# Catalyst-controlled regiodivergence and stereodivergence in formal cross-[4+2] cycloadditions: The unique effect of bismuth(III)

**DOI:** 10.1126/sciadv.adt5997

**Published:** 2025-03-26

**Authors:** Qiumeng Hou, Chenxi Cai, Shuai-Jiang Liu, Wei Huang, Cheng Peng, Gu Zhan, Bo Han

**Affiliations:** State Key Laboratory of Southwestern Chinese Medicine Resources, Hospital of Chengdu University of Traditional Chinese Medicine, School of Pharmacy, Chengdu University of Traditional Chinese Medicine, Chengdu 611137, China.

## Abstract

The [4+2] cycloaddition is crucial for constructing six-membered rings in pharmaceuticals and natural products. Cross-[4+2] cycloadditions offer greater product diversity than traditional diene-dienophile reactions due to multiple possible pathways. However, precise control over regio- and stereoselectivity for various isomers remains a great challenge. This study reports catalyst-controlled regiodivergent formal cross-cycloadditions of acyclic dienes and enones, significantly enhancing access to diverse pyrazole-fused spirooxindoles. Chiral phosphoric acid (CPA) catalysis enables endoselective [4+2] cycloadditions, while Bi(III) with a CPA ligand yields [2+4] products with high regio- and stereoselectivity. A Claisen rearrangement of the [2+4] adduct produces the exo-selective [4+2] product, further increasing stereochemical diversity and enabling the synthesis of six regio- and stereo-isomers from a single substrate set. DFT calculations reveal that Bi(III) reverses regioselectivity by repositioning reactants in the CPA pocket and stabilizing the enone oxygen’s negative charge. In addition, product **3as** demonstrates therapeutic potential against triple-negative breast cancer, with an IC_50_ of 8.5 μM in MDA-MB-453 cells.

## Teaser

Bi(III) uniquely drives regio- and stereodivergence in cross-[4+2] cycloadditions, expanding synthetic diversity.

## INTRODUCTION

Diverse compound libraries are crucial for successful drug discovery (*1*–*4*). Diversity-oriented synthesis is a powerful approach that enables researchers to efficiently explore chemical space by generating molecules with varied structures and stereochemistry (*5*–*9*). Catalyst-controlled divergent synthesis is a key method in this approach (*10*–*14*), allowing chemists to access different isomeric forms of a product from the same starting materials by simply switching the catalyst (*15*–*26*).

The [4+2] cycloaddition is fundamental for constructing six-membered rings common in pharmaceuticals and natural products (*27*, *28*). Cross-[4+2] cycloadditions of two different dienes (or a diene and an enone) offer greater product diversity than conventional diene-dienophile reactions due to multiple possible pathways (*29*–*32*). However, precisely controlling both regio- and stereoselectivity to favor various isomers remains an unattained goal, largely due to the inherent structural impact of starting materials, which limits catalyst control and often yields isomer mixtures or a single, nonswitchable product.

**Fig. 1.**
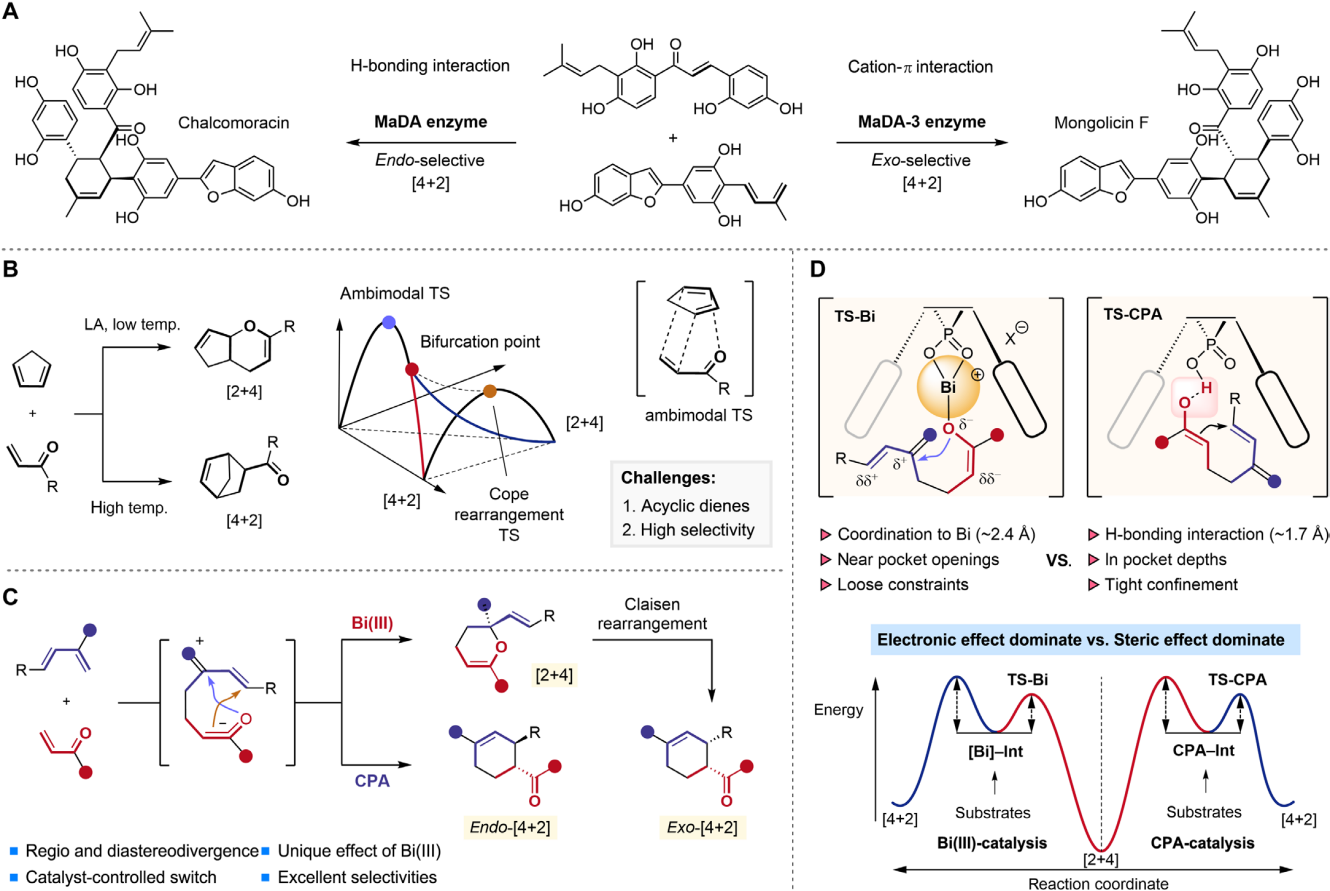
Catalyst-controlled divergence in cross-[4+2] cycloadditions: Progress, challenges, and our strategy. (**A**) Catalyst-controlled diastereodivergent cross-[4+2] cycloadditions. (**B**) Lewis acid (LA)–enabled regiodivergence in cross-[4+2] cycloadditions of Cp with enones. (**C**) This work: CPA/Bi(III)-controlled regio- and diastereodivergence in cross-[4+2] cycloadditions. (**D**) Origin of the switchable regioselectivity and the effect of Bi(III). TS, transition state.

Recent research has made strides in catalyst-controlled diastereodivergent cross-[4+2] cycloadditions (Fig. 1A) (*33*–*35*). The Lei group, for instance, developed flavin-adenine-dinucleotide–dependent enzymes catalyzing Diels-Alder reactions with opposite endo/exo selectivity and high enantioselectivity on the same substrates (*35*). The *Morus alba* Diels-Alder (MaDA) enzyme exhibits high endoselectivity, while its mutant, MaDA-3, switches to exo-selectivity.

However, catalyst-controlled regiodivergence in cross-[4+2] cycloadditions remains largely unexplored (*36*–*40*). The breakthrough has been the experimental and mechanistic study on the cross-cycloadditions of cyclopentadiene (Cp) with enones or nitroalkenes, which proceed via a single ambimodal bis-pericyclic transition state (TS), followed by a bifurcation, forming both Diels-Alder and hetero–Diels-Alder adducts (Fig. 1B) (*41*–*48*). Lewis acid catalysts can influence the bifurcating potential energy surfaces, even reversing the [4+2] selectivity of the major product at low temperatures despite moderate regioselectivity in the hetero–Diels-Alder pathway. In this research context, we aimed to develop catalyst-controlled regiodivergent cross-[4+2] cycloadditions of acyclic dienes to achieve precise regioselectivity control, with the ultimate goal of achieving both regio- and diastereodivergence, thereby significantly enhancing product diversity.

This study tackles the substantial regiodivergence challenges in cross-cycloadditions of acyclic diene and enone substrates (Fig. 1C) (*49*). Chiral phosphoric acid (CPA) facilitates high endo-[4+2] selectivity and stereoselectivity through hydrogen bonding interactions and its privileged chiral scaffold. Bismuth, the heaviest “nonradioactive” element, is affordable, is nontoxic, and has unique properties. Its sixth-period position in the periodic table results in large, diffuse, and easily polarizable valence orbitals, leading to smaller orbital overlap and weaker Bi─O bonds compared to other p-block elements. Leveraging its unique properties enables superior [2+4] selectivity in the cross-cycloadditions, in sharp contrast with rare-earth and late-transition metals (*50*–*54*). When paired with a CPA ligand, Bi(III) exhibits excellent diastereoselectivity and enantioselectivity, also maintaining good [2+4] selectivity. Moreover, a Claisen-type rearrangement of the [2+4] product yields the exo-selective [4+2] adduct, further enhancing product stereochemical diversity.

Mechanistic studies suggest that the reaction proceeds through a stepwise rather than a concerted mechanism. During the regioselectivity-determining step, catalyst-adduct complexes experience distinct transition states through hydrogen bonding with CPA or coordination to the large Bi(III) center (Fig. 1D). Bi(III) plays a crucial role in reversing regioselectivity (*55*–*57*) by adjusting reactant positioning in the CPA pocket and stabilizing the enone oxygen’s negative charge. This shifts regioselectivity from being dominated by hydrogen bonding and steric constraints to electronic effects, altering the activation energy differences between the competing formal [4+2]/[2+4] cycloaddition pathways.

## RESULTS

### Reaction optimization

Spirooxindole and pyrazole frameworks are privileged scaffolds for drug discovery (*28*). Building on our prior research in synthesizing and evaluation of these frameworks (*58*–*62*) and addressing the need for diverse structures (*63*–*65*), we selected pyrazolone-4-ylidene oxindole **1a** (*66*) and 2-trifluoroacetamido-1,3-dienes **2a** (*67*) as model substrates. First, we explored CPA as potential bifunctional catalysts, leveraging the hydrogen bond accepting and donating properties of **1a** and **2a** to influence the reaction’s regio- and stereoselectivity. CPA **C1** produced the main [4+2] cycloadduct **3aa** in toluene, with a diastereomeric ratio of 1:1 and an enantiomeric excess (ee) of 29% (Table 1, entry 1). Testing various CPA catalysts revealed the reaction was optimal with 10 mol % of the 2-naphthyl substituted H_8_-BINOL–CPA catalyst **C4** (entries 2 to 5). This reaction achieved an 87% yield of chiral spiropyrazolone **3aa** with excellent endoselectivity and high enantioselectivity at room temperature. Lowering the temperature to −10°C could further improve diastereoselectivity and enantioselectivity, affording **3aa** in 85% yield with 14:1 dr and 99% ee (entry 6).

**Table 1.**
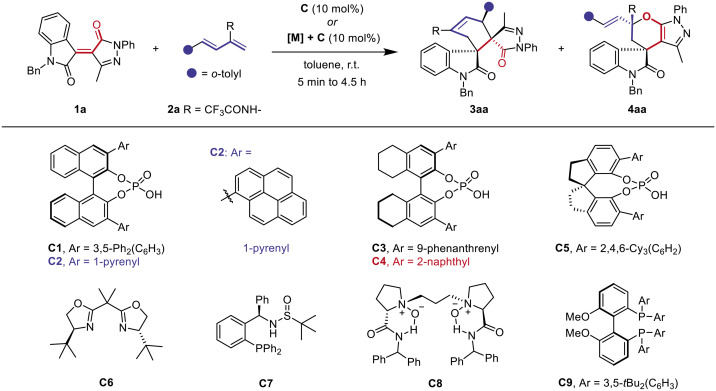
Optimization of the [4+2]/[2+4] cycloaddition conditions. Reactions conditions: **1a** (0.1 mmol), **2a** (0.15 mmol), **C** (10 mol %), and metal salt **[M]** (10 mol %) in toluene (2.0 ml) at r.t. Isolated yields were given. The dr values were determined by ^1^H NMR analysis of the crude products. The ee values were determined by high-performance liquid chromatography analysis using a chiral stationary phase.

Entry	C	[M]	*t*	Yield of 3aa (%)	Yield of 4aa (%)	dr	ee (%)
1	**C1**	–	1 hour	88	<10	1:1	29
2	**C2**	–	1 hour	83	<10	10:1	97
3	**C3**	–	4.5 hours	85	<10	4:1	0
4	**C4**	–	1 hour	87	<10	10:1	97
5	**C5**	–	4.5 hours	91	Trace	4:1	0
6*	**C4**	–	1.5 hours	85	<10	14:1	99
7	**C6**	Cu(OTf)_2_	4 hours	Trace	–	–	–
8	**C6**	Cu(CH_3_CN)BF_6_	3 hours	85	<10	>20:1	–
9	**C7**	AgOTf	4 hours	–	–	–	–
10	**C8**	Sc(OTf)_3_	3 hours	90	<10	>20:1	–
11	**C8**	La(OTf)_3_	4 hours	89	<10	>20:1	–
12	**–**	Bi(OTf)_3_	4 hours	15	79	>20:1	–
13	**C9**	Bi(OTf)_3_	1.5 hours	14	79	>20:1	0
14	**C1**	Bi(OTf)_3_	3.5 hours	<10	83	>20:1	30
15	**C2**	Bi(OTf)_3_	10 min	12	80	>20:1	99
16	**C4**	Bi(OTf)_3_	10 min	30	66	>20:1	99
17	**C5**	Bi(OTf)_3_	1.5 hours	15	81	>20:1	5
18	**C2**	BiBr_3_	3 hours	22	70	>20:1	79
19	**C2**	Bi(OAc)_3_	4 hours	64	28	>20:1	99

*At −10°C.

After establishing conditions for highly selective [4+2] cycloadditions, we explored reversing regioselectivity. Unlike previous cross–Diels-Alder reactions with enones, metal Lewis acids [Cu(OTf)_2_, Cu(CH_3_CN)BF_6_, AgOTf, Sc(OTf)_3_, and La(OTf)_3_] failed to make the desired [2+4] cycloaddition predominant (entries 7 to 11). Unexpectedly, unlike rare-earth and late-transition metals, cationic Bi(III) showed distinctive regioselectivity in the cross-cycloaddition of **1a** with **2a**. With 10 mol % Bi(OTf)_3_ in toluene, the reaction produced [2+4] cycloadduct **4aa** in 79% yield, minimizing [4+2] product **3aa** (entry 12). Encouraged, we tested chiral ligands for Bi(III). The bisphosphine **C9** maintained good regioselectivity but yielded racemic **4aa** (entry 13). Given the prominent chiral environment offered by CPAs in [4+2] cycloadditions and their compatibility with Bi(III) (*68*–*72*), we tested them as ligands. Notably, 1-pyrenyl substituted **C2** with Bi(OTf)_3_ quickly achieved high regioselectivity, diastereoselectivity, and enantioselectivity (entry 15, 80% yield, >20:1 dr, and 99% ee in 10 min). Axially chiral 1,1′-spirobiindane-7,7′-diol (SPINOL)-CPA **C5** bearing the 2,4,6-tricyclohexylphenyl groups showed low stereoselectivity despite maintaining high regioselectivity. Replacing Bi(OTf)_3_ with BiBr_3_ or Bi(OAc)_3_ will result in decreased regioselectivity of the reaction, indicating that the weakly coordinating anion is important for regioselectivity.

### Substrate scope

**Fig. 2.**
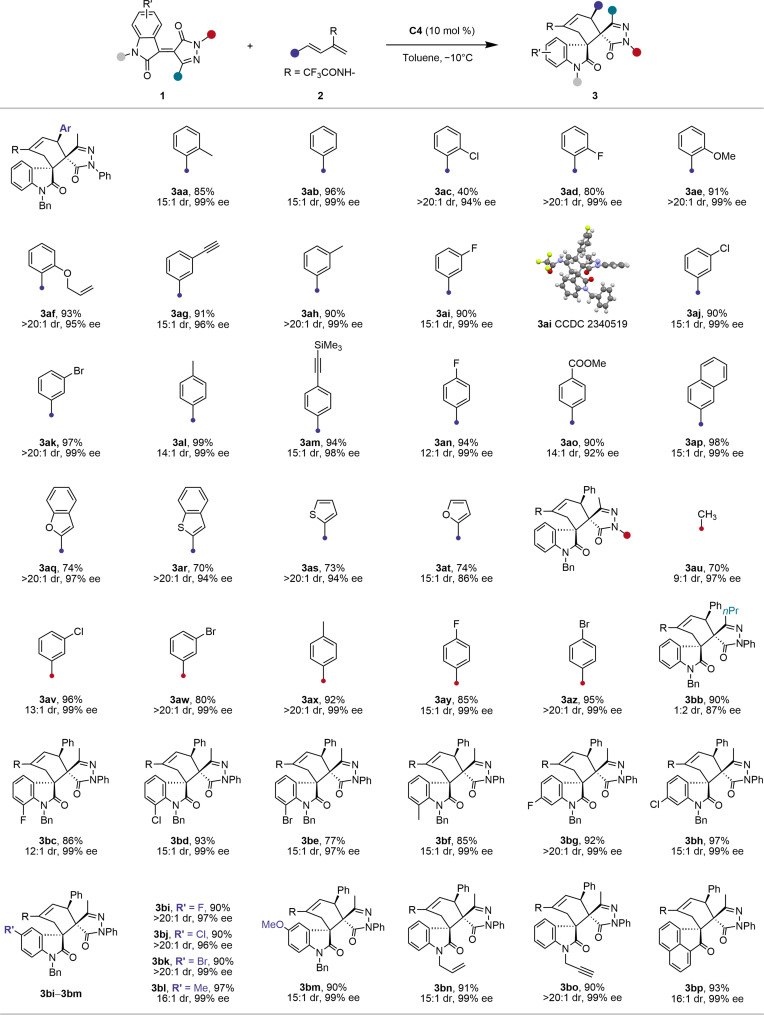
Substrate scope of the CPA-catalyzed cross-[4+2] cycloaddition. Reaction conditions: **1** (0.10 mmol), **2** (0.15 mmol), and **C4** (10 mol%) in 2.0 ml of toluene at −10°C for 1.5 to 4 hours; isolated yield.

With the regiodivergent reaction conditions established, we examined the generality of this catalyst-controlled process. Initially, a diverse array of 2-trifluoroacetamido-1,3-dienes (**2**) and pyrazolone-4-ylidene oxindoles (**1**) were explored in the CPA-controlled cross-cycloadditions (Fig. 2). A wide range of aryl-substituted dienes with electron-donating and electron-withdrawing groups (**2**) reacted smoothly with **1a**, leading to endoselective [4+2] products (**3aa**-**3ap**) with yields from 40 to 98% and ee values from 92 to 99%. Various functional groups—such as allyl, terminal alkyne, ester, and silyl groups—were well tolerated. The 1,3-dienes with heteroaromatic components such as benzofuran, benzothiophene, thiophene, and furan participated well, affording products **3aq**-**3at** in 70 to 74% yields with excellent diastereo- and enantioselectivities (15:1 to >20:1 dr, 86 to 97% ee). The structure and absolute configuration of the cross endo-[4+2] cycloadduct **3ai** were confirmed by x-ray crystallographic analysis (CCDC 2340519; see the Supplementary Materials for detailed data).

To further demonstrate the generality of this approach, optimized CPA-catalyzed conditions were applied to various pyrazolone-4-ylidene oxindoles. Substrates **1** with diverse electron-withdrawing or electron-donating groups on the *N*-phenyl ring reacted smoothly, producing endo-[4+2] cycloadducts **3au**-**3az** in 70 to 96% yield with excellent optical purity. Notably, the *N*-methyl substrate also reacted well, yielding **3au** in 70% yield with 9:1 dr and 97% ee. Modifying the methyl group at the C3 position of the pyrazolone ring did not affect the high [4+2] regioselectivity or good enantioselectivity, although diastereoselectivity was moderate (**3bb**). Increased steric hindrance between the diene’s phenyl group and the pyrazole’s propyl group during cyclization favors the formation of the exo-product **3bb′** (diastereoisomer, see Fig. 4B). We then systematically investigated substrate **1** variations on the oxindole ring. Compounds **1** with electron-withdrawing or -donating groups at the 5, 6, or 7 positions were well-tolerated in **C4**-catalyzed cycloadditions. These reactions yielded endo-[4+2] products (**3bc**-**3bm**) with high efficiency and excellent optical purity. In addition, the products **3bn** and **3bo** with allyl and propargyl substitutions on the oxindole N atom were obtained in high yields with 99% ee. It is worth noting that enones from 1,2-diketone also produced the endo-[4+2] cycloadduct **3bp** under the CPA-catalyzed standard conditions (93% yield, 16:1 dr, 99% ee).

**Fig. 3.**
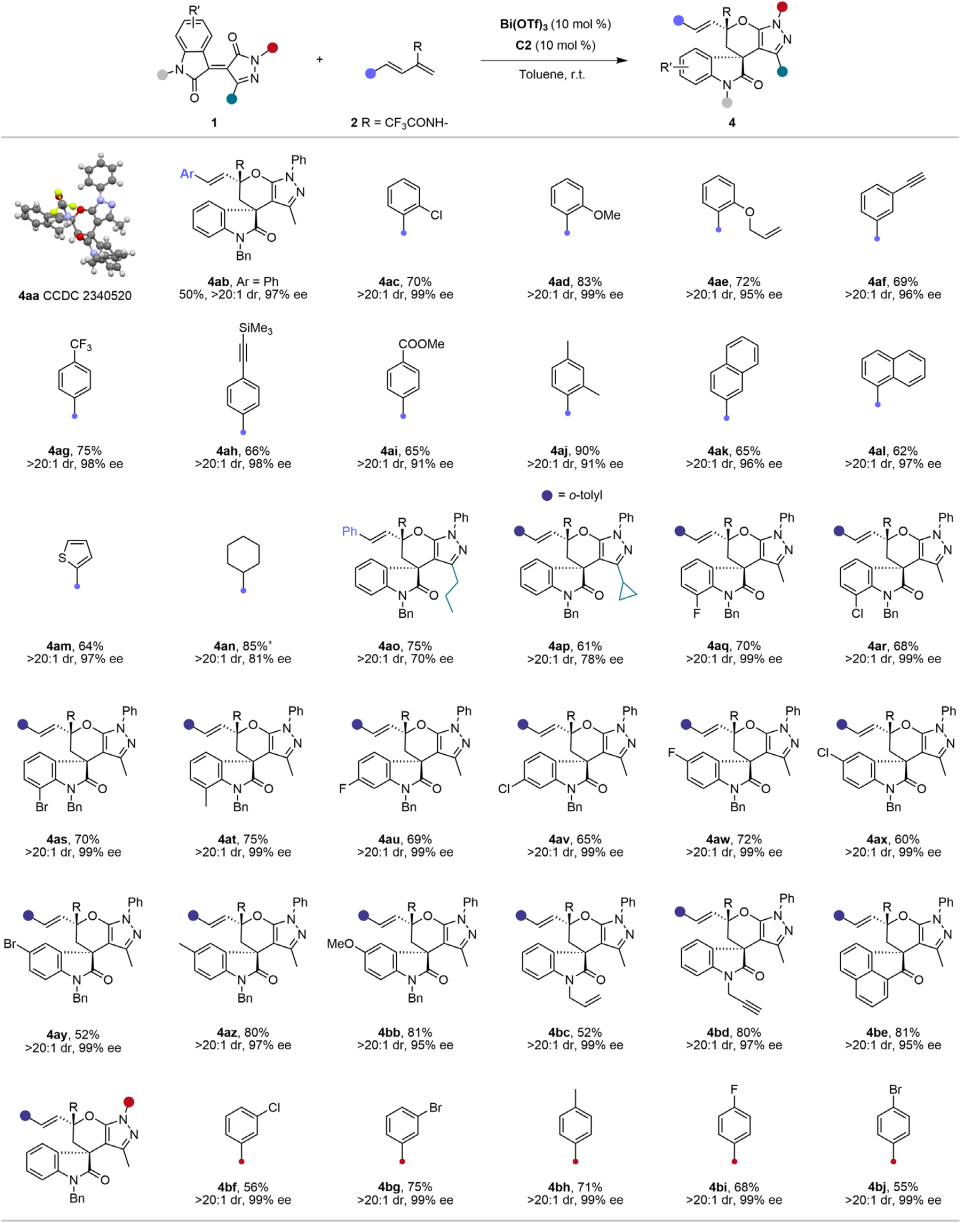
Substrate scope of the Bi(III)-catalyzed cross [2+4] cycloaddition. Reaction conditions: **1** (0.10 mmol), **2** (0.15 mmol), Bi(OTf)_3_ (10 mol%), and **C2** (10 mol%) in toluene (2.0 ml) at room temperature (r.t.) for 5 to 30 min; isolated yield. Asterisk (*) means at 0°C.

Next, we explored the scope of the Bi(III)-catalyzed cross-cycloaddition under optimal conditions. As shown in Fig. 3, the reaction accommodated various aryl and heteroaryl substituents on 2-trifluoroacetamido-1,3-dienes **2**, producing regioselective [2+4] products **4ab**-**4am** with yields of 50 to 90%, exclusive diastereoselectivity, and excellent enantioselectivity (91 to 99% ee). The [2+4] cycloadduct structure and absolute configuration of cycloadduct **4aa** were confirmed by x-ray crystallography (CCDC 2340520; see the Supplementary Materials for detailed data). Alkyl-substituted dienes **2** also reacted well; using cyclohexyl-functionalized diene, we obtained **4an** in 85% yield, >20:1 dr, and 81% ee. Pyrazolone-4-ylidene oxindoles **2** with propyl and cyclopropyl groups at C3 position afforded uncompromised regioselectivity and diastereoselectivity, with slight enantioselectivity reduction (**4ao** and **4ap**). Substrates **1** with various substituents on the oxindole scaffold also showed high regioselectivity, exclusive diastereoselectivity, and excellent enantioselectivity (95 to 99% ee) (**4aq**-**4bd**). Besides the oxindole derived substrate, [2+4] cycloadduct **4be** was also smoothly delivered from the 1,2-diketone derived enone (81% yield, >20:1 dr, 95% ee). Different substituents on the *N*-phenyl ring of pyrazole moiety did not affect the results, yielding [2+4] cycloadducts with >20:1 dr and 99% ee (**4bf**-**4bj**).

### Synthetic applications

**Fig. 4.**
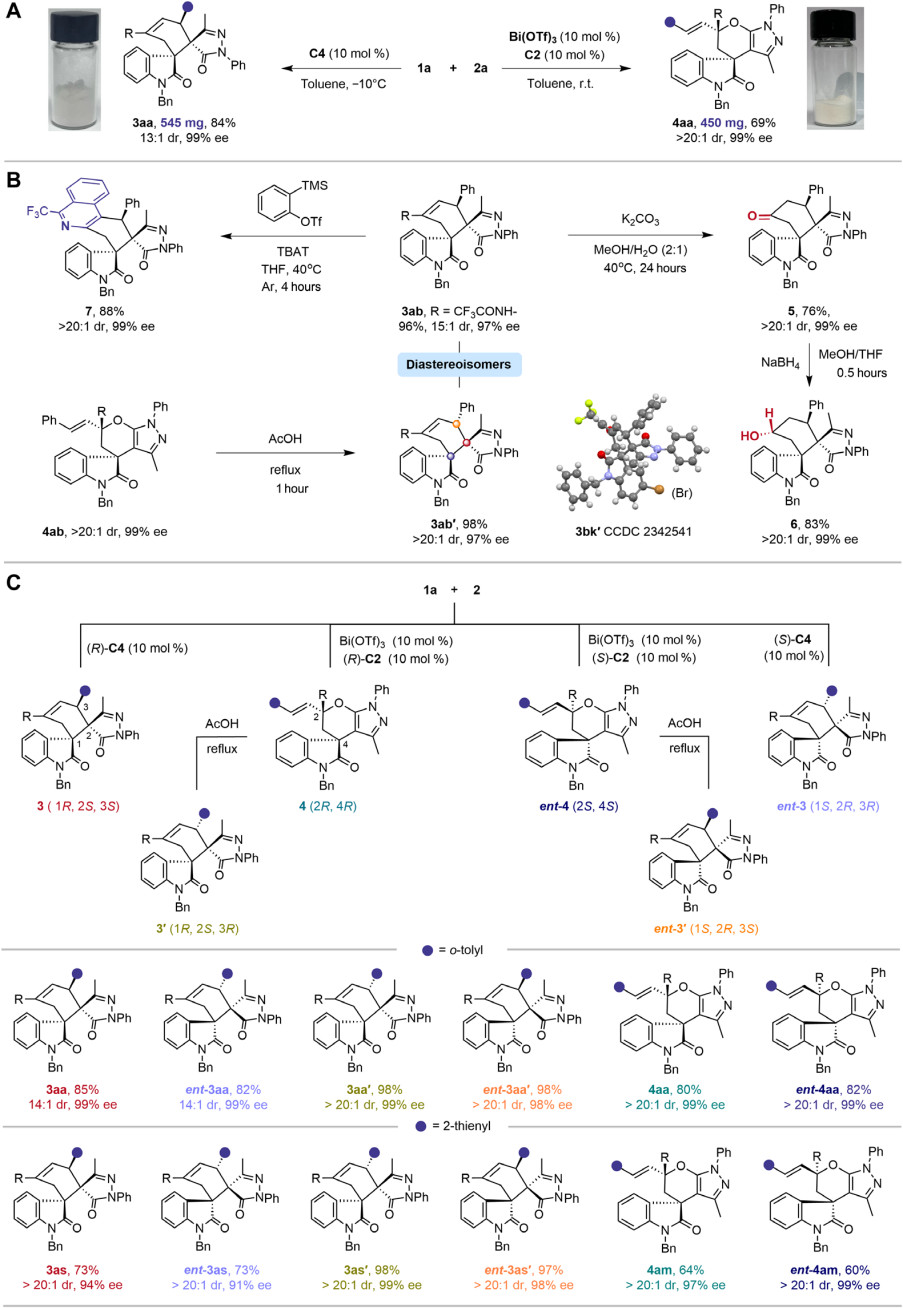
Synthetic transformations, scale-up reactions, and divergent synthesis of six isomers. (**A**) Scale-up reactions. (**B**) Synthetic transformations of [4+2] and [2+4] cycloadducts. (**C**) Regio- and stereodivergent synthesis of six isomers from **1a** and **2**. THF, tetrahydrofuran. TBAT, tetrabutylammonium difluorotriphenylsilicate.

To demonstrate the scalability of the divergent process, we conducted scale-up reactions of **1a** and **2a** using **C4** or Bi(OTf)_3_/**C2** as catalysts, respectively (Fig. 4A). The resulting products, **3aa** and **4aa**, showed uncompromised yields, regioselectivities, and stereoselectivities. The *N*-(cyclohexen-1-yl)-2,2,2-trifluoroacetamide moiety in the [4+2] cycloadduct provided a convenient handle for further elaboration (Fig. 4B). For example, **3aa** was converted to chiral cyclohexanone-fused spiropyrazolone **5** in 76% yield with >20:1 dr and 99% ee through simple hydrolysis, followed by reduction to chiral cyclohexanol-fused spiropyrazolone **6** in 83% yield with exclusive diastereoselectivity and 99% ee. In addition, the *N*-(cyclohexen-1-yl)-2,2,2-trifluoroacetamide reacted with benzyne generated in situ to provide 1-trifluoromethyl isoquinoline derivative **7**.

Heating the chiral [2+4] cycloadduct **4ab** in acetic acid triggered a Claisen rearrangement, delivering *exo*-[4+2] products **3ab′** in almost quantitative yield with exclusive diastereoselectivity and excellent enantioselectivity. This stereospecific Claisen rearrangement enables access to diastereodivergent products **3′** (*exo*-[4+2] cycloadducts) beyond the endo-[4+2] products **3** from CPA-controlled cross-cycloaddition, further enhancing the stereochemical diversity of the strategy.

By using both highly selective CPA and Bi(OTf)_3_/CPA systems for regiodivergent catalysis and a convenient Claisen rearrangement for diastereodivergent synthesis, we could easily create diverse and complementary isomeric products from the same substrate set (Fig. 4C). Starting from **1a** and **2**, chiral endo-[4+2] cycloadduct **3** and [2+4] cycloadduct **4** were efficiently constructed by using **C4** or Bi(OTf)_3_/**C2** as catalyst, respectively. The diastereoisomers, endo-[4+2] cycloadducts **3′**, were quickly obtained from **4**. By switching the (*R*)-CPA catalyst and (*R*)-CPA ligand [(*R*)-**C4**/(R)-**C2**] to their enantiomers [(*S*)-**C4**/(*S*)-**C2**], three enantiomeric products—including endo-[4+2] cycloadduct (***ent*-3**), [2+4] cycloadduct (***ent*-4**), and endo-[4+2] cycloadduct (***ent*-3′**)—were successfully obtained. All six regio- and stereoisomers were achieved with good to high yields and excellent to pure diastereoselectivities and enantioselectivities, showcasing the strategy’s capability, efficiency, and practicality in diversity-oriented synthesis. To the best of our knowledge, the catalyst-controlled switchable synthesis of regio- and diastereodivergent [4+2] cycloadducts was previously unattainable.

**Fig. 5.**
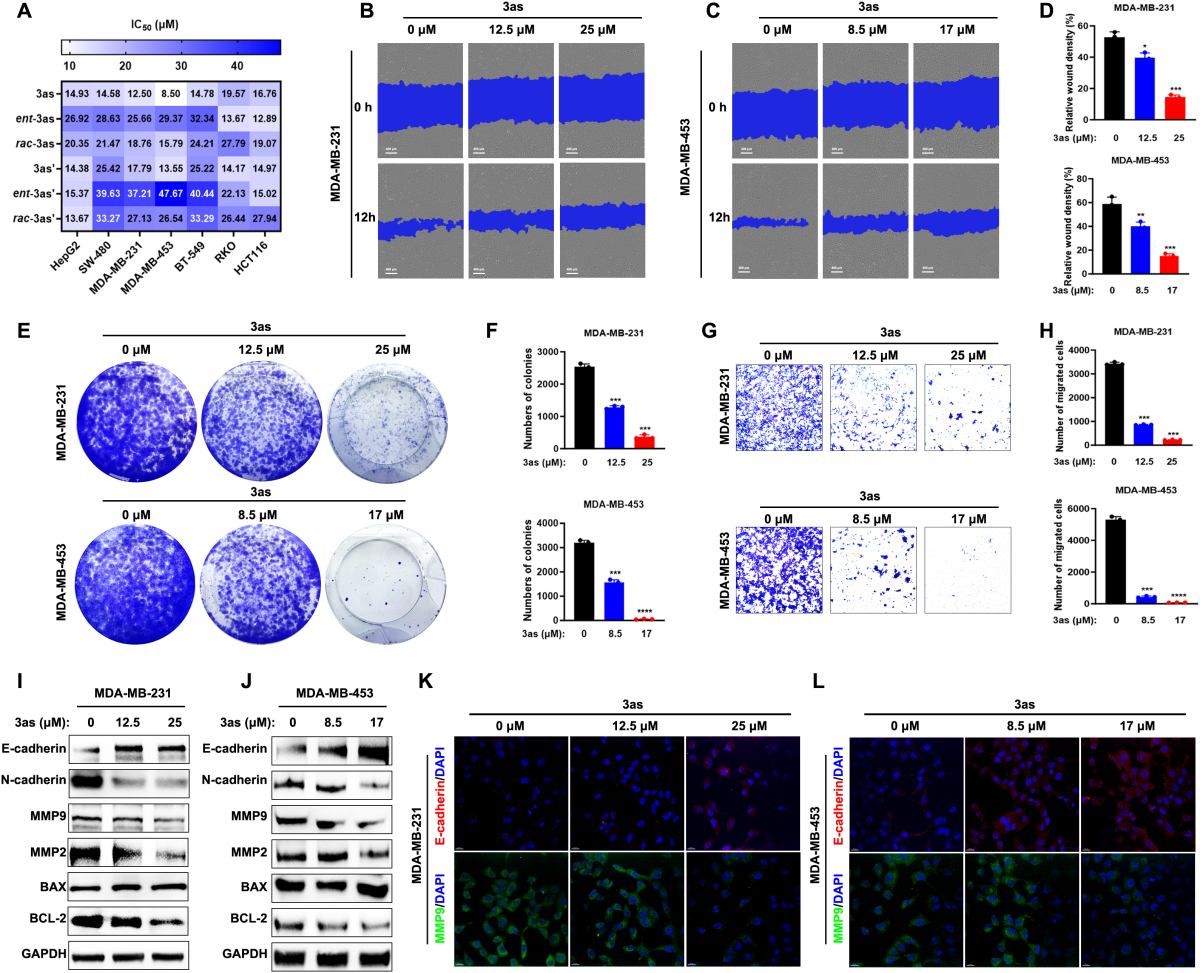
Evaluation of the antitumor activity of 3as and stereoisomers. (**A**) IC_50_ values of the **3as** series in tumor cells were determined using the MTT assay. (**B** to **D**) Cell scratch assay demonstrated that **3as** significantly inhibited the migration of TNBC cells, with corresponding quantitative data provided. Scale bars, 400 μm. (**E** and **F**) Colony formation assay confirmed that **3as** effectively suppressed the proliferation of TNBC cells, with corresponding quantitative data provided. (**G** and **H**) Transwell analysis revealed that **3as** exhibited antimigratory activity against TNBC cells, with corresponding quantitative data provided. (**I** and **J**) WB experiments indicated that **3as** modulated the expression of proteins associated with tumor metastasis and apoptosis, with glyceraldehyde-3-phosphate dehydrogenase (GAPDH) serving as a loading control. (**K** and **L**) Immunofluorescence staining further validated that **3as** regulated the expression of E-cadherin and MMP9, key markers of tumor metastasis. Scale bars, 20 μm. Data are presented as the means ± SEM. Results are consistent with at least three independent experiments. ns, not significant; *P < 0.05, **P < 0.01, ***P < 0.001, and ****P < 0.0001. Statistical significance was determined relative to the appropriate control groups.

Given the reported remarkable pharmacological activities of spirooxindoles, particularly anticancer properties, we then conducted a series of assays to evaluate the biological potential of the pyrazole-fused spirooxindole products. Using the 3-(4,5-dimethylthiazol-2-yl)-2,5-diphenyltetrazolium bromide (MTT) assay, we assessed their antitumor proliferation activities (see fig. S1 for detailed data). Most of these pyrazole-fused spirooxindole products exhibited antitumor activities, with compound **3as** showing potent cytotoxicity against two triple-negative breast cancer (TNBC) cell lines, particularly MDA-MB-453 cells, with a half-maximal inhibitory concentration (IC_50_) value of 8.5 μM. This prompted further investigation of **3as**, including its diastereomers, enantiomers (***ent*-3as**, **3as′**, and ***ent*-3as′**) and racemets of **3as** and **3as′**. The MTT assay results revealed that configurations of different chiral stereoisomers significantly influence their biological activity, with **3as** outperforming other isomers in most tumor cell lines (Fig. 5A).

On the basis of these findings, we focused on **3as** for further studies in TNBC cell lines. Because tumor metastasis is a major challenge in TNBC (*73*, *74*), we evaluated the antimigratory effects of **3as** using wound healing and Transwell assays (Fig. 5, B to D and G and H), which demonstrated its potent inhibition of tumor migration. Colony formation assays (Fig. 5, E and F) showed that **3as** suppressed TNBC cell proliferation in a concentration-dependent manner. To explore the mechanisms underlying its antimetastatic effects, we analyzed the expression of key metastasis-related proteins [E-cadherin, N-cadherin, matrix metalloproteinase 2 (MMP2), and MMP9] via Western blot (WB). **3as** up-regulated E-cadherin while down-regulating N-cadherin, MMP2, and MMP9 (Fig. 5, I and J, and fig. S1), findings further supported by immunofluorescence (Fig. 5, K and L). Regarding its antiproliferative effects, WB data suggest that **3as** induces apoptosis by increasing pro-apoptotic BAX and decreasing anti-apoptotic BCL-2 expression. Collectively, these results indicate that **3as** has good antitumor activity, effectively inhibiting both the proliferation and metastasis of TNBC. The activity differences among the compound′s stereoisomers underscore the importance of precise regio- and stereoselectivity control in complex molecular frameworks.

### Mechanism studies

**Fig. 6.**
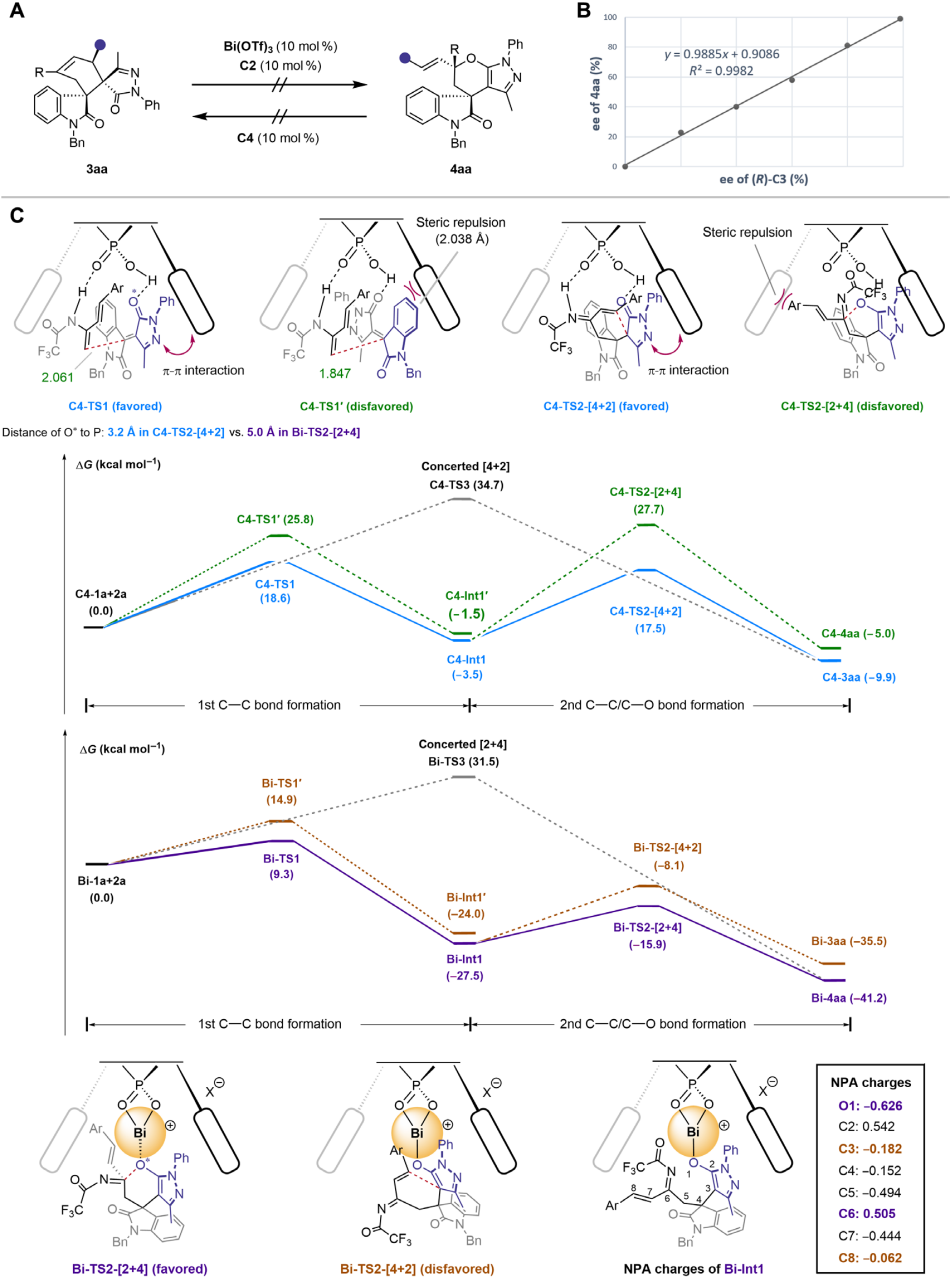
Mechanistic investigations. (**A**) Interconversion experiments. (**B**) Nonlinear effect experiment. (**C**) DFT calculations of the catalyst-controlled regiodivergent reactions between **1a** and **2a**.

To investigate the reaction mechanism, we first examined the stability and potential interconversion between the [4+2] and [2+4] products. Experiments revealed no interconversion between **3aa** and **4aa** under CPA or Bi(III)-catalysis (Fig. 6A). Extending the times of the two model reactions to 96 hours also did not yield corresponding regioisomers (see the Supplementary Materials for details). In addition, we observed a negative nonlinear effect between the ee values of the ligand **C2** and the products under optimal conditions, suggesting that only one molecule of **C2** is likely involved in the enantio-determining step (Fig. 6B).

To further explore the reaction mechanism and comprehend the origins of catalyst-controlled regioselectivity and enantioselectivity, we used density functional theory (DFT) calculations (Fig. 6C; see the “DFT calculations” section in the Supplementary Materials for details) (*75*–*79*). The results support stepwise rather than concerted pathways in both the [4+2] or [2+4] reactions, and the first C─C bond formation step determines enantioselectivity. In CPA **C4**-catalyzed reactions, the free-energy surface starts from the **C4-1a** complex, formed by a hydrogen bond between CPA’s acidic hydrogen and pyrazolone’s oxygen. Two stereoisomeric transition states, **C4-TS1** and **C4-TS1′**, lead to **3aa** and its enantiomer (***ent*-3aa**), respectively. **C4** acts as a bifunctional hydrogen bond donor and acceptor, interacting with both substrate **1a** and diene **2a**. The favored **C4-TS1** has a C─C bond distance of 2.06 Å, while the unfavored **C4-TS1′** is 1.85 Å apart. **C4-TS1** is energetically more favored than **C4-TS1′**, consistent with the experimentally observed absolute configuration of the [4+2] product. This energy difference arises from steric repulsion in **C4-TS1′** between the oxindole moiety and the 2-naphthyl group.

The favored intermediate **C4-Int1** undergoes cyclization to produce either [4+2] or [2+4] cycloadducts (**3aa** or **4aa**) through **C4-TS2**, which governs both the rate-determining and regioselectivity-determining steps. **C4-TS2-[4+2]** has a lower relative free energy (Δ*G* = 17.5 kcal/mol) compared to **C4-TS2-[2+4]** (Δ*G* = 27.7 kcal/mol), supporting the observed high regioselectivity for [4+2]. The difference between the transition states lies in the spatial arrangement of the pyrazolyl and phenyl vinyl groups relative to CPA, with **C4-TS2-[2+4]** experiencing significant steric repulsion. A strong π-π interaction between **C4**’s 2-naphthyl group and the *N*-phenyl pyrazolone moiety reduces the energy of **C4-TS2-[4+2]**. A concerted Diels-Alder pathway was also considered; however, the **C4-TS3** transition state had a much higher barrier (34.7 kcal/mol).

For Bi(III)-catalyzed [2+4] reactions, we used the smaller ligand **C4** for computational efficiency. The stepwise reaction begins with the **Bi-1a** complex, featuring a Bi─O bond between the metal center and pyrazolone’s oxygen. During the first C─C bond formation step through **Bi-TS**, the chiral environment from the BINOL framework results in stereocontrol similar to the CPA-catalyzed process, producing the same configuration for the first quaternary stereocenter in product **4aa** as in **3aa**. We further explored the subsequent C─C or C─O bond formation step to understand Bi-controlled regioselectivity. In the cyclization step’s transition states, **Bi-TS2-[4+2]** and **Bi-TS2-[2+4]**, the Bi─O bond length is 2.3 to 2.6 Å. The distances between the pyrazolone oxygen atom (O*, involved in coordination or hydrogen bonding) and the phosphorus atom in the CPA pocket were measured: 3.2 Å in **C4-TS2-[4+2]** and 5.0 Å in **Bi-TS2-[2+4]**. The primary difference from **C4-TS2-[4+2]** is that the adduct is farther from the CPA’s pocket, reducing spatial constraints and enabling the C─O pathway. The relative free energy of **Bi-TS2-[4+2]** is higher than **Bi-TS2-[2+4]**, aligning with the selective formation of **4aa**. Natural population analysis of **Bi-Int1** shows partial charge separation on the Bi─O bond (O1: −0.626; C3: −0.182; C6: 0.505; C8: −0.062), with the cationic Bi stabilizing the negative O atom. The long Bi─O bond accommodates C─O formation, allowing Bi(III)'s unique properties to reverse CPA-controlled [4+2] selectivity and enable a selective [2+4] pathway. Last, a concerted Bi-catalyzed hetero–Diels-Alder reaction was considered. The significantly higher energy of the **Bi-TS3** transition state (due to steric factors) compared to the stepwise pathway suggests that a concerted [2+4] mechanism is unlikely.

## DISCUSSION

In summary, we effectively tackled the regiodivergence challenges in cross-cycloadditions of acyclic diene and enone substrates, achieving both regio- and diastereodivergence, which greatly enhanced the diversity of pyrazole-fused spirooxindole products. CPA ensures high endo-[4+2] selectivity and stereoselectivity through hydrogen bonding and its chiral scaffold. The unique properties of Bi(III) are key to reversing [4+2] selectivity, enabling superior [2+4] selectivity. Combining cationic Bi(III) with a CPA ligand results in excellent diastereoselectivity and enantioselectivity, while maintaining good [2+4] selectivity. A Claisen-type rearrangement of the [2+4] product smoothly produces the *exo*-selective [4+2] adduct, further increasing stereochemical diversity. Using (*R*)- or (*S*)-configured CPA as the catalyst or ligand allows for the divergent synthesis of six regio- and stereoisomers from the same substrates. The reaction supports a wide range of substrates with excellent regioselectivity, diastereoselectivity, and enantioselectivity. Moreover, the pyrazole-fused spirooxindole product **3as** shows therapeutic potential against TNBC, with an IC_50_ of 8.5 μM in MDA-MB-453 cells.

Control experiments and DFT calculations provided insights into the mechanism and catalyst-controlled regioselectivities. The reaction follows a stepwise mechanism. During the regioselectivity-determining step, catalyst-adduct complexes undergo distinct transition states via hydrogen bonding with CPA or coordination to the large Bi(III) cation. Bi(III) is crucial in reversing regioselectivity by repositioning reactants in the CPA pocket and stabilizing the enone oxygen’s negative charge, shifting regioselectivity from hydrogen bonding and steric constraints to electronic effects, and altering activation energy differences between the formal [4+2]/[2+4] cycloaddition pathways.

## MATERIALS AND METHODS

### General information

Unless stated otherwise, all reactions were carried out under an atmosphere of Ar. Commercial reagents and solvents were obtained from Adamas-Beta, Macklin, Alfa Aesar, Aldrich Chemical Co., Energy Chemical, and Leyan. Pyrazolone-4-ylidene oxindole **1a** and 2-trifluoroacetamido-1,3-dienes **2a** were synthesized according to the literature procedures. Bi(OTf)_3_ was purchased from Adamas-Beta Co. and used without further treatment. Analytical thin-layer chromatography (TLC) was performed on silica gel HSGF_254_ glass plates (purchased from Jiangyou Silica Gel Development Co. Ltd., Yantai, China) containing a 254-nm fluorescent indicator. Flash column chromatography was performed over silica gel (200 to 300 mesh). ^1^H nuclear magnetic resonance (NMR), ^13^C NMR, and ^19^F NMR spectra were recorded at 25°C on a Bruker 600 MHz or JEOL 600 NMR instrument. High-resolution mass spectra were obtained using Agilent P/N G1969–90010. Melting points were recorded on BUCHI Melting Point M-565 instrument. Enantiomeric ratios were determined by high-performance liquid chromatography analysis on an Agilent 1260 Infinity II or SHIMADZU SIL-16 using chiral columns in comparison with authentic racemates. Chiral columns, Daicel Chiralpak IB Column (250 mm by 4.6 mm), Daicel Chiralpak IG Column (250 mm by 4.6 mm), Daicel Chiralpak IC Column (250 mm by 4.6 mm), and Daicel Chiralpak ID Column (250 mm by 4.6 mm) were used. Ultraviolet detection was performed at 254 nm.

### General procedure for the CPA-catalyzed cross-[4+2] cycloaddition

A 10-ml reaction tube was filled with 1 (0.1 mmol, 1.0 equiv.), 2 (0.15 mmol, 1.5 equiv.), (*R*)-**C4** (10 mol%, 6.1 mg), and toluene (2.0 ml). The mixture was stirred at −10°C for 1.5 hours, monitored by TLC. After completion, it was concentrated under reduced pressure, and the crude material was purified by column chromatography on silica gel using petroleum ether and ethyl acetate (20:1) as eluents to yield product **3**.

### General procedure for the bi(III)-catalyzed cross-[2+4] cycloaddition

Bi(OTf)_3_ (10 mol%, 6.6 mg) and (*R*)-**C2** (10 mol%, 7.5 mg) were added to a Schlenk tube, followed by toluene (2.0 ml). The solution was stirred at room temperature for 0.5 hours. Subsequently, **1** (0.1 mmol, 1.0 equiv.) and **2** (0.15 mmol, 1.5 equiv.) were introduced. The reaction proceeded for 5 to 10 min at room temperature and was monitored by TLC. Upon completion, the mixture was concentrated under reduced pressure, and the crude material was purified via column chromatography on silica gel using petroleum ether and ethyl acetate (30:1) as eluents, yielding product **4**.
