# Supplementary material for: Catalyst-controlled regiodivergence and stereodivergence in formal cross-[4+2] cycloadditions: The unique effect of bismuth(III)
Source: Sci Adv. 2025 Mar 26;11(13):eadt5997. doi: 10.1126/sciadv.adt5997 (PMC11939037; doi:10.1126/sciadv.adt5997)

Science Advances
AAAS

Supplementary Materials for

Catalyst-controlled regiodivergence and stereodivergence in formal cross-[4+2] cycloadditions: The unique effect of bismuth(III)

Qiumeng Hou *et al.*

Corresponding author: Gu Zhan, zhangu@cdutcm.edu.cn; Bo Han, hanbo@cdutcm.edu.cn

Sci. Adv. **11**, eadt5997 (2025)
DOI: 10.1126/sciadv.adt5997

This PDF file includes:

Supplementary Text
Tables S1 to S3
Figs. S1 and S2

1. General information

Enantiomeric excess was determined by HPLC analysis on an Agilent 1260 Infinity II or SHIMADZU SIL-16 using chiral columns in comparison with authentic racemates. Chiral columns, Daicel Chiralpak IB Column (250 x 4.6 mm), Daicel Chiralpak IG Column (250 x 4.6 mm), Daicel Chiralpak IC Column (250 x 4.6 mm), Daicel Chiralpak ID Column (250 x 4.6 mm). UV detection was performed at 254 nm. Nuclear magnetic resonance (NMR) spectra were recorded in $CDCl_3$ and DMSO-d_6 on Bruker 600 MHz, or JEOL 600 NMR instrument for ^{1}H, at 150 MHz for ^{13}C and at 564 MHz for ^{19}F. Proton chemical shifts are reported in parts per million (δ scale). The ^{1}H NMR chemical shifts are reported in ppm with the internal TMS signal at 0.0 ppm as standard. The ^{13}C NMR chemical shifts were given using $CDCl_3$ or DMSO-d_6 as the internal standard ($CDCl_3$: δ = 77.04 ppm, DMSO-d_6: δ = 39.98 ppm). The ^{19}F NMR chemical shifts were given by using $CDCl_3$ or DMSO-d_6. Data are reported as follows: chemical shift [multiplicity (s = singlet, d = doublet, t = triplet, q = quartet, m = multiplet, dd = doublet of doublets, td = triplet of doublets), coupling constant(s) (Hz), integration]. High-resolution mass spectra (HRMS) were obtained using Agilent P/N G1969-90010. High-resolution mass spectra were reported for the molecular ion $[M+Na]^+$. X-ray diffraction experiment was carried out on an Agilent Gemini and the data obtained were deposited at the Cambridge Crystallographic Data Centre. Analytical thin-layer chromatography was performed on silica gel $HSGF_{254}$ glass plates (purchased from Jiangyou Silica Gel Development Co. Ltd., Yantai, China) containing a 254 nm fluorescent indicator. Column chromatography was performed on silica gel (200–300 mesh) using an eluent of ethyl acetate (EA) and petroleum ether (PE). Optical rotation values were measured with instruments operating at λ = 589 nm, corresponding to the sodium D line at 25 °C. Melting points were recorded on BUCHI Melting Point M-565 instrument. Unless stated otherwise, all reactions were carried out under an atmosphere of Ar. Commercial reagents and solvents were obtained from Adamas-Beta, Macklin, Alfa Aesar, Aldrich Chemical Co., Energy Chemical, and Leyan. Pyrazolone-4-ylidene oxindole **1** (*67*) and 2-trifluoroacetamido-1,3-dienes **2** (*68*) were synthesized according to the literature procedures. $Bi(OTf)_3$ was purchased from Adamas-Beta Co. and used without further treatment.

2. Optimization of the [4+2]/[2+4] cycloaddition conditions

Table S1. **Optimization of [4+2] cycloaddition**. Reaction conditions: **1a** (0.10 mmol, 1.0 equiv.), **2a** (0.15 mmol, 1.5 equiv.), **CPA** (10 mol%) in solvent (2.0 mL) at the indicated temperature for the corresponding time.

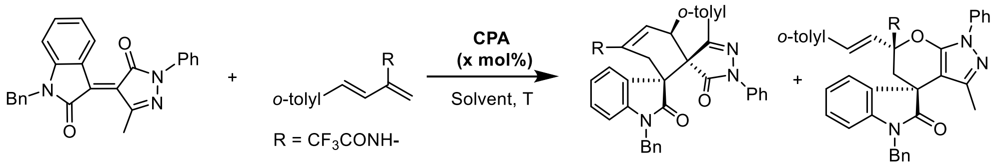

1a **2a** **3aa** **4aa**

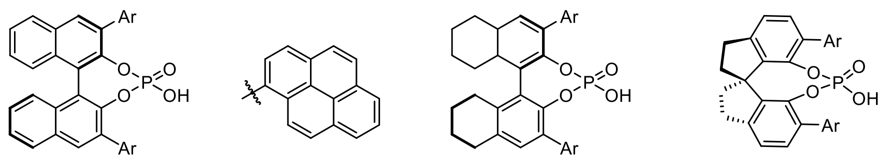

C1, Ar = 3,5-Ph$_2$(C$_6$H$_3$)
C2, Ar = pyrenyl
C2, Ar = pyrenyl
C3, Ar = 9-phenanthrenyl
C4, Ar = 2-naphthyl
C5, Ar = =2,4,6-Cy$_3$(C$_6$H$_2$)

Entry	**CPA**	Solvent	x	T(°C)	*t* (h)	Yield of **3aa** (%)*	Yield of **4aa** (%)*	dr of **3aa**†	ee of **3aa** (%)‡
1	**C1**	toluene	10	r.t.	4.5	88	<10	1:1	29
2	**C2**	toluene	10	r.t.	1.0	83	<10	10:1	97
3	**C3**	toluene	10	r.t.	4.5	85	<10	4:1	0
4	**C4**	toluene	10	r.t.	1.0	87	<10	10:1	97
5	**C5**	toluene	10	r.t.	4.5	91	trace	4:1	0
6	**C4**	DCM	10	r.t.	2	60	35	3:1	87
7	**C4**	DCE	10	r.t.	2	50	42	5:1	83
8	**C4**	THF	10	r.t.	4	98	trace	7:1	0
9	**C4**	toluene	10	0	1.5	85	<10	12:1	97
10	**C4**	toluene	10	–10	1.5	85	<10	14:1	99
11	**C4**	toluene	10	–20	4	93	trace	15:1	83
12	**C4**	toluene	5	–10	5	70	25	10:1	91

*Isolated yield. †Determined by ^{1}HNMR analysis. ‡ Determined by chiral HPLC analysis.

Table S2. **Optimization of [2+4] cycloaddition.** Reaction conditions: **1a** (0.10 mmol, 1.0 equiv.), **2a** (0.15 mmol, 1.5 equiv.), **[M]** and **C** in solvent (2.0 mL) at r.t. for the corresponding time.

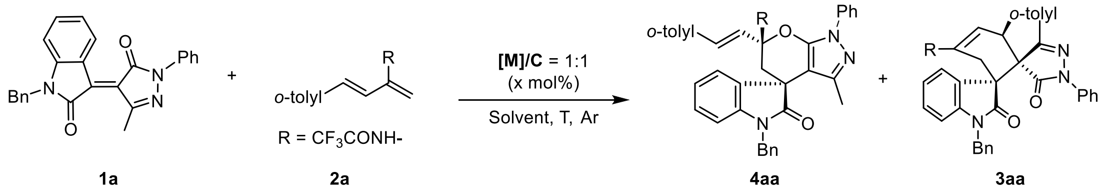

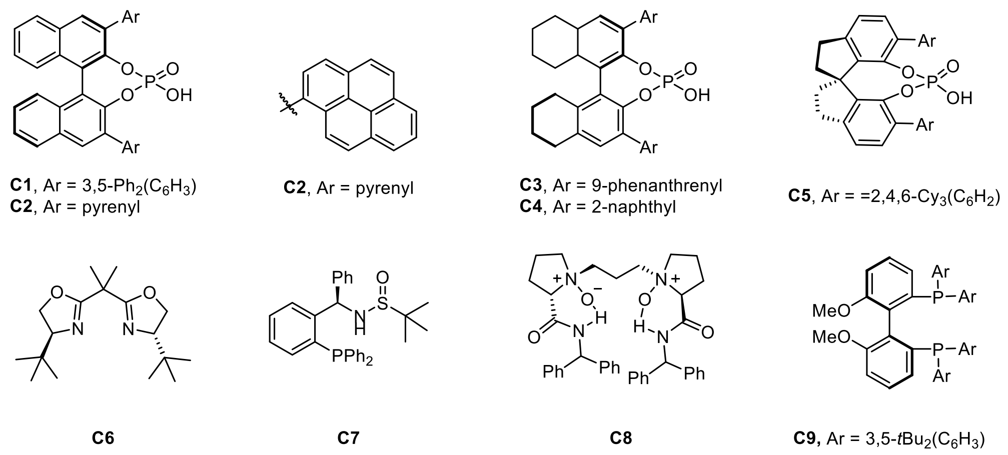

Entry	[M]	C	Solvent	x	*t*	Yield of **4aa** (%)*	Yield of **3aa** (%)*	dr of **4aa**†	ee of **4aa** (%)‡
1	$Bi(OTf)_3$	-	toluene	10	4 h	79	15	-	-
2	$Bi(OTf)_3$	**C1**	toluene	10	3.5 h	83	10	> 20:1	30
3	$Bi(OTf)_3$	**C2**	toluene	10	10 min	80	12	> 20:1	99
4	$Bi(OTf)_3$	**C3**	toluene	10	3.5 h	40	54	> 20:1	97
5	$Bi(OTf)_3$	**C4**	toluene	10	10 min	66	30	> 20:1	99
6	$Bi(OTf)_3$	**C5**	toluene	10	1.5 h	81	15	> 20:1	5
7	$BiBr_3$	**C2**	toluene	10	3 h	70	22	> 20:1	79
8	$Bi(OAc)_3$	**C2**	toluene	10	4 h	28	64	> 20:1	99
9	$Cu(OTf)_2$	**C6**	toluene	10	4 h	trace	-	-	-
10	$Cu(CH_3CN)BF_6$	**C6**	toluene	10	3 h	<10	85	> 20:1	-
11	AgOTf	**C7**	toluene	10	12 h	-	-	-	-
12	$Sc(OTf)_3$	**C8**	toluene	10	3 h	<10	90	> 20:1	-
13	$La(OTf)_3$	**C8**	toluene	10	4 h	<10	89	> 20:1	-
14	$Bi(OTf)_3$	**C9**	toluene	10	1.5 h	79	14	> 20:1	0
15	$Bi(OTf)_3$	**C2**	DCM	10	10 min	60	30	> 20:1	97
16	$Bi(OTf)_3$	**C2**	DCE	10	10 min	65	30	> 20:1	90
17	$Bi(OTf)_3$	**C2**	THF	10	10 min	NR	98	> 20:1	-

18	$Bi(OTf)_3$	**C2**	toluene	7.5	10 min	70	25	> 20:1	91
19	$Bi(OTf)_3$	**C2**	toluene	5	15 min	60	32	> 20:1	95

*Isolated yield. †Determined by ^{1}H NMR. ‡Dtermined by HPLC analys

3. General procedure for the synthesis of product 3

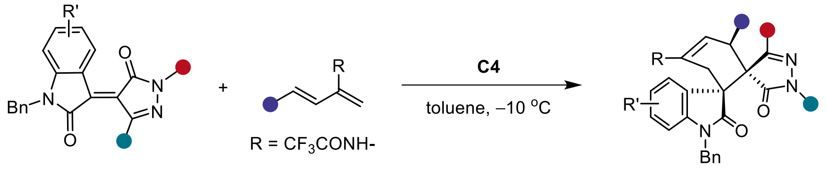

Procedure for synthesis of product **3**

In a 10 mL reaction tube, combine **1** (0.1 mmol, 1.0 equiv.), **2** (0.15 mmol, 1.5 equiv.), (*R*)-**C4** (10 mol%, 6.1 mg), and toluene (2.0 mL). Stir the mixture at −10 °C for 1.5 hours, monitoring by TLC. Upon completion, concentrate under reduced pressure and purify the crude product by silica gel column chromatography using petroleum ether and ethyl acetate (20/1) as eluents to yield **3**. Dry the product under vacuum, and analyze using ^{1}H NMR, ^{13}C NMR, HRMS, and chiral HPLC, *etc*.

Procedure for synthesis of product ***ent*-3**

In a 10 mL reaction tube, combine **1** (0.1 mmol, 1.0 equiv.), **2** (0.15 mmol, 1.5 equiv.), (*S*)-**C4** (10 mol%, 6.1 mg), and toluene (2.0 mL). Stir the mixture at −10 °C for 1.5 h, monitoring with TLC. After completion, concentrate under reduced pressure and purify the crude product using silica gel column chromatography with petroleum ether and ethyl acetate (20/1) as eluents to yield ***ent*-3**. Dry the product under vacuum and analyze using ^{1}H NMR, ^{13}C NMR, HRMS, and chiral HPLC, *etc*.

_N_-((3*R*,5'*S*,6'*S*)-1-benzyl-3''-methyl-2,5''-dioxo-1''-phenyl-5'-(*o*-tolyl)-1'',5''-dihydrodispiro [indoline-3,1'-cyclohexene-6',4''-pyrazol]-3'-yl)-2,2,2-trifluoroacetamide (3aa)

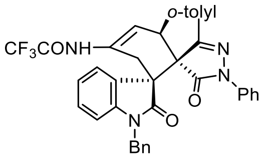

Prepared according to the general procedure, the crude product was purified by silica gel chromatography (petroleum ether/ethyl acetate 20:1) to afford **3aa** (55.5 mg, 85% yield, white solid, 14:1 dr, m.p.: 245.9 – 250.1 °C).

The enantiomeric excess was determined to be 99% by HPLC with a Daicel Chiralpak IB (*n*-hexane/2-propanol = 90:10, 1.0 mL/min, at 254 nm): t_R = 10.19 min (major), t_R = 12.61 min (minor); $[\alpha]_D^{20}$ = –550.200 (*c* = 0.10, EA).

NMR and HRMS data for the product **3aa**:

^{1}H NMR (600 MHz, Chloroform-*d*) δ (ppm): 7.59 (d, *J* = 7.8z Hz, 1H), 7.44 (s, 1H), 7.40 (d, *J* = 8.4 Hz, 2H), 7.32 (t, *J* = 7.8 Hz, 1H), 7.29 – 7.26 (m, 1H), 7.20 (t, *J* = 1.2 Hz, 1H), 7.11 (t, *J* = 7.8 Hz, 3H), 7.07 (q, *J* = 7.8 Hz, 2H), 7.02 – 7.00 (m, 3H), 6.97 (d, *J* = 7.8 Hz, 1H), 6.87 (t, *J* = 7.2 Hz, 2H), 6.77 (d, *J* = 7.8 Hz, 1H), 6.57 (s, 1H), 5.12 (d, *J* = 15.6 Hz, 1H), 5.03 (s, 1H), 4.44 (d, *J* = 15.6 Hz, 1H), 3.55 (d, *J* = 18.6 Hz, 1H), 2.78 (s, 3H), 2.40 (d, *J* = 18.0 Hz, 1H), 2.17 (s, 3H).

^{13}C NMR (150 MHz, Chloroform-*d*) δ (ppm): 174.4, 170.0, 158.7, 155.2 (q, *J* = 37.2 Hz), 142.4, 137.2, 136.9, 134.8, 130.9, 130.3, 129.7, 129.4, 129.0, 128.7, 128.5, 128.0, 127.5, 126.8, 125.9, 125.1, 124.2, 123.4, 120.4, 119.4, 118.4, 115.5 (q, *J* = 286.8 Hz), 109.4, 59.9, 51.3, 44.1, 41.7, 34.1, 20.0, 19.8.

^{19}F NMR (564 MHz, Chloroform-*d*) δ (ppm): −75.58.

HRMS (ESI-TOF) m/z: $[M+Na]^+$ Calcd for $C_{38}H_{31}F_3N_4NaO_3^+$ 671.2240, Found: 671.2236.

***N*-((3*R*,5'*S*,6'*S*)-1-benzyl-3''-methyl-2,5''-dioxo-1'',5'-diphenyl-1'',5''-dihydrodispiro[indoline-3,1'-cyclohexene-6',4''-pyrazol]-3'-yl)-2,2,2-trifluoroacetamide (3ab)**

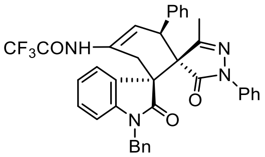

Prepared according to the general procedure, the crude product was purified by silica gel chromatography (petroleum ether/ethyl acetate 20:1) to afford **3ab** (61.2 mg, 96% yield, white solid, 15:1 dr, m.p: 254.6 – 258.9 °C).

The enantiomeric excess was determined to be 99% by HPLC with a Daicel Chiralpak IG (*n*-hexane/2-propanol = 70:30, 1.0 mL/min, at 254 nm): t_R = 8.28 min (minor), t_R = 14.65 min (major); $[\alpha]_D^{20}$ = – 780.545 (*c* = 0.11, EA).

NMR and HRMS data for the product **3ab**:

^{1}H NMR (600 MHz, Chloroform-*d*) δ (ppm): 7.56 (d, *J* = 7.8 Hz, 1H), 7.54 (d, *J* = 8.4 Hz, 2H), 7.52 (s, 1H), 7.34 (t, *J* = 7.8 Hz, 1H), 7.28 – 7.25 (m, 2H), 7.22 – 7.19 (m, 6H), 7.13 (t, *J* = 7.8 Hz, 1H), 7.06 (t, *J* = 7.8 Hz, 1H), 7.03 (d, *J* = 7.8 Hz, 2H), 6.91 (t, *J* = 7.8 Hz, 2H), 6.88 (s, 1H), 6.79 (d, *J* = 7.8 Hz,1H), 5.07 (d, *J* = 15.6 Hz, 1H), 4.60 (s, 1H), 4.48 (d, *J* = 15.6 Hz, 1H), 3.51 (d, *J* = 18.0 Hz, 1H), 2.55 (s, 3H), 2.34 (d, *J* = 18.0 Hz, 1H).

^{13}C NMR (150 MHz, Chloroform-*d*) δ (ppm): 174.3, 170.3, 158.7, 155.3 (q, *J* = 37.4 Hz), 142.2, 137.3, 136.1, 134.8, 130.1, 129.9, 129.3, 128.9, 128.7, 128.5, 128.2, 128.1, 127.5, 126.8, 125.1, 124.5, 123.4, 119.4, 118.6, 115.5 (q, *J* = 287.0 Hz), 109.4, 60.4, 51.1, 45.6, 44.1, 34.0, 18.8.

^{19}F NMR (564 MHz, Chloroform-*d*) δ (ppm): −75.54.

HRMS (ESI-TOF) m/z: $[M+Na]^+$ Calcd for $C_{37}H_{29}F_3N_4NaO_3^+$ 657.2084, Found: 657.2084.

<u>*N*-((3*R*,5'*S*,6'*S*)-1-benzyl-5'-(2-chlorophenyl)-3''-methyl-2,5''-dioxo-1''-phenyl-1'',5''-dihydrodispiro [indoline-3,1'-cyclohexene-6',4''-pyrazol]-3'-yl)-2,2,2-trifluoroacetamide (3ac)</u>

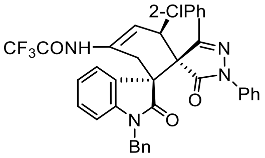

Prepared according to the general procedure, the crude product was purified by silica gel chromatography (petroleum ether/ethyl acetate 20:1) to afford **3ac** (26.8 mg, 40% yield, white solid, > 20:1 dr, m.p: 256.4 – 258.7 °C).

The enantiomeric excess was determined to be 94% by HPLC with a Daicel Chiralpak IG (*n*-hexane/2-propanol = 70:30, 1.0 mL/min, at 254 nm): t_R = 4.52 min (minor), t_R = 5.18 min (major); $[\alpha]_D^{20}$ = – 583.286 (*c* = 0.14, EA).

NMR and HRMS data for the product **3ac**:

^{1}H NMR (600 MHz, Chloroform-*d*) δ (ppm): 7.57 (d, *J* = 7.2 Hz, 1H), 7.49 (d, *J* = 8.4 Hz, 2H), 7.43 (s, 2H), 7.30 (t, *J* = 7.8 Hz, 1H), 7.22 (m, 4H), 7.28 (t, *J* = 7.8 Hz, 1H), 7.13 – 7.08 (m, 2H), 7.03 – 7.00 (m, 3H), 6.88 (t, *J* = 7.2 Hz, 2H), 6.76 (d, *J* = 7.8 Hz, 1H), 6.59 (s, 1H), 5.34 (s, 1H), 5.05 (d, *J* = 15.6 Hz, 1H), 4.51 (d, *J* = 15.6 Hz, 1H), 3.47 (d, *J* = 12.0 Hz, 1H), 2.66 (s, 3H), 2.41 (d, *J* = 7.8 Hz, 1H).

^{13}C NMR (150 MHz, Chloroform-*d*) δ (ppm): 174.4, 169.5, 158.1, 155.2 (q, *J* = 37.5 Hz), 142.3, 137.2, 134.7, 134.5, 134.2, 130.7, 130.0, 129.8, 129.5, 129.4, 128.6, 128.5, 128.0, 127.5, 126.8, 126.8, 125.1, 124.3, 123.5, 119.5, 119.2, 115.5 (q, *J* = 287.1 Hz), 109.4, 59.2, 50.9, 44.1, 42.4, 34.0, 19.8.

^{19}F NMR (564 MHz, Chloroform-*d*) δ (ppm): −75.58.

HRMS (ESI-TOF) m/z: $[M+Na]^+$ Calcd for $C_{37}H_{28}ClF_3N_4NaO_3^+$ 691.1694, Found: 691.1686.

<u>*N*-((3*R*,5'*S*,6'*S*)-1-benzyl-5'-(2-fluorophenyl)-3''-methyl-2,5''-dioxo-1''-phenyl-1'',5''-dihydrodi spiro[indoline-3,1'-cyclohexene-6',4''-pyrazol]-3'-yl)-2,2,2-trifluoroacetamide (3ad)</u>

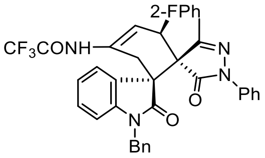

Prepared according to the general procedure, the crude product was purified by silica gel chromatography (petroleum ether/ethyl acetate 20:1) to afford **3ad** (52.3 mg, 80% yield, white solid, > 20:1 dr, m.p: 272.7 – 275.9 °C).

The enantiomeric excess was determined to be 99% by HPLC with a Daicel Chiralpak IG (*n*-hexane/2-propanol = 70:30, 1.0 mL/min, at 254 nm): t_R = 6.07 min (minor), t_R = 9.52 min (major); $[\alpha]_D^{20}$ = – 357.091 (*c* = 0.11, EA).

NMR and HRMS data for the product **3ad**:

^{1}H NMR (600 MHz, Chloroform-*d*) δ (ppm): 7.54 (d, *J* = 7.2 Hz, 1H), 7.50 (d, *J* = 7.2 Hz, 2H), 7.44 (s, 1H), 7.32 – 7.27 (m, 2H), 7.23 (t, *J* = 7.8 Hz, 2H), 7.20 – 7.14 (m, 2H), 7.10 (t, *J* = 7.8 Hz, 1H), 7.04 – 6.98 (m, 4H), 6.99 – 6.87 (m, 3H), 6.76 – 6.75 (m, 2H), 5.06 (d, *J* = 16.2 Hz, 1H), 4.84 (s, 1H), 4.49 (d, *J* = 15.6 Hz, 1H), 3.52 (d, *J* = 17.4 Hz, 1H), 2.61 (s, 3H), 2.35 (d, *J* = 18.0 Hz, 1H).

^{13}C NMR (150 MHz, Chloroform-*d*) δ (ppm): 174.3, 169.6, 160.7 (d, J_{CF}= 248.0 Hz), 158.7, 155.3 (q, *J* = 37.4 Hz), 142.2, 137.2, 134.8, 131.1 (d, J_{CF} = 3.5 Hz), 130.2 (d, J_{CF}= 8.6 Hz), 129.9, 129.5, 129.3, 128.7, 128.5, 127.5, 126.8, 125.1, 124.4, 124.2 (d, J_{CF} = 3.3 Hz), 123.5, 123.2 (d, J_{CF} = 12.9 Hz), 119.4, 118.5, 116.0 (d, J_{CF} = 22.8 Hz), 155.5 (q, *J* = 287.1 Hz), 109.4, 59.6, 50.9, 44.1, 41.2, 33.9, 18.9.

^{19}F NMR (564 MHz, Chloroform-*d*) δ (ppm): −75.57, −112.87.

HRMS (ESI-TOF) m/z: $[M+Na]^+$ Calcd for $C_{37}H_{28}F_4N_4NaO_3^+$ 675.1990, Found: 675.1994.

<u>*N*-((3*R*,5'*S*,6'*S*)-1-benzyl-5'-(2-methoxyphenyl)-3''-methyl-2,5''-dioxo-1''-phenyl-1'',5''-dihydrodi spiro[indoline-3,1'-cyclohexene-6',4''-pyrazol]-3'-yl)-2,2,2-trifluoroacetamide (3ae)</u>

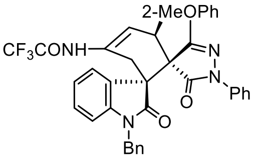

Prepared according to the general procedure, the crude product was purified by silica gel chromatography (petroleum ether/ethyl acetate 20:1) to afford **3ae** (60.5 mg, 91% yield, white solid, > 20:1 dr, m.p: 196.2 – 196.6 °C).

The enantiomeric excess was determined to be 99% by HPLC with a Daicel Chiralpak IG (*n*-hexane/2-propanol = 70:30, 1.0 mL/min, at 254 nm): tR = 5.06 min (minor), tR = 5.67 min (major); $[\alpha]_D^{20}$ = – 431.200 (*c* = 0.10, EA).

NMR and HRMS data for the product **3ae**:

^{1}H NMR (600 MHz, Chloroform-*d*) δ (ppm):7.58 – 7.56 (m, 3H), 7.40 (s, 1H), 7.38 (s, 1H), 7.31 (t, *J* = 7.8 Hz, 1H), 7.22 (t, *J* = 7.2 Hz, 1H), 7.19 – 7.15 (m, 2H), 7.09 – 7.05 (m, 2H), 7.04 (d, *J* = 7.8 Hz, 2H), 6.94 (t, *J* = 7.8 Hz, 2H), 6.88 (t, *J* = 7.8 Hz, 1H), 6.76 (d, *J* = 7.8 Hz, 1H), 6.70 (s, 1H), 6.61 (d, *J* = 7.8 Hz, 1H), 5.07 (d, *J* = 15.6 Hz, 1H), 4.96 (s, 1H), 4.43 (d, *J* = 15.6 Hz, 1H), 3.50 (d, *J* = 17.4 Hz, 1H), 3.35 (s, 3H), 2.51 (s, 3H), 2.29 (d, *J* = 10.2 Hz, 1H).

^{13}C NMR (150 MHz, Chloroform-*d*) δ (ppm): 174.6, 159.2, 157.1, 155.2 (q, *J* = 37.4 Hz), 142.2, 137.7, 134.9, 130.5, 129.4, 129.1, 128.8, 128.7, 128.7, 128.4, 127.6, 126.9, 124.7, 124.6, 124.5, 123.4, 120.9, 120.2, 118.9, 115.5 (q, *J* = 287.1 Hz), 110.3, 109.3, 59.3, 54.8, 51.3, 44.0, 40.4, 34.1, 19.3.

^{19}F NMR (564 MHz, Chloroform-*d*) δ (ppm): −75.56.

HRMS (ESI-TOF) m/z: $[M+Na]^+$ Calcd for $C_{38}H_{31}F_3N_4NaO_4^+$ 687.2190, Found: 687.2194.

***N*-((3*R*,5'*S*,6'*S*)-1-benzyl-5'-(2-(allyloxy)phenyl))-3''-methyl-2,5''-dioxo-1''-phenyl-1'',5''-dihydrodispiro[indoline-3,1'-cyclohexene-6',4''-pyrazol]-3'-yl)-2,2,2-trifluoroacetamide (3af)**

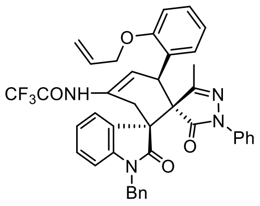

Prepared according to the general procedure, the crude product was purified by silica gel chromatography (petroleum ether/ethyl acetate 20:1) to afford **3af** (64.0 mg, 93% yield, white solid, > 20:1 dr, m.p: 150.0 – 150.2 °C).

The enantiomeric excess was determined to be 95% by HPLC with a Daicel Chiralpak IC (*n*-hexane/2-propanol = 80:20, 1.0 mL/min, at 254 nm): t_R = 4.28 min (minor), t_R = 4.82 min (major); $[\alpha]_D^{20}$ = 244.800 (*c* = 0.08, EA).

NMR and HRMS data for the product **3af***:*

^{1}H NMR (600 MHz, Chloroform-*d*) δ (ppm): 7.56 (d, *J* = 7.2 Hz, 1H), 7.51 (d, *J* = 7.2 Hz, 2H), 7.37 (s, 1H), 7.34 (t, *J* = 7.2 Hz, 2H), 7.21 (t, *J* = 7.2 Hz, 2H), 7.17 – 7.11 (m, 2H), 7.09 – 7.06 (m, 2H), 7.05 (d, *J* = 7.2 Hz, 2H), 6.94 (t, *J* = 7.8 Hz, 2H), 6.84 (t, *J* = 7.8 Hz, 1H), 6.77 (d, *J* = 8.4 Hz, 1H), 6.71 (s, 1H), 6.66 (d, *J* = 7.2 Hz, 1H), 5.87 – 5.81 (m, 1H), 5.21 (dd, *J* = 17.4, 1.8 Hz, 1H), 5.11 (d, *J* = 10.8 Hz, 1H), 5.05 – 5.03 (m, 2H), 4.51 (d, *J* = 15.6 Hz, 1H), 4.23 (d, *J* = 4.8 Hz, 2H), 3.50 (d, *J* = 18.0 Hz, 1H), 2.58 (s, 3H), 2.31 (s, 1H).

^{13}C NMR (150 MHz, Chloroform-*d*) δ (ppm): 174.6, 159.1, 156.3, 155.1 (q, *J* = 37.2 Hz), 142.2, 137.6, 135.0, 133.3, 130.8, 130.4, 129.3, 129.1, 128.7, 128.5, 128.4, 127.5, 126.9, 124.8, 124.7, 124.6, 123.3,

120.7, 120.4, 119.2, 119.1, 117.2, 115.5 (q, J = 287.1 Hz), 112.0, 109.2, 69.1, 59.6, 51.0, 44.0, 40.7, 34.0, 19.5.

^{19}F NMR (564 MHz, Chloroform-*d*) δ (ppm): -75.86.

HRMS (ESI-TOF) m/z: $[M+Na]^+$ Calcd for $C_{40}H_{33}F_3N_4NaO_4^+$ 713.2346, Found: 713.2349.

<u>*N*-((3*R*,5'*S*,6'*S*)-1-benzyl-5'-(3-ethynylphenyl)-3''-methyl-2,5''-dioxo-1''-phenyl-1'',5''-dihydrodispiro[indoline-3,1'-cyclohexene-6',4''-pyrazol]-3'-yl)-2,2,2-trifluoroacetamide (3ag)</u>

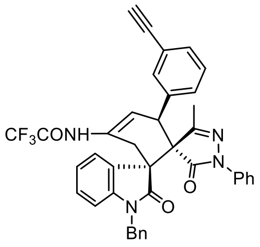

Prepared according to the general procedure, the crude product was purified by silica gel chromatography (petroleum ether/ethyl acetate 20:1) to afford **3ag** (60.0 mg, 91% yield, white solid, 14:1 dr, m.p: 251.2 – 251.9 °C).

The enantiomeric excess was determined to be 96% by HPLC with a Daicel Chiralpak IG (*n*-hexane/2-propanol = 70:30, 1.0 mL/min, at 254 nm): t_R = 7.73 min (minor), t_R = 10.61 min (major); $[\alpha]_D^{20}$ = – 236.800 (c = 0.05, EA).

NMR and HRMS data for the product **3ag***:*

^{1}H NMR (600 MHz, Chloroform-*d*) δ (ppm): 7.53 – 7.50 (m, 3H), 7.44 (s, 1H), 7.37 (s, 1H), 7.32 (t, J = 7.8 Hz, 1H), 7.29 (d, J = 7.2 Hz, 1H), 7.24 (d, J = 7.8 Hz, 2H), 7.19 (t, J = 7.8 Hz, 1H), 7.14 – 7.09 (m, 3H), 7.04 (t, J = 7.8 Hz, 1H), 7.02 (d, J = 7.2 Hz, 2H), 6.88 (t, J = 7.8 Hz, 2H), 6.82 (s, 1H), 6.77 (d, J = 7.8 Hz, 1H), 5.08 (d, J = 15.6 Hz, 1H), 4.57 (s, 1H), 4.48 (d, J = 15.6 Hz, 1H), 3.51 (d, J = 17.4 Hz, 1H), 2.97 (s, 1H), 2.54 (s, 3H), 2.37 (d, J = 17.4 Hz, 1H).

^{13}C NMR (150 MHz, Chloroform-*d*) δ (ppm): 174.2, 170.1, 158.5, 155.3 (q, J = 37.2 Hz), 142.3, 137.1, 136.5, 134.8, 131.9, 131.8, 130.7, 129.7, 129.4, 129.0, 128.7, 128.6, 128.1, 127.6, 126.8, 125.3, 124.5, 123.4, 122.6, 119.7, 117.6, 115.5 (q, J = 287.1 Hz), 109.5, 82.9, 77.6, 60.2, 51.0, 45.4, 44.1, 33.9, 18.8.

^{19}F NMR (564 MHz, Chloroform-*d*) δ (ppm): -75.55.

HRMS (ESI-TOF) m/z: $[M+Na]^+$ Calcd for $C_{39}H_{29}F_3N_4NaO_3^+$ 681.2084, Found: 681.2085.

<u>*N*-((3*R*,5'*S*,6'*S*)-1-benzyl-3''-methyl-2,5''-dioxo-1''-phenyl-5'-(*m*-tolyl)-1'',5''-dihydrodispiro[indoline-3,1'-cyclohexene-6',4''-pyrazol]-3'-yl)-2,2,2-trifluoroacetamide (3ah)</u>

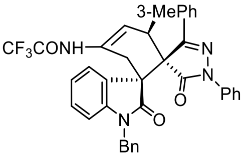

Prepared according to the general procedure, the crude product was purified by silica gel chromatography (petroleum ether/ethyl acetate 20:1) to afford **3ah** (58.6 mg, 90% yield, white solid, > 20:1 dr, m.p: 212.2 – 214.9 °C).

The enantiomeric excess was determined to be 99% by HPLC with a Daicel Chiralpak IG (*n*-hexane/2-propanol = 90:10, 1.0 mL/min, at 254 nm): t_R = 30.09 min (minor), t_R = 32.91 min (major); $[\alpha]_D^{20}$ = –494.000 (*c* = 0.10, EA).

NMR and HRMS data for the product **3ah**:

^{1}H NMR (600 MHz, Chloroform-*d*) δ (ppm): 7.54 – 7.52 (m, 3H), 7.40 (s, 1H), 7.32 (t, *J* = 7.8 Hz, 1H), 7.24 (t, *J* = 7.8 Hz, 2H), 7.19 (t, *J* = 7.8 Hz, 1H), 7.11 (t, *J* = 7.2 Hz, 1H), 7.06 (t, *J* = 7.8 Hz, 1H), 7.05 – 7.02 (m, 4H), 6.98 – 6.94 (m, 2H), 6.89 (t, *J* = 7.8 Hz, 2H), 6.84 (s, 1H), 6.76 (d, *J* = 7.8 Hz, 1H), 5.07 (d, *J* = 15.6 Hz, 1H), 4.55 (s, 1H), 4.47 (d, *J* = 15.6 Hz, 1H), 3.50 (d, *J* = 18.0 Hz, 1H), 2.52 (s, 3H), 2.33 (d, *J* = 18.0 Hz, 1H), 2.07 (s, 3H).

^{13}C NMR (150 MHz, Chloroform-*d*) δ (ppm): 174.3, 170.5, 158.9, 155.3 (q, *J* = 37.4 Hz), 142.3, 138.2, 137.3, 135.9, 134.8, 130.0, 129.9, 129.3, 129.0, 128.8, 128.7, 128.5, 128.4, 127.5, 126.8, 125.1, 124.9, 124.5, 123.4, 119.4, 118.7, 115.5 (q, *J* = 287.1 Hz), 109.4, 60.4, 51.0, 45.5, 44.1, 34.0, 21.1, 18.8.

^{19}F NMR (564 MHz, Chloroform-*d*) δ (ppm): −75.55.

HRMS (ESI-TOF) m/z: $[M+Na]^+$ Calcd for $C_{38}H_{31}F_3N_4NaO_3^+$ 671.2240, Found: 671.2231.

<u>*N*-((3*R*,5'*S*,6'*S*)-1-benzyl-5'-(3-fluorophenyl)-3''-methyl-2,5''-dioxo-1''-phenyl-1'',5''-dihydrodispiro[indoline-3,1'-cyclohexene-6',4''-pyrazol]-3'-yl)-2,2,2-trifluoroacetamide (3ai)</u>

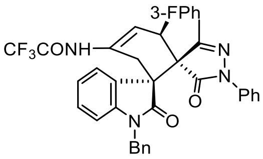

Prepared according to the general procedure, the crude product was purified by silica gel chromatography (petroleum ether/ethyl acetate 20:1) to afford **3ai** (59.0 mg, 90% yield, white solid, 15:1 dr, m.p: 207.3 – 208.4 °C).

The enantiomeric excess was determined to be 99% by HPLC with a Daicel Chiralpak IG (*n*-hexane/2-propanol = 70:30, 1.0 mL/min, at 254 nm): t_R = 6.80 min (minor), t_R = 8.86 min (major); $[\alpha]_D^{20}$ = + 610.000 (*c* = 0.10, EA).

NMR and HRMS data for the product **3ai**:

^{1}H NMR (600 MHz, Chloroform-*d*) δ (ppm): 7.53 – 7.51 (m, 3H), 7.49 (s, 1H), 7.33 (t, *J* = 7.8 Hz, 1H), 7.27 – 7.24 (m, 2H), 7.19 (t, *J* = 7.8 Hz, 1H), 7.12 (q, *J* = 7.8 Hz, 2H), 7.04 (t, *J* = 7.2 Hz, 1H), 7.01 (d, *J* = 7.2 Hz, 2H), 6.96 – 6.94 (m, 2H), 6.88 (t, *J* = 7.8 Hz, 3H), 6.85 (s, 1H), 6.77 (d, *J* = 8.4 Hz, 1H), 5.06 (d, *J* = 15.6 Hz, 1H), 4.58 (s, 1H), 4.47 (d, *J* = 15.6 Hz, 1H), 3.48 (d, *J* = 18.0 Hz, 1H), 2.53 (s, 3H), 2.32 (d, *J* = 18.0 Hz, 1H).

^{13}C NMR (150 MHz, Chloroform-*d*) δ (ppm): 174.1, 170.1, 162.6 (d, J_{CF} = 245.6 Hz), 158.5, 155.3 (q, *J* = 37.5 Hz), 142.2, 138.7 (d, J_{CF} = 6.8 Hz), 137.1, 134.7, 134.3, 130.5, 130.1 (d, *J* = 8.3 Hz), 129.6, 129.4, 128.7, 128.6, 127.6, 126.8, 125.3, 124.7, 123.9 (d, *J* = 2.7 Hz), 123.5, 119.4, 117.6, 115.5 (q, *J* = 287.1 Hz), 115.3 (d, J_{CF} = 3.3 Hz), 115.2 (d, J_{CF} = 1.4 Hz), 60.3, 51.1, 45.3, 44.1, 34.0, 18.7.

^{19}F NMR (564 MHz, Chloroform-*d*) δ (ppm): −75.57, −111.97 – −112.01.

HRMS (ESI-TOF) m/z: $[M+Na]^+$ Calcd for $C_{37}H_{28}F_4N_4NaO_3^+$ 675.1990, Found: 675.1982.

N-((3*R*,5'*S*,6'*S*)-1-benzyl-5'-(3-chlorophenyl)-3''-methyl-2,5''-dioxo-1''-phenyl-1'',5''-dihydrodispiro[indoline-3,1'-cyclohexene-6',4''-pyrazol]-3'-yl)-2,2,2-trifluoroacetamide (3aj)

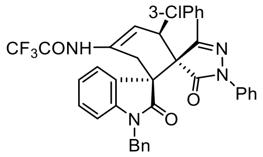

Prepared according to the general procedure, the crude product was purified by silica gel chromatography (petroleum ether/ethyl acetate 20:1) to afford **3aj** (60.6 mg, 90% yield, white solid, 15:1 dr, m.p: 254.3 – 258.8 °C).

The enantiomeric excess was determined to be 99% by HPLC with a Daicel Chiralpak IB (*n*-hexane/2-propanol = 80:20, 1.0 mL/min, at 254 nm): t_R = 6.03 min (major), t_R = 6.88 min (minor); $[\alpha]D^{20}$ = – 725.000 (*c* = 0.10, EA).

NMR and HRMS data for the product **3aj**:

^{1}H NMR (600 MHz, Chloroform-*d*) δ (ppm): 7.53 – 7.51 (m, 3H), 7.44 (s, 1H), 7.33 – 7.31 (m, 1H), 7.27 – 7.24 (m, 2H), 7.22 (t, *J* = 1.8 Hz, 1H), 7.20 – 7.17 (m, 1H), 7.16 – 7.12 (m, 2H), 7.09 (t, *J* = 7.8 Hz, 1H), 7.07 – 7.03 (m, 2H), 7.01 (d, *J* = 7.8 Hz, 2H), 6.88 (t, *J* = 7.8 Hz, 2H), 6.83 (s, 1H), 6.77 (d, *J* = 7.8 Hz, 1H), 5.08 (d, *J* = 15.6 Hz, 1H), 4.57 (s, 1H), 4.48 (d, *J* = 15.6 Hz, 1H), 3.52 – 3.48 (m, 1H), 2.53 (s, 3H), 2.35 (d, *J* = 17.4 Hz, 1H).

^{13}C NMR (150 MHz, Chloroform-*d*) δ (ppm): 174.1, 170.1, 158.5, 155.3 (q, *J* = 37.5 Hz), 142.3, 138.2, 137.1, 134.7, 134.5, 130.7, 129.8, 129.6, 129.4, 128.7, 128.6, 128.4, 128.4, 127.6, 126.8, 126.3, 125.4, 124.4, 123.5, 119.6, 117.4, 115.5 (q, *J* = 287.0 Hz), 109.5, 60.2, 51.0, 45.3, 44.1, 34.0, 18.7.

^{19}F NMR (564 MHz, Chloroform-*d*) δ (ppm): −75.55.

HRMS (ESI-TOF) m/z: $[M+Na]^+$ Calcd for $C_{37}H_{28}ClF_3N_4NaO_3^+$ 691.1694, Found: 691.1699.

<u>*N*-((3*R*,5'*S*,6'*S*)-1-benzyl-5'-(3-bromophrnyl)-3''-methyl-2,5''-dioxo-1''-phenyl-1'',5''-dihydrodispiro[indoline-3,1'-cyclohexene-6',4''-pyrazol]-3'-yl)-2,2,2-trifluoroacetamide (3ak)</u>

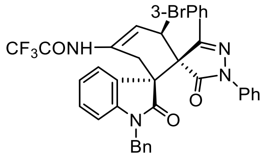

Prepared according to the general procedure, the crude product was purified by silica gel chromatography (petroleum ether/ethyl acetate 20:1) to afford **3ak** (69.5 mg, 97% yield, white solid, > 20:1 dr, m.p: 255.6 – 256.9 °C).

The enantiomeric excess was determined to be 99% by HPLC with a Daicel Chiralpak IB (*n*-hexane/2-propanol = 80:20, 1.0 mL/min, at 254 nm**)**: t_R = 6.08 min (major), t_R = 6.93 min (minor); $[\alpha]_D^{20}$ = – 686.235 (*c* = 0.17, EA).

NMR and HRMS data for the product **3ak**:

^{1}H NMR (600 MHz, Chloroform-*d*) δ (ppm): 7.53 – 7.51 (m, 3H), 7.46 (s, 1H), 7.36 (s, 1H), 7.34 – 7.29 (m, 2H), 7.28 – 7.25 (m, 2H), 7.19 (t, *J* = 7.8 Hz, 1H), 7.14 – 7.11 (m, 2H), 7.04 (t, *J* = 7.8 Hz, 2H) , 7.01 (d, *J* = 7.2 Hz, 2H), 6.88 (t, *J* = 7.8 Hz, 2H), 6.82 (s, 1H), 6.77 (d, *J* = 7.8 Hz, 1H), 5.08 (d, *J* = 15.6 Hz, 1H), 4.56 (s, 1H), 4.48 (d, *J* = 16.2 Hz, 1H), 3.49 (d, *J* = 18.0 Hz, 1H), 2.53 (s, 3H), 2.35 (d, *J* = 18.0 Hz, 1H).

^{13}C NMR (150 MHz, Chloroform-*d*) δ (ppm): 174.1, 170.0, 158.5, 155.3 (q, *J* = 37.4 Hz), 142.3, 138.5, 137.1, 134.7, 131.4, 131.3, 130.7, 130.1, 129.6, 129.4, 128.7, 128.6, 127.6, 126.8, 126.7, 125.4, 124.4, 123.5, 122.7, 119.7, 117.3, 115.5 (q, *J* = 287.3 Hz), 109.5, 60.2, 51.0, 45.3, 44.1, 33.9, 18.7.

^{19}F NMR (564 MHz, Chloroform-*d*) δ (ppm): −75.54.

HRMS (ESI-TOF) m/z: $[M+Na]^+$ Calcd for $C_{37}H_{28}BrF_3N_4NaO_3^+$ 735.1189, Found: 735.1186.

<u>*N*-((3*R*,5'*S*,6'*S*)-1-benzyl-3''-methyl-2,5''-dioxo-1''-phenyl-5'-(*p*-tolyl)-1'',5''-dihydrodispiro[indoline-3,1'-cyclohexene-6',4''-pyrazol]-3'-yl)-2,2,2-trifluoroacetamide (3al)</u>

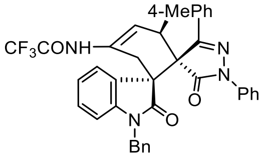

Prepared according to the general procedure, the crude product was purified by silica gel chromatography (petroleum ether/ethyl acetate 20:1) to afford **3al** (64.3 mg, 99% yield, white solid, 14:1 dr, m.p: 252.1 – 255.5 °C).

The enantiomeric excess was determined to be 99% by HPLC with a Daicel Chiralpak IG (*n*-hexane/2-propanol = 80:20, 1.0 mL/min, at 254 nm): t_R = 22.22 min (minor), t_R = 25.52 min (major); $[\alpha]_D^{20}$ = –694.545 (*c* = 0.11, EA).

NMR and HRMS data for the product **3al**:

^{1}H NMR (600 MHz, Chloroform-*d*) δ (ppm): 7.54 (t, *J* = 6.6 Hz, 3H), 7.38 (s, 1H), 7.31 (t, *J* = 7.8 Hz, 1H), 7.26 – 7.24 (m, 2H), 7.18 (t, *J* = 7.2 Hz, 1H), 7.12 (t, *J* = 7.8 Hz, 1H), 7.07 (d, *J* = 7.8 Hz, 2H), 7.04 (t, *J* = 7.8 Hz, 1H), 7.01 (d, *J* = 7.2 Hz, 2H), 6.96 (d, *J* = 7.8 Hz, 2H), 6.87 (t, *J* = 7.2 Hz, 2H), 6.83 (s, 1H), 6.76 (d, *J* = 7.8 Hz, 1H), 5.07 (d, *J* = 15.6 Hz, 1H), 4.56 (s, 1H), 4.46 (d, *J* = 16.2 Hz, 1H), 3.50 (d, *J* = 17.4 Hz, 1H), 2.53 (s, 3H), 2.32 (d, *J* = 17.4 Hz, 1H), 2.21 (s, 3H).

^{13}C NMR (150 MHz, Chloroform-*d*) δ (ppm): 174.3, 170.4, 158.9, 155.3 (q, *J* = 37.4 Hz), 142.3, 137.8, 137.4, 134.8, 132.9, 129.9, 129.9, 129.3, 129.3, 128.7, 128.5, 128.0, 127.5, 126.8, 125.1, 124.6, 123.4, 119.5, 119.0, 115.5 (q, *J* = 287.1 Hz), 109.4, 60.4, 51.2, 45.2, 44.1, 34.1, 21.0, 18.8.

^{19}F NMR (564 MHz, Chloroform-*d*) δ (ppm): −75.58.

HRMS (ESI-TOF) m/z: $[M+Na]^+$ Calcd for $C_{38}H_{31}F_3N_4NaO_3^+$ 671.2240, Found: 671.2236.

***N*-((3*R*,5'*S*,6'*S*)-1-benzyl-3''-methyl-2,5''-dioxo-1''-phenyl-5'-(4-((trimethylsilyl)ethynyl)phenyl)-1'',5''-dihydrodispiro[indoline-3,1'-cyclohexene-6',4''-pyrazol]-3'-yl)-2,2,2-trifluoroacetamide (3am)**

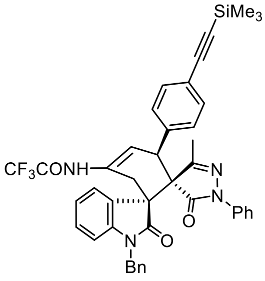

Prepared according to the general procedure, the crude product was purified by silica gel chromatography (petroleum ether/ethyl acetate 20:1) to afford **3am** (68.8 mg, 94% yield, white solid, 15:1 dr, m.p: 271.5 – 272.0 °C).

The enantiomeric excess was determined to be 98% by HPLC with a Daicel Chiralpak IG (*n*-hexane/2-propanol = 70:30, 1.0 mL/min, at 254 nm): t_R = 3.75 min (minor), t_R = 7.74 min (major); $[\alpha]_D^{20}$ = – 176.308 (*c* = 0.10, EA).

NMR and HRMS data for the product **3am**:

^{1}H NMR (600 MHz, Chloroform-*d*) δ (ppm):7.56 (d, *J* = 7.8 Hz, 2H), 7.52 (d, J = 7.2 Hz, 1H), 7.37 (s, 1H), 7.32 (t, *J* = 7.8 Hz, 1H), 7.28 – 7.25 (m, 4H), 7.18 (t, *J* = 7.8 Hz, 1H), 7.14 – 7.12 (m, 3H), 7.03 (t, *J* = 7.2 Hz, 1H), 7.00 (d, *J* = 7.8 Hz, 2H), 6.87 (t, *J* = 7.2 Hz, 2H), 6.84 (s, 1H), 6.76 (d, *J* = 7.2 Hz, 1H), 5.07 (d, *J* = 15.6 Hz, 1H), 4.58 (s, 1H), 4.45 (d, *J* = 15.6 Hz, 1H), 3.48 (d, *J* = 18.0, Hz, 1H), 2.50 (s, 3H), 2.32 (d, *J* = 18.0 Hz, 1H), 0.20 (s, 9H).

^{13}C NMR (150 MHz, Chloroform-*d*) δ (ppm): 174.1, 170.1, 158.4, 155.3 (q, *J* = 37.7 Hz), 142.3, 137.3, 136.6, 134.7, 132.1, 130.3, 129.7, 129.4, 128.7, 128.6, 128.0, 127.6, 126.8, 125.2, 124.5, 123.4, 122.9, 119.3,118.0, 115.5 (q, *J* = 287.1 Hz), 109.5, 104.5, 95.0, 60.3, 51.1, 45.3, 44.1, 34.0, 18.7, 0.1.

^{19}F NMR (564 MHz, Chloroform-*d*) δ (ppm): -75.57.

HRMS (ESI-TOF) m/z: $[M+Na]^+$ Calcd for $C_{42}H_{37}F_3N_4NaO_3Si^+$ 753.2479, Found: 753.2478.

<u>*N*-((3*R*,5'*S*,6'*S*)-1-benzyl-5'-(4-fluorophenyl)-3''-methyl-2,5''-dioxo-1''-phenyl-1'',5''-dihydrodi spiro[indoline-3,1'-cyclohexene-6',4''-pyrazol]-3'-yl)-2,2,2-trifluoroacetamide (3an)</u>

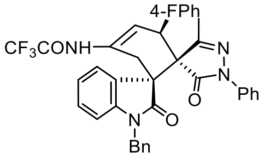

Prepared according to the general procedure, the crude product was purified by silica gel chromatography (petroleum ether/ethyl acetate 20:1) to afford **3an** (61.5 mg, 94% yield, white solid, 12:1 dr, m.p: 248.3 – 249.2 °C).

The enantiomeric excess was determined to be 99% by HPLC with a Daicel Chiralpak IG (*n*-hexane/2-propanol = 70:30, 1.0 mL/min, at 254 nm): t_R = 5.66 min (minor), t_R = 10.68 min (major); $[\alpha]_D^{20}$ = – 731.400 (*c* = 0.10, EA).

NMR and HRMS data for the product **3an**:

^{1}H NMR (600 MHz, Chloroform-*d*) δ (ppm): 7.53 (t, *J* = 6.6 Hz, 3H), 7.39 (s, 1H), 7.33 (t, *J* = 7.8 Hz, 1H), 7.27 – 7.24 (m, 2H), 7.20 – 7.15 (m, 3H), 7.12 (t, *J* = 7.8 Hz, 1H), 7.05 (t, *J* = 7.8 Hz, 1H), 7.02 (d, *J* = 7.8 Hz, 2H), 6.90 – 6.84 (m, 5H), 6.78 (d, *J* = 7.8 Hz, 1H), 5.08 (d, *J* = 15.6 Hz, 1H), 4.57 (s, 1H), 4.47 (d, *J* = 15.6 Hz, 1H), 3.48 (d, *J* = 18.0 Hz, 1H), 2.53 (s, 3H), 2.32 (d, *J* = 18.0 Hz, 1H).

^{13}C NMR (150 MHz, Chloroform-*d*) δ (ppm): 174.2, 170.2, 162.4 (d, J_{CF} = 246.0 Hz), 158.6, 155.4 (q, J = 37.4 Hz), 142.3, 137.2, 134.8, 131.9 (d, J = 3.2 Hz), 130.2, 129.8 (d, J = 8.1 Hz), 129.7, 129.4, 128.7, 128.6, 127.6, 126.8, 125.3, 124.5, 123.4, 119.3,118.4, 115.5 (q, J = 287.1 Hz), 115.5 (d, J = 21.3 Hz), 109.5, 60.4, 51.0, 44.9, 44.1, 34.1, 18.8.

^{19}F NMR (564 MHz, Chloroform-*d*) δ (ppm): −75.59, −113.59 – −113.64.

HRMS (ESI-TOF) m/z: $[M+Na]^+$ Calcd for $C_{37}H_{28}F_4N_4NaO_3^+$ 675.1990, Found: 675.1986.

methyl 4-((3*R*,6'*S*,1'*S*)-1-benzyl-3''-methyl-2,5''-dioxo-1''-phenyl-4'-(2,2,2-trifluoroacetamido)-1'',5''-dihydrodispiro[indoline-3,2'-cyclohexene-1',4''-pyrazol]-6'-yl)benzoate (3ao)

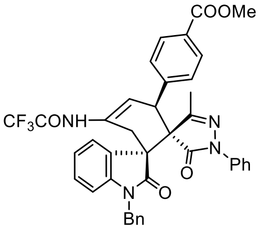

Prepared according to the general procedure, the crude product was purified by silica gel chromatography (petroleum ether/ethyl acetate 20:1) to afford **3ao** (62.4 mg, 90% yield, white solid, 14:1 dr, m.p: 235.5 – 236.0 °C).

The enantiomeric excess was determined to be 92% by HPLC with a Daicel Chiralpak IC (*n*-hexane/2-propanol = 70:30, 1.0 mL/min, at 254 nm): t_R = 5.97 min (major), t_R = 7.07 min (minor); $[\alpha]_D^{20}$ = – 278.800 (*c* = 0.05, EA).

NMR and HRMS data for the product **3ao**:

^{1}H NMR (600 MHz, Chloroform-*d*) δ (ppm): 7.84 (d, J = 7.8 Hz, 2H), 7.54 – 7.52 (m, 3H), 7.42 (s, 1H), 7.33 (t, J = 7.8, 1H), 7.28 – 7.23 (m, 4H), 7.19 (t, J = 7.8, 1H), 7.12 (t, J = 7.8 Hz, 1H), 7.04 (t, J = 7.8 Hz, 1H), 7.01 (d, J = 7.8 Hz, 2H), 6.89 – 6.86 (m, 3H), 6.78 (d, J = 7.8 Hz, 1H), 5.08 (d, J = 15.6 Hz, 1H), 4.65 (s, 1H), 4.47 (d, J = 15.6 Hz, 1H), 3.84 (s, 3H), 3.50 (d, J = 17.4 Hz, 1H), 2.53 (s, 3H), 2.35 (d, J = 17.4 Hz, 1H).

^{13}C NMR (150 MHz, Chloroform-*d*) δ (ppm): 174.1, 170.0, 166.5, 158.4, 155.4 (q, J = 37.9 Hz), 142.3, 141.5, 137.1, 134.7, 130.5, 129.9, 129.8, 129.6, 129.5, 128.7, 128.6, 128.3, 127.6, 126.8, 125.3, 124.5, 123.5, 119.3, 117.6, 115.5 (q, J = 288.0 Hz), 109.5, 60.8, 52.1, 51.1, 45.5, 44.2, 34.0, 18.7.

^{19}F NMR (564 MHz, Chloroform-*d*) δ (ppm): -75.57.

HRMS (ESI-TOF) m/z: $[M+Na]^+$ Calcd for $C_{39}H_{31}F_3N_4NaO_5^+$ 715.2139, Found: 715.2133.

N-((3R,5'S,6'S)-1-benzyl-3''-methyl-5'-(naphthalen-2-yl)-2,5''-dioxo-1''-phenyl-1'',5''-dihydrodi spiro[indoline-3,1'-cyclohexene-6',4''-pyrazol]-3'-yl)-2,2,2-trifluoroacetamide (3ap)

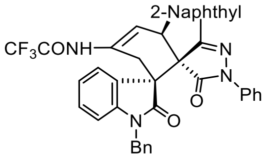

Prepared according to the general procedure, the crude product was purified by silica gel chromatography (petroleum ether/ethyl acetate 20:1) to afford **3ap** (67.2 mg, 98% yield, white solid, 15:1 dr, m,p: 212.5 – 214.9 °C).

The enantiomeric excess was determined to be 99% by HPLC with a Daicel Chiralpak IG (*n*-hexane/2-propanol = 70:30, 1.0 mL/min, at 254 nm): t_R = 8.35 min (minor), t_R = 17.42 min (major); $[\alpha]_D^{20}$ = – 309.231 (*c* = 0.13, EA).

NMR and HRMS data for the product **3ap**:

^{1}H NMR (600 MHz, DMSO-d_6) δ (ppm): 10.85 (s, 1H), 7.82 – 7.80 (m, 1H), 7.78 – 7.76 (m, 1H), 7.74 (d, *J* = 8.4 Hz, 2H), 7.61 (d, *J* = 7.2 Hz, 1H), 7.46 – 7.43 (m, 2H), 7.40 (d, *J* = 7.8 Hz, 2H), 7.34 – 7.32 (m, 1H), 7.29 (t, *J* = 7.8 Hz, 2H), 7.24 – 7.22 (m, 1H), 7.18 (t, *J* = 7.8 Hz, 1H), 7.14 (t, *J* = 7.8 Hz, 1H), 7.06 – 7.03 (m, 3H), 6.90 (d, *J* = 8.4 Hz, 1H), 6.84 (t, *J* = 7.8 Hz, 2H), 6.73 (s, 1H), 5.01 (d, *J* = 16.2 Hz, 1H), 4.69 (s, 1H), 4.61 (d, *J* = 16.2 Hz, 1H), 3.33 (s, 1H), 2.56 (d, *J* = 18.0 Hz, 1H), 2.47 (s, 3H).

^{13}C NMR (150 MHz, DMSO-d_6) δ (ppm): 174.5, 170.8, 159.7, 155.5 (q, *J* = 36.9 Hz), 142.4, 137.4, 136.2, 135.0, 133.1, 132.7, 132.1, 130.5, 129.2, 129.1, 128.8, 128.2, 128.1, 127.9, 127.6, 127.5, 127.1, 126.8, 126.7, 126.4, 125.5, 125.1, 123.6, 119.0, 118.7, 116.2 (q, *J* = 287.0 Hz), 109.7, 60.5, 51.2, 45.8, 43.4, 33.7, 18.9.

^{19}F NMR (564 MHz, DMSO-d_6) δ (ppm): −75.50.

HRMS (ESI-TOF) m/z: $[M+Na]^+$ Calcd for $C_{41}H_{31}F_3N_4NaO_3^+$ 707.2240, Found: 707.2240.

N-((3R,5'S,6'S)-5'-(benzofuran-2-yl)-1-benzyl-3''-methyl-2,5''-dioxo-1''-phenyl-1'',5''-dihydrodi spiro[indoline-3,1'-cyclohexene-6',4''-pyrazol]-3'-yl)-2,2,2-trifluoroacetamide (3aq)

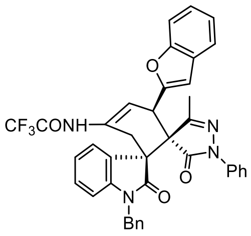

Prepared according to the general procedure, the crude product was purified by silica gel chromatography (petroleum ether/ethyl acetate 20:1) to afford **3aq** (50.2 mg, 74% yield, white solid, > 20:1 dr, m.p: 171.3 – 173.5 °C).

The enantiomeric excess was determined to be 97% by HPLC with a Daicel Chiralpak IG (*n*-hexane/2-propanol = 70:30, 1.0 mL/min, at 254 nm): t_R = 11.96 min (minor), t_R = 15.19 min (major); $[\alpha]_D^{20}$ = – 843.875 (*c* = 0.16, EA).

NMR and HRMS data for the product **3aq**:

^{1}H NMR (600 MHz, Chloroform-*d*) δ (ppm): 7.63 – 7.62 (m, 3H), 7.52 (d, *J* = 7.8 Hz, 1H), 7.45 – 7.43 (m, 1H), 7.32 – 7.27 (m, 3H), 7.19 – 7.14 (m, 2H), 7.13 – 7.10 (m, 2H), 7.05 (t, *J* = 7.2 Hz, 1H), 7.01 (d, *J* = 7.8 Hz, 3H), 6.96 (s, 1H), 6.90 (t, *J* = 7.8 Hz, 2H), 6.74 (d, *J* = 7.8 Hz, 1H), 6.60 (s, 1H), 4.99 (d, *J* = 15.6 Hz, 1H), 4.74 (s, 1H), 4.35 (d, *J* = 15.6 Hz, 1H), 3.44 (d, *J* = 17.4 Hz, 1H), 2.45 (s, 3H), 2.35 (d, *J* = 18.0 Hz, 1H).

^{13}C NMR (150 MHz, Chloroform-*d*) δ (ppm): 173.9, 170.1, 158.6, 155.3 (q, *J* = 37.5 Hz), 154.8, 153.1, 142.2, 137.6, 134.7, 130.8, 129.4, 129.3, 128.7, 128.6, 127.6, 127.6, 126.8, 125.3, 124.4, 124.4, 123.4, 122.9, 120.9, 119.6, 115.4 (q, *J* = 287.0 Hz), 113.9, 111.1, 109.6, 104.5, 58.5, 50.8, 44.1, 40.3, 33.9, 18.0.

^{19}F NMR (564 MHz, Chloroform-*d*) δ (ppm): −75.54.

HRMS (ESI-TOF) m/z: $[M+Na]^+$ Calcd for $C_{39}H_{29}F_3N_4NaO_4^+$ 697.2033, Found: 697.2042.

<u>*N*-((3*R*,5'*S*,6'*S*)-5'-(benzo[*b*]thiophen-2-yl)-1-benzyl-3''-methyl-2,5''-dioxo-1''-phenyl-1'',5''-dihydrodispiro[indoline-3,1'-cyclohexene-6',4''-pyrazol]-3'-yl)-2,2,2-trifluoroacetamide (3ar)</u>

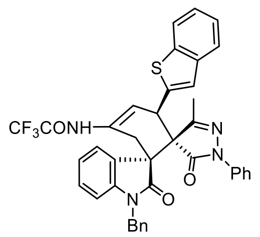

Prepared according to the general procedure, the crude product was purified by silica gel chromatography (petroleum ether/ethyl acetate 20:1) to afford **3ar** (48.3 mg, 70% yield, white solid, > 20:1 dr, m.p: 238.5 – 242.7 °C).

The enantiomeric excess was determined to be 94% by HPLC with a Daicel Chiralpak IG (*n*-hexane/2-propanol = 70:30, 1.0 mL/min, at 254 nm): t_R = 8.40 min (minor), t_R = 23.50 min (major); $[\alpha]_D^{20}$ = – 231.368 (*c* = 0.19, EA).

NMR and HRMS data for the product **3ar**:

^{1}H NMR (600 MHz, Chloroform-*d*) δ (ppm): 7.64 (d, *J* = 7.8 Hz, 1H), 7.60 (d, *J* = 7.8 Hz, 2H), 7.58 (d, *J* = 8.4 Hz, 1H), 7.55 (s, 1H), 7.51 (d, *J* = 7.2 Hz, 1H), 7.32 (t, *J* = 7.8 Hz, 1H), 7.27 – 7.25 (m, 3H), 7.22 (d, *J* = 6.6 Hz, 1H), 7.19 (t, *J* = 7.8 Hz, 1H), 7.17 (s, 1H), 7.13 (t, *J* = 7.2 Hz, 1H), 7.04 (t, *J* = 7.2 Hz, 1H), 6.99 (d, *J* = 6.0 Hz, 3H), 6.87 (t, *J* = 7.8 Hz, 2H), 6.74 (d, *J* = 7.8 Hz, 1H), 5.02 (d, *J* = 15.6 Hz, 1H), 4.87 (s, 1H), 4.36 (d, *J* = 15.6 Hz, 1H), 3.48 (d, *J* = 17.4 Hz, 1H), 2.54 (s, 3H), 2.35 (d, *J* = 17.4 Hz, 1H).

^{13}C NMR (150 MHz, Chloroform-*d*) δ (ppm): 173.1, 170.0, 158.6, 155.3 (q, *J* = 37.7 Hz), 142.3, 139.7, 139.4, 139.1, 137.4, 134.7, 130.6, 129.5, 129.3, 128.7, 128.6, 127.6, 126.8, 125.3, 124.5, 124.5, 124.4, 123.5, 123.4, 123.1, 122.2, 119.5, 117.1, 115.4 (q, *J* = 287.0 Hz), 109.6, 60.2, 51.2, 44.2, 41.4, 34.0, 18.5.

^{19}F NMR (564 MHz, Chloroform-*d*) δ (ppm): −75.49.

HRMS (ESI-TOF) m/z: $[M+Na]^+$ Calcd for $C_{39}H_{29}F_3N_4NaO_3S^+$ 712.1805, Found: 713.1809.

<u>*N*-((3*R*,5'*S*,6'*S*)-1-benzyl-3''-methyl-2,5''-dioxo-1''-phenyl-5'-(thiophen-2-yl)-1'',5''-dihydrodispiro [indoline-3,1'-cyclohexene-6',4''-pyrazol]-3'-yl)-2,2,2-trifluoroacetamide (3as)</u>

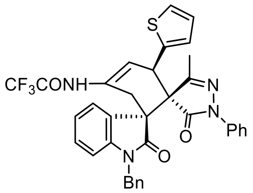

Prepared according to the general procedure, the crude product was purified by silica gel chromatography (petroleum ether/ethyl acetate 20:1) to afford **3as** (47.0 mg, 73% yield, white solid, > 20:1 dr, m.p: 205.9 – 210.6 °C).

The enantiomeric excess was determined to be 94% by HPLC with a Daicel Chiralpak IG (*n*-hexane/2-propanol = 70:30, 1.0 mL/min, at 254 nm): t_R = 9.37 min (minor), t_R = 18.78 min (major); $[\alpha]_D^{20}$ = – 350.769 (*c* = 0.13, EA).

NMR and HRMS data for the product **3as**:

^{1}H NMR (600 MHz, Chloroform-*d*) δ (ppm): 7.63 (d, *J* = 7.8 Hz, 2H), 7.49 (d, *J* = 7.2 Hz, 1H), 7.47 (s, 1H), 7.31 (t, *J* = 7.8 Hz, 1H), 7.29 – 7.25 (m, 2H), 7.18 (t, *J* = 7.8 Hz, 1H), 7.13 (t, *J* = 7.8 Hz, 1H), 7.09 (dd, *J* = 4.8, 0.6 Hz, 1H), 7.03 (t, *J* = 7.8 Hz, 1H), 7.01 (d, J = 7.8 Hz, 2H), 6.88 – 6.86 (m, 4H), 6.82 – 6.81 (m, 1H), 6.76 (d, *J* = 7.8 Hz, 1H), 5.06 (d, *J* = 15.6 Hz, 1H), 4.86 (s, 1H), 4.44 (d, *J* = 15.6 Hz, 1H), 3.48 (d, *J* = 18.0 Hz, 1H), 2.53 (s, 3H), 2.35 (d, *J* = 18.0 Hz, 1H).

^{13}C NMR (150 MHz, Chloroform-*d*) δ (ppm): 174.1, 170.0, 158.7, 155.2 (q, *J* = 37.5 Hz), 142.2, 138.5, 137.5, 134.7, 130.5, 129.5, 129.4, 128.7, 128.6, 127.6, 126.9, 126.8, 126.4, 125.2, 125.2, 124.5, 123.4, 119.4, 117.7, 115.4 (q, *J* = 287.0 Hz), 109.5, 60.4, 51.1, 44.2, 41.0, 33.9, 18.6.

^{19}F NMR (564 MHz, Chloroform-*d*) δ (ppm): −75.53.

HRMS (ESI-TOF) m/z: $[M+Na]^+$ Calcd for $C_{35}H_{27}F_3N_4NaO_3S^+$ 663.1648, Found: 663.1646.

<u>*N*-((3*R*,5'*S*,6'*S*)-1-benzyl-5'-(furan-2-yl)-3''-methyl-2,5''-dioxo-1''-phenyl-1'',5''-dihydrodispiro [indoline-3,1'-cyclohexene-6',4''-pyrazol]-3'-yl)-2,2,2-trifluoroacetamide (3at)</u>

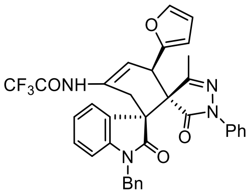

Prepared according to the general procedure, the crude product was purified by silica gel chromatography (petroleum ether/ethyl acetate 20:1) to afford **3at** (46.6 mg, 74% yield, white solid, 15:1 dr, m.p: 176.5 – 180.2 °C)

The enantiomeric excess was determined to be 86% by HPLC with a Daicel Chiralpak IB (*n*-hexane/2-propanol = 80:20, 1.0 mL/min, at 254 nm): t_R = 6.32 min (major), t_R = 7.33 min (minor); $[\alpha]_D^{20}$ = – 694.545 (*c* = 0.13, EA).

NMR and HRMS data for the product **3at**:

^{1}H NMR (600 MHz, Chloroform-*d*) δ (ppm): 7.65 – 7.63 (m, 2H), 7.46 (d, *J* = 7.8 Hz, 2H), 7.29 (q, *J* = 7.8 Hz, 3H), 7.17 – 7.13 (m, 3H), 7.04 (t, *J* = 7.8 Hz, 1H), 7.02 (d, *J* = 7.2 Hz, 2H), 6.89 (t, *J* = 7.8 Hz, 2H), 6.84 (s, 1H), 6.75 (d, *J* = 8.4 Hz, 1H), 6.18 – 6.17 (m, 1H), 6.14 (d, *J* = 3.6 Hz, 1H), 5.05 (d, *J* = 16.2 Hz, 1H), 4.66 (s, 1H), 4.46 (d, *J* = 15.6 Hz, 1H), 3.47 – 3.43 (m, 1H), 2.49 (s, 3H), 2.34 (d, *J* = 18.0 Hz, 1H).

^{13}C NMR (150 MHz, Chloroform-*d*) δ (ppm): 174.1, 170.0, 159.0, 155.2 (q, *J* = 37.5 Hz), 149.7, 142.7, 142.2, 137.6, 134.8, 130.4, 129.5, 129.4, 128.7, 128.6, 127.6, 126.8, 125.1, 124.4, 123.4, 119.4, 115.4 (q, *J* = 287.3 Hz), 114.8, 110.4, 109.5, 107.9, 58.7, 50.8, 44.2, 40.0, 33.9, 18.0.

^{19}F NMR (564 MHz, Chloroform-*d*) δ (ppm): −75.58.

HRMS (ESI-TOF) m/z: $[M+Na]^+$ Calcd for $C_{35}H_{27}F_3N_4NaO_4^+$ 647.1877, Found: 647.1872.

<u>*N*-((3*R*,5'*S*,6'*S*)-1-benzyl-1'',3''-dimethyl-2,5''-dioxo-5'-phenyl-1'',5''-dihydrodispiro[indoline-3,1'-cyclohexene-6',4''-pyrazol]-3'-yl)-2,2,2-trifluoroacetamide (3au)</u>

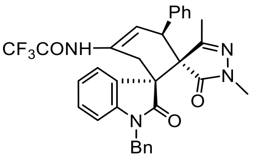

Prepared according to the general procedure, the crude product was purified by silica gel chromatography (petroleum ether/ethyl acetate 20:1) to afford **3au** (40.3 mg, 70% yield, white solid, 9:1 dr, m.p: 224.9 – 232.1 °C).

The enantiomeric excess was determined to be 97% by HPLC with a Daicel Chiralpak IG (*n*-hexane/2-propanol = 70:30, 1.0 mL/min, at 254 nm): t_R = 9.42 min (minor), t_R = 19.58 min (major); $[\alpha]_D^{20}$ = – 822.600 (*c* = 0.10, EA).

NMR and HRMS data for the product **3au**:

^{1}H NMR (600 MHz, Chloroform-*d*) δ (ppm): 7.52 (d, *J* = 7.8 Hz, 1H), 7.51 (s, 1H), 7.36 – 7.34 (m, 1H), 7.33 – 7.28 (m, 3H), 7.27 – 7.26 (m, 3H), 7.22 – 7.21 (m, 2H), 7.20 – 7.18 (m, 1H), 7.17 – 7.15 (m, 2H), 6.85 (s, 1H), 6.83 (d, *J* = 7.8 Hz, 1H), 5.02 (d, *J* = 16.2 Hz, 1H), 4.67 (d, *J* = 15.6 Hz, 1H), 4.46 (s, 1H), 3.46- 3.42 (m 1H), 2.83 (s, 3H), 2.43 (s, 3H), 2.30 (d, *J* = 18.0 Hz, 1H).

^{13}C NMR (150 MHz, Chloroform-*d*) δ (ppm): 174.3, 171.7, 158.0, 155.3 (q, *J* = 37.4 Hz), 142.2, 136.3, 135.3, 130.1, 129.8, 129.2, 128.8, 128.4, 128.2, 128.1, 127.7, 127.0, 124.6, 123.4, 118.6, 115.5 (q, *J* = 287.1 Hz), 109.3, 58.9, 50.8, 45.0, 43.9, 33.7, 30.7, 18.6.

^{19}F NMR (564 MHz, Chloroform-*d*) δ (ppm): –75.53.

HRMS (ESI-TOF) m/z: $[M+Na]^+$ Calcd for $C_{32}H_{27}F_3N_4NaO_3^+$ 595.1927, Found: 595.1928.

<u>*N*-((3*R*,5'*S*,6'*S*)-1-benzyl-1''-(3-chlorophenyl)-3''-methyl-2,5''-dioxo-5'-phenyl-1'',5''-dihydrodi spiro[indoline-3,1'-cyclohexene-6',4''-pyrazol]-3'-yl)-2,2,2-trifluoroacetamide (3av)</u>

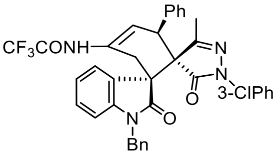

Prepared according to the general procedure, the crude product was purified by silica gel chromatography (petroleum ether/ethyl acetate 20:1) to afford **3av** (64.3 mg, 96% yield, white solid, 13:1 dr, m.p: 243.0 – 247.0 °C)

The enantiomeric excess was determined to be 99% by HPLC with a Daicel Chiralpak IG (*n*-hexane/2-propanol = 70:30, 1.0 mL/min, at 254 nm): t_R = 5.05 min (minor), t_R = 7.98 min (major); $[\alpha]_D^{20}$ = + 503.333 (*c* = 0.12, EA).

NMR and HRMS data for the product **3av**:

^{1}H NMR (600 MHz, Chloroform-*d*) δ (ppm): 7.65 (t, *J* = 1.8 Hz, 1H), 7.54 (d, *J* = 7.8 Hz, 1H), 7.43 – 7.41 (m, 1H), 7.40 (s, 1H), 7.36 (t, *J* = 7.8 Hz, 1H), 7.21 (t, *J* = 7.8 Hz, 1H), 7.18 – 7.13 (m, 6H), 7.09 – 7.06

(m, 2H), 7.04 (d, J = 7.2 Hz, 2H), 6.96 (t, J = 7.8 Hz, 2H), 6.85 (s, 1H), 6.81 (d, J = 7.8 Hz, 1H), 5.09 (d, J = 15.6 Hz, 1H), 4.57 (s, 1H), 4.47 (d, J = 15.6 Hz, 1H), 3.50 (d, J = 18.0 Hz, 1H), 2.54 (s, 3H), 2.34 (d, J = 17.4 Hz, 1H).

^{13}C NMR (150 MHz, Chloroform-*d*) δ (ppm): 174.2, 170.5, 159.2, 155.3 (q, J = 37.5 Hz), 142.3, 138.2, 135.9, 134.8, 134.3, 130.1, 129.7, 129.6, 129.4, 128.7, 128.6, 128.3, 128.1, 127.7, 126.9, 125.0, 124.6, 123.5, 119.0, 118.6, 116.9, 115.5 (q, J = 287.3 Hz), 109.5, 60.6, 51.1, 45.7, 44.1, 34.0, 18.8.

^{19}F NMR (564 MHz, Chloroform-*d*) δ (ppm): −75.57.

HRMS (ESI-TOF) m/z: $[M+Na]^+$ Calcd for $C_{37}H_{28}ClF_3N_4NaO_3^+$ 691.1694, Found: 691.1696.

<u>*N*-((3*R*,5'*S*,6'*S*)-1-benzyl-1''-(3-bromophenyl)-3''-methyl-2,5''-dioxo-5'-phenyl-1'',5''-dihydrodispiro[indoline-3,1'-cyclohexene-6',4''-pyrazol]-3'-yl)-2,2,2-trifluoroacetamide (3aw)</u>

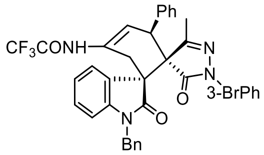

Prepared according to the general procedure, the crude product was purified by silica gel chromatography (petroleum ether/ethyl acetate 20:1) to afford **3aw** (57.3 mg, 80% yield, white solid, > 20:1 dr, m.p: 225.8 – 229.1 °C).

The enantiomeric excess was determined to be 99% by HPLC with a Daicel Chiralpak IG (*n*-hexane/2-propanol = 70:30, 1.0 mL/min, at 254 nm): t_R = 5.07 min (minor), t_R = 8.53 min (major); $[\alpha]_D^{20}$ = + 164.545 (c = 0.11, EA).

NMR and HRMS data for the product **3aw**:

^{1}H NMR (600 MHz, Chloroform-*d*) δ (ppm): 7.79 (t, J = 1.8 Hz, 1H), 7.54 (d, J = 7.2 Hz, 1H), 7.49 – 7.45 (m, 1H), 7.39 (s, 1H), 7.36 (t, J = 7.8 Hz, 1H), 7.23 – 7.19 (m, 2H), 7.17 – 7.15 (m, 5H), 7.09 – 7.07 (m, 2H), 7.04 (d, J = 7.2 Hz, 2H), 6.97 (t, J = 7.8 Hz, 2H), 6.85 (s, 1H), 6.82 (d, J = 7.8 Hz, 1H), 5.08 (d, J = 16.2 Hz, 1H), 4.57 (s, 1H), 4.47 (d, J = 15.6 Hz, 1H), 3.49 (d, J = 18.0 Hz, 1H), 2.53 (s, 3H), 2.33 (d, J = 18.0 Hz, 1H).

^{13}C NMR (150 MHz, Chloroform-*d*) δ (ppm): 174.2, 170.5, 159.2, 155.3 (q, J = 37.4 Hz), 142.3, 138.3, 135.9, 134.8, 130.1, 129.9, 129.7, 129.4, 128.7, 128.6, 128.3, 128.1, 128.0, 127.7, 126.9, 124.5, 123.5, 122.3, 121.7, 118.6, 117.4, 115.5 (q, J = 287.1 Hz), 109.5, 60.6, 51.1, 45.7, 44.1, 34.0, 18.8.

^{19}F NMR (564 MHz, Chloroform-*d*) δ (ppm): −75.56.

HRMS (ESI-TOF) m/z: $[M+Na]^+$ Calcd for $C_{37}H_{28}BrF_3N_4NaO_3^+$ 735.1189, Found: 735.1180.

***N*-((3*R*,5'*S*,6'*S*)-1-benzyl-3''-methyl-2,5''-dioxo-5'-phenyl-1''-(*p*-tolyl)-1'',5''-dihydrodispiro [indoline-3,1'-cyclohexene-6',4''-pyrazol]-3'-yl)-2,2,2-trifluoroacetamide (3ax)**

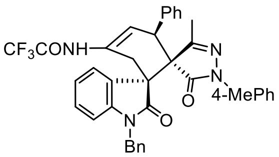

Prepared according to the general procedure, the crude product was purified by silica gel chromatography (petroleum ether/ethyl acetate 20:1) to afford **3ax** (59.8 mg, 92% yield, white solid, > 20:1 dr, m.p: 252.5 – 257.0 °C).

The enantiomeric excess was determined to be 99% by HPLC with a Daicel Chiralpak IG (*n*-hexane/2-propanol = 80:20, 1.0 mL/min, at 254 nm): t_R = 22.22 min (minor), t_R = 25.52 min (major); $[\alpha]_D^{20}$ = + 381.429 (*c* = 0.14, EA).

NMR and HRMS data for the product **3ax**:

^{1}H NMR (600 MHz, Chloroform-*d*) δ (ppm): 7.53 (d, *J* = 7.2 Hz, 1H), 7.40 (s, 1H), 7.38 (d, *J* = 9.0 Hz, 2H), 7.31 (t, *J* = 7.8 Hz, 1H), 7.20 – 7.15 (m, 6H), 7.07 (t, *J* = 7.8 Hz, 1H), 7.03 (d, *J* = 7.8 Hz, 4H), 6.93 (t, *J* = 7.8 Hz, 2H), 6.86 (s, 1H), 6.76 (d, *J* = 7.8 Hz, 1H), 5.05 (d, *J* = 15.6 Hz, 1H), 4.58 (s, 1H), 4.49 (d, *J* = 15.6 Hz, 1H), 3.50 (d, *J* = 17.4 Hz, 1H), 2.52 (s, 3H), 2.32 (d, *J* = 18.0 Hz, 1H), 2.30 (s, 3H).

^{13}C NMR (150 MHz, Chloroform-*d*) δ (ppm): 174.3, 170.1, 158.5, 155.3 (q, *J* = 37.5 Hz), 142.3, 136.1, 134.9, 134.9, 134.8, 130.1, 129.9, 129.3, 129.0, 128.7, 128.6, 128.2, 128.1, 127.5, 126.9, 124.5, 123.4, 119.4, 118.7, 115.5 (q, *J* = 287.0 Hz), 109.4, 60.3, 51.1, 45.5, 44.1, 34.1, 21.0, 18.8.

^{19}F NMR (564 MHz, Chloroform-*d*) δ (ppm): −75.58.

HRMS (ESI-TOF) m/z: $[M+Na]^+$ Calcd for $C_{38}H_{31}F_3N_4NaO_3^+$ 671.2240, Found: 671.2235.

***N*-((3*R*,5'*S*,6'*S*)-1-benzyl-1''-(4-fluorophenyl)-3''-methyl-2,5''-dioxo-5'-phenyl-1'',5''-dihydrodi spiro[indoline-3,1'-cyclohexene-6',4''-pyrazol]-3'-yl)-2,2,2-trifluoroacetamide (3ay)**

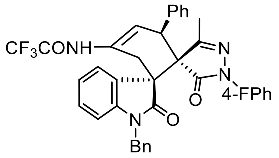

Prepared according to the general procedure, the crude product was purified by silica gel chromatography (petroleum ether/ethyl acetate 20:1) to afford **3ay** (55.6 mg, 85% yield, white solid, 15:1 dr, m.p. 268.2 – 273.6 °C).

The enantiomeric excess was determined to be 99% by HPLC with a Daicel Chiralpak IG (*n*-hexane/2-propanol =70:30, 1.0 mL/min, at 254 nm): t_R = 7.58 min (minor), t_R = 12.93 min (major); $[\alpha]_D^{20}$ = – 403.800 (*c* = 0.10, EA).

NMR and HRMS data for the product **3ay**:

^{1}H NMR (600 MHz, Chloroform-*d*) δ (ppm): 7.54 (d, *J* = 7.8 Hz, 1H), 7.48 – 7.44 (m, 2H), 7.39 (s, 1H), 7.34 (t, *J* = 7.8 Hz, 1H), 7.20 – 7.16 (m, 6H), 7.08 (t, *J* = 7.8 Hz, 1H), 7.03 (d, *J* = 7.8 Hz, 2H), 6.93 (d, *J* = 7.8 Hz, 2H), 6.92 – 6.89 (m, 2H), 6.86 (s, 1H), 6.79 (d, *J* = 7.8 Hz, 1H), 5.07 (d, *J* = 15.6 Hz, 1H), 4.57 (s, 1H), 4.49 (d, *J* = 16.2 Hz, 1H), 3.51 – 3.45 (m, 1H), 2.53 (s, 3H), 2.34 (d, *J* = 18.0 Hz, 1H).

^{13}C NMR (150 MHz, Chloroform-*d*) δ (ppm): 174.3, 170.2, 159.9 (d, J_{CF}= 243.2 Hz), 159.0, 155.3 (q, *J* = 37.4 Hz), 142.3, 136.0, 134.9, 133.4 (d, J_{CF}= 2.7 Hz), 130.1, 129.8, 129.4, 128.7, 128.5, 128.2, 128.1, 127.6, 126.8, 124.6, 123.5, 121.1 (d, J_{CF}= 8.0 Hz), 118.6, 115.5 (q, *J* = 287.0 Hz), 115.2 (d, *J* = 22.4 Hz), 109.4, 60.4, 51.1, 45.6, 44.1, 34.0, 18.8.

^{19}F NMR (564 MHz, Chloroform-*d*) δ (ppm): −75.57. −117.00 – −117.04.

HRMS (ESI-TOF) m/z: $[M+Na]^+$ Calcd for $C_{37}H_{28}F_4N_4NaO_3^+$ 675.1990, Found: 675.1982.

<u>*N*-((3*R*,5'*S*,6'*S*)-1-benzyl-1''-(4-bromophenyl)-3''-methyl-2,5''-dioxo-5'-phenyl-1'',5''-dihydrodi spiro[indoline-3,1'-cyclohexene-6',4''-pyrazol]-3'-yl)-2,2,2-trifluoroacetamide (3az)</u>

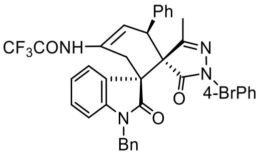

Prepared according to the general procedure, the crude product was purified by silica gel chromatography (petroleum ether/ethyl acetate 20:1) to afford **3az** (67.8 mg, 95% yield, white solid, > 20:1 dr, m.p: 255.5 – 260.1 °C).

The enantiomeric excess was determined to be 99% by HPLC with a Daicel Chiralpak IG (*n*-hexane/2-propanol =80:20, 1.0 mL/min, at 254 nm): t_R = 17.78 min (major), t_R = 21.34 min (minor); $[\alpha]_D^{20}$ = + 150.000 (*c* = 0.11, EA).

NMR and HRMS data for the product **3az**:

^{1}H NMR (600 MHz, Chloroform-*d*) δ (ppm): 7.53 (d, *J* = 7.8 Hz, 1H), 7.43 (d, *J* = 9.0 Hz, 2H), 7.36 (s, 1H), 7.34 – 7.31 (m, 3H), 7.20 (t, *J* = 7.8 Hz, 1H), 7.17 – 7.15 (m, 5H), 7.09 (t, *J* = 7.8 Hz, 1H), 7.03 (d, *J* = 7.8 Hz, 2H), 6.95 (t, *J* = 7.8 Hz, 2H), 6.85 (s, 1H), 6.81 (d, *J* = 7.8 Hz, 1H), 5.06 (d, *J* = 15.6 Hz, 1H), 4.56 (s, 1H), 4.48 (d, *J* = 15.6 Hz, 1H), 3.49 (d, *J* = 18.0 Hz, 1H), 2.53 (s, 3H), 2.34 (d, *J* = 18.0 Hz, 1H).

^{13}**C NMR** (150 MHz, Chloroform-*d*) δ (ppm): 174.2, 170.4, 159.2, 155.3 (q, *J* = 37.5 Hz), 142.3, 136.3, 135.9, 134.8, 131.5, 130.1, 129.7, 129.4, 128.7, 128.6, 128.2, 128.1, 127.7, 126.9, 124.6, 123.5, 120.6, 118.6, 117.9, 115.5 (q, *J* = 287.1 Hz), 109.4, 60.5, 51.1, 45.6, 44.1, 34.0, 18.8.

^{19}**F NMR** (564 MHz, Chloroform-*d*) δ (ppm): −75.57.

HRMS (ESI-TOF) m/z: $[M+Na]^+$ Calcd for $C_{37}H_{28}BrF_3N_4NaO_3^+$ 735.1189, Found: 735.1184.

<u>*N*-((3*R*,5'*S*,6'*S*)-1-benzyl-2,5''-dioxo-1'',3'-diphenyl-3''-propyl-1'',5''-dihydrodispiro[indoline-3,1'-cyclohexene-6',4''-pyrazol]-3'-yl)-2,2,2-trifluoroacetamide (3bb)</u>

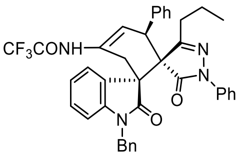

Prepared according to the general procedure, two diastereoisomers **3bb** and **3bb'** (60.0 mg, 90% yield, 1:2 dr) were obtained and separated by silica gel column chromatography (petroleum ether/ethyl acetate 20:1) to afford **3bb** and **3bb'**.

3bb (20.0 mg, 30% yield, white solid, m.p: 202.1 – 203.3 °C).

The enantiomeric excess was determined to be 87% by HPLC with a Daicel Chiralpak IG (*n*-hexane/2-propanol =80:20, 1.0 mL/min, at 254 nm): t_R = 10.46 min (minor), t_R = 13.55 min (major); $[\alpha]_D^{20}$ = + 261.000 (*c* = 0.10, EA).

NMR and HRMS data for the product **3bb**:

^{1}**H NMR** (600 MHz, Chloroform-*d*) δ (ppm): 7.57 (d, *J* = 7.8 Hz, 2H), 7.55 (d, *J* = 7.8 Hz, 1H), 7.41 (s, 1H), 7.31 (t, *J* = 7.8 Hz, 1H), 7.27 – 7.24 (m, 2H), 7.19 – 7.16 (m, 6H), 7.11 (t, *J* = 7.2 Hz, 1H), 7.05 (t, *J* = 7.8 Hz, 1H), 7.01 (d, *J* = 7.8 Hz, 2H), 6.91 – 6.89 (m, 3H), 6.75 (d, *J* = 7.8 Hz, 1H), 5.06 (d, *J* = 15.6 Hz, 1H), 4.58 (s, 1H), 4.46 (d, *J* = 16.2 Hz, 1H), 3.49 (d, *J* = 17.4 Hz, 1H), 3.09 – 3.04 (m, 1H), 2.72 – 2.66 (m, 1H), 2.29 (d, *J* = 18.0 Hz, 1H), 1.97 – 1.88 (m, 1H), 1.39 – 1.33 (m, 1H), 1.00 (t, *J* = 7.8 Hz, 3H).

^{13}**C NMR** (150 MHz, Chloroform-*d*) δ (ppm): 174.3, 170.4, 161.7, 155.3 (q, *J* = 37.2 Hz), 142.2, 137.6, 136.3, 134.8, 130.2, 129.9, 129.2, 128.7, 128.5, 128.5, 128.1, 128.1, 127.5, 126.8, 125.0, 124.6, 123.4, 119.3, 118.6, 115.5 (q, *J* = 287.1 Hz), 109.4, 60.4, 51.2, 45.5, 44.1, 34.1, 33.9, 18.3, 14.2.

^{19}**F NMR** (564 MHz, Chloroform-*d*) δ (ppm): −75.53.

HRMS (ESI-TOF) m/z: $[M+Na]^+$ Calcd for $C_{39}H_{33}F_3N_4NaO_3^+$ 685.2397, Found: 685.2395.

_N_-((3_R_,5'_R_,6'_S_)-1-benzyl-2,5''-dioxo-1'',3'-diphenyl-3''-propyl-1'',5''-dihydrodispiro[indoline-3,1'-cyclohexene-6',4''-pyrazol]-3'-yl)-2,2,2-trifluoroacetamide (3bb')

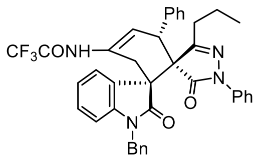

3bb' (40.0 mg, 60% yield, white solid, m.p: 219.5 – 219.9 °C).

NMR and HRMS data for the product **3bb'**:

^{1}H NMR (600 MHz, Chloroform-*d*) δ (ppm): 7.43 (d, *J* = 7.2 Hz, 2H), 7.38 (t, *J* = 7.8 Hz, 3H), 7.34 – 7.31 (m, 3H), 7.27 – 7.24 (m, 2H), 7.22 – 7.16 (m, 5H), 7.15 – 7.10 (m, 2H), 7.06 (d, *J* = 7.8 Hz, 1H), 6.89 (d, *J* = 7.8 Hz, 1H), 6.86 (t, *J* = 7.8 Hz, 1H), 6.27 (s, 1H), 5.07 (d, *J* = 15.0 Hz, 1H), 4.95 (s, 1H), 4.82 (d, *J* = 15.6 Hz, 1H), 3.87 (d, *J* = 17.4 Hz, 1H), 2.52 (d, *J* = 18.0 Hz, 1H), 2.44 (ddd, *J* = 18.0, 9.6, 6.0 Hz, 1H), 1.75 (ddd, *J* = 17.4, 8.4, 6.0 Hz, 1H), 1.52 – 1.46 (m, 1H), 1.45 – 1.39 (m, 1H), 0.74 (t, *J* = 7.2 Hz, 3H).

^{13}C NMR (150 MHz, Chloroform-*d*) δ (ppm): 176.0, 171.0, 160.7, 154.5 (q, *J* = 37.4 Hz), 141.7, 136.9, 136.7, 135.2, 129.6, 129.4, 129.2, 129.1, 128.6, 128.2, 128.2, 128.1, 128.0, 127.9, 125.6, 123.8, 123.6, 119.9, 115.8, 115.6 (q, *J* = 287.6 Hz), 109.4, 59.9, 48.2, 44.3, 42.5, 32.4, 30.3, 17.8, 13.8.

^{19}F NMR (564 MHz, Chloroform-*d*) δ (ppm): −75.72.

HRMS (ESI-TOF) m/z: $[M+Na]^+$ Calcd for $C_{39}H_{33}F_3N_4NaO_3^+$ 685.2397, Found: 685.2404.

_N_-((3_R_,5'_S_,6'_S_)-1-benzyl-7-fluoro-3''-methyl-2,5''-dioxo-1'',5'-diphenyl-1'',5''-dihydrodispiro [indoline-3,1'-cyclohexene-6',4''-pyrazol]-3'-yl)-2,2,2-trifluoroacetamide (3bc)

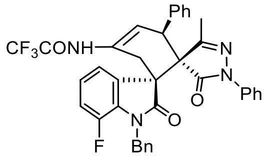

Prepared according to the general procedure, the crude product was purified by silica gel chromatography (petroleum ether/ethyl acetate 20:1) to afford **3bc** (56.3 mg, 86% yield, white solid, 12:1 dr, m.p: 262.6 – 265.4 °C).

The enantiomeric excess was determined to be 99% by HPLC with a Daicel Chiralpak IG (*n*-hexane/2-propanol = 70:30, 1.0 mL/min, at 254 nm): t_R = 7.45 min (minor), t_R = 11.60 min (major); $[\alpha]_D^{20}$ = – 203.833 (*c* = 0.12, EA).

NMR and HRMS data for the product **3bc**:

^{1}H NMR (600 MHz, Chloroform-*d*) δ (ppm): 7.51 (s, 1H), 7.46 (d, *J* = 8.4 Hz, 2H), 7.35 (d, *J* = 6.6 Hz, 1H), 7.26 – 7.23 (m, 2H), 7.16 (s, 5H), 7.14 – 7.09 (m, 3H), 7.07 (d, *J* = 7.8 Hz, 2H), 7.00 (t, *J* = 7.8 Hz,

1H), 6.88 – 6.86 (m, 3H), 5.07 (d, J = 15.6 Hz, 1H), 4.77 (d, J = 15.6 Hz, 1H), 4.54 (s, 1H), 3.47 (d, J = 18.0 Hz, 1H), 2.50 (s, 3H), 2.30 (d, J = 18.0 Hz, 1H).

^{13}C NMR (150 MHz, Chloroform-*d*) δ (ppm): 174.0, 170.1, 158.4, 155.4 (q, J = 37.5 Hz), 147.7 (d, J_{CF} = 243.5 Hz), 137.1, 136.0, 135.8, 132.7 (d, J = 2.9 Hz), 130.0, 129.1 (d, J = 8.9 Hz), 128.6, 128.5, 128.3, 128.1, 127.5, 126.9, 126.9, 125.3, 124.0 (d, J = 6.5 Hz), 120.5 (d, J_{CF} = 3.0 Hz), 119.5, 118.6, 117.5 (d, J_{CF} = 19.4 Hz), 115.5 (q, J_{CF} = 287.0 Hz), 60.3, 51.2, 45.8 (d, J_{CF} = 5.3 Hz), 45.5, 34.1, 18.8.

^{19}F NMR (564 MHz, Chloroform-*d*) δ (ppm): −75.58, −133.17 – −113.20.

HRMS (ESI-TOF) m/z: $[M+Na]^+$ Calcd for $C_{37}H_{28}F_4N_4NaO_3^+$ 675.1990, Found: 675.1994.

***N*-((3*R*,5'*S*,6'*S*)-1-benzyl-7-chloro-3''-methyl-2,5''-dioxo-1'',5'-diphenyl-1'',5''-dihydrodispiro [indoline-3,1'-cyclohexene-6',4''-pyrazol]-3'-yl)-2,2,2-trifluoroacetamide (3bd)**

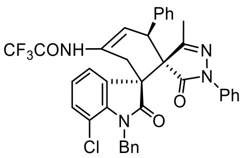

Prepared according to the general procedure, the crude product was purified by silica gel chromatography (petroleum ether/ethyl acetate 20:1) to afford **3bd** (62.3 mg, 93% yield, white solid, 15:1 dr, m.p: 246.5 – 248.2 °C).

The enantiomeric excess was determined to be 99% by HPLC with a Daicel Chiralpak IG (*n*-hexane/2-propanol = 70:30, 1.0 mL/min, at 254 nm): t_R = 9.09 min (minor), t_R = 11.67 min (major); $[\alpha]_D^{20}$ = + 422.000 (*c* = 0.10, EA).

NMR and HRMS data for the product **3bd**:

^{1}H NMR (600 MHz, Chloroform-*d*) δ (ppm): 7.52 (s, 1H), 7.48 (d, J = 9.0 Hz, 3H), 7.33 (d, J = 8.4 Hz, 1H), 7.26 – 7.23 (m, 2H), 7.17 (s, 5H), 7.16 – 7.11 (m, 2H), 6.96 (t, J = 7.8 Hz, 3H), 6.88 (s, 1H), 6.83 (t, J = 8.4 Hz, 2H), 5.27 (d, J = 16.2 Hz, 1H), 5.18 (d, J = 16.8 Hz, 1H), 4.55 (s, 1H), 3.46 (d, J = 18.0 Hz, 1H), 2.50 (s, 3H), 2.31 (d, J = 18.0 Hz, 1H).

^{13}C NMR (150 MHz, Chloroform-*d*) δ (ppm): 174.9, 170.2, 158.5, 155.4 (q, J = 37.4 Hz), 138.5, 137.1, 136.5, 135.8, 132.7, 132.0, 130.0, 128.6, 128.5, 128.4, 128.3, 128.2, 127.0, 125.9, 125.3, 124.2, 123.1, 119.5, 118.5, 115.9, 115.5 (q, J = 287.0 Hz), 60.4, 50.5, 45.5, 45.5, 34.2, 18.7.

^{19}F NMR (564 MHz, Chloroform-*d*) δ (ppm): −75.56.

HRMS (ESI-TOF) m/z: $[M+Na]^+$ Calcd for $C_{37}H_{28}ClF_3N_4NaO_3^+$ 691.1694, Found: 691.1695.

***N*-((3*R*,5'*S*,6'*S*)-1-benzyl-7-bromo-3''-methyl-2,5''-dioxo-1'',5'-diphenyl-1'',5''-dihydrodispiro [indoline-3,1'-cyclohexene-6',4''-pyrazol]-3'-yl)-2,2,2-trifluoroacetamide (3be)**

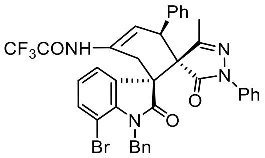

Prepared according to the general procedure, the crude product was purified by silica gel chromatography (petroleum ether/ethyl acetate 20:1) to afford **3be** (55.1 mg, 77% yield, white solid, 15:1 dr, m.p: 267.5 – 269.5 °C).

The enantiomeric excess was determined to be 97% by HPLC with a Daicel Chiralpak IG (*n*-hexane/2-propanol = 70:30, 1.0 mL/min, at 254 nm): t_R = 7.41 min (minor), t_R = 10.16 min (major); $[\alpha]_D^{20}$ = – 450.655 (*c* = 0.15, EA).

NMR and HRMS data for the product **3be**:

^{1}H NMR (600 MHz, Chloroform-*d*) δ (ppm): 7.53 (t, *J* = 8.4 Hz, 2H), 7.48 (d, *J* = 8.4 Hz, 3H), 7.24 (t, *J* = 7.8 Hz, 2H), 7.17 (s, 5H), 7.12 (t, *J* = 7.8 Hz, 1H), 7.08 (t, *J* = 7.8 Hz, 1H), 6.96 (t, *J* = 7.8 Hz, 3H), 6.86 (s, 1H), 6.83 (t, *J* = 7.8 Hz, 2H), 5.31 (d, *J* = 16.2 Hz, 1H), 5.25 (d, *J* = 16.2 Hz, 1H), 4.55 (s, 1H), 3.47 (d, *J* = 18.0 Hz, 1H), 2.50 (s, 3H), 2.36 (d, *J* = 18.0 Hz, 1H).

^{13}C NMR (150 MHz, Chloroform-*d*) δ (ppm): 175.1, 170.2, 158.5, 155.4 (q, *J* = 37.5 Hz), 139.9, 137.1, 136.5, 135.8, 135.4, 133.1, 130.1, 128.6, 128.6, 128.4, 128.3, 128.2, 126.9, 125.8, 125.3, 124.5, 123.6, 119.5, 118.6, 115.5 (q, *J* = 287.0 Hz), 102.9, 60.4, 50.4, 45.5, 45.2, 34.3, 18.7.

^{19}F NMR (564 MHz, Chloroform-*d*) δ (ppm): −75.53.

HRMS (ESI-TOF) m/z: $[M+Na]^+$ Calcd for $C_{37}H_{28}BrF_3N_4NaO_3^+$ 735.1189, Found: 735.1180.

***N*-((3*R*,5'*S*,6'*S*)-1-benzyl-7-benzyl-3''-methyl-2,5''-dioxo-1'',5'-diphenyl-1'',5''-dihydrodispiro [indoline-3,1'-cyclohexene-6',4''-pyrazol]-3'-yl)-2,2,2-trifluoroacetamide (3bf)**

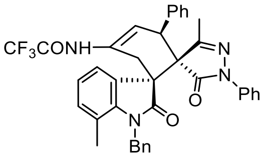

Prepared according to the general procedure, the crude product was purified by silica gel chromatography (petroleum ether/ethyl acetate 20:1) to afford **3bf** (55.5 mg, 85% yield, white solid, 15:1 dr, m.p: 285.5 – 287.1 °C).

The enantiomeric excess was determined to be 99% by HPLC with a Daicel Chiralpak IG (*n*-hexane/2-propanol = 70:30, 1.0 mL/min, at 254 nm): t_R = 8.74 min (minor), t_R = 12.28 min (major); $[\alpha]_D^{20}$ = + 780.800 (*c* = 0.10, EA).

NMR and HRMS data for the product **3bf**:

^{1}H NMR (600 MHz, Chloroform-*d*) δ (ppm): 7.55 (d, *J* = 7.8 Hz, 2H), 7.47 (s, 1H), 7.45 – 7.43 (m, 1H), 7.26 – 7.23 (m, 2H), 7.21 – 7.20 (m, 2H), 7.17 – 7.16 (m, 3H), 7.13 – 7.10 (m, 3H), 6.97 (t, *J* = 7.8 Hz, 1H), 6.90 – 6.88 (m, 3H), 6.80 (t, *J* = 7.8 Hz, 2H), 5.23 (d, *J* = 16.8 Hz, 1H), 4.85 (d, *J* = 16.8 Hz, 1H), 4.62 (s, 1H), 3.50 – 3.47 (m, 1H), 2.52 (s, 3H), 2.31 (d, *J* = 17.4 Hz, 1H), 2.27 (s, 3H).

^{13}C NMR (150 MHz, Chloroform-*d*) δ (ppm): 175.4, 170.4, 158.9, 155.3 (q, *J* = 37.5 Hz), 140.6, 137.3, 136.6, 136.1, 133.4, 130.6, 130.2, 128.7, 128.5, 128.5, 128.2, 128.2, 127.0, 125.2, 125.1, 123.4, 122.5, 120.0, 119.5, 118.5, 115.5 (q, *J* = 287.1 Hz), 60.5, 50.3, 45.5, 45.5, 34.5, 19.0, 18.8.

^{19}F NMR (564 MHz, Chloroform-*d*) δ (ppm): –75.56.

HRMS (ESI-TOF) m/z: $[M+Na]^+$ Calcd for $C_{38}H_{31}F_3N_4NaO_3^+$ 671.2240, Found: 671.2239.

<u>*N*-((3*R*,5'*S*,6'*S*)-1-benzyl-6-fluoro-3''-methyl-2,5''-dioxo-1'',5'-diphenyl-1'',5''-dihydrodispiro [indoline-3,1'-cyclohexene-6',4''-pyrazol]-3'-yl)-2,2,2-trifluoroacetamide (3bg)</u>

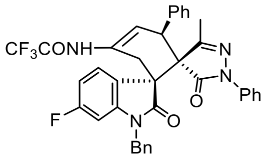

Prepared according to the general procedure, the crude product was purified by silica gel chromatography (petroleum ether/ethyl acetate 20:1) to afford **3bg** (60.2 mg, 92% yield, white solid, > 20:1 dr, m.p: 254.6 – 258.3 °C).

The enantiomeric excess was determined to be 99% by HPLC with a Daicel Chiralpak IG (*n*-hexane/2-propanol = 70:30, 1.0 mL/min, at 254 nm): t_R = 6.29 min (minor), t_R = 12.42 min (major); $[\alpha]_D^{20}$ = – 513.200 (*c* = 0.10, EA).

NMR and HRMS data for the product **3bg**:

^{1}H NMR (600 MHz, Chloroform-*d*) δ (ppm): 7.53 (s, 1H), 7.51 (d, *J* = 7.8 Hz, 2H), 7.47 (dd, *J* = 8.4, 5.4 Hz, 1H), 7.27 – 7.25 (m, 2H), 7.17 (s, 5H), 7.13 (t, *J* = 7.8 Hz, 1H), 7.07 (t, *J* = 7.8 Hz, 1H), 7.00 (d, *J* = 7.8 Hz, 2H), 6.91 (t, *J* = 7.8 Hz, 2H), 6.87 – 6.84 (m, 2H), 6.50 (d, *J* = 9.0 Hz, 1H), 5.05 (d, *J* = 15.6 Hz, 1H), 4.53 (s, 1H), 4.42 (d, *J* = 15.6 Hz, 1H), 3.49 (d, *J* = 17.4 Hz, 1H), 2.52 (s, 3H), 2.32 (d, *J* = 18.0 Hz, 1H).

^{13}C NMR (150 MHz, Chloroform-*d*) δ (ppm): 174.6, 170.2, 163.4 (d, J_{CF} = 246.0 Hz), 158.6, 155.4 (q, J = 37.4 Hz), 143.8 (d, J_{CF} = 11.6 Hz), 137.2, 135.9, 134.3, 130.1, 129.0, 128.8, 128.6, 128.3, 128.1, 127.8, 125.7, 125.7 (d, J_{CF} = 9.6 Hz), 125.3, 125.2 (d, J_{CF} = 3.2 Hz), 119.5, 118.6, 115.5 (q, J = 287.1 Hz,) 109.7 (d, J_{CF} = 22.1 Hz), 98.3 (d, J_{CF} = 27.3), 60.3, 50.8, 45.6, 44.3, 34.1, 18.8.

^{19}F NMR (564 MHz, Chloroform-*d*) δ (ppm): −75.55, −109.68 – −109.72.

HRMS (ESI-TOF) m/z: $[M+Na]^+$ Calcd for $C_{37}H_{28}F_4N_4NaO_3^+$ 675.1990, Found: 675.1989.

<u>*N*-((3*R*,5'*S*,6'*S*)-1-benzyl-6-chloro-3''-methyl-2,5''-dioxo-1'',5'-diphenyl-1'',5''-dihydrodispiro [indoline-3,1'-cyclohexene-6',4''-pyrazol]-3'-yl)-2,2,2-trifluoroacetamide (3bh)</u>

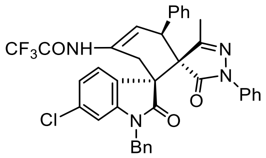

Prepared according to the general procedure, the crude product was purified by silica gel chromatography (petroleum ether/ethyl acetate 20:1) to afford **3bh** (64.8 mg, 97% yield, white solid, 15:1 dr, m.p: 280.8 – 283.7 °C).

The enantiomeric excess was determined to be 99% by HPLC with a Daicel Chiralpak IG (*n*-hexane/2-propanol = 70:30, 1.0 mL/min, at 254 nm): t_R = 6.15 min (minor), t_R = 13.98 min (major); $[\alpha]_D^{20}$ = + 509.800 (*c* = 0.10, EA).

NMR and HRMS data for the product **3bh**:

^{1}H NMR (600 MHz, Chloroform-*d*) δ (ppm): 7.51 (d, J = 7.8 Hz, 2H), 7.45 (d, J = 8.4 Hz, 1H), 7.38 (s, 1H), 7.27 – 7.24 (m, 2H), 7.18 (s, 5H), 7.16 – 7.11 (m, 2H), 7.07 (t, J = 7.8 Hz, 1H), 7.00 (d, J = 7.8 Hz, 2H), 6.91 (t, J = 7.8 Hz, 2H), 6.83 (s, 1H), 6.75 (d, J = 1.8 Hz, 1H), 5.06 (d, J = 15.6 Hz, 1H), 4.53 (s, 1H), 4.44 (d, J = 16.2 Hz, 1H), 3.50 (d, J = 18.0 Hz, 1H), 2.52 (s, 3H), 2.32 (d, J = 17.4 Hz, 1H).

^{13}C NMR (150 MHz, Chloroform-*d*) δ (ppm): 174.3, 170.2, 158.5, 155.3 (q, J = 37.5 Hz), 143.4, 137.2, 135.8, 135.2, 134.3, 130.0, 129.1, 128.8, 128.6, 128.3, 128.3, 128.1, 127.8, 126.8, 125.4, 125.3, 123.4, 119.5, 118.7, 115.5 (q, J = 287.1 Hz), 110.1, 60.3, 50.9, 45.6, 44.2, 34.0, 18.8.

^{19}F NMR (564 MHz, Chloroform-*d*) δ (ppm): −75.56.

HRMS (ESI-TOF) m/z: $[M+Na]^+$ Calcd for $C_{37}H_{28}ClF_3N_4NaO_3^+$ 691.1694, Found: 691.1704.

<u>*N*-((3*R*,5'*S*,3'*S*)-1-benzyl-5-fluoro-3''-methyl-2,5''-dioxo-1'',5'-diphenyl-1'',5''-dihydrodispiro [indoline-3,1'-cyclohexene-6',4''-pyrazol]-3'-yl)-2,2,2-trifluoroacetamide (3bi)</u>

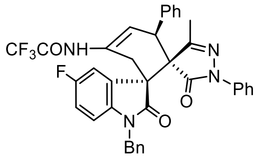

Prepared according to the general procedure, the crude product was purified by silica gel chromatography (petroleum ether/ethyl acetate 20:1) to afford **3bi** (58.7 mg, 90% yield, white solid, > 20:1 dr, m.p: 259.5 – 261.2 °C).

The enantiomeric excess was determined to be 97% by HPLC with a Daicel Chiralpak ID (*n*-hexane/2-propanol = 70:30, 1.0 mL/min, at 254 nm): t_R = 5.40 min (major), t_R = 6.52 min (minor); $[\alpha]_D^{20}$ = – 227.500 (*c* = 0.012, EA).

NMR and HRMS data for the product **3bi**:

^{1}H NMR (600 MHz, Chloroform-*d*) δ (ppm): 7.52 – 7.51 (m, 2H), 7.43 (s, 1H), 7.29 – 7.24 (m, 3H), 7.18 (s, 5H), 7.12 (t, *J* = 7.2 Hz, 1H), 7.06 (t, *J* = 7.2 Hz, 1H), 7.03 – 6.99 (m, 3H), 6.91 (t, *J* = 7.8 Hz, 2H), 6.80 (s, 1H), 6.67 (dd, *J* = 9.0, 4.2 Hz, 1H), 5.08 (d, *J* = 15.6 Hz, 1H), 4.54 (s, 1H), 4.44 (d, *J* = 15.6 Hz, 1H), 3.53 (d, *J* = 17.4 Hz, 1H), 2.53 (s, 3H), 2.37 (d, *J* = 18.0 Hz, 1H).

^{13}C NMR (150 MHz, Chloroform-*d*) δ (ppm): 174.0, 170.2, 159.6 (d, J_{CF} = 240.0 Hz), 158.6, 155.3 (q, *J* = 37.5 Hz), 138.3 (d, J_{CF} = 1.8 Hz), 137.2, 135.7, 134.5, 131.3 (d, J_{CF} = 8.1 Hz), 130.1, 128.8, 128.6, 128.6, 128.3, 128.1, 127.7, 126.8, 125.3, 119.5, 118.7, 115.5 (q, *J* = 287.0 Hz), 115.6 (d, J_{CF} = 23.1 Hz), 112.9 (d, J_{CF} = 25.4 Hz), 109.9 (d, J_{CF} = 8.1 Hz), 60.3, 51.4, 45.5, 44.3, 33.8, 18.8.

^{19}F NMR (564 MHz, Chloroform-*d*) δ (ppm): −75.54, −119.06 – −119.10.

HRMS (ESI-TOF) m/z: $[M+Na]^+$ Calcd for $C_{37}H_{28}F_4N_4NaO_3^+$ 675.1990, Found: 675.1983.

***N*-((3*R*,5'*S*,3'*S*)-1-benzyl-5-chloro-3''-methyl-2,5''-dioxo-1'',5'-diphenyl-1'',5''-dihydrodispiro [indoline-3,1'-cyclohexene-6',4''-pyrazol]-3'-yl)-2,2,2-trifluoroacetamide (3bj)**

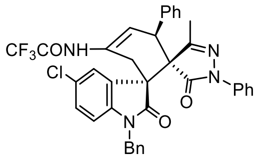

Prepared according to the general procedure, the crude product was purified by silica gel chromatography (petroleum ether/ethyl acetate 20:1) to afford **3bj** (60.2 mg, 90% yield, white solid, > 20:1 dr, m.p: 244.5 – 248.5 °C).

The enantiomeric excess was determined to be 96% by HPLC with a Daicel Chiralpak ID (*n*-hexane/2-propanol = 70:30, 1.0 mL/min, at 254 nm): t_R = 4.95 min (major), t_R = 6.50 min (minor); $[\alpha]_D^{20}$ = – 601.429 (*c* = 0.14, EA).

NMR and HRMS data for the product **3bj**:

^{1}H NMR (600 MHz, Chloroform-*d*) δ (ppm): 7.59 (s, 1H), 7.51 (d, *J* = 7.8 Hz, 2H), 7.47 (s, 1H), 7.29 – 7.25 (m, 3H), 7.18 (s, 5H), 7.13 (t, *J* = 7.2 Hz, 1H), 7.06 (t, *J* = 7.2 Hz, 1H), 6.99 (d, *J* = 7.8 Hz, 2H), 6.90 (t, *J* = 7.8 Hz, 2H), 6.79 (s, 1H), 6.68 (d, *J* = 8.4 Hz, 1H), 5.07 (d, *J* = 16.2 Hz, 1H), 4.52 (s, 1H), 4.44 (d, *J* = 15.6 Hz, 1H), 3.52 (d, *J* = 18.0 Hz, 1H), 2.53 (s, 3H), 2.36 (d, *J* = 18.0 Hz, 1H).

^{13}C NMR (150 MHz, Chloroform-*d*) δ (ppm): 173.9, 170.2, 158.5, 155.4 (q, *J* = 37.5 Hz), 140.9, 137.1, 135.7, 134.3, 131.5, 130.1, 129.3, 129.0, 128.9, 128.8, 128.6, 128.3, 128.1, 127.7, 126.8, 125.3, 124.9, 119.5, 118.8, 115.5 (q, *J* = 287.1 Hz), 110.3, 60.3, 51.2, 45.5, 44.2, 33.8, 18.7.

^{19}F NMR (564 MHz, Chloroform-*d*) δ (ppm): −75.51.

HRMS (ESI-TOF) m/z: $[M+Na]^+$ Calcd for $C_{37}H_{28}ClF_3N_4NaO_3^+$ 691.1694, Found: 691.1690.

<u>*N*-((3*R*,5'*S*,6'*S*)-1-benzyl-5-bromo-3''-methyl-2,5''-dioxo-1'',5'-diphenyl-1'',5''-dihydrodispiro [indoline-3,1'-cyclohexene-6',4''-pyrazol]-3'-yl)-2,2,2-trifluoroacetamide (3bk)</u>

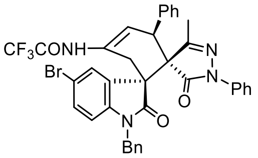

Prepared according to the general procedure, the crude product was purified by silica gel chromatography (petroleum ether/ethyl acetate 20:1) to afford **3bk** (64.3 mg, 90% yield, white solid, > 20:1 dr, m.p: 275.7 – 276.6 °C).

The enantiomeric excess was determined to be 99% by HPLC with a Daicel Chiralpak IB (*n*-hexane/2-propanol = 90:10, 1.0 mL/min, at 254 nm): t_R = 11.90 min (major), t_R = 13.50 min (minor); $[\alpha]_D^{20}$ = + 791.000 (*c* = 0.10, EA).

NMR and HRMS data for the product **3bk**:

^{1}H NMR (600 MHz, Chloroform-*d*) δ (ppm): 7.59 (d, *J* = 1.8 Hz, 1H), 7.54 (s, 1H), 7.51 (d, *J* = 7.8 Hz, 2H), 7.43 (dd, *J* = 8.4, 1.8 Hz, 1H), 7.27 – 7.25 (m, 2H), 7.20 – 7.17 (m, 5H), 7.13 (t, *J* = 7.2 Hz, 1H), 7.06 (t, *J* = 7.2 Hz, 1H), 6.99 (d, *J* = 7.2 Hz, 2H), 6.90 (t, *J* = 7.8 Hz, 2H), 6.78 (s, 1H), 6.63 (d, *J* = 8.4 Hz, 1H), 5.06 (d, *J* = 15.6 Hz, 1H), 4.51 (s, 1H), 4.43 (d, *J* = 15.6 Hz, 1H), 3.52 (d, *J* = 18.0 Hz, 1H), 2.53 (s, 3H), 2.35 (d, *J* = 18.0 Hz, 1H).

^{13}C NMR (150 MHz, Chloroform-*d*) δ (ppm): 173.8, 170.2, 158.5, 155.4 (q, *J* = 37.7 Hz), 141.4, 137.1, 135.7, 134.3, 132.2, 131.9, 130.1, 128.8, 128.6, 128.4, 128.3, 128.1, 127.7, 127.5, 126.8, 125.3, 119.5, 118.9, 116.3, 115.5 (q, *J* = 287.0 Hz), 110.7, 60.3, 51.2, 45.5, 44.2, 33.8, 18.7.

^{19}**F NMR** (564 MHz, Chloroform-*d*) δ (ppm): −75.68.

HRMS (ESI-TOF) m/z: $[M+Na]^+$ Calcd for $C_{37}H_{28}BrF_3N_4NaO_3^+$ 735.1189, Found: 735.1179.

_N_-((3*R*,5'*S*,6'*S*)-1-benzyl-3'',5-dimethyl-2,5''-dioxo-1'',5'-diphenyl-1'',5''-dihydrodispiro[indoline-3,1'-cyclohexene-6',4''-pyrazol]-3'-yl)-2,2,2-trifluoroacetamide (3bl)

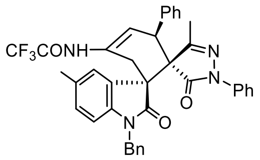

Prepared according to the general procedure, the crude product was purified by silica gel chromatography (petroleum ether/ethyl acetate 20:1) to afford **3bl** (63.2 mg, 97% yield, white solid, 16:1 dr, m.p: 242.5 – 246.4 °C).

The enantiomeric excess was determined to be 99% by HPLC with a Daicel Chiralpak IB (*n*-hexane/2-propanol = 80:20, 1.0 mL/min, at 254 nm): t_R = 5.22 min (major), t_R = 6.55 min (minor); $[\alpha]_D^{20}$ = – 758.000 (*c* = 0.11, EA).

NMR and HRMS data for the product **3bl**:

^{1}**H NMR** (600 MHz, Chloroform-*d*) δ (ppm): 7.53 (d, *J* = 7.8 Hz, 2H), 7.50 (s, 1H), 7.32 (s, 1H), 7.26 – 7.23 (m, 2H), 7.21 – 7.20 (m, 2H), 7.17 – 7.16 (m, 3H), 7.12 – 7.09 (m, 2H), 7.03 (d, *J* = 7.2 Hz, 1H), 7.00 (d, *J* = 7.2 Hz, 2H), 6.90 (s, 1H), 6.88 (t, *J* = 7.8 Hz, 2H), 6.66 (d, *J* = 7.8 Hz, 1H), 5.05 (d, *J* = 15.6 Hz, 1H), 4.60 (s, 1H), 4.45 (d, *J* = 15.6 Hz, 1H), 3.47 (d, *J* = 17.4 Hz, 1H), 2.53 (s, 3H), 2.40 (s, 3H), 2.29 (d, *J* = 17.4 Hz, 1H).

^{13}**C NMR** (150 MHz, Chloroform-*d*) δ (ppm): 174.3, 170.3, 158.8, 155.3 (q, *J* = 37.4 Hz), 139.9, 137.3, 136.2, 134.9, 132.9, 130.1, 129.9, 129.7, 128.6, 128.5, 128.5, 128.2, 128.1, 127.5, 126.8, 125.3, 125.1, 119.4, 118.6, 115.5 (q, *J* = 287.1 Hz), 109.1, 60.4, 51.2, 45.5, 44.1, 34.1, 21.6, 18.8.

^{19}**F NMR** (564 MHz, Chloroform-*d*) δ (ppm): −75.55.

HRMS (ESI-TOF) m/z: $[M+Na]^+$ Calcd for $C_{38}H_{31}F_3N_4NaO_3^+$ 671.2240, Found: 671.2250.

_N_-((3*R*,5'S,6'*S*)-1-benzyl-5-methoxy-3''-methyl-2,5''-dioxo-1'',5'-diphenyl-1'',5''-dihydrodispiro [indoline-3,1'-cyclohexene-6',4''-pyrazol]-3'-yl)-2,2,2-trifluoroacetamide (3bm)

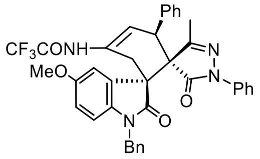

Prepared according to the general procedure, the crude product was purified by silica gel chromatography (petroleum ether/ethyl acetate 20:1) to afford **3bm** (60.2 mg, 90% yield, white solid, 15:1 dr, m.p: 286.2 – 291.2 °C).

The enantiomeric excess was determined to be 99% by HPLC with a Daicel Chiralpak IB (*n*-hexane/2-propanol = 80:20, 1.0 mL/min, at 254 nm): t_R = 6.85 min (major), t_R = 7.88 min (minor); $[\alpha]_D^{20}$ = – 311.000 (*c* = 0.10, EA).

NMR and HRMS data for the product **3bm**:

^{1}H NMR (600 MHz, Chloroform-*d*) δ (ppm): 7.53 (d, *J* = 7.8 Hz, 2H), 7.47 (s, 1H), 7.26 – 7.23 (m, 2H), 7.18 – 7.16 (m, 6H), 7.11 (t, *J* = 7.2 Hz, 1H), 7.05 (t, *J* = 7.2 Hz, 1H), 7.01 (d, *J* = 7.5 Hz, 2H), 6.88 (t, *J* = 7.8 Hz, 2H), 6.82 – 6.80 (m, 2H), 6.65 (d, *J* = 8.4 Hz, 1H), 5.06 (d, *J* = 15.6 Hz, 1H), 4.59 (s, 1H), 4.43 (d, *J* = 15.6 Hz, 1H), 3.80 (s, 3H), 3.52 – 3.48 (m, 1H), 2.53 (s, 3H), 2.33 (d, *J* = 17.4 Hz, 1H).

^{13}C NMR (150 MHz, Chloroform-*d*) δ (ppm): 173.9, 170.3, 158.8, 156.3, 155.3 (q, *J* = 37.4 Hz), 137.3, 136.0, 135.8, 134.9, 131.3, 130.2, 128.7, 128.5, 128.5, 128.2, 127.5, 126.8, 125.1, 119.5, 118.8, 118.8, 115.5 (q, *J* = 287.1 Hz), 113.3, 111.9, 109.4, 60.4, 55.7, 51.4, 45.4, 44.2, 34.0, 18.8.

^{19}F NMR (564 MHz, Chloroform-*d*) δ (ppm): −75.54.

HRMS (ESI-TOF) m/z: $[M+Na]^+$ Calcd for $C_{38}H_{31}F_3N_4NaO_4^+$ 687.2190, Found: 687.2194.

<u>*N*-((3*R*,5'*S*,6'*S*)-1-allyl-3''-methyl-2,5''-dioxo-1'',5'-diphenyl-1'',5''-dihydrodispiro[indoline-3,1'-cyclohexene-6',4''-pyrazol]-3'-yl)-2,2,2-trifluoroacetamide (3bn)</u>

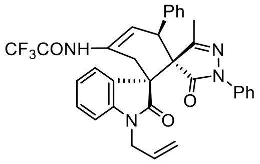

Prepared according to the general procedure, the crude product was purified by silica gel chromatography (petroleum ether/ethyl acetate 20:1) to afford **3bn** (53.1 mg, 91% yield, white solid, 15:1 dr, m.p: 225.8 – 226.2°C).

The enantiomeric excess was determined to be 99% by HPLC with a Daicel Chiralpak IG (*n*-hexane/2-propanol = 90:10, 1.0 mL/min, at 254 nm): t_R = 24.99 min (major), t_R = 27.83 min (minor); $[\alpha]_D^{20}$ = 190.182 (*c* = 0.11, EA).

NMR and HRMS data for the product **3bn***:*

^{1}H NMR (600 MHz, Chloroform-*d*) δ (ppm): 7.54 (d, *J* = 7.2 Hz, 1H), 7.52 (s, 1H), 7.48 (d, *J* = 8.4 Hz, 2H), 7.40 (t, *J* = 7.8 Hz, 1H), 7.24 – 7.20 (m, 3H), 7.18 – 7.14 (m, 5H), 7.08 (t, *J* = 7.2 Hz, 1H), 6.88 (s,

1H), 6.85 (d, J = 7.8 Hz, 1H), 5.55 (ddt, J = 15.7, 10.1, 5.0 Hz, 1H), 5.02 (d, J = 16.8 Hz, 1H), 4.86 (d, J = 10.2 Hz, 1H), 4.55 (s, 1H), 4.34 (dd, J = 16.2, 4.8 Hz, 1H), 3.99 (dd, J = 16.2, 4.8 Hz, 1H), 3.44 (d, J = 17.4 Hz, 1H), 2.49 (s, 3H), 2.28 (d, J = 17.4 Hz, 1H).

^{13}C NMR (150 MHz, Chloroform-*d*) δ (ppm): 173.8, 170.3, 158.5, 155.3 (q, J = 37.4 Hz), 142.2, 137.2, 136.1, 130.2, 130.1, 129.7, 129.3, 128.5, 128.4, 128.1, 128.1, 125.2, 124.5, 123.4, 119.4, 118.5, 117.8, 115.5 (q, J = 287.1 Hz), 109.2, 60.5, 51.2, 45.2, 42.4, 33.7, 18.7.

^{19}F NMR (564 MHz, Chloroform-*d*) δ (ppm): -75.51.

HRMS (ESI-TOF) m/z: $[M+Na]^+$ Calcd for $C_{33}H_{27}F_3N_4NaO_3^+$ 607.1927, Found: 607.1938.

2,2,2-trifluoro-*N*-((3*R*,5'*S*,6'*S*)-3''-methyl-2,5''-dioxo-1'',3'-diphenyl-1-(prop-2-yn-1-yl)-1'',5''-dihydrodispiro[indoline-3,1'-cyclohexene-6',4''-pyrazol]-3'-yl)acetamide (3bo)

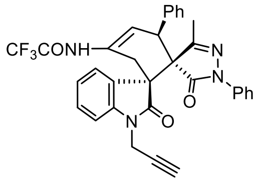

Prepared according to the general procedure, the crude product was purified by silica gel chromatography (petroleum ether/ethyl acetate 20:1) to afford **3bo** (52.5 mg, 90% yield, white solid, > 20:1 dr, m.p: 152.1 – 152.9 °C).

The enantiomeric excess was determined to be 99% by HPLC with a Daicel Chiralpak IG (*n*-hexane/2-propanol = 70:30, 1.0 mL/min, at 254 nm): t_R = 7.12 min (major), t_R = 8.56 min (minor); $[\alpha]_D^{20}$ = – 529.429 (*c* = 0.06, EA).

NMR and HRMS data for the product **3bo**:

^{1}H NMR (600 MHz, Chloroform-*d*) δ (ppm): 7.55 (d, J = 7.2 Hz, 2H), 7.46 – 7.44 (m, 3H), 7.25 – 7.20 (m, 3H), 7.18 – 7.15 (m, 5H), 7.07 (t, J = 7.8 Hz, 1H), 7.04 (d, J = 7.8 Hz, 1H), 6.89 (s, 1H), 4.54 (s, 1H), 4.32 (d, J = 2.4 Hz, 2H), 3.44 – 3.40 (m, 1H), 2.48 (s, 3H), 2.30 (d, J = 18.0 Hz, 1H), 1.84 (t, J = 2.4 Hz, 1H).

^{13}C NMR (150 MHz, Chloroform-*d*) δ (ppm): 173.3, 170.0, 158.3, 155.4 (q, J = 37.7 Hz), 141.0, 137.2, 136.1, 129.9, 129.6, 129.4, 128.5, 128.4, 128.2, 128.1, 125.2, 124.7, 123.8, 119.7, 118.5, 115.5 (q, J = 287.1 Hz), 109.3, 76.2, 72.4, 60.4, 51.2, 45.2, 33.5, 29.5, 18.7.

^{19}F NMR (564 MHz, Chloroform-*d*) δ (ppm): -75.54.

HRMS (ESI-TOF) m/z: $[M+Na]^+$ Calcd for $C_{33}H_{25}F_3N_4NaO_3^+$ 605.1771, Found: 605.1781.

2,2,2-trifluoro-*N*-((1*R*,5'*S*,6'*S*)-3''-methyl-2,5''-dioxo-1'',5'-diphenyl-1'',5''-dihydro-2*H*-dispiro

[acenaphthylene-1,1'-cyclohexane-6',4''-pyrazol]-3'-yl)acetamide (3bp)

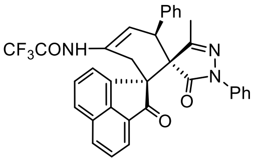

Prepared according to the general procedure, the crude product was purified by silica gel chromatography (petroleum ether/ethyl acetate 20:1) to afford **3bp** (54.0 mg, 93% yield, white solid, 16:1 dr, m.p: 186.3 – 186.7 °C).

The enantiomeric excess was determined to be 99% by HPLC with a Daicel Chiralpak IG (*n*-hexane/2-propanol = 70:30, 1.0 mL/min, at 254 nm): t_R = 8.49 min (major), t_R = 16.64 min (minor); $[\alpha]_D^{20}$ = – 217.500 (*c* = 0.10, EA).

NMR and HRMS data for the product **3bp**:

^{1}H NMR (600 MHz, Chloroform-*d*) δ (ppm): 8.17 (d, *J* = 8.4 Hz, 1H), 7.97 (dd, *J* = 7.8, 1.8 Hz, 1H), 7.85 (d, *J* = 6.6 Hz, 1H), 7.77 – 7.73 (m, 2H), 7.68 (t, *J* = 7.8 Hz, 1H), 7.51 (s, 1H), 7.18 – 7.15 (m, 7H), 7.13 – 7.11 (m 2H), 7.00 (t, *J* = 7.8 Hz, 1H), 6.92 (s, 1H), 4.64 (s, 1H), 3.52 (d, *J* = 18.6, 1H), 2.56 (s, 3H), 2.35 (d, *J* = 19.2 Hz, 1H).

^{13}C NMR (150 MHz, Chloroform-*d*) δ (ppm): 200.5, 170.3, 159.7, 155.3 (q, *J* = 37.5 Hz), 141.6, 139.6, 136.9, 136.4, 133.1, 131.0, 130.7, 130.6, 128.9, 128.5, 128.4, 128.2, 128.1, 128.0, 125.6, 125.2, 122.8, 121.7, 119.6, 118.9, 115.5 (q, *J* = 287.1 Hz), 61.0, 56.7, 46.2, 33.8, 18.8.

^{19}F NMR (564 MHz, Chloroform-*d*) δ (ppm): -75.56.

HRMS (ESI-TOF) m/z: $[M+Na]^+$ Calcd for $C_{34}H_{24}F_3N_3NaO_3^+$ 602.1662, Found: 602.1668.

4. General procedure for the synthesis of product 4

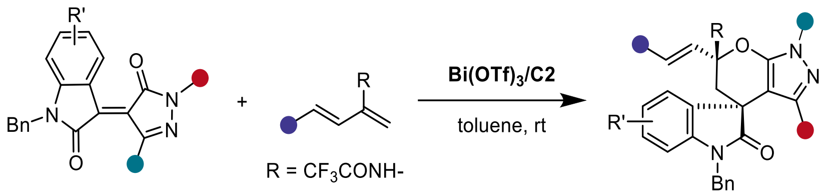

1 **2** **4**

Procedure for synthesis of product **4**

Bi(OTf)$_3$ (10 mol%, 6.6 mg) and (*R*)-**C2** (10 mol%, 7.5 mg) were added to a Schlenk tube, followed by toluene (2.0 mL) under argon. The solution was stirred at room temperature for 0.5 h. Subsequently, **1** (0.1 mmol, 1.0 equiv.) and **2** (0.15 mmol, 1.5 equiv.) were introduced. The reaction proceeded for 5–10 min at room temperature and was monitored by TLC. Upon completion, the mixture was concentrated under reduced pressure, and the crude material was purified *via* column chromatography on silica gel using petroleum ether and ethyl acetate (30/1) as eluents, yielding product **4**. All products were dried under vacuum and further analyzed by ^{1}H NMR, ^{13}C NMR, HRMS and chiral HPLC analysis, *etc*.

Procedure for synthesis of product ***ent*-4**

Bi(OTf)$_3$ (10 mol%, 6.6 mg) and (*S*)-**C2** (10 mol%, 7.5 mg) were added to a Schlenk tube, followed by toluene (2.0 mL) under argon. The solution was stirred at room temperature for 0.5 h. Subsequently, **1** (0.1 mmol, 1.0 equiv.) and **2** (0.15 mmol, 1.5 equiv.) were introduced. The reaction proceeded for 5–10 min at room temperature and was monitored by TLC. Upon completion, the mixture was concentrated under reduced pressure, and the crude material was purified *via* column chromatography on silica gel using petroleum ether and ethyl acetate (30/1) as eluents, yielding product ***ent*-4**. All products were dried under vacuum and further analyzed by ^{1}H NMR, ^{13}C NMR, HRMS and chiral HPLC analysis, *etc*.

<u>*N*-((3*R*,6'*R*)-1-benzyl-3'-methyl-6'-((*E*)-2-methylstyryl)-2-oxo-1'-phenyl-5',6'-dihydro-1'*H*-spiro[indoline-3,4'-pyrano[2,3-*c*]pyrazol]-6'-yl)-2,2,2-trifluoroacetamide (4aa)</u>

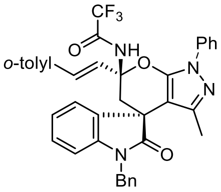

Prepared according to the general procedure, the crude product was purified by silica gel chromatography (petroleum ether/ethyl acetate 30:1) to afford **4aa** (52.0 mg, 80% yield, white solid, > 20:1 dr, m.p: 187.6 – 192.5 °C).

The enantiomeric excess was determined to be 99% by HPLC with a Daicel Chiralpak IB (*n*-hexane/2-propanol = 90:10, 1.0 mL/min, at 254 nm): t_R = 10.03 min (minor), t_R = 12.05 min (major); $[\alpha]_D^{20}$ = + 571.286 (*c* = 0.14, EA).

NMR and HRMS data for the product **4aa**:

^{1}H NMR (600 MHz, Chloroform-*d*) δ (ppm): 11.70 (s, 1H), 7.83 (d, *J* = 7.8 Hz, 2H), 7.47 – 7.43 (m, 3H), 7.42 (d, *J* = 7.8 Hz, 2H), 7.38 (t, *J* = 7.8 Hz, 2H), 7.33 (t, *J* = 7.2 Hz, 1H), 7.30 – 7.24 (m, 2H), 7.20 – 7.17 (m, 2H), 7.16 – 7.13 (m, 2H), 7.12 – 7.10 (m, 2H), 6.94 (d, *J* = 7.8 Hz, 1H), 6.50 (d, *J* = 15.6 Hz, 1H), 5.17 (d, *J* = 15.0 Hz, 1H), 4.90 (d, *J* = 15.0 Hz, 1H), 2.62 (d, *J* = 15.6 Hz, 1H), 2.58 (d, *J* = 15.0 Hz, 1H), 2.36 (s, 3H), 1.46 (s, 3H).

^{13}C NMR (150 MHz, Chloroform-*d*) δ (ppm): 179.8, 156.9 (q, *J* = 37.8 Hz), 148.9, 145.7, 142.4, 138.1, 136.1, 135.0, 134.8, 131.3, 130.4, 130.1, 129.3, 129.2, 129.1, 128.6, 128.4, 128.3, 127.9, 126.2, 126.1, 126.1, 124.4, 123.6, 120.4, 115.5 (q, *J* = 287.3 Hz), 109.8, 95.9, 88.6, 46.2, 44.7, 41.9, 19.7, 12.5.

^{19}F NMR (564 MHz, Chloroform-*d*) δ (ppm): −75.77.

HRMS (ESI-TOF) m/z: $[M+Na]^+$ Calcd for $C_{38}H_{31}F_3N_4NaO_3^+$ 671.2240, Found: 671.2239.

<u>*N*-((3*R*,6'*R*)-1-benzyl-3'-methyl-2-oxo-1'-phenyl-6'-((*E*)-styryl)-5',6'-dihydro-1'*H*-spiro[indoline-3,4'-pyrano[2,3-*c*]pyrazol]-6'-yl)-2,2,2-trifluoroacetamide (4ab)</u>

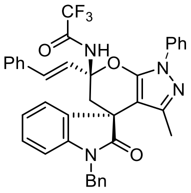

Prepared according to the general procedure, the crude product was purified by silica gel chromatography (petroleum ether/ethyl acetate 30:1) to afford **4ab** (31.8 mg, 50% yield, white solid, > 20:1 dr, m.p: 160.4 – 164.4 °C).

The enantiomeric excess was determined to be 97% by HPLC with a Daicel Chiralpak IG (*n*-hexane/2-propanol = 80:20, 1.0 mL/min, at 254 nm): t_R = 7.18 min (major), t_R = 8.06 min (minor); $[\alpha]_D^{20}$ = + 355.000 (*c* = 0.12, EA).

NMR and HRMS data for the product **4ab**:

^{1}H NMR (600 MHz, Chloroform-*d*) δ (ppm): 11.72 (s, 1H), 7.81 (d, *J* = 7.8 Hz, 2H), 7.46 (t, *J* = 7.8 Hz, 2H), 7.44 – 7.41 (m, 4H), 7.38 (t, *J* = 7.8 Hz, 2H), 7.35 – 7.32 (m, 3H), 7.30 – 7.25 (m, 3H), 7.11 – 7.09 (m, 2H), 6.93 (d, *J* = 7.8 Hz, 1H), 6.87 (d, *J* = 16.2 Hz, 1H), 6.70 (d, *J* = 16.2 Hz, 1H), 5.15 (d, *J* = 15.0 Hz, 1H), 4.92 (d, *J* = 15.6 Hz, 1H), 2.63 (d, *J* = 15.6 Hz, 1H), 2.58 (d, *J* = 15.0 Hz, 1H), 1.46 (s, 3H).

^{13}C NMR (150 MHz, Chloroform-*d*) δ (ppm): 179.8, 156.9 (q, J = 37.8 Hz), 148.9, 145.7, 142.4, 138.1, 135.4, 135.0, 131.8, 131.3, 129.3, 129.3, 129.1, 128.7, 128.5, 128.3, 127.9, 127.6, 127.0, 126.1, 124.4, 123.6, 120.5, 115.5 (q, J = 287.4 Hz), 109.8, 95.9, 88.6, 46.2, 44.6, 41.9, 12.5.

^{19}F NMR (564 MHz, Chloroform-*d*) δ (ppm): −75.68.

HRMS (ESI-TOF) m/z: $[M+Na]^+$ Calcd for $C_{37}H_{29}F_3N_4NaO_3^+$ 657.2084, Found: 657.2086.

<u>*N*-((3*R*,6'*R*)-1-benzyl-6'-((*E*)-2-chlorostyryl)-3'-methyl-2-oxo-1'-phenyl-5',6'-dihydro-1'*H*-spiro[indoline-3,4'-pyrano[2,3-*c*]pyrazol]-6'-yl)-2,2,2-trifluoroacetamide (4ac)</u>

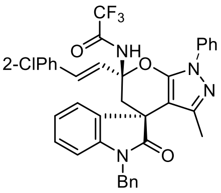

Prepared according to the general procedure, the crude product was purified by silica gel chromatography (petroleum ether/ethyl acetate 30:1) to afford **4ac** (46.8 mg, 70% yield, white solid, > 20:1 dr, m.p: 203.6 – 209.3 °C).

The enantiomeric excess was determined to be 99% by HPLC with a Daicel Chiralpak IB (*n*-hexane/2-propanol = 80:20, 1.0 mL/min, at 254 nm): t_R = 7.38 min (minor), t_R = 8.44 min (major); $[\alpha]_D^{20}$ = + 838.200 (c = 0.10, EA).

NMR and HRMS data for the product **4ac**:

^{1}H NMR (600 MHz, Chloroform-*d*) δ (ppm): 11.78 (s, 1H), 7.82 (d, J = 7.8 Hz, 2H), 7.63 – 7.62 (m, 1H), 7.46 (t, J = 7.8 Hz, 2H), 7.43 (d, J = 7.2 Hz, 2H), 7.40 – 7.36 (m, 3H), 7.34 (t, J = 7.2 Hz, 1H), 7.29 (t, J = 7.8 Hz, 2H), 7.27 – 7.22 (m, 3H), 7.15 – 7.11 (m, 2H), 6.94 (d, J = 8.4 Hz, 1H), 6.72 (d, J = 16.2 Hz, 1H), 5.18 (d, J = 15.0 Hz, 1H), 4.91 (d, J = 15.0 Hz, 1H), 2.66 (d, J = 15.6 Hz, 1H), 2.61 (d, J = 15.6 Hz, 1H), 1.46 (s, 3H).

^{13}C NMR (150 MHz, Chloroform-*d*) δ (ppm): 179.7, 157.0 (q, J = 37.8 Hz), 148.8, 145.7, 142.4, 138.1, 135.0, 133.8, 133.6, 131.2, 130.6, 129.7, 129.5, 129.3, 129.3, 129.1, 128.3, 128.2, 127.9, 127.4, 127.1, 126.1, 124.4, 123.7, 120.5, 115.5 (q, J = 287.4 Hz), 109.9, 95.8, 88.5, 46.2, 44.7, 41.7, 12.5.

^{19}F NMR (564 MHz, Chloroform-*d*) δ (ppm): −75.71.

HRMS (ESI-TOF) m/z: $[M+Na]^+$ Calcd for $C_{37}H_{28}ClF_3N_4NaO_3^+$ 691.1694, Found: 691.1691.

<u>*N*-((3*R*,6'*R*)-1-benzyl-6'-((*E*)-2-methoxystyryl)-3'-methyl-2-oxo-1'-phenyl-5',6'-dihydro-1'*H*-spiro[indoline-3,4'-pyrano[2,3-*c*]pyrazol]-6'-yl)-2,2,2-trifluoroacetamide (4ad)</u>

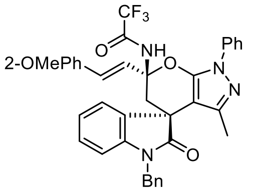

Prepared according to the general procedure, the crude product was purified by silica gel chromatography (petroleum ether/ethyl acetate 30:1) to afford **4ad** (55.0 mg, 83% yield, white solid, > 20:1 dr, m.p: 194.8 – 195.2 °C).

The enantiomeric excess was determined to be 99% by HPLC with a Daicel Chiralpak IG (*n*-hexane/2-propanol = 90:10, 1.0 mL/min, at 254 nm): t_R = 12.73 min (major), t_R = 14.17 min (minor); $[\alpha]_D^{20}$ = + 455.000 (*c* = 0.12, EA).

NMR and HRMS data for the product **4ad**:

^{1}H NMR (600 MHz, Chloroform-*d*) δ (ppm): 11.68 (s, 1H), 7.83 (d, *J* = 7.8 Hz, 2H), 7.51 (d, *J* = 7.2 Hz, 1H), 7.45 (t, *J* = 7.8 Hz, 2H), 7.42 (d, *J* = 7.2 Hz, 2H), 7.38 (t, *J* = 7.8 Hz, 2H), 7.33 (t, *J* = 7.2 Hz, 1H), 7.29 – 7.23 (m, 3H), 7.16 (d, *J* = 16.2 Hz, 1H), 7.13 – 7.09 (m, 2H), 6.96 – 6.92 (m, 2H), 6.87 (d, *J* = 8.4 Hz, 1H), 6.78 (d, *J* = 16.8 Hz, 1H), 5.16 (d, *J* = 15.0 Hz, 1H), 4.91 (d, *J* = 15.0 Hz, 1H), 3.83 (s, 3H), 2.65 (d, *J* = 15.6 Hz, 1H), 2.61 (d, *J* = 15.0 Hz, 1H), 1.46 (s, 3H).

^{13}C NMR (150 MHz, Chloroform-*d*) δ (ppm): 179.8, 157.1, 156.7 (q, *J* = 37.5 Hz), 149.0, 145.7, 142.4, 138.1, 135.1, 131.4, 129.6, 129.3, 129.2, 129.0, 128.3, 127.9, 127.9, 127.5, 126.8, 126.1, 124.5, 124.3, 123.7, 120.8, 120.5, 115.6 (q, *J* = 287.6 Hz), 111.0, 109.8, 95.9, 89.0, 55.6, 46.2, 44.6, 41.8, 12.5.

^{19}F NMR (564 MHz, Chloroform-*d*) δ (ppm): -75.71.

HRMS (ESI-TOF) m/z: $[M+Na]^+$ Calcd for $C_{38}H_{31}F_3N_4NaO_4^+$ 687.2190, Found: 687.2194.

<u>*N*-((3*R*,6'*R*)-6'-((*E*)-2-(allyloxy)styryl)-1-benzyl-3'-methyl-2-oxo-1'-phenyl-5',6'-dihydro-1'*H*-spiro[indoline-3,4'-pyrano[2,3-*c*]pyrazol]-6'-yl)-2,2,2-trifluoroacetamide (4ae)</u>

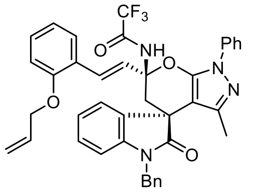

Prepared according to the general procedure, the crude product was purified by silica gel chromatography (petroleum ether/ethyl acetate 30:1) to afford **4ae** (50.0 mg, 72% yield, white solid, > 20:1 dr, m.p: 155.1 – 155.5 °C).

The enantiomeric excess was determined to be 95% by HPLC with a Daicel Chiralpak IG (*n*-hexane/2-propanol = 70:30, 1.0 mL/min, at 254 nm): t_R = 4.84 min (minor), t_R = 6.09 min (major); $[\alpha]_D^{20}$ = + 216.250 (*c* = 0.08, EA).

NMR and HRMS data for the product **4ae:**

^{1}H NMR (600 MHz, Chloroform-*d*) δ (ppm): 11.66 (s, 1H), 7.83 (d, *J* = 8.4 Hz, 2H), 7.50 (dd, *J* = 7.8, 1.8 Hz, 1H), 7.45 (t, *J* = 7.8 Hz, 2H), 7.43 – 7.41 (m, 2H), 7.38 (t, *J* = 7.8 Hz, 2H), 7.34 – 7.32 (m, 1H), 7.28 – 7.26 (m, 1H), 7.25 – 7.21 (m, 3H), 7.13 – 7.09 (m, 2H), 6.94 (q, *J* = 7.8 Hz, 2H), 6.87 (d, *J* = 7.8 Hz, 1H), 6.75 (d, *J* = 16.2 Hz, 1H), 6.07 – 6.01 (m, 1H), 5.39 (dd, *J* = 17.4, 1.8 Hz, 1H), 5.23 (dd, *J* = 10.2, 1.2 Hz, 1H), 5.16 (d, *J* = 15.0 Hz, 1H), 4.91 (d, *J* = 15.6 Hz, 1H), 4.56 (dt, *J* = 4.8, 1.8 Hz, 2H), 2.63 (d, *J* = 15.6 Hz, 1H), 2.59 (d, *J* = 15.6 Hz, 1H), 1.46 (s, 3H).

^{13}C NMR (150 MHz, Chloroform-*d*) δ (ppm): 179.8, 156.2, 156.8 (q, *J* = 37.7 Hz), 149.1, 145.7, 142.4, 138.1, 135.1, 133.2, 131.4, 129.5, 129.3, 129.2, 129.0, 128.3, 127.9, 127.8, 127.5, 126.9, 126.1, 124.9, 124.4, 123.7, 121.0, 120.5, 117.5, 115.6 (q, *J* = 287.6 Hz), 109.8, 95.9, 88.9, 69.3, 46.2, 44.7, 41.8, 12.5.

^{19}F NMR (564 MHz, Chloroform-*d*) δ (ppm): -75.71.

HRMS (ESI-TOF) m/z: $[M+Na]^+$ Calcd for $C_{40}H_{33}F_3N_4NaO_4^+$ 713.2346, Found: 713.2352.

<u>*N*-((3*R*,6'*R*)-1-benzyl-6'-((*E*)-3-ethynylstyryl)-3'-methyl-2-oxo-1'-phenyl-5',6'-dihydro-1'*H*-spiro[indoline-3,4'-pyrano[2,3-*c*]pyrazol]-6'-yl)-2,2,2-trifluoroacetamide (4af)</u>

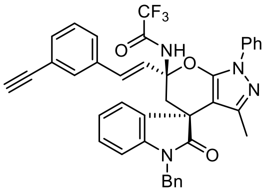

Prepared according to the general procedure, the crude product was purified by silica gel chromatography (petroleum ether/ethyl acetate 30:1) to afford **4af** (45.5 mg, 69% yield, white solid, > 20:1 dr, m.p: 170.2 – 171.0 °C).

The enantiomeric excess was determined to be 96% by HPLC with a Daicel Chiralpak IC (*n*-hexane/2-propanol = 70:30, 1.0 mL/min, at 254 nm): t_R = 5.19 min (major), t_R = 6.40 min (minor); $[\alpha]_D^{20}$ = + 236.800 (*c* = 0.05, EA).

NMR and HRMS data for the product **4af**:

^{1}H NMR (600 MHz, Chloroform-*d*) δ ppm): 11.73 (s, 1H), 7.79 (d, *J* = 7.8 Hz, 2H), 7.56 (s, 1H), 7.46 (t, *J* = 7.8 Hz, 2H), 7.42 – 7.39 (m, 5H), 7.38 – 7.37 (m, 2H), 7.34 (d, *J* = 7.2 Hz, 1H), 7.30 (d, *J* = 7.8 Hz, 1H), 7.27 – 7.25 (m, 1H), 7.11 – 7.10 (m, 2H), 6.93 (d, *J* = 7.8 Hz, 1H), 6.83 (d, *J* = 16.2 Hz, 1H), 6.69 (d,

J = 16.2 Hz, 1H), 5.14 (d, *J* = 15.0 Hz, 1H), 4.92 (d, *J* = 15.0 Hz, 1H), 3.07 (s, 1H), 2.61 (d, *J* = 15.6 Hz, 1H), 2.55 (d, *J* = 15.6 Hz, 1H), 1.46 (s, 3H).

^{13}C NMR (150 MHz, Chloroform-*d*) δ ppm): 179.7, 157.0 (q, *J* = 37.7 Hz), 148.8, 145.7, 142.4, 138.1, 135.7, 135.0, 132.0, 131.2, 130.9, 130.6, 129.3, 129.3, 129.1, 128.7, 128.6, 128.3, 127.9, 127.4, 126.2, 124.4, 123.7, 122.6, 120.6, 115.6 (q, *J* = 287.6 Hz), 109.9, 95.9, 88.4, 83.2, 77.5, 46.2, 44.7, 41.8, 12.6.

^{19}F NMR (564 MHz, Chloroform-*d*) δ ppm): -75.69.

HRMS (ESI-TOF) m/z: $[M+Na]^+$ Calcd for $C_{39}H_{29}F_3N_4NaO_3^+$ 681.2084, Found: 681.2085.

<u>*N*-((3*R*,6'*R*)-1-benzyl-3'-methyl-2-oxo-1'-phenyl-6'-((*E*)-4-(trifluoromethyl)styryl)-5',6'-dihydro-1'*H*-spiro[indoline-3,4'-pyrano[2,3-*c*]pyrazol]-6'-yl)-2,2,2-trifluoroacetamide (4ag)</u>

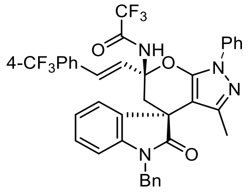

Prepared according to the general procedure, the crude product was purified by silica gel chromatography (petroleum ether/ethyl acetate 30:1) to afford **4ag** (52.9 mg, 75% yield, white solid, > 20:1 dr, m.p: 140.2 – 144.6 °C).

The enantiomeric excess was determined to be 98% by HPLC with a Daicel Chiralpak IB (*n*-hexane/2-propanol = 80:20, 1.0 mL/min, at 254 nm): t_R = 7.53 min (minor), t_R = 15.77 min (major); $[\alpha]_D^{20}$ = + 202.286 (*c* = 0.14, EA).

NMR and HRMS data for the product **4ag**:

^{1}H NMR (600 MHz, Chloroform-*d*) δ (ppm): 11.80 (s, 1H), 7.79 (d, *J* = 7.8 Hz, 2H), 7.59 (d, *J* = 8.4 Hz, 2H), 7.53 (d, *J* = 8.4 Hz, 2H), 7.46 (t, *J* = 7.8 Hz, 2H), 7.42 – 7.41 (m, 2H), 7.38 (t, *J* = 7.8 Hz, 2H), 7.34 – 7.32 (m, 1H), 7.30 – 7.25 (m, 2H), 7.12 – 7.10 (m, 2H), 6.94 – 6.90 (m, 2H), 6.77 (d, *J* = 16.2 Hz, 1H), 5.15 (d, *J* = 15.0 Hz, 1H), 4.92 (d, *J* = 15.0 Hz, 1H), 2.63 (d, *J* = 15.0 Hz, 1H), 2.56 (d, *J* = 15.0 Hz, 1H), 1.46 (s, 3H).

^{13}C NMR (150 MHz, Chloroform-*d*) δ (ppm): 179.7, 157.1 (q, *J* = 38.0 Hz), 148.7, 145.8, 142.4, 138.9, 138.1, 135.0, 131.1, 130.5, 130.3, 130.3 (q, *J* = 32.3 Hz), 129.4, 129.3, 129.1, 128.3, 127.9, 127.2, 126.2, 125.7 (q, *J* = 3.9 Hz), 124.4, 124.0 (q, *J* = 270.2 Hz), 123.6, 120.6, 115.5 (q, *J* = 287.3 Hz), 109.9, 95.8, 88.3, 46.2, 44.7, 41.7, 12.5.

^{19}F NMR (564 MHz, Chloroform-*d*) δ (ppm): –75.68, –62.62.

HRMS (ESI-TOF) m/z: $[M+Na]^+$ Calcd for $C_{38}H_{28}F_6N_4NaO_3^+$ 725.1958, Found:725.1958.

<u>*N*-((3*R*,6'*R*)-1-benzyl-3'-methyl-2-oxo-1'-phenyl-6'-((*E*)-4-((trimethylsilyl)ethynyl)styryl)-5',6'-dihydro-1'*H*-spiro[indoline-3,4'-pyrano[2,3-*c*]pyrazol]-6'-yl)-2,2,2-trifluoroacetamide (4ah)</u>

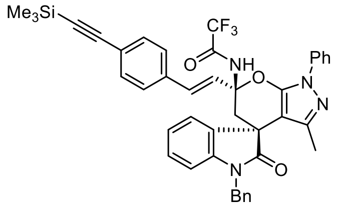

Prepared according to the general procedure, the crude product was purified by silica gel chromatography (petroleum ether/ethyl acetate 30:1) to afford **4ah** (48.0 mg, 66% yield, white solid, > 20:1 dr, m.p: 171.4 – 172.1 °C).

The enantiomeric excess was determined to be 98% by HPLC with a Daicel Chiralpak IC (*n*-hexane/2-propanol = 70:30, 1.0 mL/min, at 254 nm): t_R = 5.73 min (major), t_R = 7.16 min (minor); $[\alpha]_D^{20}$ = + 402.000 (*c* = 0.05, EA).

NMR and HRMS data for the product **4ah:**

^{1}H NMR (600 MHz, Chloroform-*d*) δ (ppm): 11.73 (s, 1H), 7.79 (d, *J* = 7.8 Hz, 2H), 7.46 (t, *J* = 7.8 Hz, 2H), 7.43 – 7.40 (m, 4H), 7.39 – 7.36 (m, 3H), 7.35 – 7.31 (m, 2H), 7.28 – 7.25 (m, 2H), 7.11 – 7.08 (m, 2H), 6.92 (d, *J* = 7.8 Hz, 1H), 6.83 (d, *J* = 16.2 Hz, 1H), 6.69 (d, *J* = 16.2 Hz, 1H), 5.14 (d, *J* = 15.0 Hz, 1H), 4.92 (d, *J* = 15.0 Hz, 1H), 2.61 (d, *J* = 15.6 Hz, 1H), 2.56 (d, *J* = 15.0 Hz, 1H), 1.46 (s, 3H), 0.25 (s, 9H).

^{13}C NMR (150 MHz, Chloroform-*d*) δ (ppm): 179.7, 157.0 (q, *J* = 37.9 Hz), 148.8, 145.7, 142.4, 138.1, 135.5, 135.0, 132.3, 131.2, 131.1, 129.3, 129.3, 129.1, 128.5, 128.3, 127.9, 126.8, 126.2, 124.4, 123.6, 123.1, 120.6, 115.5 (q, *J* = 287.1 Hz), 109.9, 104.9, 95.8, 95.4, 88.5, 46.2, 44.6, 41.9, 12.5, 0.03.

^{19}F NMR (564 MHz, Chloroform-*d*) δ (ppm): -75.68.

HRMS (ESI-TOF) m/z: $[M+Na]^+$ Calcd for $C_{42}H_{37}F_3N_4NaO_3Si^+$ 753.2479, Found: 753.2474.

<u>Methyl 4-((*E*)-2-((3*R*,6'*R*)-1-benzyl-3'-methyl-2-oxo-1'-phenyl-6'-(2,2,2-trifluoroacetamido)-5',6'-dihydro-1'*H*-spiro[indoline-3,4'-pyrano[2,3-*c*]pyrazol]-6'-yl)vinyl)benzoate (4ai)</u>

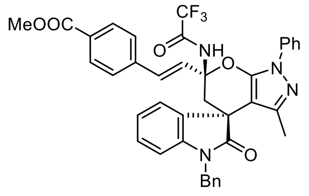

Prepared according to the general procedure, the crude product was purified by silica gel chromatography (petroleum ether/ethyl acetate 30:1) to afford **4ai** (45.0 mg, 65% yield, white solid, > 20:1 dr, m.p: 170.5 – 171.0 °C).

The enantiomeric excess was determined to be 91% by HPLC with a Daicel Chiralpak IG (*n*-hexane/2-propanol = 70:30, 1.0 mL/min, at 254 nm): t_R = 9.02 min (minor), t_R = 14.80 min (major); $[\alpha]_D^{20}$ = + 327.000 (*c* = 0.10, EA).

NMR and HRMS data for the product **4ai:**

^{1}H NMR (600 MHz, Chloroform-*d*) δ (ppm): 11.78 (s, 1H), 8.00 (d, *J* = 8.4 Hz, 2H), 7.79 (d, *J* = 8.4 Hz, 2H), 7.49 – 7.45 (m, 4H), 7.41 – 7.37 (m, 4H), 7.33 (t, *J* = 7.2 Hz, 1H), 7.29 – 7.25 (m, 2H), 7.10 (d, *J* = 6.6 Hz, 2H), 6.94 – 6.90 (m, 2H), 6.78 (d, *J* = 16.2 Hz, 1H), 5.14 (d, *J* = 15.0 Hz, 1H), 4.92 (d, *J* = 15.0 Hz, 1H), 3.91 (s, 3H), 2.62 (d, *J* = 15.6 Hz, 1H), 2.57 (d, *J* = 15.0 Hz, 1H), 1.46 (s, 3H).

^{13}C NMR (150 MHz, Chloroform-*d*) δ (ppm): 179.7, 166.7, 157.1 (q, *J* = 38.1 Hz), 148.7, 145.8, 142.4, 139.9, 138.0, 134.9, 131.1, 130.9, 130.2, 130.0, 129.9, 129.3, 129.1, 128.3, 127.9, 126.9, 126.2, 124.4, 123.6, 120.6, 115.5 (q, *J* = 287.4 Hz), 109.9, 95.9, 88.4, 52.2, 46.2, 44.7, 41.8, 12.5.

^{19}F NMR (564 MHz, Chloroform-*d*) δ (ppm): -75.67.

HRMS (ESI-TOF) m/z: $[M+Na]^+$ Calcd for $C_{39}H_{31}F_3N_4NaO_5^+$ 715.2139, Found: 715.2134.

<u>*N*-((3*R*,6'*R*)-1-benzyl-6'-((*E*)-2,4-dimethylstyryl)-3'-methyl-2-oxo-1'-phenyl-5',6'-dihydro-1'*H*-spiro[indoline-3,4'-pyrano[2,3-*c*]pyrazol]-6'-yl)-2,2,2-trifluoroacetamide (4aj)</u>

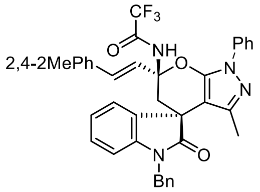

Prepared according to the general procedure, the crude product was purified by silica gel chromatography (petroleum ether/ethyl acetate 30:1) to afford **4aj** (59.7 mg, 90% yield, white solid, > 20:1 dr, m.p: 185.6 – 190.3 °C).

The enantiomeric excess was determined to be 91% by HPLC with a Daicel Chiralpak IB (*n*-hexane/2-propanol = 80:20, 1.0 mL/min, at 254 nm): t_R =5.87 min (minor), t_R = 7.41 min (major); $[\alpha]_D^{20}$ = + 245.000 (*c* = 0.10 EA).

NMR and HRMS data for the product **4aj**:

^{1}H NMR (600 MHz, Chloroform-*d*) δ (ppm): 11.71 (s, 1H), 7.83 (d, *J* = 7.8 Hz, 2H), 7.45 – 7.42 (m, 4H), 7.38 (t, *J* = 7.8 Hz, 2H), 7.33 (t, *J* = 7.2 Hz, 1H), 7.31 – 7.29 (m, 1H), 7.26 – 7.23 (m, 1H), 7.15 – 7.12 (m,

2H), 7.09 – 7.07 (m, 1H), 7.04 (d, *J* = 7.8 Hz, 2H), 6.96 (d, *J* = 7.8 Hz, 1H), 6.94 (s, 1H), 6.16 (d, *J* = 16.8 Hz, 1H), 5.17 (d, *J* = 15.6 Hz, 1H), 4.91 (d, *J* = 15.6 Hz, 1H), 2.65 (d, *J* = 15.6 Hz, 1H), 2.59 (d, *J* = 15.0 Hz, 1H), 2.33 (s, 6H), 1.46 (s, 3H).

^{13}C NMR (150 MHz, Chloroform-*d*) δ (ppm): 179.8, 157.0 (q, *J* = 37.7 Hz), 148.9, 145.7, 142.5, 138.1, 136.1, 135.1, 135.0, 132.4, 131.4, 130.5, 129.3, 129.2, 129.1, 128.3, 127.9, 127.8, 127.3, 126.1, 124.5, 123.6, 120.4, 115.5 (q, *J* = 287.4 Hz), 109.9, 95.9, 88.4, 46.3, 44.7, 42.1, 20.8, 12.5.

^{19}F NMR (564 MHz, Chloroform-*d*) δ (ppm): −75.80.

HRMS (ESI-TOF) m/z: $[M+Na]^+$ Calcd for $C_{39}H_{33}F_3N_4NaO_3^+$ 685.2397, Found: 685.2402.

***N*-((3*R*,6'*R*)-1-benzyl-3'-methyl-6'-((*E*)-2-(naphthalen-2-yl)vinyl)-2-oxo-1'-phenyl-5',6'-dihydro-1'*H*-spiro[indoline-3,4'-pyrano[2,3-*c*]pyrazol]-6'-yl)-2,2,2-trifluoroacetamide (4ak)**

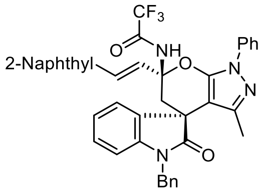

Prepared according to the general procedure, the crude product was purified by silica gel chromatography (petroleum ether/ethyl acetate 30:1) to afford **4ak** (44.6 mg, 65% yield, white solid, > 20:1 dr, m.p: 145.2 – 148.6 °C).

The enantiomeric excess was determined to be 96% by HPLC with a Daicel Chiralpak IG (*n*-hexane/2-propanol = 80:20, 1.0 mL/min, at 254 nm): t_R = 9.24 min (major), t_R = 12.29 min (minor); $[\alpha]_D^{20}$ = + 107.692 (*c* = 0.13, EA).

NMR and HRMS data for the product **4ak**:

^{1}H NMR (600 MHz, Chloroform-*d*) δ (ppm): 11.76 (s, 1H), 7.83 (d, *J* = 7.8 Hz, 2H), 7.81 – 7.80 (m, 4H), 7.63 (d, *J* = 9.6 Hz, 1H), 7.49 – 7.45 (m, 4H), 7.42 (d, *J* = 7.2 Hz, 2H), 7.39 (t, *J* = 7.8 Hz, 2H), 7.34 (t, *J* = 7.2 Hz, 1H), 7.28 – 7.26 (m, 2H), 7.14 – 7.10 (m, 2H), 7.03 (d, *J* = 16.2 Hz, 1H), 6.93 (d, *J* = 7.9 Hz, 1H), 6.82 (d, *J* = 16.2 Hz, 1H), 5.15 (d, *J* = 15.0 Hz, 1H), 4.93 (d, *J* = 15.0 Hz, 1H), 2.67 (d, *J* = 15.6 Hz, 1H), 2.62 (d, *J* = 15.6 Hz, 1H), 1.48 (s, 3H).

^{13}C NMR (150 MHz, Chloroform-*d*) δ (ppm): 179.8, 157.0 (q, *J* = 37.8 Hz), 148.9, 145.8, 142.4, 138.1, 135.0, 133.5, 133.4, 132.9, 132.0, 131.3, 129.3, 129.3, 129.1, 128.4, 128.3, 128.1, 127.9, 127.8, 127.7, 127.5, 126.4, 126.3, 126.2, 124.4, 123.6, 123.6, 120.6, 115.5 (q, *J* = 287.4 Hz), 109.8, 95.9, 88.7, 46.2, 44.7, 41.9, 12.5.

^{19}F NMR (564 MHz, Chloroform-*d*) δ (ppm): −75.65.

HRMS (ESI-TOF) m/z: $[M+Na]^+$ Calcd for $C_{41}H_{31}F_3N_4NaO_3^+$ 707.2240, Found: 707.2237.

<u>*N*-((3*R*,6'*R*)-1-benzyl-3'-methyl-6'-((*E*)-2-(naphthalen-1-yl)vinyl)-2-oxo-1'-phenyl-5',6'-dihydro-1'*H*-spiro[indoline-3,4'-pyrano[2,3-*c*]pyrazol]-6'-yl)-2,2,2-trifluoroacetamide (4al)</u>

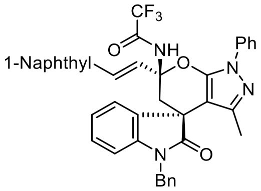

Prepared according to the general procedure, the crude product was purified by silica gel chromatography (petroleum ether/ethyl acetate 30:1) to afford **4al** (42.8 mg, 62% yield, white solid, > 20:1 dr, m.p: 158.9 – 160.3 °C).

The enantiomeric excess was determined to be 97% by HPLC with a Daicel Chiralpak IB (*n*-hexane/2-propanol = 80:20, 1.0 mL/min, at 254 nm): t_R = 8.45 min (minor), t_R = 9.71 min (major); $[\alpha]_D^{20}$ = + 220.462 (*c* = 0.13, EA).

NMR and HRMS data for the product **4al**:

^{1}H NMR (600 MHz, Chloroform-*d*) δ (ppm): 11.79 (s, 1H), 8.06 (d, *J* = 9.0 Hz, 1H), 7.85 (d, *J* = 7.8 Hz, 3H), 7.82 (d, *J* = 7.8 Hz, 1H), 7.68 – 7.65 (m, 2H), 7.54 – 7.48 (m, 2H), 7.46 (dd, *J* = 7.2, 2.4 Hz, 3H), 7.43 (d, *J* = 7.2 Hz, 2H), 7.39 (t, *J* = 7.2 Hz, 2H), 7.34 (t, *J* = 7.2 Hz, 1H), 7.31 – 7.25 (m, 2H), 7.17 (d, *J* = 7.8 Hz, 1H), 7.13 (t, *J* = 7.2 Hz, 1H), 6.95 (d, *J* = 8.4 Hz, 1H), 6.68 (d, *J* = 16.2 Hz, 1H), 5.18 (d, *J* = 15.6 Hz, 1H), 4.93 (d, *J* = 15.0 Hz, 1H), 2.72 (d, *J* = 15.0 Hz, 1H), 2.67 (d, *J* = 15.0 Hz, 1H), 1.48 (s, 3H).

^{13}C NMR (150 MHz, Chloroform-*d*) δ (ppm): 179.8, 157.1 (q, *J* = 37.8 Hz), 148.9, 145.8, 142.4, 138.1, 135.0, 133.6, 133.4, 131.3, 131.3, 130.6, 129.6, 129.3, 129.3, 129.1, 128.8, 128.6, 128.3, 127.9, 126.4, 126.2, 126.0, 125.6, 124.6, 124.4, 123.7, 123.7, 120.5, 115.6 (q, *J* = 287.3 Hz), 109.9, 95.9, 88.6, 46.3, 44.7, 41.9, 12.6.

^{19}F NMR (564 MHz, Chloroform-*d*) δ (ppm): −75.69.

HRMS (ESI-TOF) m/z: $[M+Na]^+$ Calcd for $C_{41}H_{31}F_3N_4NaO_3^+$ 707.2240, Found: 707.2250.

<u>*N*-((3*R*,6'*R*)-1-benzyl-3'-methyl-2-oxo-1'-phenyl-6'-((*E*)-2-(thiophen-2-yl)vinyl)-5',6'-dihydro-1'*H*-spiro[indoline-3,4'-pyrano[2,3-*c*]pyrazol]-6'-yl)-2,2,2-trifluoroacetamide (4am)</u>

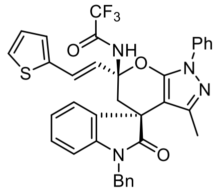

Prepared according to the general procedure, the crude product was purified by silica gel chromatography (petroleum ether/ethyl acetate 30:1) to afford **4am** (41.0 mg, 64% yield, white solid, > 20:1 dr, m.p: 150.6 – 152.6 °C).

The enantiomeric excess was determined to be 97% by HPLC with a Daicel Chiralpak IG (*n*-hexane/2-propanol = 90:10, 1.0 mL/min, at 254 nm): t_R = 12.20 min (major), t_R = 15.23 min (minor); $[\alpha]_D^{20}$ = + 530.666 (*c* = 0.15, EA).

NMR and HRMS data for the product **4am**:

^{1}H NMR (600 MHz, Chloroform-*d*) δ (ppm): 11.67 (s, 1H), 7.79 (d, *J* = 7.2 Hz, 2H), 7.47 (t, *J* = 7.2 Hz, 2H), 7.41 – 7.38 (m, 4H), 7.37 – 7.32 (m, 2H), 7.28 – 7.26 (m, 1H), 7.22 (d, *J* = 4.8 Hz, 1H), 7.10 – 7.05 (m, 3H), 7.02 – 6.97 (m, 2H), 6.92 (d, *J* = 7.8 Hz, 1H), 6.47 (d, *J* = 15.6 Hz, 1H), 5.13 (d, *J* = 15.6 Hz, 1H), 4.92 (d, *J* = 15.6 Hz, 1H), 2.60 (d, *J* = 15.6 Hz, 1H), 2.55 (d, *J* = 15.6 Hz, 1H), 1.47 (s, 3H).

^{13}C NMR (150 MHz, Chloroform-*d*) δ (ppm): 179.7, 157.0 (q, *J* = 38.0 Hz), 148.8, 145.7, 142.4, 140.3, 138.0, 135.0, 131.2, 129.3, 129.3, 129.1, 128.3, 127.9, 127.6, 127.5, 126.4, 126.3, 125.6, 125.2, 124.4, 123.6, 120.6, 115.5 (q, *J* = 287.7 Hz), 109.9, 95.9, 88.3, 46.2, 44.6, 41.9, 12.5.

^{19}F NMR (564 MHz, Chloroform-*d*) δ (ppm): −75.68.

HRMS (ESI-TOF) m/z: $[M+Na]^+$ Calcd for $C_{35}H_{27}F_3N_4NaO_3S^+$ 663.1648, Found: 663.1656.

***N*-((3*R*,6'*R*)-1-benzyl-6'-((*E*)-2-cyclohexylvinyl)-3'-methyl-2-oxo-1'-phenyl-5',6'-dihydro-1'*H*-spiro[indoline-3,4'-pyrano[2,3-*c*]pyrazol]-6'-yl)-2,2,2-trifluoroacetamide (4an)**

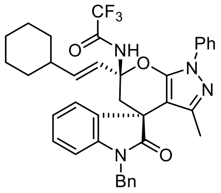

Prepared according to the general procedure, the crude product was purified by silica gel chromatography (petroleum ether/ethyl acetate 30:1) to afford **4an** (54.6 mg, 85% yield, white solid, > 20:1 dr, m.p: 168.2 – 175.4 °C).

The enantiomeric excess was determined to be 81% by HPLC with a Daicel Chiralpak IB (*n*-hexane/2-propanol = 70:30, 1.0 mL/min, at 254 nm): t_R = 5.05 min (minor), t_R = 7.26 min (major); $[\alpha]_D^{20}$ = + 205.800 (*c* = 0.10, EA).

NMR and HRMS data for the product **4an**:

^{1}H NMR (600 MHz, Chloroform-*d*) δ (ppm): 11.51 (s, 1H), 7.78 (d, *J* = 7.8 Hz, 2H), 7.44 (t, *J* = 8.4 Hz, 2H), 7.40 (d, *J* = 6.6 Hz, 2H), 7.37 (t, *J* = 7.8 Hz, 2H), 7.32 (t, *J* = 7.2 Hz, 1H), 7.28 – 7.23 (m, 2H), 7.10 (d, *J* = 4.2 Hz, 2H), 6.91 (d, *J* = 7.8 Hz, 1H), 5.99 (d, *J* = 16.2 Hz, 1H), 5.90 (dd, *J* = 15.6, 6.6 Hz, 1H), 5.13 (d, *J* = 15.6 Hz, 1H), 4.90 (d, *J* = 15.6 Hz, 1H), 2.49 (d, *J* = 15.0 Hz, 1H), 2.45 (d, *J* = 15.6 Hz, 1H), 2.10 – 2.04 (m, 1H), 1.77 – 1.71 (m, 4H), 1.66 – 1.64 (m, 2H), 1.43 (s, 3H), 1.27 – 1.23 (m, 1H), 1.19 – 1.14 (m, 3.2 Hz, 1H), 1.30 – 1.11 (m, 1H), 1.10 – 1.06(m, 1H).

^{13}C NMR (150 MHz, Chloroform-*d*) δ (ppm): 179.8, 156.7 (q, *J* = 37.4 Hz), 149.1, 145.7, 142.4, 139.0, 138.1, 135.1, 131.5, 129.2, 129.2, 129.0, 128.3, 127.9, 126.1, 126.0, 124.3, 123.6, 120.5, 115.5 (q, *J* = 287.6 Hz), 109.8, 95.8, 88.6, 46.1, 44.6, 42.0, 40.0, 32.4, 32.3, 26.1, 25.9, 25.8, 1+2.5.

^{19}F NMR (564 MHz, Chloroform-*d*) δ (ppm): −75.89.

HRMS (ESI-TOF) m/z: $[M+Na]^+$ Calcd for $C_{37}H_{35}F_3N_4NaO_3^+$ 663.2553, Found: 663.2545.

<u>*N*-((3*R*,6'*R*)-1-benzyl-2-oxo-1'-phenyl-3'-propyl-6'-((*E*)-styryl)-5',6'-dihydro-1'*H*-spiro[indoline-3,4'-pyrano[2,3-*c*]pyrazol]-6'-yl)-2,2,2-trifluoroacetamide (4ao)</u>

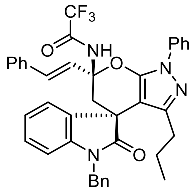

Prepared according to the general procedure, the crude product was purified by silica gel chromatography (petroleum ether/ethyl acetate 30:1) to afford **4ao** (49.8 mg, 75% yield, white solid, > 20:1 dr, m.p: 150.3 – 153.8 °C).

The enantiomeric excess was determined to be 70% by HPLC with a Daicel Chiralpak IG (*n*-hexane/2-propanol = 90:10, 1.0 mL/min, at 254 nm): t_R = 7.07 min (major), t_R = 7.78 min (minor); $[\alpha]_D^{20}$ = – 520.200 (*c* = 0.10, EA).

NMR and HRMS data for the product **4ao**:

^{1}H NMR (600 MHz, Chloroform-*d*) δ (ppm): 11.65 (s, 1H), 7.82 (d, *J* = 7.8 Hz, 2H), 7.47 – 7.44 (m, 2H), 7.44 – 7.41 (m, 4H), 7.40 – 7.37 (m, 2H), 7.33 (t, *J* = 7.8 Hz, 3H), 7.29 – 7.23 (m, 3H), 7.12 – 7.09 (m, 2H), 6.92 (d, *J* = 7.8 Hz, 1H), 6.87 (d, *J* = 16.2 Hz, 1H), 6.69 (d, *J* = 16.2 Hz, 1H), 5.07 (d, *J* = 15.0 Hz,

1H), 4.98 (d, J = 15.6 Hz, 1H), 2.61 (d, J = 15.6 Hz, 1H), 2.57 (d, J = 15.6 Hz, 1H), 1.81 – 1.76 (m, 1H), 1.70 – 1.65 (m, 1H), 1.25 – 1.21 (m, 1H), 1.12 – 1.05 (m, 1H), 0.55 (t, J = 7.2 Hz, 3H).

^{13}C NMR (150 MHz, Chloroform-d) δ (ppm): 180.0, 157.0 (q, J = 37.7 Hz), 149.7, 148.7, 142.3, 138.2, 135.5, 134.9, 131.8, 131.8, 129.5, 129.3, 129.0, 128.7, 128.5, 128.3, 127.9, 127.6, 127.0, 126.1, 124.4, 123.7, 120.7, 115.5 (q, J = 287.4 Hz), 109.8, 95.5, 88.5, 46.3, 44.7, 42.2, 29.5, 21.2, 14.0.

^{19}F NMR (564 MHz, Chloroform-*d*) δ (ppm): −75.71.

HRMS (ESI-TOF) m/z: $[M+Na]^+$ Calcd for $C_{39}H_{33}F_3N_4NaO_3^+$ 685.2397, Found: 685.2382.

<u>*N*-((3*R*,6'*R*)-1-benzyl-3'-cyclopropyl-6'-((*E*)-2-methylstyryl)-2-oxo-1'-phenyl-5',6'-dihydro-1'*H*-spiro[indoline-3,4'-pyrano[2,3-*c*]pyrazol]-6'-yl)-2,2,2-trifluoroacetamide (4ap)</u>

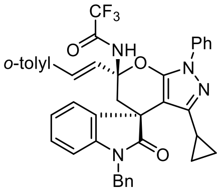

Prepared according to the general procedure, the crude product was purified by silica gel chromatography (petroleum ether/ethyl acetate 30:1) to afford **4ap** (41.2 mg, 61% yield, white solid, > 20:1 dr, m.p: 140.2 – 141.5 °C).

The enantiomeric excess was determined to be 78% by HPLC with a Daicel Chiralpak IB (*n*-hexane/2-propanol = 80:20, 1.0 mL/min, at 254 nm): t_R = 6.65 min (minor), t_R = 8.67 min (major); $[\alpha]_D^{20}$ = + 207.333 (*c* = 0.12, EA).

NMR and HRMS data for the product **4ap**:

^{1}H NMR (600 MHz, Chloroform-*d*) δ (ppm): 11.69 (s, 1H), 7.82 (d, J = 7.8 Hz, 2H), 7.48 – 7.46 (m, 1H), 7.42 (d, J = 7.2 Hz, 3H), 7.37 (t, J = 7.2 Hz, 2H), 7.33 – 7.31 (m, 1H), 7.28 – 7.25 (m, 1H), 7.23 (d, J = 7.8 Hz, 1H), 7.19 – 7.17 (m, 2H), 7.16 – 7.15 (m, 3H), 7.13 – 7.11 (m, 2H), 6.91 (d, J = 7.8 Hz, 1H), 6.50 (d, J = 15.6 Hz, 1H), 5.10 (d, J = 15.6 Hz, 1H), 4.97 (d, J = 15.0 Hz, 1H), 2.62 (d, J = 15.0 Hz, 1H), 2.59 (d, J = 15.6 Hz, 1H), 2.35 (s, 3H), 0.77 – 0.73 (m, 1H), 0.65 – 0.61 (m, 1H), 0.51 – 0.47 (m, 1H), 0.35 – 0.30 (m, 1H), 0.14 – 0.10 (m, 1H).

^{13}C NMR (150 MHz, Chloroform-*d*) δ (ppm): 180.0, 156.9 (q, J = 37.5 Hz), 150.4, 148,7, 142.5, 138.3, 136.1, 135.0, 134.8, 131.9, 130.3, 130.1, 129.2, 129.1, 129.0, 128.7, 128.4, 128.2, 127.9, 126.2, 126.1, 126.0, 124.3, 123.8, 120.4, 115.5 (q, J = 287.6 Hz), 109.7, 96.5, 88.6, 46.3, 44.7, 42.1, 19.7, 7.5, 5.9, 5.9.

^{19}F NMR (564 MHz, Chloroform-*d*) δ (ppm): −75.78.

HRMS (ESI-TOF) m/z: $[M+Na]^+$ Calcd for $C_{40}H_{33}F_3N_4NaO_3^+$ 697.2397, Found: 697.2390.

<u>*N*-((3*R*,6'*R*)-1-benzyl-7-fluoro-3'-methyl-6'-((*E*)-2-methylstyryl)-2-oxo-1'-phenyl-5',6'-dihydro-1'*H*-spiro[indoline-3,4'-pyrano[2,3-*c*]pyrazol]-6'-yl)-2,2,2-trifluoroacetamide (4aq)</u>

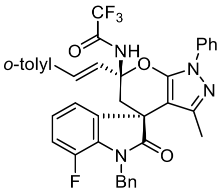

Prepared according to the general procedure, the crude product was purified by silica gel chromatography (petroleum ether/ethyl acetate 30:1) to afford **4aq** (46.4 mg, 70% yield, white solid, > 20:1 dr, m.p: 140.9 – 146.5 °C).

The enantiomeric excess was determined to be 99% by HPLC with a Daicel Chiralpak IG (*n*-hexane/2-propanol = 80:20, 1.0 mL/min, at 254 nm): t_R = 5.71 min (major), t_R = 6.46 min (minor); $[\alpha]_D^{20}$ = + 287.420 (*c* = 0.10, EA).

NMR and HRMS data for the product **4aq**:

^{1}H NMR (600 MHz, Chloroform-*d*) δ (ppm): 11.56 (s, 1H), 7.81 (d, *J* = 7.8 Hz, 2H), 7.47 – 7.43 (m, 5H), 7.37 (t, *J* = 7.8 Hz, 2H), 7.33 (d, *J* = 7.2 Hz, 1H), 7.26 – 7.24 (m, 1H), 7.21 – 7.18 (m, 2H), 7.17 – 7.15 (m, 1H), 7.12 (d, *J* = 15.6 Hz, 1H), 7.10 – 7.06 (m, 2H), 6.93 – 6.91 (m, 1H), 6.48 (d, *J* = 15.6 Hz, 1H), 5.26 (d, *J* = 15.0 Hz, 1H), 5.08 (d, *J* = 15.0 Hz, 1H), 2.60 (d, *J* = 15.0 Hz, 1H), 2.53 (d, *J* = 15.0 Hz, 1H), 2.35 (s, 3H), 1.41 (s, 3H).

^{13}C NMR (150 MHz, Chloroform-*d*) δ (ppm): 179.6, 156.9 (q, *J* = 37.7Hz), 148.8, 147.4 (d, J_{CF}= 245.0 Hz), 145.6, 138.1, 136.1 (d, J_{CF} = 8.3 Hz), 134.7, 134.3 (d, J_{CF} = 2.1 Hz), 130.4, 130.3, 129.3, 129.0 (d, J_{CF} = 8.6 Hz), 128.9, 128.4, 128.4, 128.3, 128.2, 128.2, 126.3, 126.2, 126.1, 125.1 (d, J_{CF} = 6.5 Hz), 120.4, 119.5 (d, J_{CF} = 3.0 Hz), 117.5 (d, J_{CF} = 19.5 Hz), 115.5 (q, *J* = 287.4 Hz), 95.6, 88.6, 46.5 (d, *J* = 6.0 Hz), 46.2 (d, *J* = 18.6 Hz), 42.0, 19.7, 12.5.

^{19}F NMR (564 MHz, Chloroform-*d*) δ (ppm): −75.80, −132.19 – −132.21.

HRMS (ESI-TOF) m/z: $[M+Na]^+$ Calcd for $C_{38}H_{30}F_4N_4NaO_3^+$ 689.2146, Found: 689.2142.

<u>*N*-((3*R*,6'*R*)-1-benzyl-7-chloro-3'-methyl-6'-((*E*)-2-methylstyryl)-2-oxo-1'-phenyl-5',6'-dihydro-1'*H*-spiro[indoline-3,4'-pyrano[2,3-*c*]pyrazol]-6'-yl)-2,2,2-trifluoroacetamide (4ar)</u>

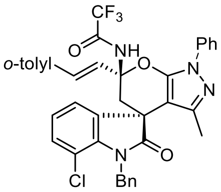

Prepared according to the general procedure, the crude product was purified by silica gel chromatography (petroleum ether/ethyl acetate 30:1) to afford **4ar** (46.5 mg, 68% yield, white solid, > 20:1 dr, m.p: 140.3 – 146.3 °C).

The enantiomeric excess was determined to be 99% by HPLC with a Daicel Chiralpak IG (*n*-hexane/2-propanol = 80:20, 1.0 mL/min, at 254 nm): t_R = 5.86 min (major), t_R = 7.22 min (minor); $[\alpha]_D^{20}$ = + 257.400 (*c* = 0.10, EA).

NMR and HRMS data for the product **4ar**:

^{1}H NMR (600 MHz, Chloroform-*d*) δ (ppm): 11.50 (s, 1H), 7.82 (d, *J* = 7.8 Hz, 2H), 7.46 – 7.44 (m, 3H), 7.39 – 7.35 (m, 4H), 7.33 – 7.30 (m, 1H), 7.29 – 7.24 (m, 2H), 7.22 – 7.8 (m, 2H), 7.17 – 7.15 (m, 1H), 7.13 (d, *J* = 16.2 Hz, 1H), 7.07 (q, *J* = 8.4Hz, 2H), 6.48 (d, *J* = 16.2 Hz, 1H), 5.52 (d, *J* = 15.6 Hz, 1H), 5.46 (d, *J* = 15.6 Hz, 1H), 2.60 (d, *J* = 15.0 Hz, 1H), 2.54 (d, *J* = 15.0 Hz, 1H), 2.36 (s, 3H), 1.54 (s, 3H).

^{13}C NMR (150 MHz, Chloroform-*d*) δ (ppm): 180.6, 156.9 (q, *J* = 37.7 Hz), 148.9, 145.5, 138.5, 138.0, 136.6, 136.1, 134.6, 134.4, 131.9, 130.4, 130.3, 129.3, 128.8, 128.4, 128.3, 127.9, 127.2, 126.3, 126.2, 126.1, 125.2, 122.4, 120.4, 116.1, 115.4 (q, *J* = 287.4 Hz), 95.5, 88.6, 46.0, 45.6, 42.3, 19.7, 12.7.

^{19}F NMR (564 MHz, Chloroform-*d*) δ (ppm): –75.82.

HRMS (ESI-TOF) m/z: $[M+Na]^+$ Calcd for $C_{38}H_{30}ClF_3N_4NaO_3^+$ 705.1851, Found: 705.1844.

***N*-((3*R*,6'*R*)-1-benzyl-7-bromo-3'-methyl-6'-((*E*)-2-methylstyryl)-2-oxo-1'-phenyl-5',6'-dihydro-1'*H*-spiro[indoline-3,4'-pyrano[2,3-*c*]pyrazol]-6'-yl)-2,2,2-trifluoroacetamide (4as)**

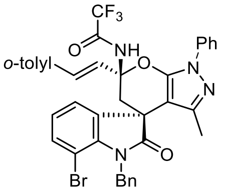

Prepared according to the general procedure, the crude product was purified by silica gel chromatography (petroleum ether/ethyl acetate 30:1) to afford **4as** (51.2 mg, 70% yield, white solid, > 20:1 dr, m.p: 140.2 – 147.5 °C).

The enantiomeric excess was determined to be 99% by HPLC with a Daicel Chiralpak IG (*n*-hexane/2-propanol = 80:20, 1.0 mL/min, at 254 nm): t_R = 5.99 min (major), t_R = 7.60 min (minor); $[\alpha]_D^{20}$ = + 323.625 (*c* = 0.16, EA).

NMR and HRMS data for the product **4as**:

^{1}H NMR (600 MHz, Chloroform-*d*) δ (ppm): 11.37 (s, 1H), 7.71 (d, *J* = 7.8 Hz, 2H), 7.39 – 7.36 (m, 2H), 7.34 (d, *J* = 8.4 Hz, 2H), 7.27 – 7.25 (m, 2H), 7.23 – 7.20 (m, 1H), 7.19 – 7.13 (m, 3H), 7.10 – 7.08 (m,

2H), 7.06 (d, J = 8.4 Hz, 1H), 7.02 (d, J = 16.2 Hz, 2H), 6.91 (t, J = 7.8 Hz, 1H), 6.37 (d, J = 16.2 Hz, 1H), 5.49 (d, J = 16.0 Hz, 1H), 5.39 (d, J = 15.6 Hz, 1H), 2.49 (d, J = 15.6 Hz, 1H), 2.44 (d, J = 15.0 Hz, 1H), 2.25 (s, 3H), 1.46 (s, 3H).

^{13}C NMR (150 MHz, Chloroform-*d*) δ (ppm): 180.8, 156.9 (q, J = 37.8 Hz), 148.9, 145.5, 139.9, 138.0, 136.5, 136.1, 135.3, 134.8, 134.6, 130.4, 130.3, 129.3, 128.8, 128.5, 128.3, 127.8, 127.0, 126.3, 126.2, 126.1, 125.6, 123.0, 120.4, 115.4 (q, J = 287.3 Hz), 103.1, 95.5, 88.6, 45.9, 45.2, 42.4, 19.7, 12.8.

^{19}F NMR (564 MHz, Chloroform-*d*) δ (ppm): −75.84.

HRMS (ESI-TOF) m/z: $[M+Na]^+$ Calcd for $C_{38}H_{30}BrF_3N_4NaO_3^+$ 749.1346, Found: 749.1344.

<u>*N*-((3R,6'*R*)-1-benzyl-3',7-dimethyl-6'-((*E*)-2-methylstyryl)-2-oxo-1'-phenyl-5',6'-dihydro-1'*H*-spiro[indoline-3,4'-pyrano[2,3-*c*]pyrazol]-6'-yl)-2,2,2-trifluoroacetamide (4at)</u>

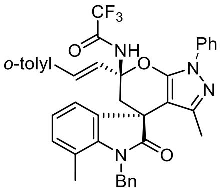

Prepared according to the general procedure, the crude product was purified by silica gel chromatography (petroleum ether/ethyl acetate 30:1) to afford **4at** (49.8 mg, 75% yield, white solid, > 20:1 dr, m.p: 131.5 – 135.7 °C).

The enantiomeric excess was determined to be 99% by HPLC with a Daicel Chiralpak IG (*n*-hexane/2-propanol = 80:20, 1.0 mL/min, at 254 nm): t_R = 5.89 min (major), t_R = 7.66 min (minor); $[\alpha]_D^{20}$ = + 319.636 (c = 0.11, EA).

NMR and HRMS data for the product **4at**:

^{1}H NMR (600 MHz, Chloroform-*d*) δ (ppm): 11.71 (s, 1H), 7.83 (d, J = 7.8 Hz, 2H), 7.47 – 7.44 (m, 3H), 7.38 (t, J = 7.8 Hz, 2H), 7.32 (t, J = 7.2 Hz, 1H), 7.26 – 7.24 (m, 3H), 7.21 – 7.18 (m, 2H), 7.16 – 7.15 (m, 1H), 7.13 (d, J = 16.2 Hz, 1H), 7.05 – 7.02 (m, 2H), 7.00 – 6.99 (m, 1H), 6.49 (d, J = 16.2 Hz, 1H), 5.33 (d, J = 16.8 Hz, 1H), 5.29 (d, J = 16.2 Hz, 1H), 2.61 (d, J = 15.6 Hz, 1H), 2.58 (d, J = 15.6 Hz, 1H), 2.39 (s, 3H), 2.36 (s, 3H), 1.64 (s, 3H).

^{13}C NMR (150 MHz, Chloroform-*d*) δ (ppm): 180.9, 156.9 (q, J = 37.7 Hz), 148.9, 145.7, 140.6, 138.1, 136.8, 136.1, 134.8, 133.2, 132.1, 130.4, 130.1, 129.3, 129.1, 128.6, 128.4, 127.8, 126.2, 126.1, 126.1, 126.0, 124.5, 121.7, 120.7, 120.5, 115.5 (q, J = 287.4 Hz), 96.2, 88.7, 45.8, 45.7, 42.7, 19.7, 18.8, 12.8.

^{19}F NMR (564 MHz, Chloroform-*d*) δ (ppm): −75.81.

HRMS (ESI-TOF) m/z: $[M+Na]^+$ Calcd for $C_{39}H_{33}F_3N_4NaO_3^+$ 685.2397, Found: 685.2387.

N-((3R,6'R)-1-benzyl-6-fluoro-3'-methyl-6'-((E)-2-methylstyryl)-2-oxo-1'-phenyl-5',6'-dihydro-1'H-spiro[indoline-3,4'-pyrano[2,3-c]pyrazol]-6'-yl)-2,2,2-trifluoroacetamide (4au)

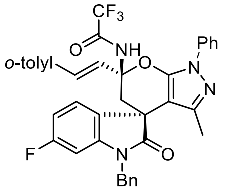

Prepared according to the general procedure, the crude product was purified by silica gel chromatography (petroleum ether/ethyl acetate 30:1) to afford **4au** (45.9 mg, 69% yield, white solid, > 20:1 dr, m.p: 178.9 – 190.4 °C).

The enantiomeric excess was determined to be 99% by HPLC with a Daicel Chiralpak IG (*n*-hexane/2-propanol = 90:00, 1.0 mL/min, at 254 nm): t_R = 8.78 min (major), t_R = 10.20 min (minor); $[\alpha]_D^{20}$ = + 558.166 (*c* = 0.10, EA).

NMR and HRMS data for the product **4au**:

^{1}H NMR (600 MHz, Chloroform-*d*) δ (ppm): 11.53 (s, 1H), 7.82 (d, *J* = 8.4 Hz, 2H), 7.46 – 7.44 (m, 3H), 7.41 – 7.39 (m, 4H), 7.37 – 7.33 (m, 1H), 7.27 – 7.24 (m, 1H), 7.21 – 7.18 (m, 2H), 7.17 – 7.15 (m, 1H), 7.13 (d, *J* = 16.2 Hz, 1H), 7.08 (dd, *J* = 7.8, 4.8 Hz, 1H), 6.81 – 6.78 (m, 1H), 6.67 (dd, *J* = 8.4, 1.8 Hz, 1H), 6.48 (d, *J* = 16.2 Hz, 1H), 5.16 (d, *J* = 15.0 Hz, 1H), 4.86 (d, *J* = 15.0 Hz, 1H), 2.60 (d, *J* = 15.0 Hz, 1H), 2.54 (d, *J* = 15.6 Hz, 1H), 2.36 (s, 3H), 1.49 (s, 3H).

^{13}C NMR (150 MHz, Chloroform-*d*) δ (ppm): 180.2, 163.2 (d, J_{CF} = 246.3 Hz), 156.9 (q, *J* = 37.8 Hz), 148.9, 145.6, 143.8 (d, J_{CF} = 11.4 Hz), 138.1, 136.1, 134.7, 134.5, 130.4, 130.2, 129.3, 129.2, 128.5, 128.4, 128.4, 127.9, 126.6 (d, J_{CF} = 3.0 Hz), 126.3, 126.2, 126.1, 124.8 (d, J_{CF}= 9.8 Hz), 120.4, 115.5 (q, *J* = 287.6 Hz), 110.7 (d, J_{CF}= 22.5 Hz), 98.8 (d, J_{CF} = 27.8 Hz), 95.6, 88.6, 45.9, 44.9, 42.0, 19.7, 12.6.

^{19}F NMR (564 MHz, Chloroform-*d*) δ (ppm): −75.80, −109.55 – −109.60.

HRMS (ESI-TOF) m/z: $[M+Na]^+$ Calcd for $C_{38}H_{30}F_4N_4NaO_3^+$ 689.2146, Found: 689.2150.

N-((3R,6'R)-1-benzyl-6-chloro-3'-methyl-6'-((E)-2-methylstyryl)-2-oxo-1'-phenyl-5',6'-dihydro-1'H-spiro[indoline-3,4'-pyrano[2,3-c]pyrazol]-6'-yl)-2,2,2-trifluoroacetamide (4av)

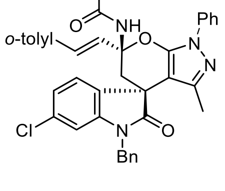

Prepared according to the general procedure, the crude product was purified by silica gel chromatography (petroleum ether/ethyl acetate 30:1) to afford **4av** (44.4 mg, 65% yield, white solid, > 20:1 dr, m.p: 140.9 – 143.5 °C).

The enantiomeric excess was determined to be 99% by HPLC with a Daicel Chiralpak IB (*n*-hexane/2-propanol = 80:20, 1.0 mL/min, at 254 nm): t_R = 7.30 min (minor), t_R = 9.07 min (major); $[\alpha]_D^{20}$ = + 504.000 (*c* = 0.11, EA).

NMR and HRMS data for the product **4av**:

^{1}H NMR (600 MHz, Chloroform-*d*) δ (ppm): 11.51 (s, 1H), 7.82 (d, *J* = 7.8 Hz, 2H), 7.47 – 7.45 (m, 3H), 7.41 (d, *J* = 4.2 Hz, 4H), 7.38 – 7.34 (m, 1H), 7.27 – 7.25 (m, 1H), 7.21 – 7.18 (m, 2H), 7.17 – 7.15 (m, 1H), 7.13 (d, *J* = 16.2 Hz, 1H), 7.11 – 7.09 (m, 1H), 7.06 (d, *J* = 8.4 Hz, 1H), 6.93 (d, *J* = 1.2 Hz, 1H), 6.48 (d, *J* = 15.6 Hz, 1H), 5.16 (d, *J* = 15.0 Hz, 1H), 4.85 (d, *J* = 15.0 Hz, 1H), 2.59 (d, *J* = 15.0 Hz, 1H), 2.53 (d, *J* = 15.6 Hz, 1H), 2.36 (s, 3H), 1.49 (s, 3H).

^{13}C NMR (150 MHz, Chloroform-*d*) δ (ppm): 179.8, 156.8 (q, *J* = 37.7 Hz), 148.9, 145.5, 143.5, 138.0, 136.1, 135.1, 134.6, 134.5, 130.4, 130.3, 129.6, 129.3, 129.2, 128.6, 128.4, 128.3, 127.9, 126.3, 126.2, 126.1, 124.6, 124.4, 120.4, 115.5 (q, *J* = 287.4 Hz), 110.5, 95.4, 88.6, 46.0, 44.8, 41.8, 19.7, 12.7.

^{19}F NMR (564 MHz, Chloroform-*d*) δ (ppm): −75.80.

HRMS (ESI-TOF) m/z: $[M+Na]^+$ Calcd for $C_{38}H_{30}ClF_3N_4NaO_3^+$ 705.1851, Found: 705.1848.

<u>*N*-((3*R*,6'*R*)-1-benzyl-5-fluoro-3'-methyl-6'-((*E*)-2-methylstyryl)-2-oxo-1'-phenyl-5',6'-dihydro-1'*H*-spiro[indoline-3,4'-pyrano[2,3-*c*]pyrazol]-6'-yl)-2,2,2-trifluoroacetamide (4aw)</u>

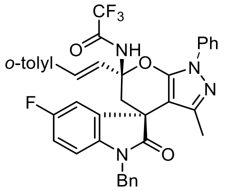

Prepared according to the general procedure, the crude product was purified by silica gel chromatography (petroleum ether/ethyl acetate 30:1) to afford **4aw** (47.9 mg, 72% yield, white solid, > 20:1 dr, m.p: 145.5 – 147.8 °C).

The enantiomeric excess was determined to be 99% by HPLC with a Daicel Chiralpak IC (*n*-hexane/2-propanol = 95:05, 1.0 mL/min, at 254 nm): t_R = 9.76 min (major), t_R = 11.43 min (minor); $[\alpha]_D^{20}$ = + 256.777 (*c* = 0.14, EA).

NMR and HRMS data for the product **4aw**:

^{1}**H NMR** (600 MHz, Chloroform-*d*) δ (ppm): 11.54 (s, 1H), 7.73 (d, *J* = 7.8 Hz, 2H), 7.36 (dd, *J* = 7.8, 4.2 Hz, 3H), 7.30 – 7.28 (m, 4H), 7.26 – 7.23 (m, 1H), 7.17 (t, *J* = 7.8 Hz, 1H), 7.12 – 7.06 (m, 3H), 7.04 (d, *J* = 16.2 Hz, 1H), 6.89 (td, *J* = 8.4, 1.8 Hz, 1H), 6.80 (dd, *J* = 7.2, 2.4 Hz, 1H), 6.76 (dd, *J* = 9.0, 4.2 Hz, 1H), 6.39 (d, *J* = 16.2 Hz, 1H), 5.07 (d, *J* = 15.0 Hz, 1H), 4.80 (d, *J* = 15.0 Hz, 1H), 2.53 (d, *J* = 15.0 Hz, 1H), 2.45 (d, *J* = 15.6 Hz, 1H), 2.27 (s, 3H), 1.41 (s, 3H).

^{13}**C NMR** (150 MHz, Chloroform-*d*) δ (ppm): 179.5, 160.2 (d, J_{CF} = 242.9 Hz), 156.9 (q, *J* = 37.8 Hz), 148.9, 145.5, 138.2 (d, J_{CF}= 1.8 Hz), 138.0, 136.1, 134.7, 134.7, 133.1 (d, J_{CF} = 7.7 Hz), 130.4, 130.3, 129.4, 129.3, 129.1, 128.4, 128.3, 127.8, 126.3, 126.2, 126.1, 120.5, 115.8 (d, J_{CF}= 23.4 Hz), 115.5 (q, *J* = 287.4 Hz), 111.8 (d, J_{CF} = 24.9 Hz), 110.7 (d, J_{CF} = 8.0 Hz), 95.4, 88.6, 46.6, 44.8, 41.9, 19.7, 12.6.

^{19}**F NMR** (564 MHz, Chloroform-*d*) δ (ppm): −75.79, −117.14 – −117.18.

HRMS (ESI-TOF) m/z: $[M+Na]^+$ Calcd for $C_{38}H_{30}F_4N_4NaO_3^+$ 689.2146, Found: 689.2152.

<u>*N*-((3*R*,6'*R*)-1-benzyl-5-chloro-3'-methyl-6'-((*E*)-2-methylstyryl)-2-oxo-1'-phenyl-5',6'-dihydro-1'*H*-spiro[indoline-3,4'-pyrano[2,3-*c*]pyrazol]-6'-yl)-2,2,2-trifluoroacetamide (4ax)</u>

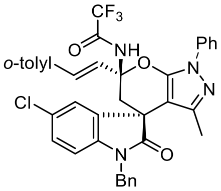

Prepared according to the general procedure, the crude product was purified by silica gel chromatography (petroleum ether/ethyl acetate 30:1) to afford **4ax** (40.8 mg, 60% yield, white solid, > 20:1 dr, m.p: 167.5 – 168.9 °C).

The enantiomeric excess was determined to be 99% by HPLC with a Daicel Chiralpak IG (*n*-hexane/2-propanol = 95:05, 1.0 mL/min, at 254 nm): t_R = 11.43 min (minor), t_R = 12.49 min (major); $[\alpha]_D^{20}$ = + 256.444 (*c* = 0.14, EA).

NMR and HRMS data for the product **4ax**:

^{1}**H NMR** (600 MHz, Chloroform-*d*) δ (ppm): 11.54 (s, 1H), 7.82 (d, *J* = 7.8 Hz, 2H), 7.45 (t, *J* = 7.8 Hz, 3H), 7.39 (d, *J* = 4.2 Hz, 4H), 7.34 – 7.33 (m, 1H), 7.27 – 7.24 (m, 2H), 7.21 – 7.18 (m, 2H), 7.17 – 7.12 (m, 3H), 6.85 (d, *J* = 8.4 Hz, 1H), 6.48 (d, *J* = 16.2 Hz, 1H), 5.16 (d, *J* = 15.6 Hz, 1H), 4.89 (d, *J* = 15.6 Hz, 1H), 2.62 (d, *J* = 15.0 Hz, 1H), 2.55 (d, *J* = 15.0 Hz, 1H), 2.36 (s, 3H), 1.51 (s, 3H).

^{13}**C NMR** (150 MHz, Chloroform-*d*) δ (ppm): 179.4, 156.8 (q, *J* = 37.8 Hz), 148.9, 145.5, 140.9, 138.0, 136.1, 134.7, 134.6, 133.1, 130.4, 130.0, 129.3, 129.3, 129.2, 128.5, 128.4, 128.3, 127.8, 126.3, 126.1, 124.1, 120.5, 115.5 (q, *J* = 287.6 Hz), 110.9, 95.3, 88.6, 46.4, 44.8, 41.8, 19.7, 12.7.

^{19}**F NMR** (564 MHz, Chloroform-*d*) δ (ppm): −75.80.

HRMS (ESI-TOF) m/z: [M+Na]$^+$ Calcd for $C_{38}H_{30}ClF_3N_4NaO_3^+$ 705.1851, Found: 705.1842.

<u>*N*-((3*R*,6'*R*)-1-benzyl-5-bromo-3'-methyl-6'-((*E*)-2-methylstyryl)-2-oxo-1'-phenyl-5',6'-dihydro-1'*H*-spiro[indoline-3,4'-pyrano[2,3-*c*]pyrazol]-6'-yl)-2,2,2-trifluoroacetamide (4ay)</u>

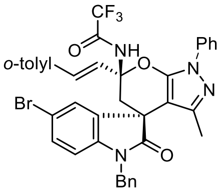

Prepared according to the general procedure, the crude product was purified by silica gel chromatography (petroleum ether/ethyl acetate 30:1) to afford **4ay** (37.9 mg, 52% yield, white solid, > 20:1 dr, m.p: 165.5 – 167.8 °C).

The enantiomeric excess was determined to be 99% by HPLC with a Daicel Chiralpak IG (*n*-hexane/2-propanol = 95:05, 1.0 mL/min, at 254 nm): t_R = 11.72 min (minor), t_R = 13.08 min (major); $[\alpha]_D^{20}$ = + 455.662 (*c* = 0.10, EA).

NMR and HRMS data for the product **4ay**:

^{1}**H NMR** (600 MHz, Chloroform-*d*) δ (ppm): 11.53 (s, 1H), 7.82 (d, *J* = 8.4 Hz, 2H), 7.45 (t, *J* = 7.8 Hz, 3H), 7.41 – 7.38 (m, 5H), 7.36 – 7.34 (m, 1H), 7.27 – 2.26 (m, 2H), 7.21 – 7.18 (m, 2H), 7.17 – 7.13 (m, 2H), 6.81 (d, *J* = 8.4 Hz, 1H), 6.48 (d, *J* = 16.2 Hz, 1H), 5.15 (d, *J* = 15.0 Hz, 1H), 4.88 (d, *J* = 15.0 Hz, 1H), 2.61 (d, *J* = 15.0 Hz, 1H), 2.56 (d, *J* = 15.6 Hz, 1H), 2.36 (s, 3H), 1.51 (s, 3H).

^{13}**C NMR** (150 MHz, Chloroform-*d*) δ (ppm): 179.3, 156.8 (q, *J* = 37.7 Hz), 148.9, 145.5, 141.4, 138.0, 136.1, 134.6, 134.5, 133.4, 132.2, 130.4, 129.3, 129.2, 128.5, 128.4, 128.2, 127.8, 126.9, 126.3, 126.1, 120.5, 117.2, 115.5 (d, *J* = 287.6 Hz), 111.3, 95.3, 88.6, 46.3, 44.8, 41.8, 19.7, 12.7.

^{19}**F NMR** (564 MHz, Chloroform-*d*) δ (ppm): −75.79.

HRMS (ESI-TOF) m/z: [M+Na]$^+$ Calcd for $C_{38}H_{30}BrF_3N_4NaO_3^+$ 749.1346, Found: 749.1349.

<u>*N*-((3*R*,6'*R*)-1-benzyl-3',5-dimethyl-6'-((*E*)-2-methylstyryl)-2-oxo-1'-phenyl-5',6'-dihydro-1'*H*-spiro[indoline-3,4'-pyrano[2,3-*c*]pyrazol]-6'-yl)-2,2,2-trifluoroacetamide (4az)</u>

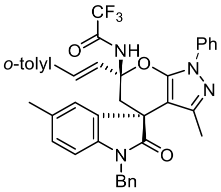

Prepared according to the general procedure, the crude product was purified by silica gel chromatography (petroleum ether/ethyl acetate 30:1) to afford **4az** (53.1 mg, 80% yield, white solid, > 20:1 dr, m.p: 156.4 −157.8 °C).

The enantiomeric excess was determined to be 97% by HPLC with a Daicel Chiralpak IB (*n*-hexane/2-propanol = 80:20, 1.0 mL/min, at 254 nm): t_R = 5.72 min (minor), t_R = 6.58 min (major); $[\alpha]_D^{20}$ = + 356.654 (*c* = 0.11, EA).

NMR and HRMS data for the product **4az**:

^{1}H NMR (600 MHz, Chloroform-*d*) δ (ppm): 11.75 (s, 1H), 7.84 (d, *J* = 7.8 Hz, 2H), 7.46 (q, *J* = 7.8 Hz, 3H), 7.41 (d, *J* = 7.2 Hz, 2H), 7.38 (t, *J* = 7.2 Hz, 2H), 7.33 (t, *J* = 7.2 Hz, 1H), 7.26 – 7.24 (m, 1H), 7.21 – 7.18 (m, 2H), 7.17 – 7.12 (m, 2H), 7.07 (d, *J* = 8.4 Hz, 1H), 6.95 (s, 1H), 6.82 (d, *J* = 8.4 Hz, 1H), 6.51 (d, *J* = 16.2 Hz, 1H), 5.15 (d, *J* = 15.6 Hz, 1H), 4.89 (d, *J* = 15.0 Hz, 1H), 2.61 (d, *J* = 15.0 Hz, 1H), 2.58 (d, *J* = 15.0 Hz, 1H), 2.37 (s, 3H), 2.28 (s, 3H), 1.48 (s, 3H).

^{13}C NMR (150 MHz, Chloroform-*d*) δ (ppm): 179.7, 156.9 (q, *J* = 37.8 Hz), 148.9, 145.8, 140.0, 138.2, 136.0, 135.1, 134.8, 134.2, 131.3, 130.3, 130.1, 129.5, 129.2, 129.0, 128.7, 128.3, 128.2, 127.9, 126.2, 126.1, 126.0, 124.2, 120.4, 115.5 (q, *J* = 287.6 Hz), 109.6, 96.1, 88.6, 46.3, 44.7, 42.0, 21.1, 19.7, 12.6.

^{19}F NMR (564 MHz, Chloroform-*d*) δ (ppm): −75.76.

HRMS (ESI-TOF) m/z: $[M+Na]^+$ Calcd for $C_{39}H_{33}F_3N_4NaO_3^+$ 685.2397, Found: 685.2400.

<u>*N*-((3*R*,6'*R*)-1-benzyl-5-methoxy-3'-methyl-6'-((*E*)-2-methylstyryl)-2-oxo-1'-phenyl-5',6'-dihydro-1'*H*-spiro[indoline-3,4'-pyrano[2,3-*c*]pyrazol]-6'-yl)-2,2,2-trifluoroacetamide (4bb)</u>

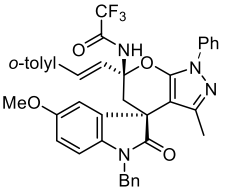

Prepared according to the general procedure, the crude product was purified by silica gel chromatography (petroleum ether/ethyl acetate 30:1) to afford **4bb** (54.9 mg, 81% yield, white solid, > 20:1 dr, m.p: 187.9 – 190.8 °C).

The enantiomeric excess was determined to be 95% by HPLC with a Daicel Chiralpak IG (*n*-hexane/2-propanol = 95:05, 1.0 mL/min, at 254 nm): t_R = 16.69 min (minor), t_R = 18.30 min (major); $[\alpha]_D^{20}$ = + 445.668 (*c* = 0.10, EA).

NMR and HRMS data for the product **4bb**:

^{1}H NMR (600 MHz, Chloroform-*d*) δ (ppm): 11.78 (s, 1H), 7.83 (d, *J* = 8.4 Hz, 2H), 7.46 (q, *J* = 7.8 Hz, 3H), 7.41 – 7.37 (m, 4H), 7.33 (t, *J* = 7.2 Hz, 1H), 7.26 – 7.24 (m, 1H), 7.21 – 7.15 (m, 3H), 7.13 (d, *J* = 15.6 Hz, 1H), 6.82 (d, *J* = 8.4 Hz, 1H), 6.80 – 6.78 (m, 1H), 6.70 (d, *J* = 1.8 Hz, 1H), 6.51 (d, *J* = 16.2 Hz, 1H), 5.14 (d, *J* = 15.0 Hz, 1H), 4.88 (d, *J* = 15.6 Hz, 1H), 3.73 (s, 3H), 2.62 (d, *J* = 15.0 Hz, 1H), 2.55 (d, *J* = 15.0 Hz, 1H), 2.36 (s, 3H), 1.50 (s, 3H).

^{13}C NMR (150 MHz, Chloroform-*d*) δ (ppm): 179.4, 157.3, 156.9 (q, *J* = 37.5 Hz), 148.9, 145.8, 138.2, 136.1, 135.6, 135.1, 134.8, 132.6, 130.4, 130.1, 129.2, 129.0, 128.6, 128.4, 128.3, 127.9, 126.2, 126.1, 126.1, 120.4, 115.5 (q, *J* = 287.6 Hz), 113.9, 110.5, 110.4, 95.9, 88.6, 55.9, 46.6, 44.7, 42.1, 19.7, 12.6.

^{19}F NMR (564 MHz, Chloroform-*d*) δ (ppm): −75.78.

HRMS (ESI-TOF) m/z: $[M+Na]^+$ Calcd for $C_{39}H_{33}F_3N_4NaO_4^+$ 701.2346, Found: 701.2355.

*N*-((3*R*,6'*R*)-1-allyl-3'-methyl-6'-((*E*)-2-methylstyryl)-2-oxo-1'-phenyl-5',6'-dihydro-1'*H*-spiro[indoline-3,4'-pyrano[2,3-*c*]pyrazol]-6'-yl)-2,2,2-trifluoroacetamide (4bc)

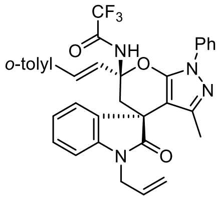

Prepared according to the general procedure, the crude product was purified by silica gel chromatography (petroleum ether/ethyl acetate 30:1) to afford **4bc** (36.2 mg, 60% yield, white solid, > 20:1 dr, m.p: 204.1 – 204.9 °C).

The enantiomeric excess was determined to be 98% by HPLC with a Daicel Chiralpak IC (*n*-hexane/2-propanol = 70:30, 1.0 mL/min, at 254 nm): t_R = 4.69 min (major), t_R = 5.10 min (minor); $[\alpha]_D^{20}$ = + 539.667 (*c* = 0.06, EA).

NMR and HRMS data for the product **4bc:**

^{1}H NMR (600 MHz, Chloroform-*d*) δ (ppm): 11.62 (s, 1H), 7.82 (d, *J* = 8.4 Hz, 2H), 7.46 – 7.43 (m, 3H), 7.36 – 7.33 (m, 1H), 7.26 – 7.24 (m, 1H), 7.19 – 7.17 (m, 2H), 7.16 – 7.14 (m, 3H), 7.11 (d, *J* = 16.2 Hz, 1H), 6.99 (d, *J* = 7.8 Hz, 1H), 6.49 (d, *J* = 16.2 Hz, 1H), 5.95 – 5.88 (m, 1H), 5.41 (d, *J* = 17.4 Hz, 1H), 5.36 (d, *J* = 10.2 Hz, 1H), 4.59 (dd, *J* = 15.6, 5.4 Hz, 1H), 4.40 (dd, *J* = 15.6, 6.0 Hz, 1H), 2.58 (s, 2H), 2.34 (s, 3H), 1.59 (s, 3H).

^{13}C NMR (150 MHz, Chloroform-*d*) δ (ppm): 179.2, 156.9 (q, *J* = 37.8 Hz), 148.9, 145.6, 142.4, 138.1, 136.0, 134.7, 131.3, 130.7, 130.3, 130.1, 129.3, 129.3, 128.6, 128.4, 126.2, 126.1, 124.4, 123.6, 120.5, 119.3, 115.5 (q, *J* = 287.4 Hz), 109.8, 95.9, 88.7, 46.2, 43.2, 41.8, 19.7, 12.6.

^{19}**F NMR** (564 MHz, Chloroform-*d*) δ (ppm): -75.83.

HRMS (ESI-TOF) m/z: [M+Na]$^+$ Calcd for $C_{34}H_{29}F_3N_4NaO_3^+$ 621.2084, Found: 621.2090.

2,2,2-trifluoro-*N*-((3*R*,6'*R*)-3'-methyl-6'-((*E*)-2-methylstyryl)-2-oxo-1'-phenyl-1-(prop-2-yn-1-yl)-5',6'-dihydro-1'*H*-spiro[indoline-3,4'-pyrano[2,3-*c*]pyrazol]-6'-yl)acetamide (4bd)

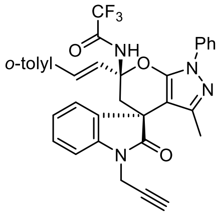

Prepared according to the general procedure, the crude product was purified by silica gel chromatography (petroleum ether/ethyl acetate 30:1) to afford **4bd** (38.8 mg, 67% yield, white solid, > 20:1 dr, m.p: 192.9 – 193.3 °C).

The enantiomeric excess was determined to be 98% by HPLC with a Daicel Chiralpak IB (*n*-hexane/2-propanol = 80:20, 1.0 mL/min, at 254 nm): t_R = 5.76 min (minor), t_R = 10.13 min (major); $[\alpha]_D^{20}$ = + 207.000 (*c* = 0.11, EA).

NMR and HRMS data for the product **4bd**:

^{1}**H NMR** (600 MHz, Chloroform-*d*) δ (ppm): 11.46 (s, 1H), 7.81 (d, *J* = 7.8 Hz, 2H), 7.47 – 7.43 (m, 3H), 7.41 (t, *J* = 7.8 Hz, 1H), 7.26 – 7.24 (m, 1H), 7.21 – 7.17 (m, 4H), 7.17 – 7.14 (m, 2H), 7.11 (d, *J* = 16.2 Hz, 1H), 6.49 (d, *J* = 16.2 Hz, 1H), 4.80 (dd, *J* = 17.4, 2.4 Hz, 1H), 4.51 (dd, *J* = 17.4, 2.4 Hz, 1H), 2.61 (d, *J* = 15.0 Hz, 1H), 2.56 (d, *J* = 15.6 Hz, 1H), 2.35 (s, 1H), 2.34 (s, 3H), 1.59 (s, 3H).

^{13}**C NMR** (150 MHz, Chloroform-*d*) δ (ppm): 178.7, 156.8 (q, *J* = 38.0 Hz), 148.7, 145.8, 141.3, 138.1, 136.0, 134.7, 131.1, 130.4, 130.2, 129.4, 129.3, 128.5, 128.4, 126.3, 126.2, 126.1, 124.8, 123.6, 120.5, 115.5 (q, *J* = 287.3 Hz), 109.9, 95.8, 88.6, 75.8, 73.4, 46.2, 41.5, 29.9, 19.7, 12.5.

^{19}**F NMR** (564 MHz, Chloroform-*d*) δ (ppm): -75.89.

HRMS (ESI-TOF) m/z: [M+Na]$^+$ Calcd for $C_{34}H_{27}F_3N_4NaO_4^+$ 619.1927, Found: 619.1937.

2,2,2-trifluoro-*N*-((1*R*,6'*R*)-3'-methyl-6'-((*E*)-2-methylstyryl)-2-oxo-1'-phenyl-5',6'-dihydro-1'*H*,2*H*-spiro [acenaphthylene-1,4'-pyrano[2,3-*c*]pyrazol]-6'-yl)acetamide (4be)

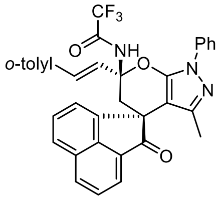

Prepared according to the general procedure, the crude product was purified by silica gel chromatography (petroleum ether/ethyl acetate 30:1) to afford **4be** (40.0 mg, 67% yield, white solid, > 20:1 dr, m.p: 180.6 – 181.0 °C).

The enantiomeric excess was determined to be 92% by HPLC with a Daicel Chiralpak IG (*n*-hexane/2-propanol = 70:30, 1.0 mL/min, at 254 nm): t_R = 5.36 min (major), t_R = 7.15 min (minor); $[\alpha]_D^{20}$ = + 307.000 (*c* = 0.10, EA).

NMR and HRMS data for the product **4be**:

^{1}H NMR (600 MHz, Chloroform-*d*) δ (ppm): 11.20 (s, 1H), 8.29 (d, *J* = 8.4 Hz, 1H), 8.18 (d, *J* = 6.6 Hz, 1H), 7.94 (d, *J* = 8.4 Hz, 1H), 7.89 (t, *J* = 7.2 Hz, 1H), 7.84 (d, *J* = 7.8 Hz, 2H), 7.71 (t, *J* = 8.4 Hz, 1H), 7.48 – 7.42 (m, 4H), 7.27 – 7.24 (m, 1H), 7.19 – 7.16 (m, 2H), 7.16 – 7.12 (m, 2H), 6.52 (d, *J* = 16.2 Hz, 1H), 2.79 (d, *J* = 15.6 Hz, 1H), 2.68 (d, *J* = 15.6 Hz, 1H), 2.34 (s, 3H), 1.19 (s, 3H).

^{13}C NMR (150 MHz, Chloroform-*d*) δ (ppm): 208.3, 156.9 (q, *J* = 37.5 Hz), 148.5, 145.8, 142.1, 141.1, 138.1, 136.0, 134.7, 133.5, 130.6, 130.4, 130.3, 130.1, 129.4, 129.3, 129.0, 128.5, 128.4, 126.2, 126.1, 126.1, 125.1, 123.9, 120.5, 120.4, 115.6 (q, *J* = 288.3 Hz), 97.6, 88.5, 51.3, 42.0, 19.7, 12.7.

^{19}F NMR (564 MHz, Chloroform-*d*) δ (ppm): -75.83.

HRMS (ESI-TOF) m/z: $[M+Na]^+$ Calcd for $C_{35}H_{26}F_3N_3NaO_3^+$ 616.1818, Found: 616.1825.

<u>*N*-((3*R*,6'*R*)-1-benzyl-1'-(3-chlorophenyl)-3'-methyl-6'-((*E*)-2-methylstyryl)-2-oxo-5',6'-dihydro-1'*H*-spiro[indoline-3,4'-pyrano[2,3-*c*]pyrazol]-6'-yl)-2,2,2-trifluoroacetamide (4bf)</u>

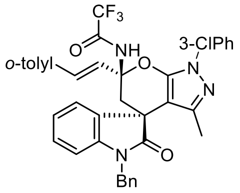

Prepared according to the general procedure, the crude product was purified by silica gel chromatography (petroleum ether/ethyl acetate 30:1) to afford **4bf** (38.6 mg, 56% yield, white solid, > 20:1 dr, m.p: 175.2 – 178.1 °C).

The enantiomeric excess was determined to be 99% by HPLC with a Daicel Chiralpak IC (*n*-hexane/2-propanol = 90:10, 1.0 mL/min, at 254 nm): t_R = 6.52 min (major), t_R = 7.58 min (minor); $[\alpha]_D^{20}$ = + 157.000 (*c* = 0.10, EA).

NMR and HRMS data for the product **4bf**:

^{1}H NMR (600 MHz, Chloroform-*d*) δ (ppm): 11.66 (s, 1H), 7.89 (s, 1H), 7.78 (d, *J* = 7.8 Hz, 1H), 7.46 – 7.45 (m, 1H), 7.42 (d, *J* = 7.2 Hz, 2H), 7.40 – 7.36 (m, 3H), 7.33 (t, *J* = 7.2 Hz, 1H), 7.30 – 7.27 (m, 1H), 7.22 (d, *J* = 7.2 Hz, 1H), 7.20 – 7.18 (m, 2H), 7.17 – 7.14 (m, 2H), 7.12 – 7.10 (m, 2H), 6.94 (d, *J* = 7.8 Hz, 1H), 6.47 (d, *J* = 15.6 Hz, 1H), 5.16 (d, *J* = 15.0 Hz, 1H), 4.91 (d, *J* = 15.0 Hz, 1H), 2.62 (d, *J* = 15.6 Hz, 1H), 2.58 (d, *J* = 15.0 Hz, 1H), 2.38 (s, 3H), 1.44 (s, 3H).

^{13}C NMR (150 MHz, Chloroform-*d*) δ (ppm): 179.6, 156.9 (q, *J* = 37.7 Hz), 149.1, 146.3, 142.4, 139.9, 139.1, 136.2, 135.0, 134.9, 134.7, 131.1, 130.4, 130.4, 129.4, 129.1, 128.4, 128.3, 128.3, 127.9, 126.2, 126.1, 126.1, 124.5, 123.6, 120.6, 118.1, 115.5 (q, *J* = 287.6 Hz), 109.9, 96.3, 88.8, 46.2, 44.7, 41.9, 19.8, 12.5.

^{19}F NMR (564 MHz, Chloroform-*d*) δ (ppm): −75.79.

HRMS (ESI-TOF) m/z: $[M+Na]^+$ Calcd for $C_{38}H_{30}ClF_3N_4NaO_3^+$ 705.1851, Found: 705.1857.

<u>*N*-((3*R*,6'*R*)-1-benzyl-1'-(3-bromophenyl)-3'-methyl-6'-((*E*)-2-methylstyryl)-2-oxo-5',6'-dihydro-1'*H*-spiro[indoline-3,4'-pyrano[2,3-*c*]pyrazol]-6'-yl)-2,2,2-trifluoroacetamide (4bg)</u>

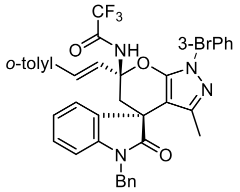

Prepared according to the general procedure, the crude product was purified by silica gel chromatography (petroleum ether/ethyl acetate 30:1) to afford **4bg** (54.6 mg, 75% yield, white solid, > 20:1 dr, m.p: 168.5 – 170.7 °C).

The enantiomeric excess was determined to be 99% by HPLC with a Daicel Chiralpak IG (*n*-hexane/2-propanol = 90:10, 1.0 mL/min, at 254 nm): t_R = 9.20 min (major), t_R = 12.01 min (minor); $[\alpha]_D^{20}$ = + 211.789 (*c* = 0.19, EA).

NMR and HRMS data for the product **4bg**:

^{1}H NMR (600 MHz, Chloroform-*d*) δ (ppm): 11.66 (s, 1H), 8.04 (t, *J* = 1.8 Hz, 1H), 7.83 – 7.82 (m, 1H), 7.46 – 7.45 (m, 1H), 7.42 (d, *J* = 7.2 Hz, 2H), 7.38 (t, *J* = 7.2 Hz, 3H), 7.35 – 7.32 (m, 2H), 7.30 – 7.27 (m, 1H), 7.22 – 7.18 (m, 2H), 7.17 – 7.14 (m, 2H), 7.12 – 7.10 (m, 2H), 6.94 (d, *J* = 8.4 Hz, 1H), 6.47 (d, *J* =

16.2 Hz, 1H), 5.16 (d, J = 15.0 Hz, 1H), 4.91 (d, J = 15.0 Hz, 1H), 2.62 (d, J = 15.6 Hz, 1H), 2.58 (d, J = 15.6 Hz, 1H), 2.38 (s, 3H), 1.44 (s, 3H).

^{13}C NMR (150 MHz, Chloroform-*d*) δ (ppm): 179.6, 156.9 (q, J = 37.7 Hz), 149.1, 146.3, 142.4, 139.2, 136.2, 135.0, 134.7, 131.1, 130.6, 130.4, 130.4, 129.4, 129.1, 129.0, 128.4, 128.3, 128.3, 127.9, 126.2, 126.1, 124.5, 123.6, 123.4, 122.8, 118.6, 115.5 (q, J = 287.6 Hz), 109.9, 96.3, 88.8, 46.2, 44.7, 41.9, 19.8, 12.5.

^{19}F NMR (564 MHz, Chloroform-*d*) δ (ppm): −75.78.

HRMS (ESI-TOF) m/z: $[M+Na]^+$ Calcd for $C_{38}H_{30}BrF_3N_4NaO_3^+$ 749.1346, Found: 749.1346.

<u>*N*-((3*R*,6'*R*)-1-benzyl-3'-methyl-6'-((*E*)-2-methylstyryl)-2-oxo-1'-(p-tolyl)-5',6'-dihydro-1'*H*-spiro[indoline-3,4'-pyrano[2,3-*c*]pyrazol]-6'-yl)-2,2,2-trifluoroacetamide (4bh)</u>

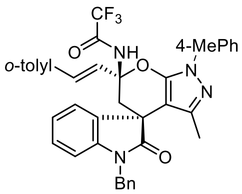

Prepared according to the general procedure, the crude product was purified by silica gel chromatography (petroleum ether/ethyl acetate 30:1) to afford **4bh** (47.5 mg, 71% yield, white solid, > 20:1 dr, m.p: 206.4 – 209.3 °C).

The enantiomeric excess was determined to be 99% by HPLC with a Daicel Chiralpak IB (*n*-hexane/2-propanol = 80:20, 1.0 mL/min, at 254 nm): t_R = 6.81min (minor), t_R = 8.18 min (major); $[\alpha]_D^{20}$ = + 261.250 (c = 0.16, EA).

NMR and HRMS data for the product **4bh**:

^{1}H NMR (600 MHz, Chloroform-*d*) δ (ppm): 11.68 (s, 1H), 7.68 (d, J = 7.8 Hz, 2H), 7.47 – 7.45 (m, 1H), 7.42 (d, J = 7.2 Hz, 2H), 7.38 (t, J = 7.8 Hz, 2H), 7.33 (t, J = 7.2 Hz, 1H), 7.28 (d, J = 7.2 Hz, 1H), 7.24 (d, J = 8.4 Hz, 2H), 7.21 – 7.17 (m, 2H), 7.16 (d, J = 7.2 Hz, 1H), 7.13 – 7.10 (m, 3H), 6.93 (d, J = 7.8 Hz, 1H), 6.49 (d, J = 16.2 Hz, 1H), 5.17 (d, J = 15.6 Hz, 1H), 4.90 (d, J = 15.0 Hz, 1H), 2.60 (d, J = 15.0 Hz, 1H), 2.57 (d, J = 15.0 Hz, 1H), 2.37 (s, 3H), 2.36 (s, 3H), 1.45 (s, 3H).

^{13}C NMR (150 MHz, Chloroform-*d*) δ (ppm): 179.8, 156.9 (q, J = 37.5 Hz), 148.7, 145.4, 142.4, 136.0, 135.9, 135.6, 135.0, 134.8, 131.4, 130.3, 130.1, 129.8, 129.2, 129.1, 128.7, 128.3, 128.3, 127.9, 126.2, 126.2, 124.4, 123.7, 120.5, 115.5 (q, J = 287.6 Hz), 109.8, 95.7, 88.6, 46.2, 44.7, 42.0, 21.0, 19.7, 12.5.

^{19}F NMR (564 MHz, Chloroform-*d*) δ (ppm): −75.79.

HRMS (ESI-TOF) m/z: $[M+Na]^+$ Calcd for $C_{39}H_{33}F_3N_4NaO_3^+$ 685.2397, Found: 685.2391.

_N_-((3_R_,6'_R_)-1-benzyl-1'-(4-fluorophenyl)-3'-methyl-6'-((_E_)-2-methylstyryl)-2-oxo-5',6'-dihydro-1'_H_-spiro[indoline-3,4'-pyrano[2,3-_c_]pyrazol]-6'-yl)-2,2,2-trifluoroacetamide (4bi)

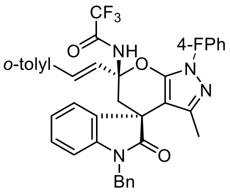

Prepared according to the general procedure, the crude product was purified by silica gel chromatography (petroleum ether/ethyl acetate 30:1) to afford **4bi** (45.3 mg, 68% yield, white solid, > 20:1 dr, m.p: 164.8 – 167.5 °C).

The enantiomeric excess was determined to be 99% by HPLC with a Daicel Chiralpak IG (*n*-hexane/2-propanol = 90:10, 1.0 mL/min, at 254 nm): t_R = 8.90 min (major), t_R = 9.94 min (minor); $[\alpha]D^{20}$ = + 324.400 (*c* = 0.10, EA).

NMR and HRMS data for the product **4bi**:

^{1}H NMR (600 MHz, Chloroform-*d*) δ (ppm): 11.70 (s, 1H), 7.78 (dd, *J* = 9.0, 4.8 Hz, 2H), 7.47 – 7.45 (m, 1H), 7.42 (d, *J* = 7.2 Hz, 2H), 7.38 (t, *J* = 7.8 Hz, 2H), 7.33 (t, *J* = 7.2 Hz, 1H), 7.30 – 7.27 (m, 1H), 7.21 – 7.19 (m, 2H), 7.15 (t, *J* = 8.4 Hz, 3H), 7.12 (s, 2H), 7.11 – 7.09 (m, 1H), 6.94 (d, *J* = 7.8 Hz, 1H), 6.49 (d, *J* = 16.2 Hz, 1H), 5.17 (d, *J* = 15.6 Hz, 1H), 4.90 (d, *J* = 15.0 Hz, 1H), 2.61 (d, *J* = 15.6 Hz, 1H), 2.58 (d, *J* = 15.0 Hz, 1H), 2.35 (s, 3H), 1.44 (s, 3H).

^{13}C NMR (150 MHz, Chloroform-*d*) δ (ppm): 179.7, 160.8 (d, J_{CF} = 243.9 Hz), 157.0 (q, *J* = 37.7 Hz), 148.7, 145.8, 142.4, 136.1, 135.0, 134.7, 134.2 (d, J_{CF} =2.9 Hz), 131.2, 130.4, 130.2, 129.3, 129.1, 128.4, 128.4, 128.3, 127.9, 126.3, 126.1, 124.4, 123.6, 122.3 (d, J_{CF} = 8.3 Hz), 116.0 (d, J_{CF} = 22.8 Hz), 115.5 (q, *J* = 287.4 Hz), 109.9, 95.9, 88.8, 46.2, 44.7, 41.9, 19.7, 12.5.

^{19}F NMR (564 MHz, Chloroform-*d*) δ (ppm): −75.80, −116.15 – −116.19.

HRMS (ESI-TOF) m/z: $[M+Na]^+$ Calcd for $C_{38}H_{30}F_4N_4NaO_3^+$ 689.2146, Found: 689.2152.

_N_-((3_R_,6'_R_)-1-benzyl-1'-(4-bromophenyl)-3'-methyl-6'-((_E_)-2-methylstyryl)-2-oxo-5',6'-dihydro-1'_H_-spiro[indoline-3,4'-pyrano[2,3-_c_]pyrazol]-6'-yl)-2,2,2-trifluoroacetamide (4bj)

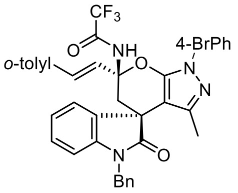

Prepared according to the general procedure, the crude product was purified by silica gel chromatography (petroleum ether/ethyl acetate 30:1) to afford **4bj** (40.0 mg, 55% yield, white solid, > 20:1 dr, m.p: 168.5 – 173.2 °C).

The enantiomeric excess was determined to be 99% by HPLC with a Daicel Chiralpak IB (*n*-hexane/2-propanol = 80:20, 1.0 mL/min, at 254 nm): t_R = 7.40 min (minor), t_R = 8.98 min (major); $[\alpha]_D^{20}$ = + 316.182 (*c* = 0.11, EA).

NMR and HRMS data for the product **4bj**:

^{1}H NMR (600 MHz, Chloroform-*d*) δ (ppm): 11.68 (s, 1H), 7.74 (d, *J* = 9.0 Hz, 2H), 7.56 (d, *J* = 8.4 Hz, 2H), 7.47 – 7.45 (m, 1H), 7.41 (d, *J* = 7.8 Hz, 2H), 7.38 (t, *J* = 7.8 Hz, 2H), 7.33 (t, *J* = 7.2 Hz, 1H), 7.29 (t, *J* = 7.8 Hz, 1H), 7.22 – 7.18 (m, 2H), 7.17 – 7.16 (m, 1H), 7.12 – 7.10 (m, 3H), 6.94 (d, *J* = 7.8 Hz, 1H), 6.49 (d, *J* = 16.2 Hz, 1H), 5.17 (d, *J* = 15.6 Hz, 1H), 4.89 (d, *J* = 15.0 Hz, 1H), 2.61 (d, *J* = 15.6 Hz, 1H), 2.58 (d, *J* = 15.6 Hz, 1H), 2.36 (s, 3H), 1.43 (s, 3H).

^{13}C NMR (150 MHz, Chloroform-*d*) δ (ppm): 179.6, 157.0 (q, *J* = 37.7 Hz), 148.9, 146.1, 142.4, 137.2, 136.1, 135.0, 134.6, 132.3, 131.1, 130.4, 130.3, 129.4, 129.1, 128.5, 128.4, 128.3, 127.9, 126.3, 126.1, 124.5, 123.6, 121.7, 119.3, 115.5 (q, *J* = 287.4 Hz), 109.9, 96.2, 88.9, 46.2, 44.7, 41.8, 19.8, 12.5.

^{19}F NMR (564 MHz, Chloroform-*d*) δ (ppm): −75.80.

HRMS (ESI-TOF) m/z: $[M+Na]^+$ Calcd for $C_{38}H_{30}BrF_3N_4NaO_3^+$ 749.1346, Found: 749.1346.

<u>*N*-((3*R*,6'*R*)-1-benzyl-5-bromo-3'-methyl-2-oxo-1'-phenyl-6'-((*E*)-styryl)-5',6'-dihydro-1'*H*-spiro[indoline-3,4'-pyrano[2,3-*c*]pyrazol]-6'-yl)-2,2,2-trifluoroacetamide (4bk)</u>

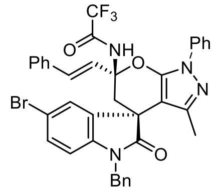

Prepared according to the general procedure, the crude product was purified by silica gel chromatography (petroleum ether/ethyl acetate 30:1) to afford **4bk** (32.5 mg, 45% yield, white solid, > 20:1 dr, m.p: 197.1 – 202.4 °C).

The enantiomeric excess was determined to be 94% by HPLC with a Daicel Chiralpak IG (*n*-hexane/2-propanol = 80:20, 1.0 mL/min, at 254 nm): t_R = 7.65 min (major), t_R = 8.97 min (minor); $[\alpha]_D^{20}$ = + 559.000 (*c* = 0.10, EA).

NMR and HRMS data for the product **4bk**:

^{1}H NMR (600 MHz, Chloroform-d) δ (ppm): 11.41 (s, 1H), 7.68 (d, *J* = 7.8 Hz, 2H), 7.35 (t, *J* = 7.8 Hz,

2H), 7.31 (d, J = 7.2 Hz, 2H), 7.28 – 7.26 (m, 4H), 7.22 (t, J = 7.8 Hz, 3H), 7.17 (t, J = 7.8 Hz, 2H), 7.14 – 7.12 (m, 2H), 6.76 (d, J = 16.2 Hz, 1H), 6.68 (d, J = 7.8 Hz, 1H), 6.56 (d, J = 16.2 Hz, 1H), 5.01 (d, J = 15.0 Hz, 1H), 4.78 (d, J = 15.0 Hz, 1H), 2.51 (d, J = 15.0 Hz, 1H), 2.43 (d, J = 15.0 Hz, 1H), 1.40 (s, 3H).

^{13}C NMR (150 MHz, Chloroform-d) δ (ppm): 179.2, 156.6 (q, J = 38.0 Hz), 148.9, 145.5, 141.4, 138.0, 135.3, 134.5, 133.3, 132.2, 132.1, 129.3, 129.2, 128.7, 128.6, 128.5, 127.8, 127.3, 127.0, 126.9, 126.3, 120.6, 117.1, 115.5 (q, J = 287.4 Hz), 111.3, 95.3, 88.6, 46.3, 44.7, 41.8, 12.7.

^{19}F NMR (377 MHz, Chloroform-d) δ (ppm): −75.72.

HRMS (ESI-TOF) m/z: $[M+Na]^+$ Calcd for $C_{37}H_{28}BrF_3N_4NaO_3^+$ 735.1189, Found: 735.1184.

5. Scale-up synthesis

5.1. Procedure for the scaled synthesis of product **3aa**

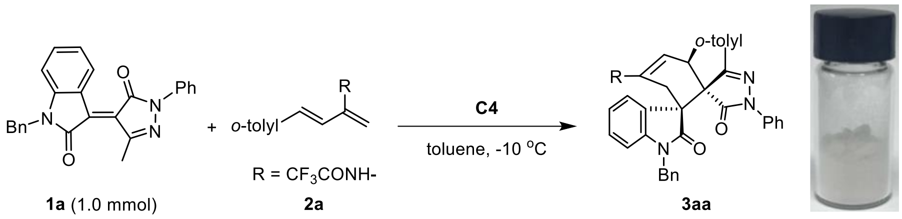

A 50 mL reaction tube was filled with **1a** (1.0 mmol, 1.0 equiv., 393.5 mg), **2a** (1.5 mmol, 1.5 equiv., 382.8 mg), (*R*)-**C4** (10 mol%, 61.0 mg) and toluene (20.0 mL). The mixture was stirred at −10 ℃ for 1.5 h, monitored by TLC. After completion, it was concentrated under reduced pressure, and the crude material was purified by column chromatography on silica gel using petroleum ether and ethyl acetate (20/1) as eluents to yield product **3aa** (545.0 mg, 13:1 dr, 84% yield, 99% ee).

5.2. Procedure for the scaled synthesis of product **4aa**

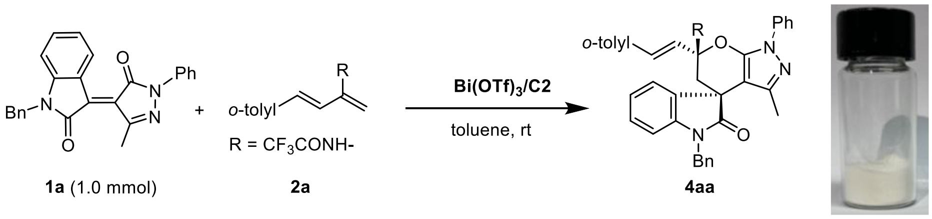

$Bi(OTf)_3$ (10 mol%, 66.0 mg) and (*R*)-**C2** (10 mol%, 75.0 mg) were added to a Schlenk tube, followed by toluene (2.0 mL) under argon. The solution was stirred at room temperature for 0.5 h. Subsequently, **1a** (1.0 mmol, 1.0 equiv., 393.5 mg) and **2a** (1.5 mmol, 1.5 equiv., 382.8 mg) were introduced. The reaction proceeded for 1 h at room temperature and was monitored by TLC. Upon completion, the mixture was concentrated under reduced pressure, and the crude material was purified via column chromatography on silica gel using petroleum ether and ethyl acetate (30/1) as eluents, yielding product **4aa** (450.0 mg, 69% yield, > 20:1 dr, 99% ee).

6. Synthetic transformations of the products

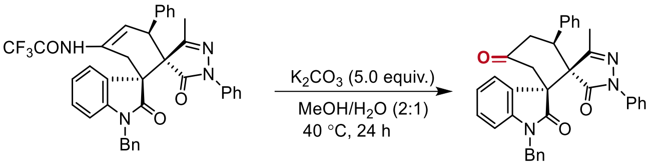

A 10 mL reaction tube was filled with **3ab** (0.1 mmol, 1.0 equiv., 63.4 mg), K_2CO_3 (0.5 mmol, 5.0 equiv., 69.0 mg), MeOH (2.0 mL) and H_2O (1 mL). The reaction mixture was stirred at 40 °C for 24 h, monitored by TLC. After completion, the reaction mixture was diluted with water, extracted with EtOAc. The organic phase was washed with brine, dried over anhydrous Na_2SO_4, filtered, and concentrated in vacuo. Then the crude material was purified by column chromatography on silica gel using petroleum ether and ethyl acetate (10/1) as eluents to yield product **5** (41.3 mg, 76% yield, > 20:1 dr, 99% ee).

(2'*S*,3*R*,3'*S*)-1-benzyl-3''-methyl-1'',3'-diphenyldispiro[indoline-3,1'-cyclohexane-2',4''-pyrazole]-2,5',5''(1''*H*)-trione (5)

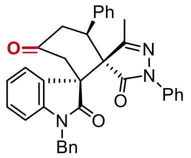

Prepared according to the general procedure, the crude product was purified by silica gel chromatography (petroleum ether/ethyl acetate 10:1) to afford **5** (41.3 mg, 76% yield, white solid, > 20:1 dr, m.p: 156.7 – 163.2 °C).

The enantiomeric excess was determined to be 99% by HPLC with a Daicel Chiralpak IB (*n*-hexane/2-propanol = 80:20, 1.0 mL/min, at 254 nm): t_R = 8.42 min (minor), t_R = 11.60 min (major); $[\alpha]_D^{20}$ = + 791.000 (c = 0.10, EA).

NMR and HRMS data for the product **5**:

^{1}H NMR (600 MHz, Chloroform-*d*) δ (ppm): 7.49 – 7.47 (m, 2H), 7.40 – 7.34 (m, 6H), 7.32 – 7.30 (m, 1H), 7.26 – 7.25(m, 2H), 7.22 – 7.18 (m, 6H), 6.90 – 6.87 (m, 2H), 5.09 (d, *J* = 15.0 Hz, 1H), 4.79 – 4.75 (m, 2H), 4.20 (d, *J* = 15.0 Hz, 1H), 3.84 (t, *J* = 14.4 Hz, 1H), 2.78 (d, *J* = 15.6 Hz, 1H), 2.23 (d, *J* = 15.0 Hz, 1H), 1.70 (s, 3H).

^{13}C NMR (150 MHz, Chloroform-*d*) δ (ppm): 205.9, 175.9, 172.1, 158.8, 141.6, 137.0, 136.7, 134.8, 129.7, 129.0, 128.9, 128.7, 128.3, 128.3, 128.1, 128.0, 127.0, 126.1, 124.0, 123.7, 120.2, 109.7, 60.9, 51.9, 44.3, 42.9, 41.7, 41.6, 14.8.

HRMS (ESI-TOF) m/z: $[M+Na]^+$ Calcd for $C_{35}H_{29}N_3NaO_3^+$ 562.2101, Found: 562.1997.

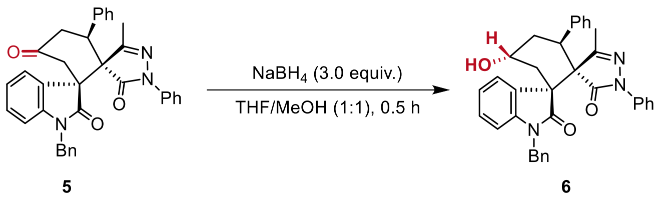

A 10 mL reaction tube was filled with **5** (0.055 mmol, 1.0 equiv., 30.0 mg), $NaBH_4$ (0.165 mmol. 3.0 equiv., 6.3 mg), THF (1 mL) and MeOH (1 mL). The reaction mixture was stirred at room temperature for 0.5 h, monitored by TLC. After completion, the reaction mixture was diluted with water, extracted with EtOAc. The organic phase was washed with brine, dried over anhydrous Na_2SO_4, filtered, and concentrated in vacuo. Then the crude material was purified by column chromatography on silica gel using petroleum ether and ethyl acetate (8/1) as eluents to yield product **6** (25.2 mg, 83% yield, > 20:1 dr, 99% ee).

(2'*S*,3*R*,3'*S*,5'*R*)-1-benzyl-5'-hydroxy-3''-methyl-1'',3'-diphenyldispiro[indoline-3,1'-cyclohexane-2',4''-pyrazole]-2,5''(1''*H*)-dione (6)

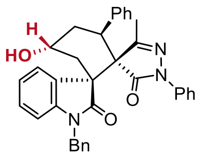

Prepared according to the general procedure, the crude product was purified by silica gel chromatography (petroleum ether/ethyl acetate 8:1) to afford **6** (25.2 mg, 83% yield, white solid, > 20:1 dr, m.p: 156.7 – 163.2 °C).

The enantiomeric excess was determined to be 99% by HPLC with a Daicel Chiralpak IG (*n*-hexane/2-propanol = 70:30, 1.0 mL/min, at 254 nm): t_R = 10.81 min (minor), t_R = 28.81 min (major); $[\alpha]_D^{20}$ = + 591.600 (*c* = 0.10, EA).

NMR and HRMS data for the product **6**:

^{1}H NMR (600 MHz, Chloroform-*d*) δ (ppm): 7.43 – 7.42 (m, 2H), 7.36 (d, *J* = 4.2 Hz, 4H), 7.34 – 7.30 (m, 4H), 7.25 – 7.24 (m, 2H), 7.19 – 7.14 (m, 5H), 6.90 – 6.87 (m, 1H), 6.83 (d, *J* = 7.8 Hz, 1H), 5.76 (s, 1H), 5.26 (d, *J* = 15.0 Hz, 1H), 4.70 (d, *J* = 15.6 Hz, 1H), 4.50 (dd, *J* = 13.8, 3.6 Hz, 1H), 4.42 (s, 1H), 3.37 (td, *J* = 14.4, 4.2 Hz, 1H), 3.30 (dd, *J* = 15.0, 4.2 Hz, 1H), 2.10 (d, *J* = 13.8 Hz, 1H), 1.88 (d, *J* = 15.0 Hz, 1H), 1.77 (s, 3H).

^{13}C NMR (150 MHz, Chloroform-*d*) δ (ppm): 179.9, 172.0, 159.0, 141.2, 139.0, 136.9, 134.5, 129.3, 129.1, 129.0, 128.7, 128.5, 128.2, 128.1, 127.8, 127.7, 125.8, 124.8, 123.9, 120.3, 109.5, 66.7, 61.1, 50.6, 44.4, 37.4, 33.4, 33.1, 15.3.

HRMS (ESI-TOF) m/z: [M+Na]$^+$ Calcd for $C_{35}H_{31}N_3NaO_3^+$ 564.2258, Found: 564.2263.

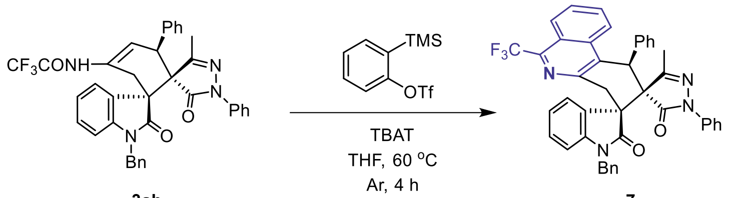

3ab 7

3ab (0.1 mmol, 1.0 equiv., 63.4 mg), silyl aryl triflate (0.2 mmol, 2.0 equiv., 59.6 mg) and TBAT (0.2 mmol, 2.0 equiv.,107.9 mg) were added to a Schlenk tube, followed by THF (2.0 mL) under argon. The solution was stirred at 60 °C for 4 h and was monitored by TLC. Upon completion, the mixture was concentrated under reduced pressure, and the crude material was purified via column chromatography on silica gel using petroleum ether and ethyl acetate (10/1) as eluents, yielding product **7** (61.3 mg, 88% yield, > 20:1 dr, 99% ee).

(1'*R*,2'*S*,3*R*)-1-benzyl-3''-methyl-1',1''-diphenyl-6'-(trifluoromethyl)-1',4'-dihydrodispiro[indoline-3,3'-phenanthridine-2',4''-pyrazole]-2,5''(1''*H*)-dione (7)

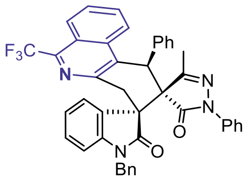

Prepared according to the general procedure, the crude product was purified by silica gel chromatography (petroleum ether/ethyl acetate 10:1) to afford **7** (63.1 mg, 88% yield, white solid, > 20:1 dr, m.p: 142,4 – 147.5 °C).

The enantiomeric excess was determined to be 99% by HPLC with a Daicel Chiralpak ID (*n*-hexane/2-propanol = 80:20, 1.0 mL/min, at 254 nm): t_R = 19.09 min (minor), t_R = 25.22 min (major); $[\alpha]_D^{20}$ = + 610.000 (*c* = 0.16, EA).

NMR and HRMS data for the product **7**:

^{1}**H NMR** (600 MHz, Chloroform-*d*) δ (ppm): 8.16 (d, *J* = 8.4 Hz, 1H), 8.13 (d, *J* = 7.2 Hz, 1H), 7.85 (t, *J* = 7.8 Hz, 1H), 7.67 (t, *J* = 7.2 Hz, 1H), 7.58 (d, *J* = 7.8 Hz, 2H), 7.29 – 7.25 (m, 3H), 7.14 (t, *J* = 7.8 Hz, 1H), 7.11 (t, *J* = 7.8 Hz, 1H), 7.08 (d, *J* = 7.2 Hz, 5H), 7.02 (t, *J* = 7.8 Hz, 1H), 6.99 (d, *J* = 4.2 Hz, 2H), 6.95 (t, *J* = 7.8 Hz, 2H), 6.79 (d, *J* = 7.8 Hz, 1H), 6.54 (d, *J* = 7.8 Hz, 1H), 5.71 (s, 1H), 5.13 (d, *J* = 15.6 Hz, 1H), 4.57 (d, *J* = 15.6 Hz, 1H), 4.49 (d, *J* = 18.6 Hz, 1H), 3.31 (d, *J* = 18.6 Hz, 1H), 2.32 (s, 3H).

^{13}C NMR (150 MHz, Chloroform-*d*) δ (ppm): 174.5, 170.7, 158.5, 155.2, 147.7, 142,5, 138.7, 137.3, 134.9, 130.7, 129.5, 129.4, 129.3, 129.3, 129.0, 128.7, 128.6, 128.2, 128.0, 128.0, 127.9, 127.5, 126.9, 126.8, 125.3, 124.9, 124.9, 124.0, 123.8, 123.7 (q, J = 276.5 Hz), 123.2, 119.5, 109.6, 62.8, 51.3, 49.4, 44.2, 39.6, 20.5.

^{19}F NMR (564 MHz, Chloroform-*d*) δ (ppm): −52.69.

HRMS (ESI-TOF) m/z: $[M+Na]^+$ Calcd for $C_{43}H_{33}F_3N_4NaO_3^+$ 715.2291, Found: 715.2281.

Procedure for the claisen rearrangement of product **4**

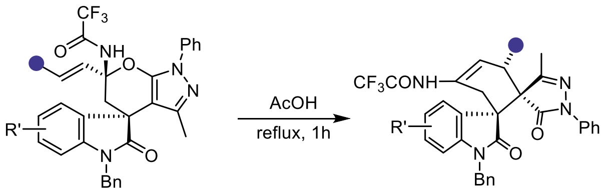

A 10 mL reaction tube was filled with **4** (0.05 mmol) and AcOH (1 mL). The reaction mixture was refluxed for 1 h, monitored by TLC. After completion, the reaction mixture was diluted with water, extracted with EtOAc. The organic phase was washed with brine, dried over anhydrous Na_2SO_4, filtered, and concentrated in vacuo. Then the crude material was purified by column chromatography on silica gel using petroleum ether and ethyl acetate (10/1) as eluents to yield product **3'**.

Procedure for the claisen rearrangement of product ***ent-4***

A 10 mL reaction tube was filled with ***ent*-4** (0.05 mmol) and AcOH (1 mL). The reaction mixture was refluxed for 1 h, monitored by TLC. After completion, the reaction mixture was diluted with water, extracted with EtOAc. The organic phase was washed with brine, dried over anhydrous Na_2SO_4, filtered, and concentrated in vacuo. Then the crude material was purified by column chromatography on silica gel using petroleum ether and ethyl acetate (10/1) as eluents to yield product ***ent*-3'**.

<u>*N*-((3*R*,5'*R*,6'*S*)-1-benzyl-3''-methyl-2,5''-dioxo-1''-phenyl-5'-(*o*-tolyl)-1'',5''-dihydrodispiro [indoline-3,1'-cyclohexene-6',4''-pyrazol]-3'-yl)-2,2,2-trifluoroacetamide (3aa')</u>

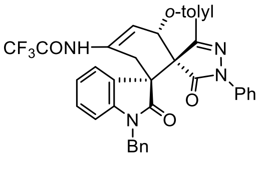

Prepared according to the general procedure, the crude product was purified by silica gel chromatography (petroleum ether/ethyl acetate 10:1) to afford **3aa'** (31.8 mg, 98% yield, white solid, > 20:1 dr, m.p: 180.3 – 185.6 °C).

The enantiomeric excess was determined to be 99% by HPLC with a Daicel Chiralpak IB (*n*-hexane/2-propanol =90:10, 1.0 mL/min, at 254 nm): t_R = 6.05 min (minor), t_R = 6.47 min (major); $[\alpha]_D^{20}$ = + 559.000 (*c* = 0.10, EA).

NMR and HRMS data for the product **3aa'**:

^{1}H NMR (600 MHz, Chloroform-*d*) δ(ppm): 7.44 (s, 1H), 7.40 (t, *J* = 7.8 Hz, 5H), 7.37 (t, *J* = 7.8 Hz, 2H), 7.32 – 7.27 (m, 3H), 7.22 – 7.19 (m, 1H), 7.14 (dd, *J* = 13.8, 7.2 Hz, 2H), 7.05 – 6.99 (m, 3H), 6.89 – 6.86 (m, 2H), 6.10 (s, 1H), 5.38 (s, 1H), 5.15 (d, *J* = 15.0 Hz, 1H), 4.77 (d, *J* = 15.6 Hz, 1H), 3.94 – 3.90 (m, 1H), 2.57 (d, *J* = 18.0 Hz, 1H), 2.36 (s, 3H), 1.76 (s, 3H).

^{13}C NMR (150 MHz, Chloroform-*d*) δ (ppm): 176.1, 171.5, 158.2, 154.5 (q, *J* = 36.9 Hz), 141.6, 136.8, 136.1, 135.3, 135.0, 130.6, 130.2, 129.6, 129.2, 129.0, 128.7, 128.2, 128.0, 127.8, 127.7, 126.1, 125.7, 124.1, 123.7, 119.9, 117.1, 115.6 (q, *J* = 287.3 Hz), 109.4, 59.4, 48.4, 44.3, 37.6, 32.1, 20.0, 15.6.

^{19}F NMR (564 MHz, Chloroform-*d*) δ (ppm): −75.71.

HRMS (ESI-TOF) m/z: $[M+Na]^+$ Calcd for $C_{38}H_{31}F_3N_4NaO_3^+$ 671.2240, Found: 671.2235.

<u>*N*-((3*R*,5'*R*,6'*S*)-1-benzyl-3''-methyl-2,5''-dioxo-1''-phenyl-5'-(thiophen-2-yl)-1'',5''-dihydrodispiro [indoline-3,1'-cyclohexene-6',4''-pyrazol]-3'-yl)-2,2,2-trifluoroacetamide (3as')</u>

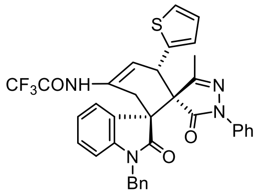

Prepared according to the general procedure, the crude product was purified by silica gel chromatography (petroleum ether/ethyl acetate 10:1) to afford **3as'** (35.1 mg, 98% yield, white solid, 20:1 dr, m.p: 187.3 – 189.5 °C).

The enantiomeric excess was determined to be 99% by HPLC with a Daicel Chiralpak IG (*n*-hexane/2-propanol = 80:20, 1.0 mL/min, at 254 nm): t_R = 9.00 min (major), t_R = 10.43 min (minor); $[\alpha]_D^{20}$ = + 560.443 (*c* = 0.11, EA).

NMR and HRMS data for the product **3as'**:

^{1}H NMR (600 MHz, Chloroform-*d*) δ (ppm): 7.43 – 7.40 (m, 4H), 7.38 – 7.36(m, 3H), 7.32 – 7.28 (m, 3H), 7.21 (t, *J* = 7.8 Hz, 1H), 7.15 (t, *J* = 7.8 Hz, 1H), 7.08 – 7.06 (m, 2H), 6.95 (d, *J* = 3.6 Hz, 1H), 6.90

(d, J = 7.8 Hz, 1H), 6.86 (t, J = 7.8 Hz, 1H), 6.84 – 6.83 (m, 1H), 6.30 (s, 1H), 5.25 (s, 1H), 5.11 (d, J = 15.0 Hz, 1H), 4.79 (d, J = 15.0 Hz, 1H), 3.79 (d, J = 17.4 Hz, 1H), 2.57 (d, J = 18.0 Hz, 1H), 1.82 (s, 3H).

^{13}C NMR (150 MHz, Chloroform-*d*) δ (ppm): 175.7, 170.8, 158.0, 154.6 (q, J = 36.5 Hz), 141.6, 138.8, 136.8, 135.0, 129.7, 129.6, 129.0, 128.7, 128.2, 128.1, 127.5, 127.0, 126.8, 125.8, 125.1, 123.8, 123.7, 119.9, 115.5 (q, J = 287.6 Hz), 115.2, 109.4, 59.8, 48.2, 44.3, 37.4, 31.8, 14.8.

^{19}F NMR (564 MHz, Chloroform-*d*) δ (ppm): −75.72.

HRMS (ESI-TOF) m/z: $[M+Na]^+$ Calcd for $C_{35}H_{27}F_3N_4NaO_3S^+$ 663.1648, Found: 663.1647.

<u>*N*-((3*R*,5'*R*,6'*S*)-1-benzyl-5-bromo-3''-methyl-2,5''-dioxo-1'',5'-diphenyl-1'',5''-dihydrodispiro [indoline-3,1',3'-cyclohexene-6',4''-pyrazol]-3'-yl)-2,2,2-trifluoroacetamide (3bk')</u>

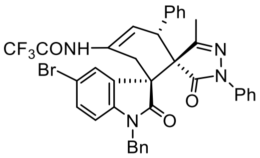

Prepared according to the general procedure, the crude product was purified by silica gel chromatography (petroleum ether/ethyl acetate 10:1) to afford **3bk'** (35.0 mg, 98% yield, white solid, > 20:1 dr, m.p: 197.1 – 202.4 °C).

The enantiomeric excess was determined to be 97% by HPLC with a Daicel Chiralpak IB (*n*-hexane/2-propanol =90:10, 1.0 mL/min, at 254 nm): t_R = 14.95 min (major), t_R = 10.82 min (minor); $[\alpha]_D^{20}$ = + 559.000 (c = 0.10, EA).

NMR and HRMS data for the product **3bk'**:

^{1}H NMR (600 MHz, Chloroform-d) δ (ppm): 7.45 (s, 1H), 7.37 (d, J = 4.2 Hz, 4H), 7.35 – 7.32 (m, 2H), 7.29 – 7.26 (m, 2H), 7.23 – 7.21 (m, 5H), 7.19 (d, J = 7.8 Hz, 3H), 7.15 (t, J = 7.2 Hz, 1H), 6.77 (d, J = 8.4 Hz, 1H), 6.26 (s, 1H), 5.13 (d, J = 15.6 Hz, 1H), 4.87 (s, 1H), 4.75 (d, J = 15.0 Hz, 1H), 3.82 (d, J = 18.0 Hz, 1H), 2.57 (d, J = 17.4 Hz, 1H), 1.83 (s, 3H).

^{13}C NMR (150 MHz, Chloroform-d) δ (ppm): 175.2, 170.6, 157.5, 154.6 (q, J = 37.1 Hz), 140.6, 136.4, 136.3, 134.6, 132.6, 130.1, 129.7, 129.3, 129.1, 129.1, 128.7, 128.4, 128.1, 128.0, 127.3, 126.3, 121.0, 116.4, 115.7, 115.6 (q, J = 287.4 Hz), 110.8, 59.6, 48.3, 44.4, 41.9, 31.6, 14.9.

^{19}F NMR (377 MHz, Chloroform-d) δ (ppm): −75.68.

HRMS (ESI-TOF) m/z: $[M+Na]^+$ Calcd for $C_{37}H_{28}BrF_3N_4NaO_3^+$ 735.1189, Found: 735.1188.

7. Interconversion experiments

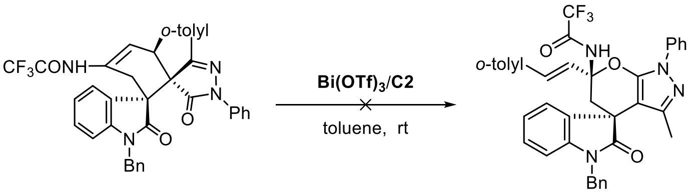

3aa 4aa

3aa (0.05 mmol, 28.0 mg), Bi(OTf)3 (10 mol%, 3.3 mg), (*R*)-**C2** (10 mol%, 3.8 mg) were dissolved in toluene (1.0 mL), and the mixture was stirred at r.t. for 24 h. No reaction was observed.

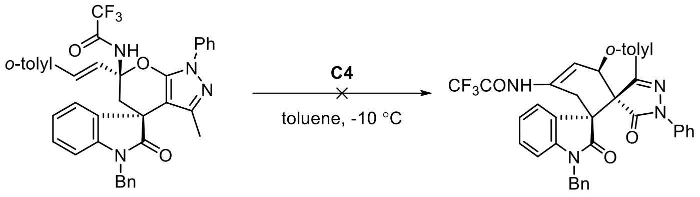

4aa 3aa

4aa (0.03 mmol, 20.0 mg) and (*R*)-**C4** (10 mol%, 1.8 mg) were dissolved in toluene (1.0 mL), and the mixture was stirred at r.t. for 24 h. No reaction was observed.

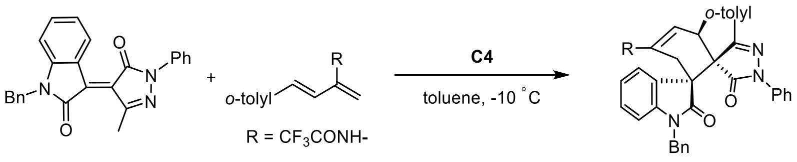

1a 2a 3aa

A 10 mL reaction tube was charged with **1a** (0.1 mmol, 1.0 equiv., 39.3 mg), **2a** (0.15 mmol, 1.5 equiv., 38.2 mg), (*R*)-**C4** (10 mol%, 6.1 mg) and toluene (2.0 mL). The reaction mixture was stirred at −10 °C for 96 h. No generation of **4aa** was observed.

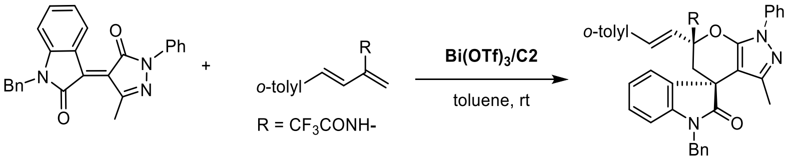

1a 2a 4aa

Bi(OTf)3 (10 mol%, 6.6 mg) and (*R*)-**C2** (10 mol%, 7.5 mg) were added to a Schlenk tube, followed by toluene (2.0 mL) under argon. The solution was stirred at room temperature for 0.5 h. Subsequently, **1** (0.1 mmol, 1.0 equiv., 39.3 mg) and **2** (0.15 mmol, 1.5 equiv., 38.2 mg) were introduced. The reaction proceeded at room temperature for 96 h. No generation of **3aa** was observed.

8. Nonlinear effect experiment

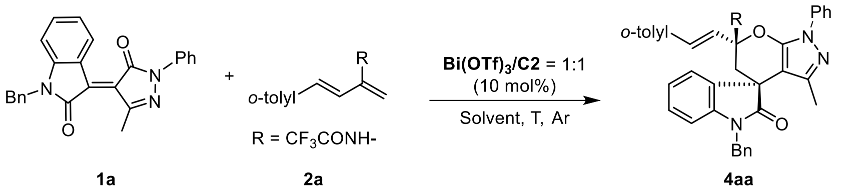

Entry	er of **C2**	ee of **4aa**‡
1	50:50	0
2	60:40	23
3	70:30	40
4	80:20	58
5	90:10	81
6	99.5:0.5	99

Reaction condition: **1a** (0.05 mmol, 1.0 equiv., 20.0 mg), **2a** (0.075 mmol, 1.5 equiv., 19.1 mg), $Bi(OTf)_3$ (10 mol%) and (*R*)-**C2** (10 mol%) in 1.0 mL toluene at r.t. for 5 min. ‡The ee determined by HPLC analysis.

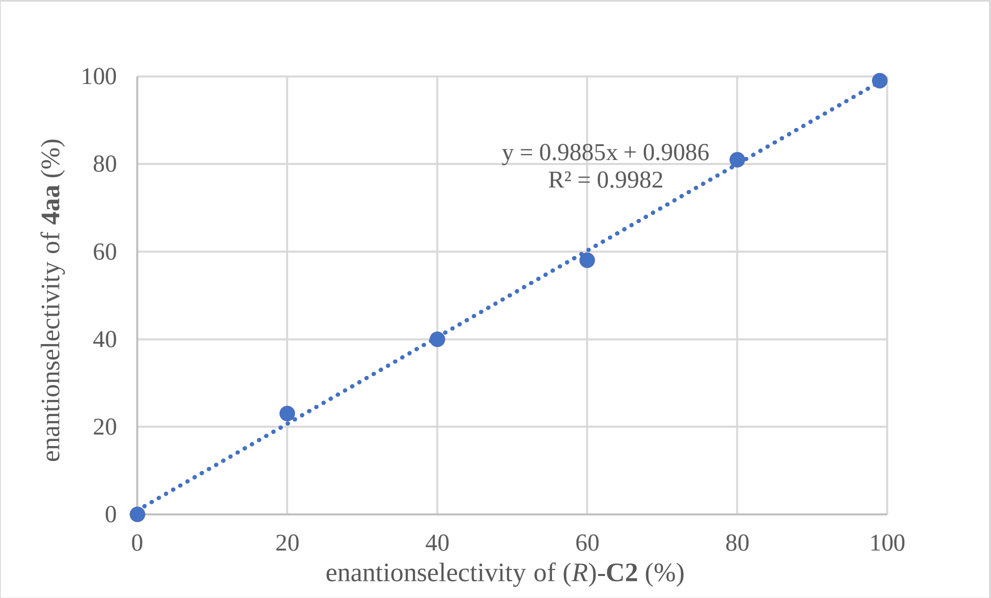

Fig. S1. Nonlinear effect experiment

9. X-ray crystal structure and data

Crystal of 3ai: to a 5 mL tube containing **3ai** (20.0 mg) was added a 4:1 mixture of *n*-hexane (4 mL), and $CDCl_3$ (1 mL). A clear solution was obtained through ultrasound treatment and was kept at room temperature to get the crystals of **3ai**, which was characterized by X-ray diffraction using an Agilent Gemini instrument. CCDC 2340519 (**3ai**) contains the supplementary crystallographic data for this paper. These data can be obtained free of charge via www.ccdc.cam.ac.uk/data_request/cif.

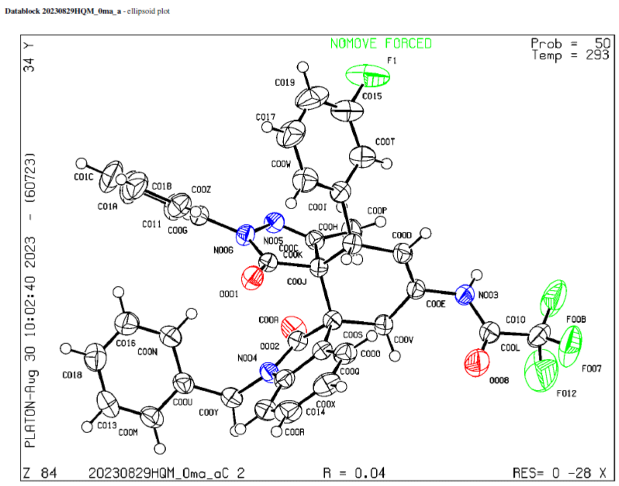

≡

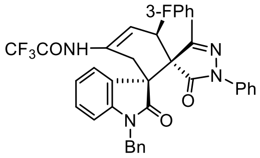

3ai, CCDC 2340519

(Ellipsoid contour probability 50%)

Identification code	20230829HQM
Empirical formula	$C_{37}H_{28}F_4N_4O_3$
Formula weight	652.63
Temperature/K	293.15
Crystal system	monoclinic
Space group	C 1 2 1
a/Å	25.5022(9)
b/Å	10.5499(3)
c/Å	13.6715(4)
α/°	90
β/°	122.3960(10)
γ/°	90
Volume/Å^3	3105.79(17)
Z	4
ρ_{calc}g/cm^3	1.396
μ/mm^{-1}	0.894

F(000)	1352
Radiation	CuKα (λ = 1.54178)
2Θ range for data collection/°	3.83 to 68.34
Index ranges	$-30 \leq h \leq 30$, $-11 \leq k \leq 12$, $-16 \leq l \leq 16$
Reflections collected	34338
Independent reflections	5597 [R_{int} = 0.0652, R_{sigma} = 0.0368]
Data/restraints/parameters	5597/1/438
Goodness-of-fit on F^2	1.009
Final R indexes [I>=2σ (I)]	R_1 = 0.0396, wR_2 = 0.1028
Final R indexes [all data]	R_1 = 0.0438, wR_2 = 0.1045
Largest diff. peak and hole / e Å^{-3}	0.294/-0.292
Flack parameter	0.005(5)

Crystal of 4aa: to a 5 mL tube containing **4aa** (20.0 mg) was added a 4:1 mixture of *n*-hexane (4 mL), and EA (1 mL). A clear solution was obtained through ultrasound treatment and was kept at room temperature to get the crystals of **4aa**, which was characterized by X-ray diffraction using an Agilent Gemini instrument. CCDC 2340520 (**4aa**) contains the supplementary crystallographic data for this paper. These data can be obtained free of charge via www.ccdc.cam.ac.uk/data_request/cif.

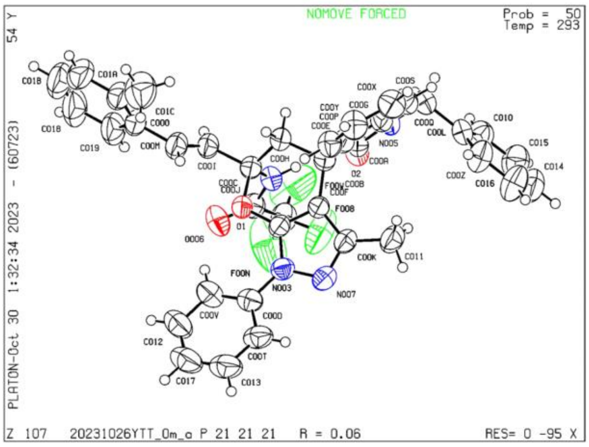

≡

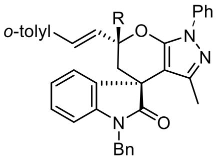

R = CF_3CONH-

4aa, CCDC 2340520

(Ellipsoid contour probability 50%)

Identification code	20231026YTT
Empirical formula	$C_{37}H_{31}F_3N_4O_3$
Formula weight	648.67
Temperature/K	293.15

Crystal system	orthorhombic
Space group	P 21 21 21
a/Å	12.2070(3)
b/Å	14.2542(4)
c/Å	18.6904(5)
α/°	90
β/°	90
γ/°	90
Volume/Å^3	3252.15(15)
Z	4
ρ_{calc}g/cm^3	1.325
μ/mm^{-1}	0.804
F(000)	1352
Radiation	CuKα (λ = 1.54178)
2Θ range for data collection/°	3.90 to 68.58
Index ranges	$-14 \leq h \leq 11$, $-17 \leq k \leq 17$, $-22 \leq l \leq 22$
Reflections collected	27353
Independent reflections	5959 [R_{int} = 0.0590, R_{sigma} = 0.0390]
Data/restraints/parameters	5959/0/435
Goodness-of-fit on F^2	1.067
Final R indexes [I>=2σ (I)]	R_1 = 0.0599, wR_2 = 0.1511
Final R indexes [all data]	R_1 = 0.0754, wR_2 = 0.1699
Largest diff. peak and hole / e Å^{-3}	0.239/-0.312
Flack parameter	0.01(11)

Crystal of 3bk': to a 5 mL tube containing **3bk'** (20.0 mg) was added a 4:1 mixture of *n*-hexane (4 mL), and EA (1 mL). A clear solution was obtained through ultrasound treatment and was kept at room temperature to get the crystals of **3bk'**, which was characterized by X-ray diffraction using an Agilent Gemini instrument. CCDC 2342541 (**3bk'**) contains the supplementary crystallographic data for this paper. These data can be obtained free of charge via www.ccdc.cam.ac.uk/data_request/cif.

Datablock 20230829HQM_0ma_a - ellipsoid plot

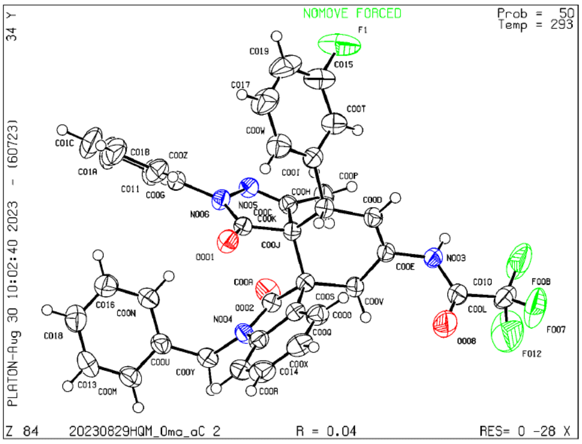

≡

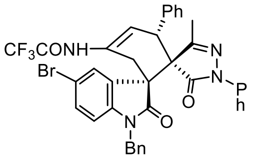

R = CF_3CONH-

3bk', CCDC (2342541)

(Ellipsoid contour probability 50%)

Identification code	20231227YTT
Empirical formula	$C_{78}H_{64}Br_2F_6N_8O_8$
Formula weight	1515.19
Temperature/K	293.15
Crystal system	triclinic
Space group	P-1
a/Å	11.6007(3)
b/Å	13.5353(4)
c/Å	24.7336(7)
α/°	92.977(2)
β/°	90.516(2)
γ/°	112.7210(10)
Volume/Å^3	3575.64(17)
Z	2
ρ_{calc}g/cm^3	1.407
μ/mm^{-1}	2.095
F(000)	1552
Radiation	CuKα (λ = 1.54178)
2Θ range for data collection/°	3.55 to 68.60
Index ranges	$-13 \le h \le 13$, $-14 \le k \le 16$, $-28 \le l \le 29$
Reflections collected	83357
Independent reflections	12818 [R_{int} = 0.0764, R_{sigma} = 0.0462]
Data/restraints/parameters	12818/2/938

Goodness-of-fit on F^2	1.047
Final R indexes [I>=2σ (I)]	R_1 = 0.0606, wR_2 = 0.1522
Final R indexes [all data]	R_1 = 0.0999, wR_2 = 0.1852
Largest diff. peak and hole / e $Å^{-3}$	0.630/-0.821

10. DFT calculations

Computational methods:

All density functional theory (DFT) calculations were performed with Gaussian 16 program software. A SMD solvation model was utilized to simulate the solvent effect of toluene solution. The Geometry of catalyst models optimizations were operated to locate all of the stationary points in toluene solution, using PBE0 density functional theory method including the DFT-D3 dispersion correction with BJ-damping and the def2-SVP basis set. All pertinent spin states including low and high spin were evaluated for the complexes in their reactant, transition and product states. The lowest free energy spin multiplicities were selected to calculate thermodynamics (*ΔG*) and free energy barriers (*ΔG*‡) for the studied reactions. Single-point calculations for all stationary points were performed with the same functional and a larger basis set, def2-TZVP basis set, to obtain more accurate free energies. The SMD implicit solvation model was used to account for the solvation effect of toluene. Atomic charges were analyzed by natural populationanalysis·(NPA) with·larger basis·set·def2-TZVP which is same as the basis set used in energy calculation to investigate the electronic properties for optimized structures.

Cartesian coordinates of DFT-computed structures:

C4-1a+2a:

C	3.4406	7.2167	0.0750	C	-3.7868	-0.1532	1.2602
C	3.2424	5.8151	0.6737	C	-2.5279	0.0020	1.8464
C	2.3789	4.9257	-0.1743	C	-2.3939	0.7544	3.0099
C	1.1730	5.4920	-0.6241	C	-3.5148	1.3436	3.5984
C	0.9374	6.9405	-0.3086	C	-4.7964	1.1707	3.0623
C	2.1868	7.7601	-0.6336	C	-5.7887	-0.7945	0.1837
H	3.7507	7.8942	0.8854	C	-4.2719	-0.9344	0.1402
H	4.2078	5.3458	0.9033	H	-1.6618	-0.4726	1.4008
C	2.6582	3.5755	-0.4496	H	-1.4033	0.8680	3.4528
C	0.2558	4.7184	-1.3277	H	-3.3969	1.9413	4.5053
H	0.0643	7.3177	-0.8612	H	-5.6714	1.6148	3.5380
H	2.0226	8.8166	-0.3724	N	-6.0741	0.0407	1.2426
C	0.4762	3.3586	-1.5718	O	-6.6183	-1.2500	-0.5695
C	1.6884	2.8203	-1.1269	C	-3.6025	-1.6454	-0.8186
H	-0.6731	5.1721	-1.6828	C	-4.0610	-2.6936	-1.7350
C	3.9099	2.8950	-0.0209	C	-2.1705	-1.4512	-1.1987
C	5.1781	3.3208	-0.4510	C	-5.3444	-3.4425	-1.7916
C	3.8178	1.7331	0.7661	H	-6.1426	-2.8332	-2.2321
C	5.4231	4.4474	-1.4142	H	-5.1876	-4.3462	-2.3947
C	6.3197	2.6108	-0.0415	H	-5.6868	-3.7253	-0.7866

C	4.9355	0.9700	1.1318	O	-1.3385	-0.6655	-0.7624
C	6.5597	5.3825	-0.9719	N	-3.0915	-3.0681	-2.5088
H	4.5070	5.0142	-1.6223	N	-1.9826	-2.3095	-2.2542
C	7.6523	3.1318	-0.4959	C	-0.8757	-2.4228	-3.1238
C	6.1863	1.4709	0.7395	C	-1.0547	-3.0653	-4.3556
C	7.7230	4.6444	-0.2856	C	0.3719	-1.8927	-2.7883
H	6.9178	5.9285	-1.8582	C	0.0121	-3.1642	-5.2443
H	8.4672	2.6210	0.0381	H	-2.0308	-3.4835	-4.5988
H	7.0866	0.9338	1.0465	C	1.4269	-1.9998	-3.6889
H	8.6898	5.0304	-0.6429	H	0.5349	-1.4142	-1.8290
H	0.6982	7.0466	0.7659	C	1.2587	-2.6304	-4.9195
H	2.3334	7.7301	-1.7249	H	-0.1367	-3.6704	-6.2012
H	4.2793	7.1953	-0.6369	H	2.3978	-1.5882	-3.4044
H	2.7262	5.9213	1.6450	H	2.0960	-2.7189	-5.6148
H	5.7094	3.9838	-2.3756	C	-8.2697	4.6135	1.5540
H	6.1609	6.1466	-0.2879	C	-7.4284	4.0513	0.5939
H	7.6956	4.8340	0.7992	C	-8.8222	3.8035	2.5450
H	7.7896	2.9026	-1.5692	C	-7.1454	2.6880	0.6254
C	-0.5678	2.5195	-2.1992	H	-6.9880	4.6779	-0.1854
C	-1.8767	2.6114	-1.7616	C	-8.5331	2.4398	2.5762
C	-0.2549	1.6086	-3.2444	H	-9.4776	4.2363	3.3045
C	-2.9031	1.8171	-2.3266	C	-7.6958	1.8685	1.6143
H	-2.1285	3.2869	-0.9399	H	-6.4802	2.2508	-0.1213
C	-1.2296	0.8285	-3.8148	H	-8.9617	1.8109	3.3620
H	0.7766	1.5278	-3.5900	C	-7.4151	0.3856	1.6381
C	-4.2497	1.8994	-1.8837	H	-7.6200	-0.0183	2.6438
C	-2.5742	0.8988	-3.3698	H	-8.0760	-0.1433	0.9333
H	-0.9724	0.1279	-4.6125	H	-8.4909	5.6831	1.5323
C	-5.2236	1.1026	-2.4401	C	3.9568	-4.8244	-3.0337
H	-4.4924	2.6134	-1.0919	C	3.2950	-5.6757	-3.9146
C	-3.5961	0.0730	-3.9051	C	1.9697	-6.0308	-3.6646
C	-4.8911	0.1690	-3.4488	C	1.3260	-5.5274	-2.5431
H	-6.2586	1.1701	-2.0982	C	1.9787	-4.6638	-1.6476
H	-3.3332	-0.6491	-4.6826	C	3.3271	-4.3073	-1.8973
H	-5.6688	-0.4761	-3.8631	H	4.9955	-4.5450	-3.2313
C	4.8636	-0.3432	1.8146	H	3.8137	-6.0611	-4.7960
C	3.9477	-0.6505	2.8052	H	1.4356	-6.6948	-4.3481
C	5.7980	-1.3460	1.4222	H	0.2851	-5.8027	-2.3628
C	3.9334	-1.9264	3.4251	C	4.0765	-3.3822	-0.9840
H	3.2079	0.0846	3.1223	H	3.5735	-2.4084	-0.8743
C	5.8058	-2.5883	2.0023	H	4.1689	-3.7973	0.0319
H	6.5019	-1.1284	0.6167	H	5.0935	-3.2034	-1.3589
C	2.9986	-2.2500	4.4433	C	1.2858	-4.0814	-0.5024
C	4.8737	-2.9213	3.0165	C	-0.0263	-4.1936	-0.2145
H	6.5221	-3.3447	1.6711	H	1.8937	-3.4482	0.1462
C	2.9807	-3.5036	5.0100	C	-0.7056	-3.4690	0.8499
H	2.2716	-1.4944	4.7432	H	-0.6854	-4.8057	-0.8349
C	4.8348	-4.2029	3.6225	C	-2.0117	-3.6814	1.1155
C	3.9060	-4.4901	4.5960	H	-2.5682	-3.0936	1.8427
H	2.2417	-3.7417	5.7782	H	-2.5347	-4.4868	0.5981
H	5.5540	-4.9602	3.2991	N	0.0545	-2.4644	1.4828
H	3.8798	-5.4826	5.0524	H	0.6792	-1.9200	0.8652

O	1.9038	1.4542	-1.2653	C	0.0390	-1.9773	2.7431
O	2.5503	1.3245	1.1588	C	-0.6284	-2.8056	3.8705
P	1.6096	0.5887	0.0778	O	0.5589	-0.9252	3.0550
O	1.8567	-0.8564	-0.1508	F	-0.1114	-2.4491	5.0389
O	0.1763	1.0064	0.5784	F	-0.4495	-4.1141	3.7250
H	-0.5178	0.4929	0.0881	F	-1.9436	-2.5623	3.9344
C	-4.9128	0.4106	1.9090				

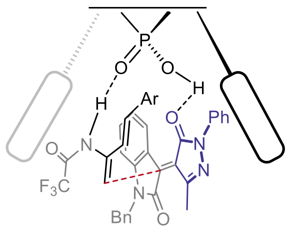

C4-TS1:

C	6.1265	-5.1126	-2.3058	C	-3.4562	-0.2534	-1.5528
C	5.6471	-3.7111	-1.9631	C	-2.2717	-0.7701	-2.0571
C	4.4476	-3.6892	-1.0459	C	-2.3118	-1.7270	-3.0759
C	3.5903	-4.7998	-0.9712	C	-3.5298	-2.1712	-3.5848
C	3.9417	-6.1121	-1.6317	C	-4.7363	-1.6315	-3.1255
C	4.9589	-5.9777	-2.7545	C	-5.2657	1.0508	-0.7457
H	6.9013	-5.0603	-3.0866	C	-3.7922	0.7380	-0.4884
H	6.4664	-3.1099	-1.5510	H	-1.3175	-0.4205	-1.6864
C	4.1050	-2.5112	-0.3526	H	-1.3721	-2.1220	-3.4669
C	2.3943	-4.6982	-0.2562	H	-3.5494	-2.9340	-4.3666
H	4.3489	-6.7872	-0.8562	H	-5.6941	-1.9577	-3.5337
H	4.4825	-5.5124	-3.6353	N	-5.7250	0.0990	-1.6235
C	1.9982	-3.5240	0.3830	O	-5.9229	1.9671	-0.3068
C	2.8833	-2.4424	0.3280	C	-3.4150	0.5834	0.8739
H	1.7223	-5.5598	-0.2130	C	-4.1041	0.8798	2.1077
C	4.9650	-1.2996	-0.3495	C	-2.0815	0.1756	1.2949
C	6.2508	-1.2885	0.2130	C	-5.5137	1.2701	2.3981
C	4.4203	-0.0939	-0.8158	H	-6.2326	0.5718	1.9447
C	6.8891	-2.4630	0.9002	H	-5.6470	1.2551	3.4878
C	6.9813	-0.0885	0.2433	H	-5.7598	2.2639	2.0036
C	5.0944	1.1297	-0.7183	O	-1.1086	-0.1767	0.6154
C	8.3539	-2.6813	0.4868	N	-3.3137	0.6928	3.1356
H	6.3015	-3.3811	0.7692	N	-2.0939	0.3147	2.6708
C	8.3727	-0.1533	0.8046	C	-1.0574	0.0967	3.5951
C	6.4031	1.0889	-0.2169	C	-1.3825	-0.1576	4.9328
C	9.1101	-1.3651	0.2313	C	0.2806	0.1530	3.2014
H	8.8511	-3.2647	1.2771	C	-0.3684	-0.3882	5.8570
H	8.9186	0.7774	0.5895	H	-2.4326	-0.1641	5.2239
H	6.9732	2.0203	-0.1670	C	1.2841	-0.0888	4.1341
H	10.1292	-1.4192	0.6440	H	0.5350	0.3927	2.1734
H	3.0214	-6.5974	-1.9934	C	0.9705	-0.3671	5.4631
H	5.3027	-6.9744	-3.0723	H	-0.6300	-0.5901	6.8988
H	6.6031	-5.5702	-1.4199	H	2.3244	-0.0560	3.8016
H	5.3544	-3.1887	-2.8928	H	1.7625	-0.5570	6.1914
H	6.8626	-2.2451	1.9833	C	-9.0241	-3.5959	-0.6322

H	8.3918	-3.3083	-0.4178	C	-8.1121	-2.9250	0.1823
H	9.2233	-1.2051	-0.8528	C	-9.3059	-3.1011	-1.9039
H	8.3245	-0.2400	1.9063	C	-7.4879	-1.7666	-0.2730
C	0.6701	-3.4106	1.0303	H	-7.8810	-3.3093	1.1788
C	-0.4800	-3.5369	0.2757	C	-8.6762	-1.9433	-2.3592
C	0.5621	-3.1573	2.4217	H	-10.0146	-3.6236	-2.5507
C	-1.7621	-3.3705	0.8542	C	-7.7642	-1.2641	-1.5484
H	-0.4050	-3.7138	-0.8003	H	-6.7624	-1.2525	0.3628
C	-0.6674	-3.0254	3.0173	H	-8.8940	-1.5641	-3.3621
H	1.4715	-3.0562	3.0167	C	-7.1001	0.0038	-2.0307
C	-2.9490	-3.4212	0.0790	H	-7.1733	0.0794	-3.1285
C	-1.8601	-3.0999	2.2536	H	-7.5982	0.8896	-1.6045
H	-0.7346	-2.8181	4.0875	H	-9.5111	-4.5069	-0.2769
C	-4.1756	-3.1911	0.6555	C	0.7355	3.5932	4.8504
H	-2.8734	-3.6204	-0.9923	C	-0.4403	3.5882	5.5971
C	-3.1402	-2.8701	2.8204	C	-1.6775	3.5211	4.9568
C	-4.2713	-2.9062	2.0380	C	-1.7225	3.4443	3.5755
H	-5.0781	-3.2194	0.0410	C	-0.5414	3.4259	2.8047
H	-3.2084	-2.6395	3.8863	C	0.7193	3.5157	3.4577
H	-5.2491	-2.7085	2.4851	H	1.6989	3.6424	5.3637
C	4.4455	2.4296	-1.0002	H	-0.3890	3.6305	6.6877
C	3.6491	2.6578	-2.1057	H	-2.6035	3.5029	5.5341
C	4.6278	3.4827	-0.0596	H	-2.6920	3.3541	3.0857
C	3.0144	3.9087	-2.3071	C	2.0081	3.5022	2.6940
H	3.4786	1.8651	-2.8352	H	2.1207	2.5853	2.0943
C	4.0152	4.6995	-0.2219	H	2.0734	4.3451	1.9884
H	5.2385	3.2980	0.8270	H	2.8635	3.5727	3.3791
C	2.1986	4.1567	-3.4416	C	-0.5871	3.2146	1.3780
C	3.1820	4.9481	-1.3413	C	-1.7161	3.0877	0.6214
H	4.1527	5.4885	0.5223	H	0.3804	3.0866	0.8871
C	1.5513	5.3606	-3.5948	C	-1.7707	2.6608	-0.7399
H	2.0746	3.3614	-4.1780	H	-2.6939	3.2513	1.0767
C	2.5068	6.1811	-1.5296	C	-3.0513	2.4878	-1.2864
C	1.7039	6.3821	-2.6280	H	-3.1874	2.2423	-2.3367
H	0.9133	5.5284	-4.4654	H	-3.8346	3.1081	-0.8461
H	2.6342	6.9700	-0.7835	N	-0.5939	2.2495	-1.3428
H	1.1836	7.3338	-2.7596	H	0.1503	1.9183	-0.7081
O	2.5015	-1.2194	0.8699	C	-0.2348	2.0594	-2.6491
O	3.1260	-0.1348	-1.3144	C	-1.0733	2.6969	-3.7836
P	1.9411	-0.1471	-0.2211	O	0.7534	1.4419	-2.9601
O	1.6264	1.1687	0.3933	F	-0.3106	2.8673	-4.8500
O	0.7628	-0.8652	-0.9708	F	-1.5814	3.8800	-3.4443
H	-0.0488	-0.7663	-0.3844	F	-2.0812	1.8917	-4.1382
C	-4.6721	-0.6567	-2.1402				

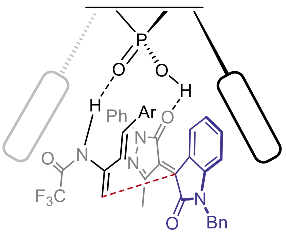

C4-TS1':

C	7.7726	2.2993	-2.4878	C	-2.9833	0.5217	-0.8696
C	6.8901	1.7528	-1.3527	C	-1.6747	0.2042	-1.2009
C	5.4243	1.8078	-1.6699	C	-1.4153	-0.5131	-2.3749
C	4.9432	3.0310	-2.1801	C	-2.4512	-0.8769	-3.2303
C	5.9623	4.1016	-2.4426	C	-3.7736	-0.5287	-2.9395
C	7.1532	3.5132	-3.2070	C	-5.1077	1.1772	-0.0112
H	8.7550	2.5621	-2.0596	C	-3.6098	1.2019	0.2939
H	7.2076	0.7386	-1.0641	H	-0.8581	0.4644	-0.5385
C	4.5256	0.7640	-1.4032	H	-0.3849	-0.7922	-2.6096
C	3.5854	3.2058	-2.4522	H	-2.2356	-1.4472	-4.1378
H	5.5117	4.9353	-3.0066	H	-4.5895	-0.8205	-3.6023
H	7.9140	4.2942	-3.3740	N	-5.2455	0.5658	-1.2389
C	2.6626	2.1973	-2.1683	O	-6.0290	1.5702	0.6657
C	3.1639	1.0198	-1.6163	C	-3.0580	2.4421	0.8364
H	3.2301	4.1407	-2.8939	C	-3.6409	3.7443	0.9212
C	4.9047	-0.5796	-0.8896	C	-1.7727	2.6069	1.3442
C	5.7289	-1.4554	-1.6078	C	-4.9575	4.2648	0.4569
C	4.3149	-1.0399	0.2994	H	-5.7844	3.9173	1.0953
C	6.3298	-1.1486	-2.9483	H	-4.9248	5.3661	0.4712
C	5.9562	-2.7525	-1.1132	H	-5.1797	3.9347	-0.5708
C	4.4641	-2.3480	0.7765	O	-0.9572	1.6055	1.5541
C	7.8143	-1.5437	-3.0375	N	-2.7795	4.6117	1.4392
H	6.1902	-0.0935	-3.2292	N	-1.6394	3.9257	1.7120
C	6.8788	-3.6310	-1.9076	C	-0.5888	4.6199	2.3499
C	5.3293	-3.1799	0.0513	C	-0.9067	5.7671	3.0907
C	8.1407	-2.8487	-2.2857	C	0.7416	4.2134	2.2403
H	8.0802	-1.6347	-4.1045	C	0.1031	6.4904	3.7157
H	7.1368	-4.5387	-1.3367	H	-1.9506	6.0761	3.1577
H	5.5064	-4.1984	0.4087	C	1.7430	4.9397	2.8831
H	8.8111	-3.4845	-2.8882	H	1.0200	3.3426	1.6556
H	6.3118	4.5245	-1.4797	C	1.4344	6.0808	3.6203
H	6.7921	3.2106	-4.2057	H	-0.1563	7.3840	4.2907
H	7.9642	1.4983	-3.2217	H	2.7792	4.6020	2.7903
H	7.0475	2.3823	-0.4541	H	2.2239	6.6489	4.1189
H	5.7646	-1.7360	-3.6989	C	-7.5206	-3.8433	-2.1168
H	8.4358	-0.7250	-2.6365	C	-7.0811	-3.2884	-0.9137
H	8.6843	-2.6169	-1.3529	C	-7.6473	-3.0329	-3.2439
H	6.3671	-3.9728	-2.8293	C	-6.7605	-1.9346	-0.8452
C	1.2089	2.2804	-2.4364	H	-6.9899	-3.9143	-0.0219
C	0.4102	3.3134	-1.9956	C	-7.3248	-1.6776	-3.1712
C	0.6251	1.2425	-3.2186	H	-7.9941	-3.4583	-4.1896
C	-0.9864	3.3283	-2.2663	C	-6.8719	-1.1175	-1.9750
H	0.8390	4.1223	-1.3992	H	-6.4333	-1.5005	0.1043
C	-0.7099	1.2526	-3.5299	H	-7.4148	-1.0493	-4.0629
H	1.2652	0.4352	-3.5848	C	-6.5135	0.3487	-1.8925
C	-1.8350	4.3510	-1.7697	H	-6.4833	0.7941	-2.9040
C	-1.5639	2.2733	-3.0446	H	-7.2664	0.9022	-1.3026
H	-1.1393	0.4577	-4.1458	H	-7.7696	-4.9063	-2.1729
C	-3.1870	4.3256	-2.0235	C	-1.1986	-5.4262	-2.7184
H	-1.4026	5.1514	-1.1636	C	-2.4306	-5.6105	-3.3425
C	-2.9589	2.2758	-3.2901	C	-3.5573	-4.9294	-2.8781

C	-3.7540	3.2799	-2.7886	C	-3.4373	-4.0744	-1.7918
H	-3.8310	5.1087	-1.6155	C	-2.2020	-3.8851	-1.1482
H	-3.3920	1.4575	-3.8723	C	-1.0545	-4.5694	-1.6233
H	-4.8316	3.2709	-2.9740	H	-0.3198	-5.9580	-3.0949
C	3.6683	-2.8870	1.8984	H	-2.5102	-6.2872	-4.1979
C	3.4177	-2.1907	3.0652	H	-4.5272	-5.0611	-3.3640
C	3.0883	-4.1788	1.7397	H	-4.3208	-3.5369	-1.4371
C	2.5975	-2.7316	4.0876	C	0.2979	-4.3806	-0.9963
H	3.8505	-1.2008	3.2216	H	0.6224	-3.3239	-1.0217
C	2.2862	-4.7247	2.7091	H	0.3139	-4.6887	0.0637
H	3.2568	-4.7247	0.8074	H	1.0591	-4.9749	-1.5251
C	2.3256	-2.0153	5.2821	C	-2.0690	-2.9633	-0.0370
C	2.0112	-4.0192	3.9087	C	-2.9906	-2.0799	0.4124
H	1.8375	-5.7115	2.5605	H	-1.0932	-2.9211	0.4613
C	1.5126	-2.5512	6.2526	C	-2.6503	-1.0897	1.4302
H	2.7571	-1.0186	5.4073	H	-3.9658	-1.9835	-0.0689
C	1.1719	-4.5449	4.9238	C	-3.6423	-0.0420	1.6584
C	0.9283	-3.8269	6.0717	H	-3.5560	0.5853	2.5150
H	1.3011	-1.9851	7.1633	H	-4.6321	-0.4396	1.5659
H	0.7226	-5.5318	4.7788	N	-1.5362	-1.2469	2.0661
H	0.2796	-4.2392	6.8495	H	0.6599	-1.1286	1.4816
O	2.2088	0.0941	-1.1890	C	-0.9274	-0.6835	3.1218
O	3.4947	-0.1466	0.9666	C	-1.7304	-0.2847	4.3859
P	2.0179	0.1354	0.4167	O	0.2844	-0.5622	3.1702
O	1.1174	-1.1456	0.5899	F	-1.0005	-0.4249	5.4791
O	1.5323	1.3987	1.0423	F	-2.8351	-1.0167	4.5296
H	0.0344	1.5324	1.3823	F	-2.1006	0.9961	4.3100
C	-4.0120	0.1633	-1.7581				

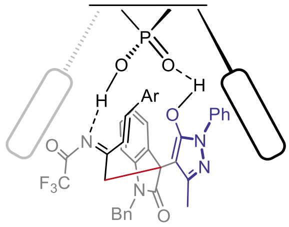

C4-Int1:

C	7.2183	-3.2712	-2.7664	C	-3.6435	1.1012	-0.8669
C	6.2797	-2.0542	-2.7954	C	-2.5573	1.2353	-1.7196
C	4.8680	-2.3875	-2.4048	C	-2.7445	1.1152	-3.1044
C	4.3136	-3.5236	-3.0228	C	-4.0096	0.8711	-3.6275
C	5.2059	-4.3075	-3.9401	C	-5.1268	0.7782	-2.7879
C	6.5543	-4.5678	-3.2661	C	-5.3186	1.3139	0.7846
H	8.1063	-3.0331	-3.3719	C	-3.7871	1.1328	0.6482
H	6.6834	-1.2310	-2.1909	H	-1.5486	1.4453	-1.3673
C	4.0783	-1.6100	-1.5396	H	-1.8734	1.2101	-3.7544
C	2.9984	-3.8935	-2.7561	H	-4.1436	0.7672	-4.7066
H	4.7236	-5.2514	-4.2339	H	-6.1257	0.6140	-3.1944
H	7.2228	-5.1033	-3.9574	N	-5.8883	0.9884	-0.4190
C	2.1685	-3.1065	-1.9533	O	-5.9157	1.6886	1.7664
C	2.7245	-1.9556	-1.3883	C	-3.4023	-0.2023	1.2738

H	2.5851	-4.7969	-3.2128	C	-3.9541	-0.9214	2.3854
C	4.6063	-0.4542	-0.7627	C	-2.2122	-0.8812	1.0123
C	5.6486	-0.5910	0.1721	C	-5.2172	-0.7425	3.1599
C	3.9784	0.7968	-0.8864	H	-6.1100	-0.8311	2.5246
C	6.3149	-1.8901	0.5266	H	-5.2556	-1.5253	3.9286
C	6.0671	0.5285	0.9114	H	-5.2761	0.2432	3.6400
C	4.3767	1.9277	-0.1600	O	-1.2402	-0.6593	0.1393
C	7.8447	-1.7726	0.6004	N	-3.1521	-1.9087	2.7528
H	6.0169	-2.6996	-0.1514	N	-2.0922	-1.8821	1.9223
C	7.1817	0.3220	1.8950	C	-1.0110	-2.7691	2.1463
C	5.4544	1.7606	0.7204	C	-1.2759	-3.9928	2.7658
C	8.3255	-0.4600	1.2480	C	0.2976	-2.4157	1.8148
H	8.2313	-2.6428	1.1526	C	-0.2251	-4.8591	3.0515
H	7.5373	1.2880	2.2833	H	-2.3060	-4.2404	3.0218
H	5.8175	2.6245	1.2815	C	1.3377	-3.2945	2.0996
H	9.1104	-0.6593	1.9934	H	0.5103	-1.4649	1.3383
H	5.3679	-3.7351	-4.8720	C	1.0864	-4.5152	2.7212
H	6.3794	-5.2469	-2.4166	H	-0.4360	-5.8152	3.5371
H	7.5865	-3.4245	-1.7412	H	2.3563	-3.0044	1.8318
H	6.2410	-1.6718	-3.8314	H	1.9080	-5.1980	2.9501
H	5.9379	-2.1836	1.5235	C	-8.8711	-2.8500	-1.8812
H	8.2608	-1.8499	-0.4151	C	-7.8857	-2.7819	-0.8958
H	8.7823	0.1859	0.4820	C	-9.3353	-1.6773	-2.4731
H	6.7984	-0.2422	2.7660	C	-7.3663	-1.5497	-0.5052
C	0.7710	-3.5090	-1.6483	H	-7.5216	-3.6988	-0.4266
C	-0.3108	-2.7732	-2.0814	C	-8.8181	-0.4440	-2.0781
C	0.5541	-4.6690	-0.8565	H	-10.1020	-1.7209	-3.2502
C	-1.6306	-3.1231	-1.6998	C	-7.8340	-0.3675	-1.0896
H	-0.1545	-1.8746	-2.6817	H	-6.5822	-1.5048	0.2554
C	-0.7114	-5.0243	-0.4613	H	-9.1827	0.4744	-2.5473
H	1.4162	-5.2525	-0.5265	C	-7.3111	0.9763	-0.6410
C	-2.7543	-2.3515	-2.0971	H	-7.5785	1.7514	-1.3783
C	-1.8335	-4.2466	-0.8426	H	-7.7654	1.2701	0.3185
H	-0.8639	-5.8926	0.1837	H	-9.2735	-3.8179	-2.1888
C	-4.0123	-2.6414	-1.6205	C	2.5441	-0.9612	4.8669
H	-2.6043	-1.5063	-2.7729	C	1.7014	-1.8290	5.5570
C	-3.1431	-4.5264	-0.3753	C	0.3161	-1.6947	5.4509
C	-4.2052	-3.7345	-0.7432	C	-0.2085	-0.6983	4.6452
H	-4.8646	-2.0240	-1.9143	C	0.6300	0.1875	3.9390
H	-3.2894	-5.3738	0.2995	C	2.0394	0.0586	4.0559
H	-5.2044	-3.9501	-0.3602	H	3.6273	-1.0790	4.9530
C	3.6992	3.2393	-0.2573	H	2.1287	-2.6215	6.1760
C	3.2004	3.7392	-1.4473	H	-0.3508	-2.3821	5.9743
C	3.5845	4.0447	0.9131	H	-1.2916	-0.6281	4.5297
C	2.6291	5.0326	-1.5266	C	2.9821	0.9524	3.3064
H	3.2546	3.1407	-2.3573	H	2.8588	0.8443	2.2189
C	3.0397	5.3036	0.8658	H	2.8160	2.0140	3.5451
H	3.9247	3.6435	1.8697	H	4.0250	0.7130	3.5532
C	2.1211	5.5500	-2.7465	C	0.0850	1.1553	3.0123
C	2.5655	5.8468	-0.3550	C	-1.2220	1.3392	2.6867
H	2.9623	5.9027	1.7770	H	0.8113	1.7646	2.4727
C	1.5918	6.8176	-2.8055	C	-1.6373	2.1799	1.5918

H	2.1482	4.9164	-3.6365	H	-2.0156	0.8161	3.2208
C	2.0296	7.1553	-0.4500	C	-3.1183	2.3334	1.3729
C	1.5512	7.6308	-1.6484	H	-3.3439	3.2413	0.7999
H	1.1961	7.2012	-3.7488	H	-3.6158	2.4158	2.3510
H	1.9872	7.7737	0.4504	N	-0.7461	2.6880	0.7816
H	1.1340	8.6387	-1.7109	H	0.5312	1.7047	0.3783
O	1.9326	-1.1544	-0.5884	C	-0.8834	3.7098	-0.1151
O	2.8773	0.8766	-1.7197	C	-1.5046	5.0371	0.4024
P	1.4771	0.3160	-1.1090	O	-0.4559	3.7017	-1.2410
O	1.2778	1.0247	0.2798	F	-0.7400	6.0635	0.0705
O	0.3957	0.3780	-2.1117	F	-1.6753	5.0506	1.7259
H	-1.5929	-0.3620	-0.7161	F	-2.7024	5.2144	-0.1654
C	-4.9215	0.9179	-1.4229				

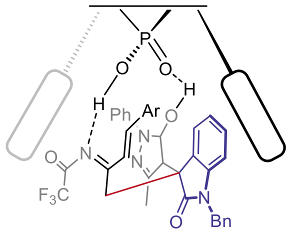

C4-Int1':

C	-8.3005	-1.4948	-1.8817	C	2.9984	-0.6547	-0.9448
C	-7.2613	-0.9841	-0.8690	C	1.7080	-0.2534	-1.2521
C	-5.8512	-1.3497	-1.2364	C	1.4772	0.4615	-2.4324
C	-5.6344	-2.6923	-1.5941	C	2.5241	0.7373	-3.3097
C	-6.8435	-3.5801	-1.6677	C	3.8259	0.3021	-3.0355
C	-7.9568	-2.8834	-2.4555	C	5.0731	-1.4442	-0.0920
H	-9.2832	-1.5141	-1.3810	C	3.5906	-1.2668	0.3124
H	-7.3725	0.0986	-0.7073	H	0.8937	-0.4527	-0.5588
C	-4.7611	-0.4680	-1.1526	H	0.4655	0.8102	-2.6557
C	-4.3496	-3.1416	-1.8911	H	2.3338	1.3079	-4.2235
H	-6.5850	-4.5466	-2.1305	H	4.6488	0.5270	-3.7168
H	-8.8536	-3.5248	-2.4838	N	5.2409	-0.8691	-1.3304
C	-3.2379	-2.2988	-1.7873	O	5.9697	-1.8870	0.5886
C	-3.4767	-0.9831	-1.3865	C	2.9411	-2.5433	0.7857
H	-4.1958	-4.1762	-2.2124	C	3.4413	-3.8761	0.8913
C	-4.8879	0.9728	-0.8035	C	1.6507	-2.6165	1.2978
C	-5.6044	1.8832	-1.5955	C	4.7189	-4.4944	0.4303
C	-4.1665	1.4631	0.2953	H	5.5722	-4.1968	1.0582
C	-6.3213	1.5277	-2.8664	H	4.6051	-5.5897	0.4659
C	-5.5967	3.2479	-1.2565	H	4.9575	-4.1992	-0.6050
C	-4.0857	2.8252	0.6171	O	0.8830	-1.5682	1.4930
C	-7.7268	2.1478	-2.9525	N	2.5372	-4.6746	1.4276
H	-6.3654	0.4396	-3.0208	N	1.4473	-3.9177	1.6898
C	-6.4118	4.1681	-2.1197	C	0.3660	-4.5510	2.3379
C	-4.8479	3.6967	-0.1745	C	0.6342	-5.7006	3.0933
C	-7.8000	3.5666	-2.3553	C	-0.9457	-4.0915	2.2236
H	-8.0312	2.1572	-4.0127	C	-0.4050	-6.3727	3.7281
H	-6.4904	5.1647	-1.6551	H	1.6644	-6.0521	3.1626
H	-4.8447	4.7647	0.0634	C	-1.9776	-4.7655	2.8757

H	-8.3919	4.2310	-3.0070	H	-1.1871	-3.2192	1.6253
H	-7.2003	-3.8055	-0.6434	C	-1.7184	-5.9090	3.6282
H	-7.6130	-2.7815	-3.4992	H	-0.1822	-7.2695	4.3143
H	-8.3935	-0.7705	-2.7081	H	-2.9986	-4.3845	2.7767
H	-7.4688	-1.4559	0.1109	H	-2.5316	-6.4372	4.1342
H	-5.7129	1.9250	-3.7017	C	7.9147	3.3123	-2.2571
H	-8.4476	1.4941	-2.4334	C	7.4341	2.8077	-1.0471
H	-8.3213	3.5366	-1.3830	C	7.9523	2.4875	-3.3807
H	-5.9035	4.3127	-3.0936	C	6.9843	1.4914	-0.9690
C	-1.8684	-2.7600	-2.1047	H	7.4118	3.4436	-0.1572
C	-1.3403	-3.8999	-1.5331	C	7.5016	1.1692	-3.2973
C	-1.0882	-2.0344	-3.0479	H	8.3307	2.8730	-4.3322
C	-0.0217	-4.3319	-1.8290	C	7.0069	0.6601	-2.0946
H	-1.9294	-4.4730	-0.8119	H	6.6262	1.0951	-0.0135
C	0.1759	-2.4503	-3.3824	H	7.5237	0.5299	-4.1864
H	-1.5159	-1.1443	-3.5176	C	6.5155	-0.7662	-1.9932
C	0.5554	-5.4676	-1.2058	H	6.4399	-1.2194	-2.9969
C	0.7576	-3.5890	-2.7681	H	7.2189	-1.3746	-1.4006
H	0.7617	-1.8954	-4.1200	H	8.2654	4.3466	-2.3209
C	1.8476	-5.8435	-1.4916	C	2.0309	5.5467	-2.8956
H	-0.0346	-6.0300	-0.4763	C	3.2718	5.5228	-3.5295
C	2.0864	-3.9990	-3.0413	C	4.2726	4.6633	-3.0713
C	2.6209	-5.1027	-2.4153	C	4.0182	3.8412	-1.9836
H	2.2852	-6.7078	-0.9857	C	2.7722	3.8579	-1.3300
H	2.6825	-3.4135	-3.7483	C	1.7518	4.7267	-1.7979
H	3.6505	-5.4079	-2.6250	H	1.2505	6.2192	-3.2656
C	-3.1630	3.3598	1.6445	H	3.4569	6.1775	-4.3865
C	-2.9745	2.7727	2.8830	H	5.2494	4.6308	-3.5623
C	-2.3965	4.5164	1.3205	H	4.8033	3.1647	-1.6348
C	-2.0438	3.2922	3.8170	C	0.3914	4.7757	-1.1574
H	-3.5483	1.8886	3.1664	H	-0.1116	3.7930	-1.1778
C	-1.4821	5.0379	2.2016	H	0.4409	5.0843	-0.0981
H	-2.5133	4.9716	0.3330	H	-0.2573	5.4950	-1.6828
C	-1.8417	2.6868	5.0841	C	2.5020	2.9703	-0.2146
C	-1.2728	4.4440	3.4726	C	3.2928	1.9756	0.2522
H	-0.8929	5.9179	1.9247	H	1.5312	3.0660	0.2802
C	-0.9211	3.1982	5.9686	C	2.8339	1.0499	1.2835
H	-2.4167	1.7916	5.3372	H	4.2537	1.7530	-0.2186
C	-0.3264	4.9457	4.4020	C	3.7072	-0.1636	1.4651
C	-0.1546	4.3366	5.6245	H	3.5704	-0.6672	2.4280
H	-0.7670	2.7153	6.9375	H	4.7345	0.2319	1.4637
H	0.2641	5.8269	4.1313	N	1.7345	1.3232	1.8952
H	0.5781	4.7307	6.3352	H	-0.5311	1.2254	1.2905
O	-2.3770	-0.1540	-1.1314	C	1.0799	0.8598	2.9637
O	-3.4440	0.5402	1.0390	C	1.8542	0.5084	4.2572
P	-2.0581	-0.0081	0.4462	O	-0.1376	0.8069	3.0038
O	-1.0262	1.1836	0.4272	F	1.1249	0.7601	5.3295
O	-1.6360	-1.2470	1.1670	F	2.9939	1.1952	4.3513
H	-0.1152	-1.4409	1.3638	F	2.1602	-0.7927	4.2662
C	4.0363	-0.3870	-1.8463				

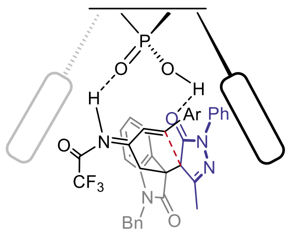

C4-TS2-[4+2]:

C	8.2765	-2.8703	-1.2023	C	-2.7913	0.5530	-1.6187
C	7.1940	-1.7928	-1.3904	C	-1.5492	0.6216	-2.2244
C	5.8137	-2.2927	-1.0756	C	-1.3197	-0.1206	-3.3898
C	5.4662	-3.5264	-1.6477	C	-2.3371	-0.9009	-3.9396
C	6.5174	-4.2409	-2.4432	C	-3.6220	-0.9157	-3.3840
C	7.8328	-4.2738	-1.6579	C	-4.8623	1.1923	-0.7089
H	9.1772	-2.5514	-1.7548	C	-3.3432	1.2701	-0.4048
H	7.4463	-0.8878	-0.8148	H	-0.7750	1.2765	-1.8143
C	4.8646	-1.5789	-0.3322	H	-0.3386	-0.0806	-3.8679
C	4.1931	-4.0411	-1.4578	H	-2.1405	-1.4910	-4.8383
H	6.1883	-5.2631	-2.6974	H	-4.4295	-1.4833	-3.8474
H	8.6202	-4.7510	-2.2655	N	-5.0501	0.1549	-1.6090
C	3.1967	-3.3334	-0.7712	O	-5.7312	1.8975	-0.2613
C	3.5464	-2.0823	-0.2372	C	-3.1267	0.4352	0.8660
H	3.9350	-5.0094	-1.8955	C	-4.2291	-0.2641	1.6085
C	5.2096	-0.2937	0.3188	C	-2.0656	-0.6669	0.7827
C	6.2063	-0.1472	1.2937	C	-5.5965	0.1951	1.9775
C	4.4592	0.8367	-0.0290	H	-6.3137	0.0535	1.1527
C	7.0080	-1.2811	1.8614	H	-5.9502	-0.3994	2.8351
C	6.4461	1.1198	1.8561	H	-5.6167	1.2627	2.2398
C	4.6777	2.1078	0.5139	O	-1.0007	-0.6521	0.2178
C	8.5077	-0.9510	1.9585	N	-3.8770	-1.4642	1.9425
H	6.8532	-2.2154	1.2980	N	-2.5955	-1.7179	1.5065
C	7.5225	1.2096	2.8975	C	-1.9357	-2.8574	2.0234
C	5.7086	2.2282	1.4498	C	-2.7046	-3.9499	2.4368
C	8.7885	0.4951	2.4117	C	-0.5484	-2.8563	2.2073
H	8.9746	-1.6722	2.6514	C	-2.0844	-5.0342	3.0498
H	7.7393	2.2626	3.1455	H	-3.7854	-3.9299	2.2919
H	5.9166	3.2082	1.8858	C	0.0551	-3.9496	2.8209
H	9.5530	0.5101	3.2068	H	0.0589	-2.0165	1.8719
H	6.6744	-3.7140	-3.4058	C	-0.7052	-5.0373	3.2502
H	7.6848	-4.9221	-0.7756	H	-2.6888	-5.8849	3.3737
H	8.5700	-2.9101	-0.1393	H	1.1389	-3.9446	2.9629
H	7.1900	-1.4779	-2.4540	H	-0.2215	-5.8893	3.7323
H	6.6221	-1.4792	2.8823	C	-7.0725	-4.4897	-1.5703
H	8.9814	-1.1222	0.9766	C	-6.4075	-3.7684	-0.5792
H	9.2045	1.0758	1.5693	C	-7.4844	-3.8425	-2.7348
H	7.1687	0.7332	3.8340	C	-6.1580	-2.4086	-0.7510
C	1.8756	-3.9848	-0.6464	H	-6.0690	-4.2660	0.3331
C	0.7048	-3.3763	-1.0413	C	-7.2367	-2.4809	-2.9033
C	1.8206	-5.3271	-0.1748	H	-7.9976	-4.4017	-3.5207
C	-0.5422	-4.0443	-0.9673	C	-6.5769	-1.7514	-1.9106
H	0.7288	-2.3698	-1.4536	H	-5.6142	-1.8631	0.0234
C	0.6279	-6.0019	-0.0974	H	-7.5580	-1.9808	-3.8213

H	2.7454	-5.8137	0.1446	C	-6.3444	-0.2703	-2.0941
C	-1.7493	-3.4090	-1.3601	H	-6.4497	0.0019	-3.1639
C	-0.5885	-5.3813	-0.4843	H	-7.0992	0.3191	-1.5362
H	0.5997	-7.0280	0.2770	H	-7.2647	-5.5570	-1.4386
C	-2.9501	-4.0696	-1.2678	C	-4.2411	1.9562	5.3053
H	-1.7107	-2.3807	-1.7277	C	-4.8825	3.1597	5.0306
C	-1.8423	-6.0402	-0.4013	C	-4.6876	3.7744	3.7961
C	-2.9975	-5.3985	-0.7828	C	-3.8653	3.1679	2.8526
H	-3.8763	-3.5696	-1.5624	C	-3.2430	1.9404	3.1026
H	-1.8741	-7.0647	-0.0229	C	-3.4139	1.3306	4.3641
H	-3.9613	-5.9082	-0.7124	H	-4.3773	1.4854	6.2826
C	3.8218	3.2444	0.1334	H	-5.5226	3.6232	5.7850
C	3.4915	3.5240	-1.1762	H	-5.1700	4.7270	3.5667
C	3.3430	4.1007	1.1643	H	-3.7065	3.6674	1.8959
C	2.6973	4.6509	-1.5076	C	-2.7473	0.0397	4.7464
H	3.8369	2.8700	-1.9811	H	-3.1001	-0.8064	4.1338
C	2.5840	5.2045	0.8703	H	-1.6499	0.0882	4.6416
H	3.5738	3.8546	2.2035	H	-2.9582	-0.2127	5.7971
C	2.3264	4.9343	-2.8477	C	-2.2961	1.3670	2.1262
C	2.2442	5.5156	-0.4707	C	-1.3501	2.2776	1.4683
H	2.2144	5.8491	1.6718	H	-1.7323	0.5548	2.5840
C	1.5387	6.0216	-3.1404	C	-1.5543	2.8821	0.2922
H	2.6661	4.2634	-3.6404	H	-0.3877	2.3762	1.9777
C	1.4439	6.6373	-0.8034	C	-2.8907	2.7333	-0.3728
C	1.0952	6.8831	-2.1094	H	-2.8831	3.1187	-1.4048
H	1.2478	6.2265	-4.1732	H	-3.6650	3.3090	0.1663
H	1.0921	7.2906	-0.0017	N	-0.4640	3.4966	-0.3613
H	0.4665	7.7412	-2.3583	H	0.4311	3.0186	-0.2495
O	2.5862	-1.3440	0.4469	C	-0.4521	4.4615	-1.3265
O	3.3900	0.6484	-0.8966	C	-1.6059	5.4971	-1.3453
P	2.0850	0.0844	-0.1384	O	0.4519	4.6122	-2.1144
O	1.5083	0.9434	0.8990	F	-1.1154	6.7162	-1.5043
O	1.2115	-0.2102	-1.4352	F	-2.3385	5.5199	-0.2319
H	0.2698	-0.3786	-1.1619	F	-2.4356	5.2713	-2.3650
C	-3.8332	-0.1568	-2.2397				

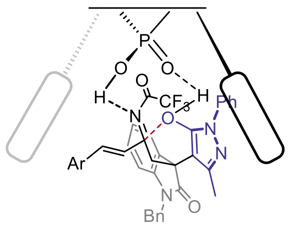

C4-TS2-[2+4]:

C	8.0998	-1.6474	-2.8822	C	-3.6024	-0.1469	-1.1059
C	6.8588	-0.8013	-2.5583	C	-2.5529	-0.7598	-1.7725
C	5.7897	-1.6014	-1.8705	C	-2.6355	-0.9196	-3.1602
C	5.4351	-2.8046	-2.5086	C	-3.7695	-0.4926	-3.8539
C	6.2475	-3.2032	-3.7074	C	-4.8553	0.0846	-3.1854
C	7.7448	-3.0494	-3.4210	C	-5.2400	0.7362	0.3776
H	8.7173	-1.0936	-3.6094	C	-3.7835	0.2230	0.3442

H	7.1343	0.0963	-1.9869	H	-1.6634	-1.0962	-1.2346
C	5.0914	-1.1873	-0.7223	H	-1.8032	-1.3806	-3.6983
C	4.3497	-3.5483	-2.0522	H	-3.8210	-0.6219	-4.9389
H	6.0123	-4.2383	-4.0052	H	-5.7574	0.3798	-3.7254
H	8.3192	-3.2709	-4.3361	N	-5.6969	0.7626	-0.9179
C	3.5928	-3.1277	-0.9537	O	-5.8525	1.1067	1.3521
C	4.0225	-1.9793	-0.2824	C	-3.4982	-0.8537	1.3480
H	4.0535	-4.4606	-2.5790	C	-4.2672	-1.7272	2.1602
C	5.3124	0.1273	-0.0619	C	-2.2017	-1.0513	1.7670
C	6.5315	0.5161	0.5171	C	-5.7334	-1.9919	2.1277
C	4.2273	1.0222	0.0152	H	-5.9832	-2.7318	1.3457
C	7.7410	-0.3688	0.6248	H	-6.0662	-2.3958	3.0963
C	6.6540	1.7868	1.1059	H	-6.2984	-1.0717	1.9150
C	4.3300	2.2942	0.5881	O	-1.1528	-0.3915	1.2607
C	9.0233	0.3599	0.1968	N	-3.4813	-2.3807	3.0020
H	7.6137	-1.3013	0.0555	N	-2.2200	-1.9603	2.7867
C	7.9761	2.1144	1.7370	C	-1.1616	-2.5342	3.5234
C	5.5715	2.6619	1.1162	C	-1.4018	-3.6778	4.2943
C	9.1405	1.7774	0.7986	C	0.1064	-1.9567	3.5074
H	9.8916	-0.2603	0.4757	C	-0.3549	-4.2567	5.0070
H	8.0139	3.1737	2.0394	H	-2.4103	-4.0910	4.3282
H	5.6773	3.6500	1.5746	C	1.1488	-2.5545	4.2117
H	10.0898	1.8931	1.3479	H	0.3074	-1.0467	2.9478
H	5.9735	-2.5599	-4.5669	C	0.9298	-3.7102	4.9594
H	8.0291	-3.8175	-2.6818	H	-0.5489	-5.1514	5.6062
H	8.7152	-1.7469	-1.9723	H	2.1382	-2.0910	4.1674
H	6.4299	-0.4325	-3.5103	H	1.7496	-4.1763	5.5135
H	7.8410	-0.6736	1.6846	C	-9.5284	-1.7834	-3.0606
H	9.0377	0.4264	-0.9038	C	-8.6385	-2.1253	-2.0413
H	9.1582	2.5253	-0.0121	C	-9.5901	-0.4641	-3.5082
H	8.0798	1.5201	2.6657	C	-7.8179	-1.1531	-1.4736
C	2.2997	-3.7539	-0.5967	H	-8.5815	-3.1582	-1.6854
C	1.3528	-3.9065	-1.5916	C	-8.7643	0.5063	-2.9406
C	1.9817	-4.1611	0.7263	H	-10.2810	-0.1877	-4.3104
C	0.0715	-4.4437	-1.3229	C	-7.8750	0.1718	-1.9160
H	1.5821	-3.5782	-2.6101	H	-7.1204	-1.4275	-0.6766
C	0.7493	-4.6939	1.0181	H	-8.8103	1.5391	-3.3025
H	2.7277	-4.0502	1.5164	C	-7.0080	1.2339	-1.2819
C	-0.9193	-4.5676	-2.3332	H	-6.9155	2.1050	-1.9551
C	-0.2443	-4.8378	0.0133	H	-7.4624	1.5909	-0.3416
H	0.5170	-5.0084	2.0404	H	-10.1707	-2.5469	-3.5092
C	-2.1750	-5.0428	-2.0322	C	-1.4897	1.3848	1.1006
H	-0.6666	-4.2753	-3.3572	C	-2.9743	1.4937	0.7896
C	-1.5439	-5.3371	0.2884	H	-3.0498	2.2832	0.0305
C	-2.4890	-5.4316	-0.7080	H	-3.5380	1.8747	1.6487
H	-2.9363	-5.1177	-2.8143	C	-0.6829	1.8988	-0.0924
H	-1.7830	-5.6392	1.3120	C	-1.1603	2.0801	-1.3305
H	-3.4881	-5.8135	-0.4796	H	0.3559	2.1687	0.1454
C	3.1465	3.1826	0.7108	C	-0.4072	2.4445	-2.5324
C	2.5538	3.7277	-0.4118	H	-2.2172	1.8868	-1.5191
C	2.5926	3.4597	1.9879	C	0.9584	2.1570	-2.6471
C	1.3852	4.5250	-0.3208	C	-1.0770	3.0839	-3.6003

H	2.9720	3.5237	-1.4009	C	1.6753	2.5184	-3.7832
C	1.4698	4.2441	2.1106	H	1.4638	1.6321	-1.8364
H	3.0492	3.0109	2.8742	C	-0.3404	3.4426	-4.7322
C	0.7414	5.0512	-1.4704	C	-2.5440	3.3975	-3.5234
C	0.8211	4.7776	0.9679	C	1.0248	3.1720	-4.8290
H	1.0335	4.4194	3.0975	H	2.7422	2.2849	-3.8503
C	-0.4196	5.7792	-1.3494	H	-0.8497	3.9544	-5.5552
H	1.1695	4.8480	-2.4564	H	-3.1534	2.4761	-3.5760
C	-0.3782	5.5302	1.0597	H	-2.8042	3.9131	-2.5824
C	-0.9875	6.0170	-0.0745	H	-2.8515	4.0474	-4.3585
H	-0.9126	6.1690	-2.2446	H	1.5757	3.4638	-5.7283
H	-0.8079	5.7180	2.0488	N	-1.0351	2.1204	2.2307
H	-1.9123	6.5965	0.0067	H	-0.0338	2.3435	2.1687
O	3.2996	-1.5525	0.8169	C	-1.4846	2.4856	3.4703
O	2.9791	0.5835	-0.4311	C	-2.8562	2.0371	4.0248
P	2.2265	-0.3629	0.6538	O	-0.7999	3.1558	4.2070
O	1.8668	0.2925	1.9291	F	-2.8216	2.0861	5.3420
O	1.0413	-1.0294	-0.1589	F	-3.1749	0.7972	3.6678
H	0.1741	-0.9579	0.3107	F	-3.8186	2.8636	3.6027
C	-4.7496	0.2413	-1.8078				

C4-3aa:

C	7.8539	-3.7428	-0.9226	C	-2.8662	0.9316	-1.5507
C	6.9233	-2.5471	-1.1875	C	-1.6500	0.9109	-2.2166
C	5.4713	-2.8744	-0.9780	C	-1.5615	0.2612	-3.4547
C	5.0222	-4.0612	-1.5756	C	-2.6898	-0.3414	-4.0138
C	6.0391	-4.9019	-2.2909	C	-3.9403	-0.2595	-3.3894
C	7.2797	-5.0869	-1.4134	C	-4.8059	1.7140	-0.4865
H	8.8228	-3.5345	-1.4069	C	-3.2834	1.5930	-0.2525
H	7.2356	-1.6744	-0.5950	H	-0.7858	1.4328	-1.7949
C	4.5567	-2.0453	-0.3095	H	-0.6048	0.2340	-3.9823
C	3.6844	-4.4134	-1.4780	H	-2.6067	-0.8621	-4.9720
H	5.6076	-5.8773	-2.5698	H	-4.8324	-0.6833	-3.8555
H	8.0454	-5.6594	-1.9635	N	-5.1480	0.7965	-1.4562
C	2.7285	-3.5903	-0.8633	O	-5.5651	2.4705	0.0688
C	3.1891	-2.3848	-0.3056	C	-3.0560	0.6816	1.0174
H	3.3436	-5.3482	-1.9335	C	-4.2446	-0.0080	1.6744
C	5.0008	-0.8007	0.3685	C	-2.1685	-0.5419	0.7242
C	5.9139	-0.7933	1.4362	C	-5.5475	0.5447	2.1493
C	4.4472	0.4158	-0.0519	H	-6.3106	0.5306	1.3541
C	6.4960	-2.0246	2.0708	H	-5.9030	-0.0864	2.9783
C	6.2684	0.4285	2.0326	H	-5.4549	1.5859	2.4857
C	4.8044	1.6477	0.5065	O	-1.1204	-0.5857	0.1133
C	8.0085	-1.9050	2.3213	N	-3.9949	-1.2466	1.9053
H	6.2704	-2.9295	1.4874	N	-2.7640	-1.5776	1.3969
C	7.2391	0.3739	3.1771	C	-2.1997	-2.8053	1.8078
C	5.7361	1.6203	1.5518	C	-3.0567	-3.8426	2.1834
C	8.4371	-0.5086	2.8155	C	-0.8147	-2.9492	1.9371
H	8.2978	-2.6815	3.0495	C	-2.5272	-5.0194	2.7056
H	7.5705	1.3886	3.4524	H	-4.1346	-3.7062	2.0838
H	6.0451	2.5680	2.0033	C	-0.3011	-4.1322	2.4601

H	9.1115	-0.5932	3.6842
H	6.3291	-4.4036	-3.2368
H	6.9892	-5.7066	-0.5475
H	8.0619	-3.8089	0.1579
H	7.0350	-2.2453	-2.2470
H	5.9892	-2.1607	3.0459
H	8.5492	-2.1440	1.3907
H	9.0093	0.0075	2.0257
H	6.7313	-0.0448	4.0681
C	1.3351	-4.0948	-0.8357
C	0.2646	-3.3667	-1.3101
C	1.1031	-5.4222	-0.3756
C	-1.0479	-3.8971	-1.3286
H	0.4295	-2.3697	-1.7143
C	-0.1574	-5.9678	-0.3864
H	1.9459	-6.0052	0.0073
C	-2.1507	-3.1410	-1.8030
C	-1.2709	-5.2247	-0.8552
H	-0.3185	-6.9856	-0.0182
C	-3.4193	-3.6717	-1.8007
H	-1.9800	-2.1233	-2.1629
C	-2.5896	-5.7459	-0.8656
C	-3.6413	-4.9864	-1.3265
H	-4.2627	-3.0754	-2.1597
H	-2.7574	-6.7620	-0.4948
H	-4.6565	-5.3946	-1.3271
C	4.2564	2.9423	0.0489
C	4.2506	3.2945	-1.2877
C	3.8071	3.8891	1.0101
C	3.8561	4.5873	-1.7091
H	4.5873	2.5772	-2.0415
C	3.4044	5.1464	0.6293
H	3.7754	3.5989	2.0643
C	3.8750	4.9693	-3.0752
C	3.4336	5.5401	-0.7323
H	3.0546	5.8625	1.3797
C	3.4935	6.2349	-3.4563
H	4.1947	4.2361	-3.8223
C	3.0438	6.8351	-1.1555
C	3.0732	7.1767	-2.4874
H	3.5085	6.5167	-4.5134
H	2.7084	7.5567	-0.4042
H	2.7657	8.1779	-2.8037
O	2.3111	-1.5057	0.3234
O	3.4238	0.3511	-0.9994
P	2.0190	-0.0321	-0.2973
O	1.5189	0.9399	0.6963
O	1.1017	-0.2778	-1.5745
H	0.1628	-0.3769	-1.2832
C	-4.0066	0.4089	-2.1721
H	-0.1430	-2.1486	1.6304
C	-1.1493	-5.1685	2.8527
H	-3.2037	-5.8269	3.0012
H	0.7830	-4.2384	2.5600
H	-0.7352	-6.0934	3.2644
C	-7.7348	-3.5649	-1.7257
C	-6.9355	-3.0298	-0.7153
C	-8.1175	-2.7602	-2.7988
C	-6.5238	-1.7000	-0.7766
H	-6.6175	-3.6524	0.1262
C	-7.7065	-1.4284	-2.8557
H	-8.7364	-3.1722	-3.6014
C	-6.9110	-0.8829	-1.8432
H	-5.8768	-1.3052	0.0107
H	-8.0063	-0.8034	-3.7038
C	-6.4993	0.5706	-1.9018
H	-6.6196	0.9530	-2.9321
H	-7.1392	1.1900	-1.2502
H	-8.0544	-4.6103	-1.6808
C	-3.8707	1.9117	5.5849
C	-4.3572	3.2083	5.4497
C	-4.1229	3.9089	4.2683
C	-3.4181	3.2944	3.2390
C	-2.9543	1.9782	3.3485
C	-3.1627	1.2742	4.5569
H	-4.0360	1.3698	6.5216
H	-4.9066	3.6755	6.2725
H	-4.4828	4.9345	4.1469
H	-3.2242	3.8578	2.3254
C	-2.6602	-0.1267	4.7909
H	-3.1419	-0.8580	4.1191
H	-1.5690	-0.2122	4.6434
H	-2.8729	-0.4419	5.8247
C	-2.1881	1.3384	2.2040
C	-1.1027	2.1943	1.6326
H	-1.6711	0.4768	2.6466
C	-1.2727	2.8925	0.5066
H	-0.1115	2.1230	2.0848
C	-2.6421	2.9807	-0.1124
H	-2.6161	3.4414	-1.1097
H	-3.3071	3.6066	0.4995
N	-0.1334	3.3626	-0.1705
H	0.6809	2.7359	-0.1187
C	-0.0160	4.3347	-1.1103
C	-0.9719	5.5483	-1.0336
O	0.8436	4.3585	-1.9587
F	-0.2700	6.6600	-1.1997
F	-1.6185	5.6450	0.1307
F	-1.8853	5.4904	-2.0067

C4-4aa:

C	8.0074	-0.6908	-3.3152	C	-3.7958	0.3539	-1.0160
C	6.7790	-0.0050	-2.6971	C	-2.8319	0.0581	-1.9661
C	5.7442	-0.9956	-2.2424	C	-3.0126	0.5048	-3.2804
C	5.3674	-1.9493	-3.2051	C	-4.1612	1.2138	-3.6331
C	6.1301	-1.9503	-4.4996	C	-5.1716	1.4665	-2.6980
C	7.6356	-1.8772	-4.2284	C	-5.3350	0.4472	0.7923
H	8.5815	0.0674	-3.8742	C	-3.8814	0.0417	0.4615
H	7.0781	0.6805	-1.8921	H	-1.9461	-0.5242	-1.7037
C	5.0912	-0.9663	-0.9963	H	-2.2474	0.2928	-4.0320
C	4.2994	-2.8064	-2.9592	H	-4.2889	1.5615	-4.6623
H	5.8835	-2.8467	-5.0920	H	-6.0924	1.9771	-2.9874
H	8.1830	-1.8194	-5.1842	N	-5.8490	1.0830	-0.3116
C	3.5991	-2.7709	-1.7491	O	-5.9014	0.3003	1.8507
C	4.0620	-1.8911	-0.7656	C	-3.5213	-1.3507	0.8571
H	3.9701	-3.5023	-3.7371	C	-4.2071	-2.5858	1.0117
C	5.3104	0.1234	-0.0077	C	-2.2602	-1.6109	1.3307
C	6.5527	0.4347	0.5701	C	-5.6110	-2.9148	0.6422
C	4.2049	0.9197	0.3421	H	-5.7705	-2.7997	-0.4451
C	7.7768	-0.4287	0.4411	H	-5.8326	-3.9570	0.9185
C	6.6735	1.5589	1.4062	H	-6.3228	-2.2504	1.1614
C	4.3135	2.0696	1.1234	O	-1.2263	-0.7545	1.4022
C	9.0697	0.3660	0.1834	N	-3.4121	-3.4944	1.5478
H	7.6402	-1.2127	-0.3190	N	-2.2152	-2.8992	1.7625
C	8.0303	1.8344	1.9885	C	-1.1722	-3.6216	2.3835
C	5.5728	2.3803	1.6420	C	-1.3615	-4.9863	2.6337
C	9.0978	1.7307	0.8967	C	0.0291	-3.0094	2.7548
H	9.9213	-0.2558	0.5074	C	-0.3394	-5.7326	3.2121
H	8.0513	2.8291	2.4632	H	-2.3165	-5.4409	2.3695
H	5.6844	3.2751	2.2620	C	1.0489	-3.7722	3.3200
H	10.0958	1.9229	1.3252	H	0.1945	-1.9424	2.6257
H	5.8251	-1.0771	-5.1098	C	0.8782	-5.1362	3.5462
H	7.9390	-2.8250	-3.7519	H	-0.4988	-6.7988	3.3993
H	8.6697	-1.0427	-2.5065	H	1.9847	-3.2705	3.5821
H	6.3073	0.6300	-3.4718	H	1.6820	-5.7298	3.9906
H	7.8851	-0.9643	1.4040	C	-9.9048	-0.3552	-3.0125
H	9.2010	0.5177	-0.9008	C	-8.9582	-1.0892	-2.2965
H	8.9099	2.5377	0.1672	C	-9.9551	1.0309	-2.8680
H	8.2472	1.0983	2.7876	C	-8.0702	-0.4405	-1.4410
C	2.2955	-3.4543	-1.5897	H	-8.9096	-2.1768	-2.4052
C	1.2955	-3.1360	-2.4889	C	-9.0614	1.6777	-2.0144
C	1.9925	-4.2940	-0.4861	H	-10.6907	1.6155	-3.4287
C	-0.0373	-3.5715	-2.3001	C	-8.1142	0.9486	-1.2903
H	1.5138	-2.4605	-3.3219	H	-7.3273	-1.0213	-0.8864
C	0.7164	-4.7672	-0.2979	H	-9.0996	2.7675	-1.9115
H	2.7795	-4.5475	0.2281	C	-7.1704	1.6548	-0.3447
C	-1.0925	-3.1591	-3.1557	H	-7.1006	2.7248	-0.6092
C	-0.3379	-4.4021	-1.1769	H	-7.5447	1.5925	0.6915
H	0.4906	-5.4050	0.5613	H	-10.6008	-0.8637	-3.6861
C	-2.3908	-3.5430	-2.9067	C	-1.5269	0.6868	1.3937
H	-0.8582	-2.5135	-4.0084	C	-3.0393	0.9468	1.4053
C	-1.6823	-4.7982	-0.9605	H	-3.1751	2.0097	1.1555

C	-2.6872	-4.3723	-1.7995	H	-3.4575	0.7894	2.4076
H	-3.1983	-3.2003	-3.5604	C	-0.8991	1.2405	0.1336
H	-1.9144	-5.4280	-0.0984	C	-1.0458	2.4854	-0.3326
H	-3.7221	-4.6666	-1.6024	H	-0.3147	0.5325	-0.4533
C	3.1281	2.9309	1.3732	C	-0.4789	2.9216	-1.6144
C	2.6740	3.7890	0.3913	H	-1.6524	3.2168	0.2148
C	2.4522	2.8851	2.6208	C	0.7581	2.4298	-2.0539
C	1.5499	4.6266	0.6014	C	-1.2044	3.8193	-2.4276
H	3.1826	3.8263	-0.5764	C	1.2905	2.8261	-3.2772
C	1.3671	3.6974	2.8594	H	1.3284	1.7543	-1.4101
H	2.7853	2.1787	3.3850	C	-0.6533	4.2048	-3.6526
C	1.0589	5.4970	-0.4060	C	-2.5520	4.3271	-2.0037
C	0.8797	4.5799	1.8619	C	0.5840	3.7221	-4.0789
H	0.8557	3.6426	3.8234	H	2.2632	2.4403	-3.5970
C	-0.0467	6.2827	-0.1768	H	-1.2126	4.8973	-4.2900
H	1.5613	5.5167	-1.3779	H	-3.2560	3.4929	-1.8327
C	-0.2613	5.3988	2.0676	H	-2.4880	4.9101	-1.0665
C	-0.7153	6.2321	1.0704	H	-2.9870	4.9815	-2.7761
H	-0.4202	6.9421	-0.9656	H	0.9937	4.0455	-5.0406
H	-0.7725	5.3555	3.0344	N	-0.8351	1.2676	2.5219
H	-1.5948	6.8610	1.2390	H	0.0301	1.7520	2.2718
O	3.4282	-1.8663	0.4672	C	-0.8763	1.0617	3.8728
O	2.9362	0.5235	-0.0684	C	-2.0590	0.3157	4.5374
P	2.3376	-0.7222	0.7920	O	-0.0349	1.5106	4.6140
O	2.1270	-0.5022	2.2356	F	-1.7393	0.0097	5.7777
O	1.0510	-1.0968	-0.0565	F	-2.4012	-0.8082	3.9097
H	0.2353	-1.1501	0.5026	F	-3.1317	1.1148	4.5690
C	-4.9695	1.0162	-1.3983				

Bi-1a+2a:

C	-8.9197	-2.3588	-0.8925	C	4.8146	-0.5805	0.9342
C	-7.4334	-2.2915	-1.2898	C	3.4947	-0.8472	1.3184
C	-6.6140	-1.3762	-0.4361	C	3.2214	-1.9143	2.1681
C	-7.1683	-0.1040	-0.1932	C	4.2649	-2.7107	2.6517
C	-8.5667	0.1440	-0.6722	C	5.5990	-2.4426	2.3292
C	-9.4529	-1.0453	-0.3060	C	6.9343	0.2589	0.3210
H	-9.4919	-2.6447	-1.7869	C	5.4313	0.4767	0.1637
H	-6.9915	-3.2927	-1.3608	H	2.6905	-0.2119	0.9622
C	-5.3143	-1.6843	0.0234	H	2.1876	-2.1137	2.4572
C	-6.4259	0.8481	0.4733	H	4.0399	-3.5519	3.3114
H	-8.9579	1.0798	-0.2482	H	6.4089	-3.0533	2.7288
H	-10.4837	-0.8635	-0.6424	N	7.0873	-0.8721	1.0934
C	-5.1051	0.6153	0.9168	O	7.8414	0.8850	-0.1699
C	-4.5687	-0.6749	0.6548	C	4.8822	1.4730	-0.6048
H	-6.8918	1.8113	0.6892	C	5.4398	2.7340	-1.1073
C	-4.7208	-3.0281	-0.2070	C	3.5135	1.4473	-1.1783
C	-5.2900	-4.2027	0.3179	C	6.7205	3.4224	-0.7992
C	-3.4922	-3.1225	-0.8671	H	7.5619	2.9482	-1.3186
C	-6.5344	-4.2522	1.1554	H	6.6307	4.4701	-1.1129
C	-4.6250	-5.4302	0.1477	H	6.9507	3.3771	0.2740
C	-2.7717	-4.3219	-0.9802	O	2.6454	0.5715	-1.0875
C	-7.4942	-5.3748	0.7293	N	4.5596	3.3547	-1.8285

H	-7.0519	-3.2846	1.1877	N	3.4304	2.5855	-1.9306
C	-5.2888	-6.6505	0.7136	C	2.4243	2.9913	-2.8398
C	-3.3865	-5.4741	-0.4804	C	2.7464	3.9690	-3.7887
C	-6.7693	-6.6725	0.3310	C	1.1399	2.4361	-2.8323
H	-8.1935	-5.5571	1.5586	C	1.7948	4.3699	-4.7208
H	-4.7797	-7.5626	0.3703	H	3.7449	4.4040	-3.7802
H	-2.8585	-6.4279	-0.5574	C	0.1982	2.8455	-3.7713
H	-7.2568	-7.5446	0.7902	H	0.8622	1.6977	-2.0895
H	-8.5556	0.2769	-1.7698	C	0.5178	3.8100	-4.7222
H	-9.4915	-1.1129	0.7929	H	2.0593	5.1342	-5.4549
H	-9.0684	-3.1696	-0.1651	H	-0.8006	2.4039	-3.7442
H	-7.3686	-1.8684	-2.3101	H	-0.2269	4.1339	-5.4514
H	-6.2083	-4.4472	2.1928	C	9.1316	-5.4287	0.1959
H	-8.1088	-5.0283	-0.1145	C	8.4184	-4.5541	-0.6240
H	-6.8370	-6.8182	-0.7582	C	9.5857	-4.9929	1.4397
H	-5.1936	-6.6347	1.8149	C	8.1642	-3.2513	-0.2021
C	-4.4286	1.7268	1.5943	H	8.0572	-4.8881	-1.5995
C	-4.7383	3.0390	1.1635	C	9.3262	-3.6901	1.8621
C	-3.4693	1.5653	2.6249	H	10.1404	-5.6732	2.0899
C	-4.1153	4.1675	1.7152	C	8.6175	-2.8067	1.0436
H	-5.4556	3.1537	0.3501	H	7.5992	-2.5702	-0.8436
C	-2.8631	2.6669	3.1950	H	9.6768	-3.3564	2.8430
H	-3.2244	0.5671	2.9862	C	8.3708	-1.3894	1.4986
C	-4.4184	5.4737	1.2541	H	8.4673	-1.3226	2.5951
C	-3.1520	3.9942	2.7569	H	9.1178	-0.7068	1.0642
H	-2.1443	2.5278	4.0066	H	9.3301	-6.4512	-0.1331
C	-3.7929	6.5755	1.8172	C	-2.3219	5.3468	-2.5955
H	-5.1474	5.6001	0.4503	C	-1.5450	6.3932	-3.0788
C	-2.5320	5.1274	3.3085	C	-0.3043	6.6652	-2.4937
C	-2.8527	6.4022	2.8431	C	0.1397	5.8888	-1.4370
H	-4.0286	7.5793	1.4594	C	-0.6310	4.8237	-0.9340
H	-1.7994	5.0063	4.1100	C	-1.8935	4.5449	-1.5319
H	-2.3647	7.2734	3.2851	H	-3.2891	5.1344	-3.0582
C	-1.3376	-4.3029	-1.3475	H	-1.9045	6.9981	-3.9143
C	-0.8738	-3.6587	-2.4755	H	0.3160	7.4828	-2.8664
C	-0.4010	-4.7276	-0.3597	H	1.1107	6.1104	-0.9908
C	0.4800	-3.2487	-2.5758	C	-2.7639	3.4203	-1.0678
H	-1.5693	-3.3587	-3.2615	H	-2.2631	2.4387	-1.0851
C	0.9070	-4.3237	-0.4050	H	-3.0322	3.5706	-0.0144
H	-0.7640	-5.2964	0.4987	H	-3.6657	3.3566	-1.6908
C	0.9642	-2.5017	-3.6821	C	-0.1462	3.9871	0.1505
C	1.3617	-3.4910	-1.4642	C	1.0786	4.0342	0.7263
H	1.6000	-4.5827	0.3985	H	-0.8460	3.2252	0.5048
C	2.2228	-1.9386	-3.6599	C	1.5480	3.0999	1.7331
H	0.3060	-2.3478	-4.5406	H	1.8179	4.7731	0.4094
C	2.6539	-2.8805	-1.4655	C	2.7499	3.2506	2.3291
C	3.0620	-2.1049	-2.5350	H	3.1595	2.5178	3.0222
H	2.5735	-1.3508	-4.5104	H	3.3330	4.1506	2.1298
H	3.3241	-3.0658	-0.6225	N	0.7149	1.9860	1.9485
H	4.0510	-1.6430	-2.5238	H	0.2857	1.5930	1.1113
O	-3.2455	-0.9343	0.9850	C	0.4450	1.2357	3.0448
O	-2.9331	-1.9228	-1.3114	C	0.7560	1.8099	4.4486

P	-2.1465	-1.0868	-0.1898	O	-0.0824	0.1474	2.9805
O	-0.9387	-1.8705	0.3510	F	-0.0290	1.2143	5.3312
O	-1.6098	0.2032	-0.8144	F	0.5391	3.1202	4.5163
Bi	0.6220	-0.6621	-0.7864	F	2.0228	1.5649	4.7966
C	5.8556	-1.3698	1.4873				

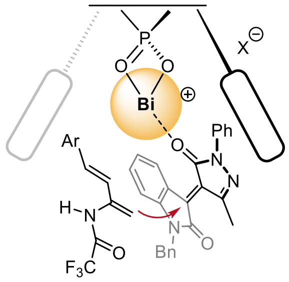

Bi-TS1:

C	9.7804	-1.3158	0.5174	C	-4.5660	-0.5042	-0.8701
C	8.2972	-1.5155	0.8746	C	-3.4938	-0.2789	-1.7279
C	7.3896	-0.4917	0.2557	C	-3.1396	-1.2571	-2.6625
C	7.7946	0.8495	0.3867	C	-3.8530	-2.4544	-2.7307
C	9.1443	1.1014	0.9918	C	-4.9298	-2.7058	-1.8789
C	10.1730	0.1620	0.3602	C	-6.3509	-0.6155	0.7180
H	10.3848	-1.7937	1.3034	C	-5.1705	0.2406	0.2495
H	7.9725	-2.5399	0.6512	H	-2.9017	0.6331	-1.6596
C	6.1542	-0.7885	-0.3437	H	-2.3034	-1.0811	-3.3414
C	6.9694	1.8753	-0.0618	H	-3.5699	-3.2119	-3.4650
H	9.4341	2.1539	0.8575	H	-5.4775	-3.6464	-1.9300
H	11.1688	0.3442	0.7912	N	-6.3204	-1.7506	-0.0326
C	5.7037	1.6212	-0.6087	O	-7.1364	-0.3315	1.5941
C	5.3304	0.2856	-0.7316	C	-4.4034	0.8182	1.3444
H	7.3093	2.9108	0.0146	C	-4.9127	1.3896	2.4912
C	5.6615	-2.1806	-0.5140	C	-2.9455	0.8080	1.5188
C	6.2940	-3.1914	-1.2657	C	-6.3140	1.6583	2.8938
C	4.4324	-2.4877	0.0594	H	-6.8017	0.7167	3.1972
C	7.5572	-3.0045	-2.0523	H	-6.3231	2.3705	3.7288
C	5.6801	-4.4617	-1.3843	H	-6.9242	2.0553	2.0714
C	3.7847	-3.7024	-0.0916	O	-2.0406	0.3704	0.7822
C	8.5581	-4.1548	-1.8549	N	-3.9116	1.7554	3.3449
H	8.0265	-2.0336	-1.8475	N	-2.7717	1.4585	2.7712
C	6.4150	-5.5009	-2.1774	C	-1.5534	1.6748	3.4472
C	4.4349	-4.7068	-0.8054	C	-1.4329	1.3336	4.7992
C	7.8872	-5.5378	-1.7623	C	-0.4934	2.2375	2.7357
H	9.2771	-4.1277	-2.6875	C	-0.2077	1.5408	5.4288
H	5.9450	-6.4870	-2.0485	H	-2.2858	0.9109	5.3319
H	3.9524	-5.6788	-0.9344	C	0.7160	2.4422	3.3810
H	8.4278	-6.2715	-2.3785	H	-0.6376	2.5964	1.7073
H	9.1006	0.9241	2.0825	C	0.8693	2.0813	4.7197
H	10.2467	0.4173	-0.7092	H	-0.0933	1.2738	6.4812
H	10.0133	-1.8559	-0.4126	H	1.5344	2.8858	2.8212
H	8.1863	-1.4048	1.9690	H	1.8277	2.2370	5.2185
H	7.2659	-2.9742	-3.1182	C	-5.5783	-6.5853	1.2367
H	9.1457	-3.9734	-0.9424	C	-5.2446	-5.4220	1.9317
H	7.9368	-5.9083	-0.7259	C	-6.4876	-6.5220	0.1818

H	6.3415	-5.2579	-3.2537
C	4.8278	2.7386	-1.0181
C	4.7138	3.8460	-0.1989
C	4.1514	2.7372	-2.2695
C	3.9740	4.9885	-0.5902
H	5.2114	3.8548	0.7744
C	3.4248	3.8295	-2.6758
H	4.2379	1.8664	-2.9212
C	3.8806	6.1432	0.2319
C	3.3253	4.9904	-1.8630
H	2.9339	3.8263	-3.6522
C	3.1922	7.2547	-0.1954
H	4.3792	6.1402	1.2046
C	2.6213	6.1516	-2.2730
C	2.5588	7.2599	-1.4604
H	3.1373	8.1422	0.4389
H	2.1364	6.1559	-3.2527
H	2.0239	8.1524	-1.7920
C	2.3945	-3.6757	0.3605
C	2.1257	-3.1879	1.6157
C	1.3308	-3.9137	-0.5509
C	0.8344	-2.7624	1.9643
H	2.9385	-3.0297	2.3283
C	0.0573	-3.5070	-0.2461
H	1.5524	-4.3354	-1.5332
C	0.5813	-2.1452	3.2105
C	-0.2150	-2.8490	0.9876
H	-0.7516	-3.6292	-0.9706
C	-0.6393	-1.5818	3.4679
H	1.3820	-2.0998	3.9534
C	-1.4669	-2.2382	1.2821
C	-1.6650	-1.6094	2.4921
H	-0.8058	-1.1097	4.4278
H	-2.2723	-2.2854	0.5429
H	-2.6258	-1.1389	2.6952
O	4.0502	0.0109	-1.2392
O	3.7246	-1.4788	0.7312
P	2.9027	-0.5338	-0.2794
O	1.8306	-1.2832	-1.0814
O	2.1280	0.5102	0.5425
Bi	0.0613	-0.4609	0.1868
C	-5.2725	-1.7204	-0.9609
C	-5.8167	-4.2068	1.5713
H	-4.5381	-5.4600	2.7622
C	-7.0469	-5.2982	-0.1849
H	-6.7585	-7.4280	-0.3648
C	-6.7115	-4.1315	0.5054
H	-5.5650	-3.3088	2.1424
H	-7.7502	-5.2500	-1.0212
C	-7.3058	-2.8088	0.1062
H	-7.8563	-2.9042	-0.8449
H	-8.0188	-2.4557	0.8686
H	-5.1315	-7.5407	1.5197
C	0.3546	4.6909	0.2129
C	0.0606	5.2223	1.4673
C	-1.2569	5.2517	1.9455
C	-2.2716	4.7348	1.1616
C	-1.9948	4.1931	-0.1170
C	-0.6521	4.1774	-0.6062
H	1.3910	4.6759	-0.1363
H	0.8724	5.6176	2.0806
H	-1.4787	5.6729	2.9271
H	-3.2973	4.7586	1.5331
C	-0.3067	3.5960	-1.9456
H	-0.5693	2.5236	-2.0042
H	-0.8258	4.1159	-2.7666
H	0.7700	3.6801	-2.1350
C	-3.0066	3.5799	-0.9208
C	-4.2660	3.2110	-0.5160
H	-2.6763	3.2873	-1.9234
C	-5.1466	2.3825	-1.2570
H	-4.5718	3.3980	0.5131
C	-6.1312	1.6409	-0.4970
H	-6.9104	1.1277	-0.9895
H	-6.4697	2.1554	0.3694
N	-5.0068	2.2626	-2.5816
H	-4.2444	2.8025	-2.9972
C	-5.5737	1.4626	-3.6035
C	-7.0182	0.9182	-3.4789
O	-4.9598	1.3117	-4.6149
F	-7.4742	0.6662	-4.6808
F	-7.8026	1.8187	-2.8919
F	-7.0482	-0.2023	-2.7621

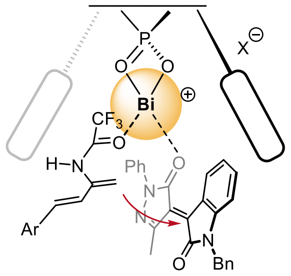

Bi-TS1':

C	6.9577	-5.5889	0.1387	C	-4.7153	1.0892	-1.3206
C	6.3331	-4.3329	-0.4999	C	-3.5786	1.0875	-2.1097
C	4.9979	-3.9750	0.0765	C	-3.5767	0.3853	-3.3186
C	4.0801	-5.0312	0.2063	C	-4.7142	-0.2999	-3.7486
C	4.5607	-6.4134	-0.1106	C	-5.8786	-0.3020	-2.9819
C	5.9187	-6.6367	0.5669	C	-6.5310	1.3522	0.2117
H	7.6661	-6.0248	-0.5906	C	-5.0291	1.6807	0.0036
H	7.0398	-3.4840	-0.4765	H	-2.6989	1.6333	-1.7706
C	4.5952	-2.6733	0.3926	H	-2.6841	0.3792	-3.9490
C	2.7966	-4.7805	0.6598	H	-4.6893	-0.8574	-4.6890
H	3.8314	-7.1764	0.2175	H	-6.7542	-0.8583	-3.3119
H	6.2851	-7.6563	0.3534	N	-6.9080	0.5259	-0.8445
C	2.3463	-3.4737	0.9124	O	-7.2409	1.6602	1.1331
C	3.2540	-2.4418	0.7250	C	-4.5556	2.9848	0.4011
H	2.1108	-5.6162	0.8233	C	-5.2489	4.1581	0.6730
C	5.5193	-1.5396	0.3888	C	-3.1384	3.3053	0.5942
C	6.5774	-1.5428	1.3051	C	-6.6753	4.5011	0.5397
C	5.2962	-0.4215	-0.4525	H	-7.2736	4.0538	1.3564
C	6.8255	-2.5956	2.3452	H	-6.8207	5.5962	0.5731
C	7.4380	-0.4395	1.3587	H	-7.0938	4.1213	-0.4090
C	6.1704	0.6730	-0.4258	O	-2.1365	2.6680	0.3502
C	8.3072	-3.0071	2.4233	N	-4.3675	5.1516	1.1305
H	6.1807	-3.4809	2.2144	N	-3.1380	4.6493	1.1299
C	8.5661	-0.4704	2.3422	C	-2.0640	5.3277	1.7625
C	7.2211	0.6256	0.5097	C	-2.3181	6.5246	2.4528
C	9.2779	-1.8272	2.2566	C	-0.7673	4.7957	1.7255
H	8.4741	-3.5102	3.3935	C	-1.2698	7.1876	3.0806
H	9.2813	0.3535	2.1617	H	-3.3341	6.9277	2.4941
H	7.9244	1.4664	0.5353	C	0.2679	5.4720	2.3630
H	10.0800	-1.8735	3.0144	H	-0.5585	3.8503	1.2237
H	4.6618	-6.5281	-1.2102	C	0.0244	6.6685	3.0363
H	5.7672	-6.5821	1.6627	H	-1.4681	8.1198	3.6177
H	7.5636	-5.3031	1.0191	H	1.2771	5.0530	2.3386
H	6.1619	-4.5457	-1.5804	H	0.8411	7.1920	3.5374
H	6.5330	-2.1559	3.3252	C	-7.4133	-4.3510	-0.1580
H	8.5191	-3.7644	1.6448	C	-6.9152	-3.3681	0.6983
H	9.7774	-1.8951	1.2709	C	-8.1677	-3.9780	-1.2676
H	8.1653	-0.3146	3.3658	C	-7.1953	-2.0314	0.4371
C	0.9823	-3.3195	1.3958	H	-6.3222	-3.6460	1.5721
C	-0.0378	-4.0703	0.8795	C	-8.4315	-2.6327	-1.5286
C	0.7334	-2.4397	2.4890	H	-8.5411	-4.7416	-1.9509
C	-1.3512	-3.9845	1.4088	C	-7.9611	-1.6428	-0.6707
H	0.1461	-4.7681	0.0574	H	-6.7996	-1.2830	1.1127
C	-0.5182	-2.3525	3.0307	H	-9.0210	-2.3656	-2.4097
H	1.5641	-1.8811	2.9281	C	-8.1960	-0.1691	-0.9073
C	-2.4221	-4.7577	0.8881	H	-8.6962	0.0224	-1.8801
C	-1.5941	-3.1048	2.4915	H	-8.8431	0.2631	-0.1089
H	-0.6972	-1.7274	3.9055	H	-7.2085	-5.4054	0.0392
C	-3.6848	-4.6418	1.4141	C	-2.3942	-4.0977	-3.9743
H	-2.2269	-5.4478	0.0658	C	-3.6402	-4.6878	-4.1688
C	-2.8998	-3.0145	3.0219	C	-4.6788	-4.4557	-3.2648
C	-3.9268	-3.7582	2.4908	C	-4.4691	-3.6250	-2.1780

H	-4.5052	-5.2341	1.0049	C	-3.2165	-3.0139	-1.9638
H	-3.0810	-2.3457	3.8642	C	-2.1473	-3.2689	-2.8817
H	-4.9349	-3.6842	2.9041	H	-1.5921	-4.2942	-4.6912
C	6.1358	1.9368	-1.1606	H	-3.8026	-5.3430	-5.0320
C	6.0840	1.9775	-2.5332	H	-5.6564	-4.9303	-3.4179
C	6.1220	3.1646	-0.4422	H	-5.2653	-3.4498	-1.4518
C	5.9686	3.1900	-3.2466	C	-0.7973	-2.6592	-2.7375
H	6.1751	1.0468	-3.0974	H	-0.8018	-1.5503	-2.7162
C	5.9400	4.3521	-1.1007	H	-0.3097	-2.9793	-1.8028
H	6.2270	3.1453	0.6429	H	-0.1270	-2.9612	-3.5602
C	5.9667	3.2157	-4.6610	C	-2.9811	-2.1148	-0.8863
C	5.8382	4.3939	-2.5186	C	-3.8552	-1.3972	-0.0780
H	5.9162	5.2902	-0.5462	H	-1.9182	-1.9176	-0.7353
C	5.8580	4.4015	-5.3208	C	-3.4270	-0.3084	0.7023
H	6.0819	2.2807	-5.1961	H	-4.9463	-1.5754	-0.1495
C	5.6653	5.6095	-3.2340	C	-4.3875	0.6186	1.2924
C	5.6824	5.6099	-4.6028	H	-4.0610	1.3256	2.0347
H	5.8826	4.4358	-6.4052	H	-5.2871	0.1338	1.6702
H	5.5757	6.5405	-2.6748	N	-2.0517	0.0166	0.6709
H	5.5887	6.5438	-5.1513	H	-1.5130	-0.3481	-0.1399
O	2.8413	-1.1345	0.9416	C	-1.2529	0.7116	1.5506
O	4.0950	-0.4608	-1.2239	C	-1.7051	1.0604	2.9939
P	2.8166	-0.1154	-0.2795	O	-0.1024	1.0743	1.2985
O	2.8369	1.3136	0.3422	F	-0.6736	1.0552	3.8189
O	1.4844	-0.0898	-1.0682	F	-2.6208	0.2355	3.4699
Bi	0.8480	2.1211	-0.4664	F	-2.2183	2.2864	3.0327
C	-5.8728	0.4041	-1.7866				

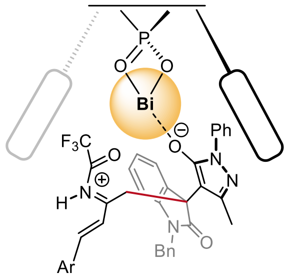

Bi-Int1:

C	9.7688	-0.8433	0.8778	C	-4.6738	-0.3555	-0.7060
C	8.2770	-1.1308	1.1200	C	-3.6974	0.1003	-1.5849
C	7.3603	-0.1506	0.4465	C	-3.3486	-0.6769	-2.6917
C	7.6789	1.2097	0.6228	C	-3.9720	-1.9064	-2.9098
C	8.9636	1.5271	1.3301	C	-4.9518	-2.3853	-2.0373
C	10.0875	0.6565	0.7672	C	-6.2944	-0.8604	0.9694
H	10.3388	-1.2985	1.7017	C	-5.3021	0.2623	0.5274
H	8.0288	-2.1679	0.8616	H	-3.1745	1.0388	-1.4021
C	6.1914	-0.5112	-0.2469	H	-2.5871	-0.3193	-3.3876
C	6.8360	2.1959	0.1250	H	-3.6943	-2.5075	-3.7785
H	9.2024	2.5958	1.2294	H	-5.4274	-3.3504	-2.2075
H	11.0363	0.8876	1.2735	N	-6.2453	-1.8558	0.0381
C	5.6320	1.8805	-0.5161	O	-7.0251	-0.8097	1.9280
C	5.3416	0.5218	-0.6791	C	-4.3620	0.6066	1.6479
H	7.1122	3.2477	0.2360	C	-4.6251	0.9859	2.9590

C	5.8026	-1.9313	-0.4706	C	-2.9106	0.6109	1.5501
C	6.5822	-2.8550	-1.1876	C	-5.8807	1.1852	3.7271
C	4.5526	-2.3535	-0.0061	H	-6.3176	0.2175	4.0085
C	7.8839	-2.5265	-1.8571	H	-5.6551	1.7589	4.6349
C	6.1026	-4.1648	-1.3871	H	-6.6546	1.7082	3.1494
C	4.0254	-3.6280	-0.2440	O	-2.1373	0.3462	0.6320
C	8.9694	-3.5840	-1.6020	N	-3.4551	1.2222	3.6381
H	8.2404	-1.5240	-1.5888	N	-2.4612	1.0250	2.8096
C	6.9956	-5.1079	-2.1386	C	-1.1049	1.0847	3.2066
C	4.8436	-4.5357	-0.9228	C	-0.7255	0.5254	4.4257
C	8.4254	-5.0243	-1.6003	C	-0.1694	1.7003	2.3655
H	9.7494	-3.4634	-2.3685	C	0.6143	0.5709	4.7996
H	6.6102	-6.1357	-2.0739	H	-1.4801	0.0614	5.0629
H	4.4733	-5.5455	-1.1183	C	1.1697	1.7365	2.7540
H	9.0813	-5.6857	-2.1852	H	-0.4985	2.2164	1.4592
H	8.8490	1.3314	2.4123	C	1.5619	1.1638	3.9635
H	10.2257	0.9320	-0.2905	H	0.9214	0.1325	5.7513
H	10.1007	-1.3553	-0.0380	H	1.9047	2.2106	2.1013
H	8.0792	-1.0430	2.2035	H	2.6122	1.1874	4.2607
H	7.6804	-2.4886	-2.9424	C	-4.9848	-6.7263	0.4644
H	9.4593	-3.3786	-0.6382	C	-4.6943	-5.6544	1.3093
H	8.4229	-5.4213	-0.5731	C	-5.9827	-6.5957	-0.5004
H	6.9899	-4.8385	-3.2108	C	-5.3998	-4.4606	1.1853
C	4.7317	2.9591	-0.9868	H	-3.9168	-5.7497	2.0709
C	4.4937	4.0576	-0.1819	C	-6.6773	-5.3928	-0.6310
C	4.1579	2.9289	-2.2906	H	-6.2183	-7.4324	-1.1619
C	3.7292	5.1620	-0.6363	C	-6.3879	-4.3146	0.2084
H	4.9099	4.0911	0.8288	H	-5.1883	-3.6305	1.8640
C	3.4108	3.9816	-2.7567	H	-7.4505	-5.2911	-1.3976
H	4.3412	2.0676	-2.9329	C	-7.1273	-3.0078	0.0709
C	3.5091	6.3101	0.1709	H	-7.7492	-3.0079	-0.8386
C	3.1861	5.1334	-1.9581	H	-7.7967	-2.8462	0.9294
H	3.0011	3.9549	-3.7700	H	-4.4339	-7.6646	0.5602
C	2.8012	7.3869	-0.3142	C	-0.0473	5.1247	0.5295
H	3.9262	6.3318	1.1811	C	-0.2040	5.4167	1.8830
C	2.4577	6.2574	-2.4276	C	-1.4371	5.2266	2.5200
C	2.2724	7.3616	-1.6262	C	-2.5005	4.7317	1.7925
H	2.6498	8.2693	0.3116	C	-2.3631	4.4287	0.4137
H	2.0540	6.2369	-3.4435	C	-1.1075	4.6393	-0.2358
H	1.7209	8.2260	-2.0029	H	0.9238	5.2808	0.0573
C	2.5943	-3.8678	0.0358	H	0.6502	5.7978	2.4473
C	2.0209	-3.5326	1.2457	H	-1.5542	5.4620	3.5790
C	1.7527	-4.2496	-1.0465	H	-3.4606	4.5859	2.2891
C	0.6168	-3.4100	1.3799	C	-0.8877	4.3326	-1.6877
H	2.6517	-3.2642	2.0945	H	-1.0472	3.2664	-1.9197
C	0.3891	-4.1394	-0.9554	H	-1.5553	4.9204	-2.3370
H	2.2118	-4.5523	-1.9895	H	0.1437	4.5762	-1.9704
C	0.0175	-2.9486	2.5833	C	-3.4327	3.8434	-0.3355
C	-0.2171	-3.6458	0.2324	C	-4.5941	3.2965	0.1628
H	-0.2435	-4.3768	-1.8136	H	-3.2343	3.7368	-1.4064
C	-1.3312	-2.6760	2.6358	C	-5.5104	2.5161	-0.5833
H	0.6490	-2.7865	3.4603	H	-4.7641	3.2972	1.2388

C	-1.6066	-3.3375	0.3169	C	-6.2553	1.4831	0.1720
C	-2.1417	-2.8526	1.4910	H	-7.1321	1.1035	-0.3535
H	-1.7762	-2.3129	3.5650	H	-6.6087	1.9234	1.1141
H	-2.2489	-3.4995	-0.5530	N	-5.5433	2.6118	-1.9206
H	-3.2030	-2.6150	1.5362	H	-4.8787	3.2706	-2.3299
O	4.1140	0.1995	-1.2748	C	-6.1878	1.9446	-2.9895
O	3.7553	-1.3936	0.6242	C	-7.5690	1.2692	-2.7967
P	2.9545	-0.4401	-0.3926	O	-5.7030	2.0126	-4.0769
O	1.9644	-1.2165	-1.2763	F	-8.1559	1.1857	-3.9633
O	2.1045	0.5204	0.4668	F	-8.3249	1.9935	-1.9742
Bi	0.1233	-0.5464	-0.0187	F	-7.4428	0.0445	-2.2892
C	-5.2906	-1.5931	-0.9452				-

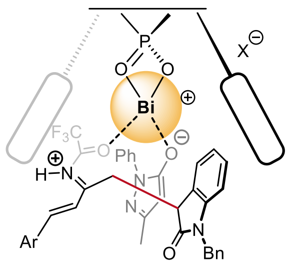

Bi-Int1':

C	-8.4264	3.8817	-0.5363	C	4.1026	-0.4606	-1.3106
C	-7.3197	2.9640	-1.0907	C	2.8730	-0.4594	-1.9491
C	-6.0997	2.8962	-0.2148	C	2.6448	0.4128	-3.0199
C	-5.5955	4.1364	0.2211	C	3.6602	1.2637	-3.4561
C	-6.4058	5.3582	-0.1024	C	4.9243	1.2392	-2.8611
C	-7.8768	5.0978	0.2274	C	6.1145	-0.7897	-0.0864
H	-9.0483	4.2093	-1.3856	C	4.6033	-1.1265	-0.0451
H	-7.7177	1.9655	-1.3210	H	2.0882	-1.1416	-1.6188
C	-5.4103	1.7087	0.1001	H	1.6722	0.4239	-3.5208
C	-4.4078	4.1988	0.9408	H	3.4714	1.9591	-4.2781
H	-6.0242	6.2310	0.4513	H	5.7171	1.9024	-3.2111
H	-8.4780	5.9957	0.0098	N	6.2915	0.1580	-1.0630
C	-3.6581	3.0508	1.2199	O	6.9564	-1.1711	0.6884
C	-4.1916	1.8336	0.7905	C	4.2941	-2.5880	0.0790
H	-4.0362	5.1674	1.2873	C	5.1001	-3.7136	0.0477
C	-5.8973	0.3609	-0.3187	C	2.9592	-3.1060	0.3474
C	-7.1462	-0.1773	0.0483	C	6.5369	-3.9073	-0.2800
C	-5.0391	-0.4638	-1.0534	H	7.1758	-3.5847	0.5574
C	-8.1368	0.5019	0.9481	H	6.7170	-4.9736	-0.4836
C	-7.4967	-1.4802	-0.3649	H	6.8335	-3.3154	-1.1595
C	-5.3268	-1.7885	-1.4163	O	1.8770	-2.5022	0.3286
C	-9.5768	0.4371	0.4099	N	4.3706	-4.8314	0.3486
H	-7.8457	1.5377	1.1722	N	3.1175	-4.4835	0.5514
C	-8.8542	-1.9785	0.0381	C	2.1955	-5.4309	1.0736
C	-6.5982	-2.2651	-1.0808	C	2.6764	-6.6960	1.4296
C	-9.9165	-0.9140	-0.2465	C	0.8433	-5.1237	1.2564
H	-10.2612	0.6465	1.2483	C	1.8003	-7.6465	1.9422
H	-9.0921	-2.9187	-0.4847	H	3.7349	-6.9232	1.2984
H	-6.8790	-3.2865	-1.3542	C	-0.0217	-6.0812	1.7792
H	-10.9001	-1.2758	0.0942	H	0.4657	-4.1355	1.0120

H	-6.3069	5.5961	-1.1797	C	0.4485	-7.3481	2.1189
H	-7.9534	4.9257	1.3149	H	2.1848	-8.6329	2.2149
H	-9.0929	3.3051	0.1250	H	-1.0748	-5.8264	1.9277
H	-6.9821	3.3785	-2.0602	H	-0.2324	-8.0974	2.5308
H	-8.1034	-0.0355	1.9153	C	7.3331	4.9268	0.1536
H	-9.7311	1.2477	-0.3201	C	7.0176	3.8630	1.0021
H	-9.9907	-0.7925	-1.3406	C	7.7247	4.6703	-1.1594
H	-8.8494	-2.2123	1.1207	C	7.0912	2.5532	0.5340
C	-2.3396	3.1389	1.8857	H	6.7257	4.0557	2.0386
C	-1.4385	4.1028	1.4684	C	7.7888	3.3567	-1.6286
C	-1.9665	2.2582	2.9393	H	7.9812	5.4975	-1.8274
C	-0.1455	4.2113	2.0399	C	7.4721	2.2874	-0.7882
H	-1.7155	4.7942	0.6669	H	6.8791	1.7207	1.2138
C	-0.7251	2.3473	3.5199	H	8.0964	3.1643	-2.6616
H	-2.6827	1.5158	3.2968	C	7.5416	0.8578	-1.2712
C	0.7944	5.1802	1.6008	H	7.8077	0.8090	-2.3394
C	0.2280	3.3046	3.0794	H	8.3042	0.2961	-0.7080
H	-0.4601	1.6753	4.3420	H	7.2809	5.9556	0.5211
C	2.0524	5.2397	2.1557	C	1.8252	5.3387	-2.7249
H	0.5074	5.8781	0.8085	C	3.0952	5.9113	-2.8133
C	1.5279	3.3978	3.6381	C	4.1369	5.4278	-2.0174
C	2.4230	4.3398	3.1815	C	3.8991	4.3824	-1.1398
H	2.7711	5.9834	1.7999	C	2.6165	3.8025	-1.0263
H	1.8098	2.7060	4.4376	C	1.5555	4.2900	-1.8456
H	3.4236	4.4006	3.6194	H	1.0195	5.7182	-3.3608
C	-4.2196	-2.6606	-1.8644	H	3.2708	6.7377	-3.5080
C	-3.2259	-2.2069	-2.7145	H	5.1358	5.8676	-2.0798
C	-4.0280	-3.9072	-1.1987	H	4.7201	4.0177	-0.5177
C	-1.9514	-2.8263	-2.7362	C	0.1871	3.6720	-1.8195
H	-3.3598	-1.2891	-3.2879	H	0.2171	2.6019	-2.0915
C	-2.8319	-4.5756	-1.2523	H	-0.2813	3.7319	-0.8229
H	-4.8328	-4.2984	-0.5713	H	-0.4825	4.1809	-2.5303
C	-0.8388	-2.2348	-3.4093	C	2.3719	2.7078	-0.1289
C	-1.7317	-4.0193	-1.9592	C	3.3309	1.8859	0.4414
H	-2.6974	-5.5129	-0.7044	H	1.3187	2.4851	0.0636
C	0.4261	-2.7978	-3.3267	C	3.0705	0.6753	1.0876
H	-1.0023	-1.3261	-3.9967	H	4.3878	2.1086	0.2691
C	-0.4188	-4.5800	-1.9126	C	4.1625	-0.3203	1.2885
C	0.6363	-3.9805	-2.5760	H	3.9631	-1.0611	2.0678
H	1.2638	-2.3323	-3.8525	H	5.0374	0.2378	1.6468
H	-0.2587	-5.4968	-1.3382	N	1.7448	0.2781	1.1920
H	1.6350	-4.4218	-2.5262	H	1.1110	0.7149	0.5100
O	-3.4371	0.6734	1.0537	C	1.1066	-0.5647	1.9947
O	-3.7572	0.0288	-1.3314	C	1.6723	-1.0187	3.3472
P	-2.7378	-0.1242	-0.1166	O	-0.0268	-1.0202	1.6633
O	-2.5143	-1.5872	0.3519	F	0.6736	-1.2287	4.1762
O	-1.3374	0.3252	-0.6216	F	2.4836	-0.1068	3.8631
Bi	-0.4334	-1.7650	-0.4086	F	2.3488	-2.1631	3.2151
C	5.1279	0.3614	-1.8016				

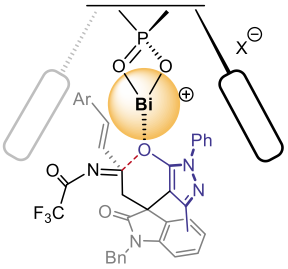

Bi-TS2-[2+4]:

C	-9.6843	-0.2767	0.0025	C	5.0307	-1.2291	0.2496
C	-8.3425	-0.4026	-0.7394	C	4.2247	-2.0827	-0.4737
C	-7.2320	0.3704	-0.0902	C	4.6336	-3.4011	-0.6970
C	-7.5310	1.7097	0.2291	C	5.8513	-3.8373	-0.1734
C	-8.9504	2.1572	0.0433	C	6.6933	-2.9766	0.5397
C	-9.8967	1.1099	0.6314	C	6.2155	0.5672	1.2498
H	-10.4879	-0.5022	-0.7143	C	4.8428	0.2263	0.5973
H	-8.0743	-1.4547	-0.8975	H	3.3191	-1.6655	-0.9023
C	-5.9325	-0.1231	0.1208	H	4.0219	-4.0828	-1.2943
C	-6.5323	2.5586	0.6902	H	6.1750	-4.8656	-0.3477
H	-9.1060	3.1398	0.5114	H	7.6633	-3.3198	0.8990
H	-10.9410	1.4329	0.5115	N	6.9452	-0.5938	1.3240
C	-5.2167	2.1132	0.8566	O	6.5545	1.6568	1.6392
C	-4.9670	0.7704	0.6045	C	4.5287	1.0659	-0.6099
H	-6.7630	3.6040	0.9093	C	5.2454	1.5241	-1.7670
C	-5.5195	-1.5015	-0.2377	C	3.2600	1.5147	-0.8445
C	-6.1161	-2.6537	0.3060	C	6.6915	1.3659	-2.0598
C	-4.4337	-1.6705	-1.1040	H	6.9597	0.3032	-2.1671
C	-7.1753	-2.6379	1.3680	H	6.9452	1.8904	-2.9890
C	-5.6551	-3.9285	-0.0775	H	7.2947	1.7872	-1.2416
C	-3.9029	-2.9241	-1.4352	O	2.1412	1.3590	-0.1507
C	-8.3317	-3.6093	1.0837	N	4.4442	2.1579	-2.6079
H	-7.5481	-1.6247	1.5621	N	3.2171	2.1638	-2.0376
C	-6.3539	-5.1131	0.5201	C	2.0672	2.5922	-2.7062
C	-4.5730	-4.0502	-0.9401	C	2.0796	2.7955	-4.0876
C	-7.8701	-4.9309	0.4444	C	0.8781	2.8424	-1.9765
H	-8.8545	-3.7989	2.0326	C	0.9250	3.2568	-4.7179
H	-6.0388	-6.0403	0.0203	H	3.0037	2.6284	-4.6413
H	-4.2093	-5.0481	-1.1983	C	-0.2787	3.2888	-2.6376
H	-8.3698	-5.7854	0.9232	H	0.9231	2.8745	-0.8891
H	-9.1594	2.2850	-1.0348	C	-0.2606	3.4933	-4.0101
H	-9.7097	1.0620	1.7161	H	0.9559	3.4386	-5.7948
H	-9.7506	-1.0471	0.7854	H	-1.1592	3.4635	-2.0119
H	-8.4670	0.0245	-1.7505	H	-1.1501	3.8539	-4.5293
H	-6.6783	-2.9549	2.3029	C	11.3346	-1.8659	-0.8677
H	-9.0673	-3.1228	0.4263	C	10.3980	-0.8979	-1.2312
H	-8.1628	-4.9557	-0.6165	C	11.2767	-2.4387	0.4014
H	-6.0520	-5.2105	1.5792	C	9.4093	-0.5087	-0.3312
C	-4.0953	3.0314	1.1215	H	10.4401	-0.4420	-2.2233
C	-3.9246	4.1196	0.2895	C	10.2816	-2.0513	1.2987
C	-3.1348	2.7492	2.1312	H	12.0062	-3.1971	0.6945
C	-2.7611	4.9218	0.3719	C	9.3391	-1.0838	0.9409
H	-4.6672	4.3377	-0.4821	H	8.6771	0.2451	-0.6224

C	-1.9735	3.4753	2.2066	H	10.2343	-2.5124	2.2894
H	-3.3560	1.9406	2.8352	C	8.2645	-0.6703	1.9171
C	-2.5331	6.0240	-0.4914	H	8.2347	-1.3745	2.7640
C	-1.7528	4.5617	1.3149	H	8.4661	0.3320	2.3254
H	-1.2187	3.2784	2.9694	H	12.1088	-2.1725	-1.5748
C	-1.3513	6.7265	-0.4343	C	2.3321	0.7536	1.3882
H	-3.3105	6.3028	-1.2073	C	3.8089	0.5388	1.7156
C	-0.5475	5.3119	1.3571	H	3.8675	-0.2519	2.4736
C	-0.3437	6.3613	0.4905	H	4.1527	1.4636	2.1972
H	-1.1852	7.5706	-1.1077	C	1.4321	-0.4490	1.2610
H	0.2062	5.0476	2.1041	C	0.3654	-0.7206	2.0757
H	0.5861	6.9334	0.5289	H	1.9143	-1.2860	0.7424
C	-2.5552	-3.0034	-2.0189	C	-0.2968	-1.9822	2.2010
C	-2.0922	-2.0936	-2.9520	H	-0.0345	0.1059	2.6748
C	-1.6321	-3.9239	-1.4428	C	0.2654	-3.1229	1.5987
C	-0.7058	-1.9179	-3.1602	C	-1.5094	-2.0844	2.9314
H	-2.7845	-1.4320	-3.4713	C	-0.3416	-4.3588	1.7000
C	-0.2878	-3.8318	-1.6802	H	1.2180	-3.0429	1.0721
H	-2.0015	-4.6773	-0.7479	C	-2.1165	-3.3405	3.0070
C	-0.1830	-0.8407	-3.9550	C	-2.1256	-0.8827	3.5924
C	0.2287	-2.7835	-2.4876	C	-1.5474	-4.4636	2.4026
H	0.4017	-4.5345	-1.2071	H	0.1215	-5.2415	1.2505
C	1.1873	-0.6433	-4.0731	H	-3.0549	-3.4387	3.5564
H	-0.8850	-0.1950	-4.4901	H	-1.4669	-0.4642	4.3687
C	1.6191	-2.5572	-2.6548	H	-2.3333	-0.0952	2.8479
C	2.0960	-1.5048	-3.4163	H	-3.0824	-1.1407	4.0632
H	1.5610	0.1823	-4.6819	H	-2.0432	-5.4329	2.4927
H	2.2805	-3.2706	-2.1553	N	1.7612	1.8543	2.1238
H	3.1715	-1.3463	-3.5217	H	1.2103	2.5148	1.5824
O	-3.6393	0.3227	0.7861	C	1.6186	2.1160	3.4562
O	-3.7589	-0.5163	-1.5540	C	2.3221	1.2227	4.5075
P	-2.7237	0.0564	-0.4709	O	0.9504	3.0465	3.8309
O	-1.6166	-0.9655	-0.1248	F	1.7295	1.3664	5.6692
O	-1.9131	1.2397	-1.0768	F	3.5998	1.5651	4.6204
Bi	0.0540	0.1084	-1.2185	F	2.2699	-0.0748	4.1675
C	6.2719	-1.6632	0.7277				

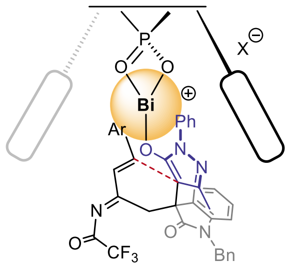

Bi-TS2-[4+2]:

C	9.1243	1.2781	2.0647	C	-4.0352	-0.4507	-1.6336
C	7.7832	0.5309	1.9460	C	-3.2954	0.1869	-2.6228
C	6.7544	1.2688	1.1573	C	-2.7839	-0.5700	-3.6852
C	6.5817	2.6362	1.4997	C	-3.0358	-1.9404	-3.7587
C	7.5377	3.2173	2.4969	C	-3.8185	-2.5839	-2.7938
C	8.9654	2.8035	2.1400	C	-5.2898	-1.2222	0.2197

H	9.6384	0.8973	2.9587	C	-4.6846	0.0735	-0.3810
H	7.9224	-0.4934	1.5799	H	-3.1229	1.2652	-2.5920
C	5.9176	0.6636	0.1905	H	-2.1984	-0.0803	-4.4658
C	5.5854	3.3741	0.8986	H	-2.6383	-2.5186	-4.5961
H	7.4396	4.3117	2.5294	H	-4.0525	-3.6467	-2.8619
H	9.6706	3.2301	2.8673	N	-5.1309	-2.2445	-0.6833
C	4.7037	2.8087	-0.0535	O	-5.8803	-1.3143	1.2716
C	4.8790	1.4311	-0.3512	C	-3.7063	0.6883	0.6532
H	5.5050	4.4386	1.1268	C	-4.2614	0.7497	2.0553
C	6.0578	-0.7582	-0.2324	C	-2.3761	-0.0579	0.9006
C	7.2286	-1.2402	-0.8486	C	-5.5331	1.3576	2.5298
C	4.9743	-1.6328	-0.1040	H	-6.3794	0.7179	2.2534
C	8.4275	-0.3930	-1.1642	H	-5.4987	1.4298	3.6244
C	7.2827	-2.5738	-1.2903	H	-5.6922	2.3562	2.0979
C	4.9893	-2.9642	-0.5655	O	-1.4626	-0.3683	0.0768
C	9.7530	-1.0713	-0.7846	N	-3.5077	0.1437	2.8869
H	8.3535	0.6049	-0.7114	N	-2.3821	-0.3070	2.2247
C	8.5673	-3.0231	-1.9220	C	-1.4293	-0.9153	3.0804
C	6.1776	-3.4105	-1.1489	C	-1.8726	-1.8861	3.9849
C	9.7601	-2.5834	-1.0706	C	-0.0908	-0.5493	3.0020
H	10.5646	-0.5664	-1.3292	C	-0.9351	-2.4909	4.8187
H	8.5683	-4.1134	-2.0622	H	-2.9329	-2.1395	4.0324
H	6.2328	-4.4405	-1.5114	C	0.8432	-1.1888	3.8202
H	10.6966	-2.8785	-1.5656	H	0.2293	0.2192	2.2939
H	7.2779	2.8478	3.5057	C	0.4197	-2.1454	4.7409
H	9.2127	3.2549	1.1665	H	-1.2614	-3.2437	5.5395
H	9.7621	1.0144	1.2089	H	1.8946	-0.9045	3.6789
H	7.3567	0.4284	2.9623	H	1.1392	-2.6429	5.3950
H	8.4195	-0.2227	-2.2556	C	-3.7937	-6.6164	1.3997
H	9.9560	-0.9010	0.2836	C	-4.2931	-5.5398	2.1395
H	9.7232	-3.1422	-0.1225	C	-4.0098	-6.6868	0.0238
H	8.6499	-2.5774	-2.9302	C	-5.0034	-4.5045	1.5280
C	3.7224	3.6689	-0.6907	H	-4.1492	-5.5275	3.2229
C	3.2363	4.7784	0.0412	C	-4.6962	-5.6492	-0.6025
C	3.2418	3.4776	-2.0179	H	-3.6396	-7.5360	-0.5545
C	2.3091	5.6746	-0.4948	C	-5.1721	-4.5556	0.1301
H	3.5630	4.9238	1.0711	H	-5.4390	-3.7106	2.1921
C	2.3586	4.3717	-2.5731	H	-4.8726	-5.6824	-1.6820
H	3.6051	2.6381	-2.6070	C	-5.9233	-3.4513	-0.5651
C	1.7987	6.7571	0.2721	H	-6.2753	-3.7170	-1.5713
C	1.8581	5.4869	-1.8378	H	-6.7991	-3.2032	0.0502
H	2.0259	4.2393	-3.6052	H	-3.2554	-7.4190	1.9098
C	0.8689	7.6231	-0.2753	C	-0.3310	4.2015	1.5598
H	2.1433	6.8917	1.3002	C	-0.7915	4.5702	2.8201
C	0.9285	6.3851	-2.3731	C	-2.1174	4.3248	3.1695
C	0.4376	7.4395	-1.5986	C	-2.9508	3.6577	2.2781
H	0.4705	8.4475	0.3178	C	-2.4809	3.2357	1.0281
H	0.5850	6.2515	-3.4013	C	-1.1739	3.5683	0.6371
H	-0.2911	8.1296	-2.0289	H	0.7008	4.4284	1.2772
C	3.7131	-3.7206	-0.5376	H	-0.1203	5.0648	3.5255
C	2.9598	-3.6636	0.6151	H	-2.5013	4.6338	4.1441
C	3.1066	-4.2566	-1.7085	H	-3.9801	3.4546	2.5711

C	1.5684	-3.8782	0.5852	C	-0.7178	3.3435	-0.7674
H	3.4032	-3.2772	1.5337	H	-0.9822	2.3504	-1.1657
C	1.7556	-4.5331	-1.7514	H	-1.1924	4.0816	-1.4333
H	3.7054	-4.3651	-2.6157	H	0.3639	3.4770	-0.8353
C	0.7926	-3.5324	1.7054	C	-3.3465	2.4277	0.0920
C	0.9214	-4.2454	-0.6341	C	-4.6073	3.0835	-0.3134
H	1.2998	-4.8785	-2.6822	H	-2.7674	2.2032	-0.8115
C	-0.5688	-3.3704	1.6306	C	-5.7230	2.4477	-0.7030
H	1.3068	-3.2738	2.6267	H	-4.5654	4.1757	-0.3746
C	-0.5050	-4.1425	-0.6639	C	-5.9197	0.9627	-0.6149
C	-1.2237	-3.6309	0.4201	H	-6.4029	0.5945	-1.5308
H	-1.0662	-2.9675	2.5011	H	-6.6150	0.7639	0.2096
H	-1.0612	-4.4072	-1.5705	N	-6.7488	3.2233	-1.2689
H	-2.2731	-3.5021	0.2460	H	-6.4382	4.0647	-1.7489
O	3.9986	0.8168	-1.1992	C	-8.1124	3.1442	-1.3184
O	3.7926	-1.1051	0.4196	C	-8.8512	2.0840	-0.4701
P	2.9371	-0.3062	-0.6933	O	-8.7710	3.9099	-1.9783
O	2.5178	-1.1387	-1.8912	F	-10.1163	2.4170	-0.3548
O	1.6931	0.2515	0.0039	F	-8.3287	1.9877	0.7595
Bi	0.2493	-1.2638	-1.1307	F	-8.7827	0.8793	-1.0438
C	-4.3202	-1.8276	-1.7397				

Bi-4aa:

C	-9.7326	0.3607	0.0758	C	5.1163	-1.2541	0.1421
C	-8.4088	0.0590	-0.6472	C	4.3076	-2.1739	-0.5029
C	-7.2252	0.7375	-0.0219	C	4.7530	-3.4917	-0.6548
C	-7.3758	2.1161	0.2284	C	6.0046	-3.8656	-0.1657
C	-8.7408	2.7003	0.0100	C	6.8434	-2.9429	0.4663
C	-9.7960	1.7891	0.6388	C	6.2809	0.5967	1.0807
H	-10.5534	0.1904	-0.6369	C	4.9069	0.2074	0.4681
H	-8.2561	-1.0217	-0.7527	H	3.3457	-1.8608	-0.9089
C	-5.9870	0.1142	0.2228	H	4.1305	-4.2289	-1.1657
C	-6.2923	2.8804	0.6468	H	6.3484	-4.8944	-0.2912
H	-8.7944	3.7150	0.4291	H	7.8331	-3.2346	0.8175
H	-10.7991	2.2161	0.4952	N	7.0421	-0.5391	1.1602
C	-5.0310	2.3080	0.8482	O	6.5908	1.7012	1.4557
C	-4.9346	0.9260	0.6778	C	4.5325	1.0678	-0.6907
H	-6.4166	3.9547	0.8030	C	5.1927	1.6083	-1.8412
C	-5.7211	-1.3118	-0.1164	C	3.2359	1.4511	-0.8612
C	-6.4312	-2.4072	0.4077	C	6.6380	1.5480	-2.1750
C	-4.6674	-1.5881	-0.9927	H	6.9621	0.5077	-2.3378
C	-7.4951	-2.3019	1.4595	H	6.8366	2.1259	-3.0856
C	-6.1003	-3.7183	-0.0021	H	7.2382	1.9648	-1.3526
C	-4.2665	-2.8770	-1.3566	O	2.1675	1.1234	-0.1080
C	-8.7423	-3.1416	1.1382	N	4.3293	2.2220	-2.6314
H	-7.7621	-1.2582	1.6661	N	3.1204	2.1366	-2.0284
C	-6.9254	-4.8355	0.5649	C	1.9390	2.4763	-2.6964
C	-5.0372	-3.9404	-0.8701	C	1.9418	2.5738	-4.0884
C	-8.4130	-4.4910	0.4758	C	0.7455	2.7038	-1.9761
H	-9.2985	-3.2947	2.0746	C	0.7582	2.8787	-4.7599
H	-6.7070	-5.7790	0.0446	H	2.8810	2.4248	-4.6223
H	-4.7793	-4.9649	-1.1497	C	-0.4369	2.9939	-2.6777

H	-9.0074	-5.2983	0.9274	H	0.7793	2.8698	-0.8969
H	-8.9327	2.7969	-1.0744	C	-0.4401	3.0634	-4.0662
H	-9.6193	1.7713	1.7259	H	0.7747	2.9637	-5.8488
H	-9.8842	-0.3629	0.8910	H	-1.3379	3.1686	-2.0946
H	-8.4808	0.4472	-1.6791	H	-1.3605	3.2967	-4.6044
H	-7.0478	-2.6854	2.3943	C	11.4913	-1.6684	-1.0004
H	-9.4097	-2.5646	0.4813	C	10.5140	-0.7480	-1.3802
H	-8.6897	-4.4629	-0.5894	C	11.4485	-2.2342	0.2724
H	-6.6461	-4.9901	1.6234	C	9.5017	-0.3973	-0.4911
C	-3.8080	3.1027	1.0770	H	10.5440	-0.2968	-2.3748
C	-3.4850	4.1192	0.1960	C	10.4299	-1.8853	1.1591
C	-2.9089	2.7867	2.1286	H	12.2099	-2.9556	0.5777
C	-2.2569	4.8171	0.2923	C	9.4490	-0.9629	0.7861
H	-4.1654	4.3631	-0.6236	H	8.7414	0.3254	-0.7915
C	-1.7164	3.4565	2.2614	H	10.3976	-2.3374	2.1546
H	-3.1812	1.9917	2.8242	C	8.3610	-0.5693	1.7554
C	-1.8920	5.8290	-0.6335	H	8.3528	-1.2560	2.6169
C	-1.3436	4.4705	1.3345	H	8.5323	0.4461	2.1450
H	-1.0359	3.2292	3.0844	H	12.2865	-1.9433	-1.6971
C	-0.6694	6.4559	-0.5409	C	2.3876	0.6667	1.2601
H	-2.5964	6.1038	-1.4226	C	3.8868	0.4692	1.6050
C	-0.0961	5.1406	1.4126	H	3.9640	-0.3351	2.3462
C	0.2366	6.1063	0.4859	H	4.2202	1.4020	2.0777
H	-0.3972	7.2318	-1.2595	C	1.5844	-0.6153	1.2792
H	0.5855	4.8855	2.2281	C	0.5179	-0.8720	2.0906
H	1.1984	6.6197	0.5538	H	2.0682	-1.4316	0.7298
C	-2.9386	-3.0386	-1.9713	C	-0.1420	-2.1456	2.2709
C	-2.4306	-2.1410	-2.8962	H	0.1197	-0.0342	2.6713
C	-2.0678	-4.0256	-1.4238	C	0.4196	-3.3221	1.7434
C	-1.0353	-2.0245	-3.0860	C	-1.3648	-2.2063	2.9834
H	-3.0863	-1.4335	-3.4039	C	-0.2119	-4.5457	1.8920
C	-0.7198	-3.9943	-1.6531	H	1.3783	-3.2727	1.2199
H	-2.4760	-4.7749	-0.7432	C	-1.9961	-3.4460	3.1019
C	-0.4578	-0.9447	-3.8308	C	-1.9741	-0.9889	3.6066
C	-0.1498	-2.9441	-2.4192	C	-1.4349	-4.6033	2.5653
H	-0.0678	-4.7351	-1.1899	H	0.2453	-5.4547	1.4958
C	0.9214	-0.8179	-3.9448	H	-2.9457	-3.5034	3.6387
H	-1.1157	-0.2382	-4.3455	H	-1.3217	-0.5701	4.3895
C	1.2538	-2.7650	-2.5424	H	-2.1518	-0.2028	2.8602
C	1.7812	-1.7251	-3.2893	H	-2.9363	-1.2309	4.0747
H	1.3386	-0.0011	-4.5351	H	-1.9468	-5.5606	2.6879
H	1.9112	-3.4862	-2.0489	N	1.8188	1.7422	2.0559
H	2.8633	-1.6083	-3.3829	H	1.2093	2.3733	1.5396
O	-3.6627	0.3416	0.8770	C	1.7889	2.0405	3.3883
O	-3.8889	-0.5168	-1.4435	C	2.5684	1.1886	4.4250
P	-2.7928	-0.0103	-0.3988	O	1.1382	2.9681	3.8029
O	-1.7052	-1.0385	-0.0176	F	2.0314	1.3652	5.6101
O	-1.9813	1.1193	-1.0881	F	3.8431	1.5591	4.4694
Bi	-0.0254	-0.0317	-1.1885	F	2.5191	-0.1188	4.1373
C	6.3827	-1.6382	0.6045				

Bi-3aa:

C	9.1083	1.8487	1.7160	80	-3.9696	-0.4148	-1.5174
C	7.7985	1.0390	1.7004	81	-3.3456	0.3242	-2.5131
C	6.7250	1.6482	0.8634	82	-2.7924	-0.3359	-3.6166
C	6.5000	3.0363	1.0693	83	-2.8908	-1.7214	-3.7322
C	7.4520	3.7584	1.9728	84	-3.5534	-2.4782	-2.7608
C	8.8892	3.3669	1.6297	85	-5.1223	-1.3863	0.3165
H	9.6506	1.5860	2.6355	86	-4.6747	-0.0164	-0.2375
H	7.9778	-0.0112	1.4401	87	-3.2958	1.4118	-2.4572
C	5.8933	0.9095	-0.0111	88	-2.2954	0.2418	-4.3986
C	5.4448	3.6620	0.4380	89	-2.4633	-2.2253	-4.6018
H	7.3034	4.8450	1.8957	90	-3.6511	-3.5597	-2.8598
H	9.5892	3.8955	2.2922	91	-4.8189	-2.3539	-0.6079
C	4.5643	2.9576	-0.4156	92	-5.6985	-1.5744	1.3638
C	4.8106	1.5723	-0.5976	93	-3.8005	0.7362	0.7993
H	5.3106	4.7366	0.5718	94	-4.3576	0.7791	2.2114
C	6.0889	-0.5465	-0.2570	95	-2.4711	0.0650	1.0686
C	7.2593	-1.0891	-0.8180	96	-5.6890	1.2525	2.6575
C	5.0188	-1.4098	0.0123	97	-6.4283	0.4557	2.4879
C	8.4413	-0.2841	-1.2743	98	-5.6530	1.4670	3.7330
C	7.3277	-2.4733	-1.0670	99	-6.0123	2.1458	2.1086
C	5.0459	-2.7800	-0.2704	100	-1.6331	-0.2316	0.1953
C	9.7811	-0.8877	-0.8232	101	-3.5042	0.3490	3.0693
H	8.3649	0.7700	-0.9750	102	-2.3578	-0.0461	2.3922
C	8.6070	-2.9945	-1.6515	103	-1.2309	-0.4714	3.1344
C	6.2337	-3.2936	-0.7973	104	-1.4220	-1.2703	4.2608
C	9.8056	-2.4253	-0.8906	105	0.0427	-0.0559	2.7459
H	10.5773	-0.4578	-1.4488	106	-0.3115	-1.6820	4.9918
H	8.6172	-4.0937	-1.6367	107	-2.4345	-1.5505	4.5564
H	6.2912	-4.3611	-1.0263	108	1.1445	-0.4907	3.4766
H	10.7389	-2.7761	-1.3542	109	0.1807	0.6126	1.8957
H	7.2316	3.4840	3.0205	110	0.9717	-1.3018	4.5972
H	9.1019	3.7203	0.6084	111	-0.4504	-2.3045	5.8782
H	9.7454	1.5195	0.8829	112	2.1440	-0.1775	3.1679
H	7.3910	1.0250	2.7298	113	1.8378	-1.6311	5.1754
H	8.4142	-0.2784	-2.3789	114	-3.1948	-7.1167	0.5012
H	9.9970	-0.5645	0.2064	115	-3.4977	-6.1358	1.4481
H	9.7898	-2.8438	0.1277	116	-3.6188	-6.9636	-0.8189
H	8.6693	-2.6940	-2.7135	117	-4.2178	-5.0035	1.0766
C	3.4644	3.6498	-1.0614	118	-3.1655	-6.2530	2.4822
C	2.8700	4.7546	-0.3974	119	-4.3440	-5.8304	-1.1885
C	2.9490	3.2749	-2.3329	120	-3.3910	-7.7312	-1.5621
C	1.7767	5.4427	-0.9380	121	-4.6455	-4.8409	-0.2466
H	3.2276	5.0434	0.5923	122	-4.4244	-4.2122	1.8056
C	1.9168	3.9761	-2.9031	123	-4.6962	-5.7253	-2.2194
H	3.3964	2.4416	-2.8705	124	-5.4833	-3.6484	-0.6244
C	1.1332	6.4918	-0.2241	125	-5.9101	-3.7868	-1.6291
C	1.2882	5.0648	-2.2281	126	-6.3181	-3.5578	0.0862
H	1.5549	3.6985	-3.8967	127	-2.6324	-8.0055	0.7960
C	0.0487	7.1501	-0.7780	128	-0.4031	3.8094	1.8347
H	1.5067	6.7644	0.7669	129	-0.9387	4.3067	3.0192
C	0.1992	5.7573	-2.7699	130	-2.3045	4.1854	3.2608
C	-0.4138	6.7858	-2.0507	131	-3.1041	3.5203	2.3372

H	-0.4478	7.9502	-0.2261	132	-2.5669	2.9706	1.1682
H	-0.1762	5.4864	-3.7584	133	-1.1990	3.1581	0.8820
H	-1.2655	7.3129	-2.4860	134	0.6643	3.9304	1.6342
C	3.7788	-3.5430	-0.2088	135	-0.2916	4.8027	3.7458
C	2.9744	-3.5201	0.9113	136	-2.7490	4.5941	4.1705
C	3.2673	-4.1078	-1.4113	137	-4.1743	3.4324	2.5323
C	1.5989	-3.8414	0.8226	138	-0.5881	2.7452	-0.4285
H	3.3587	-3.1366	1.8594	139	-0.8311	1.7123	-0.7007
C	1.9388	-4.4346	-1.5303	140	-0.9449	3.3930	-1.2472
H	3.9246	-4.1850	-2.2802	141	0.5050	2.8114	-0.3939
C	0.7287	-3.6664	1.9297	142	-3.4352	2.1527	0.2299
C	1.0449	-4.2113	-0.4485	143	-4.6469	2.8606	-0.2834
H	1.5395	-4.7861	-2.4843	144	-2.7978	1.9270	-0.6381
C	-0.6365	-3.6769	1.7553	145	-5.7808	2.2481	-0.6588
H	1.1584	-3.4464	2.9089	146	-4.5629	3.9459	-0.3883
C	-0.3765	-4.2228	-0.5964	147	-5.9768	0.7646	-0.5152
C	-1.1973	-3.8930	0.4741	148	-6.4248	0.3670	-1.4384
H	-1.2973	-3.4960	2.6037	149	-6.6930	0.5472	0.2884
H	-0.8104	-4.4824	-1.5659	150	-6.7773	3.0091	-1.2988
H	-2.2792	-3.8304	0.3707	151	-6.4365	3.8042	-1.8339
O	3.9100	0.8619	-1.3467	152	-8.1401	2.9619	-1.3463
O	3.8473	-0.8308	0.4921	153	-8.8913	1.9636	-0.4353
P	2.8973	-0.2049	-0.6525	154	-8.7828	3.6915	-2.0606
O	2.4382	-1.2026	-1.6970	155	-10.1521	2.3192	-0.3315
O	1.6745	0.4245	0.0257	156	-8.3585	1.9385	0.7948
Bi7	0.2029	-1.2935	-0.7361	157	-8.8412	0.7240	-0.9289
C	-4.0874	-1.8078	-1.6667				

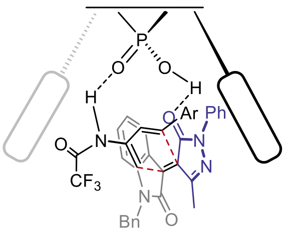

Concerted [4+2]-C4-TS3:

C	8.0005	-3.4823	-0.8848	C	-2.8644	0.8861	-1.5262
C	7.0405	-2.3132	-1.1641	C	-1.6412	0.9554	-2.1728
C	5.5962	-2.6761	-0.9577	C	-1.4990	0.3530	-3.4299
C	5.1832	-3.8788	-1.5504	C	-2.5827	-0.2940	-4.0260
C	6.2258	-4.6975	-2.2543	C	-3.8437	-0.3062	-3.4181
C	7.4649	-4.8446	-1.3683	C	-4.8611	1.4976	-0.4526
H	8.9664	-3.2522	-1.3652	C	-3.3779	1.3510	-0.2180
H	7.3283	-1.4279	-0.5785	H	-0.8128	1.5059	-1.7195
C	4.6548	-1.8663	-0.3001	H	-0.5348	0.3963	-3.9425
C	3.8546	-4.2658	-1.4603	H	-2.4564	-0.7769	-4.9991
H	5.8209	-5.6854	-2.5293	H	-4.7024	-0.7668	-3.9113
H	8.2487	-5.4012	-1.9092	N	-5.1351	0.6024	-1.4646
C	2.8737	-3.4648	-0.8570	O	-5.6711	2.1815	0.1248
C	3.2968	-2.2447	-0.3025	C	-3.0881	0.4916	0.9692
H	3.5418	-5.2115	-1.9133	C	-4.1827	-0.2201	1.7057
C	5.0637	-0.5975	0.3575	C	-2.0987	-0.6446	0.7201

C	5.9869	-0.5364	1.4157	C	-5.5005	0.2674	2.2092
C	4.4750	0.5940	-0.0845	H	-6.2722	0.2438	1.4231
C	6.6019	-1.7347	2.0823	H	-5.8196	-0.3977	3.0264
C	6.3215	0.7112	1.9700	H	-5.4445	1.3028	2.5717
C	4.8132	1.8511	0.4289	O	-1.0551	-0.6299	0.0997
C	8.1132	-1.5760	2.3186	N	-3.8781	-1.4524	1.9067
H	6.3911	-2.6619	1.5290	N	-2.6397	-1.7186	1.3789
C	7.3028	0.7126	3.1069	C	-2.0106	-2.9212	1.7709
C	5.7613	1.8777	1.4585	C	-2.8095	-4.0057	2.1405
C	8.5161	-0.1562	2.7648	C	-0.6191	-2.9918	1.8945
H	8.4227	-2.3227	3.0693	C	-2.2155	-5.1562	2.6523
H	7.6154	1.7421	3.3469	H	-3.8933	-3.9267	2.0452
H	6.0575	2.8444	1.8769	C	-0.0413	-4.1485	2.4084
H	9.1979	-0.1991	3.6308	H	0.0077	-2.1532	1.5927
H	6.5088	-4.1982	-3.2019	C	-0.8313	-5.2314	2.7959
H	7.1849	-5.4649	-0.4994	H	-2.8466	-6.0011	2.9438
H	8.2038	-3.5351	0.1972	H	1.0470	-4.1970	2.5049
H	7.1495	-2.0182	-2.2258	H	-0.3668	-6.1353	3.2005
H	6.1053	-1.8509	3.0651	C	-7.4530	-3.8969	-1.8124
H	8.6538	-1.8335	1.3932	C	-6.7247	-3.3182	-0.7725
H	9.0722	0.3460	1.9548	C	-7.8445	-3.1132	-2.8977
H	6.8104	0.3128	4.0152	C	-6.3914	-1.9662	-0.8178
C	1.4930	-4.0044	-0.8390	H	-6.4007	-3.9235	0.0793
C	0.4081	-3.3013	-1.3186	C	-7.5134	-1.7586	-2.9378
C	1.2910	-5.3388	-0.3858	H	-8.4078	-3.5592	-3.7228
C	-0.8910	-3.8635	-1.3462	C	-6.7888	-1.1704	-1.8967
H	0.5511	-2.2987	-1.7164	H	-5.7987	-1.5348	-0.0072
C	0.0446	-5.9153	-0.4067	H	-7.8203	-1.1495	-3.7949
H	2.1451	-5.9022	0.0014	C	-6.4642	0.3056	-1.9340
C	-2.0096	-3.1323	-1.8228	H	-6.5909	0.6935	-2.9612
C	-1.0839	-5.1979	-0.8788	H	-7.1492	0.8769	-1.2845
H	-0.0939	-6.9377	-0.0423	H	-7.7105	-4.9598	-1.7804
C	-3.2652	-3.6938	-1.8277	C	-3.8259	1.5630	5.6528
H	-1.8621	-2.1089	-2.1774	C	-4.3771	2.8384	5.5753
C	-2.3894	-5.7510	-0.8973	C	-4.1969	3.5926	4.4181
C	-3.4573	-5.0154	-1.3595	C	-3.4835	3.0505	3.3543
H	-4.1218	-3.1172	-2.1875	C	-2.9587	1.7534	3.4037
H	-2.5342	-6.7722	-0.5311	C	-3.1079	0.9983	4.5897
H	-4.4625	-5.4475	-1.3664	H	-3.9456	0.9800	6.5716
C	4.2031	3.1009	-0.0685	H	-4.9321	3.2479	6.4246
C	4.0713	3.3482	-1.4228	H	-4.6044	4.6047	4.3419
C	3.7852	4.0989	0.8541	H	-3.3281	3.6596	2.4631
C	3.5392	4.5682	-1.9042	C	-2.5287	-0.3809	4.7670
H	4.3820	2.5908	-2.1474	H	-2.9869	-1.1165	4.0844
C	3.2720	5.2963	0.4154	H	-1.4381	-0.4034	4.5929
H	3.8572	3.8944	1.9262	H	-2.7004	-0.7372	5.7950
C	3.3766	4.8207	-3.2906	C	-2.1250	1.2342	2.2943
C	3.1372	5.5705	-0.9688	C	-1.1699	2.1387	1.6457
H	2.9431	6.0503	1.1377	H	-1.6005	0.3450	2.6219
C	2.8255	6.0020	-3.7286	C	-1.4433	2.8703	0.5639
H	3.6802	4.0492	-4.0054	H	-0.1616	2.1364	2.0642
C	2.5877	6.7841	-1.4520	C	-2.7954	2.9697	-0.0005

C	2.4284	6.9933	-2.8016	H	-2.8751	3.3552	-0.9981
H	2.6910	6.1792	-4.8000	H	-3.5509	3.3751	0.6419
H	2.2683	7.5424	-0.7306	N	-0.3878	3.5135	-0.1111
H	1.9881	7.9273	-3.1634	H	0.4922	2.9890	-0.1458
O	2.3881	-1.3911	0.3153	C	-0.4201	4.5959	-0.9320
O	3.4439	0.4856	-1.0164	C	-1.5032	5.6730	-0.6795
P	2.0495	0.0741	-0.3069	O	0.3939	4.8267	-1.7921
O	1.5230	1.0283	0.6882	F	-0.9328	6.8688	-0.7311
O	1.1486	-0.1877	-1.5925	F	-2.1033	5.5619	0.5082
H	0.2071	-0.3043	-1.3206	F	-2.4462	5.6272	-1.6229
C	-3.9640	0.3144	-2.1799				

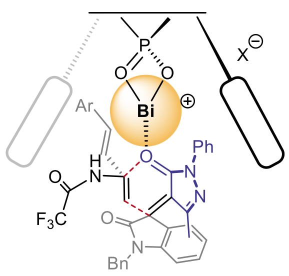

Concerted [2+4]-Bi-TS3:

C	-9.5478	0.2554	-0.0985	C	4.9176	-1.3915	0.2099
C	-8.2095	-0.0347	-0.7985	C	4.1499	-2.3706	-0.3984
C	-7.0487	0.6891	-0.1802	C	4.6548	-3.6682	-0.5273
C	-7.2352	2.0694	0.0284	C	5.9261	-3.9803	-0.0499
C	-8.6107	2.6156	-0.2205	C	6.7242	-3.0066	0.5539
C	-9.6508	1.6967	0.4231	C	6.0454	0.5082	1.1140
H	-10.3565	0.0449	-0.8143	C	4.7178	0.0687	0.4596
H	-8.0296	-1.1139	-0.8724	H	3.1758	-2.1561	-0.8080
C	-5.7999	0.1011	0.0974	H	4.0583	-4.4392	-1.0209
C	-6.1763	2.8689	0.4469	H	6.3144	-4.9947	-0.1610
H	-8.6931	3.6393	0.1719	H	7.7298	-3.2425	0.9025
H	-10.6622	2.0955	0.2580	N	6.8401	-0.5990	1.2025
C	-4.9087	2.3321	0.6909	O	6.3185	1.6290	1.4715
C	-4.7707	0.9481	0.5412	C	4.2916	0.9581	-0.6431
H	-6.3292	3.9420	0.5855	C	5.0031	1.5175	-1.7598
C	-5.5081	-1.3340	-0.1724	C	3.0036	1.3842	-0.8417
C	-6.2306	-2.3867	0.4228	C	6.4548	1.4345	-2.0545
C	-4.4375	-1.6568	-1.0212	H	6.7638	0.3956	-2.2471
C	-7.3087	-2.2135	1.4527	H	6.6896	2.0418	-2.9369
C	-5.9026	-3.7206	0.1151	H	7.0378	1.8106	-1.2007
C	-4.0403	-2.9795	-1.2675	O	1.9448	1.1045	-0.1340
C	-8.5516	-3.0776	1.1748	N	4.1911	2.1894	-2.5514
H	-7.5825	-1.1598	1.5854	N	2.9658	2.1383	-1.9824
C	-6.7303	-4.8007	0.7440	C	1.8315	2.6009	-2.6480
C	-4.8295	-3.9954	-0.7165	C	1.8708	2.7977	-4.0333
C	-8.2157	-4.4694	0.6109	C	0.6365	2.8744	-1.9307
H	-9.1191	-3.1666	2.1128	C	0.7395	3.2644	-4.7002
H	-6.4996	-5.7761	0.2918	H	2.8078	2.6163	-4.5597
H	-4.5658	-5.0361	-0.9163	C	-0.5043	3.2949	-2.6518
H	-8.8146	-5.2458	1.1088	H	0.6552	3.0013	-0.8337
H	-8.7931	2.6792	-1.3088	C	-0.4642	3.4913	-4.0194

H	-9.4851	1.7127	1.5121
H	-9.6911	-0.4479	0.7355
H	-8.2792	0.3230	-1.8413
H	-6.8727	-2.5317	2.4170
H	-9.2119	-2.5499	0.4713
H	-8.4789	-4.5147	-0.4572
H	-6.4667	-4.8819	1.8146
C	-3.7198	3.1670	0.9735
C	-3.3309	4.1678	0.1067
C	-2.9131	2.8900	2.1095
C	-2.1122	4.8708	0.2923
H	-3.9408	4.3917	-0.7725
C	-1.7362	3.5631	2.3262
H	-3.2590	2.1217	2.8041
C	-1.6777	5.8656	-0.6244
C	-1.2762	4.5514	1.4107
H	-1.1153	3.3397	3.1968
C	-0.4648	6.4943	-0.4520
H	-2.3189	6.1246	-1.4707
C	-0.0385	5.2236	1.5686
C	0.3603	6.1693	0.6493
H	-0.1389	7.2555	-1.1640
H	0.5787	4.9860	2.4381
H	1.3140	6.6863	0.7787
C	-2.7179	-3.3039	-1.8215
C	-2.1040	-2.5614	-2.8038
C	-1.9611	-4.3138	-1.1554
C	-0.7008	-2.6120	-2.9511
H	-2.6779	-1.8478	-3.3954
C	-0.6111	-4.4518	-1.3352
H	-2.4670	-4.9470	-0.4268
C	-0.0415	-1.6608	-3.7803
C	0.0864	-3.5357	-2.1779
H	-0.0588	-5.2112	-0.7738
C	1.3590	-1.6311	-3.8545
H	-0.6582	-0.9963	-4.3899
C	1.4896	-3.4702	-2.2684
C	2.1222	-2.5379	-3.0913
H	1.8599	-0.9056	-4.5025
H	2.1029	-4.1503	-1.6851
H	3.2145	-2.5065	-3.1579
O	-3.4759	0.4073	0.7436
O	-3.7063	-0.5685	-1.5488
P	-2.6297	0.0630	-0.5596
O	-1.4748	-0.8958	-0.2497
O	-1.8916	1.2869	-1.1912
Bi	0.0896	0.2699	-1.4878
C	6.2065	-1.7228	0.6684

H	0.7981	3.4393	-5.7766
H	-1.4046	3.4599	-2.0650
H	-1.3503	3.8374	-4.5539
C	11.3273	-1.5740	-0.9771
C	10.3170	-0.6887	-1.3523
C	11.3058	-2.1447	0.2942
C	9.2954	-0.3787	-0.4593
H	10.3281	-0.2318	-2.3447
C	10.2772	-1.8351	1.1850
H	12.0931	-2.8392	0.5958
C	9.2635	-0.9471	0.8169
H	8.5115	0.3183	-0.7567
H	10.2647	-2.2913	2.1790
C	8.1648	-0.5848	1.7883
H	8.1805	-1.2644	2.6551
H	8.3076	0.4378	2.1701
H	12.1310	-1.8173	-1.6757
C	2.1969	0.4745	1.6480
C	3.5899	0.3726	1.7922
H	3.8363	-0.4869	2.3904
H	4.0578	1.2928	2.1125
C	1.2770	-0.6438	1.4162
C	0.2016	-0.8515	2.2359
H	1.6936	-1.4829	0.8558
C	-0.5479	-2.0800	2.3869
H	-0.1339	-0.0082	2.8464
C	-0.0849	-3.2488	1.7751
C	-1.7589	-2.1286	3.1281
C	-0.7642	-4.4481	1.8589
H	0.8383	-3.1980	1.1977
C	-2.4458	-3.3431	3.1940
C	-2.2938	-0.9133	3.8118
C	-1.9632	-4.4956	2.5707
H	-0.3612	-5.3351	1.3672
H	-3.3846	-3.3853	3.7506
H	-1.6157	-0.5497	4.5997
H	-2.4187	-0.1229	3.0629
H	-3.2664	-1.1121	4.2793
H	-2.5228	-5.4310	2.6468
N	1.6546	1.6830	2.1533
H	1.0570	2.2671	1.5738
C	1.6741	2.0827	3.4657
C	2.4640	1.2600	4.5196
O	1.0720	3.0527	3.8500
F	1.9779	1.5166	5.7113
F	3.7521	1.5829	4.4989
F	2.3562	-0.0577	4.2973

11. Experimental procedures and results of bioassays

Cell culture and reagents

The HepG2, SW-480, MDA-MB-231, MDA-MB-453, BT549, RKO and HCT116 cells utilized in this study were procured from American Type Culture Collection (Manassas, VA, USA), and were cultured in Gibco™ Dulbecco's Modified Eagle Medium (DMEM) or RPMI-1640 supplemented with 10% fetal bovine serum (FBS) and incubated in an incubator maintained at 37 °C with 95% air and 5% CO_2.

The Methylthiazolyldiphenyl-tetrazolium bromide (MTT) was purchased from Solarbio (M8180). The following antibodies were applied to this study: GAPDH (ab8245, Abcam), BCL-2 (ER0602, HuaBio), BAX (ET1603-34, HuaBio), MMP2 (ET1606-4, HuaBio), N-cadherin (ET1607-37, HuaBio), MMP9 (RT1401, HuaBio), E-cadherin (ET1607-75, HuaBio).

Cellular toxicity assay

The cells were seeded in a 96-well plate at a density of 5 × 10^^{3} cells per well and incubated in a humidified cell culture incubator with 5% CO_2 at 37 °C for 24 hours prior to drug treatment. The concentrations of the compounds used were 100, 50, 25, 12.5, 6.25, 3.12, and 1.56 μM. Following treatment, 0.5 mg/mL of MTT solution was added to each well, and the cells were incubated at 37 °C for 4 hours. Subsequently, the medium was carefully aspirated, and dimethyl sulfoxide (DMSO) was added to dissolve the formazan crystals at 37 °C for 10 minutes. Finally, the absorbance of the resulting dye was measured at 490 nm using a microplate reader (BioTek, USA).

Colony formation and cell-migration assays

For the cloning formation experiment, 1000 cells were seeded in a 6-well plate and incubated for 24 h. The cells were then treated continuously with a compound for 2 weeks. After treatment, the medium was aspirated, and the wells were washed three times with PBS. Cells were fixed with 1 mL of methanol for 15 min, followed by discarding the methanol and washing again with PBS three times. Finally, cells were stained with crystal violet solution.

To determine cell migration ability, a wound healing assay was conducted using Incucyte® Live-Cell Analysis. Once cell confluence in a 96-well plate reached 90-100%, scratches were generated using a 96-Well Woundmaker Tool (Sartorius, 4563). Cells were treated with compounds and imaged every 4 h.

Transwell for migration assay

500 μL culture medium with 10% FBS was added in lower chamber. Single cell suspension with 2000 cells was seeded on upper chamber in 200 uL non-serum culture medium for cell migration, after being incubated 24 h, upper chamber cells were fixed with 4% paraformaldehyde for 10 min and stained with 0.1% crystal violet for another 10 min. Cells that passed through membrane were counted under light microscope (Nikon, Ni-U, Japan).

Western blotting (WB) analysis

The western blot assay is conducted under ice-cold conditions. Cells are lysed with RIPA buffer (EA0002) and a protease inhibitor (EA0006), both from Shandong Sparkjade Biotechnology Co., Ltd., then centrifuged at 12,000 rpm for 15 min at 4 °C. Protein concentration is measured using the BCA kit (EC0001-A, Shandong Sparkjade). Next, 30 μg of protein is separated by SDS-PAGE and transferred to PVDF membranes. Membranes are blocked with 5% skim milk, incubated with primary antibodies, followed by HRP-conjugated secondary antibodies. Visualization is achieved using ECL (ED0015-C, Shandong Sparkjade). Protein bands are analyzed densitometrically with ImageJ software.

Immunofluorescence

MDA-MB-231 and MDA-MB-453 cells were cultured in laser dishes and treated with compounds for 24 h. After treatment, cells were fixed in 4% paraformaldehyde for 30 min, permeabilized with 0.5% Triton X-100 for 30 min, and blocked with 5% goat serum for 1 h. The cells were then incubated overnight at 4°C with E-cadherin (ET1607-75, HuaBio) and MMP9 antibodies (RT1401, HuaBio). Following three washes with PBS, cells were incubated with Alexa 546-conjugated anti-mouse (A11030, Invitrogen), Alexa 488-conjugated anti-rabbit (A11034, Invitrogen) antibodies, and DAPI solution (10 mg/mL). After another three washes with PBS, images were captured using an LSM800 microscope (Zeiss) and analyzed with ZEN software.

Statistical Analysis

All the presented data and results were confirmed by at least three independent experiments. The data are expressed as means ± SEM and analyzed with GraphPad Prism 8.0 software. Statistical comparisons were made by one-way ANOVA and Student's t test. $P < 0.05$ was considered statistically significant.

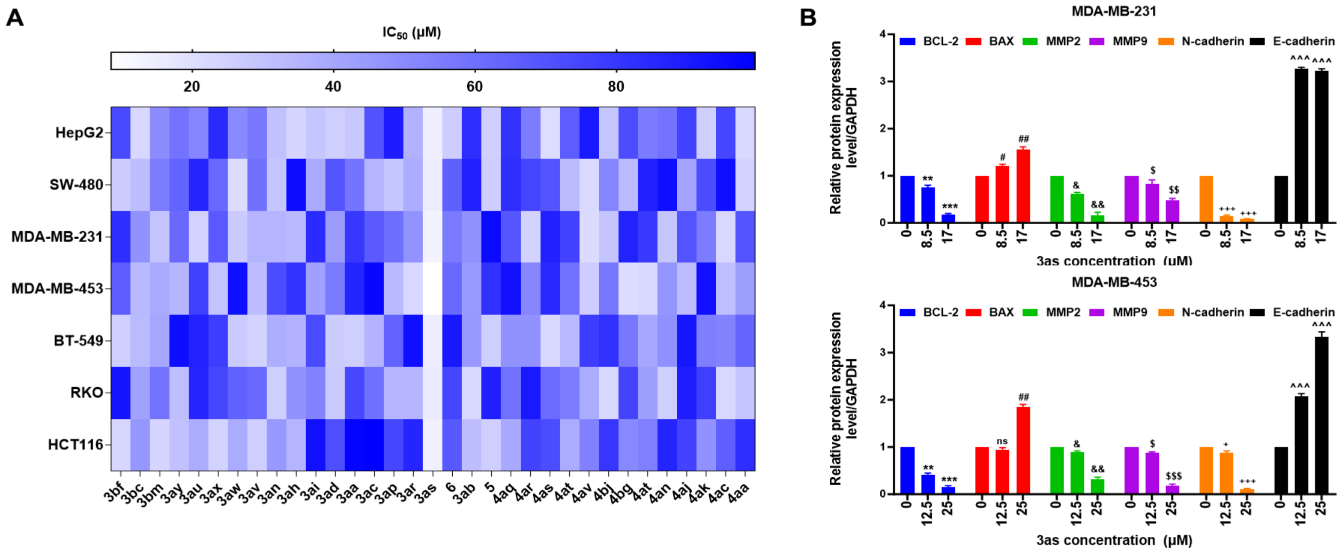

Fig. S2. Evaluation of the antitumor activity of the pyrazole-fused spirooxindole products. (A) The IC_{50} values of the products were determined using the MTT assay. (B) The quantitative analysis of the WB experimental results depicted in Figs. 5I-J.

Table S3. The IC_{50} values of the series of compounds across various tumor cell lines

Compd Num.	IC_{50} (μM)						
	HepG2	SW-480	MDA-MB-231	MDA-MB-453	BT-549	RKO	HCT116
3bf	73.44±1.21	26.49±0.35	83.04±2.11	66.7±1.57	25.93±1.04	92.08±2.07	23.13±1.15
3bc	22.79±2.21	31.96±1.28	47.33±2.34	33.31±1.22	33±0.97	41.93±1.67	45.77±2.54
3bm	49.34±0.27	54.97±2.11	29.43±1.68	38.51±2.87	40.27±1.43	58.14±2.56	23.23±3.01
3ay	58.22±0.45	63.98±2.13	62.03±2.47	34.29±0.76	94.25±2.12	31.24±1.37	30.53±2.50
3au	51.83±1.02	86.56±3.47	23.93±2.01	76.5±2.11	87.33±2.78	86.08±1.09	37.92±1.67
3ax	84.37±3.01	62.12±1.58	67.45±2.26	29.72±1.32	77.66±0.76	74.44±4.12	24.13±3.15
3aw	50.1±2.09	20.67±1.05	27.13±1.12	93.85±1.35	27.41±1.76	64.73±1.41	38.42±1.27
3av	56.62±2.32	59.39±2.12	34.77±1.23	31.89±1.21	24.4±0.68	61.8±2.29	25.49±1.45
3an	30.47±1.24	29.09±1.43	34.78±1.34	72.72±2.05	37.16±1.41	25.36±1.23	45.06±2.55
3ah	23.29±2.13	95.01±1.38	33.48±3.24	81.05±0.56	35.74±1.24	47.27±1.46	34.86±3.27
3ai	28.54±4.31	38.71±3.21	84.05±0.34	42.19±0.42	72.78±3.42	52.67±3.21	93.58±1.56
3ad	25.36±3.28	69.47±3.67	43.58±2.53	55.72±4.45	26.59±0.45	27.38±1.24	71.37±3.21
3aa	28±2.41	58.09±2.56	79.71±0.42	86.69±3.98	25.56±0.34	73.49±0.34	98.14±4.31
3ac	70.18±1.24	34.84±0.43	67.24±1.35	96.61±3.21	35.33±1.57	59.33±1.26	99.55±1.66
3ap	89.41±3.21	28.42±0.56	59.87±3.56	33.47±0.34	64.24±4.32	35.26±3.21	74.47±3.22
3ar	36.09±4.76	37.57±3.21	46.84±3.27	22.44±0.67	93.87±3.21	34.45±2.23	86.24±3.13
3as	14.23±0.32	14.58±1.24	12.50±0.04	8.50±0.07	14.78±0.32	15.57±1.27	13.76±3.21
6	26.21±3.20	64.97±2.34	55.94±3.11	61.74±2.12	90.64±3.24	74.62±3.23	68.07±2.05
3ab	83.02±3.41	87.63±1.23	47.69±0.65	44.86±2.84	44.99±3.21	22±3.29	31.65±1.12

**5**	25.04±2.10	26.29±2.11	95.82±0.56	82.23±3.25	25.97±3.23	87.96±0.45	25.33±0.34
**4aq**	81.21±3.21	83.04±0.45	68.57±0.45	93.34±0.54	47.25±3.22	58.91±2.60	38.96±0.45
**4ar**	55.13±0.34	74.71±2.34	24.21±1.45	51.68±0.31	47.6±0.57	90.36±3.21	75.71±0.78
**4as**	20.58±3.42	69.56±2.34	87.76±4.31	85.19±0.46	22.52±1.13	61.16±2.14	72.13±1.07
**4at**	66.5±2.13	32.57±4.90	75.34±3.24	59.01±4.32	33.35±0.35	55.13±3.24	38.35±4.33
**4av**	90.52±0.32	21.4±0.43	21.11±3.34	26.28±1.34	77.66±4.31	37.8±1.75	53.43±
**4bj**	28.54±3.24	51.96±3.21	29.32±4.30	57.08±0.31	81.91±2.05	22.46±2.18	80.16±2.47
**4bg**	71.76±0.98	33±2.27	87.11±2.15	20.65±3.22	56.59±3.22	75.26±2.09	50.44±3.78
**4at**	55.5±1.23	87.63±2.45	79.19±0.64	21.84±2.31	39.72±3.23	42.96±3.26	53.92±0.45
**4an**	58.31±3.41	94.19±0.34	28.51±1.24	46.11±3.24	47.84±0.53	25.61±0.56	86.45±1.24
**4aj**	76.03±3.22	40.76±1.54	69.62±4.21	32.20±3.21	91.09±3.24	89.11±4.31	75.74±2.34
**4ak**	25.37±1.24	72.4±2.31	55.24±3.12	93.63±4.31	54.28±3.24	76.73±3.13	47.63±1.65
**4ac**	74.54±2.11	93.94±2.12	22.53±1.08	31.18±2.01	52.58±3.21	22.32±2.13	65.52±1.75
**4aa**	22.04±1.08	24±0.53	69.8±1.24	42.81±2.11	63.55±3.56	29.18±1.76	82.84±3.21

Original Western Blot Image

Figure 5I	Figure 5J
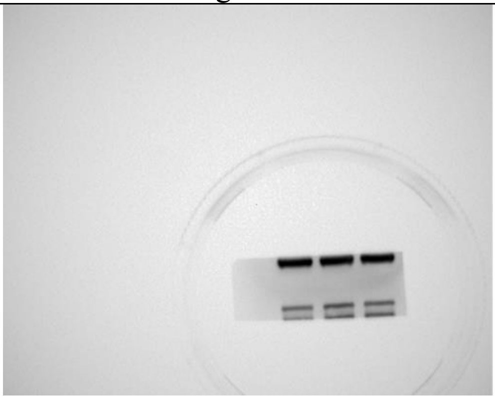<br>GAPDH	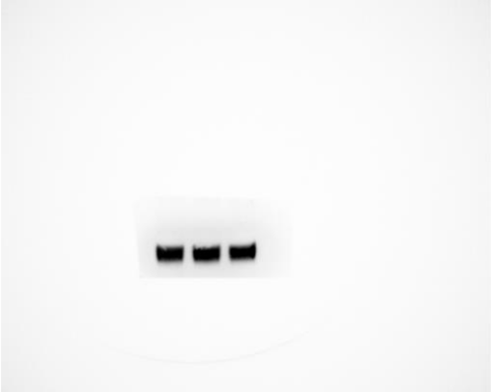<br>GAPDH
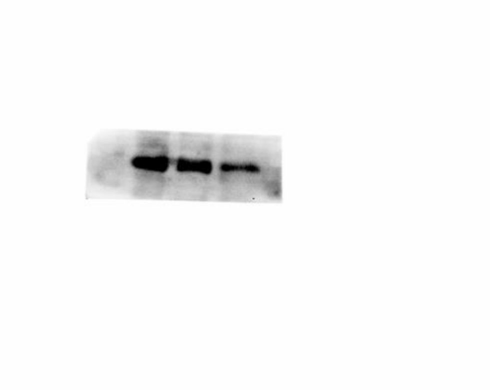<br>BCL-2	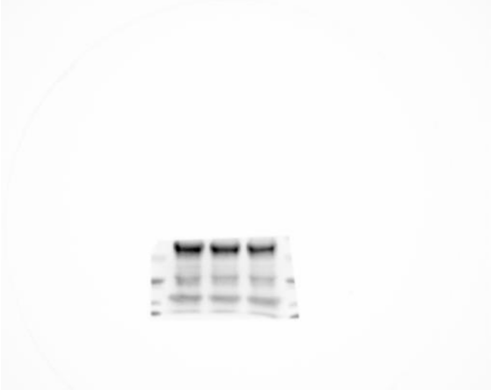<br>BCL-2

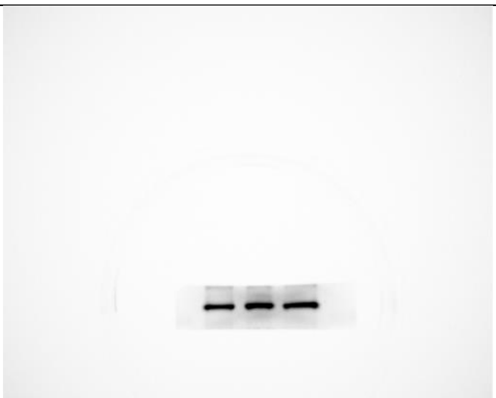<br>BAX	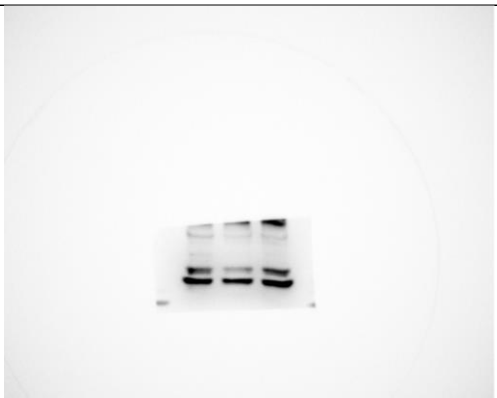<br>BAX
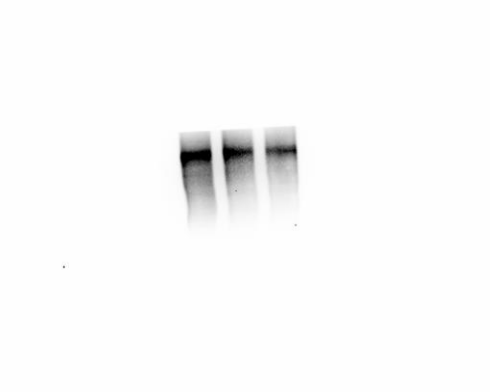<br>MMP2	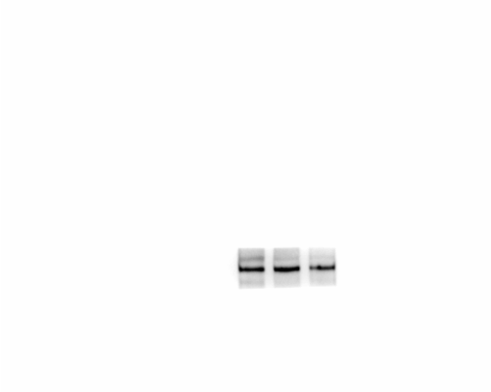<br>MMP2
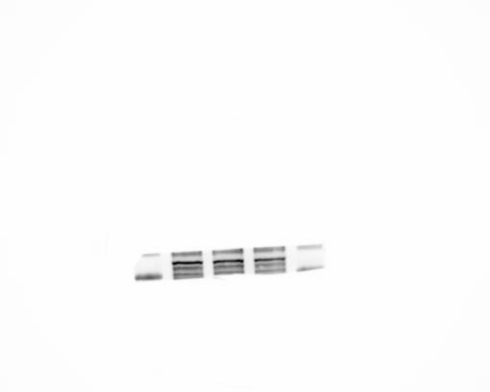<br>MMP9	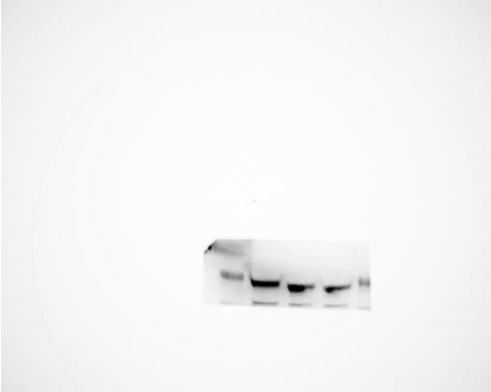<br>MMP9
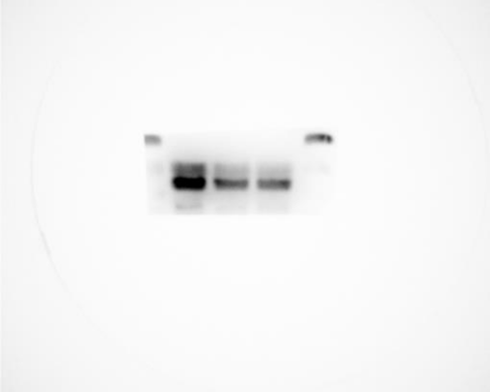<br>N-cadherin	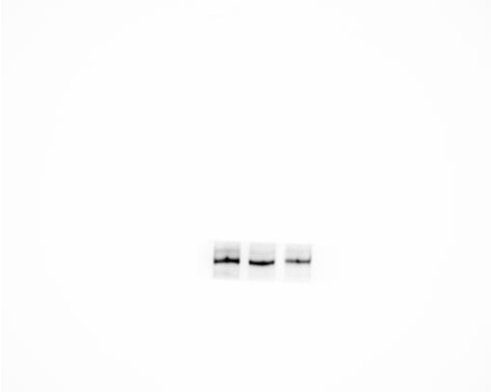<br>N-cadherin

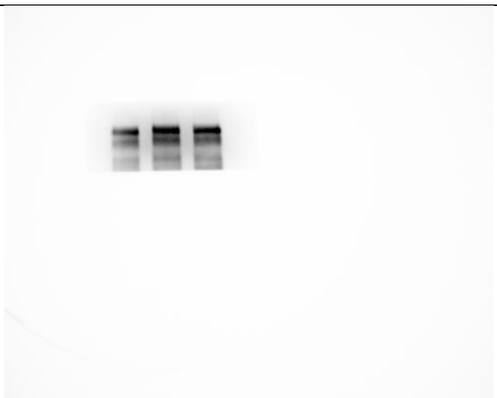 E-cadherin	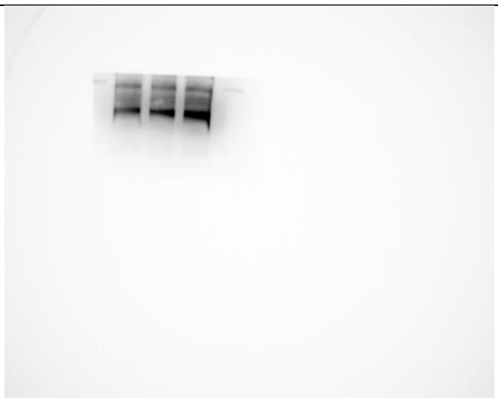 E-cadherin

12. NMR and HPLC spectra

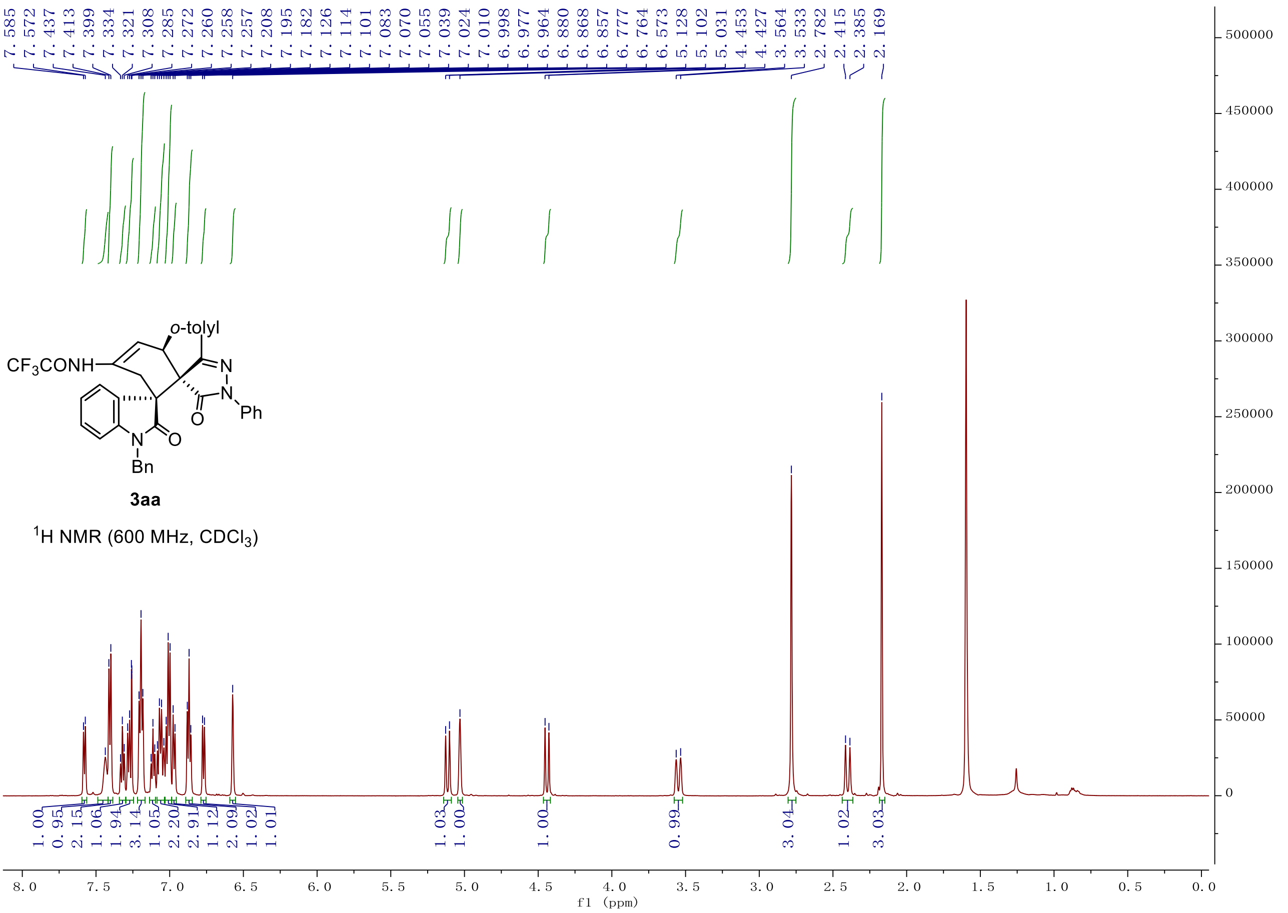

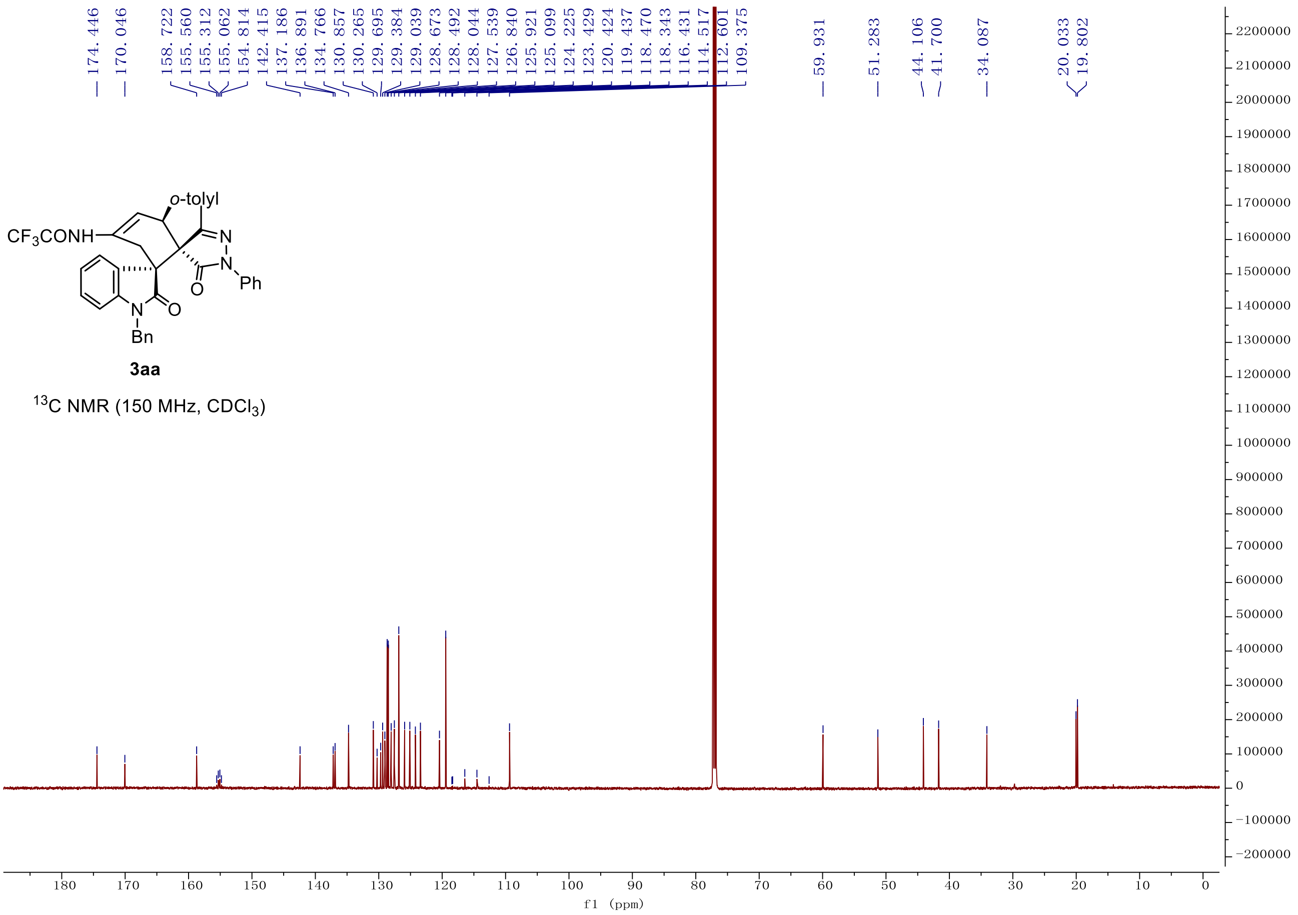

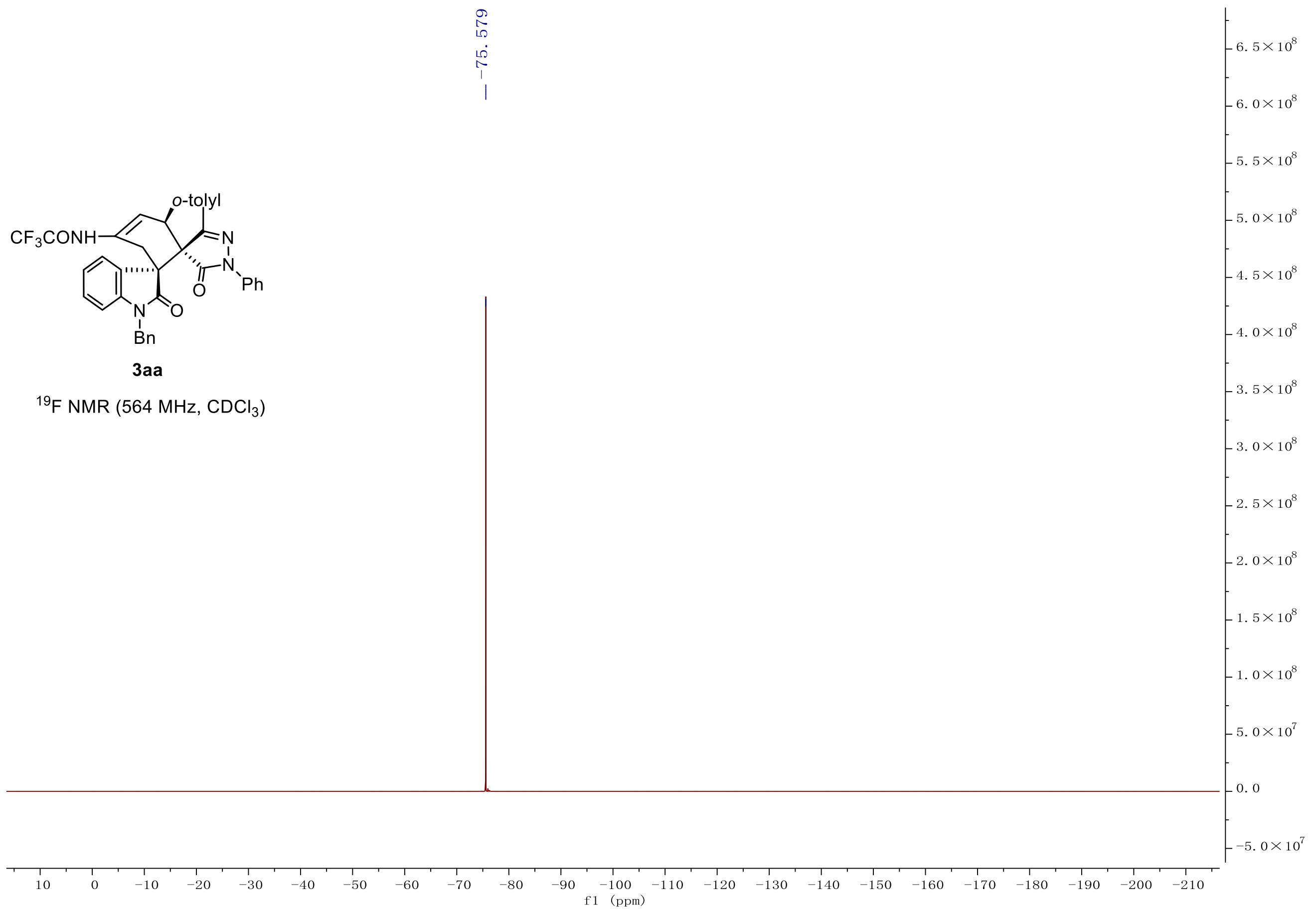

Peak Analysis Report

Detector A Channel 1 254nm

No.	Ret. Time	Height (mAu)	Area (mAu*min)	Rel. Area (%)
1	10.311	331963	9109364	50.283
2	12.532	255407	9006975	49.717
Total		587370	18116340	100.000

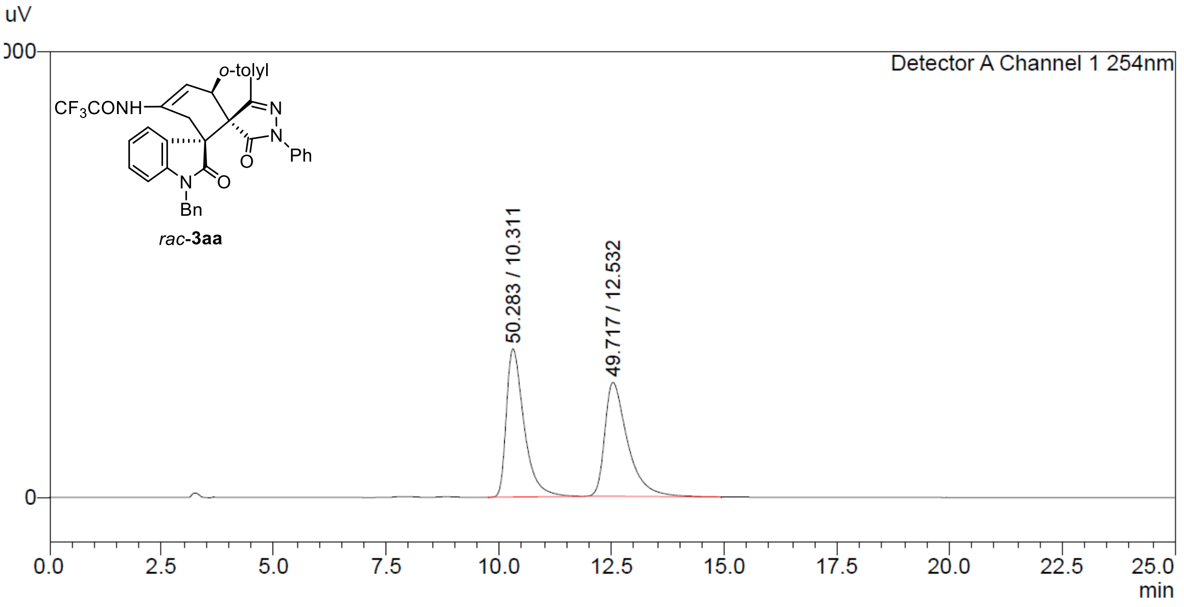

Peak Analysis Report

Detector A Channel 1 254nm

No.	Ret. Time	Height (mAu)	Area (mAu*min)	Rel. Area (%)
1	10.196	339821	9223994	99.714
2	12.611	886	26479	0.286
Total		340707	9250473	100.000

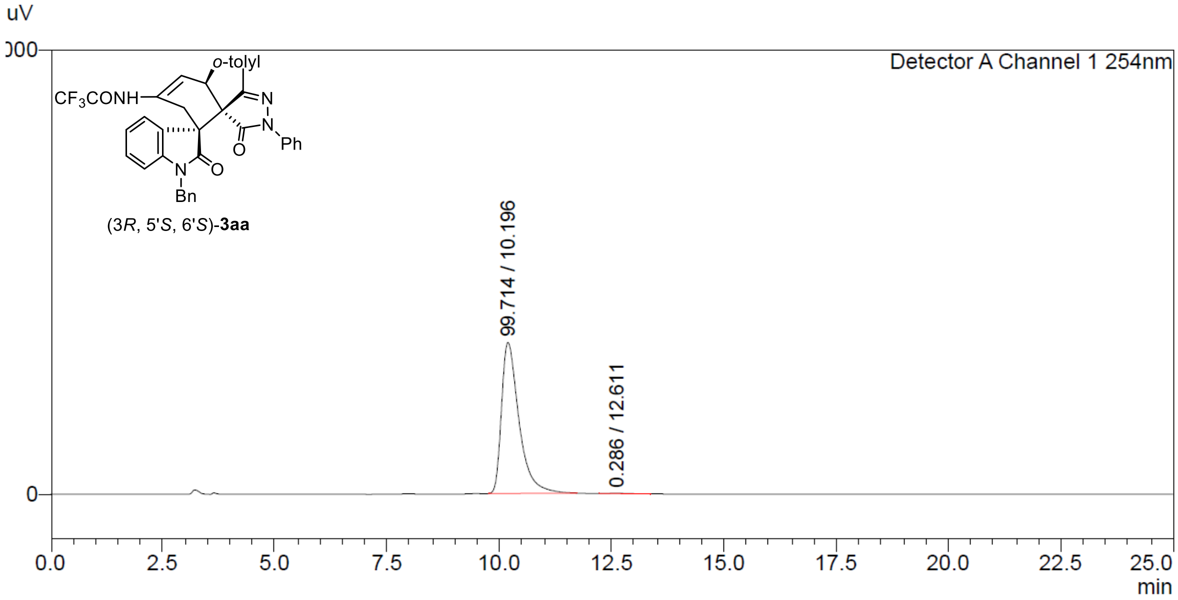

Peak Analysis Report

Detector A Channel 1 254nm

No.	Ret. Time	Height (mAu)	Area (mAu*min)	Rel. Area (%)
1	10.302	1266	36458	0.101
2	12.328	1099203	36207657	99.899
Total		1100469	36244115	100.000

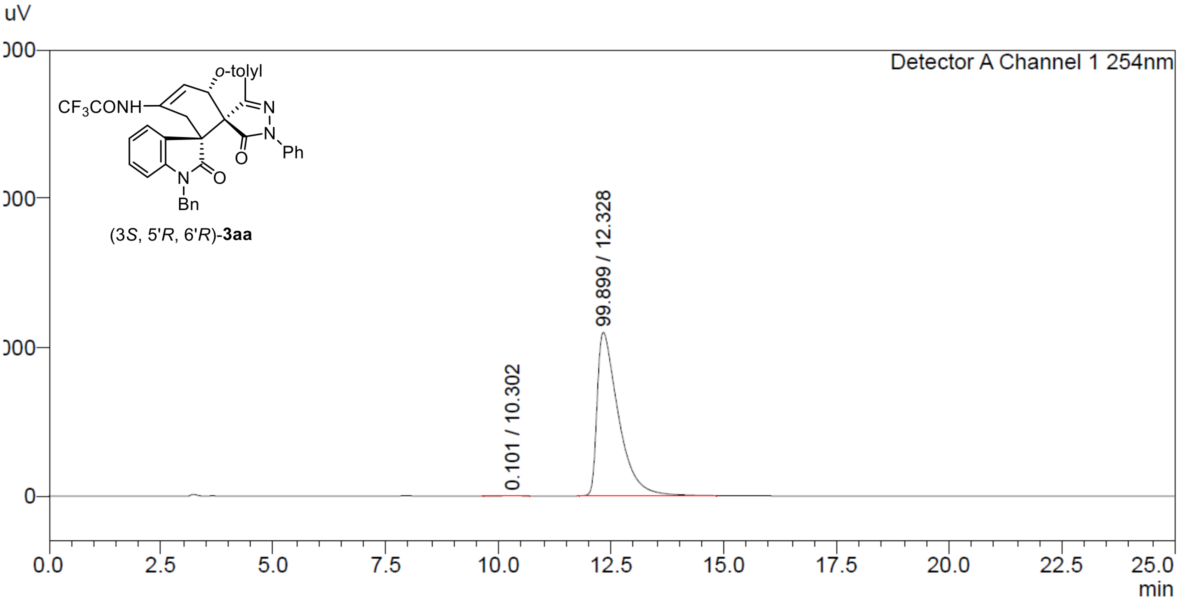

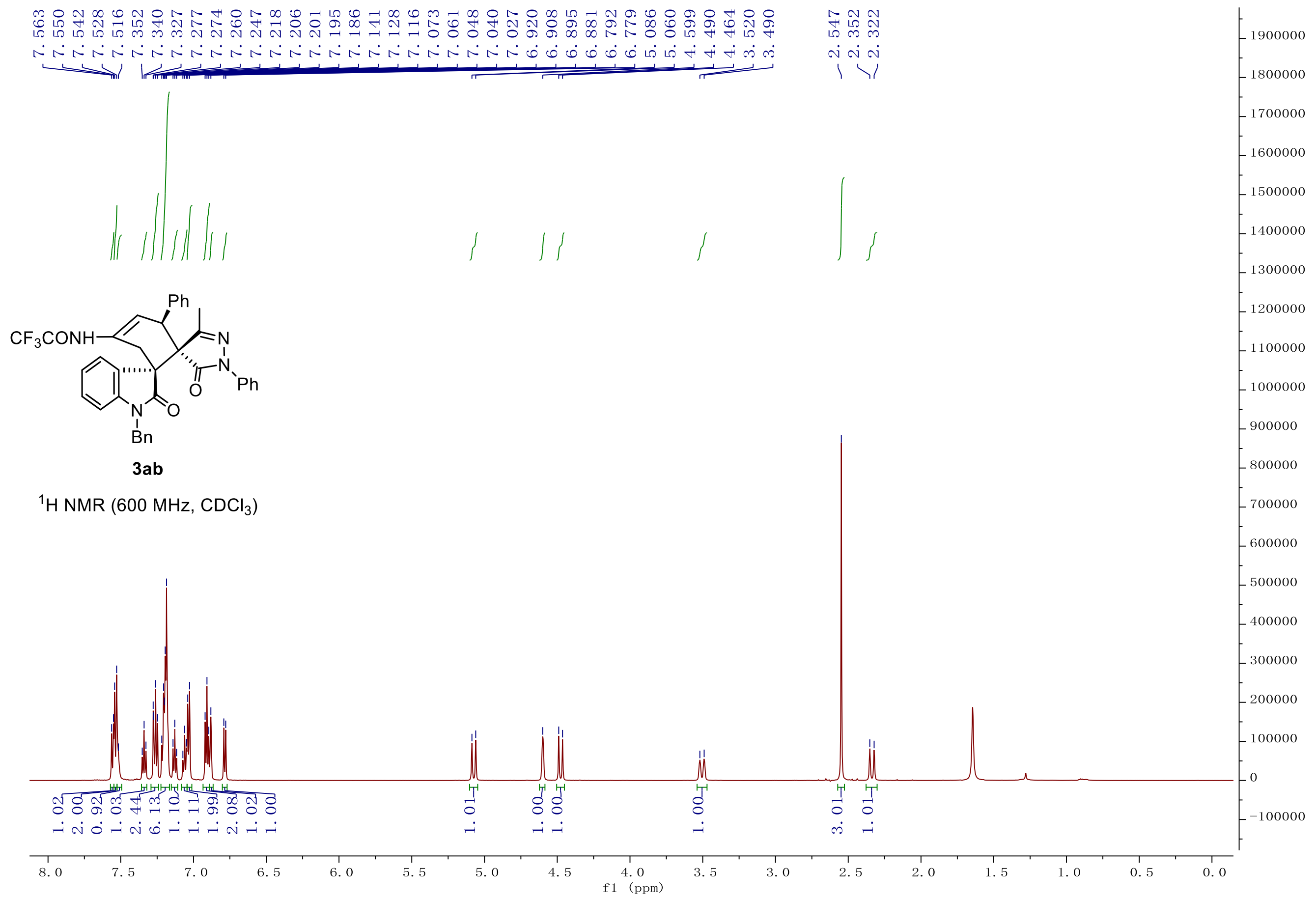
7.563 7.550 7.542 7.528 7.516 7.352 7.340 7.327 7.277 7.274 7.260 7.247 7.218 7.206 7.201 7.195 7.186 7.141 7.128 7.116 7.073 7.061 7.048 7.040 7.027 6.920 6.908 6.895 6.881 6.792 6.779 5.086 5.060 4.599 4.490 4.464 3.520 3.490
2.547 2.352 2.322
CF3CONH
Ph
N
N
Ph
O
N
O
Bn
3ab
1H NMR (600 MHz, CDCl3)
1.02 2.00 0.92 1.03 2.44 6.13 1.10 1.11 1.99 2.08 1.02 1.00
1.01 1.00 1.00 1.00 3.01 1.01
f1 (ppm)

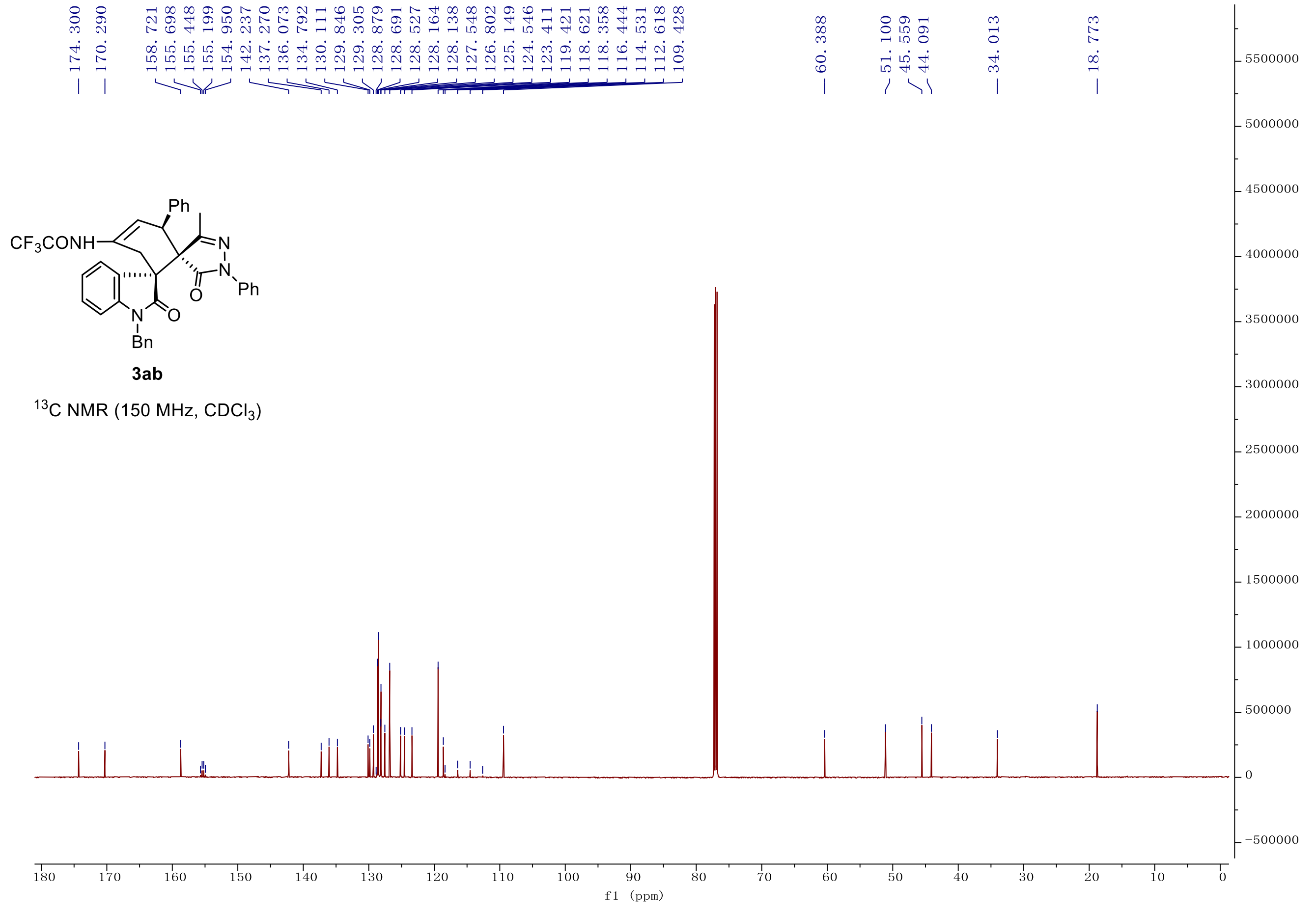
174.300 170.290
158.721 155.698 155.448 155.199 154.950 142.237 137.270 136.073 134.792 130.111 129.846 129.305 128.879 128.691 128.527 128.164 128.138 127.548 126.802 125.149 124.546 123.411 119.421 118.621 118.358 116.444 114.531 112.618 109.428
60.388 51.100 45.559 44.091 34.013 18.773
CF3CONH
Ph
N
N
Ph
O
N
O
Bn
3ab
13C NMR (150 MHz, CDCl3)
f1 (ppm)

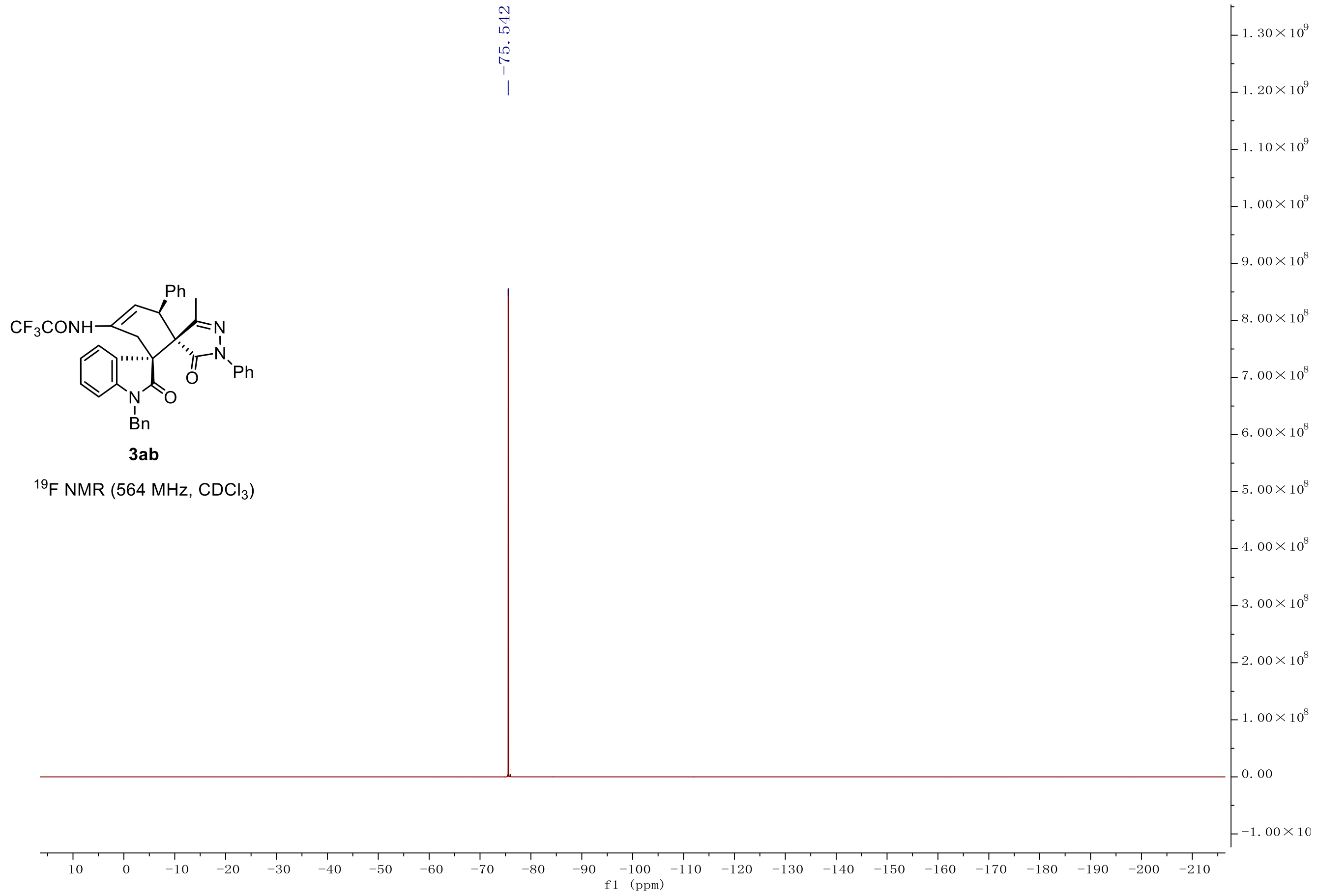

Peak Analysis Report

Detector A Channel 1 254nm

No.	Ret. Time	Height (mAu)	Area (mAu*min)	Rel. Area (%)
1	8.062	284577	8070755	50.500
2	14.567	132088	7911059	49.500
Total		416665	15981814	100.000

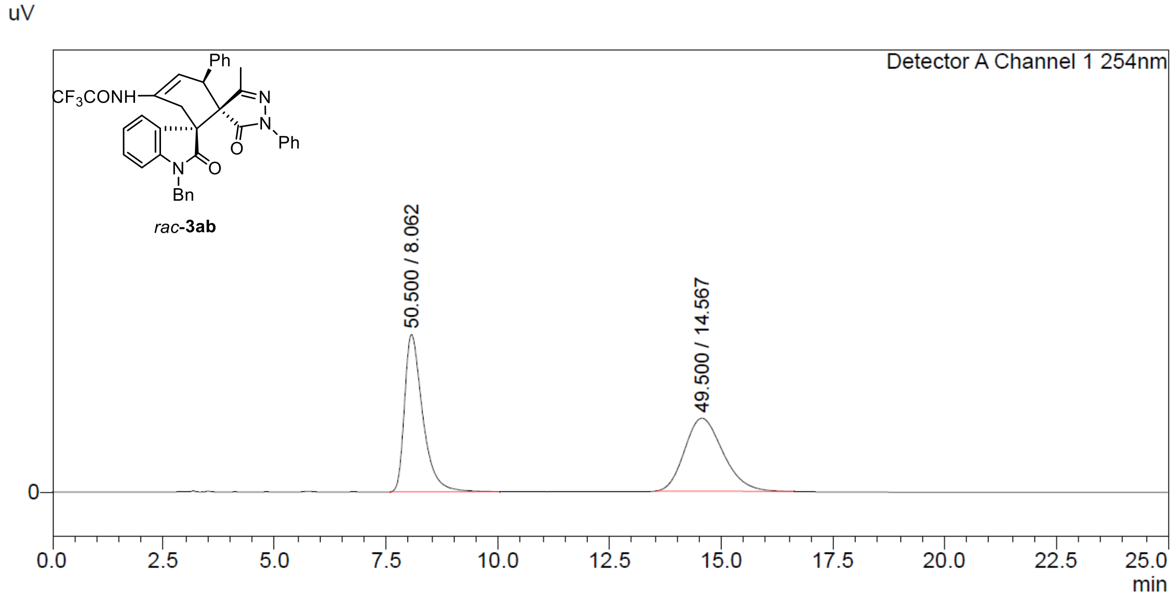

Peak Analysis Report

Detector A Channel 1 254nm

No.	Ret. Time	Height (mAu)	Area (mAu*min)	Rel. Area (%)
1	8.283	162	3304	0.015
2	14.654	349303	21646607	99.985
Total		349465	21649912	100.000

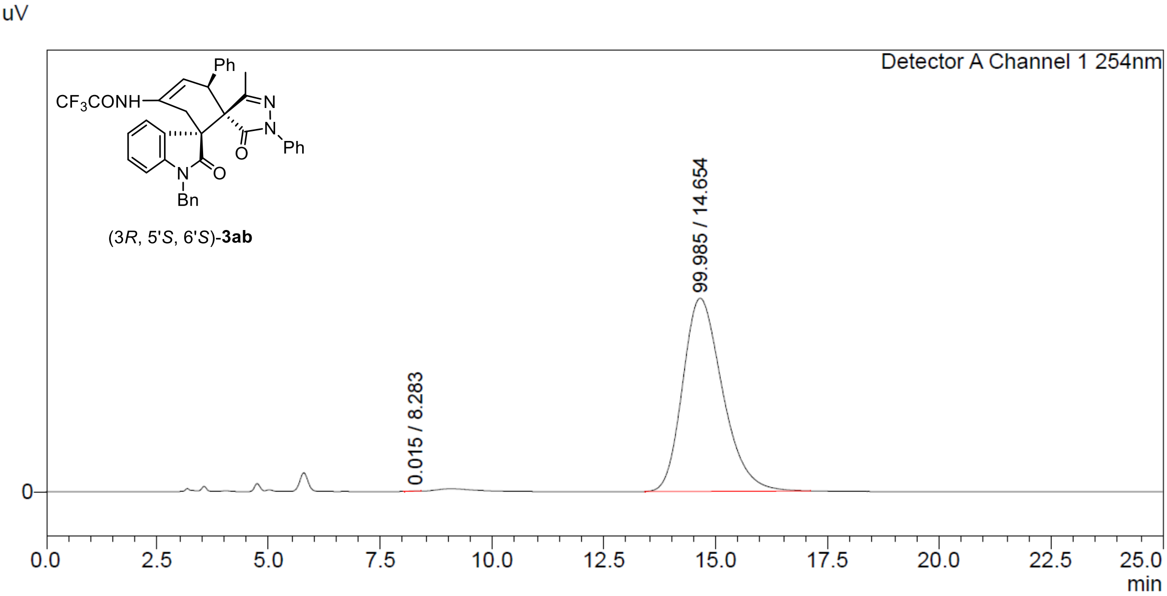

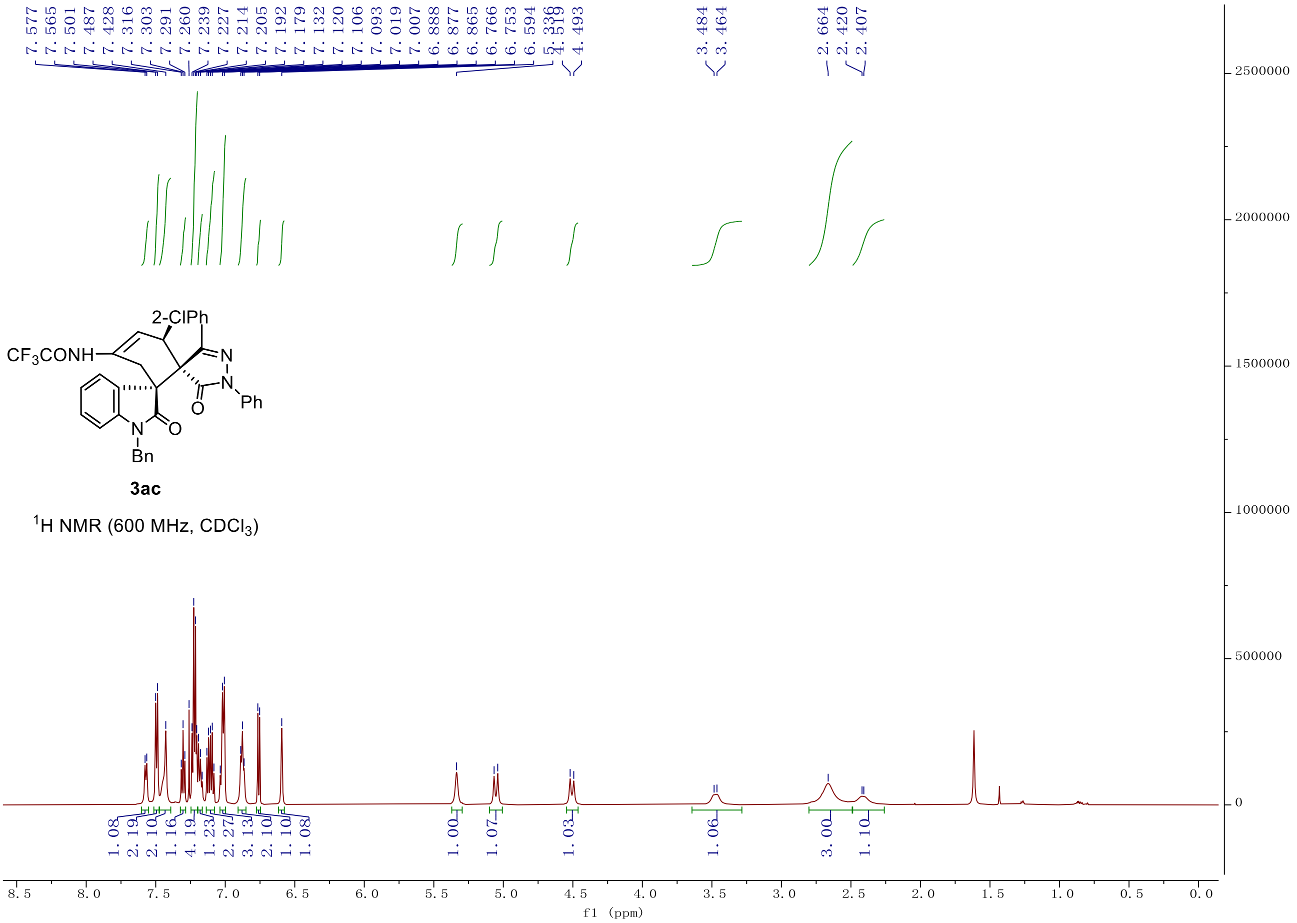

^{1}H NMR (600 MHz, $CDCl_3$)

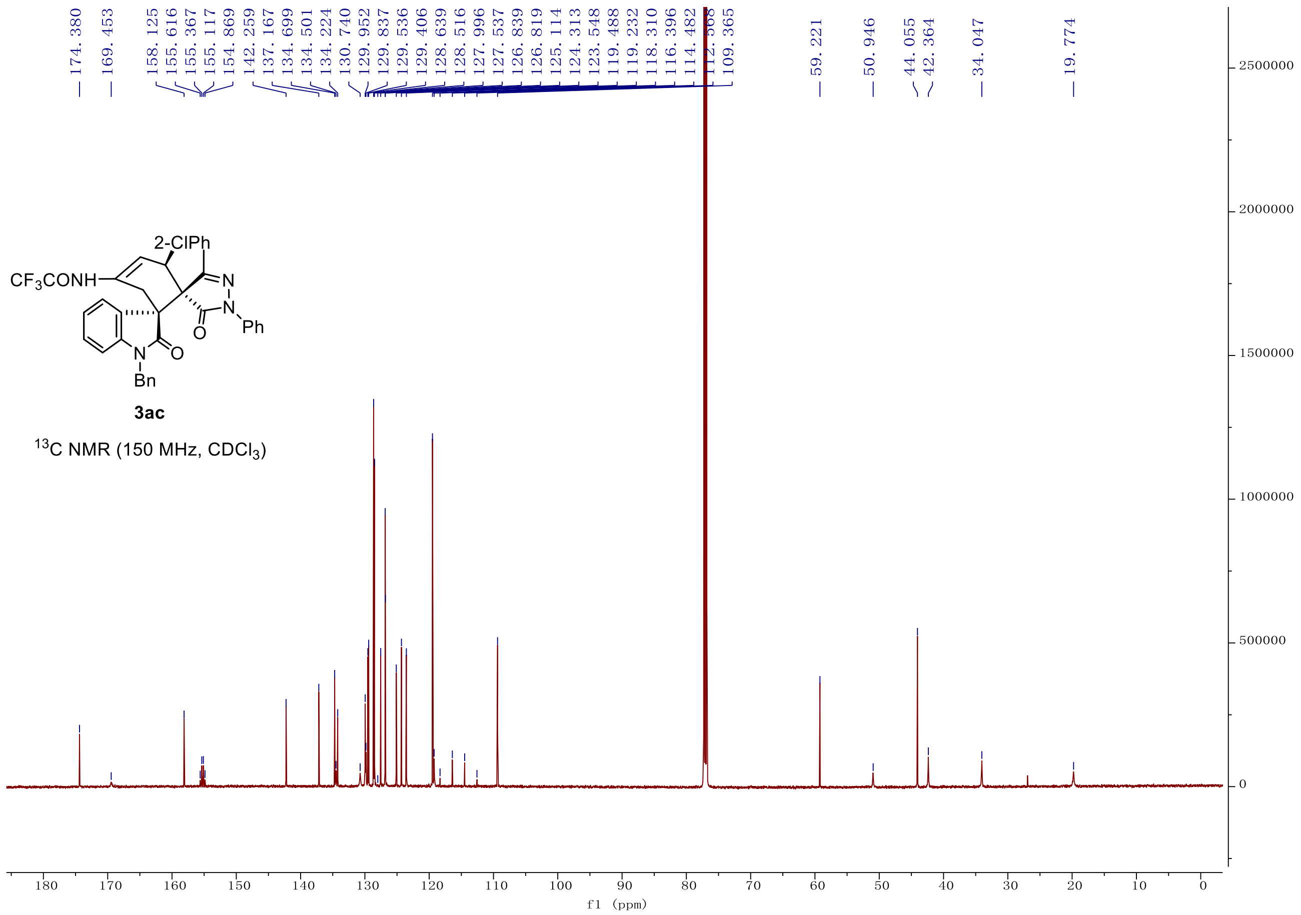

3ac

^{13}C NMR (150 MHz, $CDCl_3$)

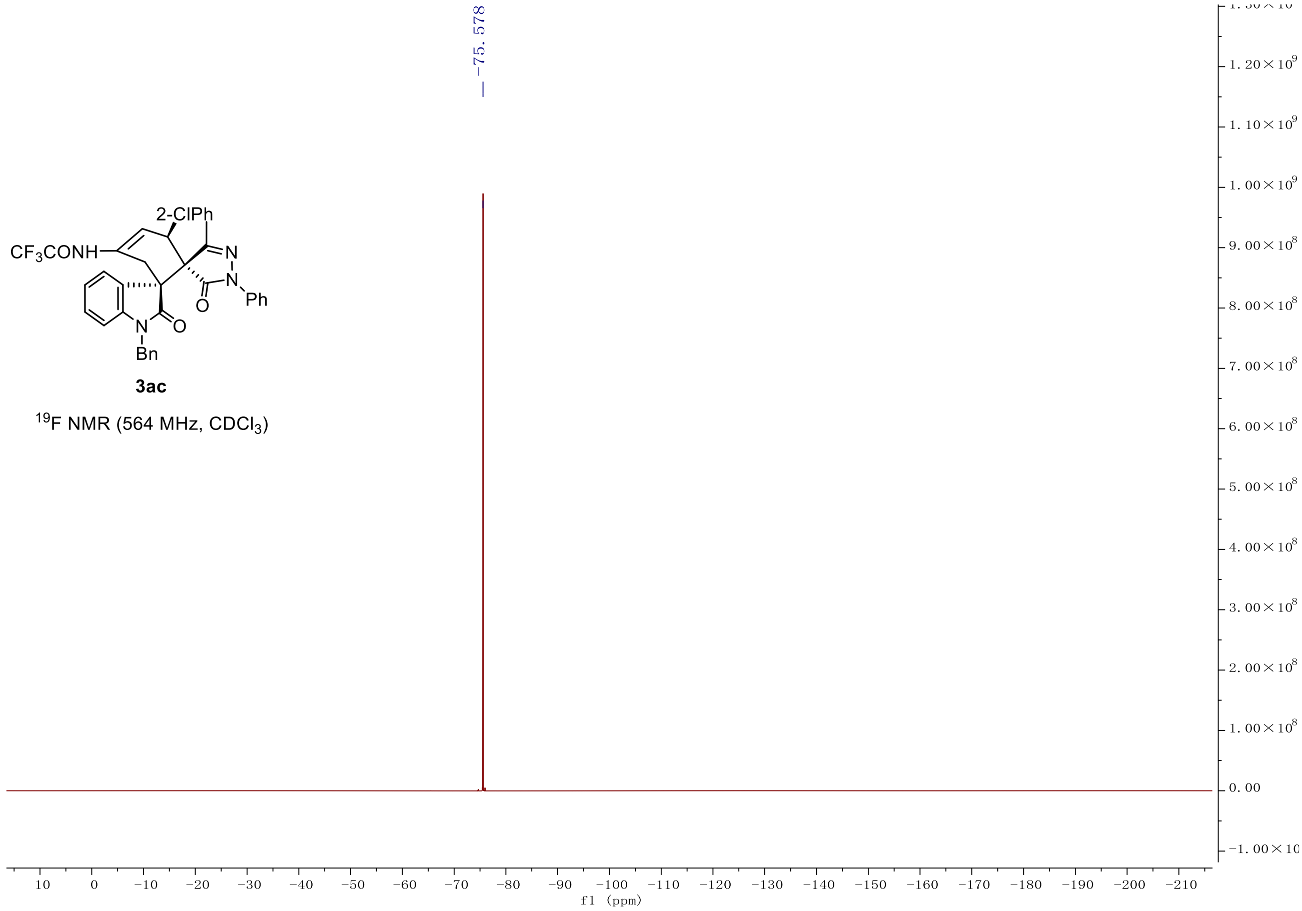

3ac

^{19}F NMR (564 MHz, $CDCl_3$)

Signal: VWD1 B, Wavelength=254 nm

RT [min]	Type	Width [min]	Area	Height	Area%	Name
4.512	VB R	0.1629	3847.8647	365.1318	50.0380	
5.189	BB	0.2135	3842.0225	281.0481	49.9620	

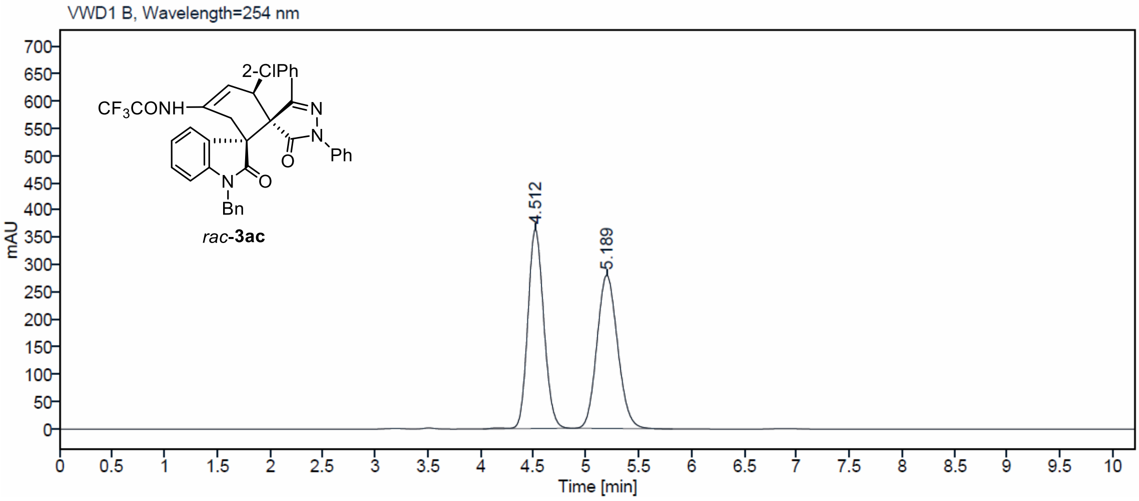

Signal: VWD1 B, Wavelength=254 nm

RT [min]	Type	Width [min]	Area	Height	Area%	Name
4.522	VV	0.2001	160.4421	12.1485	2.9867	
5.186	MM	0.2329	5211.3706	372.9256	97.0133	
		Sum	5371.8127			

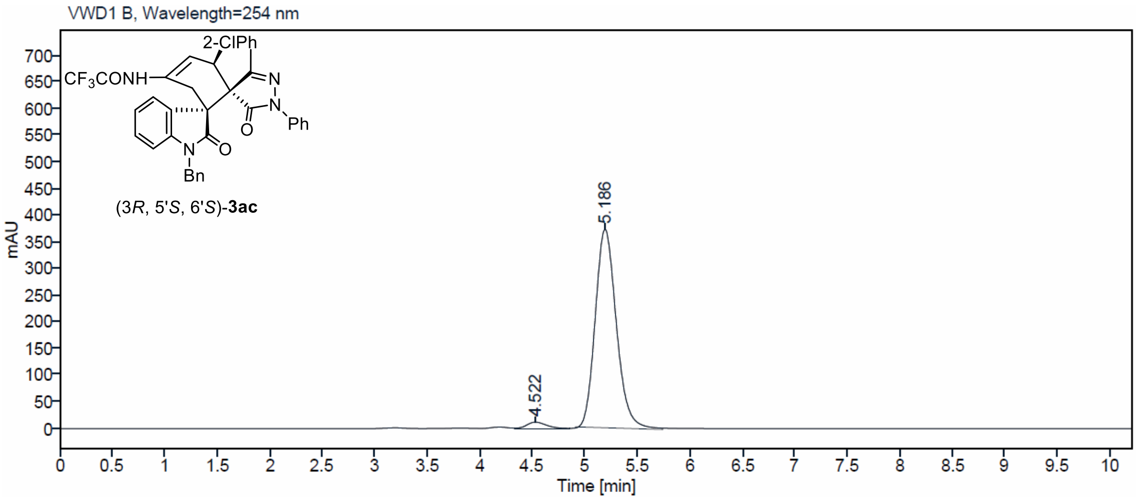

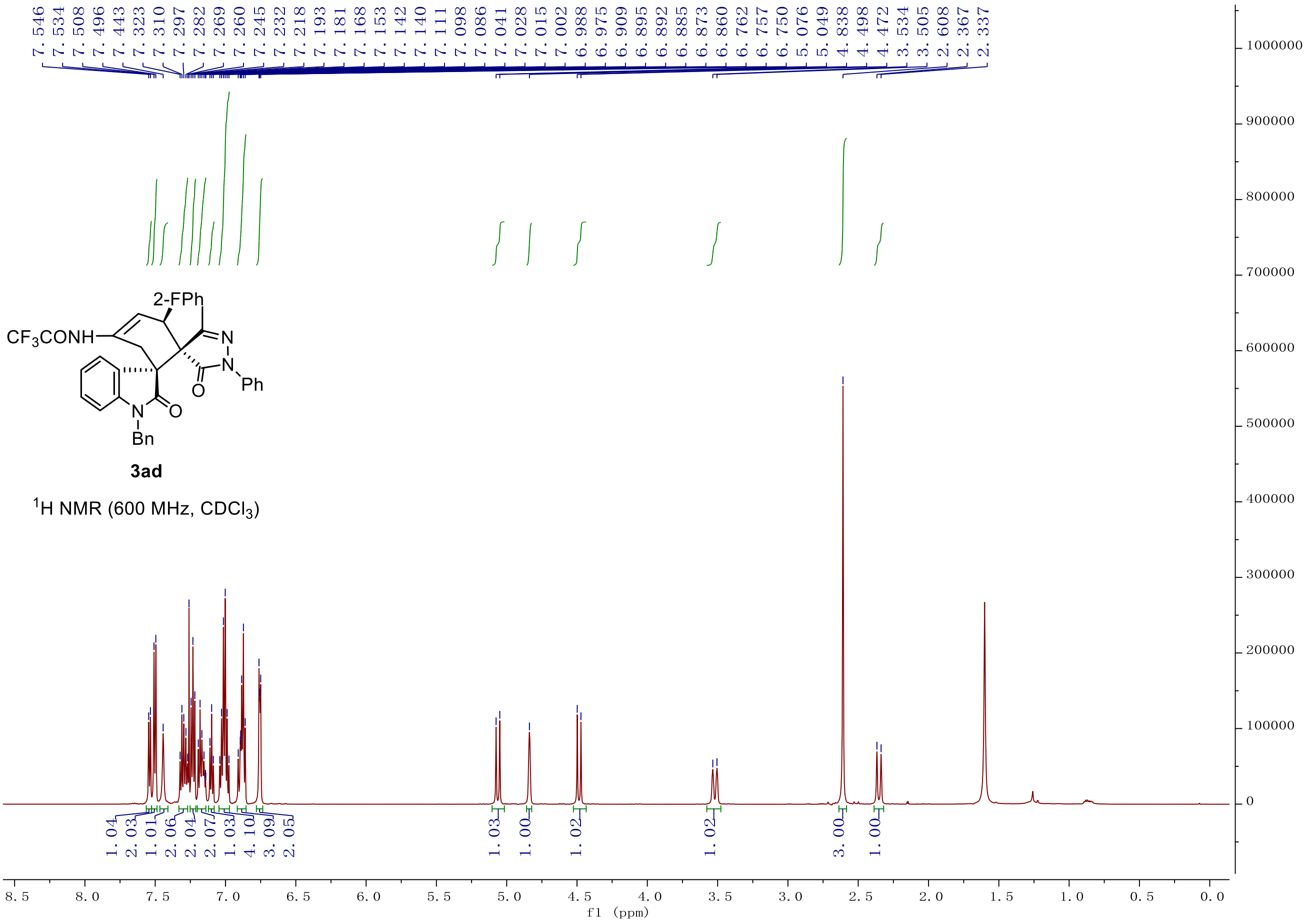

3ad

^{1}H NMR (600 MHz, $CDCl_3$)

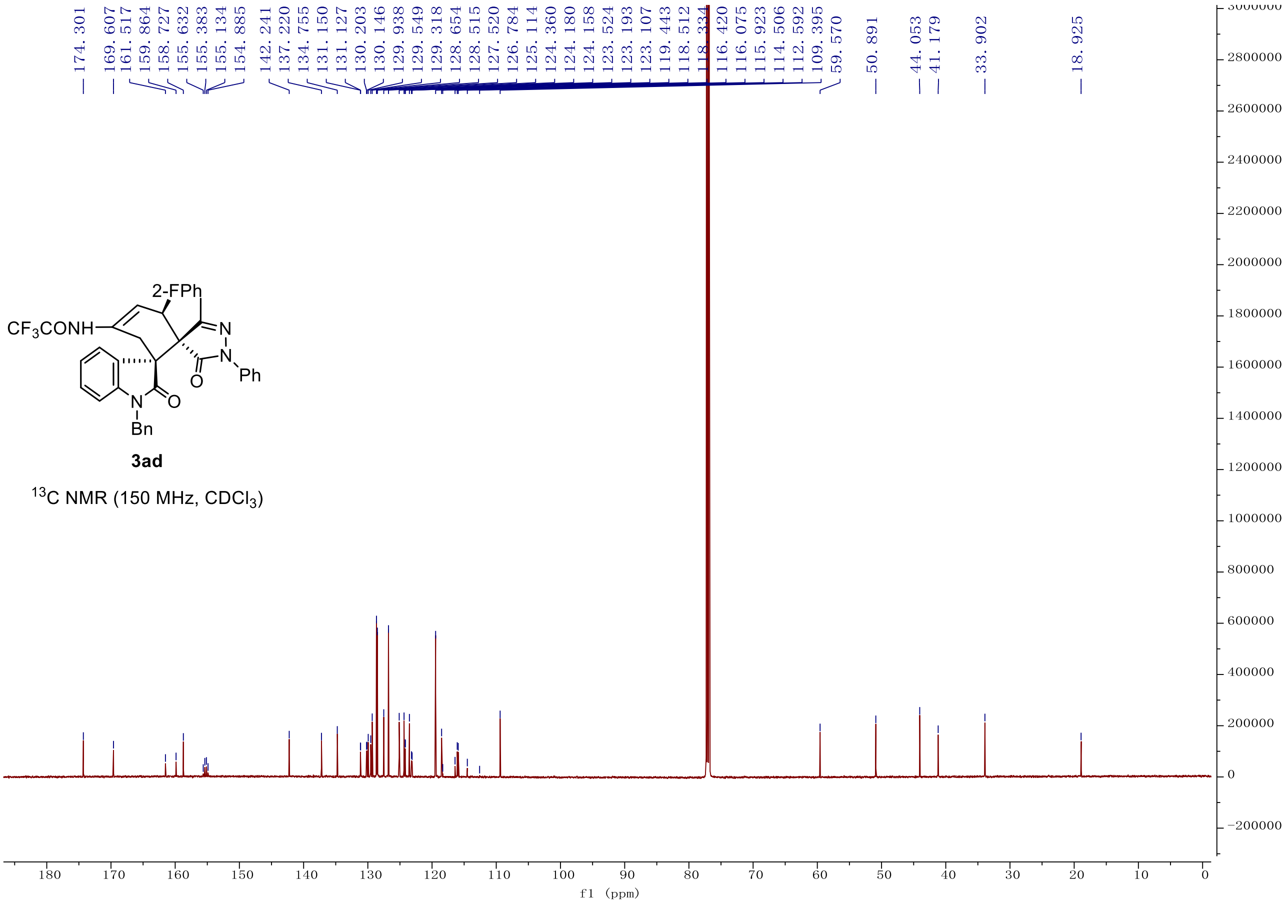

3ad

^{13}C NMR (150 MHz, $CDCl_3$)

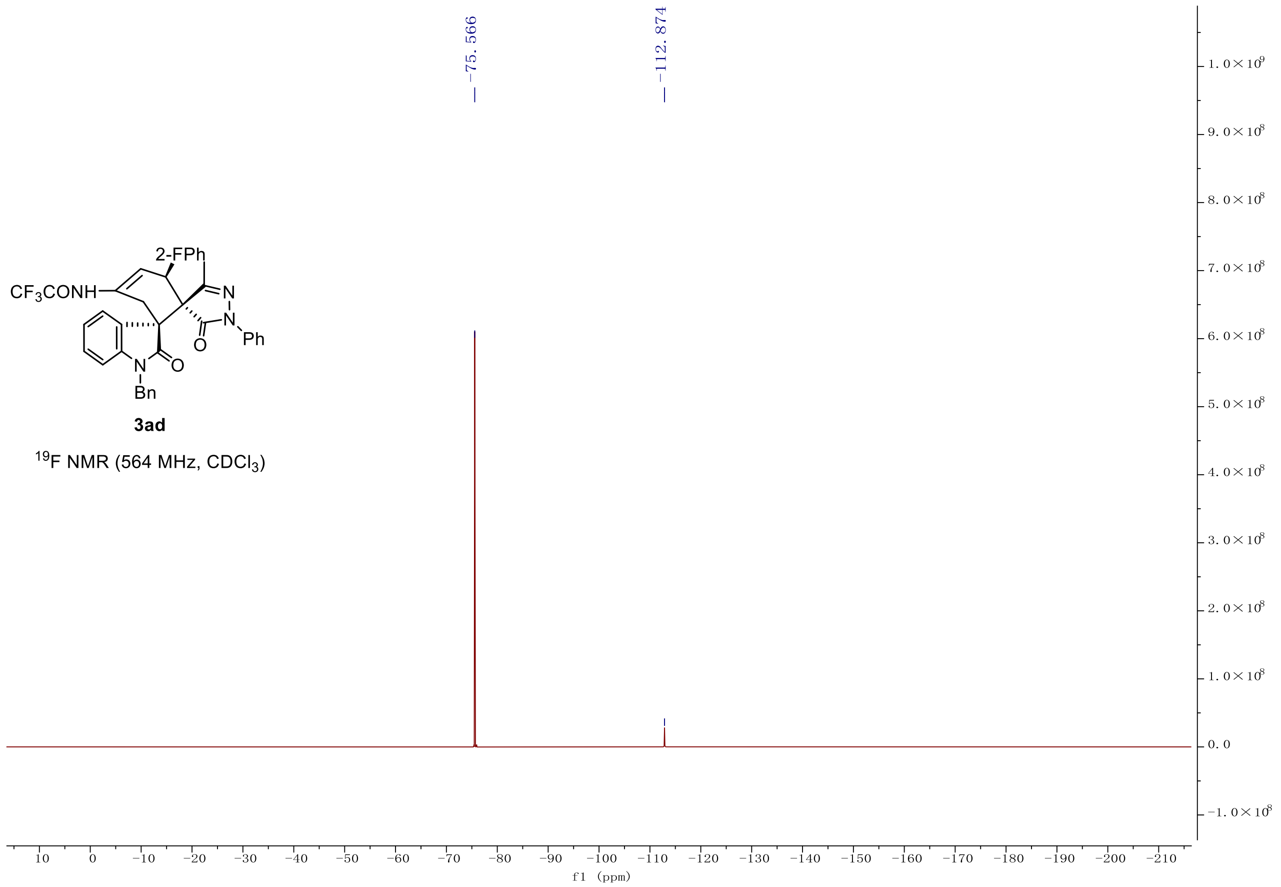

Peak Analysis Report

Detector A Channel 1 254nm

No.	Ret. Time	Height (mAu)	Area (mAu*min)	Rel. Area (%)
1	6.075	652915	8948328	49.497
2	9.523	359963	9130113	50.503
Total		1012878	18078440	100.000

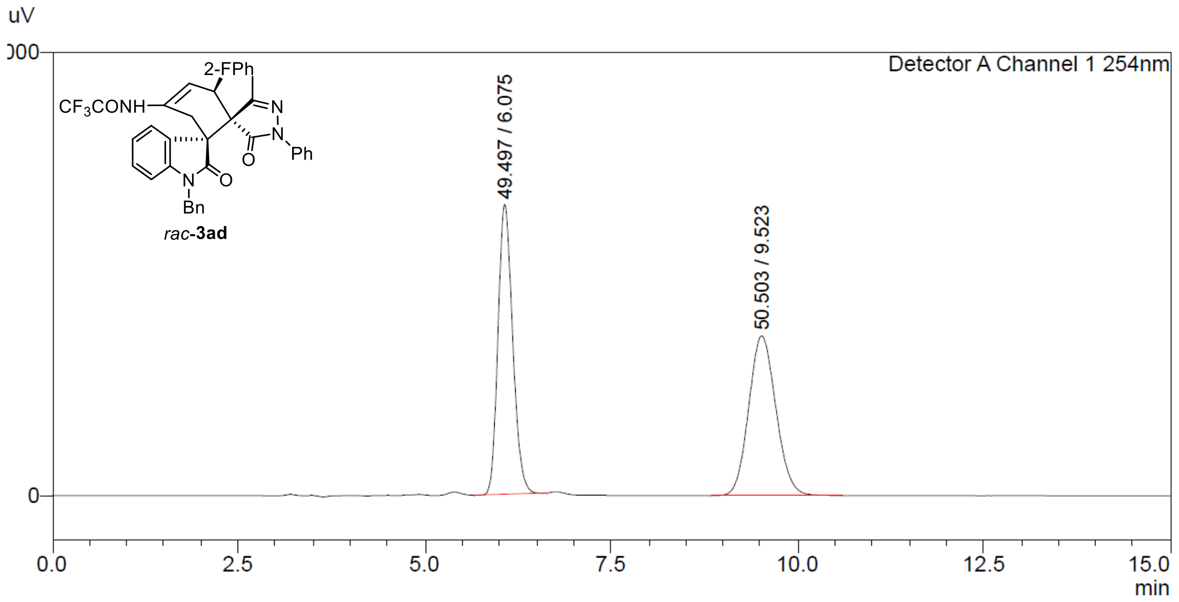

Peak Analysis Report

Detector A Channel 1 254nm

No.	Ret. Time	Height (mAu)	Area (mAu*min)	Rel. Area (%)
1	6.078	3372	48799	0.200
2	9.496	936141	24306814	99.800
Total		939513	24355613	100.000

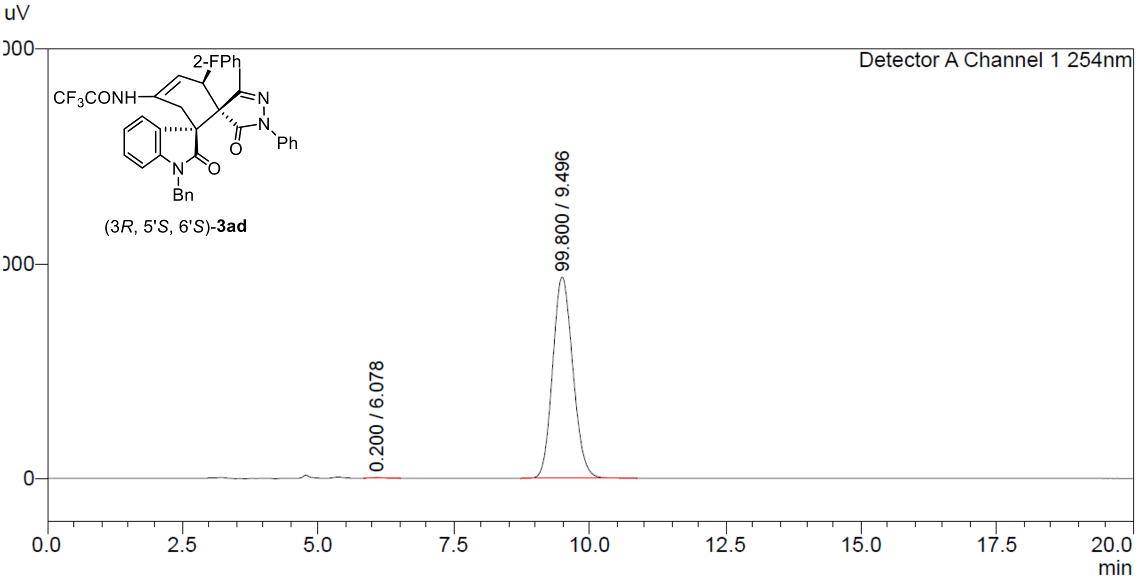

(3R, 5'S, 6'S)-3ad

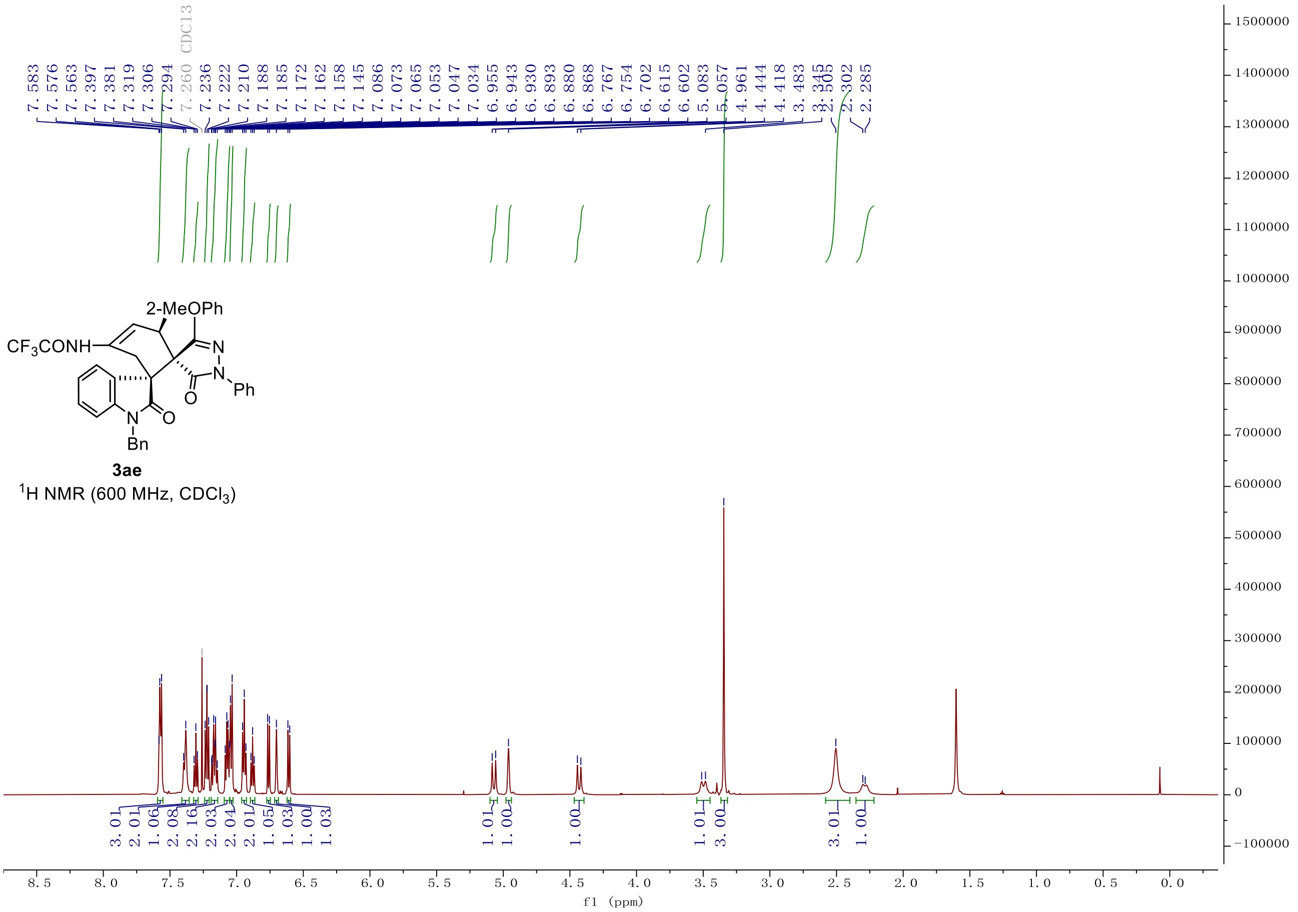

3ae

^{1}H NMR (600 MHz, $CDCl_3$)

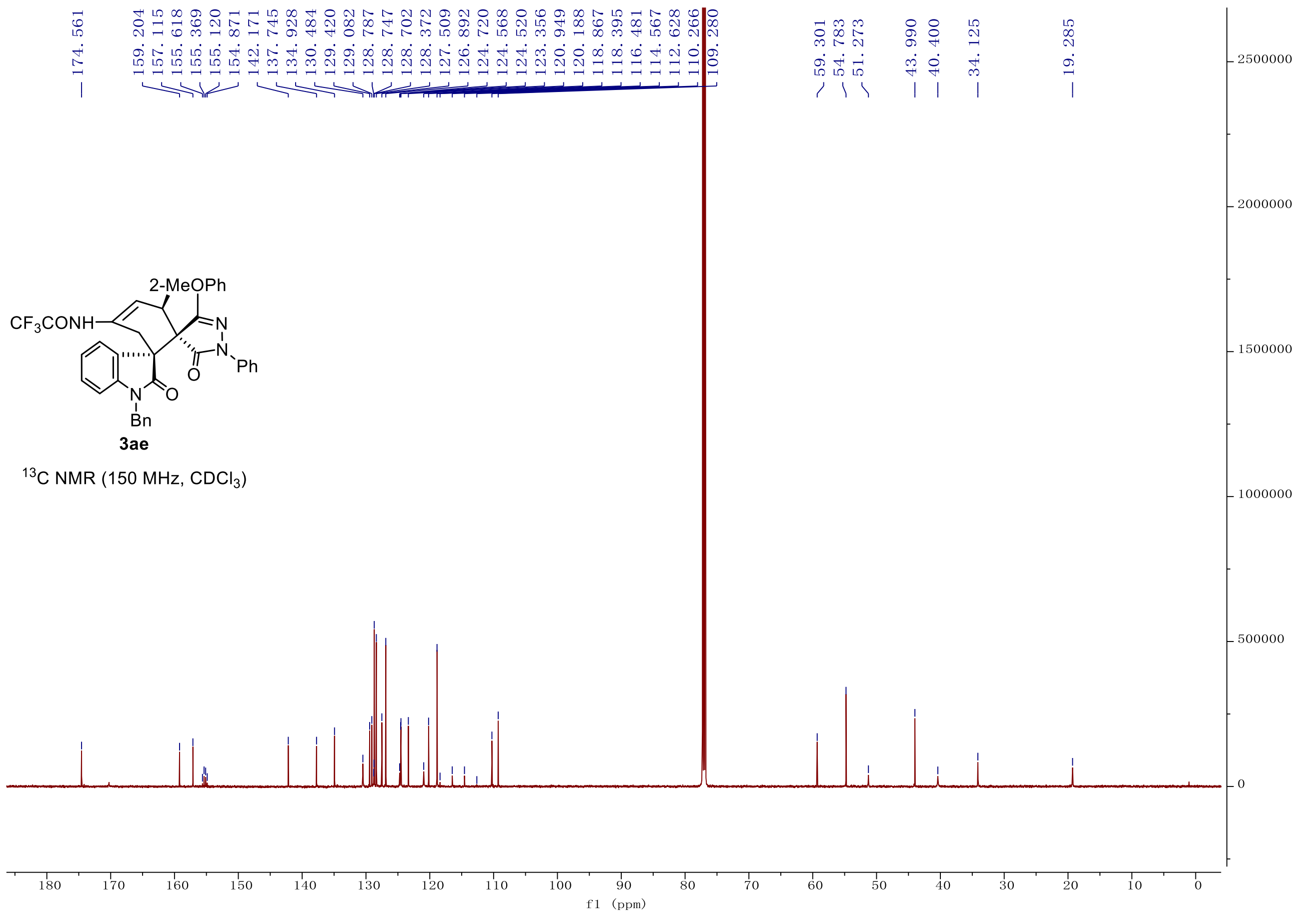
174.561
159.204
157.115
155.618
155.369
155.120
154.871
142.171
137.745
134.928
130.484
129.420
129.082
128.787
128.747
128.702
128.372
127.509
126.892
124.720
124.568
124.520
123.356
120.949
120.188
118.867
118.395
116.481
114.567
112.628
110.266
109.280
59.301
54.783
51.273
43.990
40.400
34.125
19.285
2-MeOPh
CF_3CONH
N
N
Ph
O
N
O
Bn
3ae
^{13}C NMR (150 MHz, $CDCl_3$)
f1 (ppm)

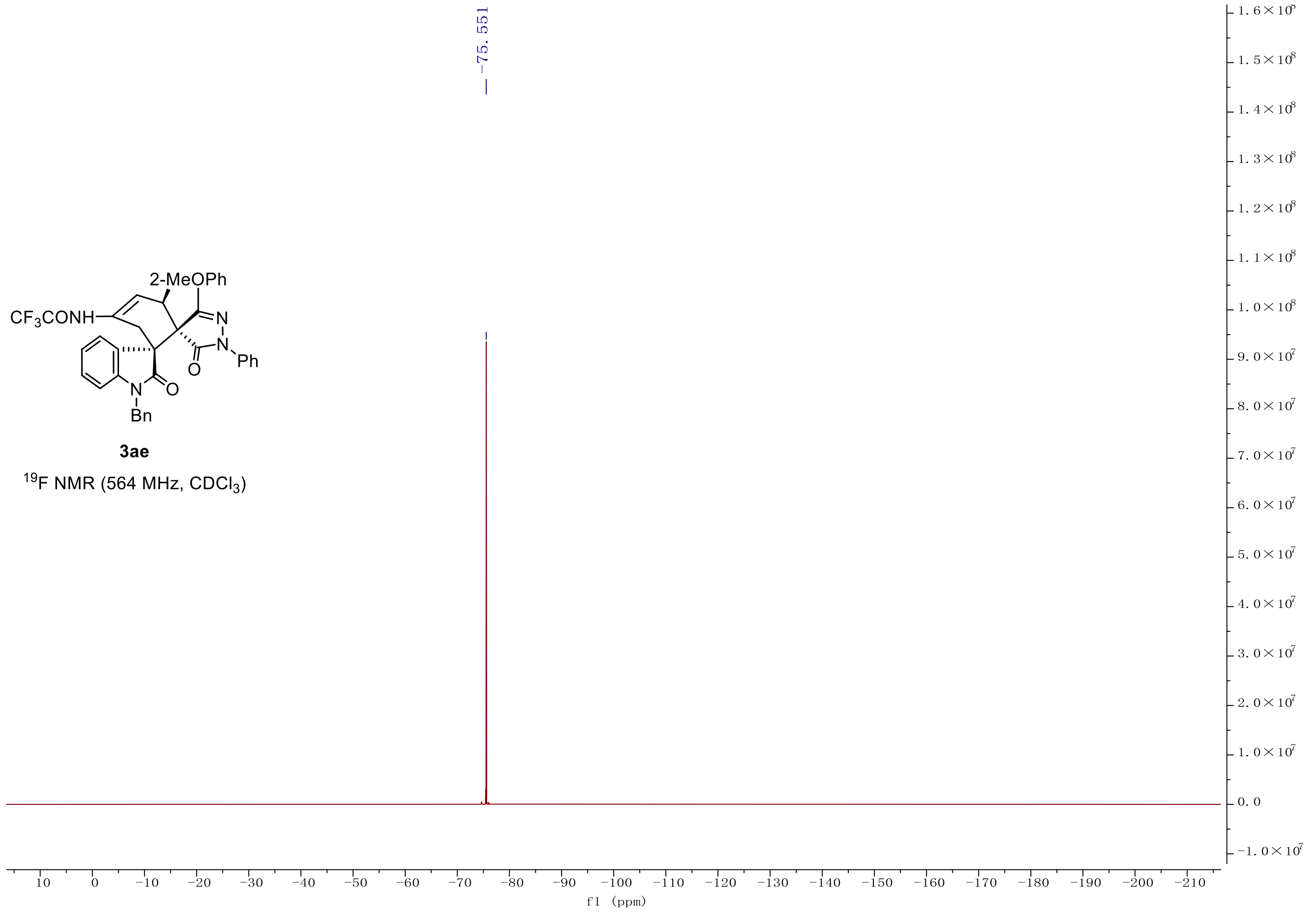
−75.551
2-MeOPh
CF_3CONH
N
N
Ph
O
N
O
Bn
3ae
^{19}F NMR (564 MHz, $CDCl_3$)
f1 (ppm)

Peak Analysis Report

Detector A Channel 1 254nm

No.	Ret. Time	Height (mAu)	Area (mAu*min)	Rel. Area (%)
1	5.116	92244	1245965	50.136
2	5.790	82398	1239218	49.864
Total		174642	2485183	100.000

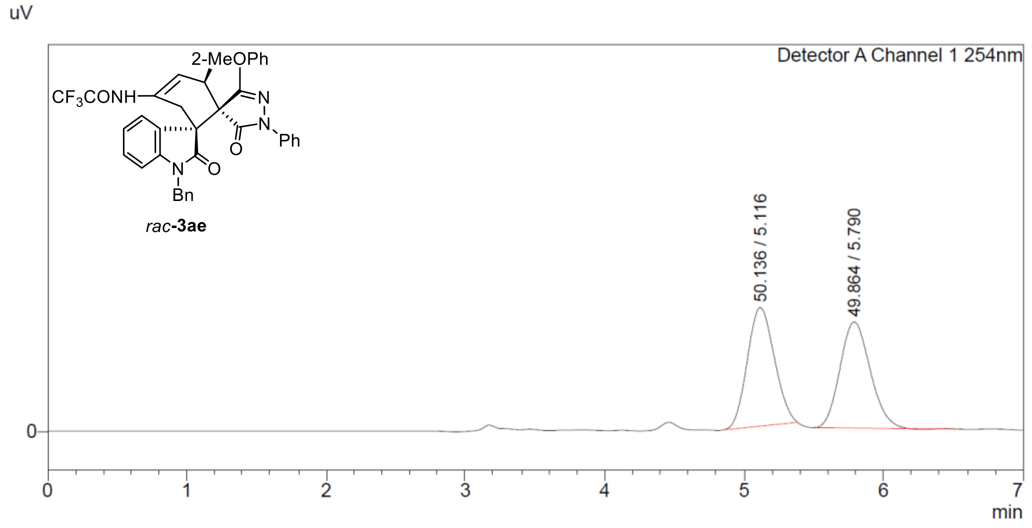

Peak Analysis Report

Detector A Channel 1 254nm

No.	Ret. Time	Height (mAu)	Area (mAu*min)	Rel. Area (%)
1	5.067	3009	21718	0.162
2	5.675	800892	13385204	99.838
Total		803901	13406922	100.000

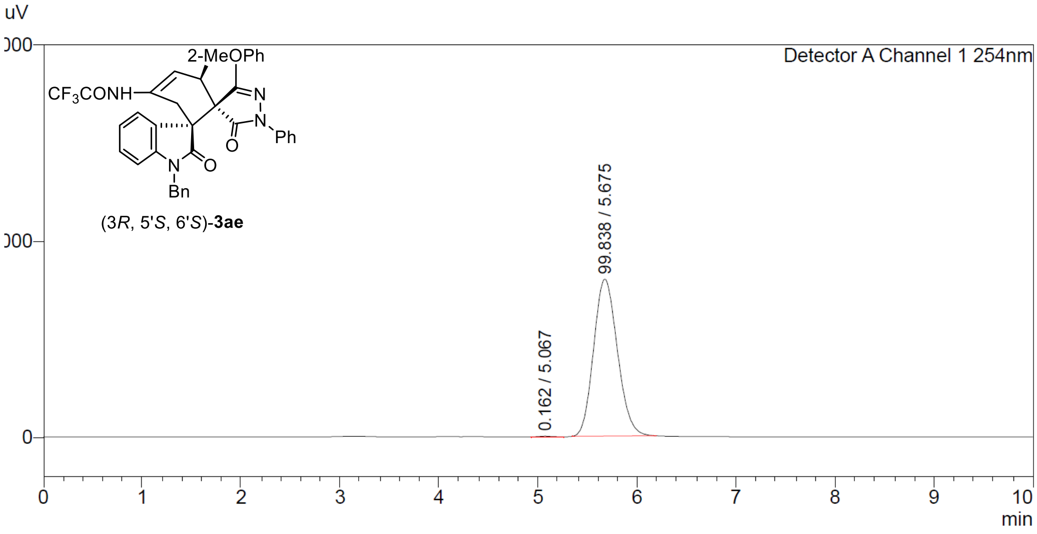

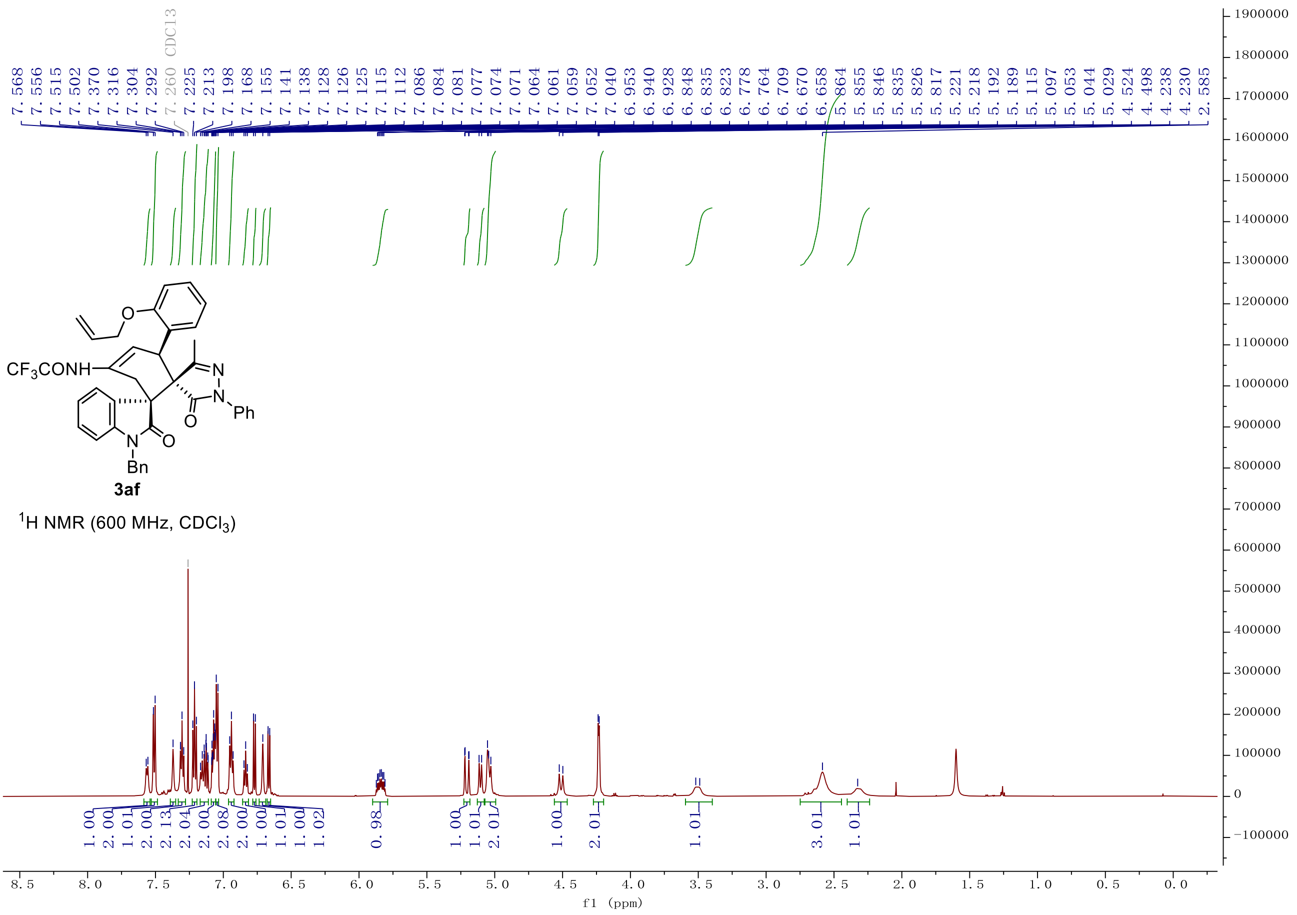
7.568 7.556 7.515 7.502 7.370 7.316 7.304 7.292 7.260 CDCl3 7.225 7.213 7.198 7.168 7.155 7.141 7.138 7.128 7.126 7.125 7.115 7.112 7.086 7.084 7.081 7.077 7.074 7.071 7.064 7.061 7.059 7.052 7.040 6.953 6.940 6.928 6.848 6.835 6.823 6.778 6.764 6.709 6.670 6.658 5.864 5.855 5.846 5.835 5.826 5.817 5.221 5.218 5.192 5.189 5.115 5.097 5.053 5.044 5.029 4.524 4.498 4.238 4.230 2.585
CF3CONH
N
N
Ph
O
O
N
Bn
3af
1H NMR (600 MHz, CDCl3)
1.00 2.00 1.01 2.00 2.13 2.04 2.00 2.08 2.00 1.00 1.01 1.00 1.02 0.98 1.00 1.01 2.01 1.00 2.01 1.01 3.01 1.01
f1 (ppm)

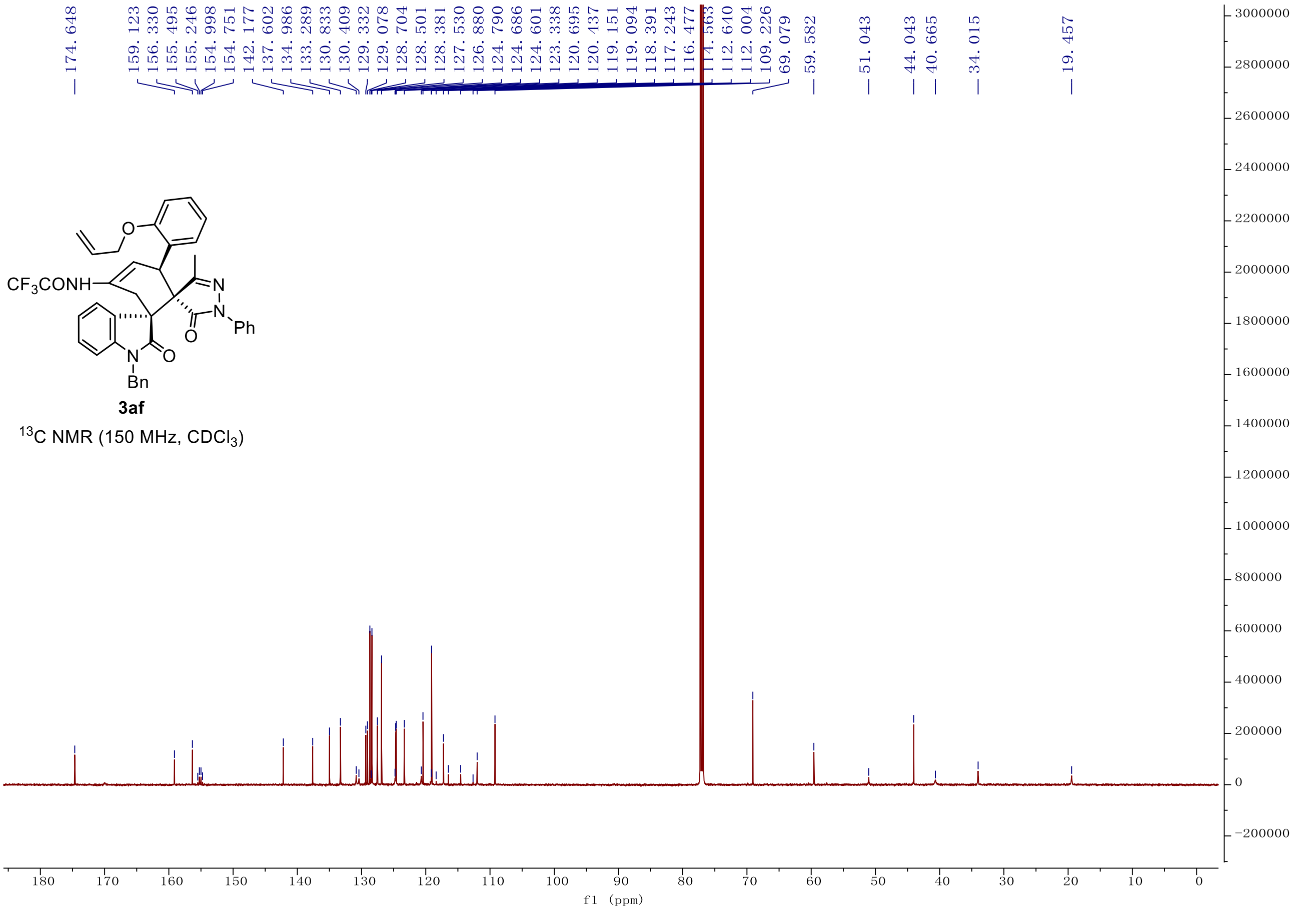
174.648 159.123 156.330 155.495 155.246 154.998 154.751 142.177 137.602 134.986 133.289 130.833 130.409 129.332 129.078 128.704 128.501 128.381 127.530 126.880 124.790 124.686 124.601 123.338 120.695 120.437 119.151 119.094 118.391 117.243 116.477 114.563 112.640 112.004 109.226 69.079 59.582 51.043 44.043 40.665 34.015 19.457
CF3CONH
N
N
Ph
O
O
N
Bn
3af
13C NMR (150 MHz, CDCl3)
f1 (ppm)

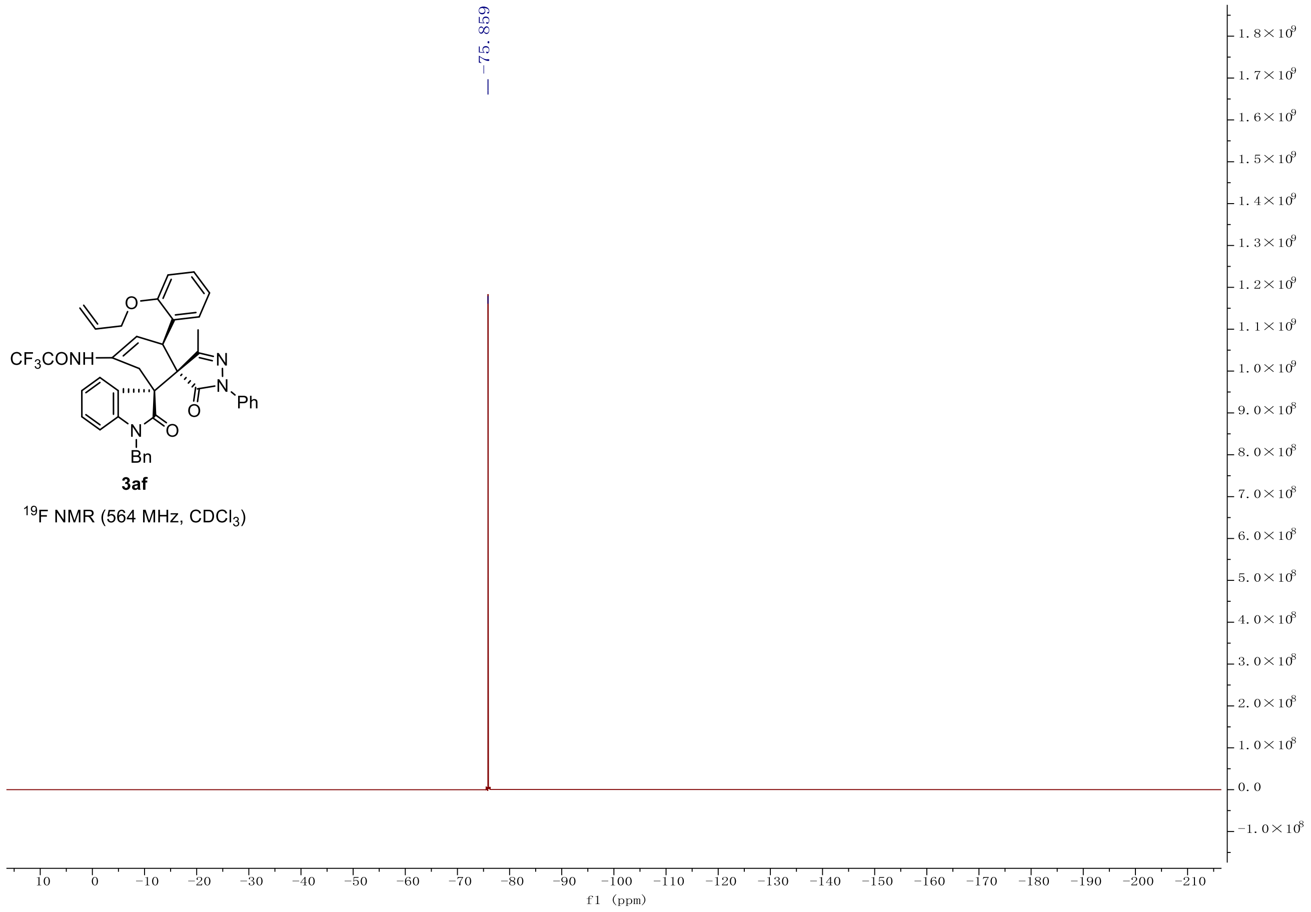

Signal: VWD1 B, Wavelength=254 nm

RT [min]Type	Area	Width[min]	Area%
4.231	8062.3730	0.227	49.0081
4.845	8388.7285	0.221	50.9919
总和	16451.1016		

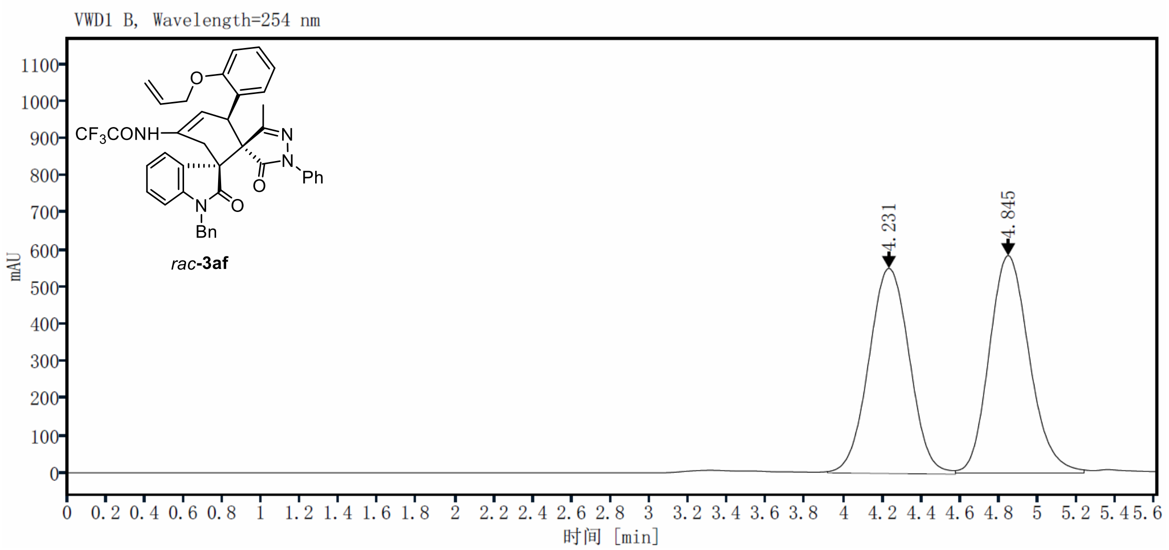

Signal: VWD1 B, Wavelength=254 nm

RT [min]Type	Area	Width[min]	Area%
4.280	976.5025	0.446	2.1278
4.827	44915.5781	0.231	97.8722
总和	45892.0806		

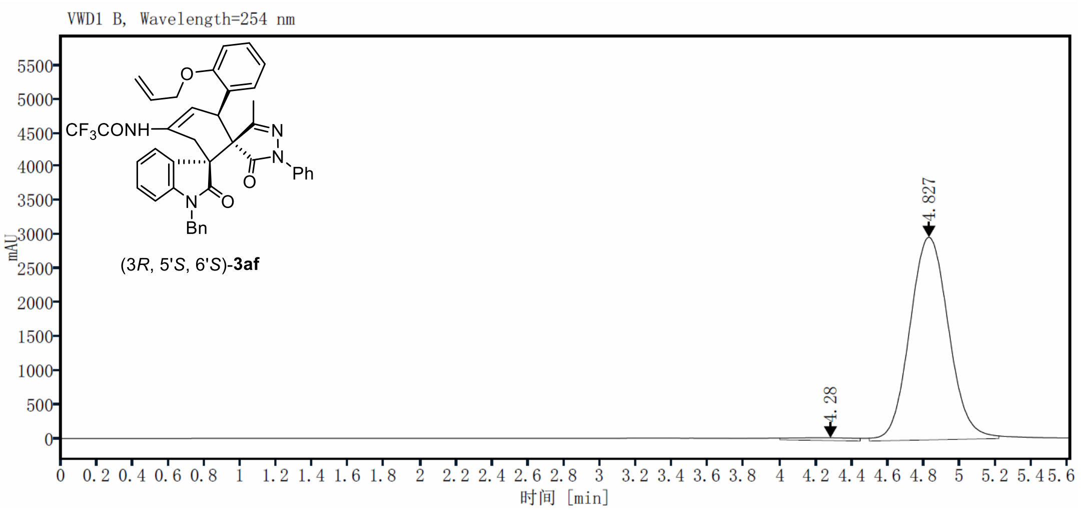

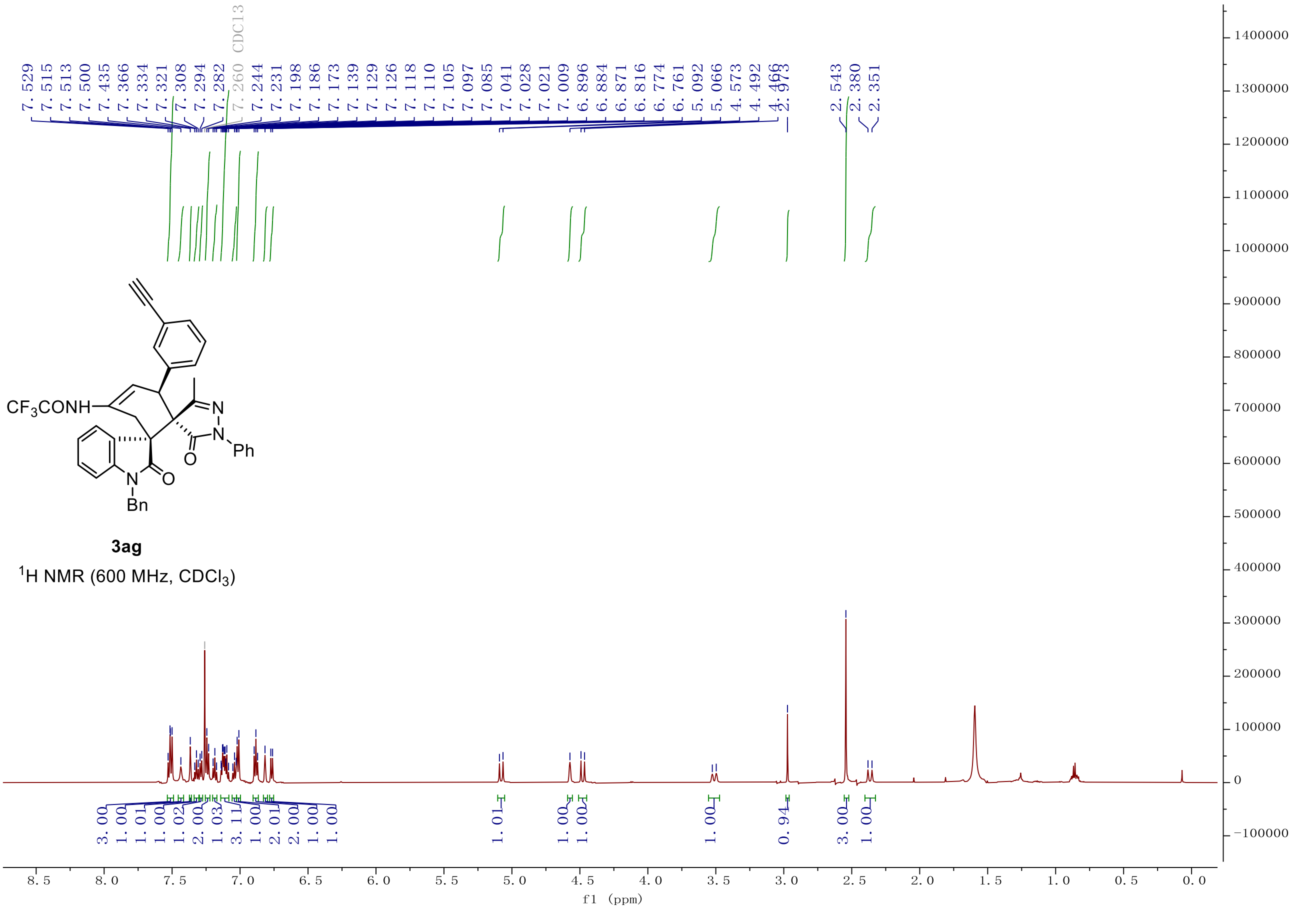

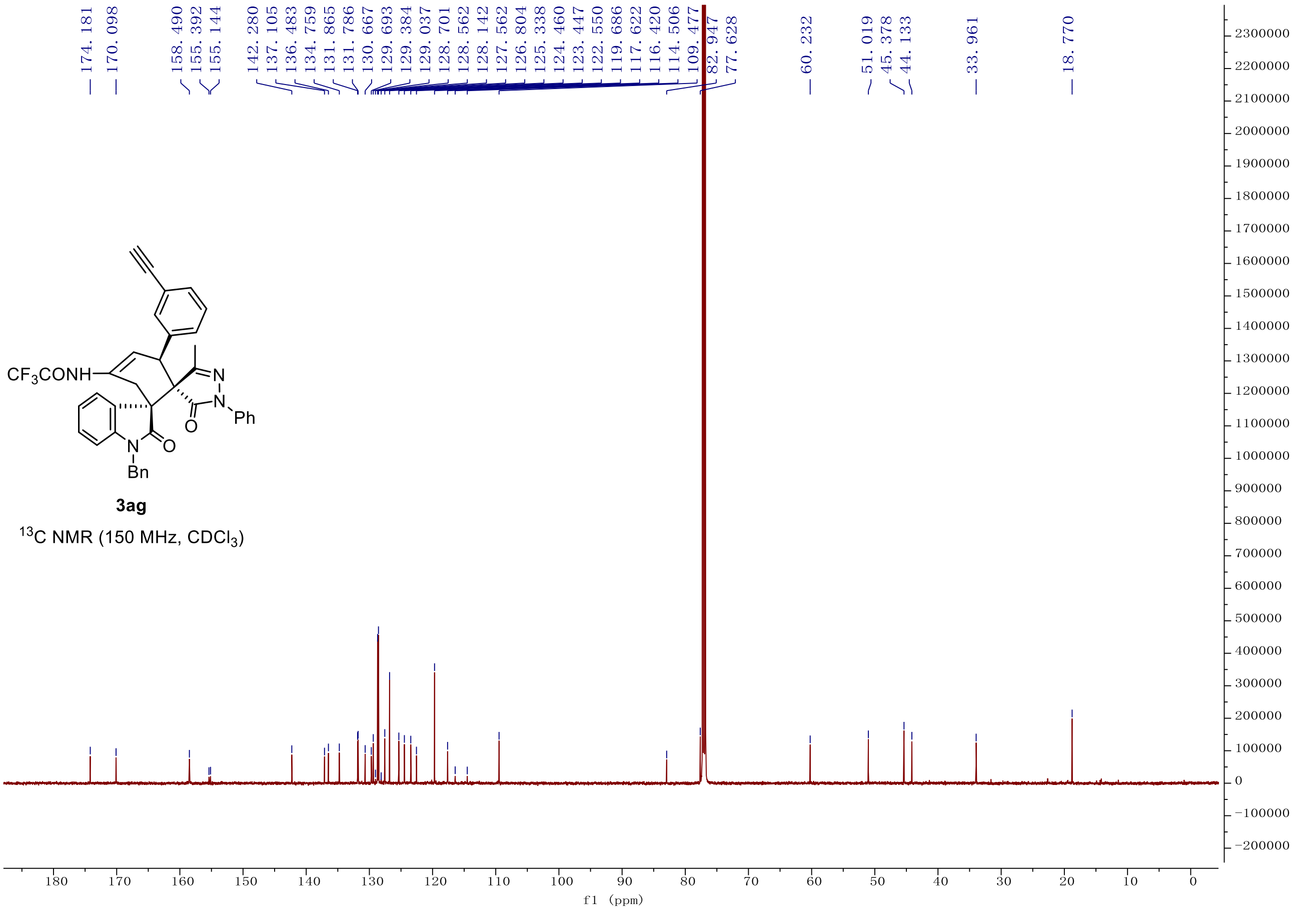
174.181
170.098
158.490
155.392
155.144
142.280
137.105
136.483
134.759
131.865
131.786
130.667
129.693
129.384
129.037
128.701
128.562
128.142
127.562
126.804
125.338
124.460
123.447
122.550
119.686
117.622
116.420
114.506
109.477
82.947
77.628
60.232
51.019
45.378
44.133
33.961
18.770
CF_3CONH
N
Ph
O
Bn
3ag
^{13}C NMR (150 MHz, $CDCl_3$)
f1 (ppm)

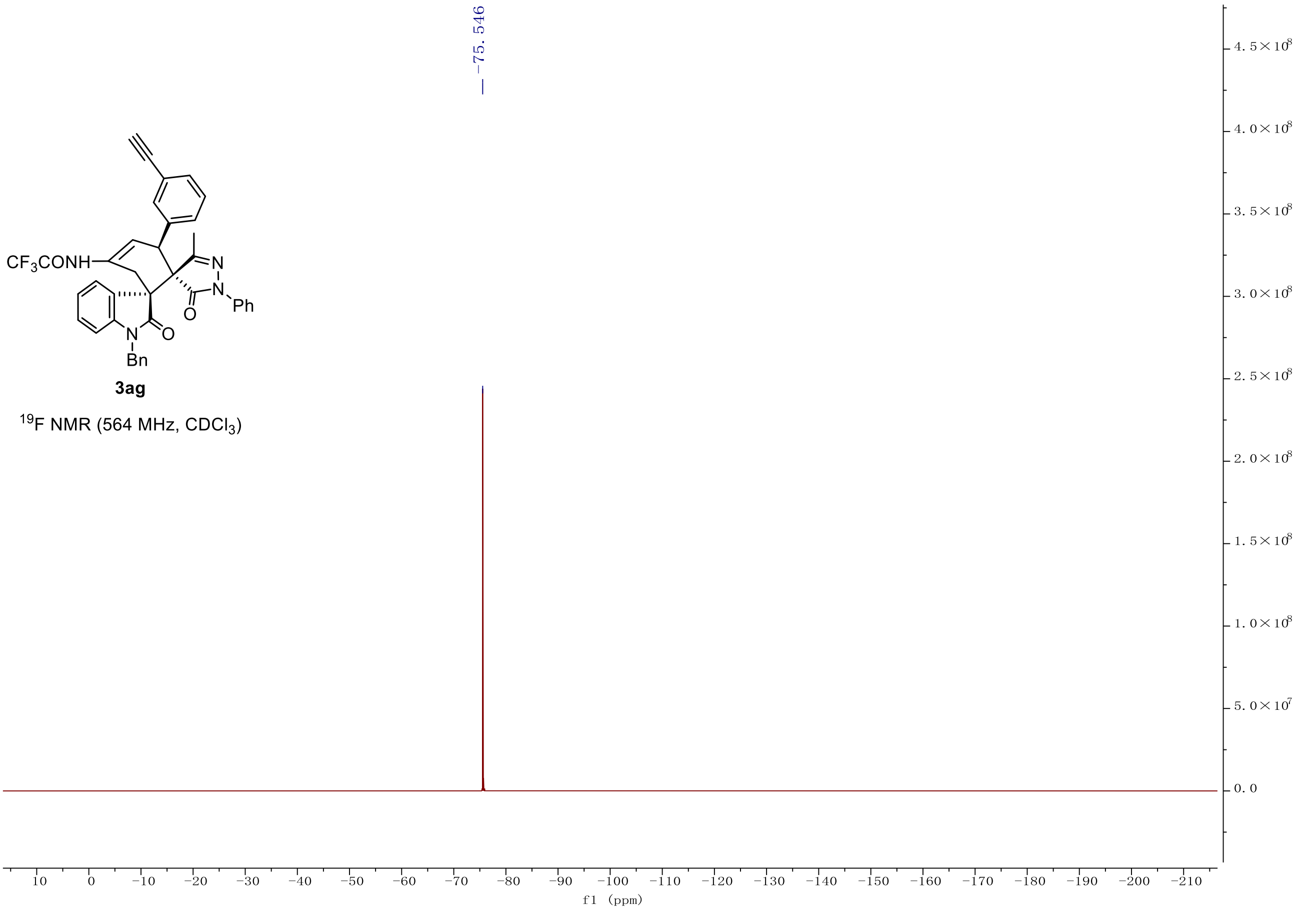
−75.546
CF_3CONH
N
Ph
O
Bn
3ag
^{19}F NMR (564 MHz, $CDCl_3$)
f1 (ppm)

Peak Analysis Report

Detector A Channel 1 254nm

No.	Ret. Time	Height (mAu)	Area (mAu*min)	Rel. Area (%)
1	7.639	188614	5298775	50.832
2	10.592	94674	5125406	49.168
Total		283288	10424181	100.000

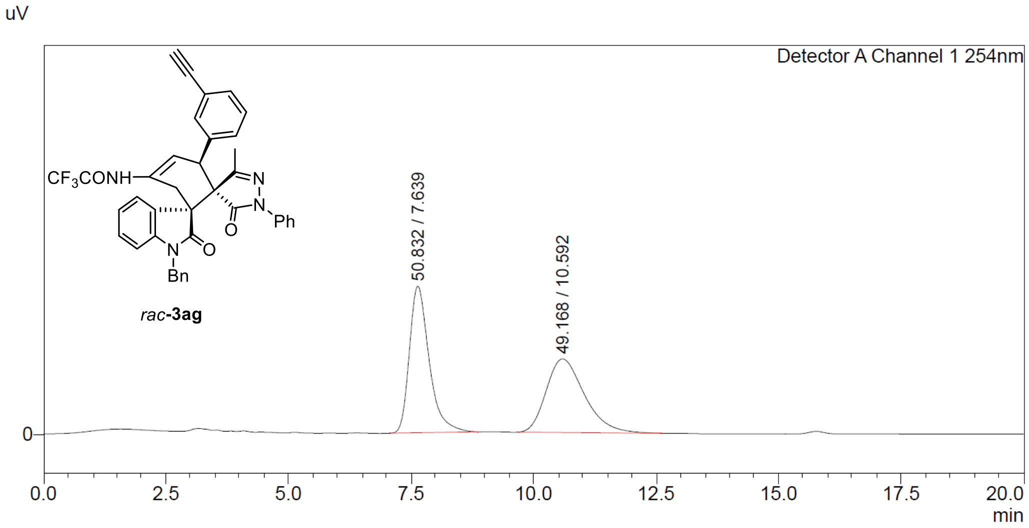

Peak Analysis Report

Detector A Channel 1 254nm

No.	Ret. Time	Height (mAu)	Area (mAu*min)	Rel. Area (%)
1	7.731	15920	444005	1.806
2	10.614	433788	24135058	98.194
Total		449708	24579063	100.000

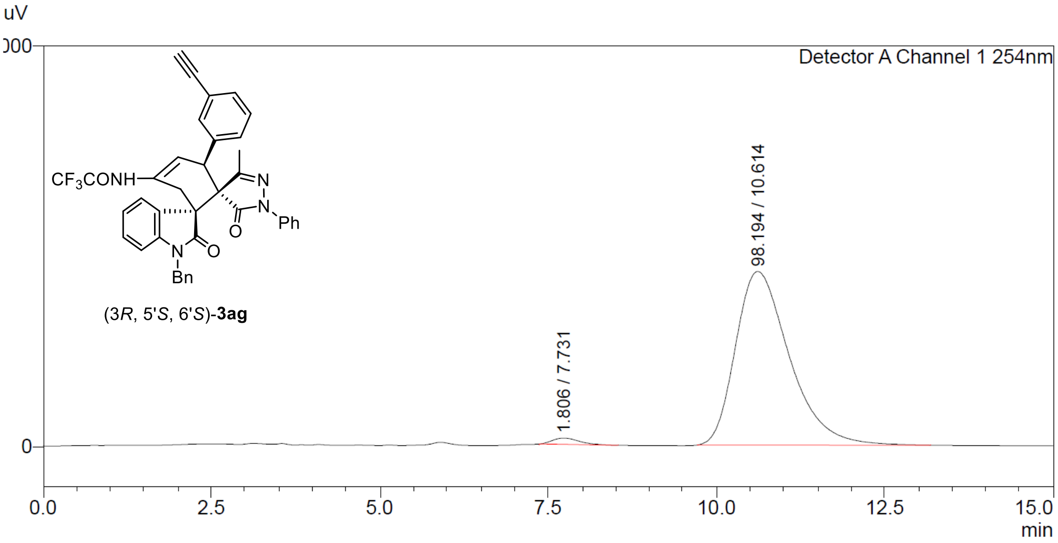

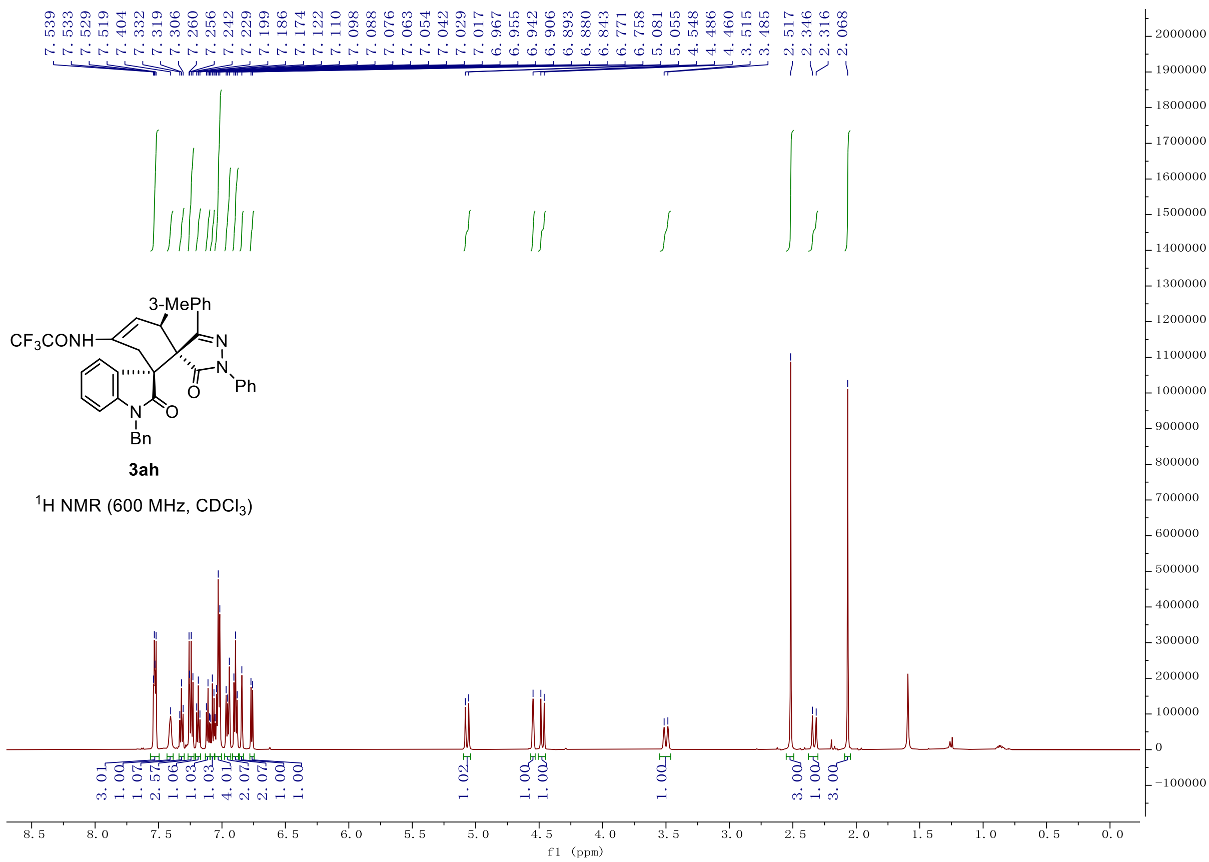

3-MePh
CF3CONH
N
N
Ph
O
N
O
Bn
3ah
1H NMR (600 MHz, CDCl3)
f1 (ppm)

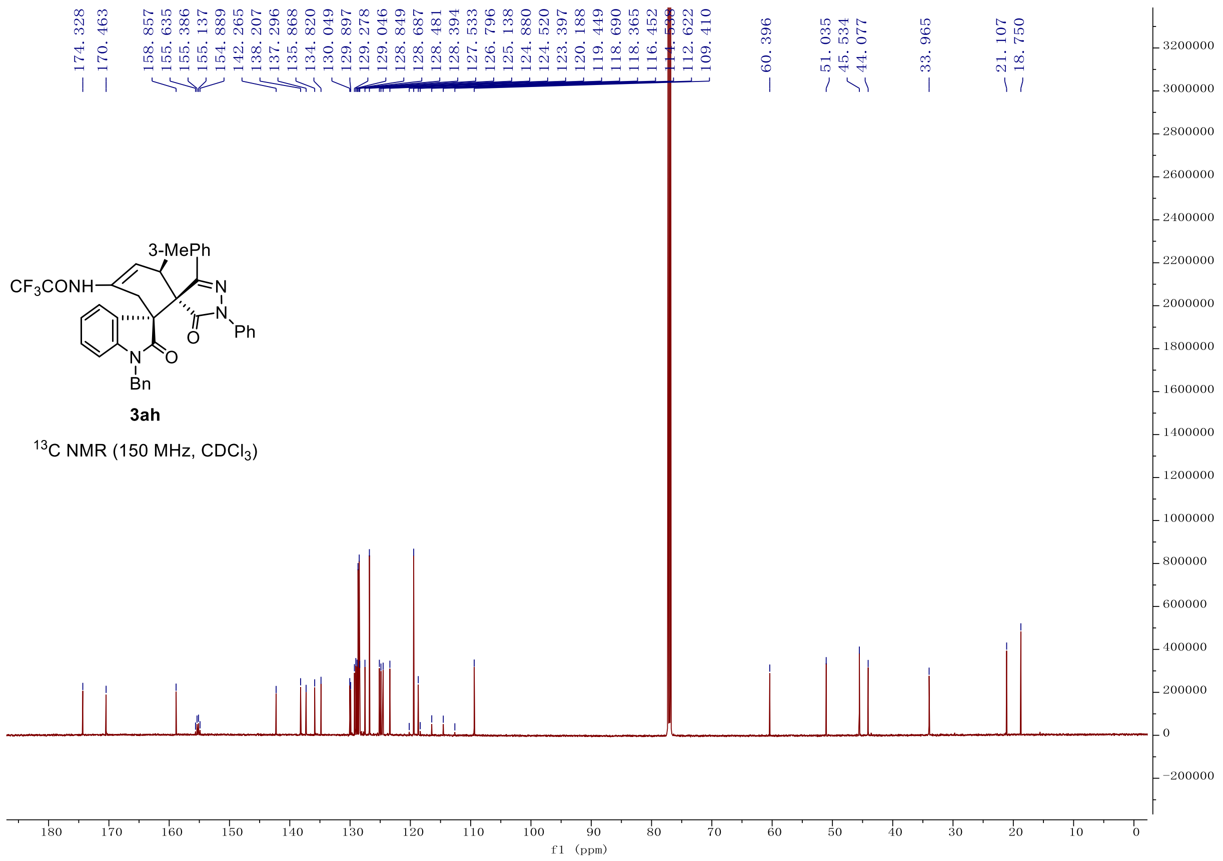

3-MePh
CF3CONH
N
N
Ph
O
N
O
Bn
3ah
13C NMR (150 MHz, CDCl3)
f1 (ppm)

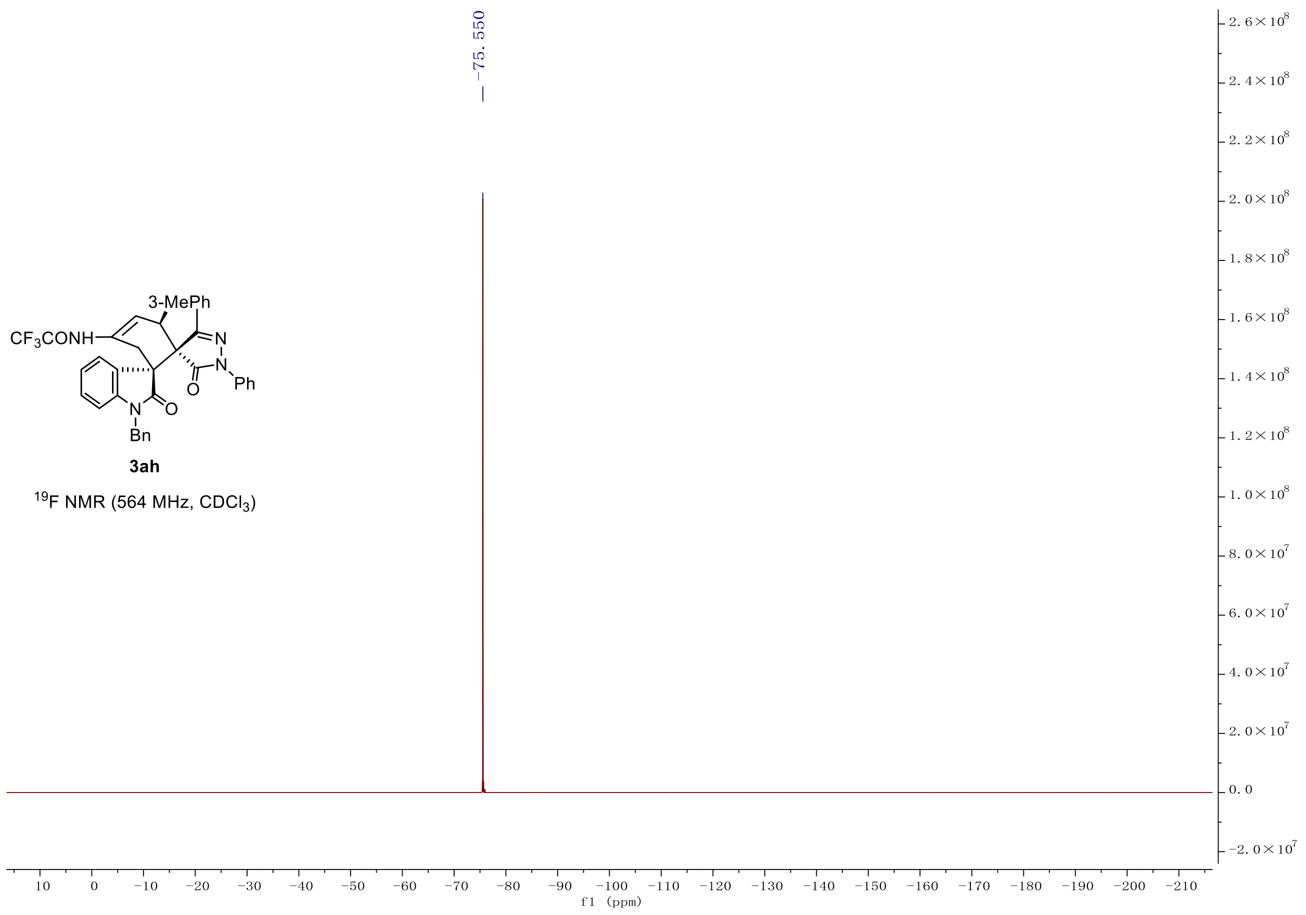

Peak Analysis Report

Detector A Channel 1 254nm

No.	Ret. Time	Height (mAu)	Area (mAu*min)	Rel. Area (%)
1	29.151	39684	3593193	50.300
2	33.160	36397	3550364	49.700
Total		76080	7143557	100.000

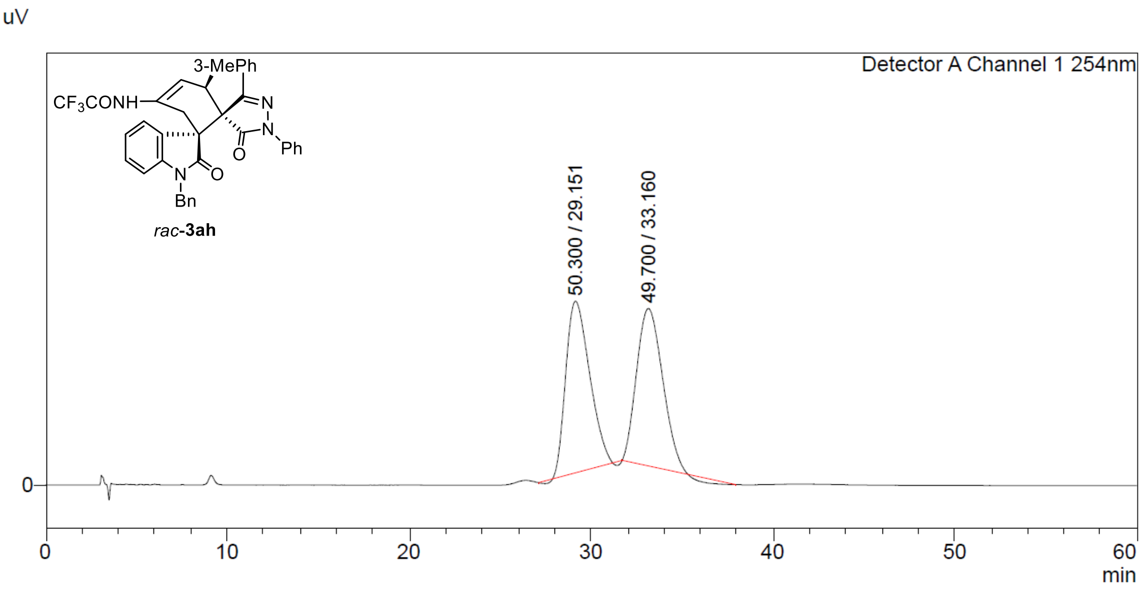

Peak Analysis Report

Detector A Channel 1 254nm

No.	Ret. Time	Height (mAu)	Area (mAu*min)	Rel. Area (%)
1	30.094	43	3343	0.017
2	32.910	176412	19531985	99.983
Total		176455	19535328	100.000

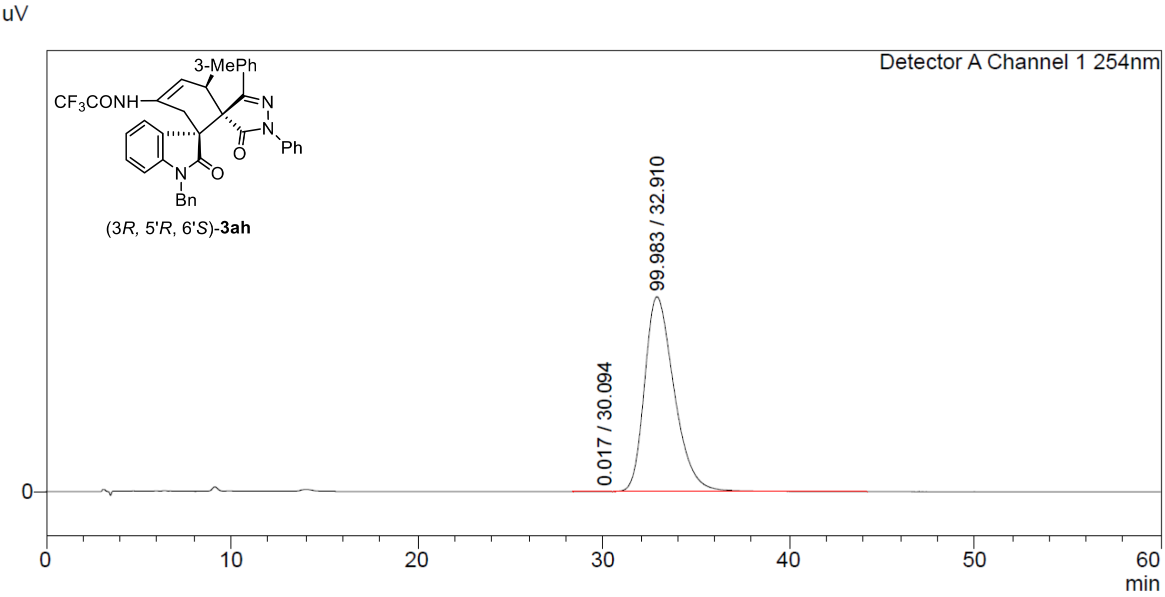

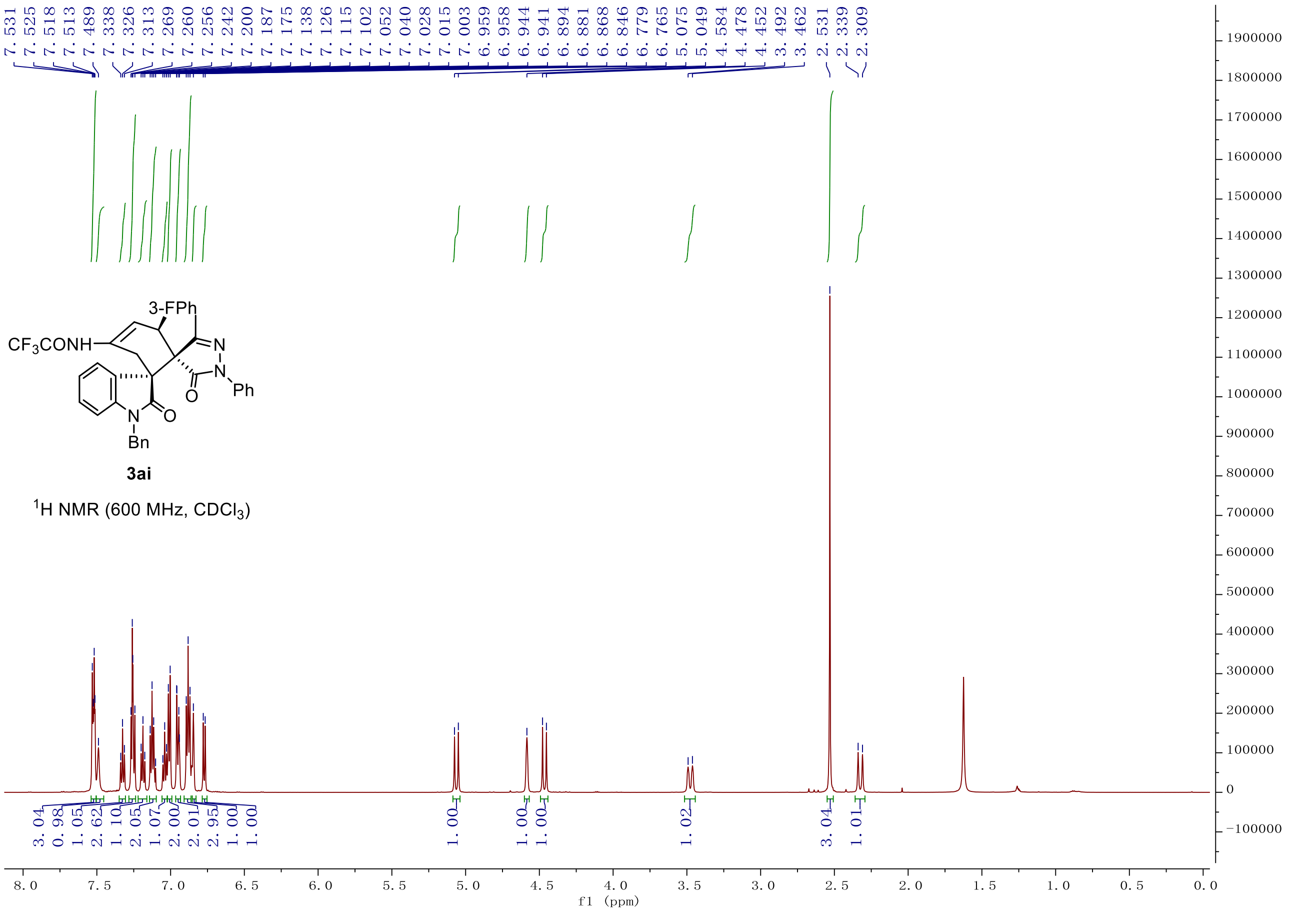

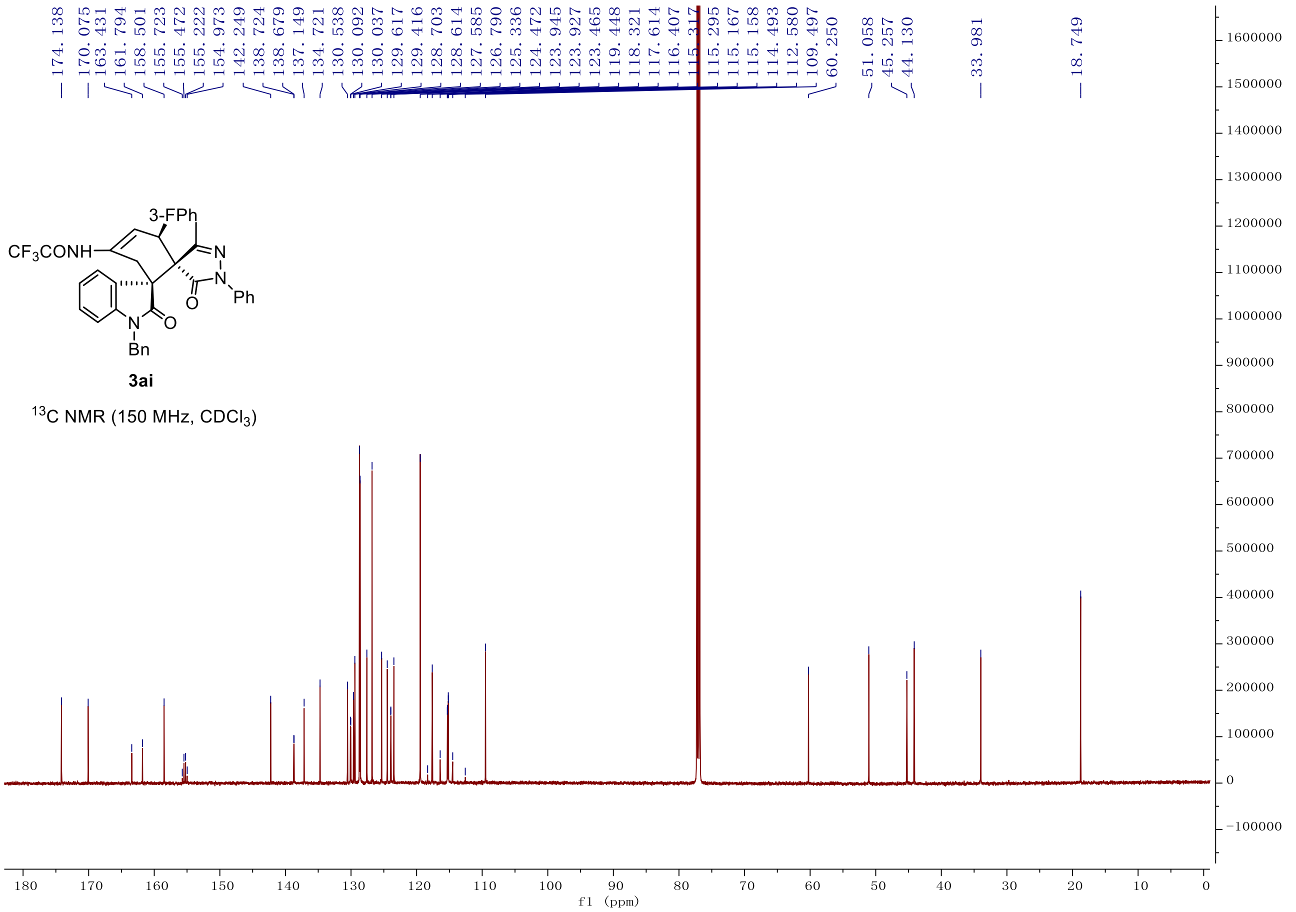

174.138
170.075
163.431
161.794
158.501
155.723
155.472
155.222
154.973
142.249
138.724
138.679
137.149
134.721
130.538
130.092
130.037
129.617
129.416
128.703
128.614
127.585
126.790
125.336
124.472
123.945
123.927
123.465
119.448
118.321
117.614
116.407
115.317
115.295
115.167
115.158
114.493
112.580
109.497
60.250
51.058
45.257
44.130
33.981
18.749
3-FPh
CF3CONH
N
N
Ph
O
N
O
Bn
3ai
13C NMR (150 MHz, CDCl3)
f1 (ppm)

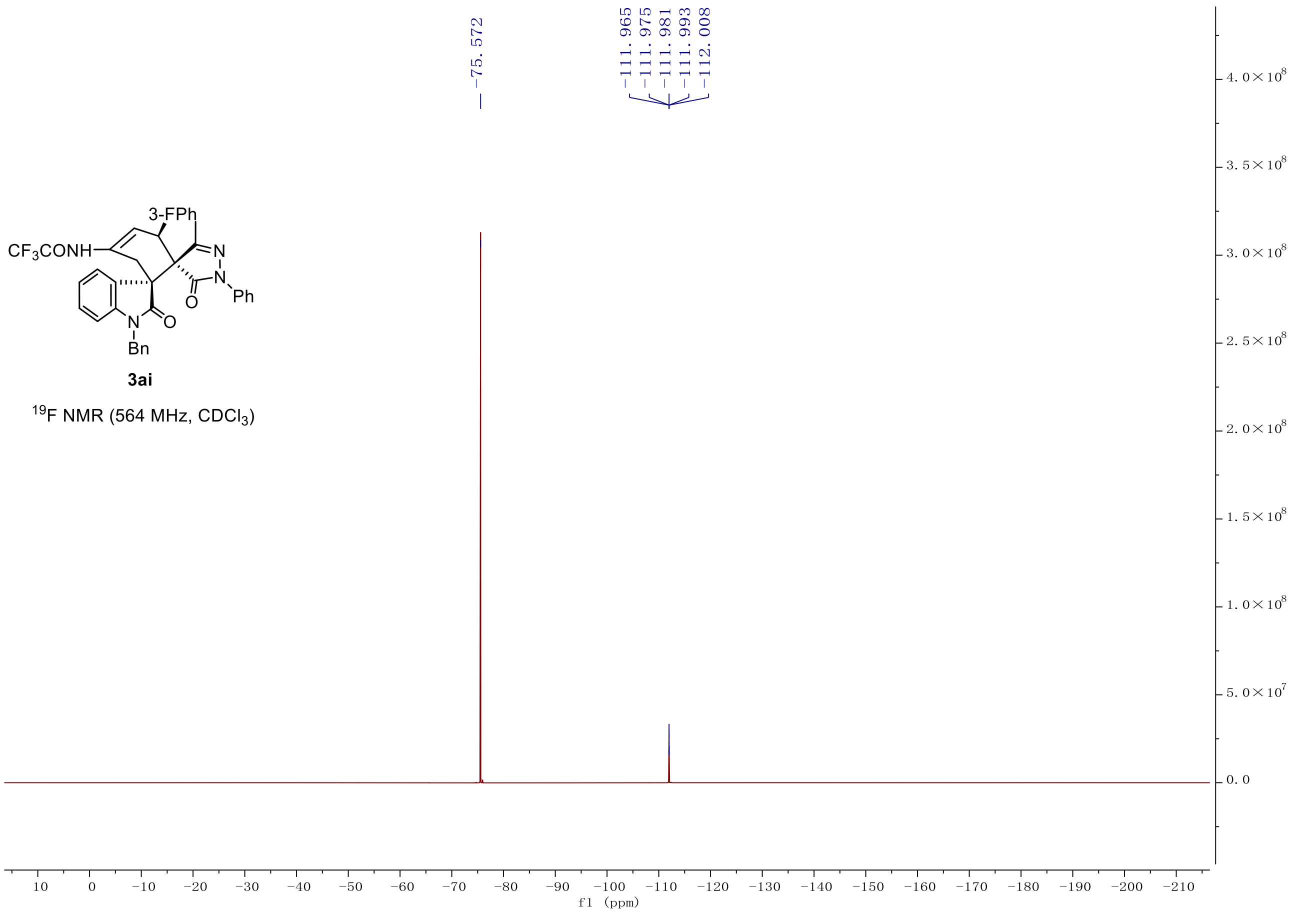

-75.572
-111.965
-111.975
-111.981
-111.993
-112.008
3-FPh
CF3CONH
N
N
Ph
O
N
O
Bn
3ai
19F NMR (564 MHz, CDCl3)
f1 (ppm)

Signal: VWD1 B, Wavelength=254 nm

RT [min]	Type	Width [min]	Area	Height	Area%	Name
6.775	VV R	0.3216	7196.5142	341.9181	50.0622	
8.966	VB	0.4981	7178.6367	222.7019	49.9378	
		Sum	14375.1509			

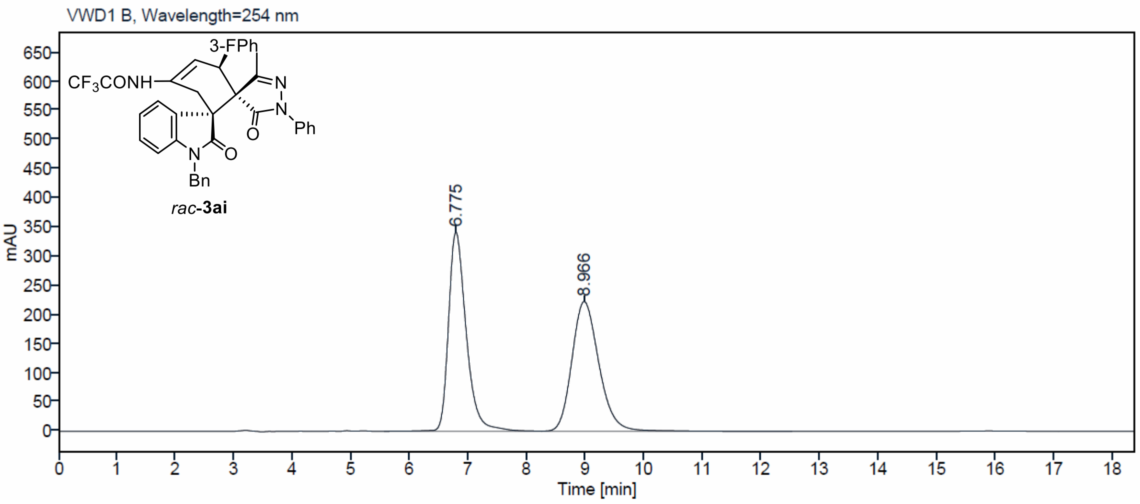

Signal: VWD1 B, Wavelength=254 nm

RT [min]	Type	Width [min]	Area	Height	Area%	Name
6.808	MM	0.3821	436.2452	19.0267	0.6286	
8.863	MM	0.5234	68966.9297	2196.0613	99.3714	
		Sum	69403.1749			

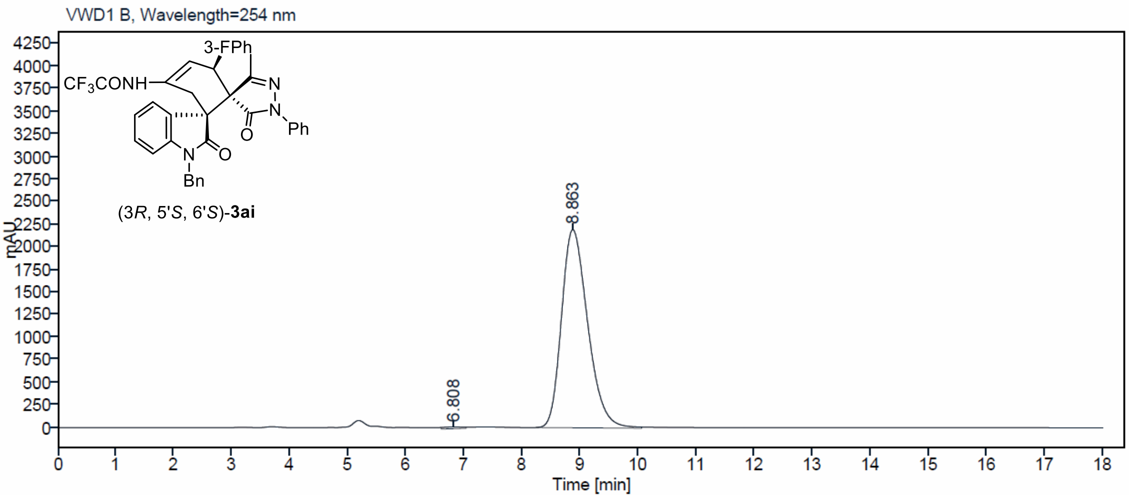

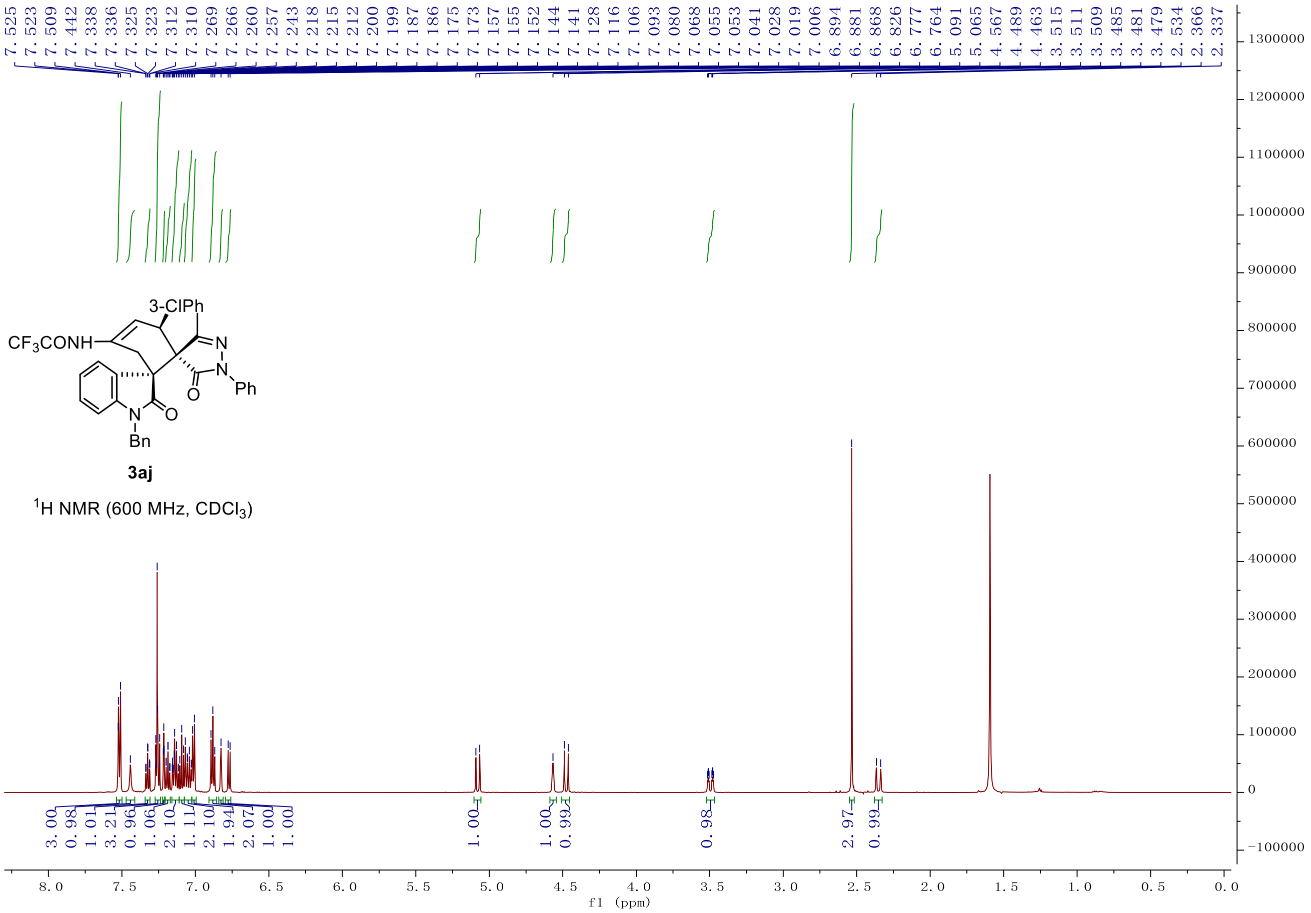
7.525 7.523 7.509 7.442 7.338 7.336 7.325 7.323 7.312 7.310 7.269 7.266 7.260 7.257 7.243 7.218 7.215 7.212 7.200 7.199 7.187 7.186 7.175 7.173 7.157 7.155 7.152 7.144 7.141 7.128 7.116 7.106 7.093 7.080 7.068 7.055 7.053 7.041 7.028 7.019 7.006 6.894 6.881 6.868 6.826 6.777 6.764 5.091 5.065 4.567 4.489 4.463 3.515 3.511 3.509 3.485 3.481 2.534 2.366 2.337
3-ClPh
CF3CONH
N
N
Ph
O
O
N
Bn
3aj
1H NMR (600 MHz, CDCl3)
3.00 0.98 1.01 3.21 0.96 1.06 2.10 1.11 2.10 1.94 2.07 1.00 1.00 1.00 1.00 0.99 0.98 2.97 0.99
f1 (ppm)

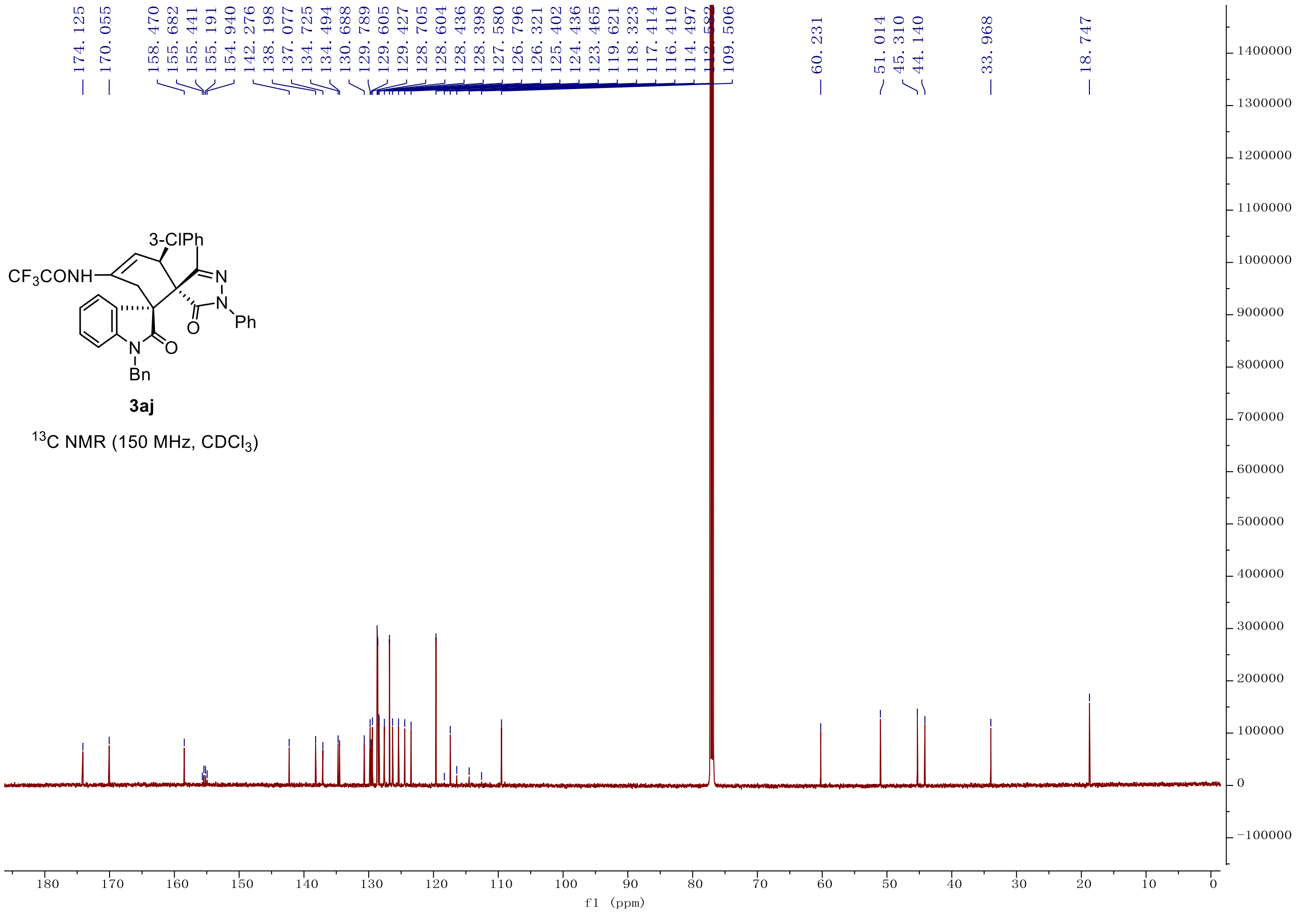
174.125 170.055 158.470 155.682 155.441 155.191 154.940 142.276 138.198 137.077 134.725 134.494 130.688 129.789 129.605 129.427 128.705 128.604 128.436 128.398 127.580 126.796 126.321 125.402 124.436 123.465 119.621 118.323 117.414 116.410 114.497 109.506 60.231 51.014 45.310 44.140 33.968 18.747
3-ClPh
CF3CONH
N
N
Ph
O
O
N
Bn
3aj
13C NMR (150 MHz, CDCl3)
f1 (ppm)

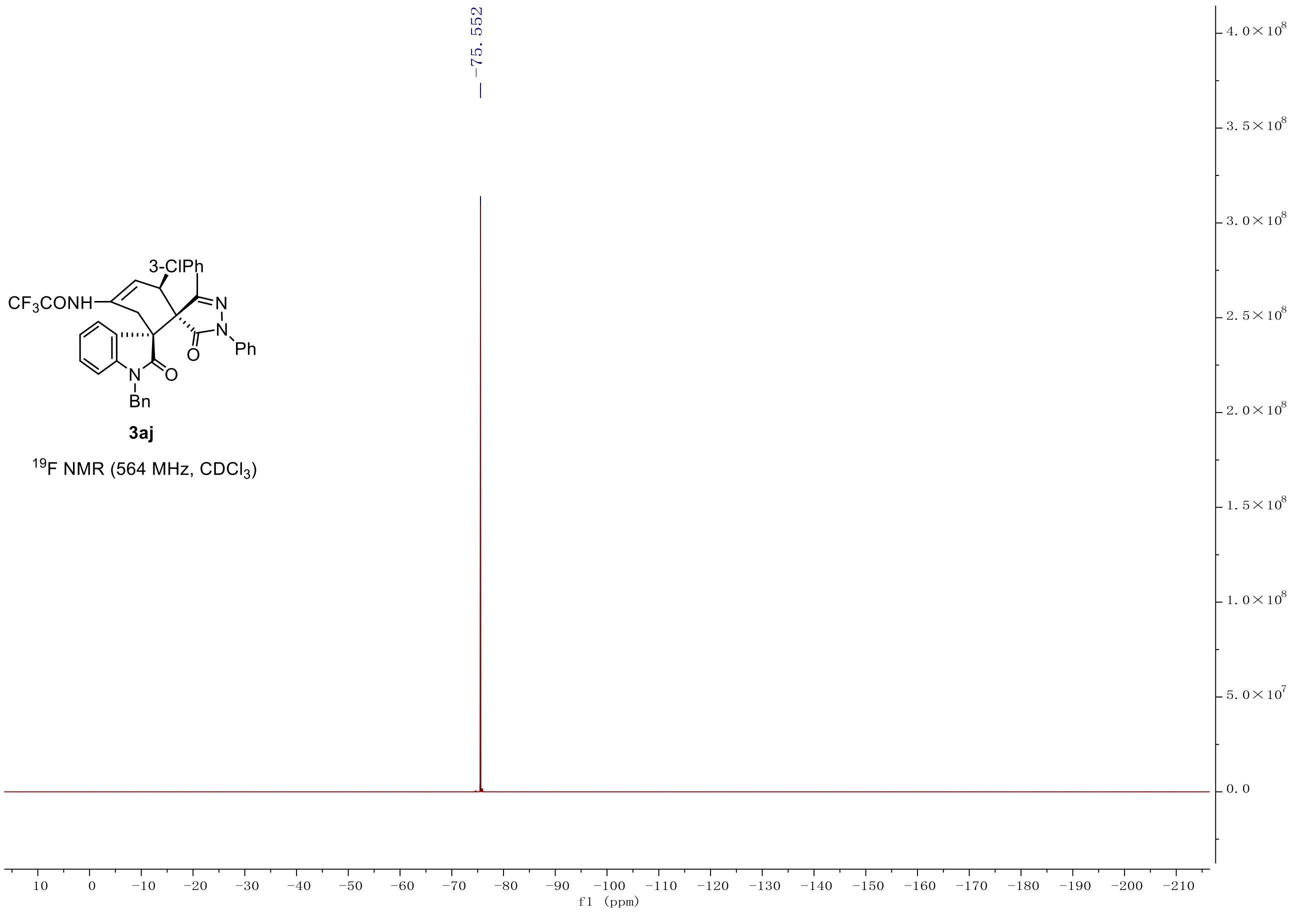

Signal: VWD1 B, Wavelength=254 nm

RT [min]	Type	Width [min]	Area	Height	Area%	Name
6.046	MM	0.2723	3755.7942	229.8593	50.7917	
6.827	MM	0.2167	3638.7083	279.8285	49.2083	
		Sum	7394.5024			

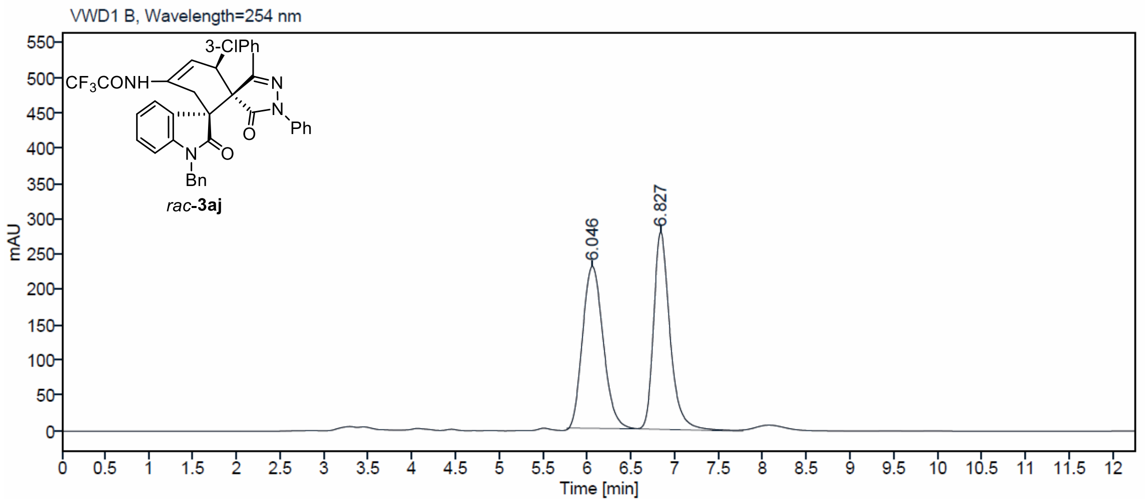

Signal: VWD1 B, Wavelength=254 nm

RT [min]	Type	Width [min]	Area	Height	Area%	Name
6.031	MM	0.2347	12835.7852	911.4442	99.7901	
6.889	MM	0.1410	26.9969	3.1900	0.2099	
		Sum	12862.7820			

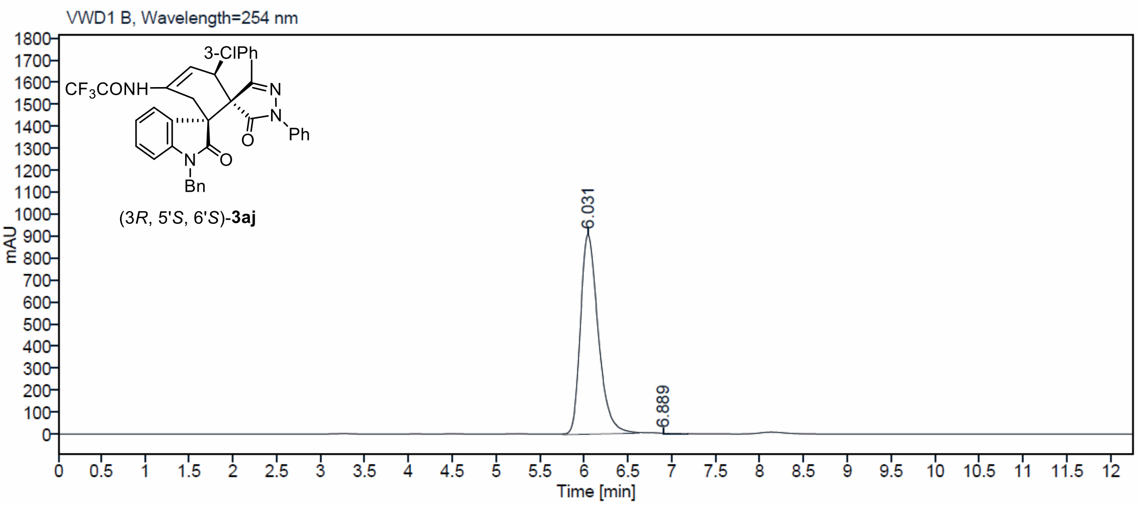

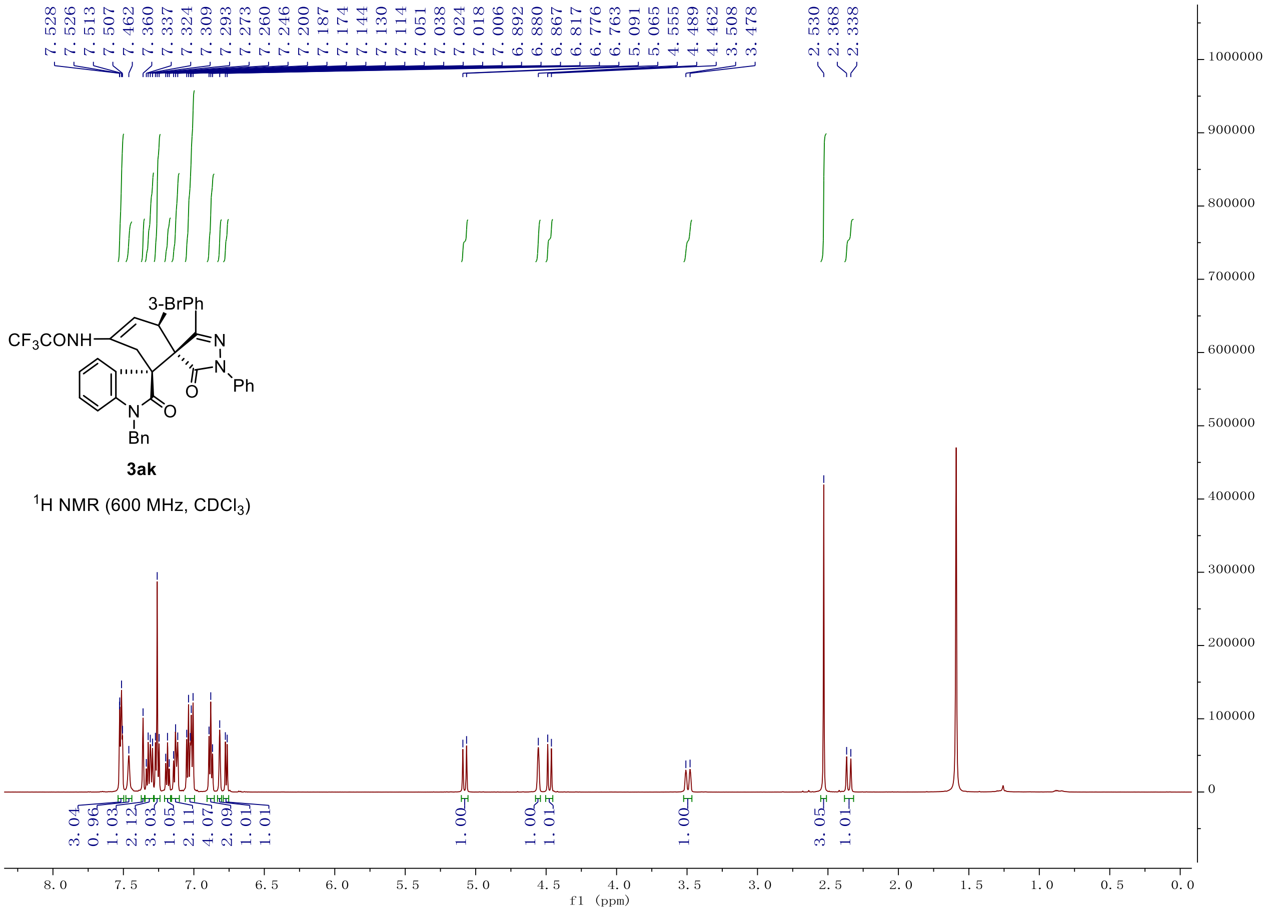

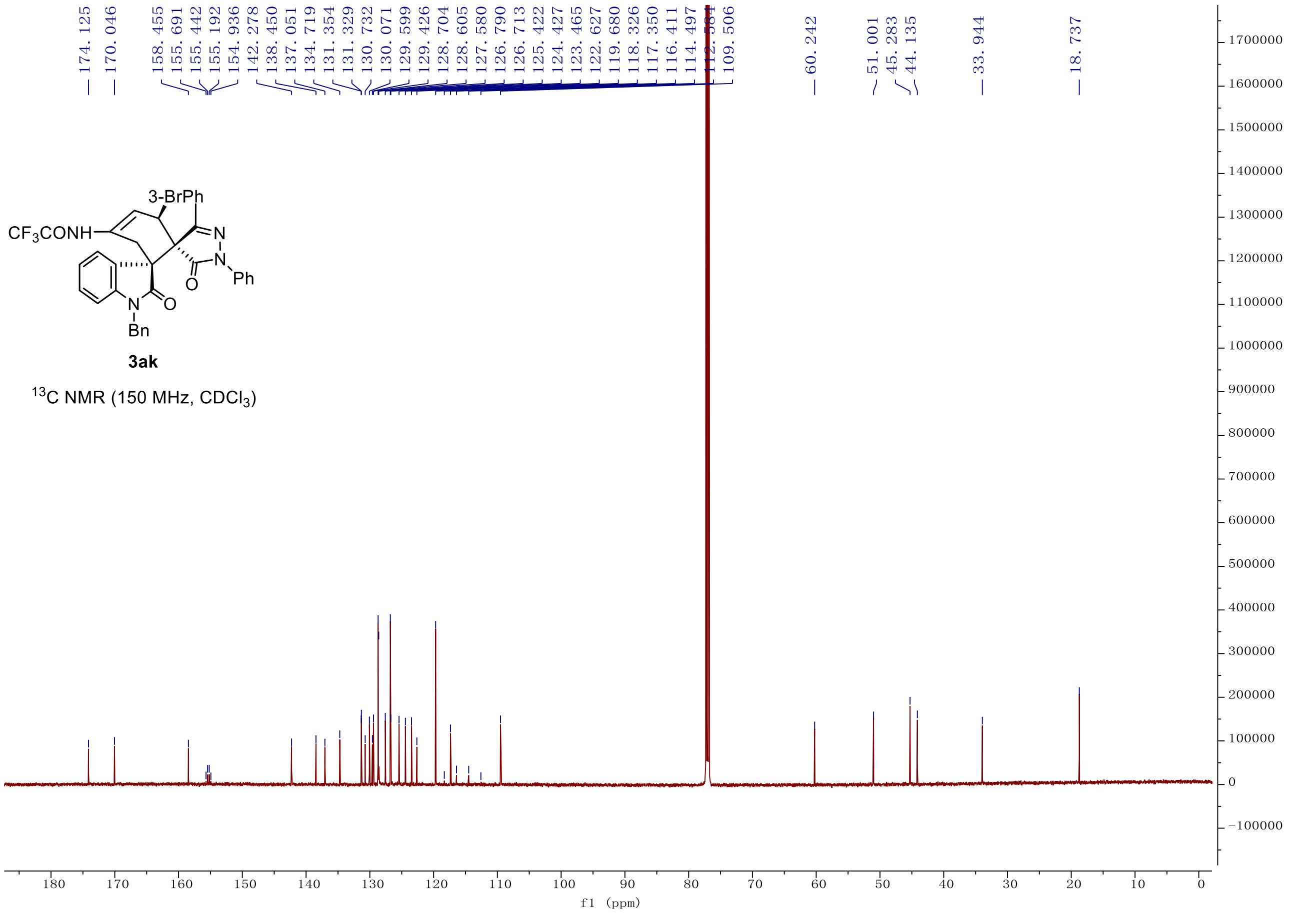
174.125
170.046
158.455
155.691
155.442
155.192
154.936
142.278
138.450
137.051
134.719
131.354
131.329
130.732
130.071
129.599
129.426
128.704
128.605
127.580
126.790
126.713
125.422
124.427
123.465
122.627
119.680
118.326
117.350
116.411
114.497
109.506
60.242
51.001
45.283
44.135
33.944
18.737
3-BrPh
CF3CONH
N
N
Ph
O
N
O
Bn
3ak
13C NMR (150 MHz, CDCl3)
f1 (ppm)

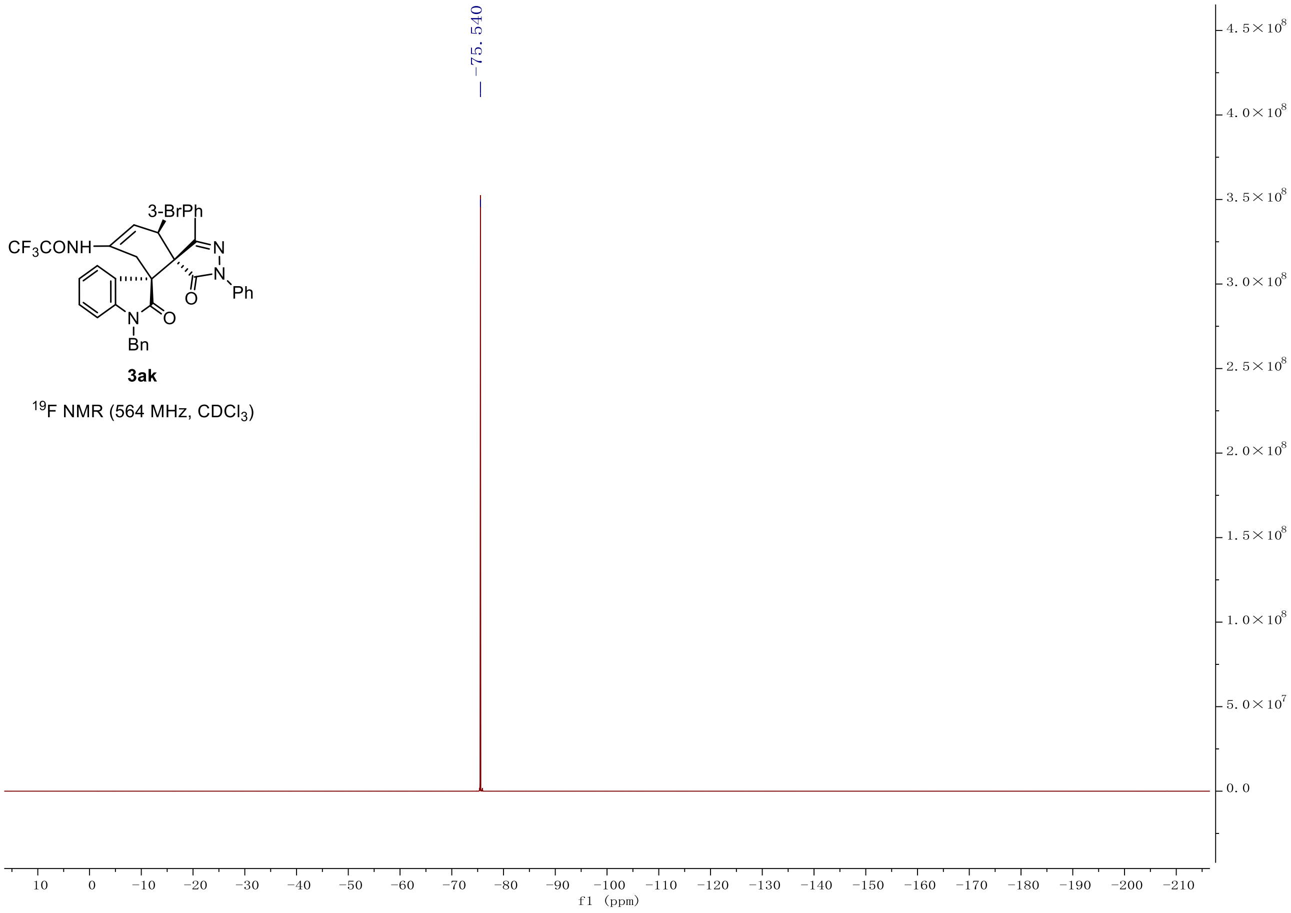
-75.540
3-BrPh
CF3CONH
N
N
Ph
O
N
O
Bn
3ak
19F NMR (564 MHz, CDCl3)
f1 (ppm)

Signal: VWD1 B, Wavelength=254 nm

RT [min]	Type	Width [min]	Area	Height	Area%	Name
6.157	MM	0.2289	6388.5879	465.1016	50.2745	
6.916	MM	0.2114	6318.8354	498.1213	49.7255	
		Sum	12707.4233			

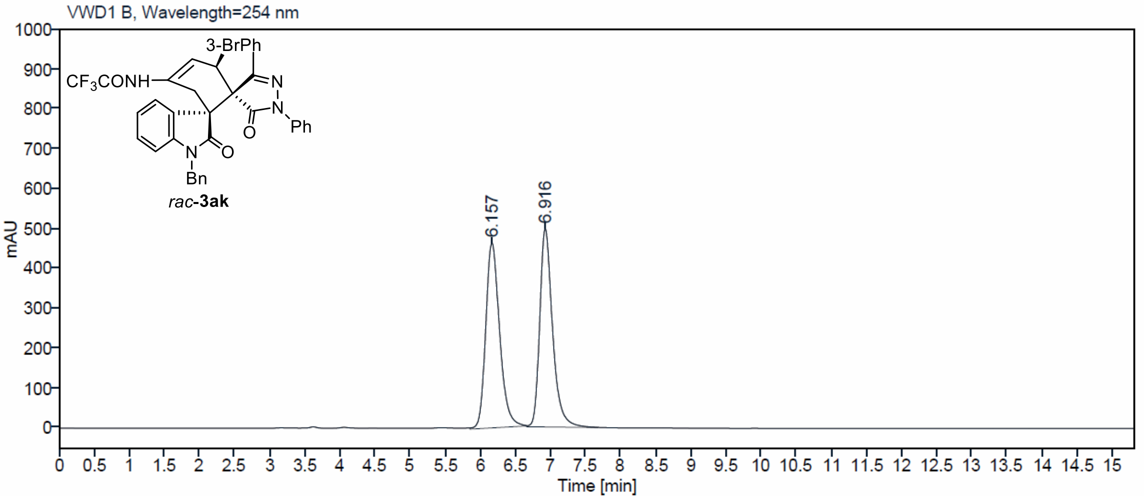

Signal: VWD1 B, Wavelength=254 nm

RT [min]	Type	Width [min]	Area	Height	Area%	Name
6.089	MM	0.2286	35370.0625	2578.5850	99.1337	
6.933	MM	0.3025	309.0965	17.0291	0.8663	
		Sum	35679.1590			

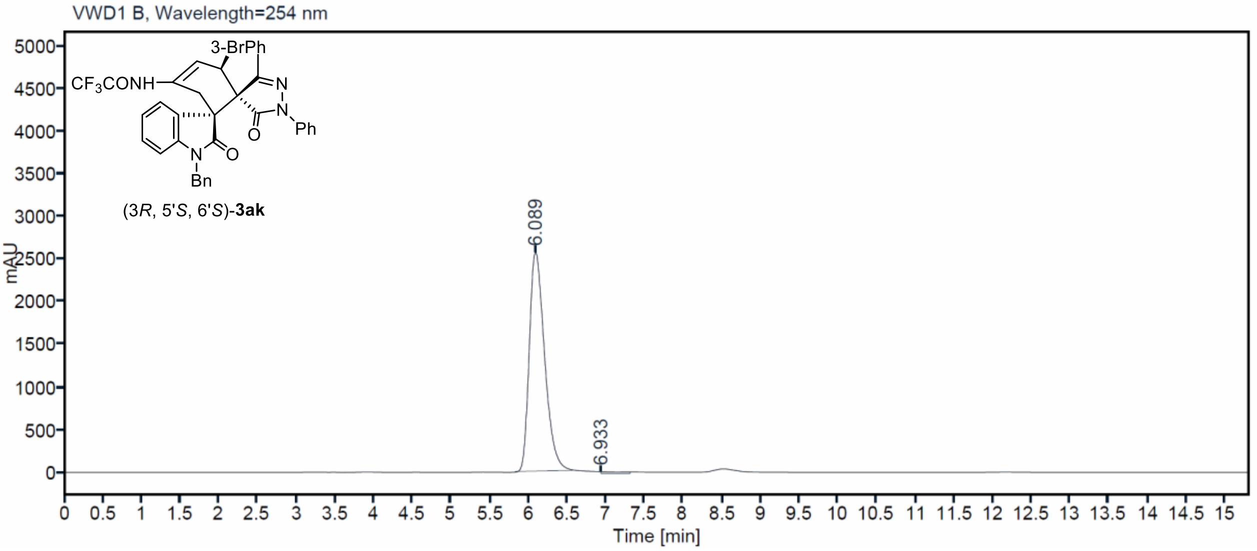

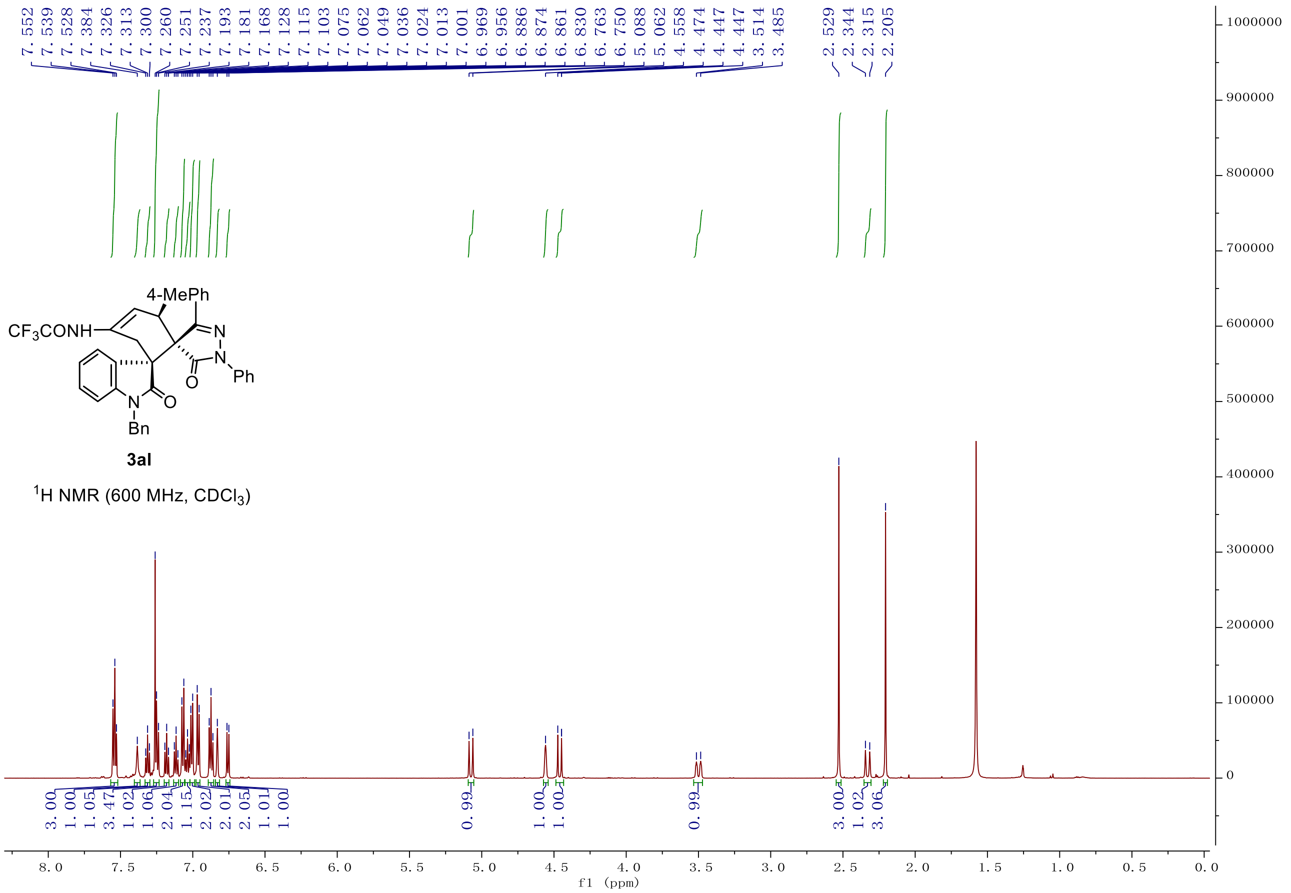
7.552 7.539 7.528 7.384 7.326 7.313 7.300 7.260 7.251 7.237 7.193 7.181 7.168 7.128 7.115 7.103 7.075 7.062 7.049 7.036 7.024 7.013 7.001 6.969 6.956 6.886 6.874 6.861 6.830 6.763 6.750 5.088 5.062 4.558 4.474 4.447 4.447 3.514 3.485
2.529 2.344 2.315 2.205
4-MePh
CF3CONH
N
N
Ph
O
N
O
Bn
3al
1H NMR (600 MHz, CDCl3)
3.00 1.00 1.05 3.47 1.02 1.06 2.04 1.15 2.02 2.01 2.05 1.01 1.00
0.99 1.00 1.00 0.99 3.00 1.02 3.06
f1 (ppm)

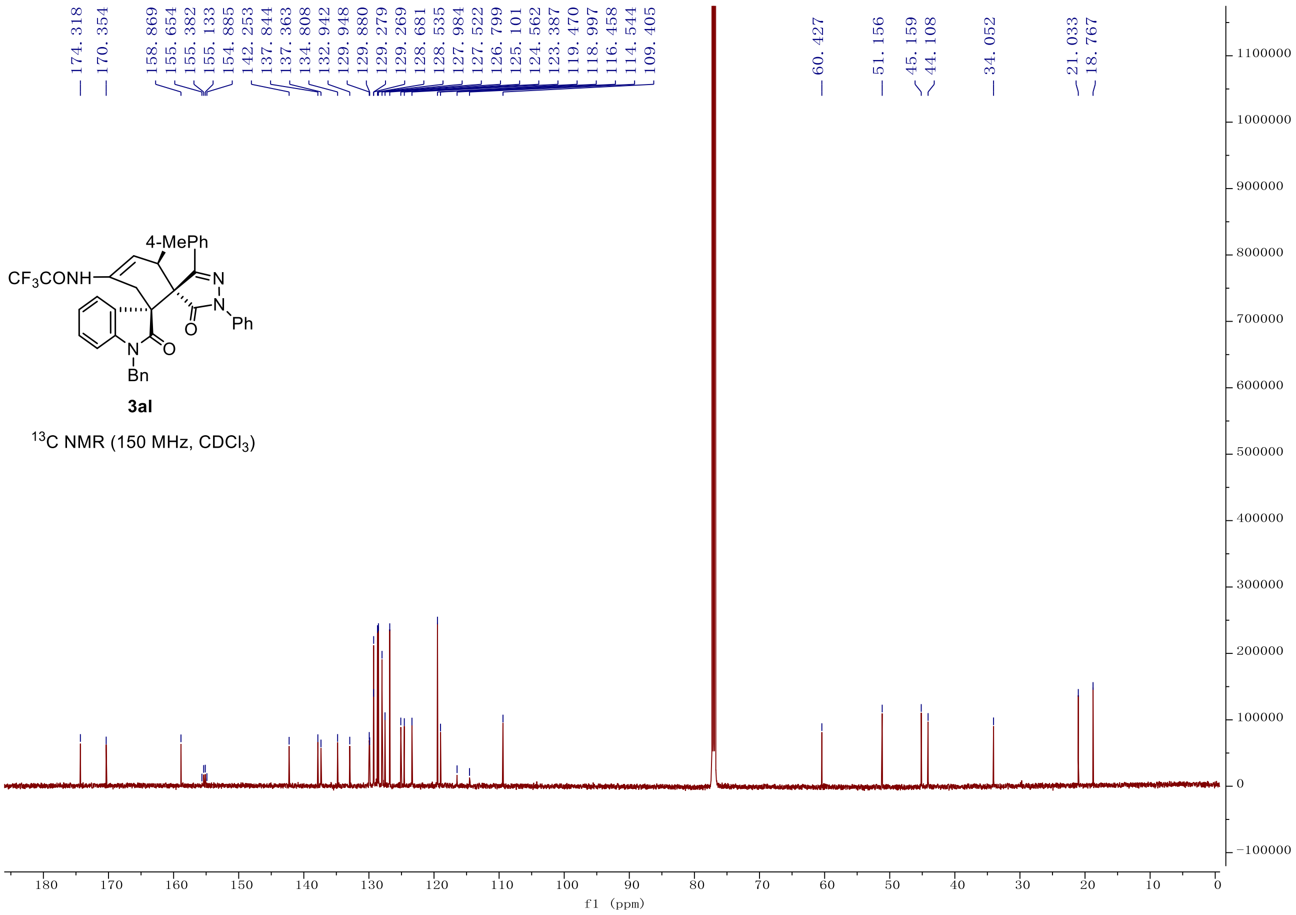
174.318 170.354 158.869 155.654 155.382 155.133 154.885 142.253 137.844 137.363 134.808 132.942 129.948 129.880 129.279 129.269 128.681 128.535 127.984 127.522 126.799 125.101 124.562 123.387 119.470 118.997 116.458 114.544 109.405
60.427 51.156 45.159 44.108 34.052 21.033 18.767
4-MePh
CF3CONH
N
N
Ph
O
N
O
Bn
3al
13C NMR (150 MHz, CDCl3)
f1 (ppm)

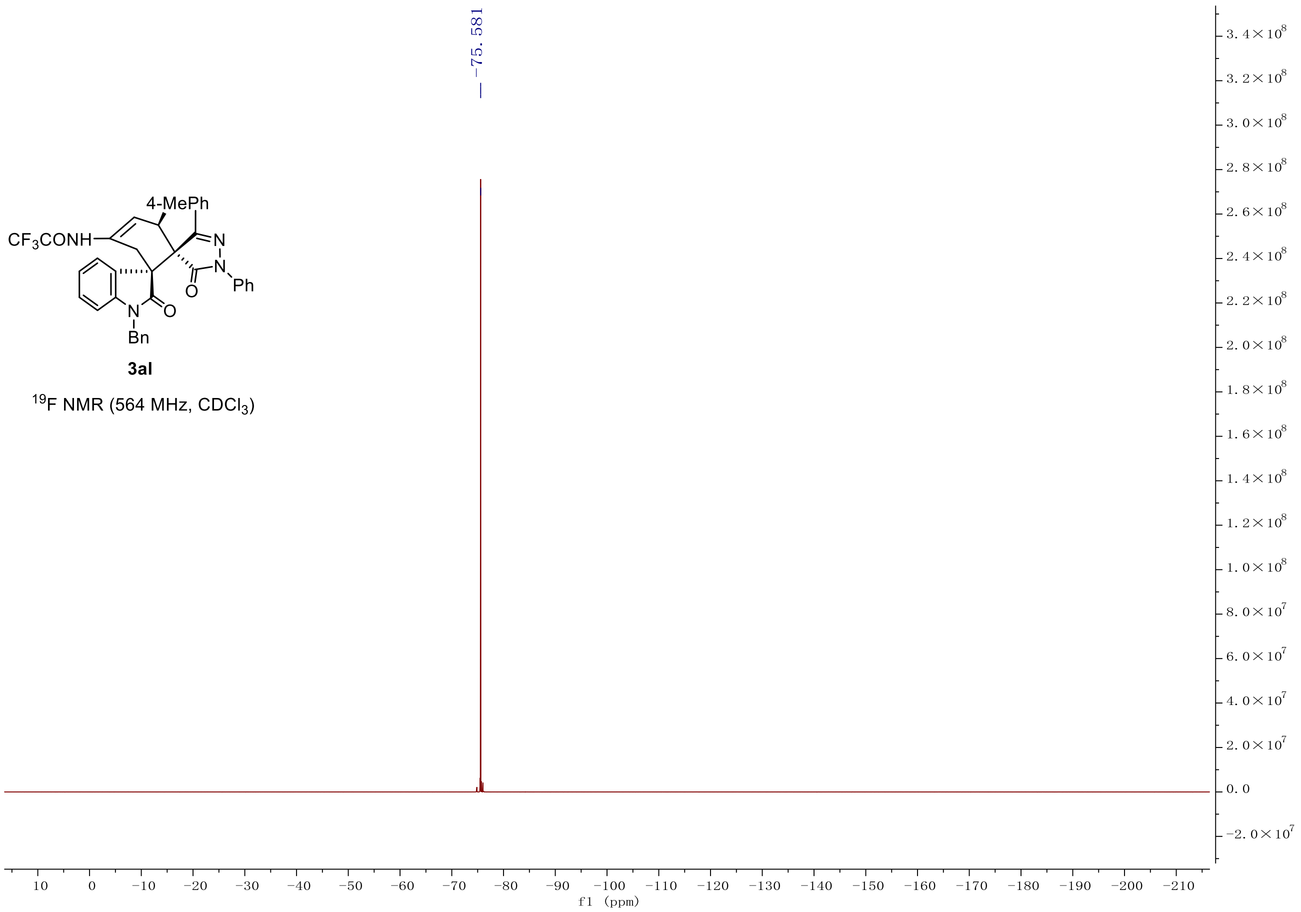

Peak Analysis Report

Detector A Channel 1 254nm

No.	Ret. Time	Height (mAu)	Area (mAu*min)	Rel. Area (%)
1	22.176	2278	196446	49.639
2	25.693	1964	199307	50.361
Total		4242	395753	100.000

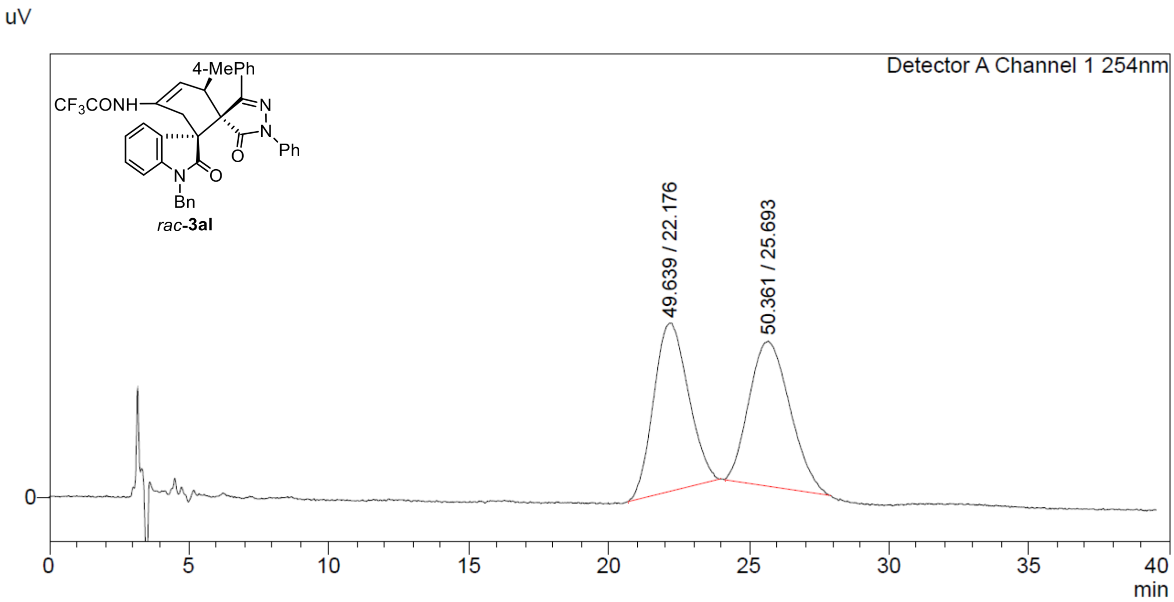

Peak Analysis Report

Detector A Channel 1 254nm

No.	Ret. Time	Height (mAu)	Area (mAu*min)	Rel. Area (%)
1	22.221	77	3982	0.044
2	25.524	82488	9110543	99.956
Total		82565	9114525	100.000

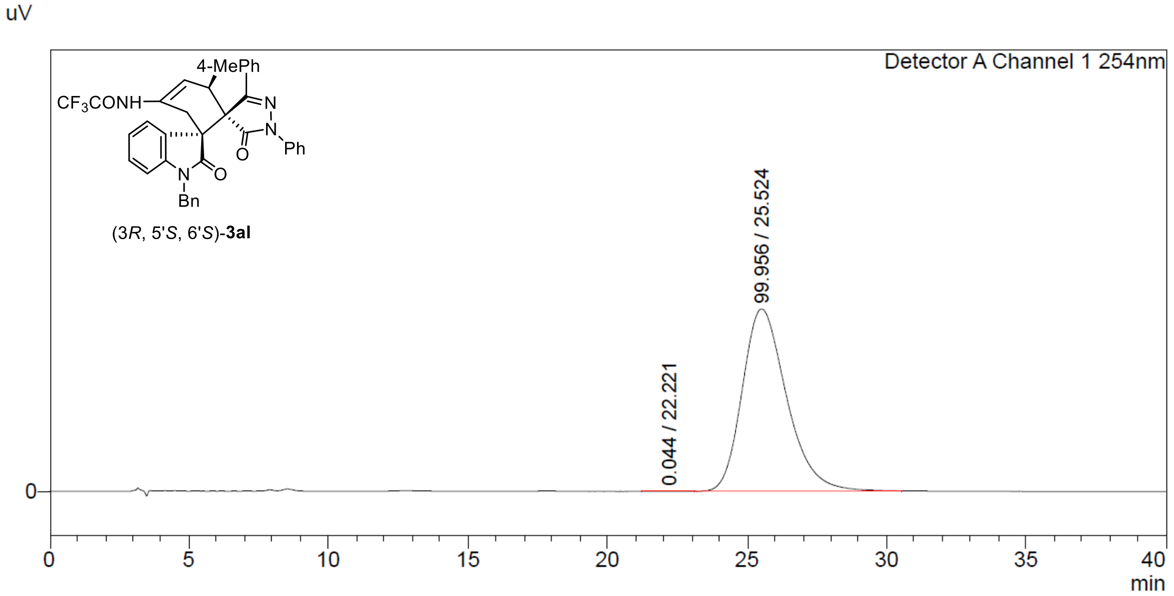

(3*R*, 5'*S*, 6'*S*)-**3al**

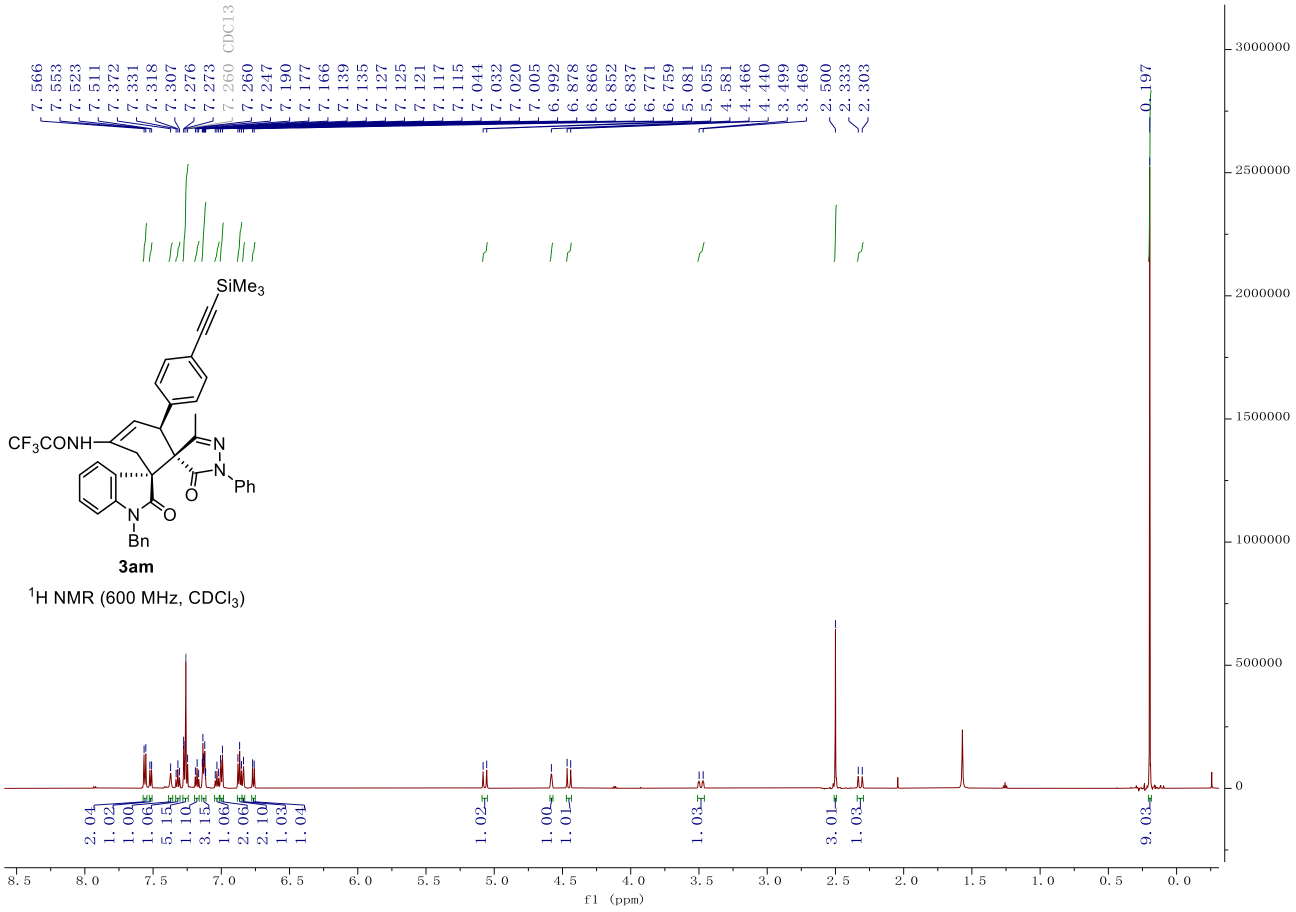

3am

^{1}H NMR (600 MHz, CDCl$_3$)

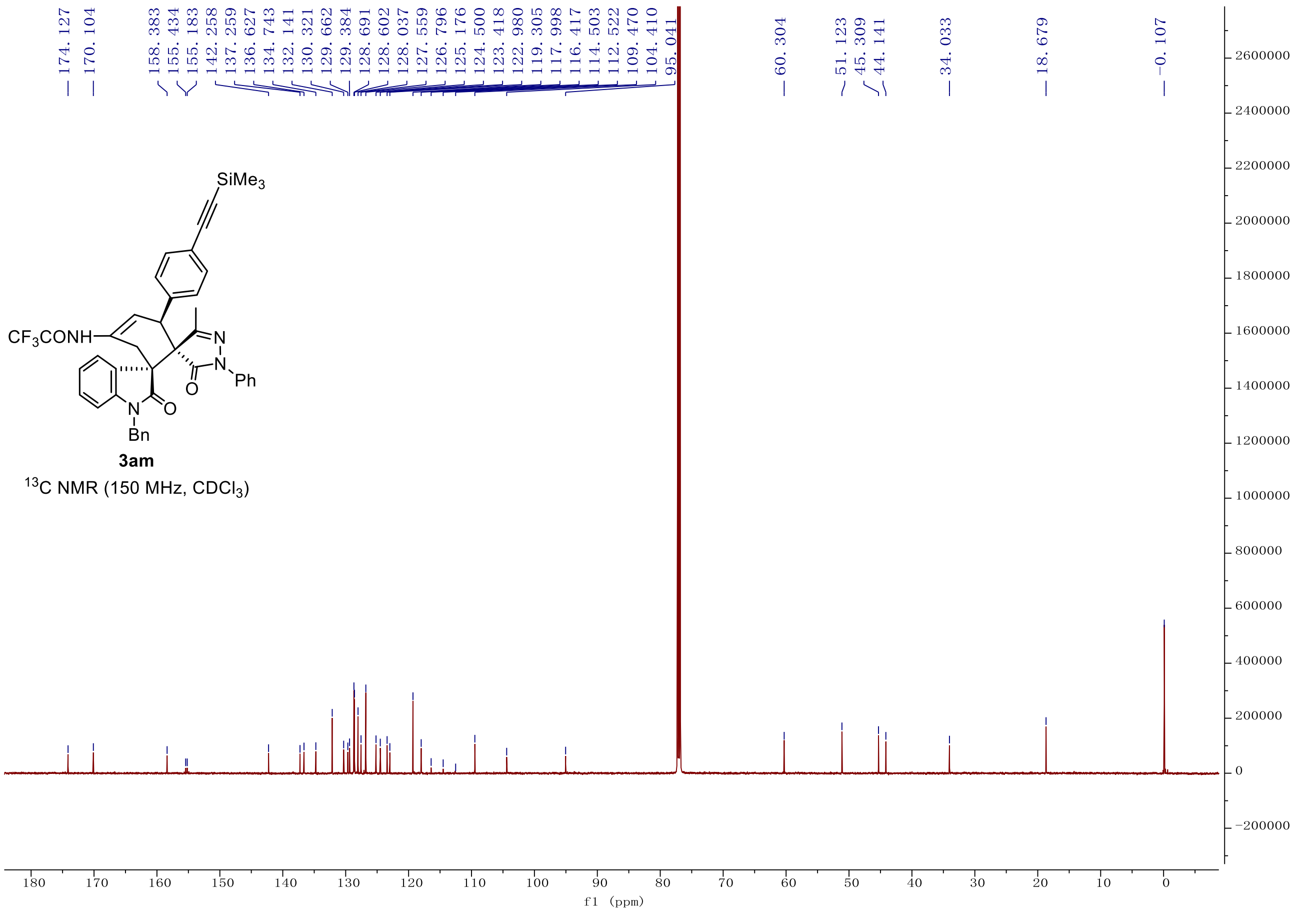

174.127
170.104
158.383
155.434
155.183
142.258
137.259
136.627
134.743
132.141
130.321
129.662
129.384
128.691
128.602
128.037
127.559
126.796
125.176
124.500
123.418
122.980
119.305
117.998
116.417
114.503
112.522
109.470
104.410
95.041
60.304
51.123
45.309
44.141
34.033
18.679
-0.107
SiMe3
CF3CONH
N
N
Ph
O
N
O
Bn
3am
13C NMR (150 MHz, CDCl3)
f1 (ppm)

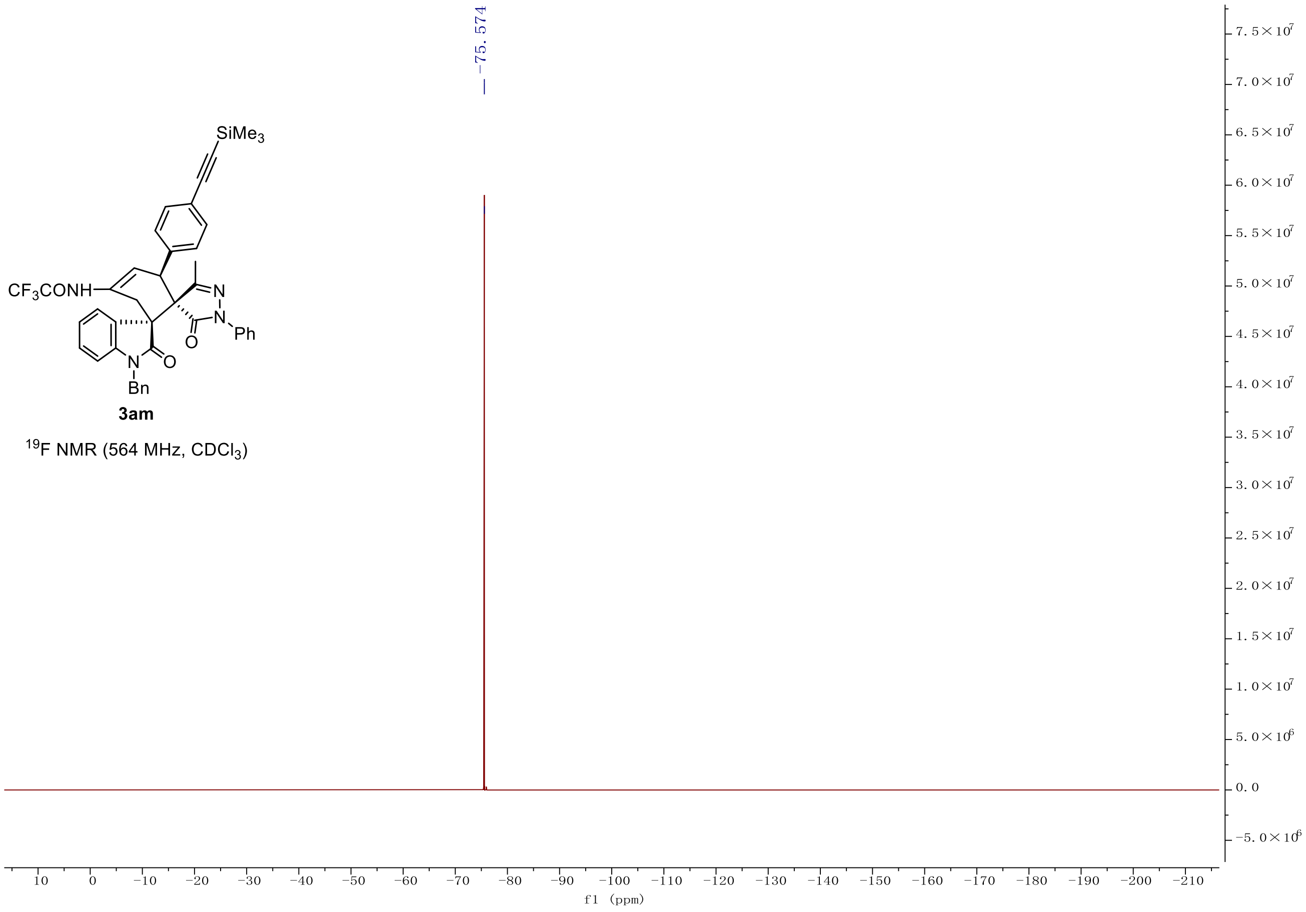

-75.574
SiMe3
CF3CONH
N
N
Ph
O
N
O
Bn
3am
19F NMR (564 MHz, CDCl3)
f1 (ppm)

Peak Analysis Report

Detector A Channel 1 254nm

No.	Ret. Time	Height (mAu)	Area (mAu*min)	Rel. Area (%)
1	3.762	708260	5943551	50.850
2	7.833	135742	5744861	49.150
Total		844002	11688412	100.000

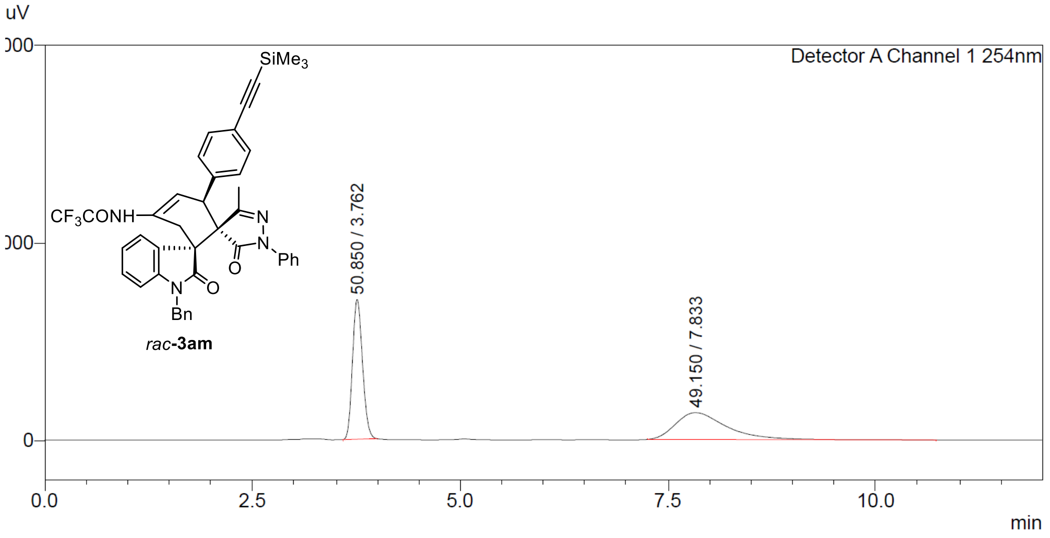

Peak Analysis Report

Detector A Channel 1 254nm

No.	Ret. Time	Height (mAu)	Area (mAu*min)	Rel. Area (%)
1	3.754	32195	277038	0.888
2	7.747	745728	30925035	99.112
Total		777922	31202073	100.000

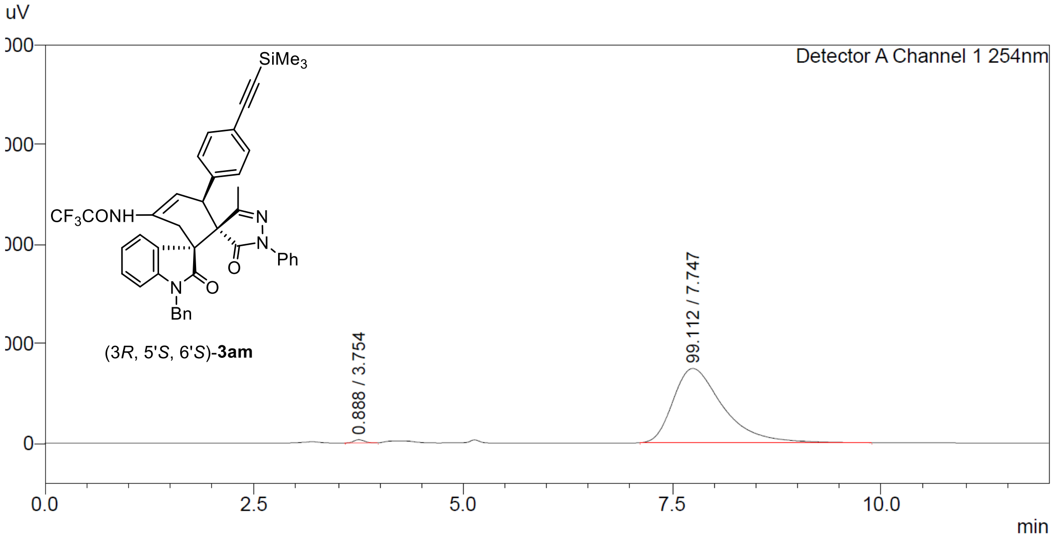

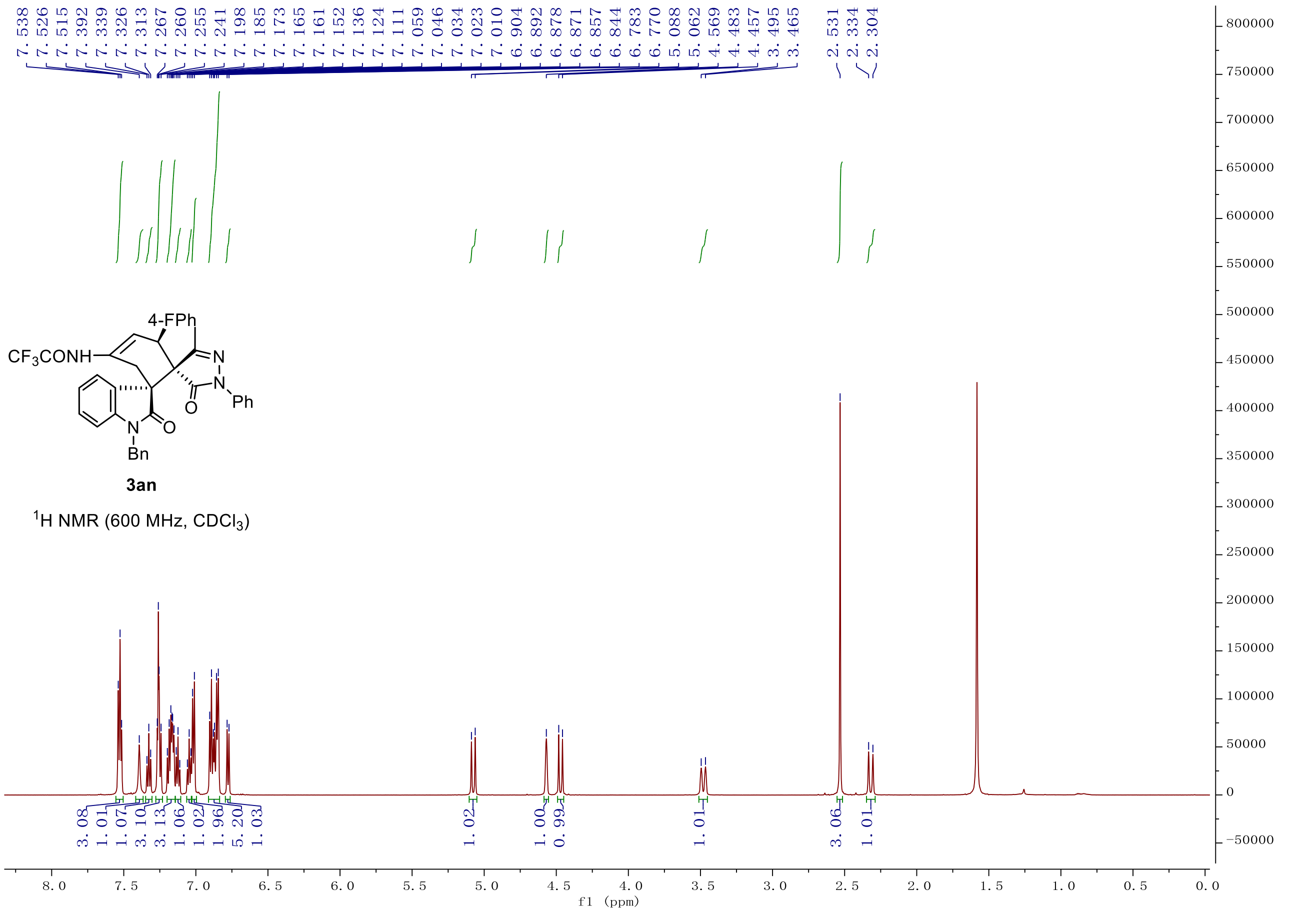

3an

^{1}H NMR (600 MHz, $CDCl_3$)

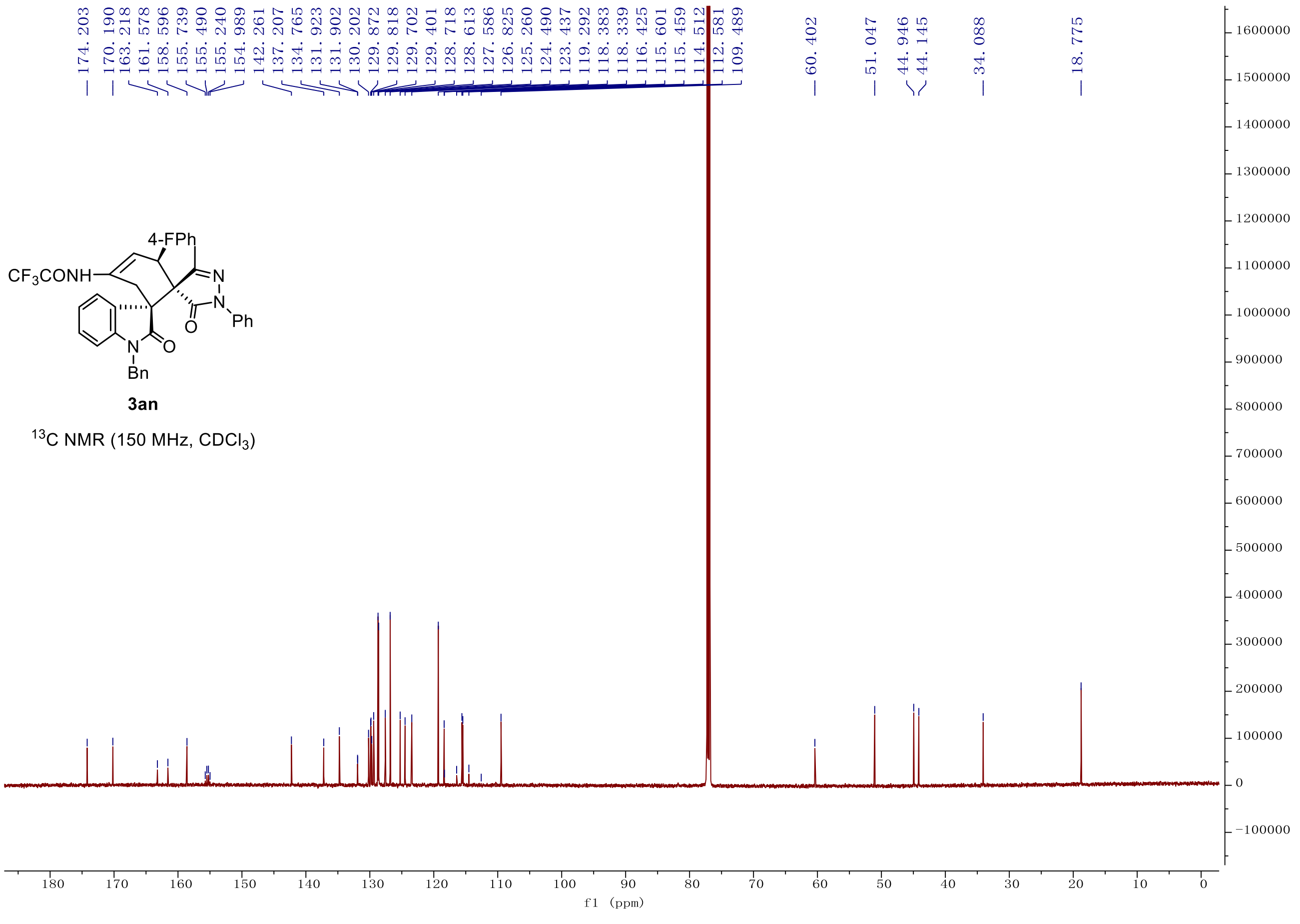

3an

^{13}C NMR (150 MHz, $CDCl_3$)

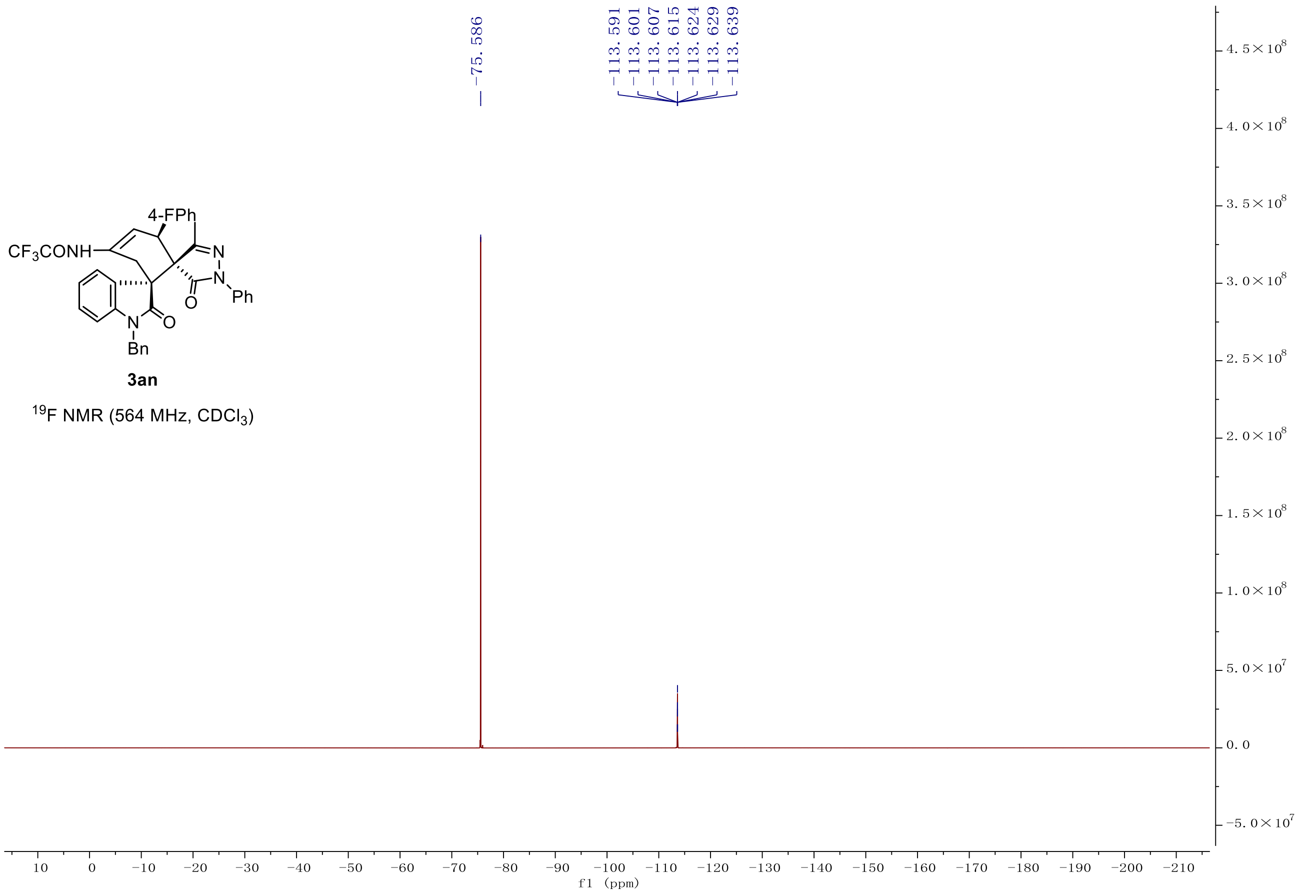

Signal: VWD1 B, Wavelength=254 nm

RT [min]	Type	Width [min]	Area	Height	Area%	Name
5.682	BB	0.2702	658.0364	37.5916	49.6318	
10.687	MM	0.6574	667.7986	16.9309	50.3682	
		Sum	1325.8350			

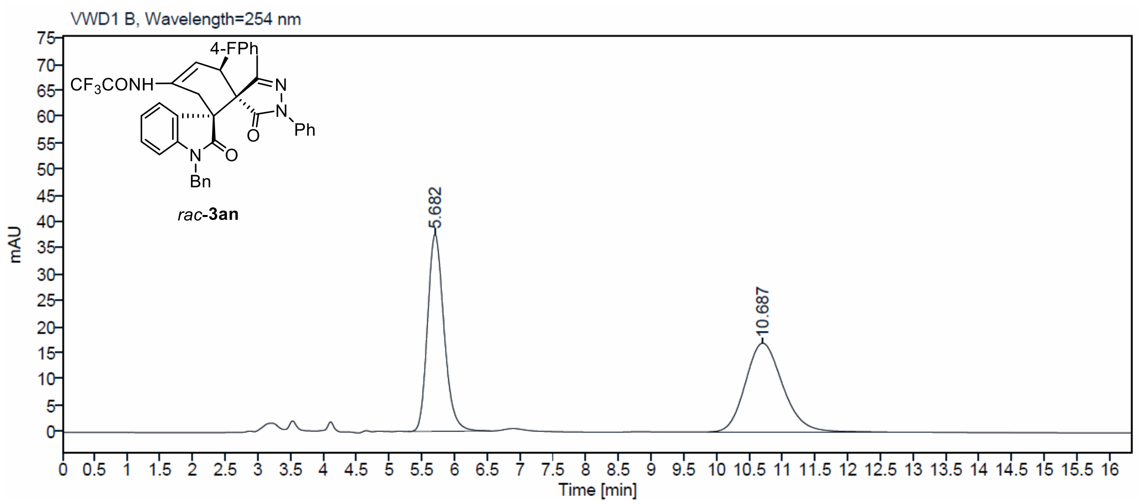

Signal: VWD1 B, Wavelength=254 nm

RT [min]	Type	Width [min]	Area	Height	Area%	Name
5.667	MM	0.2022	19.3437	1.5942	0.2684	
10.681	MM	0.6408	7187.1528	186.9324	99.7316	
		Sum	7206.4965			

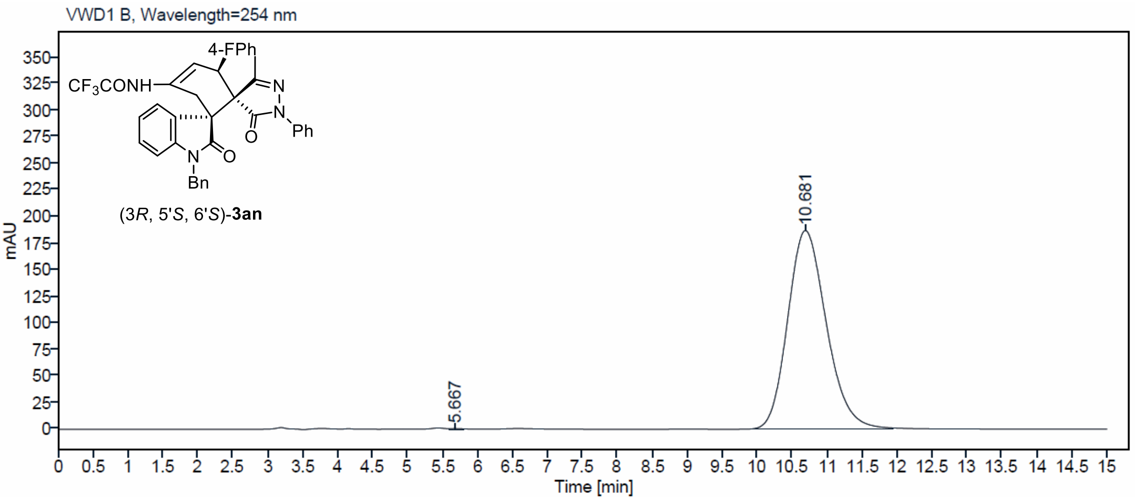

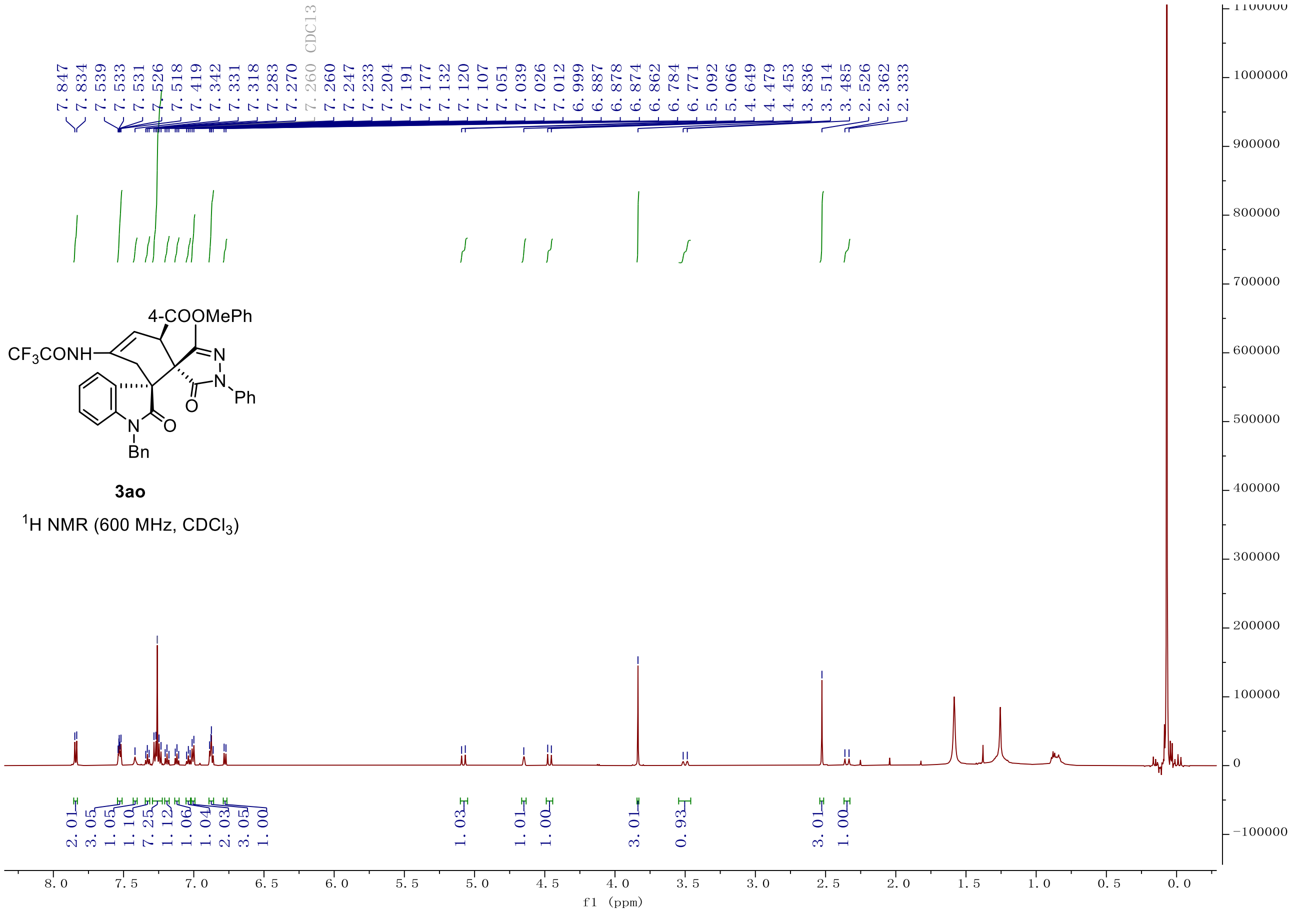

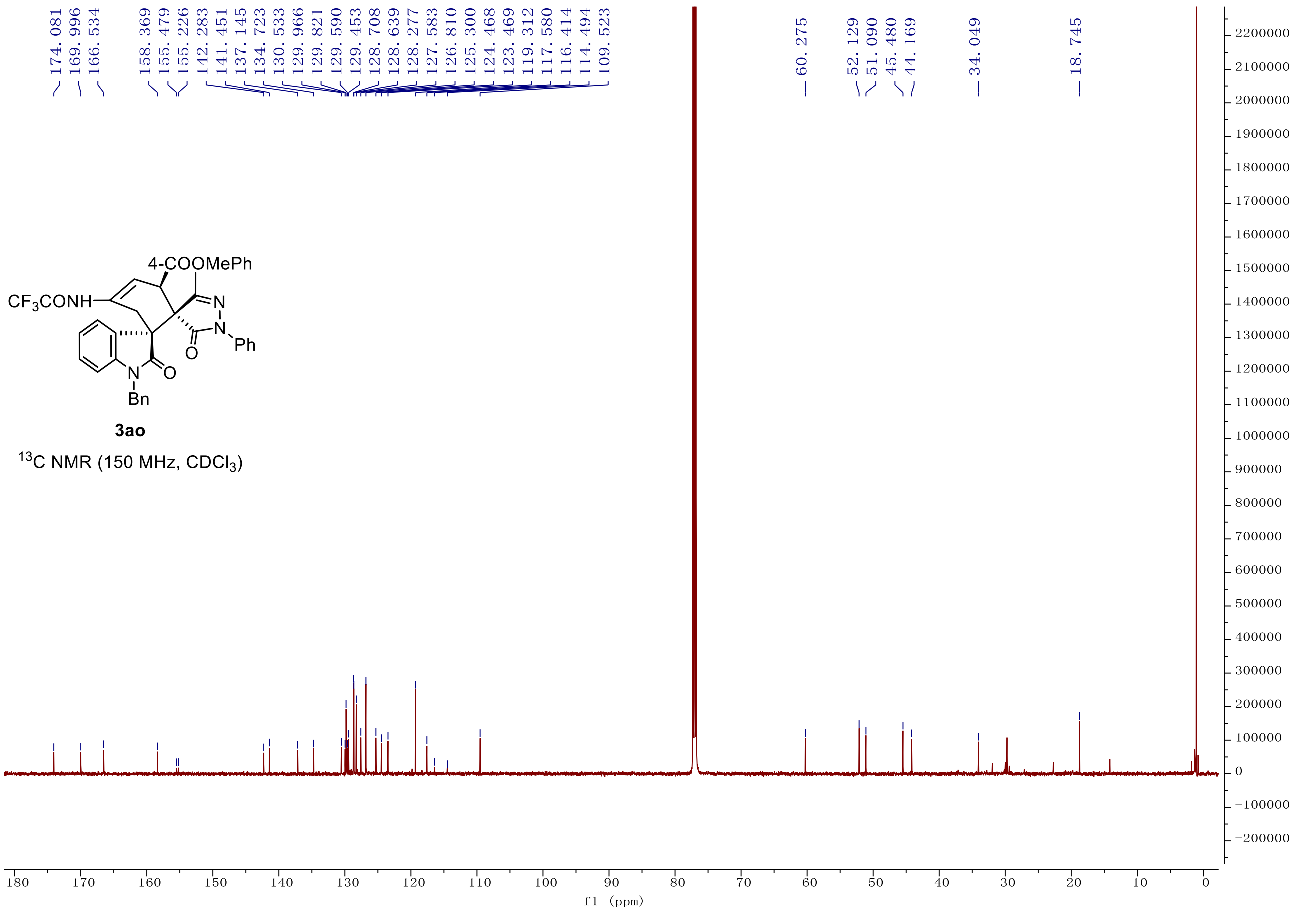
174.081
169.996
166.534
158.369
155.479
155.226
142.283
141.451
137.145
134.723
130.533
129.966
129.821
129.590
129.453
128.708
128.639
128.277
127.583
126.810
125.300
124.468
123.469
119.312
117.580
116.414
114.494
109.523
60.275
52.129
51.090
45.480
44.169
34.049
18.745
4-COOMePh
CF_3CONH
N
N
Ph
O
N
O
Bn
3ao
^{13}C NMR (150 MHz, $CDCl_3$)
f1 (ppm)

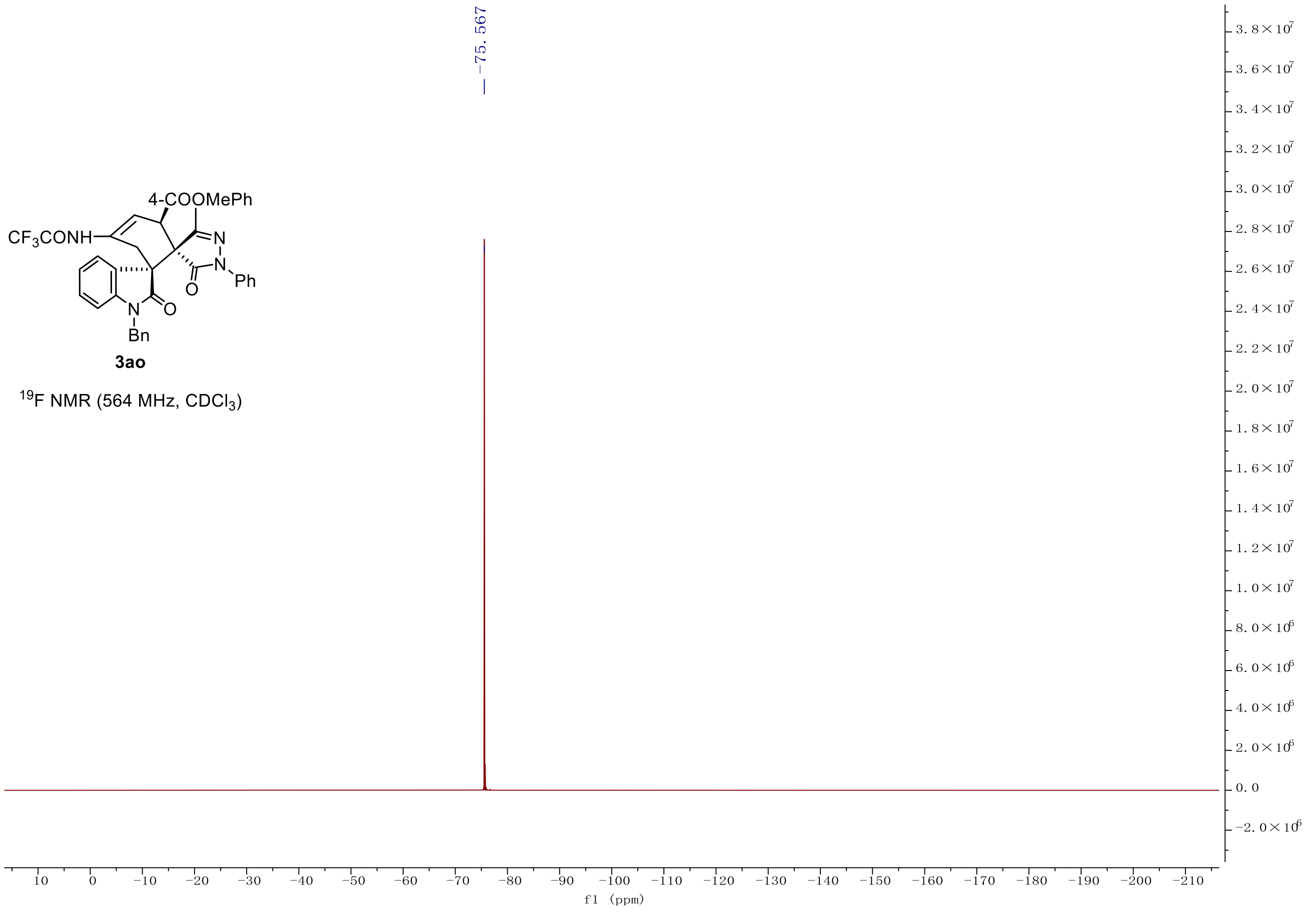
-75.567
4-COOMePh
CF_3CONH
N
N
Ph
O
N
O
Bn
3ao
^{19}F NMR (564 MHz, $CDCl_3$)
f1 (ppm)

Signal: VWD1 B, Wavelength=254 nm

RT [min]Type	Area	Width[min]	Area%
6.046	5921.0571	0.260	49.6022
7.138	6016.0190	0.313	50.3978
总和	11937.0762		

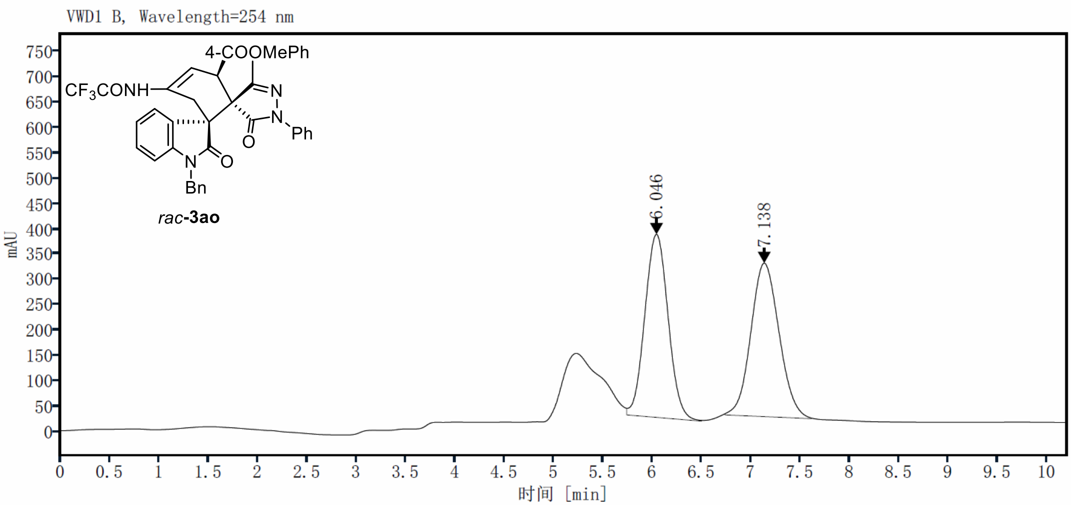

Signal: VWD1 B, Wavelength=254 nm

RT [min]Type	Area	Width[min]	Area%
5.978	10070.1914	0.300	96.1776
7.074	400.2175	0.280	3.8224
总和	10470.4089		

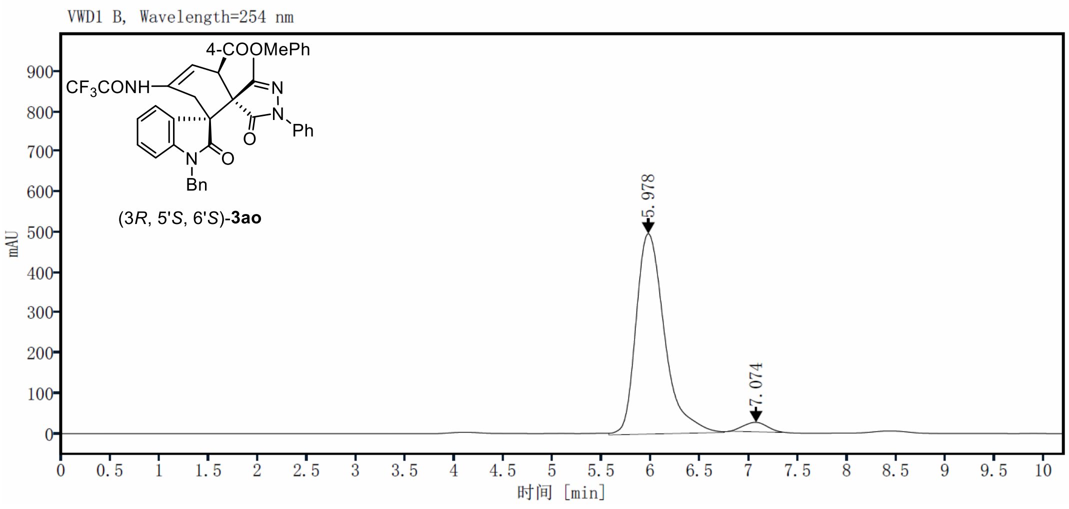

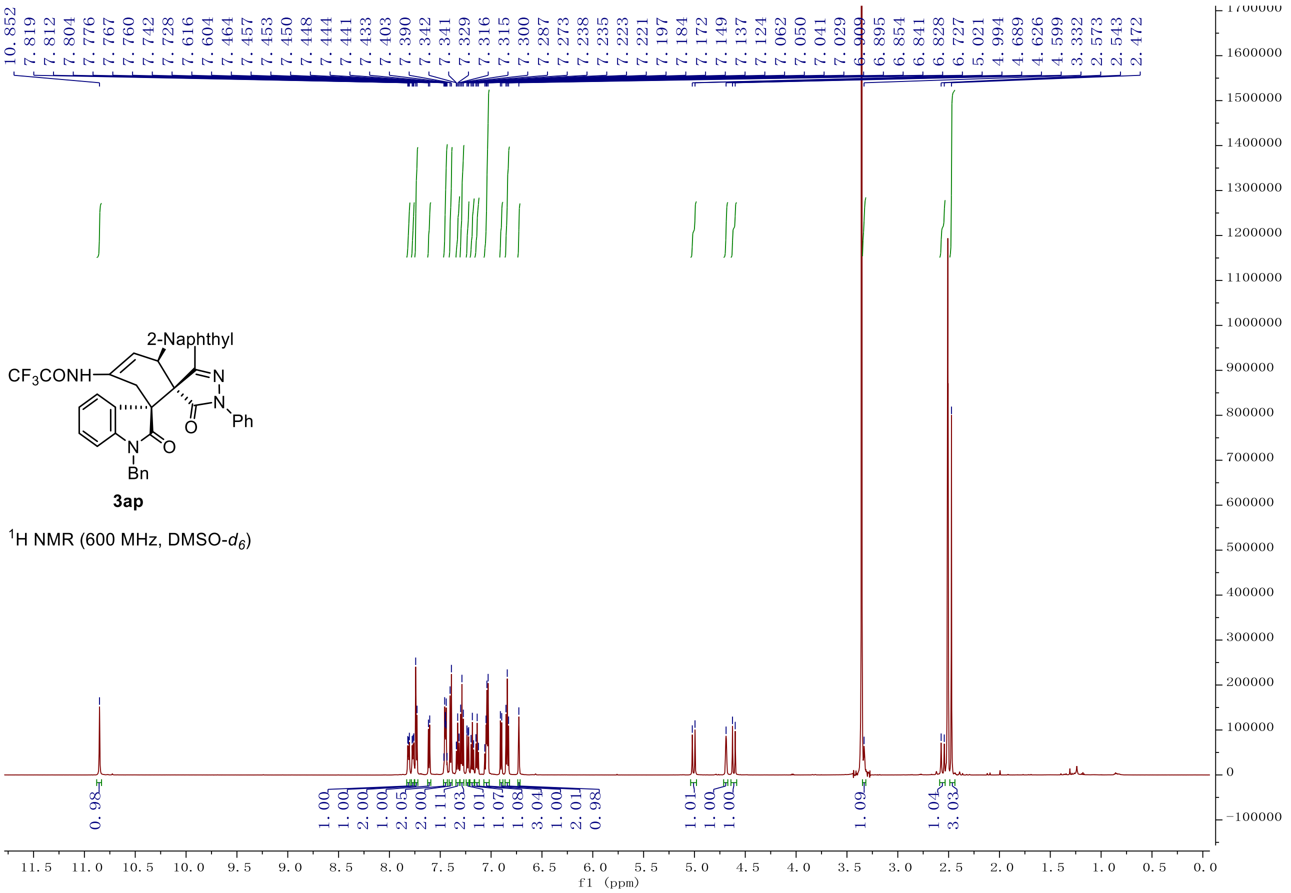
10.852
7.819
7.812
7.804
7.776
7.767
7.760
7.742
7.728
7.616
7.604
7.464
7.457
7.453
7.450
7.448
7.444
7.441
7.433
7.403
7.390
7.342
7.341
7.329
7.316
7.315
7.300
7.287
7.273
7.238
7.235
7.223
7.221
7.197
7.184
7.172
7.149
7.137
7.124
7.062
7.050
7.041
7.029
6.909
6.895
6.854
6.841
6.828
6.727
5.021
4.994
4.689
4.626
4.599
3.332
2.573
2.543
2.472
2-Naphthyl
CF_3CONH
N
N
Ph
O
N
O
Bn
3ap
^{1}H NMR (600 MHz, DMSO-d_6)
0.98
1.00
1.00
2.00
1.00
2.05
2.00
1.11
2.03
1.01
1.07
1.08
3.04
1.00
2.01
0.98
1.01
1.00
1.00
1.09
1.04
3.03
f1 (ppm)

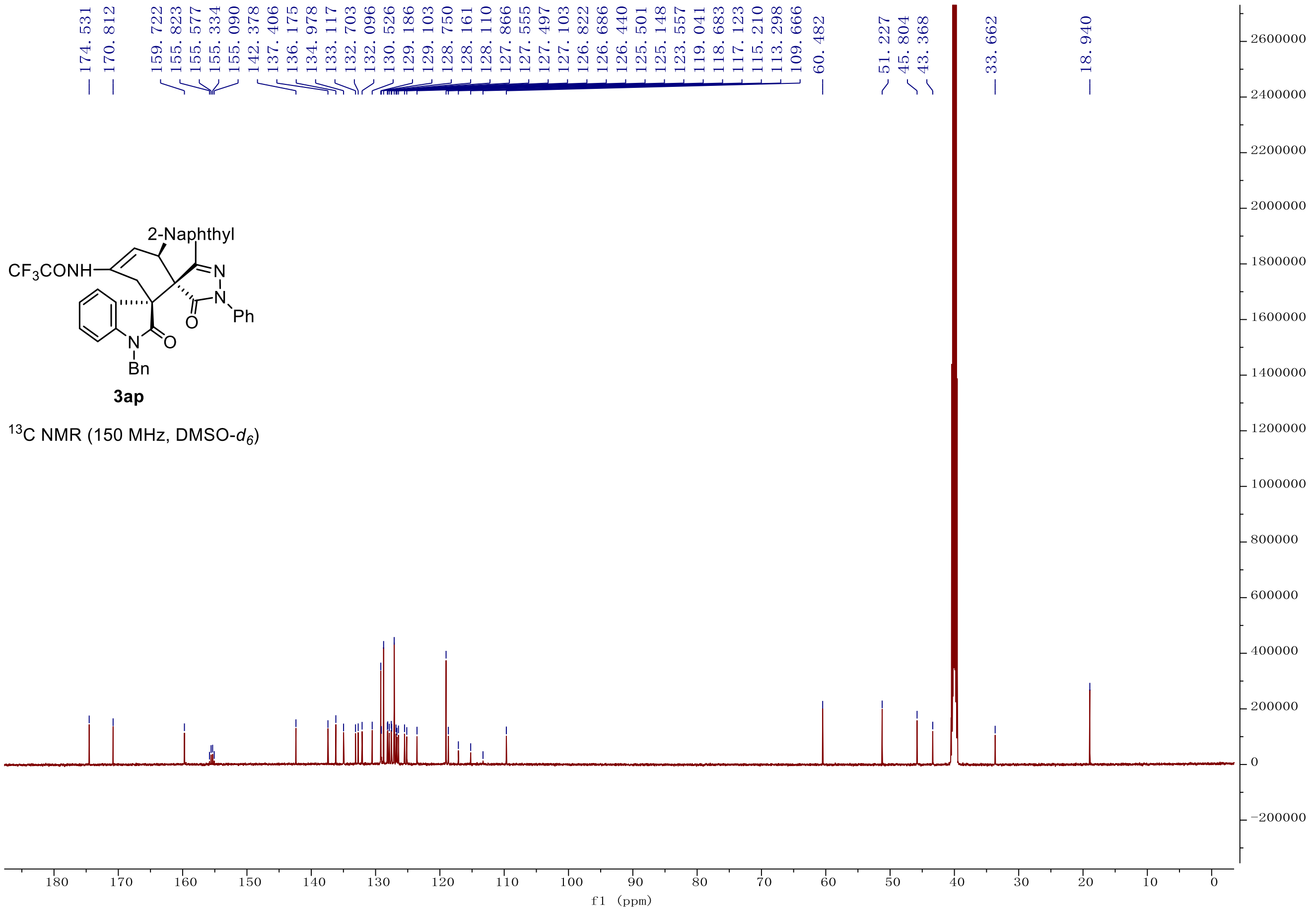
174.531
170.812
159.722
155.823
155.577
155.334
155.090
142.378
137.406
136.175
134.978
133.117
132.703
132.096
130.526
129.186
129.103
128.750
128.161
128.110
127.866
127.555
127.497
127.103
126.822
126.686
126.440
125.501
125.148
123.557
119.041
118.683
117.123
115.210
113.298
109.666
60.482
51.227
45.804
43.368
33.662
18.940
2-Naphthyl
CF_3CONH
N
N
Ph
O
N
O
Bn
3ap
^{13}C NMR (150 MHz, DMSO-d_6)
f1 (ppm)

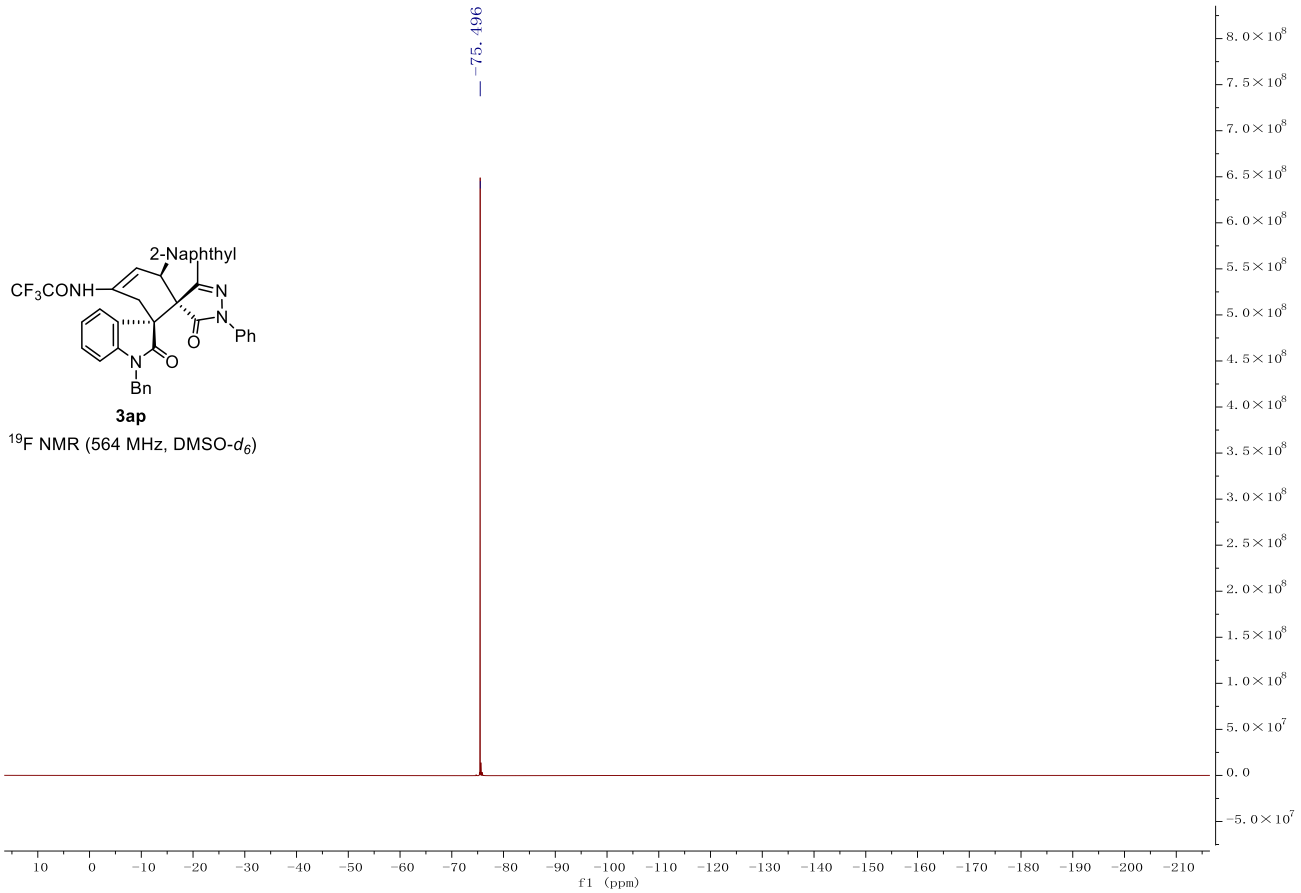

Peak Analysis Report

Detector A Channel 1 254nm

No.	Ret. Time	Height (mAu)	Area (mAu*min)	Rel. Area (%)
1	8.447	81212	2672533	50.805
2	17.424	29664	2587873	49.195
Total		110877	5260406	100.000

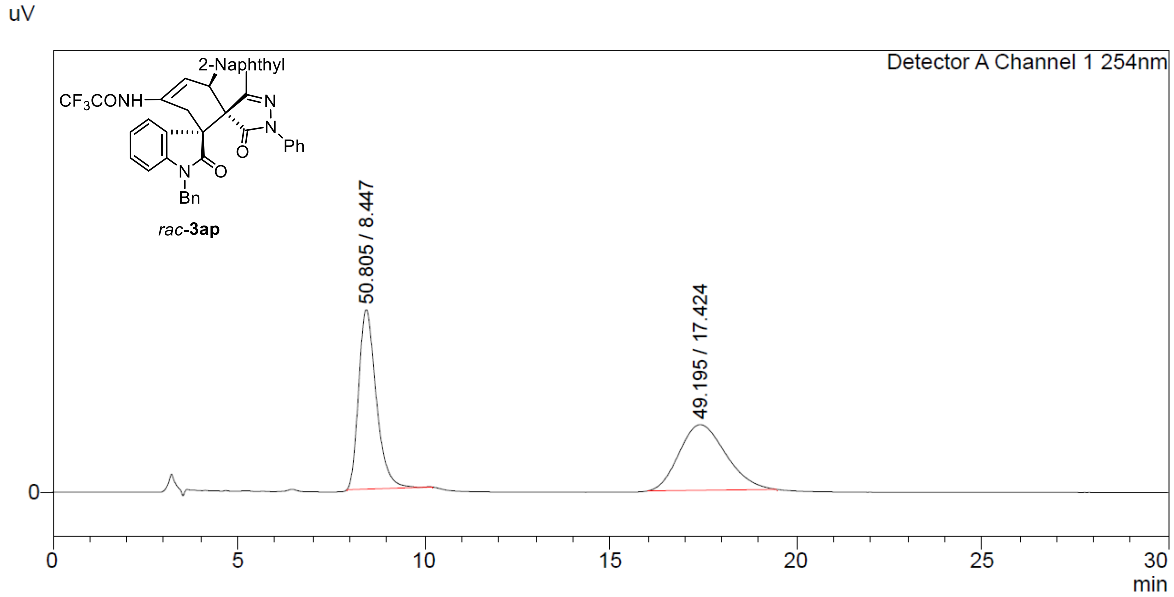

Peak Analysis Report

Detector A Channel 1 254nm

No.	Ret. Time	Height (mAu)	Area (mAu*min)	Rel. Area (%)
1	8.351	67	2259	0.071
2	17.420	34315	3162138	99.929
Total		34381	3164397	100.000

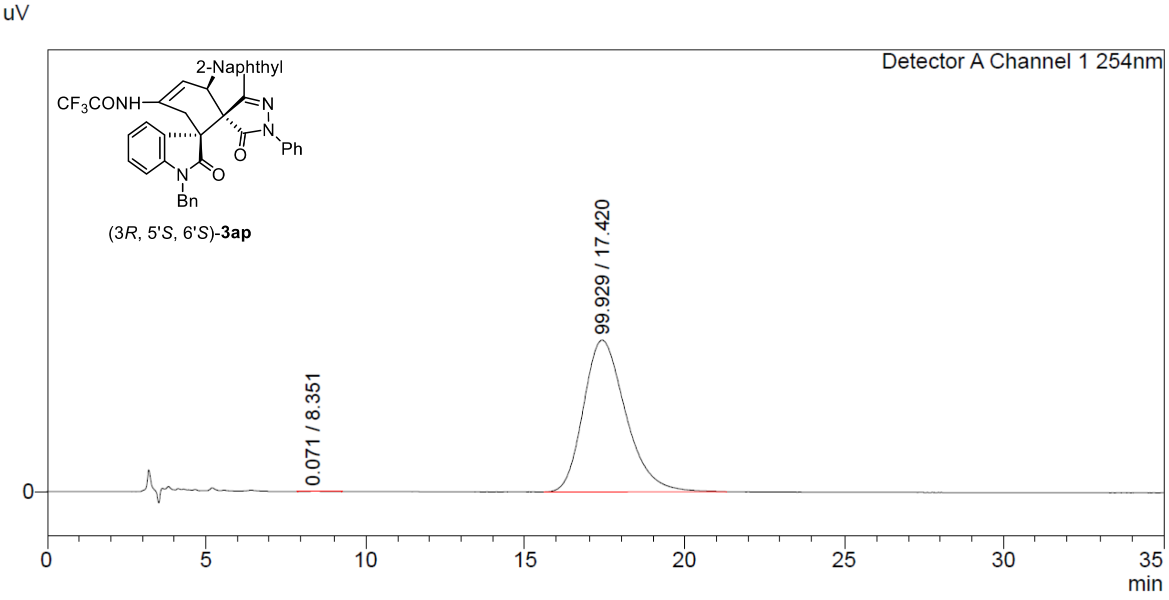

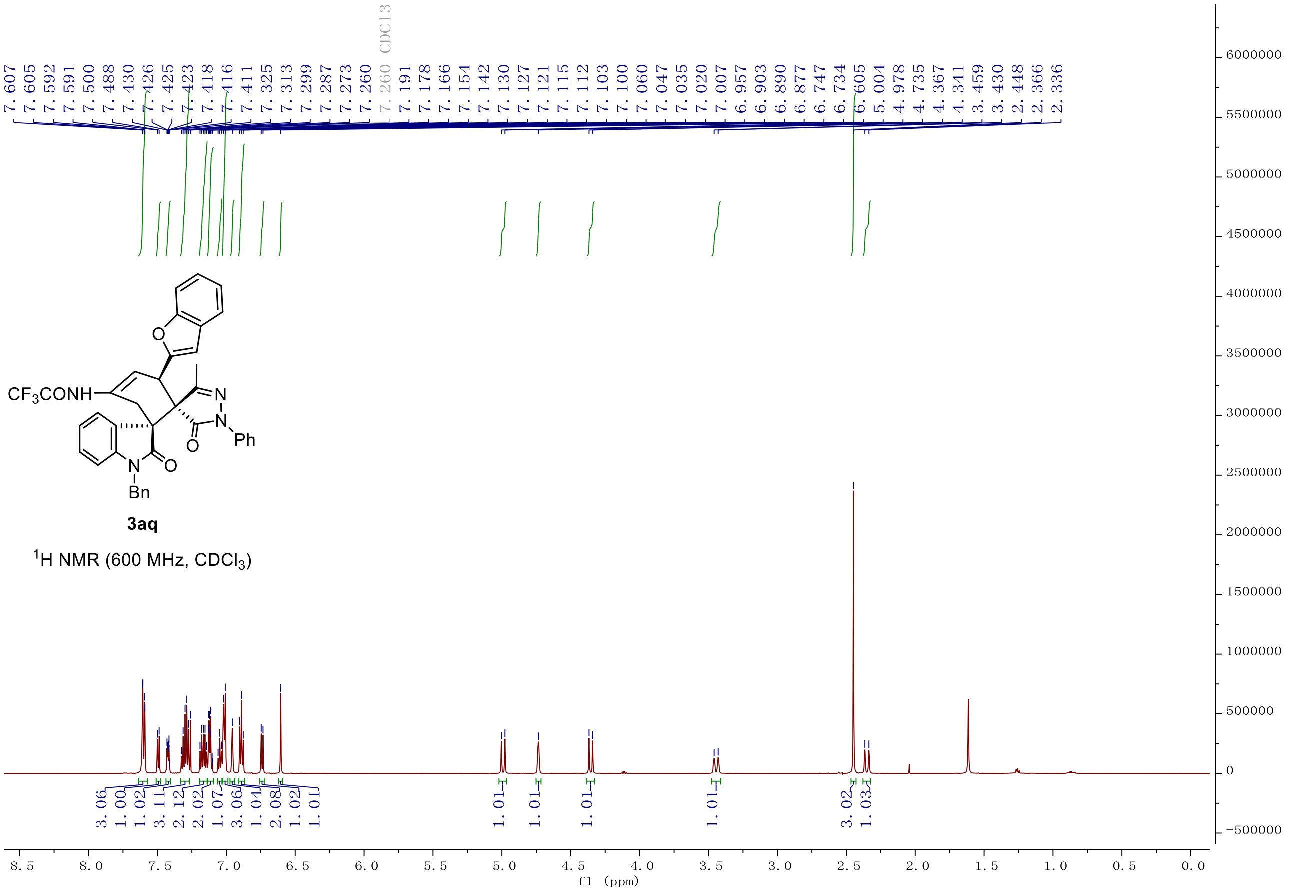

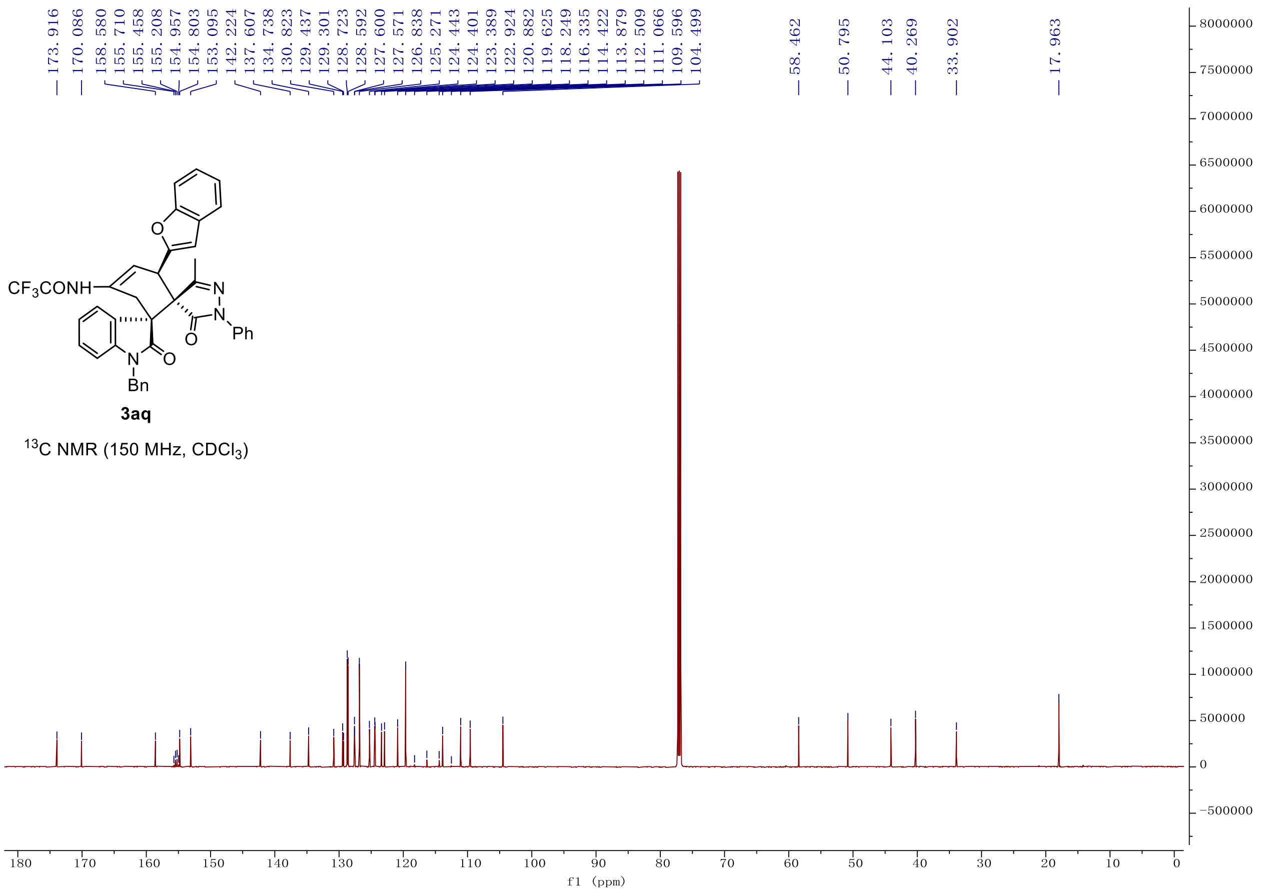
173.916
170.086
158.580
155.710
155.458
155.208
154.957
154.803
153.095
142.224
137.607
134.738
130.823
129.437
129.301
128.723
128.592
127.600
127.571
126.838
125.271
124.443
124.401
123.389
122.924
120.882
119.625
118.249
116.335
114.422
113.879
112.509
111.066
109.596
104.499
58.462
50.795
44.103
40.269
33.902
17.963
CF3CONH
O
N
N
Ph
O
N
O
Bn
3aq
13C NMR (150 MHz, CDCl3)
f1 (ppm)

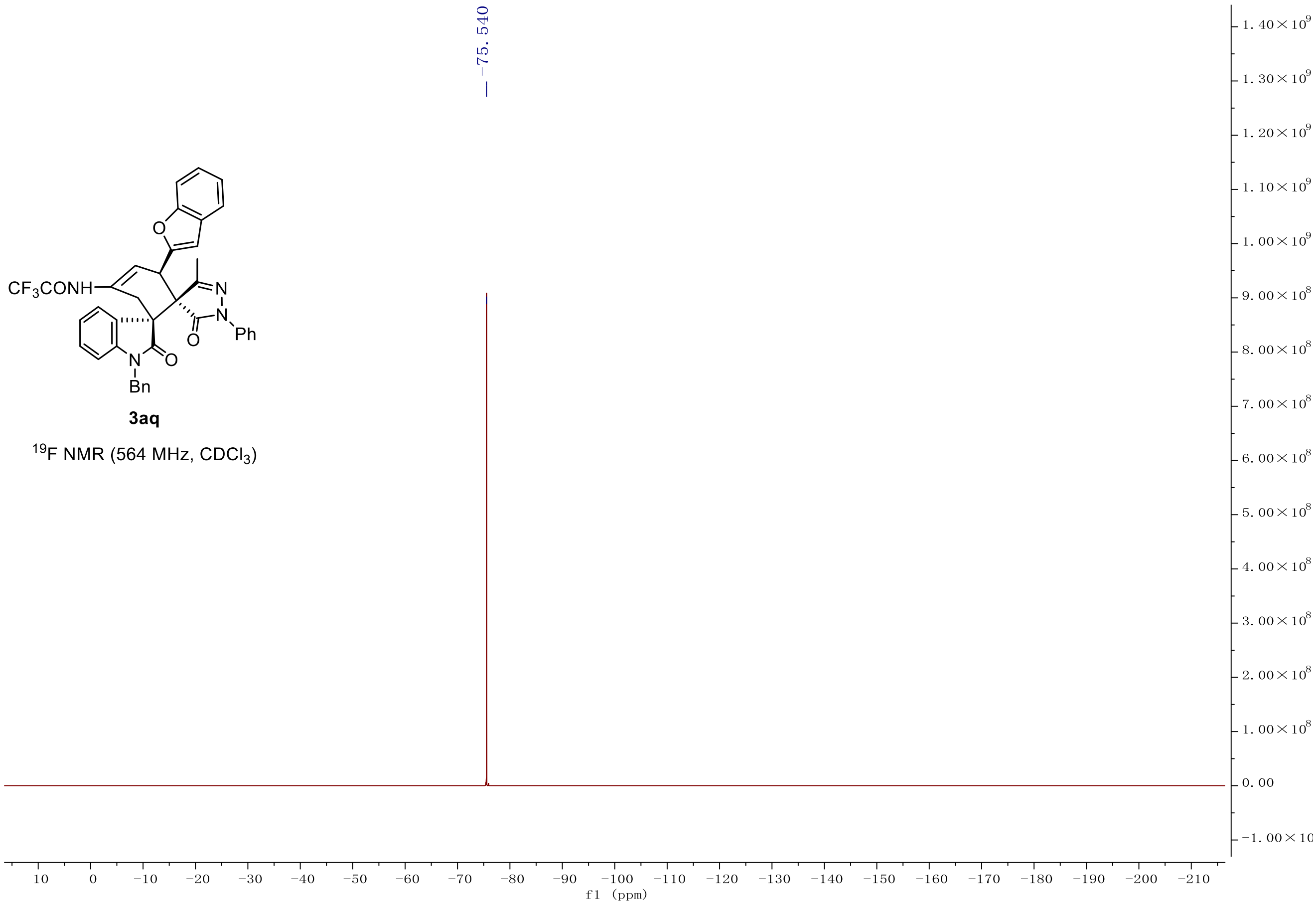
-75.540
CF3CONH
O
N
N
Ph
O
N
O
Bn
3aq
19F NMR (564 MHz, CDCl3)
f1 (ppm)

Peak Analysis Report

Detector A Channel 1 254nm

No.	Ret. Time	Height (mAu)	Area (mAu*min)	Rel. Area (%)
1	11.884	296673	14675309	49.378
2	15.201	226075	15045261	50.622
Total		522748	29720570	100.000

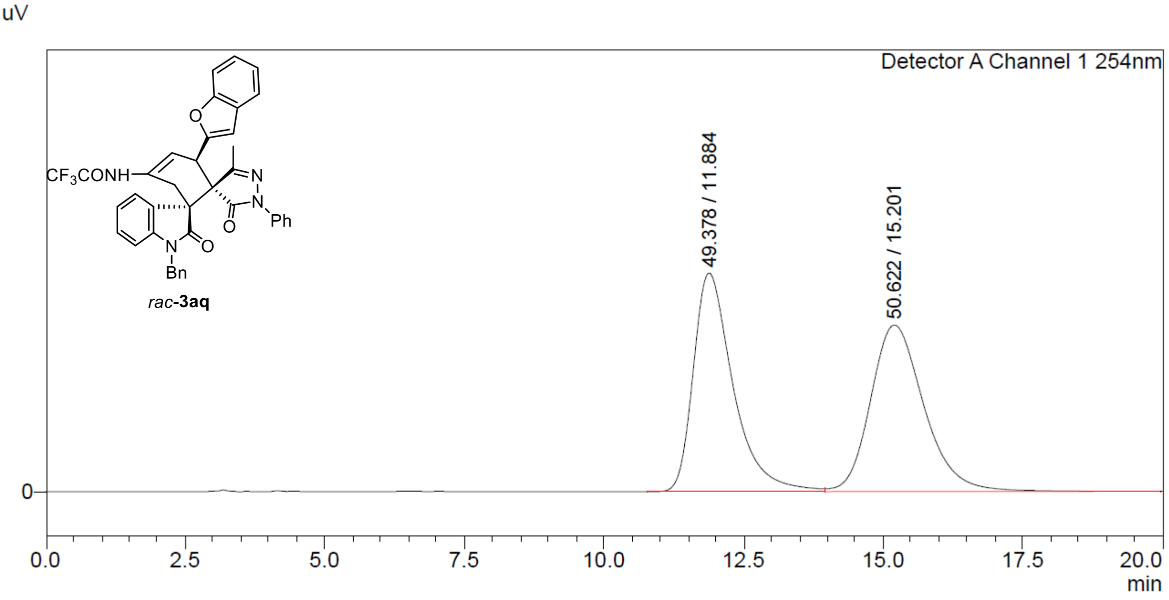

Peak Analysis Report

Detector A Channel 1 254nm

No.	Ret. Time	Height (mAu)	Area (mAu*min)	Rel. Area (%)
1	11.967	728	37678	1.507
2	15.191	37974	2462813	98.493
Total		38702	2500490	100.000

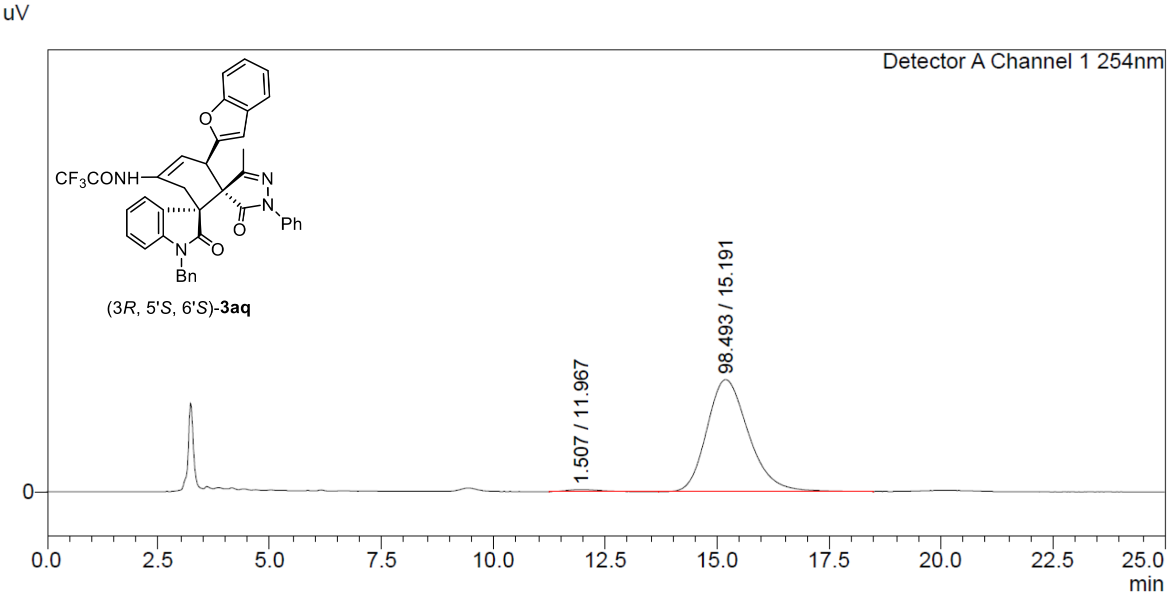

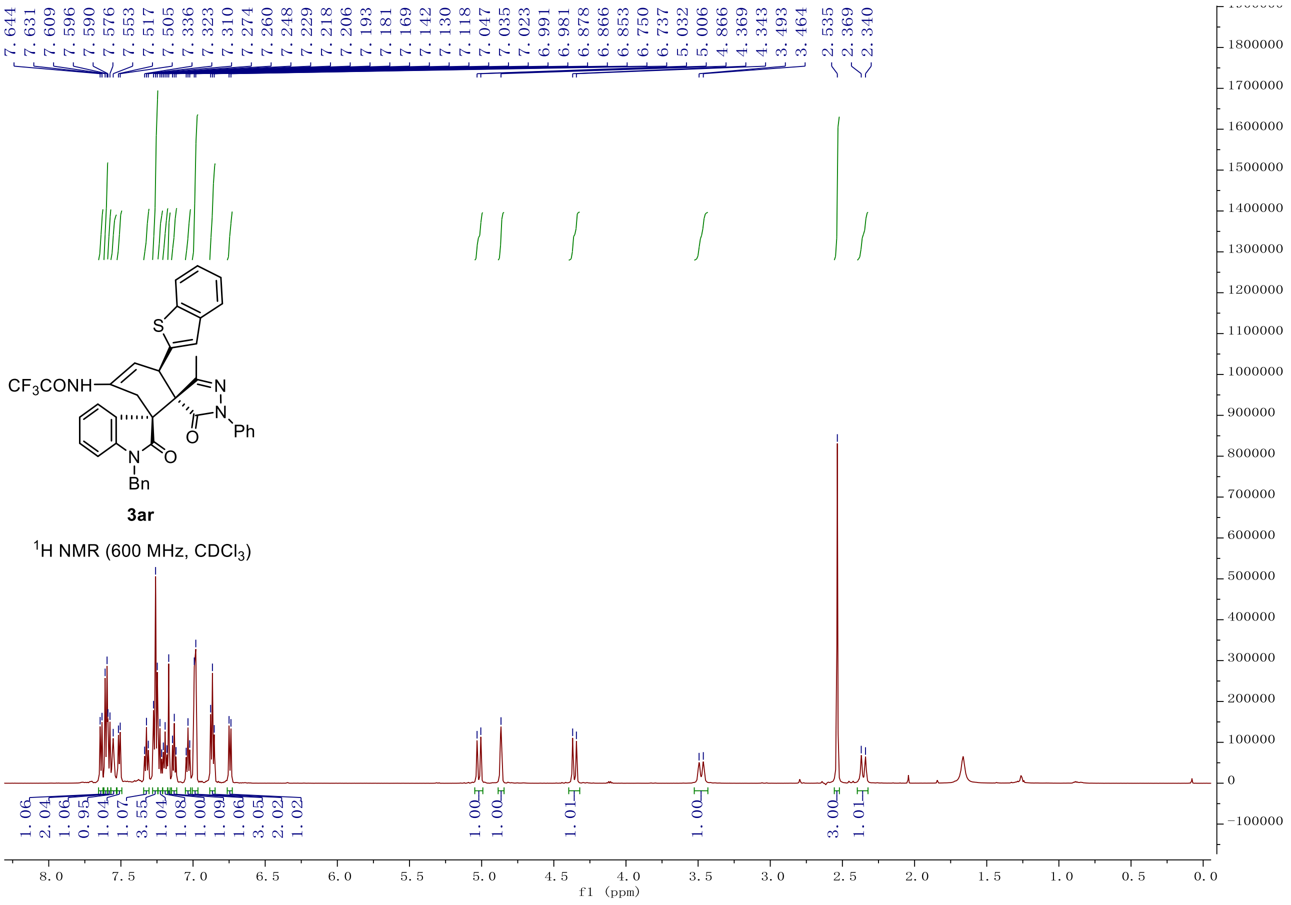

3ar

^{1}H NMR (600 MHz, $CDCl_3$)

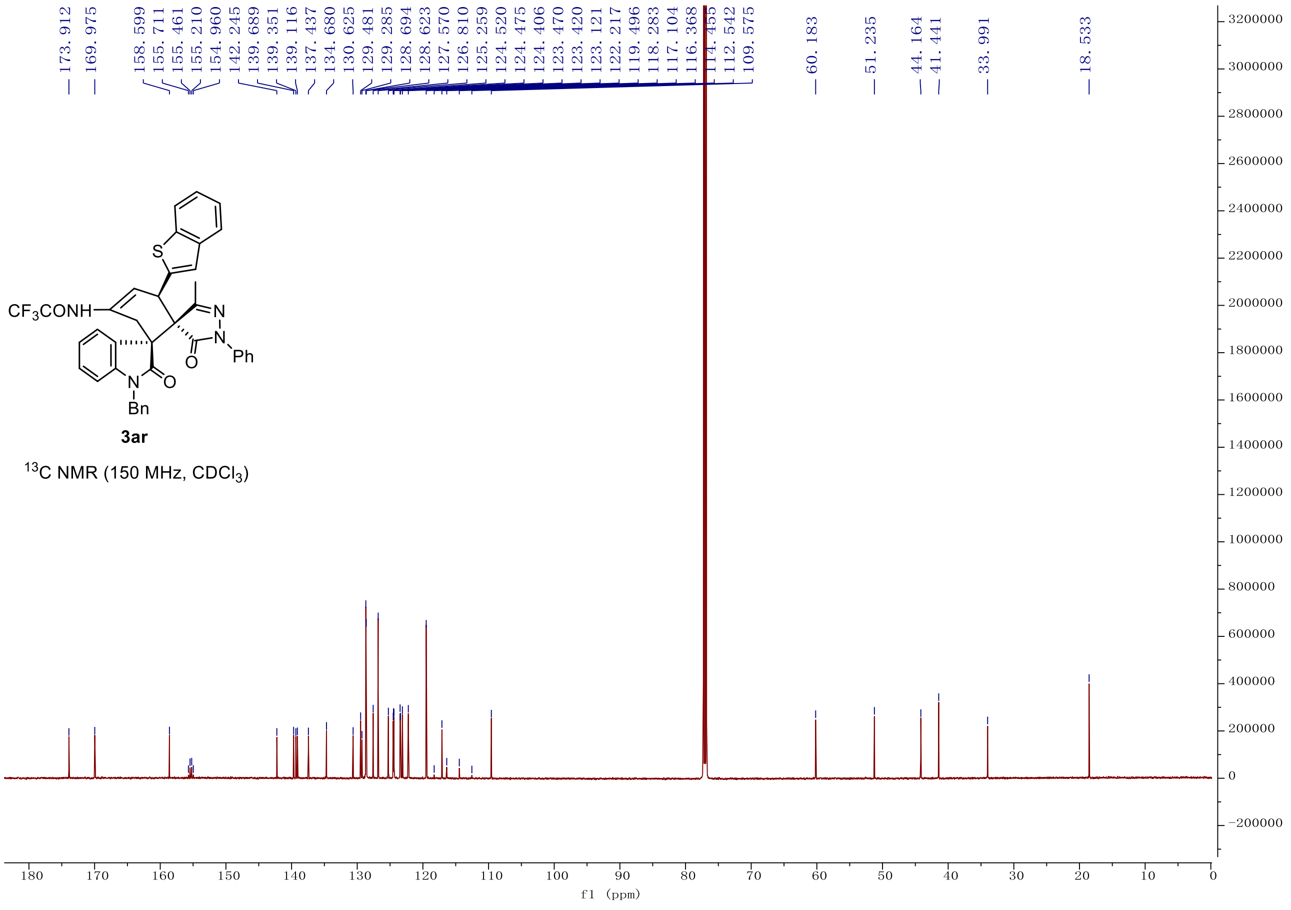

3ar

^{13}C NMR (150 MHz, $CDCl_3$)

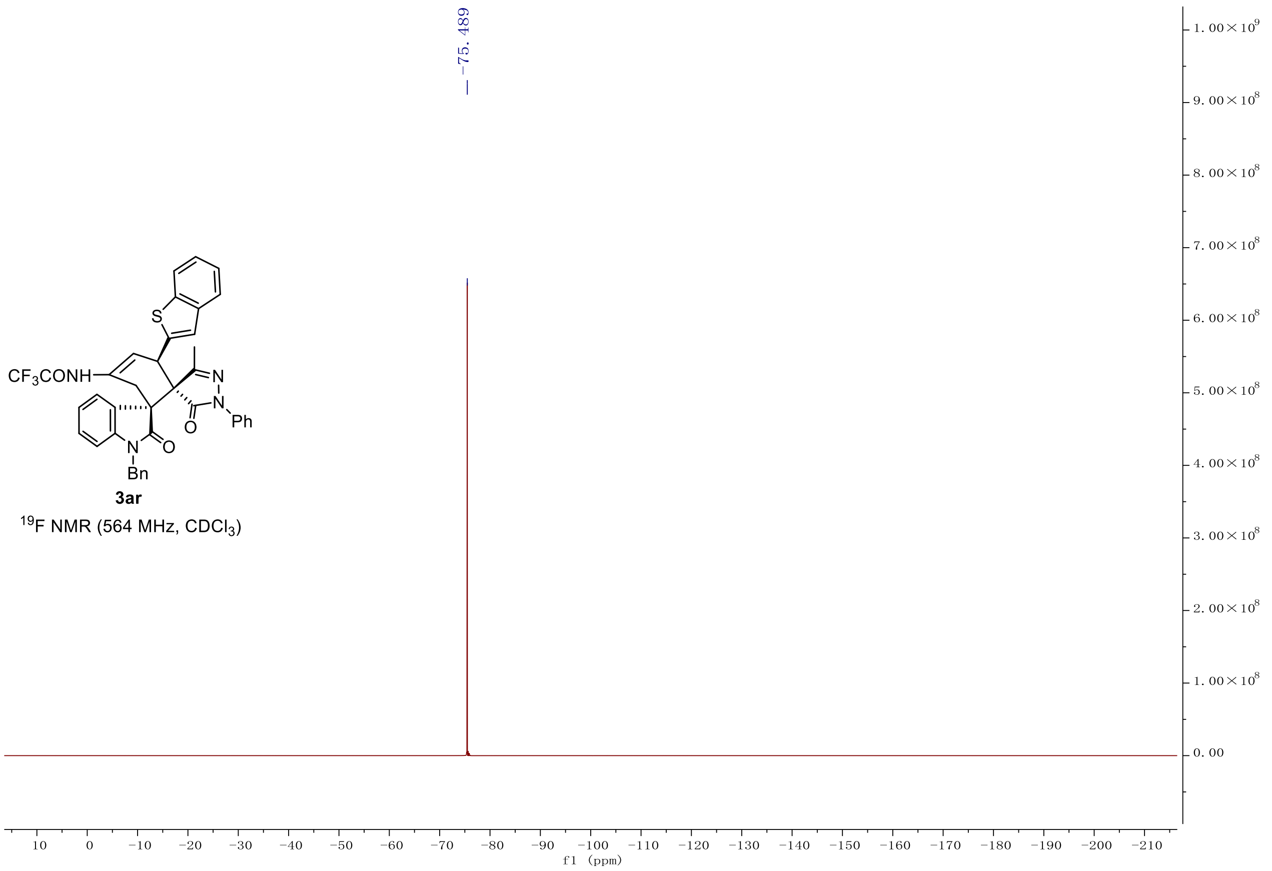

Peak Analysis Report

Detector A Channel 1 254nm

No.	Ret. Time	Height (mAu)	Area (mAu*min)	Rel. Area (%)
1	8.346	336228	10759212	50.800
2	23.598	102555	10420511	49.200
Total		438783	21179723	100.000

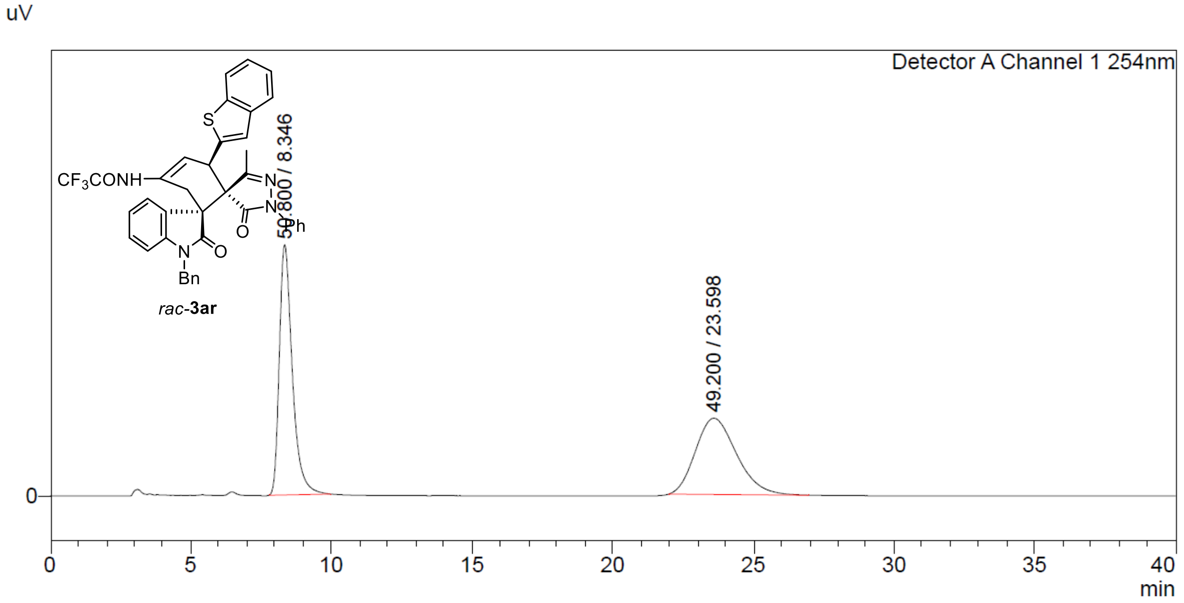

Peak Analysis Report

Detector A Channel 1 254nm

No.	Ret. Time	Height (mAu)	Area (mAu*min)	Rel. Area (%)
1	8.402	14705	569293	2.779
2	23.505	193087	19918723	97.221
Total		207792	20488016	100.000

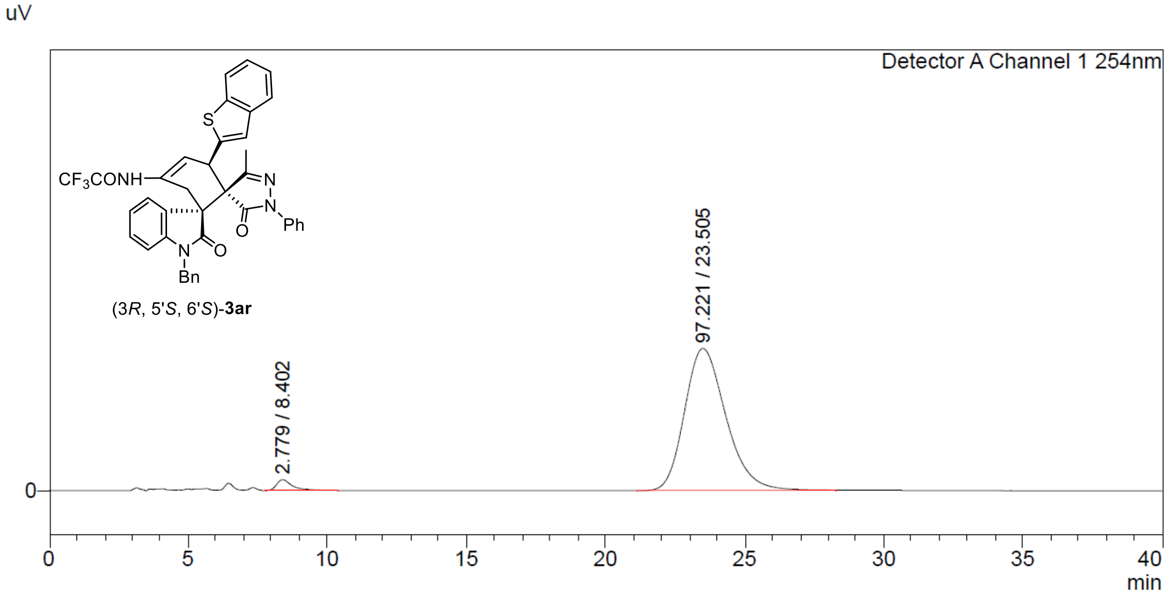

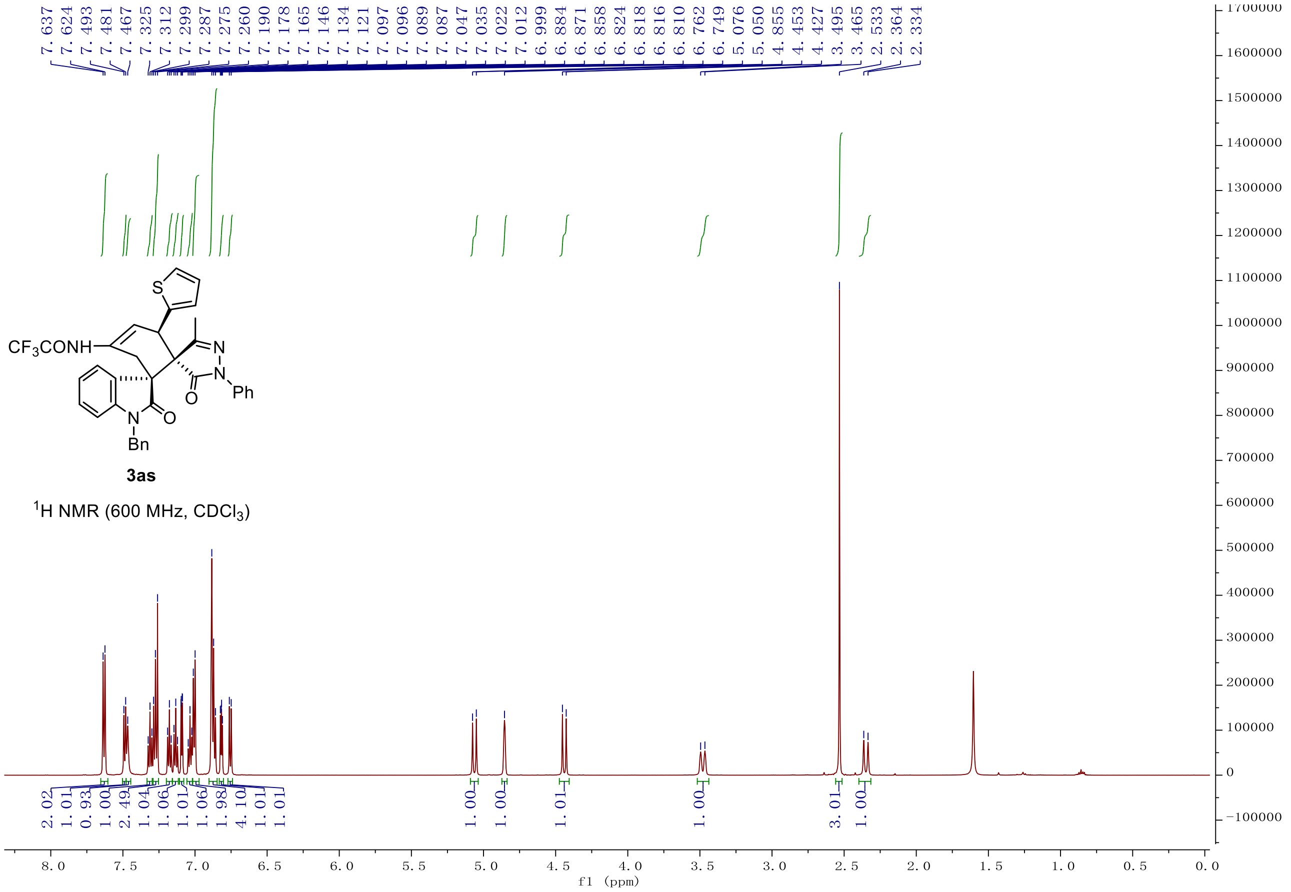

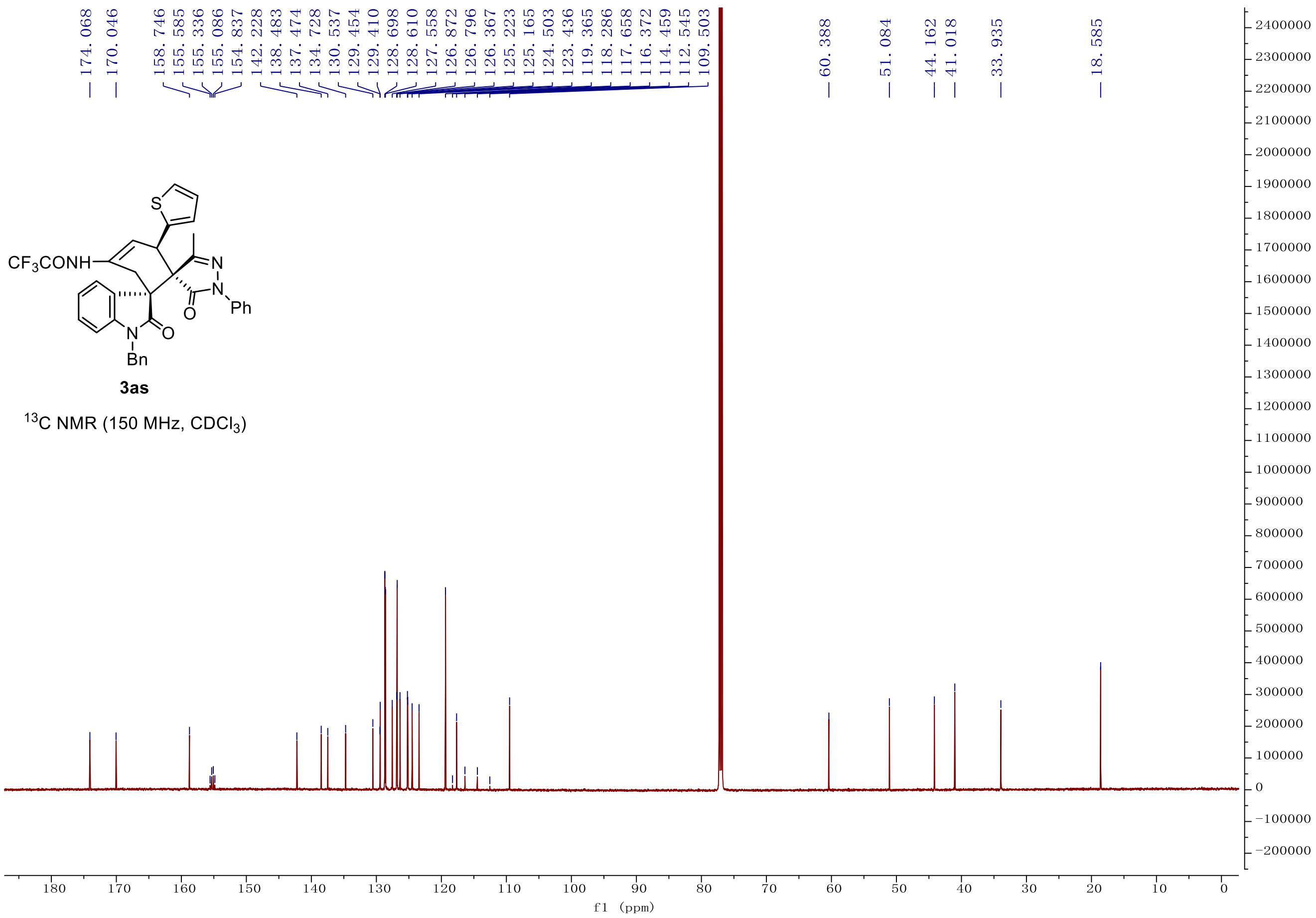

3as

^{13}C NMR (150 MHz, $CDCl_3$)

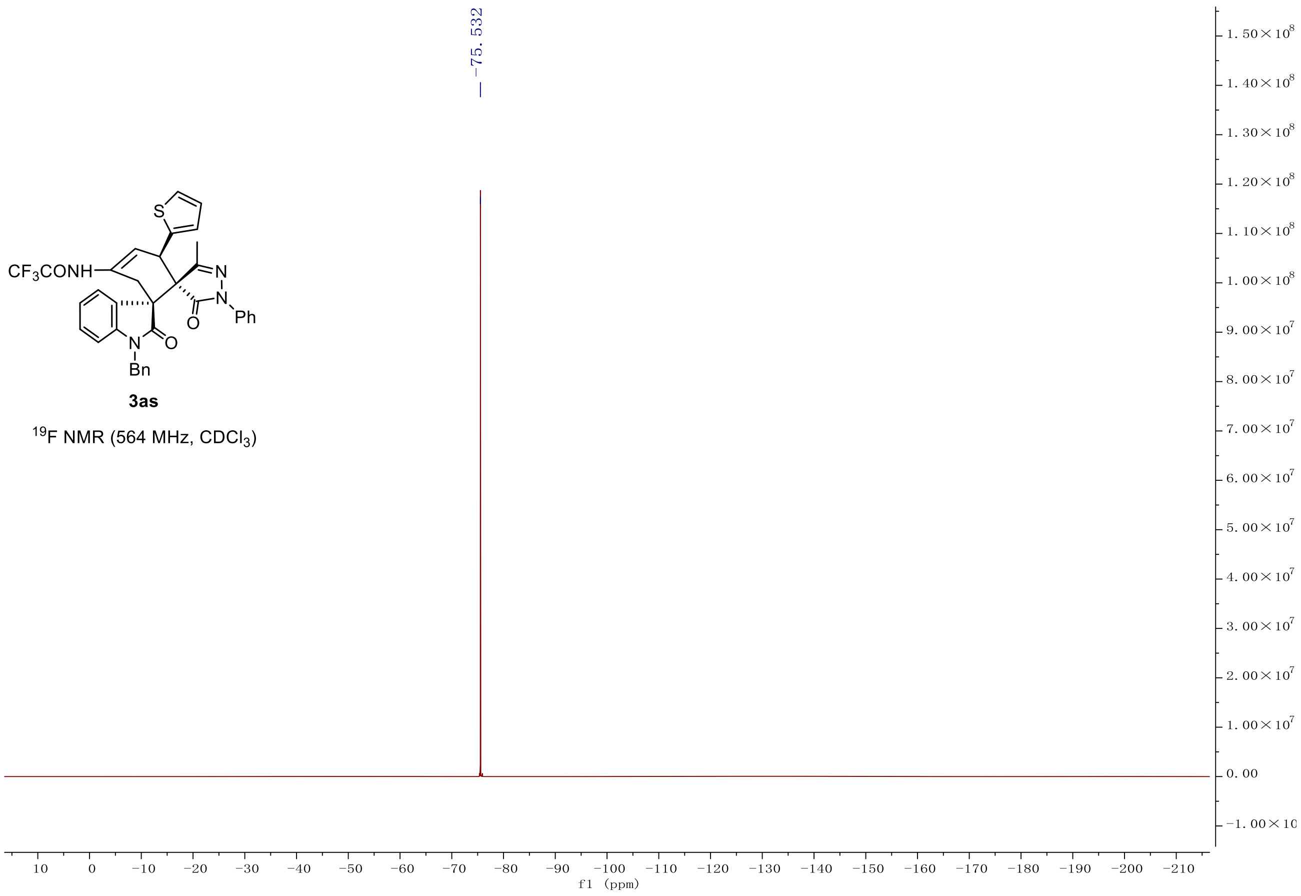

3as

^{19}F NMR (564 MHz, $CDCl_3$)

Peak Analysis Report

Detector A Channel 1 254nm

No.	Ret. Time	Height (mAu)	Area (mAu*min)	Rel. Area (%)
1	9.169	303936	10792147	49.771
2	18.990	146937	10891637	50.229
Total		450873	21683784	100.000

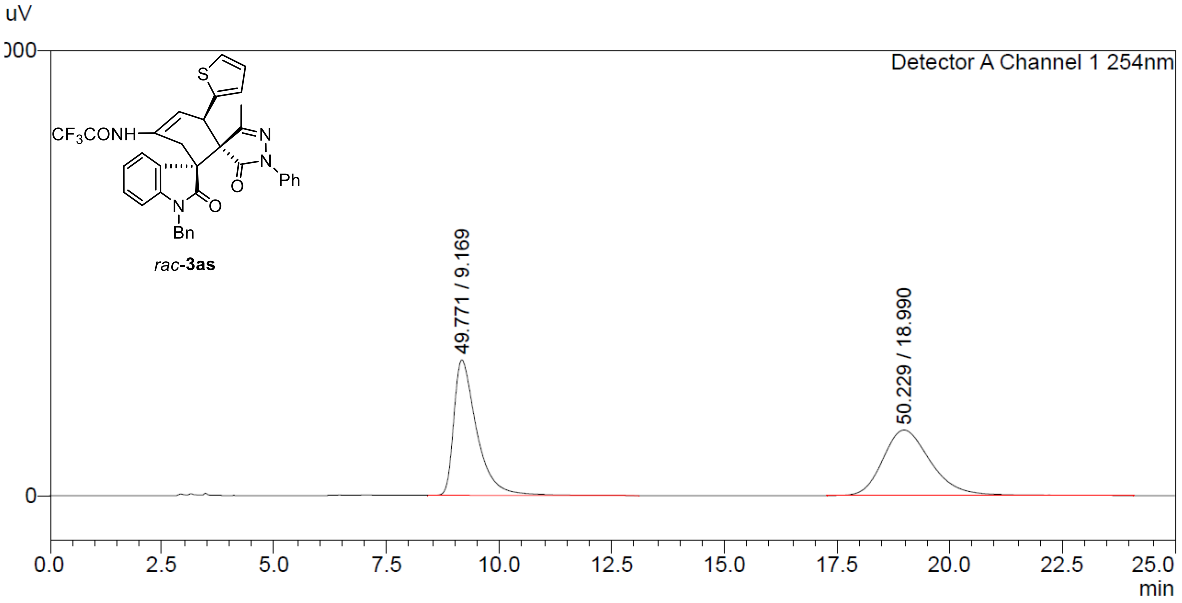

Peak Analysis Report

Detector A Channel 1 254nm

No.	Ret. Time	Height (mAu)	Area (mAu*min)	Rel. Area (%)
1	9.376	10825	442415	2.959
2	18.786	210157	14507899	97.041
Total		220982	14950314	100.000

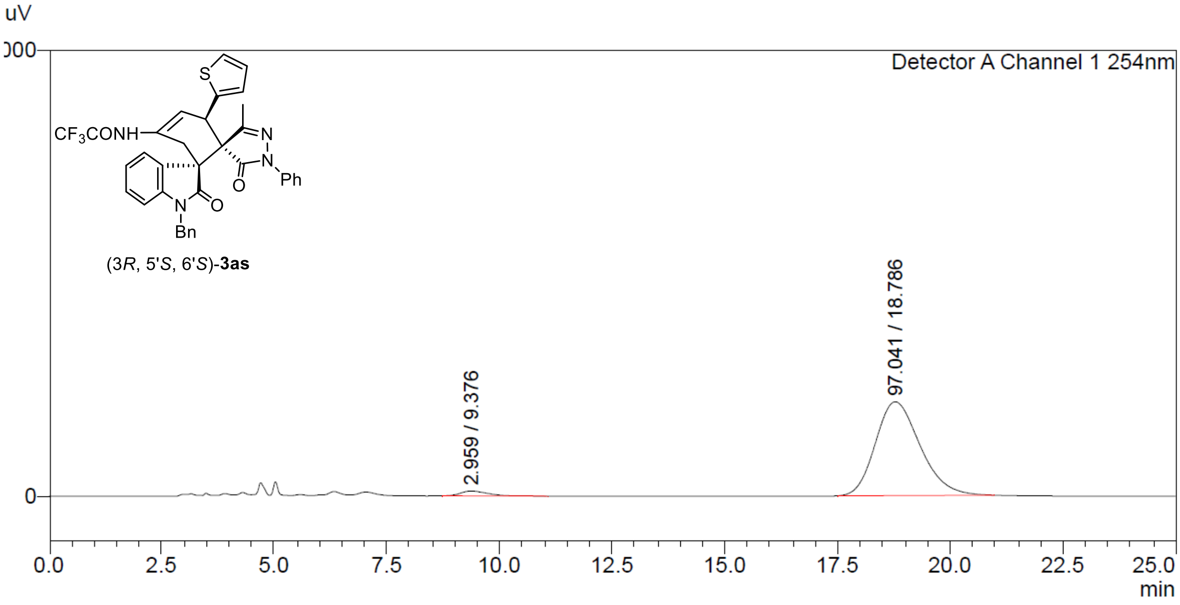

Peak Analysis Report

Detector A Channel 1 254nm

No.	Ret. Time	Height (mAu)	Area (mAu*min)	Rel. Area (%)
1	9.093	451365	15414313	95.803
2	18.939	12386	675279	4.197
Total		463750	16089592	100.000

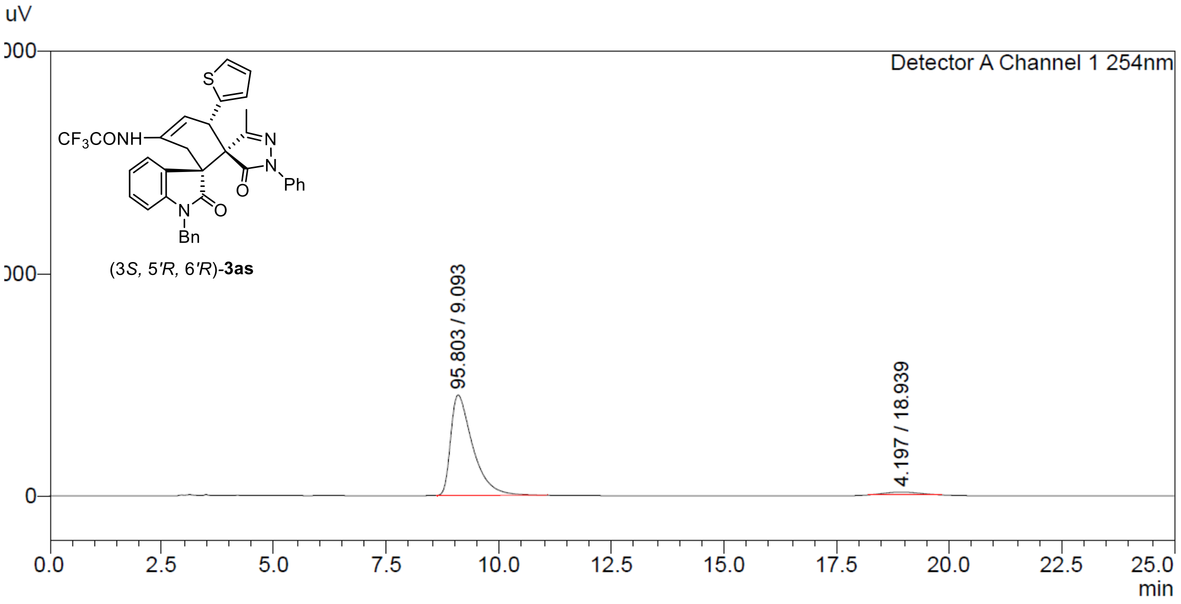

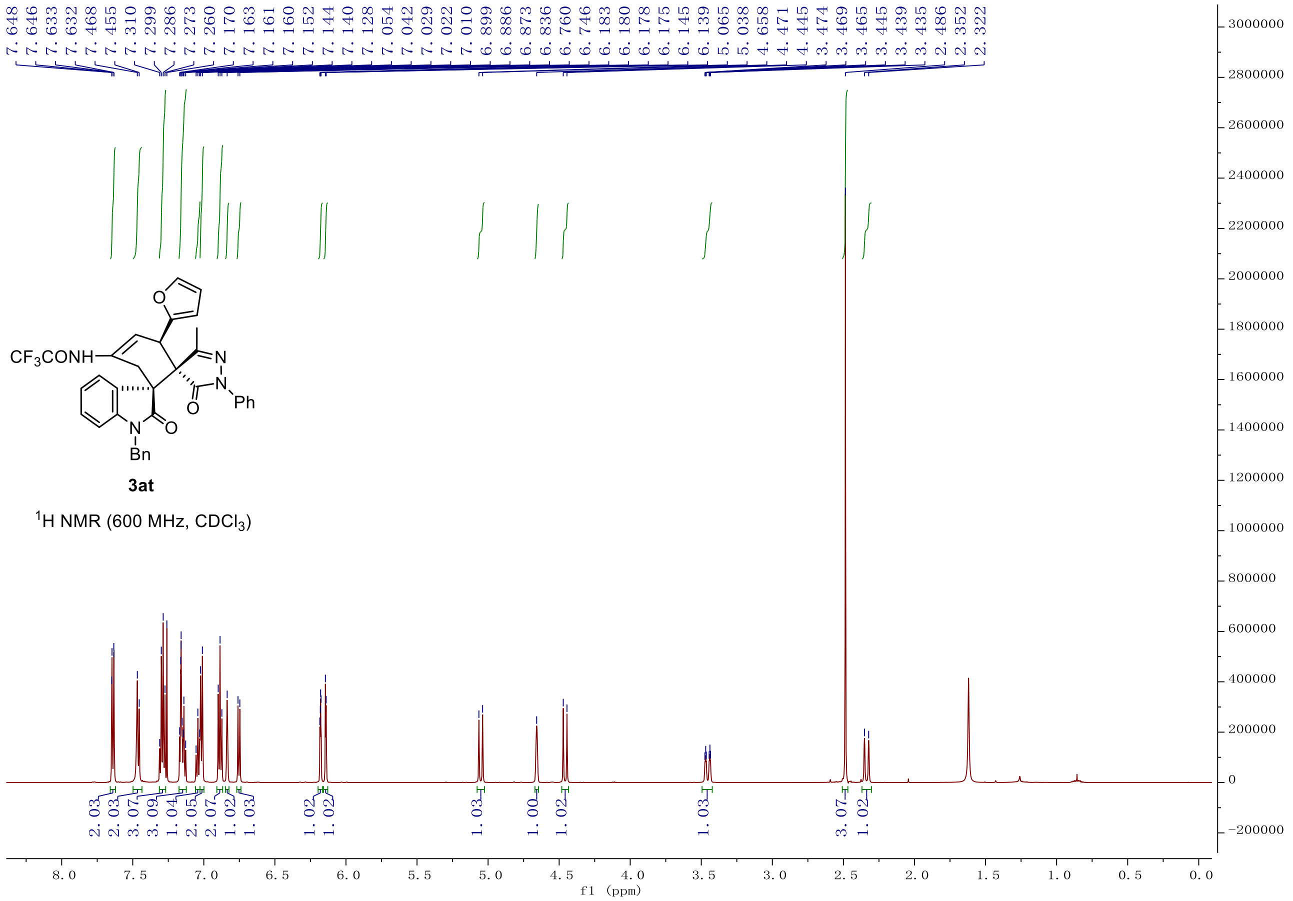

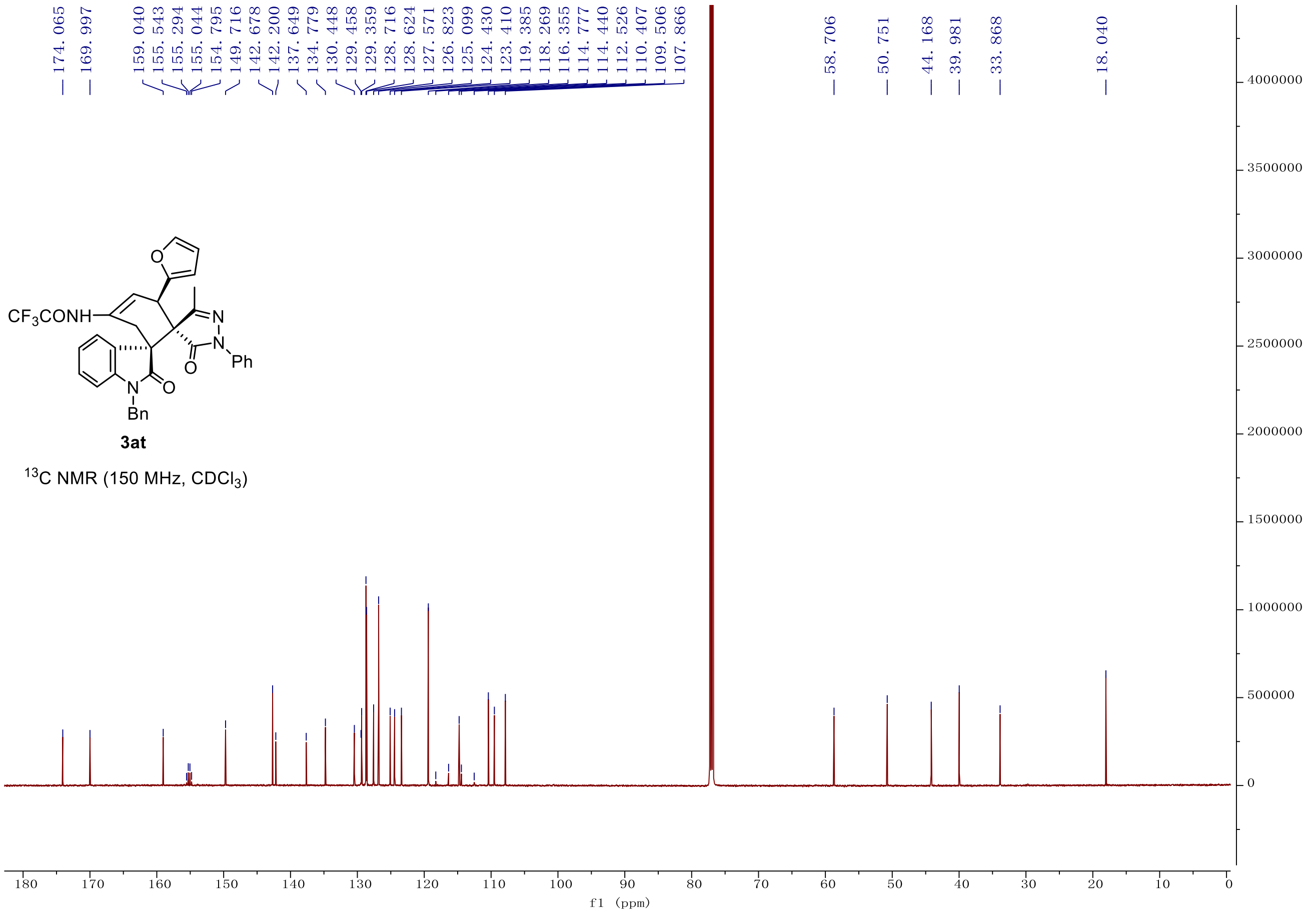

3at

^{13}C NMR (150 MHz, $CDCl_3$)

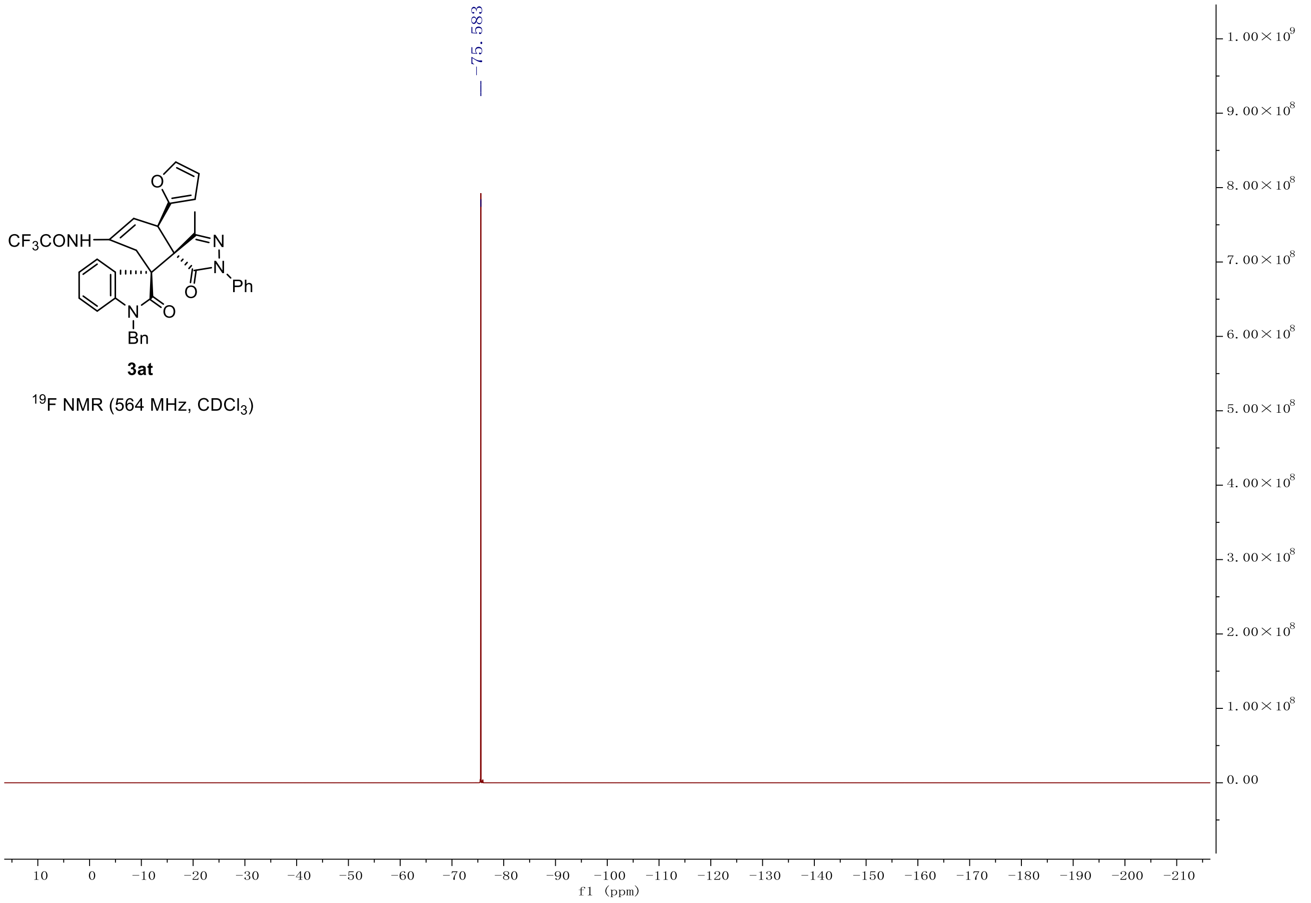

3at

^{19}F NMR (564 MHz, $CDCl_3$)

Peak Analysis Report

Detector A Channel 1 254nm

No.	Ret. Time	Height (mAu)	Area (mAu*min)	Rel. Area (%)
1	6.327	469695	6994096	50.873
2	7.288	385753	6754116	49.127
Total		855448	13748212	100.000

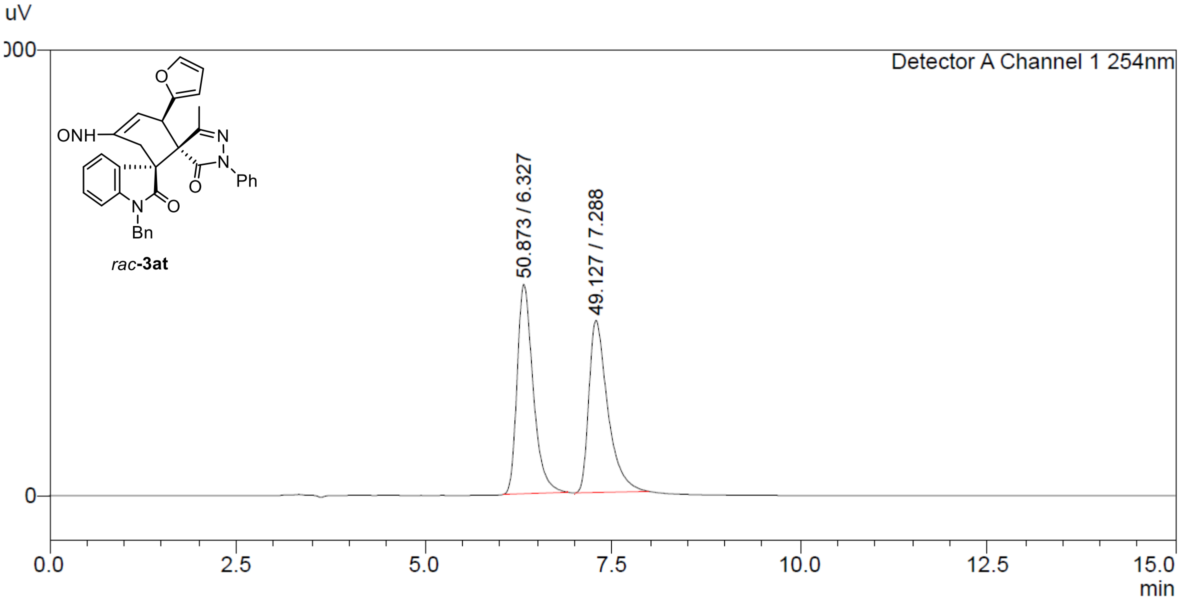

Peak Analysis Report

Detector A Channel 1 254nm

No.	Ret. Time	Height (mAu)	Area (mAu*min)	Rel. Area (%)
1	6.320	699317	10485506	93.968
2	7.332	44016	673050	6.032
Total		743333	11158556	100.000

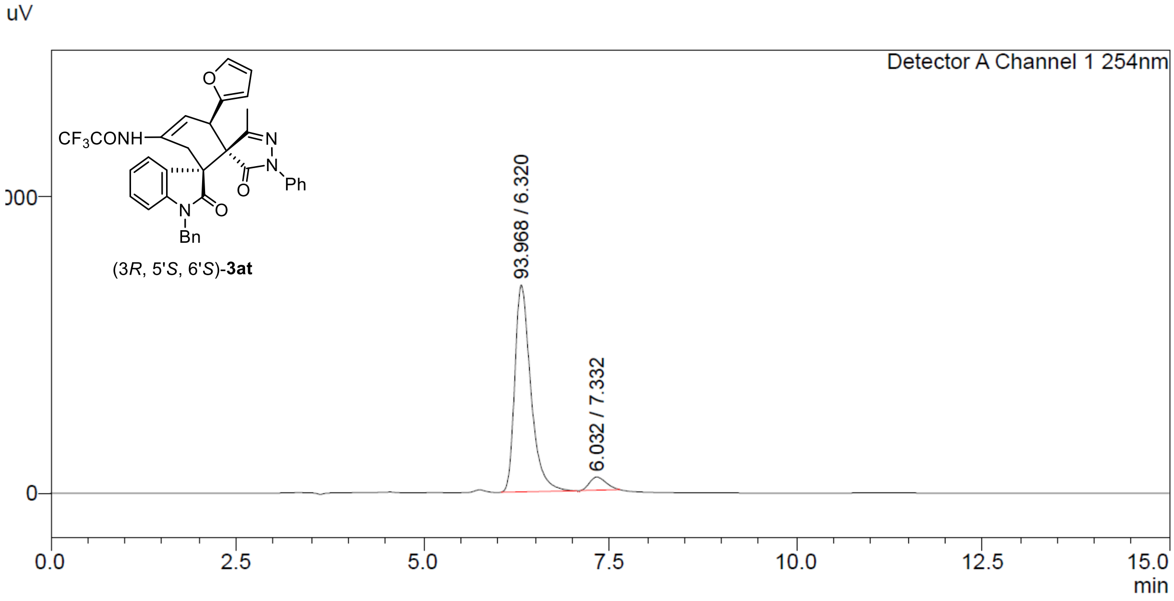

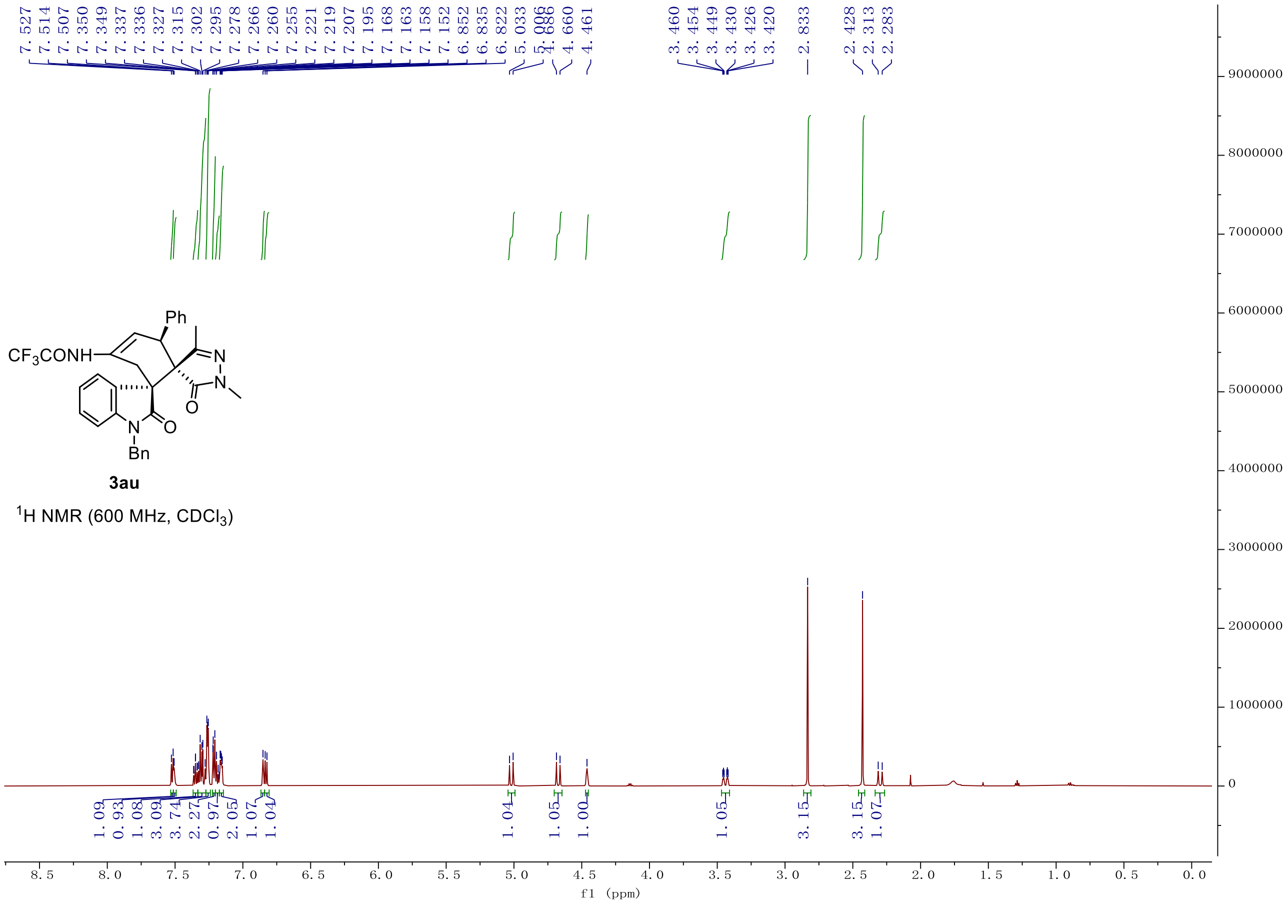

7.527 7.514 7.507 7.350 7.349 7.337 7.336 7.327 7.315 7.302 7.295 7.278 7.266 7.260 7.255 7.221 7.219 7.207 7.195 7.168 7.163 7.158 7.152 6.852 6.835 6.822 5.033 5.006 4.686 4.660 4.461 3.460 3.454 3.449 3.430 3.426 3.420 2.833 2.428 2.313 2.283
1.09 0.93 1.08 3.09 3.74 2.27 0.97 2.05 1.07 1.04 1.04 1.05 1.00 1.05 3.15 3.15 1.07
CF3CONH
Ph
N
N
O
N
O
Bn
3au
1H NMR (600 MHz, CDCl3)
f1 (ppm)

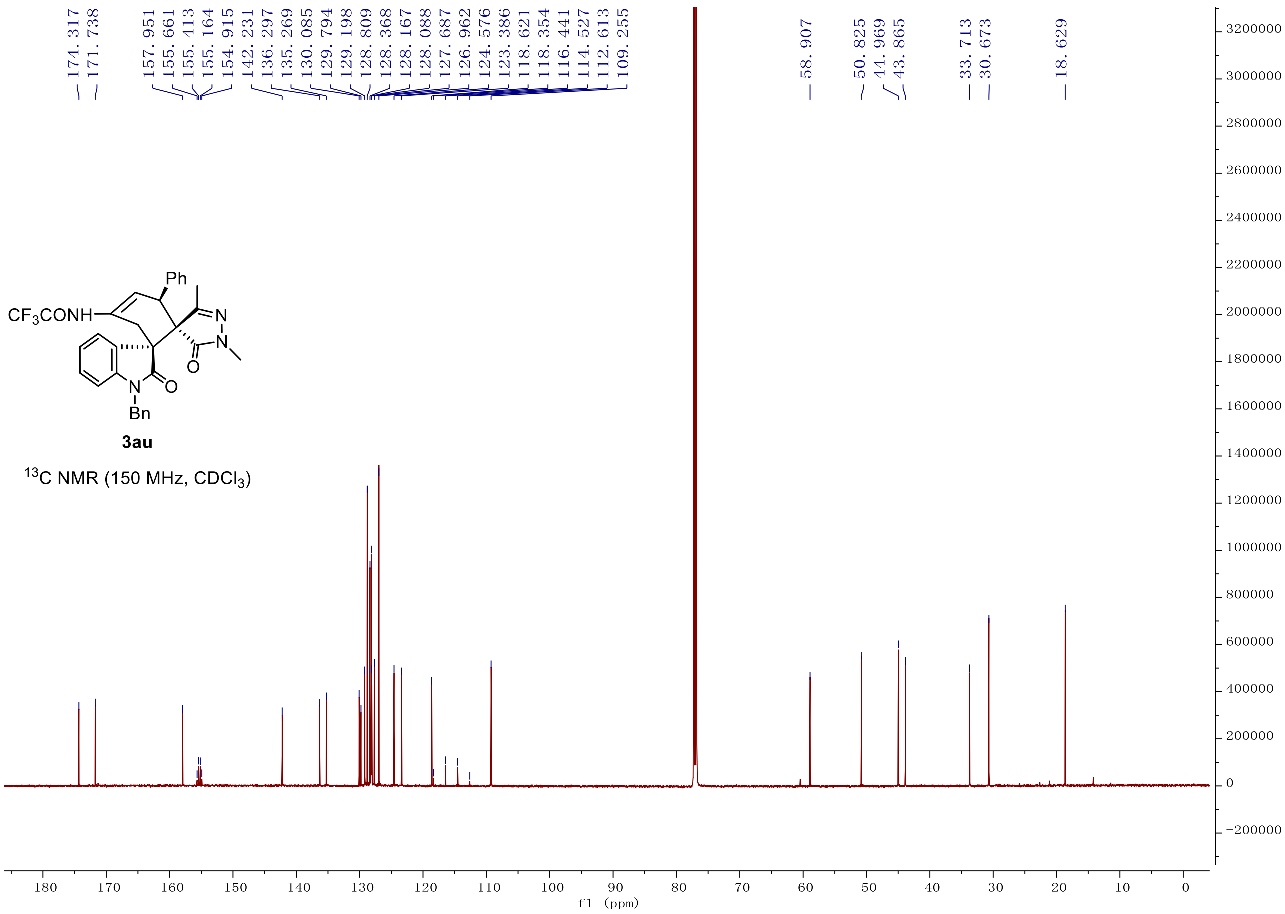

174.317 171.738 157.951 155.661 155.413 155.164 154.915 142.231 136.297 135.269 130.085 129.794 129.198 128.809 128.368 128.167 128.088 127.687 126.962 124.576 123.386 118.621 118.354 116.441 114.527 112.613 109.255 58.907 50.825 44.969 43.865 33.713 30.673 18.629
CF3CONH
Ph
N
N
O
N
O
Bn
3au
13C NMR (150 MHz, CDCl3)
f1 (ppm)

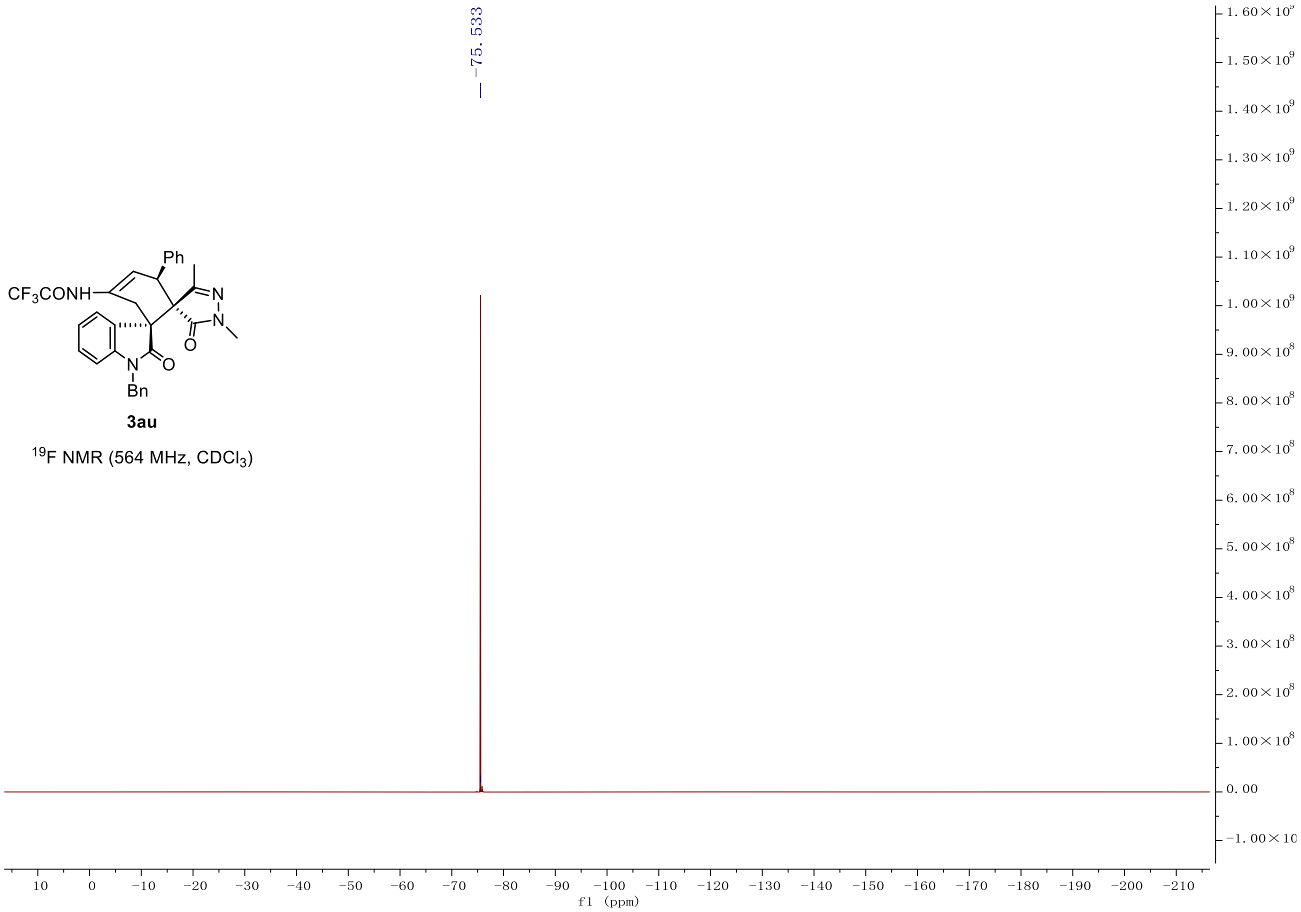

3au

^{19}F NMR (564 MHz, $CDCl_3$)

Peak Analysis Report

Detector A Channel 1 254nm

No.	Ret. Time	Height (mAu)	Area (mAu*min)	Rel. Area (%)
1	9.320	193796	5133967	50.287
2	19.587	67620	5075456	49.713
Total		261416	10209423	100.000

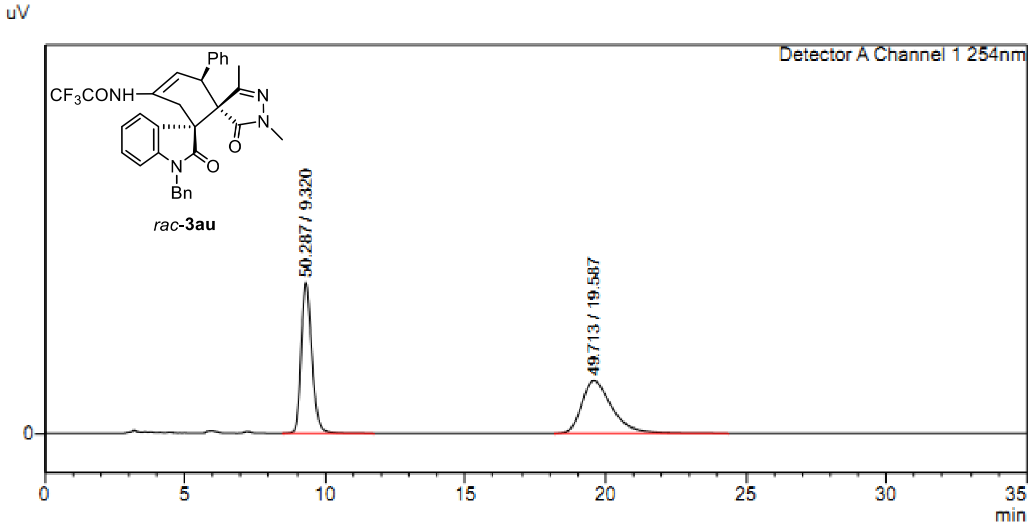

Peak Analysis Report

Detector A Channel 1 254nm

No.	Ret. Time	Height (mAu)	Area (mAu*min)	Rel. Area (%)
1	9.421	3659	91510	1.037
2	19.580	117826	8731427	98.963
Total		121485	8822938	100.000

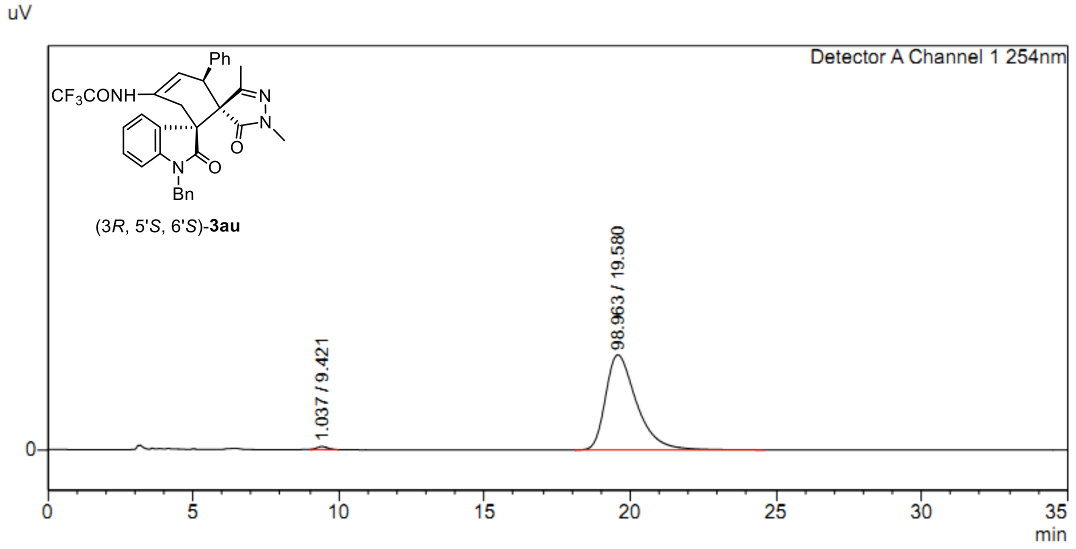

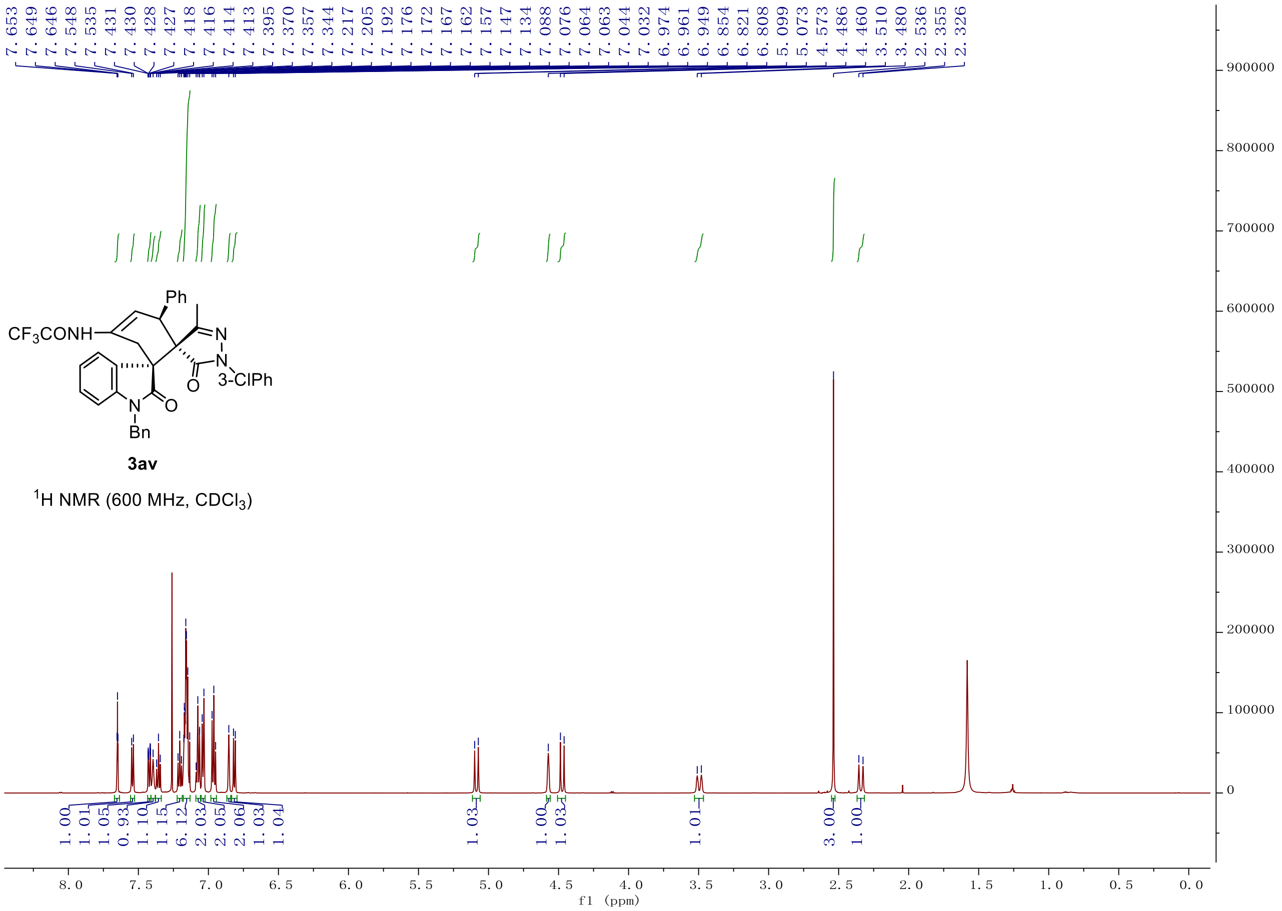

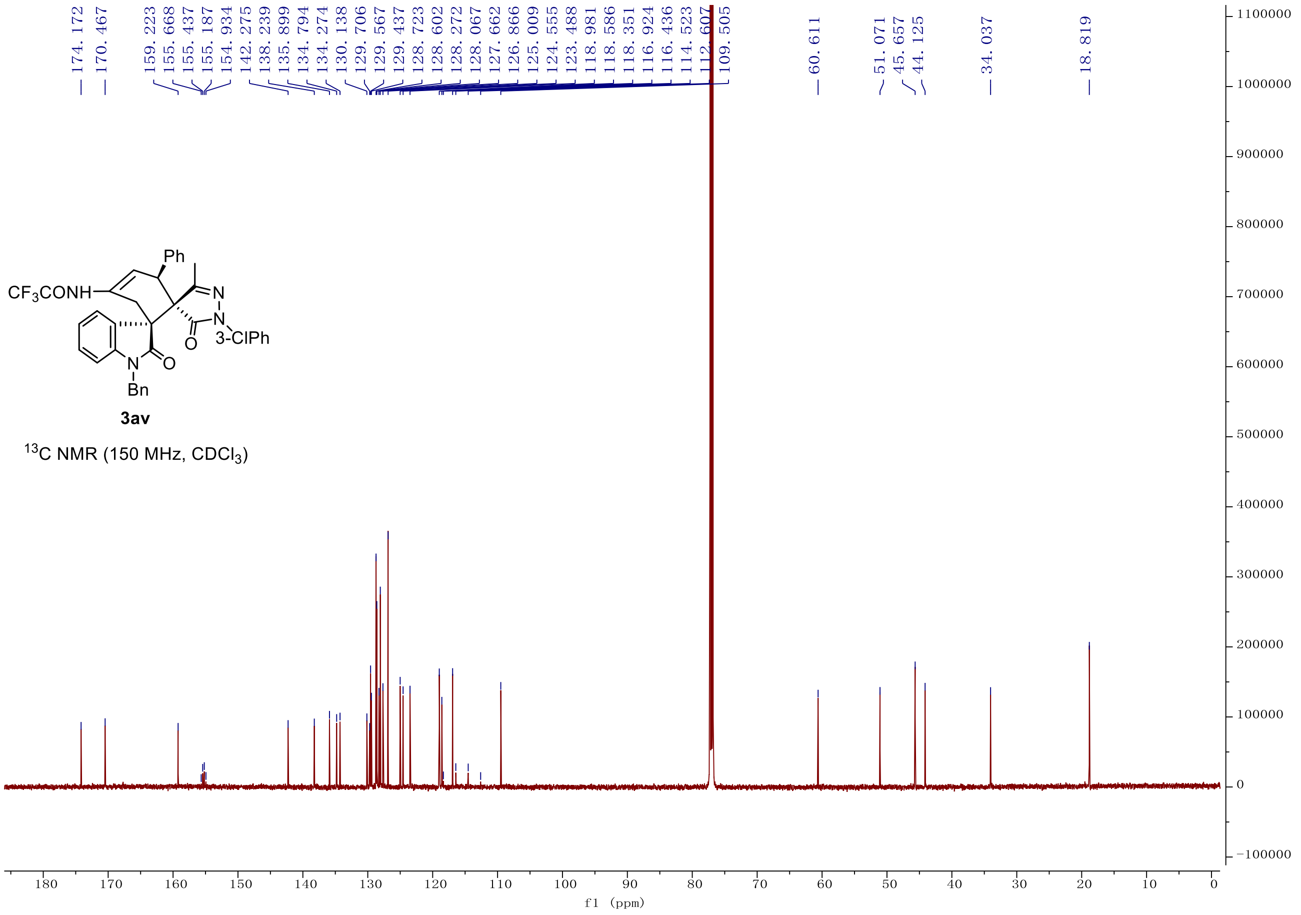
174.172
170.467
159.223
155.668
155.437
155.187
154.934
142.275
138.239
135.899
134.794
134.274
130.138
129.706
129.567
129.437
128.723
128.602
128.272
128.067
127.662
126.866
125.009
124.555
123.488
118.981
118.586
118.351
116.924
116.436
114.523
112.607
109.505
60.611
51.071
45.657
44.125
34.037
18.819
Ph
CF_3CONH
N
N
3-ClPh
O
N
O
Bn
3av
^{13}C NMR (150 MHz, $CDCl_3$)
f1 (ppm)

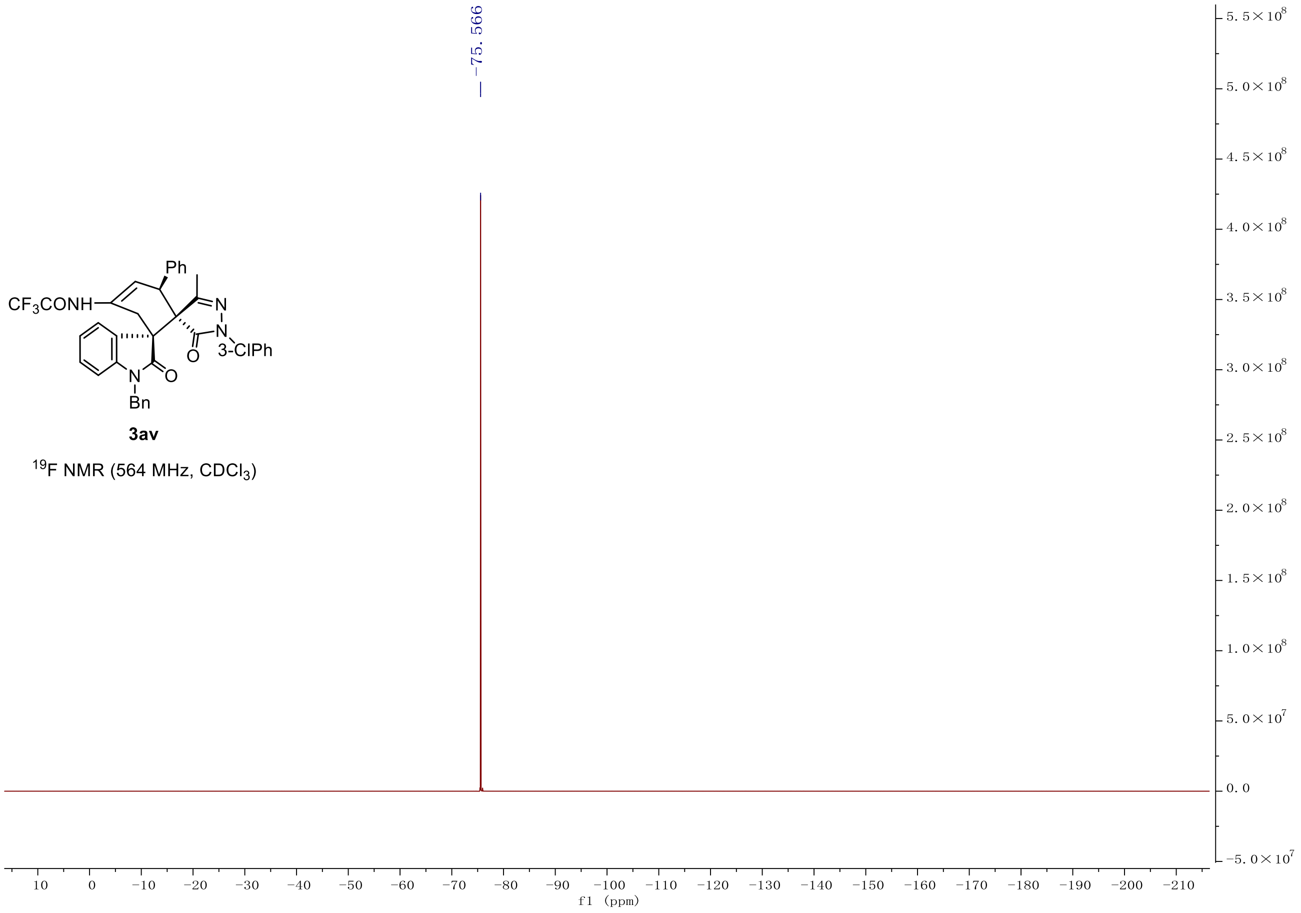
−75.566
Ph
CF_3CONH
N
N
3-ClPh
O
N
O
Bn
3av
^{19}F NMR (564 MHz, $CDCl_3$)
f1 (ppm)

Peak Analysis Report

Detector A Channel 1 254nm

No.	Ret. Time	Height (mAu)	Area (mAu*min)	Rel. Area (%)
1	5.010	682386	8499435	50.081
2	7.847	384000	8471798	49.919
Total		1066386	16971233	100.000

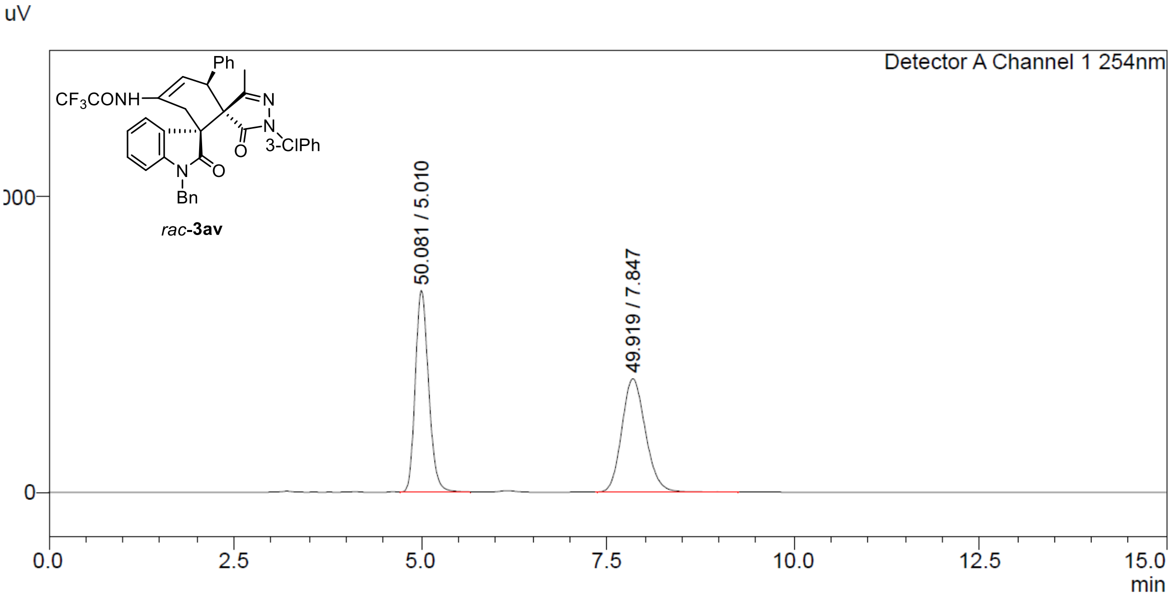

Peak Analysis Report

Detector A Channel 1 254nm

No.	Ret. Time	Height (mAu)	Area (mAu*min)	Rel. Area (%)
1	5.055	4233	72040	0.617
2	7.989	503167	11603170	99.383
Total		507399	11675210	100.000

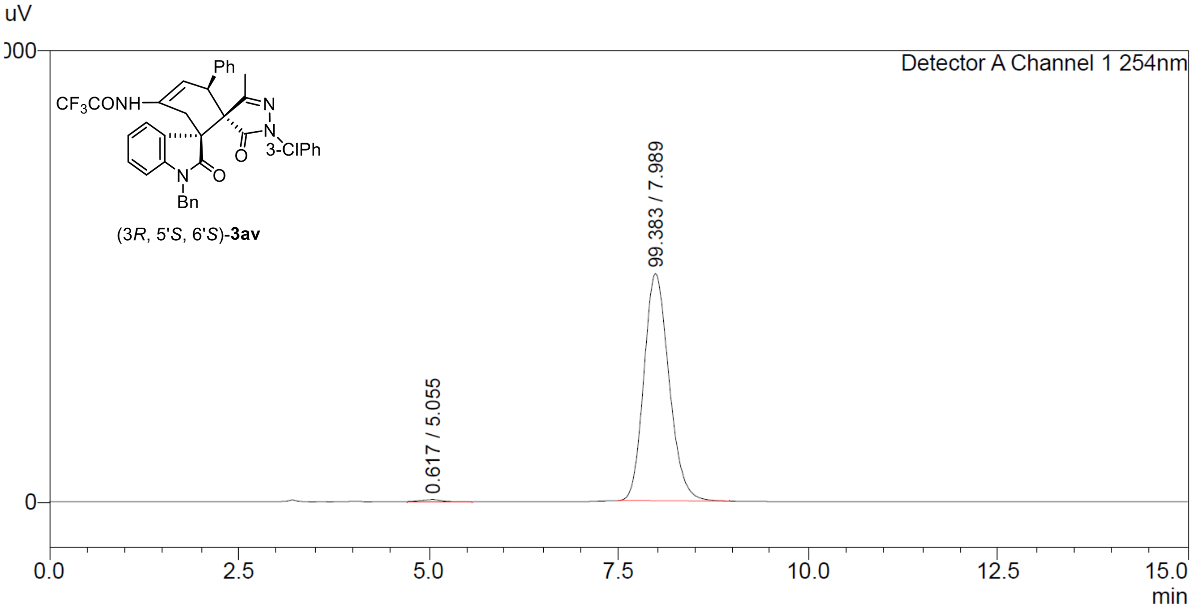

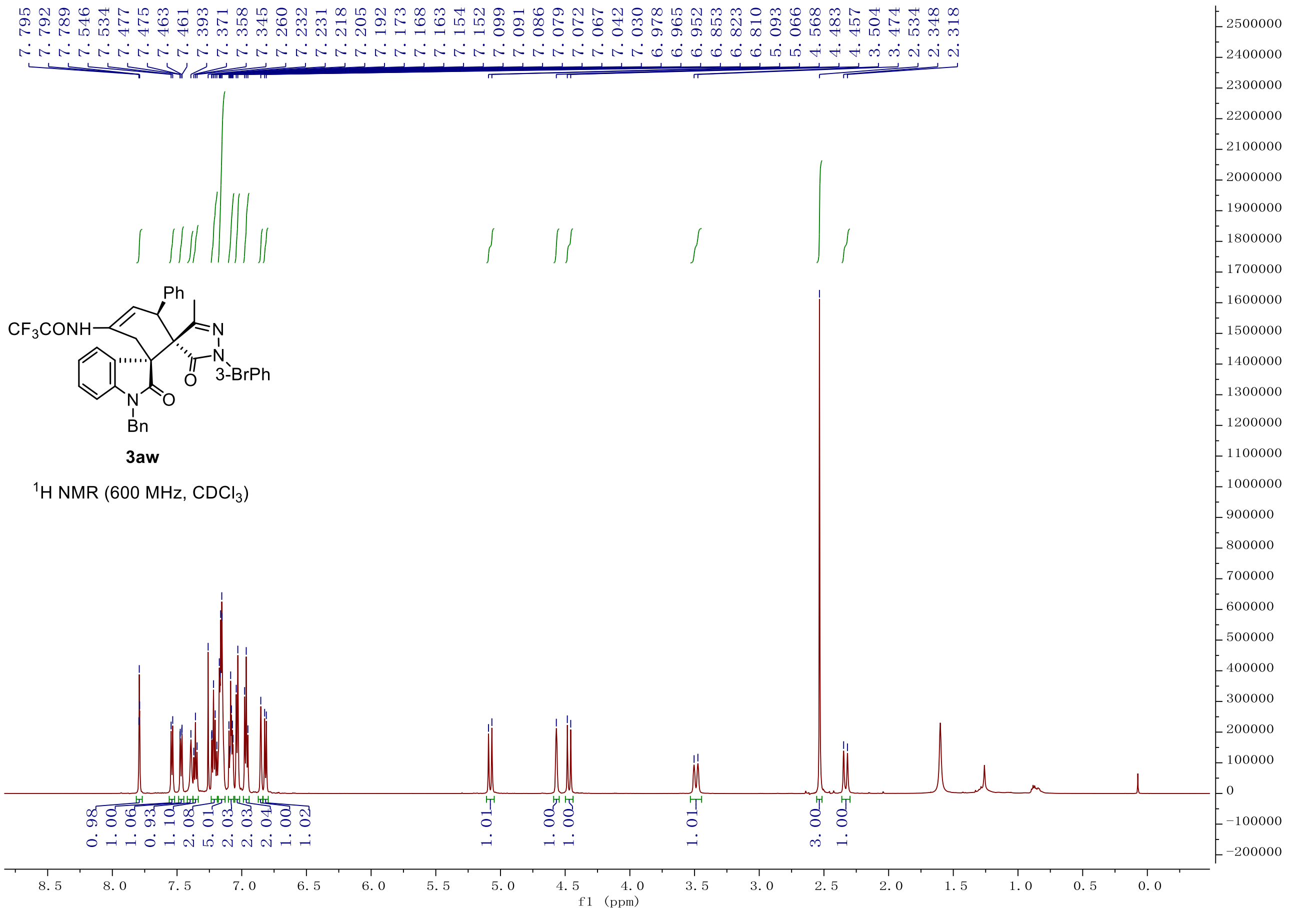
7.795
7.792
7.789
7.546
7.534
7.477
7.475
7.463
7.461
7.393
7.371
7.358
7.345
7.260
7.232
7.231
7.218
7.205
7.192
7.173
7.168
7.163
7.154
7.152
7.099
7.091
7.086
7.079
7.072
7.067
7.042
7.030
6.978
6.965
6.952
6.853
6.823
6.810
5.093
5.066
4.568
4.483
4.457
3.504
3.474
2.534
2.348
2.318
CF3CONH
Ph
N
N
3-BrPh
O
N
O
Bn
3aw
1H NMR (600 MHz, CDCl3)
0.98
1.00
1.06
0.93
1.10
2.08
5.01
2.03
2.03
2.04
1.00
1.02
1.01
1.00
1.00
1.01
3.00
1.00
f1 (ppm)

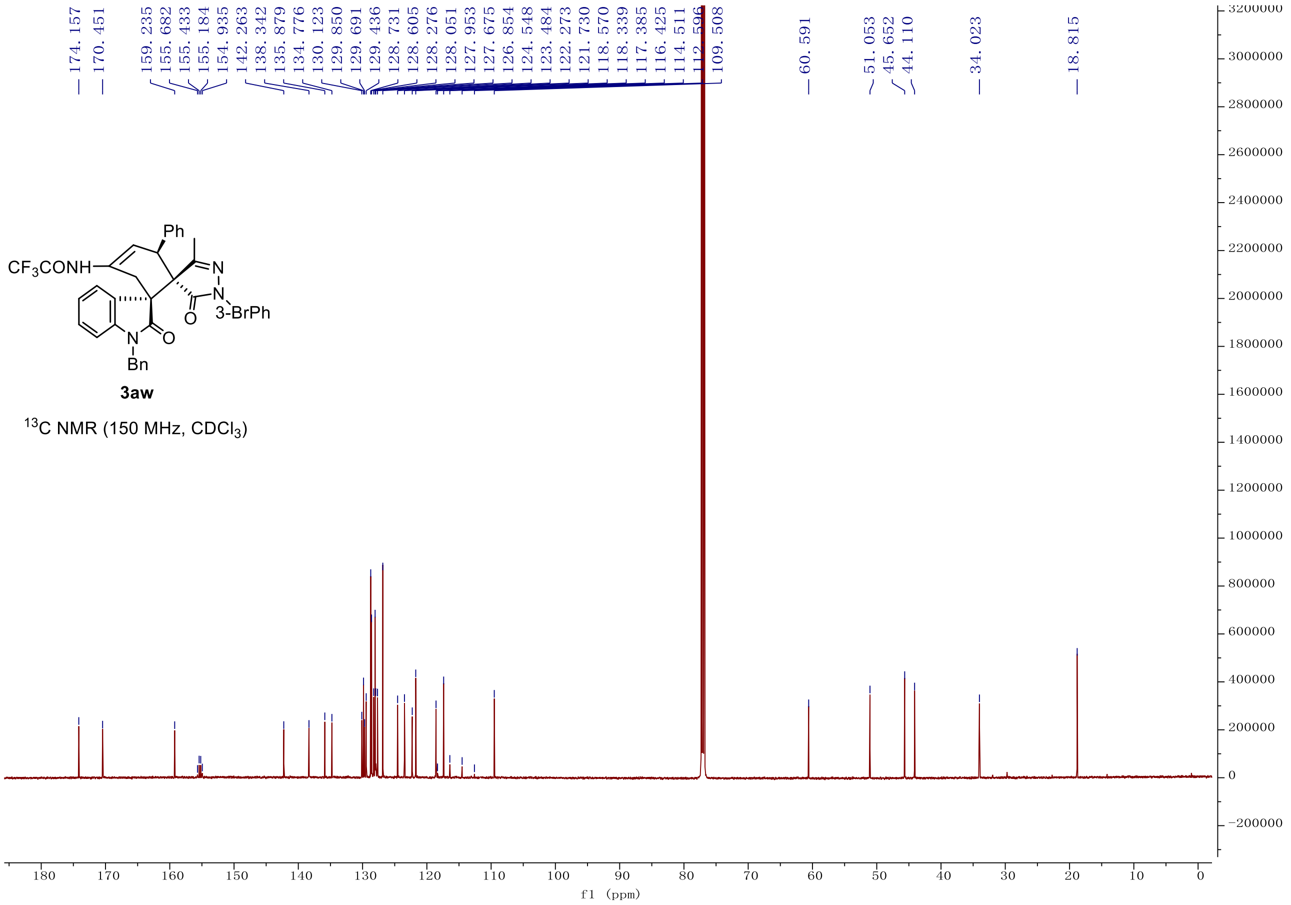
174.157
170.451
159.235
155.682
155.433
155.184
154.935
142.263
138.342
135.879
134.776
130.123
129.850
129.691
129.436
128.731
128.605
128.276
128.051
127.953
127.675
126.854
124.548
123.484
122.273
121.730
118.570
118.339
117.385
116.425
114.511
112.596
109.508
60.591
51.053
45.652
44.110
34.023
18.815
CF3CONH
Ph
N
N
3-BrPh
O
N
O
Bn
3aw
13C NMR (150 MHz, CDCl3)
f1 (ppm)

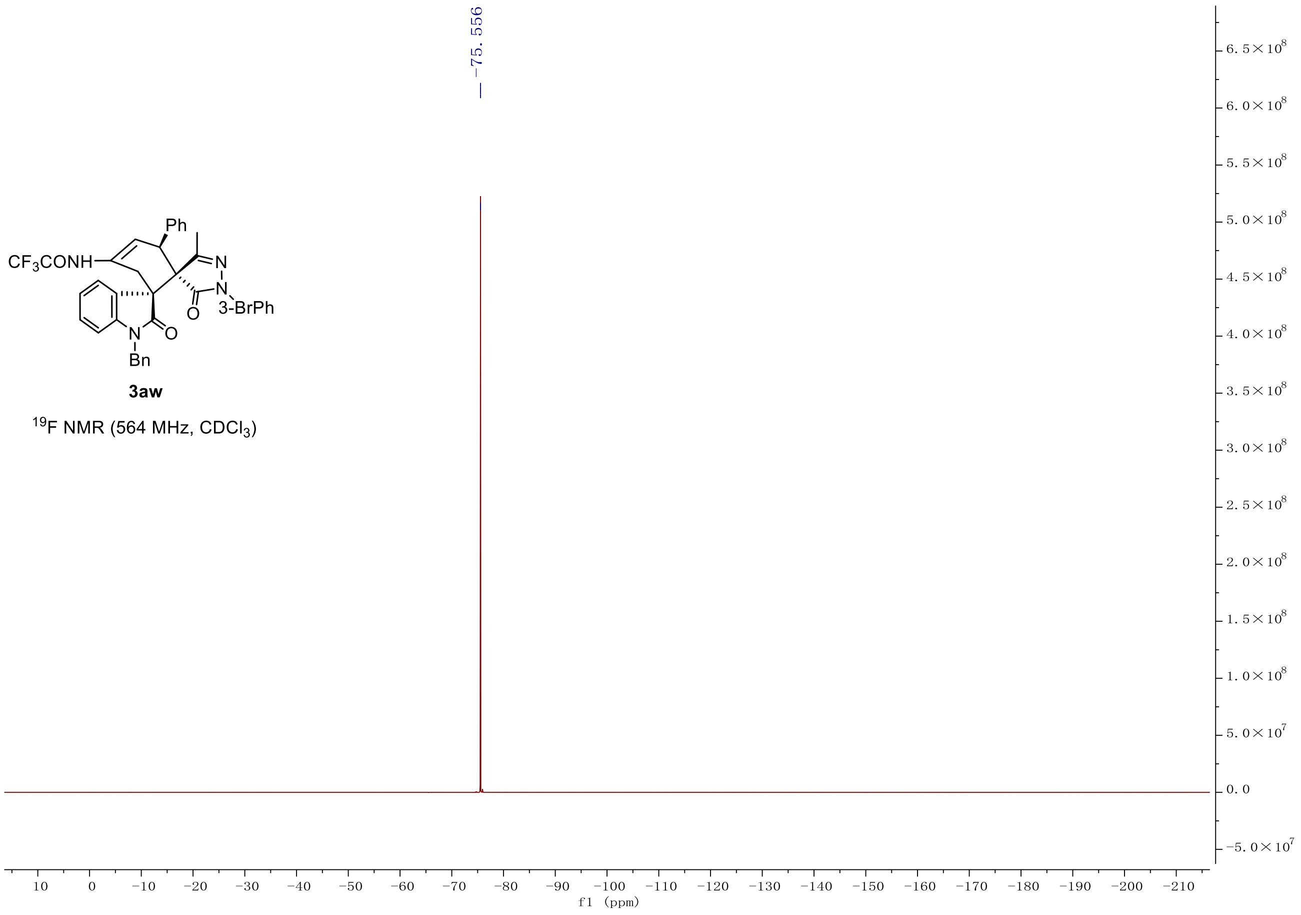

Peak Analysis Report

Detector A Channel 1 254nm

No.	Ret. Time	Height (mAu)	Area (mAu*min)	Rel. Area (%)
1	5.099	847497	10748793	49.818
2	8.561	395628	10827288	50.182
Total		1243125	21576081	100.000

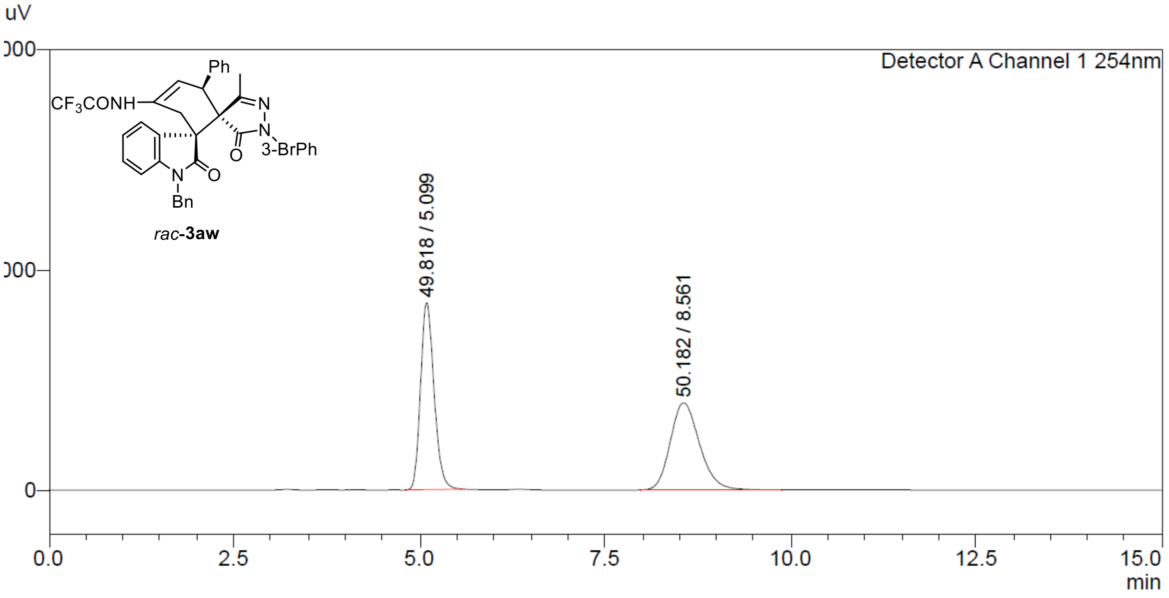

Peak Analysis Report

Detector A Channel 1 254nm

No.	Ret. Time	Height (mAu)	Area (mAu*min)	Rel. Area (%)
1	5.078	24161	236237	0.878
2	8.536	971769	26665013	99.122
Total		995930	26901250	100.000

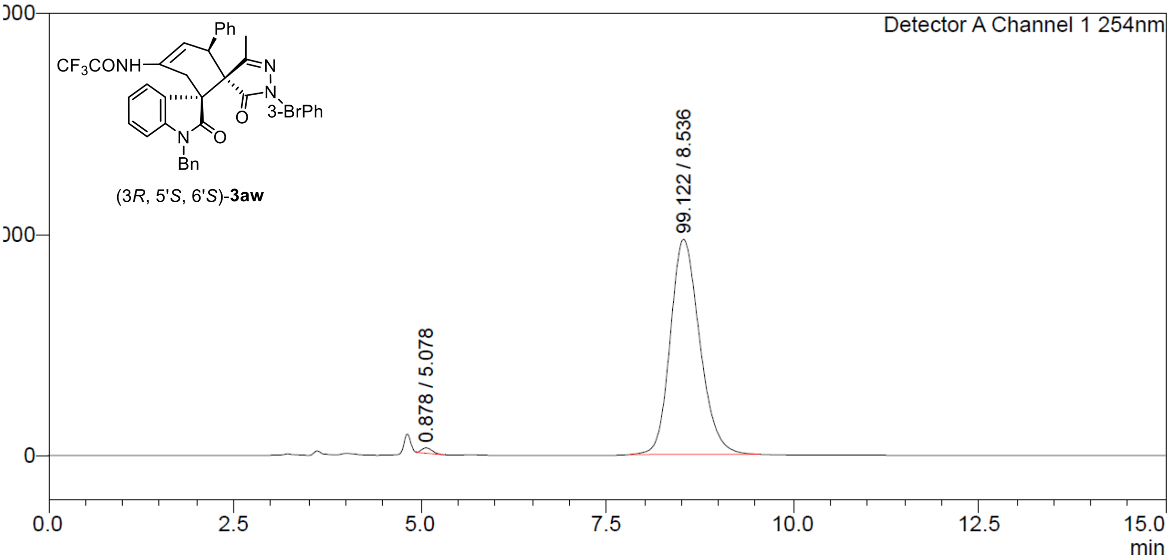

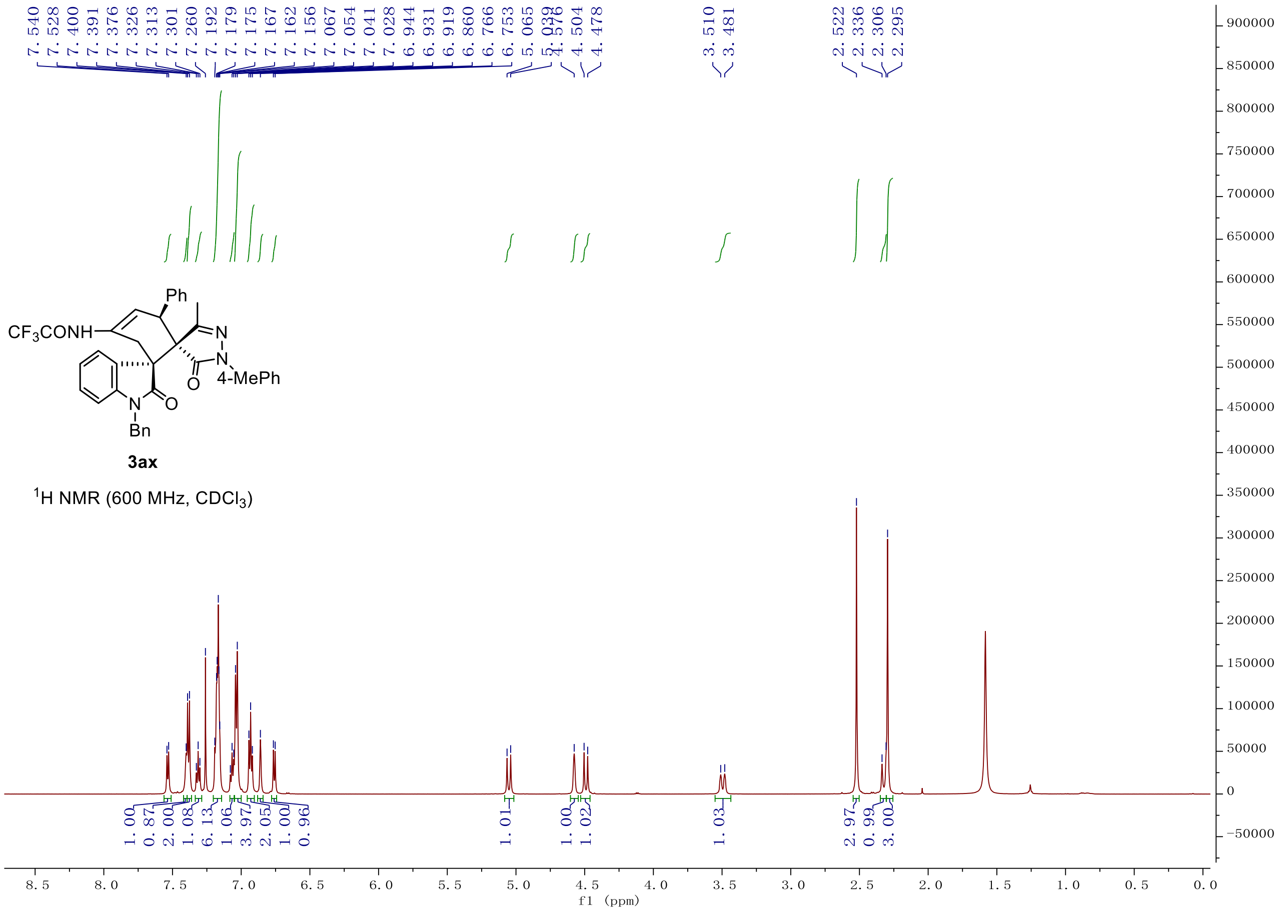

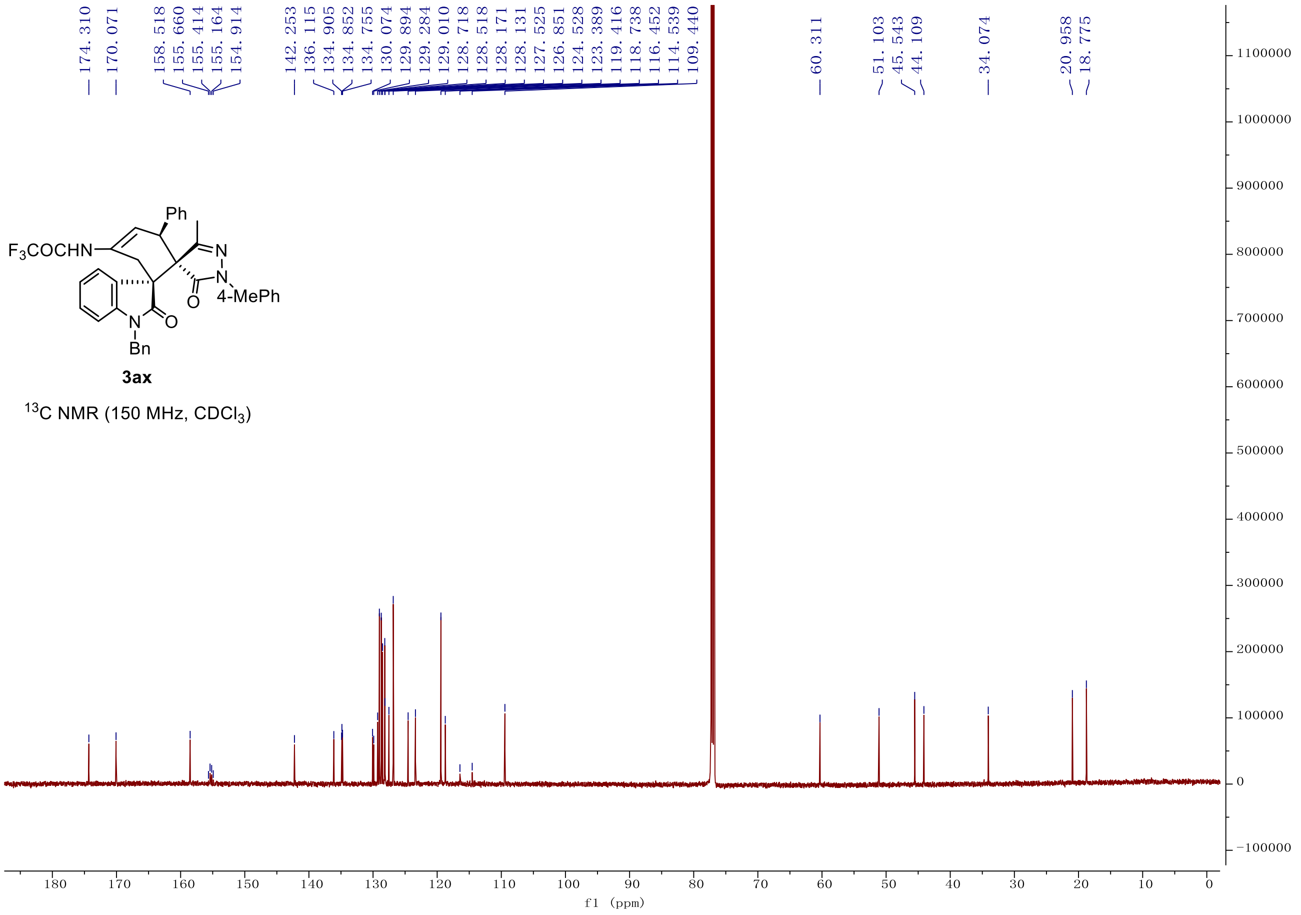

174.310
170.071
158.518
155.660
155.414
155.164
154.914
142.253
136.115
134.905
134.852
134.755
130.074
129.894
129.284
129.010
128.718
128.518
128.171
128.131
127.525
126.851
124.528
123.389
119.416
118.738
116.452
114.539
109.440
60.311
51.103
45.543
44.109
34.074
20.958
18.775
F3COCHN
Ph
N
N
4-MePh
O
N
O
Bn
3ax
13C NMR (150 MHz, CDCl3)
f1 (ppm)

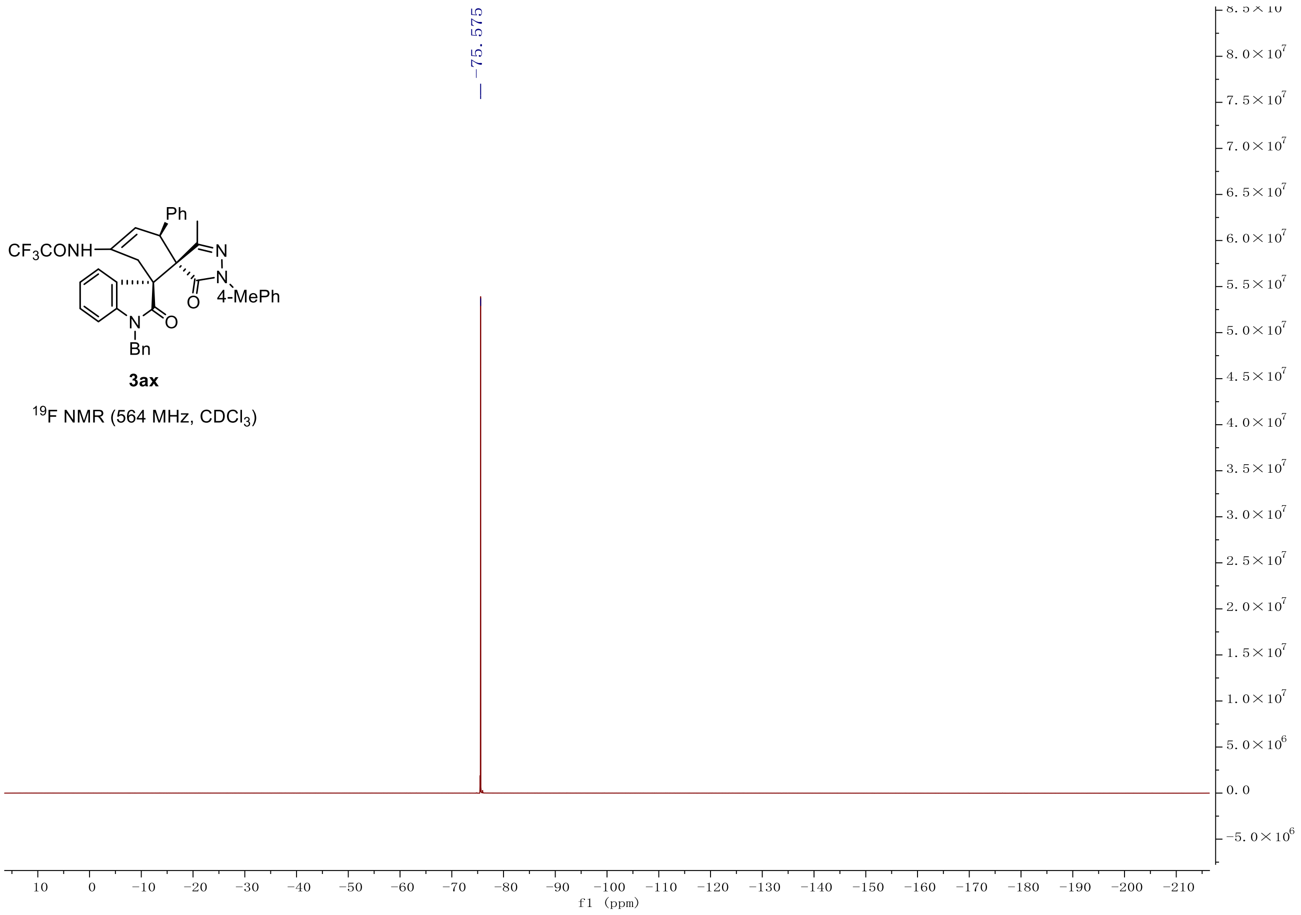

-75.575
CF3CONH
Ph
N
N
4-MePh
O
N
O
Bn
3ax
19F NMR (564 MHz, CDCl3)
f1 (ppm)

Peak Analysis Report

Detector A Channel 1 254nm

No.	Ret. Time	Height (mAu)	Area (mAu*min)	Rel. Area (%)
1	22.176	2278	196446	49.639
2	25.693	1964	199307	50.361
Total		4242	395753	100.000

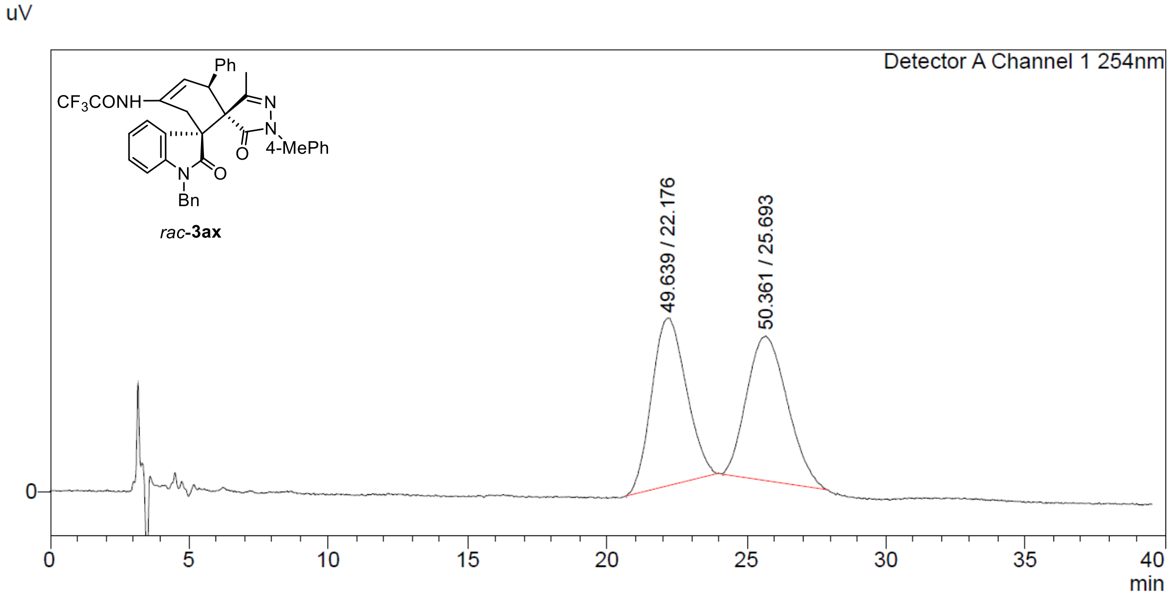

Peak Analysis Report

Detector A Channel 1 254nm

No.	Ret. Time	Height (mAu)	Area (mAu*min)	Rel. Area (%)
1	22.221	77	3982	0.044
2	25.524	82488	9110543	99.956
Total		82565	9114525	100.000

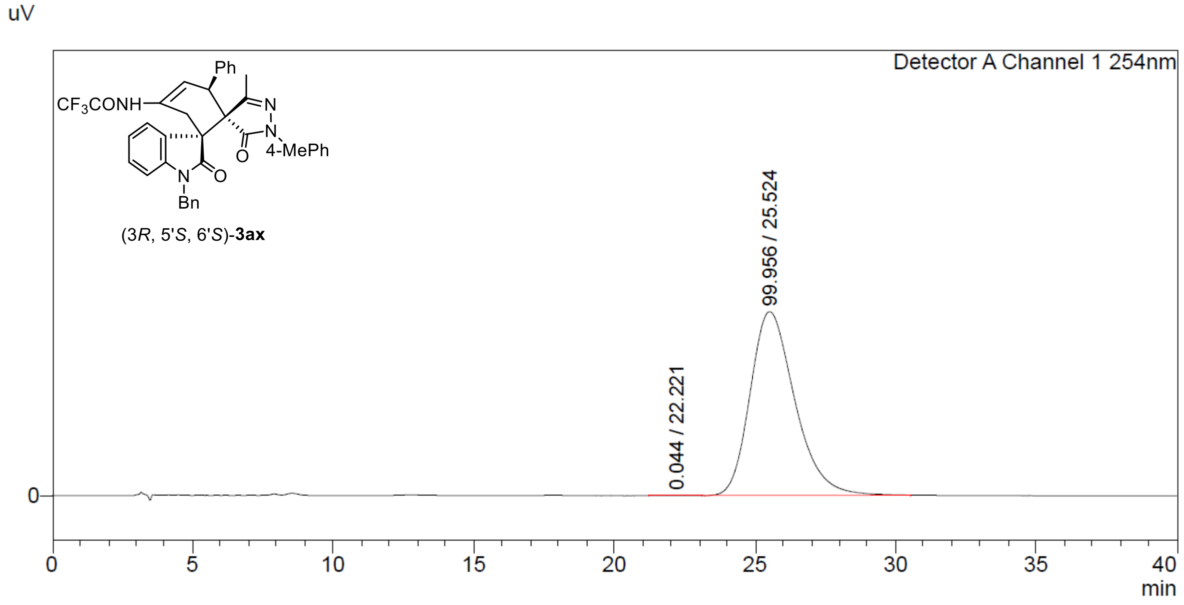

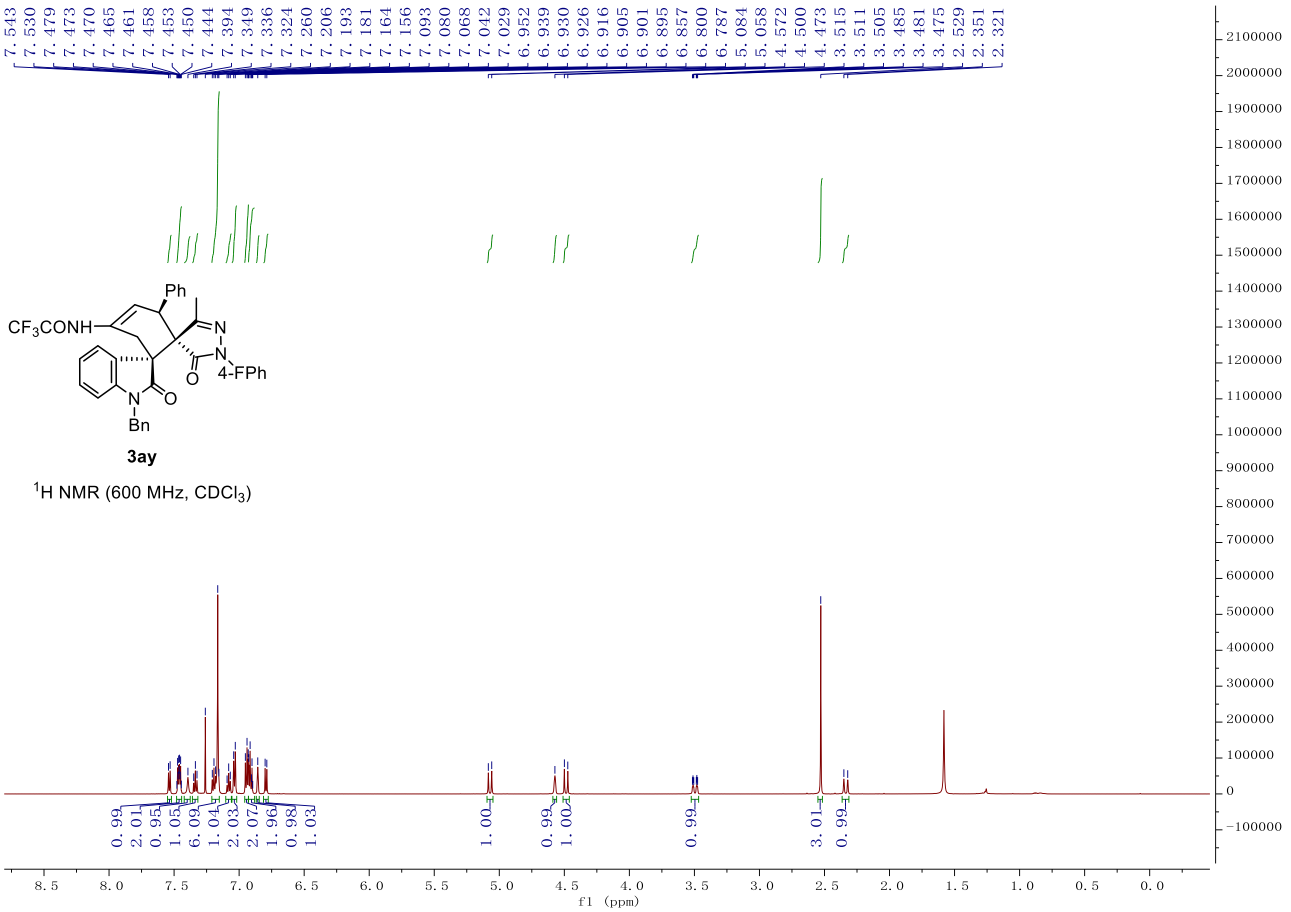
7.543 7.530 7.479 7.473 7.470 7.465 7.461 7.458 7.453 7.450 7.444 7.394 7.349 7.336 7.324 7.260 7.206 7.193 7.181 7.164 7.156 7.093 7.080 7.068 7.042 7.029 6.952 6.939 6.930 6.926 6.916 6.905 6.901 6.895 6.857 6.800 6.787 5.084 5.058 4.572 4.500 4.473 3.515 3.511 3.505 3.485 3.481 3.475 2.529 2.351 2.321
0.99 2.01 0.95 1.05 6.09 1.04 2.03 2.07 1.96 0.98 1.03 1.00 0.99 1.00 0.99 3.01 0.99
CF3CONH
Ph
N
N
4-FPh
O
N
O
Bn
3ay
1H NMR (600 MHz, CDCl3)
f1 (ppm)

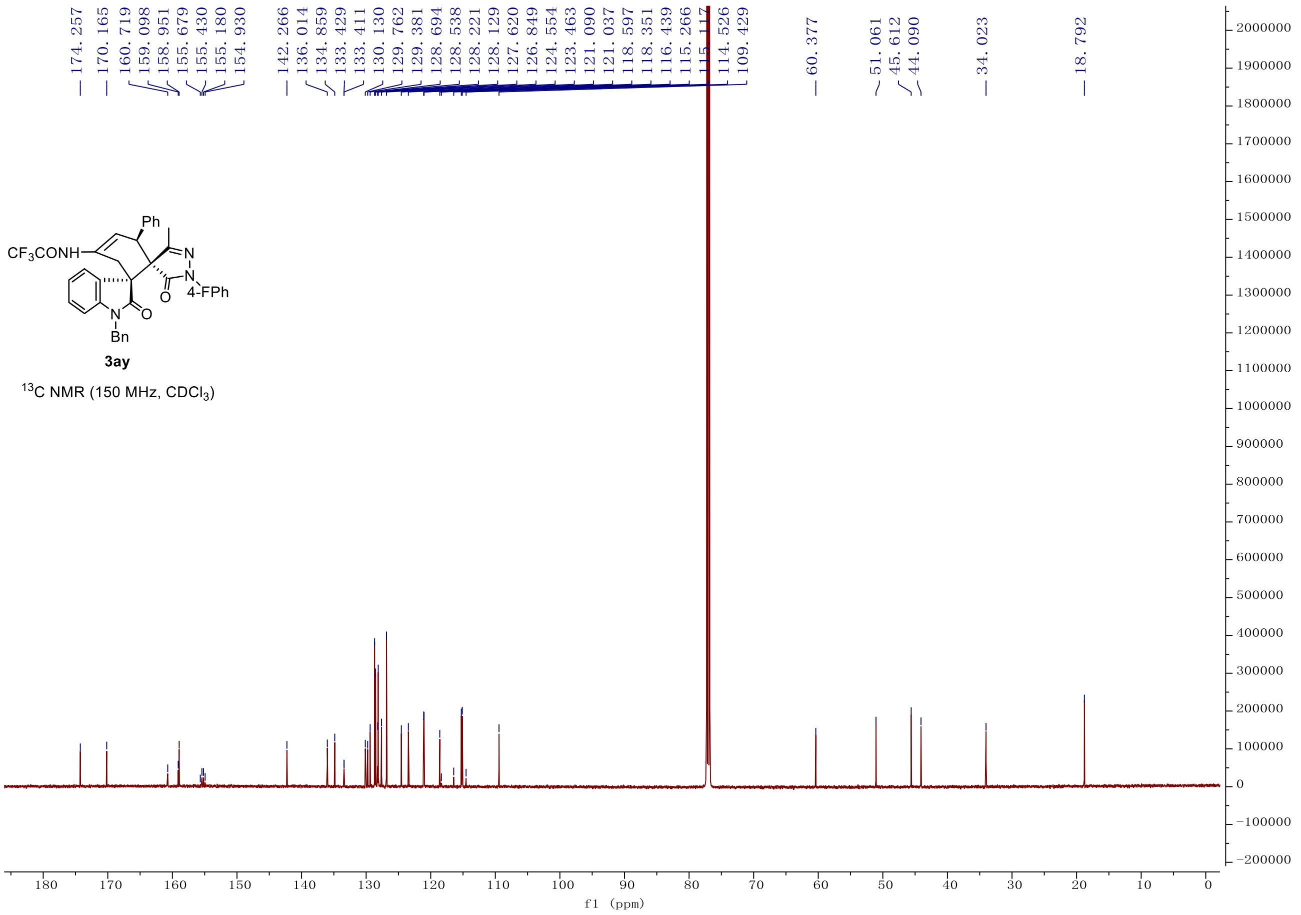
174.257 170.165 160.719 159.098 158.951 155.679 155.430 155.180 154.930 142.266 136.014 134.859 133.429 133.411 130.130 129.762 129.381 128.694 128.538 128.221 128.129 127.620 126.849 124.554 123.463 121.090 121.037 118.597 118.351 116.439 115.266 115.117 114.526 109.429 60.377 51.061 45.612 44.090 34.023 18.792
CF3CONH
Ph
N
N
4-FPh
O
N
O
Bn
3ay
13C NMR (150 MHz, CDCl3)
f1 (ppm)

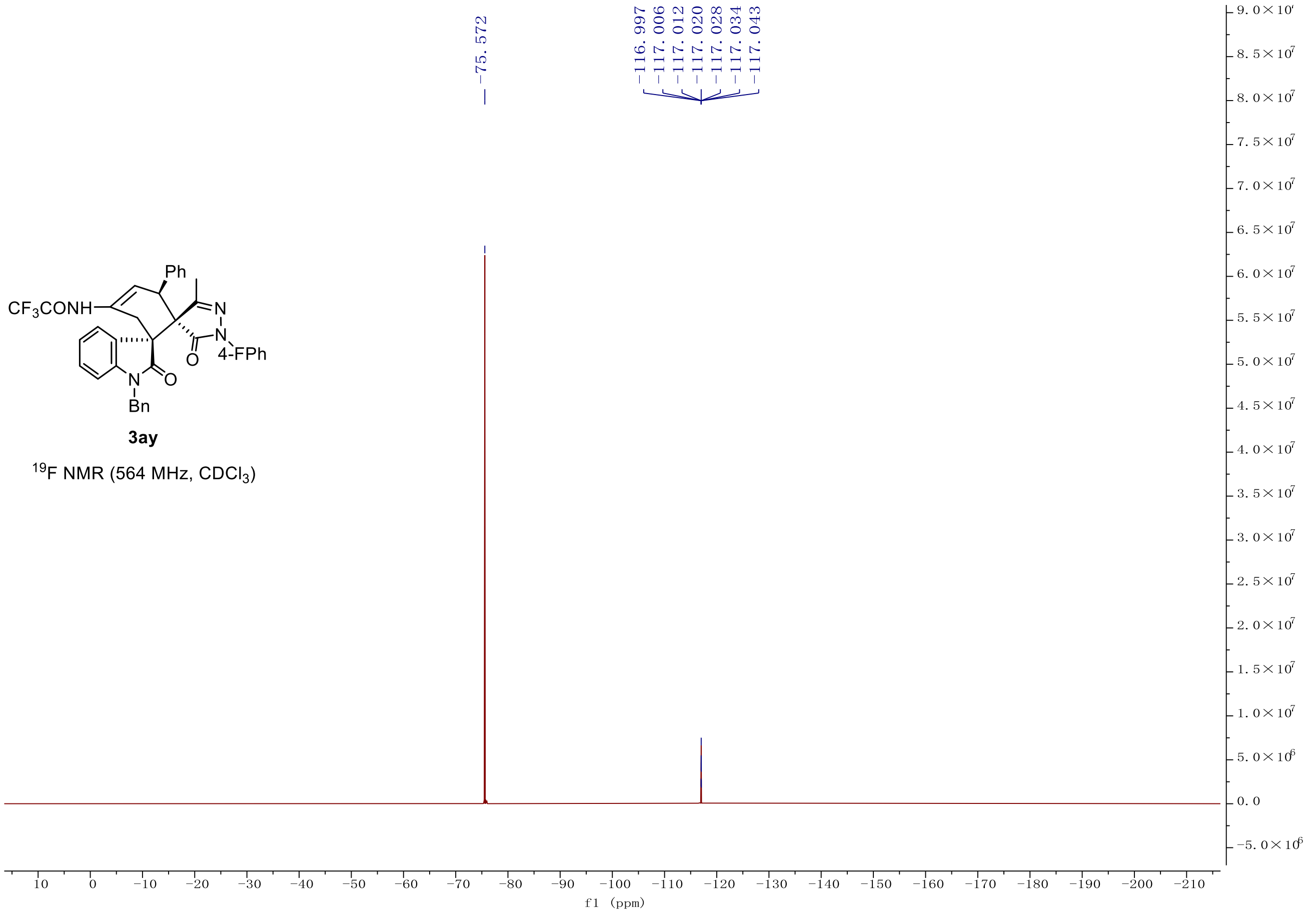

Signal: VWD1 B, Wavelength=254 nm

RT [min]	Type	Width [min]	Area	Height	Area%	Name
7.471	MM	0.4049	15808.1982	650.6248	50.4773	
12.965	MM	0.8127	15509.2139	318.0589	49.5227	
		Sum	31317.4121			

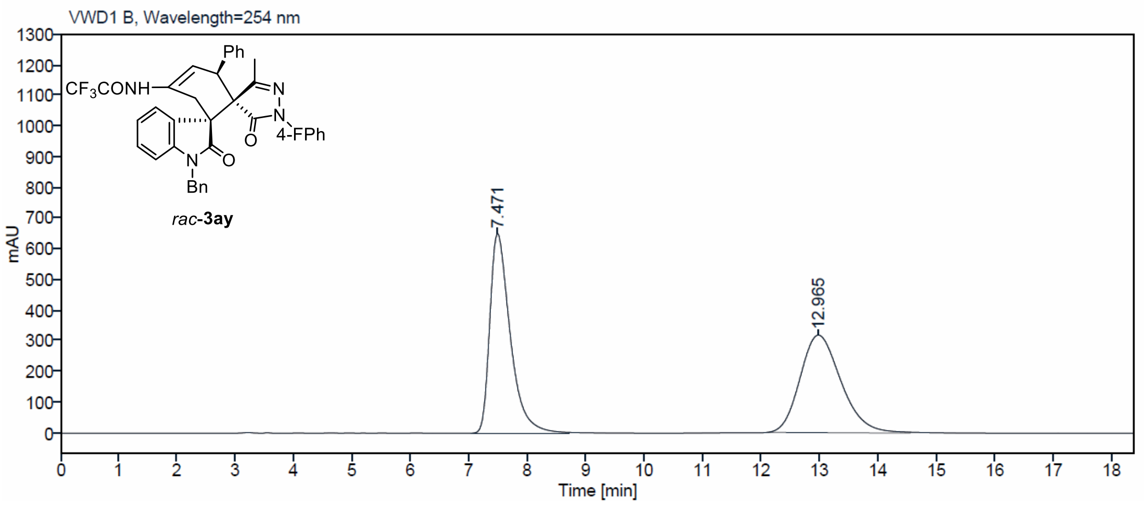

Signal: VWD1 B, Wavelength=254 nm

RT [min]	Type	Width [min]	Area	Height	Area%	Name
7.586	MM	0.4404	128.6687	4.8693	0.7130	
12.936	MM	0.8320	17918.4316	358.9603	99.2870	
		Sum	18047.1004			

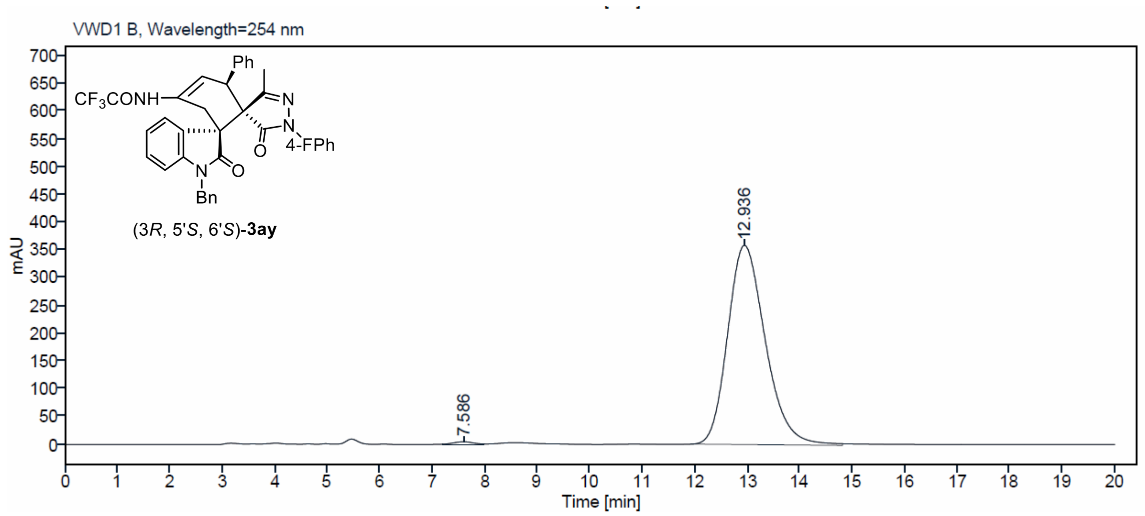

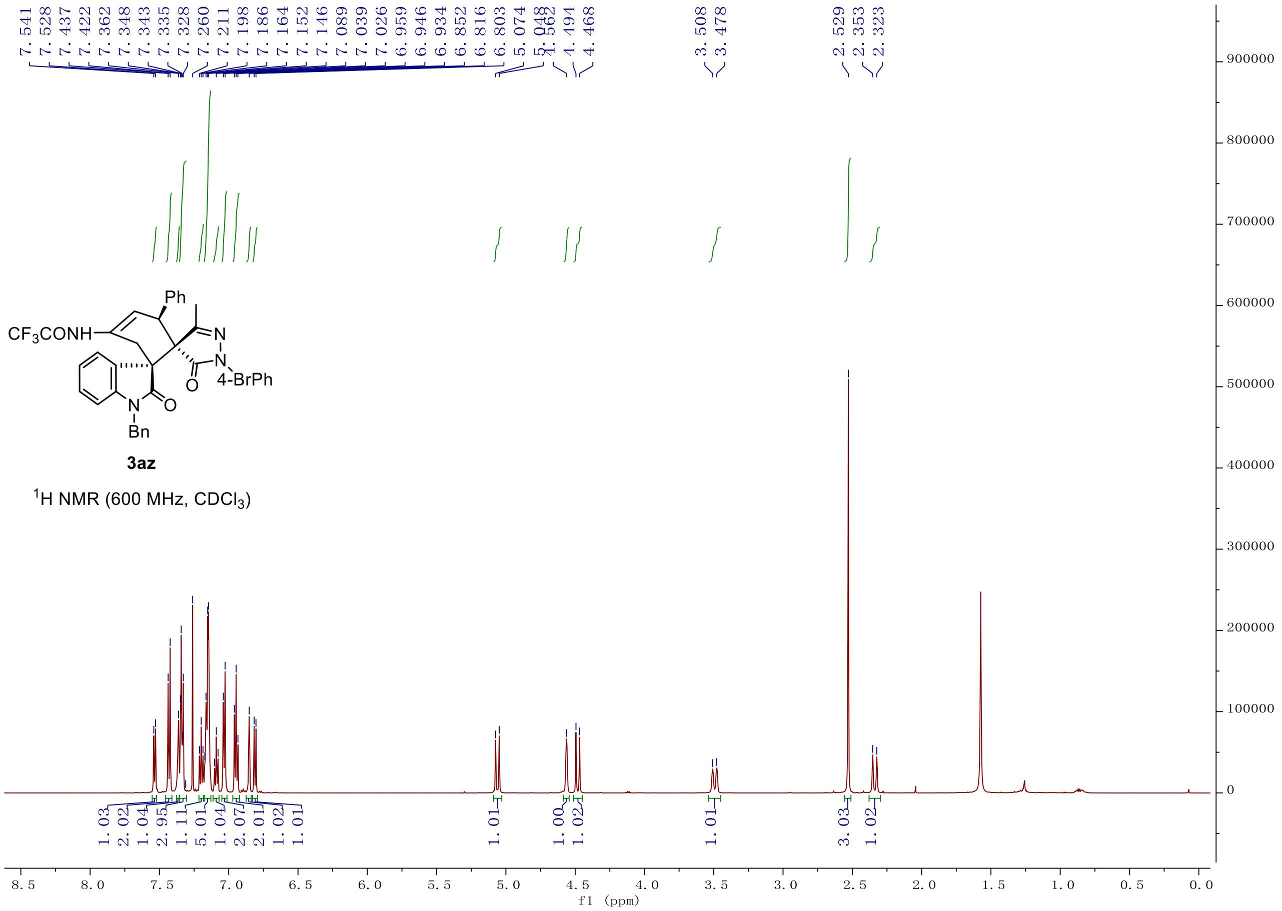

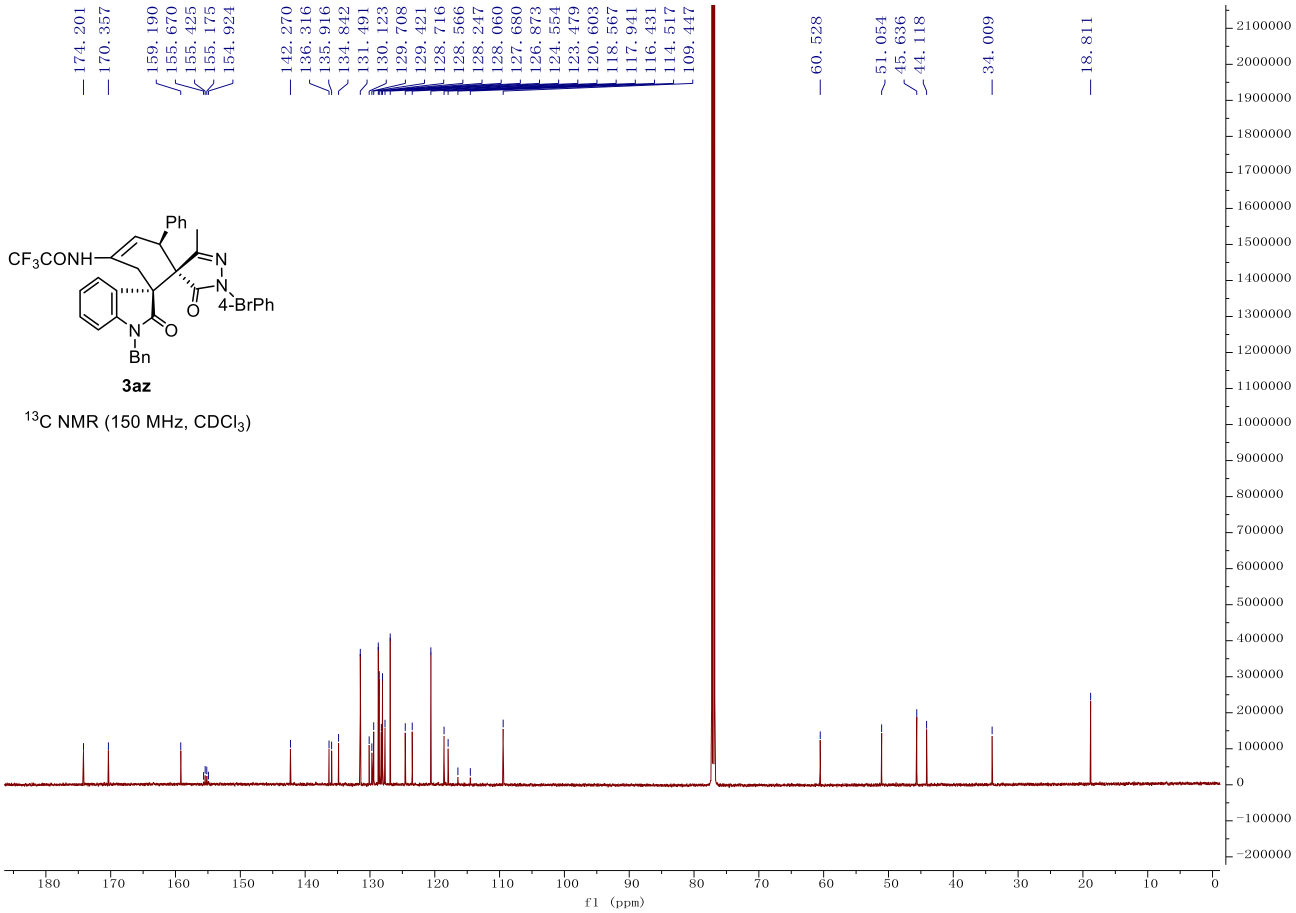

3az

^{13}C NMR (150 MHz, $CDCl_3$)

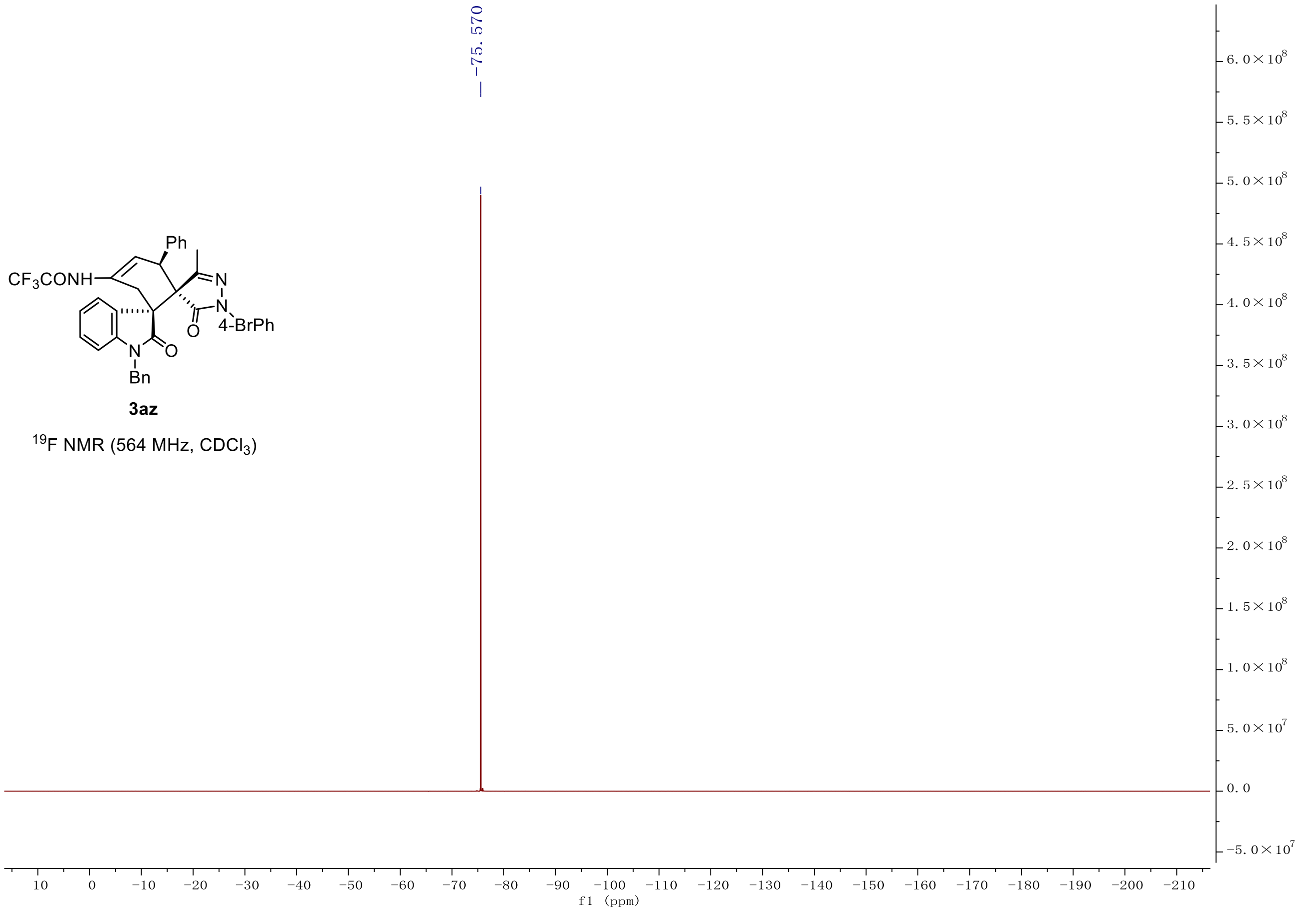

3az

^{19}F NMR (564 MHz, $CDCl_3$)

Peak Analysis Report

Detector A Channel 1 254nm

No.	Ret. Time	Height (mAu)	Area (mAu*min)	Rel. Area (%)
1	17.980	127941	8152169	50.785
2	21.329	88188	7900104	49.215
Total		216129	16052273	100.000

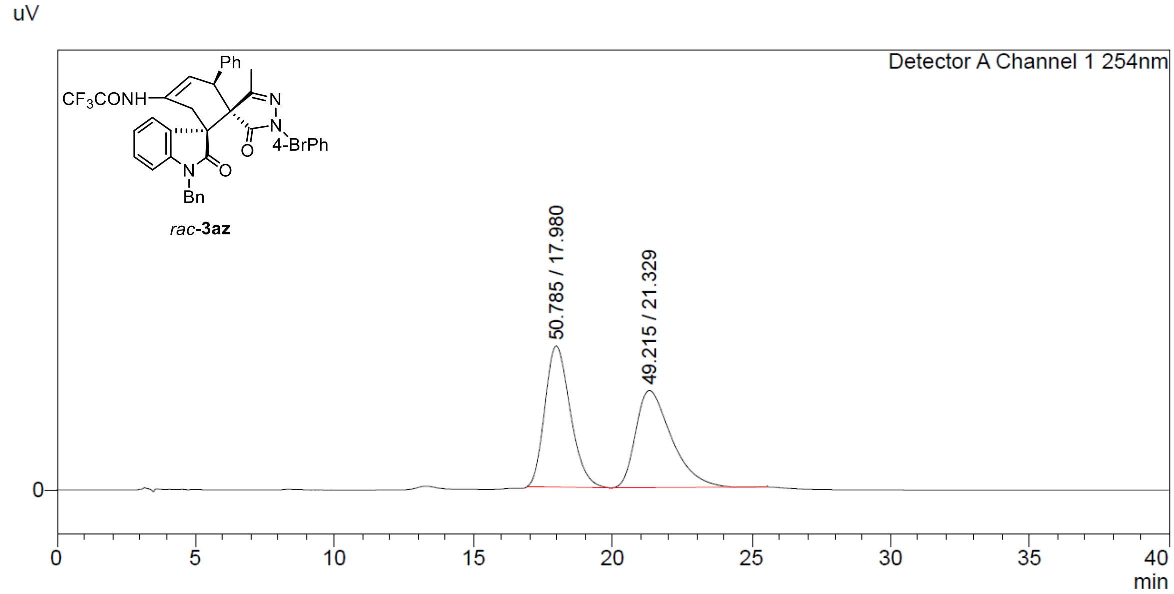

Peak Analysis Report

Detector A Channel 1 254nm

No.	Ret. Time	Height (mAu)	Area (mAu*min)	Rel. Area (%)
1	17.788	299620	18849168	99.983
2	21.344	31	3209	0.017
Total		299651	18852377	100.000

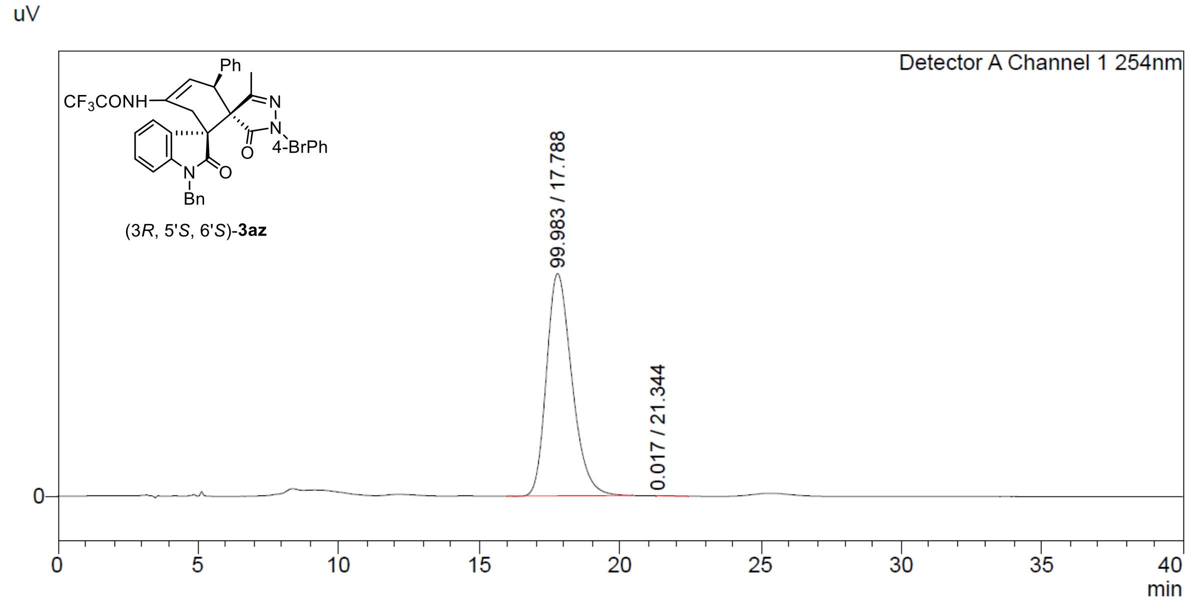

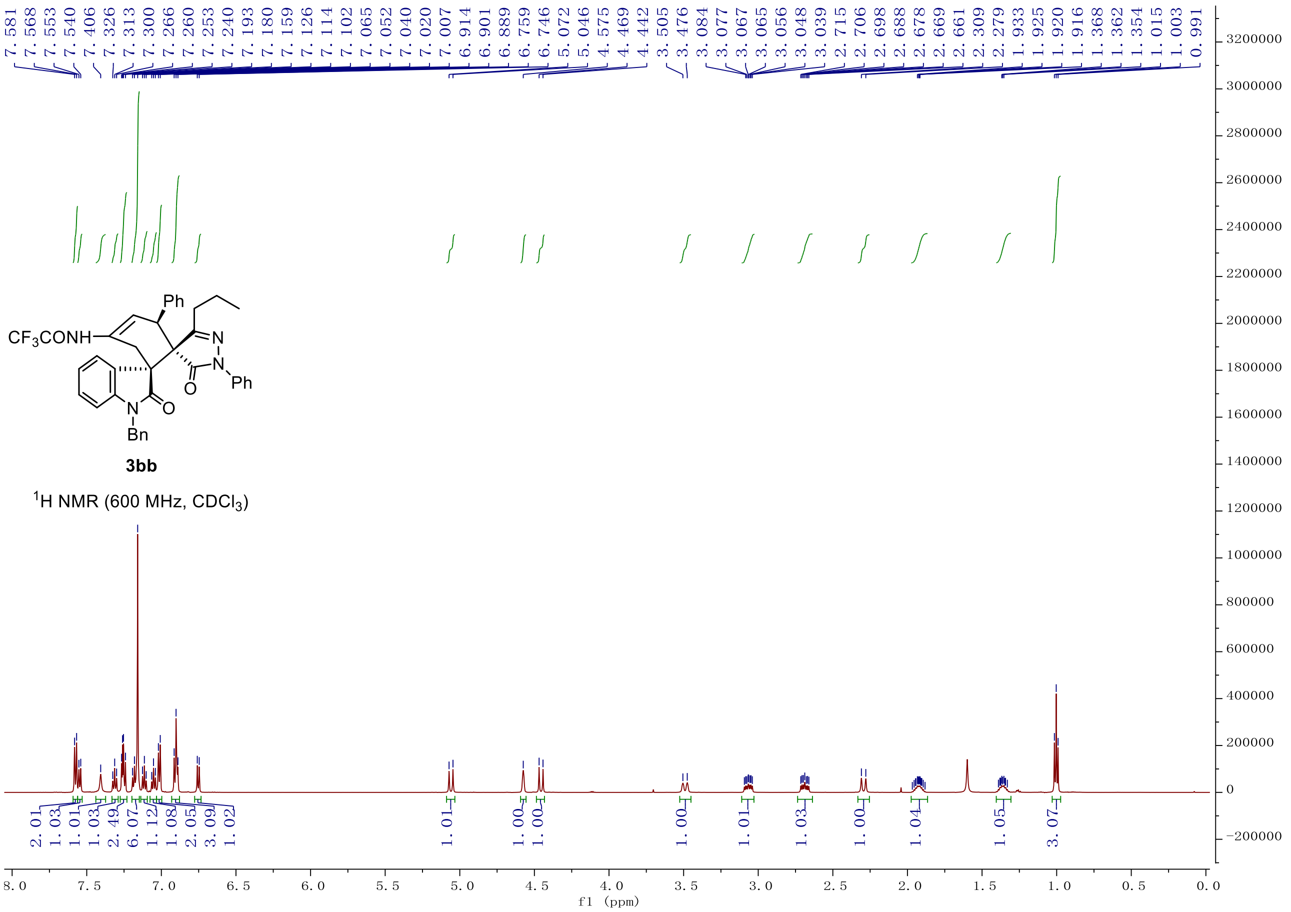

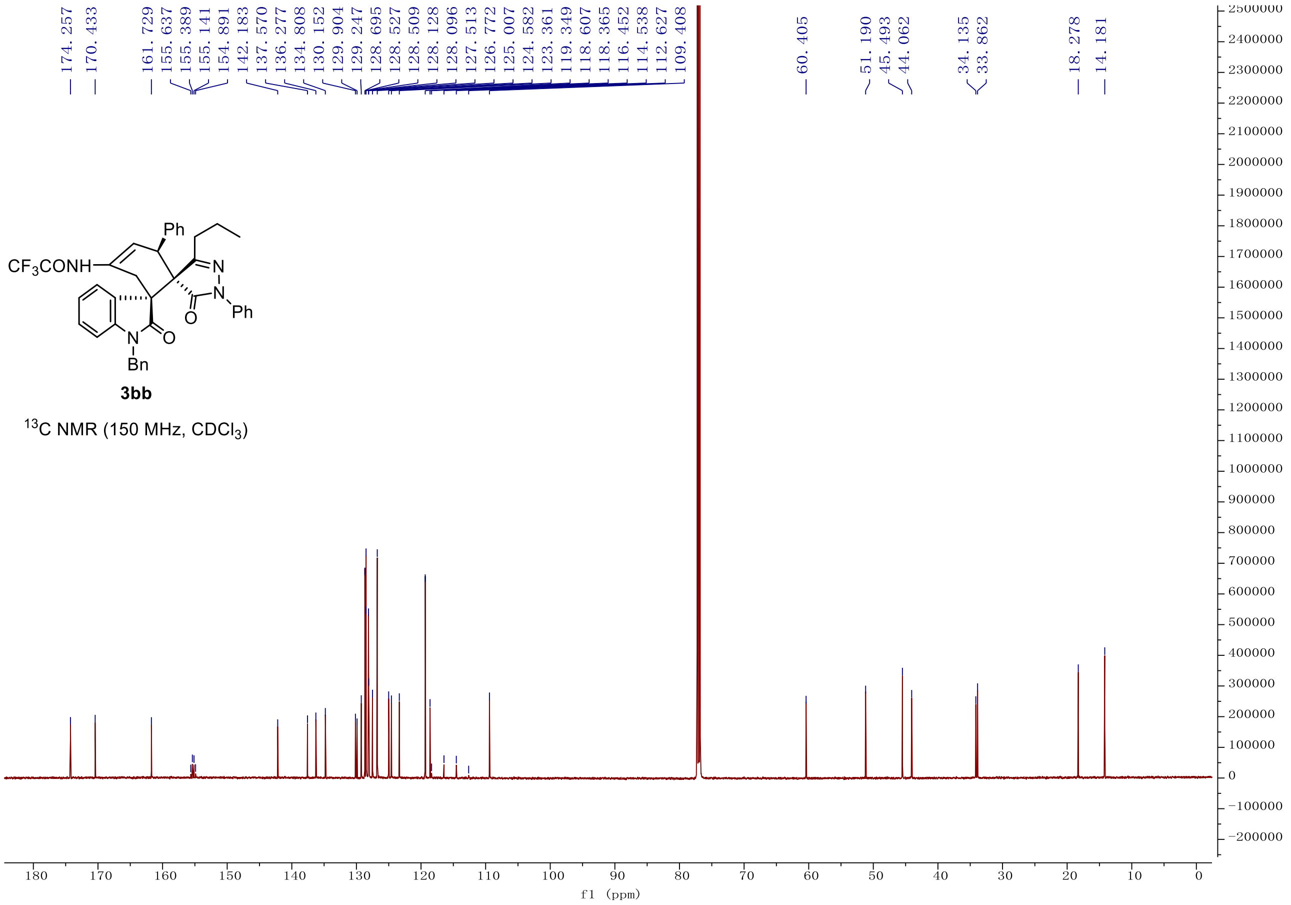

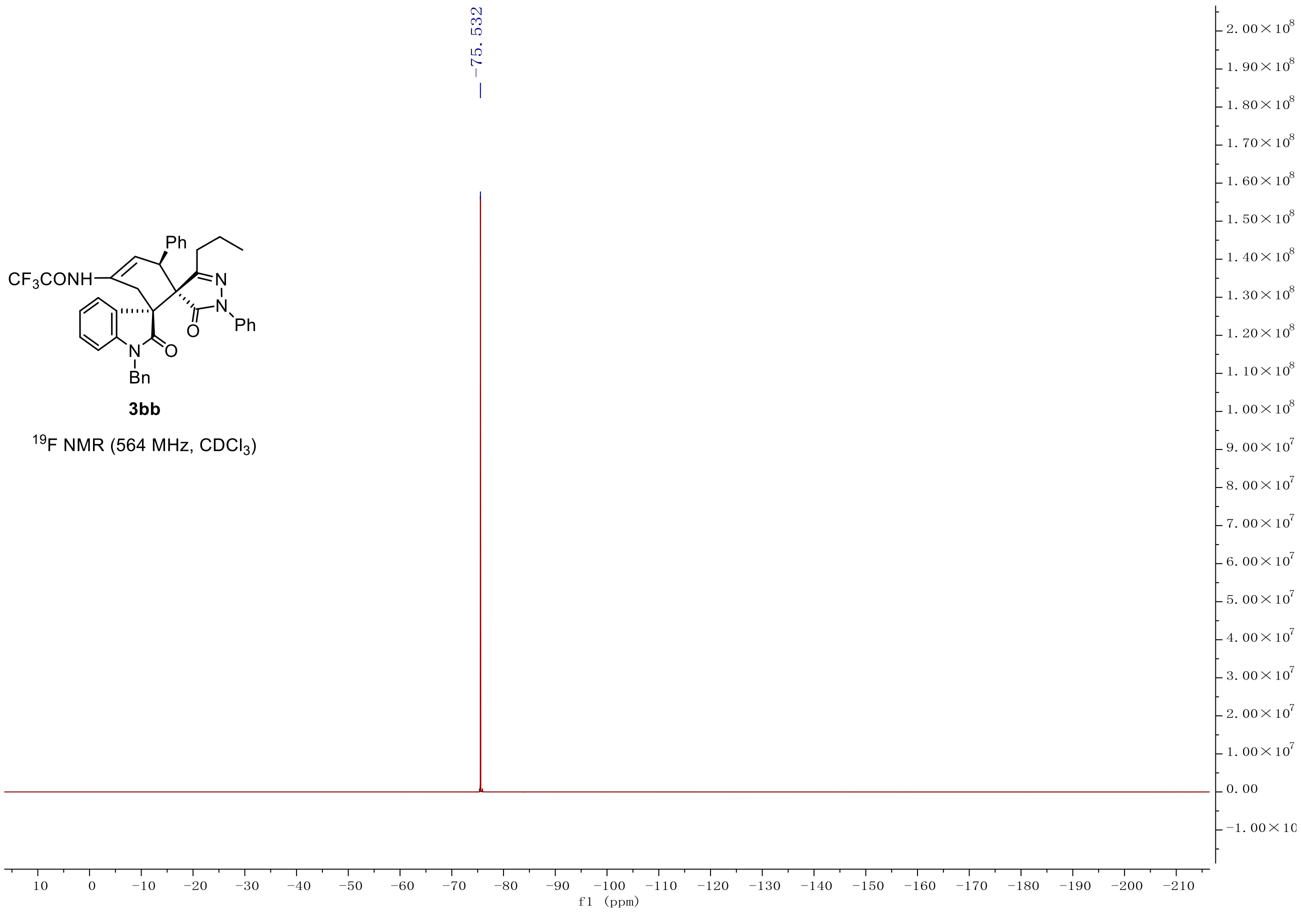

Peak Analysis Report

Detector A Channel 1 254nm

No.	Ret. Time	Height (mAu)	Area (mAu*min)	Rel. Area (%)
1	10.325	546660	19176611	49.877
2	13.532	260307	19271267	50.123
Total		806967	38447877	100.000

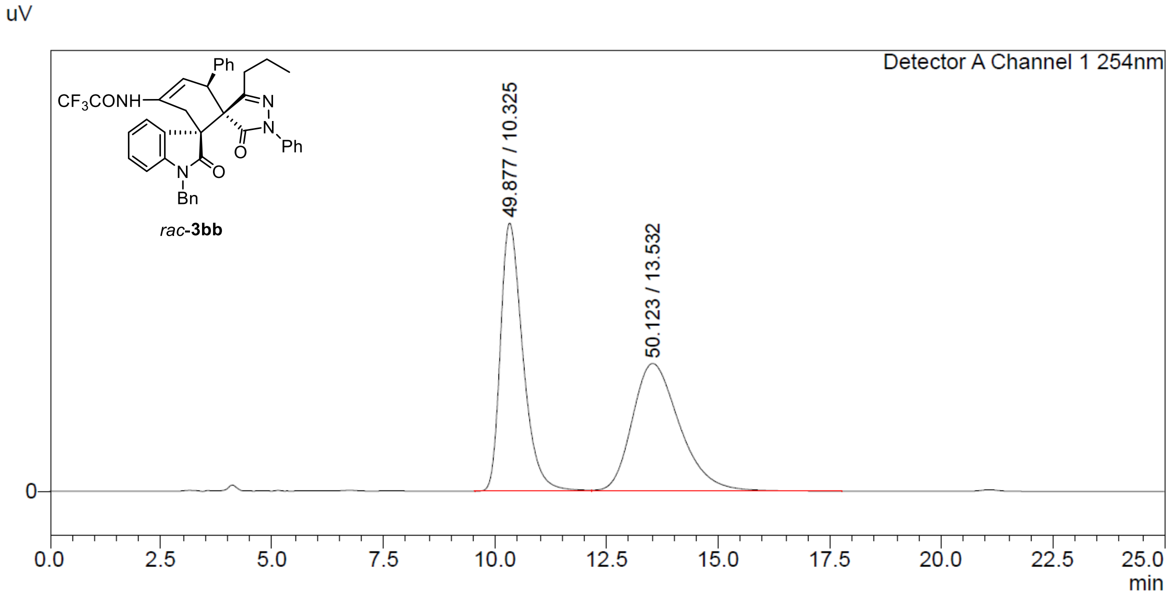

Peak Analysis Report

Detector A Channel 1 254nm

No.	Ret. Time	Height (mAu)	Area (mAu*min)	Rel. Area (%)
1	10.466	43451	1585090	6.397
2	13.551	319238	23192079	93.603
Total		362689	24777169	100.000

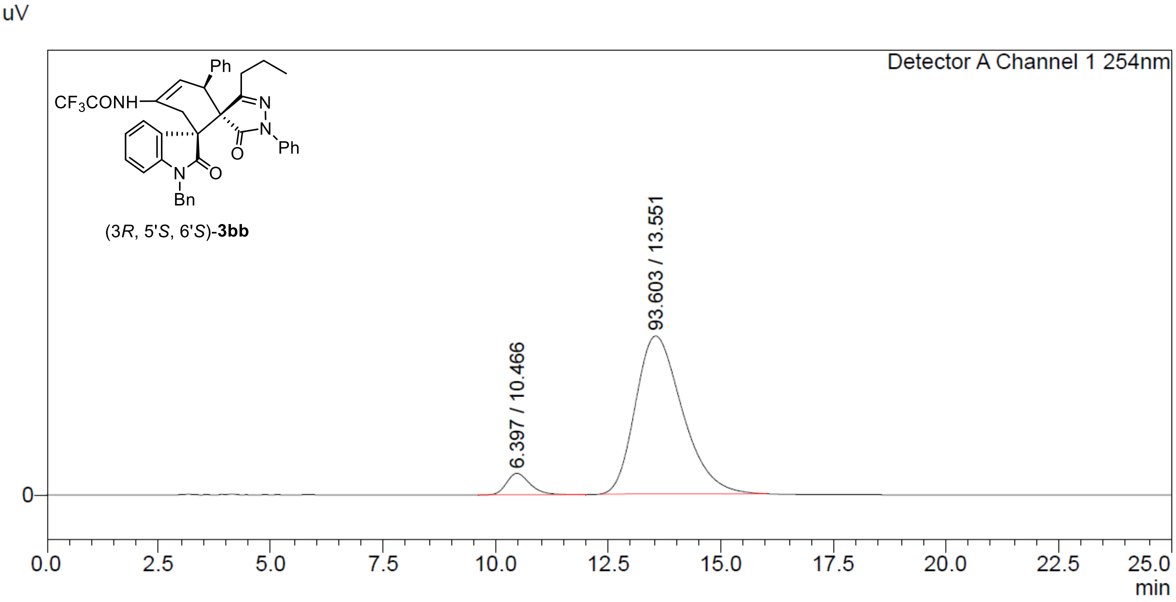

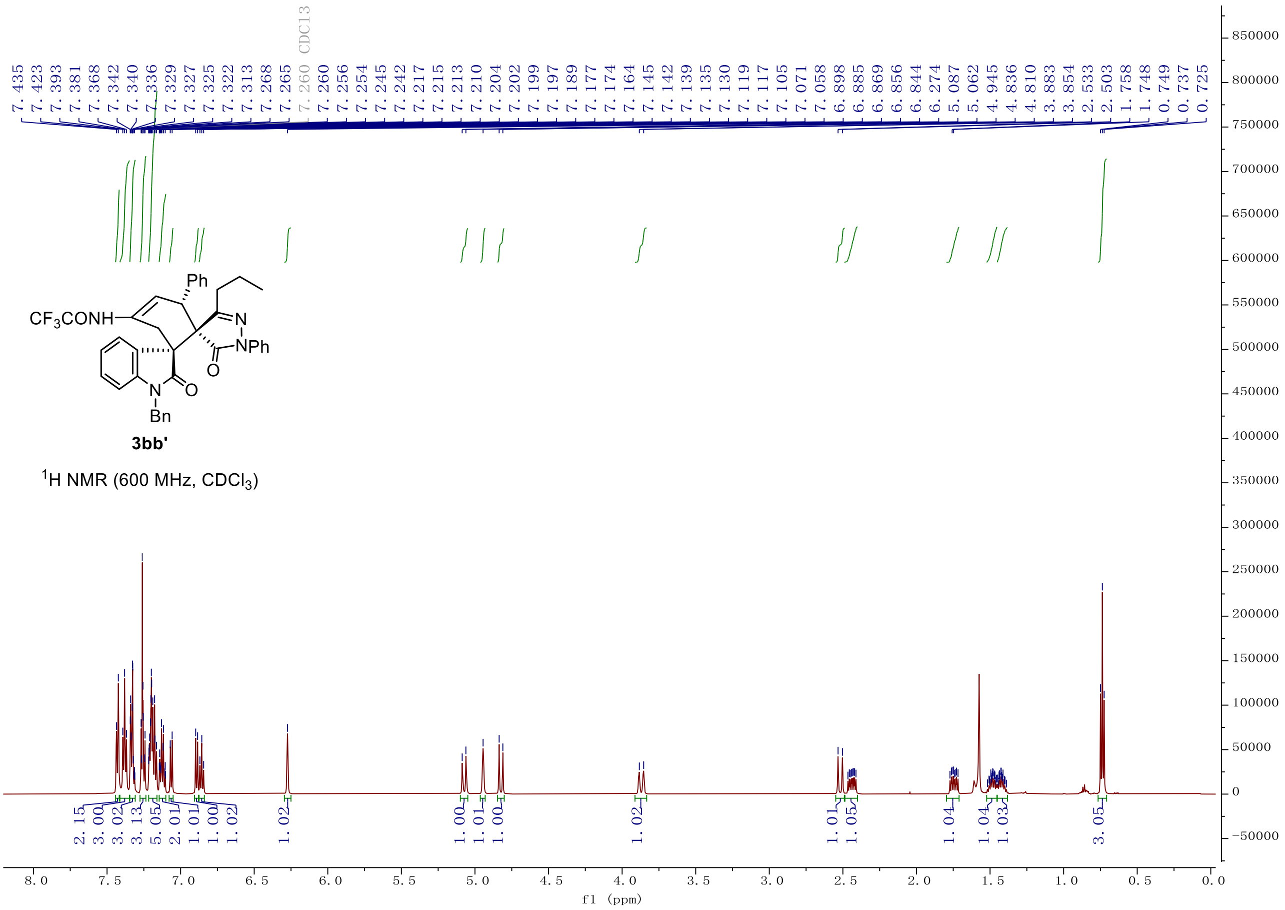

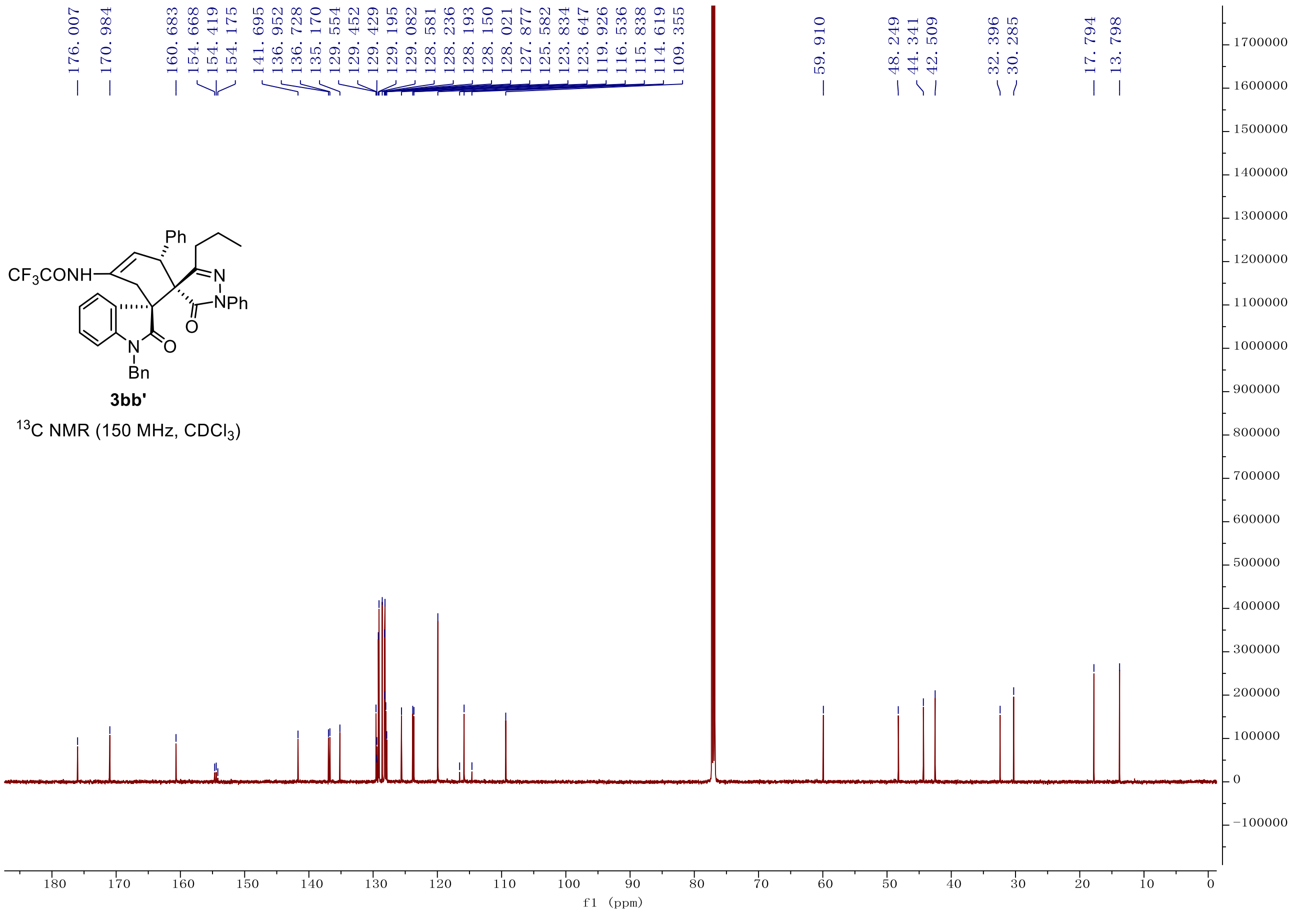
176.007
170.984
160.683
154.668
154.419
154.175
141.695
136.952
136.728
135.170
129.554
129.452
129.429
129.195
129.082
128.581
128.236
128.193
128.150
128.021
127.877
125.582
123.834
123.647
119.926
116.536
115.838
114.619
109.355
59.910
48.249
44.341
42.509
32.396
30.285
17.794
13.798
CF3CONH
Ph
N
NPh
O
N
O
Bn
3bb'
13C NMR (150 MHz, CDCl3)
f1 (ppm)

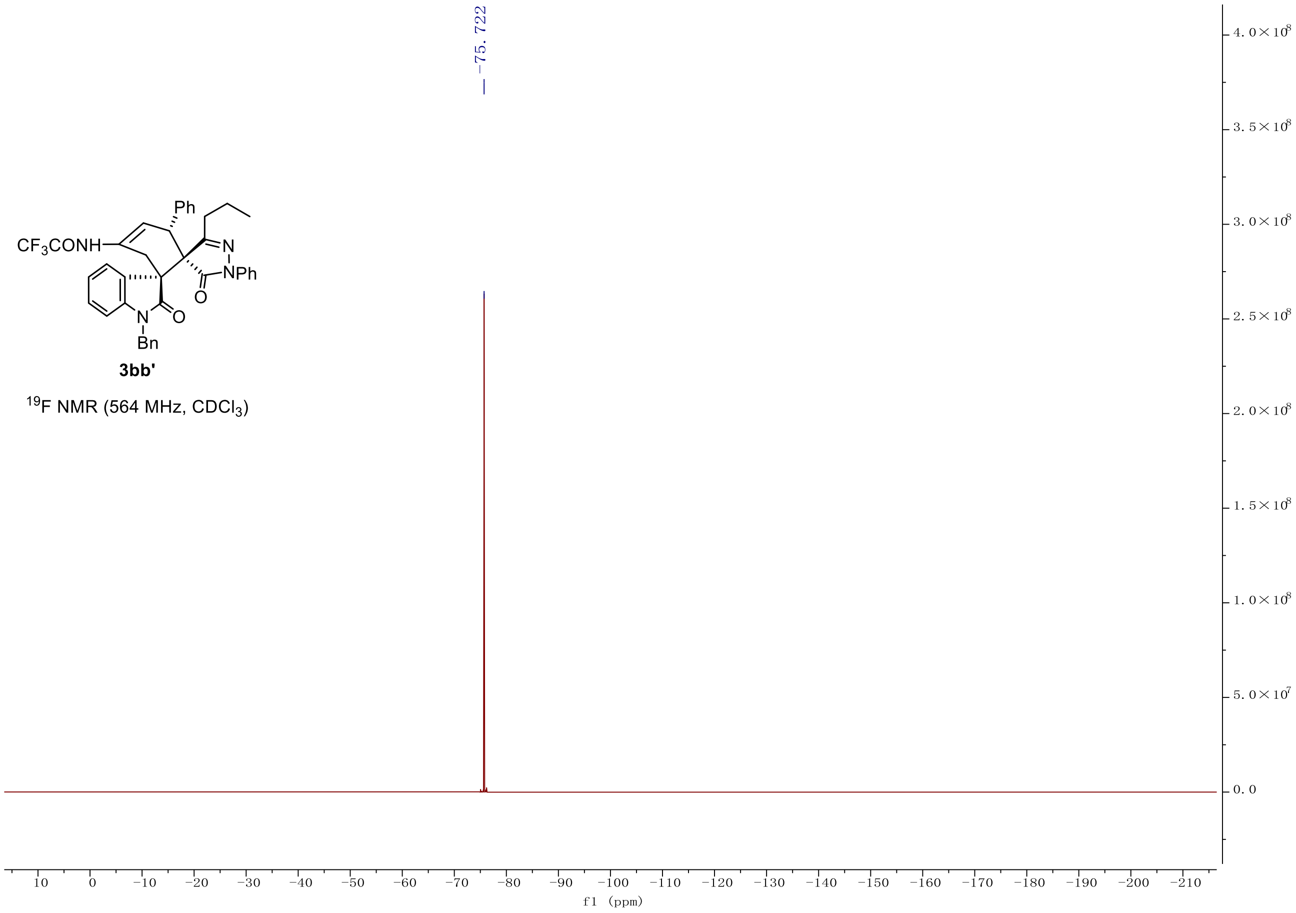
-75.722
CF3CONH
Ph
N
NPh
O
N
O
Bn
3bb'
19F NMR (564 MHz, CDCl3)
f1 (ppm)

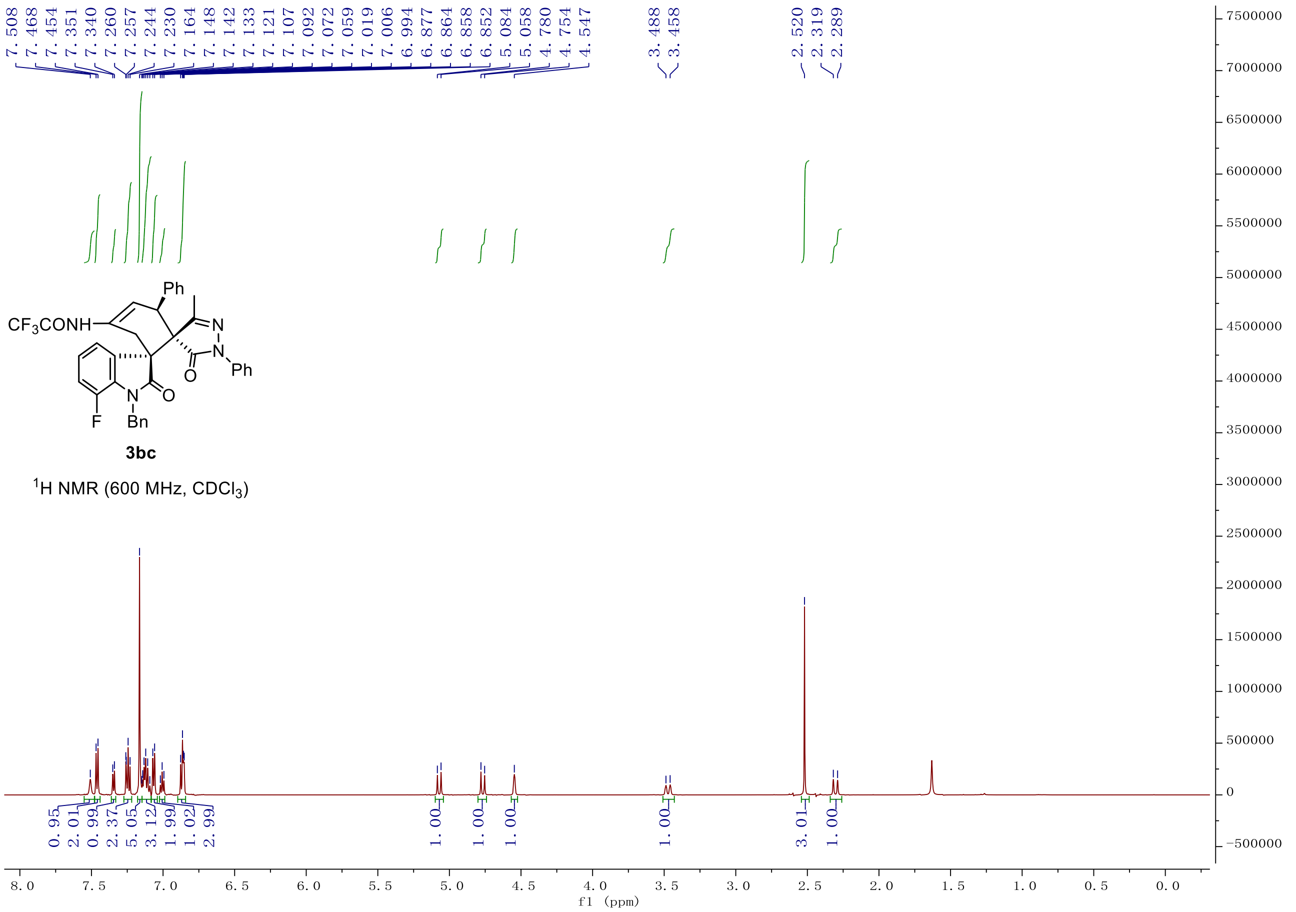
7.508 7.468 7.454 7.351 7.340 7.260 7.257 7.244 7.230 7.164 7.148 7.142 7.133 7.121 7.107 7.092 7.072 7.059 7.019 7.006 6.994 6.877 6.864 6.858 6.852 5.084 5.058 4.780 4.754 4.547
3.488 3.458
2.520 2.319 2.289
CF3CONH
Ph
N
N
Ph
O
N
O
F
Bn
3bc
1H NMR (600 MHz, CDCl3)
0.95 2.01 0.99 2.37 5.05 3.12 1.99 1.02 2.99
1.00 1.00 1.00
1.00
3.01 1.00
f1 (ppm)

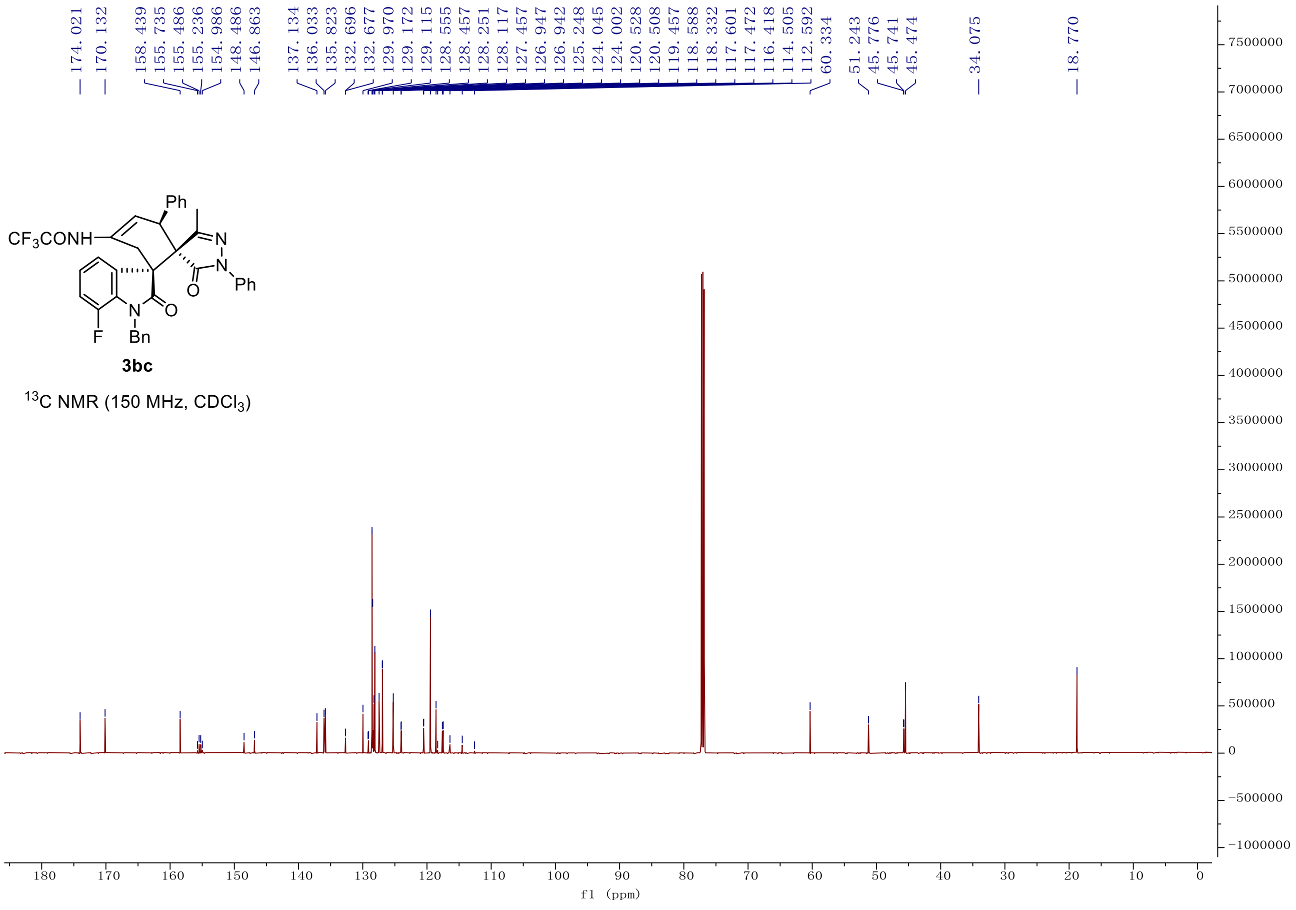
174.021
170.132
158.439 155.735 155.486 155.236 154.986 148.486 146.863
137.134 136.033 135.823 132.696 132.677 129.970 129.172 129.115 128.555 128.457 128.251 128.117 127.457 126.947 126.942 125.248 124.045 124.002 120.528 120.508 119.457 118.588 118.332 117.601 117.472 116.418 114.505 112.592
60.334
51.243 45.776 45.741 45.474
34.075
18.770
CF3CONH
Ph
N
N
Ph
O
N
O
F
Bn
3bc
13C NMR (150 MHz, CDCl3)
f1 (ppm)

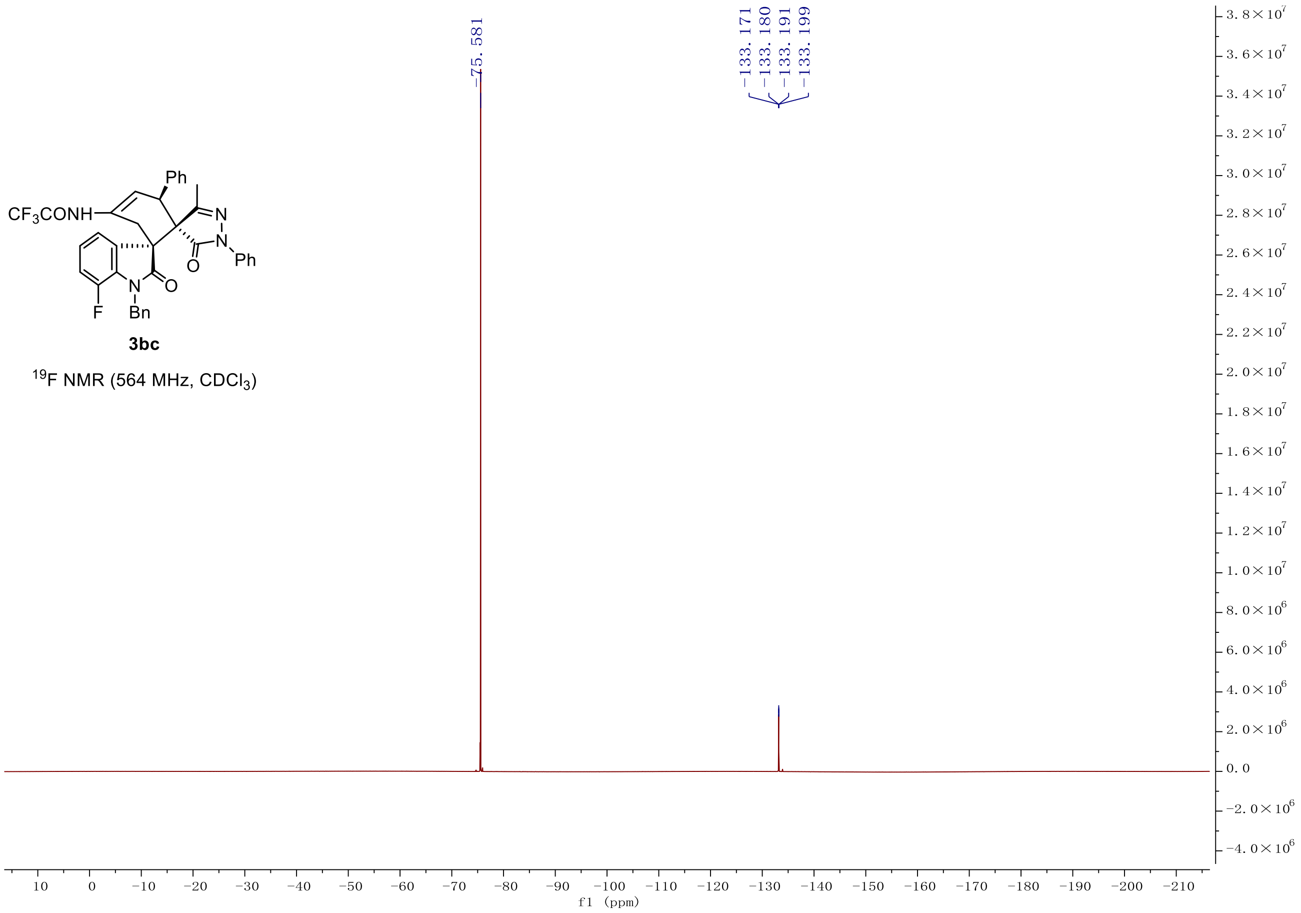

Peak Analysis Report

Detector A Channel 1 254nm

No.	Ret. Time	Height (mAu)	Area (mAu*min)	Rel. Area (%)
1	7.240	977354	21371150	49.978
2	11.605	601354	21389966	50.022
Total		1578708	42761116	100.000

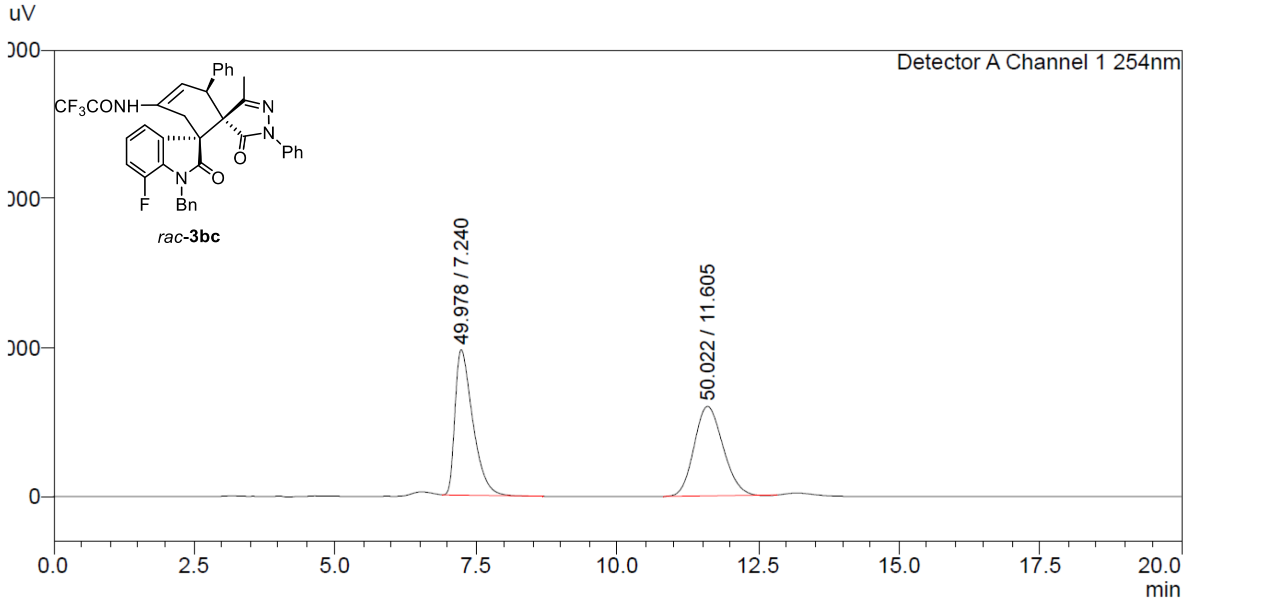

Peak Analysis Report

Detector A Channel 1 254nm

No.	Ret. Time	Height (mAu)	Area (mAu*min)	Rel. Area (%)
1	7.452	7942	173157	0.356
2	11.608	1347483	48513136	99.644
Total		1355425	48686293	100.000

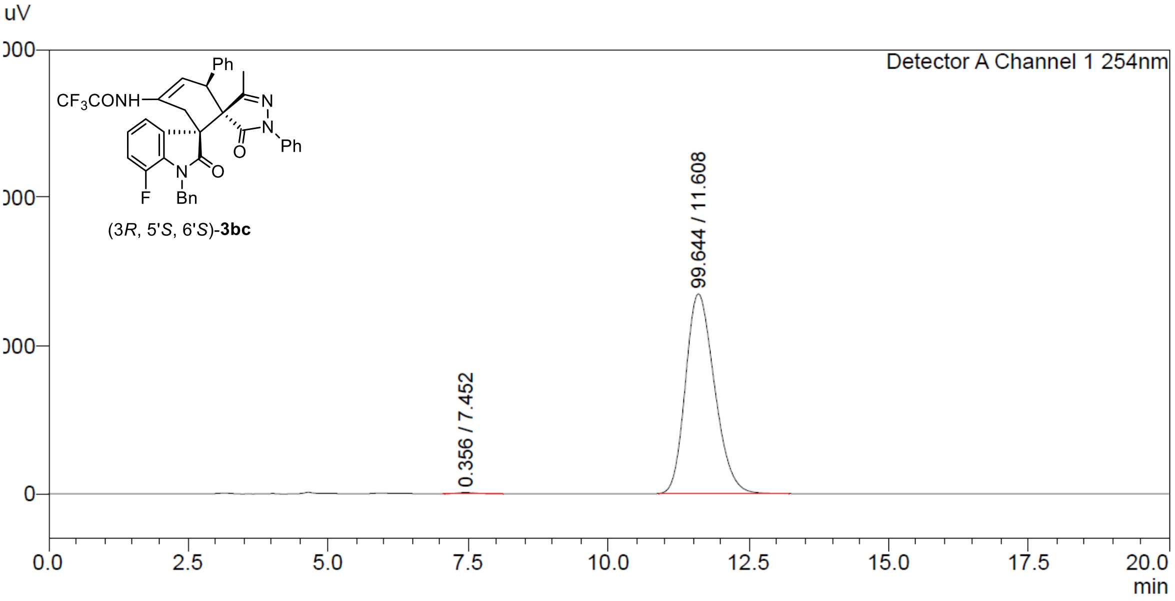

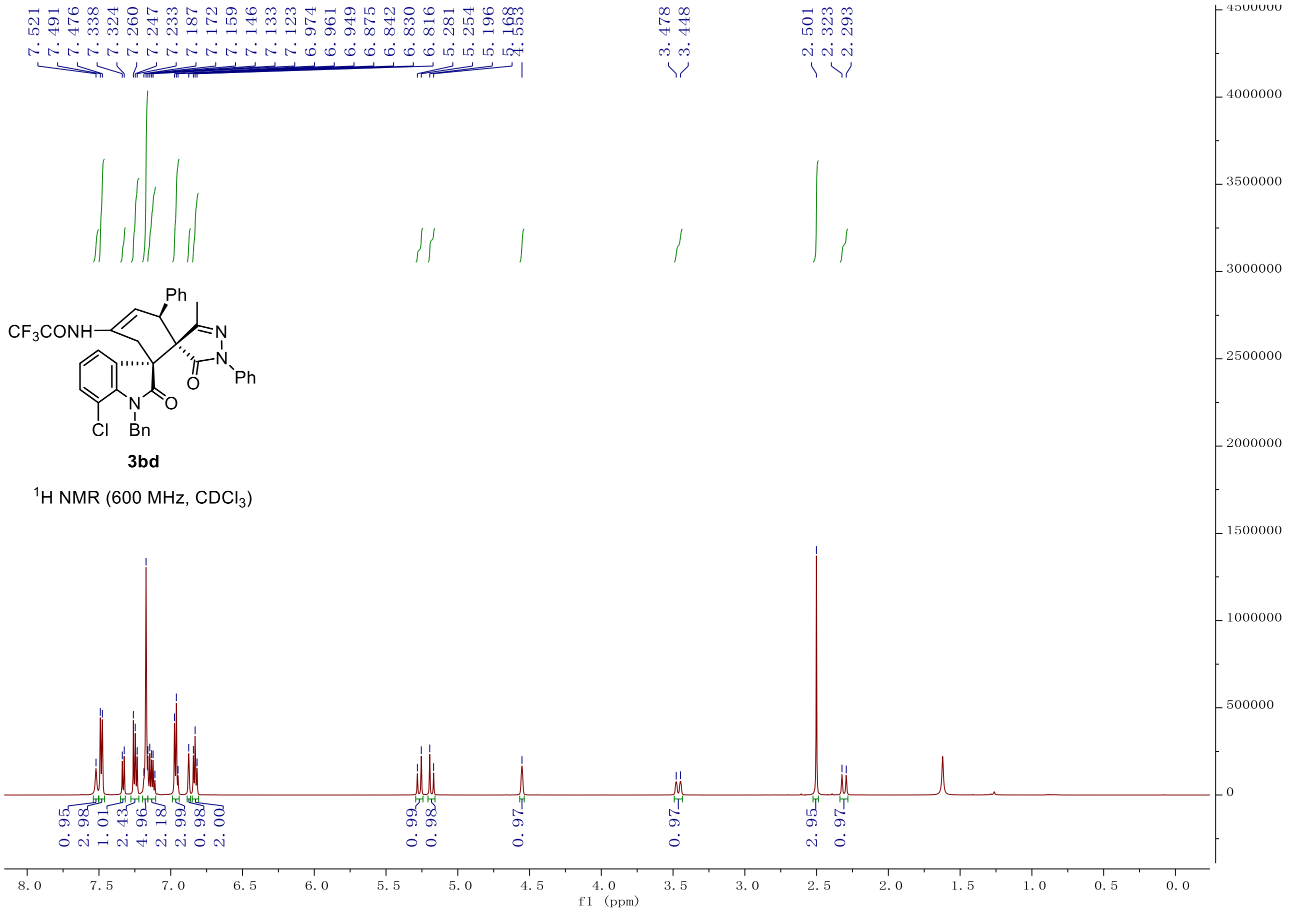

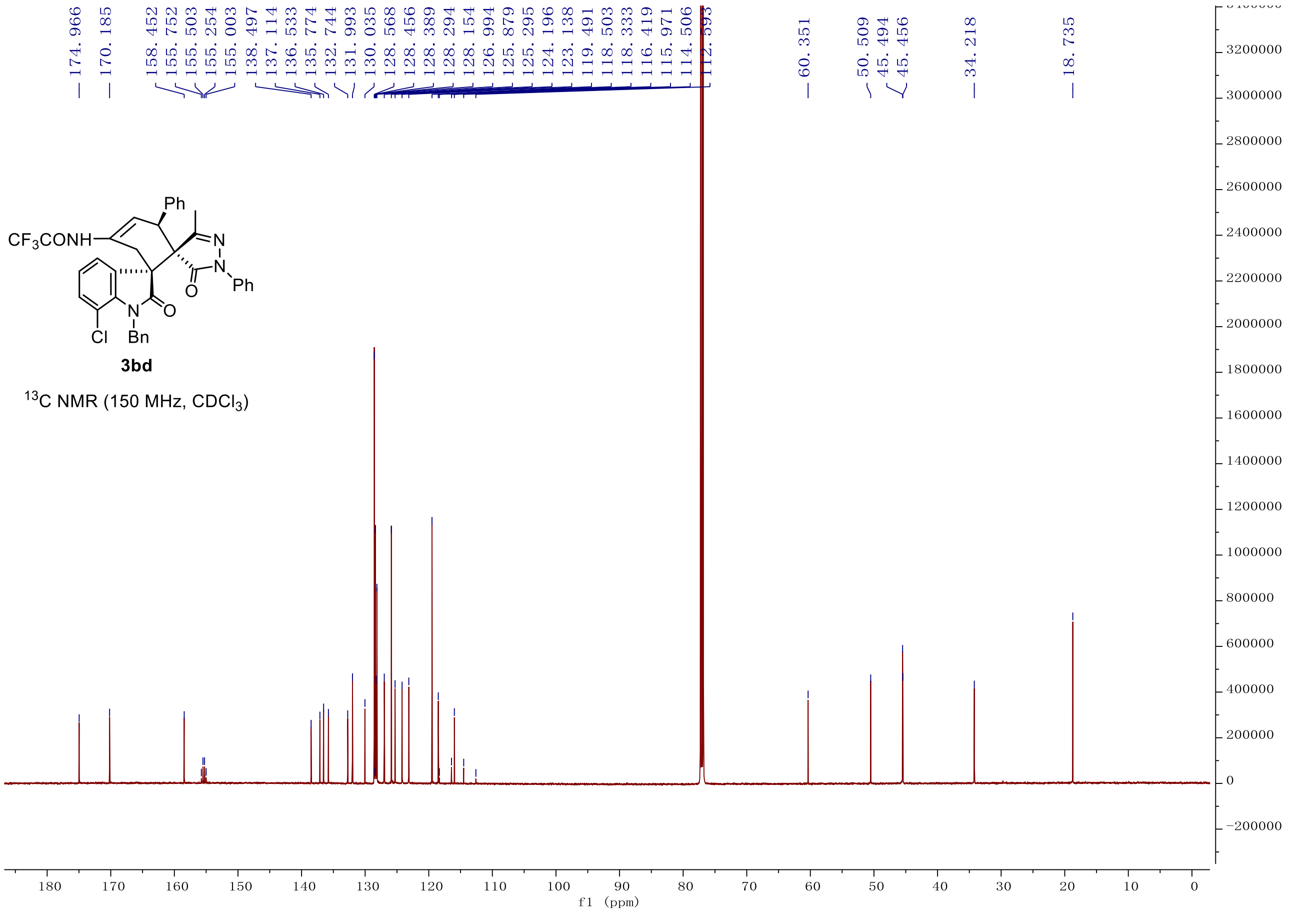
174.966
170.185
158.452
155.752
155.503
155.254
155.003
138.497
137.114
136.533
135.774
132.744
131.993
130.035
128.568
128.456
128.389
128.294
128.154
126.994
125.879
125.295
124.196
123.138
119.491
118.503
118.333
116.419
115.971
114.506
112.593
60.351
50.509
45.494
45.456
34.218
18.735
CF3CONH
Ph
N
N
Ph
O
N
O
Cl
Bn
3bd
13C NMR (150 MHz, CDCl3)
f1 (ppm)

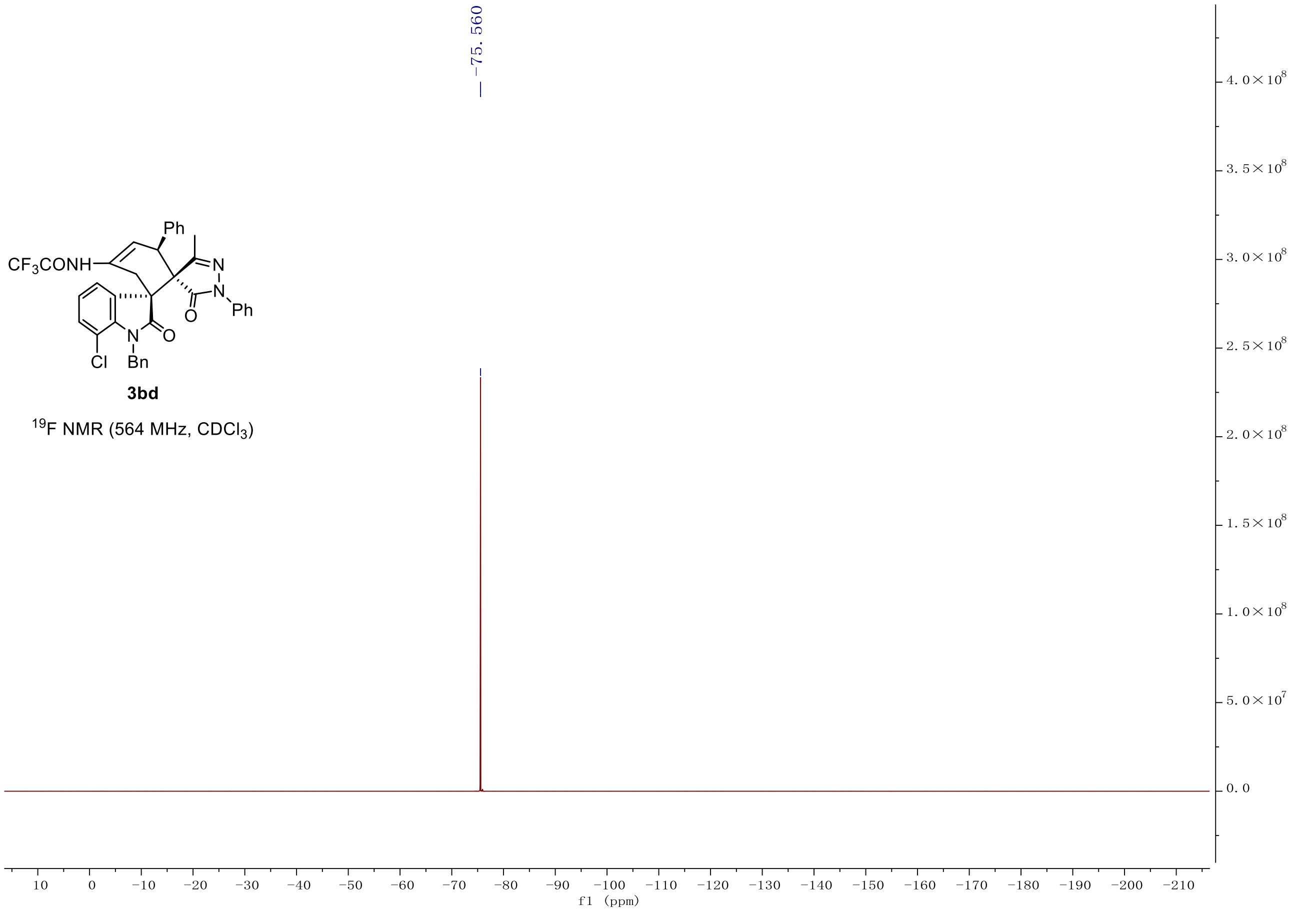
-75.560
CF3CONH
Ph
N
N
Ph
O
N
O
Cl
Bn
3bd
19F NMR (564 MHz, CDCl3)
f1 (ppm)

Signal: VWD1 B, Wavelength=254 nm

RT [min]	Type	Width [min]	Area	Height	Area%	Name
9.037	MM	0.7115	10338.0801	242.1520	49.7817	
11.611	MM	0.7568	10428.7529	229.6696	50.2183	
		Sum	20766.8330			

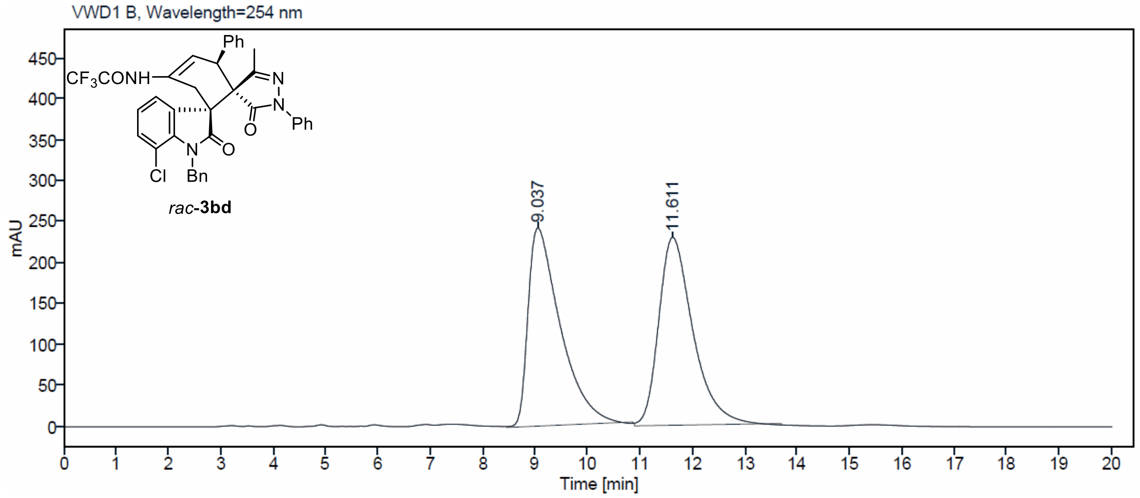

Signal: VWD1 B, Wavelength=254 nm

RT [min]	Type	Width [min]	Area	Height	Area%	Name
9.096	MM	0.0949	2.2755	0.3997	0.0473	
11.678	BB	0.7265	4812.8164	99.4654	99.9527	
		Sum	4815.0919			

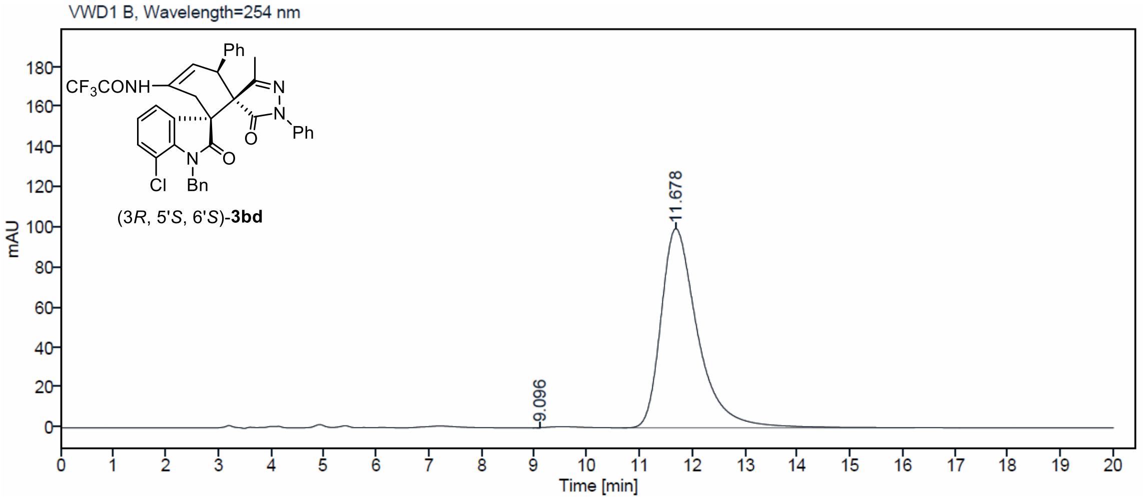

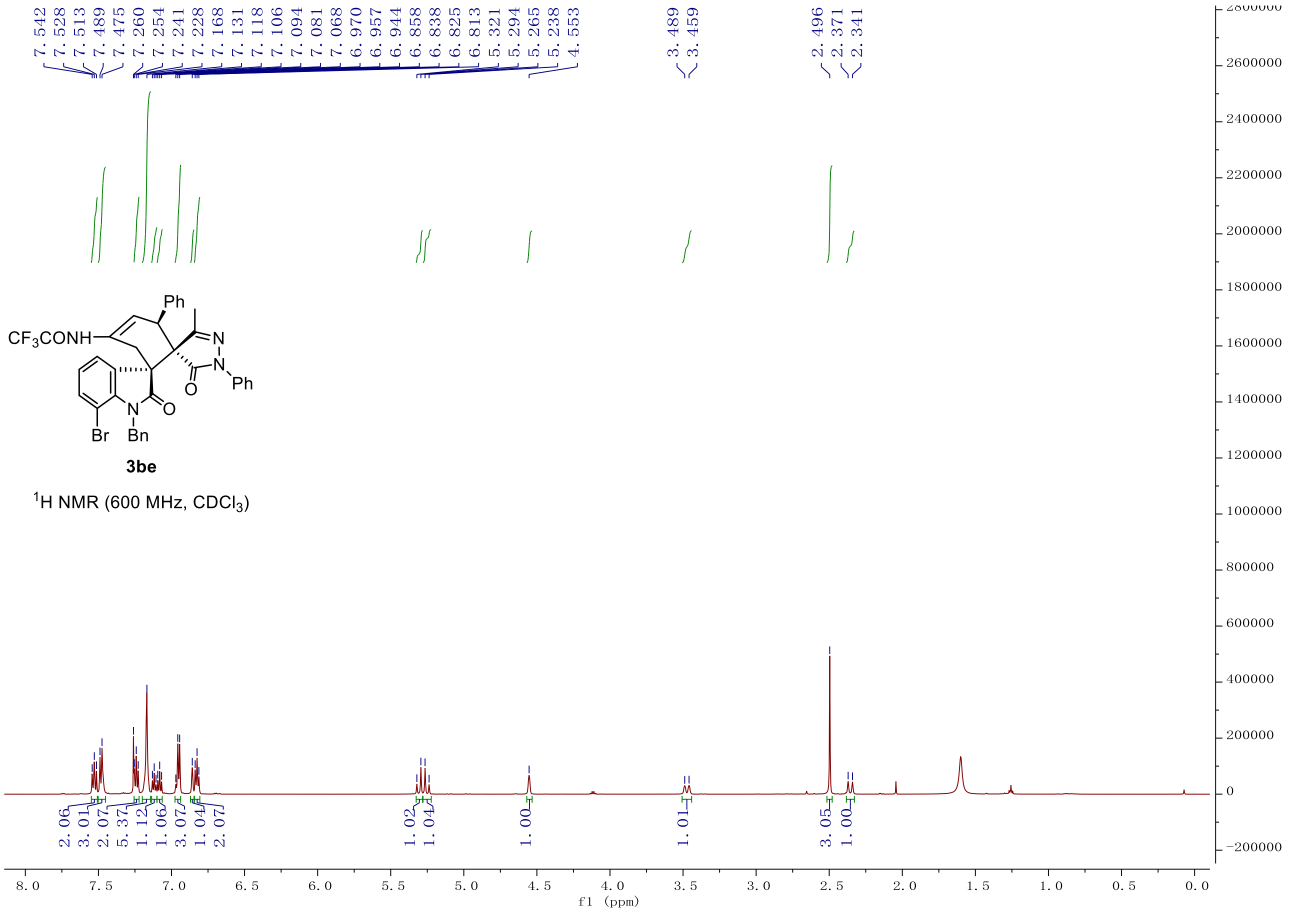
7.542 7.528 7.513 7.489 7.475 7.260 7.254 7.241 7.228 7.168 7.131 7.118 7.106 7.094 7.081 7.068 6.970 6.957 6.944 6.858 6.838 6.825 6.813 5.321 5.294 5.265 5.238 4.553
3.489 3.459
2.496 2.371 2.341
2.06 3.01 2.07 5.37 1.12 1.06 3.07 1.04 2.07
1.02 1.04
1.00
1.01
3.05 1.00
CF3CONH
Ph
N
N
Ph
O
N
O
Br
Bn
3be
1H NMR (600 MHz, CDCl3)
f1 (ppm)

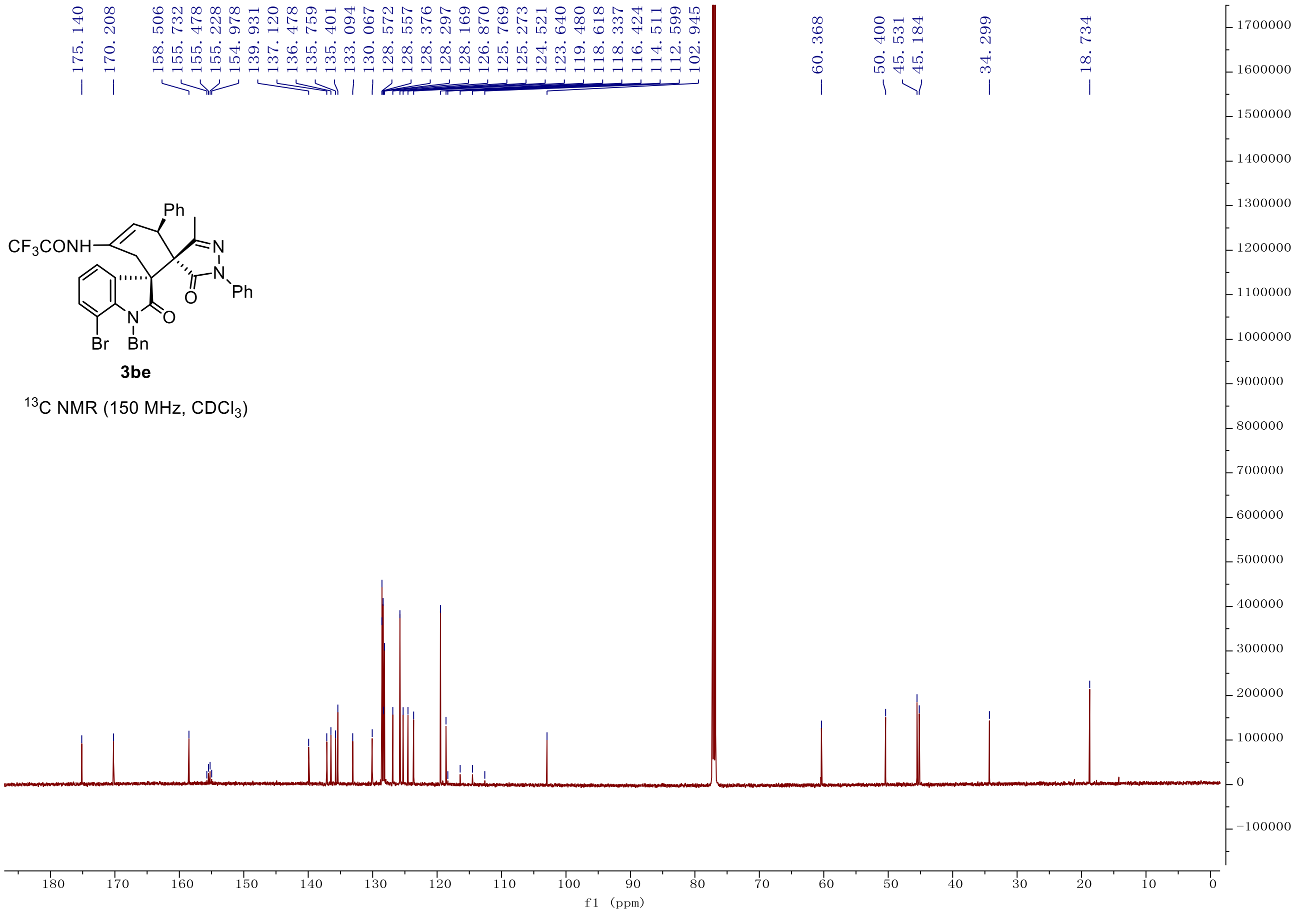
175.140
170.208
158.506 155.732 155.478 155.228 154.978 139.931 137.120 136.478 135.759 135.401 133.094 130.067 128.572 128.557 128.376 128.297 128.169 126.870 125.769 125.273 124.521 123.640 119.480 118.618 118.337 116.424 114.511 112.599 102.945
60.368
50.400 45.531 45.184
34.299
18.734
CF3CONH
Ph
N
N
Ph
O
N
O
Br
Bn
3be
13C NMR (150 MHz, CDCl3)
f1 (ppm)

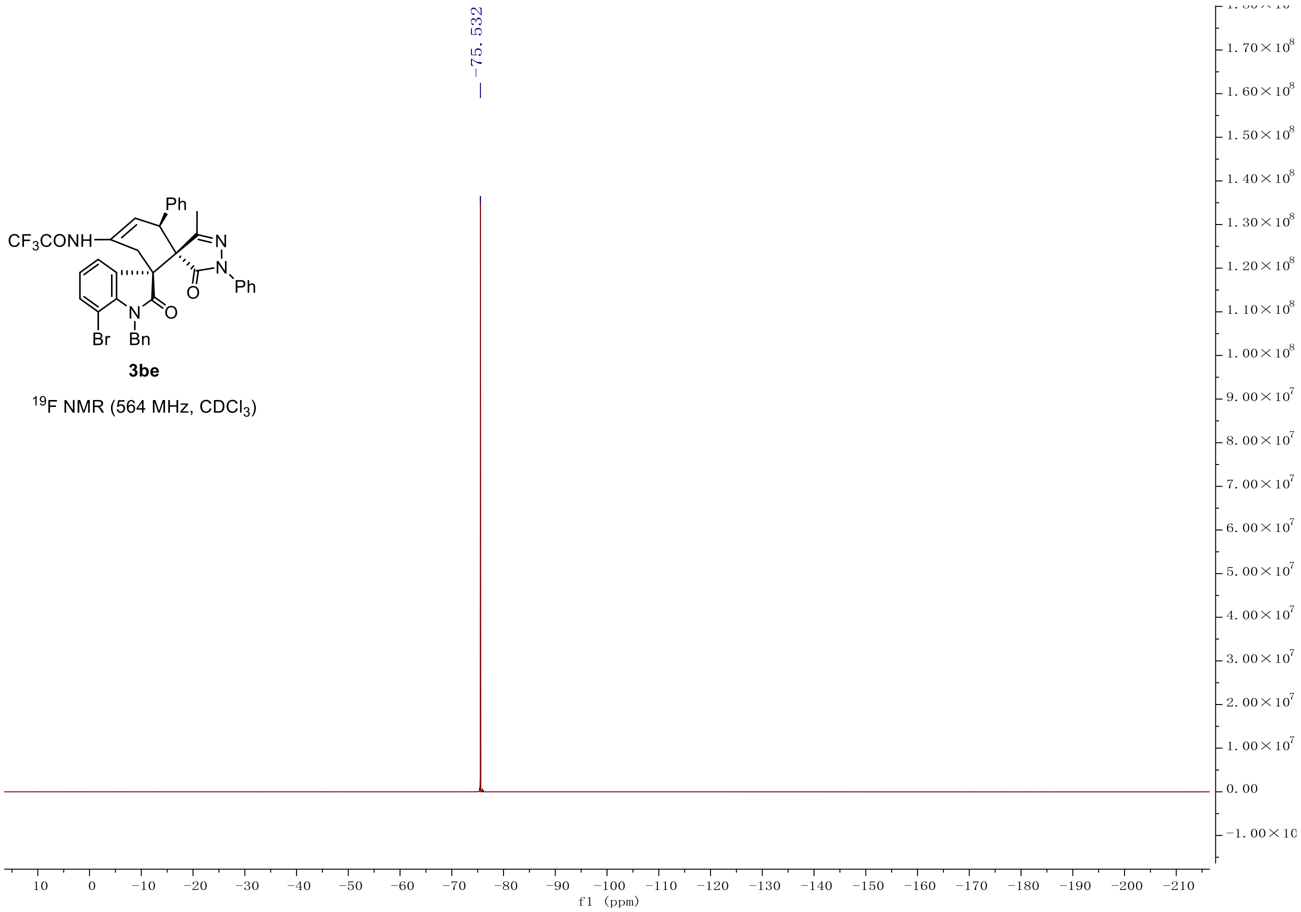

Peak Analysis Report

Detector A Channel 1 254nm

No.	Ret. Time	Height (mAu)	Area (mAu*min)	Rel. Area (%)
1	7.187	697631	16375667	50.654
2	10.319	465245	15952937	49.346
Total		1162876	32328603	100.000

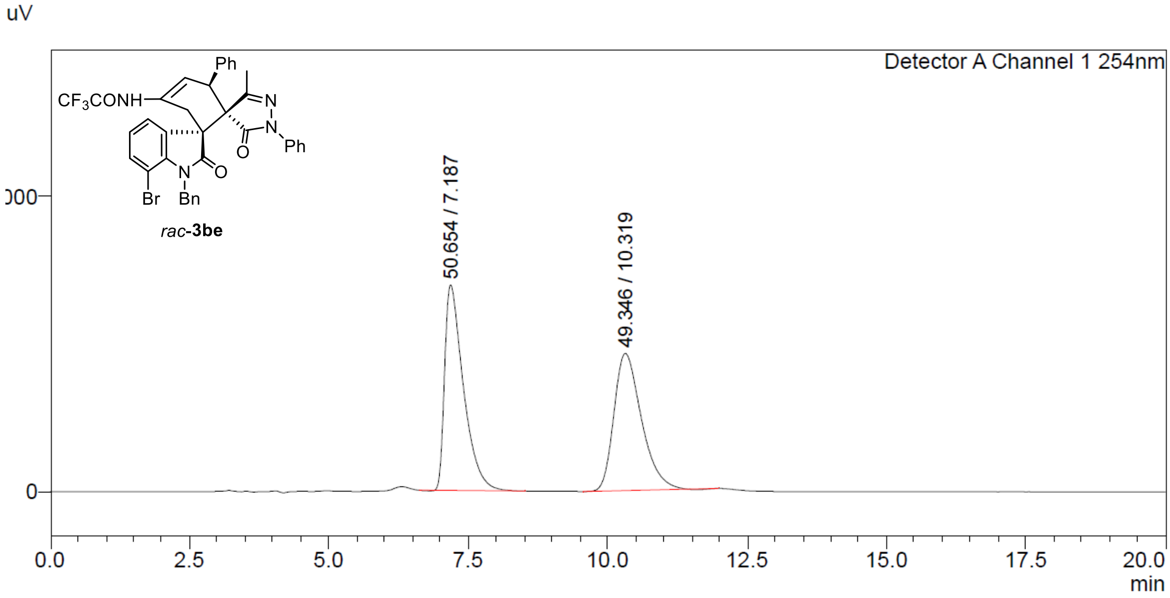

Peak Analysis Report

Detector A Channel 1 254nm

No.	Ret. Time	Height (mAu)	Area (mAu*min)	Rel. Area (%)
1	7.411	29772	606840	1.216
2	10.169	1468370	49315328	98.784
Total		1498142	49922168	100.000

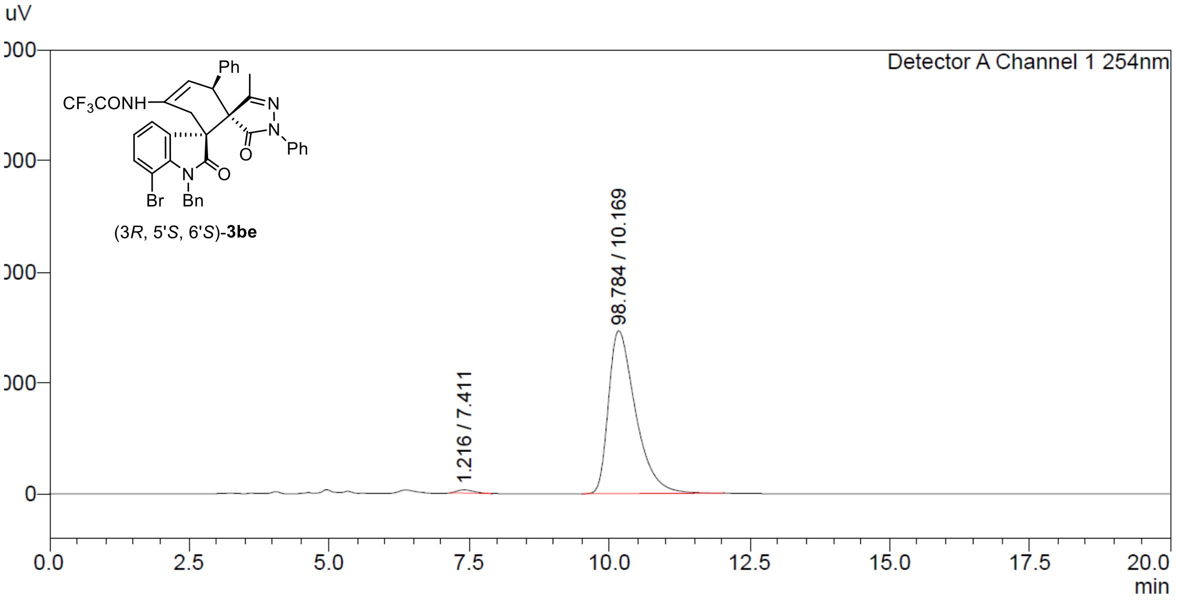

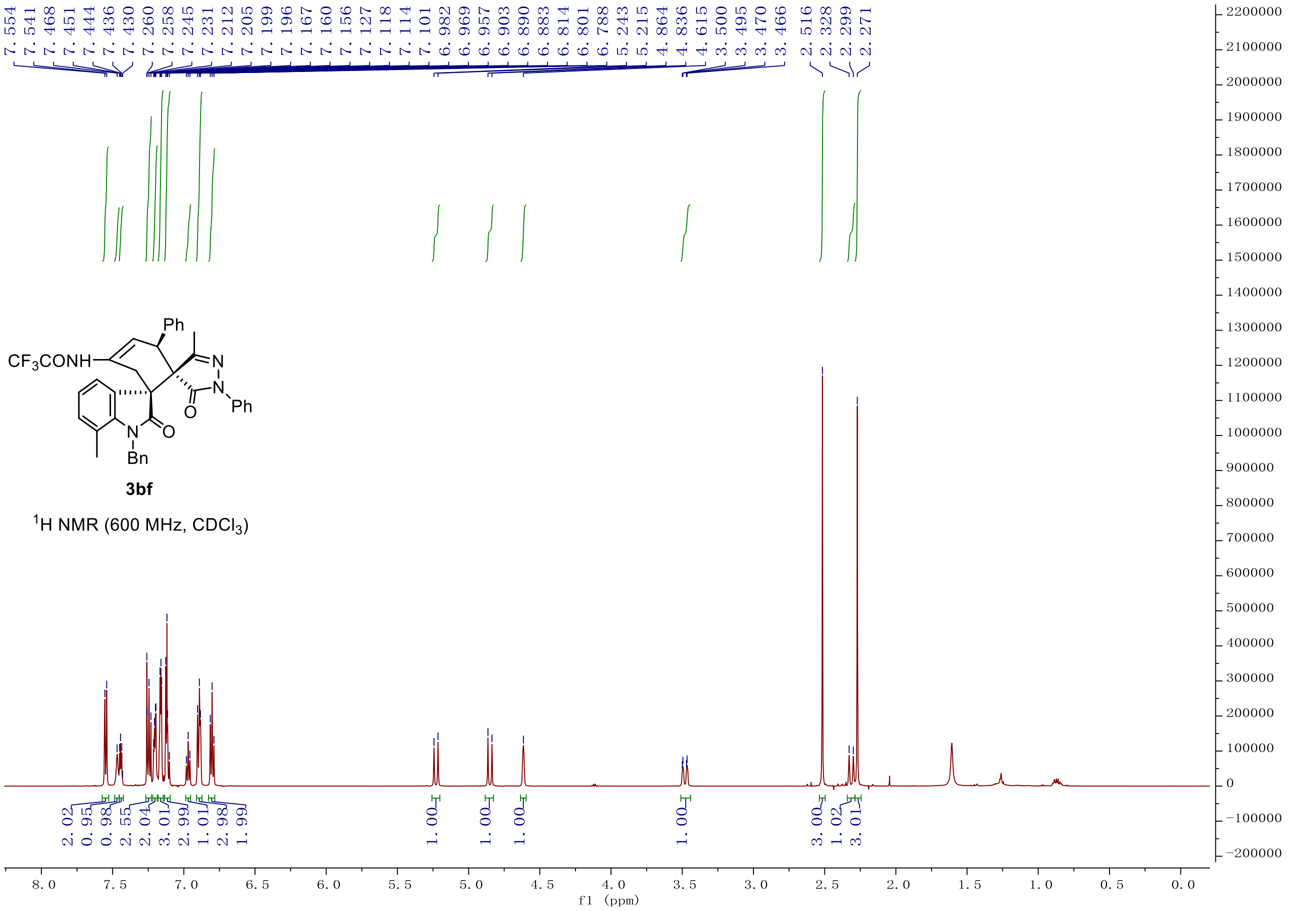

3bf

^{1}H NMR (600 MHz, $CDCl_3$)

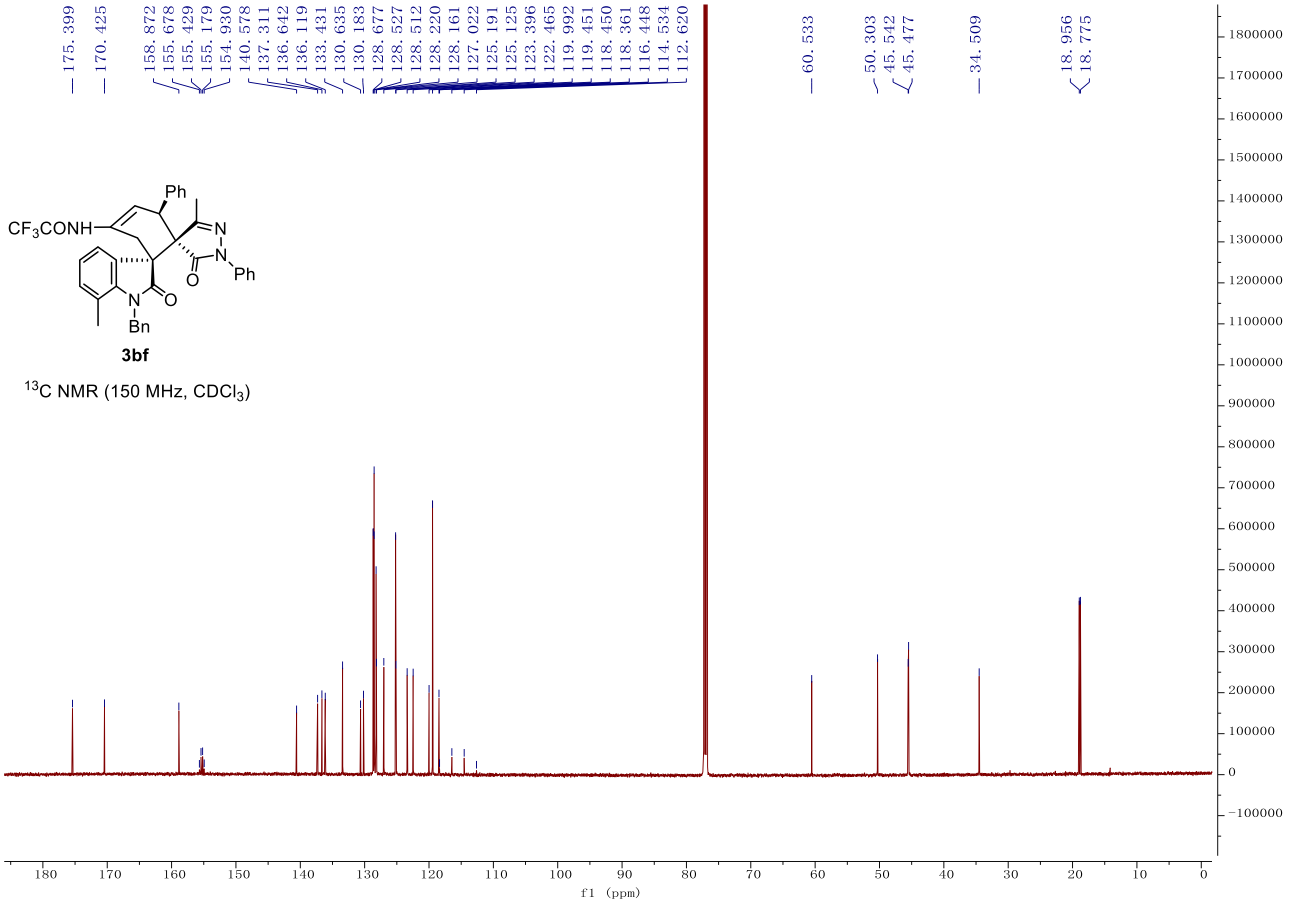

^{13}C NMR (150 MHz, $CDCl_3$)

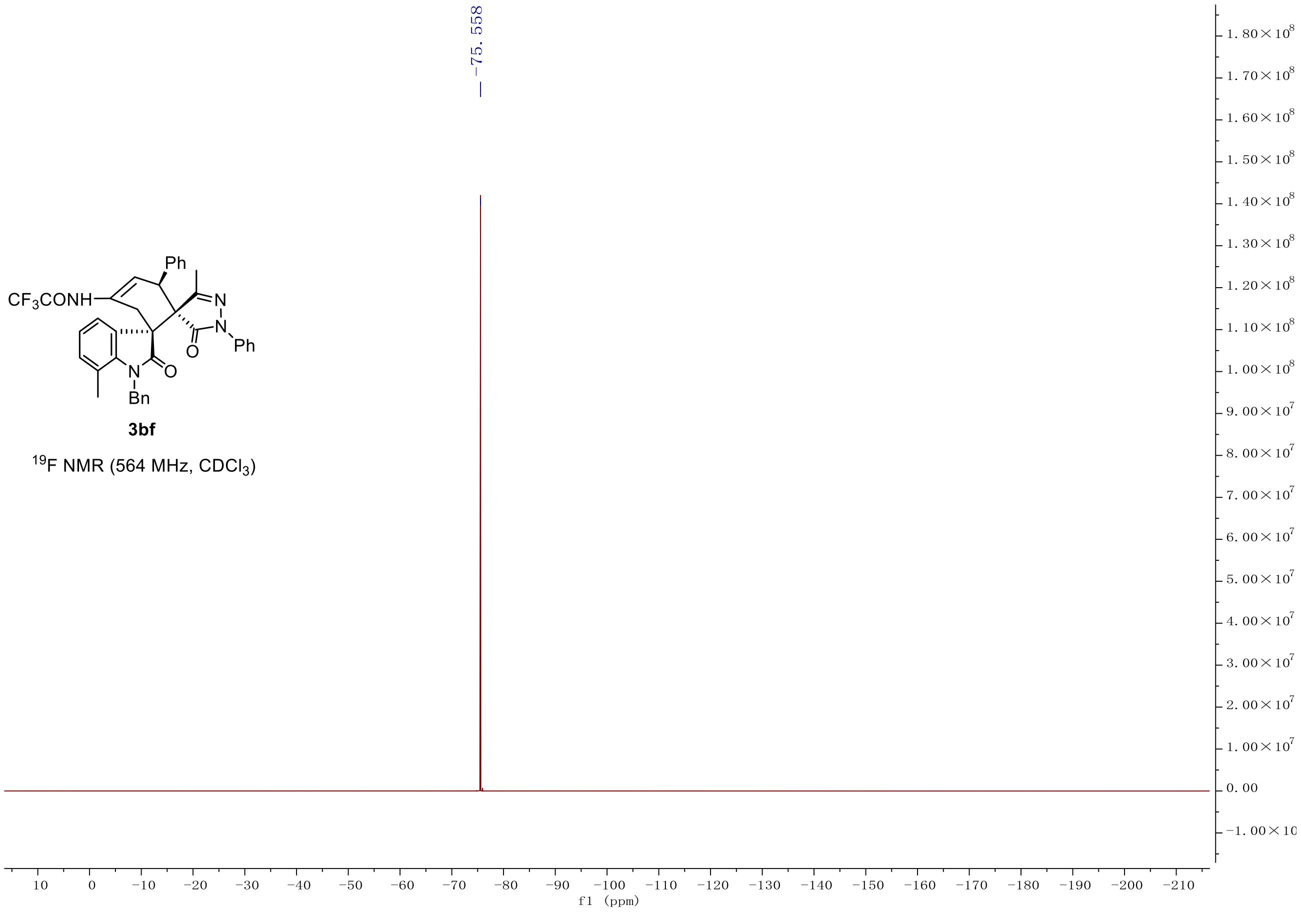

^{19}F NMR (564 MHz, $CDCl_3$)

Peak Analysis Report

Detector A Channel 1 254nm

No.	Ret. Time	Height (mAu)	Area (mAu*min)	Rel. Area (%)
1	8.552	195714	8622316	50.109
2	12.463	154311	8584808	49.891
Total		350025	17207124	100.000

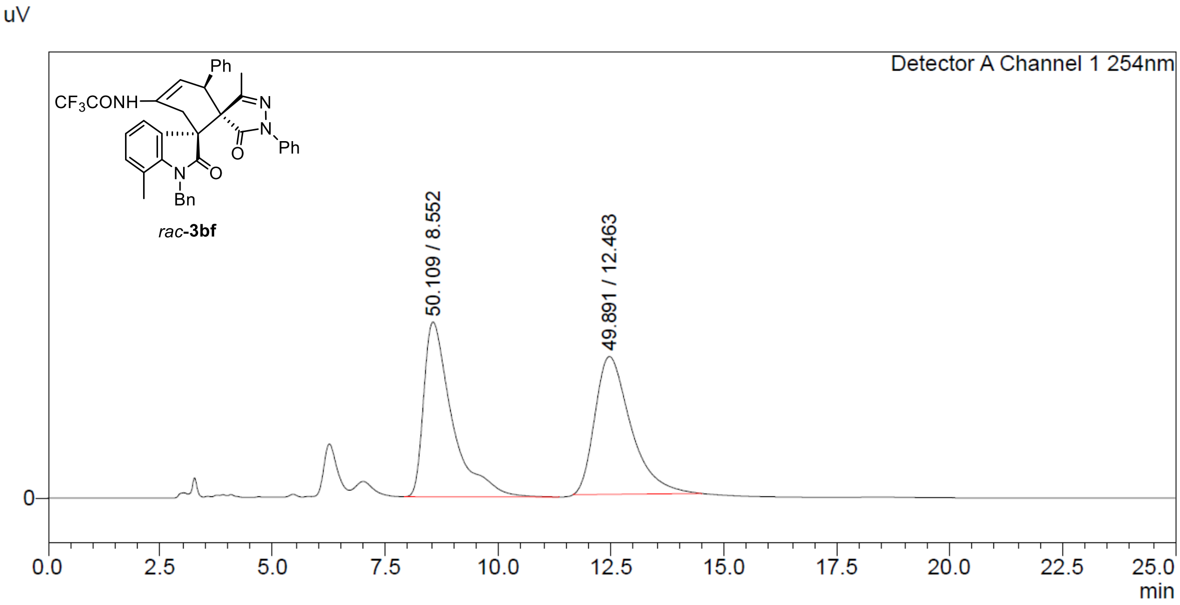

Peak Analysis Report

Detector A Channel 1 254nm

No.	Ret. Time	Height (mAu)	Area (mAu*min)	Rel. Area (%)
1	8.747	7774	319962	0.743
2	12.285	825341	42729694	99.257
Total		833115	43049656	100.000

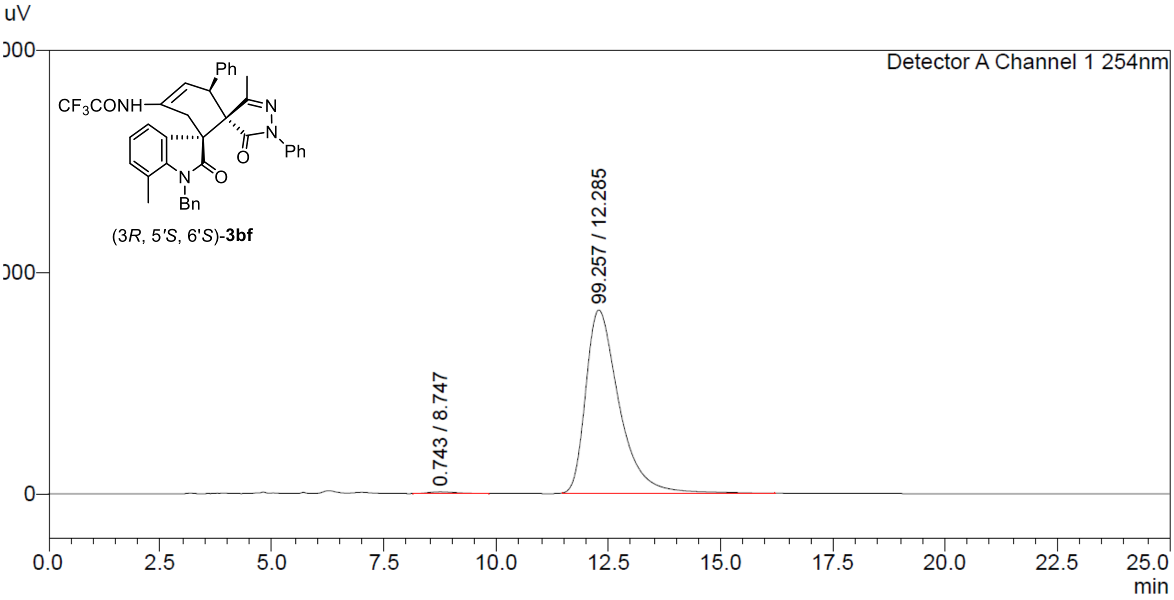

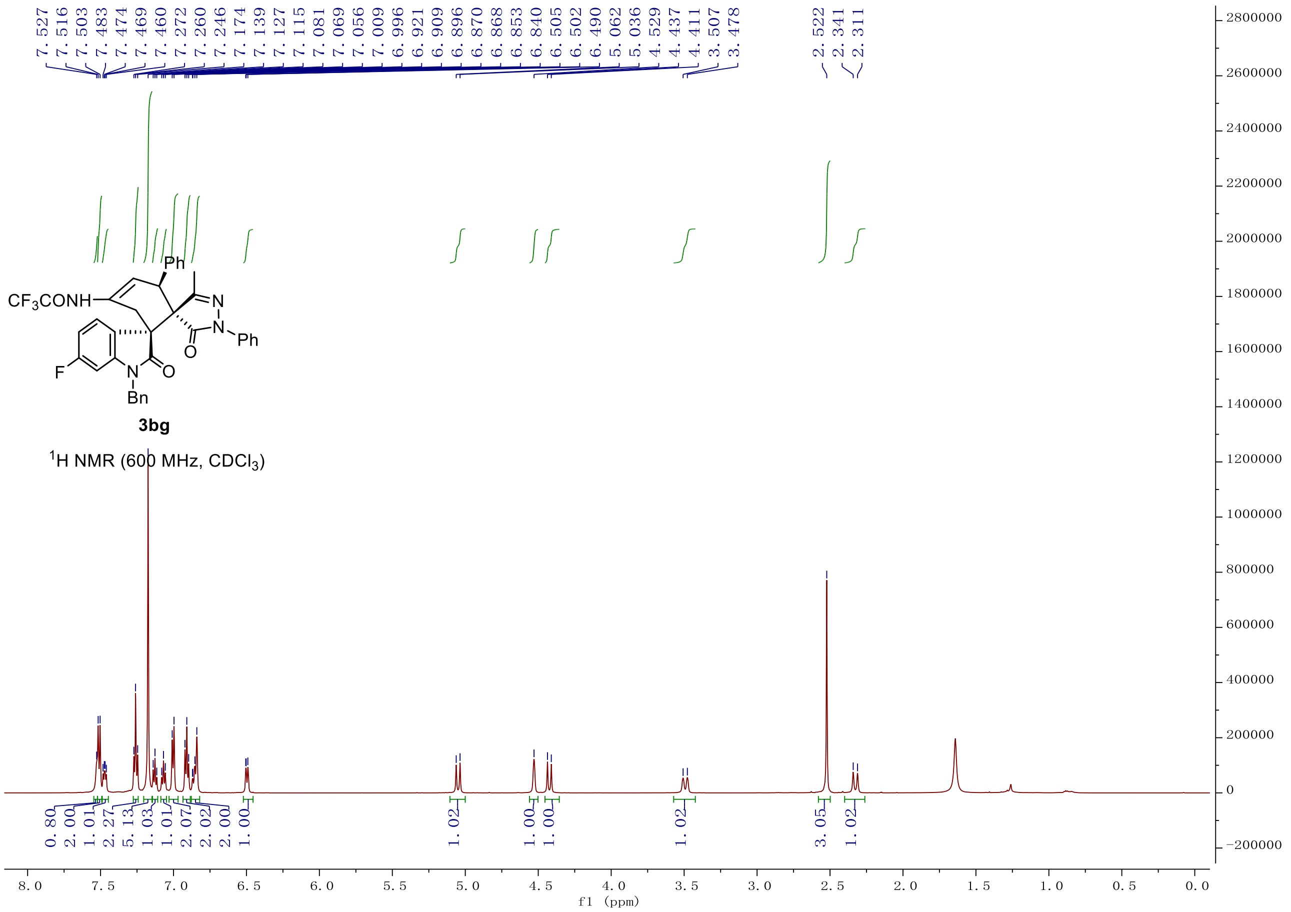

7.527 7.516 7.503 7.483 7.474 7.469 7.460 7.272 7.260 7.246 7.174 7.139 7.127 7.115 7.081 7.069 7.056 7.009 6.996 6.921 6.909 6.896 6.870 6.868 6.853 6.840 6.505 6.502 6.490 5.062 5.036 4.529 4.437 4.411 3.507 3.478
2.522 2.341 2.311
CF3CONH
Ph
N
N
Ph
O
F
N
O
Bn
3bg
1H NMR (600 MHz, CDCl3)
0.80 2.00 1.01 2.27 5.13 1.03 1.01 2.07 2.02 2.00 1.00 1.02 1.00 1.00 1.02 3.05 1.02
f1 (ppm)

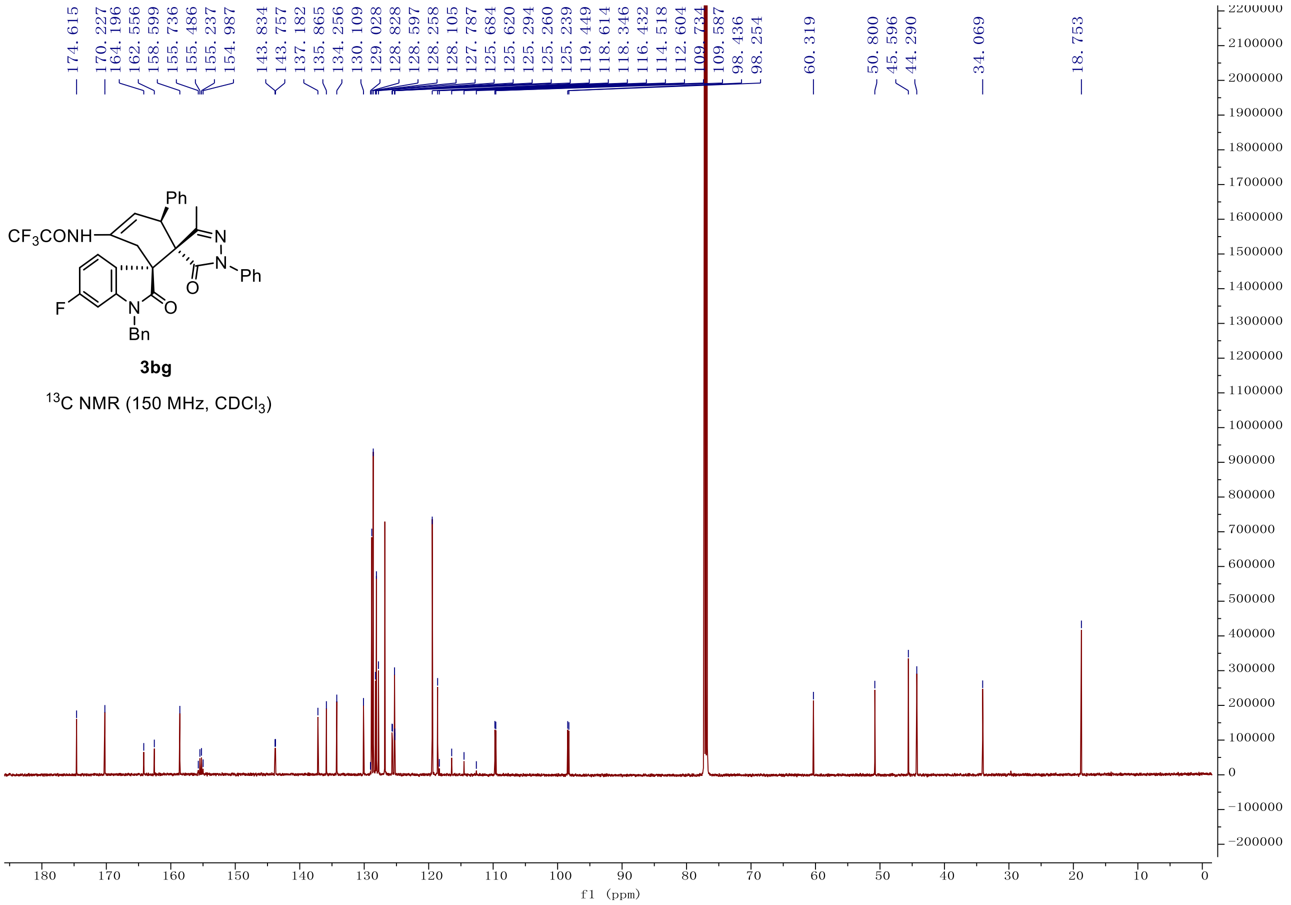

174.615 170.227 164.196 162.556 158.599 155.736 155.486 155.237 154.987 143.834 143.757 137.182 135.865 134.256 130.109 129.028 128.828 128.597 128.258 128.105 127.787 125.684 125.620 125.294 125.260 125.239 119.449 118.614 118.346 116.432 114.518 112.604 109.734 109.587 98.436 98.254
60.319 50.800 45.596 44.290 34.069 18.753
CF3CONH
Ph
N
N
Ph
O
F
N
O
Bn
3bg
13C NMR (150 MHz, CDCl3)
f1 (ppm)

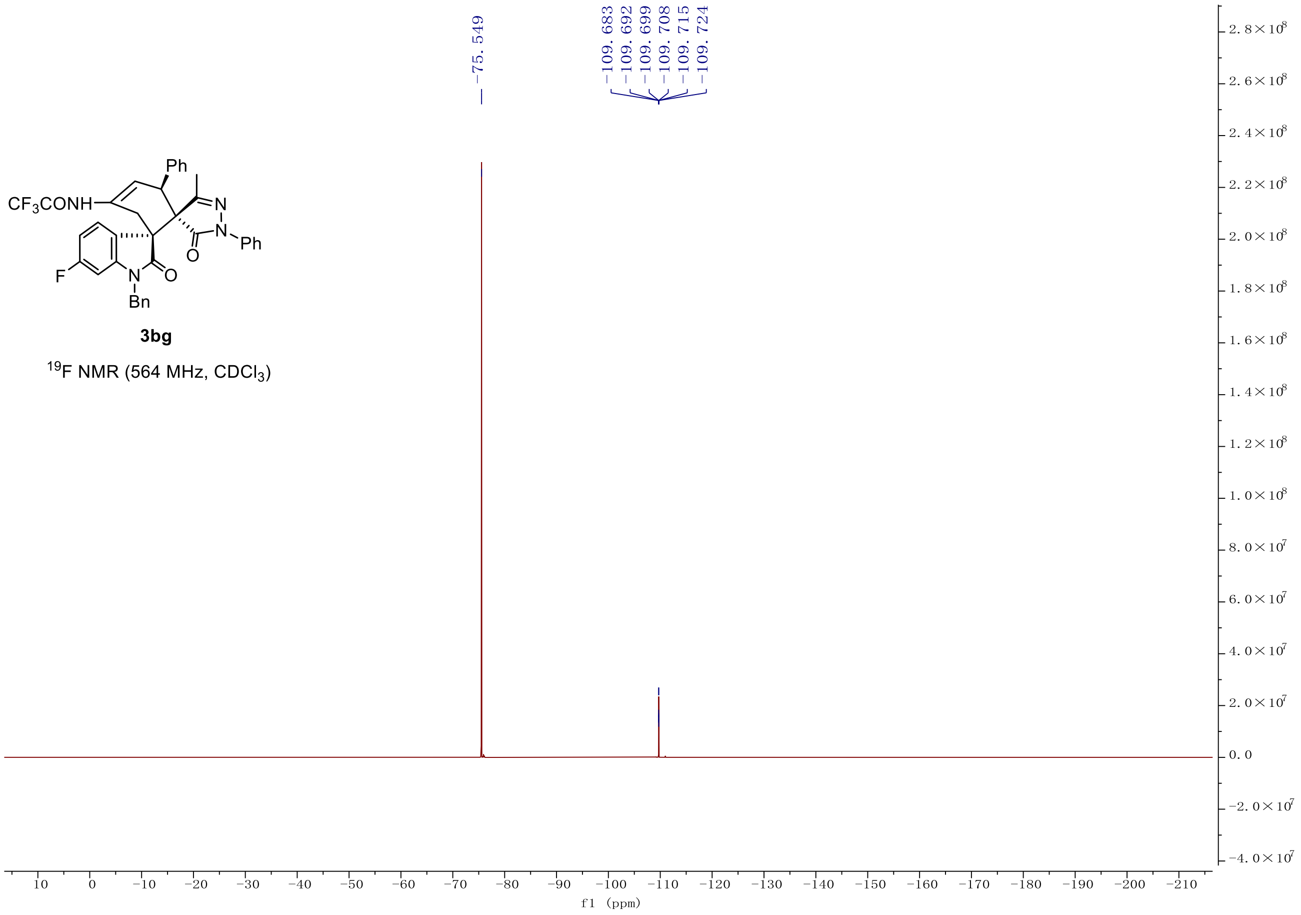

Signal: VWD1 B, Wavelength=254 nm

RT [min]	Type	Width [min]	Area	Height	Area%	Name
6.293	MM	0.3182	135.9990	7.1231	50.5204	
12.475	MM	0.9064	133.1974	2.4493	49.4796	

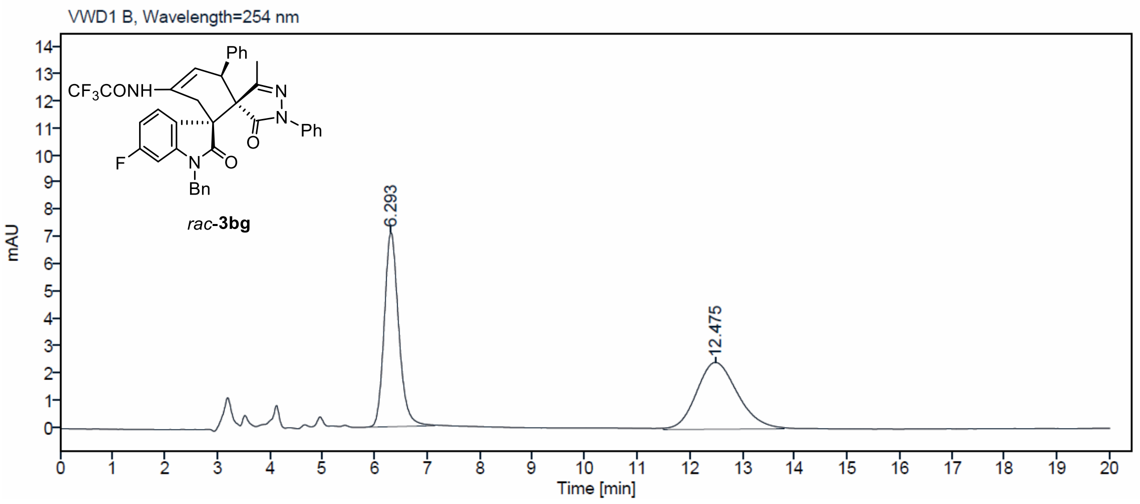

Signal: VWD1 B, Wavelength=254 nm

RT [min]	Type	Width [min]	Area	Height	Area%	Name
6.298	MM	0.4668	79.2207	2.8287	0.6059	
12.429	BB	0.8236	12996.0664	243.1525	99.3941	
		Sum	13075.2872			

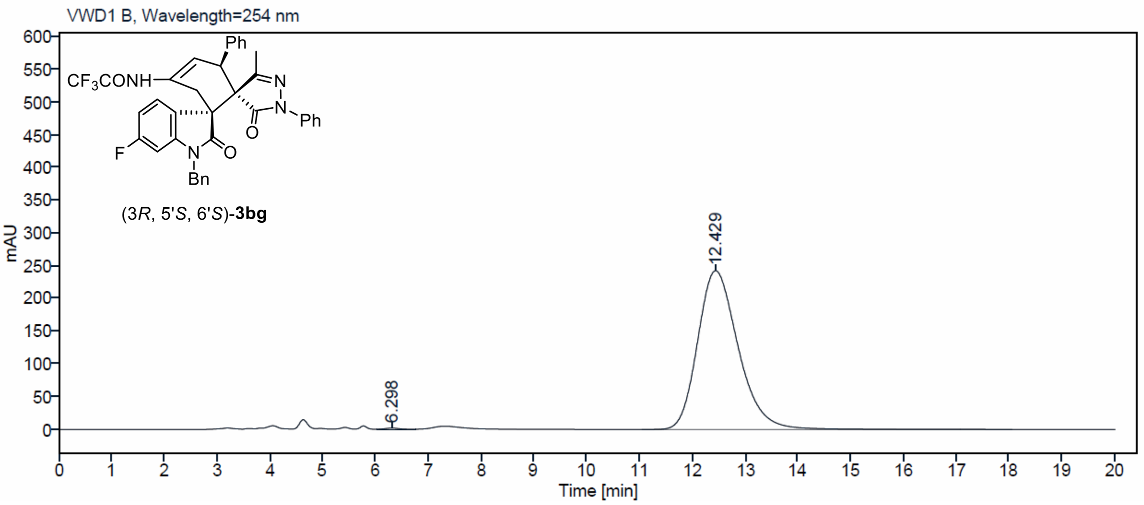

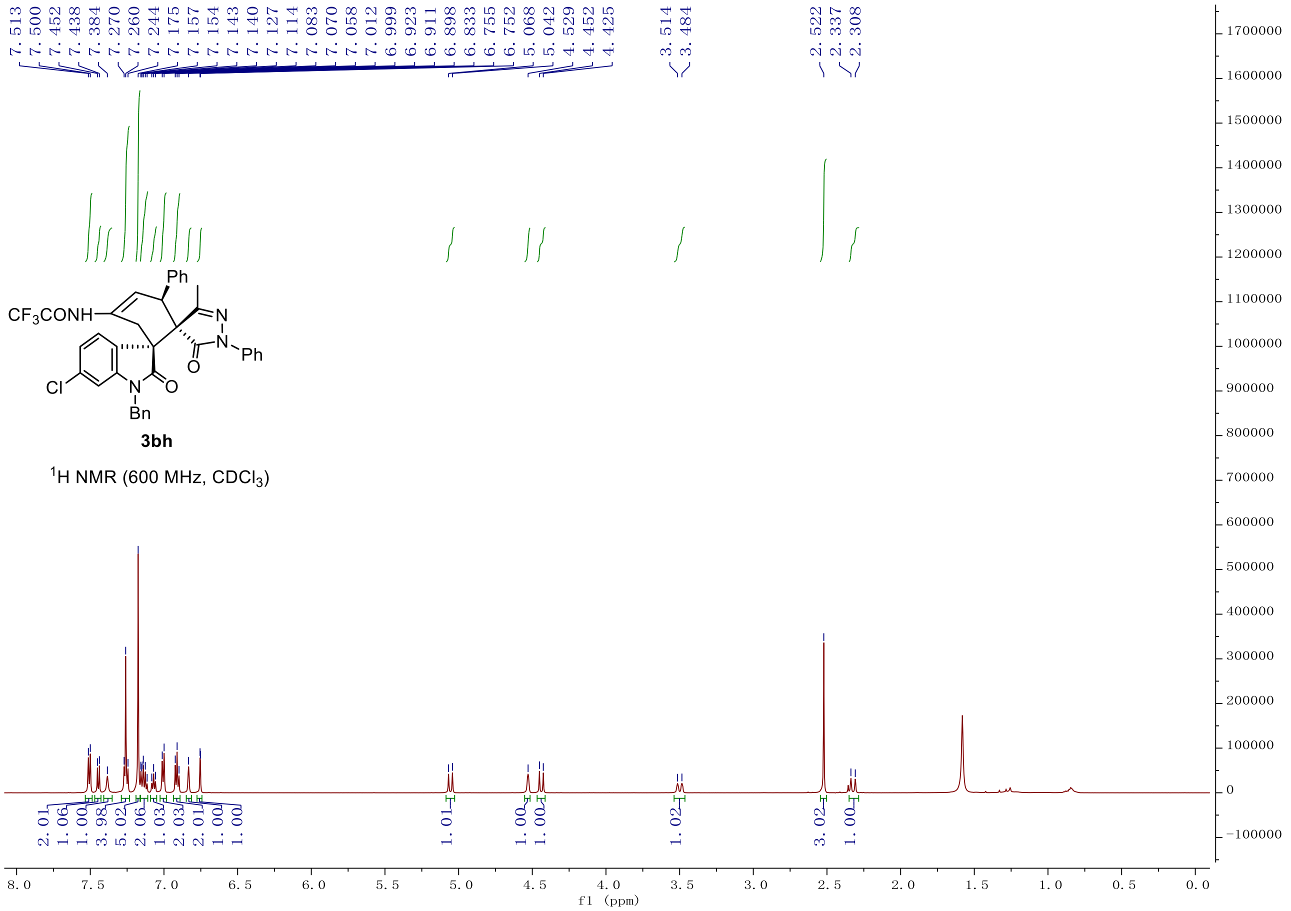

3bh

^{13}C NMR (150 MHz, $CDCl_3$)

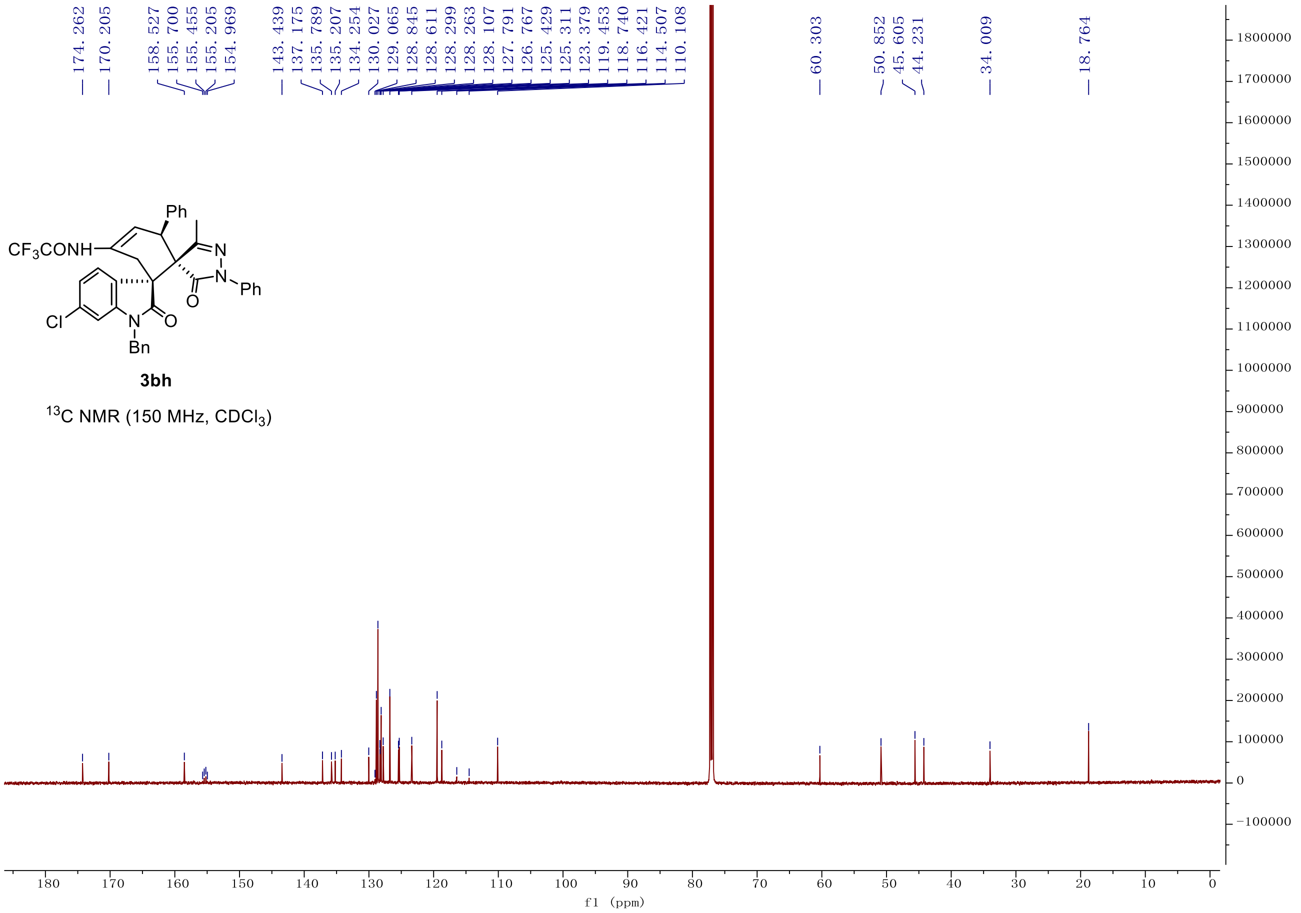

3bh

^{19}F NMR (564 MHz, $CDCl_3$)

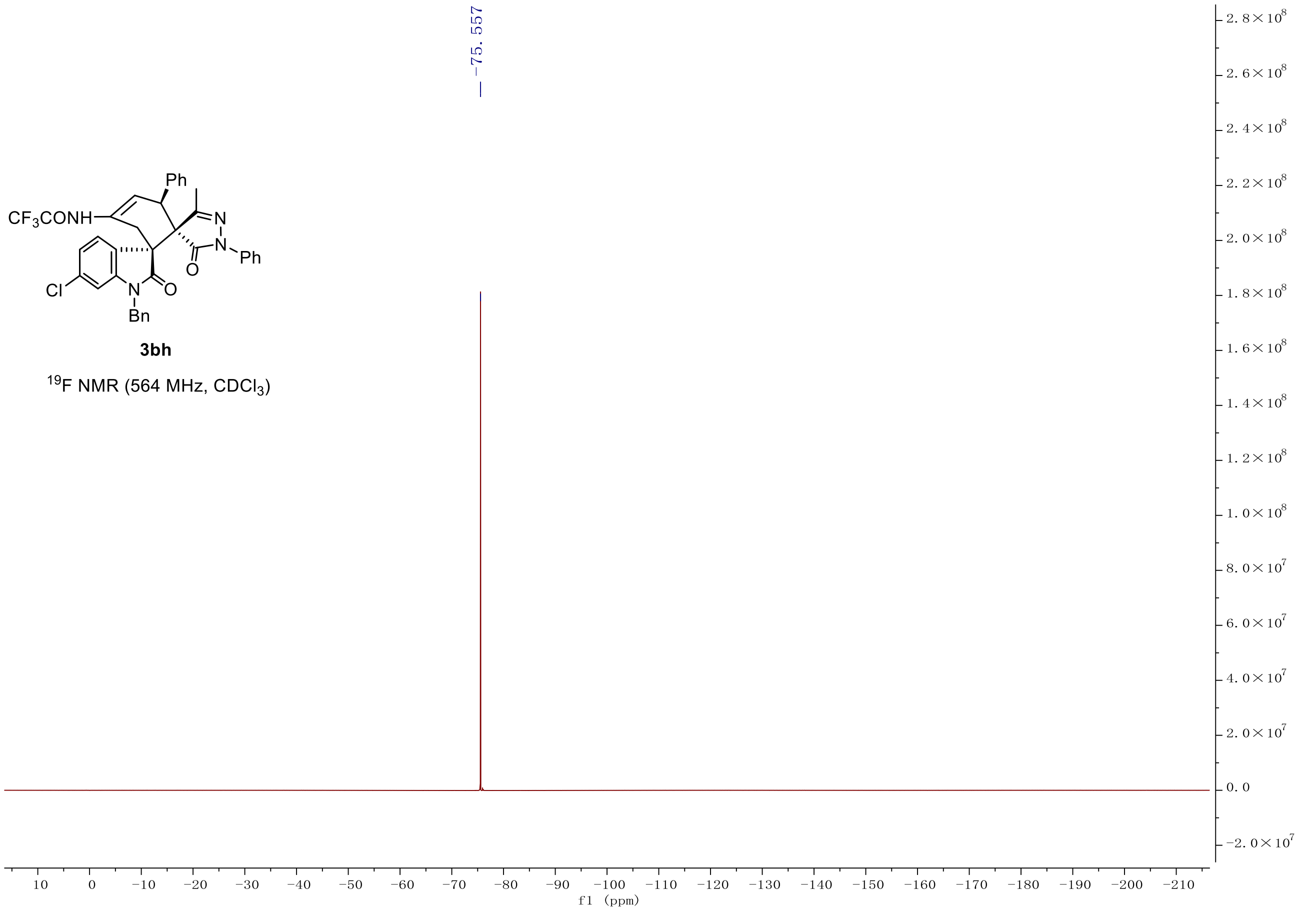

Peak Analysis Report

Detector A Channel 1 254nm

No.	Ret. Time	Height (mAu)	Area (mAu*min)	Rel. Area (%)
1	6.020	103148	1893875	49.408
2	14.020	22525	1939251	50.592
Total		125673	3833126	100.000

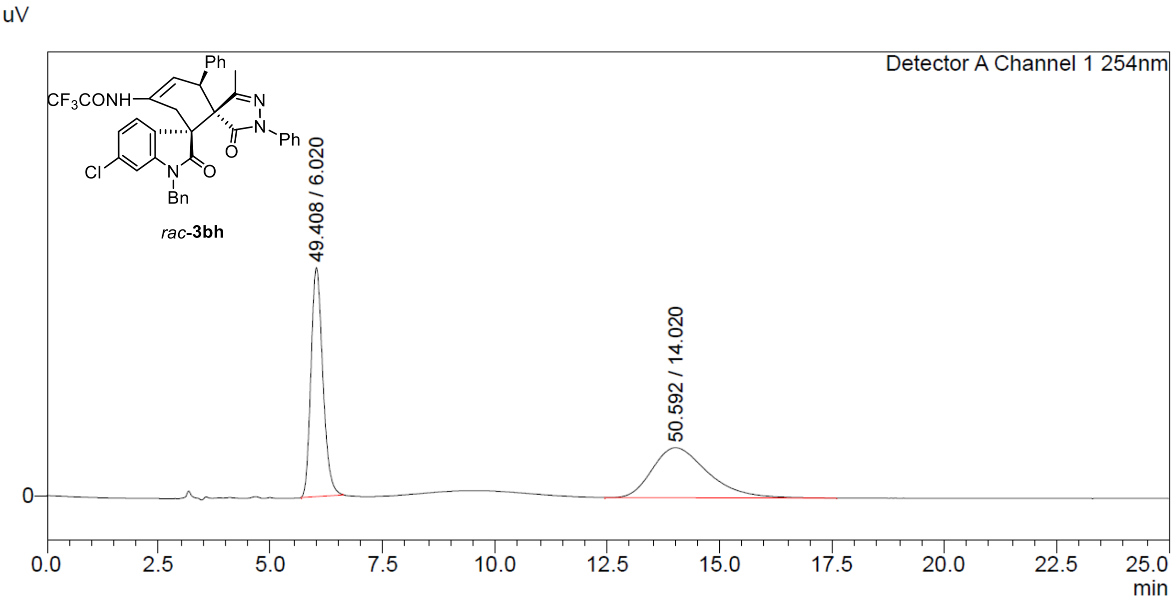

Peak Analysis Report

Detector A Channel 1 254nm

No.	Ret. Time	Height (mAu)	Area (mAu*min)	Rel. Area (%)
1	6.152	237	2068	0.045
2	13.985	53854	4592949	99.955
Total		54091	4595017	100.000

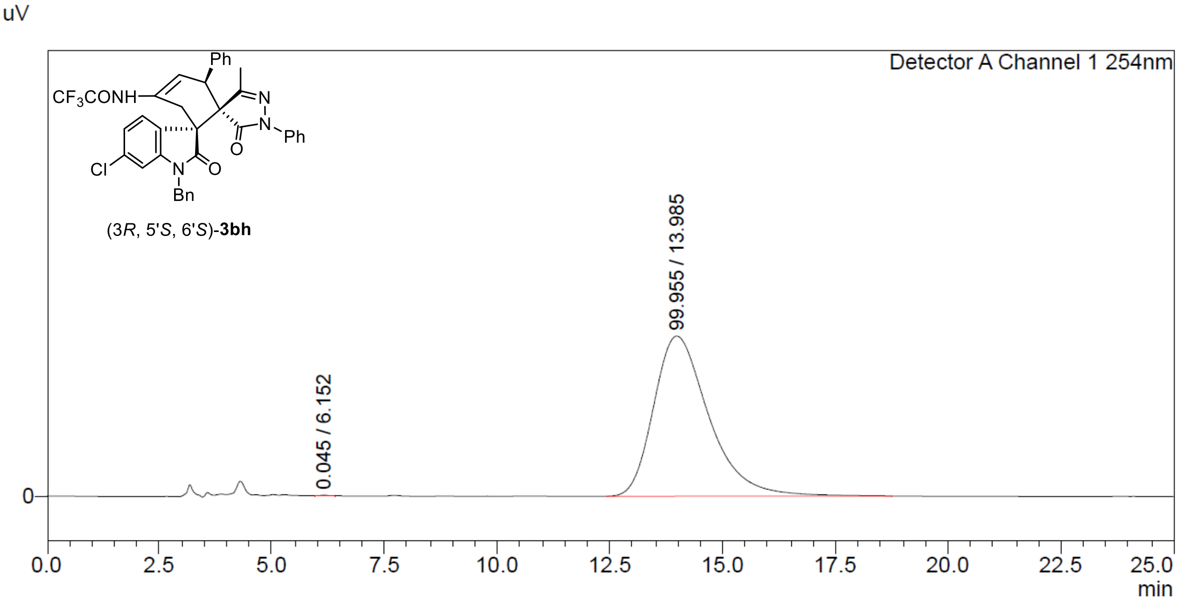

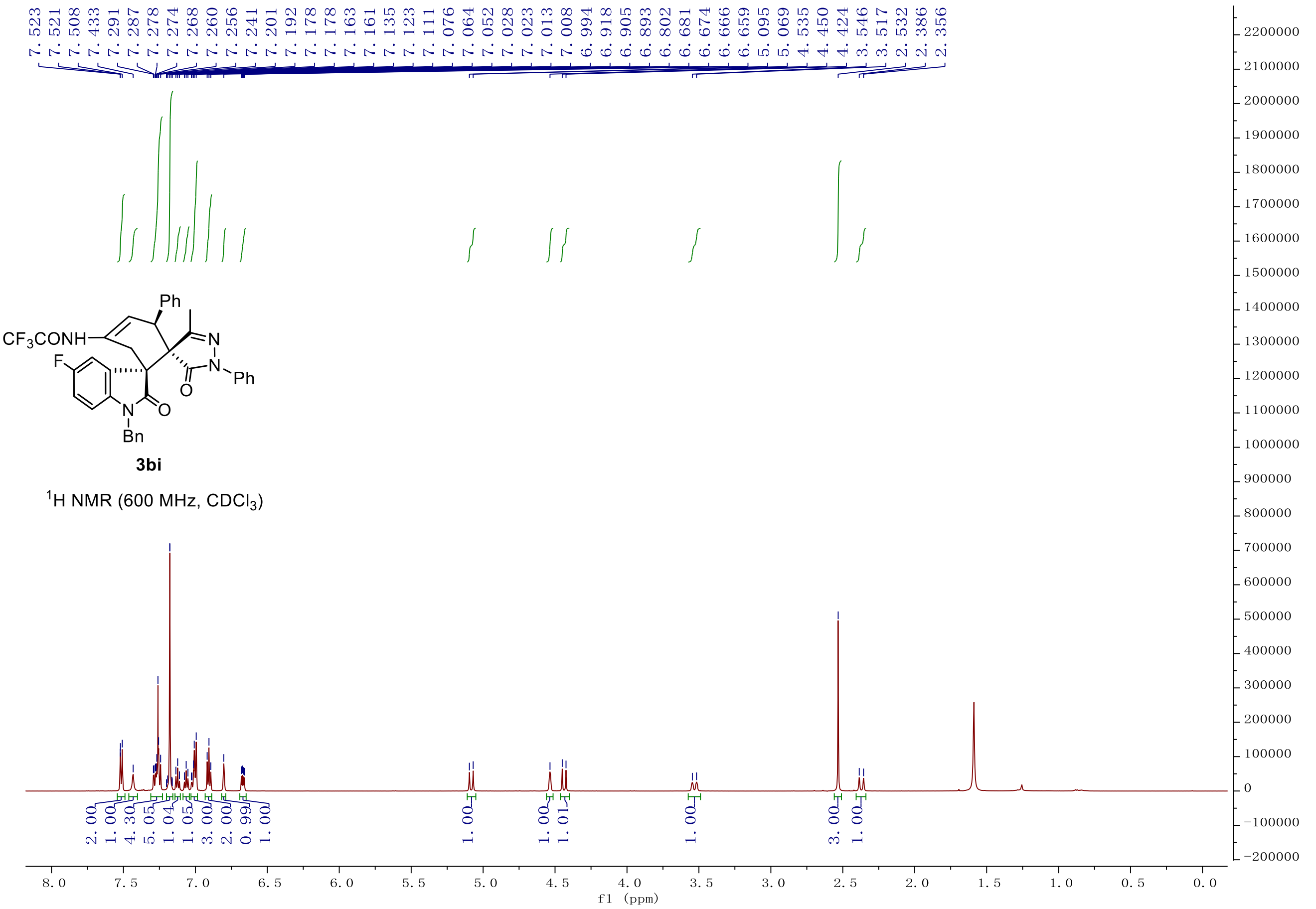

3bi

^{1}H NMR (600 MHz, $CDCl_3$)

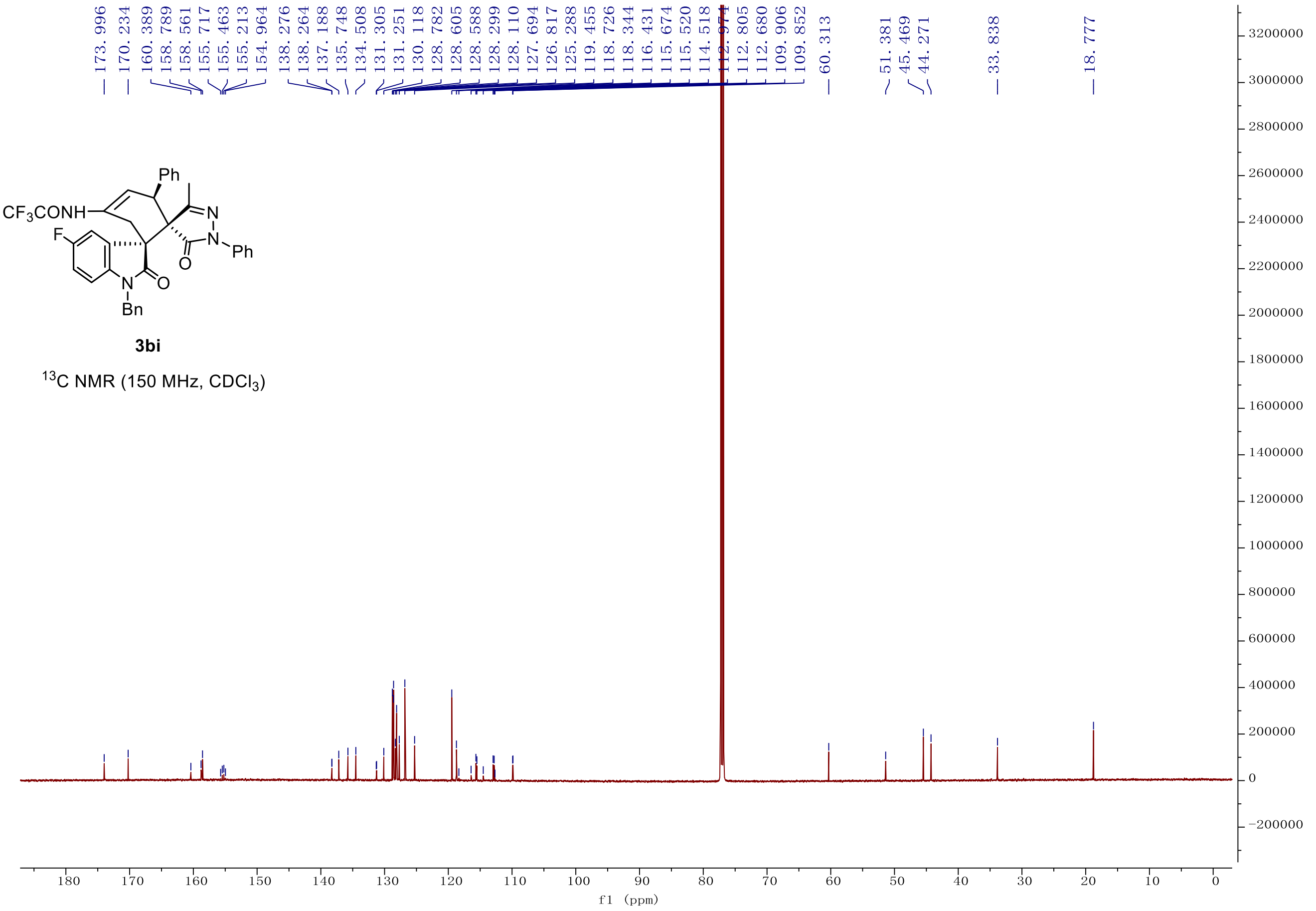

3bi

^{13}C NMR (150 MHz, $CDCl_3$)

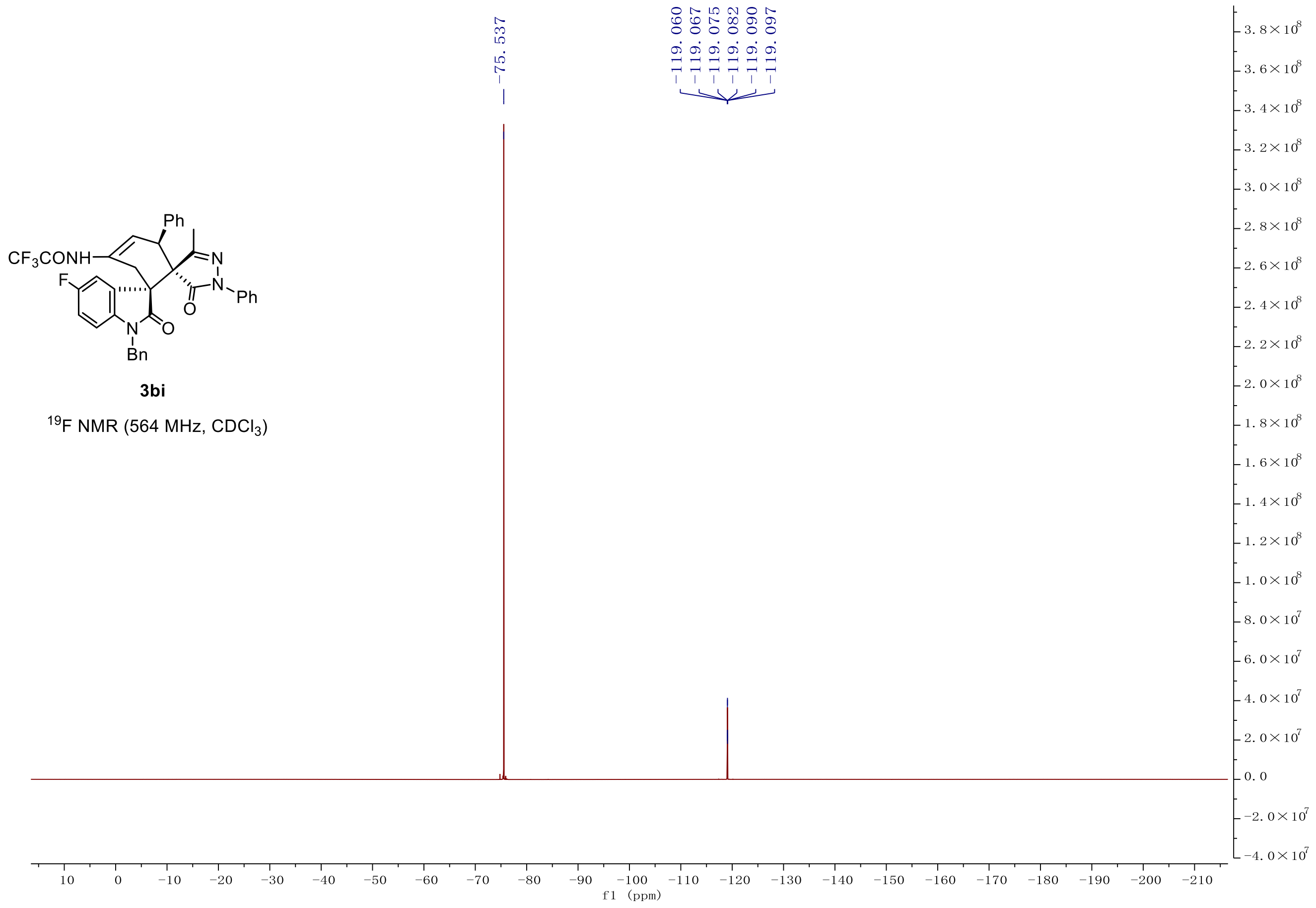

Signal: VWD1 B, Wavelength=254 nm

RT [min]	Type	Width [min]	Area	Height	Area%	Name
5.399	MM	0.2362	1804.5787	127.3341	50.2474	
6.549	MM	0.5015	1786.8063	59.3872	49.7526	
		Sum	3591.3850			

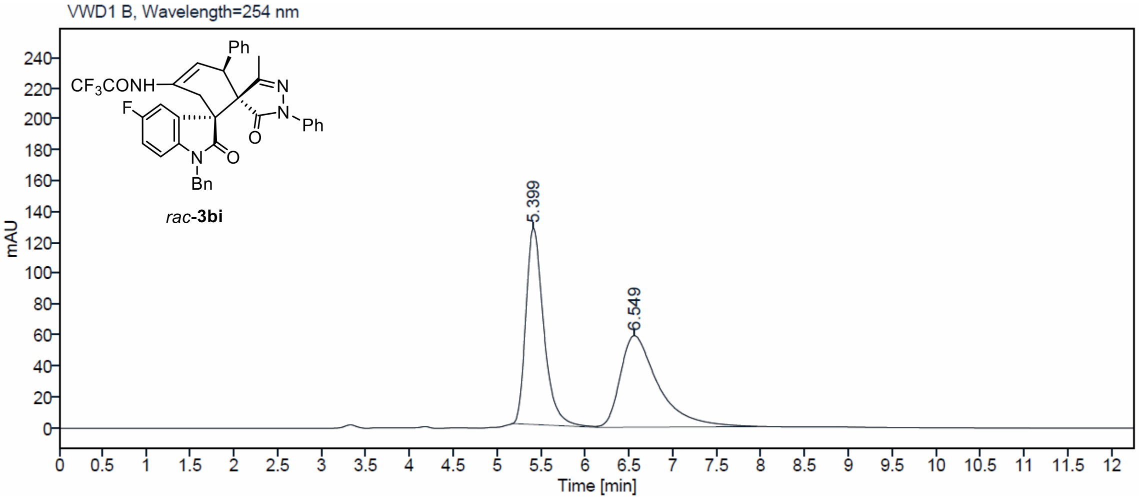

Signal: VWD1 B, Wavelength=254 nm

RT [min]	Type	Width [min]	Area	Height	Area%	Name
5.401	MM	0.2398	3777.6741	262.5912	98.5942	
6.526	MM	0.6682	53.8646	1.3435	1.4058	
		Sum	3831.5387			

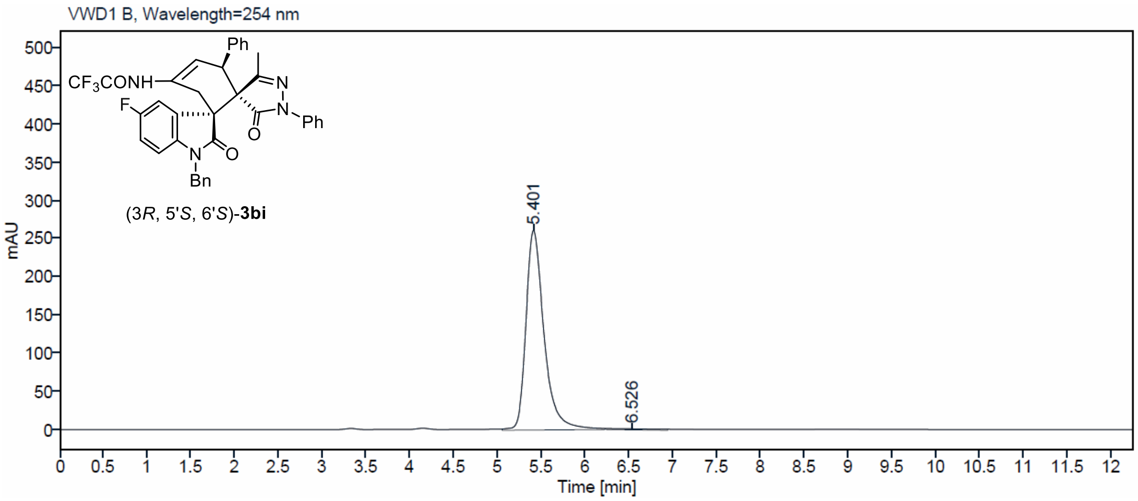

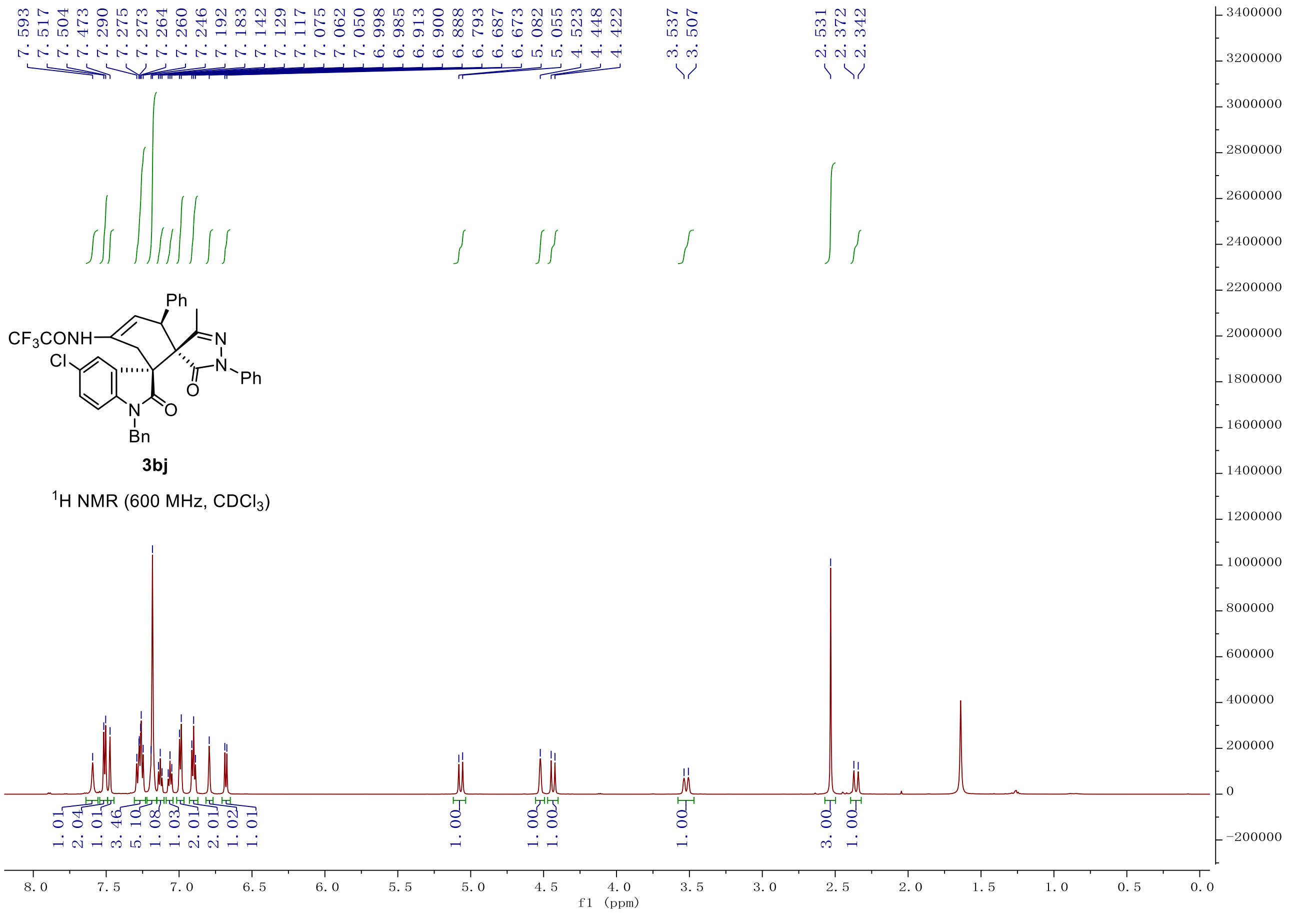

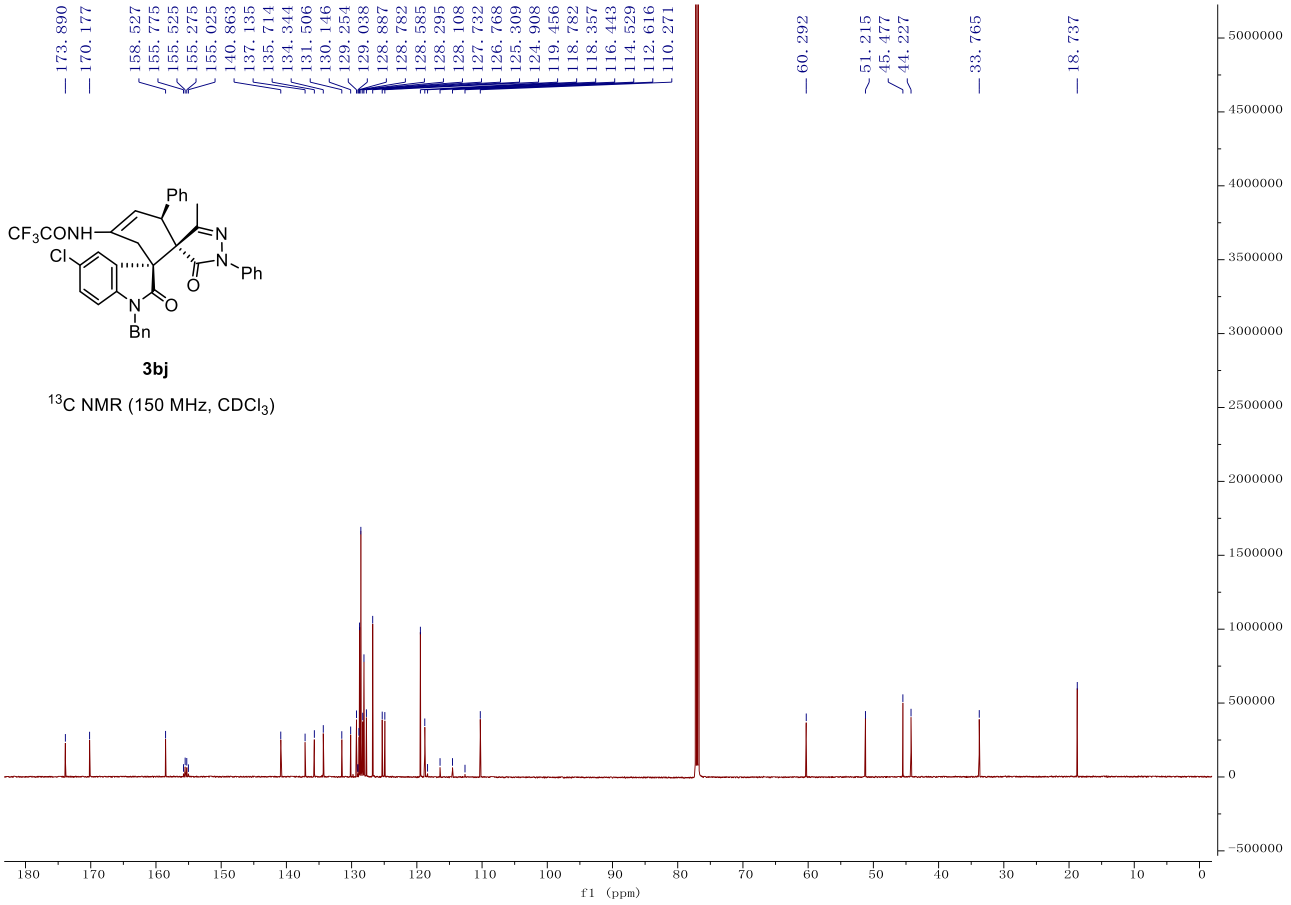
173.890
170.177
158.527
155.775
155.525
155.275
155.025
140.863
137.135
135.714
134.344
131.506
130.146
129.254
129.038
128.887
128.782
128.585
128.295
128.108
127.732
126.768
125.309
124.908
119.456
118.782
118.357
116.443
114.529
112.616
110.271
60.292
51.215
45.477
44.227
33.765
18.737
Ph
CF3CONH
Cl
N
N
Ph
O
N
O
Bn
3bj
13C NMR (150 MHz, CDCl3)
f1 (ppm)

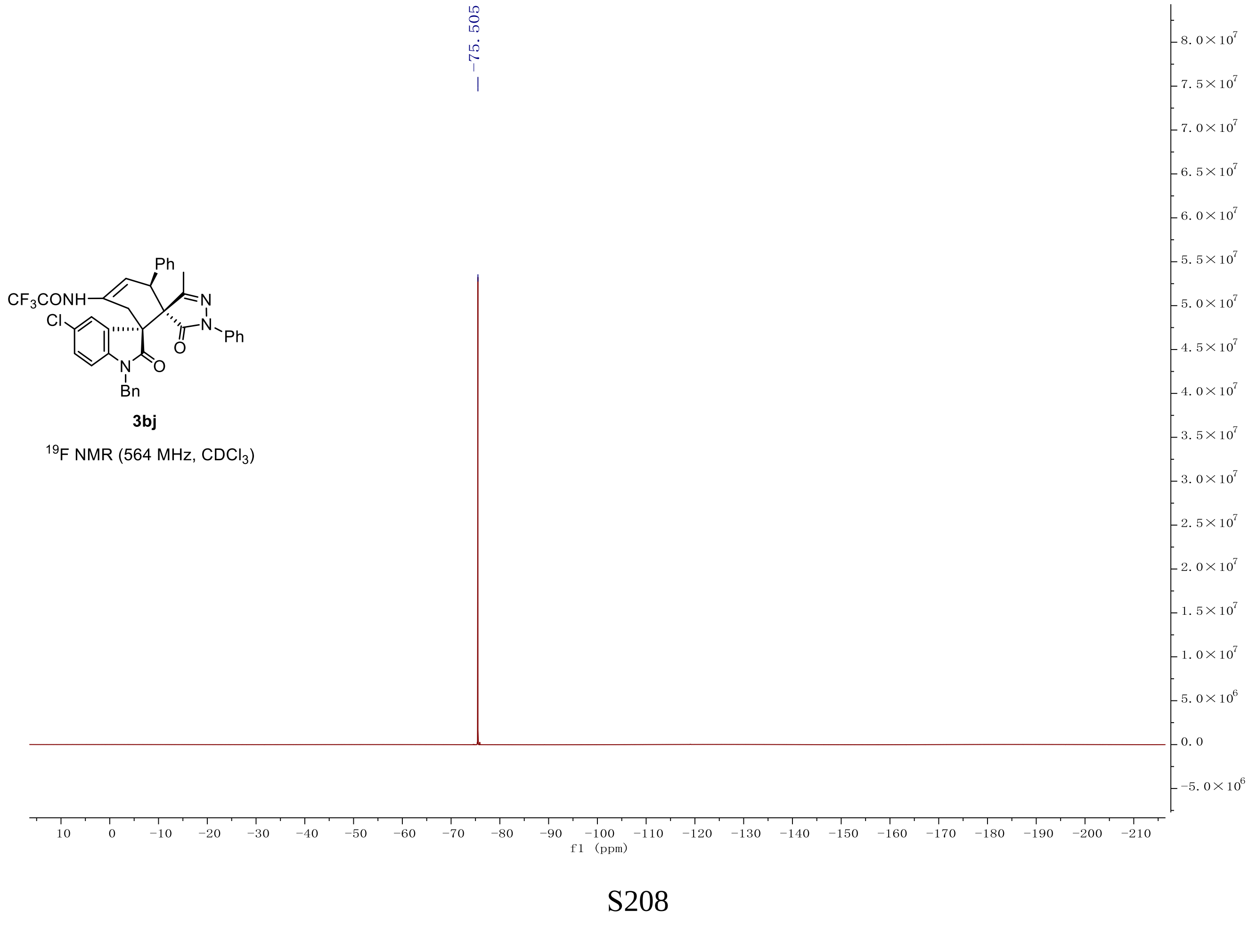
-75.505
Ph
CF3CONH
Cl
N
N
Ph
O
N
O
Bn
3bj
19F NMR (564 MHz, CDCl3)
f1 (ppm)

Signal: VWD1 B, Wavelength=254 nm

RT [min]	Type	Width [min]	Area	Height	Area%	Name
4.954	VV R	0.2409	3474.1848	199.7296	59.1674	
6.577	VB	0.4911	2397.6064	71.8863	40.8326	
		Sum	5871.7913			

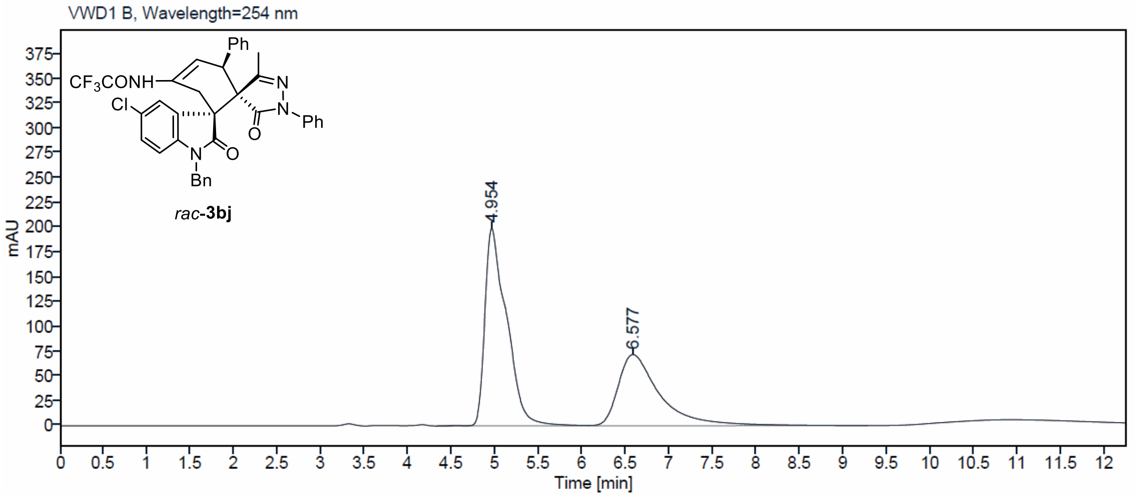

Signal: VWD1 B, Wavelength=254 nm

RT [min]	Type	Width [min]	Area	Height	Area%	Name
4.950	MM	0.2150	3893.1956	301.7892	98.1576	
6.508	MM	0.7110	73.0755	1.7130	1.8424	
		Sum	3966.2710			

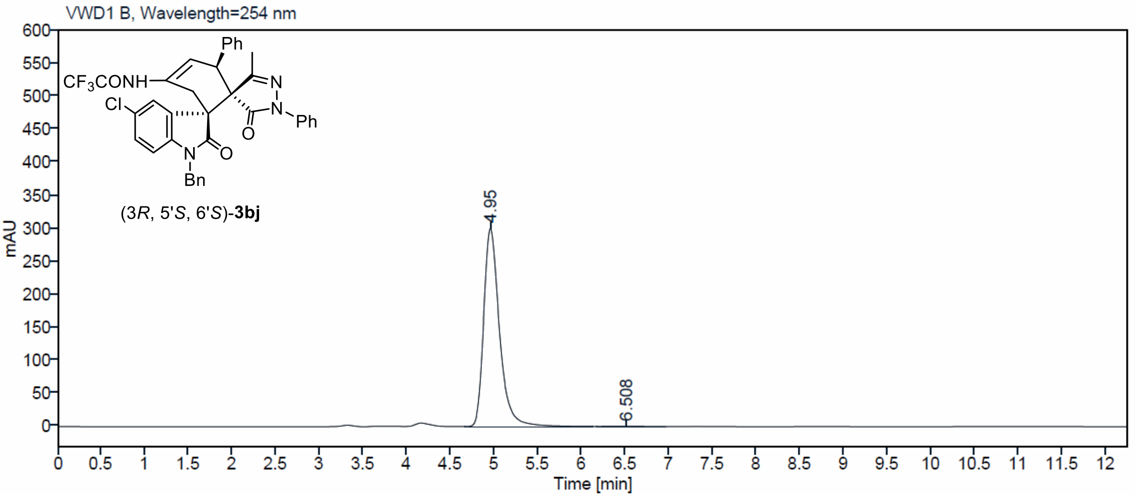

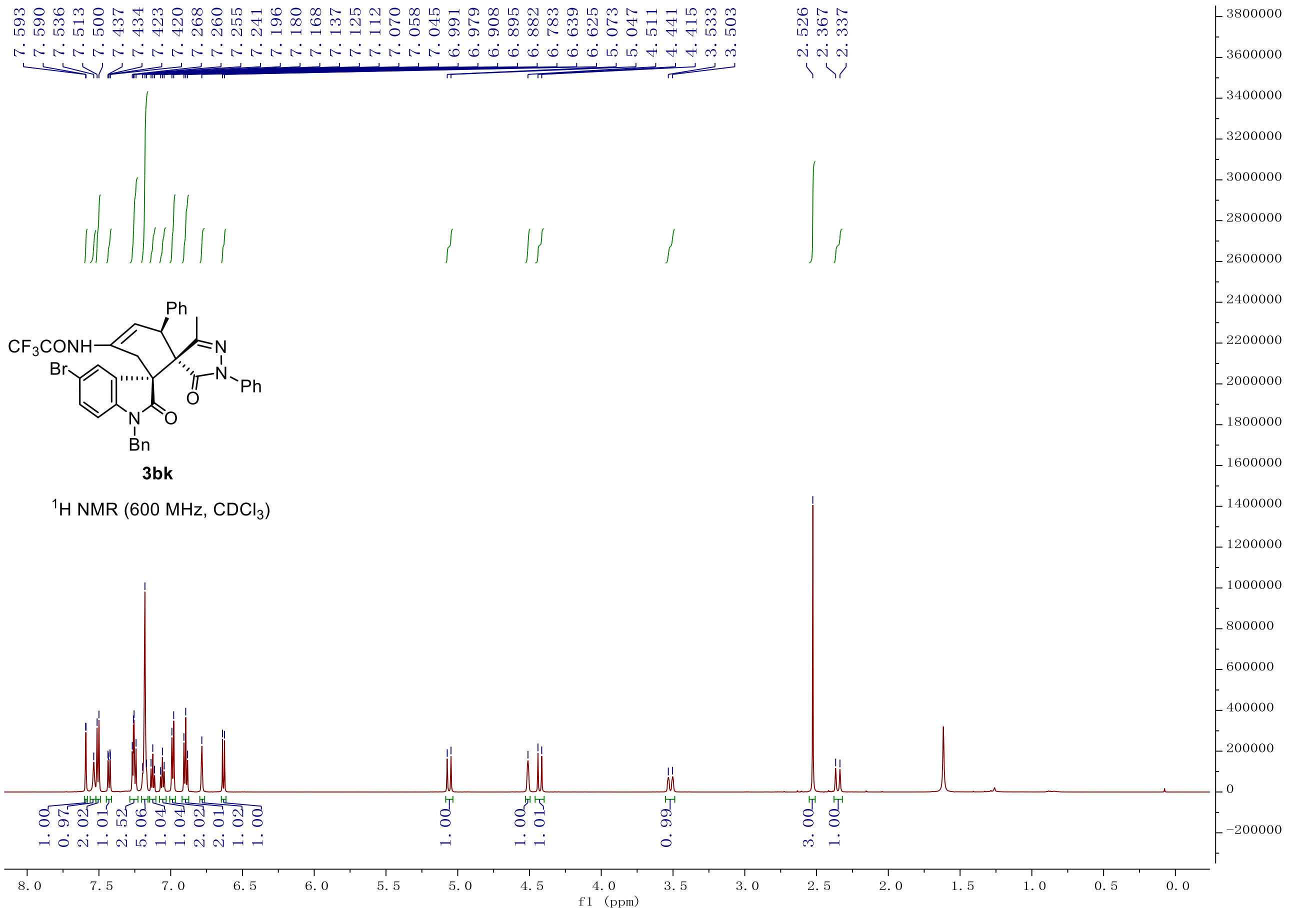
7.593 7.590 7.536 7.513 7.500 7.437 7.434 7.423 7.420 7.268 7.260 7.255 7.241 7.196 7.180 7.168 7.137 7.125 7.112 7.070 7.058 7.045 6.991 6.979 6.908 6.895 6.882 6.783 6.639 6.625 5.073 5.047 4.511 4.441 4.415 3.533 3.503 2.526 2.367 2.337
CF3CONH
Br
Ph
N
N
Ph
O
N
O
Bn
3bk
1H NMR (600 MHz, CDCl3)
1.00 0.97 2.02 1.01 2.52 5.06 1.04 1.04 2.02 2.01 1.02 1.00 1.00 1.00 1.01 0.99 3.00 1.00
f1 (ppm)

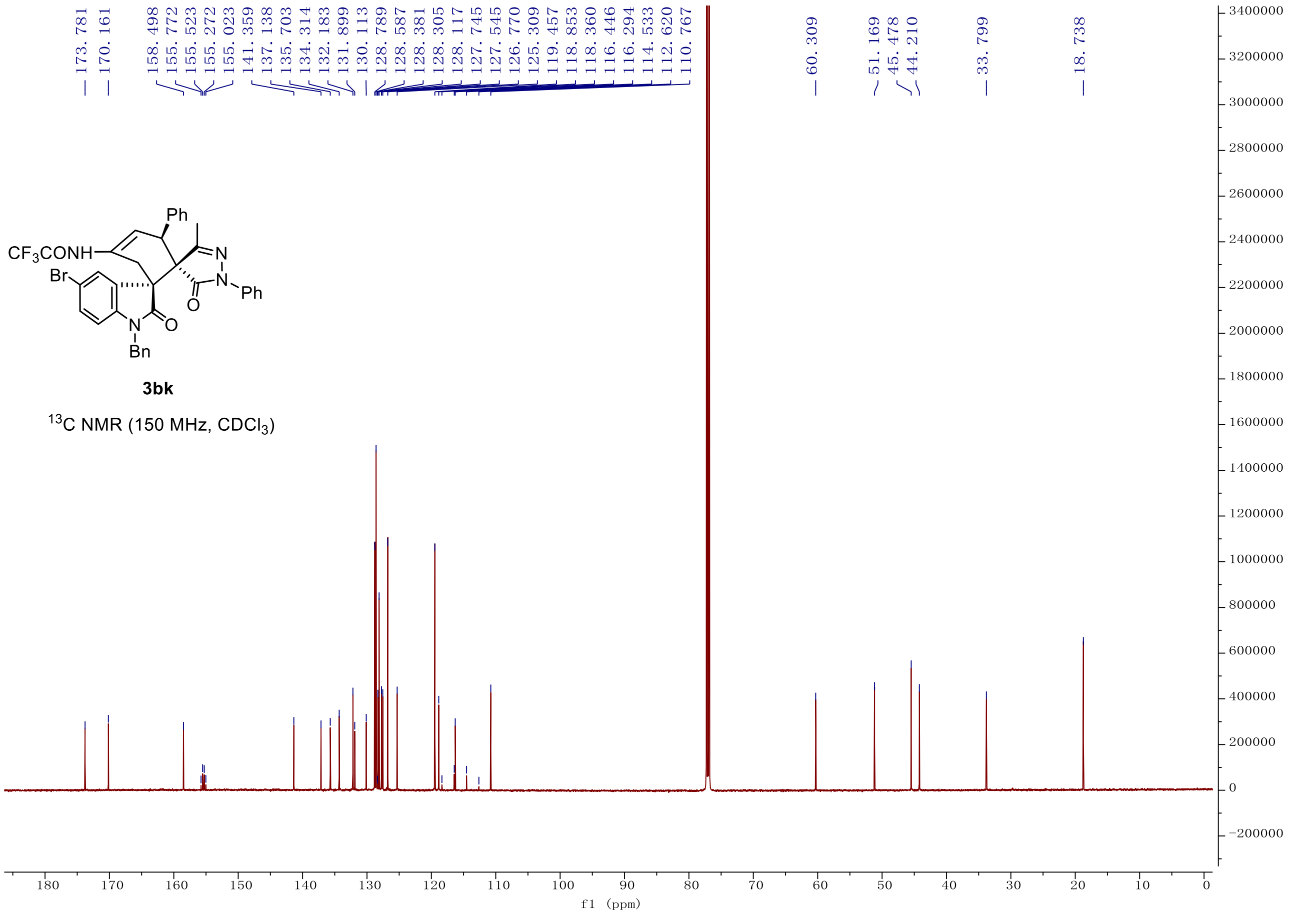
173.781 170.161 158.498 155.772 155.523 155.272 155.023 141.359 137.138 135.703 134.314 132.183 131.899 130.113 128.789 128.587 128.381 128.305 128.117 127.745 127.545 126.770 125.309 119.457 118.853 118.360 116.446 116.294 114.533 112.620 110.767 60.309 51.169 45.478 44.210 33.799 18.738
CF3CONH
Br
Ph
N
N
Ph
O
N
O
Bn
3bk
13C NMR (150 MHz, CDCl3)
f1 (ppm)

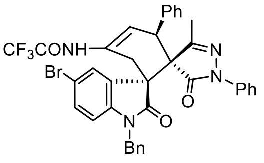

3bk

^{19}F NMR (564 MHz, $CDCl_3$)

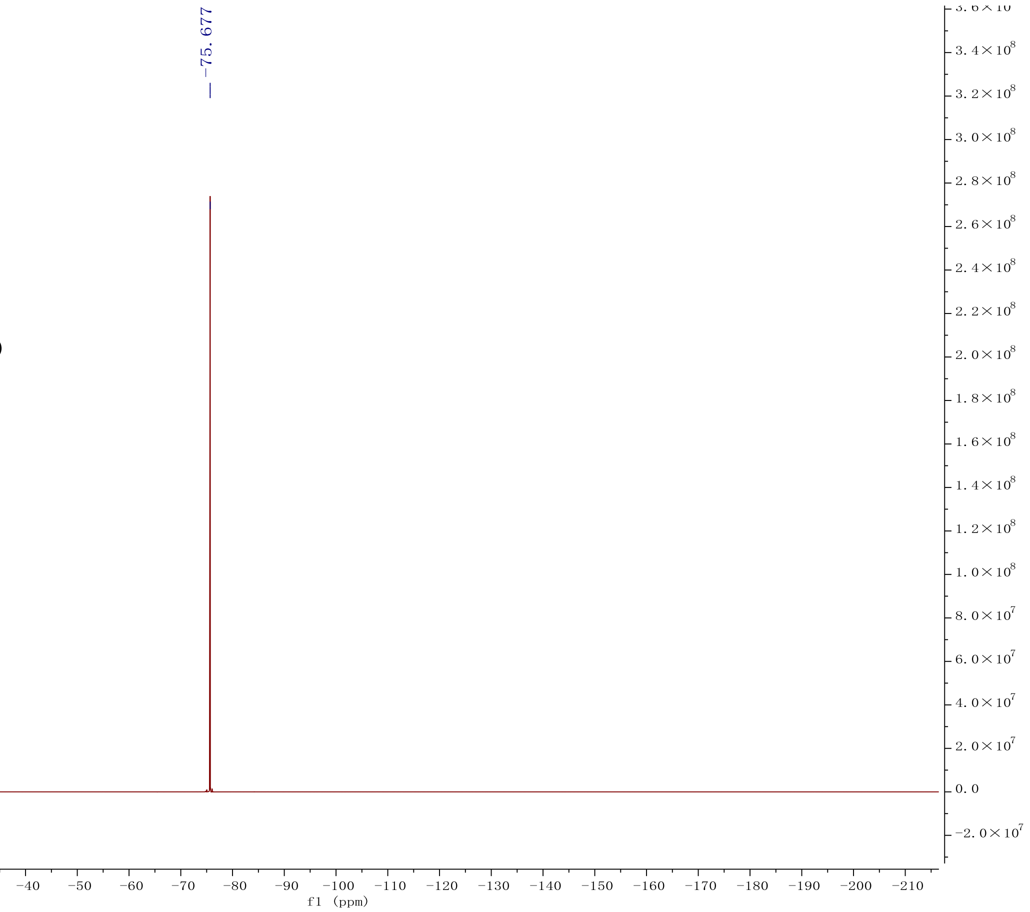

Peak Analysis Report

Detector A Channel 1 254nm

No.	Ret. Time	Height (mAu)	Area (mAu*min)	Rel. Area (%)
1	10.868	43174	1365309	22.944
2	12.052	58553	1665261	27.985
3	14.000	42745	1629970	27.392
4	15.421	33873	1290029	21.679
Total		178346	5950569	100.000

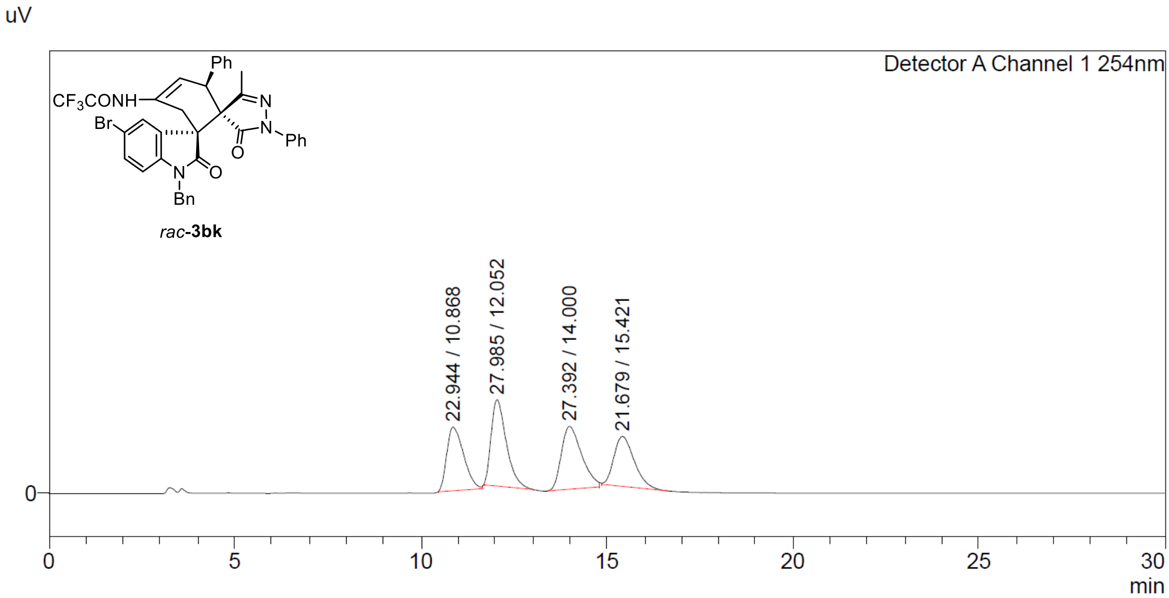

Peak Analysis Report

Detector A Channel 1 254nm

No.	Ret. Time	Height (mAu)	Area (mAu*min)	Rel. Area (%)
1	11.902	1022257	29840610	99.383
2	13.504	126	185119	0.617
Total		1022383	30025730	100.000

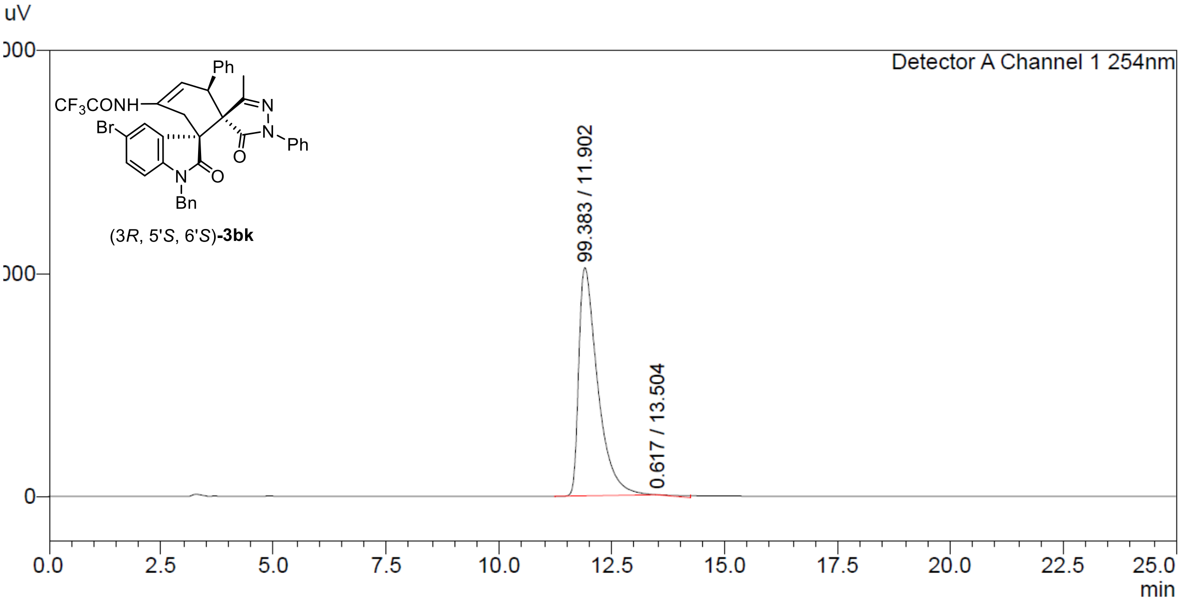

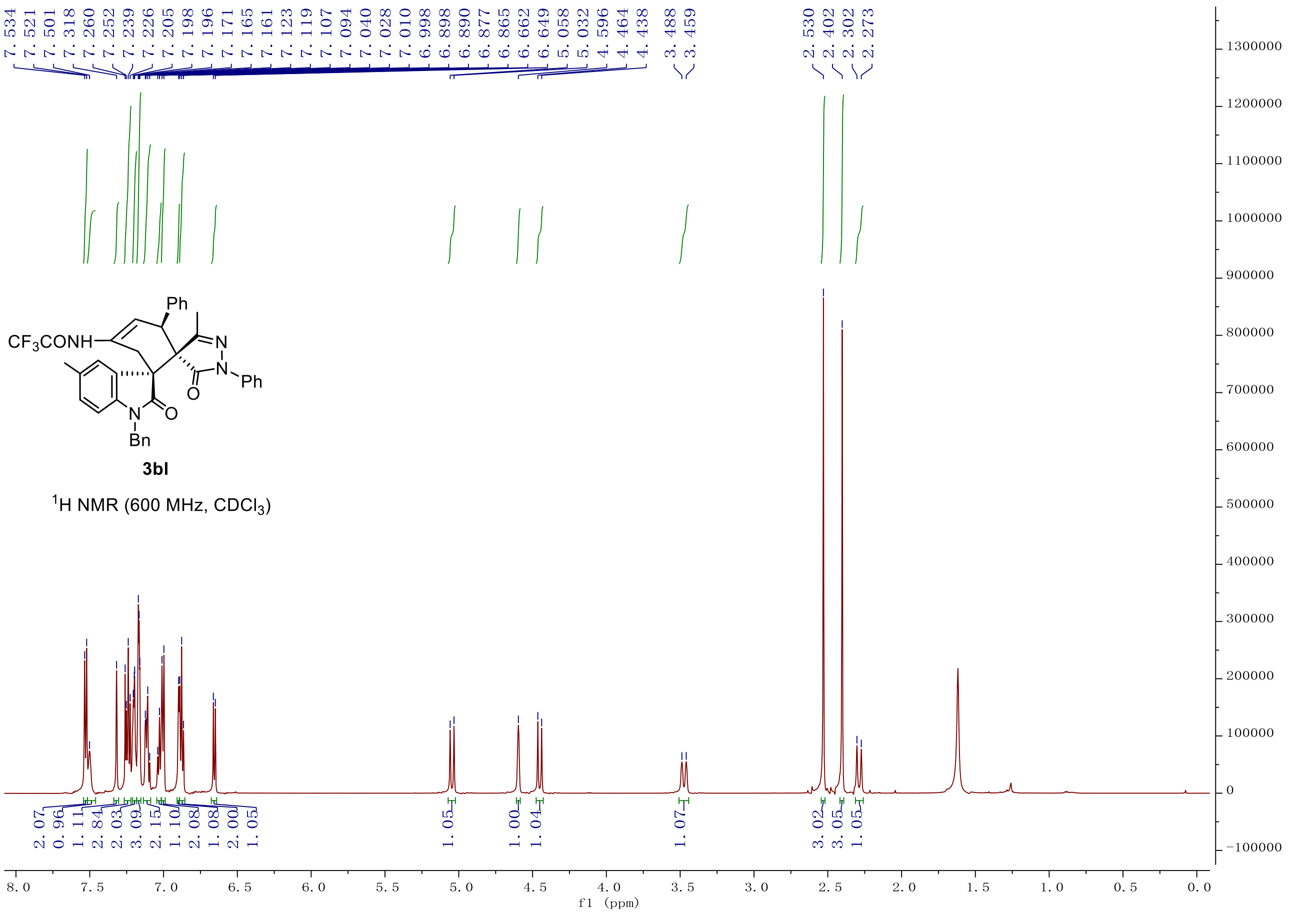

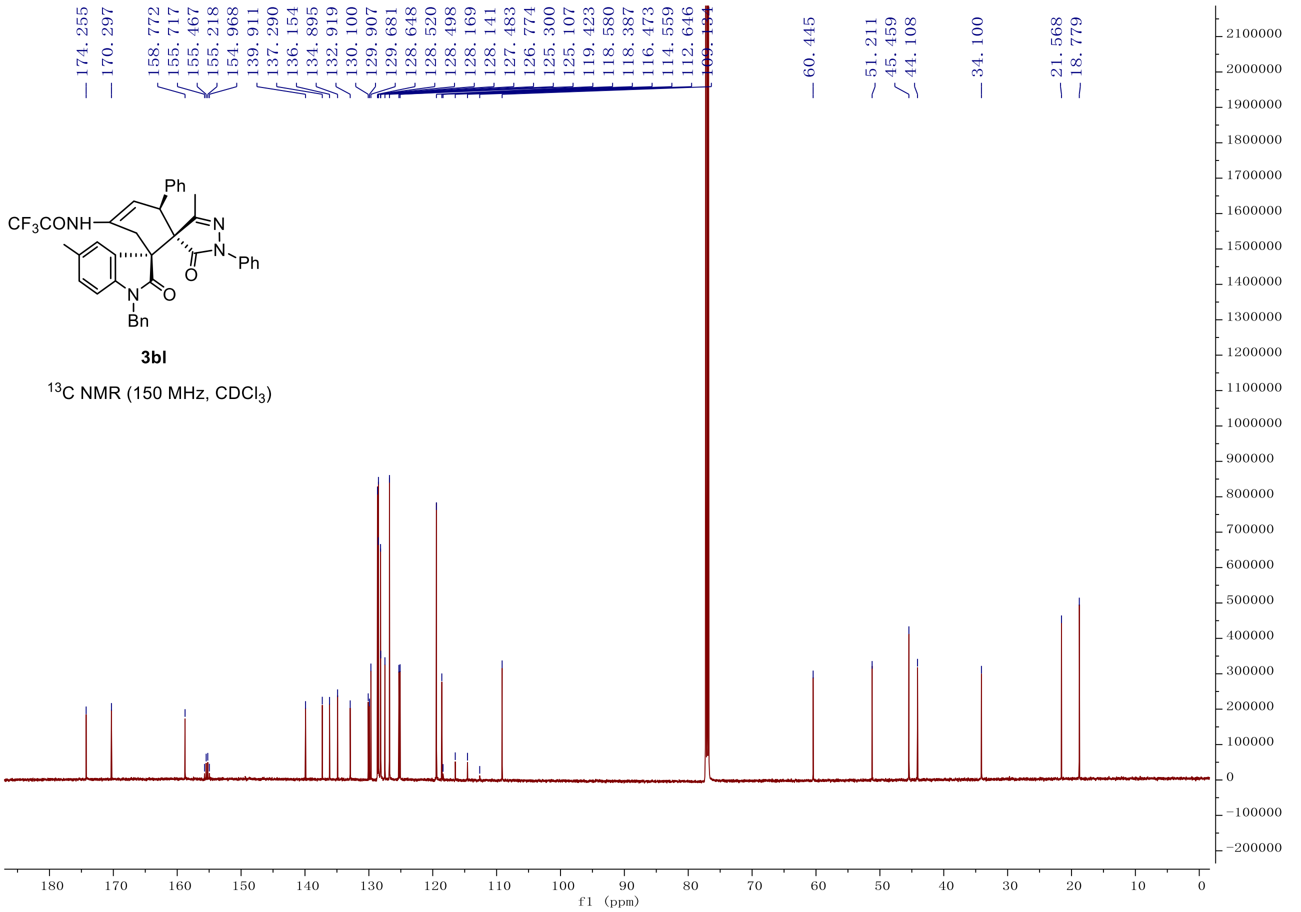
3bl
13C NMR (150 MHz, CDCl3)
f1 (ppm)

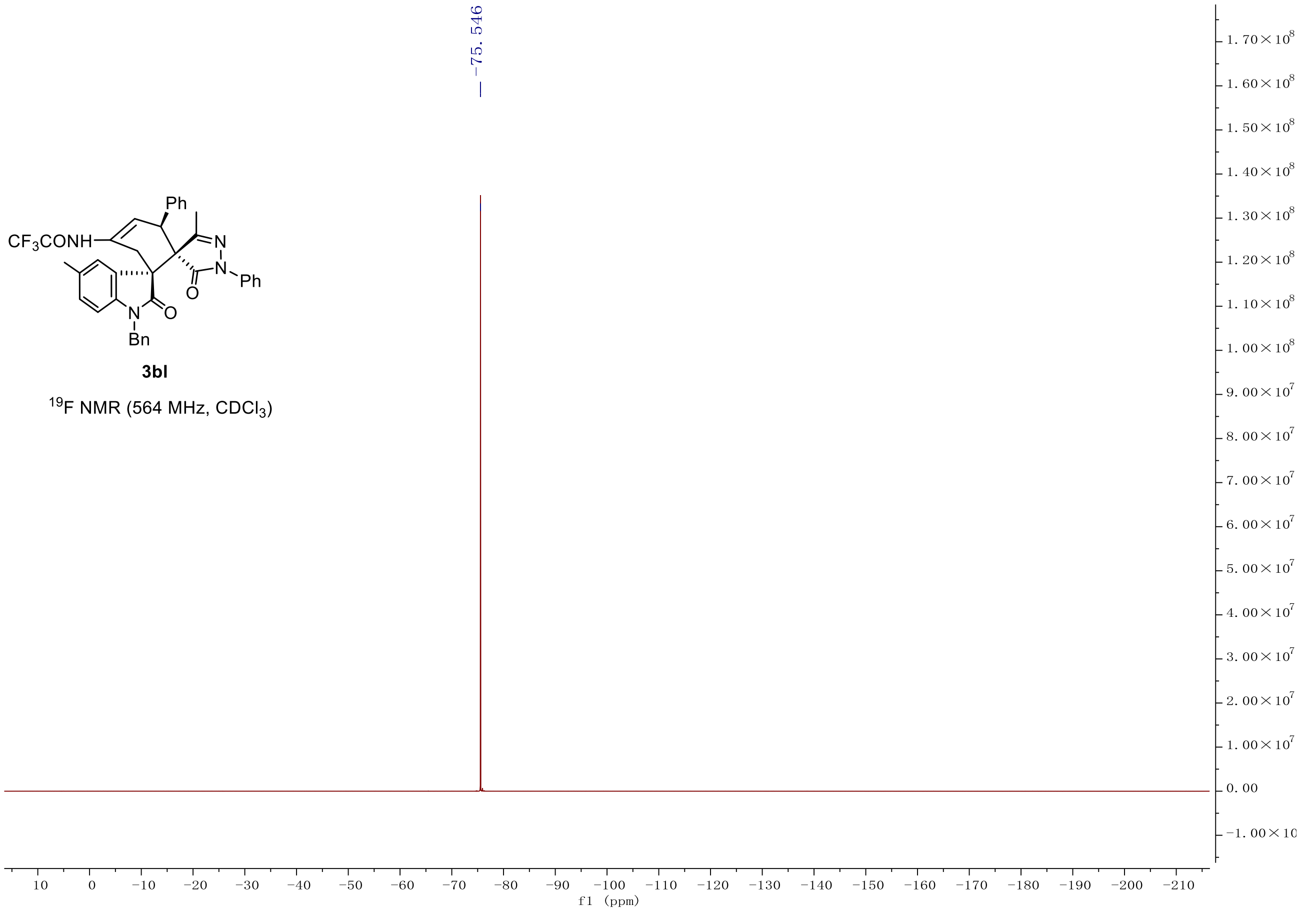
3bl
19F NMR (564 MHz, CDCl3)
−75.546
f1 (ppm)

Signal: VWD1 B, Wavelength=254 nm

RT [min]	Type	Width [min]	Area	Height	Area%	Name
5.246	MM	0.2221	12067.0674	905.4670	49.8433	
6.182	MM	0.3089	12142.9570	655.1857	50.1567	
		Sum	24210.0244			

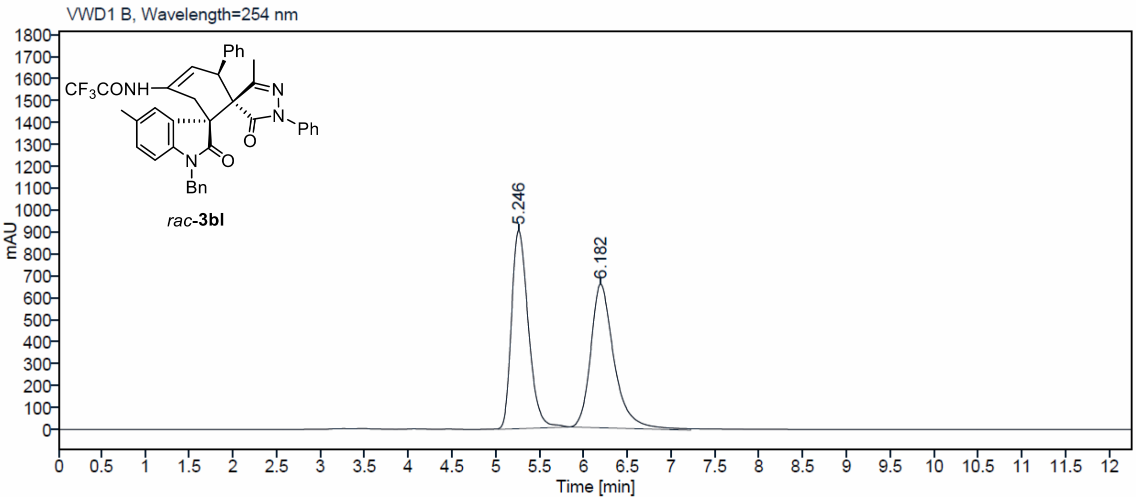

Signal: VWD1 B, Wavelength=254 nm

RT [min]	Type	Width [min]	Area	Height	Area%	Name
5.229	MM	0.2217	28422.5996	2136.8914	99.4339	
6.555	MM	0.1530	161.8278	17.6302	0.5661	
		Sum	28584.4274			

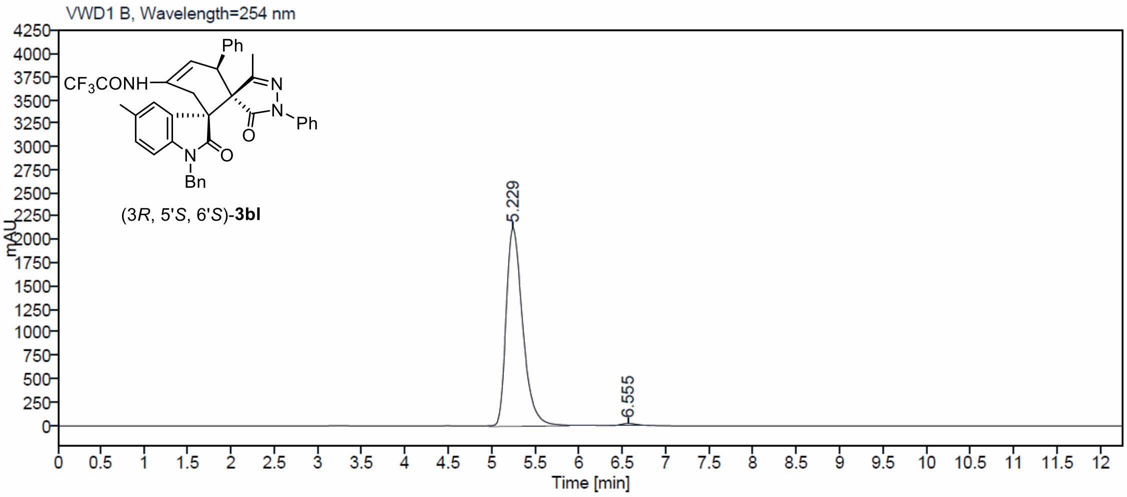

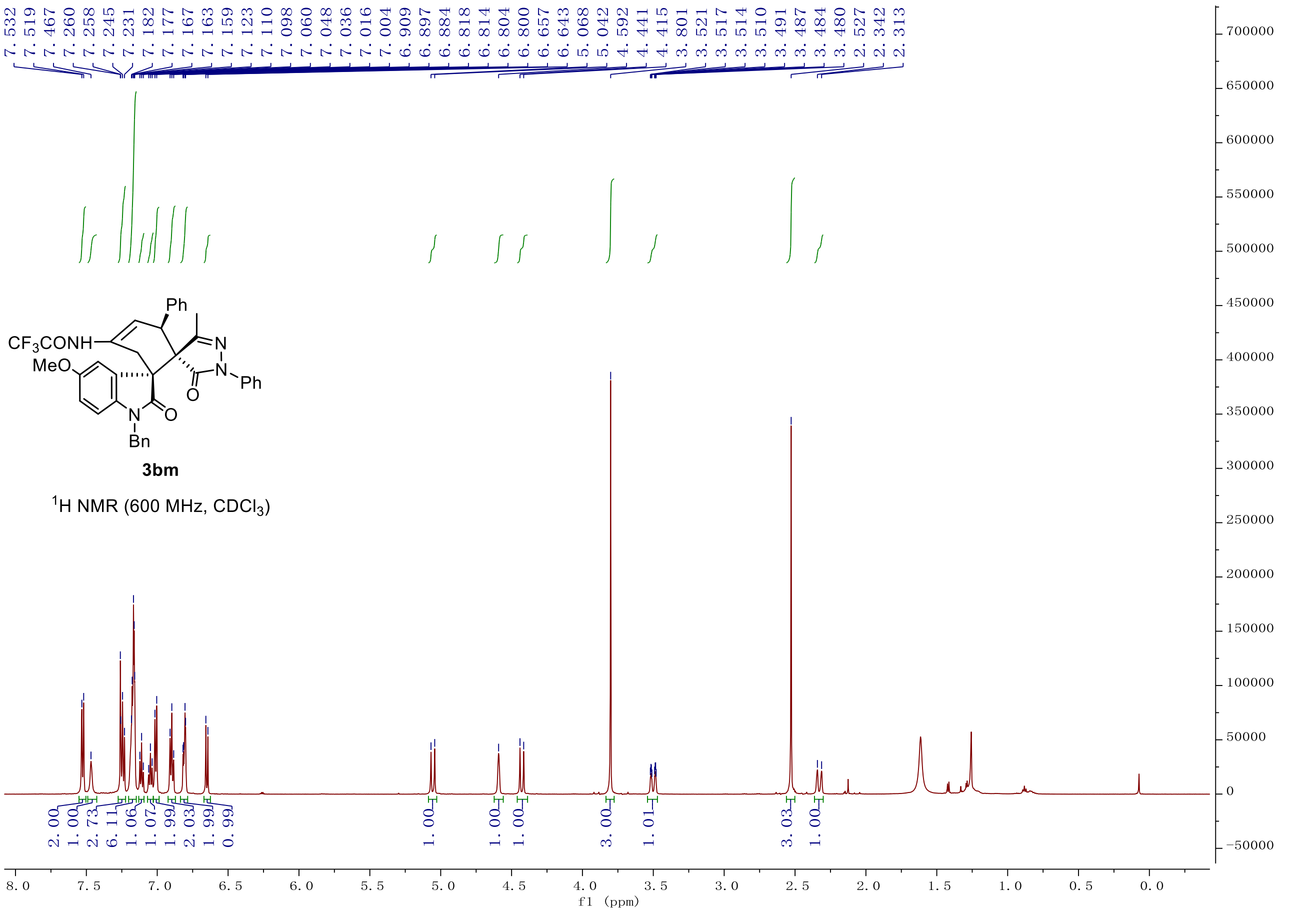
7.532 7.519 7.467 7.260 7.258 7.245 7.231 7.182 7.177 7.167 7.163 7.159 7.123 7.110 7.098 7.060 7.048 7.036 7.016 7.004 6.909 6.897 6.884 6.818 6.814 6.804 6.800 6.657 6.643 5.068 5.042 4.592 4.441 4.415 3.801 3.521 3.517 3.514 3.510 3.491 3.487 3.484 3.480 2.527 2.342 2.313
CF3CONH
MeO
Ph
N
O
Bn
3bm
1H NMR (600 MHz, CDCl3)
2.00 1.00 2.73 6.11 1.06 1.07 1.99 2.03 1.99 0.99 1.00 1.00 1.00 3.00 1.01 3.03 1.00
f1 (ppm)

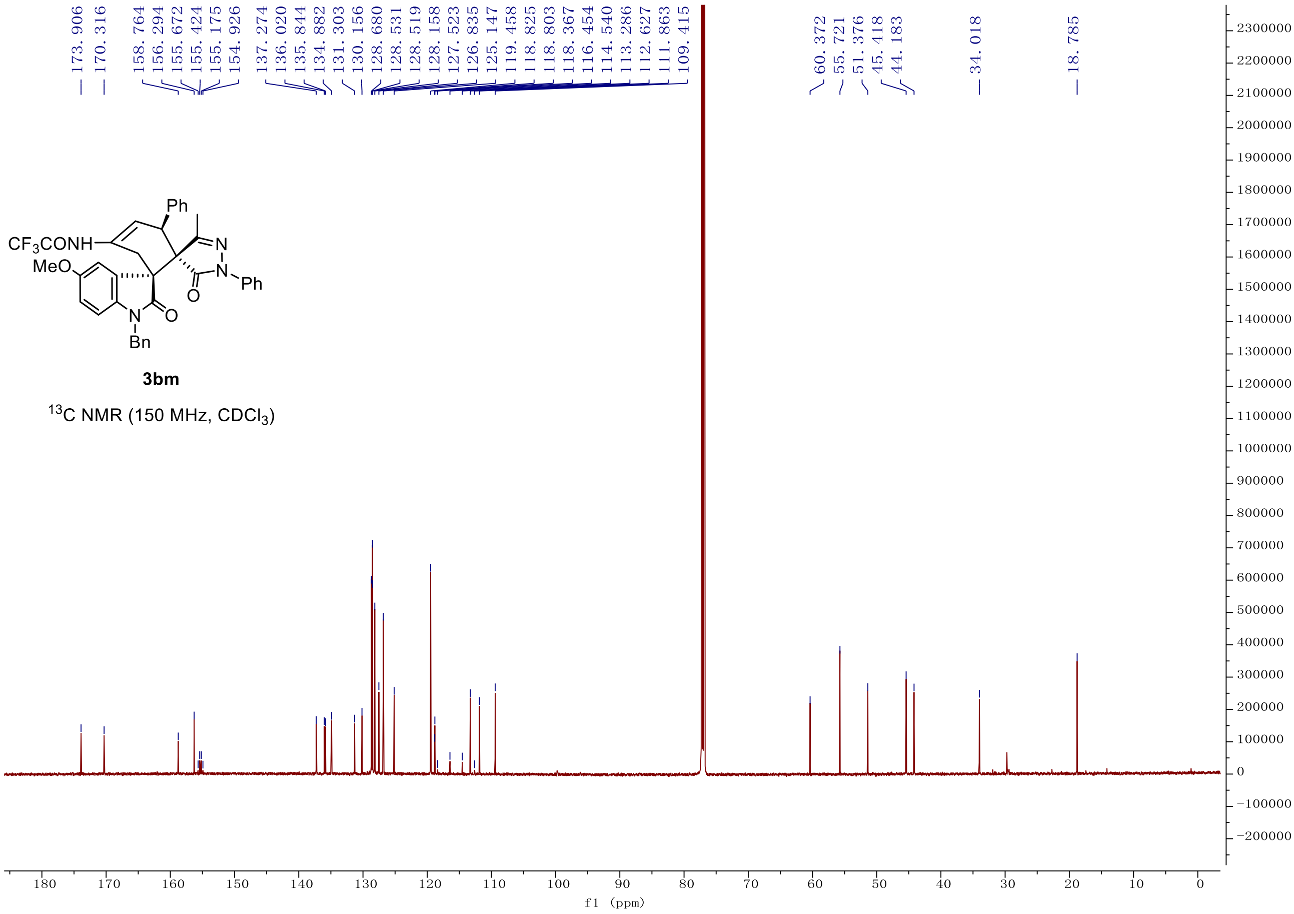
173.906 170.316 158.764 156.294 155.672 155.424 155.175 154.926 137.274 136.020 135.844 134.882 131.303 130.156 128.680 128.531 128.519 128.158 127.523 126.835 125.147 119.458 118.825 118.803 118.367 116.454 114.540 113.286 112.627 111.863 109.415 60.372 55.721 51.376 45.418 44.183 34.018 18.785
CF3CONH
MeO
Ph
N
O
Bn
3bm
13C NMR (150 MHz, CDCl3)
f1 (ppm)

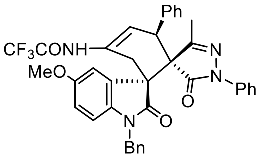

3bm

^{19}F NMR (564 MHz, $CDCl_3$)

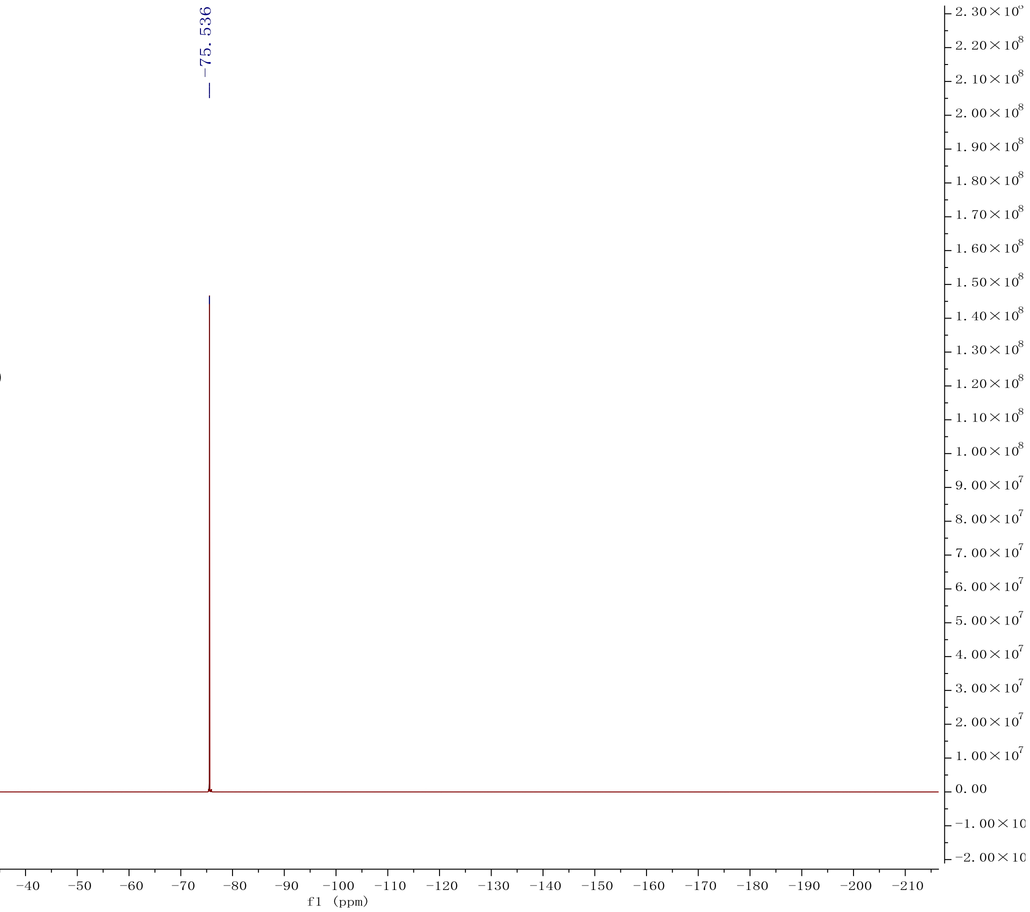

Peak Analysis Report

Detector A Channel 1 254nm

No.	Ret. Time	Height (mAu)	Area (mAu*min)	Rel. Area (%)
1	6.870	460448	7289799	49.605
2	7.839	386615	7405768	50.395
Total		847063	14695567	100.000

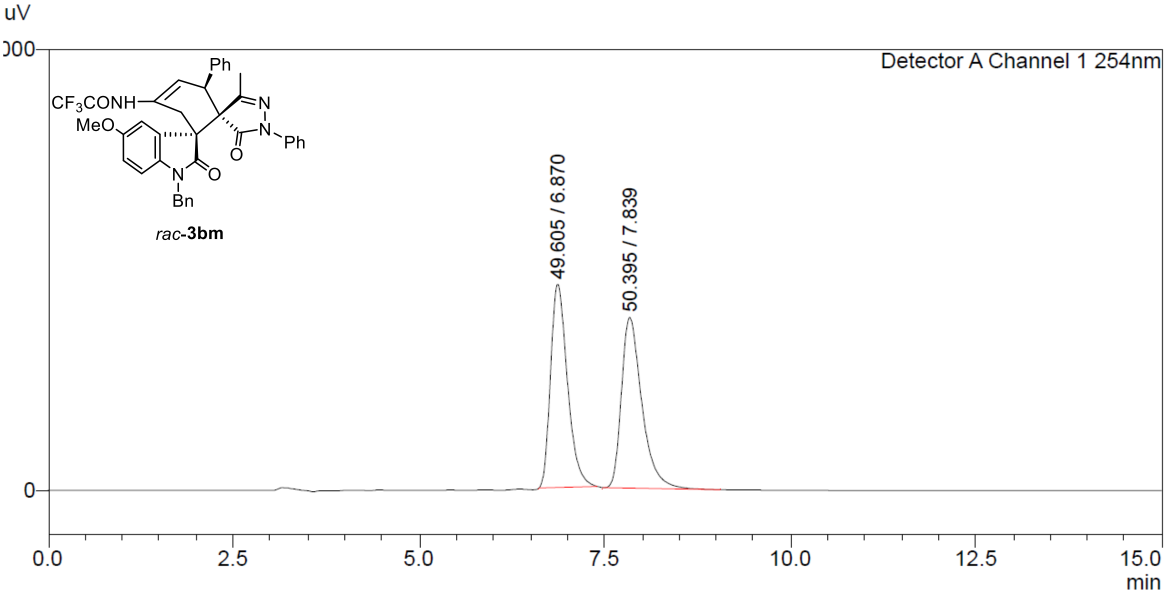

Peak Analysis Report

Detector A Channel 1 254nm

No.	Ret. Time	Height (mAu)	Area (mAu*min)	Rel. Area (%)
1	6.856	1688145	25068124	99.763
2	7.887	4344	59499	0.237
Total		1692489	25127623	100.000

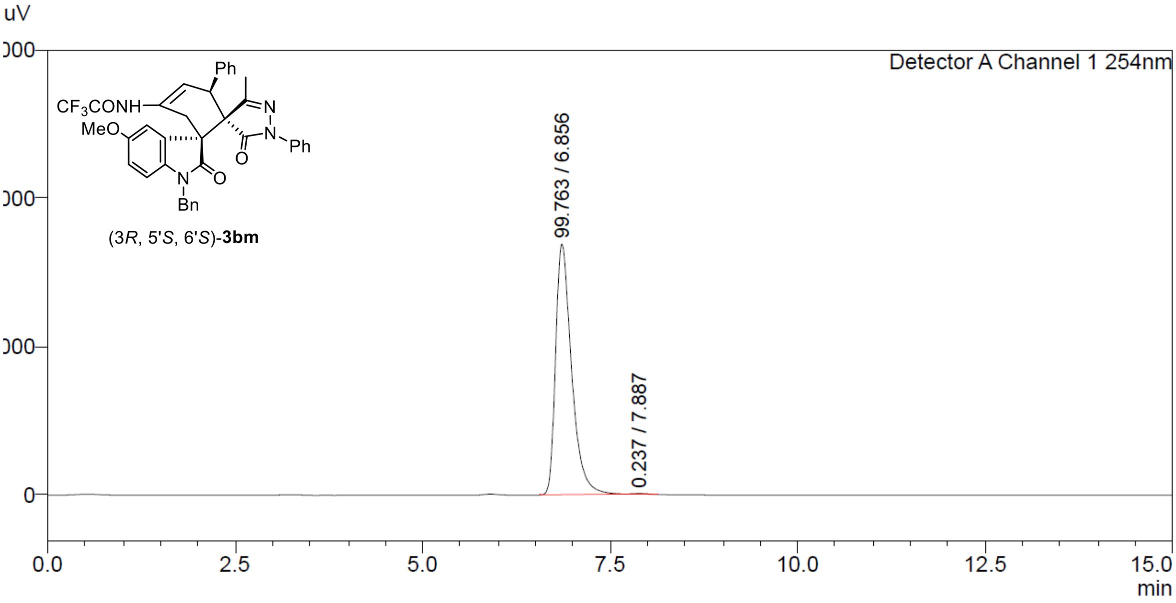

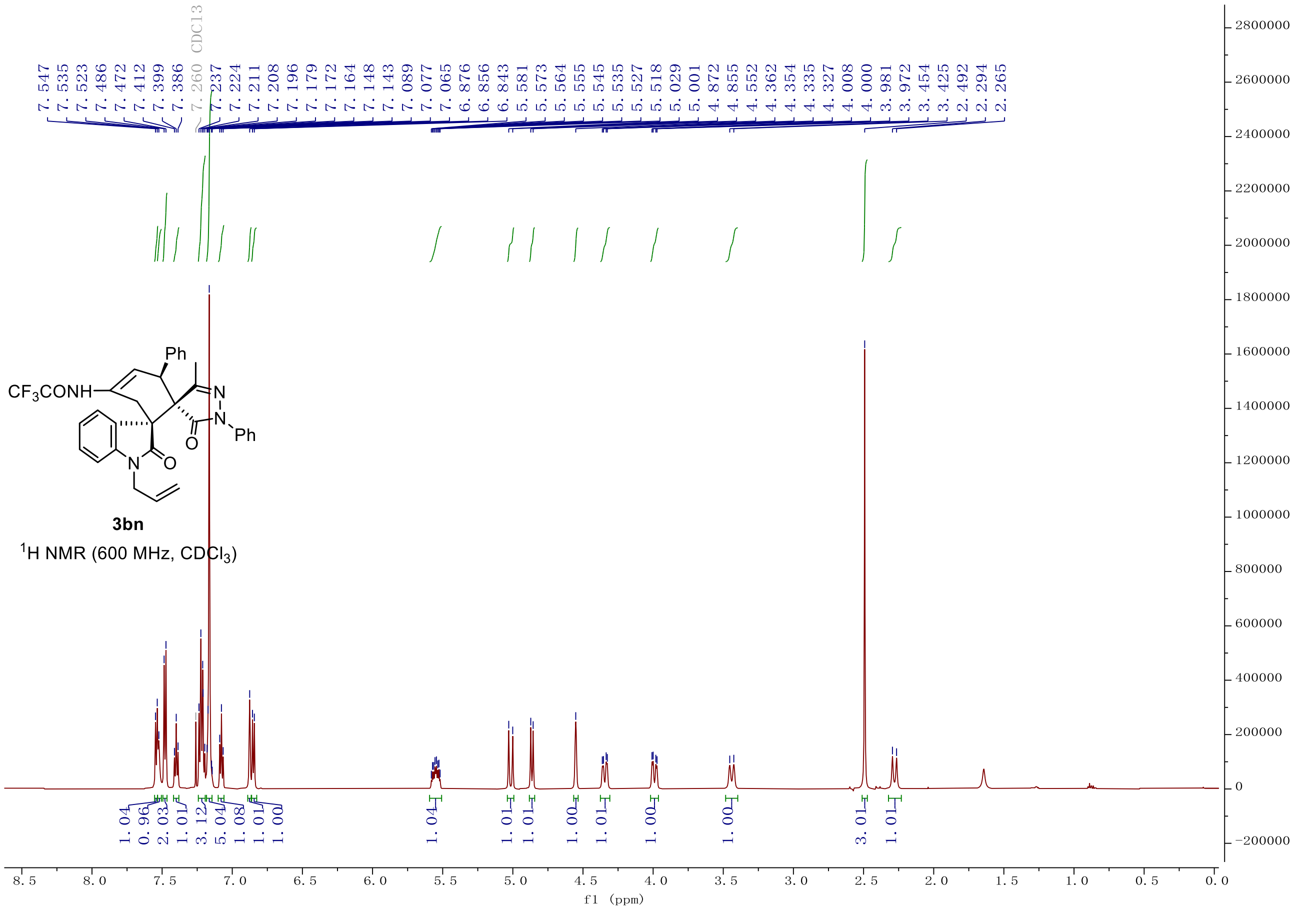

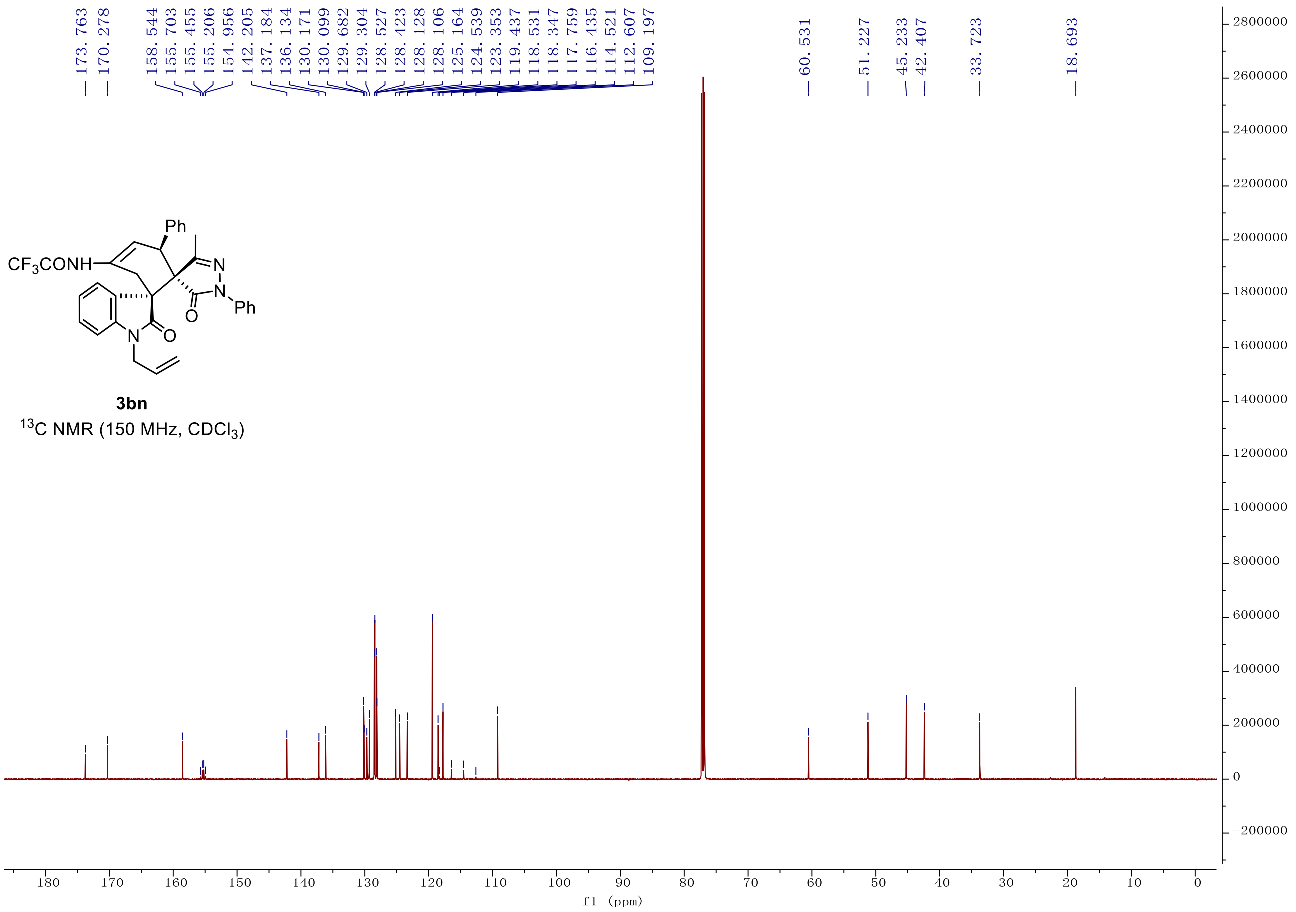

3bn

^{13}C NMR (150 MHz, $CDCl_3$)

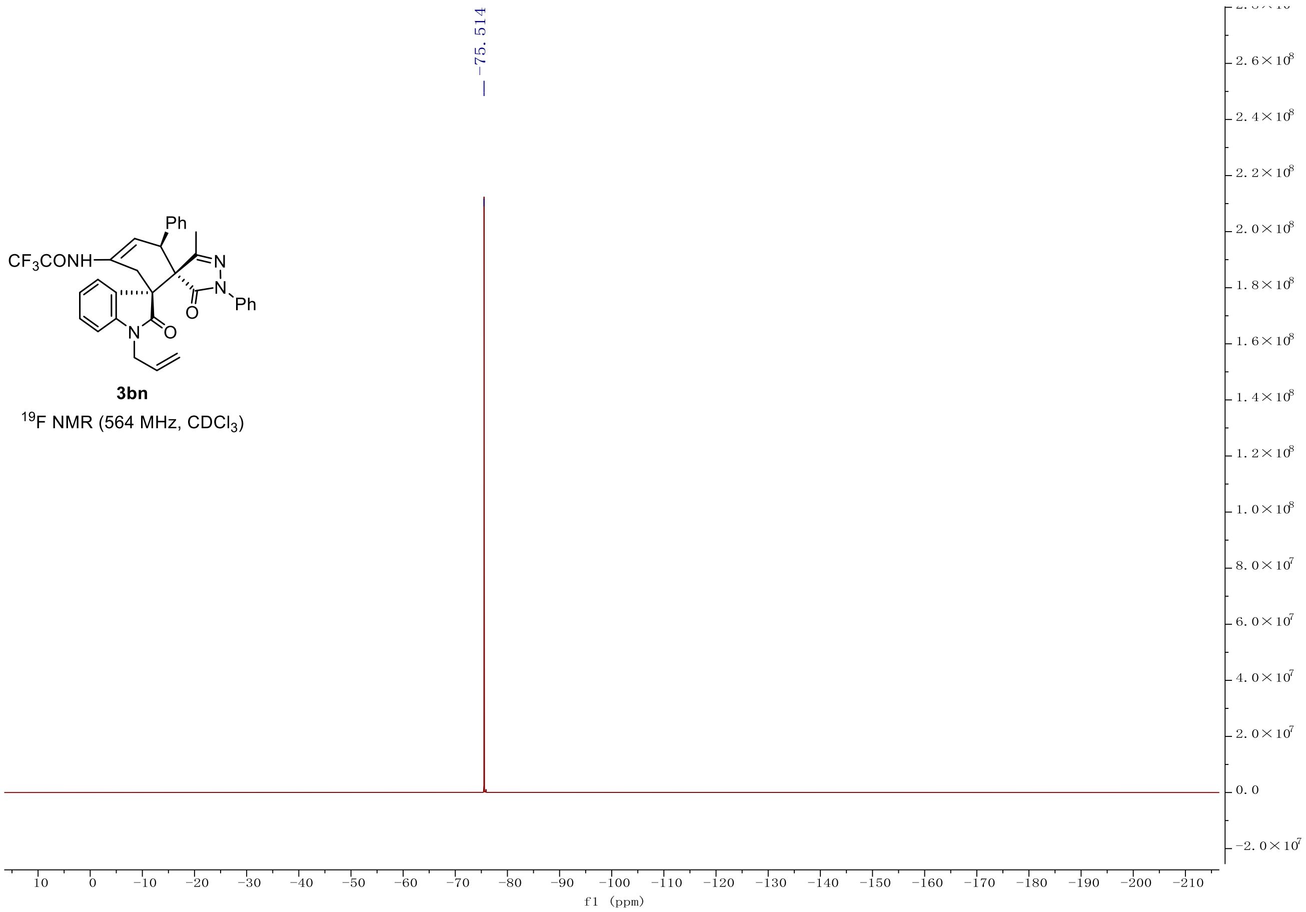

3bn

^{19}F NMR (564 MHz, $CDCl_3$)

Signal: VWD1 B, Wavelength=254 nm

RT [min]Type	Area	Width[min]	Area%
25.333	1902.6930	1.120	50.3155
27.863	1878.8325	1.660	49.6845
总和	3781.5255		

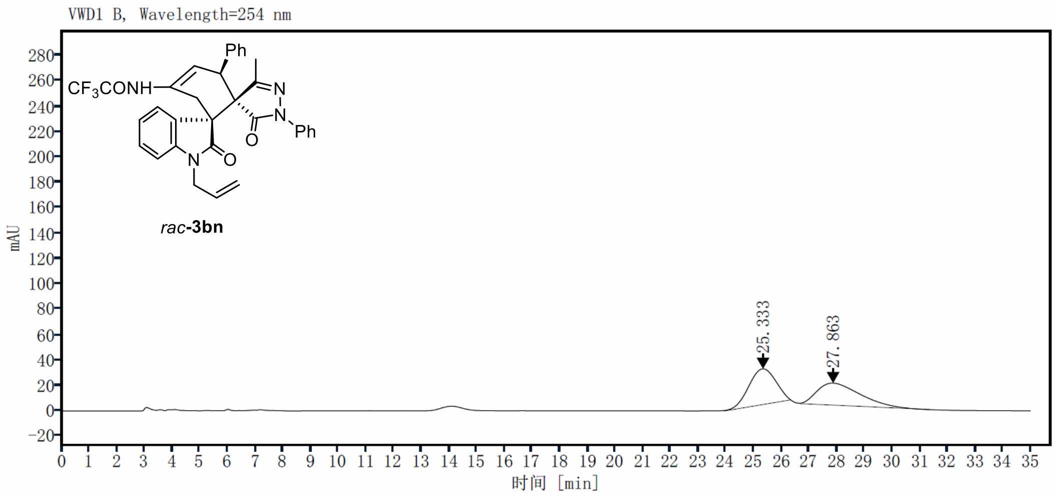

Signal: VWD1 B, Wavelength=254 nm

RT [min]Type	Area	Width[min]	Area%
24.992	27276.3457	1.080	99.5395
27.835	126.1939	0.450	0.4605
总和	27402.5396		

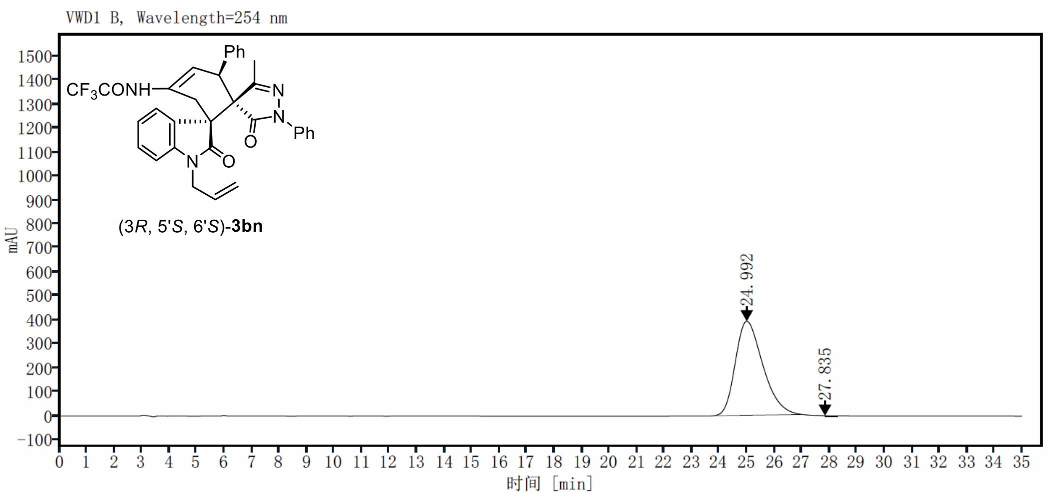

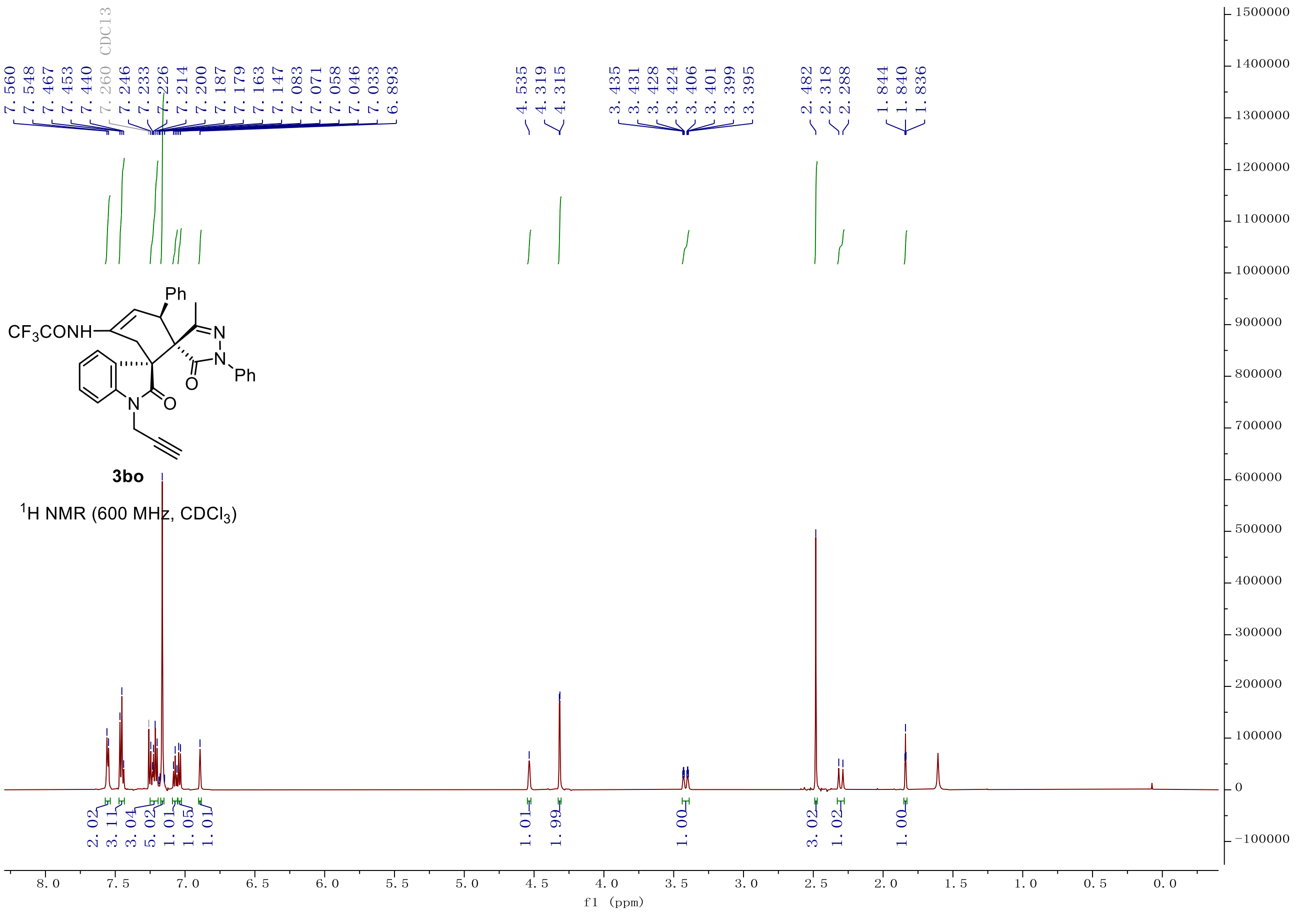

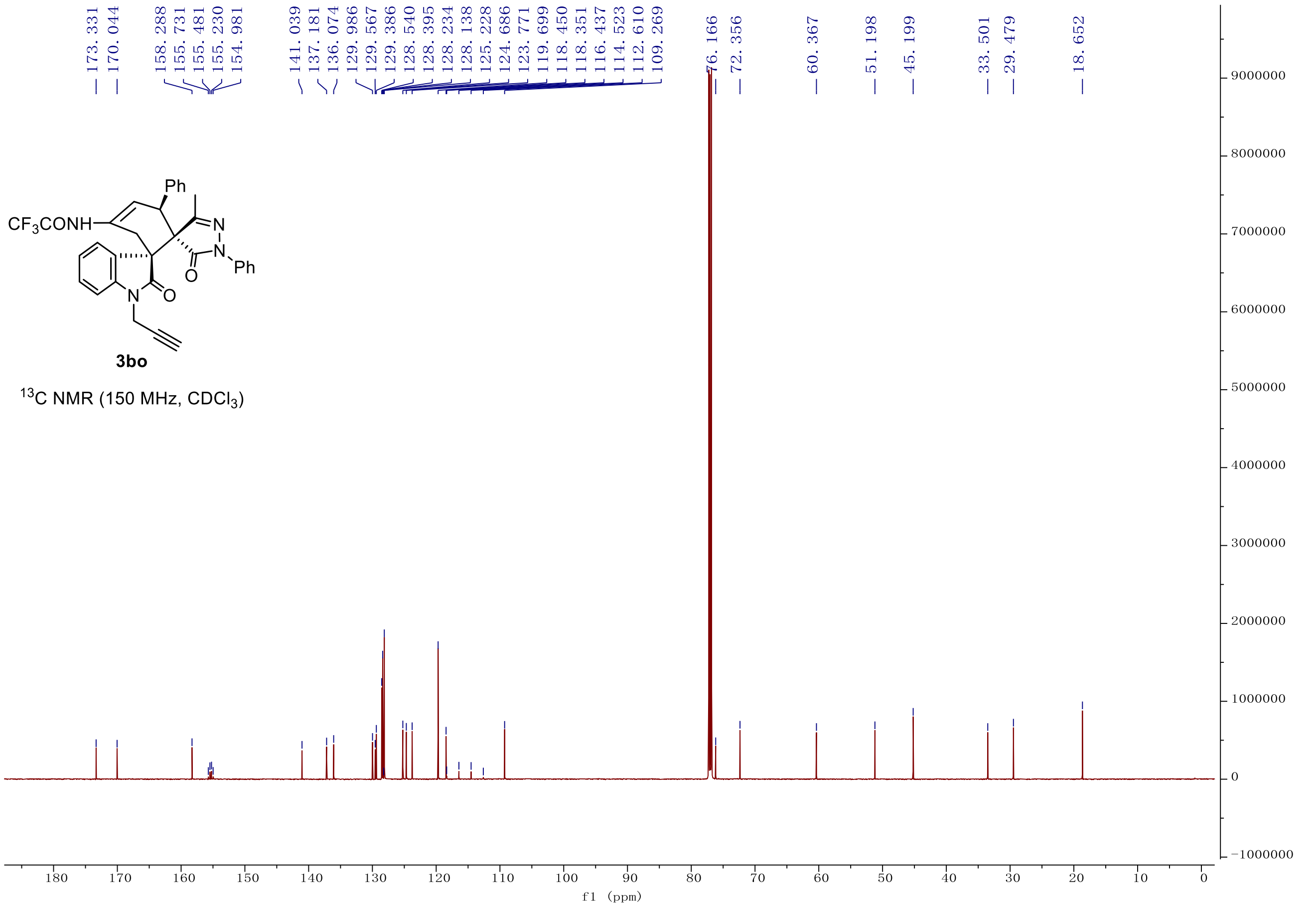
173.331
170.044
158.288
155.731
155.481
155.230
154.981
141.039
137.181
136.074
129.986
129.567
129.386
128.540
128.395
128.234
128.138
125.228
124.686
123.771
119.699
118.450
118.351
116.437
114.523
112.610
109.269
76.166
72.356
60.367
51.198
45.199
33.501
29.479
18.652
CF3CONH
Ph
N
Ph
O
N
O
3bo
13C NMR (150 MHz, CDCl3)
f1 (ppm)

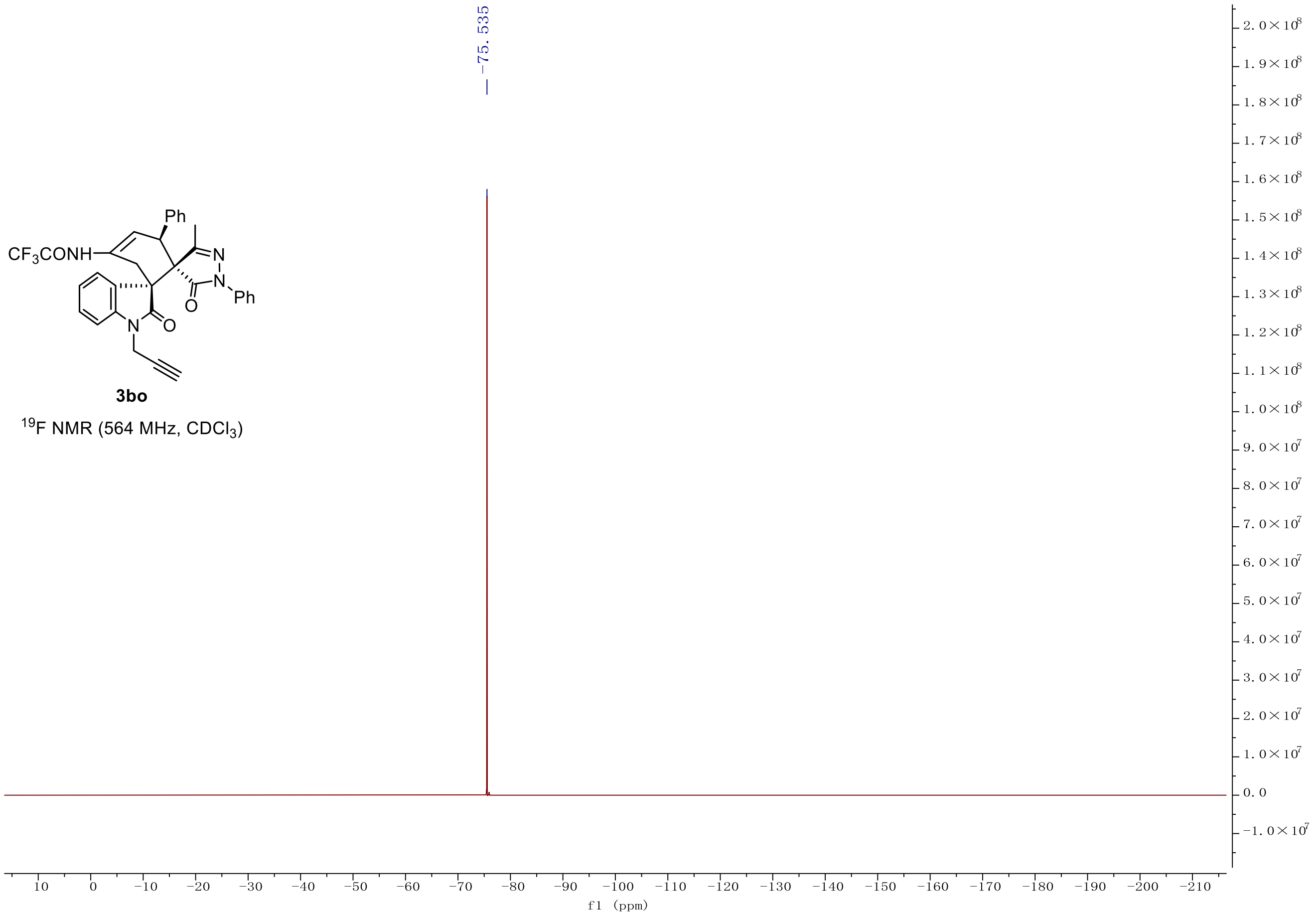
-75.535
CF3CONH
Ph
N
Ph
O
N
O
3bo
19F NMR (564 MHz, CDCl3)
f1 (ppm)

Peak Analysis Report

Detector A Channel 1 254nm

No.	Ret. Time	Height (mAu)	Area (mAu*min)	Rel. Area (%)
1	7.163	97911	2380580	50.503
2	8.692	55773	2333189	49.497
Total		153685	4713770	100.000

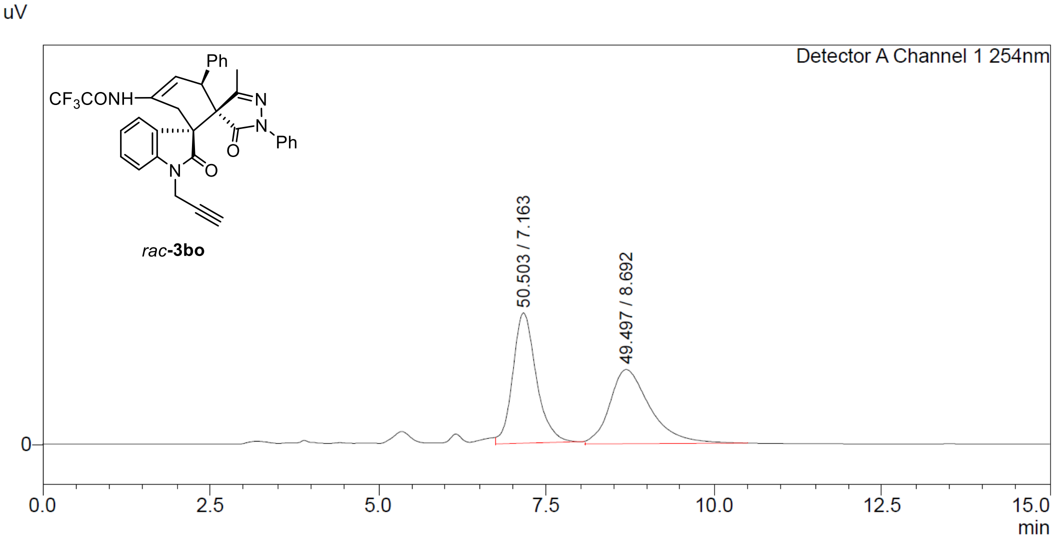

Peak Analysis Report

Detector A Channel 1 254nm

No.	Ret. Time	Height (mAu)	Area (mAu*min)	Rel. Area (%)
1	7.121	1114452	24829098	99.841
2	8.563	2024	39433	0.159
Total		1116476	24868531	100.000

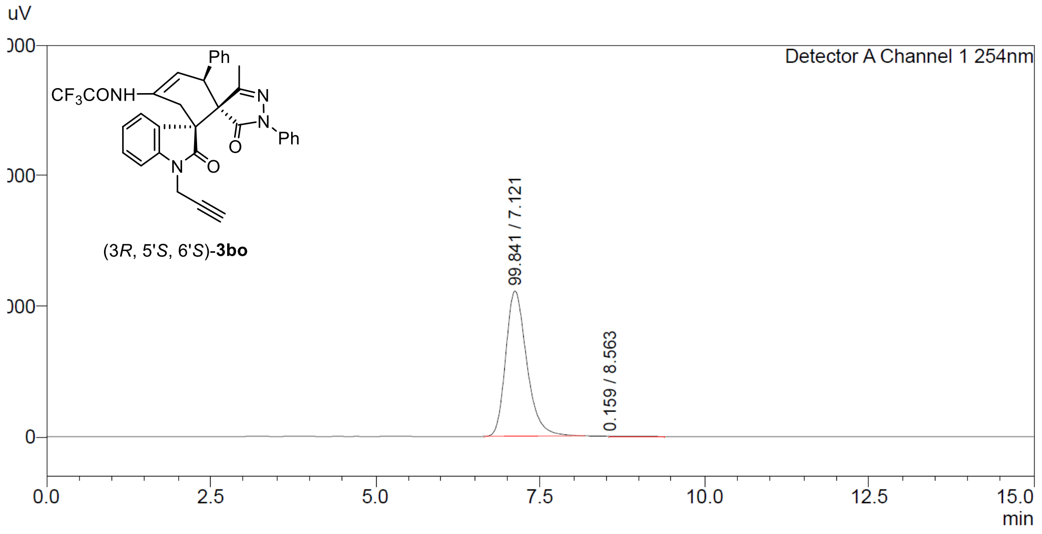

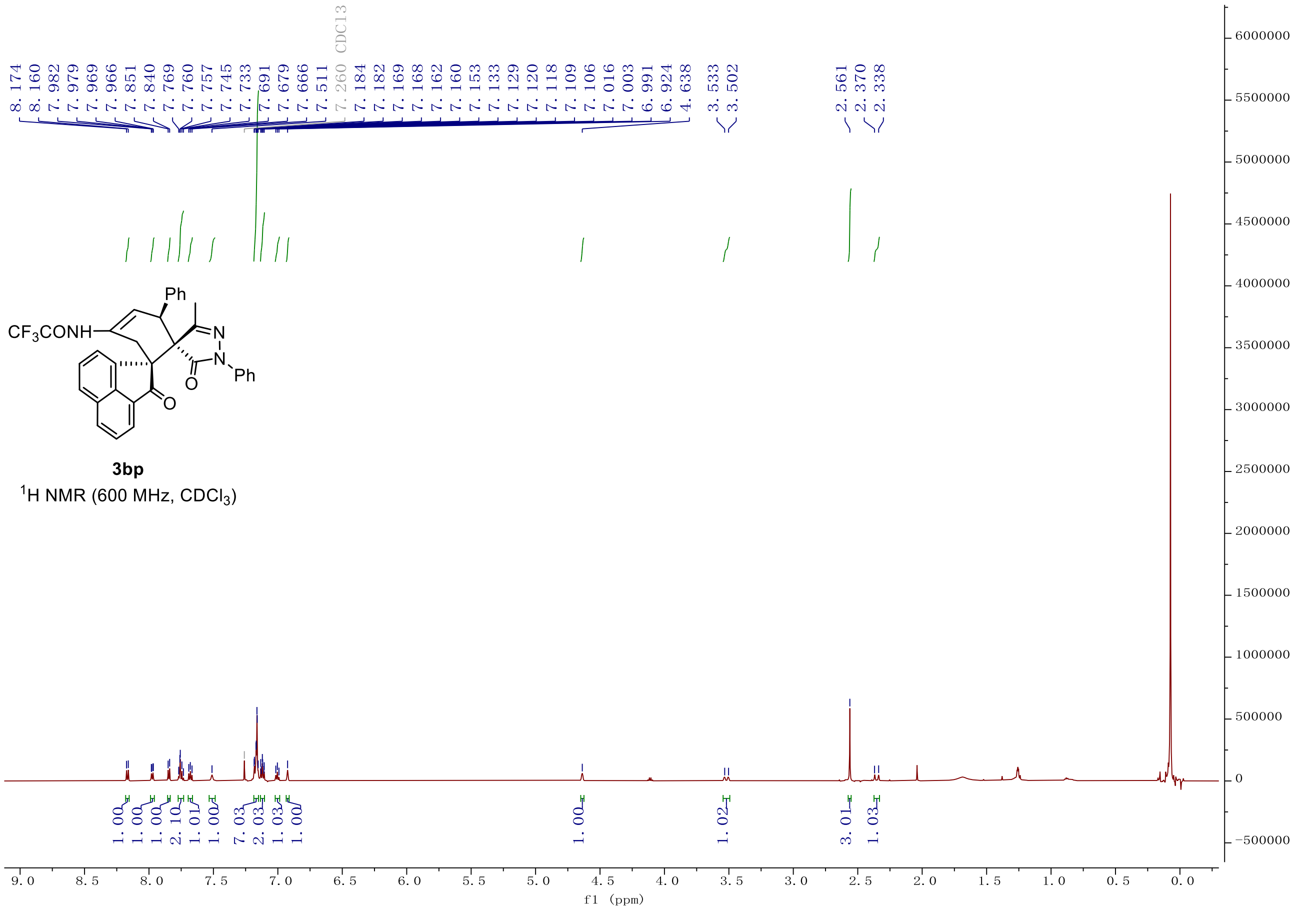

3bp

^1H NMR (600 MHz, $CDCl_3$)

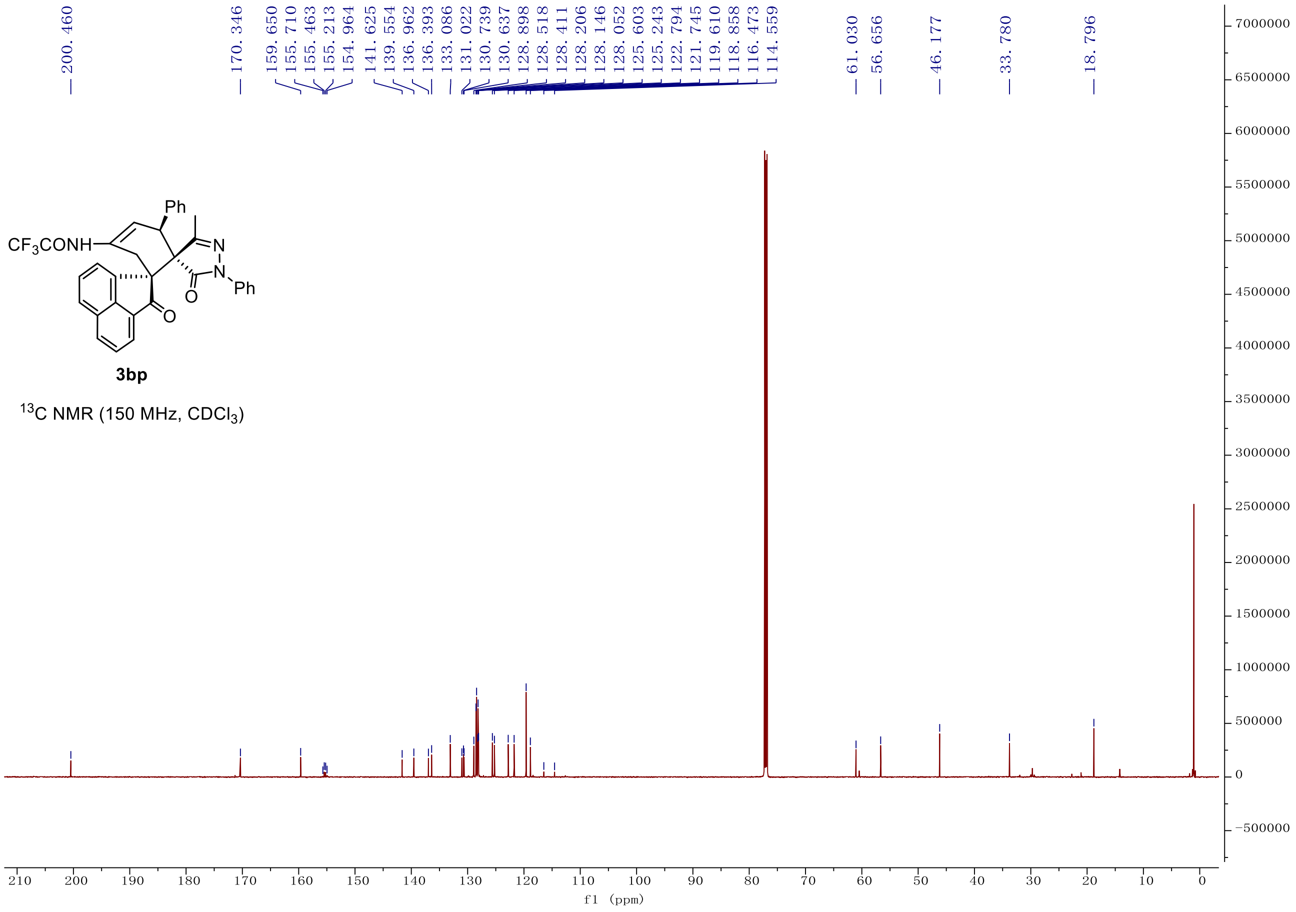

3bp

^{13}C NMR (150 MHz, $CDCl_3$)

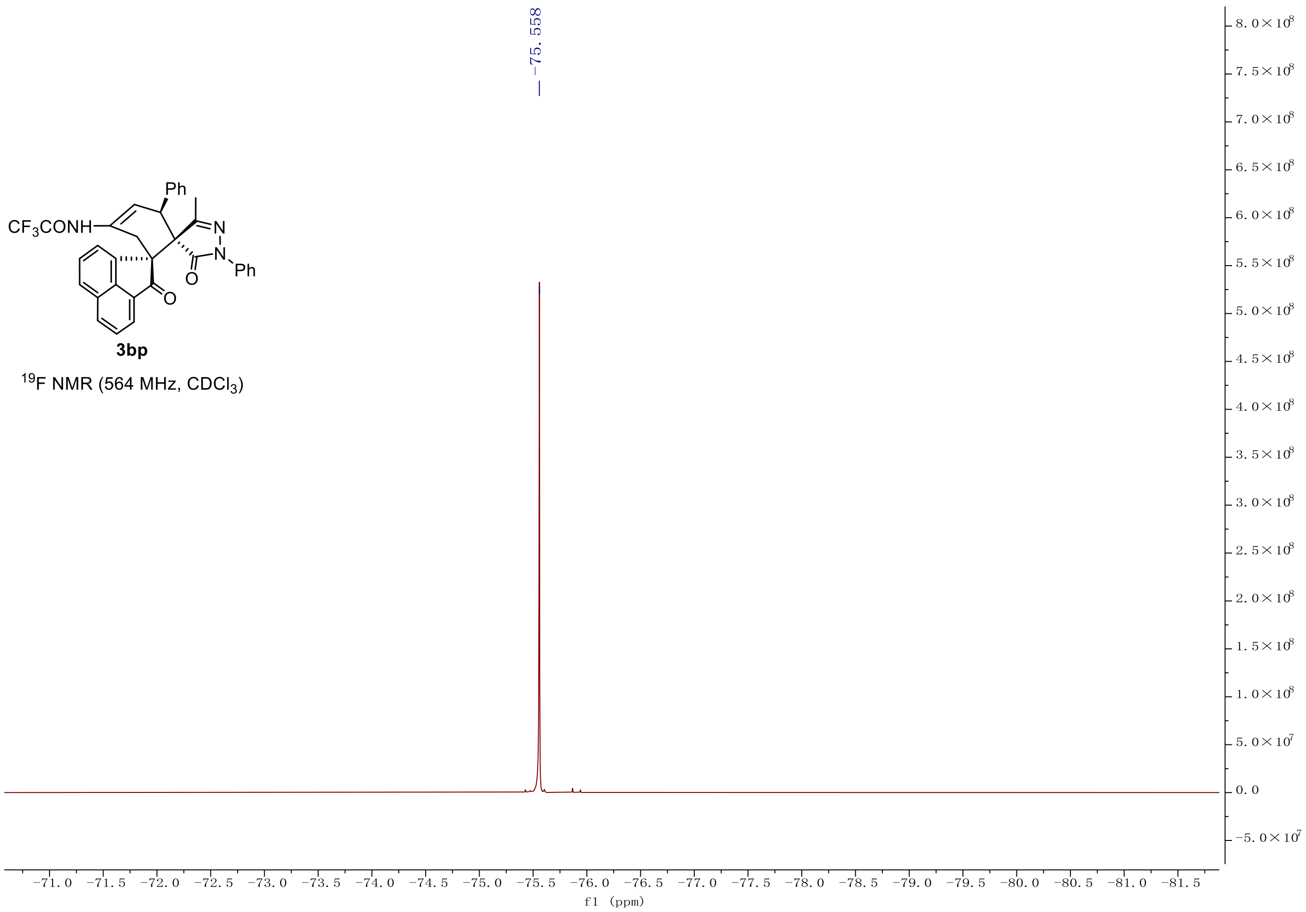

Signal: VWD1 B, Wavelength=254 nm

RT [min]Type	Area	Width[min]	Area%
8.788	9700.7061	0.760	49.3572
16.608	9953.3828	1.733	50.6428
总和	19654.0889		

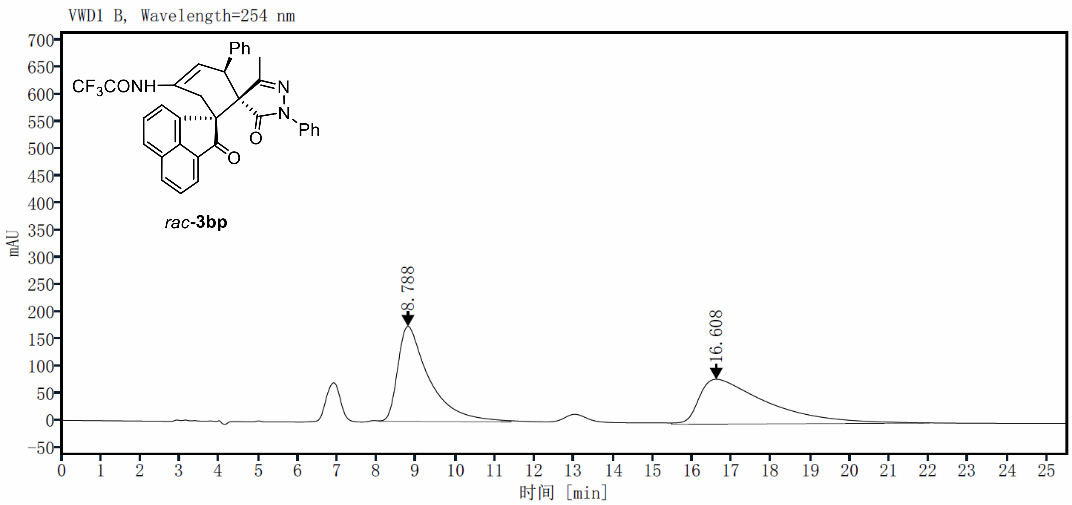

Signal: VWD1 B, Wavelength=254 nm

RT [min]Type	Area	Width[min]	Area%
8.499	23057.4297	0.629	99.5896
16.649	95.0196	0.392	0.4104
总和	23152.4493		

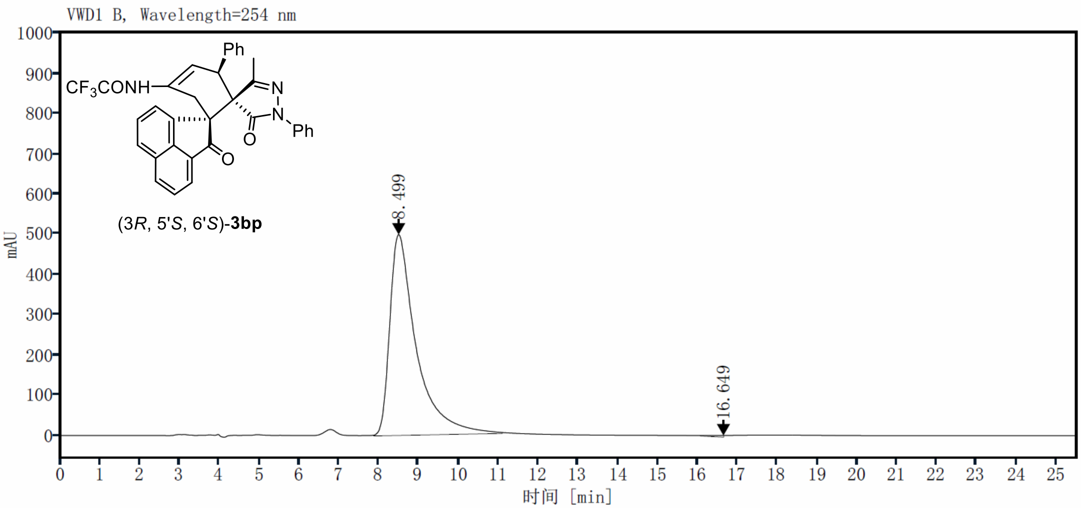

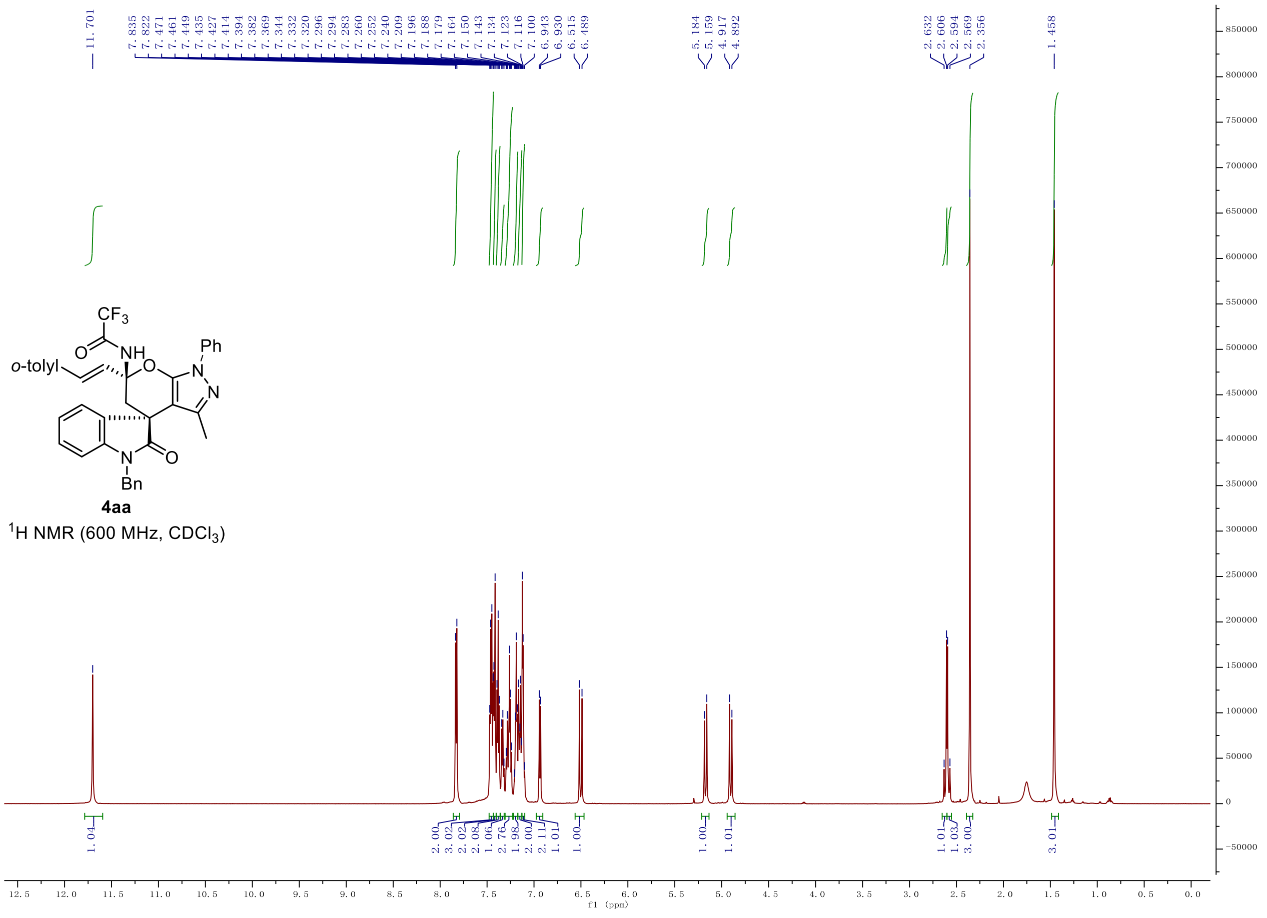

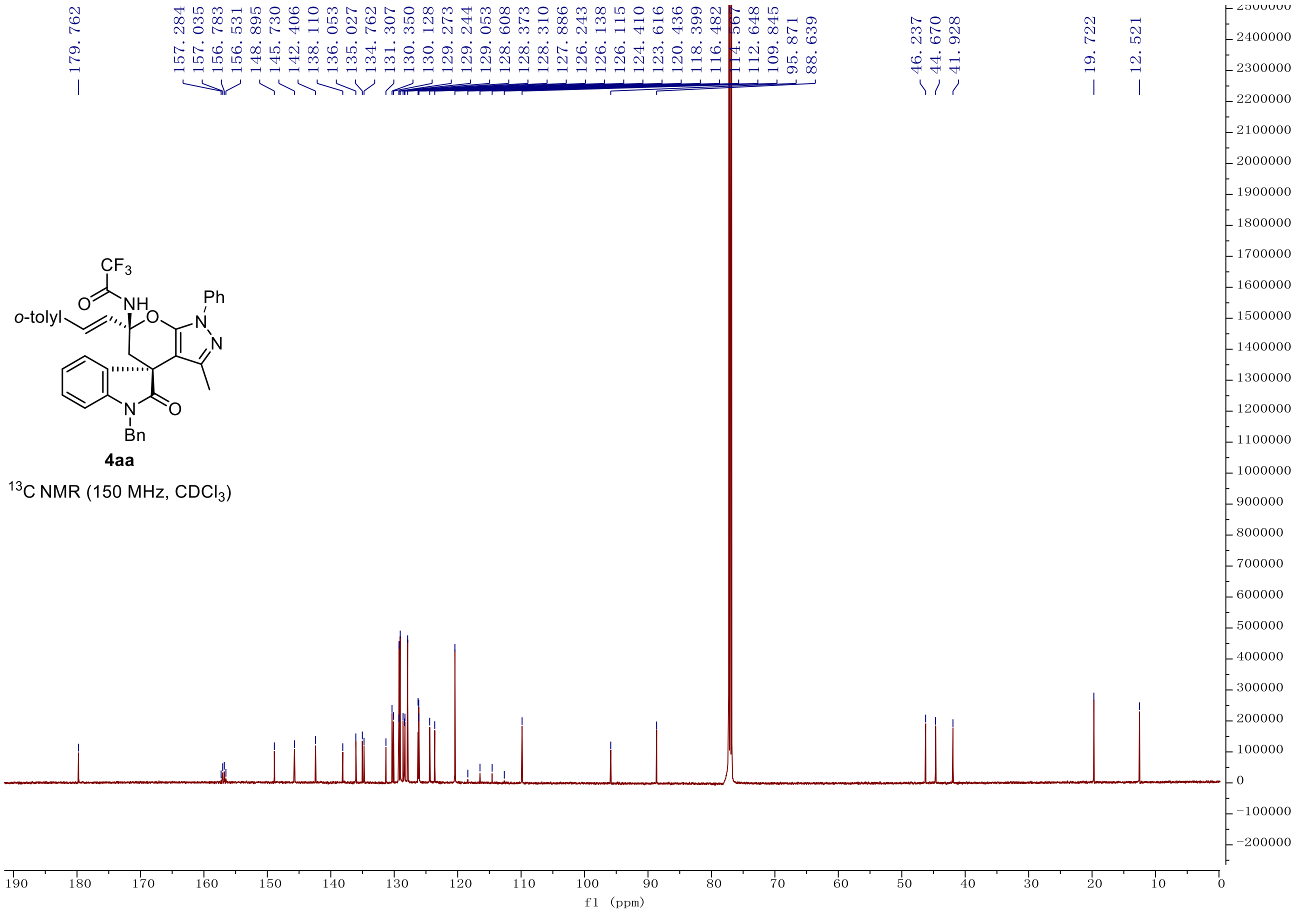

^{13}C NMR (150 MHz, $CDCl_3$)

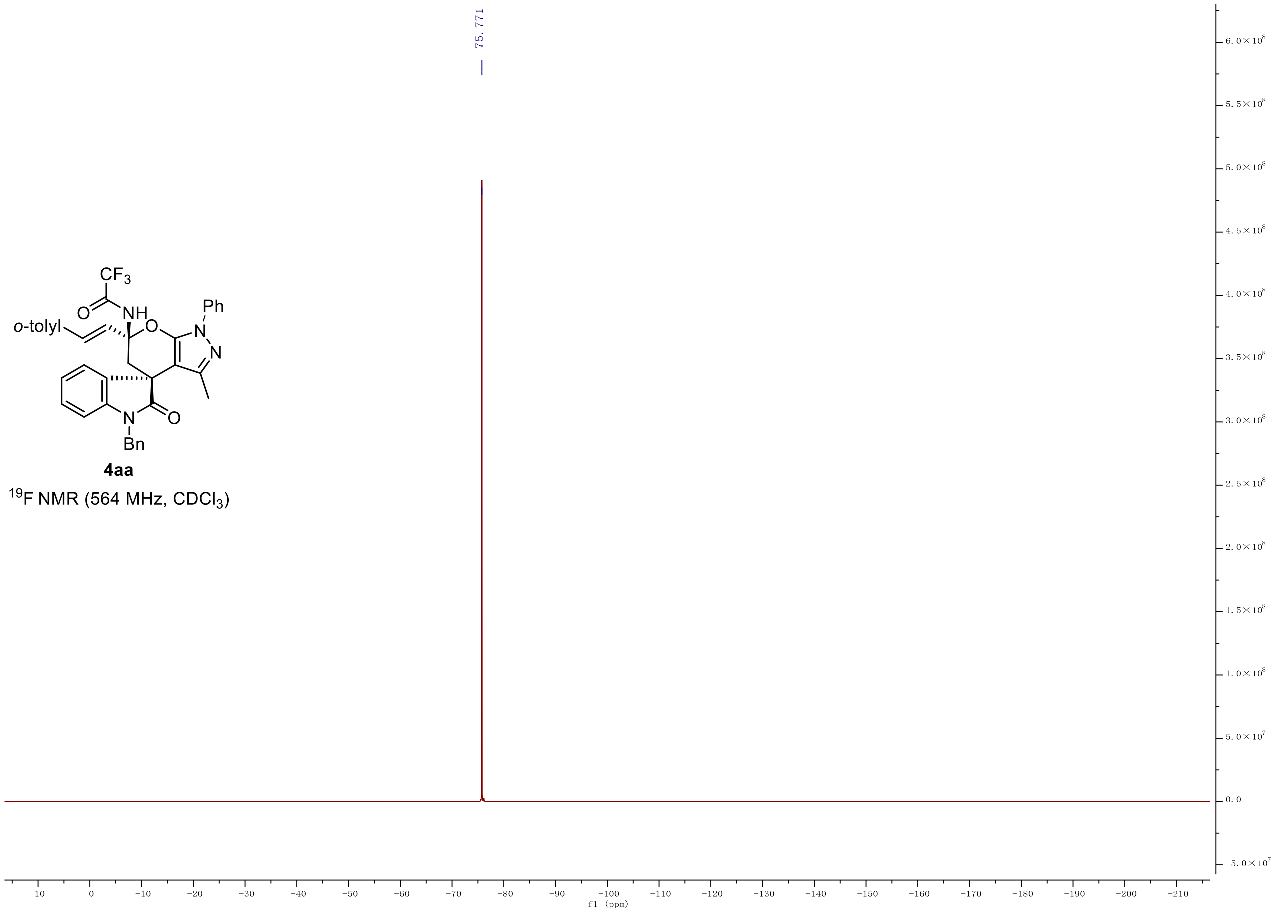

^{19}F NMR (564 MHz, $CDCl_3$)

Peak Analysis Report

Detector A Channel 1 254nm

No.	Ret. Time	Height (mAu)	Area (mAu*min)	Rel. Area (%)
1	9.841	931272	19079461	50.192
2	12.149	718471	18933849	49.808
Total		1649743	38013310	100.000

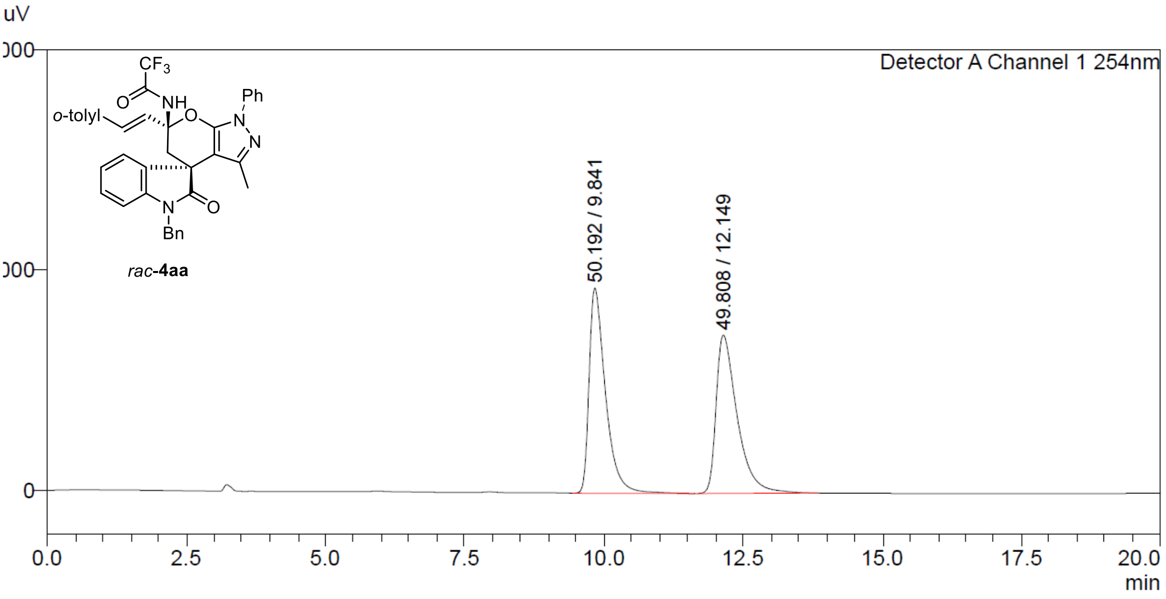

Peak Analysis Report

Detector A Channel 1 254nm

No.	Ret. Time	Height (mAu)	Area (mAu*min)	Rel. Area (%)
1	10.030	3442	46983	0.112
2	12.055	1529127	41845133	99.888
Total		1532569	41892116	100.000

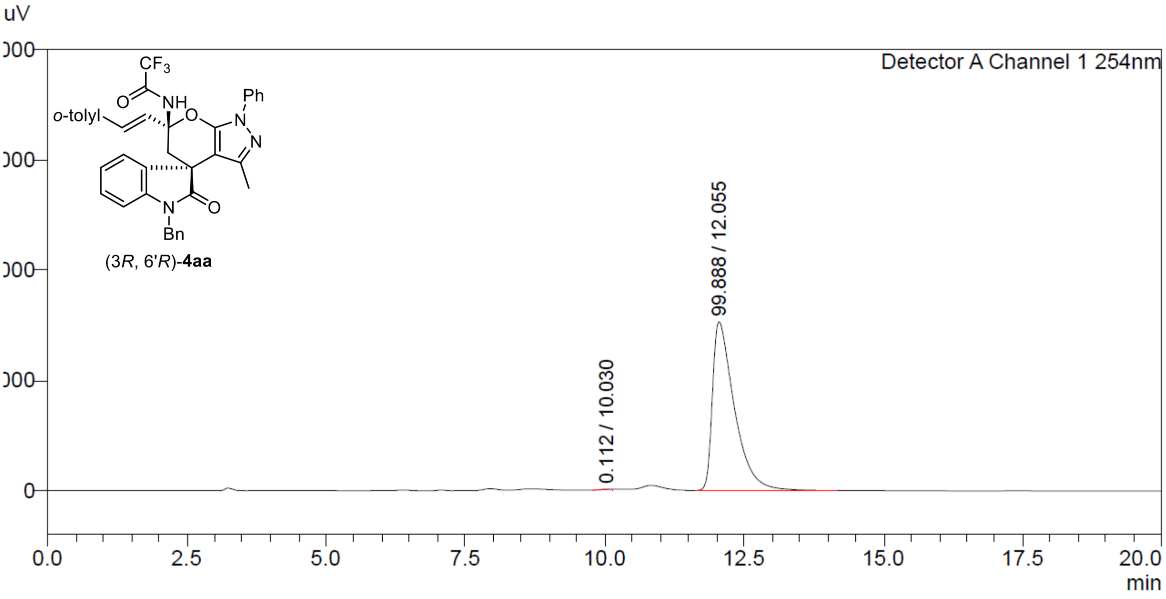

Peak Analysis Report

Detector A Channel 1 254nm

No.	Ret. Time	Height (mAu)	Area (mAu*min)	Rel. Area (%)
1	9.784	1405489	27714802	99.958
2	12.282	555	11517	0.042
Total		1406044	27726319	100.000

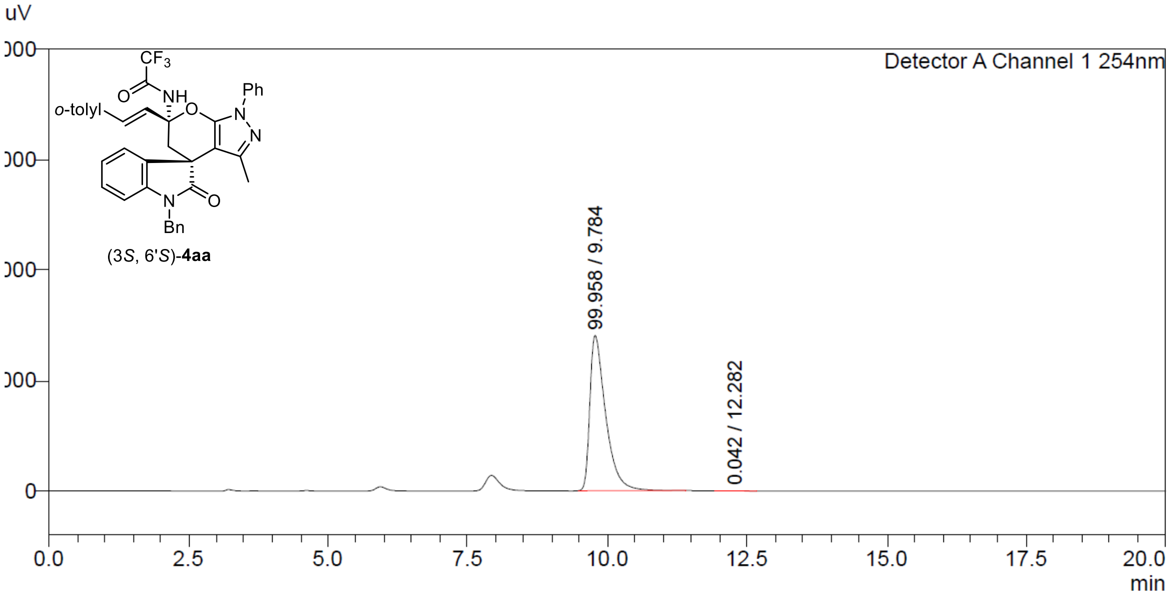

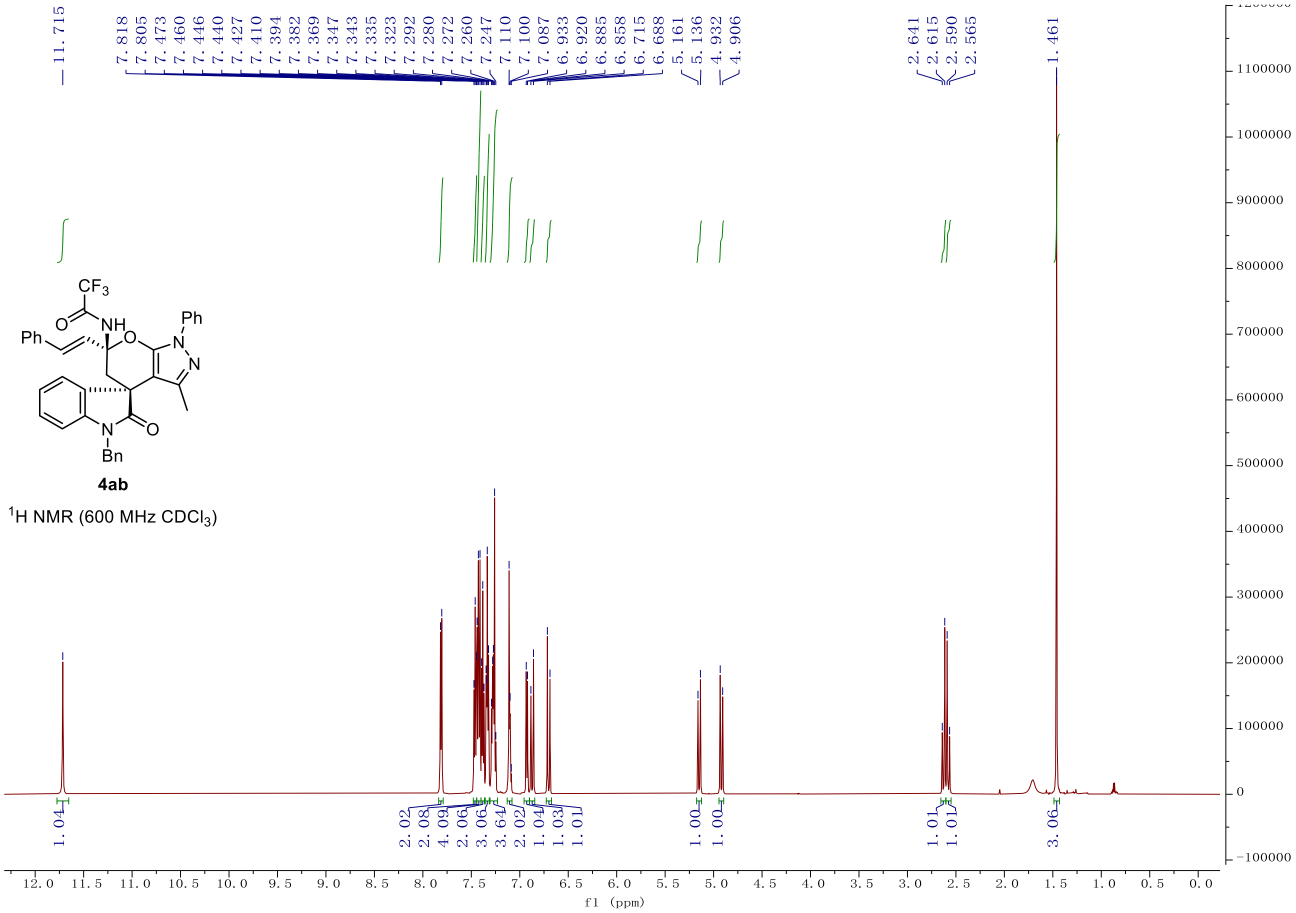

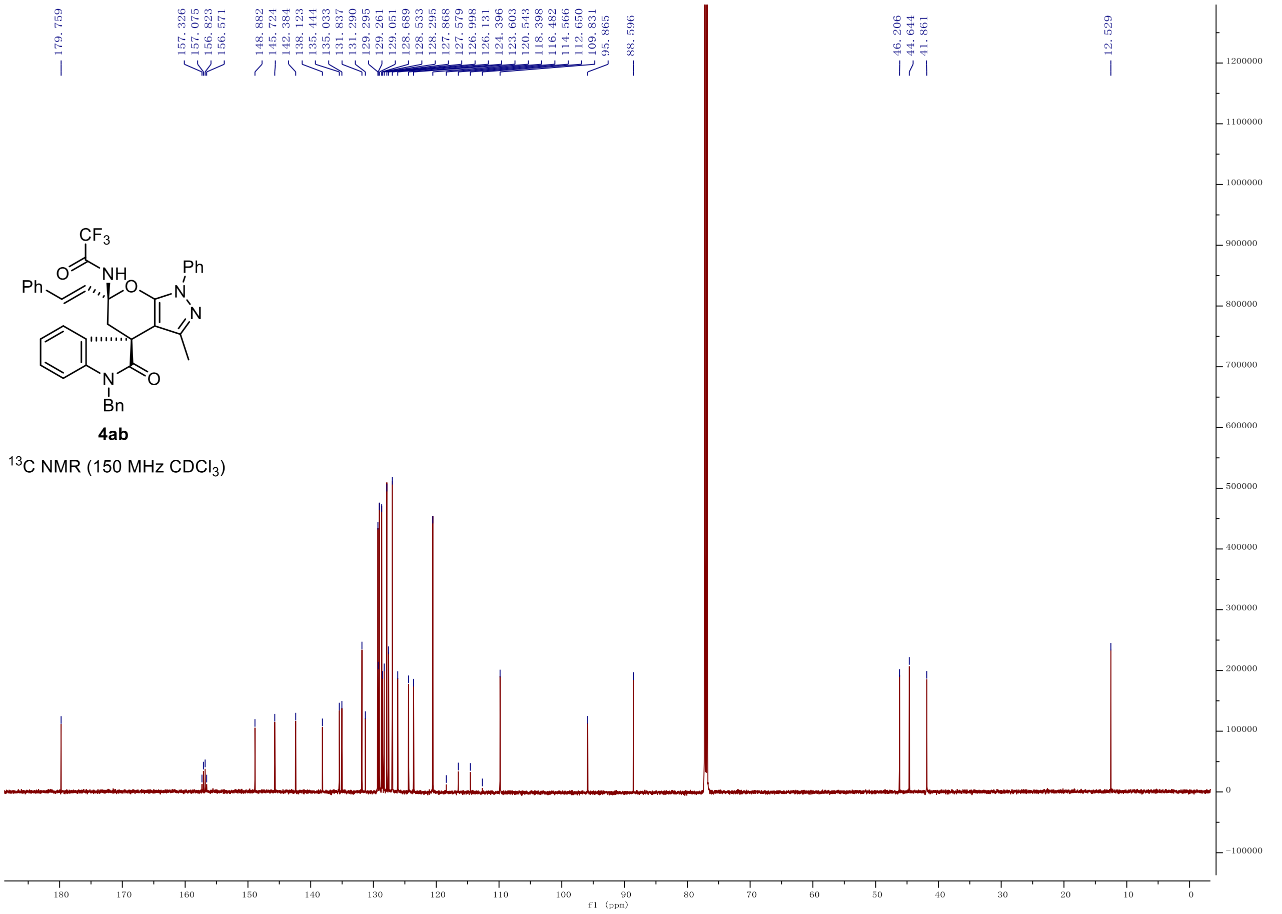
179.759
157.326
157.075
156.823
156.571
148.882
145.724
142.384
138.123
135.444
135.033
131.837
131.290
129.295
129.261
129.051
128.689
128.533
128.295
127.868
127.579
126.998
126.131
124.396
123.603
120.543
118.398
116.482
114.566
112.650
109.831
95.865
88.596
46.206
44.644
41.861
12.529
CF3
O
NH
Ph
O
N
N
Ph
N
O
Bn
4ab
^{13}C NMR (150 MHz CDCl$_3$)
f1 (ppm)

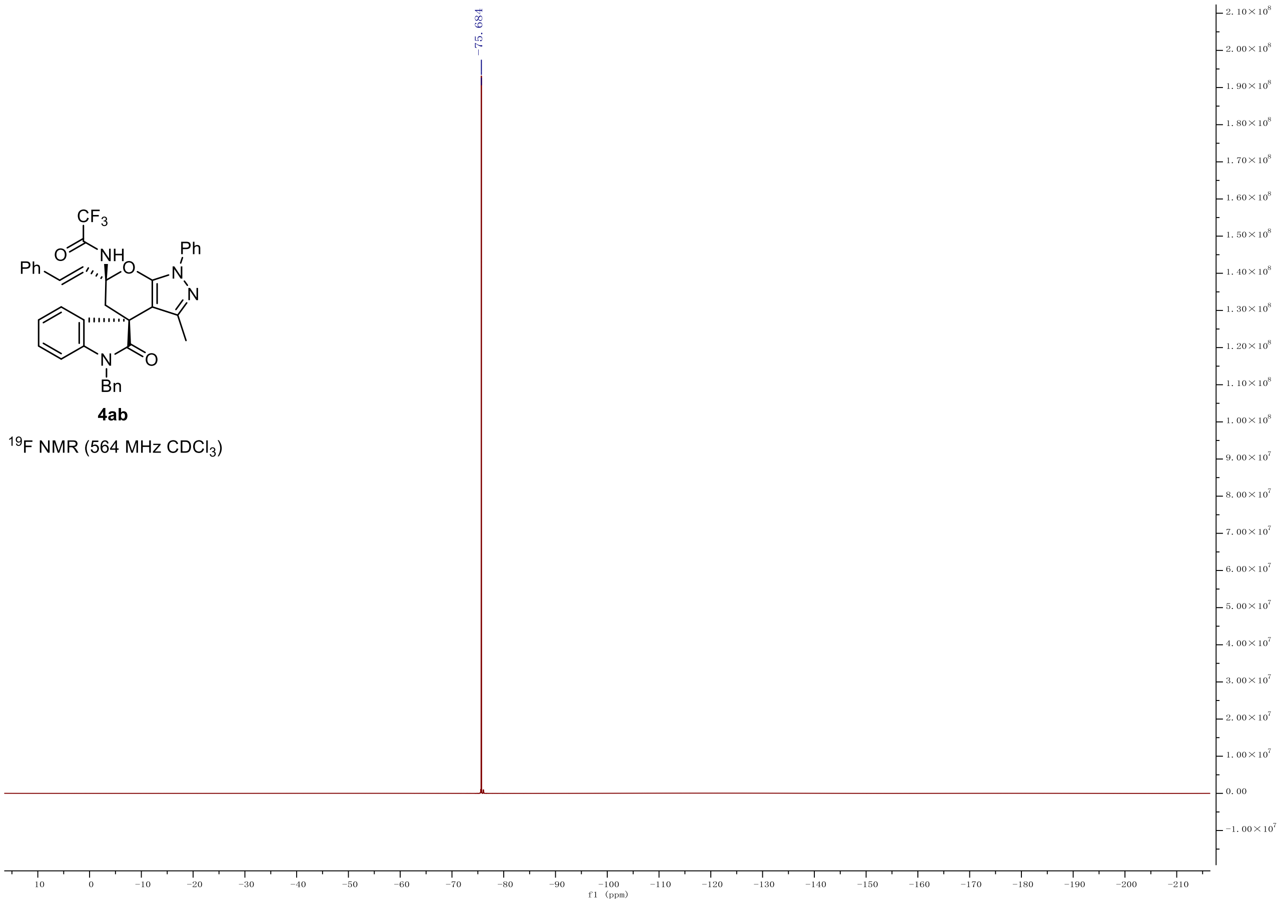
-75.684
CF3
O
NH
Ph
O
N
N
Ph
N
O
Bn
4ab
^{19}F NMR (564 MHz CDCl$_3$)
f1 (ppm)

Peak Analysis Report

Detector A Channel 1 254nm

No.	Ret. Time	Height (mAu)	Area (mAu*min)	Rel. Area (%)
1	7.156	893565	13431328	49.062
2	8.035	763936	13944675	50.938
Total		1657501	27376004	100.000

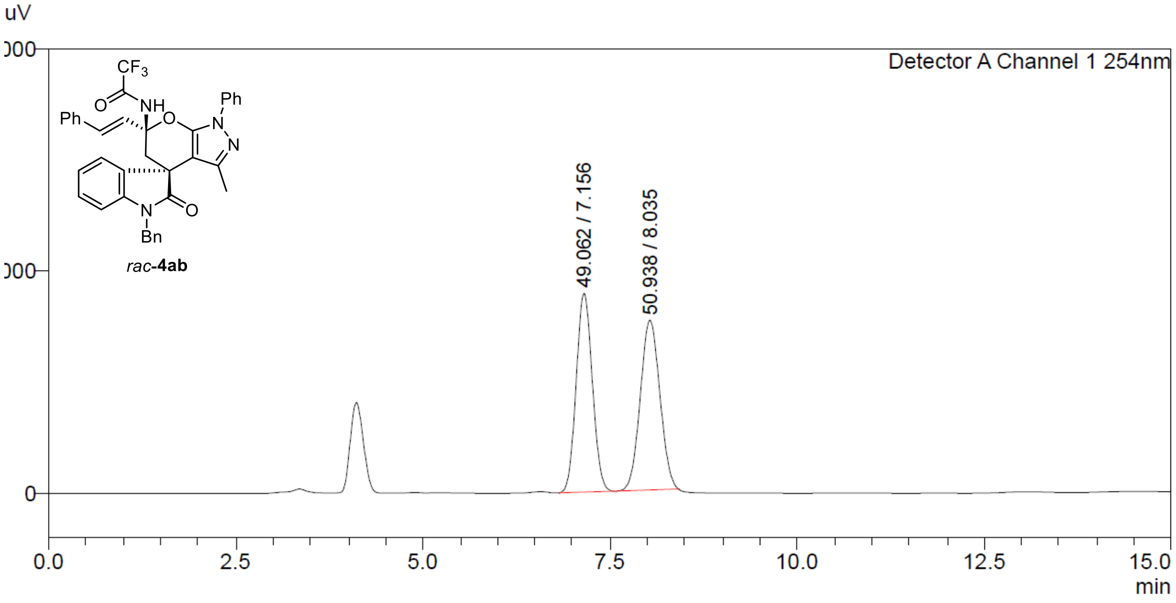

Peak Analysis Report

Detector A Channel 1 254nm

No.	Ret. Time	Height (mAu)	Area (mAu*min)	Rel. Area (%)
1	7.183	659586	9418814	98.514
2	8.063	8630	142107	1.486
Total		668216	9560921	100.000

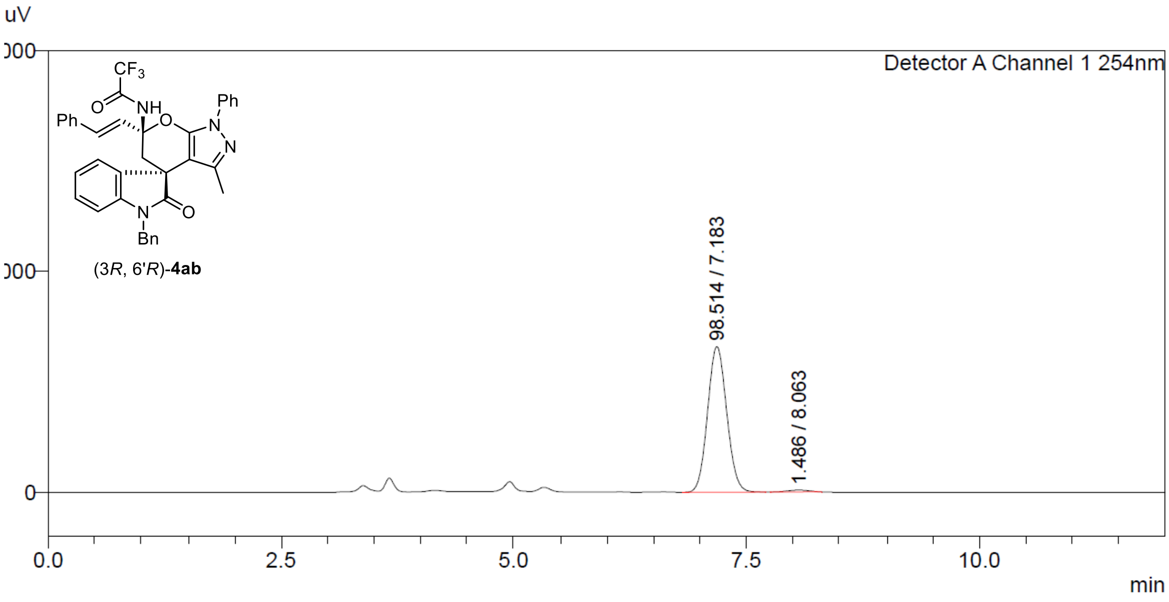

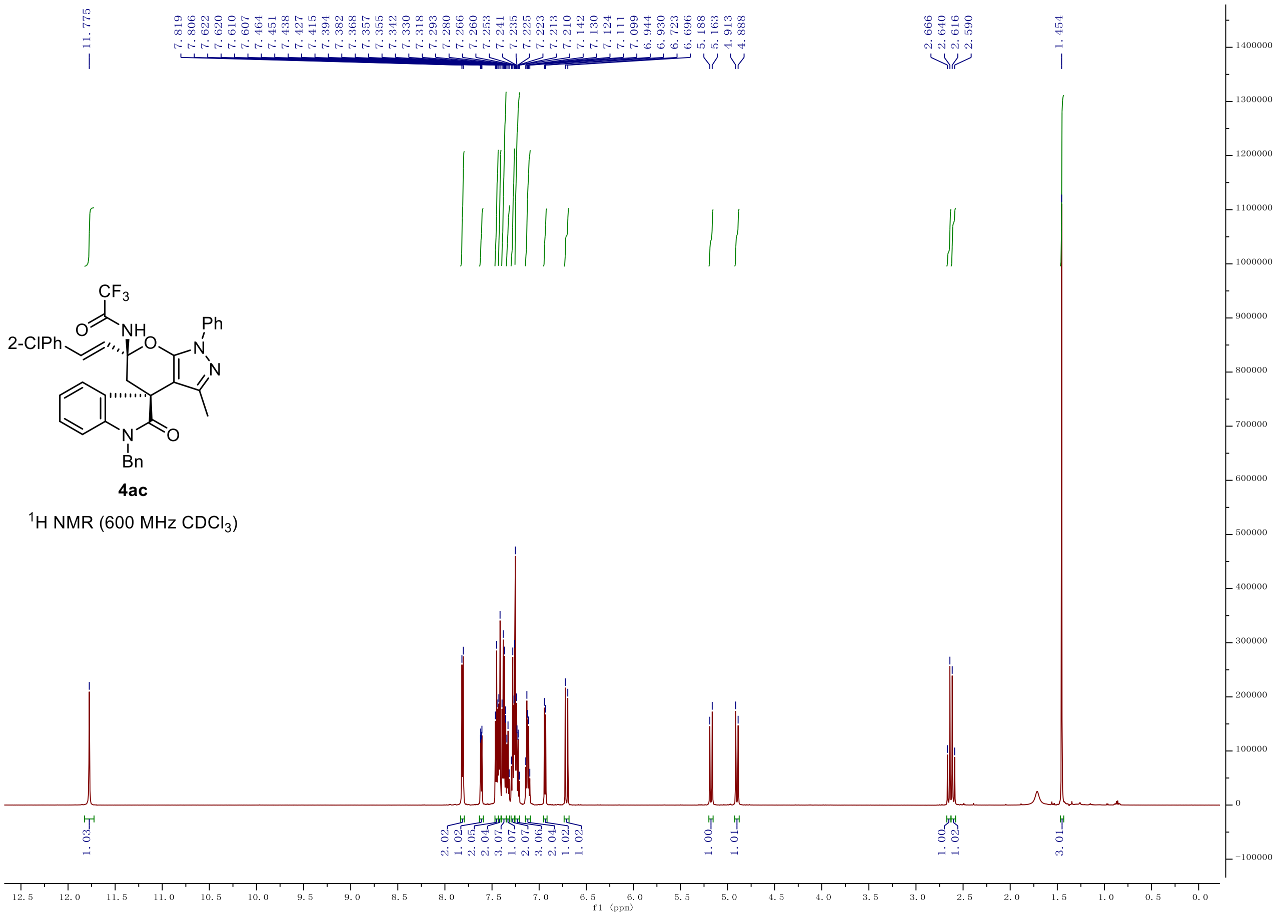
11.775
7.819 7.806 7.622 7.620 7.610 7.607 7.464 7.451 7.438 7.427 7.415 7.394 7.382 7.368 7.357 7.355 7.342 7.330 7.318 7.293 7.280 7.266 7.260 7.253 7.241 7.235 7.225 7.223 7.213 7.210 7.142 7.130 7.124 7.111 7.099 6.944 6.930 6.723 6.696
5.188 5.163 4.913 4.888
2.666 2.640 2.616 2.590
1.454
CF3
O
NH
Ph
2-ClPh
O
N
N
N
O
Bn
4ac
1H NMR (600 MHz CDCl3)
1.03 2.02 1.02 2.05 2.04 3.07 1.07 2.07 3.06 2.04 1.02 1.02 1.00 1.01 1.00 1.02 3.01
f1 (ppm)

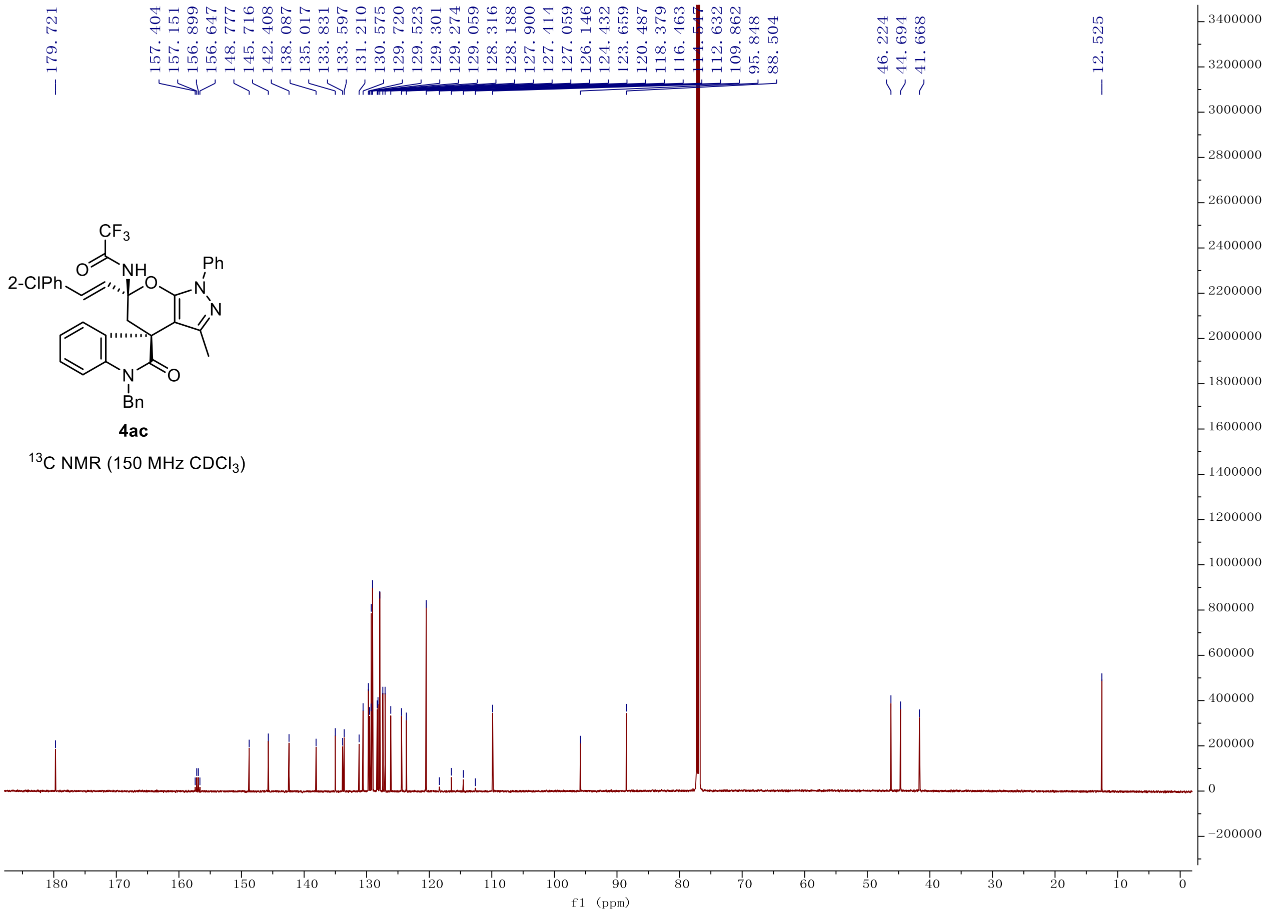
179.721
157.404 157.151 156.899 156.647 148.777 145.716 142.408 138.087 135.017 133.831 133.597 131.210 130.575 129.720 129.523 129.301 129.274 129.059 128.316 128.188 127.900 127.414 127.059 126.146 124.432 123.659 120.487 118.379 116.463 112.632 109.862 95.848 88.504
46.224 44.694 41.668
12.525
CF3
O
NH
Ph
2-ClPh
O
N
N
N
O
Bn
4ac
13C NMR (150 MHz CDCl3)
f1 (ppm)

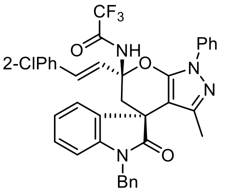

4ac

^{19}F NMR (564 MHz $CDCl_3$)

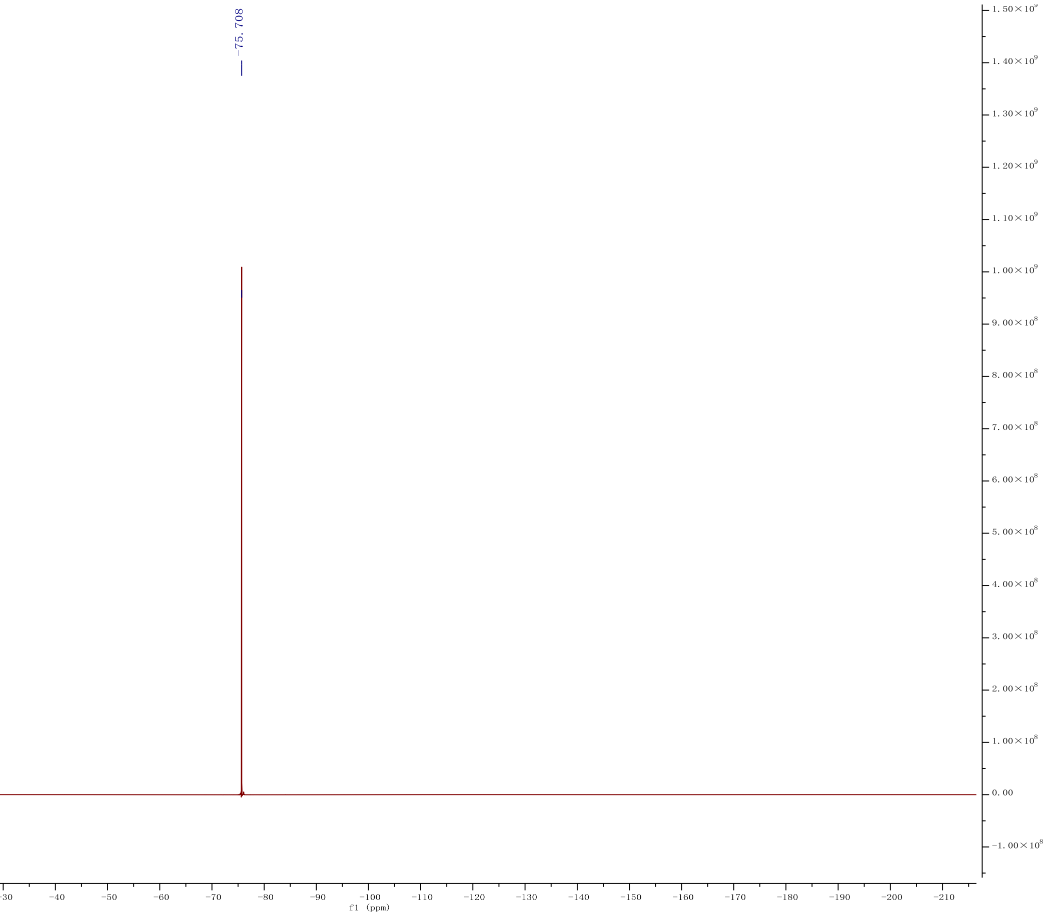

Peak Analysis Report

Detector A Channel 1 254nm

No.	Ret. Time	Height (mAu)	Area (mAu*min)	Rel. Area (%)
1	5.564	434816	4258877	16.488
2	5.937	439231	4306776	16.673
3	7.313	623960	8719568	33.756
4	8.442	517999	8545695	33.083
Total		2016006	25830916	100.000

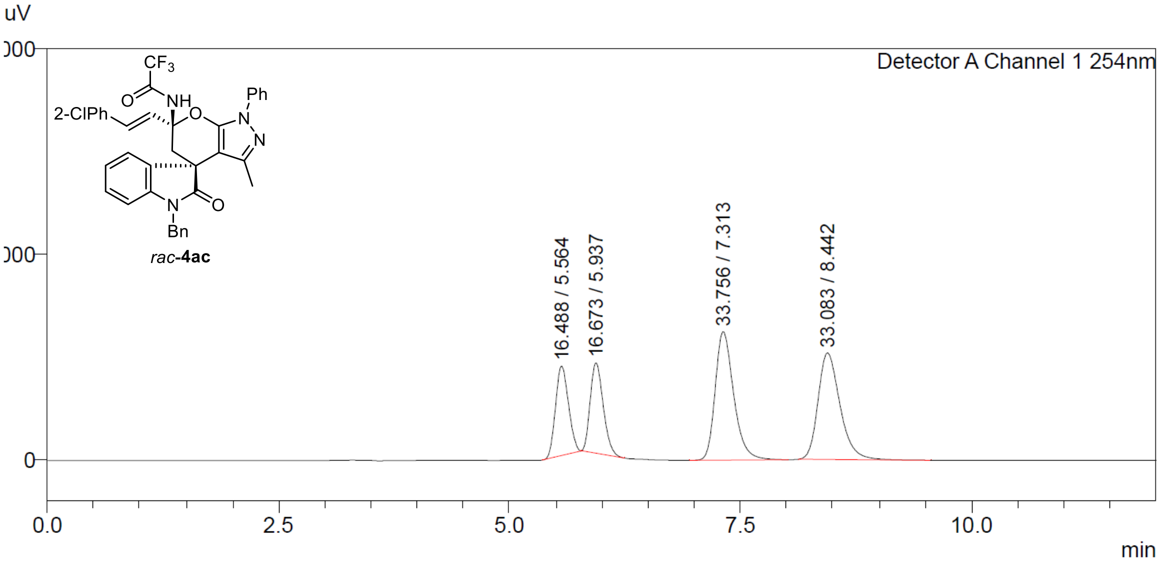

Peak Analysis Report

Detector A Channel 1 254nm

No.	Ret. Time	Height (mAu)	Area (mAu*min)	Rel. Area (%)
1	7.386	3952	53025	0.155
2	8.443	2028734	34184271	99.845
Total		2032686	34237297	100.000

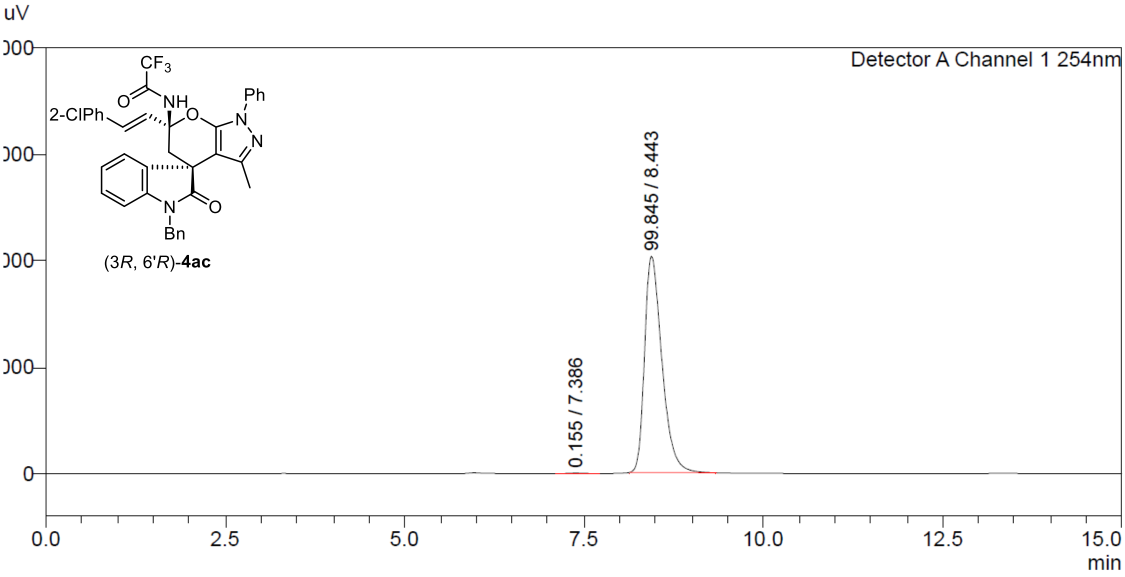

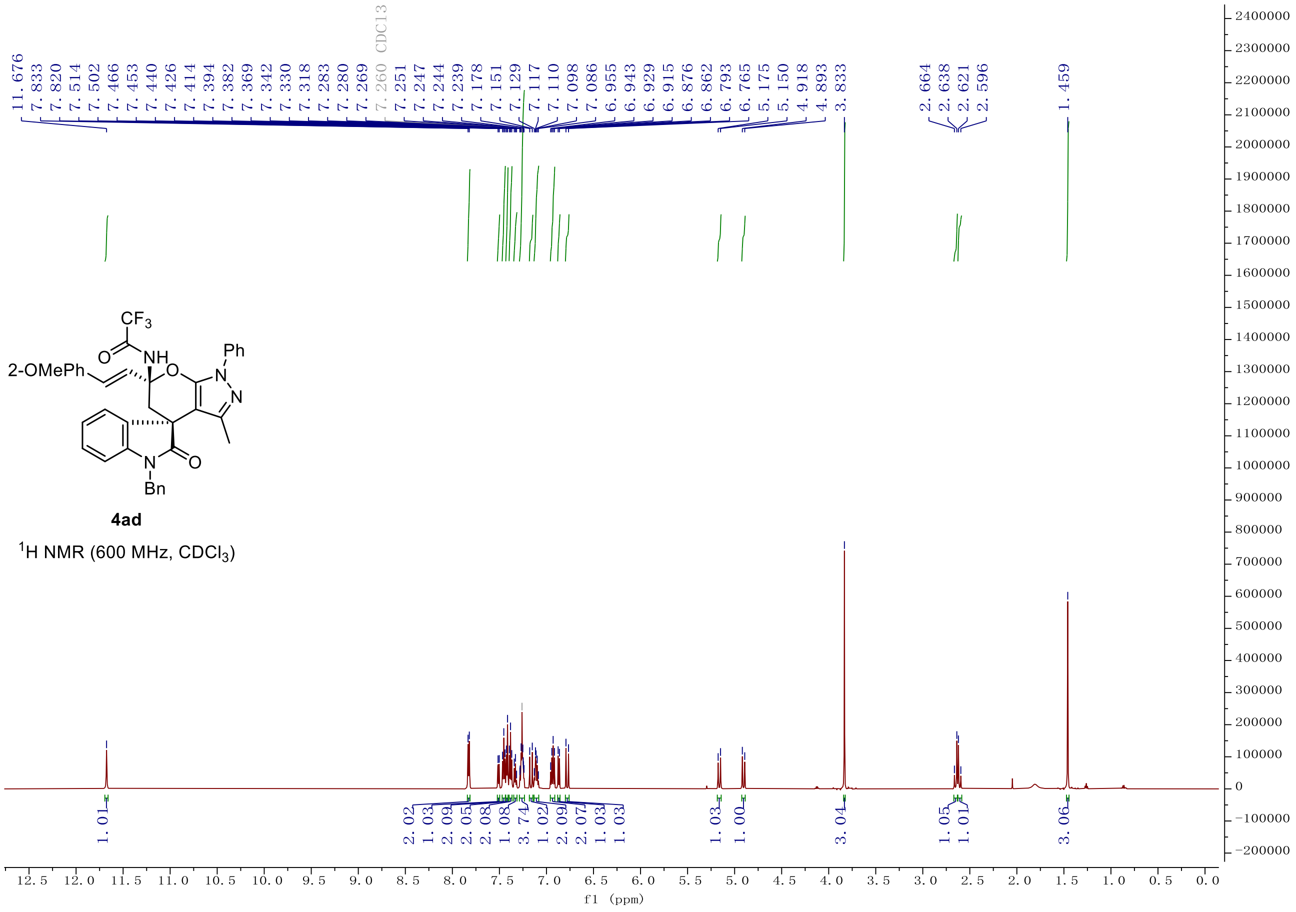

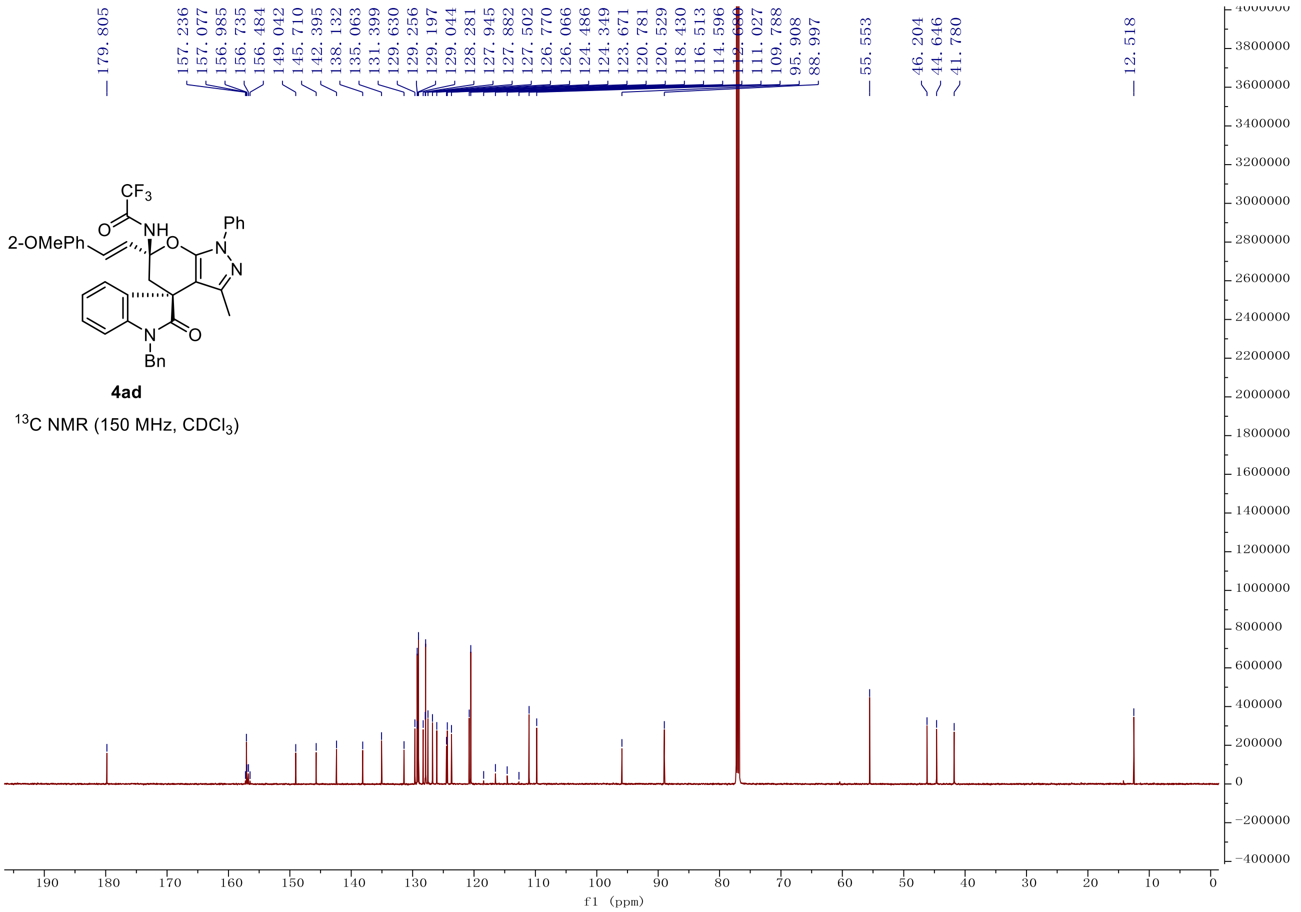

4ad

^{13}C NMR (150 MHz, $CDCl_3$)

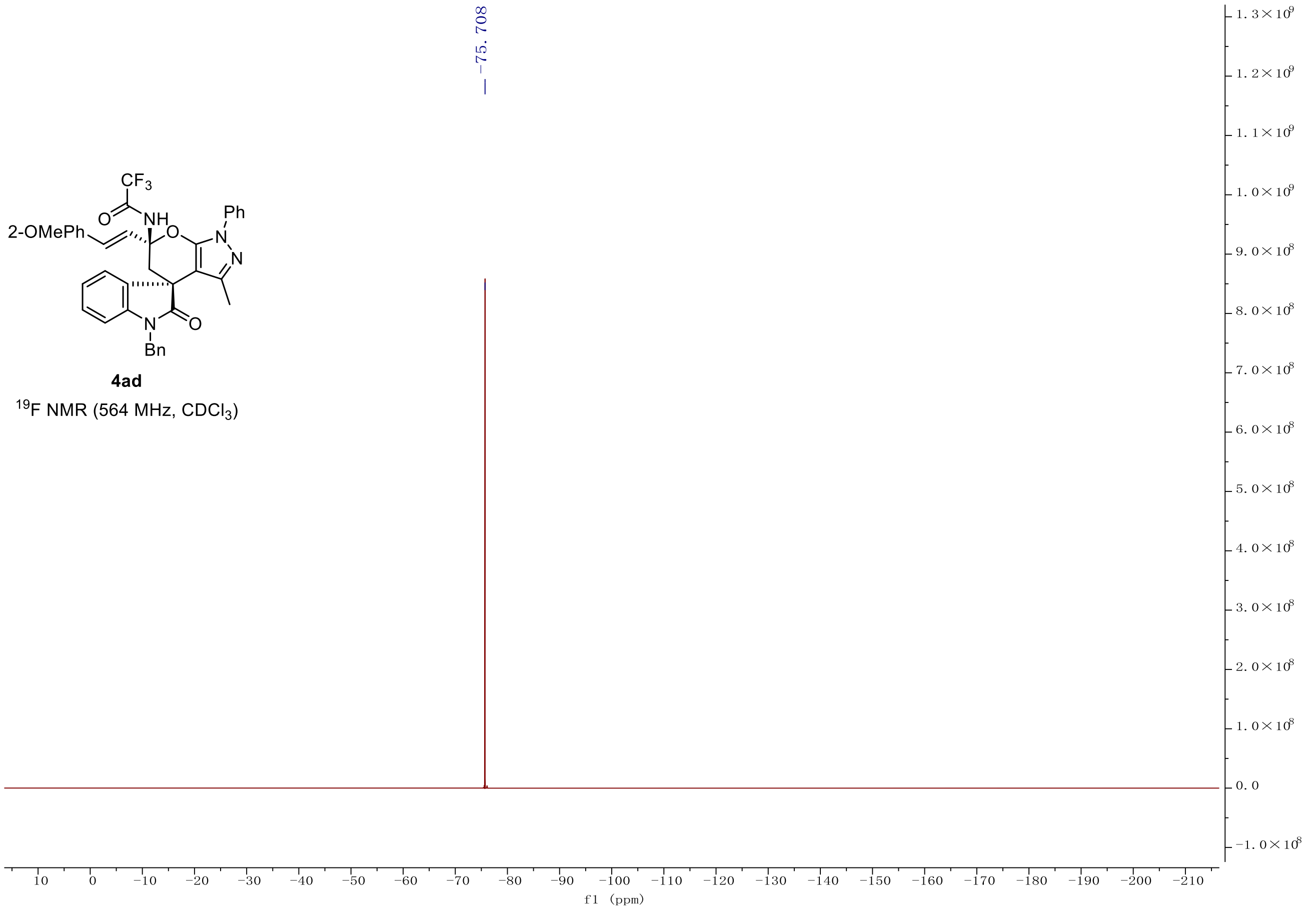

4ad

^{19}F NMR (564 MHz, $CDCl_3$)

Peak Analysis Report

Detector A Channel 1 254nm

No.	Ret. Time	Height (mAu)	Area (mAu*min)	Rel. Area (%)
1	12.504	247630	7899719	49.748
2	14.162	219045	7979702	50.252
Total		466676	15879421	100.000

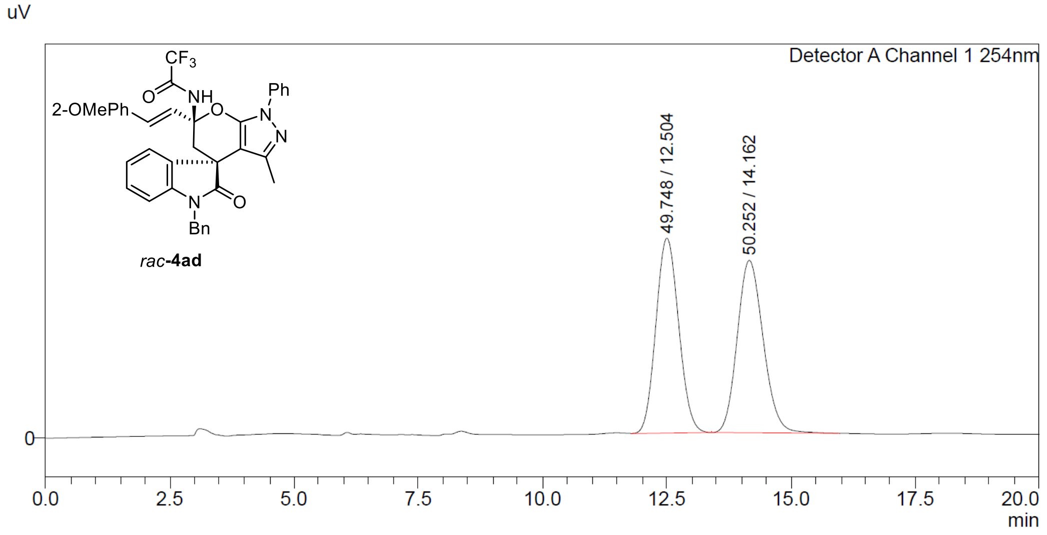

Peak Analysis Report

Detector A Channel 1 254nm

No.	Ret. Time	Height (mAu)	Area (mAu*min)	Rel. Area (%)
1	12.730	878339	28973312	99.906
2	14.171	1352	27357	0.094
Total		879691	29000669	100.000

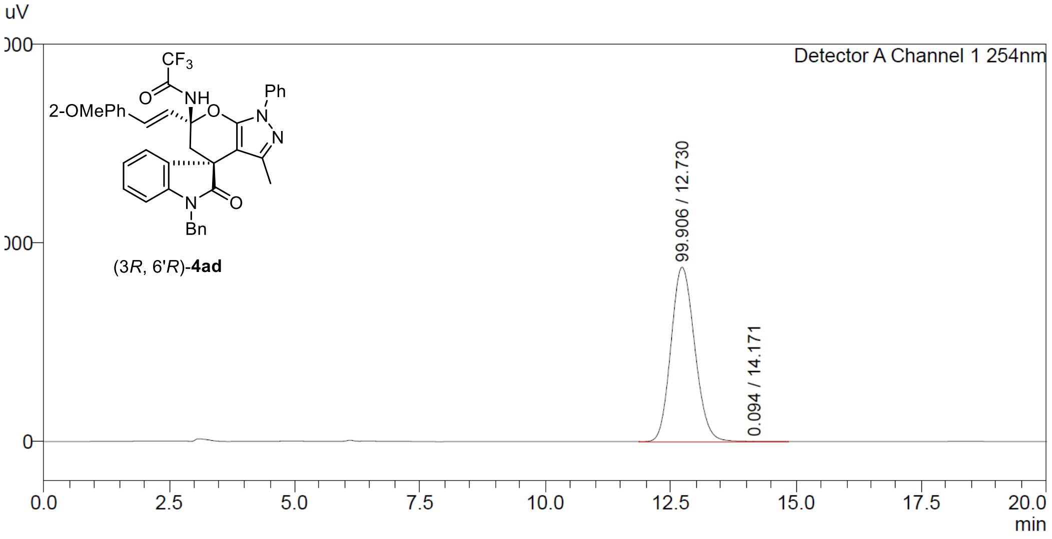

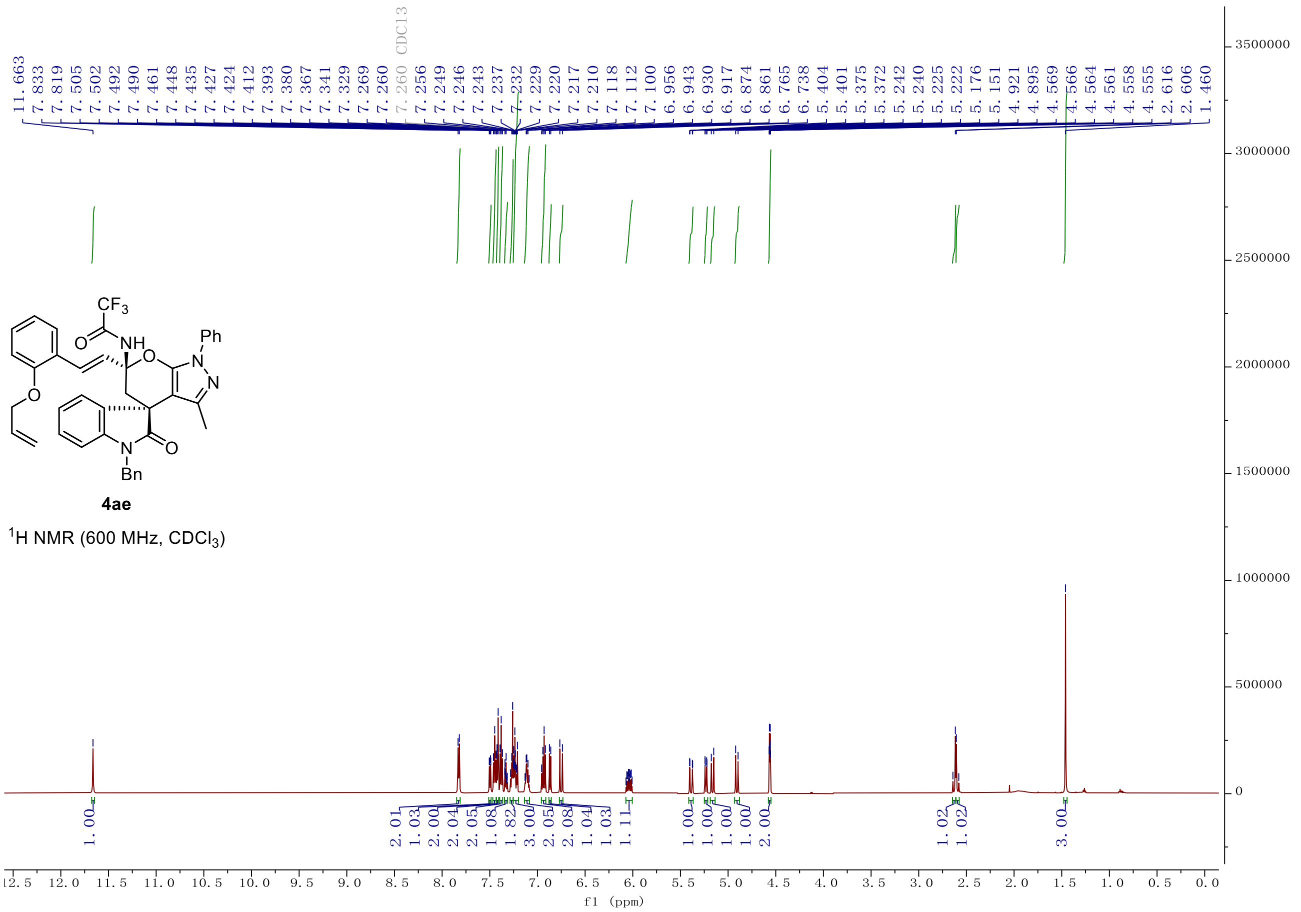
11.663
7.833
7.819
7.505
7.502
7.492
7.490
7.461
7.448
7.435
7.427
7.424
7.412
7.393
7.380
7.367
7.341
7.329
7.269
7.260
7.260 CDCl3
7.256
7.249
7.246
7.243
7.237
7.232
7.229
7.220
7.217
7.210
7.118
7.112
7.100
6.956
6.943
6.930
6.917
6.874
6.861
6.765
6.738
5.404
5.401
5.375
5.372
5.242
5.240
5.225
5.222
5.176
5.151
4.921
4.895
4.569
4.566
4.564
4.561
4.558
4.555
2.616
2.606
1.460
CF3
NH
O
Ph
N
N
O
O
N
Bn
O
4ae
1H NMR (600 MHz, CDCl3)
1.00
2.01
1.03
2.00
2.04
2.05
1.08
1.82
3.00
2.05
2.08
1.04
1.03
1.11
1.00
1.00
1.00
1.00
2.00
1.02
1.02
3.00
f1 (ppm)

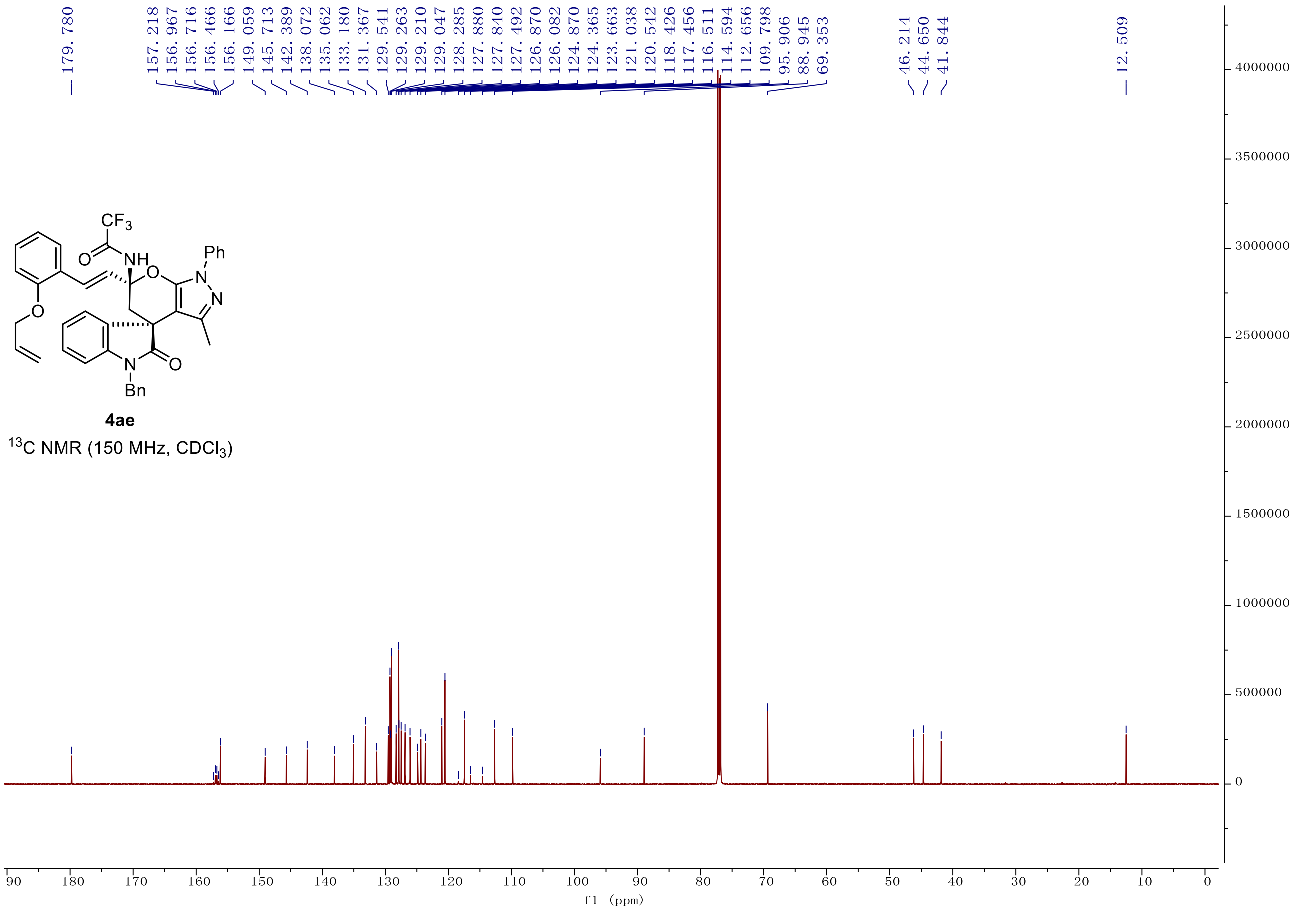
179.780
157.218
156.967
156.716
156.466
156.166
149.059
145.713
142.389
138.072
135.062
133.180
131.367
129.541
129.263
129.210
129.047
128.285
127.880
127.840
127.492
126.870
126.082
124.870
124.365
123.663
121.038
120.542
118.426
117.456
116.511
114.594
112.656
109.798
95.906
88.945
69.353
46.214
44.650
41.844
12.509
CF3
NH
O
Ph
N
N
O
O
N
Bn
O
4ae
13C NMR (150 MHz, CDCl3)
f1 (ppm)

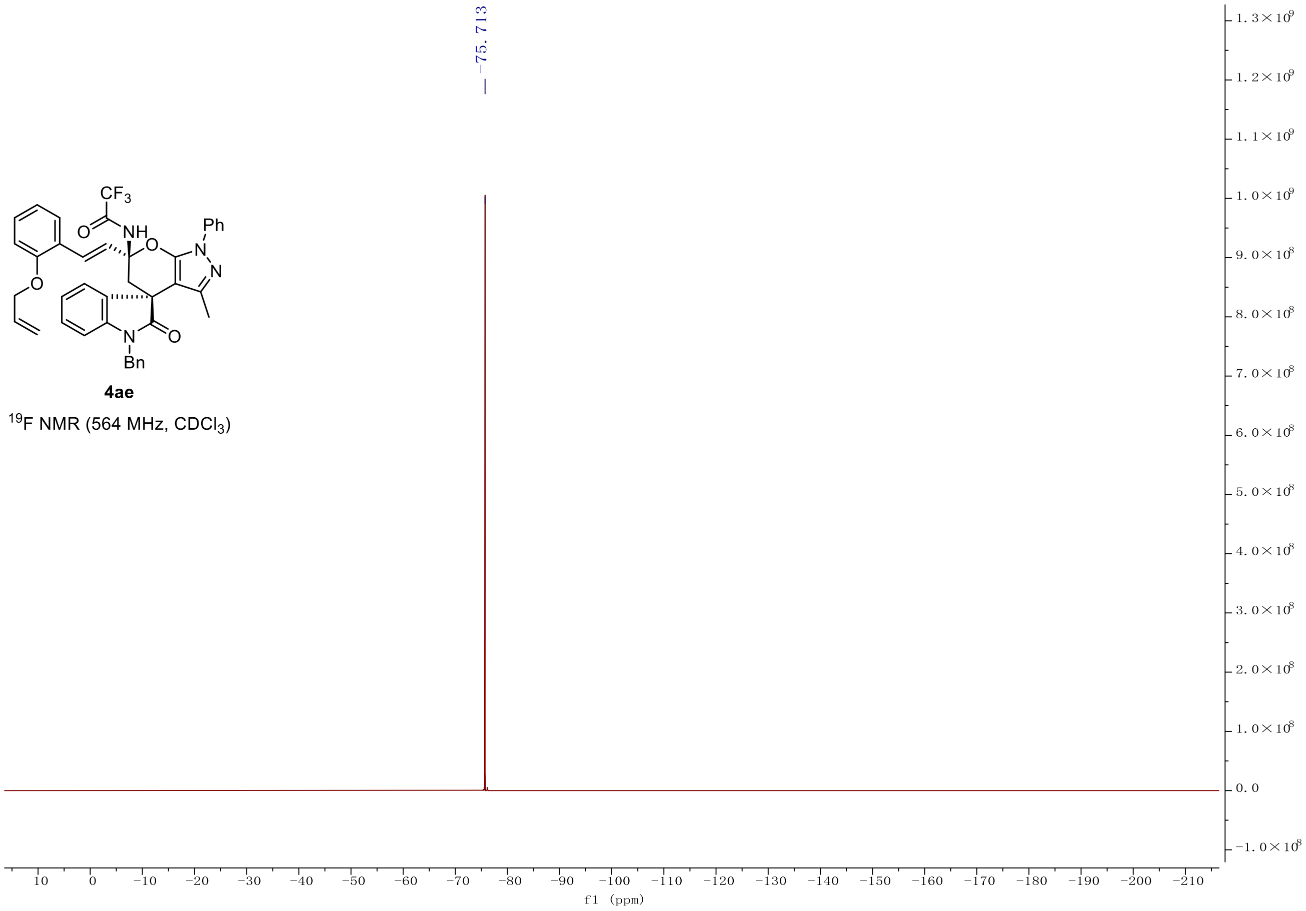

Peak Analysis Report

Detector A Channel 1 254nm

No.	Ret. Time	Height (mAu)	Area (mAu*min)	Rel. Area (%)
1	4.844	133031	1920212	49.579
2	6.093	77898	1952849	50.421
Total		210928	3873062	100.000

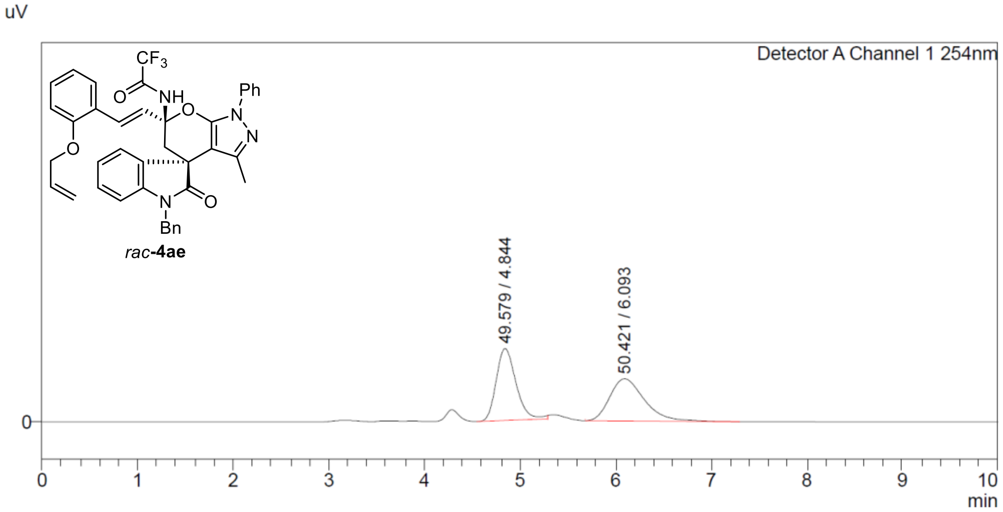

Peak Analysis Report

Detector A Channel 1 254nm

No.	Ret. Time	Height (mAu)	Area (mAu*min)	Rel. Area (%)
1	4.842	38347	502033	2.328
2	6.097	1348661	21058529	97.672
Total		1387008	21560562	100.000

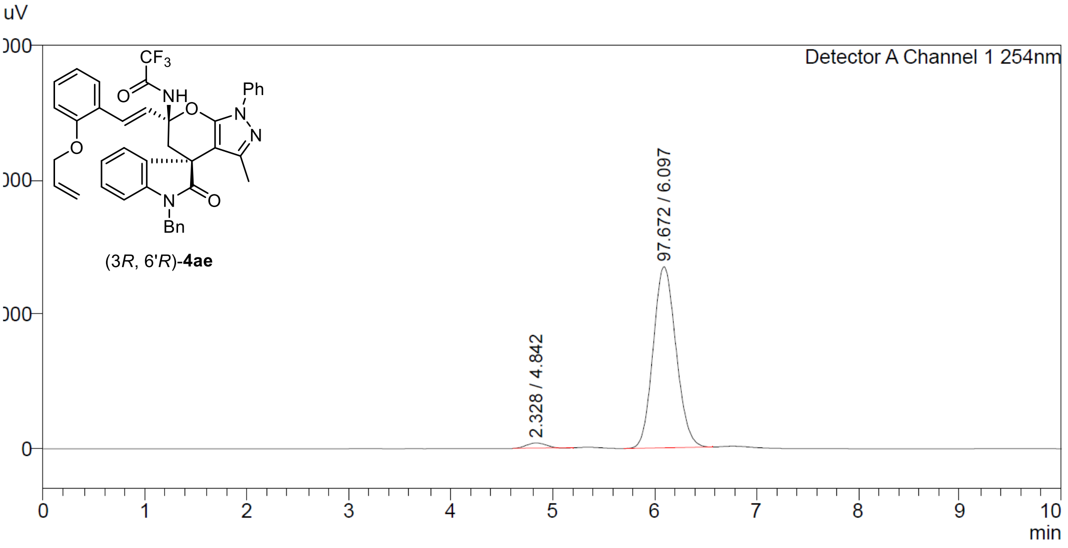

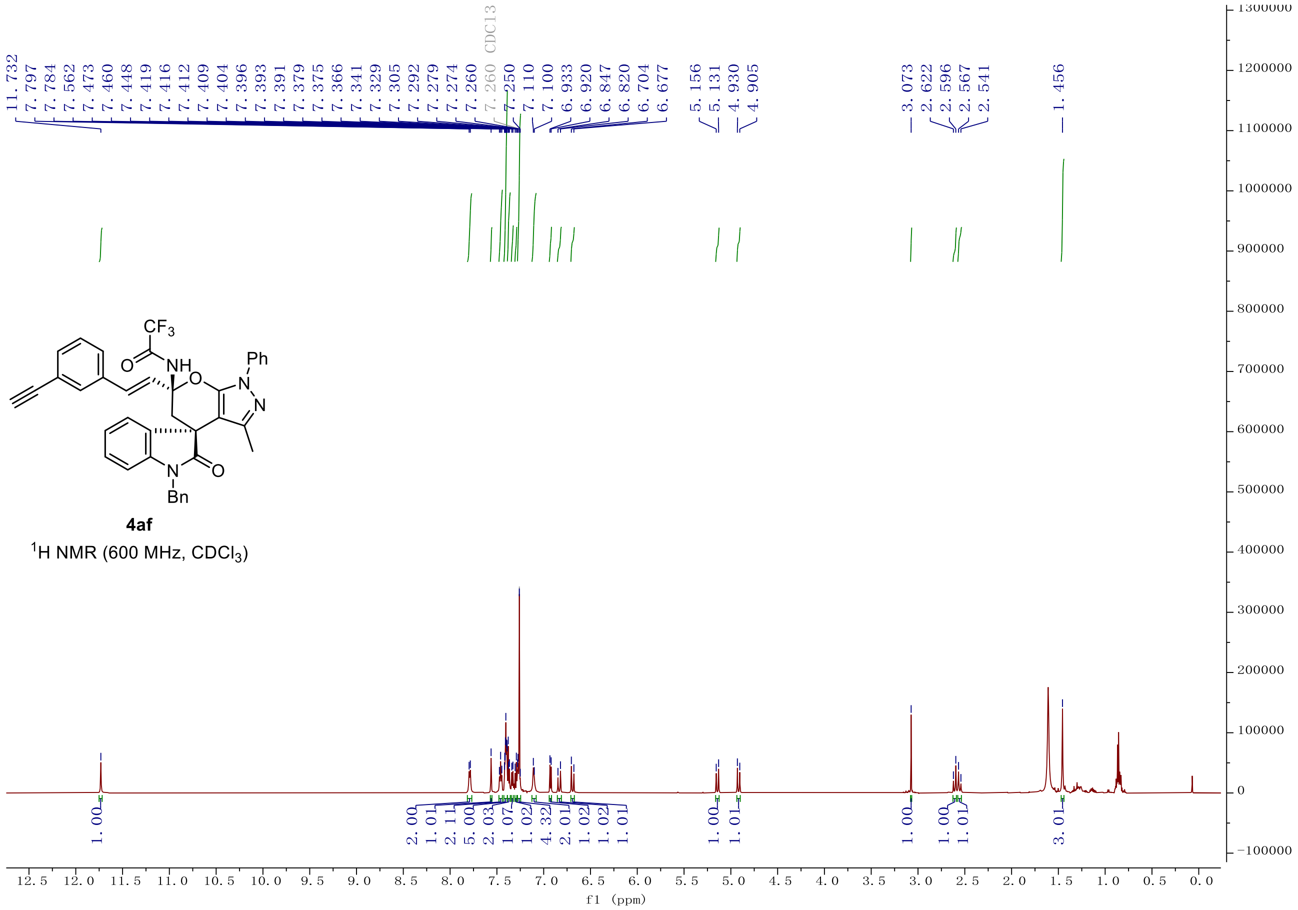

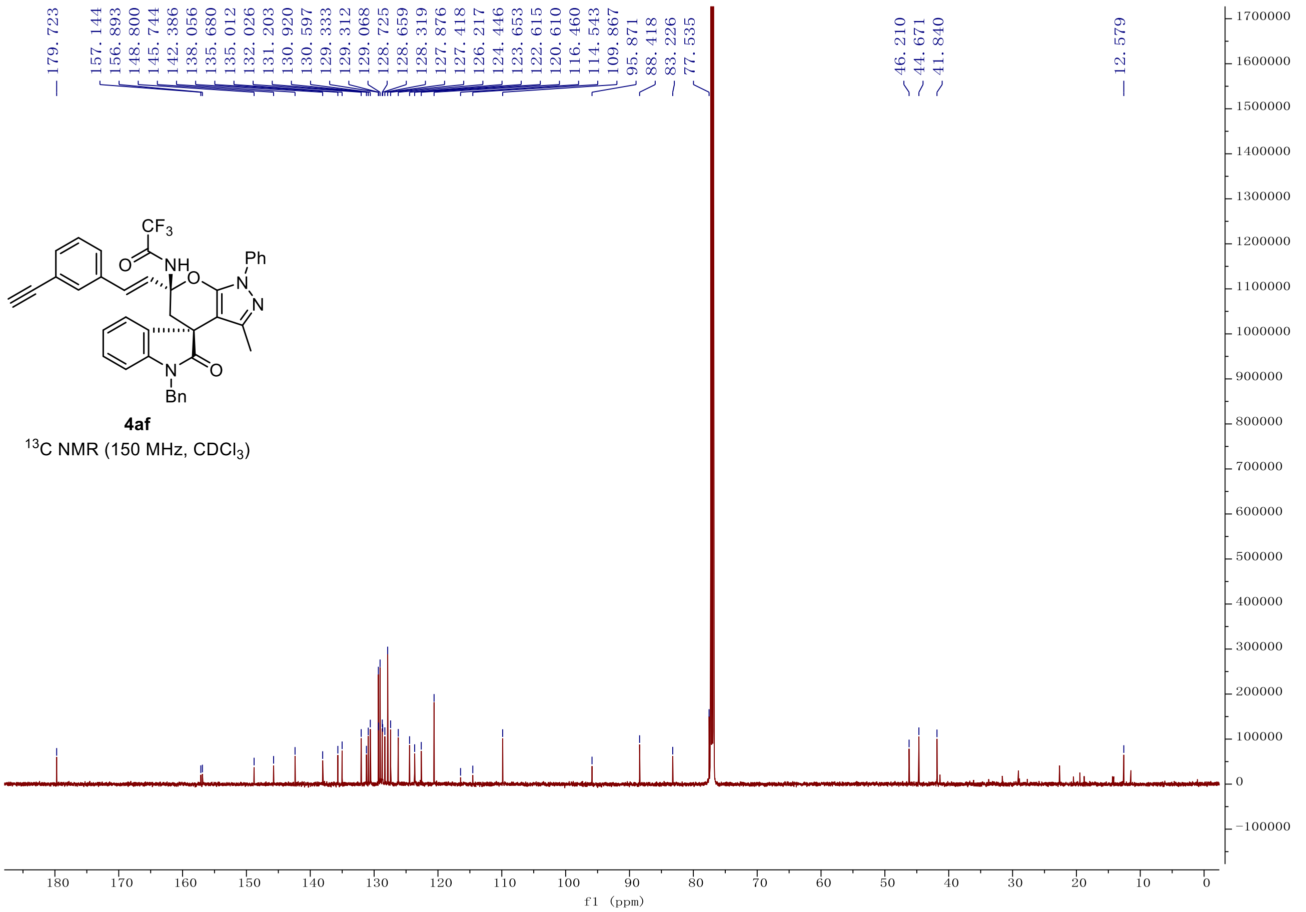

4af

^{13}C NMR (150 MHz, $CDCl_3$)

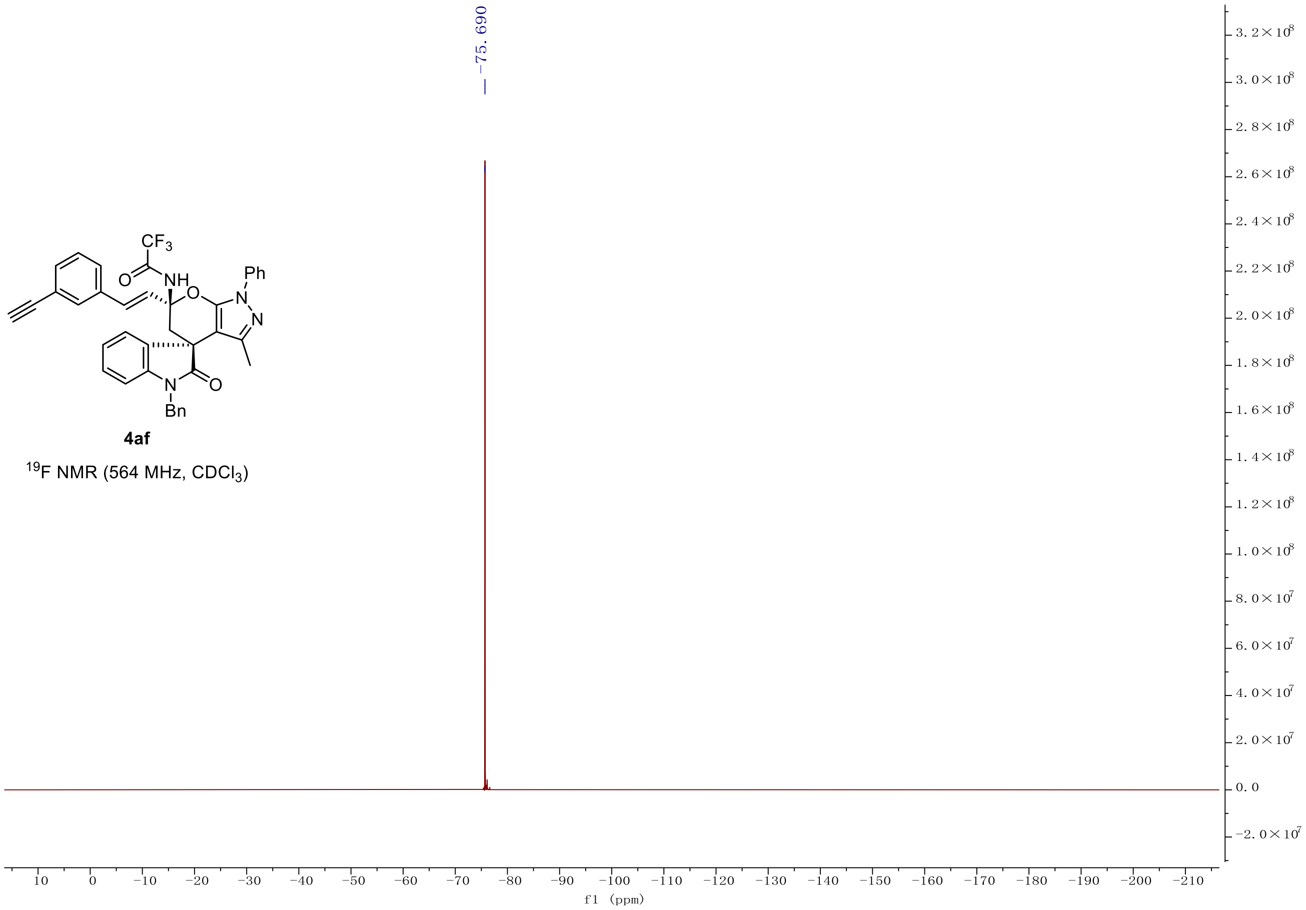

4af

^{19}F NMR (564 MHz, $CDCl_3$)

Peak Analysis Report

Detector A Channel 1 254nm

No.	Ret. Time	Height (mAu)	Area (mAu*min)	Rel. Area (%)
1	5.187	1180421	13746901	50.585
2	6.381	867381	13429168	49.415
Total		2047802	27176069	100.000

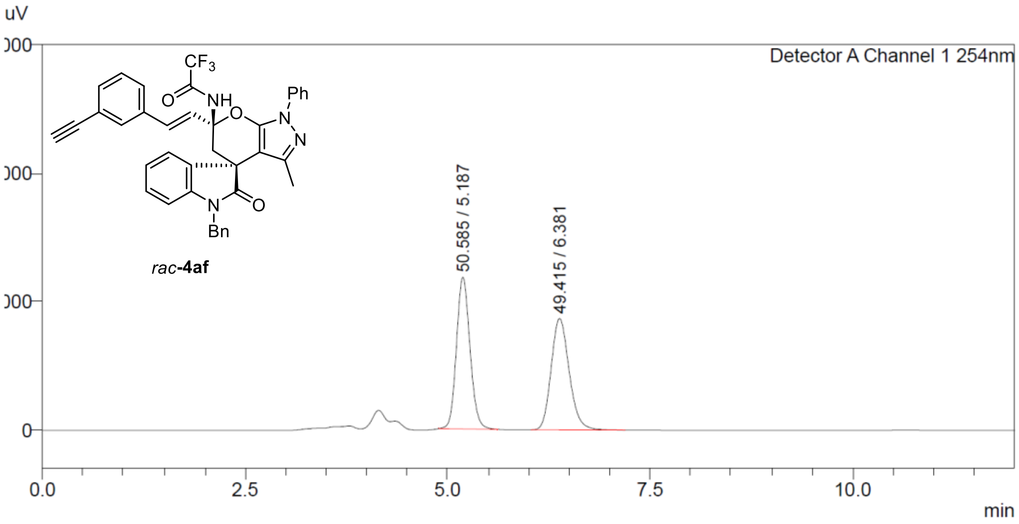

Peak Analysis Report

Detector A Channel 1 254nm

No.	Ret. Time	Height (mAu)	Area (mAu*min)	Rel. Area (%)
1	5.195	1943416	22065327	98.452
2	6.400	26822	347045	1.548
Total		1970237	22412372	100.000

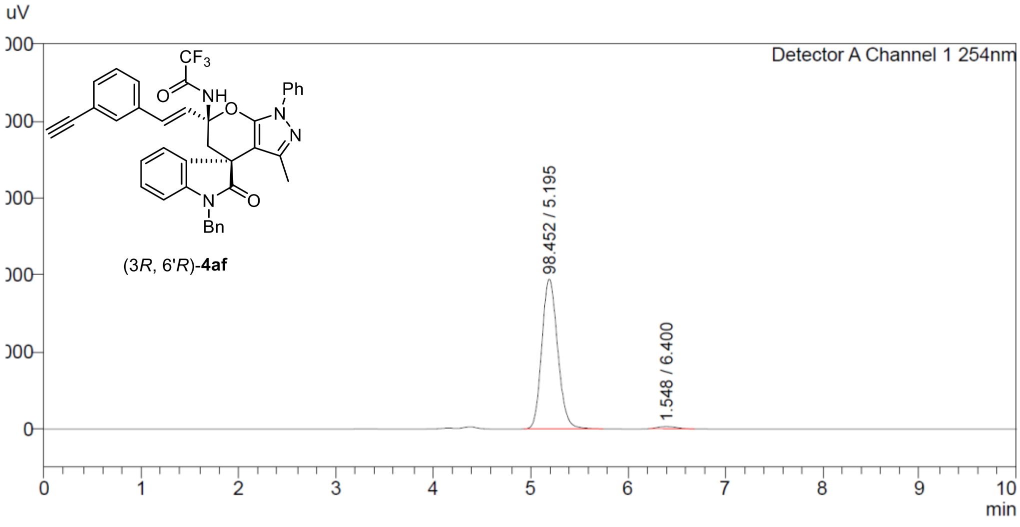

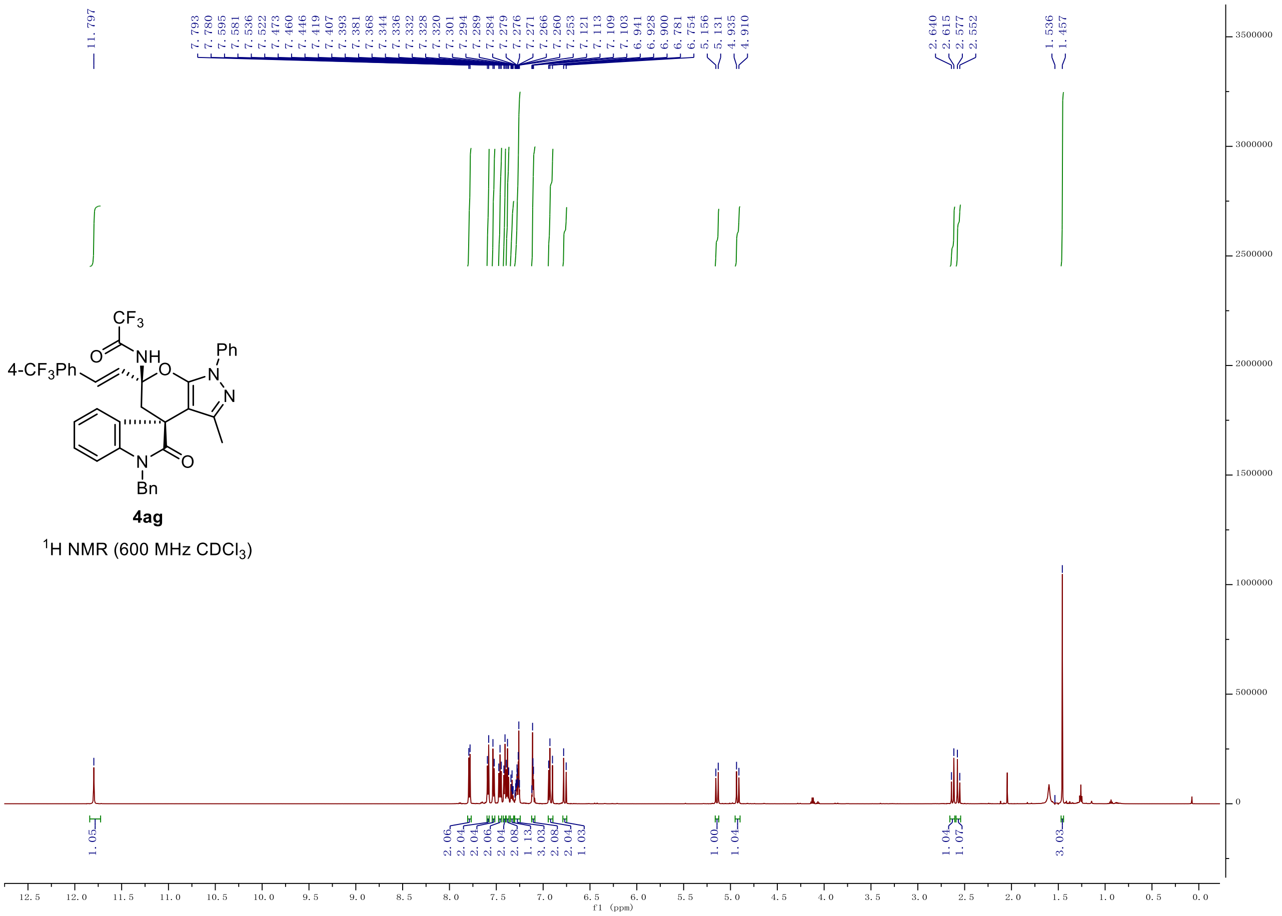

4ag

^{1}H NMR (600 MHz $CDCl_3$)

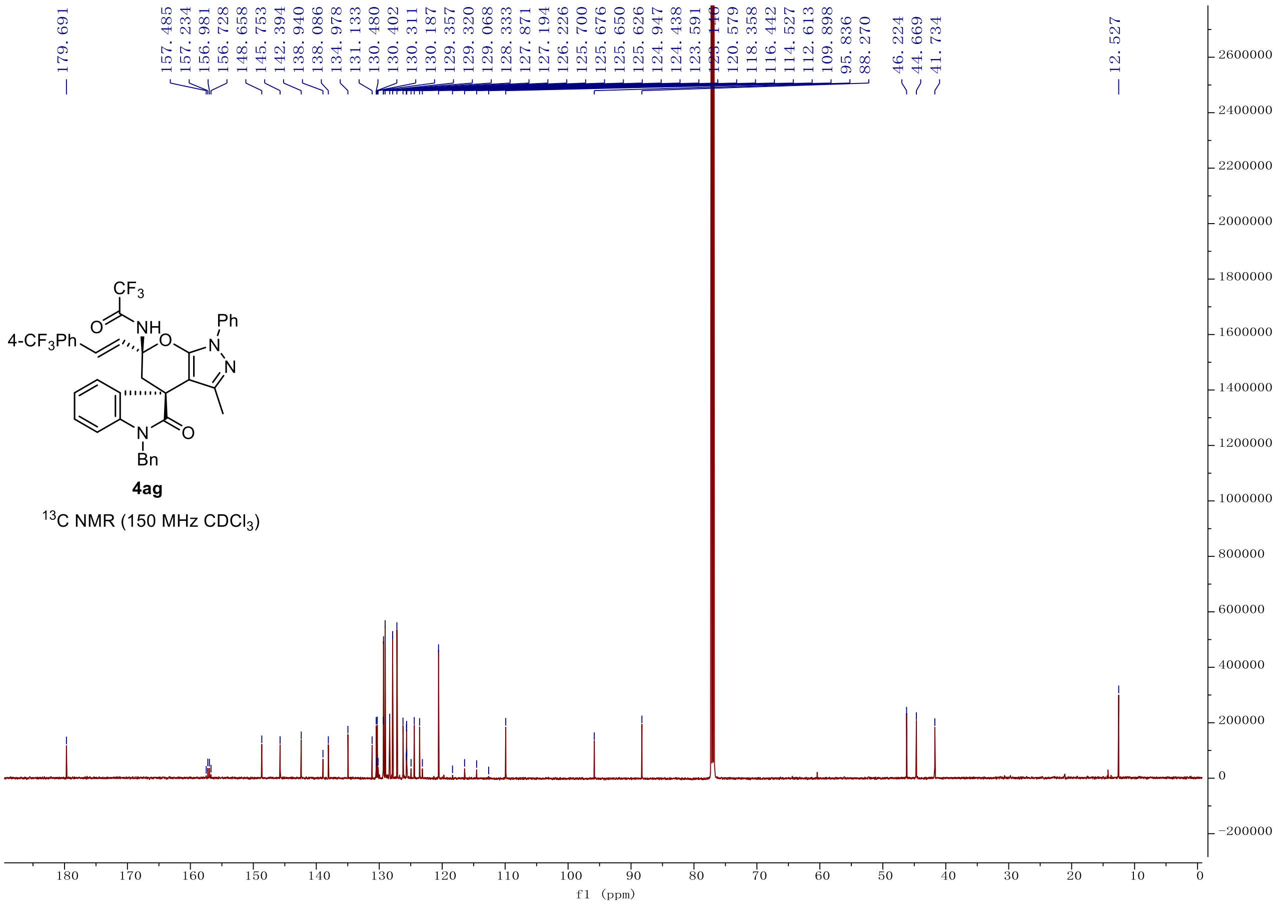

4ag

^{13}C NMR (150 MHz $CDCl_3$)

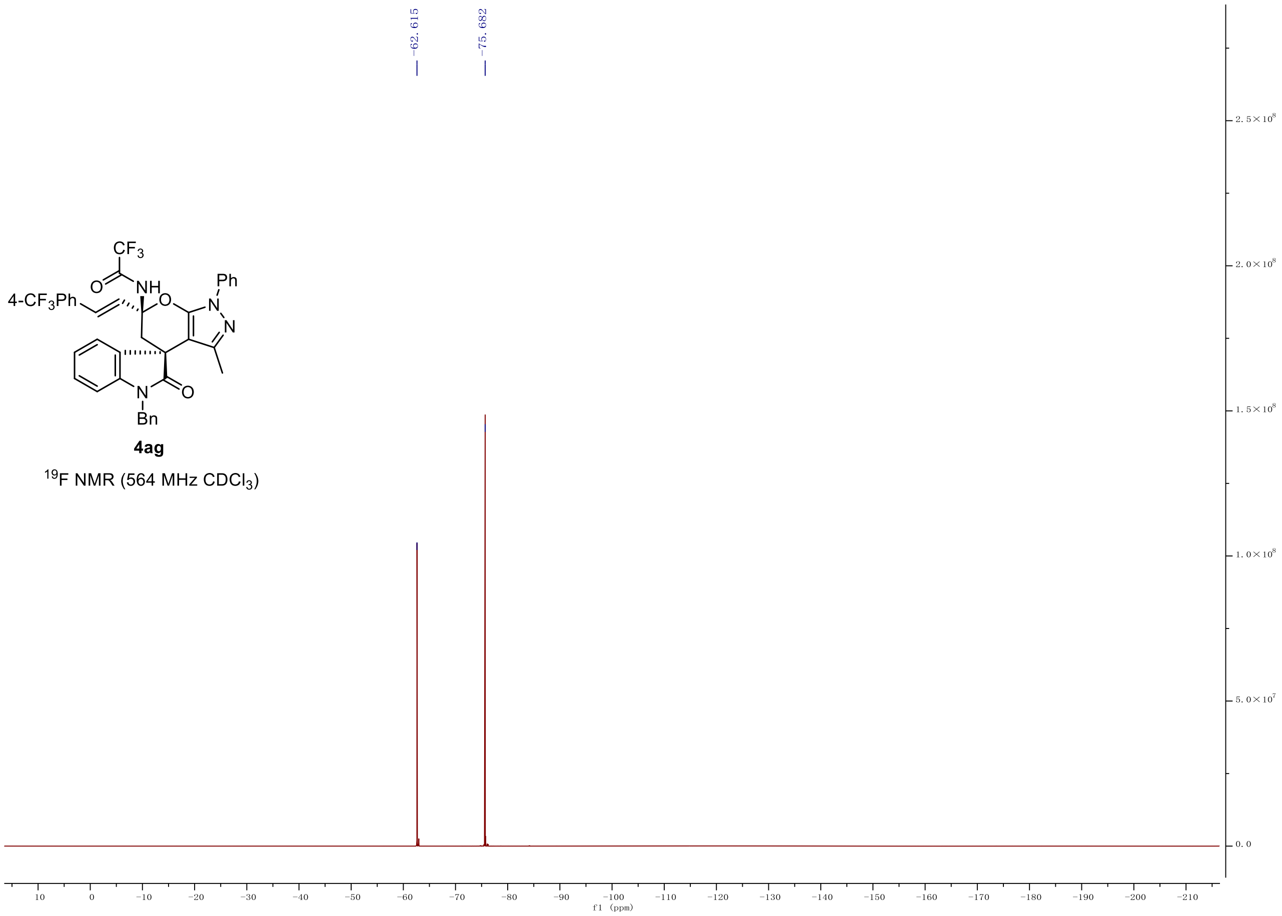

Peak Analysis Report

Detector A Channel 1 254nm

No.	Ret. Time	Height (mAu)	Area (mAu*min)	Rel. Area (%)
1	7.468	741920	11825090	50.283
2	15.662	362621	11691850	49.717
Total		1104541	23516940	100.000

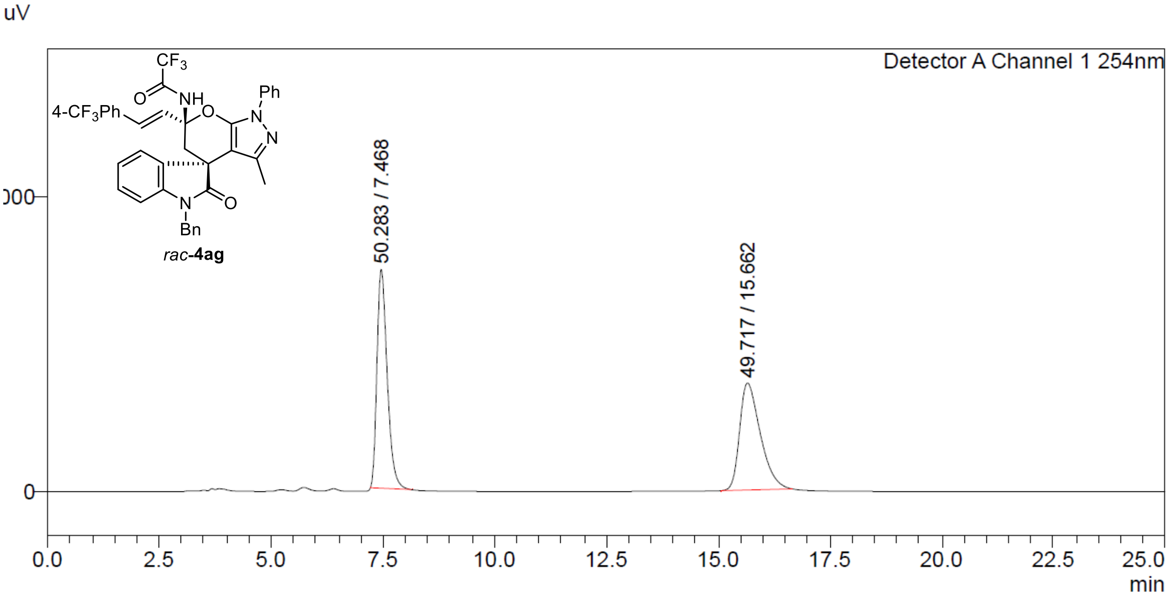

Peak Analysis Report

Detector A Channel 1 254nm

No.	Ret. Time	Height (mAu)	Area (mAu*min)	Rel. Area (%)
1	7.538	2911	47882	0.706
2	15.772	203655	6736633	99.294
Total		206566	6784515	100.000

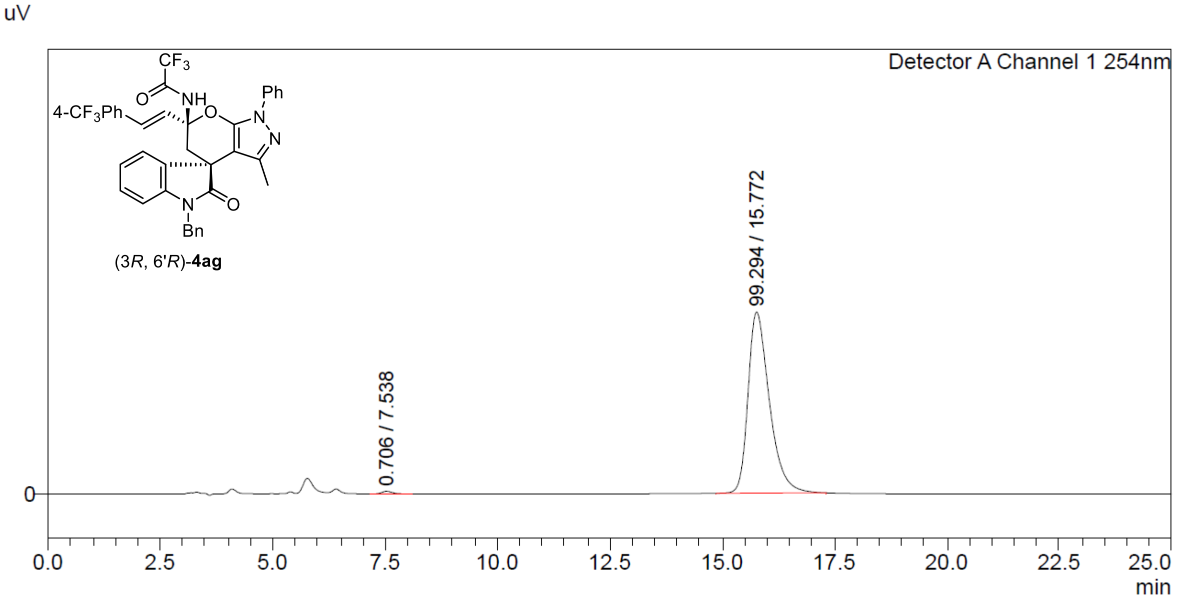

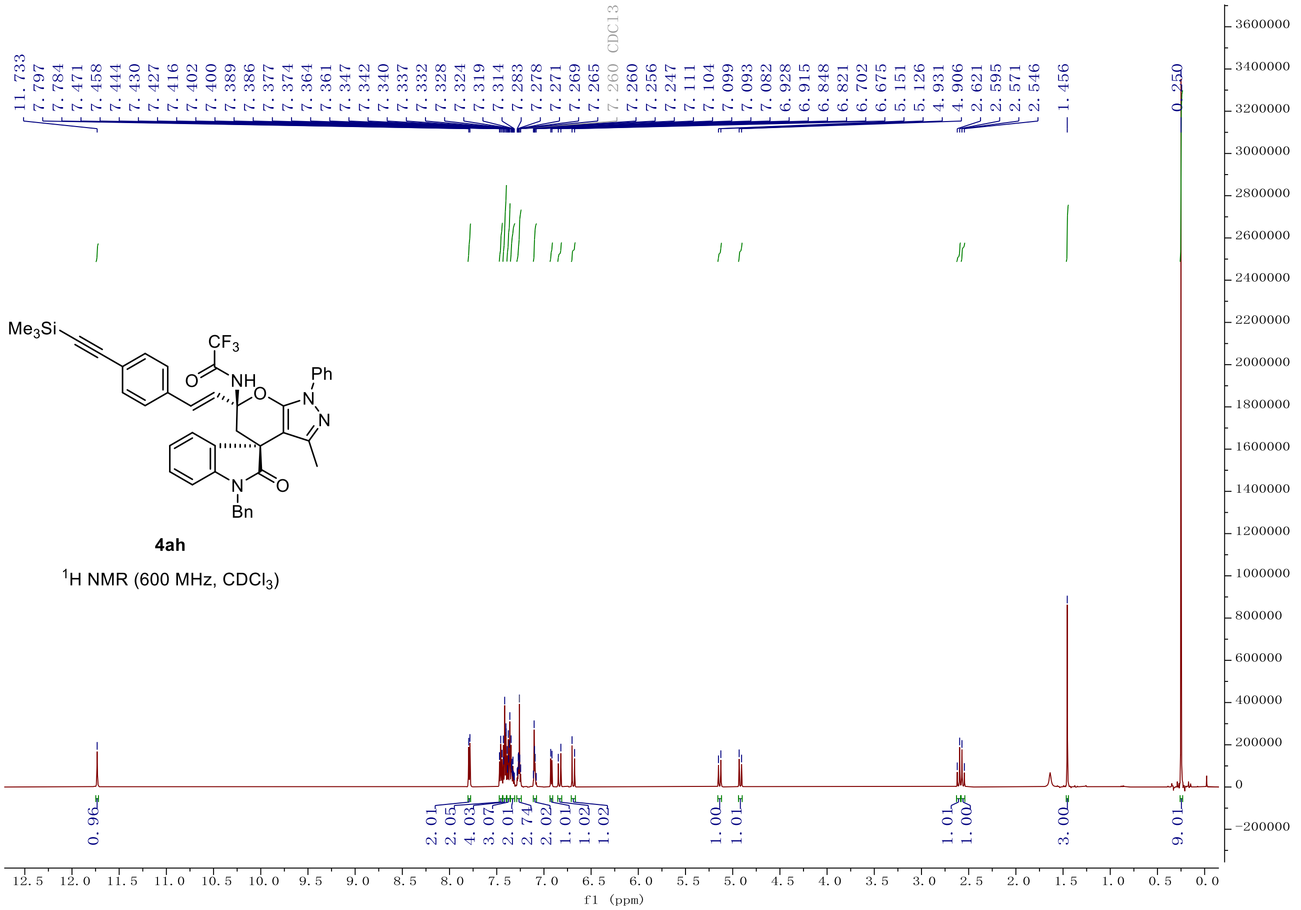

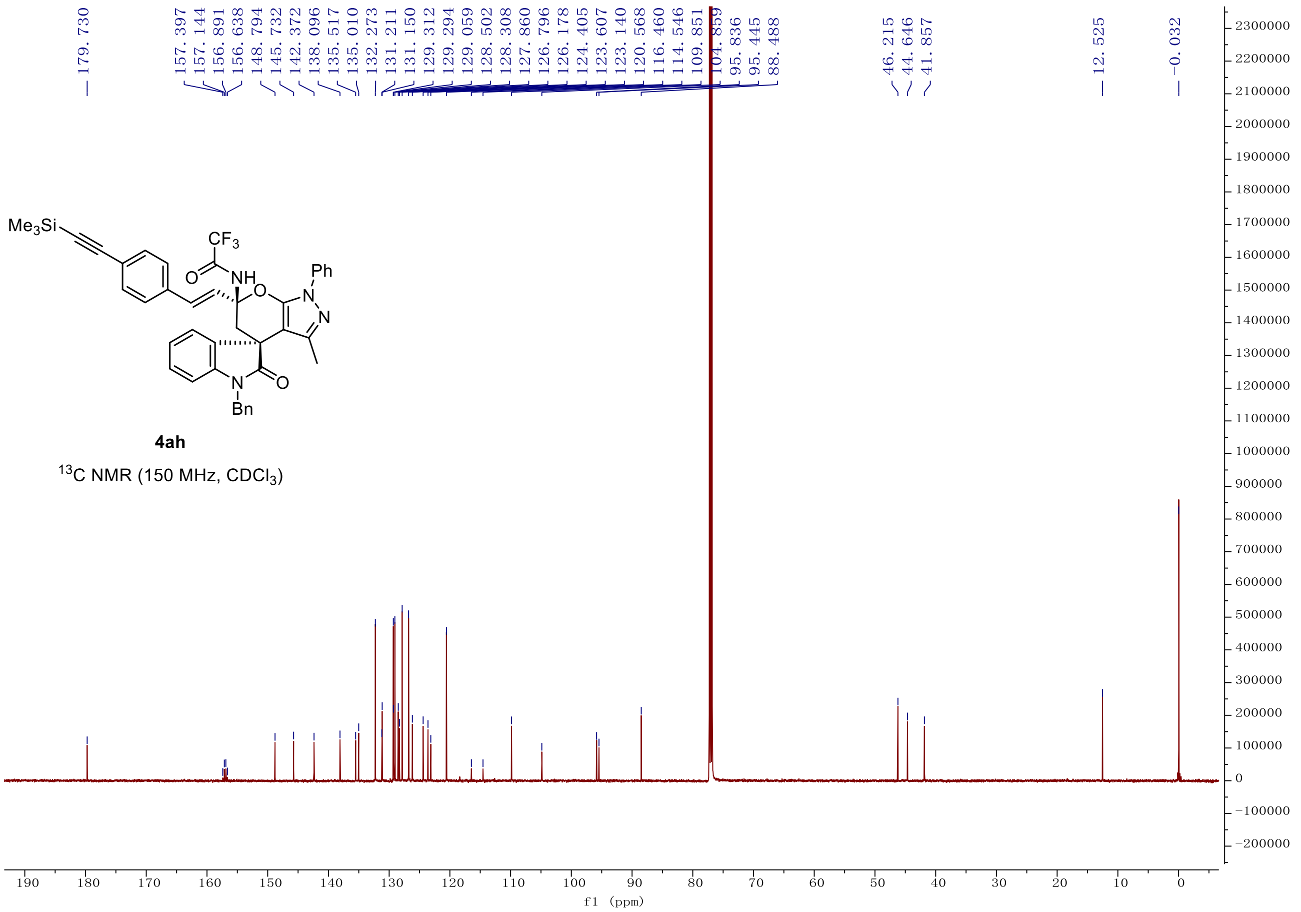
179.730
157.397
157.144
156.891
156.638
148.794
145.732
142.372
138.096
135.517
135.010
132.273
131.211
131.150
129.312
129.294
129.059
128.502
128.308
127.860
126.796
126.178
124.405
123.607
123.140
120.568
116.460
114.546
109.851
104.859
95.836
95.445
88.488
46.215
44.646
41.857
12.525
-0.032
Me3Si
CF3
O
NH
O
Ph
N
N
N
O
Bn
4ah
13C NMR (150 MHz, CDCl3)
f1 (ppm)

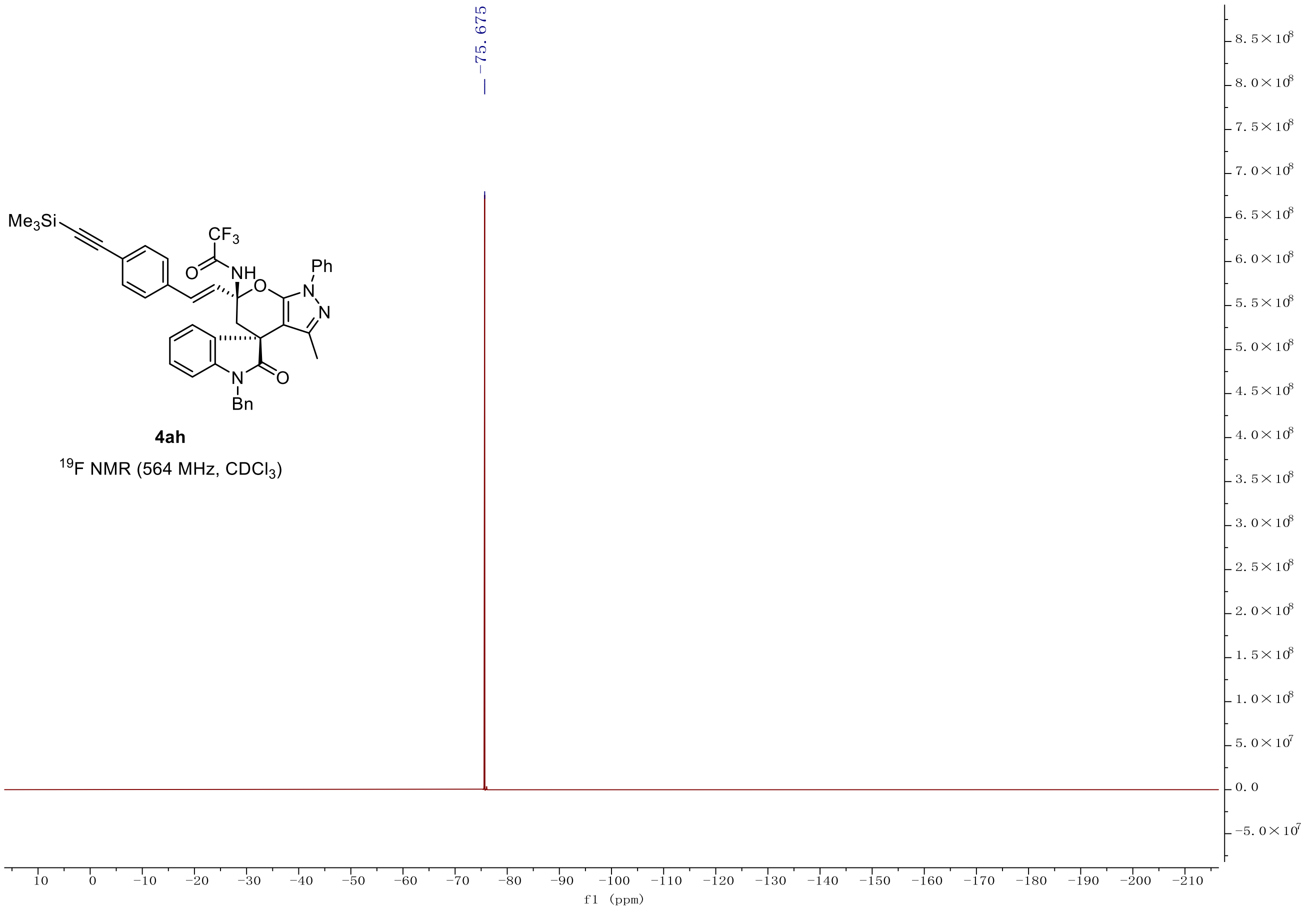
-75.675
Me3Si
CF3
O
NH
O
Ph
N
N
N
O
Bn
4ah
19F NMR (564 MHz, CDCl3)
f1 (ppm)

Peak Analysis Report

Detector A Channel 1 254nm

No.	Ret. Time	Height (mAu)	Area (mAu*min)	Rel. Area (%)
1	5.797	161923	2820301	49.114
2	7.310	124360	2922093	50.886
Total		286283	5742394	100.000

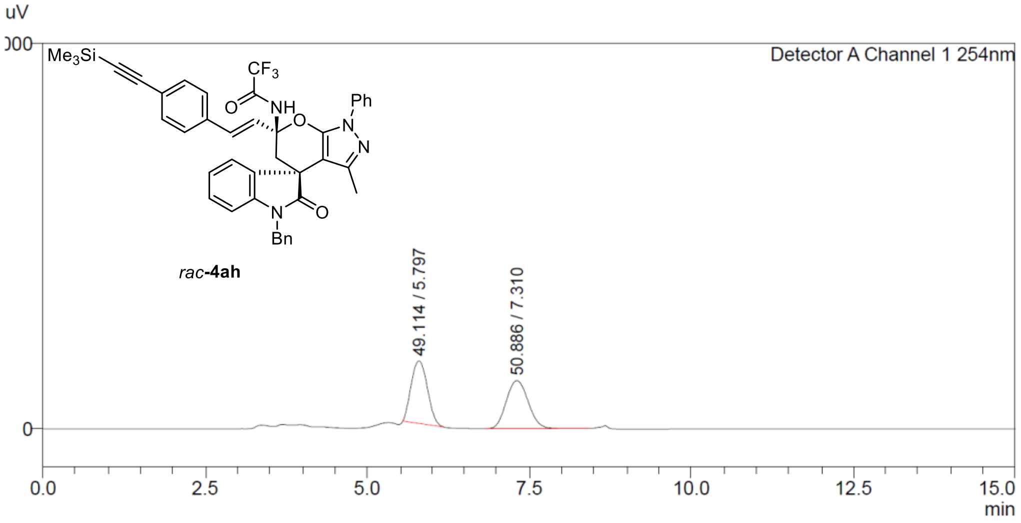

Peak Analysis Report

Detector A Channel 1 254nm

No.	Ret. Time	Height (mAu)	Area (mAu*min)	Rel. Area (%)
1	5.739	540095	9092913	99.191
2	7.161	3166	74155	0.809
Total		543261	9167068	100.000

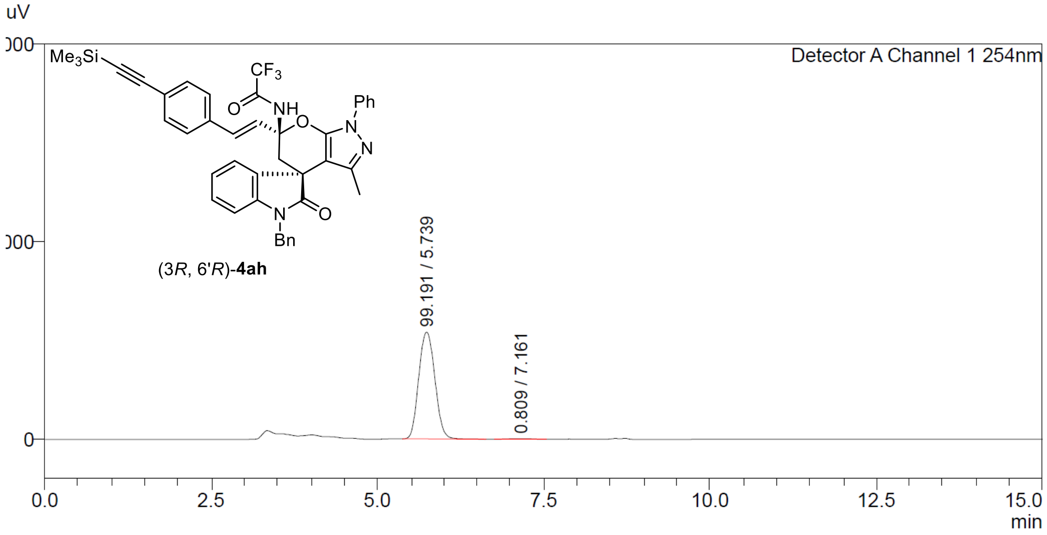

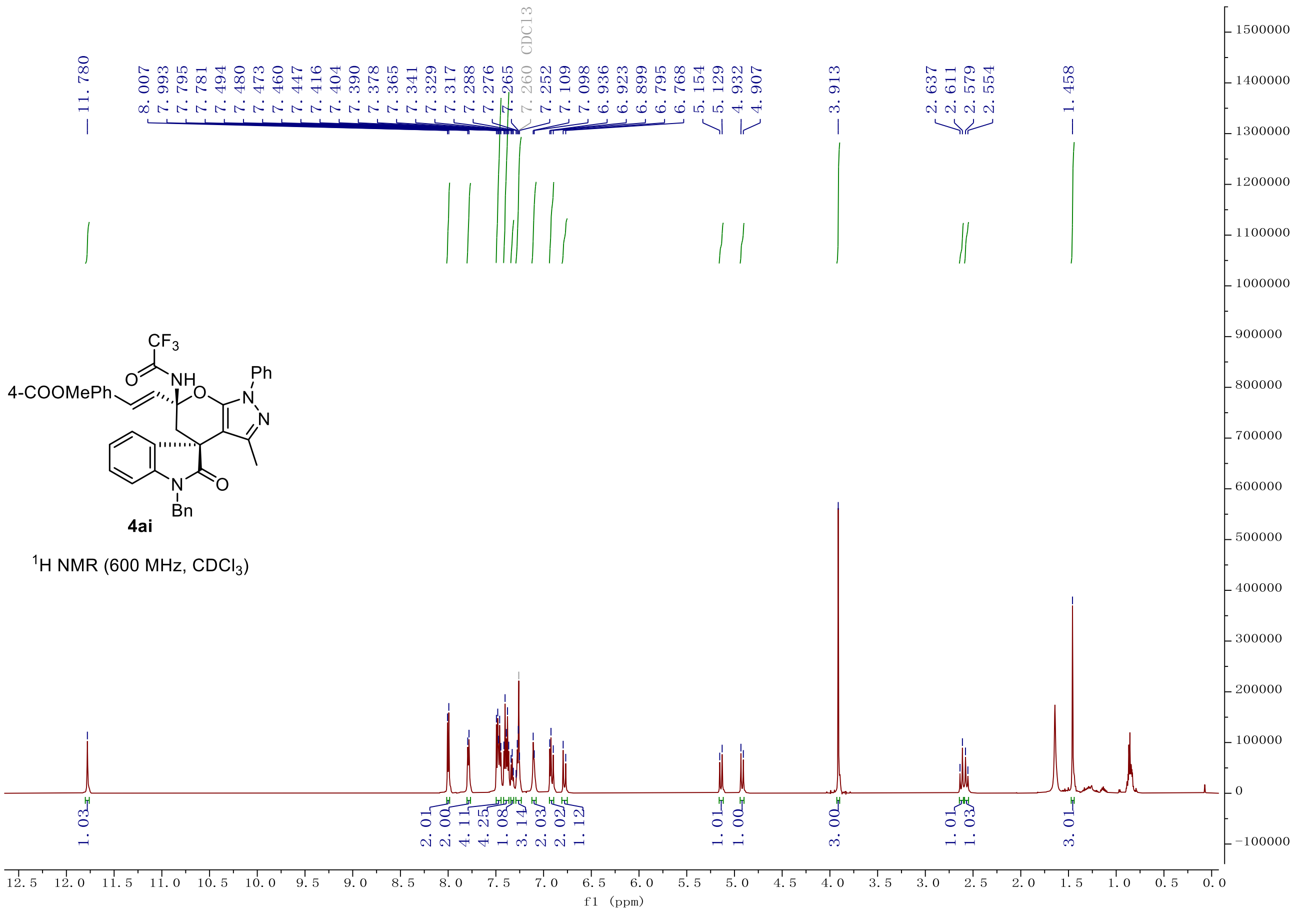
11.780
8.007 7.993 7.795 7.781 7.494 7.480 7.473 7.460 7.447 7.416 7.404 7.390 7.378 7.365 7.341 7.329 7.317 7.288 7.276 7.265 7.260 CDCl3 7.252 7.109 7.098 6.936 6.923 6.899 6.795 6.768
5.154 5.129 4.932 4.907
3.913
2.637 2.611 2.579 2.554
1.458
1.03 2.01 2.00 4.11 4.25 1.08 3.14 2.03 2.02 1.12 1.01 1.00 3.00 1.01 1.03 3.01
CF3
O
NH
Ph
4-COOMePh
O
N
N
N
O
Bn
4ai
1H NMR (600 MHz, CDCl3)
f1 (ppm)

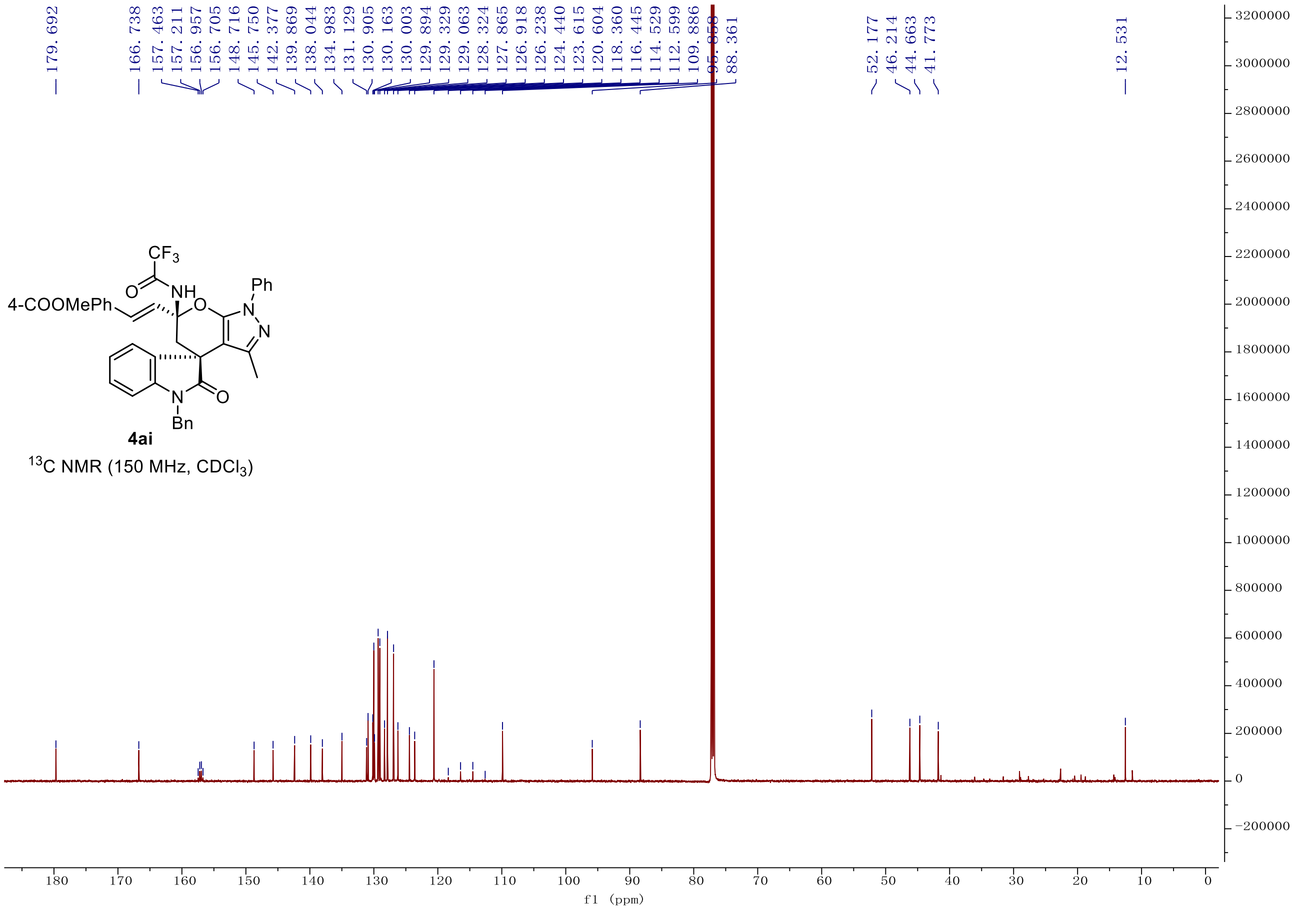
179.692
166.738 157.463 157.211 156.957 156.705 148.716 145.750 142.377 139.869 138.044 134.983 131.129 130.905 130.163 130.003 129.894 129.329 129.063 128.324 127.865 126.918 126.238 124.440 123.615 120.604 118.360 116.445 114.529 112.599 109.886 95.858 88.361
52.177 46.214 44.663 41.773
12.531
CF3
O
NH
Ph
4-COOMePh
O
N
N
N
O
Bn
4ai
13C NMR (150 MHz, CDCl3)
f1 (ppm)

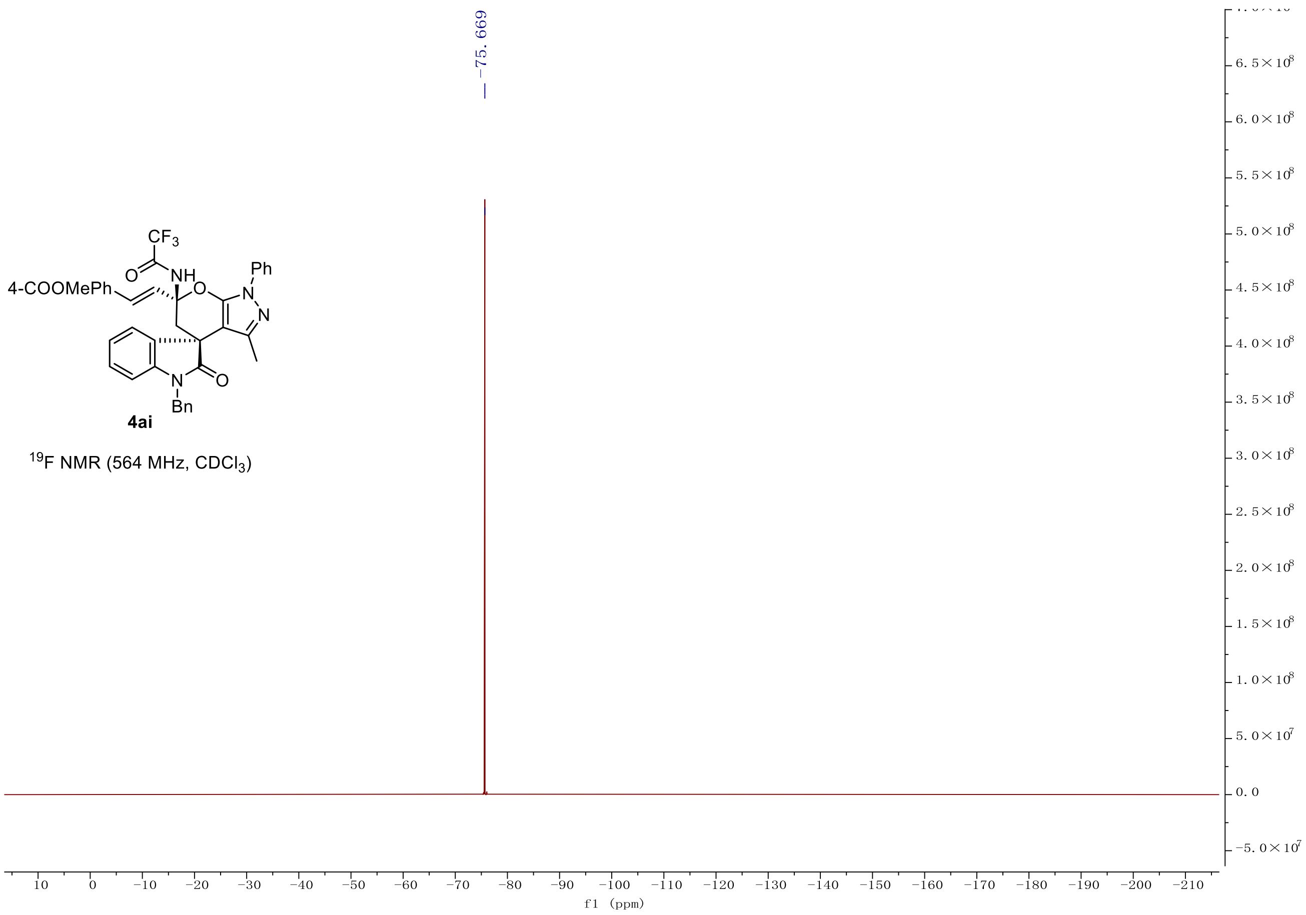

Signal: VWD1 B, Wavelength=254 nm

RT [min]Type	Area	Width[min]	Area%
9.002	19919.5000	0.374	50.5310
14.691	19500.8730	0.703	49.4690
总和	39420.3730		

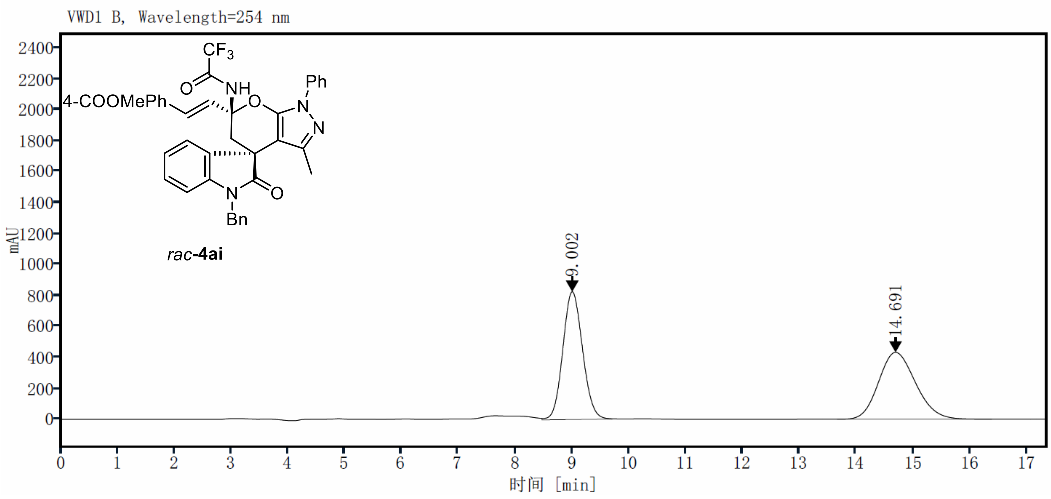

Signal: VWD1 B, Wavelength=254 nm

RT [min]Type	Area	Width[min]	Area%
9.024	31437.8750	0.348	95.9094
14.804	1340.8350	1.287	4.0906
总和	32778.7100		

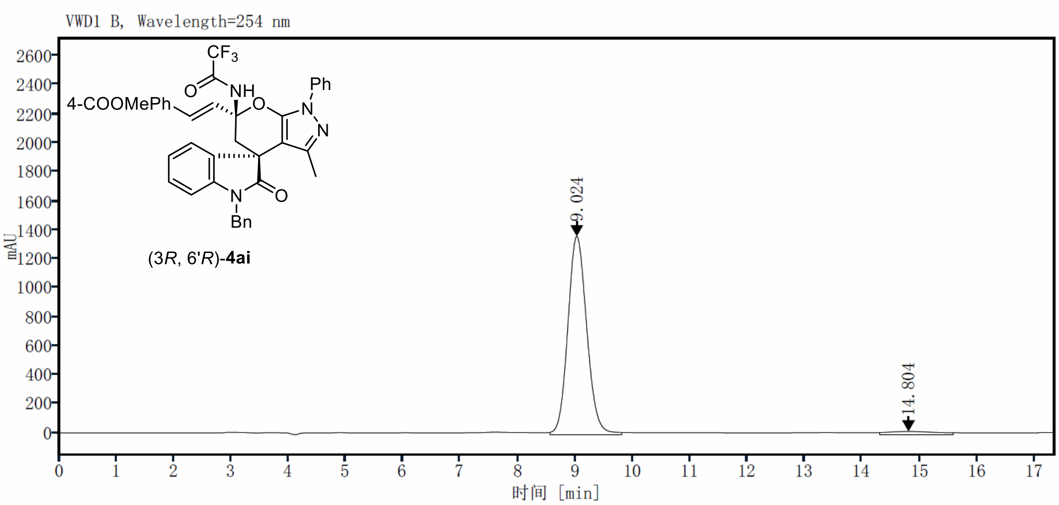

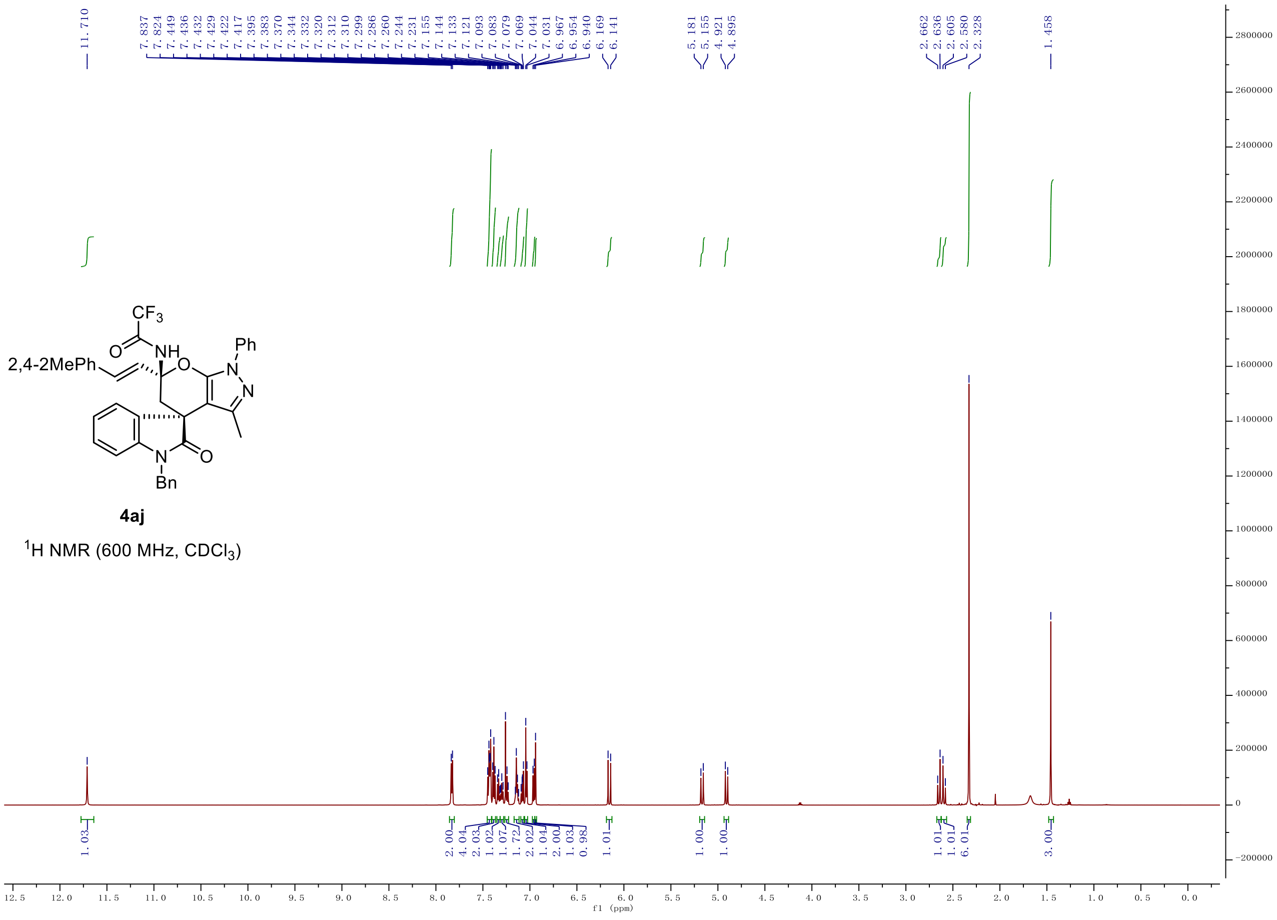

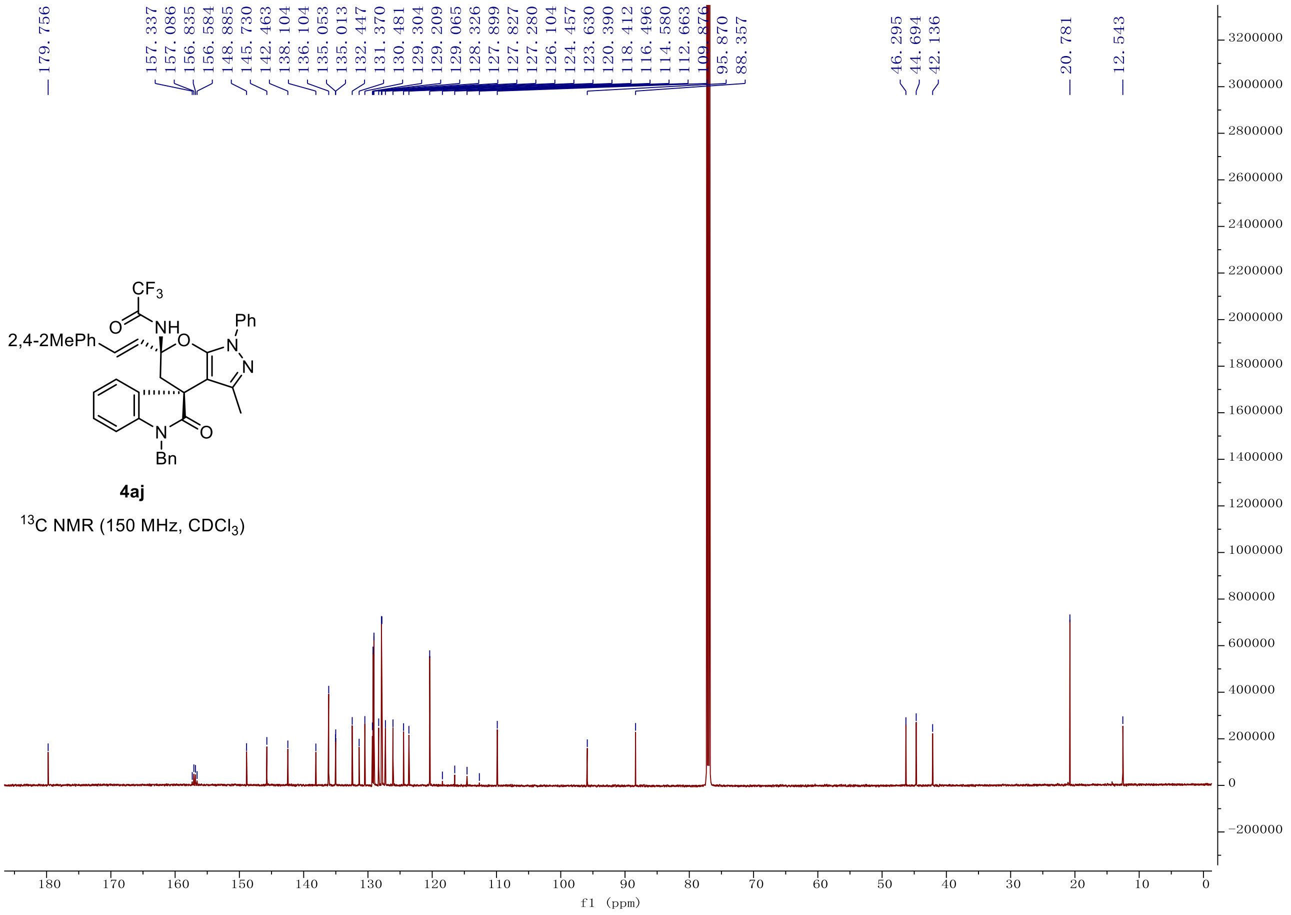
179.756
157.337
157.086
156.835
156.584
148.885
145.730
142.463
138.104
136.104
135.053
135.013
132.447
131.370
130.481
129.304
129.209
129.065
128.326
127.899
127.827
127.280
126.104
124.457
123.630
120.390
118.412
116.496
114.580
112.663
109.876
95.870
88.357
46.295
44.694
42.136
20.781
12.543
CF3
NH
O
Ph
2,4-2MePh
O
N
N
N
O
Bn
4aj
13C NMR (150 MHz, CDCl3)
f1 (ppm)

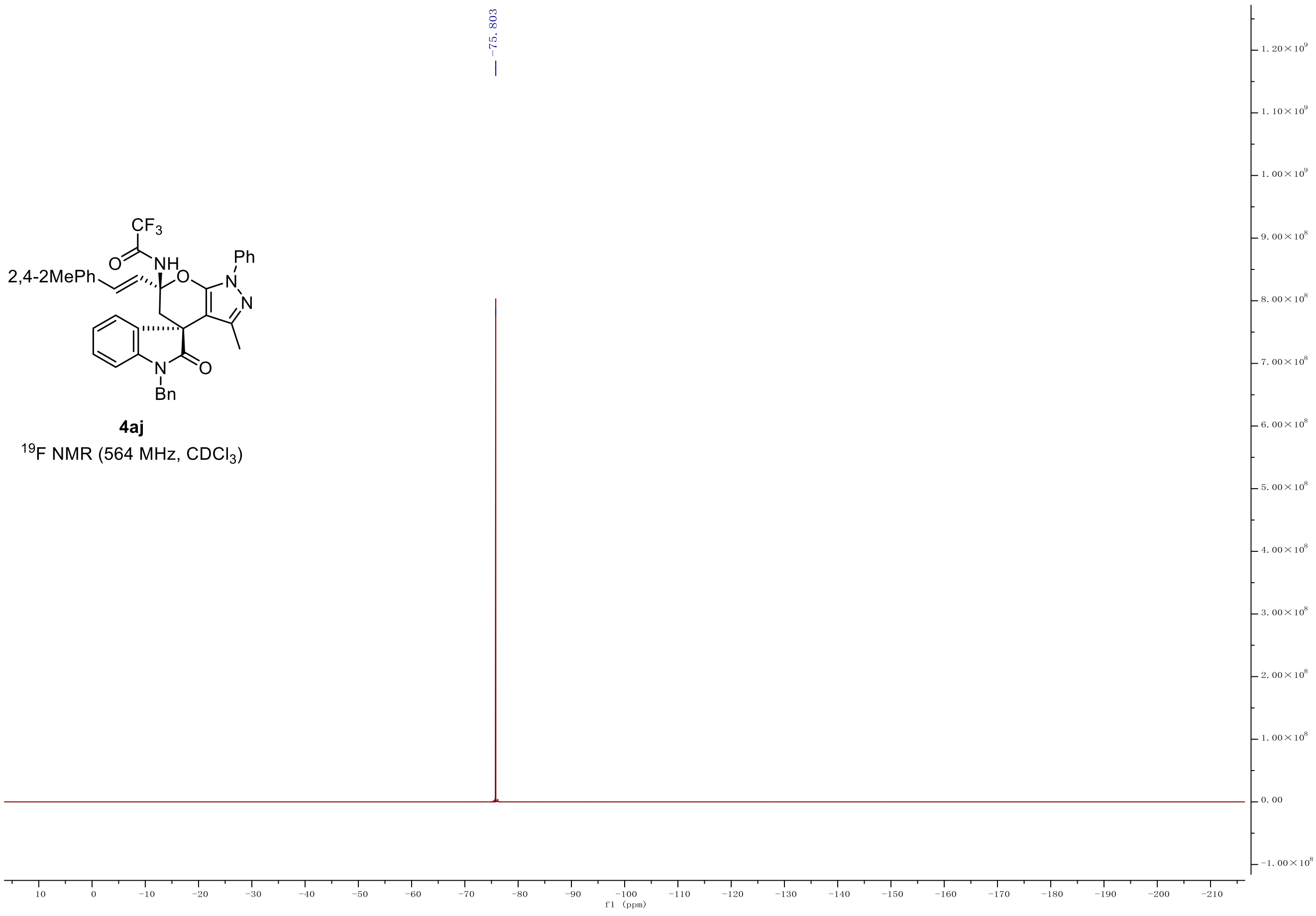
-75.803
CF3
NH
O
Ph
2,4-2MePh
O
N
N
N
O
Bn
4aj
19F NMR (564 MHz, CDCl3)
f1 (ppm)

Peak Analysis Report

Detector A Channel 1 254nm

No.	Ret. Time	Height (mAu)	Area (mAu*min)	Rel. Area (%)
1	5.840	1025144	10793732	49.998
2	7.395	783800	10794465	50.002
Total		1808944	21588197	100.000

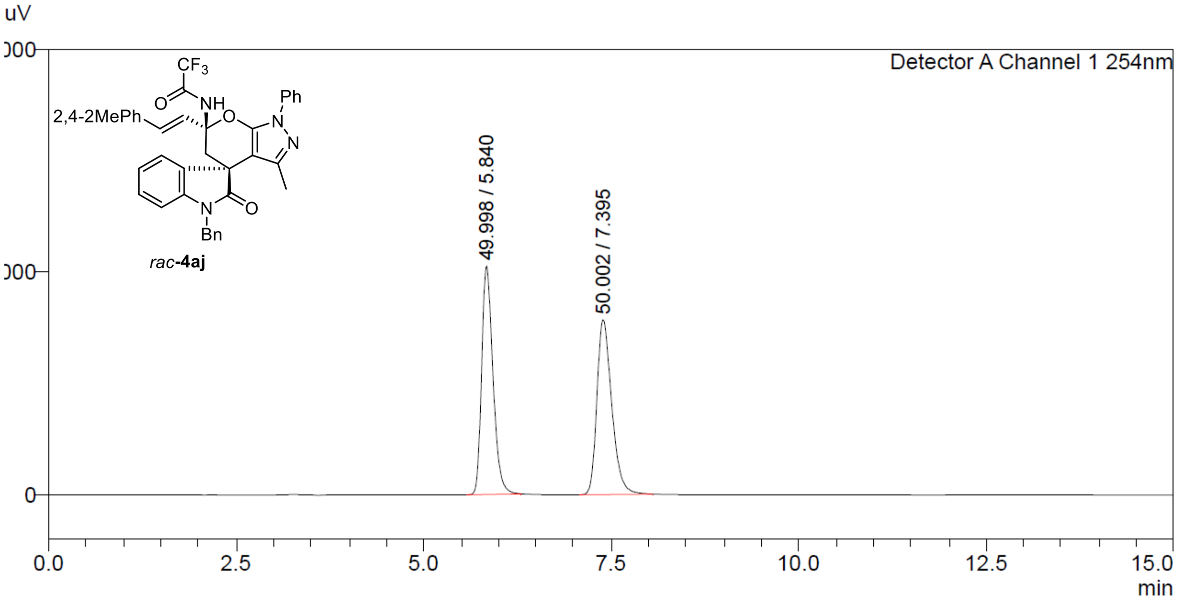

Peak Analysis Report

Detector A Channel 1 254nm

No.	Ret. Time	Height (mAu)	Area (mAu*min)	Rel. Area (%)
1	5.872	38334	363009	4.059
2	7.415	629855	8579684	95.941
Total		668188	8942692	100.000

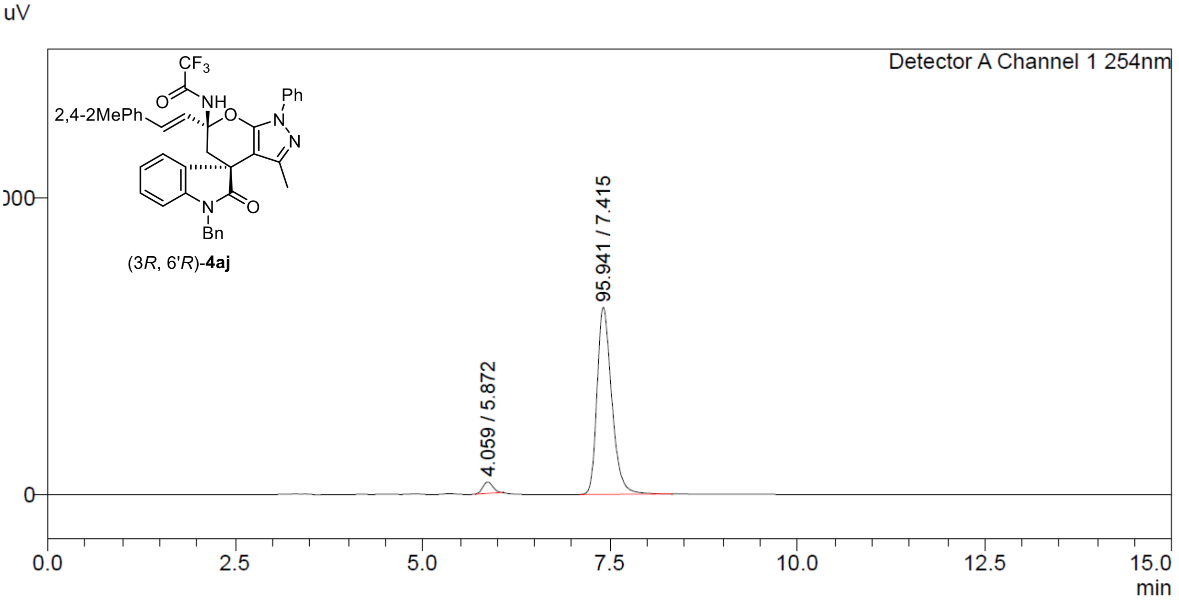

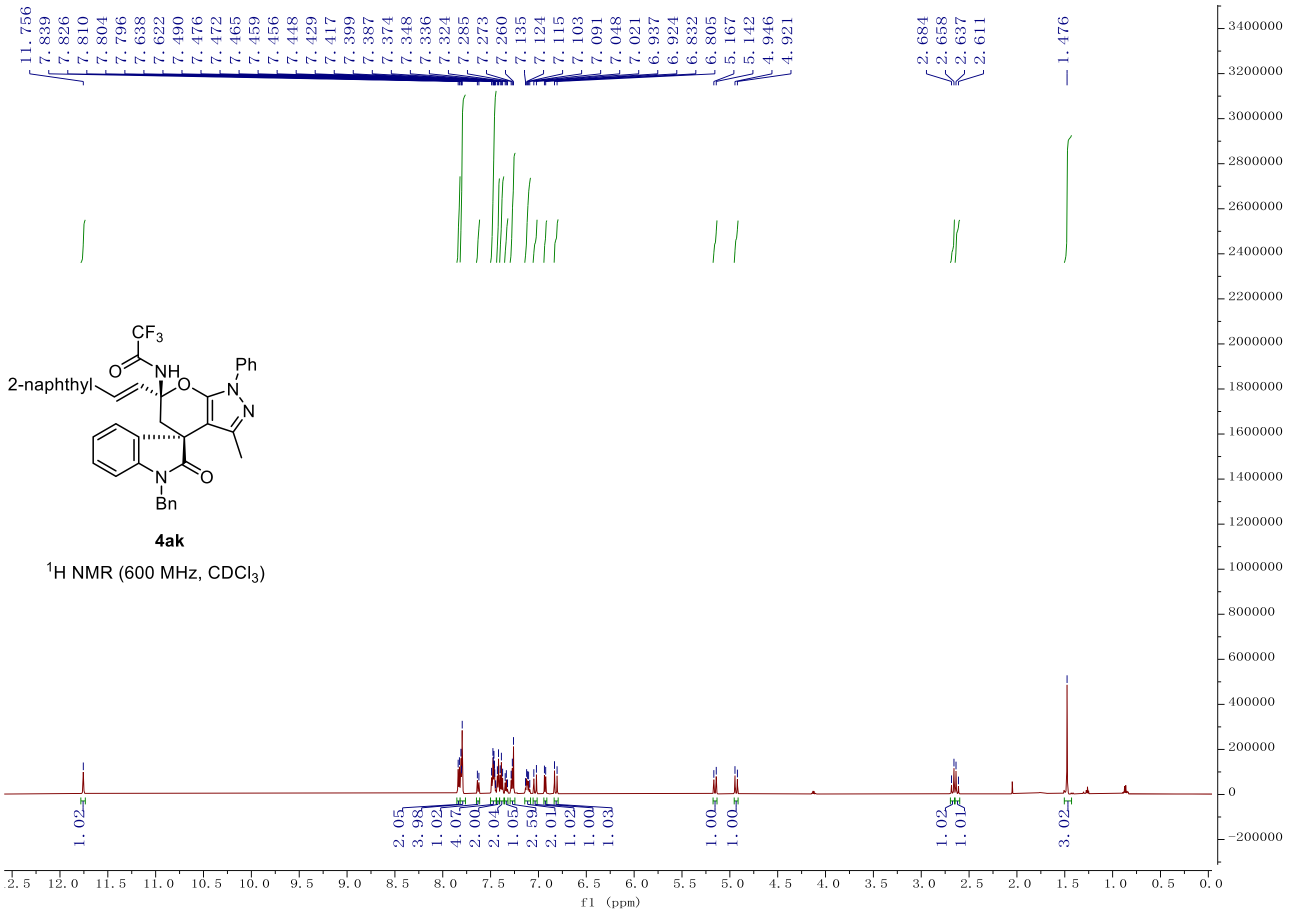

4ak

^{1}H NMR (600 MHz, $CDCl_3$)

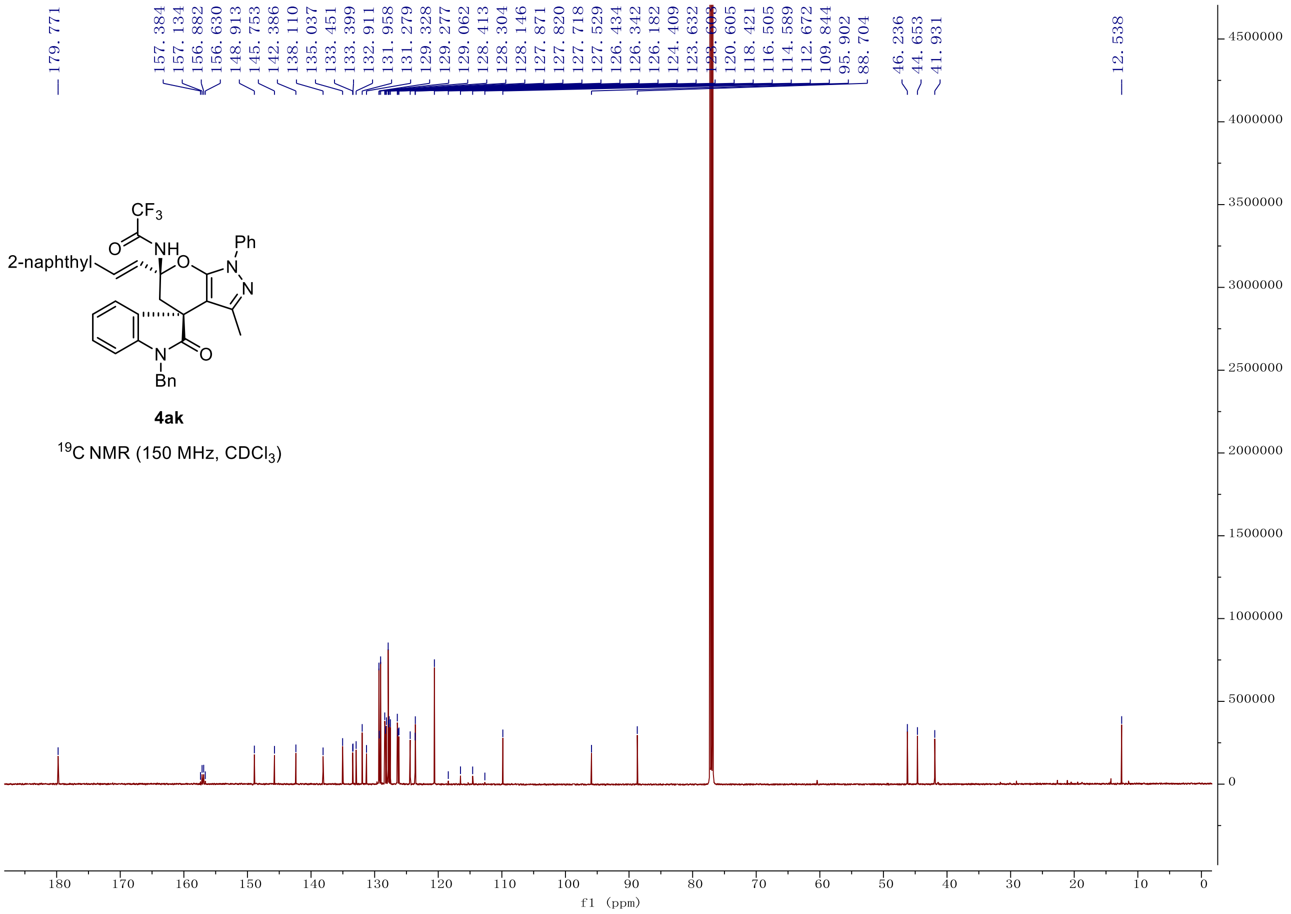

4ak

^{19}C NMR (150 MHz, $CDCl_3$)

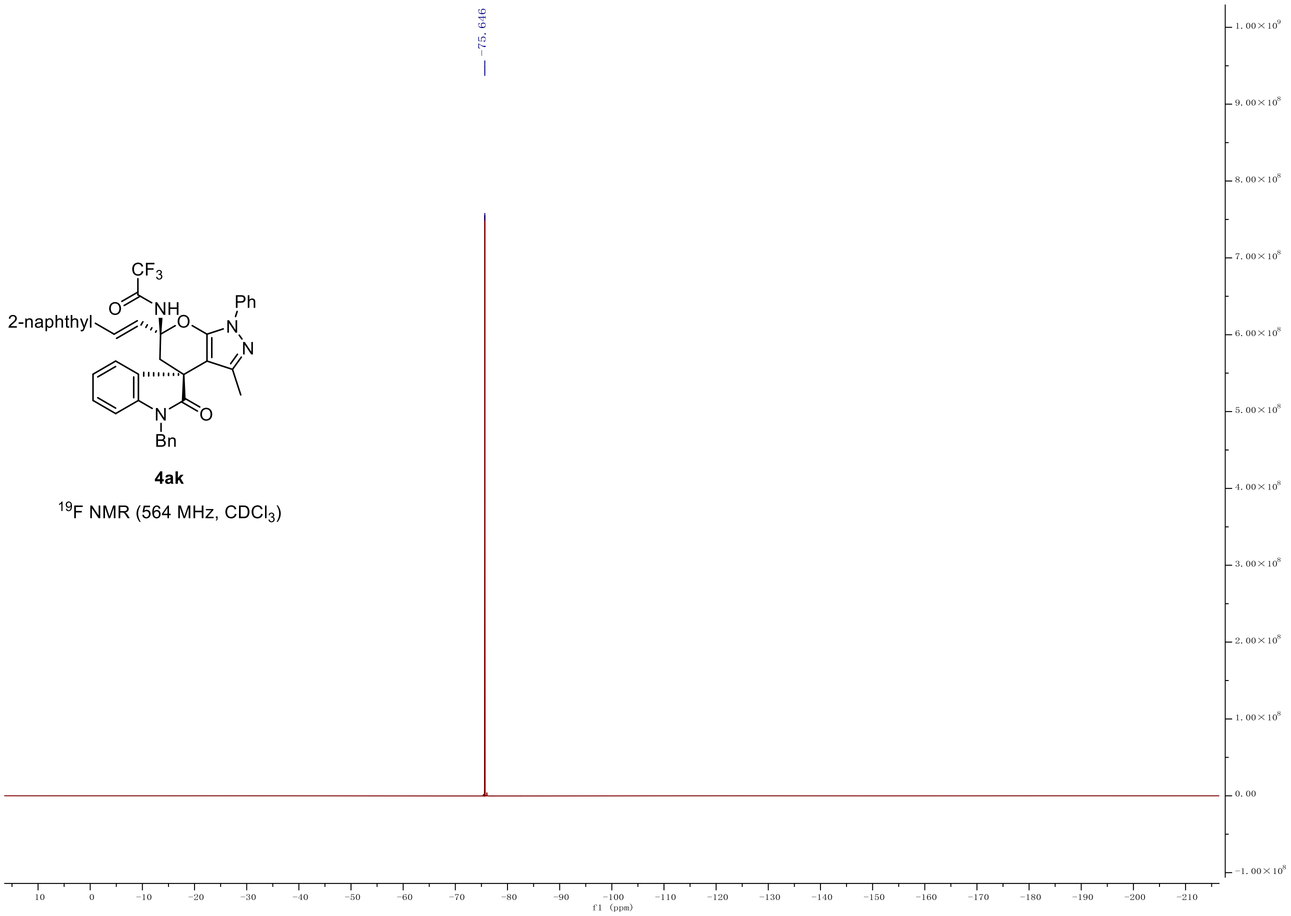

Peak Analysis Report

Detector A Channel 1 254nm

No.	Ret. Time	Height (mAu)	Area (mAu*min)	Rel. Area (%)
1	9.261	94307	1883519	50.436
2	12.306	55941	1850982	49.564
Total		150248	3734501	100.000

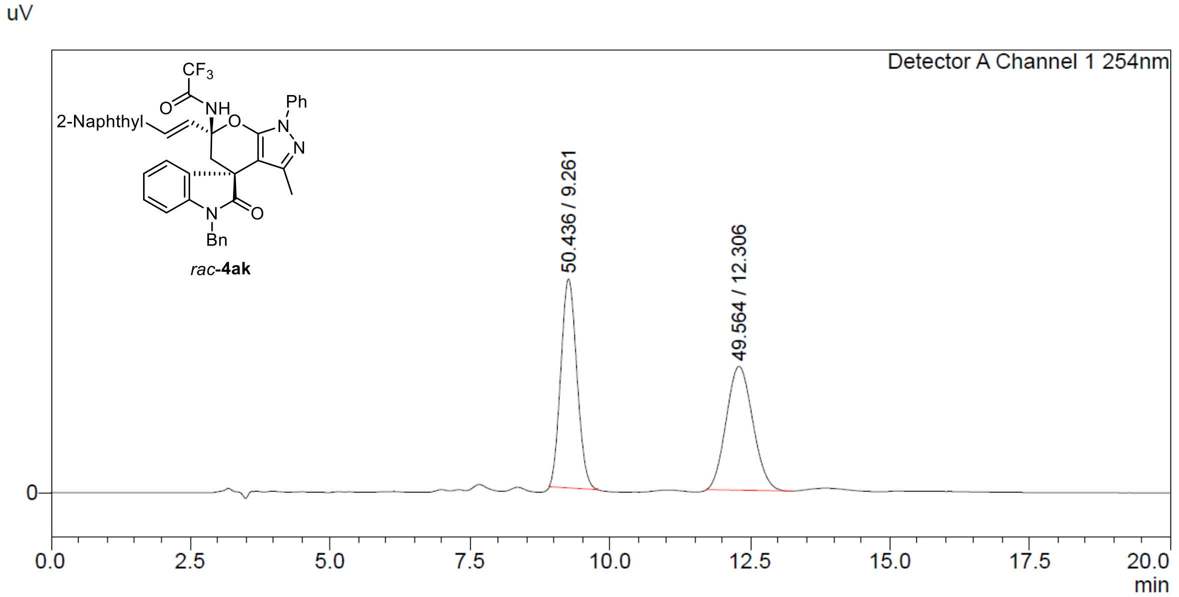

Peak Analysis Report

Detector A Channel 1 254nm

No.	Ret. Time	Height (mAu)	Area (mAu*min)	Rel. Area (%)
1	9.247	486534	9945419	98.297
2	12.292	5819	172331	1.703
Total		492354	10117750	100.000

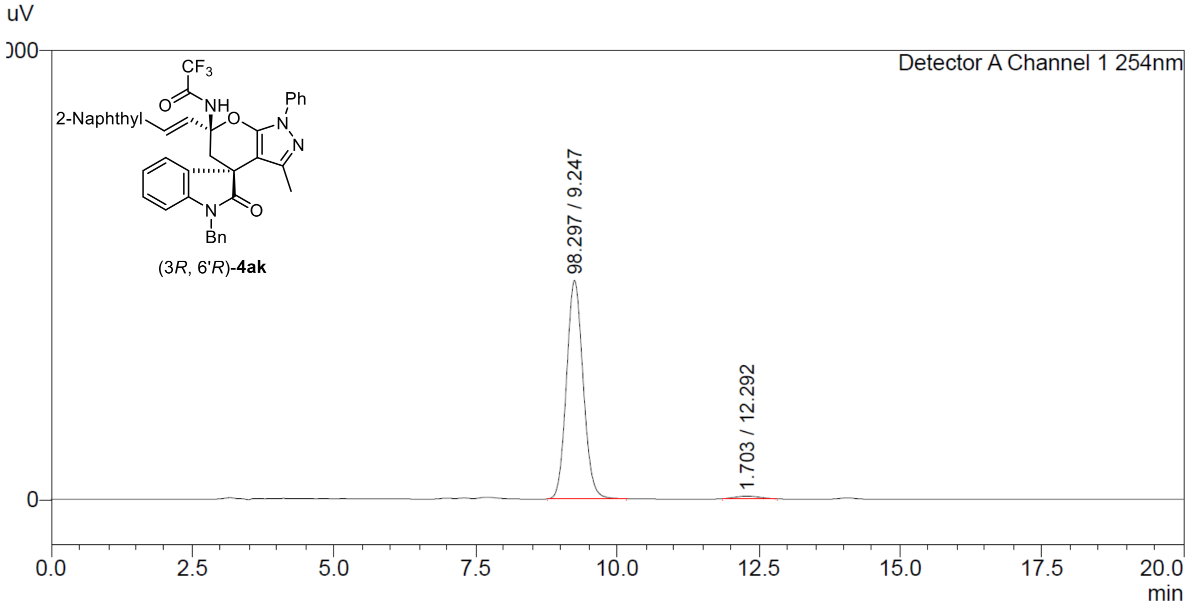

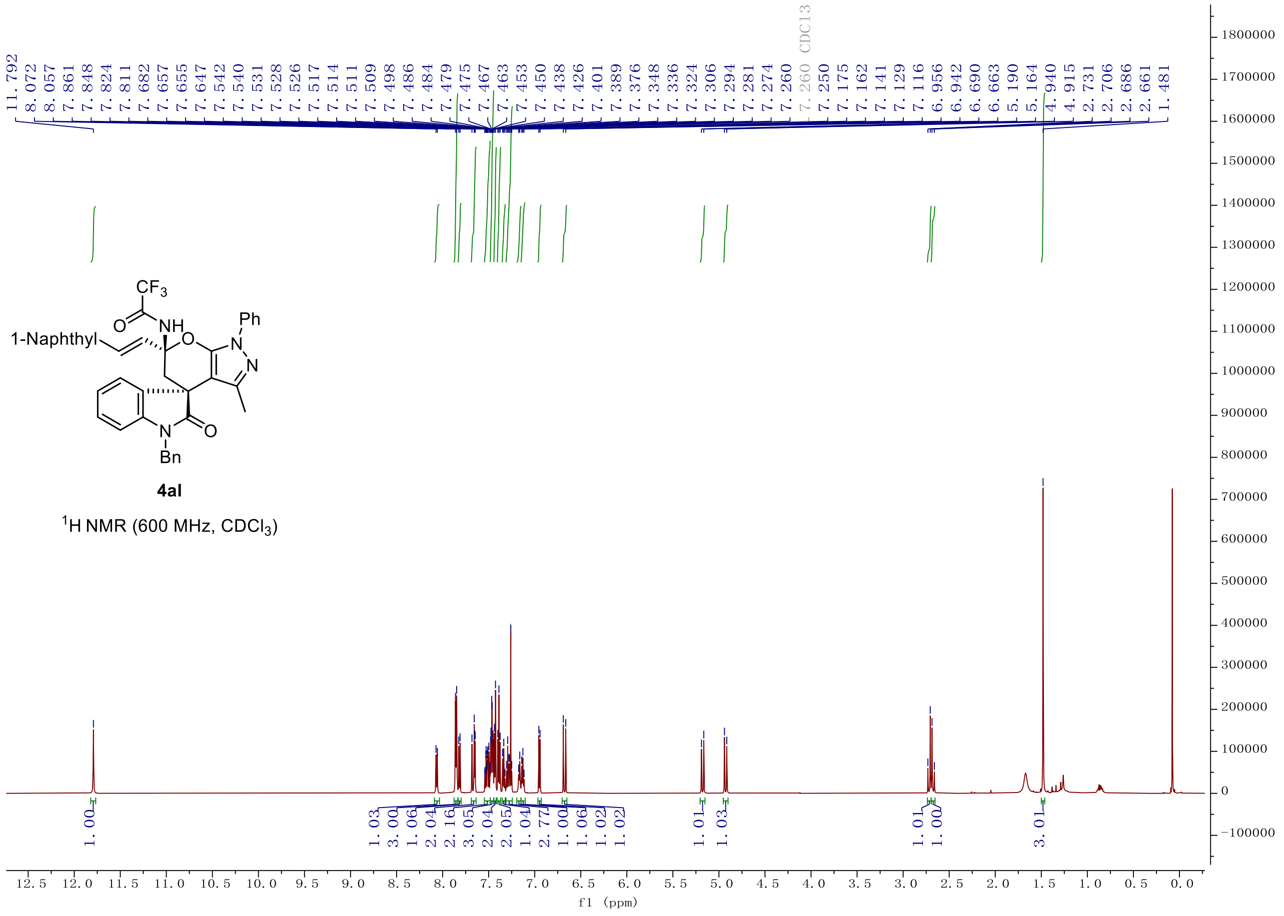

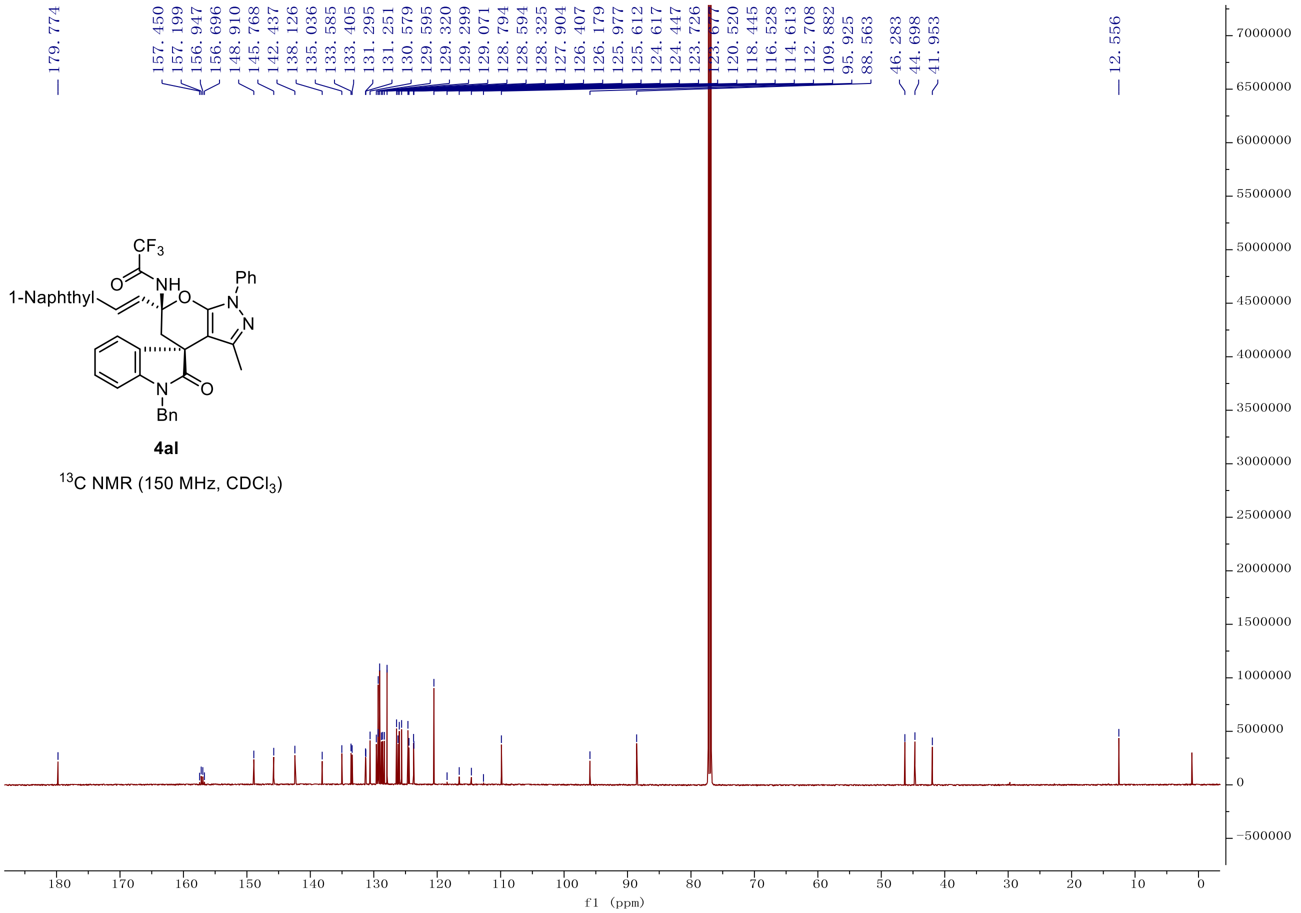

4al

^{13}C NMR (150 MHz, $CDCl_3$)

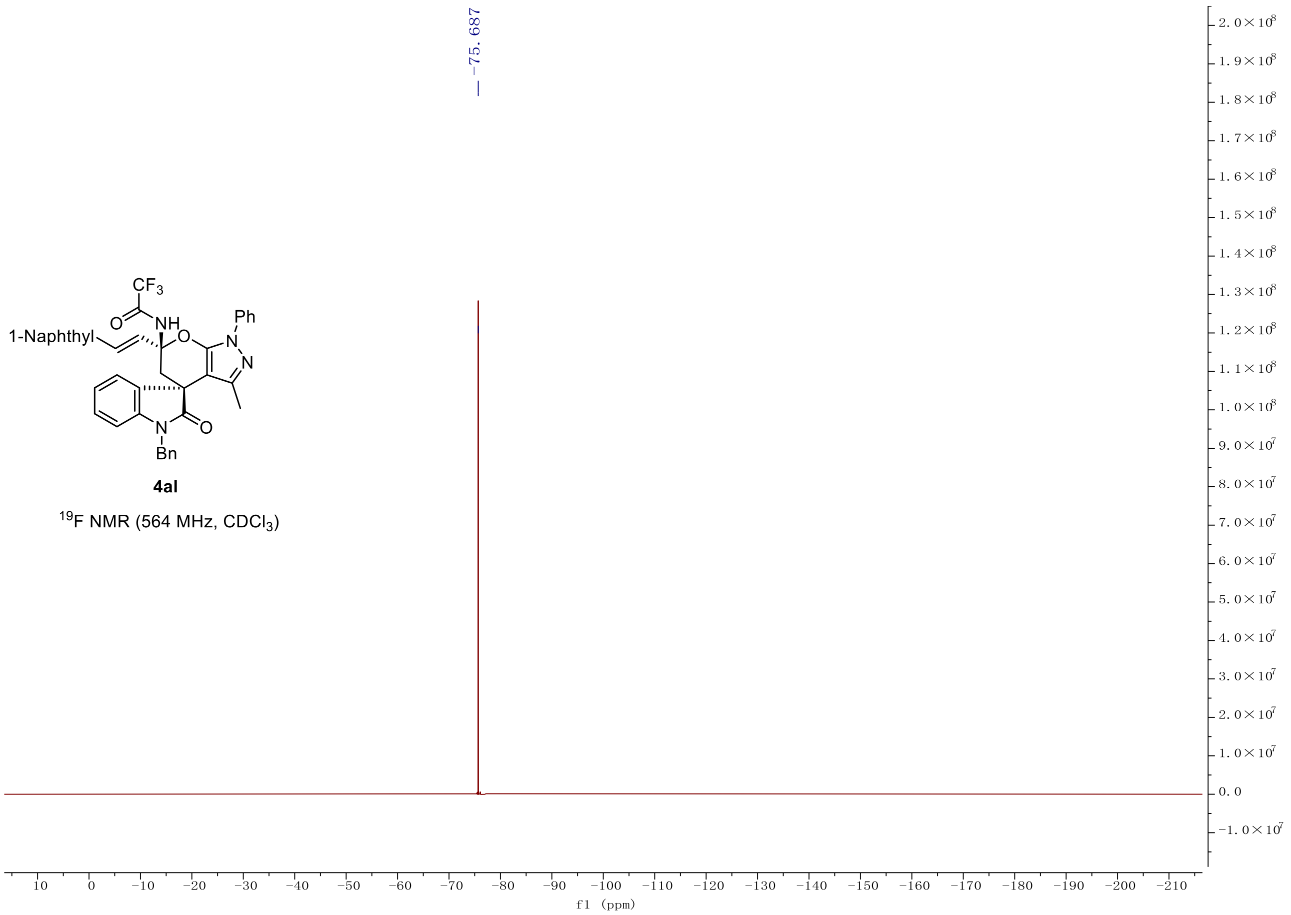

4al

^{19}F NMR (564 MHz, $CDCl_3$)

Signal: VWD1 B, Wavelength=254 nm

RT [min]Type	Area	Width[min]	Area%
8.393	2953.2983	0.243	50.0981
9.703	2941.7278	0.277	49.9019
总和	5895.0261		

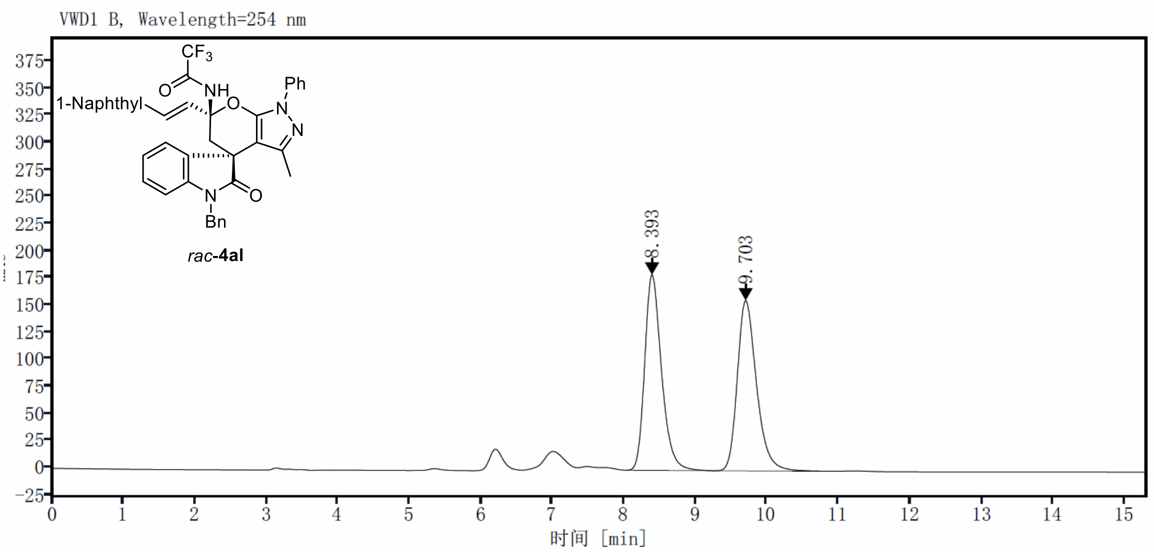

Signal: VWD1 A, Wavelength=254 nm

RT [min]Type	Area	Width[min]	Area%
8.290	448.6785	0.312	1.3366
10.068	33118.9063	0.351	98.6634
总和	33567.5848		

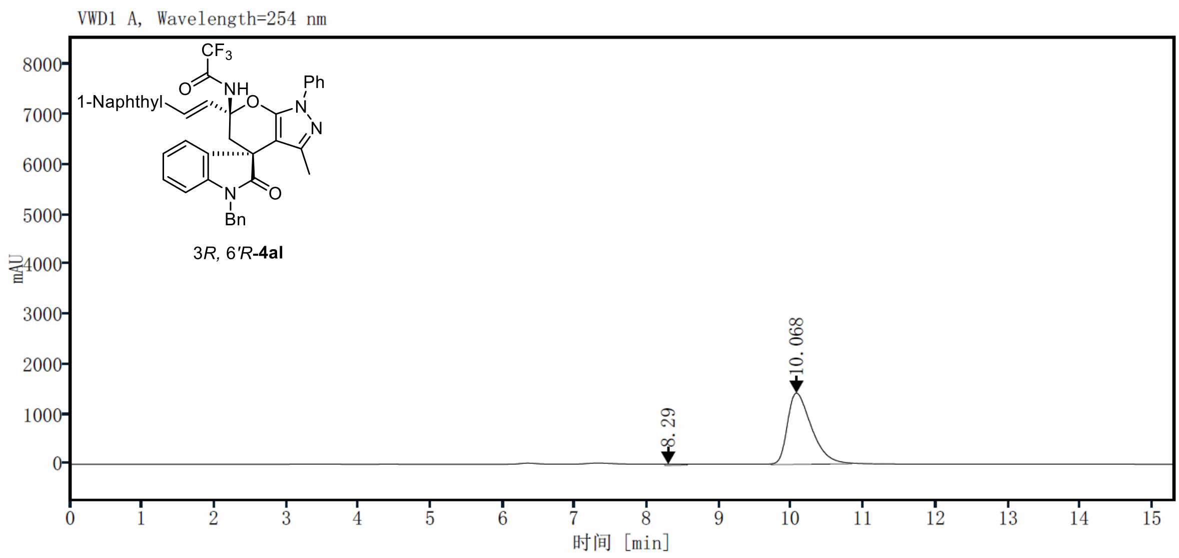

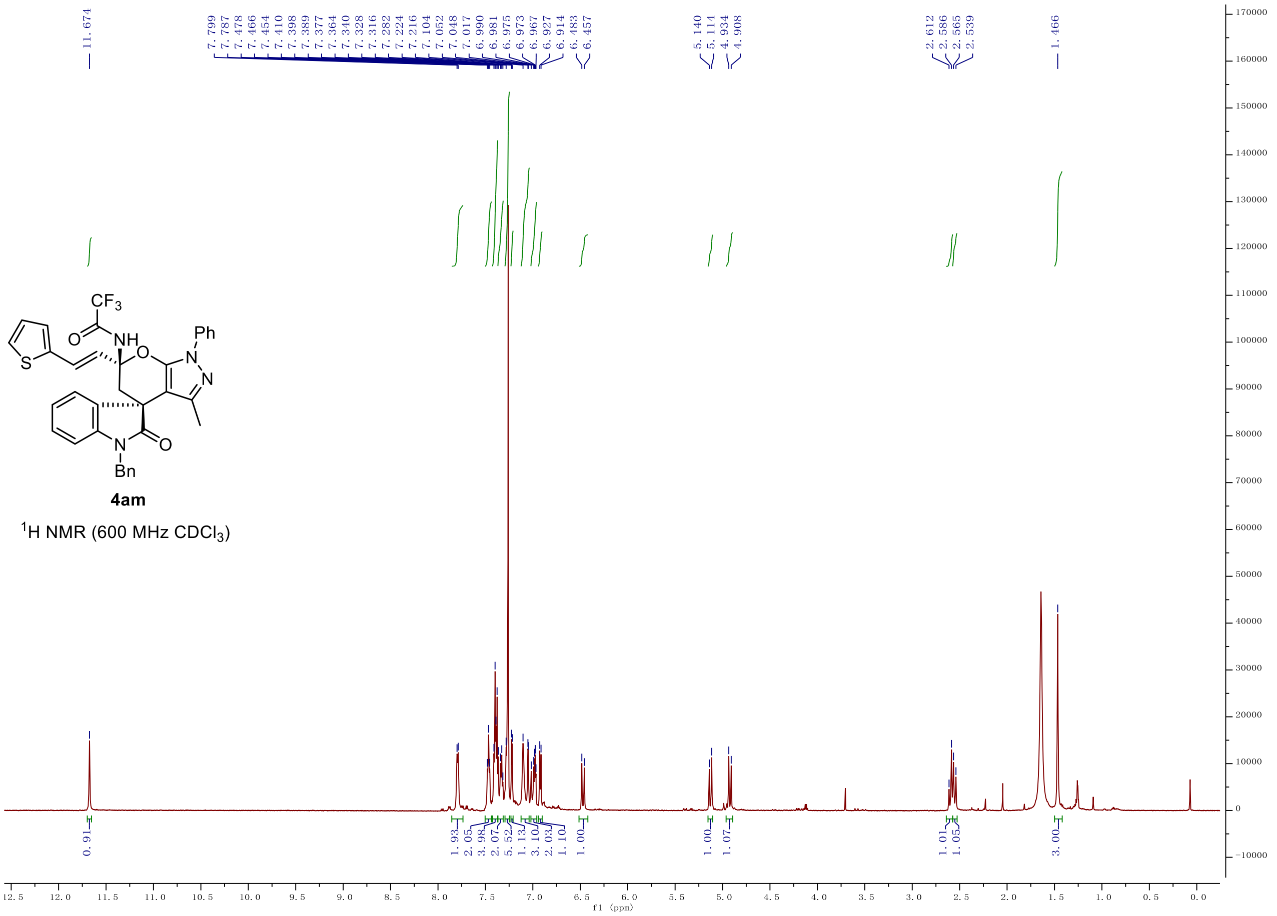

4am

^{1}H NMR (600 MHz $CDCl_3$)

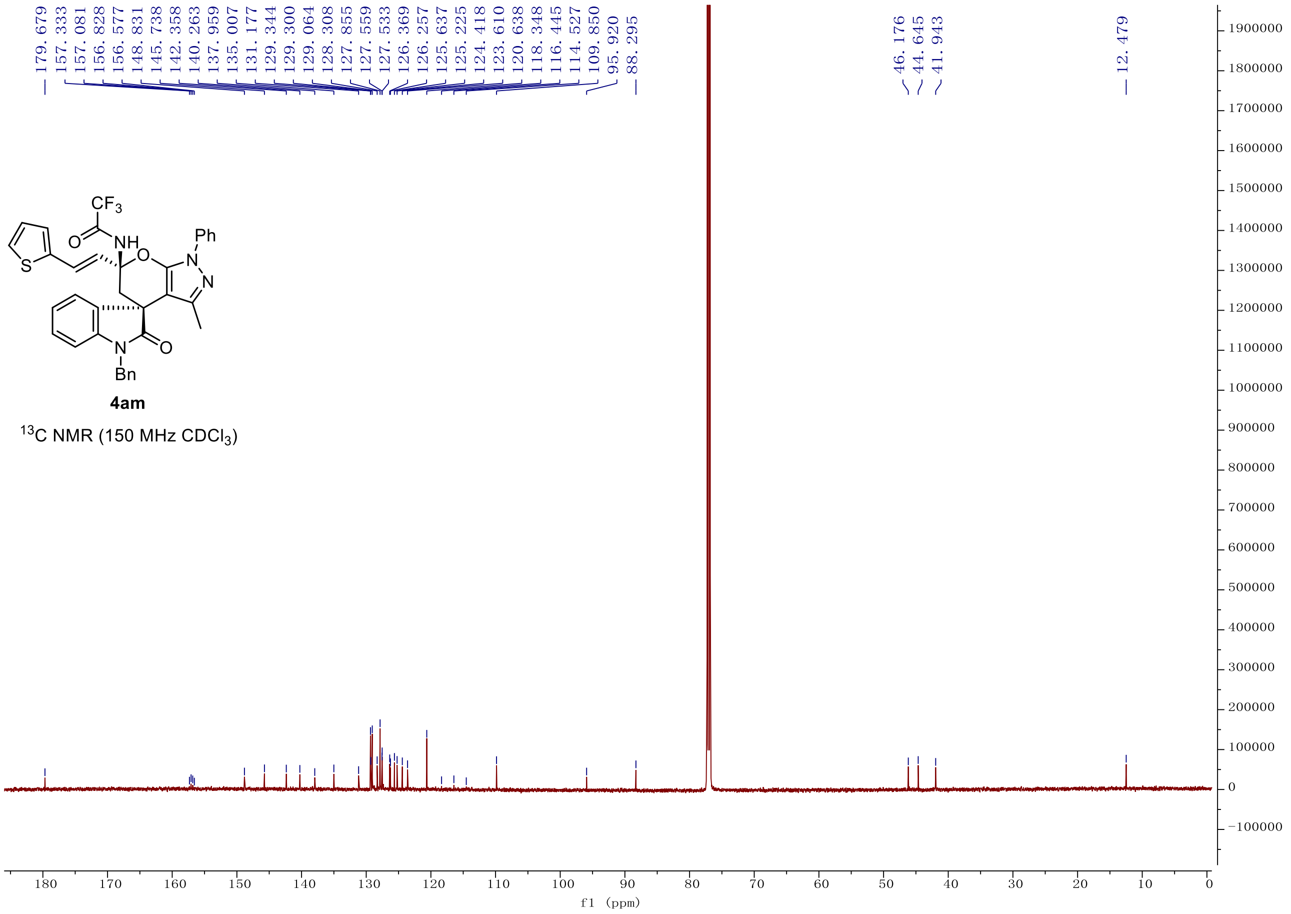

4am

^{13}C NMR (150 MHz $CDCl_3$)

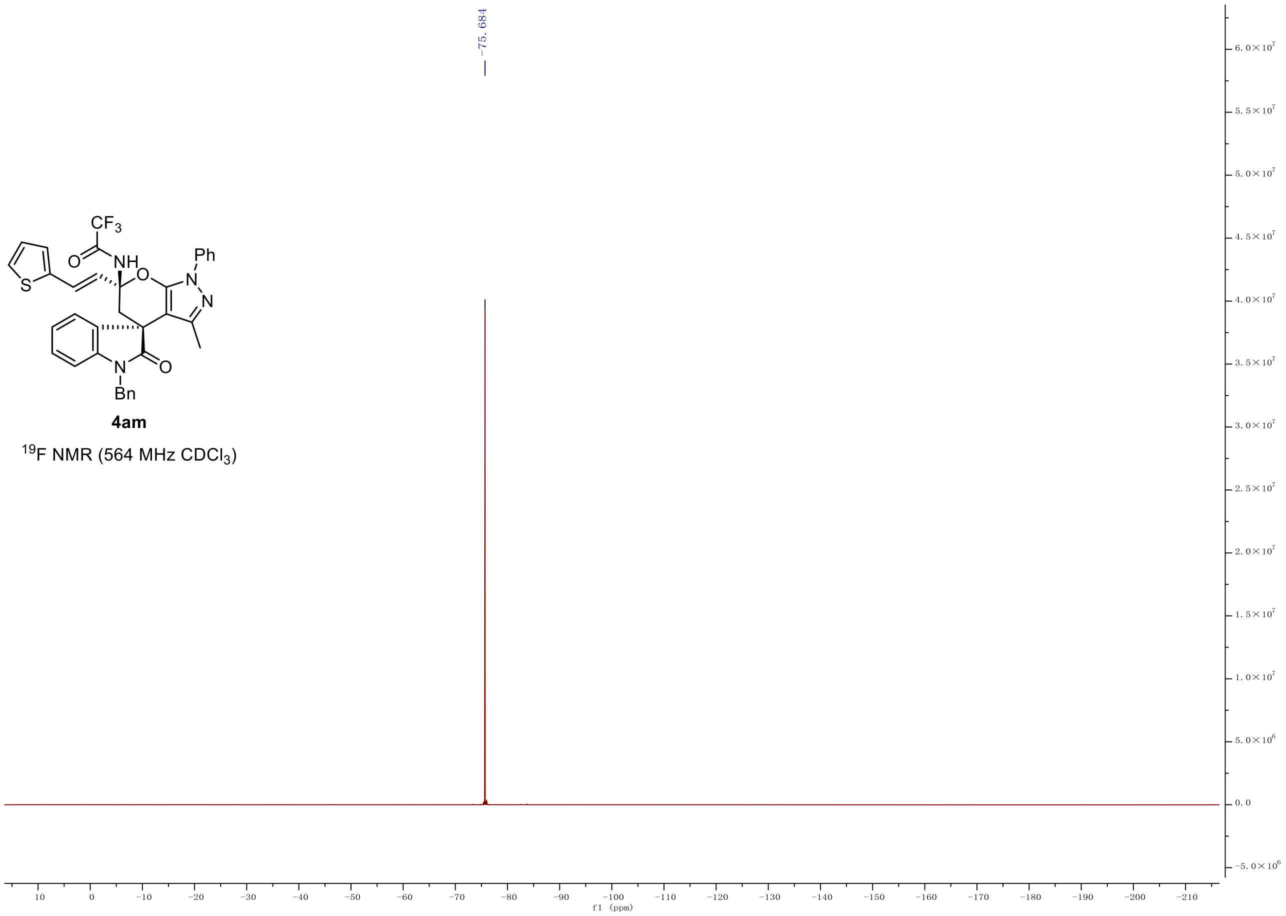

Peak Analysis Report

Detector A Channel 1 254nm

No.	Ret. Time	Height (mAu)	Area (mAu*min)	Rel. Area (%)
1	12.195	229252	5955737	49.076
2	15.187	167282	6180105	50.924
Total		396533	12135843	100.000

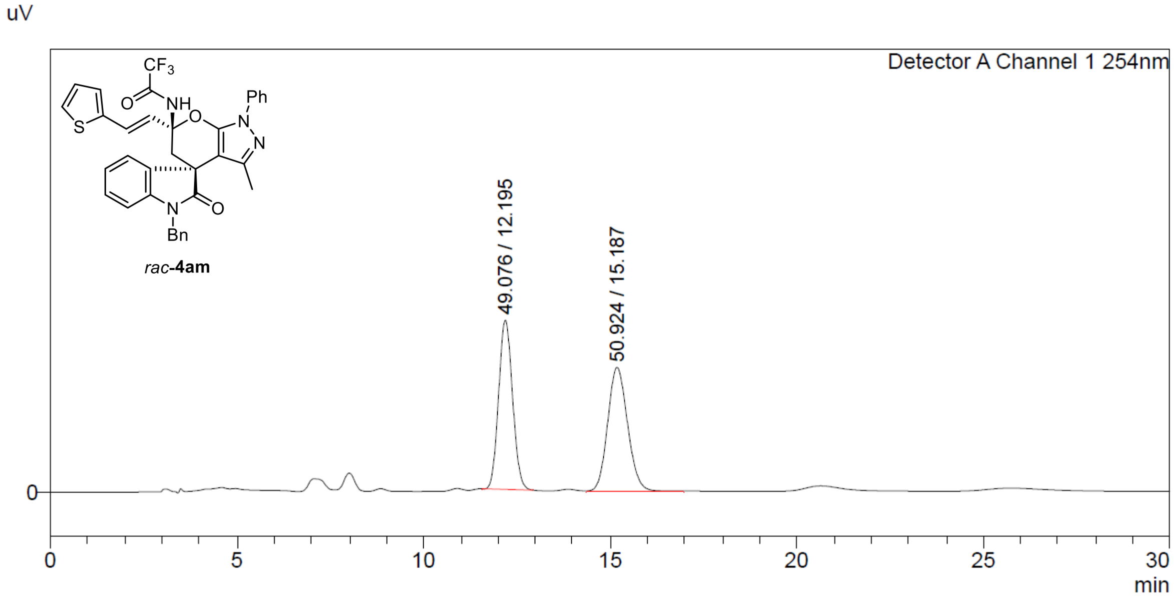

Peak Analysis Report

Detector A Channel 1 254nm

No.	Ret. Time	Height (mAu)	Area (mAu*min)	Rel. Area (%)
1	12.204	169929	4524535	98.727
2	15.236	1564	58317	1.273
Total		171493	4582852	100.000

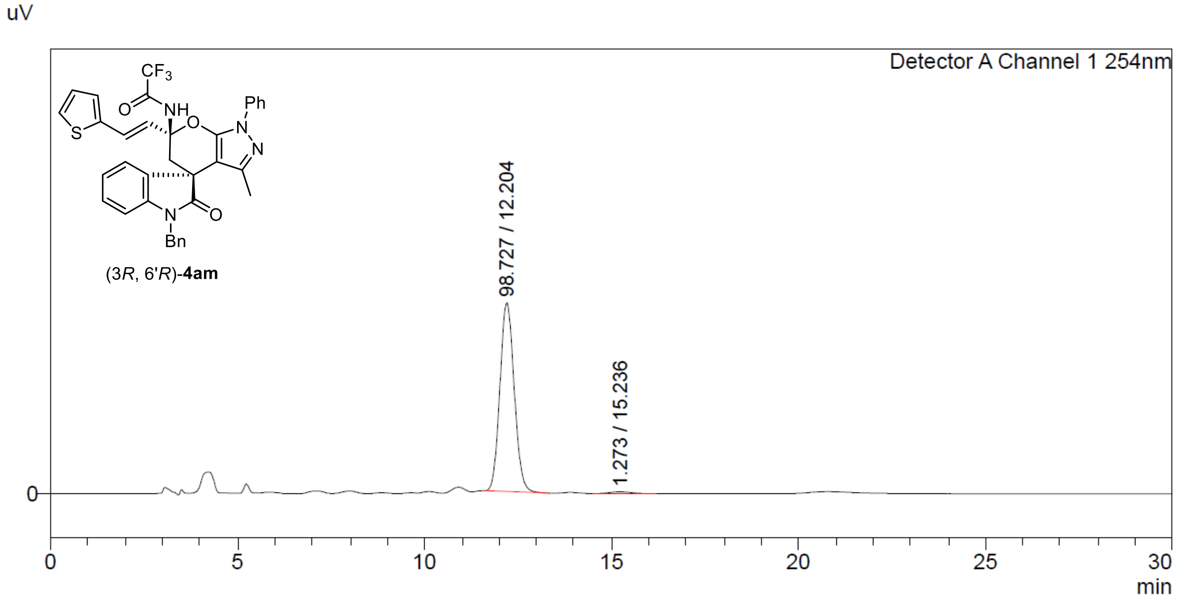

Peak Analysis Report

Detector A Channel 1 254nm

No.	Ret. Time	Height (mAu)	Area (mAu*min)	Rel. Area (%)
1	12.245	5292	71593	0.236
2	15.133	808319	30241325	99.764
Total		813610	30312918	100.000

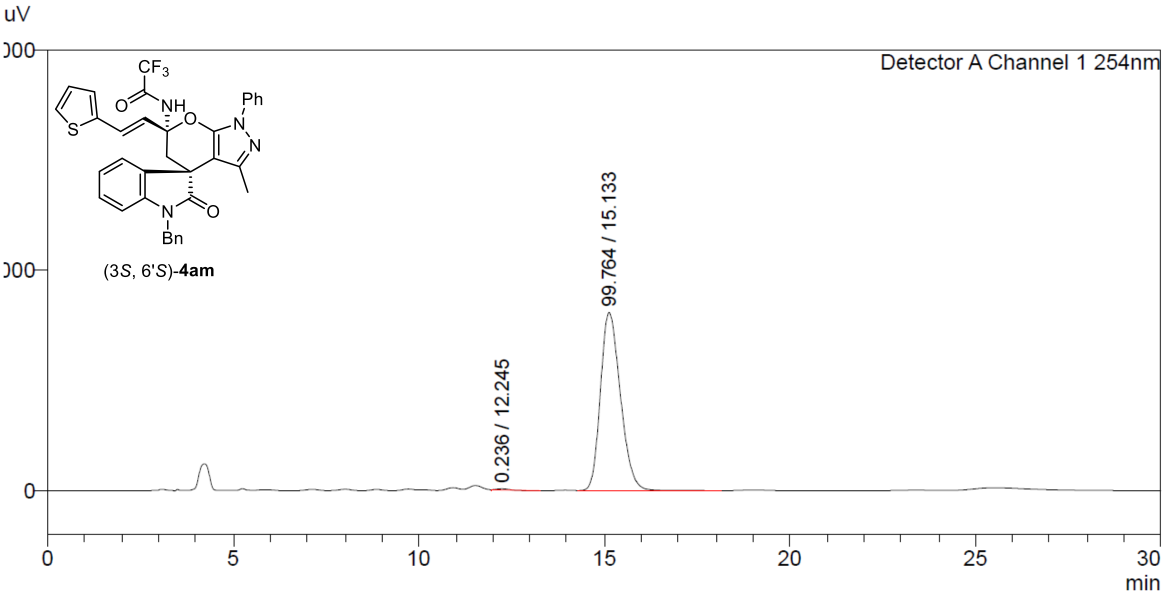

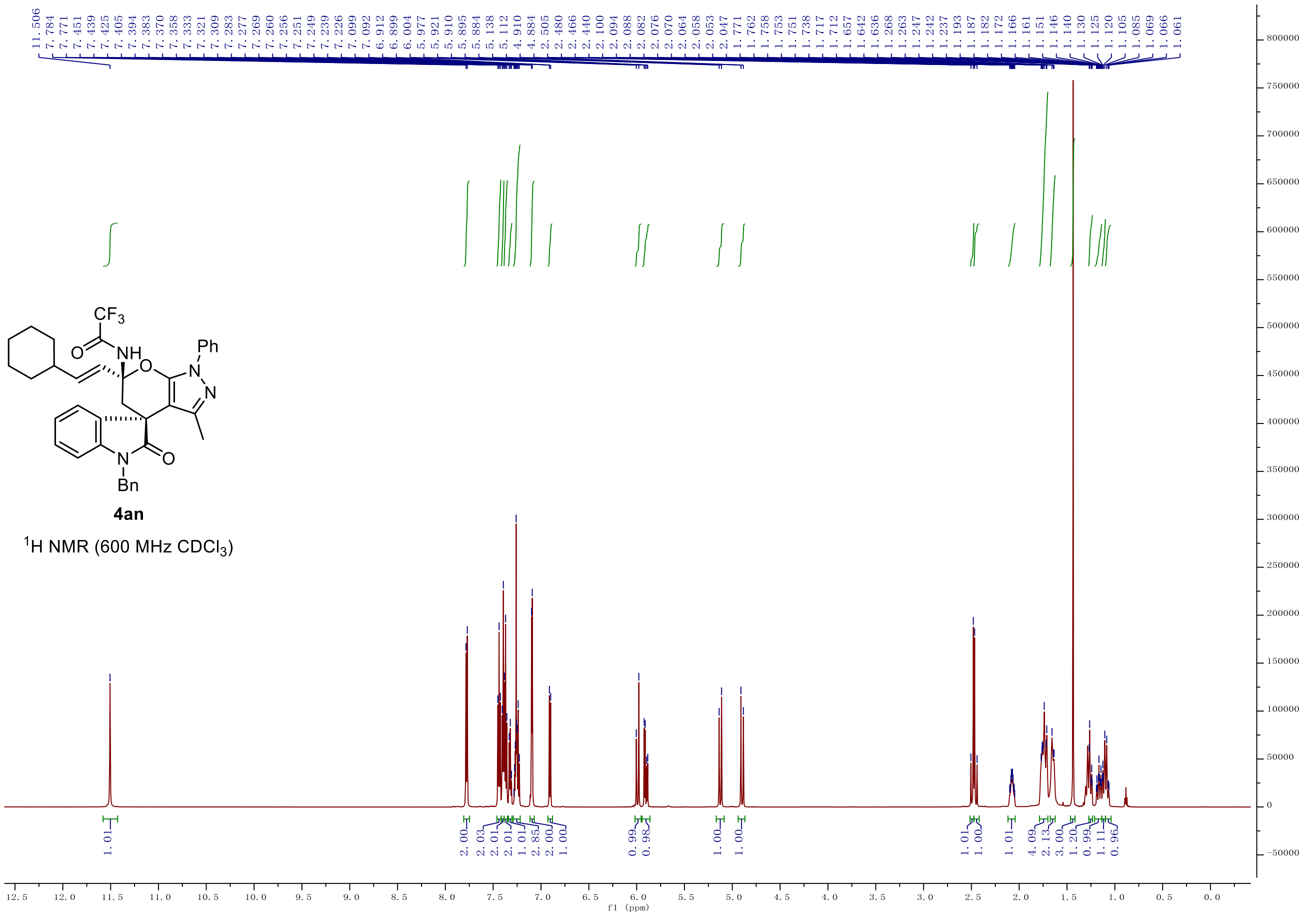
11.506 7.784 7.771 7.451 7.439 7.425 7.405 7.394 7.383 7.370 7.358 7.333 7.321 7.309 7.283 7.277 7.269 7.260 7.256 7.251 7.249 7.239 7.226 7.099 7.092 6.912 6.899 6.004 5.977 5.921 5.910 5.895 5.884 5.138 5.112 4.910 4.884 2.505 2.480 2.466 2.440 2.100 2.094 2.088 2.082 2.076 2.070 2.064 2.058 2.053 2.047 1.771 1.762 1.758 1.753 1.751 1.738 1.717 1.712 1.657 1.642 1.636 1.268 1.263 1.247 1.242 1.237 1.193 1.187 1.182 1.172 1.166 1.161 1.151 1.146 1.140 1.130 1.125 1.120 1.105 1.085 1.069 1.066 1.061
CF3
O
NH
O
Ph
N
N
N
O
Bn
4an
^{1}H NMR (600 MHz CDCl$_3$)
1.01 2.00 2.03 2.01 2.01 1.01 2.85 2.00 1.00 0.99 0.98 1.00 1.00 1.01 1.00 1.01 4.09 2.13 3.00 1.20 0.99 1.11 0.96
f1 (ppm)

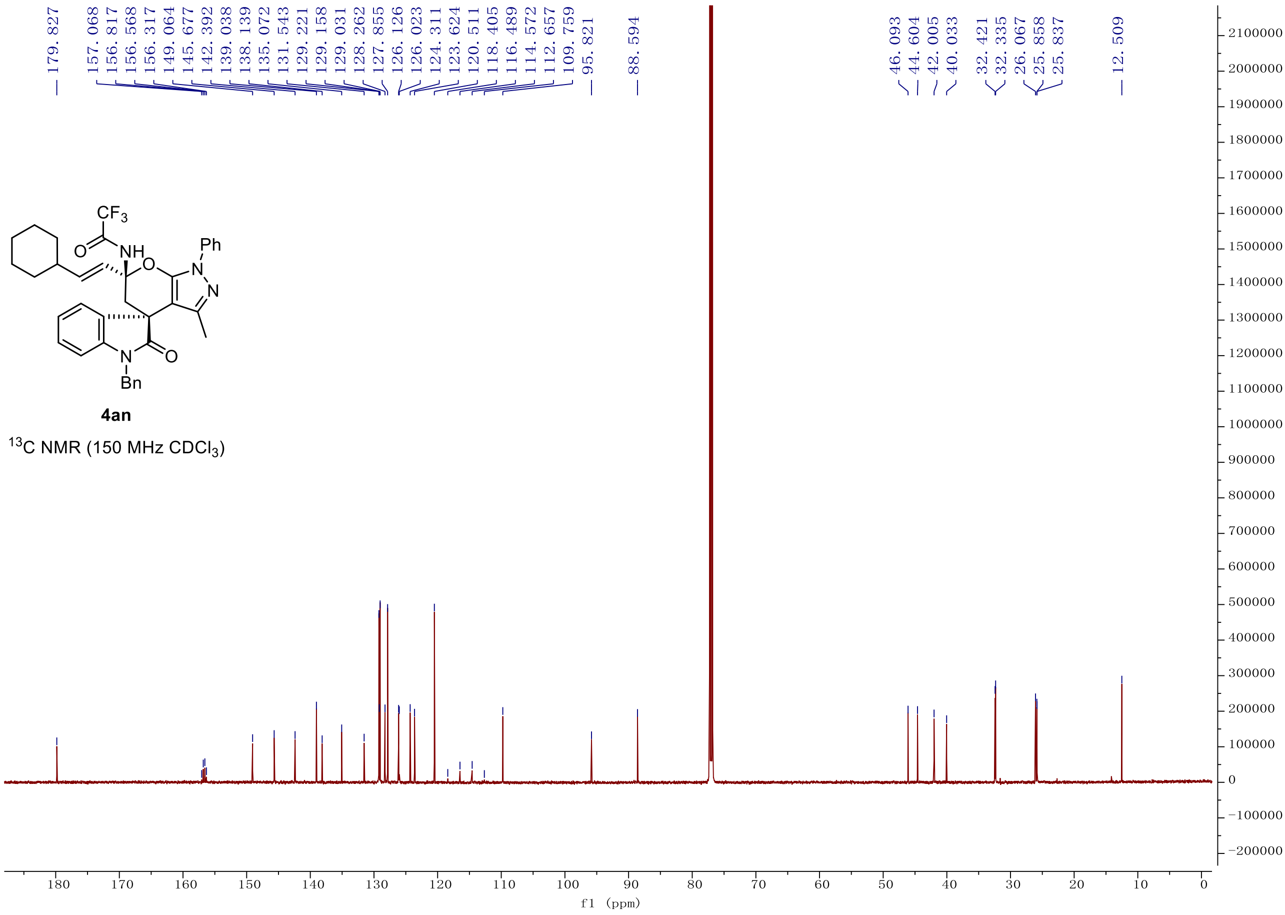
179.827 157.068 156.817 156.568 156.317 149.064 145.677 142.392 139.038 138.139 135.072 131.543 129.221 129.158 129.031 128.262 127.855 126.126 126.023 124.311 123.624 120.511 118.405 116.489 114.572 112.657 109.759 95.821 88.594 46.093 44.604 42.005 40.033 32.421 32.335 26.067 25.858 25.837 12.509
CF3
O
NH
O
Ph
N
N
N
O
Bn
4an
^{13}C NMR (150 MHz CDCl$_3$)
f1 (ppm)

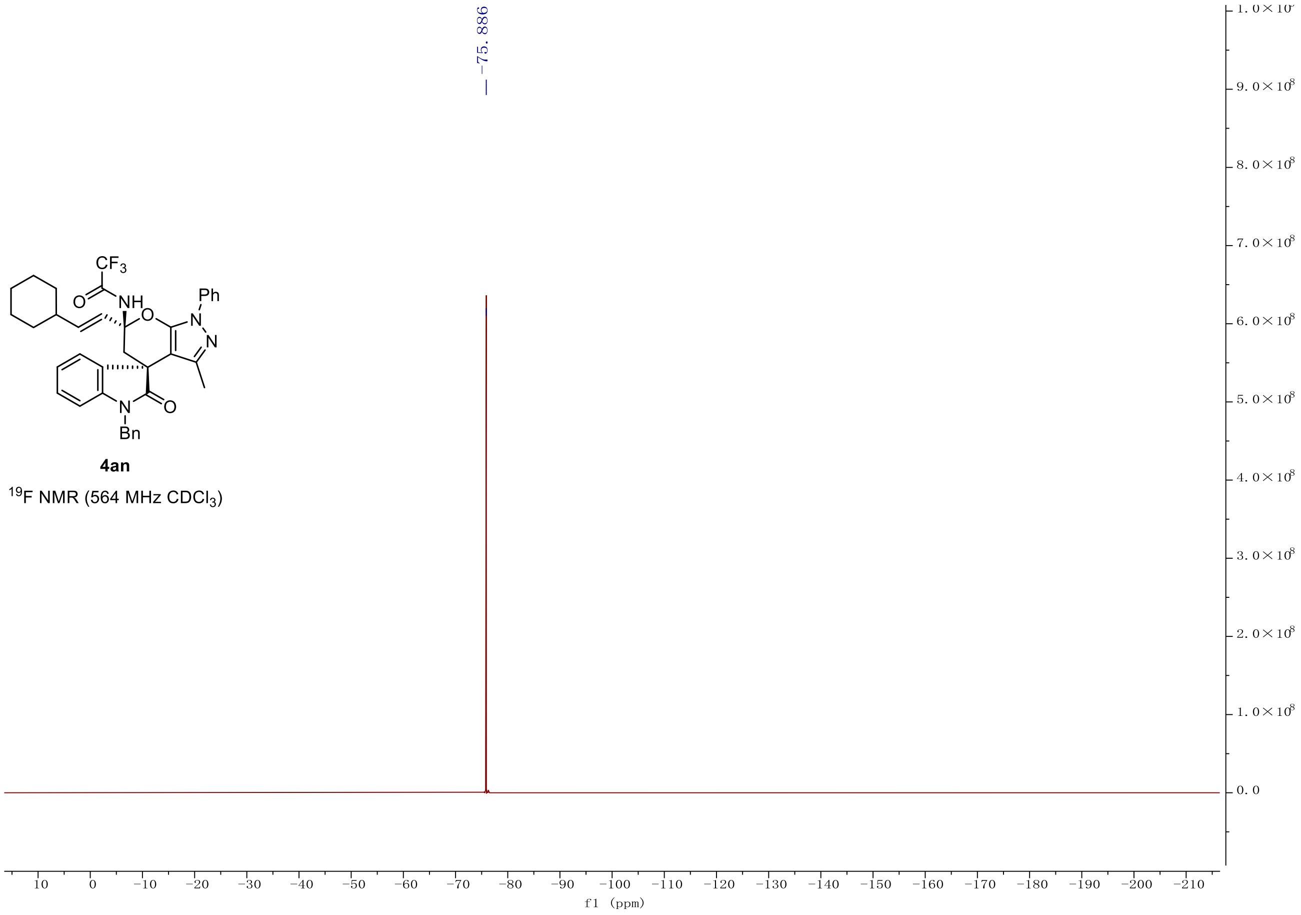

信号: VWD1 B, Wavelength=254 nm

保留时间 [min]	峰面积	峰宽 (50%)	峰面积%
4.898	10478.6191	0.166	49.5598
7.141	10664.7793	0.297	50.4402
总和	21143.3984		

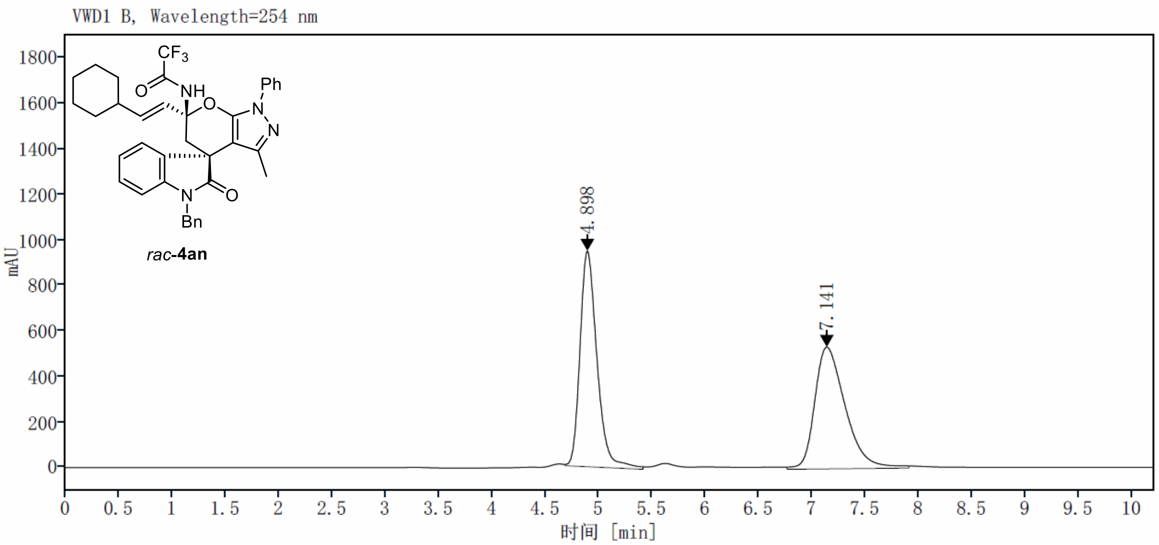

信号:　　VWD1 B, Wavelength=254 nm

保留时间 [min]	峰面积	峰宽 (50%)	峰面积%
5.059	4030.9563	0.150	27.5188
7.270	10617.0439	0.222	72.4812
**总和**	**14648.0002**		

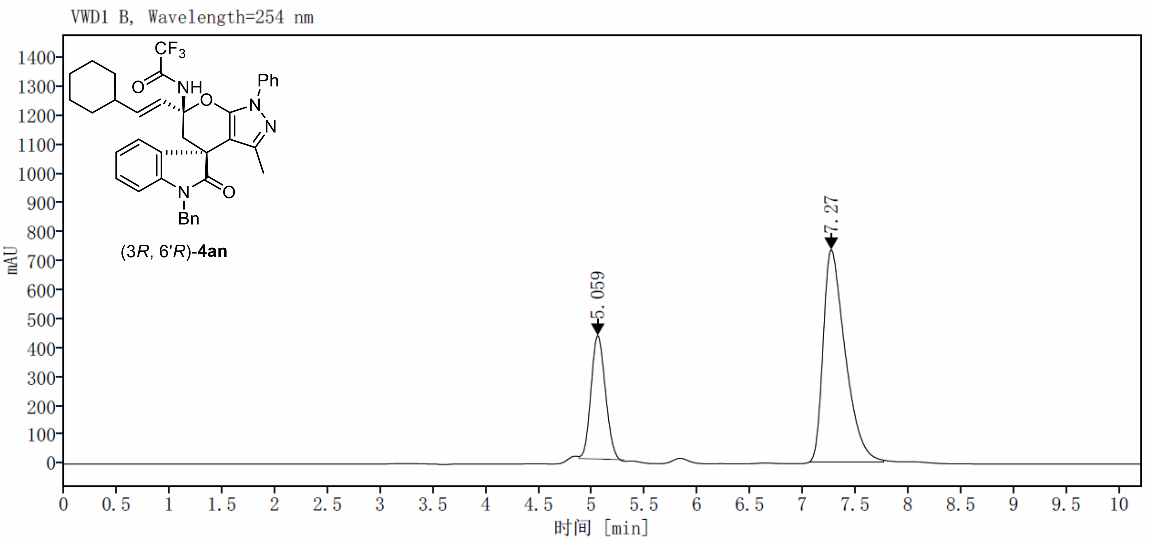

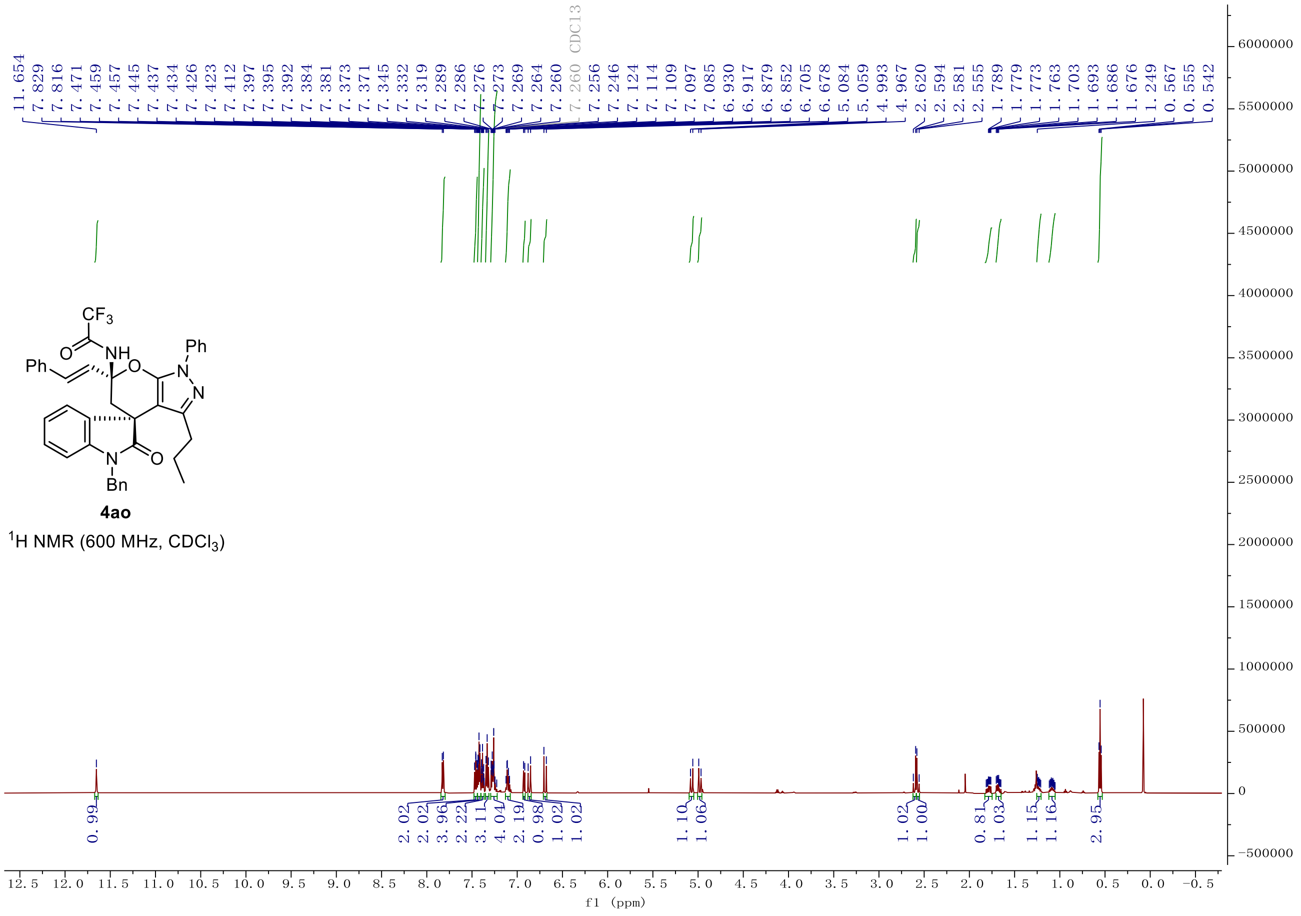

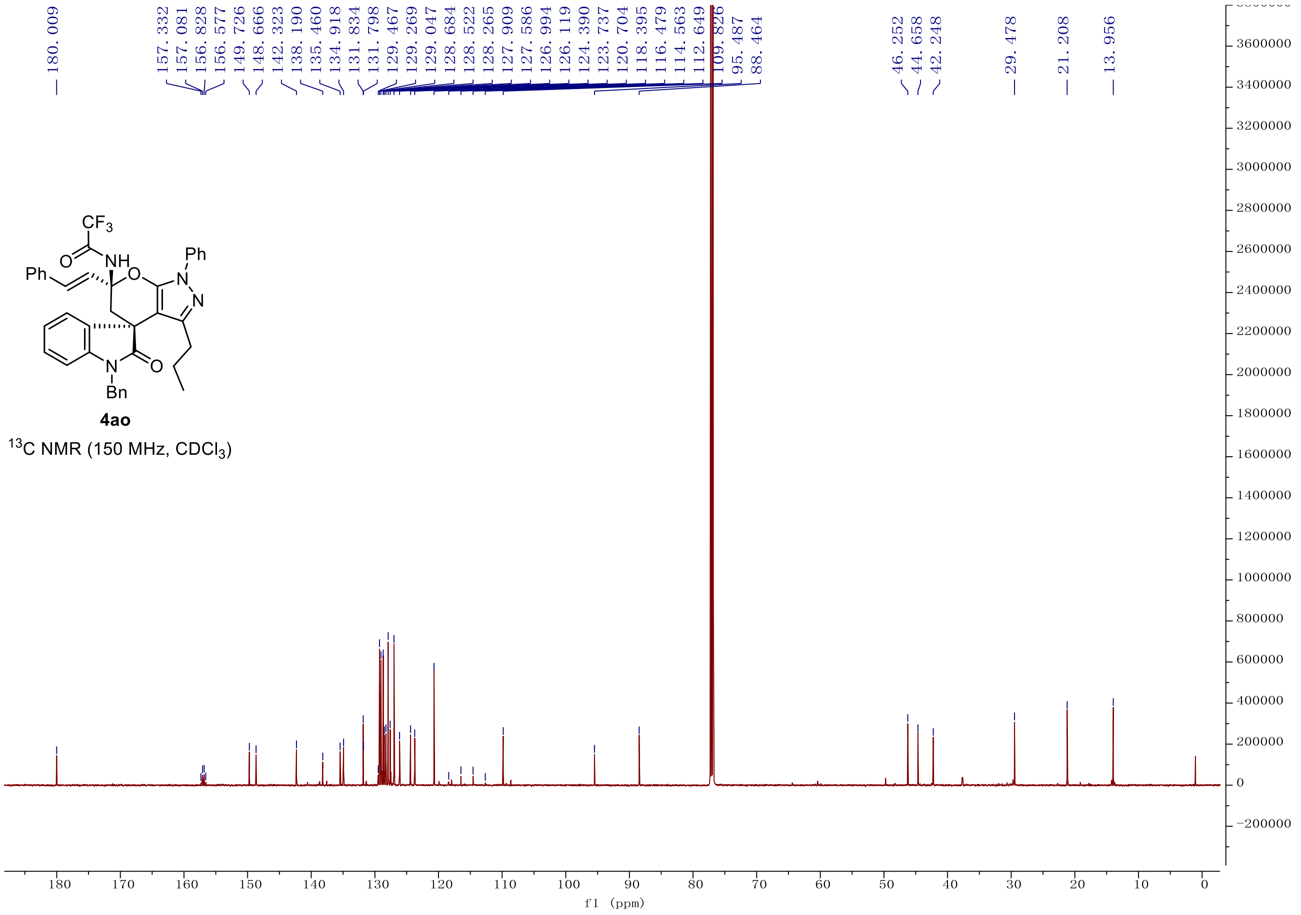

4ao

^{13}C NMR (150 MHz, $CDCl_3$)

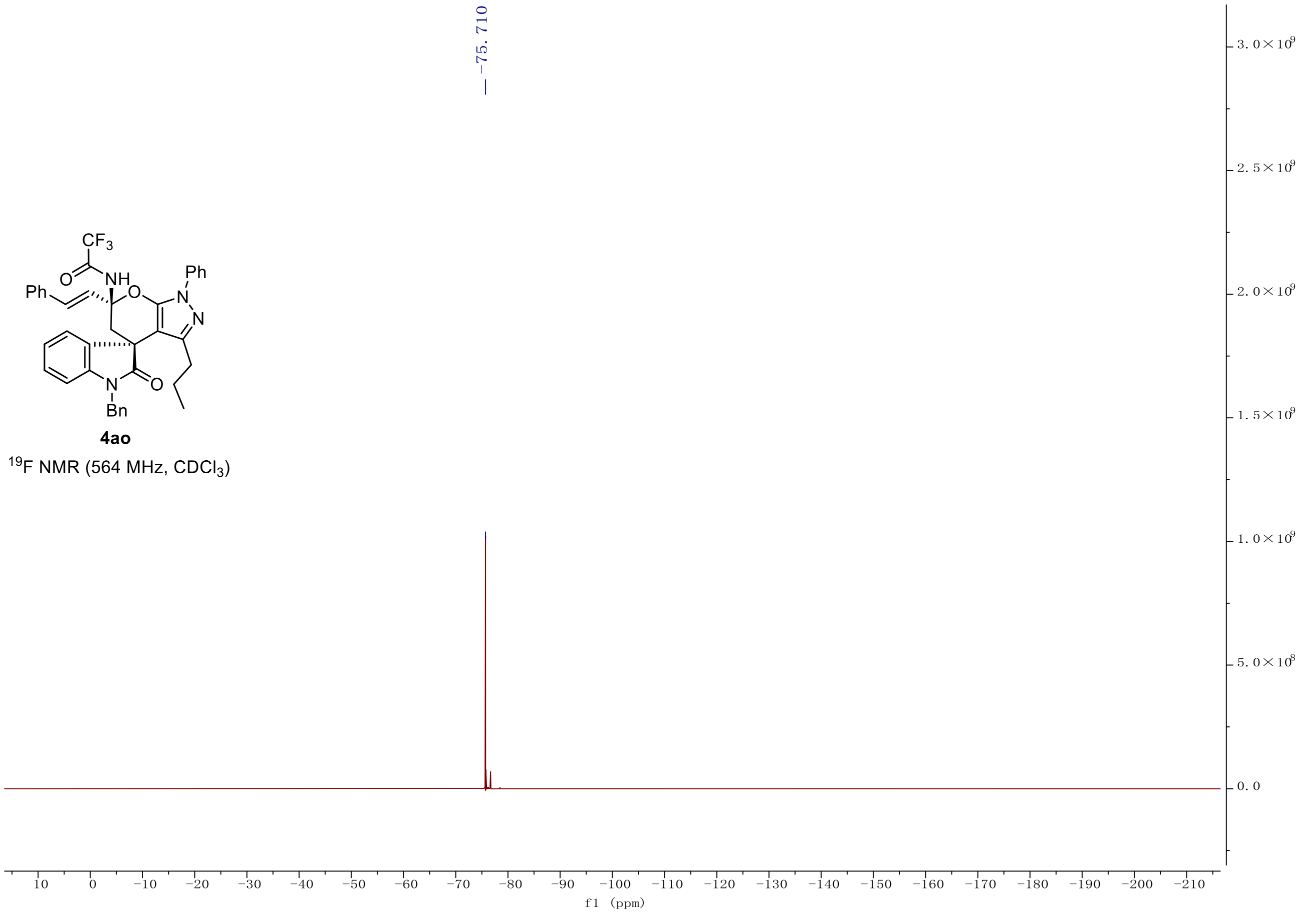

4ao

^{19}F NMR (564 MHz, $CDCl_3$)

Signal: VWD1 B, Wavelength=254 nm

RT [min]Type	Area	Width[min]	Area%
7.103	15159.8662	0.244	50.2360
7.821	15017.4082	0.182	49.7640
总和	30177.2744		

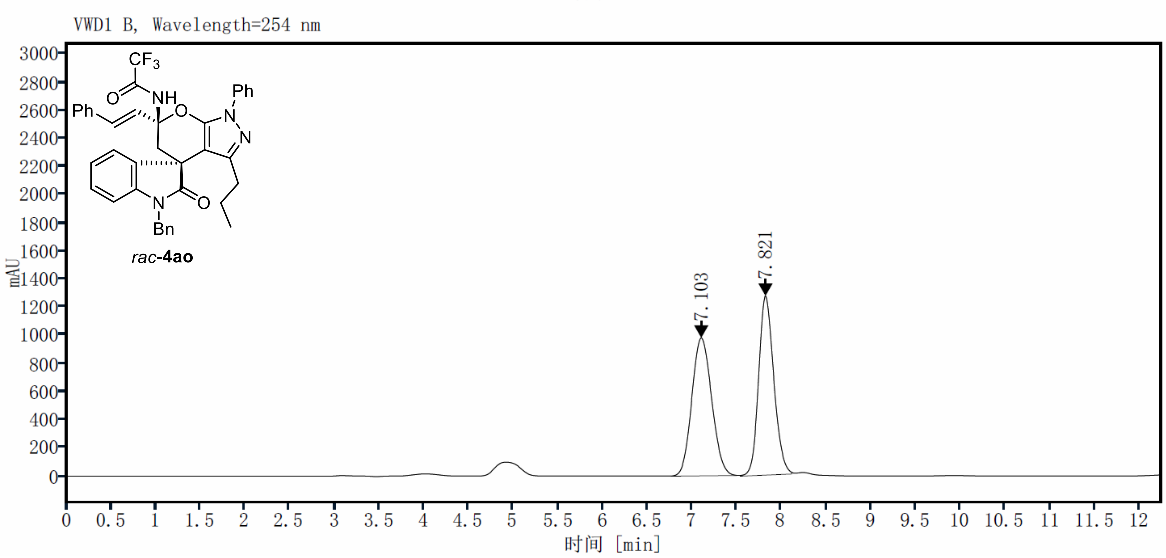

Signal: VWD1 B, Wavelength=254 nm

RT [min]Type	Area	Width[min]	Area%
7.072	12679.1309	0.235	85.4553
7.776	2158.0125	0.209	14.5447
总和	14837.1433		

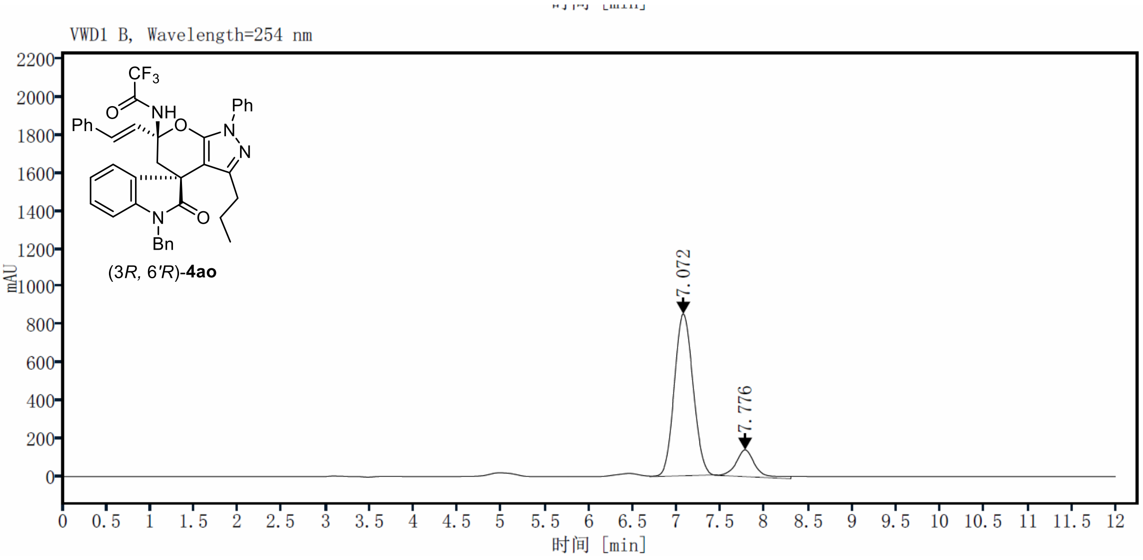

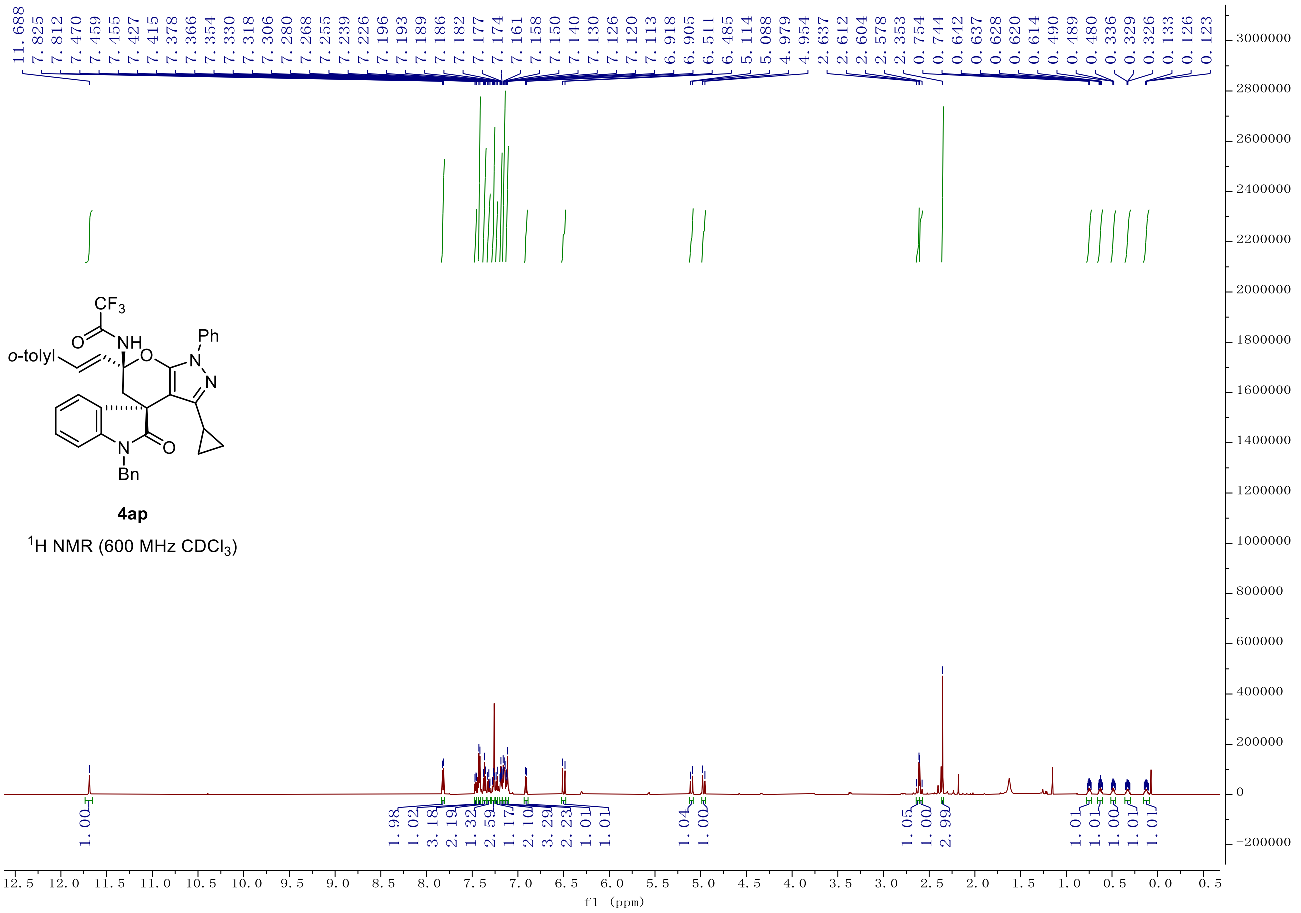
11.688
7.825
7.812
7.470
7.459
7.455
7.427
7.415
7.378
7.366
7.354
7.330
7.318
7.306
7.280
7.268
7.255
7.239
7.226
7.196
7.193
7.189
7.186
7.182
7.177
7.174
7.161
7.158
7.150
7.140
7.130
7.126
7.120
7.113
6.918
6.905
6.511
6.485
5.114
5.088
4.979
4.954
2.637
2.612
2.604
2.578
2.353
0.754
0.744
0.642
0.637
0.628
0.620
0.614
0.490
0.489
0.480
0.336
0.329
0.326
0.133
0.126
0.123
CF3
O
NH
Ph
o-tolyl
O
N
N
N
O
Bn
4ap
1H NMR (600 MHz CDCl3)
1.00
1.98
1.02
3.18
2.19
1.32
2.59
1.17
2.10
3.29
2.23
1.01
1.01
1.04
1.00
1.05
1.00
2.99
1.01
1.01
1.00
1.01
1.01
f1 (ppm)

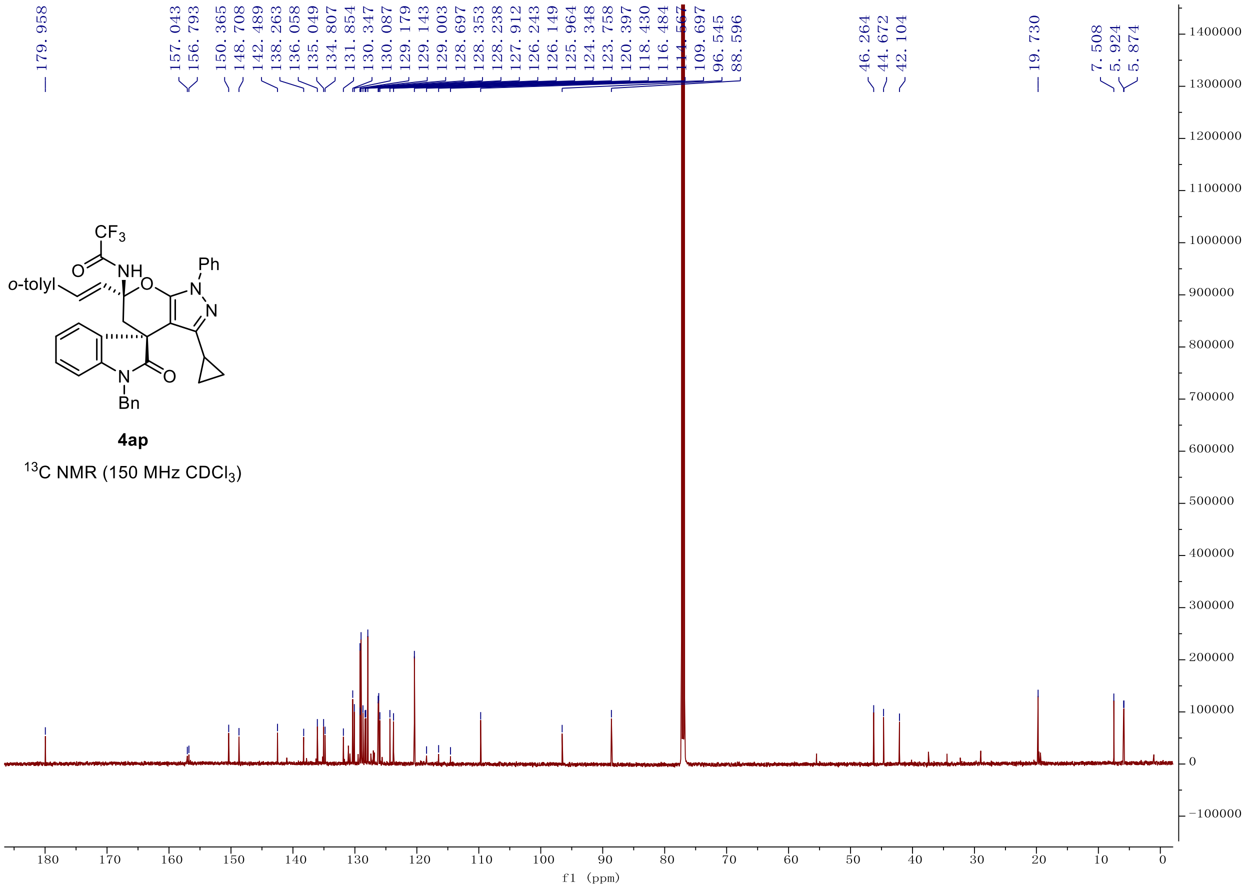
179.958
157.043
156.793
150.365
148.708
142.489
138.263
136.058
135.049
134.807
131.854
130.347
130.087
129.179
129.143
129.003
128.697
128.353
128.238
127.912
126.243
126.149
125.964
124.348
123.758
120.397
118.430
116.484
114.567
109.697
96.545
88.596
46.264
44.672
42.104
19.730
7.508
5.924
5.874
CF3
O
NH
Ph
o-tolyl
O
N
N
N
O
Bn
4ap
13C NMR (150 MHz CDCl3)
f1 (ppm)

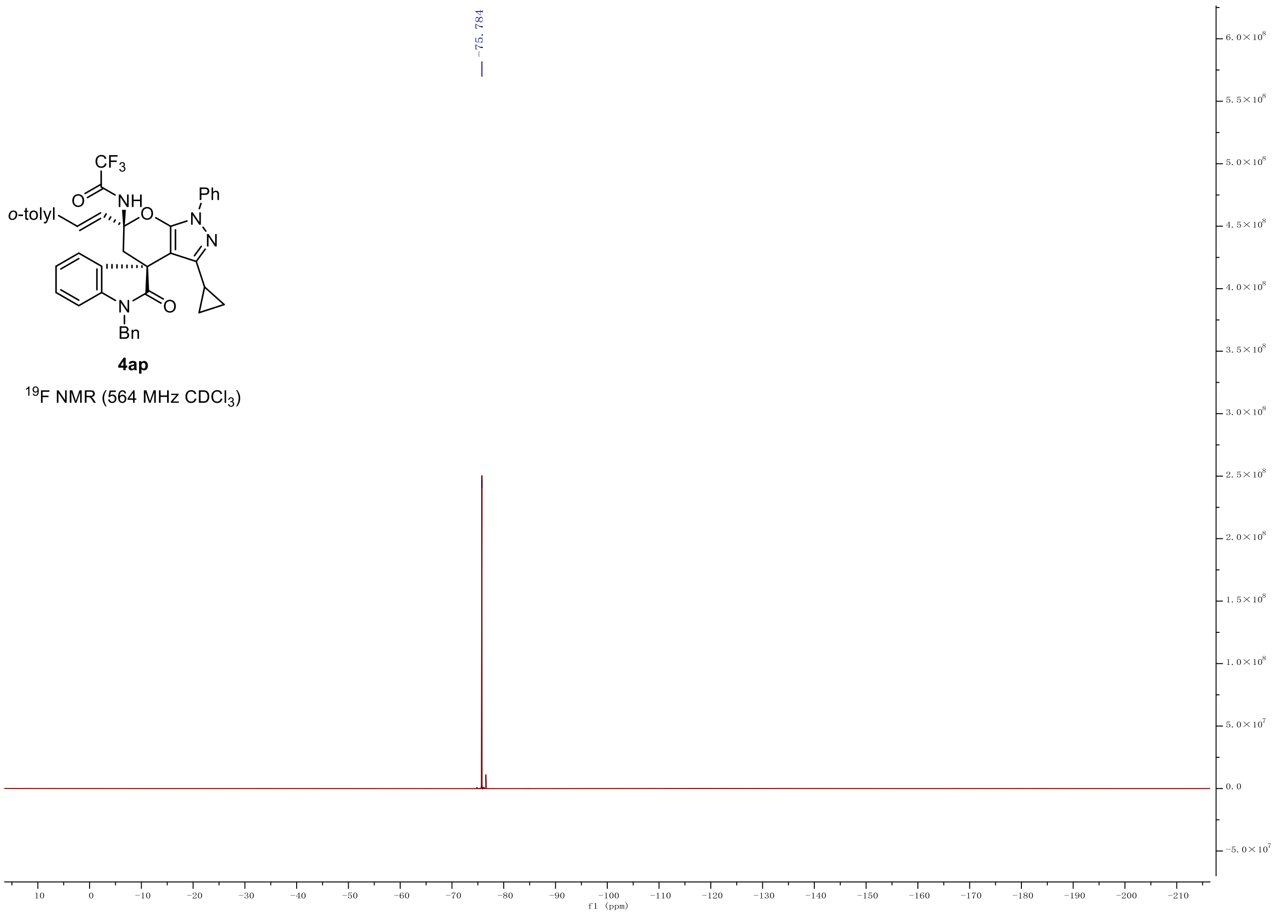

Peak Analysis Report

Detector A Channel 1 254nm

No.	Ret. Time	Height (mAu)	Area (mAu*min)	Rel. Area (%)
1	6.911	204774	3142878	50.317
2	9.164	152146	3103236	49.683
Total		356920	6246114	100.000

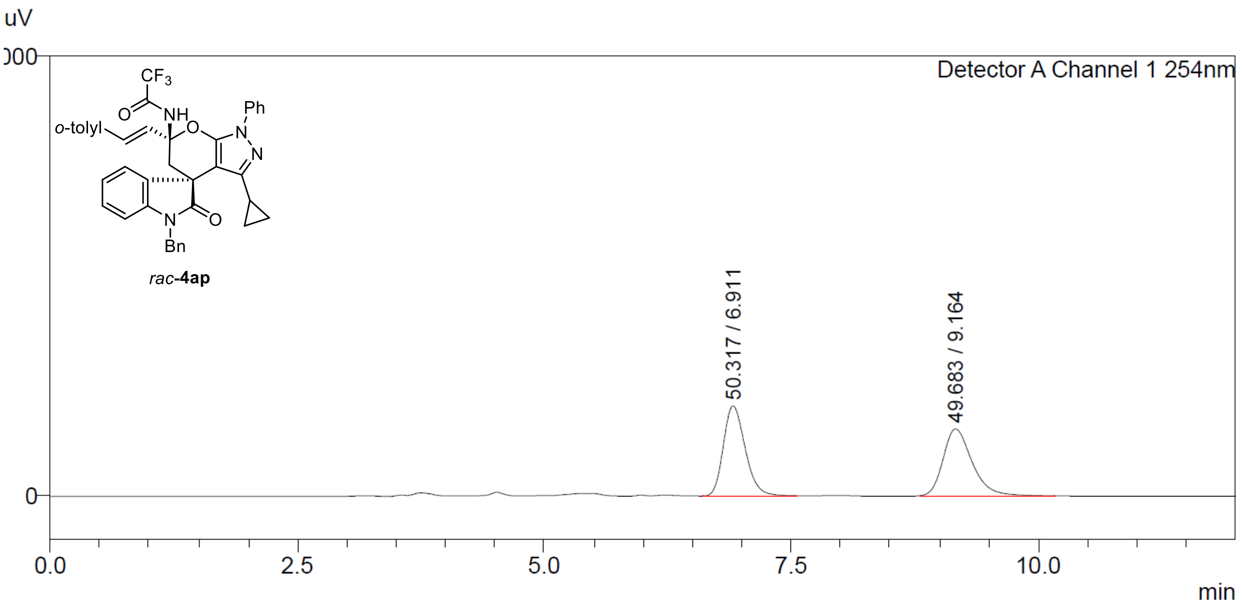

Peak Analysis Report

Detector A Channel 1 254nm

No.	Ret. Time	Height (mAu)	Area (mAu*min)	Rel. Area (%)
1	6.656	285604	3412383	10.821
2	8.677	1517947	28121294	89.179
Total		1803551	31533677	100.000

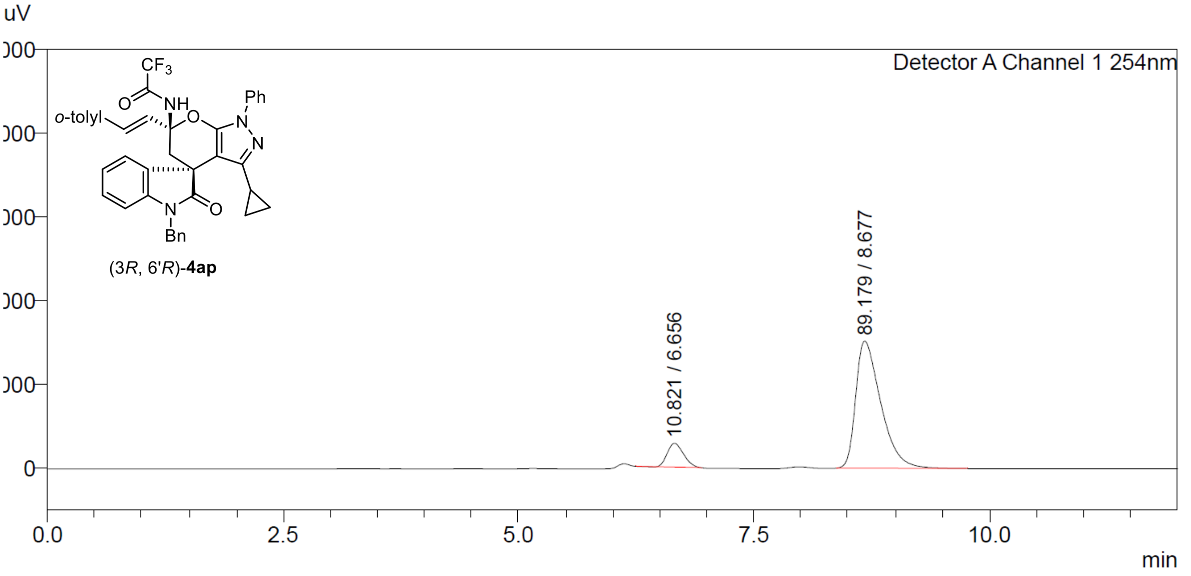

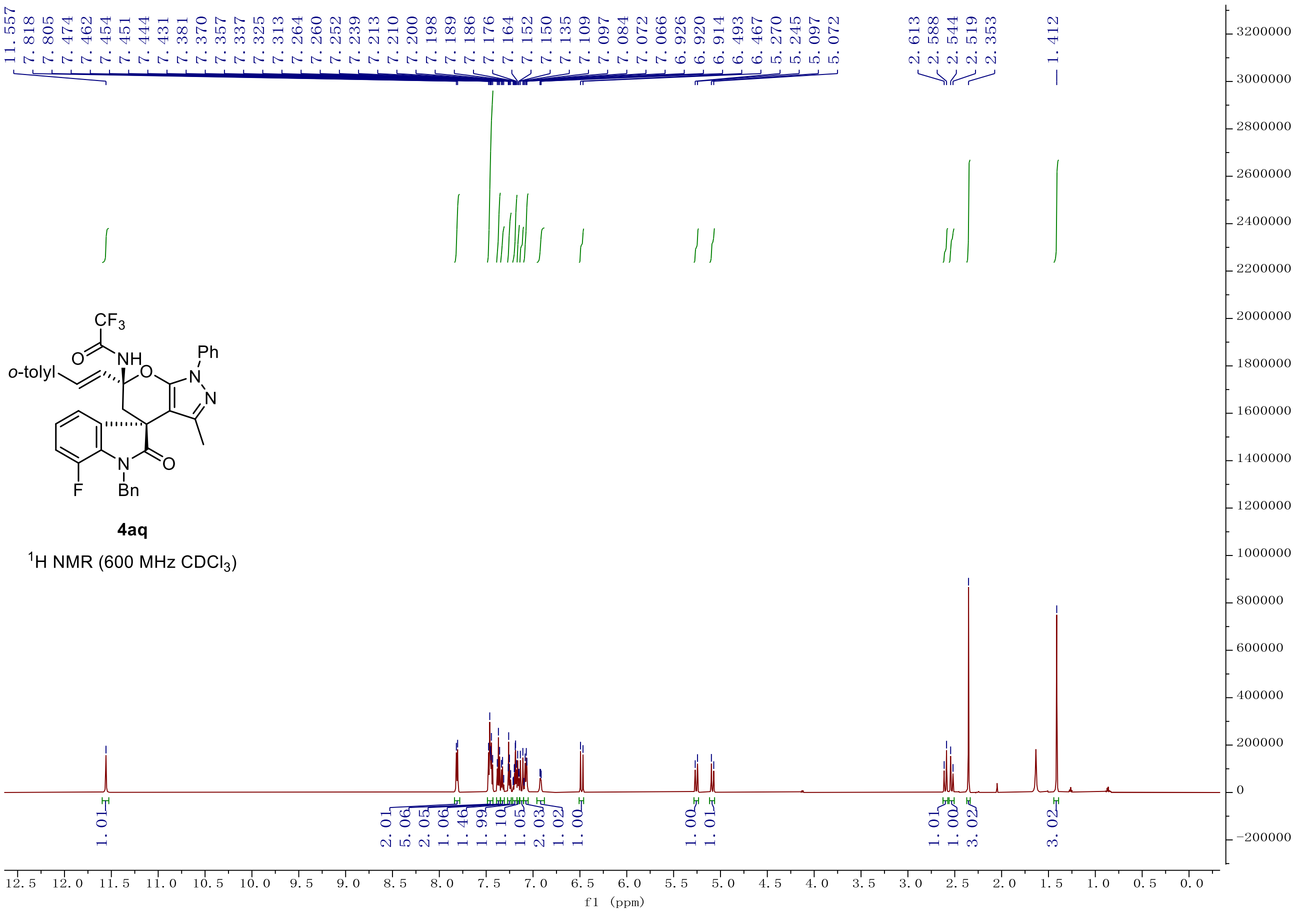

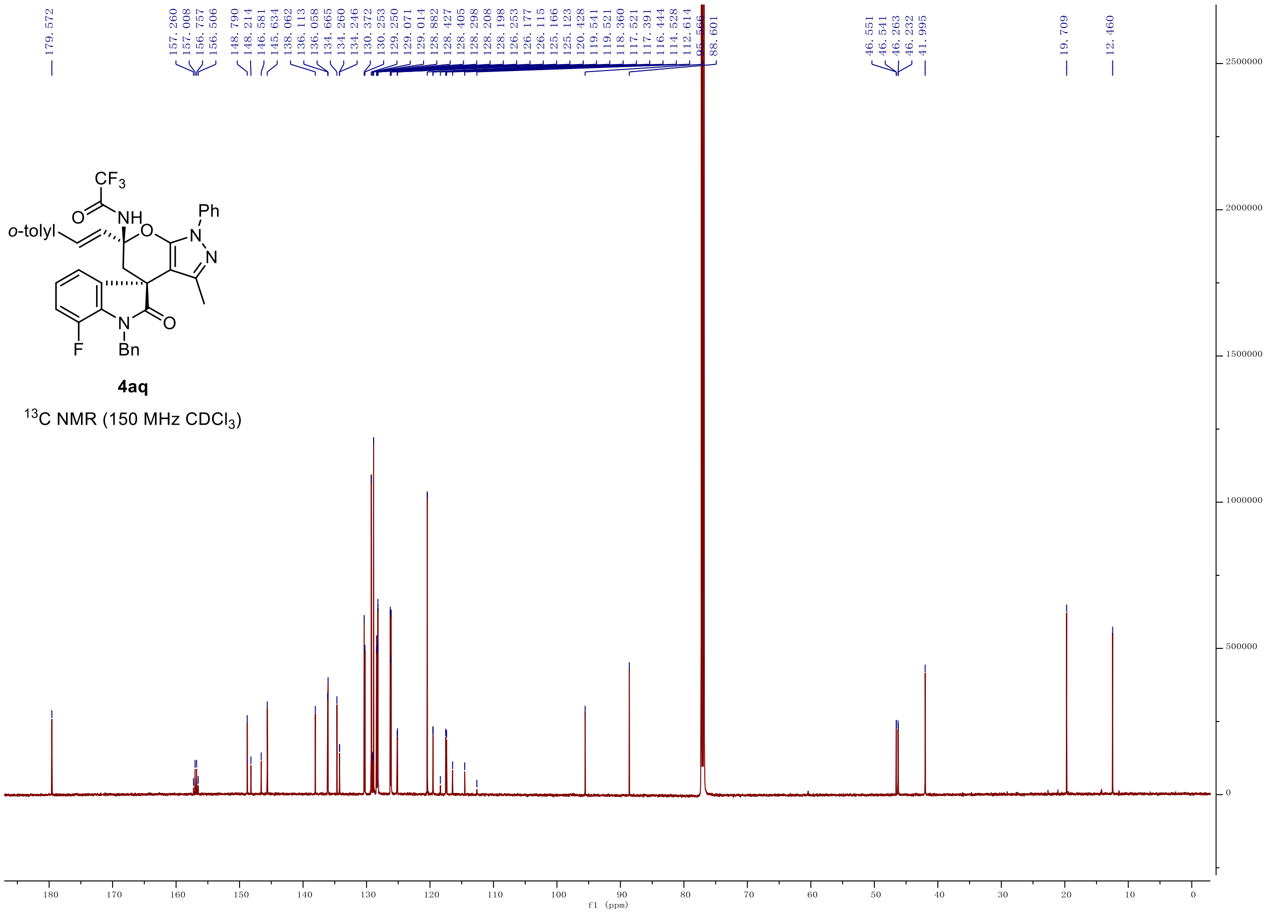
179.572
157.260
157.008
156.757
156.506
148.790
148.214
146.581
145.634
138.062
136.113
136.058
134.665
134.260
134.246
130.372
130.253
129.250
129.071
129.014
128.882
128.427
128.405
128.298
128.208
128.198
126.253
126.177
126.115
125.166
125.123
120.428
119.541
119.521
118.360
117.521
117.391
116.444
114.528
112.614
95.566
88.601
46.551
46.541
46.263
46.232
41.995
19.709
12.460
CF3
NH
Ph
o-tolyl
O
N
F
Bn
4aq
13C NMR (150 MHz CDCl3)
f1 (ppm)

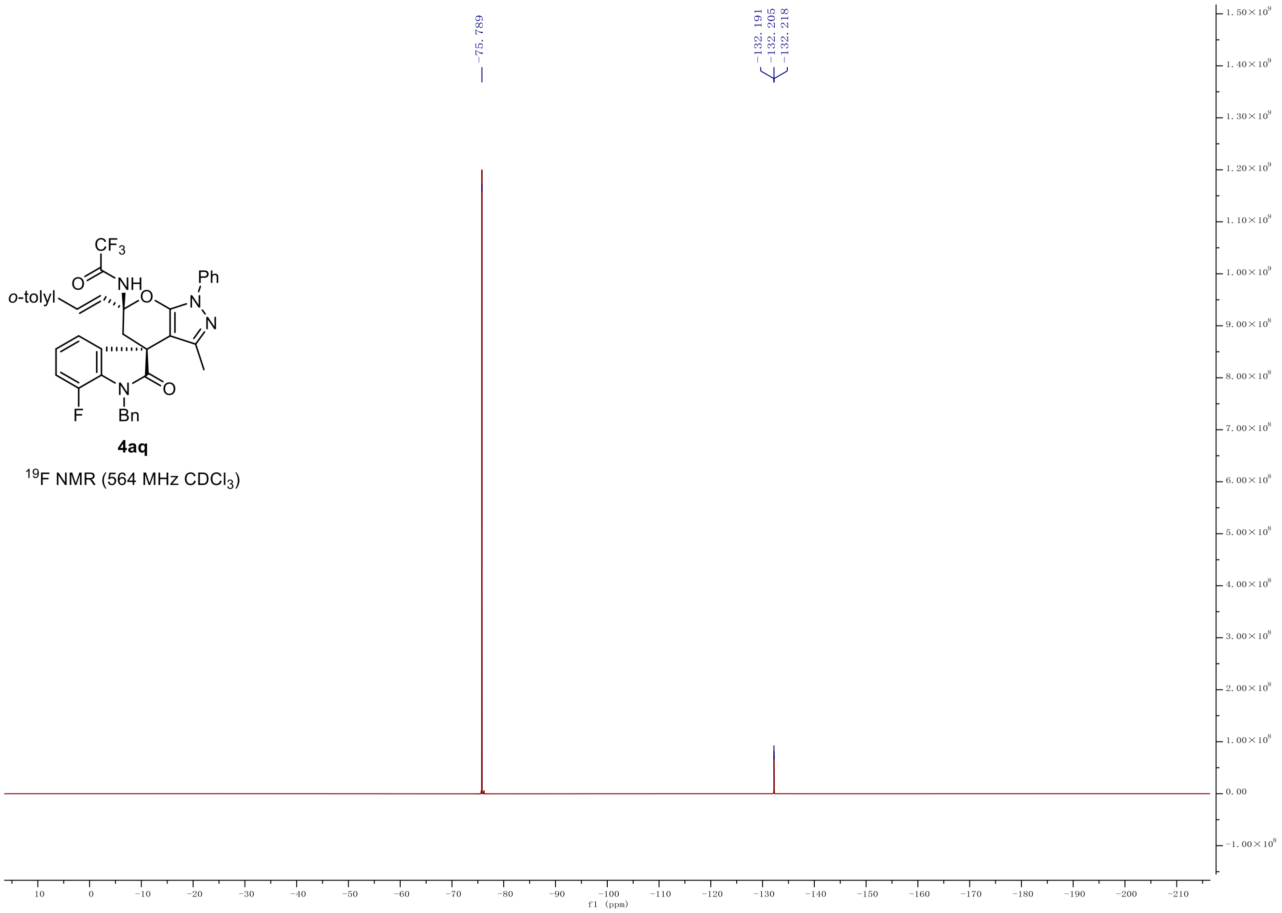
-75.789
-132.191
-132.205
-132.218
CF3
NH
Ph
o-tolyl
O
N
F
Bn
4aq
19F NMR (564 MHz CDCl3)
f1 (ppm)

Peak Analysis Report

Detector A Channel 1 254nm

No.	Ret. Time	Height (mAu)	Area (mAu*min)	Rel. Area (%)
1	5.720	2237059	26571539	49.808
2	6.464	1770690	26776279	50.192
Total		4007749	53347818	100.000

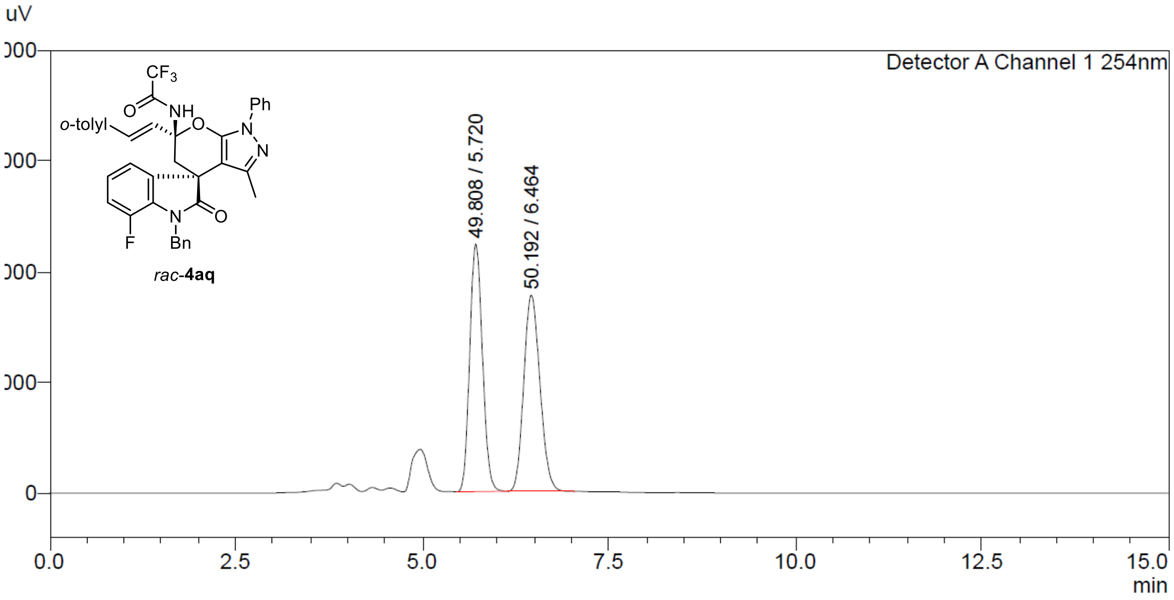

Peak Analysis Report

Detector A Channel 1 254nm

No.	Ret. Time	Height (mAu)	Area (mAu*min)	Rel. Area (%)
1	5.718	2255588	26816318	99.608
2	6.467	8953	105456	0.392
Total		2264541	26921774	100.000

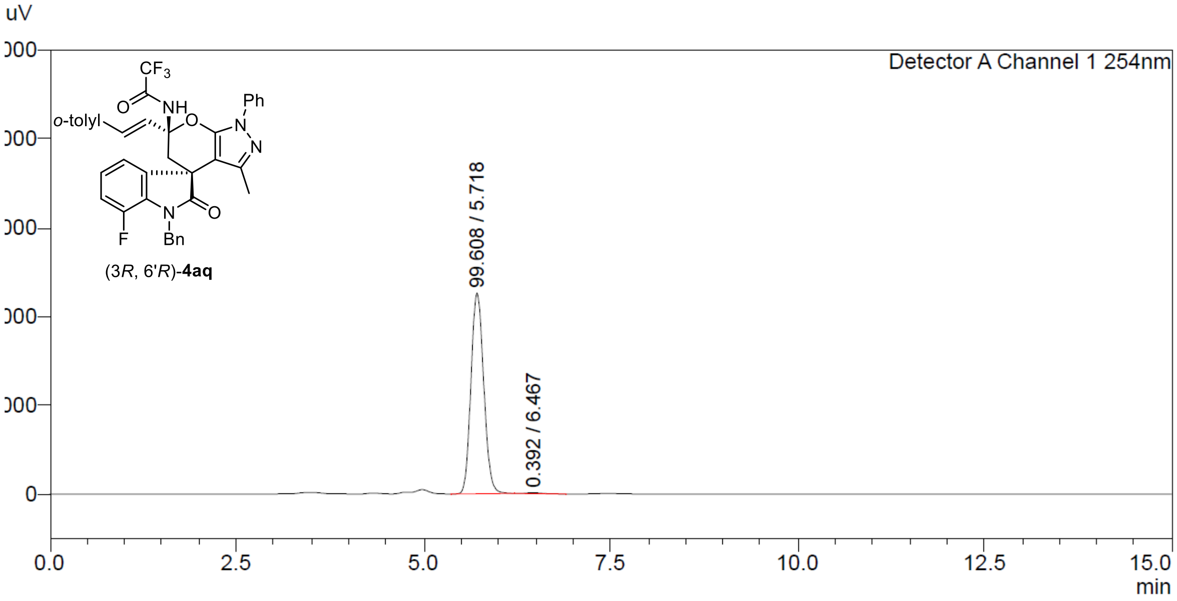

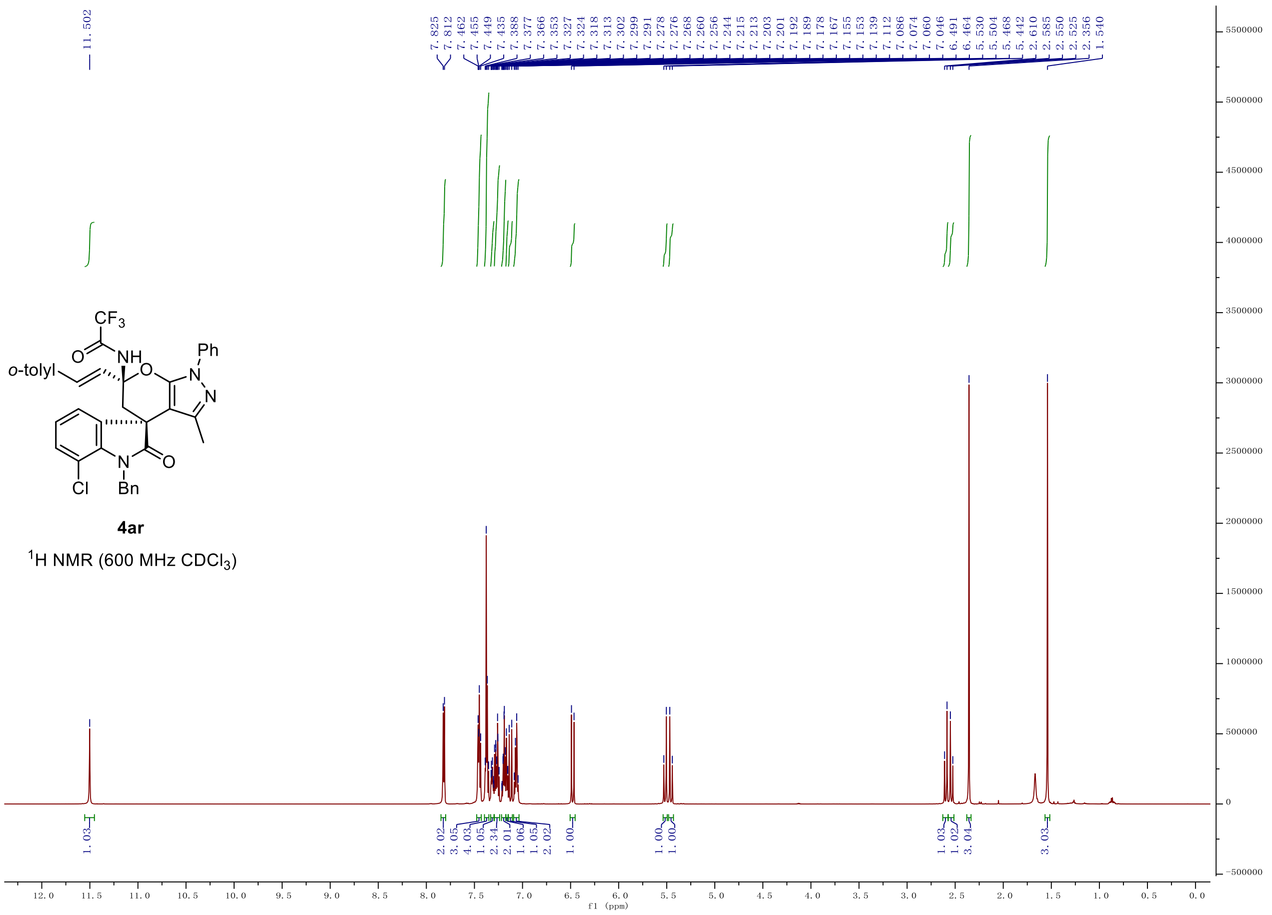
4ar
^{1}H NMR (600 MHz CDCl$_3$)

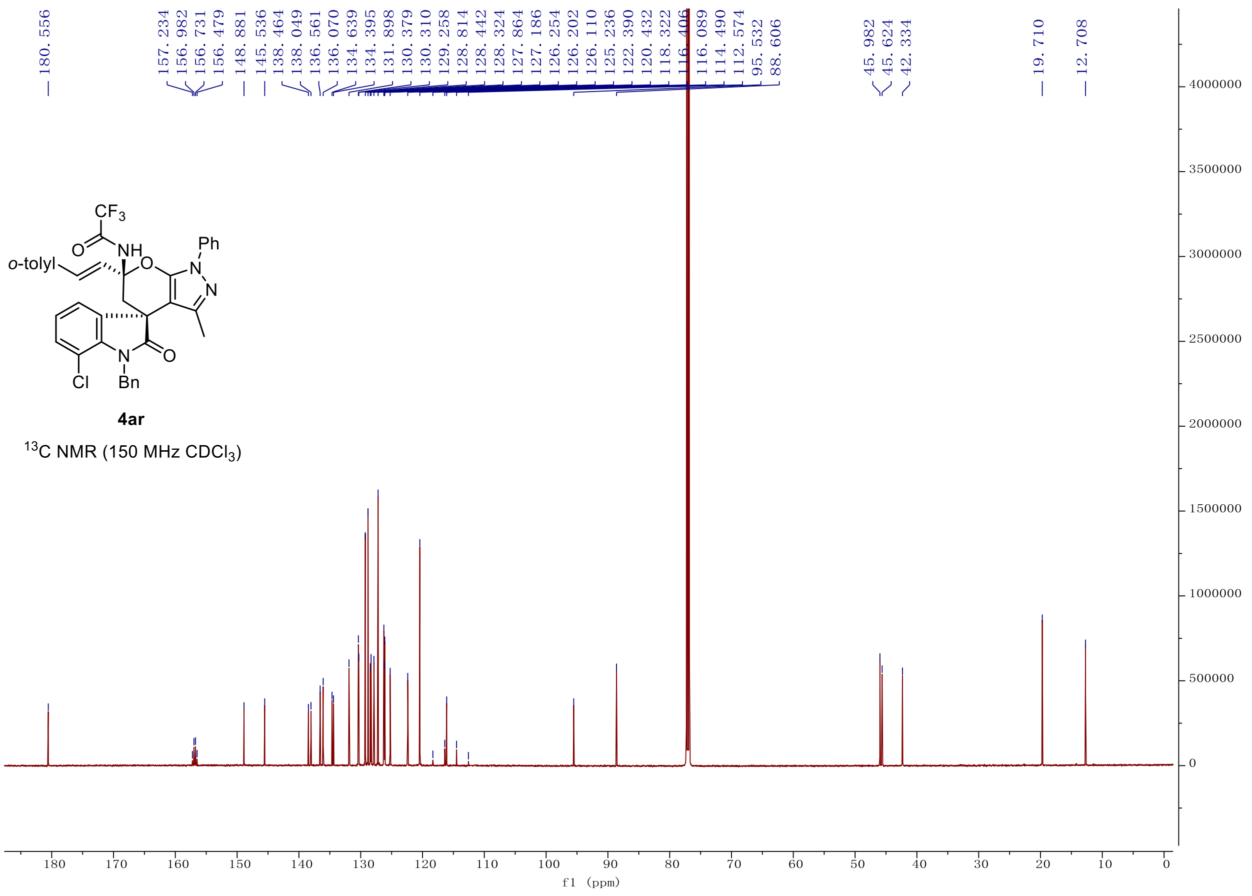
4ar
^{13}C NMR (150 MHz CDCl$_3$)

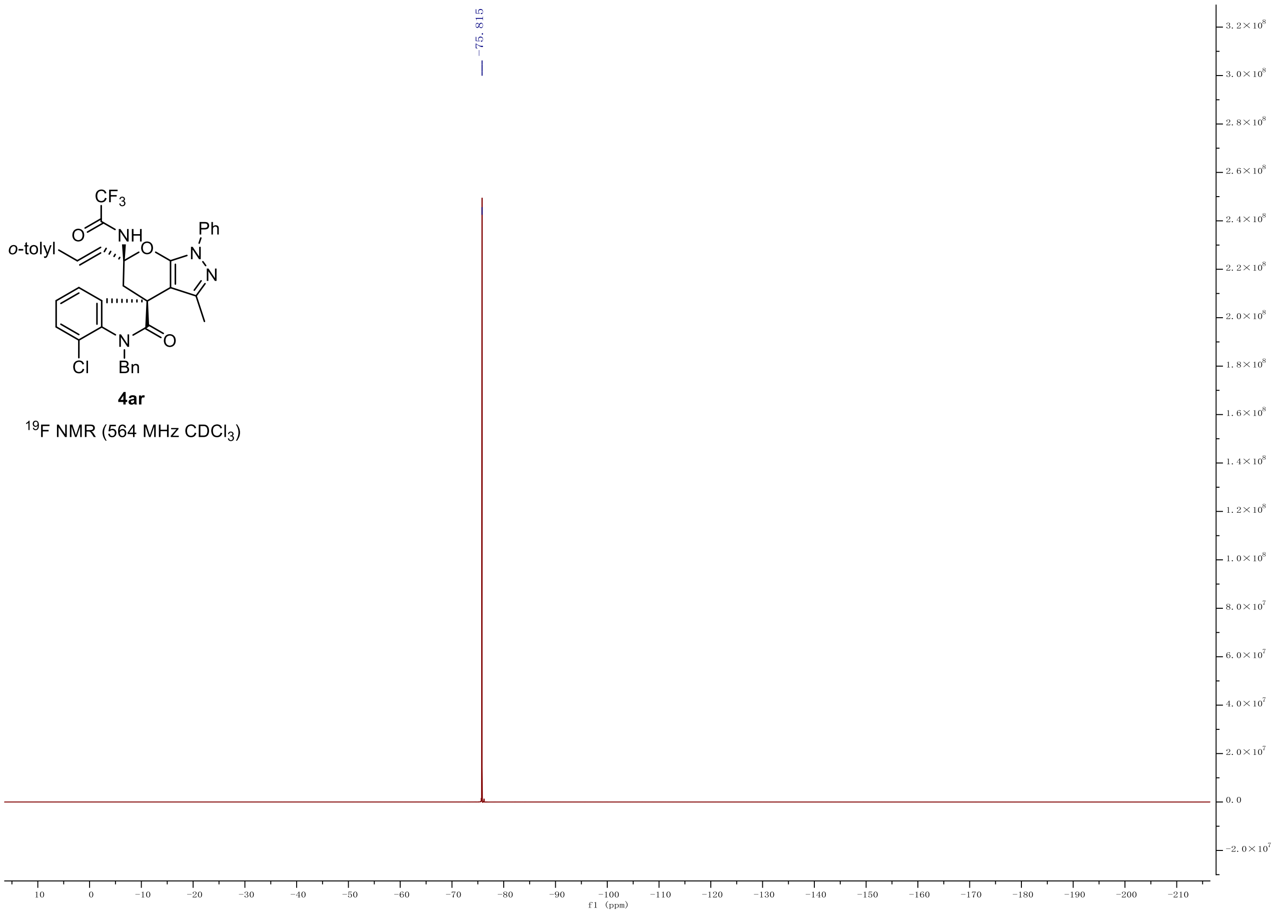

Peak Analysis Report

Detector A Channel 1 254nm

No.	Ret. Time	Height (mAu)	Area (mAu*min)	Rel. Area (%)
1	5.882	2929963	39880659	49.505
2	7.223	2007491	40678386	50.495
Total		4937455	80559046	100.000

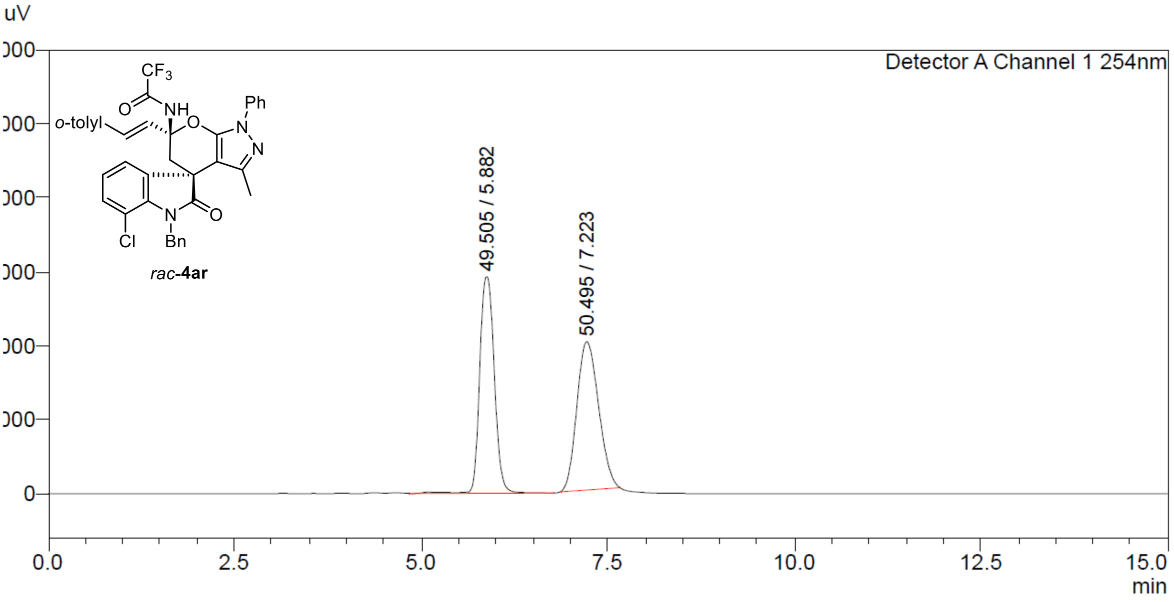

Peak Analysis Report

Detector A Channel 1 254nm

No.	Ret. Time	Height (mAu)	Area (mAu*min)	Rel. Area (%)
1	5.869	299467	3460201	99.780
2	7.221	604	7637	0.220
Total		300072	3467837	100.000

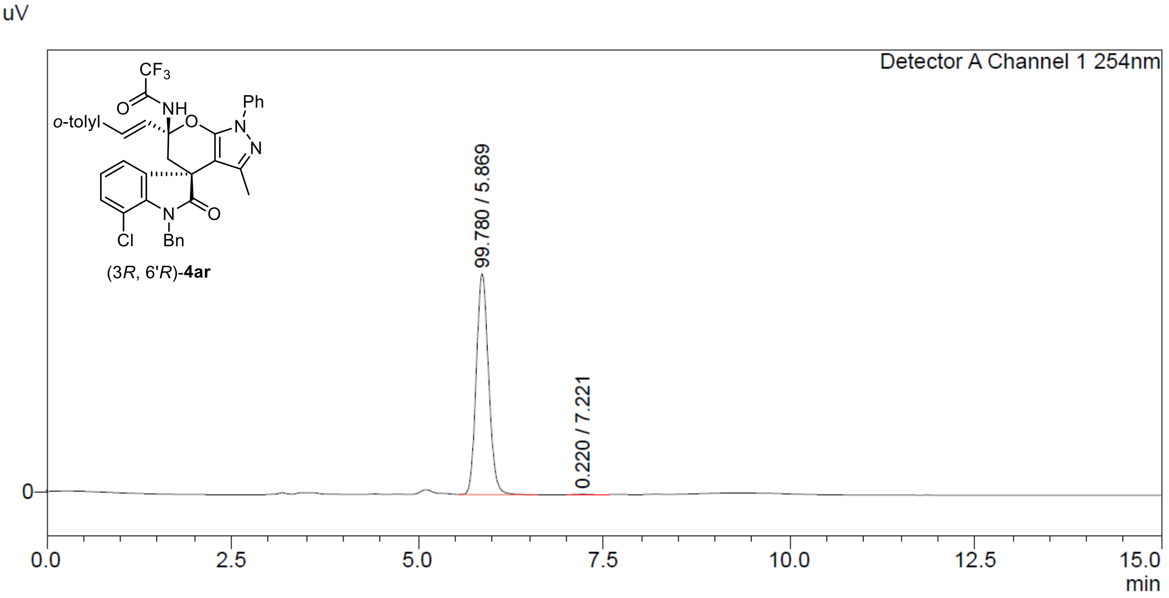

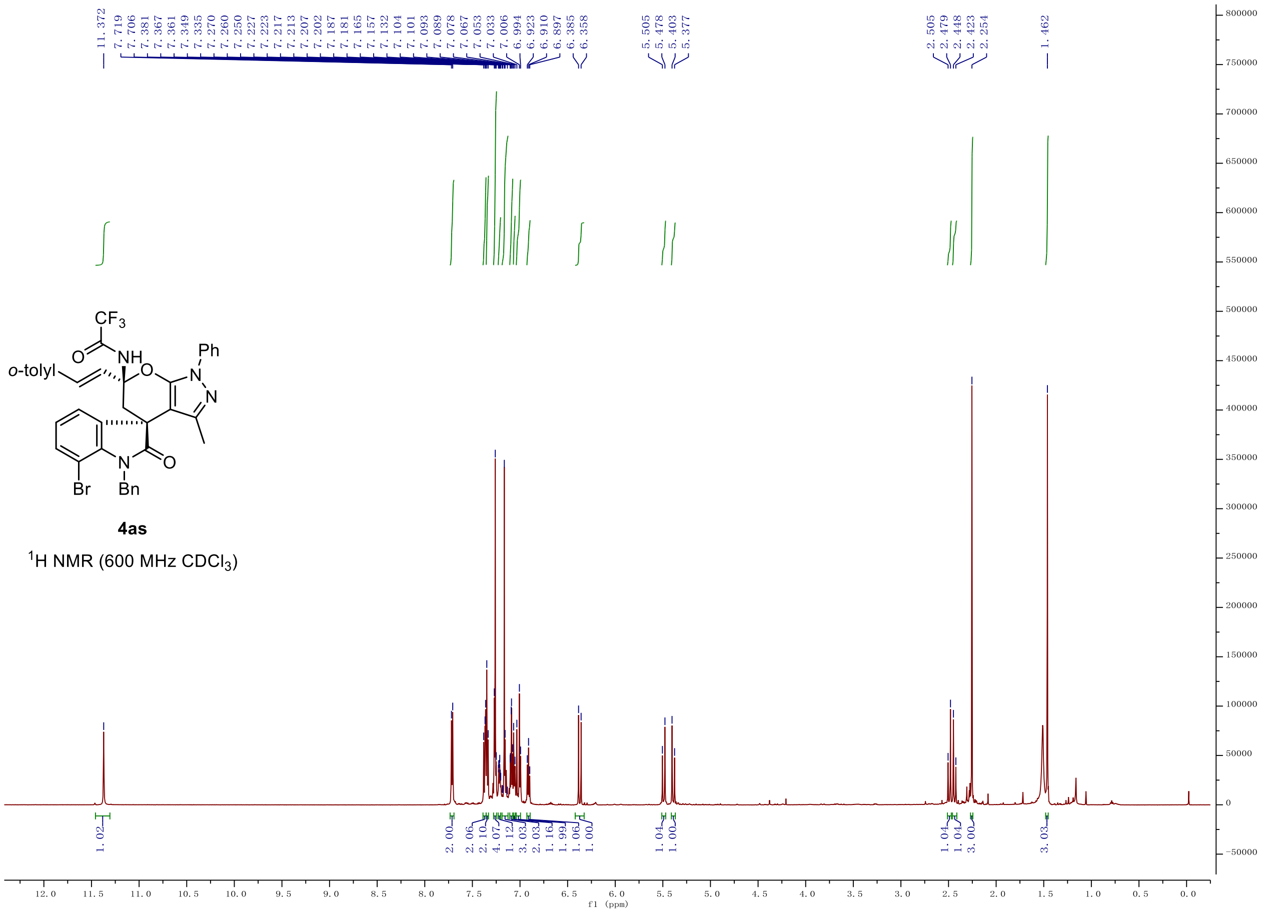

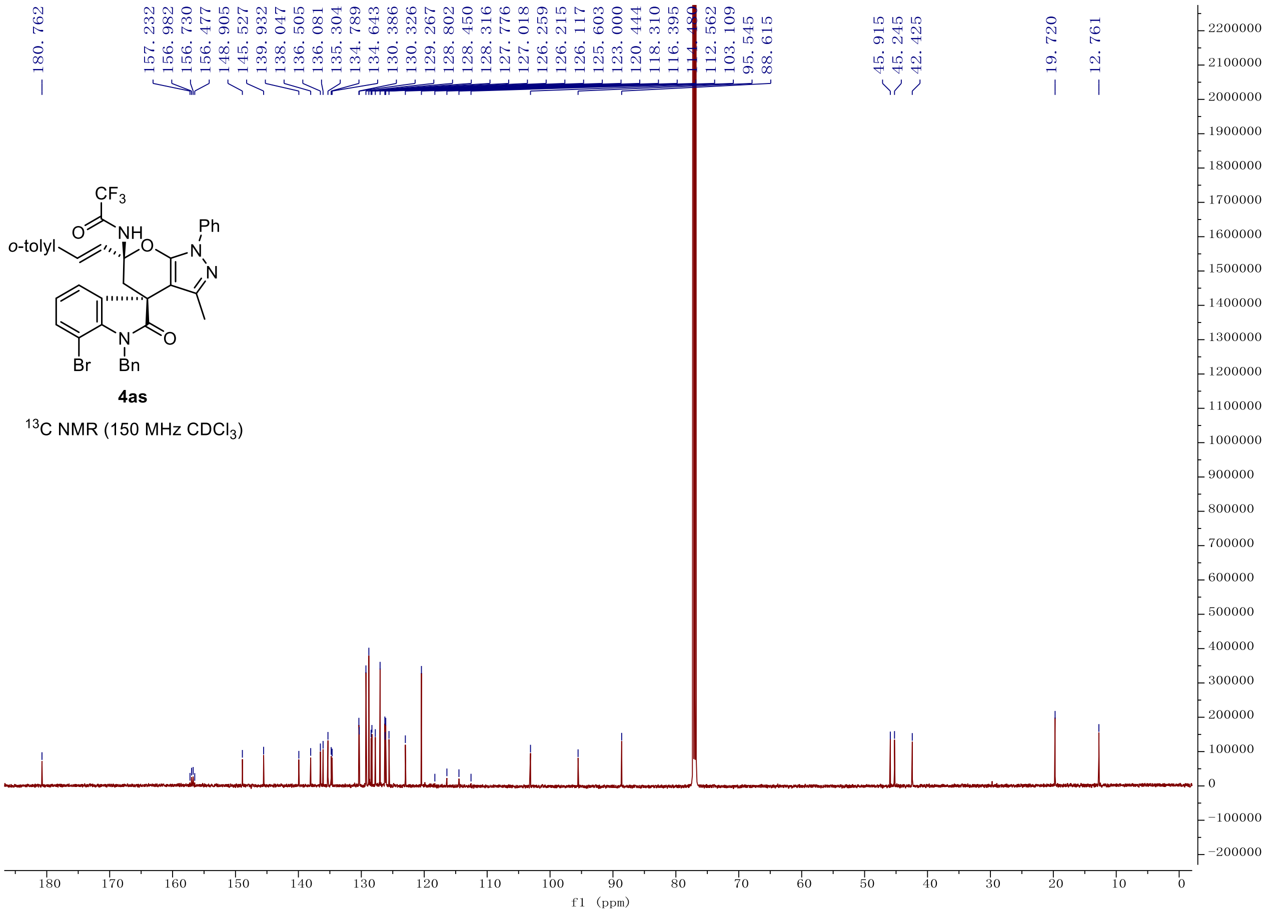

180.762
157.232
156.982
156.730
156.477
148.905
145.527
139.932
138.047
136.505
136.081
135.304
134.789
134.643
130.386
130.326
129.267
128.802
128.450
128.316
127.776
127.018
126.259
126.215
126.117
125.603
123.000
120.444
118.310
116.395
114.480
112.562
103.109
95.545
88.615
45.915
45.245
42.425
19.720
12.761
CF3
NH
O
o-tolyl
Ph
N
Br
Bn
4as
13C NMR (150 MHz CDCl3)
f1 (ppm)

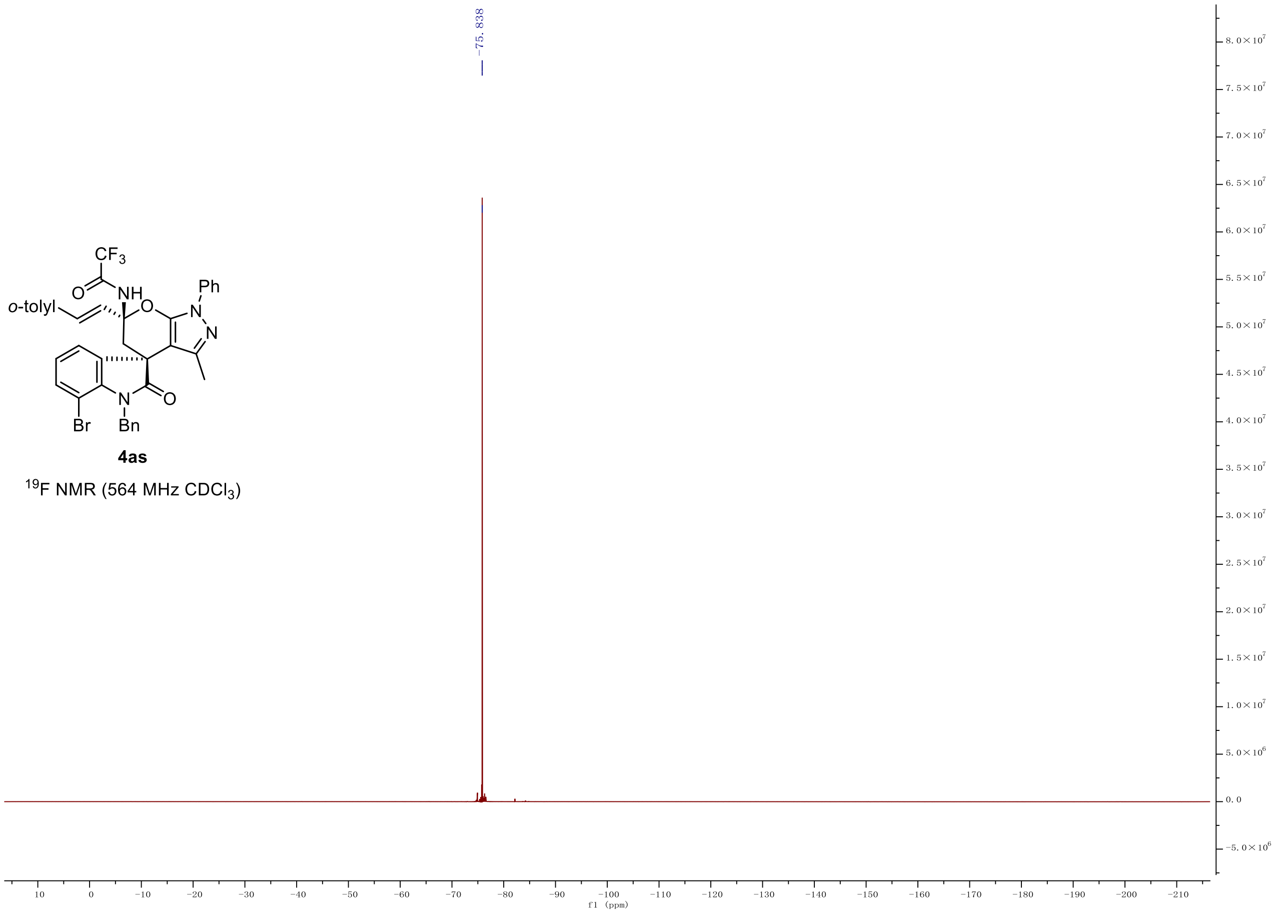

-75.838
CF3
NH
O
o-tolyl
Ph
N
Br
Bn
4as
19F NMR (564 MHz CDCl3)
f1 (ppm)

Peak Analysis Report

Detector A Channel 1 254nm

No.	Ret. Time	Height (mAu)	Area (mAu*min)	Rel. Area (%)
1	6.010	1416784	15993520	49.305
2	7.618	756964	16444706	50.695
Total		2173748	32438226	100.000

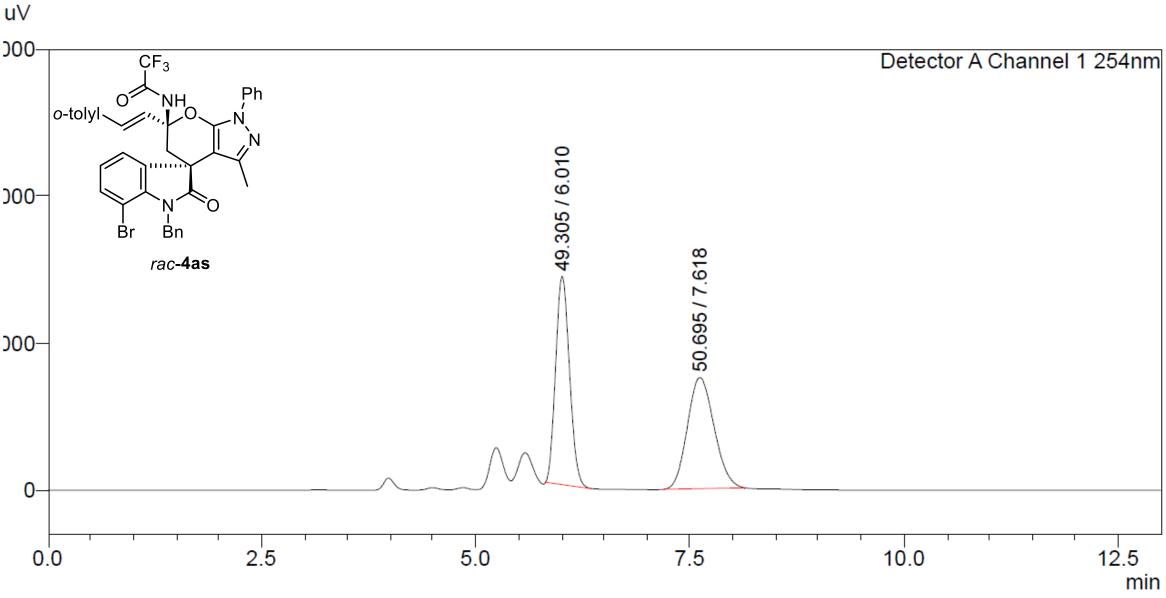

Peak Analysis Report

Detector A Channel 1 254nm

No.	Ret. Time	Height (mAu)	Area (mAu*min)	Rel. Area (%)
1	5.994	2324294	28140098	99.641
2	7.603	6778	101459	0.359
Total		2331072	28241557	100.000

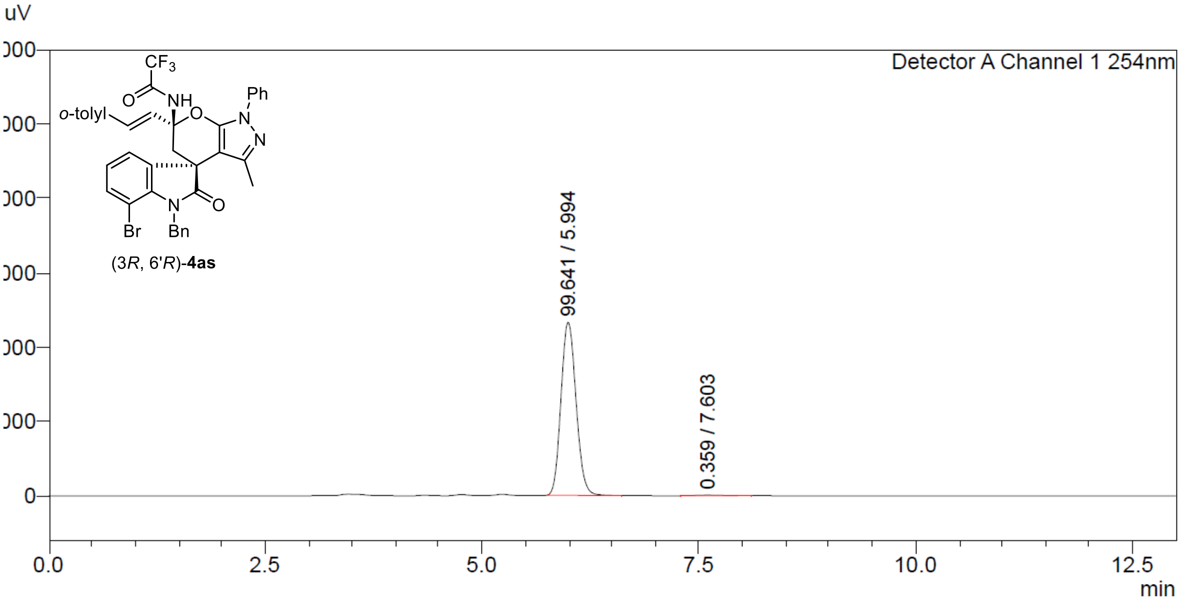

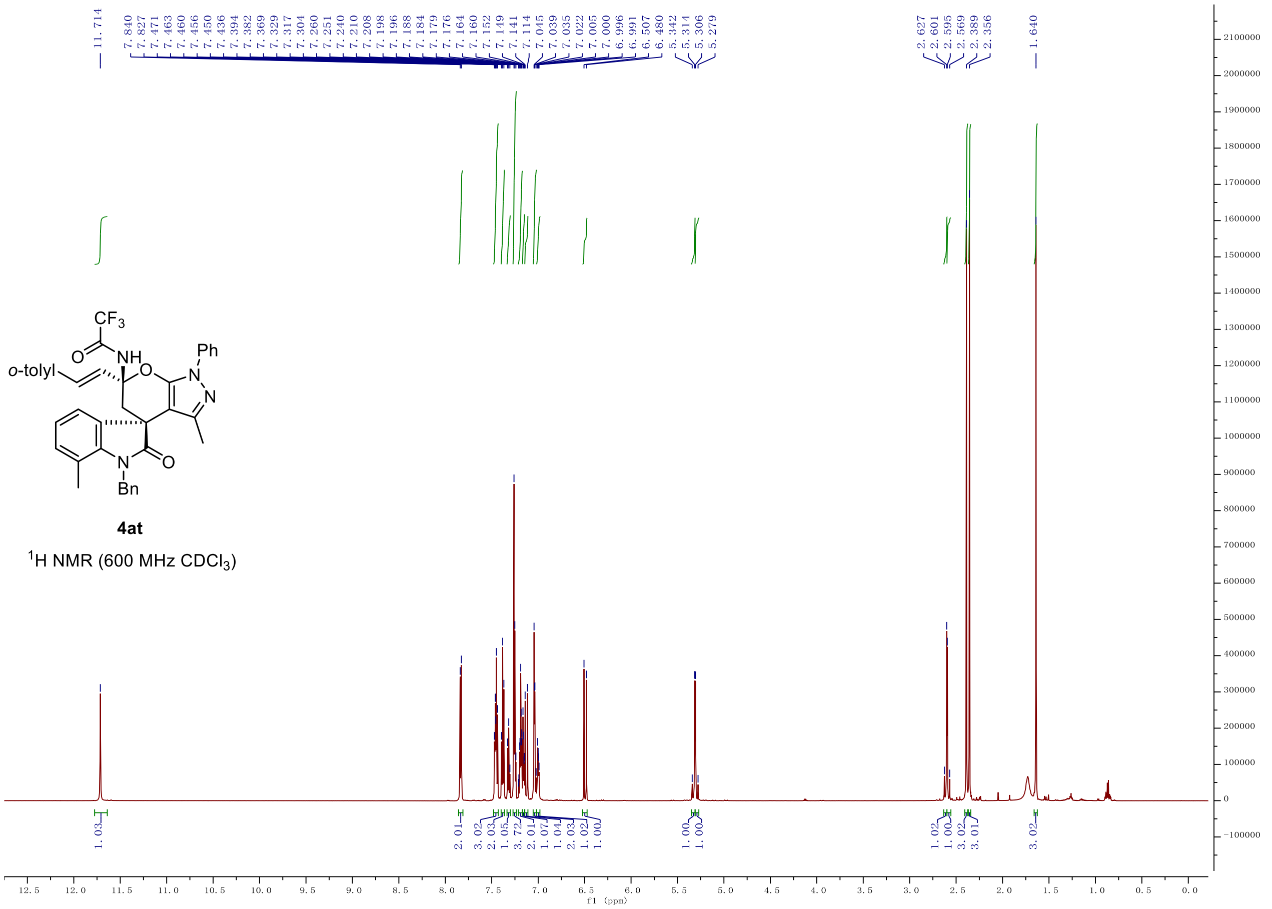

4at

^{1}H NMR (600 MHz CDCl$_3$)

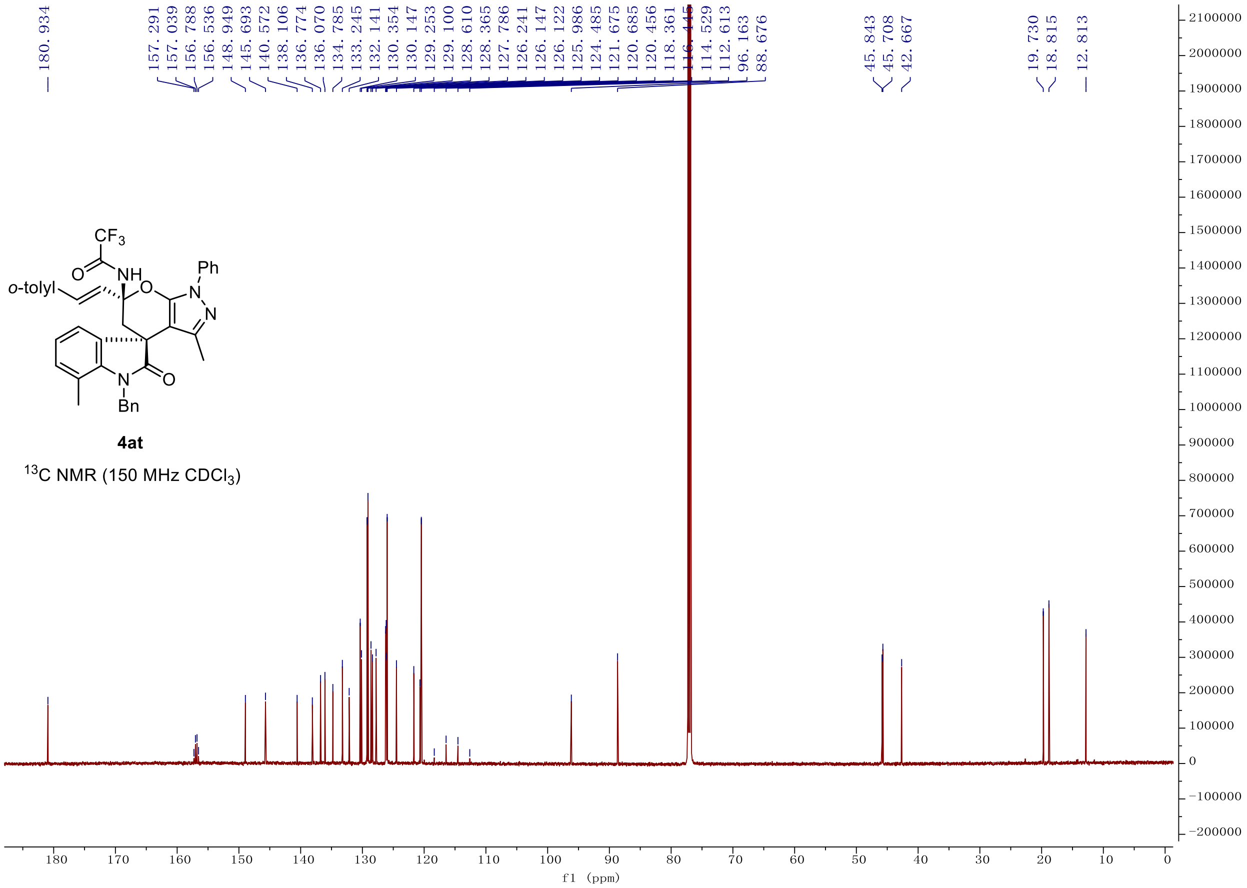

4at

^{13}C NMR (150 MHz CDCl$_3$)

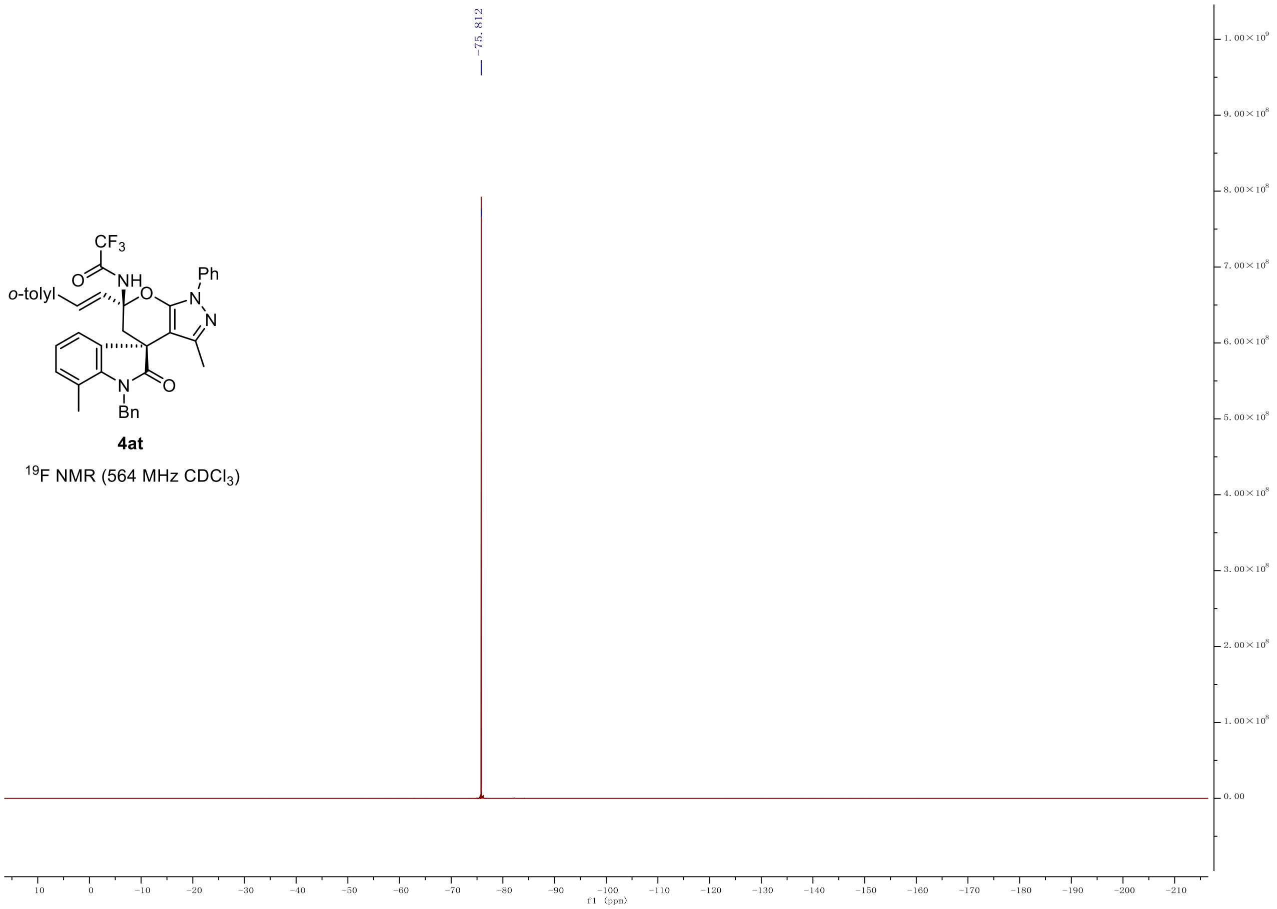

Peak Analysis Report

Detector A Channel 1 254nm

No.	Ret. Time	Height (mAu)	Area (mAu*min)	Rel. Area (%)
1	5.902	2390831	29253824	50.266
2	7.638	1180951	28944165	49.734
Total		3571781	58197989	100.000

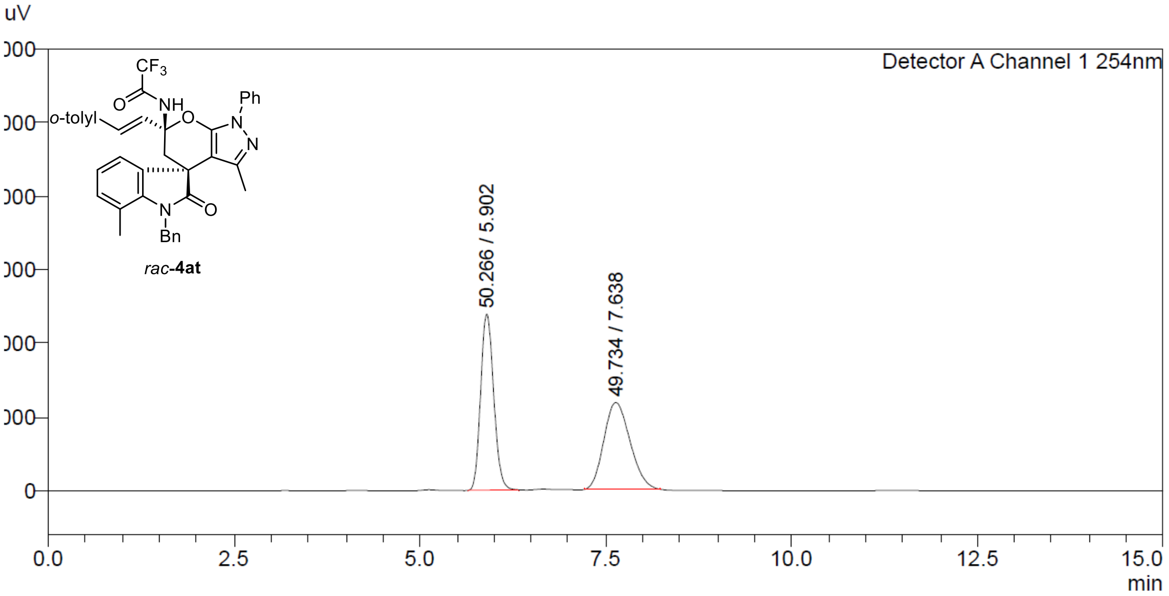

Peak Analysis Report

Detector A Channel 1 254nm

No.	Ret. Time	Height (mAu)	Area (mAu*min)	Rel. Area (%)
1	5.898	2609025	32914756	99.447
2	7.661	8160	183073	0.553
Total		2617185	33097829	100.000

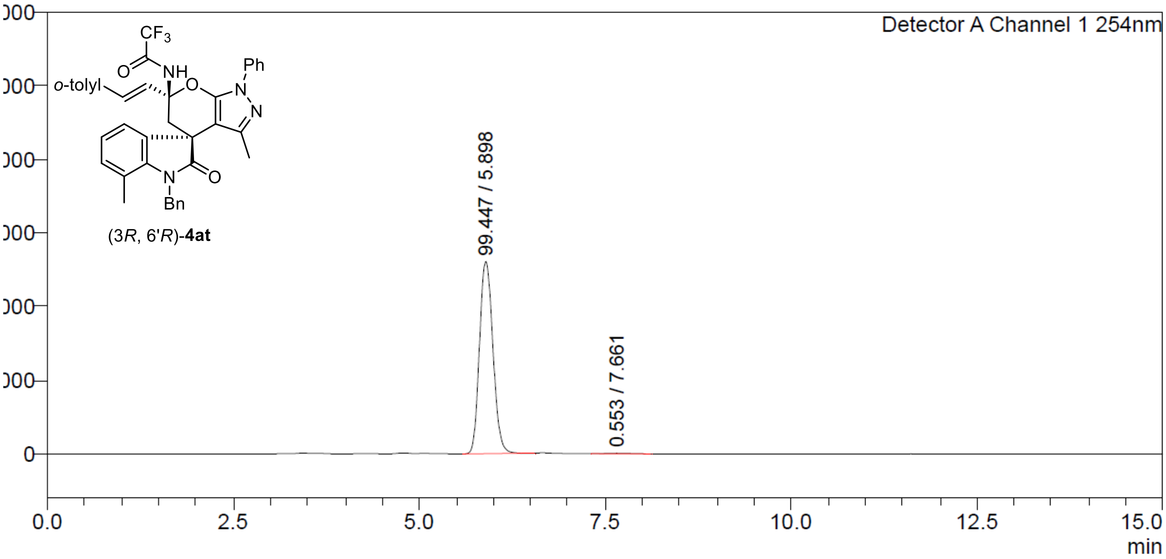

(3*R*, 6'*R*)-**4at**

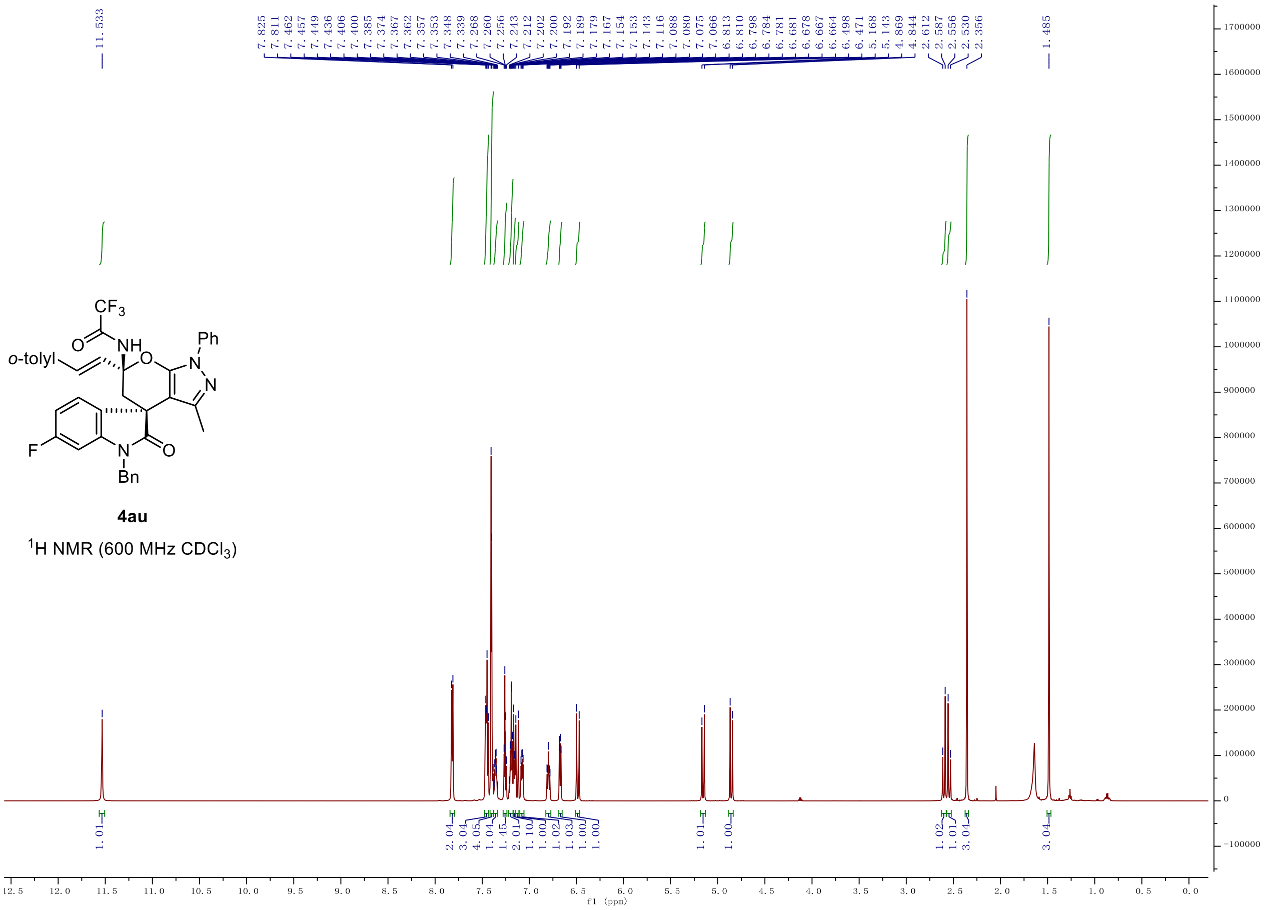

4au

^{1}H NMR (600 MHz CDCl$_3$)

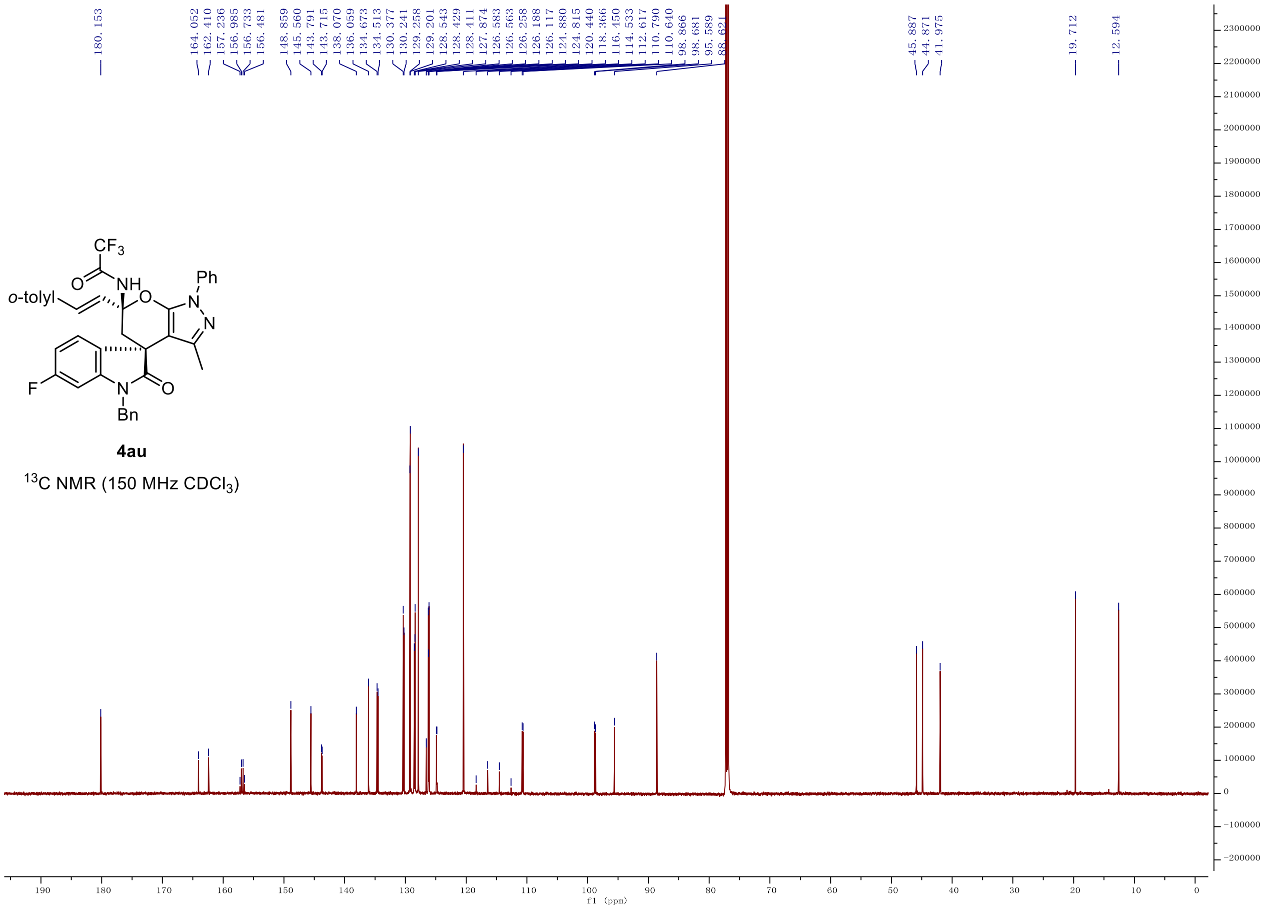
180.153
164.052
162.410
157.236
156.985
156.733
156.481
148.859
145.560
143.791
143.715
138.070
136.059
134.673
134.513
130.377
130.241
129.258
129.201
128.543
128.429
128.411
127.874
126.583
126.563
126.258
126.188
126.117
124.880
124.815
120.440
118.366
116.450
114.533
112.617
110.790
110.640
98.866
98.681
95.589
88.621
45.887
44.871
41.975
19.712
12.594
CF3
O
NH
Ph
o-tolyl
O
N
N
F
N
O
Bn
4au
13C NMR (150 MHz CDCl3)
f1 (ppm)

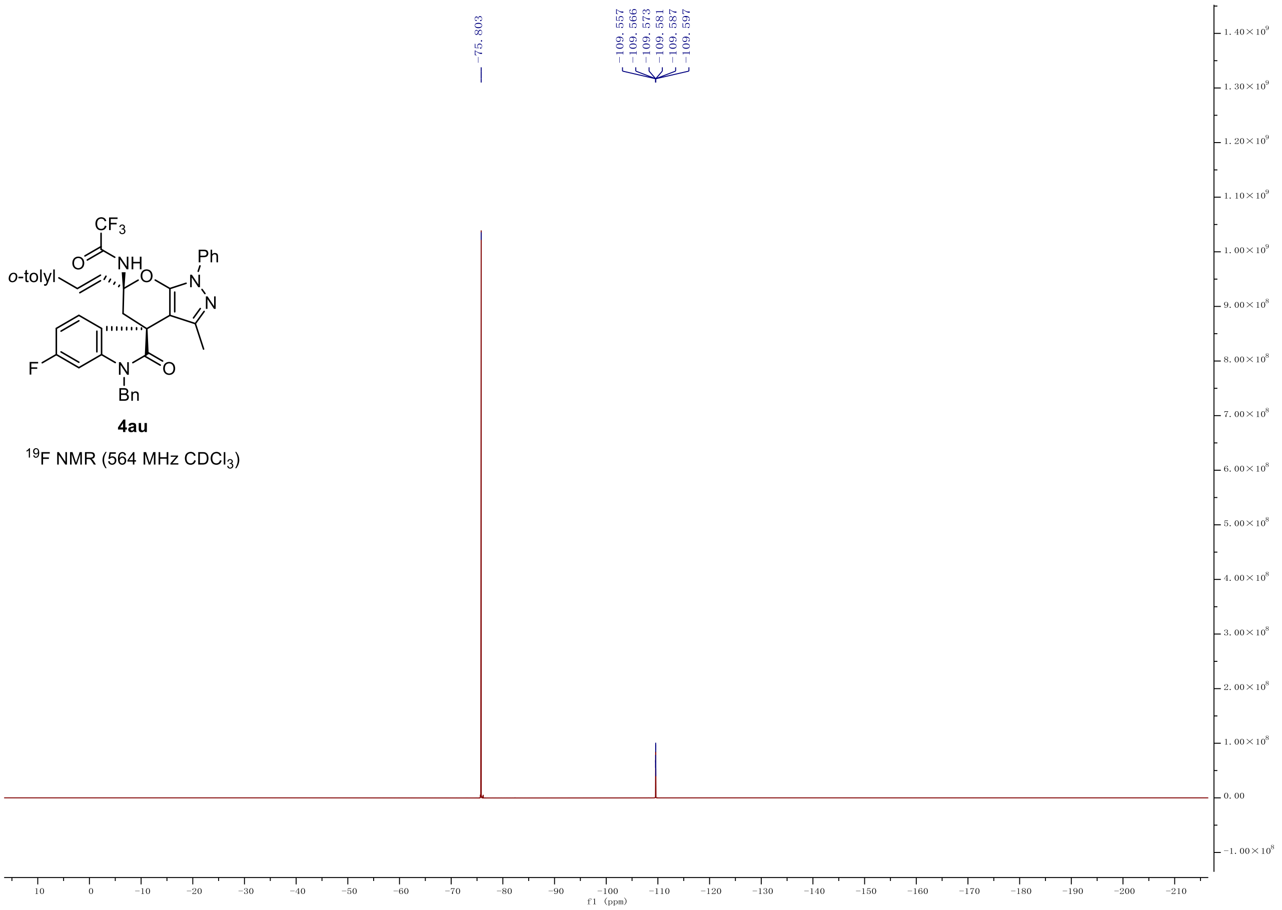
-75.803
-109.557
-109.566
-109.573
-109.581
-109.587
-109.597
CF3
O
NH
Ph
o-tolyl
O
N
N
F
N
O
Bn
4au
19F NMR (564 MHz CDCl3)
f1 (ppm)

Peak Analysis Report

Detector A Channel 1 254nm

No.	Ret. Time	Height (mAu)	Area (mAu*min)	Rel. Area (%)
1	8.827	945679	17434047	50.247
2	10.225	707307	17262872	49.753
Total		1652986	34696919	100.000

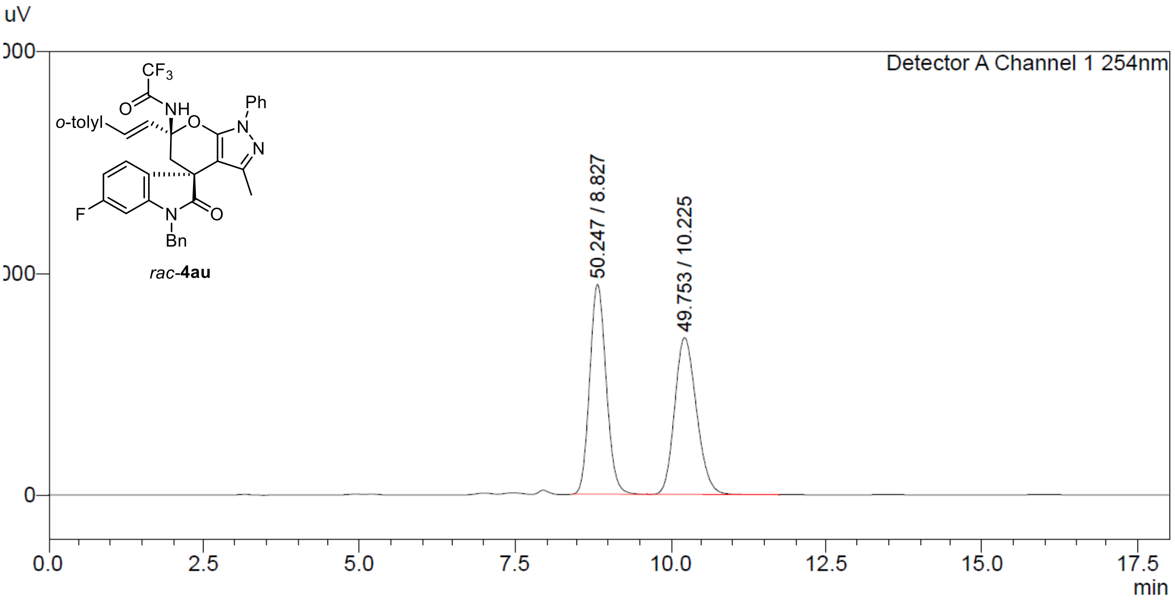

Peak Analysis Report

Detector A Channel 1 254nm

No.	Ret. Time	Height (mAu)	Area (mAu*min)	Rel. Area (%)
1	8.784	470059	8921775	99.796
2	10.202	791	18277	0.204
Total		470850	8940052	100.000

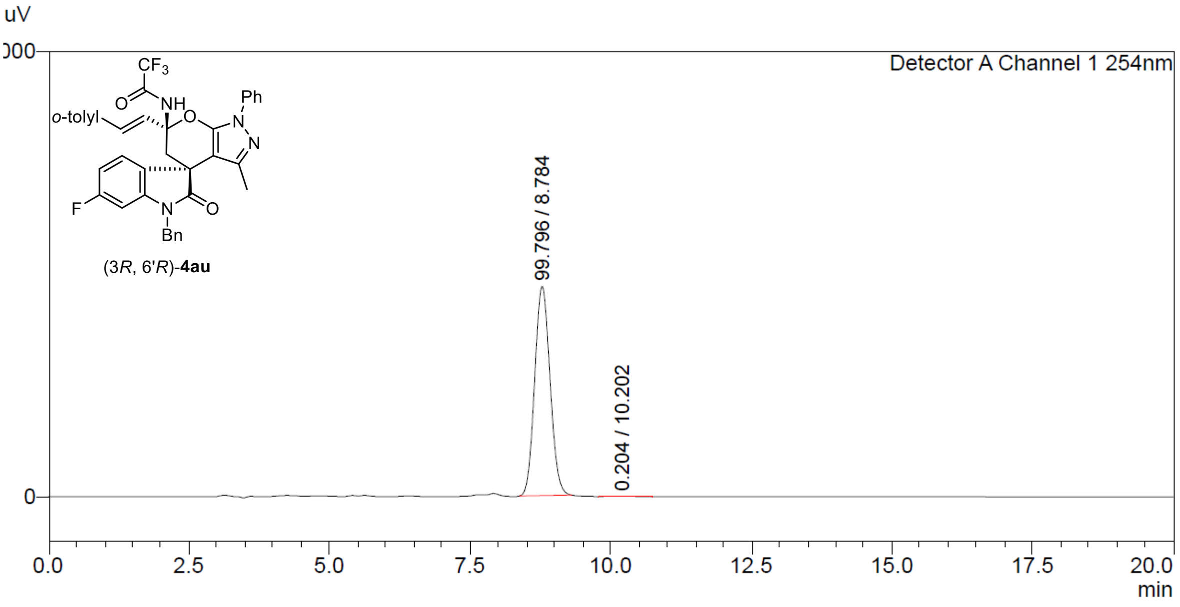

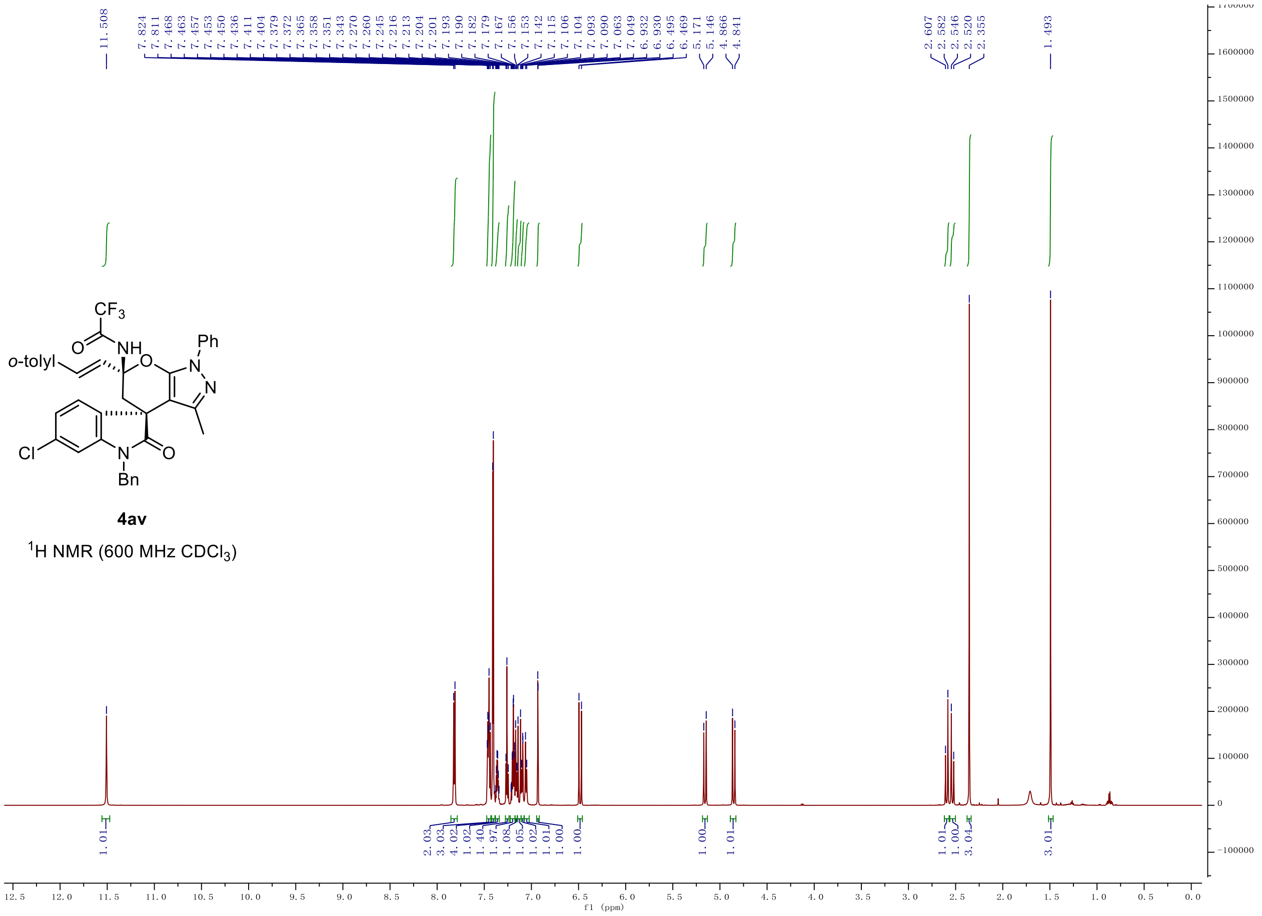

4av

^{1}H NMR (600 MHz CDCl$_3$)

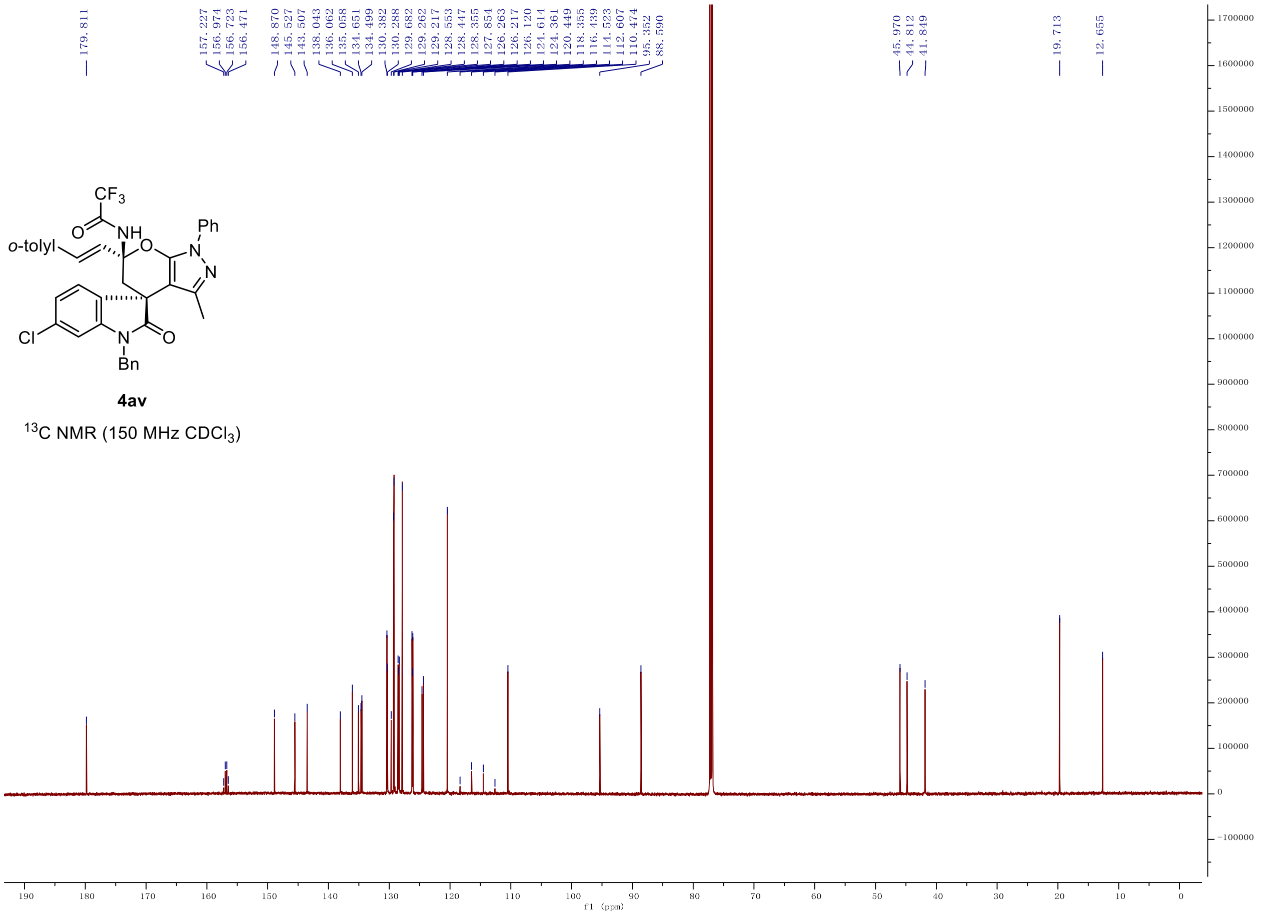

4av

^{13}C NMR (150 MHz CDCl$_3$)

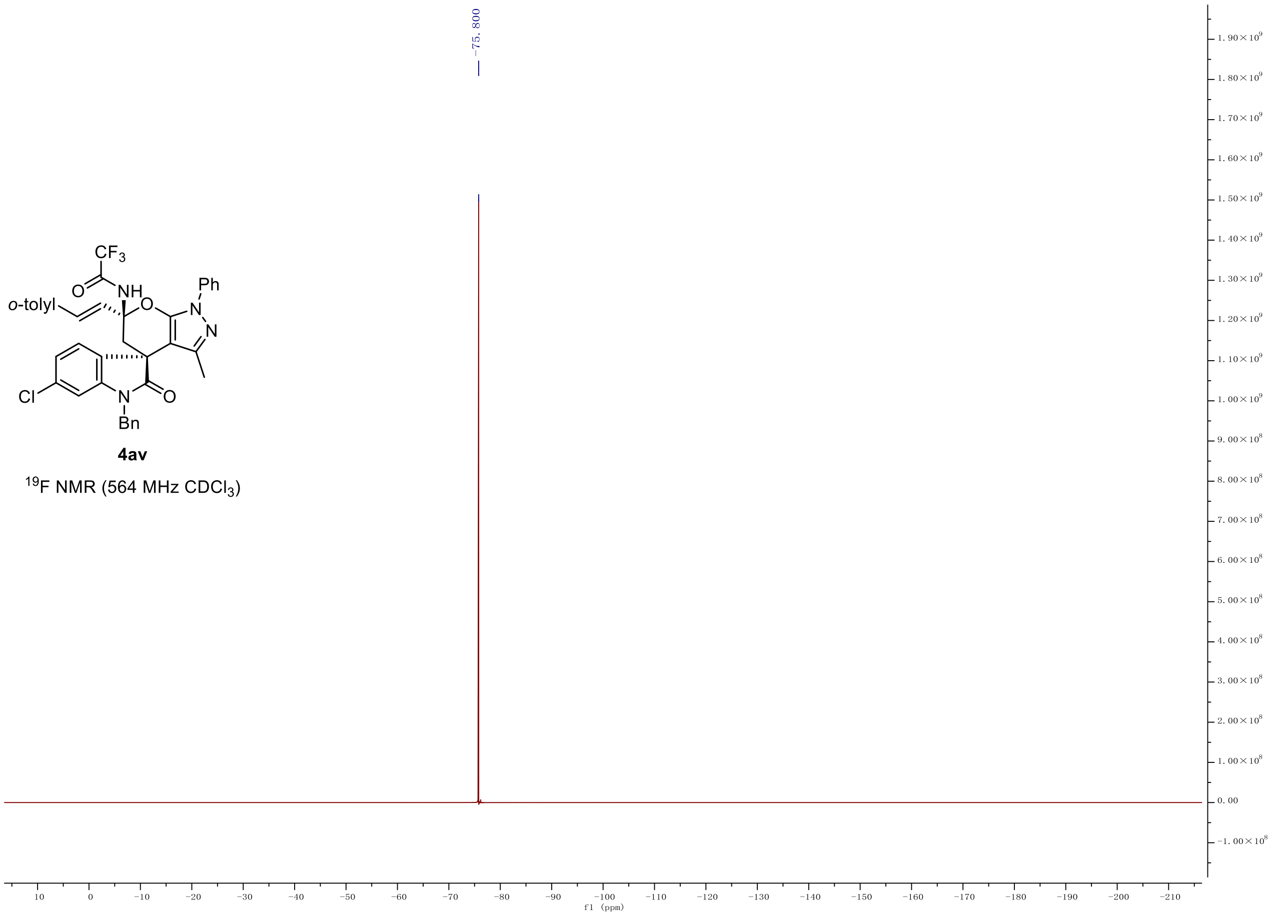

4av

^{19}F NMR (564 MHz CDCl$_3$)

Peak Analysis Report

Detector A Channel 1 254nm

No.	Ret. Time	Height (mAu)	Area (mAu*min)	Rel. Area (%)
1	7.217	1231303	18735013	49.801
2	9.096	1109759	18884914	50.199
Total		2341062	37619927	100.000

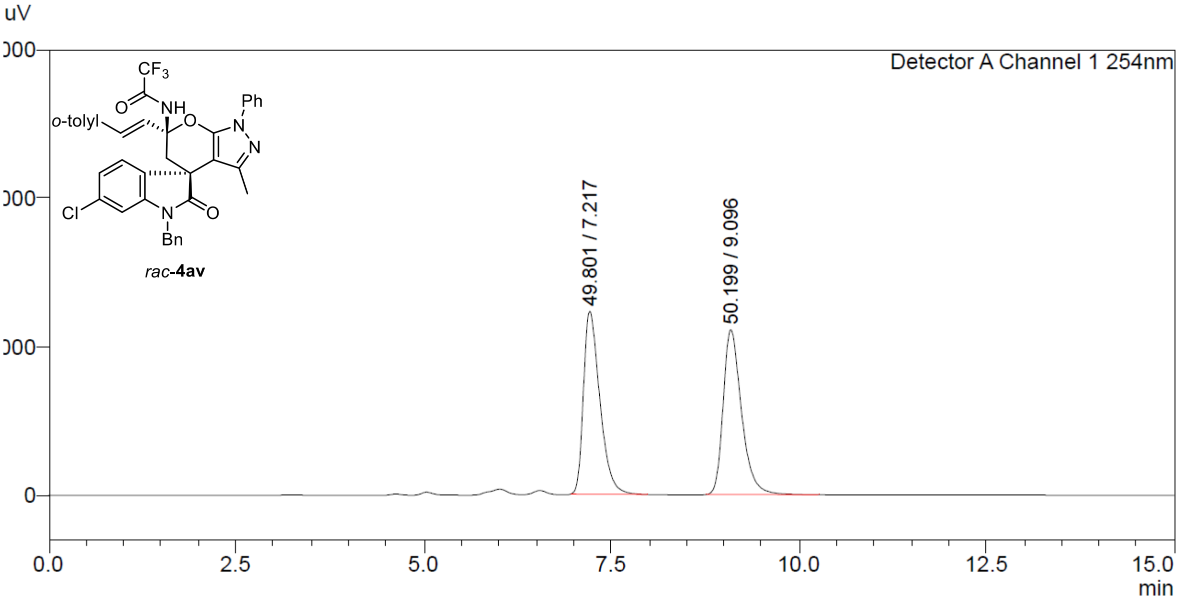

Peak Analysis Report

Detector A Channel 1 254nm

No.	Ret. Time	Height (mAu)	Area (mAu*min)	Rel. Area (%)
1	7.307	6604	80857	0.246
2	9.074	1884592	32787580	99.754
Total		1891197	32868437	100.000

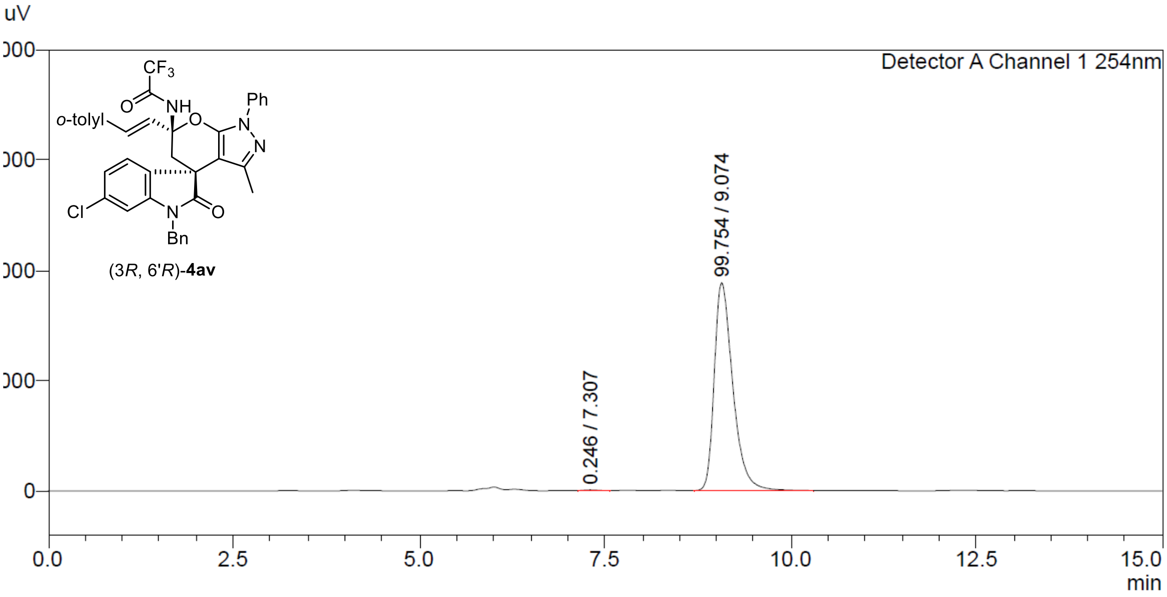

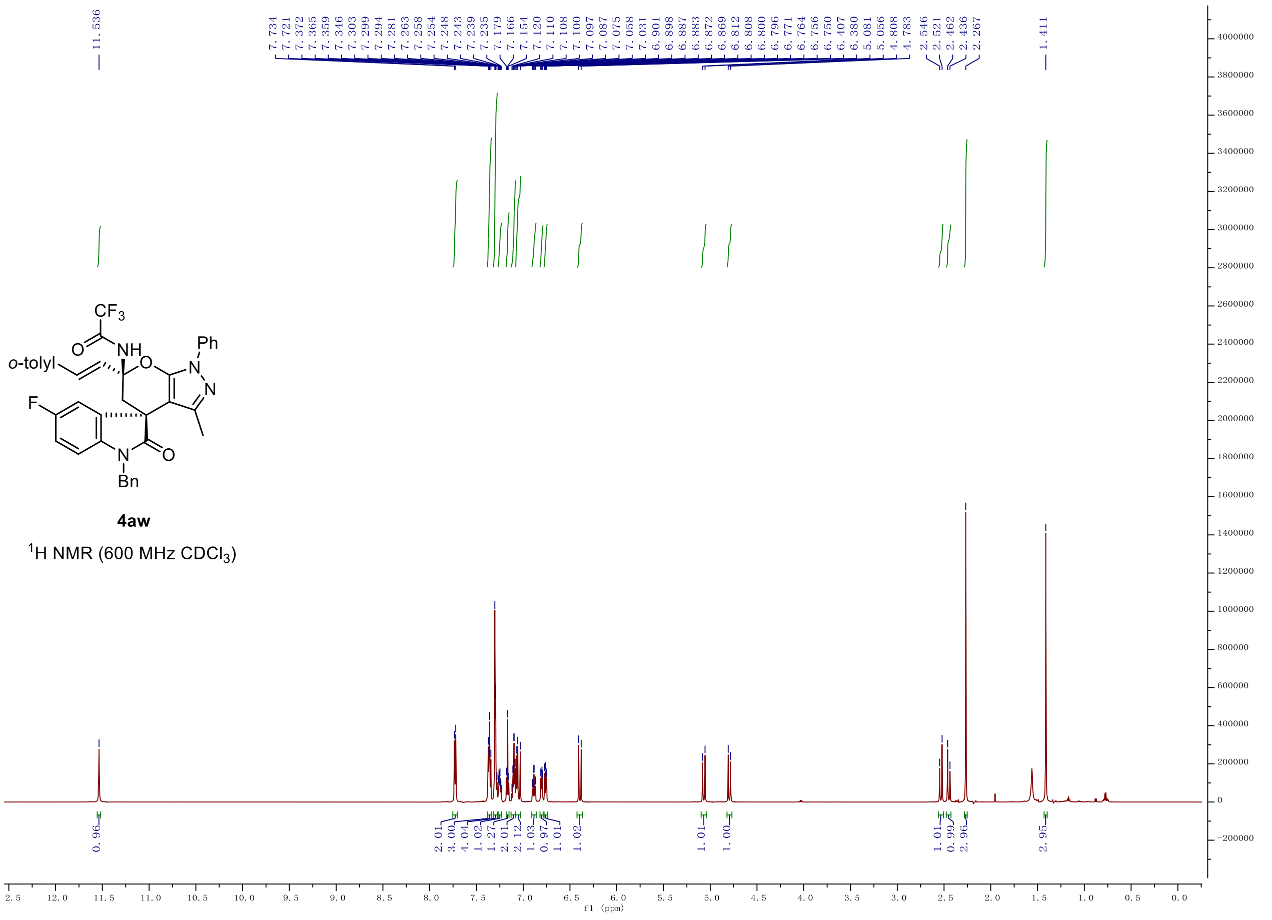

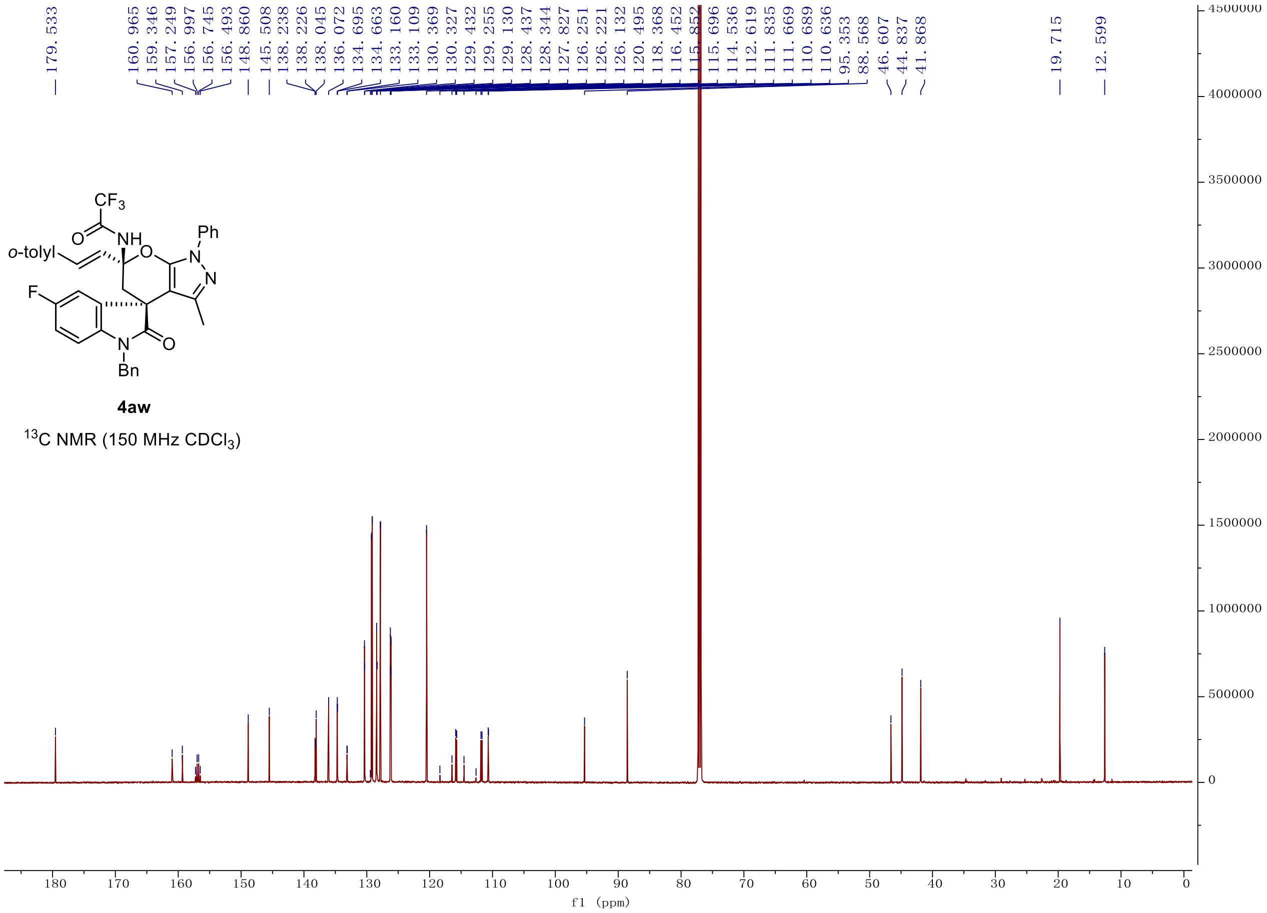
179.533
160.965
159.346
157.249
156.997
156.745
156.493
148.860
145.508
138.238
138.226
138.045
136.072
134.695
134.663
133.160
133.109
130.369
130.327
129.432
129.255
129.130
128.437
128.344
127.827
126.251
126.221
126.132
120.495
118.368
116.452
115.852
115.696
114.536
112.619
111.835
111.669
110.689
110.636
95.353
88.568
46.607
44.837
41.868
19.715
12.599
CF3
NH
O
Ph
o-tolyl
N
F
Bn
4aw
13C NMR (150 MHz CDCl3)
f1 (ppm)

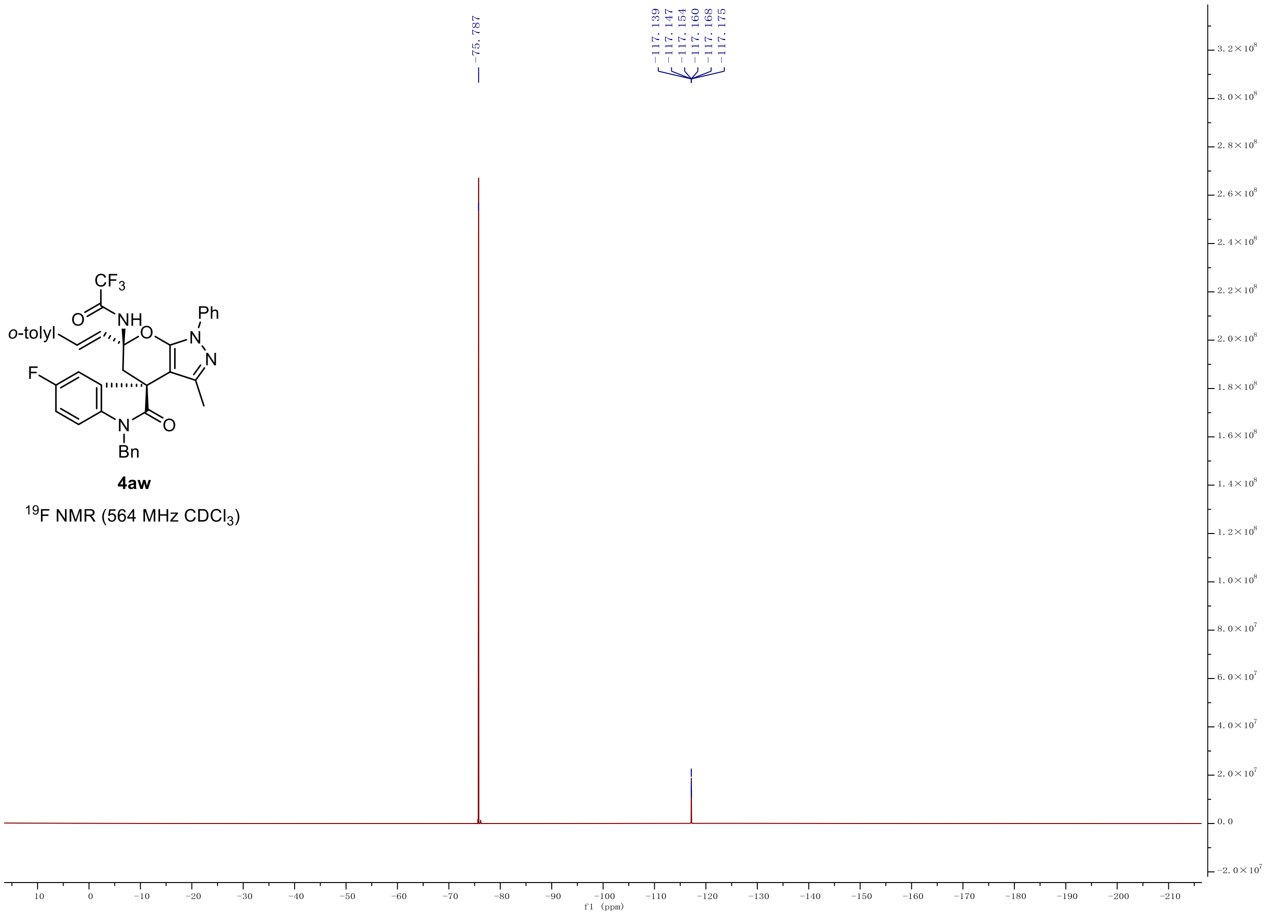
-75.787
-117.139
-117.147
-117.154
-117.160
-117.168
-117.175
CF3
NH
O
Ph
o-tolyl
N
F
Bn
4aw
19F NMR (564 MHz CDCl3)
f1 (ppm)

Peak Analysis Report

Detector A Channel 1 254nm

No.	Ret. Time	Height (mAu)	Area (mAu*min)	Rel. Area (%)
1	9.757	409185	13248201	49.413
2	11.490	393623	13562814	50.587
Total		802808	26811015	100.000

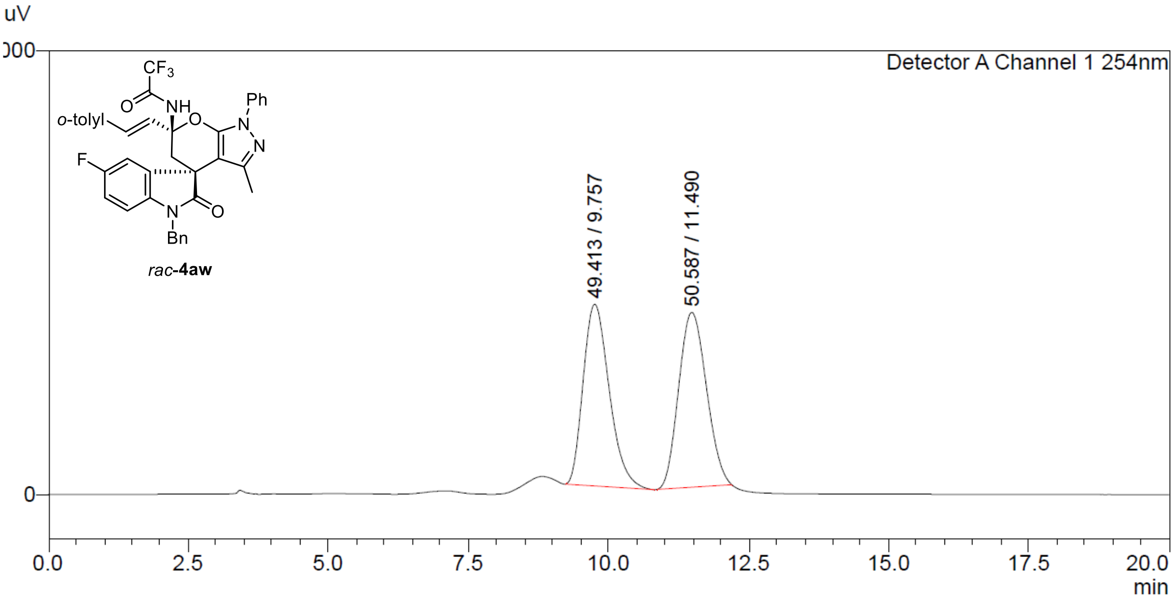

Peak Analysis Report

Detector A Channel 1 254nm

No.	Ret. Time	Height (mAu)	Area (mAu*min)	Rel. Area (%)
1	9.762	1959265	60520744	99.888
2	11.430	2233	67635	0.112
Total		1961498	60588379	100.000

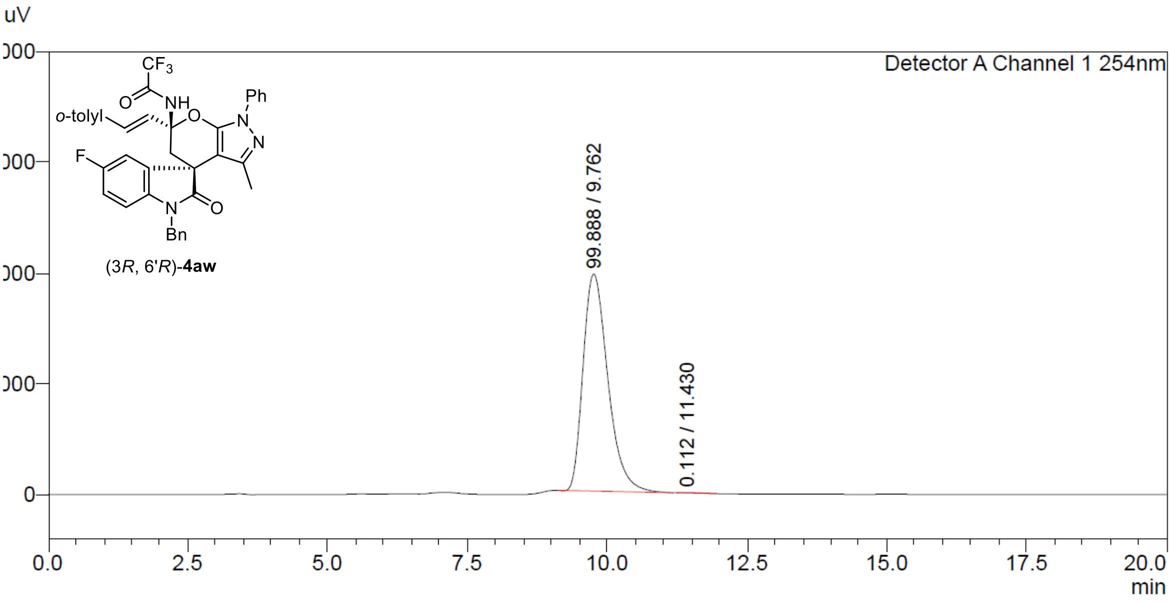

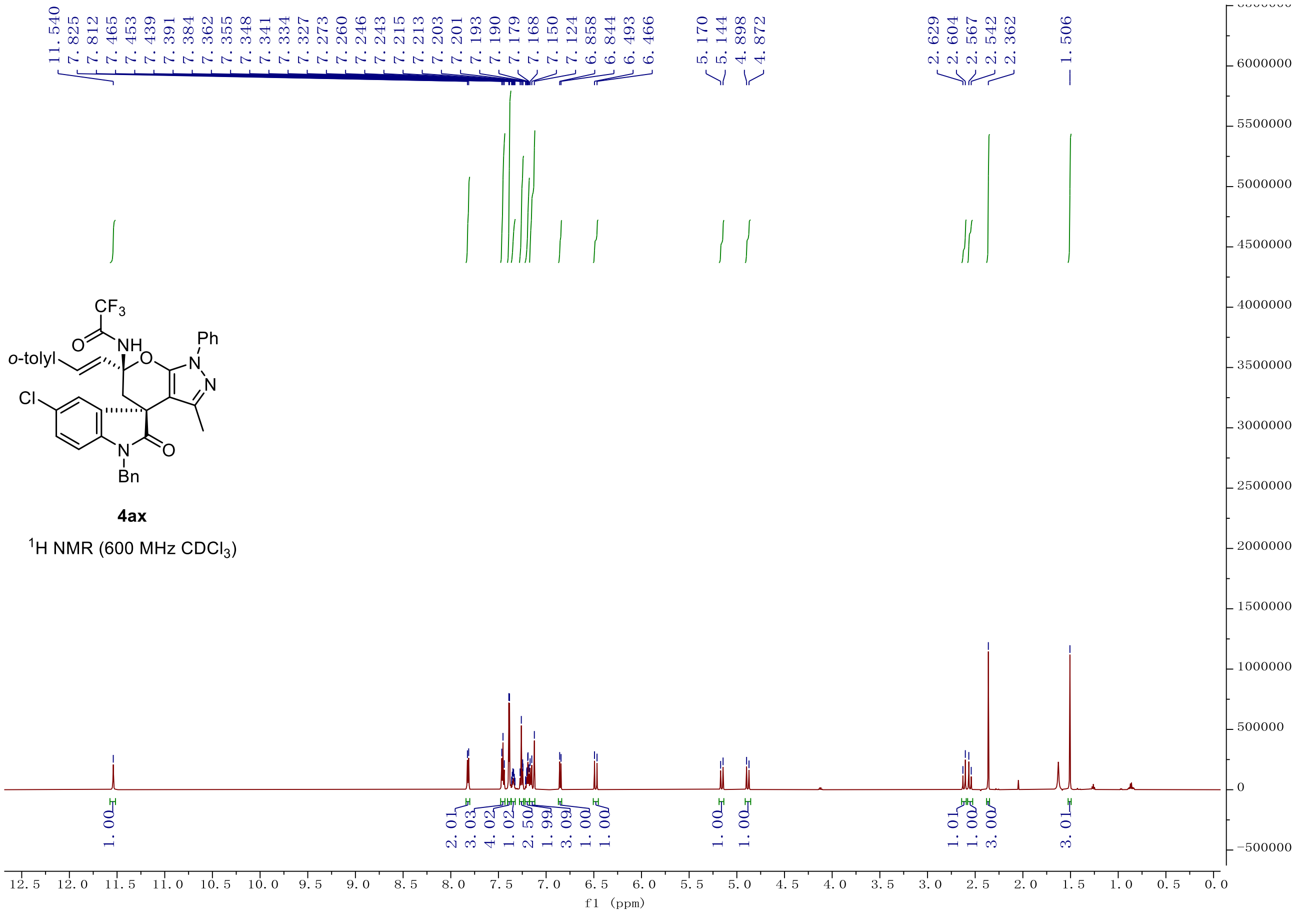

11.540
7.825
7.812
7.465
7.453
7.439
7.391
7.384
7.362
7.355
7.348
7.341
7.334
7.327
7.273
7.260
7.246
7.243
7.215
7.213
7.203
7.201
7.193
7.190
7.179
7.168
7.150
7.124
6.858
6.844
6.493
6.466
5.170
5.144
4.898
4.872
2.629
2.604
2.567
2.542
2.362
1.506
4ax
^{1}H NMR (600 MHz CDCl$_3$)
f1 (ppm)

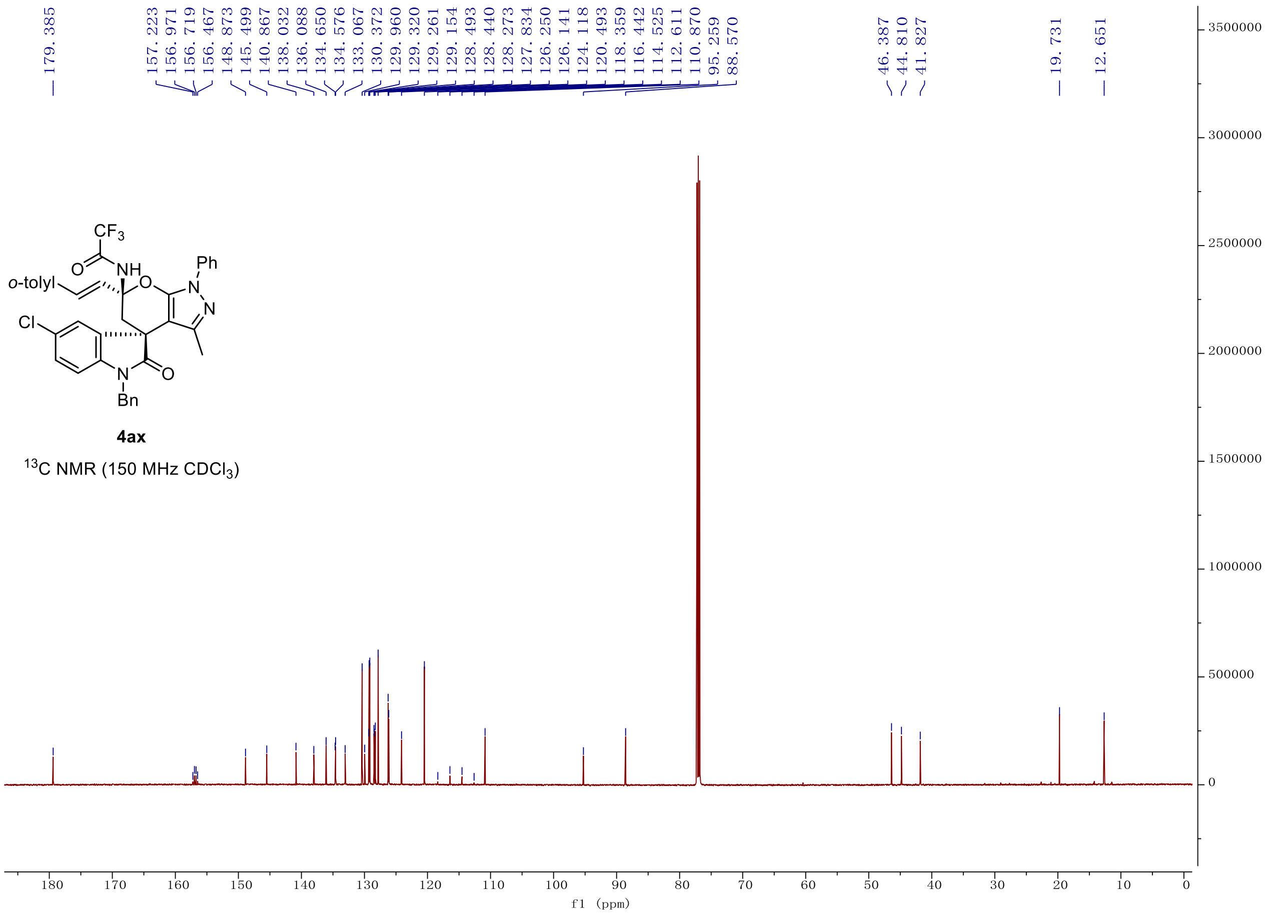

179.385
157.223
156.971
156.719
156.467
148.873
145.499
140.867
138.032
136.088
134.650
134.576
133.067
130.372
129.960
129.320
129.261
129.154
128.493
128.440
128.273
127.834
126.250
126.141
124.118
120.493
118.359
116.442
114.525
112.611
110.870
95.259
88.570
46.387
44.810
41.827
19.731
12.651
4ax
^{13}C NMR (150 MHz CDCl$_3$)
f1 (ppm)

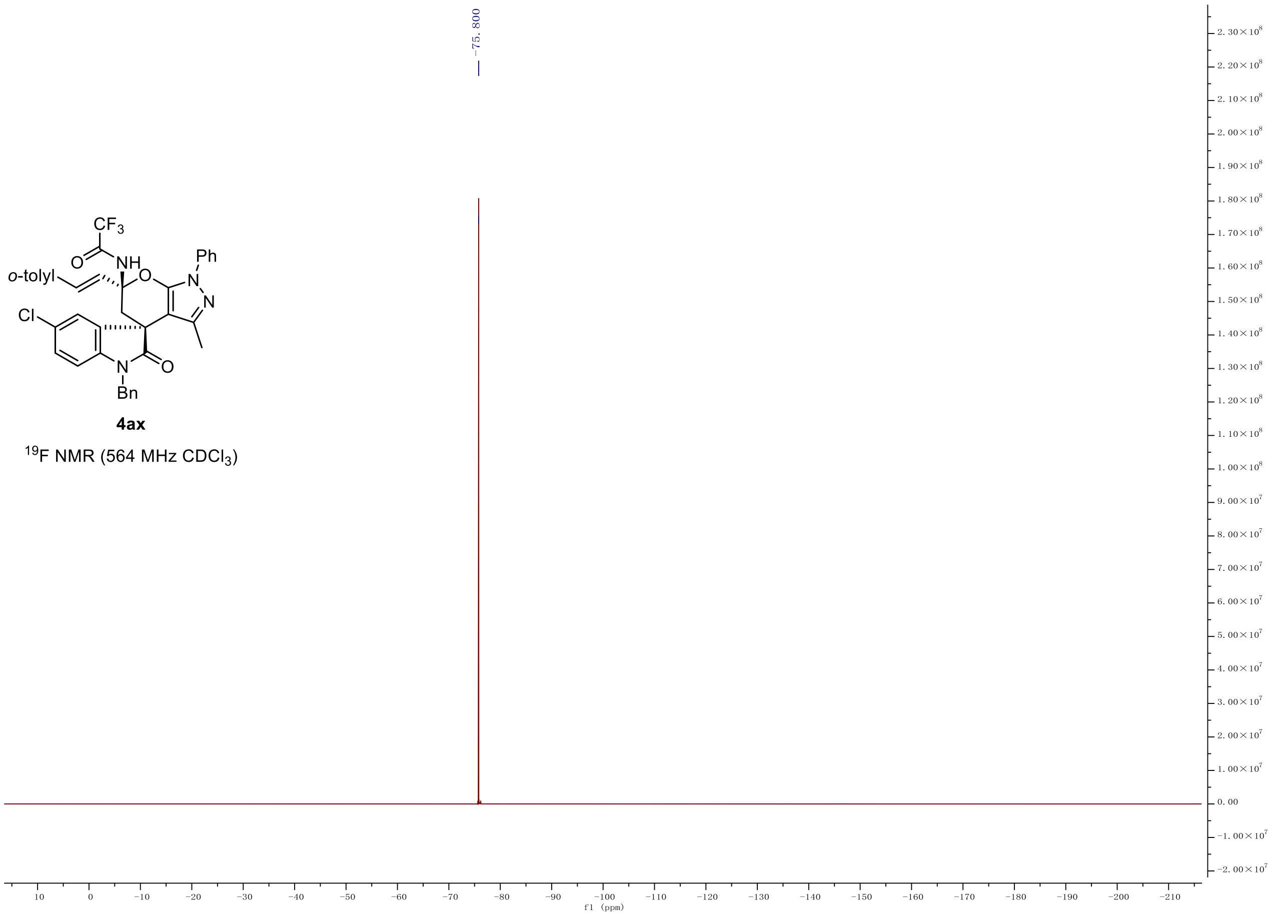

Peak Analysis Report

Detector A Channel 1 254nm

No.	Ret. Time	Height (mAu)	Area (mAu*min)	Rel. Area (%)
1	11.389	575924	15196988	50.055
2	12.504	548421	15163580	49.945
Total		1124345	30360568	100.000

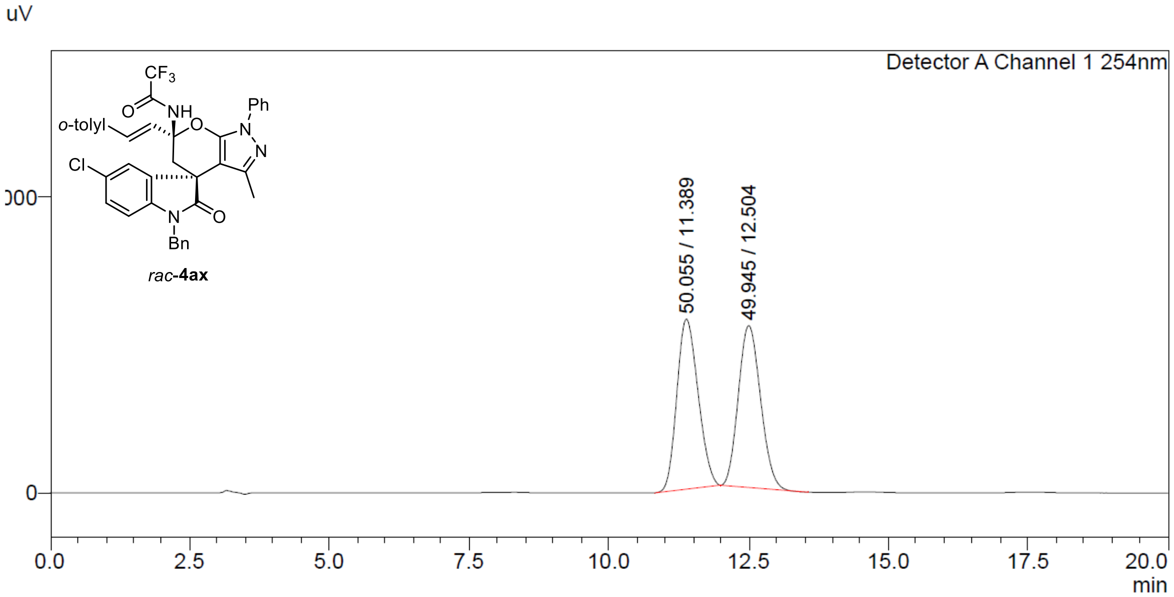

Peak Analysis Report

Detector A Channel 1 254nm

No.	Ret. Time	Height (mAu)	Area (mAu*min)	Rel. Area (%)
1	11.436	4893	125260	0.483
2	12.490	882917	25783050	99.517
Total		887811	25908311	100.000

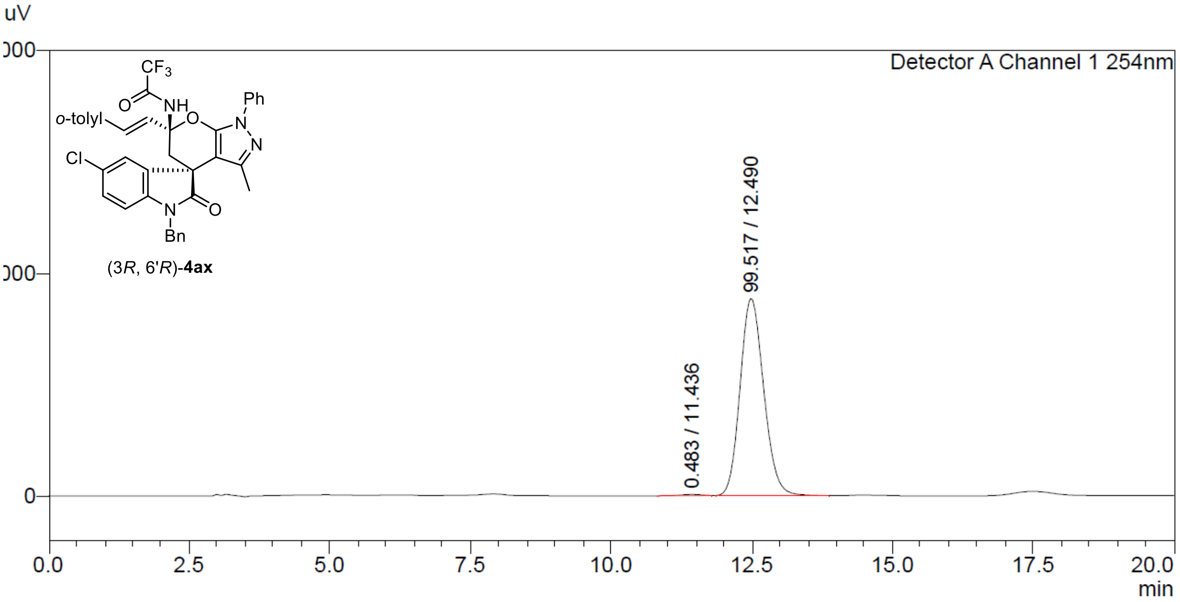

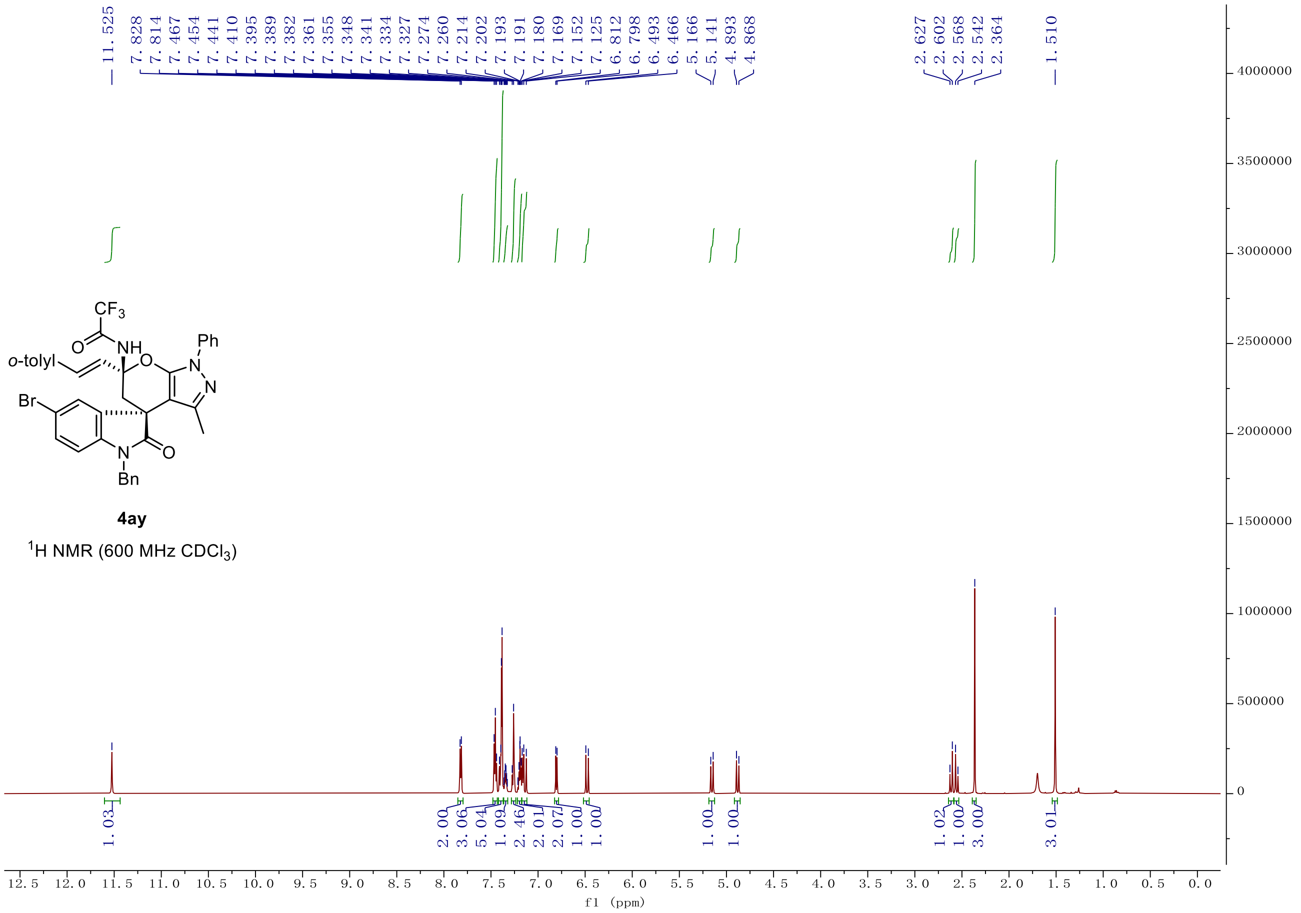

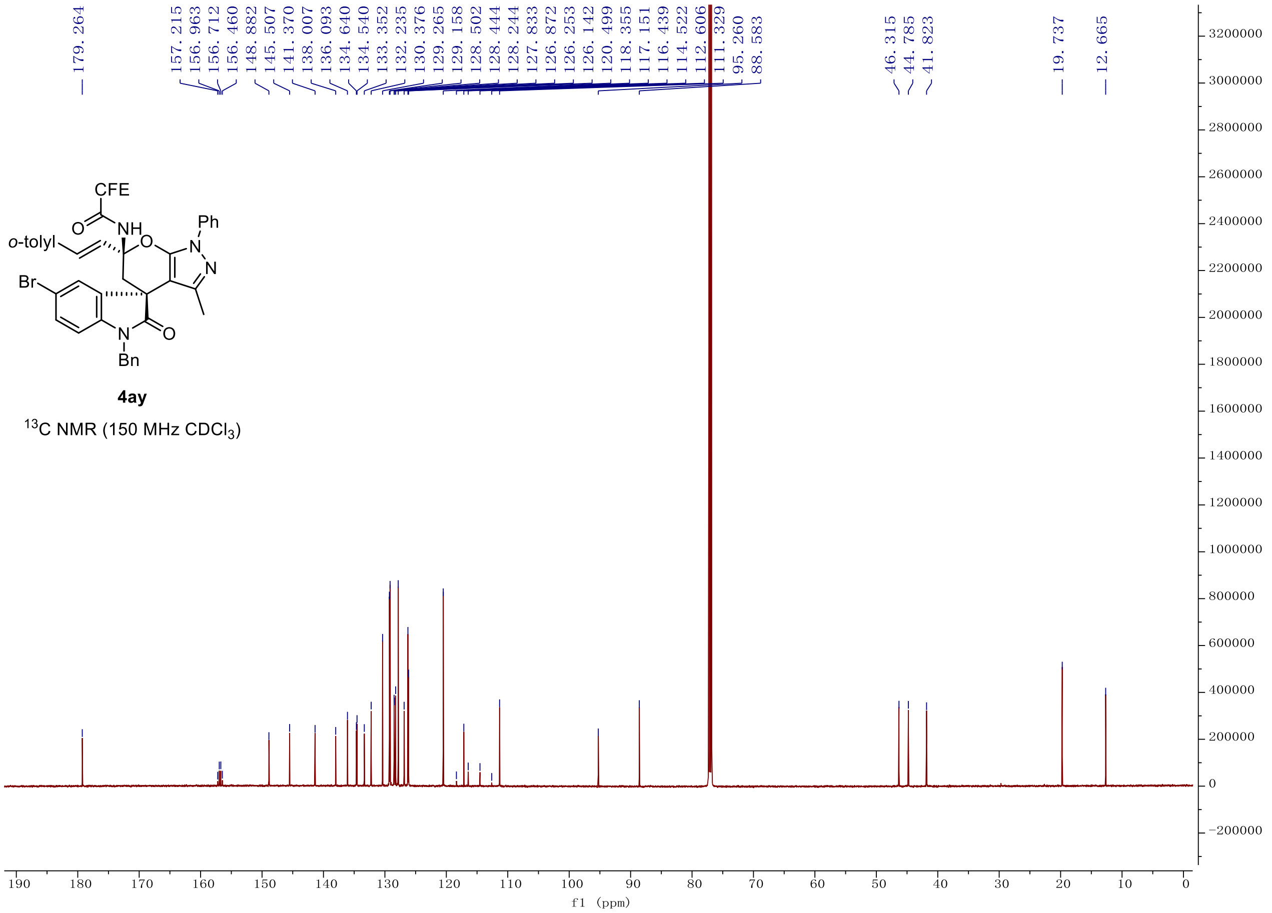
179.264
157.215
156.963
156.712
156.460
148.882
145.507
141.370
138.007
136.093
134.640
134.540
133.352
132.235
130.376
129.265
129.158
128.502
128.444
128.244
127.833
126.872
126.253
126.142
120.499
118.355
117.151
116.439
114.522
112.606
111.329
95.260
88.583
46.315
44.785
41.823
19.737
12.665
CFE
NH
O
Ph
o-tolyl
O
N
N
Br
N
O
Bn
4ay
13C NMR (150 MHz CDCl3)
f1 (ppm)

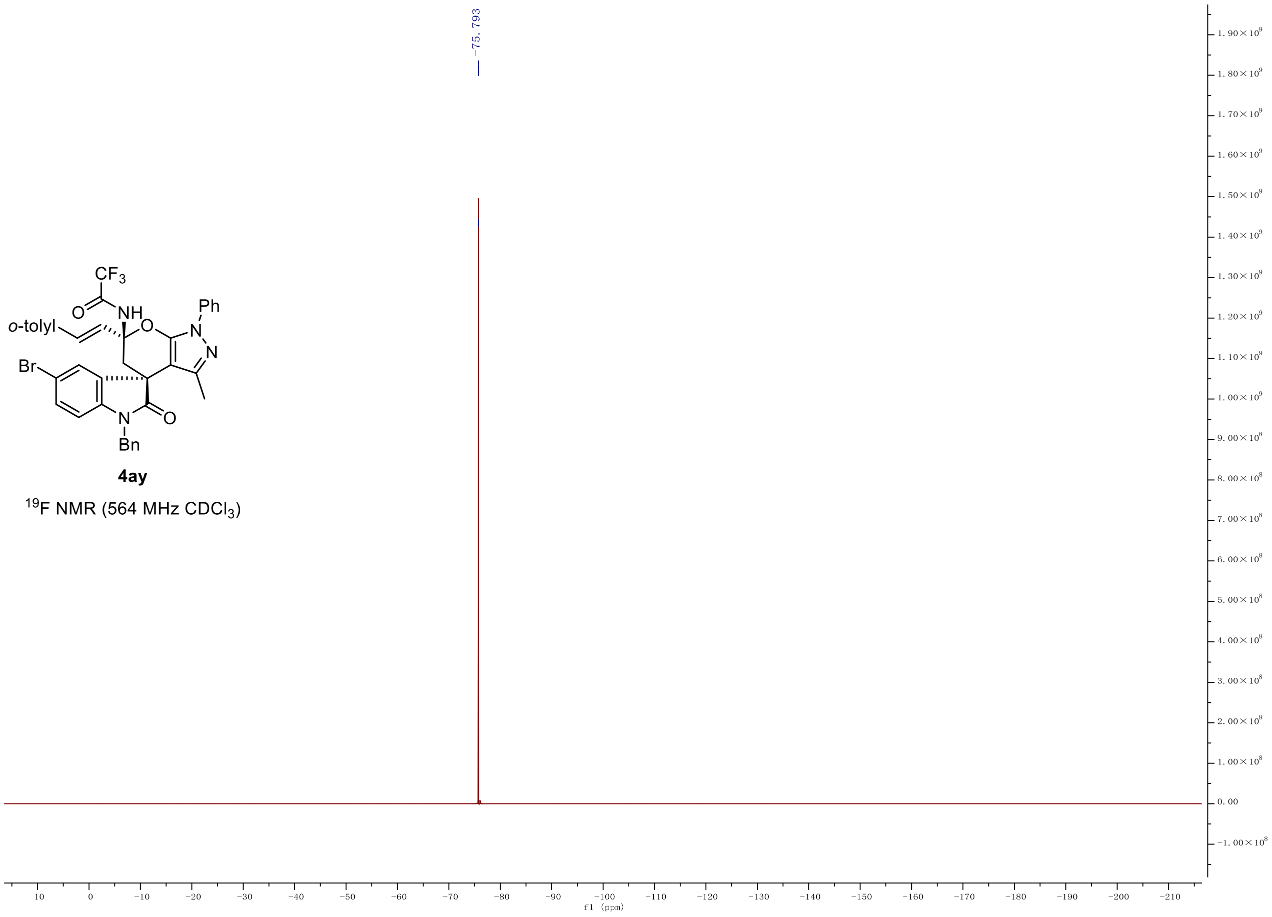
-75.793
CF3
O
NH
Ph
o-tolyl
O
N
N
Br
N
O
Bn
4ay
19F NMR (564 MHz CDCl3)
f1 (ppm)

Peak Analysis Report

Detector A Channel 1 254nm

No.	Ret. Time	Height (mAu)	Area (mAu*min)	Rel. Area (%)
1	11.665	365077	10282281	49.575
2	13.126	341663	10458418	50.425
Total		706739	20740698	100.000

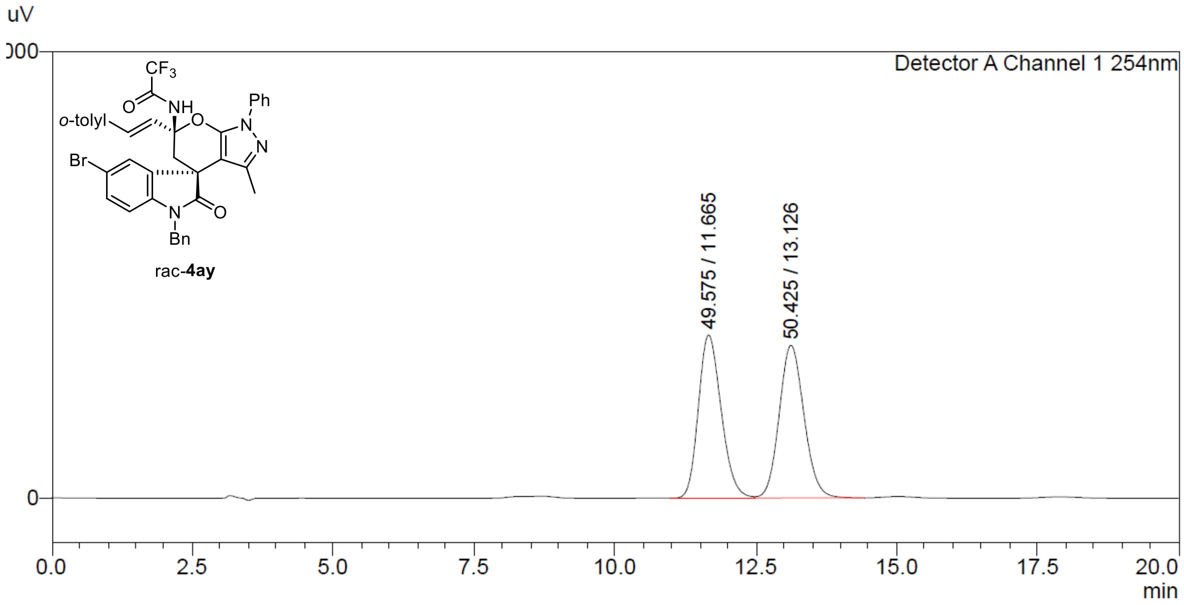

Peak Analysis Report

Detector A Channel 1 254nm

No.	Ret. Time	Height (mAu)	Area (mAu*min)	Rel. Area (%)
1	11.721	3415	87700	0.232
2	13.083	1169996	37662159	99.768
Total		1173412	37749859	100.000

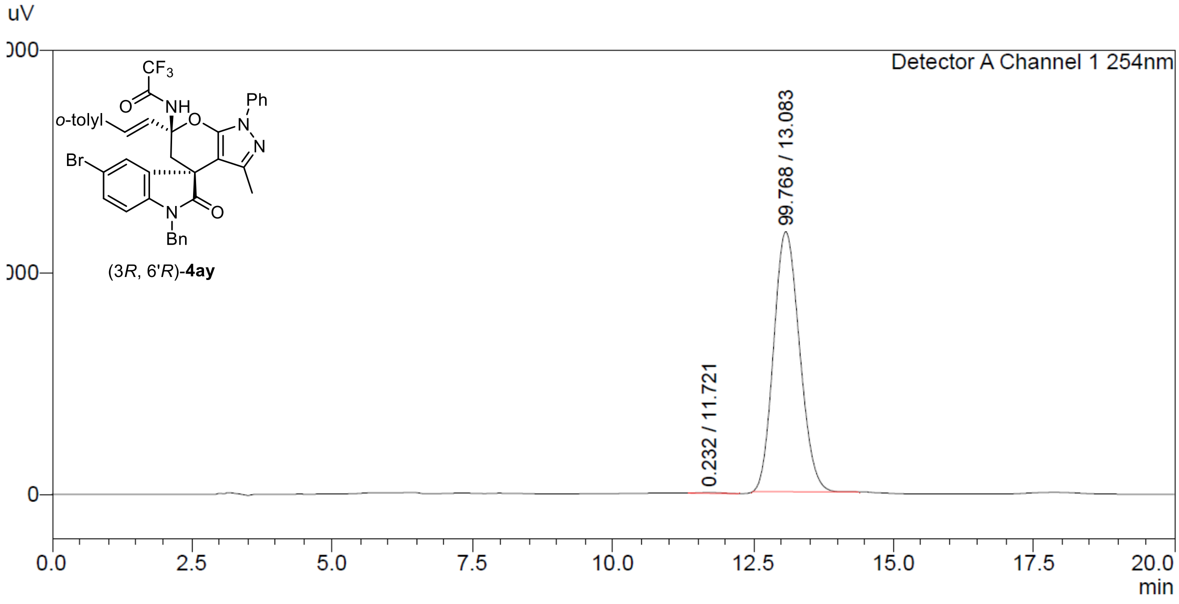

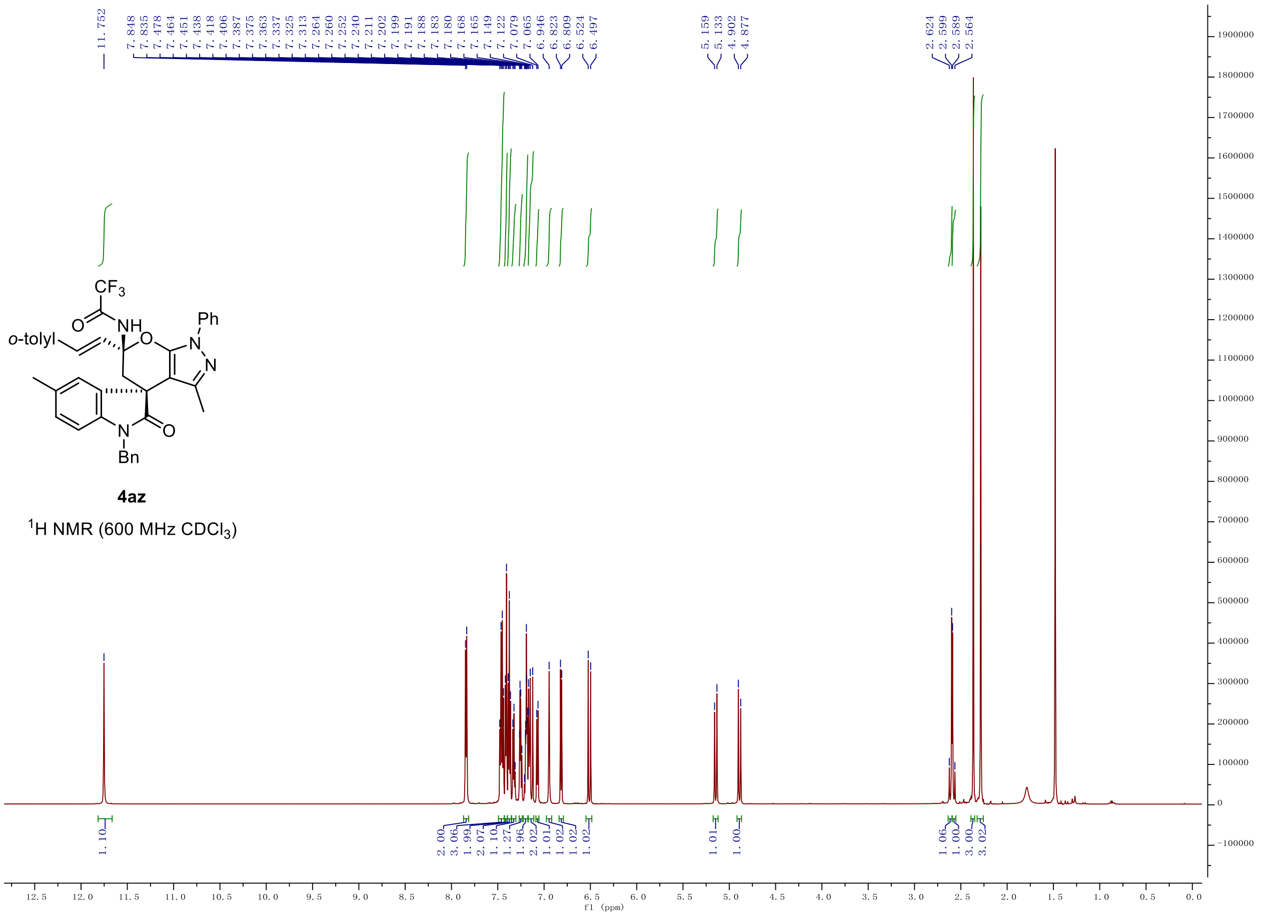

4az

^1H NMR (600 MHz CDCl$_3$)

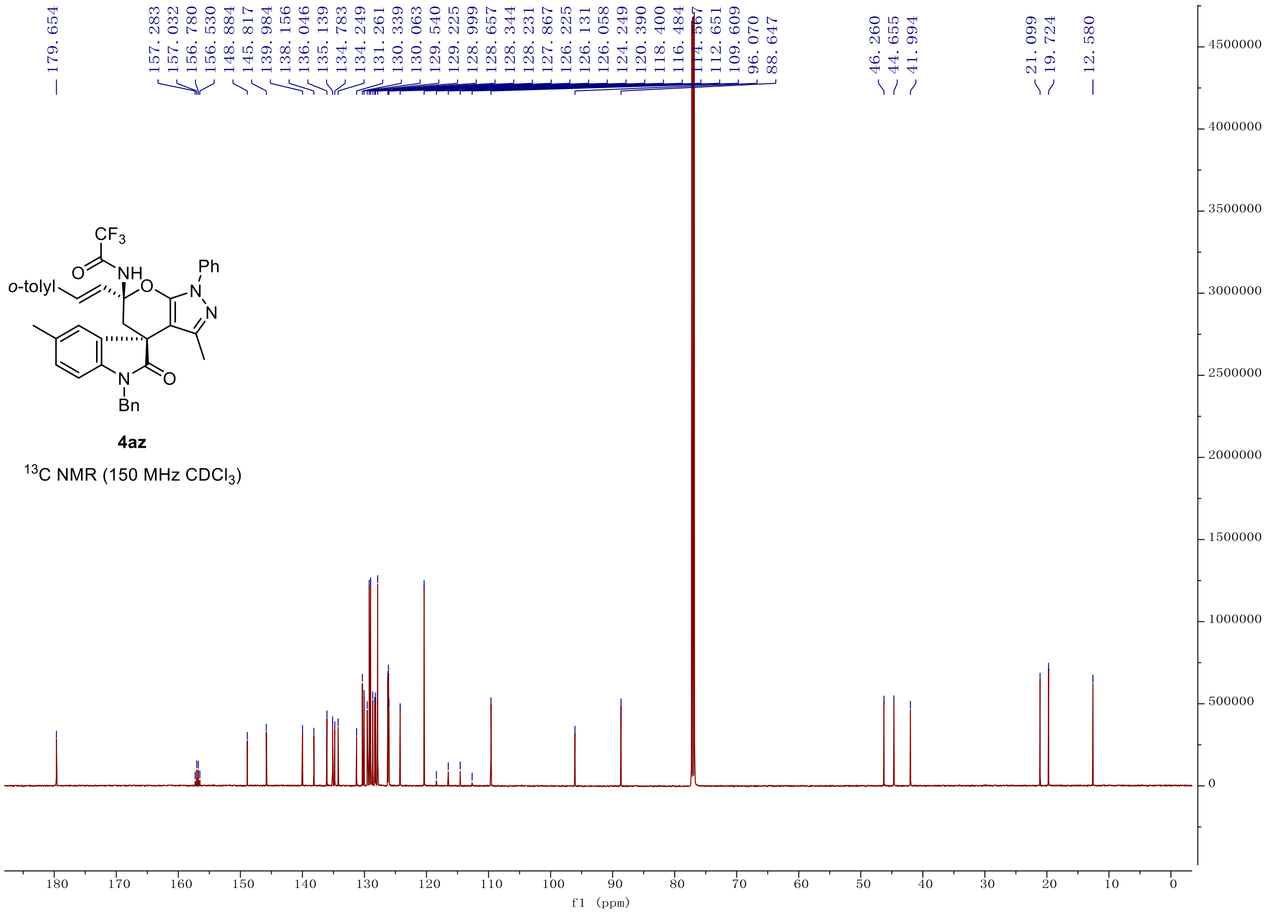

4az

^{13}C NMR (150 MHz CDCl$_3$)

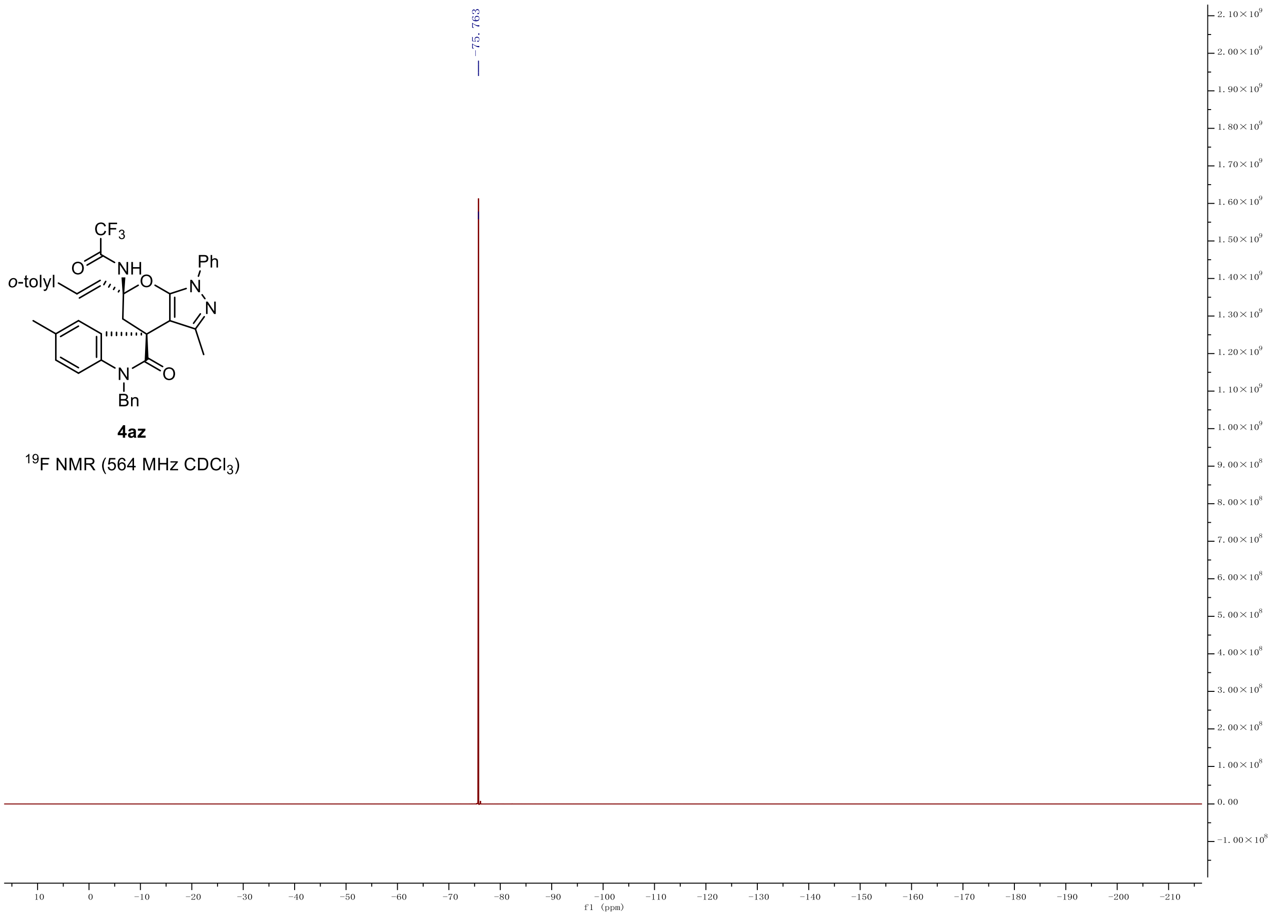

Peak Analysis Report

Detector A Channel 1 254nm

No.	Ret. Time	Height (mAu)	Area (mAu*min)	Rel. Area (%)
1	5.728	2441235	25232722	49.681
2	6.586	1743915	25556597	50.319
Total		4185150	50789319	100.000

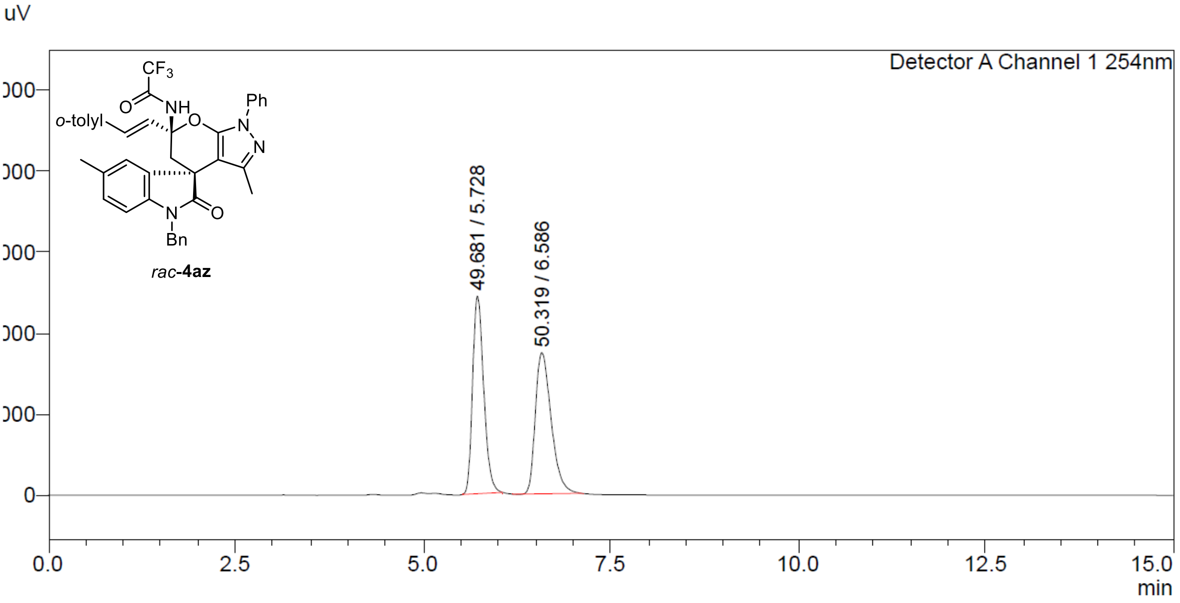

Peak Analysis Report

Detector A Channel 1 254nm

No.	Ret. Time	Height (mAu)	Area (mAu*min)	Rel. Area (%)
1	5.720	51904	519862	1.202
2	6.580	2656817	42732495	98.798
Total		2708721	43252357	100.000

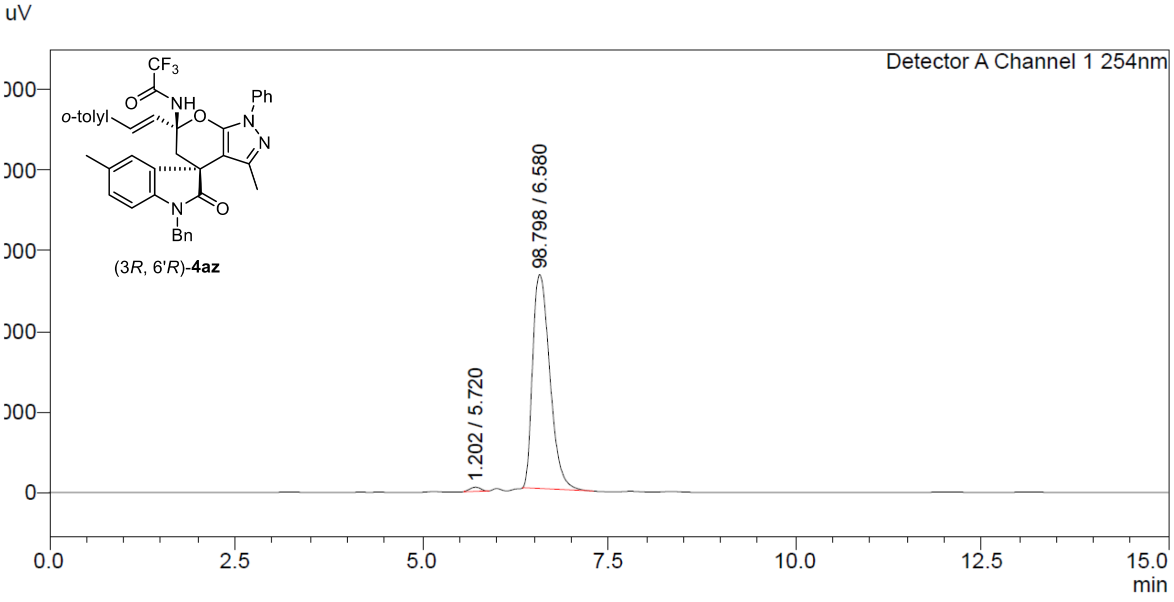

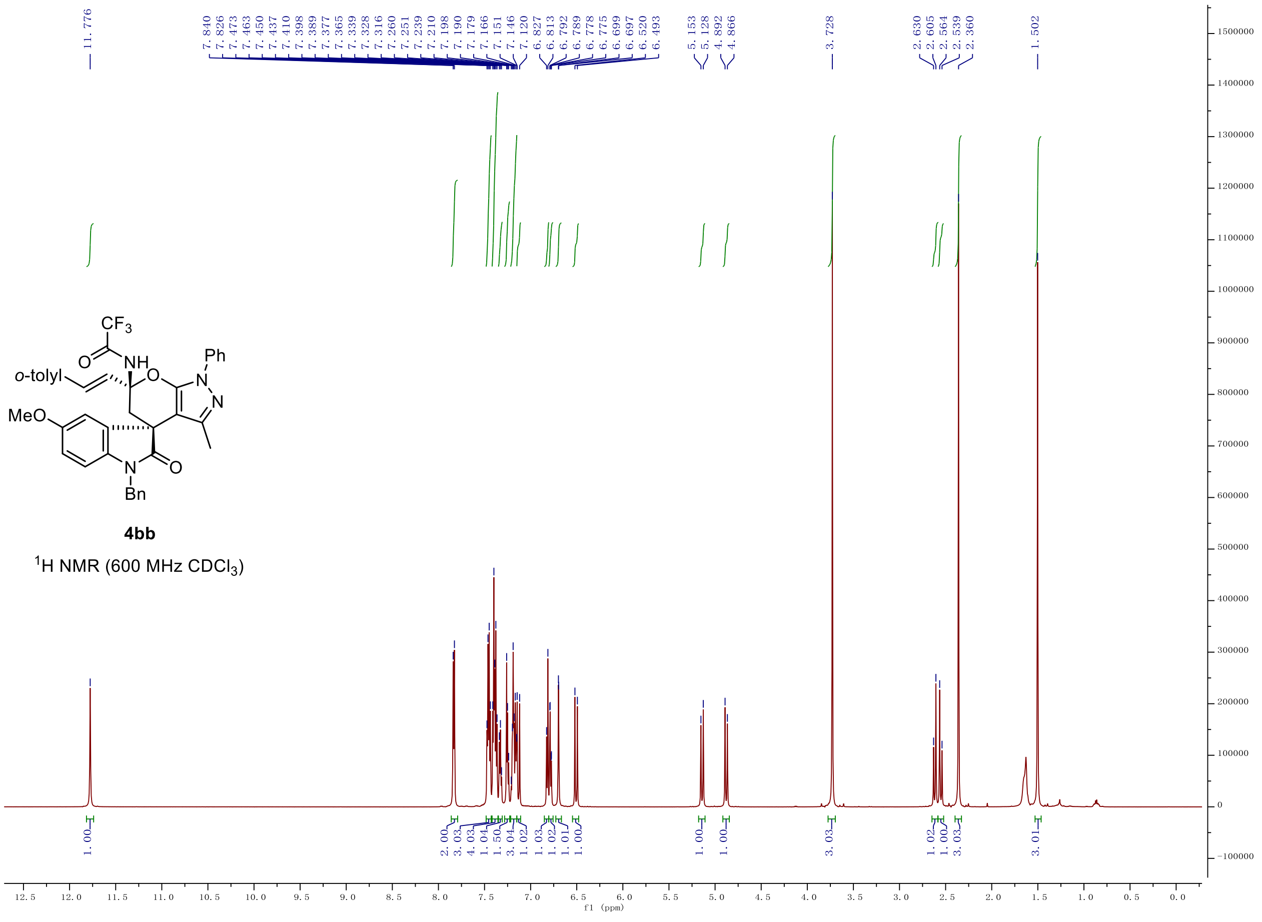

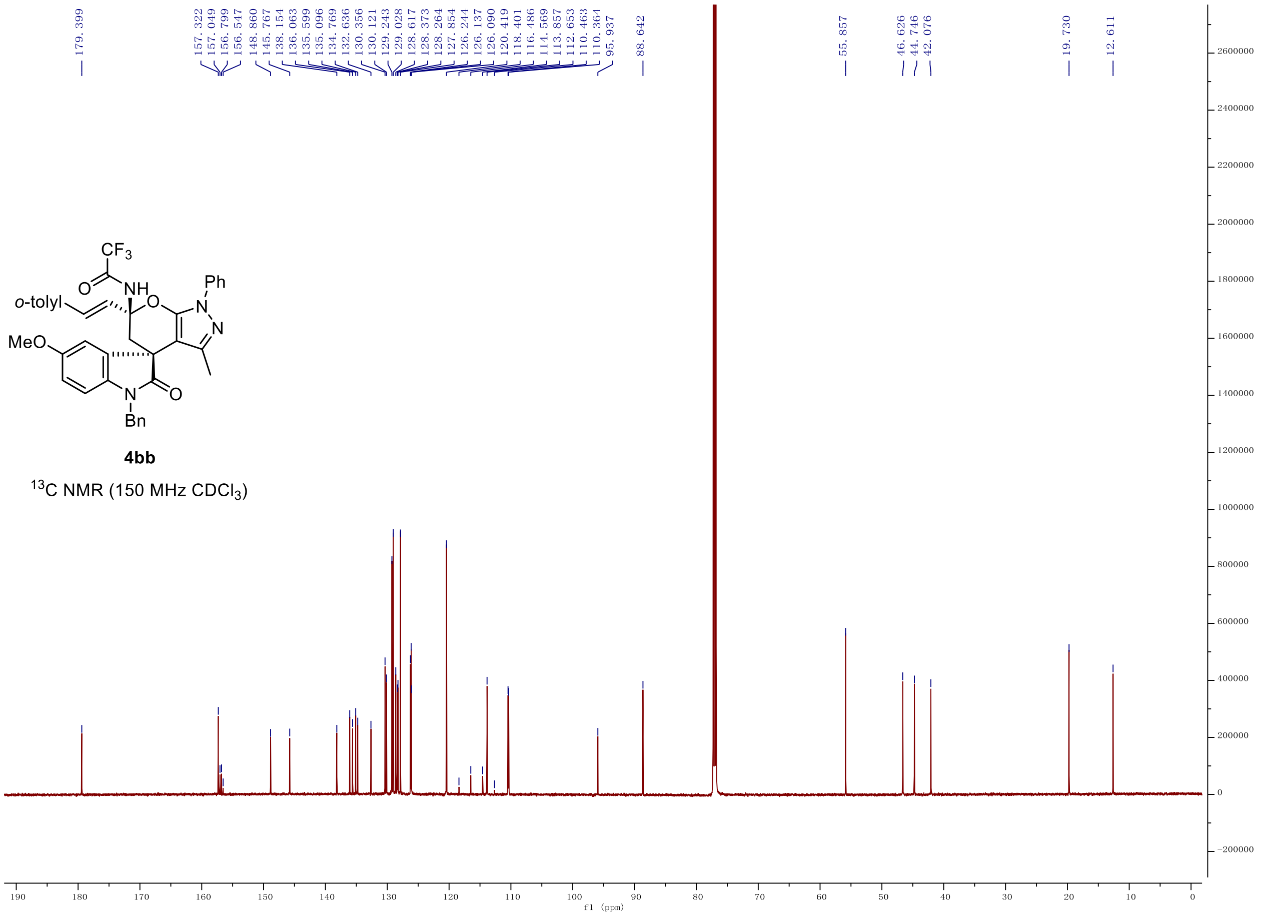
179.399
157.322
157.049
156.799
156.547
148.860
145.767
138.154
136.063
135.599
135.096
134.769
132.636
130.356
130.121
129.243
129.028
128.617
128.373
128.264
127.854
126.244
126.137
126.090
120.419
118.401
116.486
114.569
113.857
112.653
110.463
110.364
95.937
88.642
55.857
46.626
44.746
42.076
19.730
12.611
CF3
O
NH
o-tolyl
O
Ph
N
N
MeO
N
O
Bn
4bb
13C NMR (150 MHz CDCl3)
f1 (ppm)

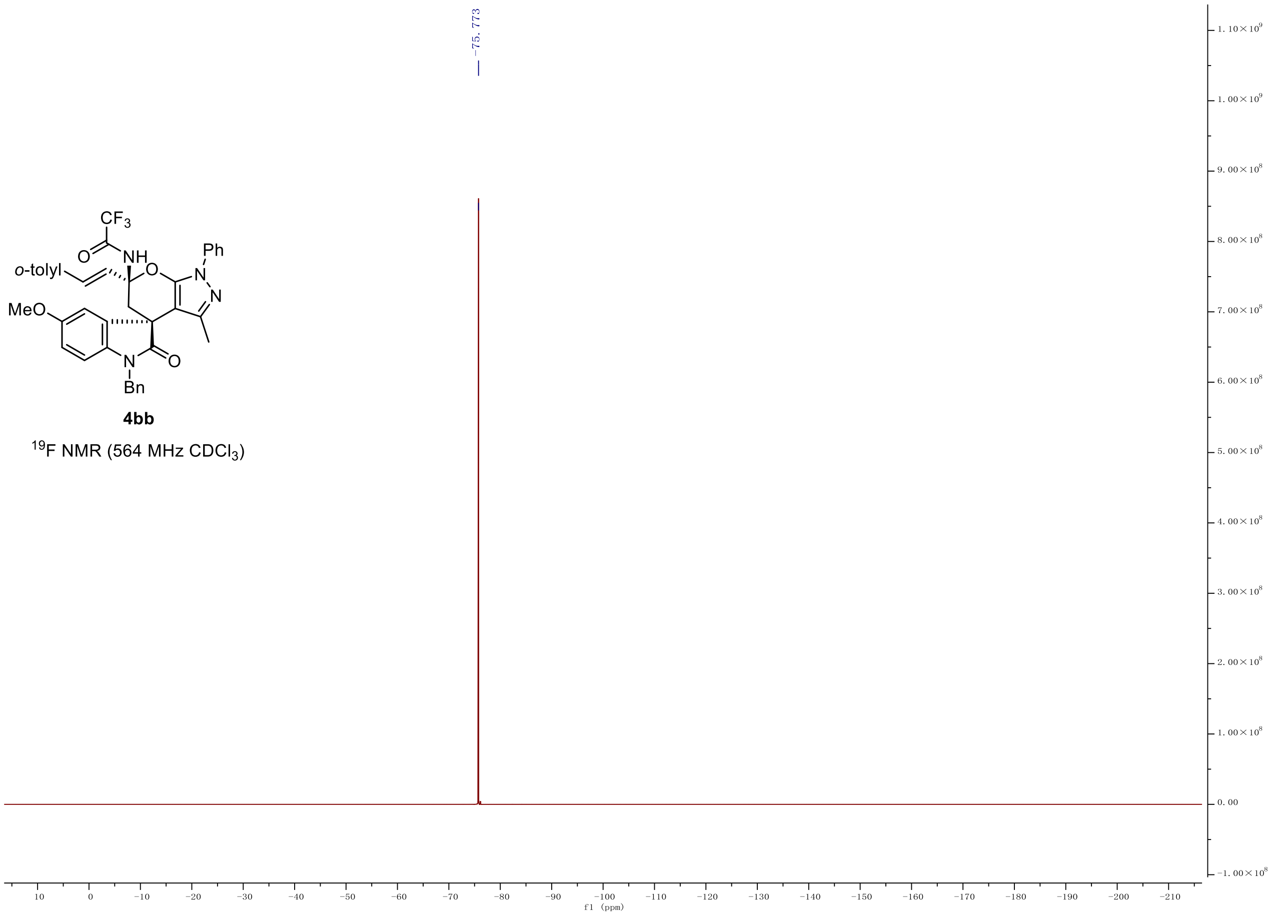
-75.773
CF3
O
NH
o-tolyl
O
Ph
N
N
MeO
N
O
Bn
4bb
19F NMR (564 MHz CDCl3)
f1 (ppm)

Peak Analysis Report

Detector A Channel 1 254nm

No.	Ret. Time	Height (mAu)	Area (mAu*min)	Rel. Area (%)
1	16.501	713162	25354012	49.967
2	18.398	613963	25387245	50.033
Total		1327125	50741256	100.000

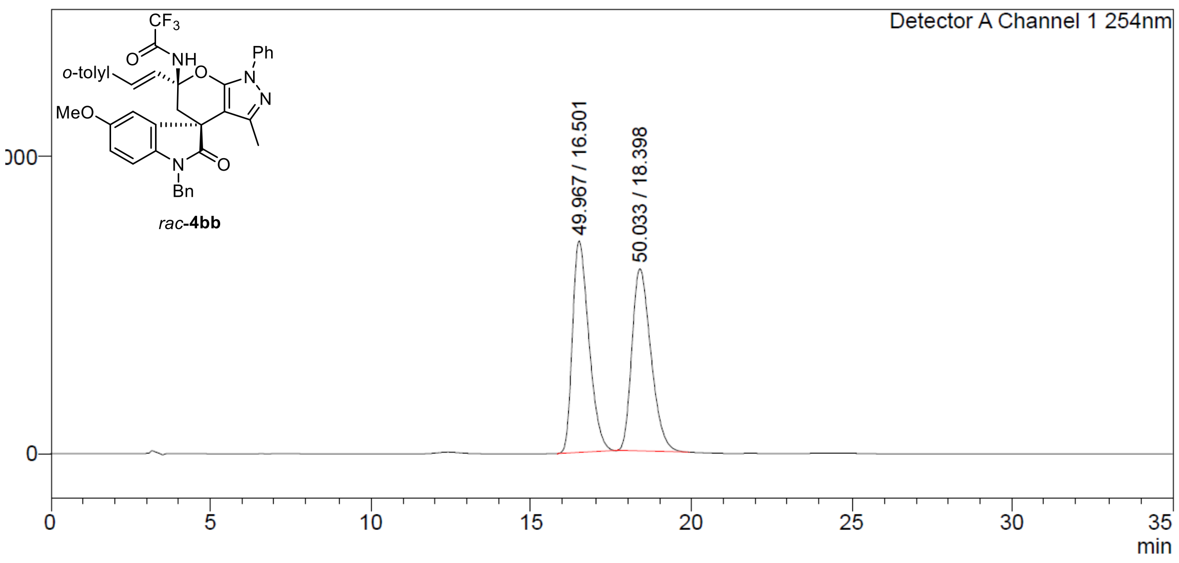

Peak Analysis Report

Detector A Channel 1 254nm

No.	Ret. Time	Height (mAu)	Area (mAu*min)	Rel. Area (%)
1	16.692	41652	1465424	2.452
2	18.308	1429986	58291405	97.548
Total		1471638	59756829	100.000

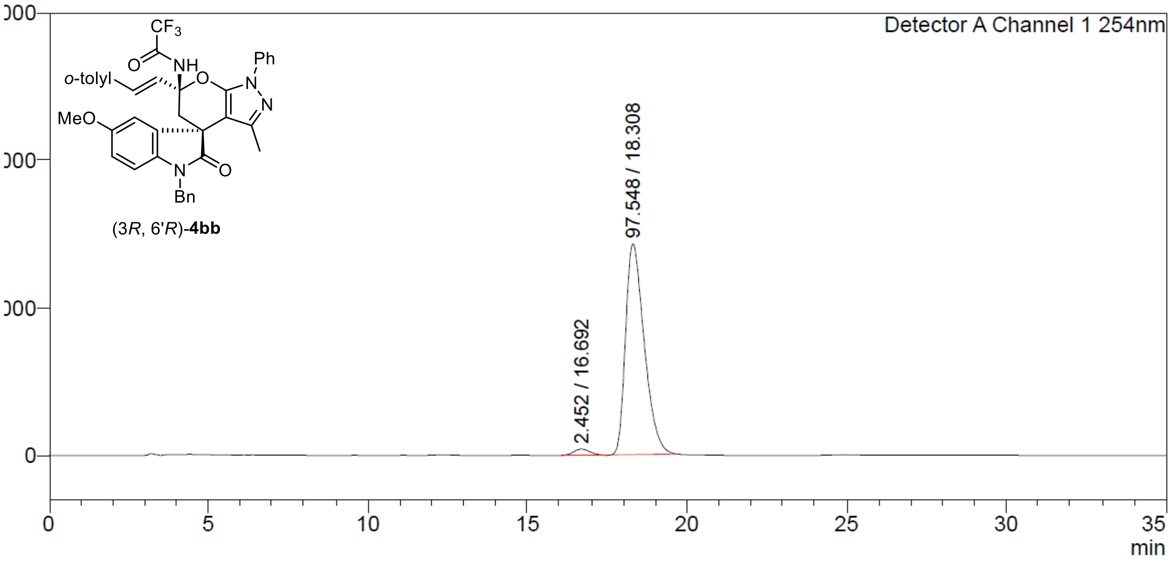

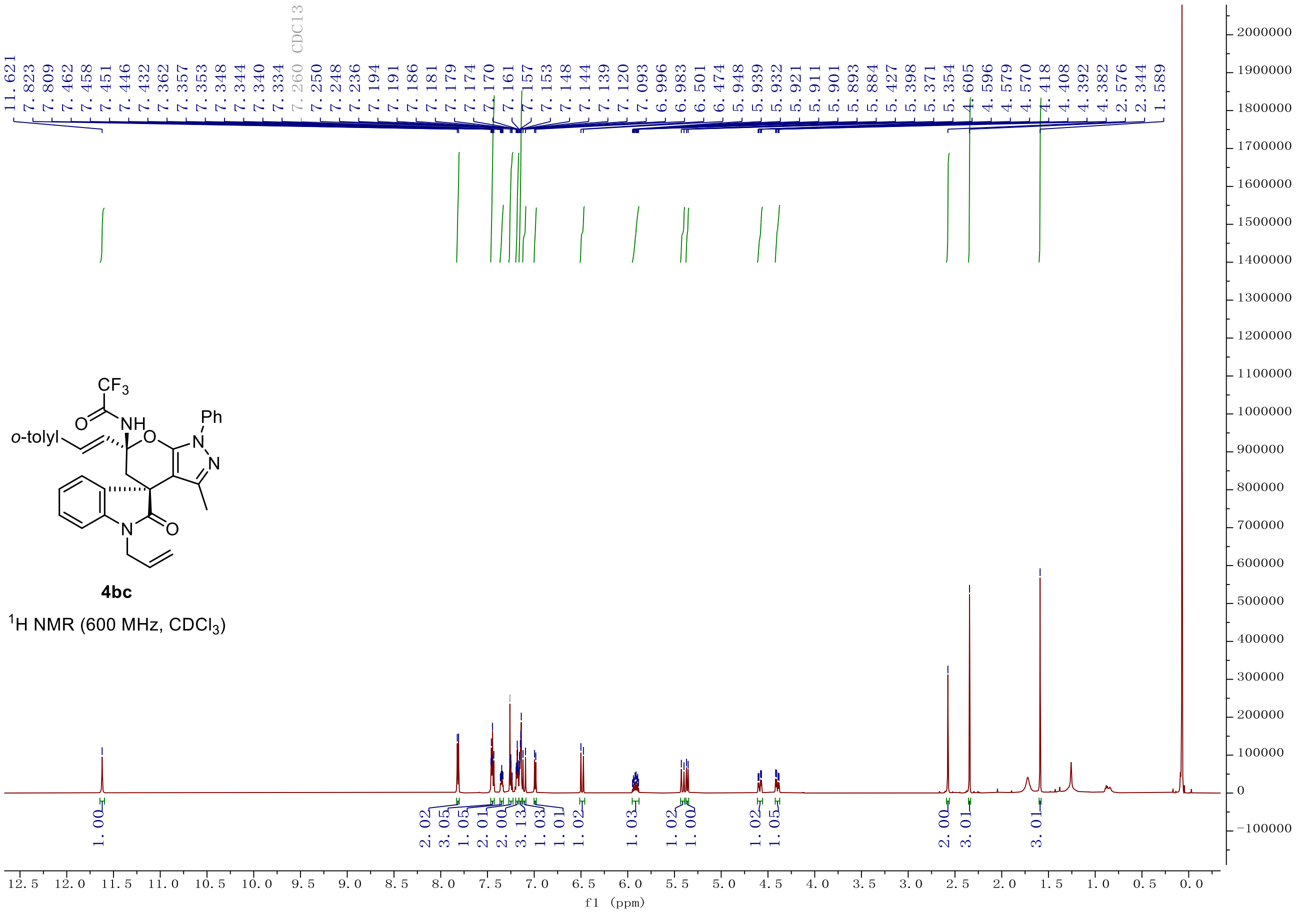
11.621 7.823 7.809 7.462 7.458 7.451 7.446 7.432 7.362 7.357 7.353 7.348 7.344 7.340 7.334 7.260 CDCl3 7.250 7.248 7.236 7.194 7.191 7.186 7.181 7.179 7.174 7.170 7.161 7.157 7.153 7.148 7.144 7.139 7.120 7.093 6.996 6.983 6.501 6.474 5.948 5.939 5.932 5.921 5.911 5.901 5.893 5.884 5.427 5.398 5.371 5.354 4.605 4.596 4.579 4.570 4.418 4.408 4.392 4.382 2.576 2.344 1.589
1.00 2.02 3.05 1.05 2.01 2.00 3.13 1.03 1.01 1.02 1.03 1.02 1.00 1.02 1.05 2.00 3.01 3.01
CF3
NH
Ph
o-tolyl
4bc
1H NMR (600 MHz, CDCl3)
f1 (ppm)

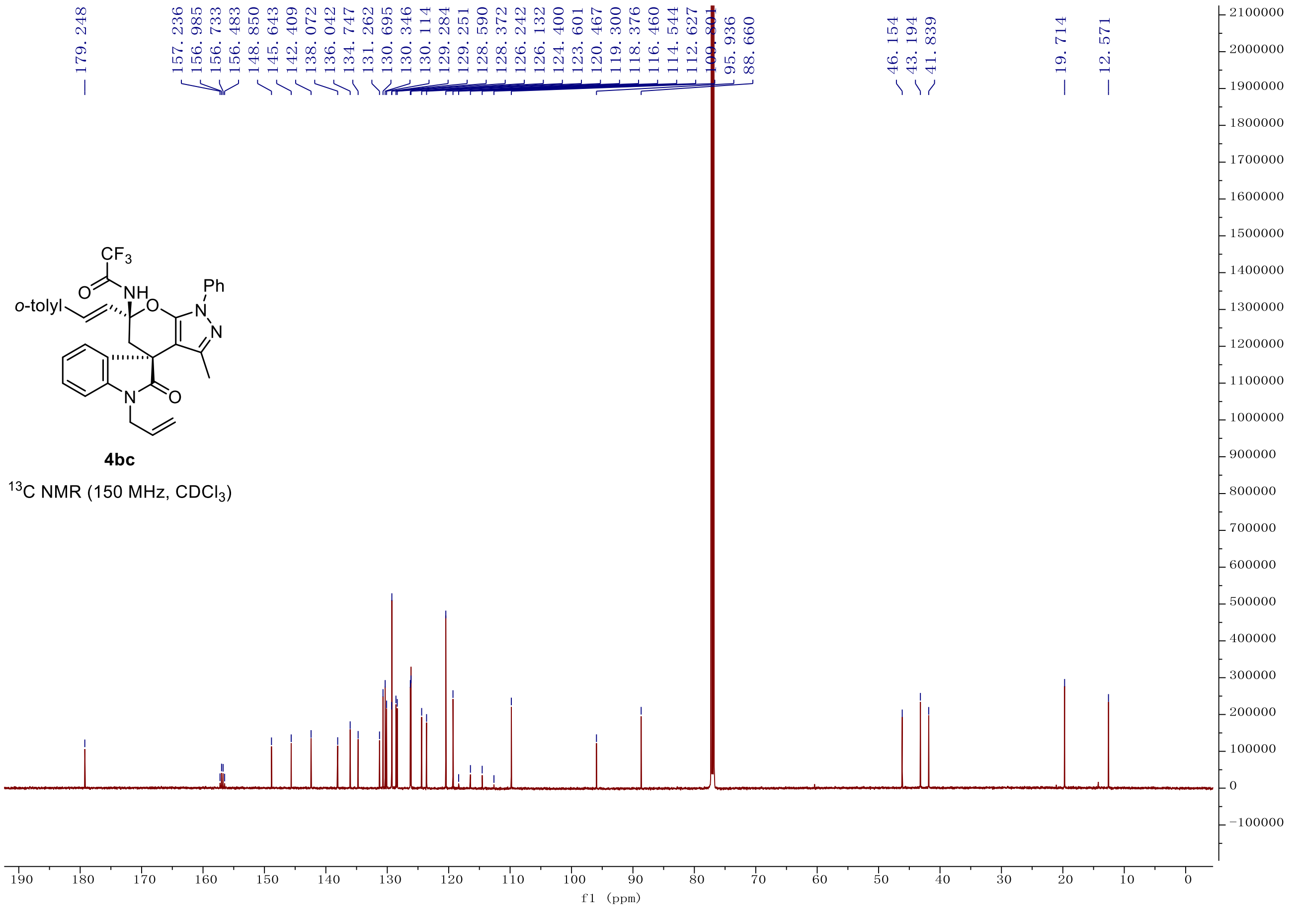
179.248 157.236 156.985 156.733 156.483 148.850 145.643 142.409 138.072 136.042 134.747 131.262 130.695 130.346 130.114 129.284 129.251 128.590 128.372 126.242 126.132 124.400 123.601 120.467 119.300 118.376 116.460 114.544 112.627 109.8[illegible] 95.936 88.660 46.154 43.194 41.839 19.714 12.571
CF3
NH
Ph
o-tolyl
4bc
13C NMR (150 MHz, CDCl3)
f1 (ppm)

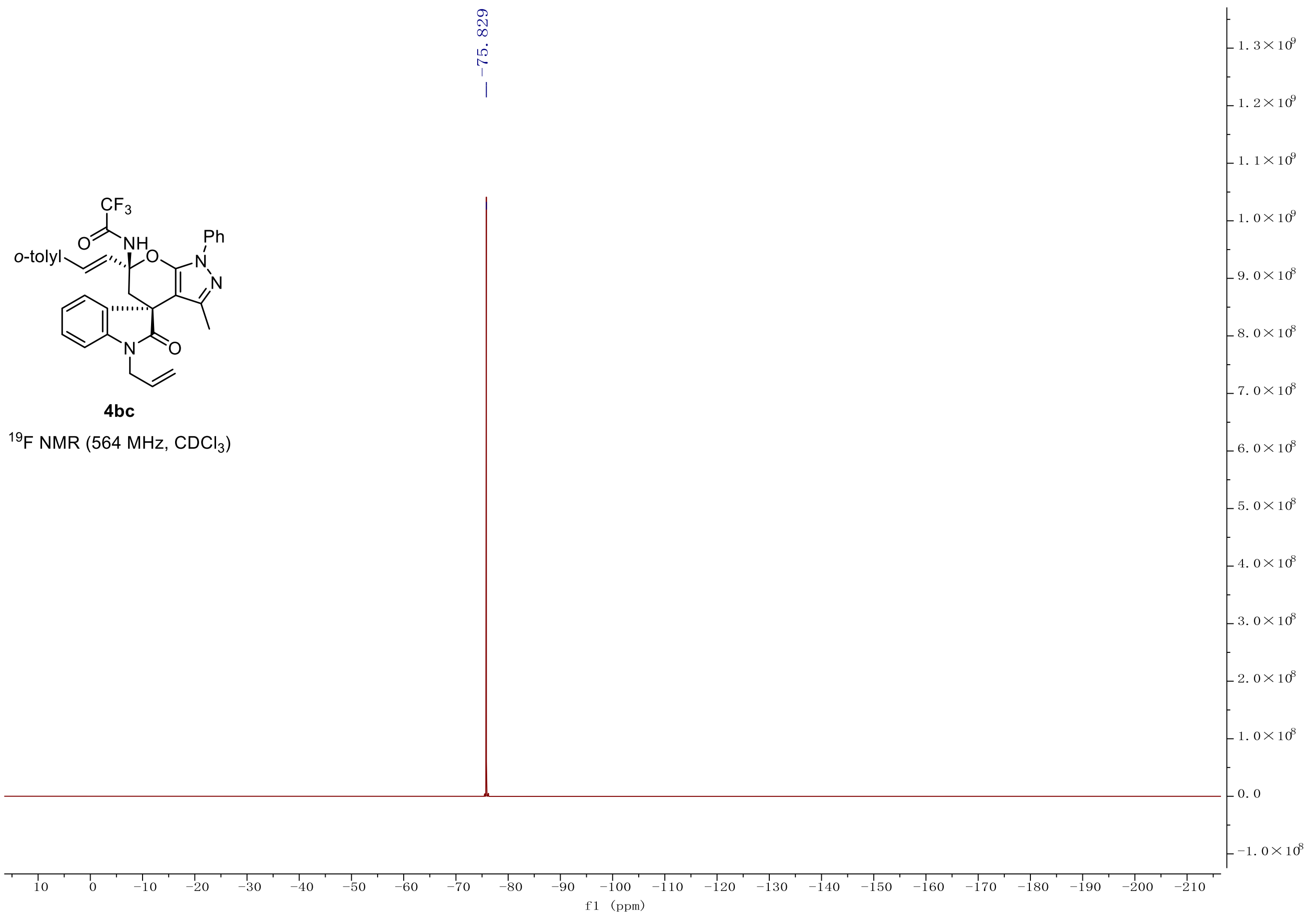

4bc

^{19}F NMR (564 MHz, $CDCl_3$)

Peak Analysis Report

Detector A Channel 1 254nm

No.	Ret. Time	Height (mAu)	Area (mAu*min)	Rel. Area (%)
1	4.672	1119733	9469398	50.226
2	5.070	961762	9384314	49.774
Total		2081495	18853711	100.000

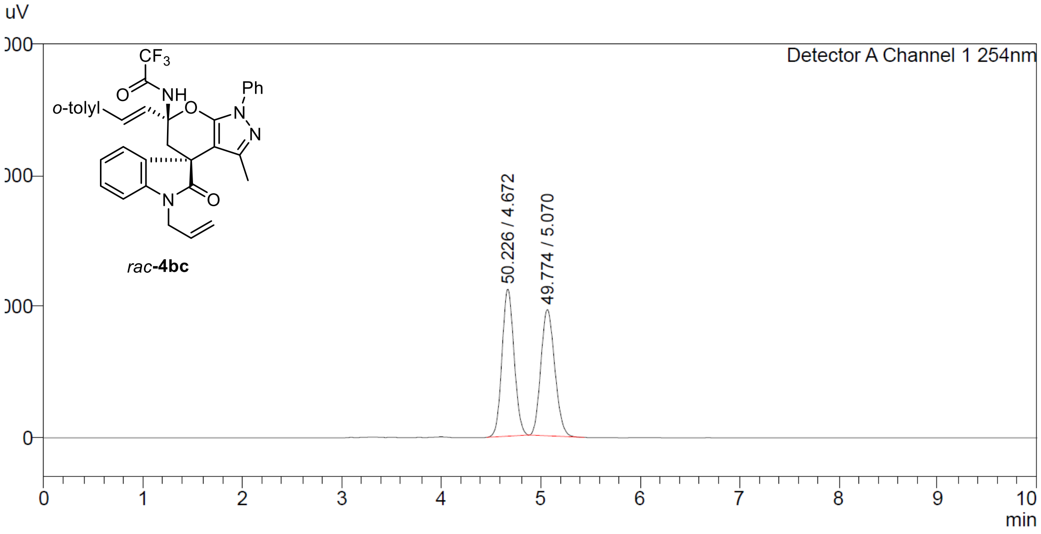

Peak Analysis Report

Detector A Channel 1 254nm

No.	Ret. Time	Height (mAu)	Area (mAu*min)	Rel. Area (%)
1	4.694	1223823	9700358	99.164
2	5.103	10352	81818	0.836
Total		1234175	9782177	100.000

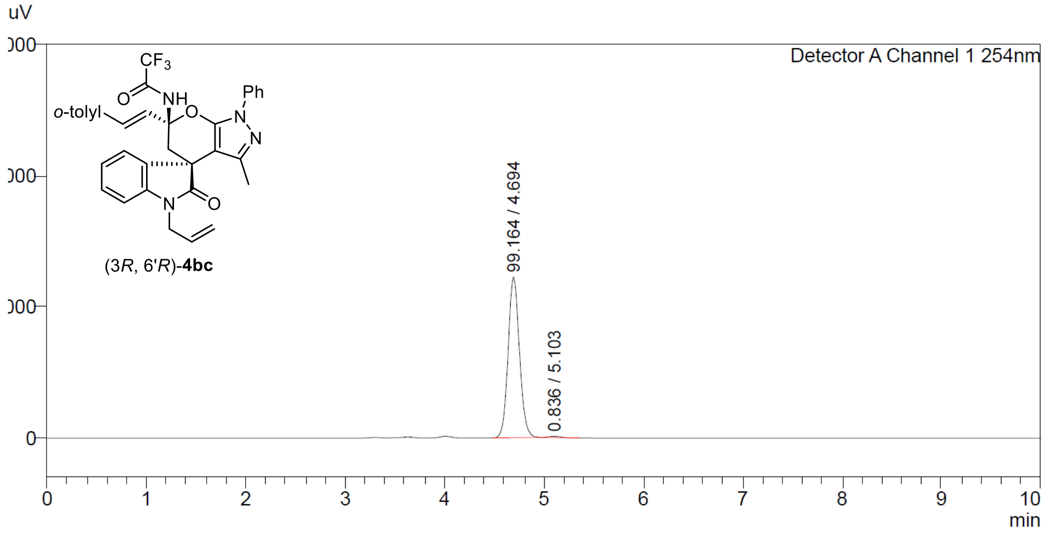

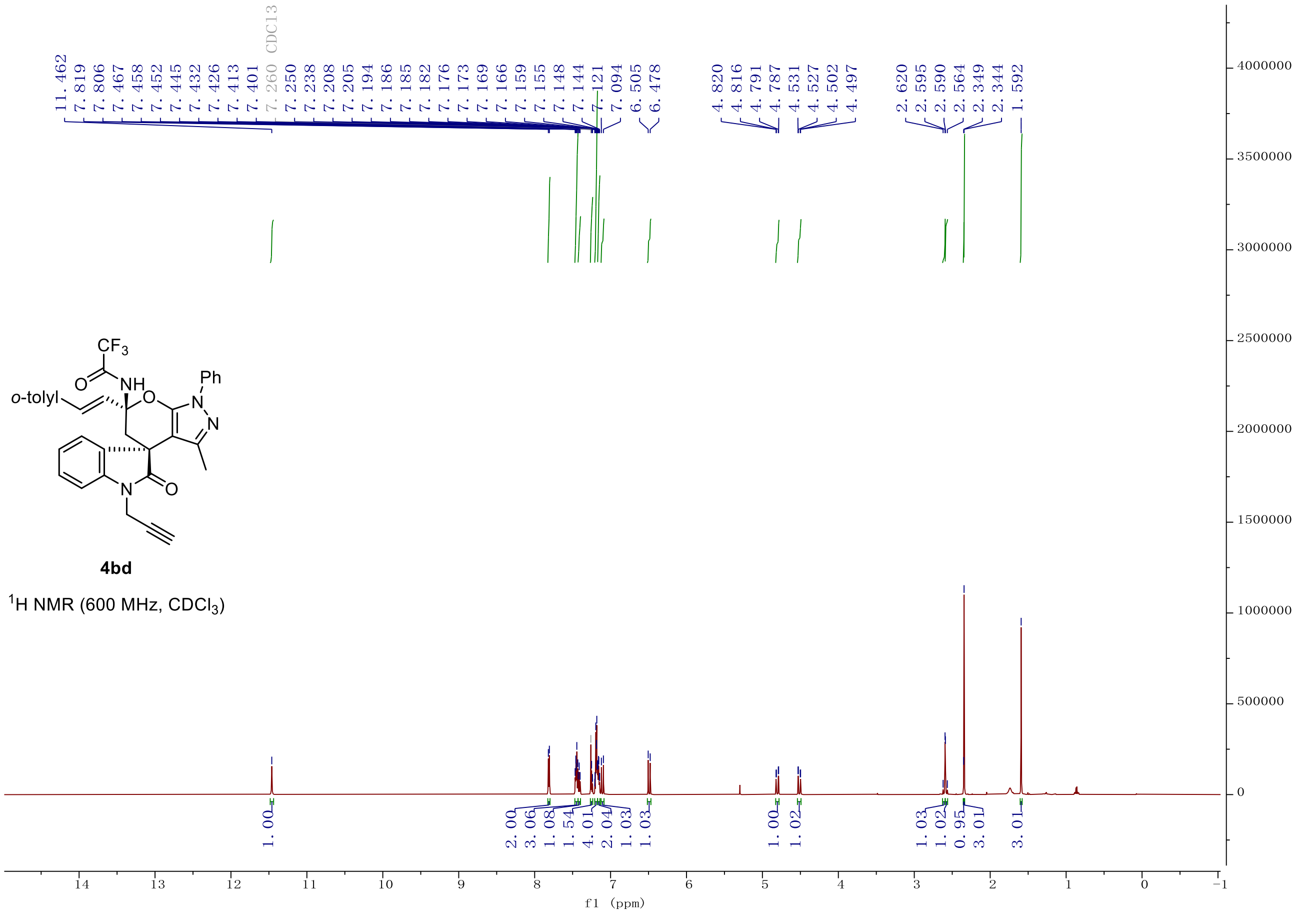

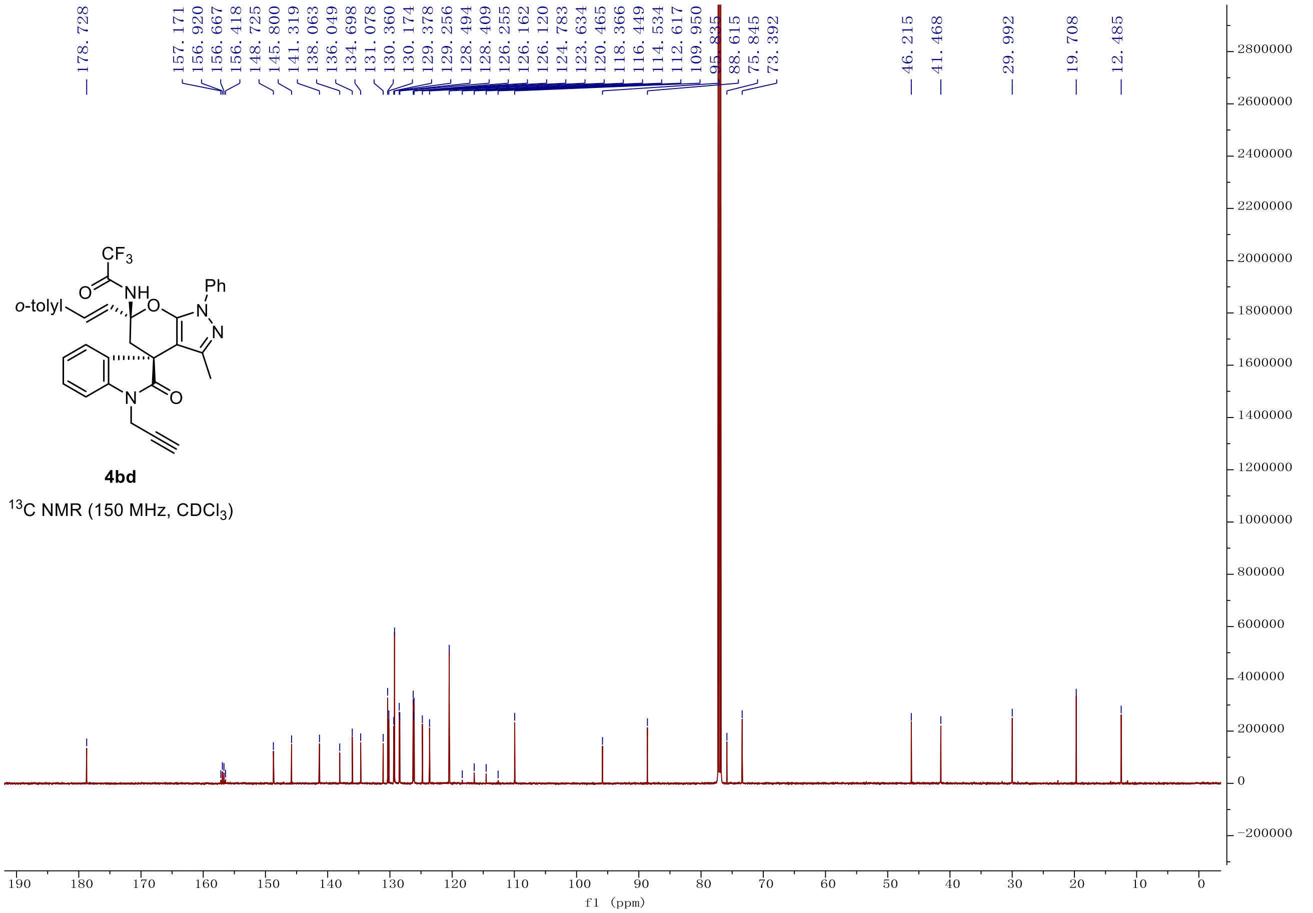
178.728
157.171
156.920
156.667
156.418
148.725
145.800
141.319
138.063
136.049
134.698
131.078
130.360
130.174
129.378
129.256
128.494
128.409
126.255
126.162
126.120
124.783
123.634
120.465
118.366
116.449
114.534
112.617
109.950
95.835
88.615
75.845
73.392
46.215
41.468
29.992
19.708
12.485
CF3
O
NH
O
Ph
N
N
o-tolyl
N
O
4bd
13C NMR (150 MHz, CDCl3)
f1 (ppm)

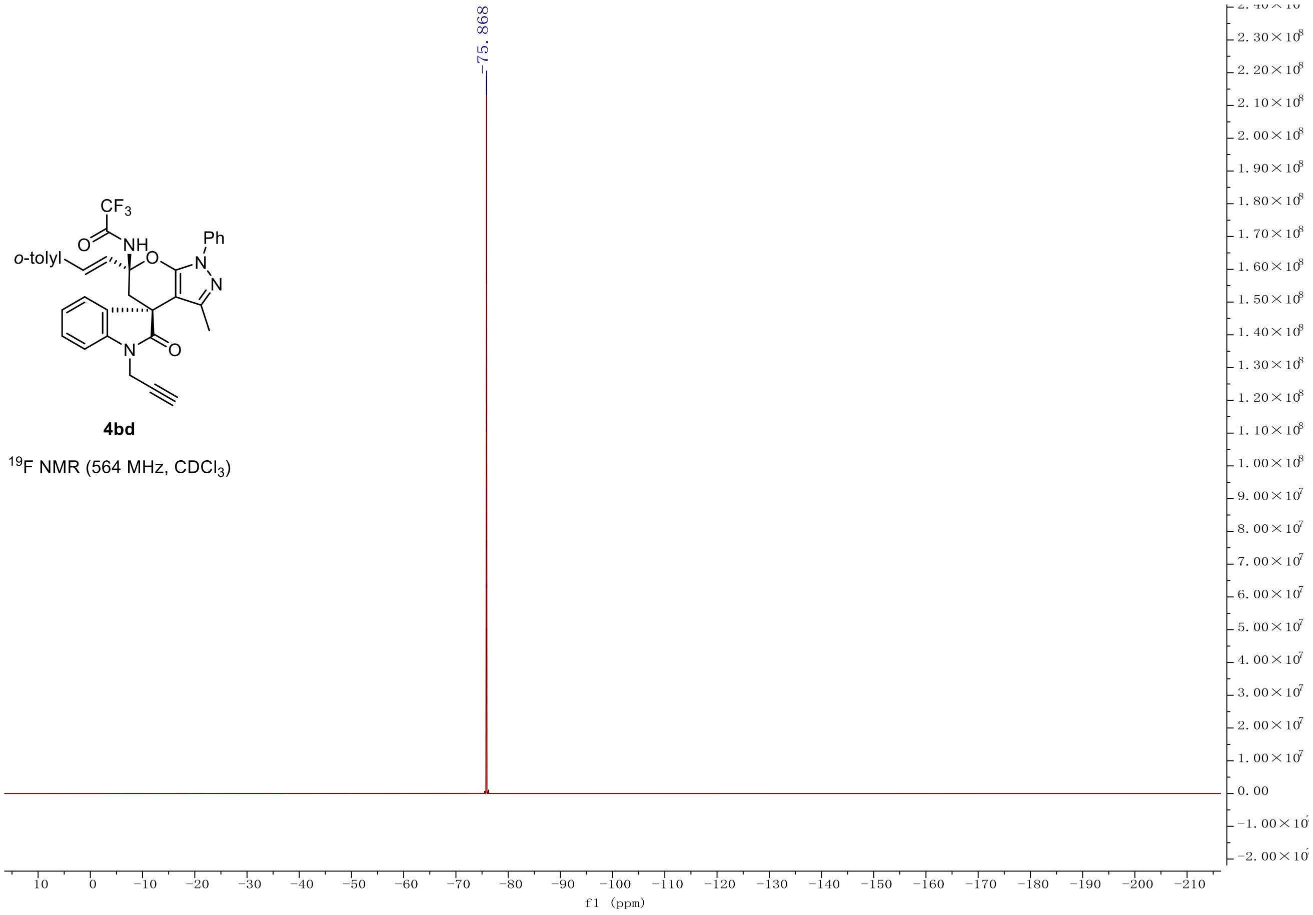
-75.868
CF3
O
NH
O
Ph
N
N
o-tolyl
N
O
4bd
19F NMR (564 MHz, CDCl3)
f1 (ppm)

Signal: VWD1 B, Wavelength=254 nm

RT [min]Type	Area	Width[min]	Area%
7.580	5783.6167	0.277	50.2259
10.405	5731.5879	0.407	49.7741
总和	11515.2046		

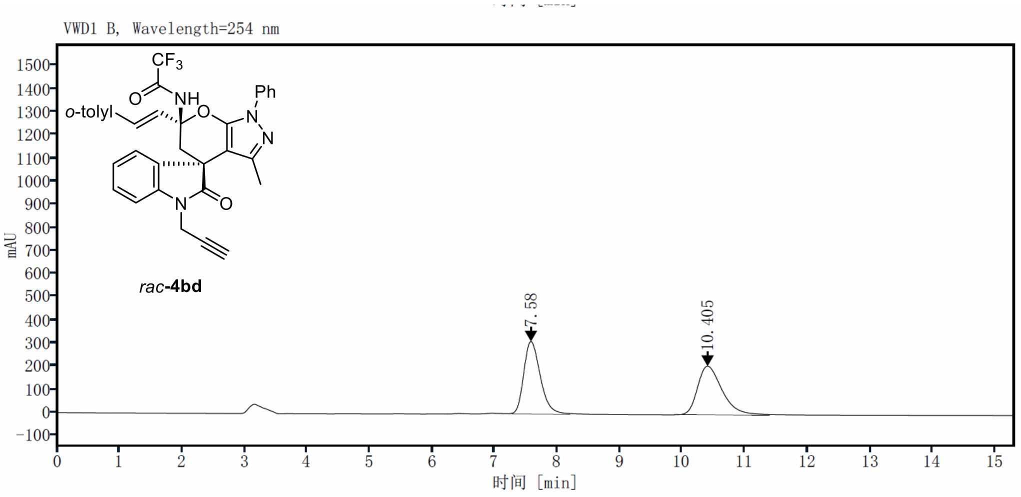

Signal: VWD1 B, Wavelength=254 nm

RT [min]Type	Area	Width[min]	Area%
7.563	450.5632	0.484	0.7894
10.138	56627.1094	0.449	99.2106
总和	57077.6726		

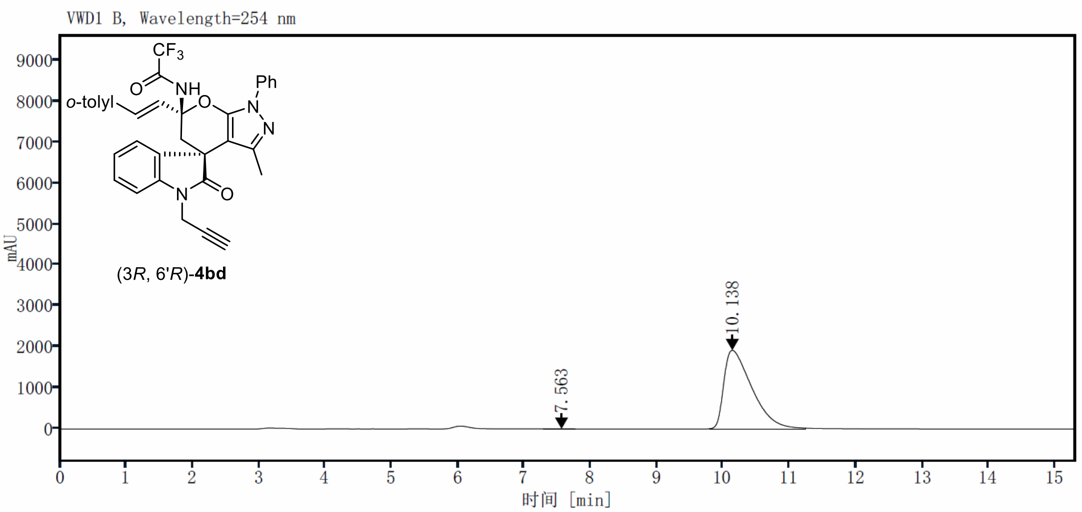

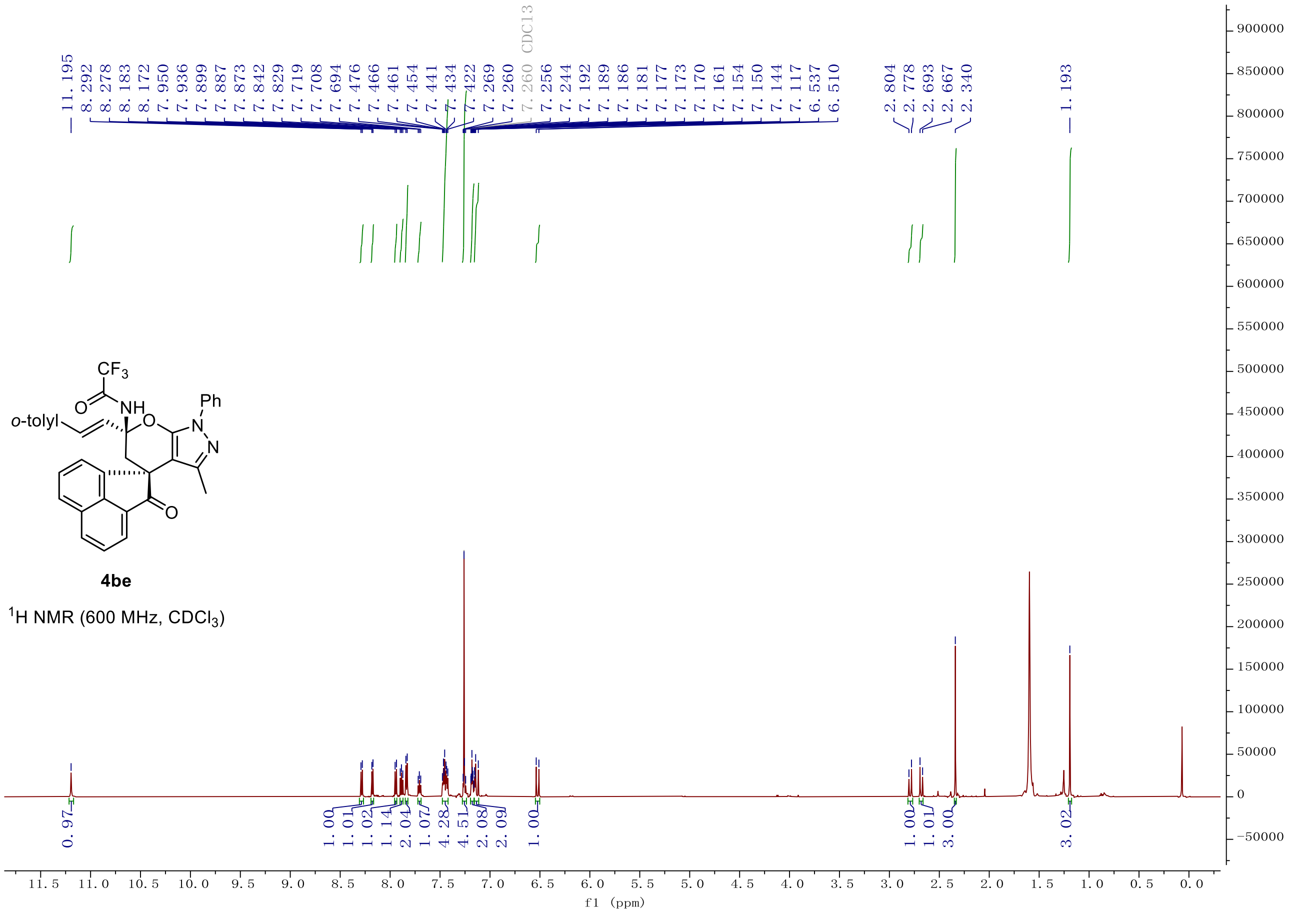
11.195
8.292
8.278
8.183
8.172
7.950
7.936
7.899
7.887
7.873
7.842
7.829
7.719
7.708
7.694
7.476
7.466
7.461
7.454
7.441
7.434
7.422
7.269
7.260
7.260 CDCl3
7.256
7.244
7.192
7.189
7.186
7.181
7.177
7.173
7.170
7.161
7.154
7.150
7.144
7.117
6.537
6.510
2.804
2.778
2.693
2.667
2.340
1.193
CF3
O
NH
Ph
o-tolyl
O
N
N
O
4be
1H NMR (600 MHz, CDCl3)
0.97
1.00
1.01
1.02
1.14
2.04
1.07
4.28
4.51
2.08
2.09
1.00
1.00
1.01
3.00
3.02
f1 (ppm)

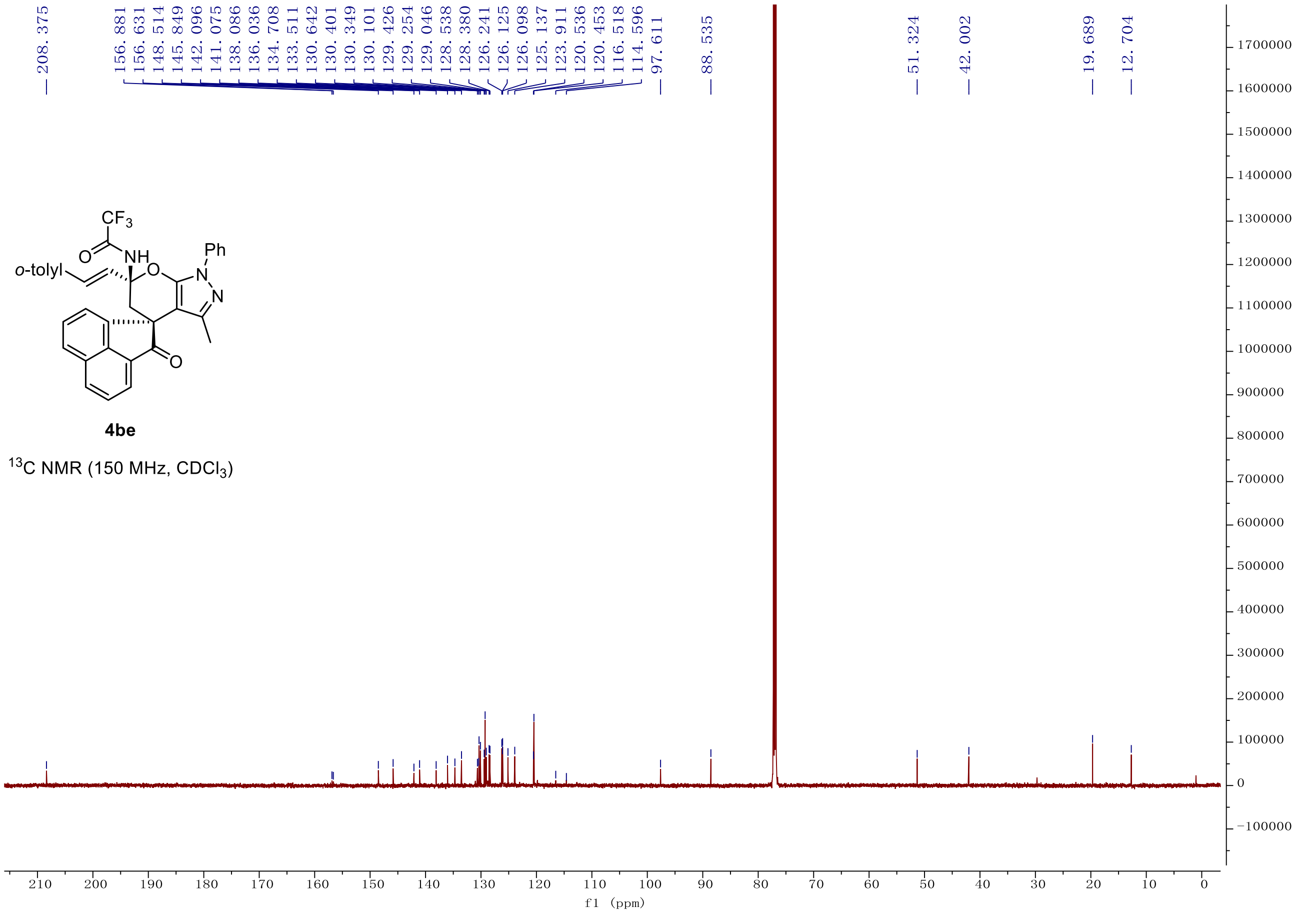
208.375
156.881
156.631
148.514
145.849
142.096
141.075
138.086
136.036
134.708
133.511
130.642
130.401
130.349
130.101
129.426
129.254
129.046
128.538
128.380
126.241
126.125
126.098
125.137
123.911
120.536
120.453
116.518
114.596
97.611
88.535
51.324
42.002
19.689
12.704
CF3
O
NH
Ph
o-tolyl
O
N
N
O
4be
13C NMR (150 MHz, CDCl3)
f1 (ppm)

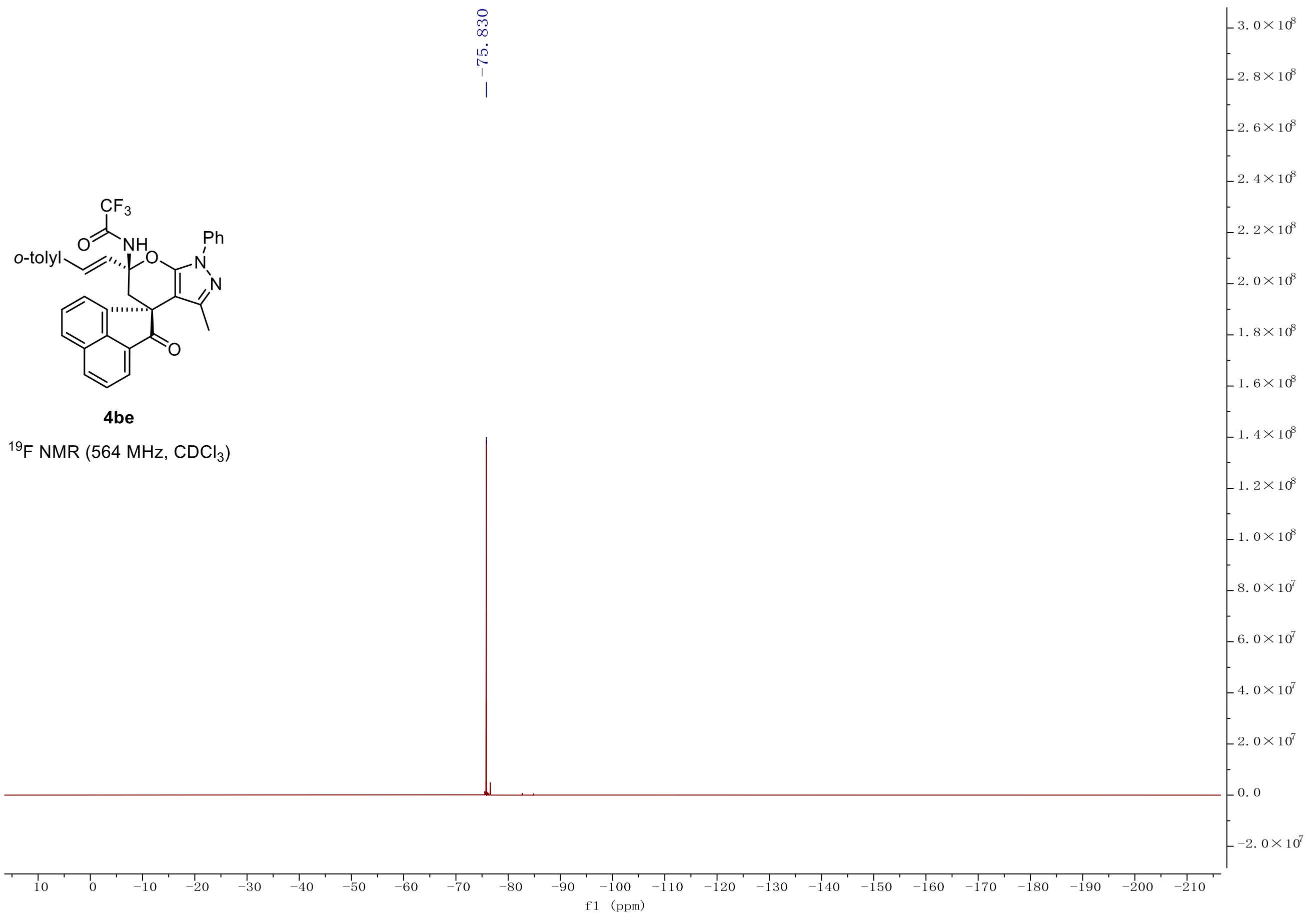

Signal: VWD1 B, Wavelength=254 nm

RT [min]Type	Area	Width[min]	Area%
5.381	18252.9375	0.192	50.4048
7.200	17959.7422	0.282	49.5952
总和	36212.6797		

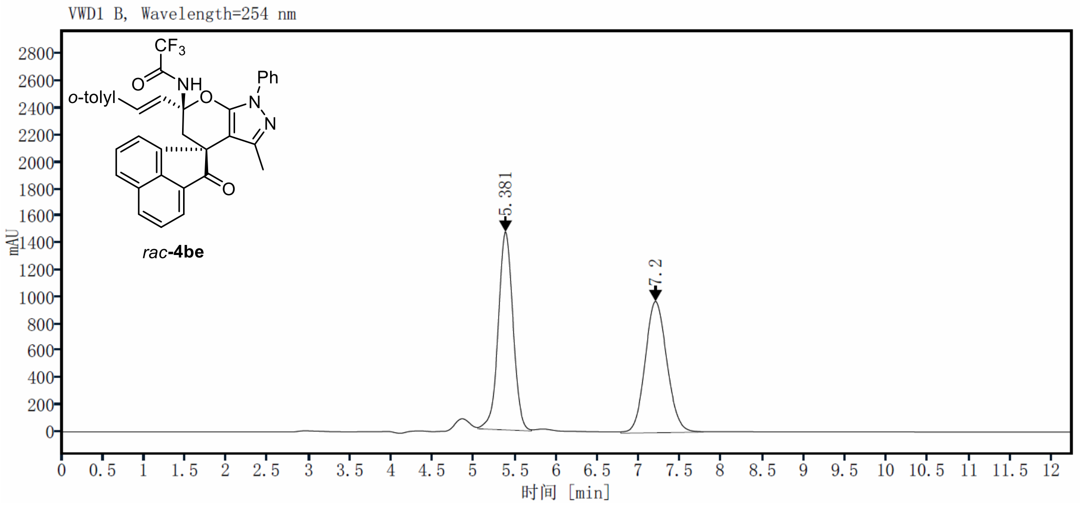

Signal: VWD1 B, Wavelength=254 nm

RT [min]Type	Area	Width[min]	Area%
5.360	7496.0410	0.187	96.1668
7.158	298.7884	0.549	3.8332
总和	7794.8294		

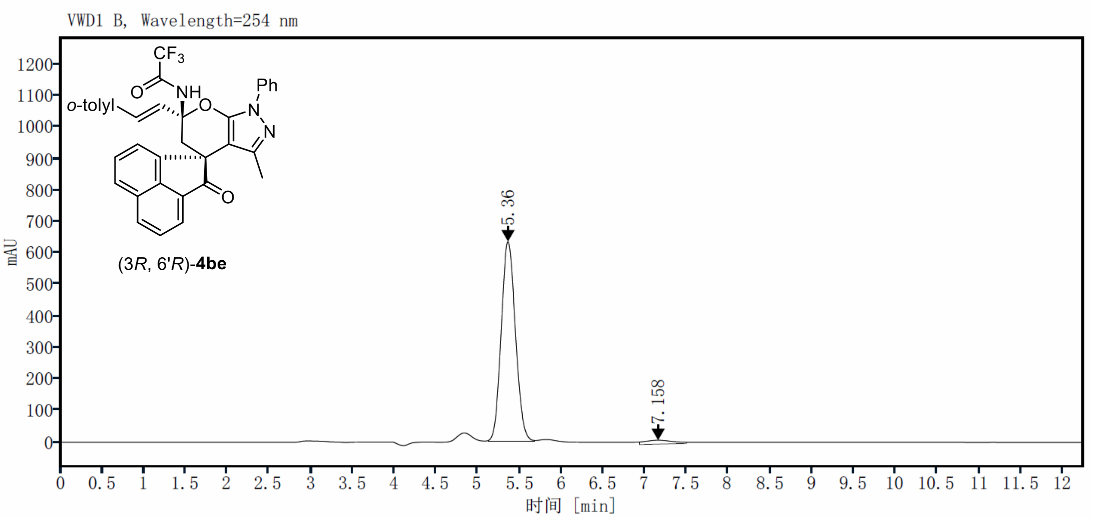

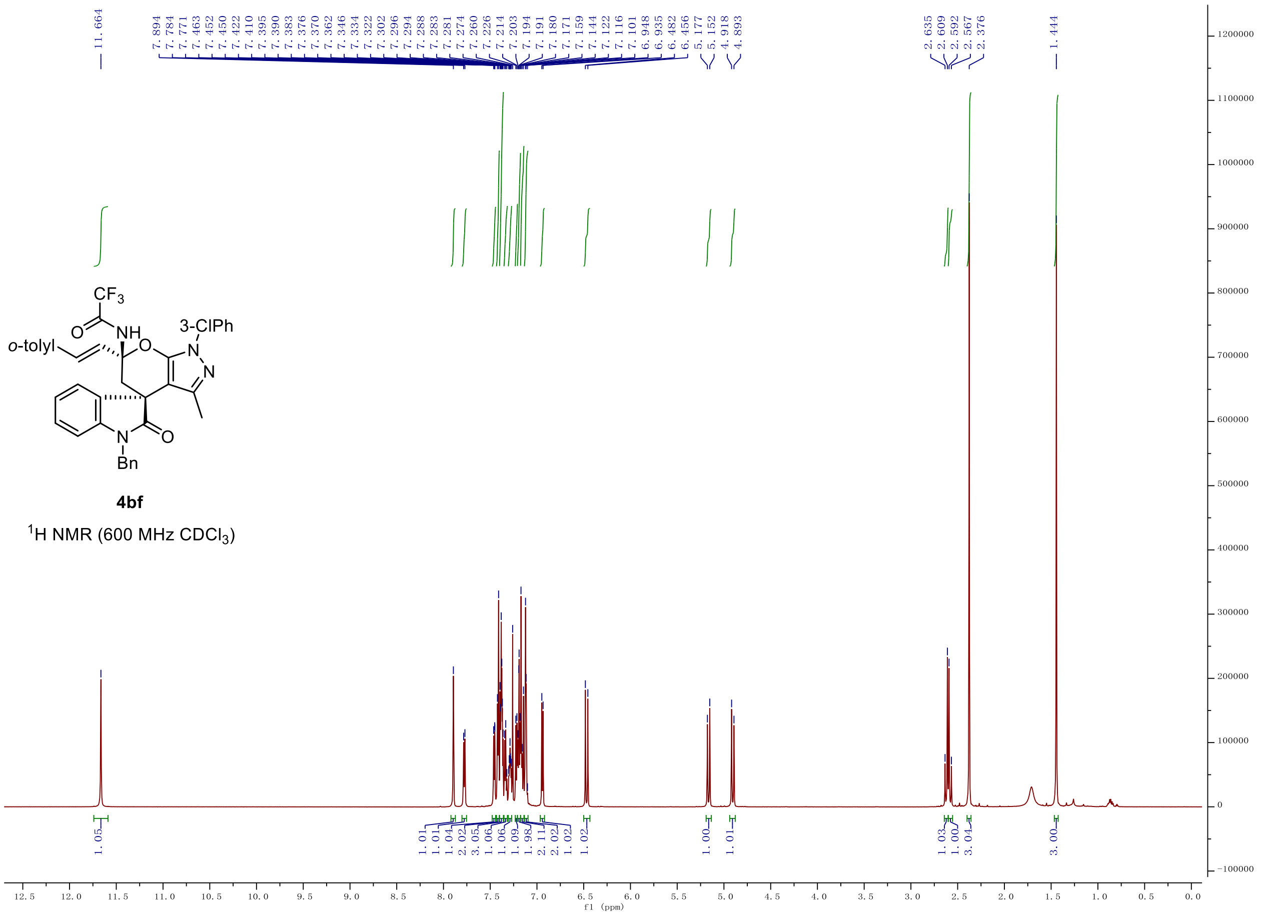

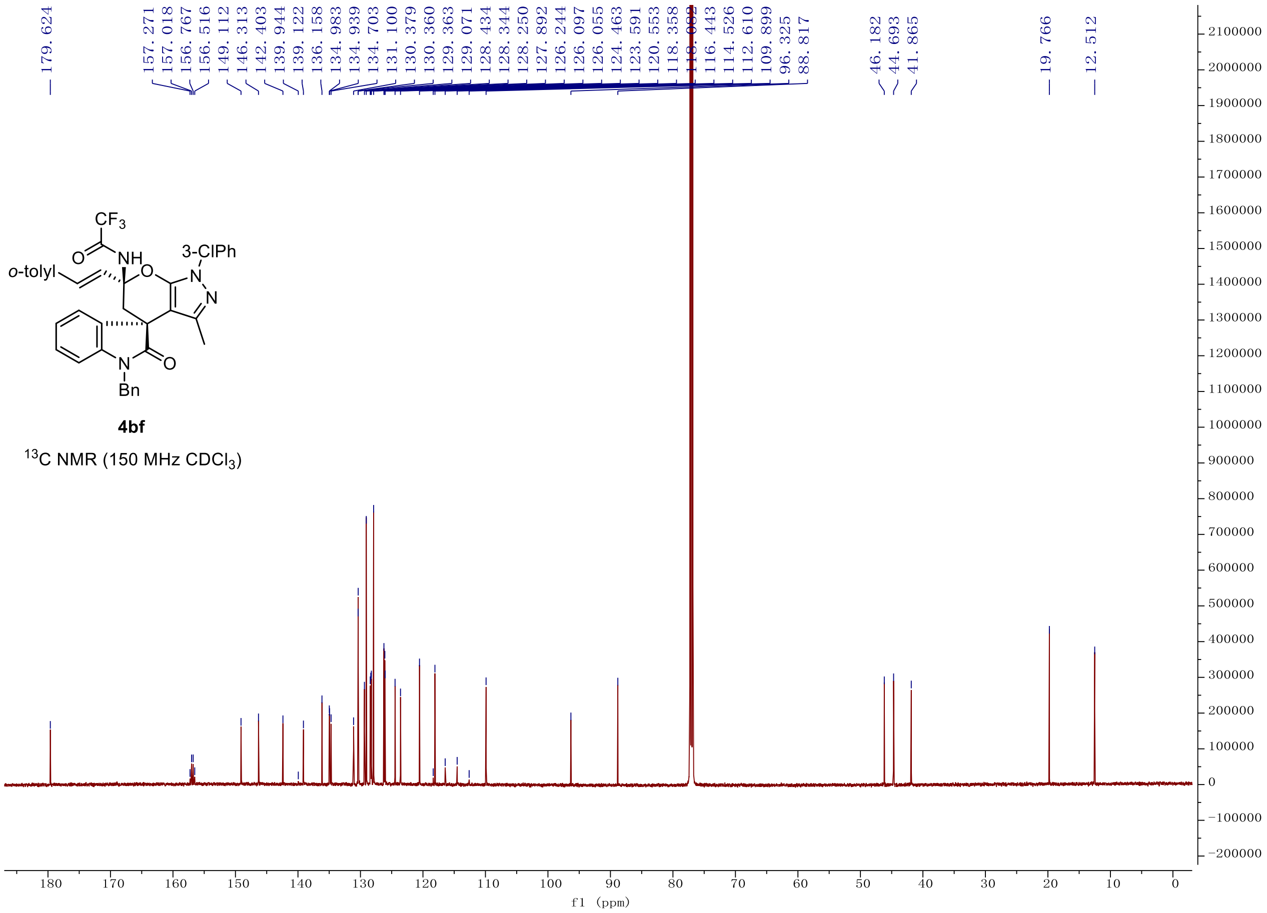
179.624
157.271
157.018
156.767
156.516
149.112
146.313
142.403
139.944
139.122
136.158
134.983
134.939
134.703
131.100
130.379
130.360
129.363
129.071
128.434
128.344
128.250
127.892
126.244
126.097
126.055
124.463
123.591
120.553
118.358
[illegible]
116.443
114.526
112.610
109.899
96.325
88.817
46.182
44.693
41.865
19.766
12.512
CF3
NH
O
o-tolyl
3-ClPh
N
N
O
N
Bn
4bf
13C NMR (150 MHz CDCl3)
f1 (ppm)

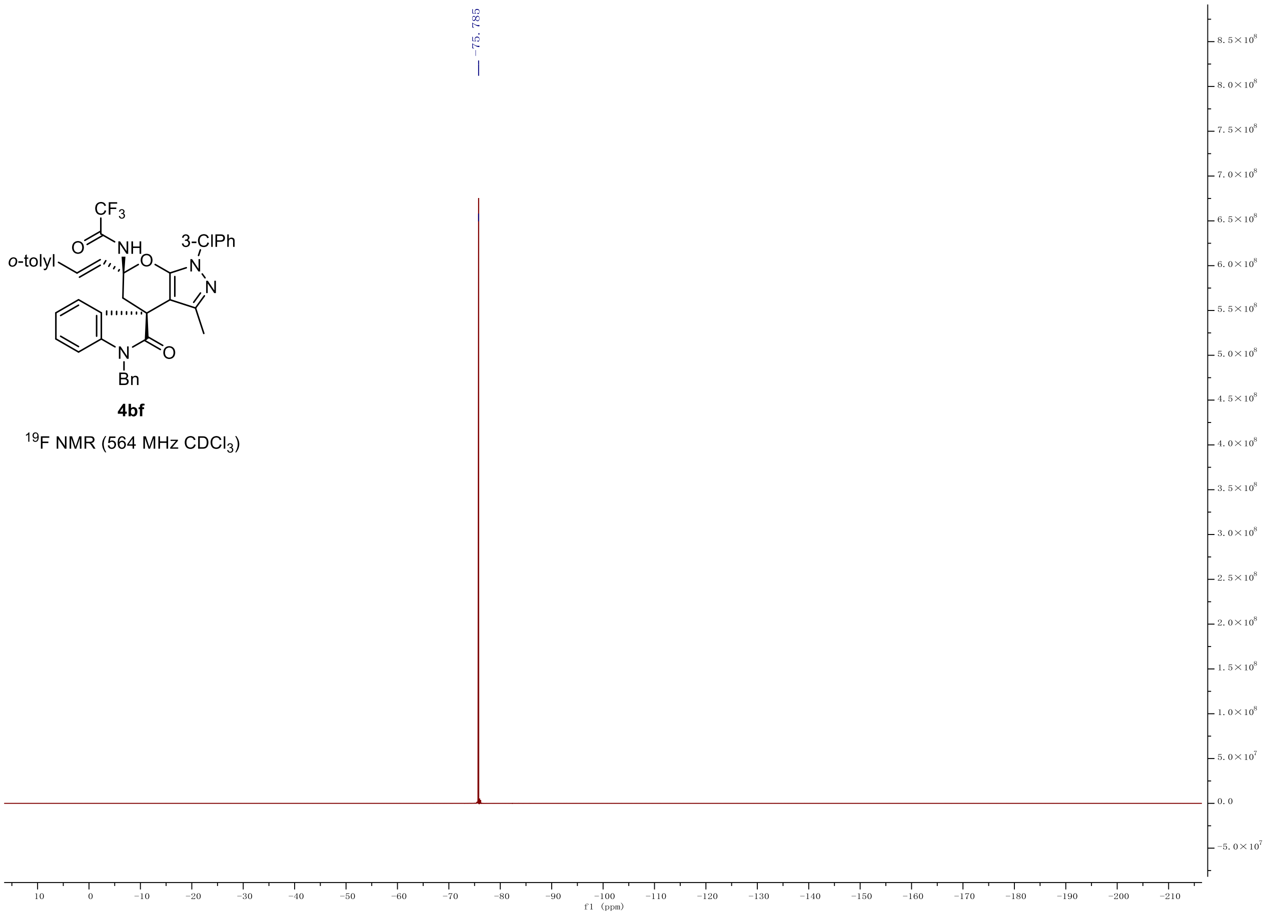
-75.785
CF3
NH
O
o-tolyl
3-ClPh
N
N
O
N
Bn
4bf
19F NMR (564 MHz CDCl3)
f1 (ppm)

Peak Analysis Report

Detector A Channel 1 254nm

No.	Ret. Time	Height (mAu)	Area (mAu*min)	Rel. Area (%)
1	6.522	686291	12754042	50.694
2	7.586	526732	12405006	49.306
Total		1213023	25159049	100.000

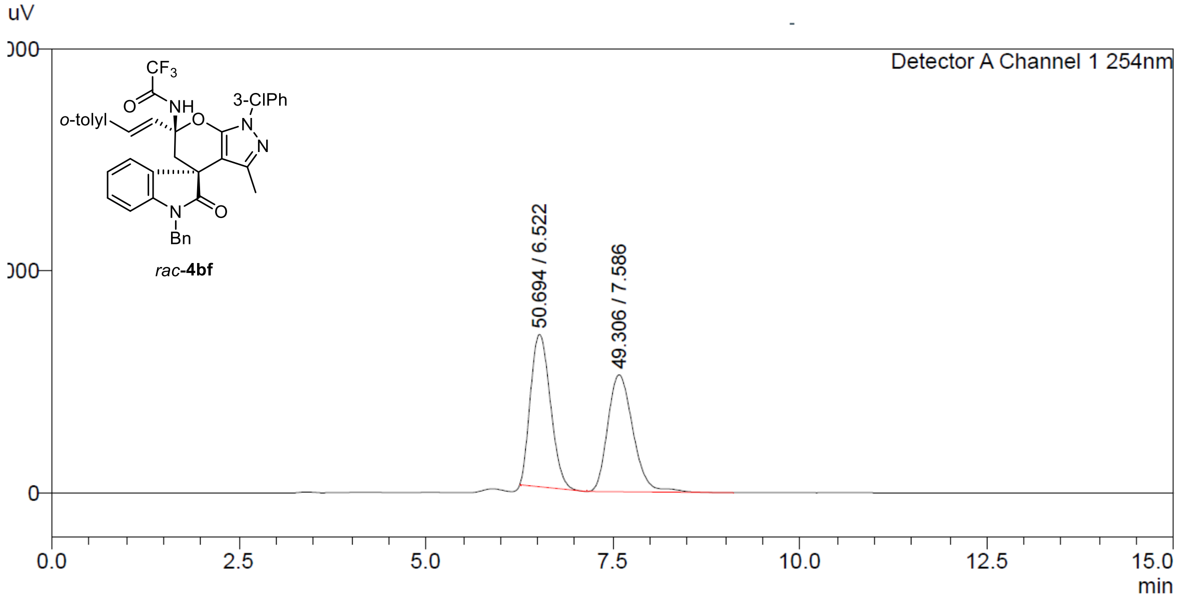

Peak Analysis Report

Detector A Channel 1 254nm

No.	Ret. Time	Height (mAu)	Area (mAu*min)	Rel. Area (%)
1	6.523	2484387	48562632	99.203
2	7.581	18157	390190	0.797
Total		2502543	48952822	100.000

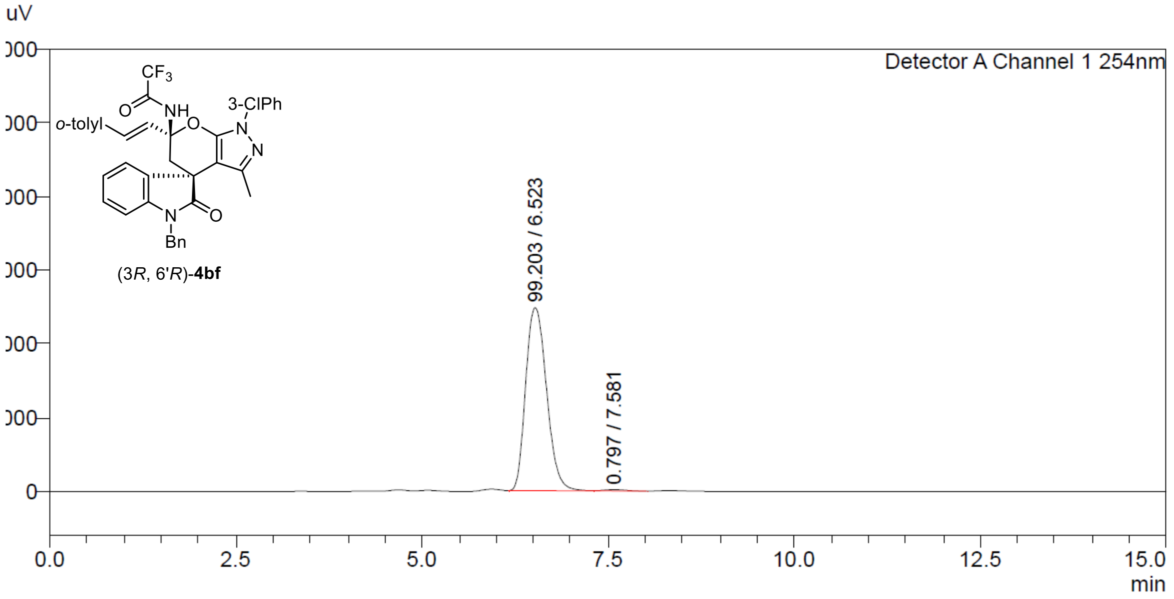

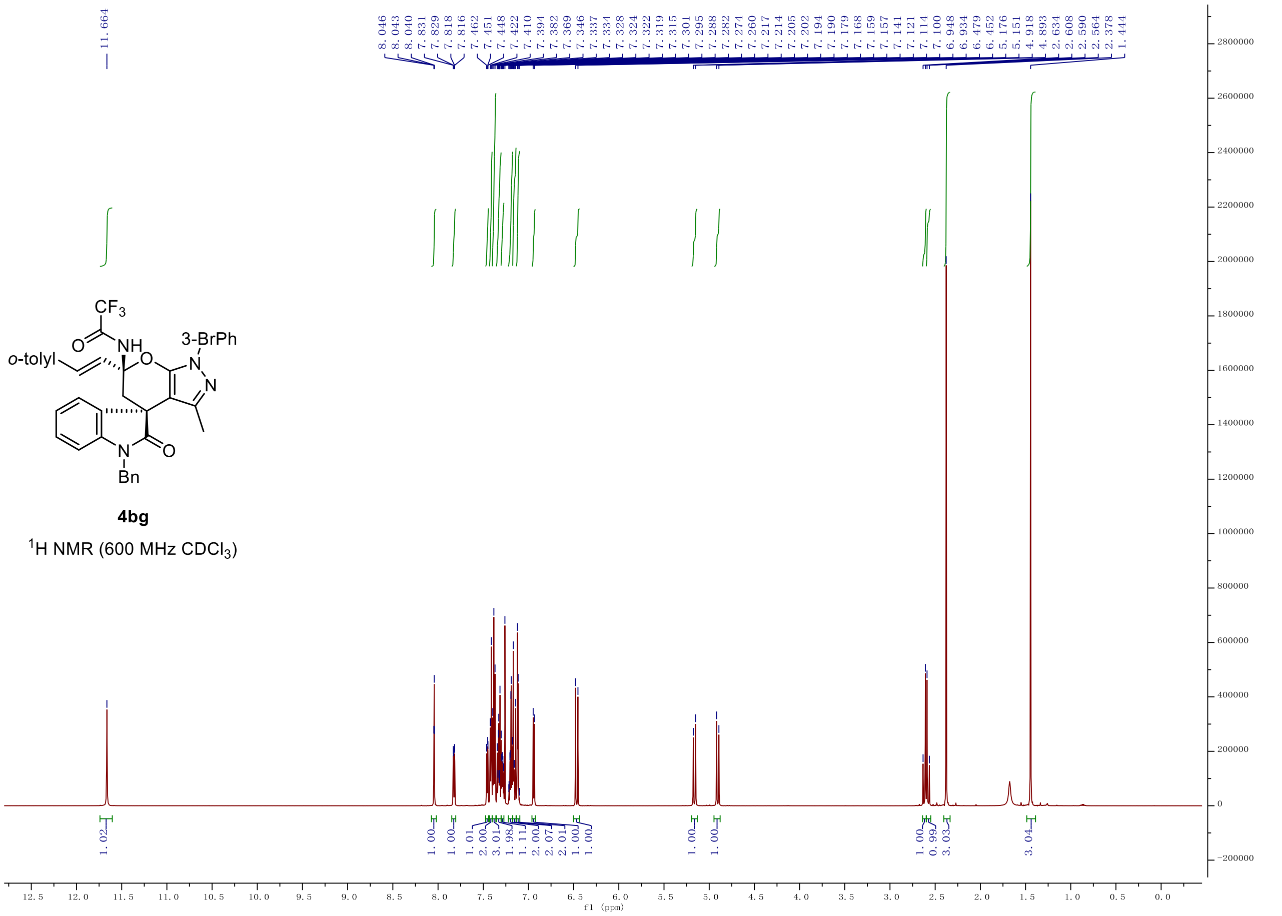
11.664
8.046
8.043
8.040
7.831
7.829
7.818
7.816
7.462
7.451
7.448
7.422
7.410
7.394
7.382
7.369
7.346
7.337
7.334
7.328
7.324
7.322
7.319
7.315
7.301
7.295
7.288
7.282
7.274
7.260
7.217
7.214
7.205
7.202
7.194
7.190
7.179
7.168
7.159
7.157
7.141
7.121
7.114
7.100
6.948
6.934
6.479
6.452
5.176
5.151
4.918
4.893
2.634
2.608
2.590
2.564
2.378
1.444
CF3
NH
O
3-BrPh
o-tolyl
N
N
O
N
Bn
4bg
1H NMR (600 MHz CDCl3)
f1 (ppm)

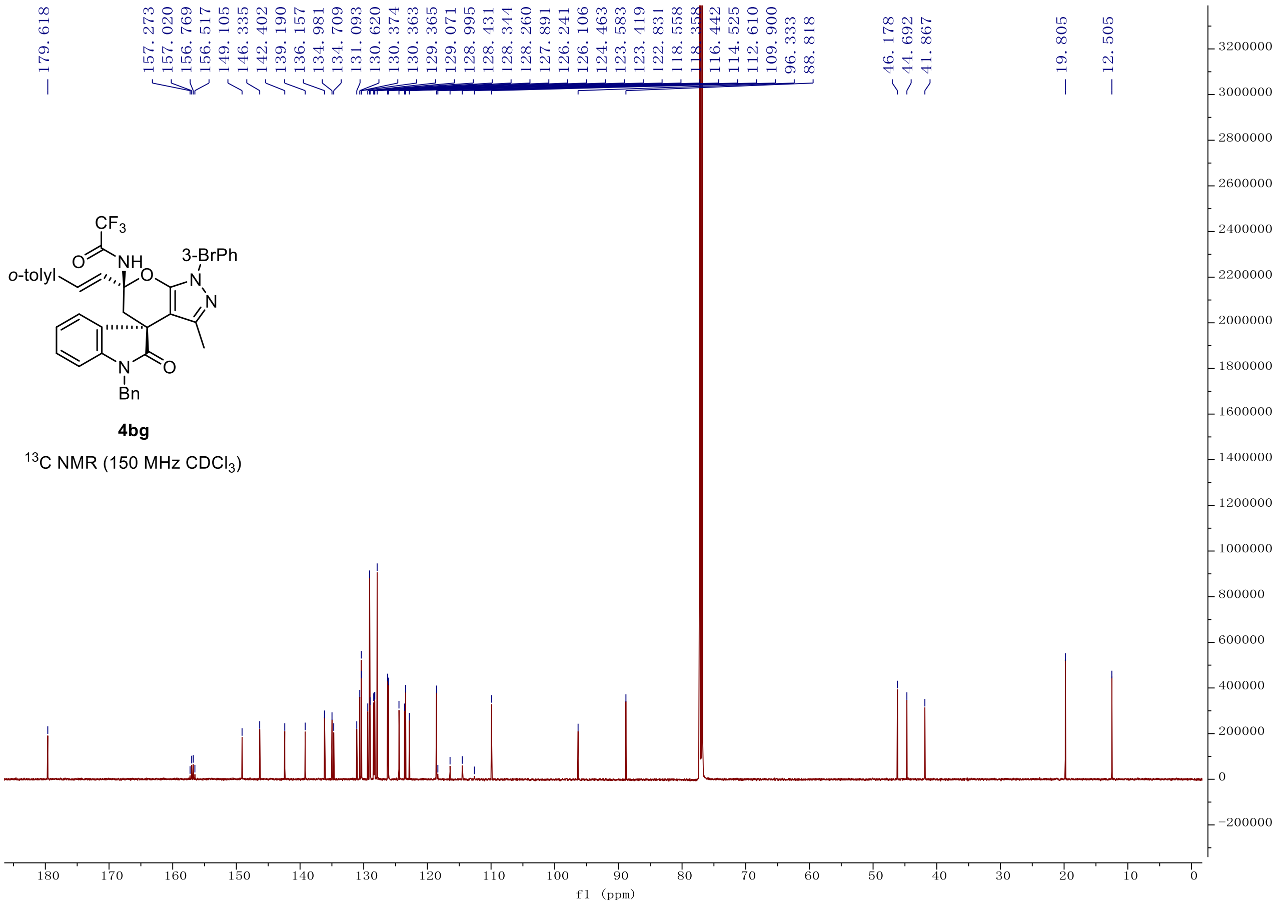
179.618
157.273
157.020
156.769
156.517
149.105
146.335
142.402
139.190
136.157
134.981
134.709
131.093
130.620
130.374
130.363
129.365
129.071
128.995
128.431
128.344
128.260
127.891
126.241
126.106
124.463
123.583
123.419
122.831
118.558
118.358
116.442
114.525
112.610
109.900
96.333
88.818
46.178
44.692
41.867
19.805
12.505
CF3
NH
O
3-BrPh
o-tolyl
N
N
O
N
Bn
4bg
13C NMR (150 MHz CDCl3)
f1 (ppm)

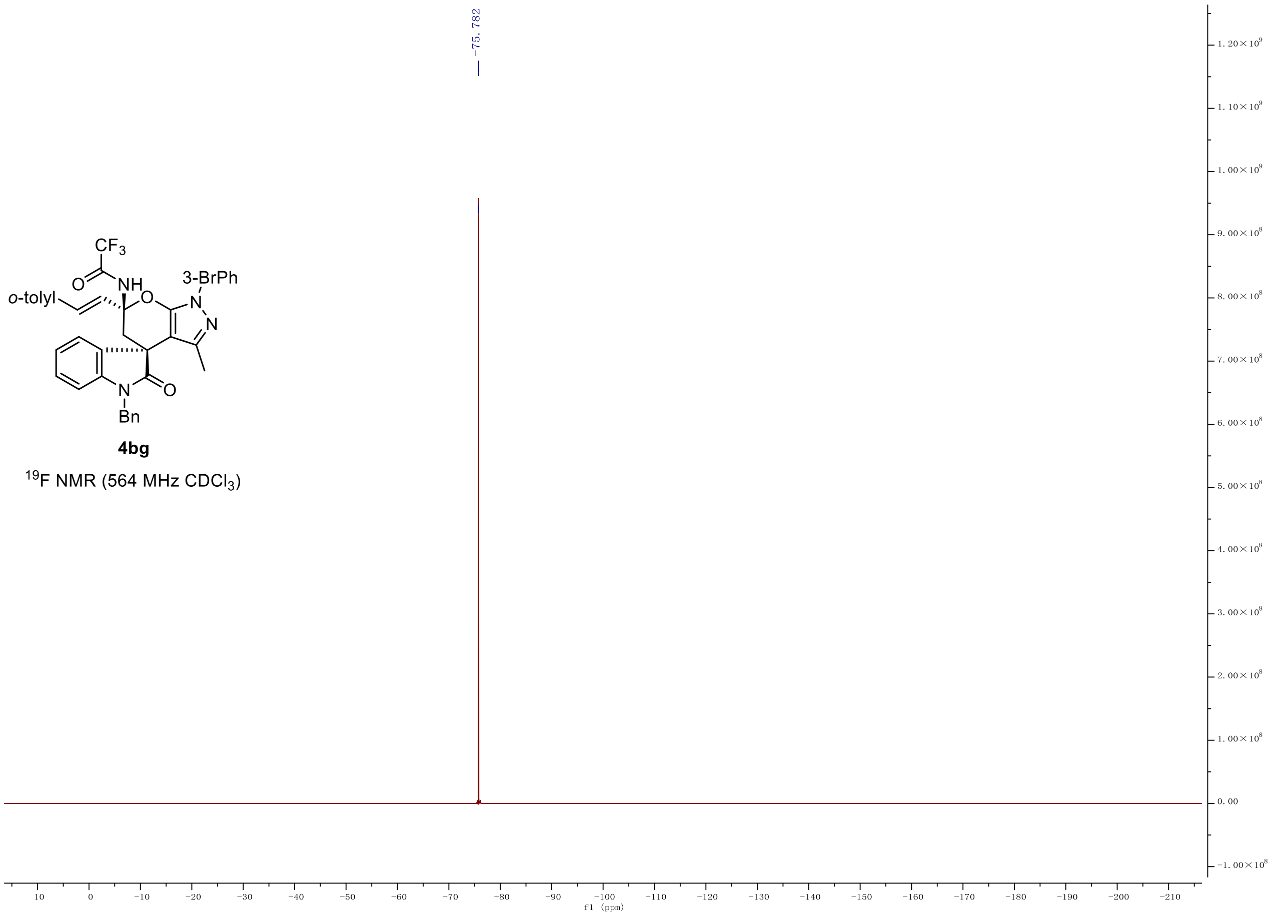

Peak Analysis Report

Detector A Channel 1 254nm

No.	Ret. Time	Height (mAu)	Area (mAu*min)	Rel. Area (%)
1	9.208	1285775	26479384	50.884
2	11.922	654962	25558926	49.116
Total		1940737	52038311	100.000

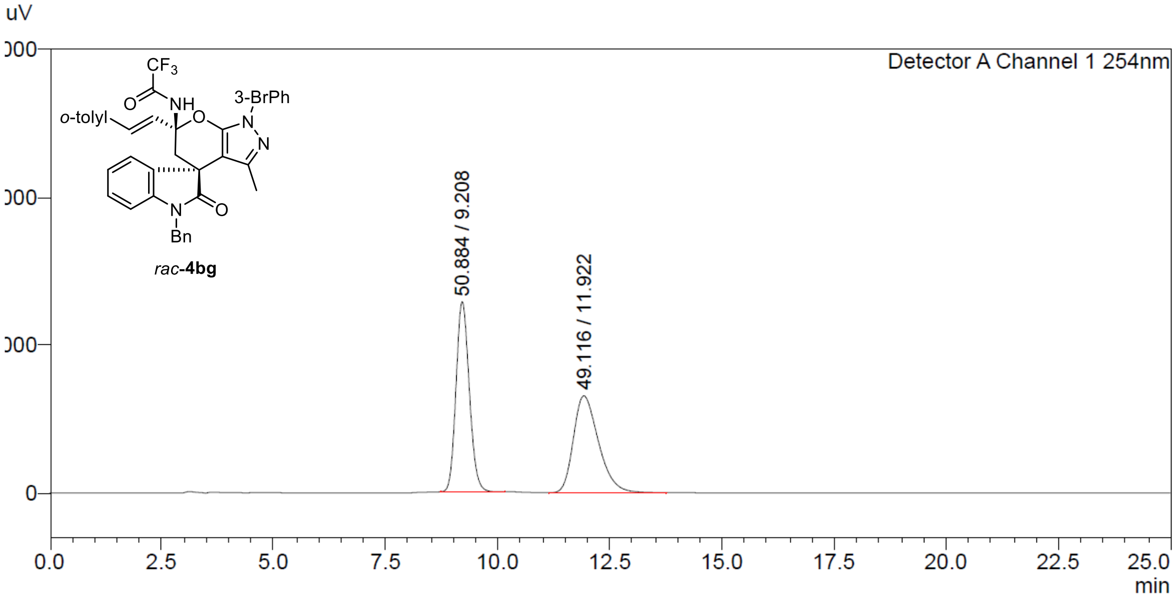

Peak Analysis Report

Detector A Channel 1 254nm

No.	Ret. Time	Height (mAu)	Area (mAu*min)	Rel. Area (%)
1	9.204	788884	15519655	99.609
2	12.018	1686	60876	0.391
Total		790570	15580531	100.000

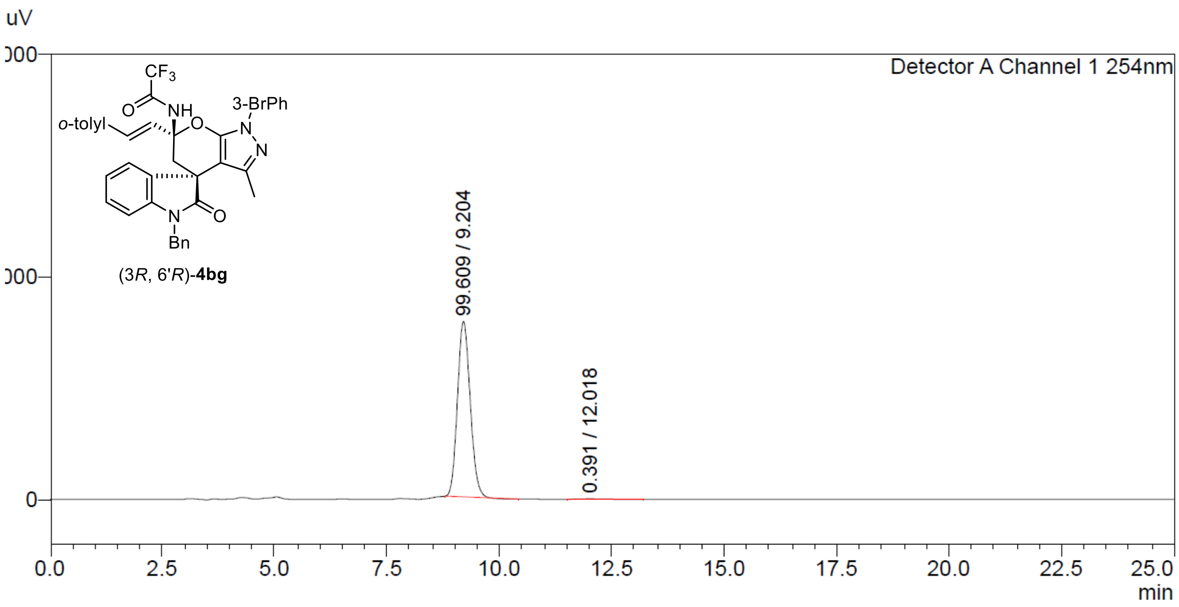

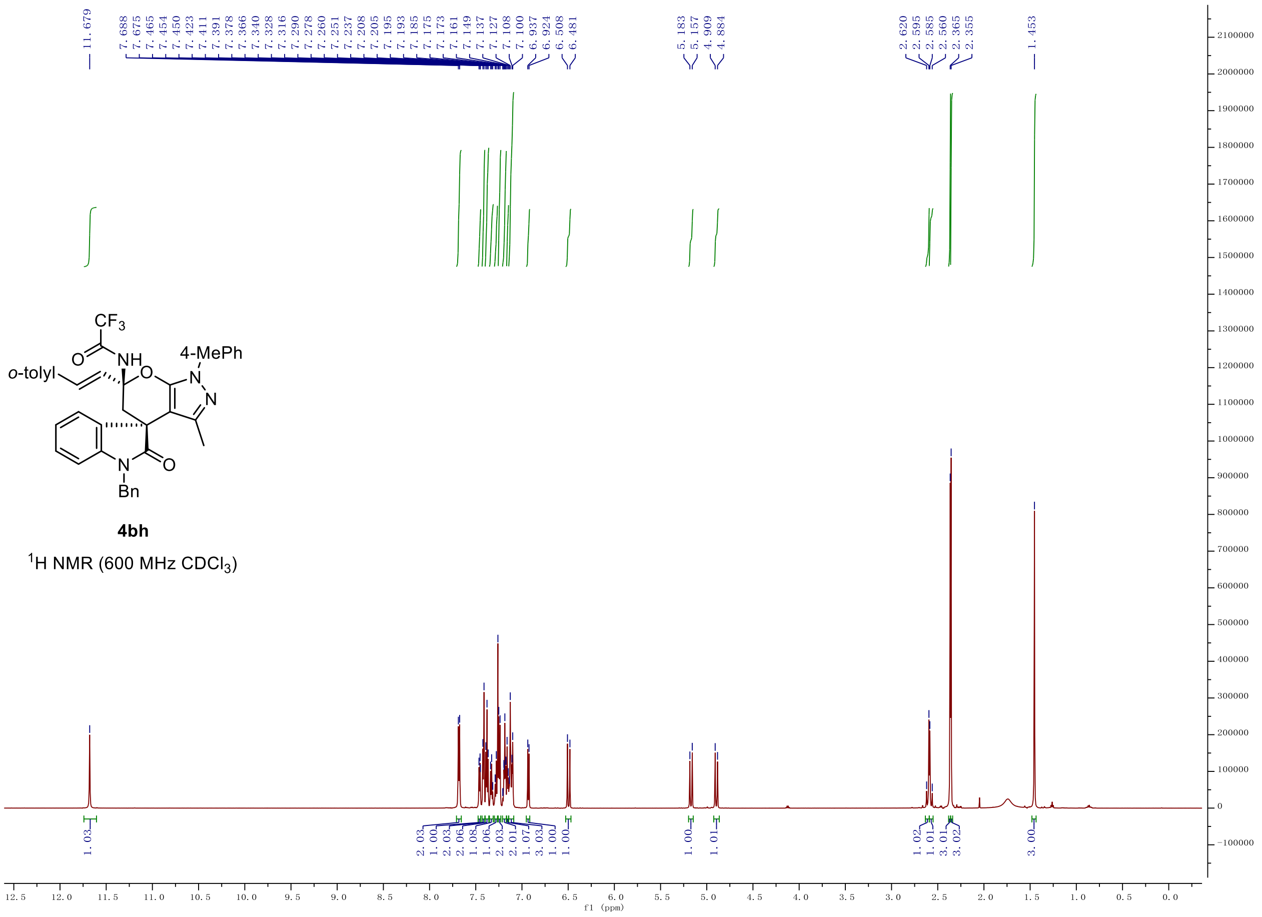

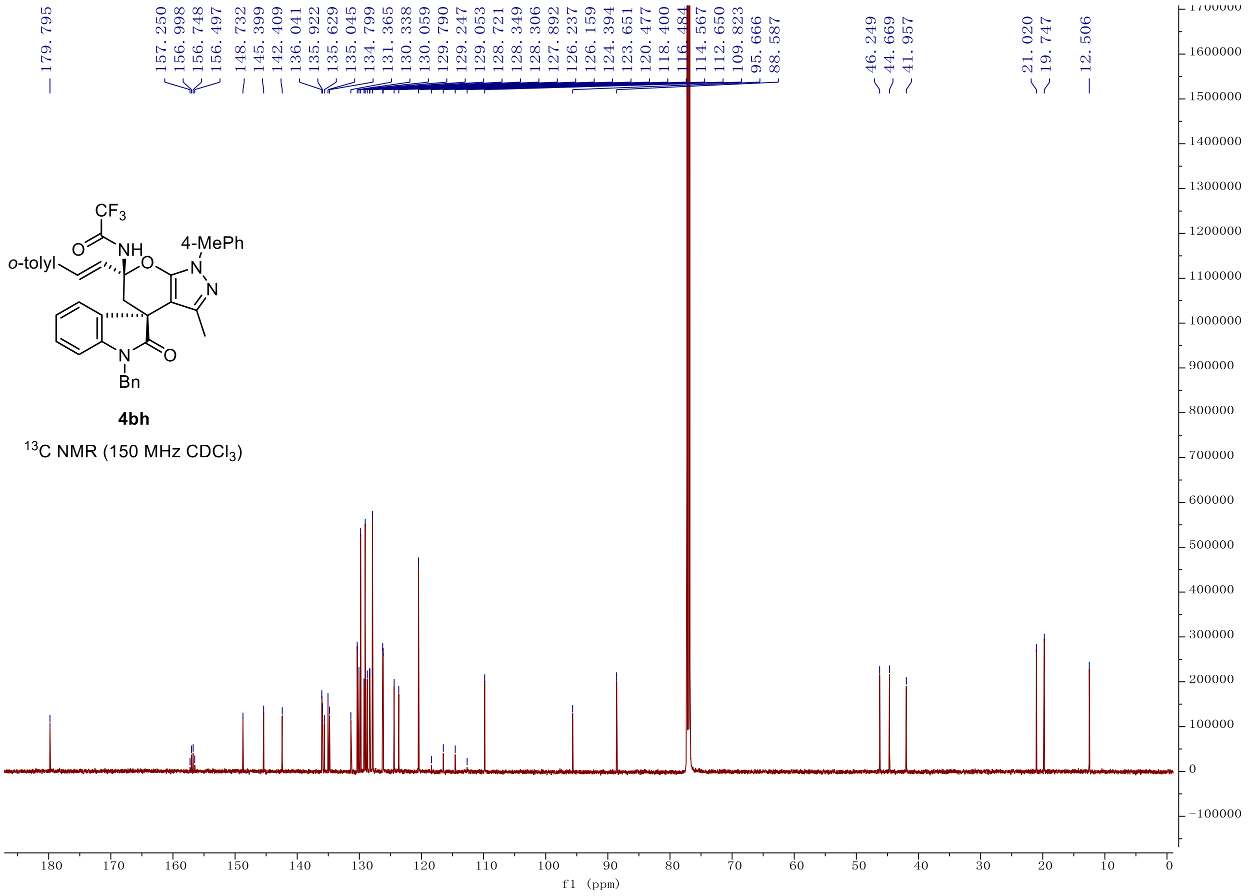

4bh
^{13}C NMR (150 MHz CDCl$_3$)

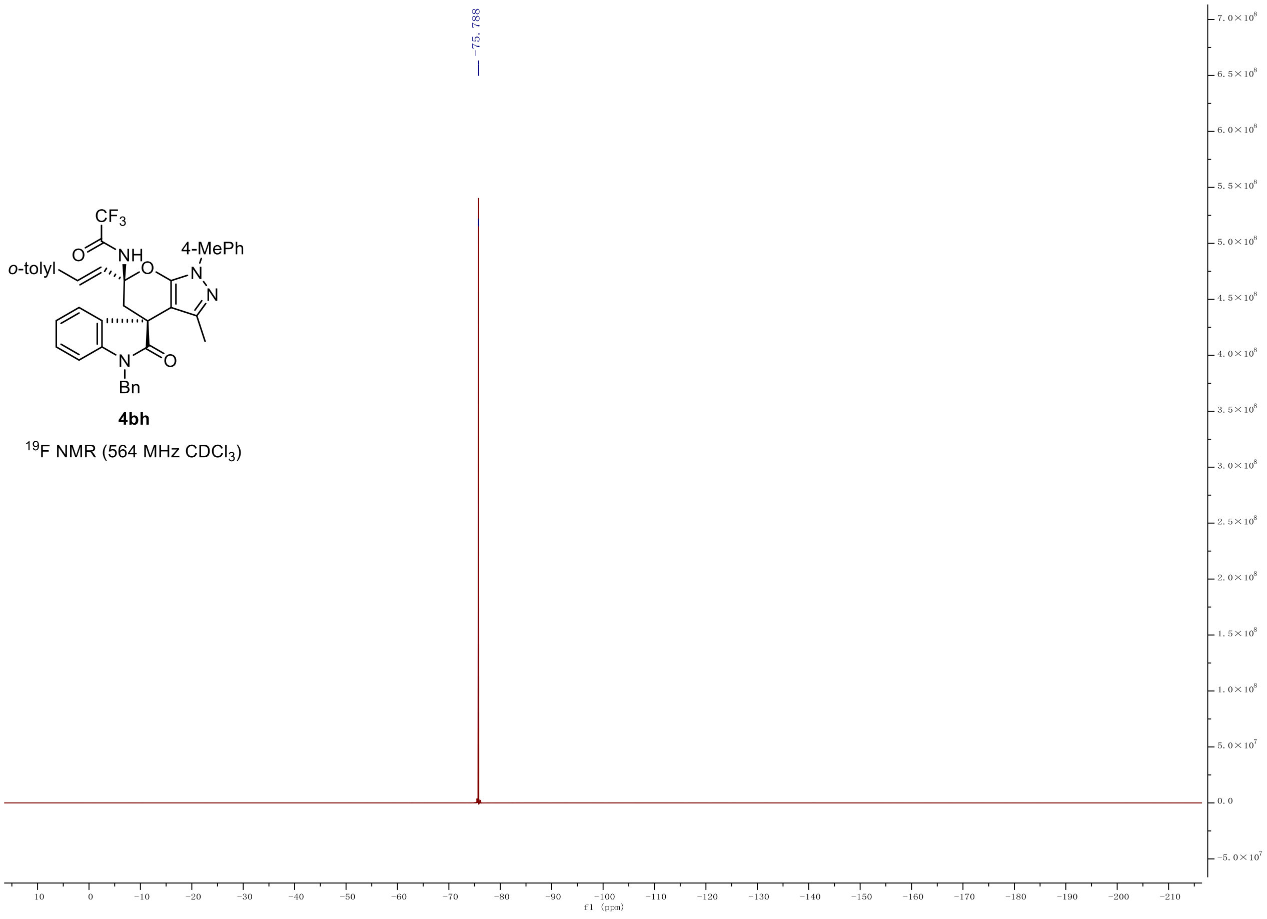

4bh
^{19}F NMR (564 MHz CDCl$_3$)

Peak Analysis Report

Detector A Channel 1 254nm

No.	Ret. Time	Height (mAu)	Area (mAu*min)	Rel. Area (%)
1	6.794	1014019	13175522	50.283
2	8.205	805011	13027066	49.717
Total		1819031	26202588	100.000

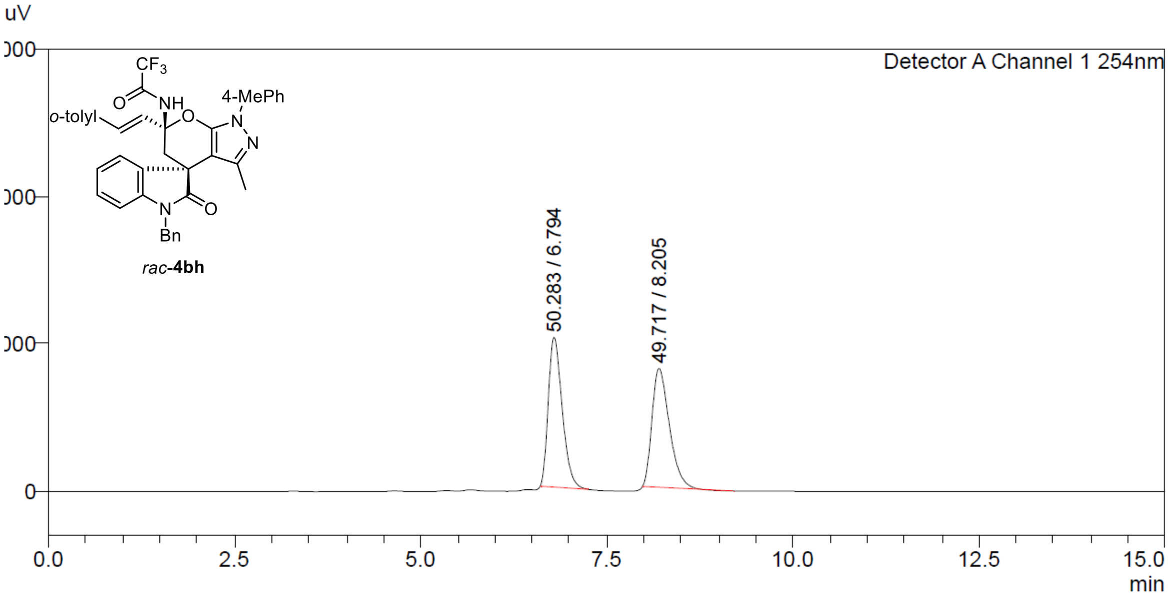

Peak Analysis Report

Detector A Channel 1 254nm

No.	Ret. Time	Height (mAu)	Area (mAu*min)	Rel. Area (%)
1	6.819	1556	11742	0.073
2	8.187	922308	16008237	99.927
Total		923864	16019979	100.000

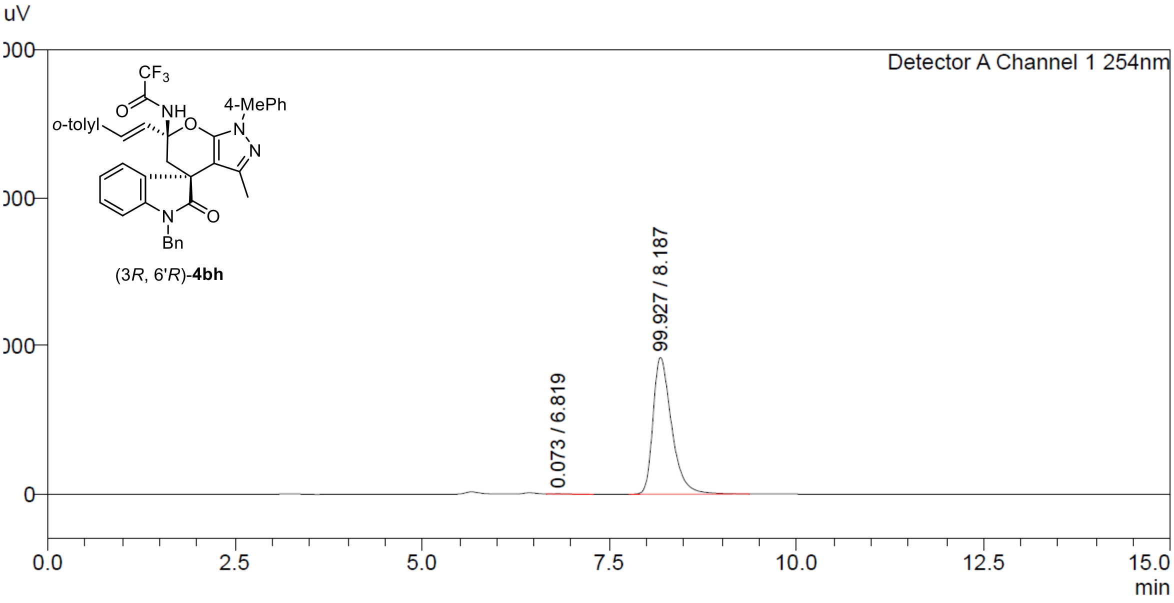

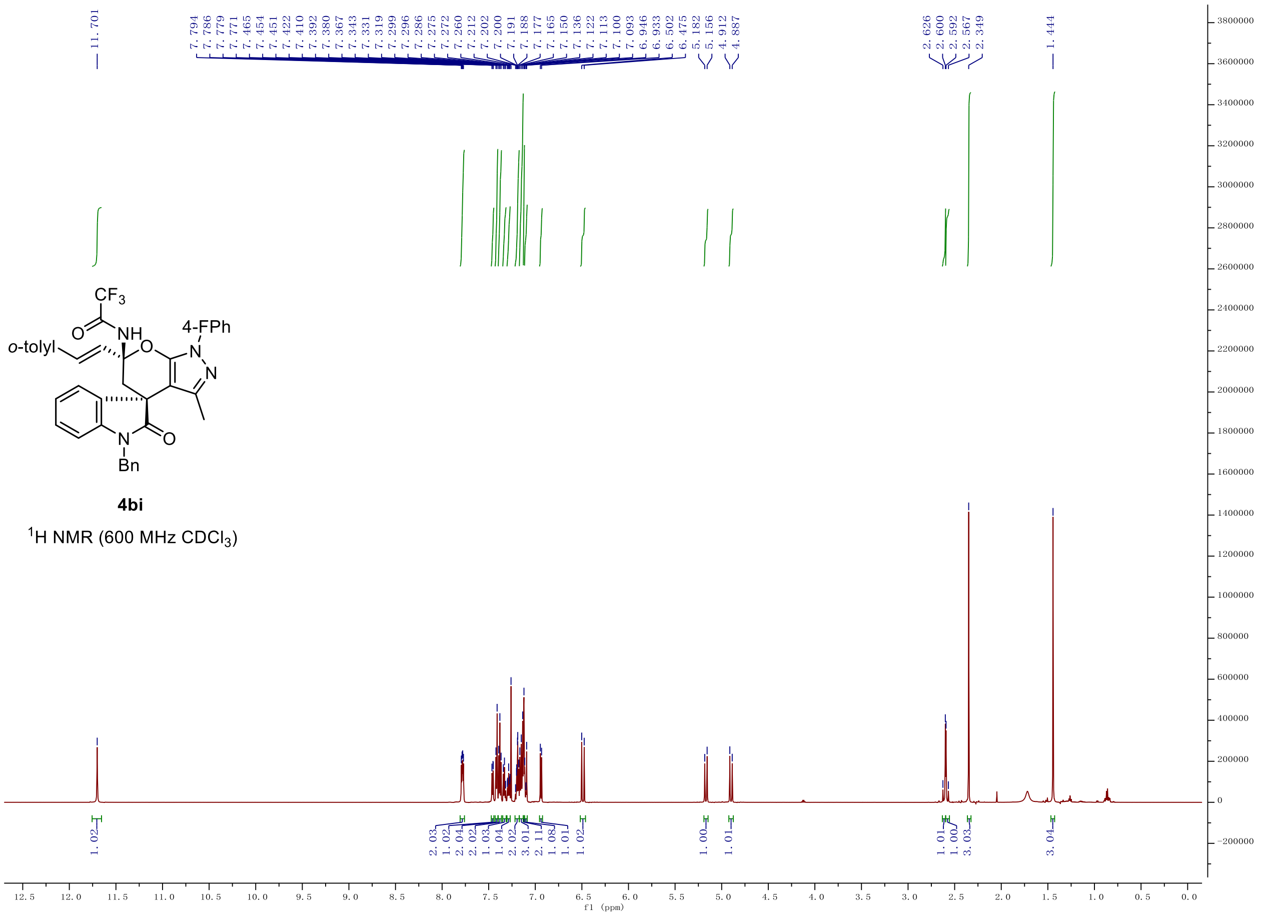

4bi

^{1}H NMR (600 MHz CDCl$_3$)

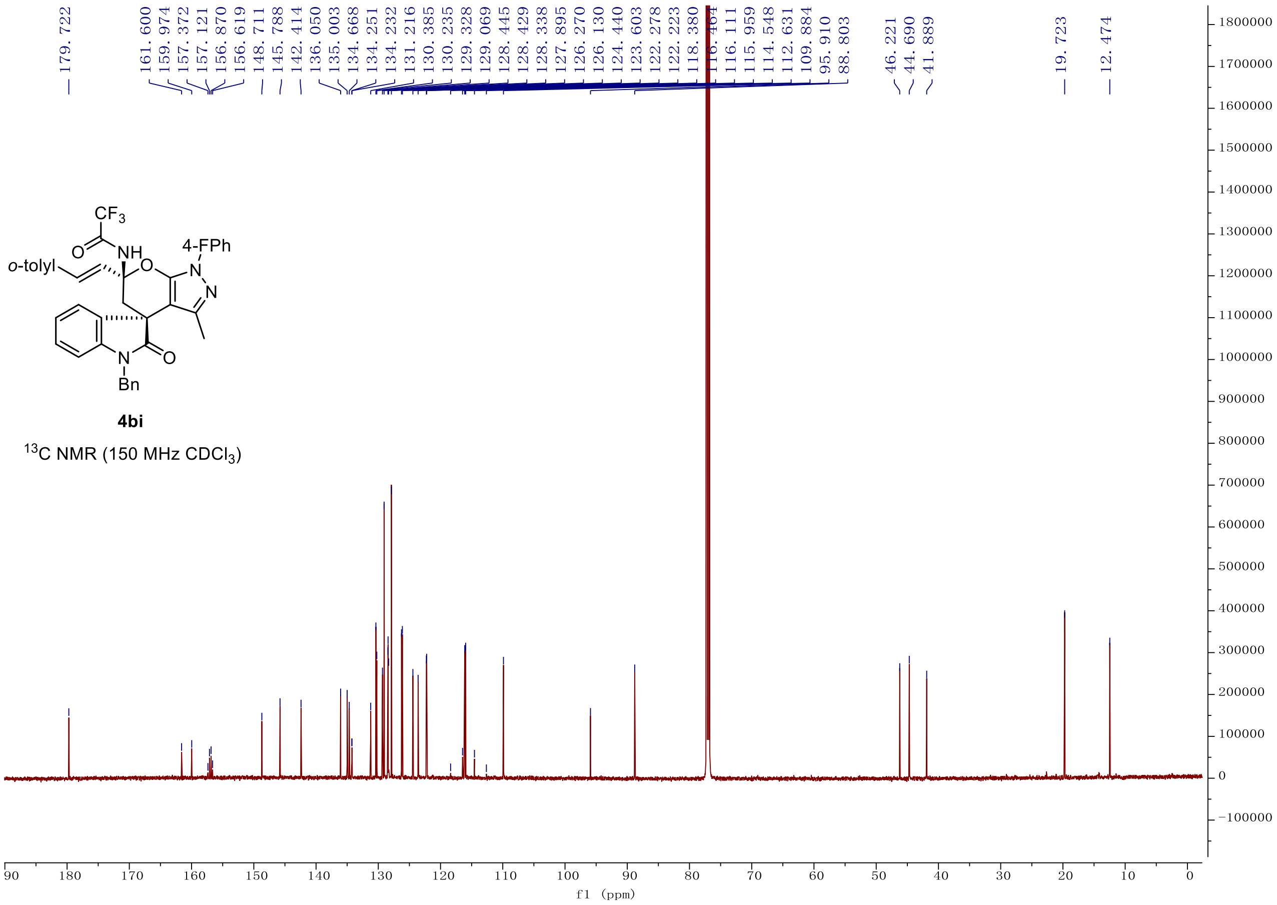

4bi

^{13}C NMR (150 MHz CDCl$_3$)

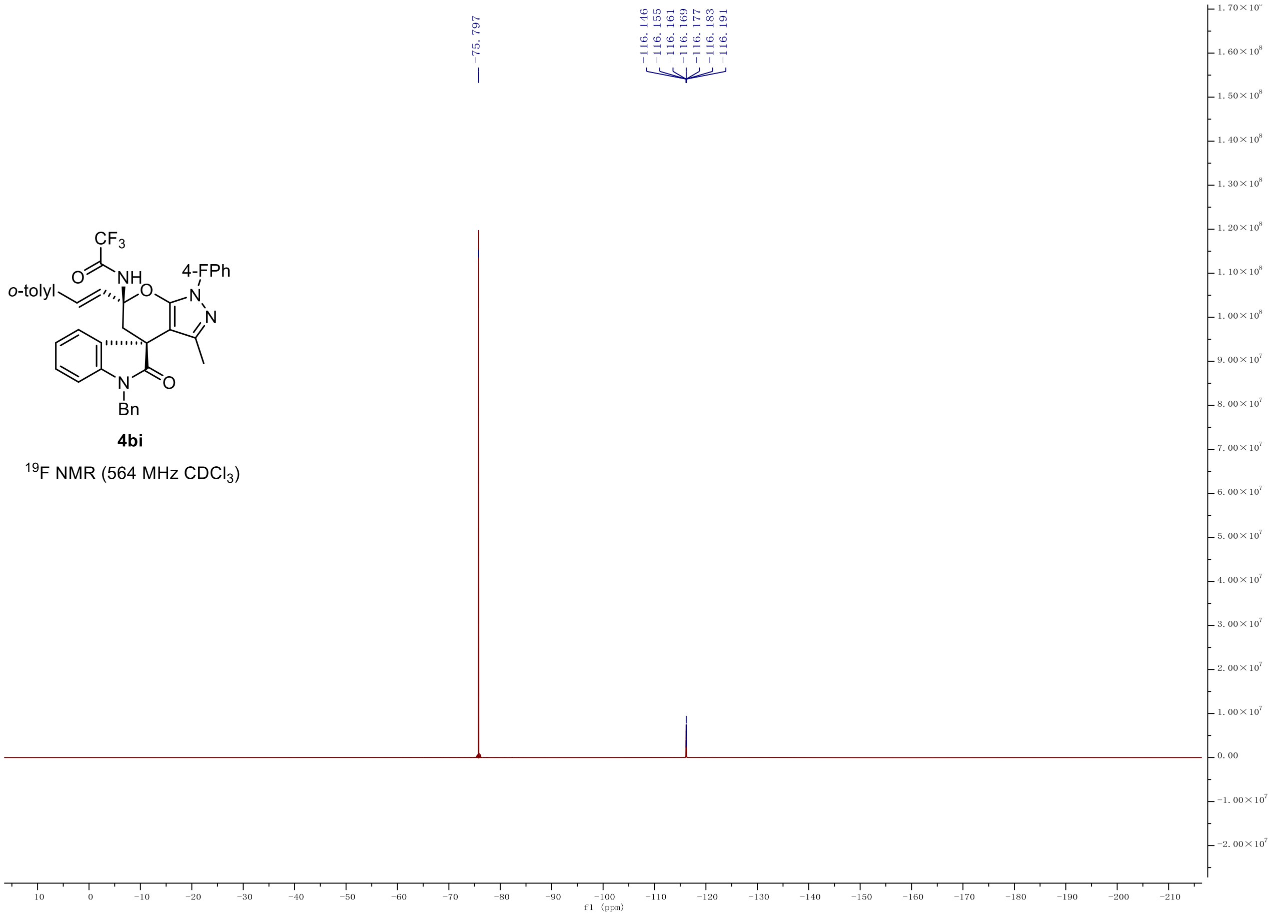

Peak Analysis Report

Detector A Channel 1 254nm

No.	Ret. Time	Height (mAu)	Area (mAu*min)	Rel. Area (%)
1	8.905	265755	5411887	50.337
2	9.944	209221	5339438	49.663
Total		474975	10751325	100.000

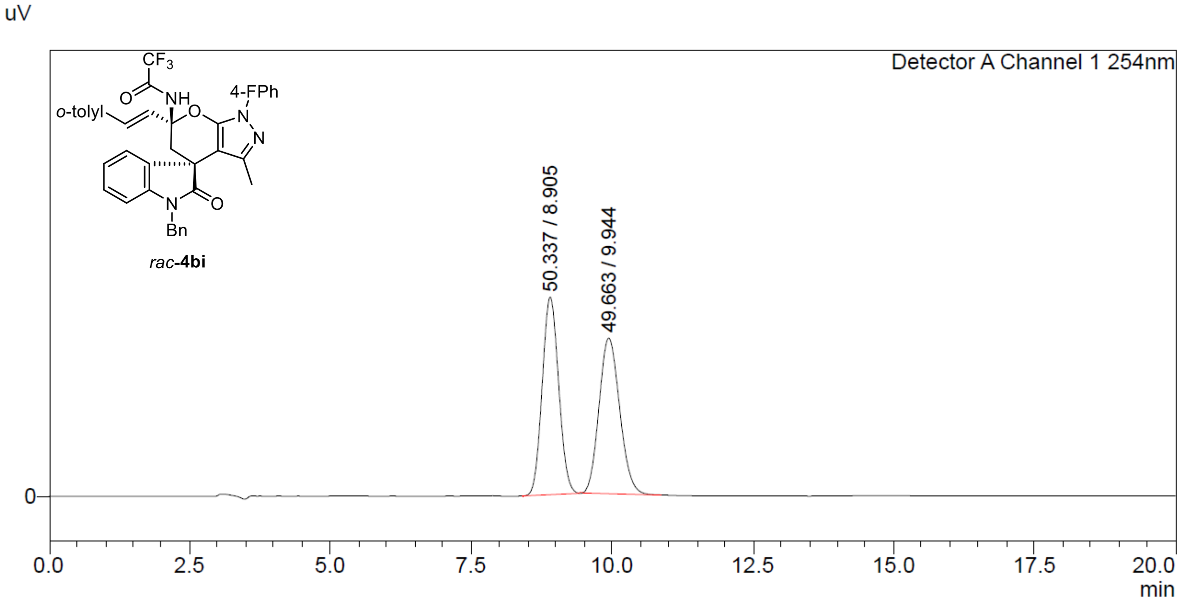

Peak Analysis Report

Detector A Channel 1 254nm

No.	Ret. Time	Height (mAu)	Area (mAu*min)	Rel. Area (%)
1	8.904	706180	13624245	99.566
2	9.946	2807	59436	0.434
Total		708987	13683681	100.000

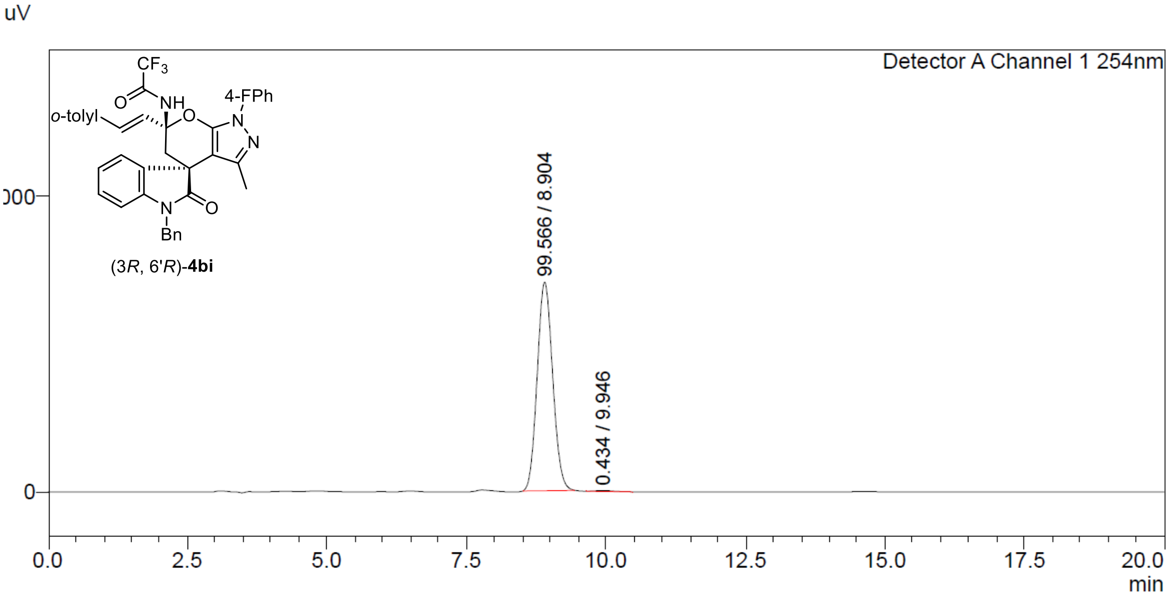

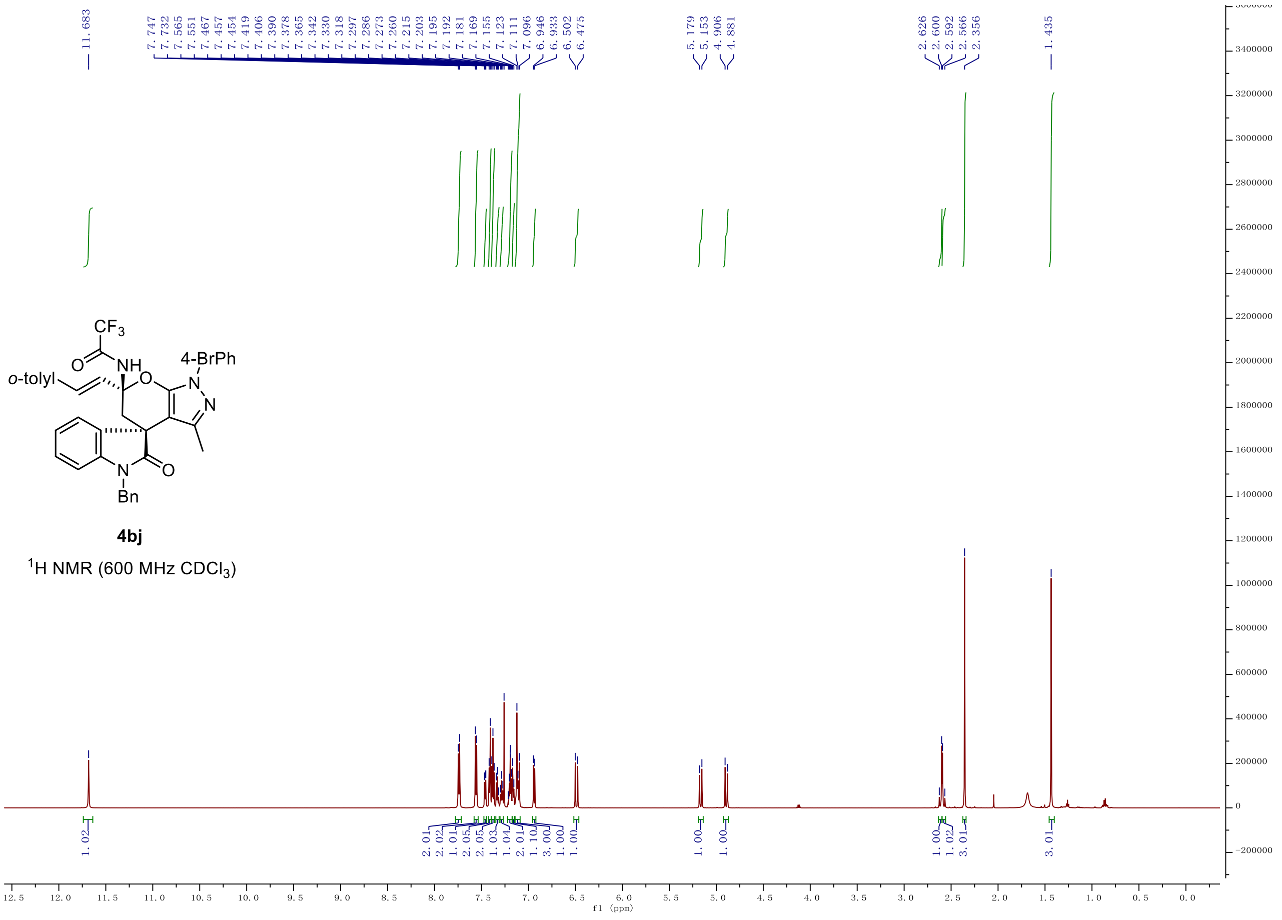

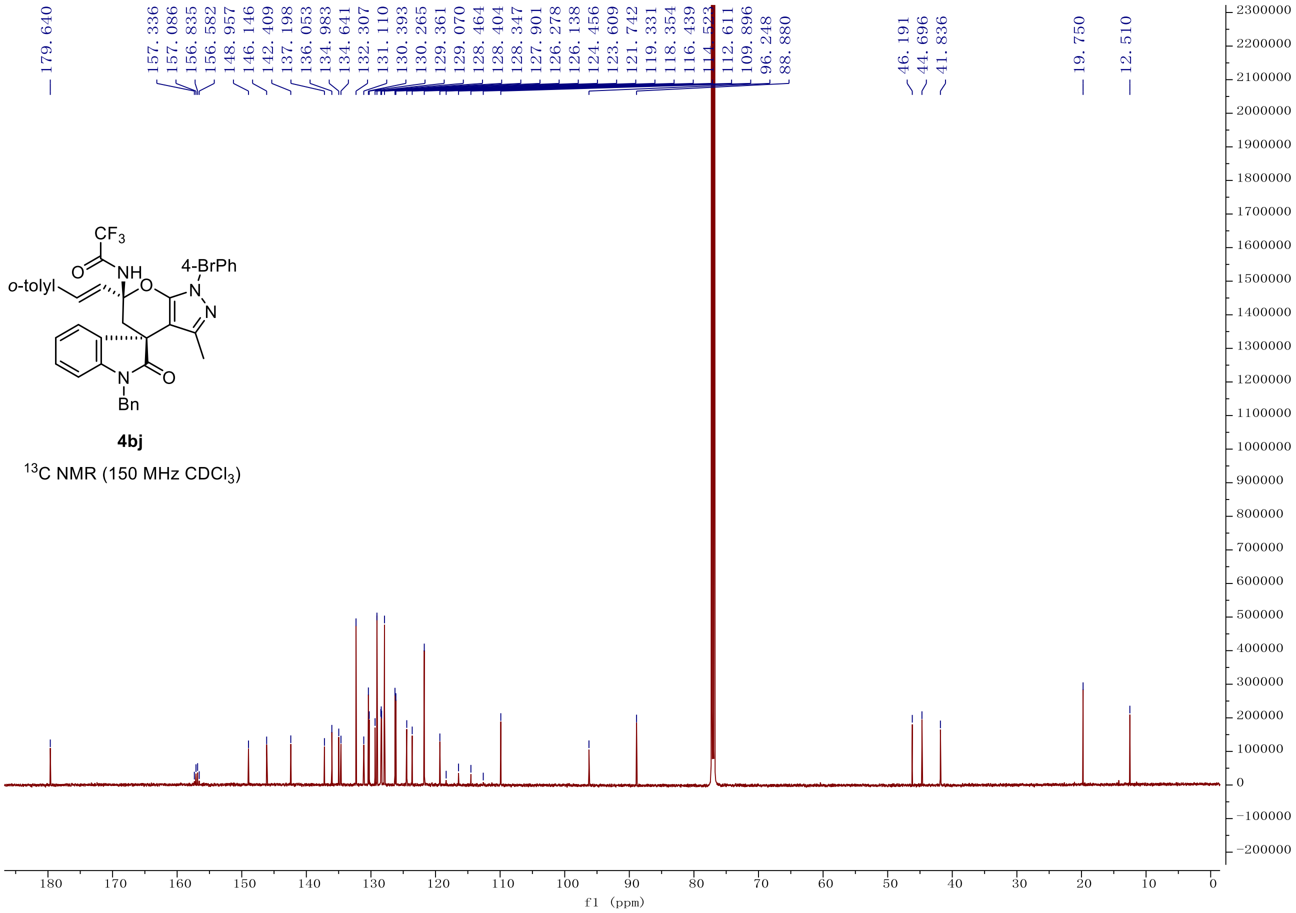

4bj

^{13}C NMR (150 MHz $CDCl_3$)

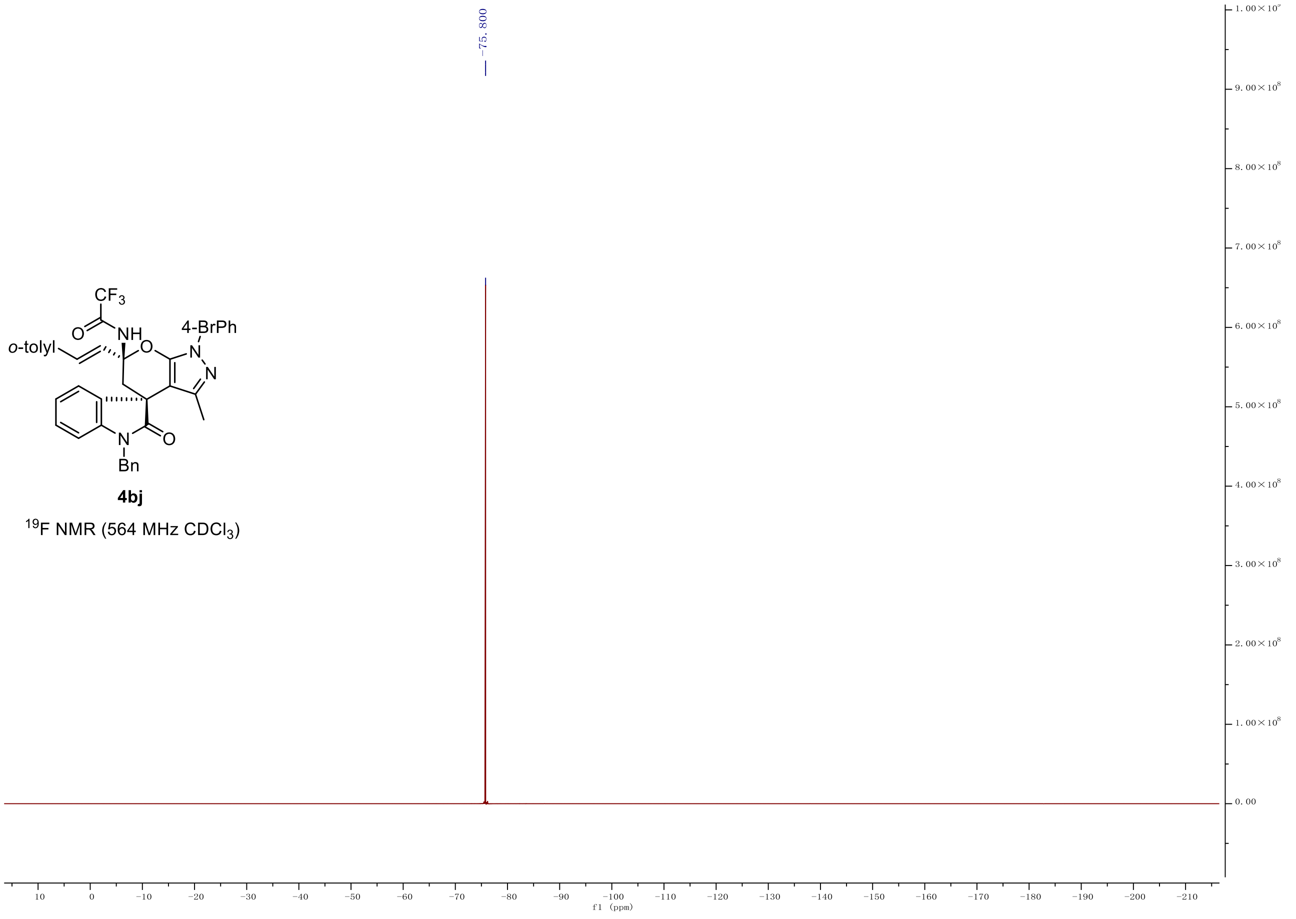

4bj

^{19}F NMR (564 MHz $CDCl_3$)

Peak Analysis Report

Detector A Channel 1 254nm

No.	Ret. Time	Height (mAu)	Area (mAu*min)	Rel. Area (%)
1	7.357	1017358	13892125	49.149
2	9.025	819043	14372983	50.851
Total		1836401	28265108	100.000

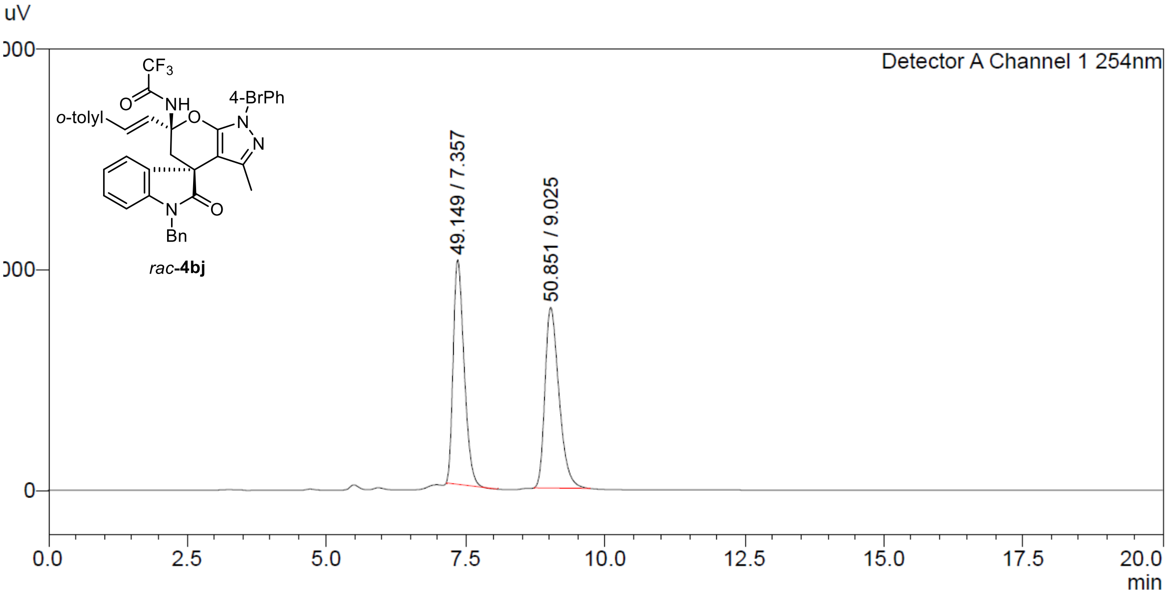

Peak Analysis Report

Detector A Channel 1 254nm

No.	Ret. Time	Height (mAu)	Area (mAu*min)	Rel. Area (%)
1	7.406	4921	57798	0.251
2	8.983	1353094	22994645	99.749
Total		1358015	23052443	100.000

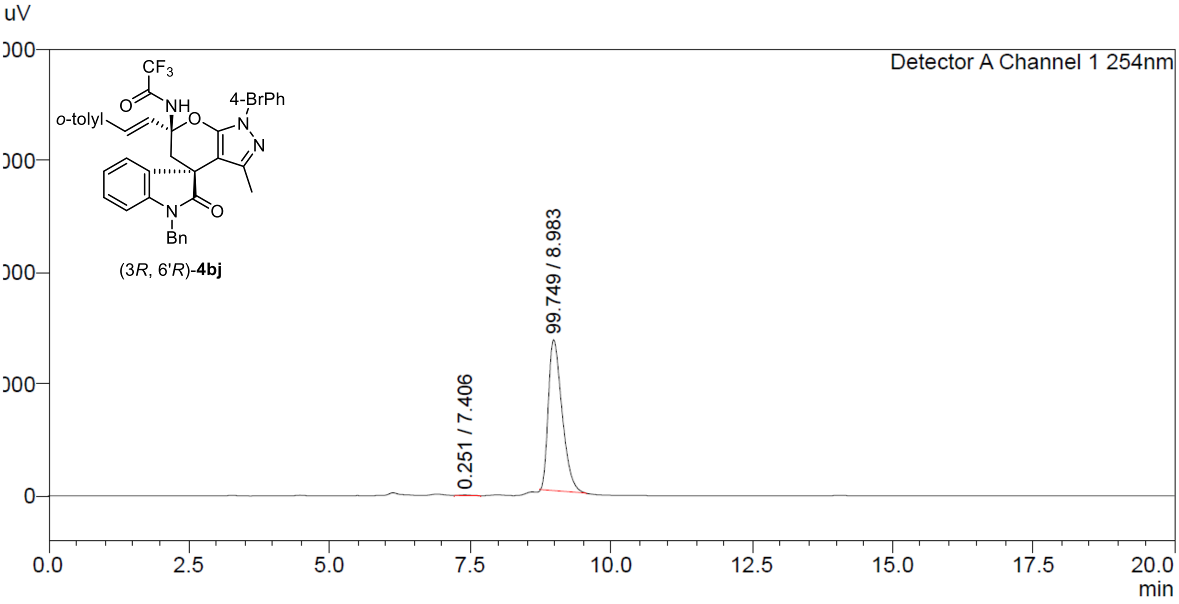

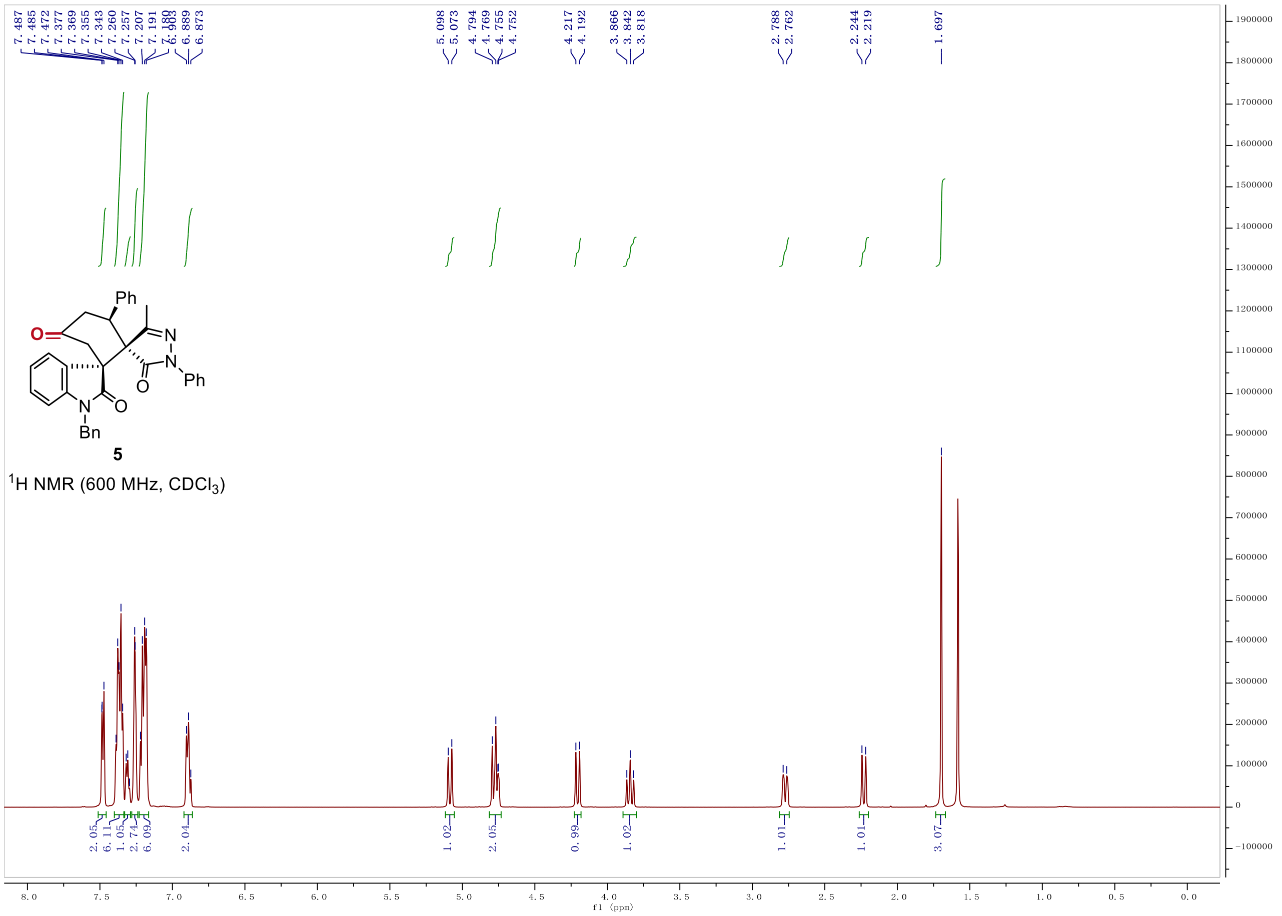
7.487
7.485
7.472
7.377
7.369
7.355
7.343
7.260
7.257
7.207
7.191
7.180
6.903
6.889
6.873
5.098
5.073
4.794
4.769
4.755
4.752
4.217
4.192
3.866
3.842
3.818
2.788
2.762
2.244
2.219
1.697
Ph
O
N
N
Ph
O
N
O
Bn
5
^{1}H NMR (600 MHz, CDCl$_3$)
2.05
6.11
1.05
2.74
6.09
2.04
1.02
2.05
0.99
1.02
1.01
1.01
3.07
f1 (ppm)

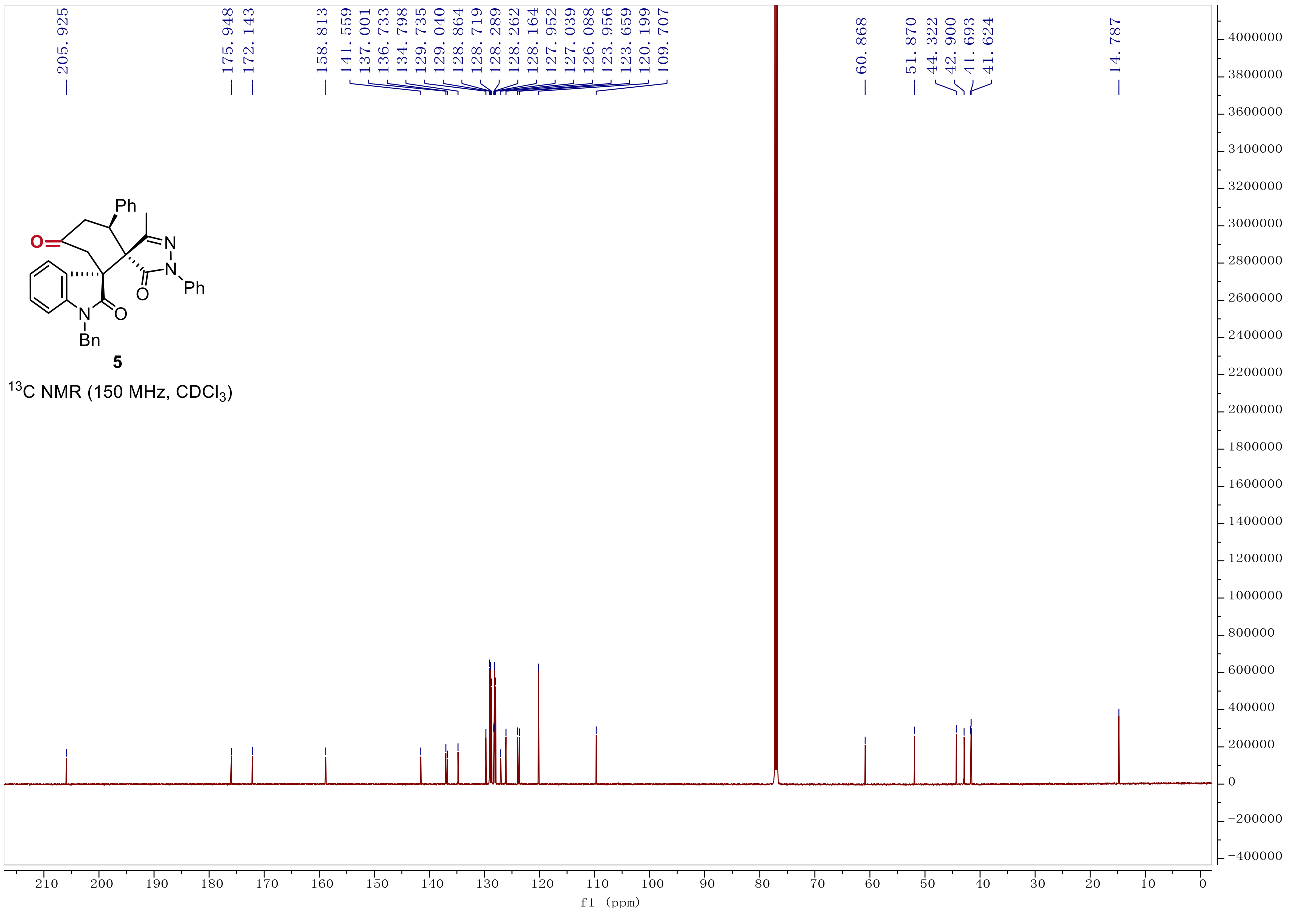
205.925
175.948
172.143
158.813
141.559
137.001
136.733
134.798
129.735
129.040
128.864
128.719
128.289
128.262
128.164
127.952
127.039
126.088
123.956
123.659
120.199
109.707
60.868
51.870
44.322
42.900
41.693
41.624
14.787
Ph
O
N
N
Ph
O
N
O
Bn
5
^{13}C NMR (150 MHz, CDCl$_3$)
f1 (ppm)

Peak Analysis Report

Detector A Channel 1 254nm

No.	Ret. Time	Height (mAu)	Area (mAu*min)	Rel. Area (%)
1	8.355	484731	7522178	49.556
2	11.608	361951	7657007	50.444
Total		846683	15179185	100.000

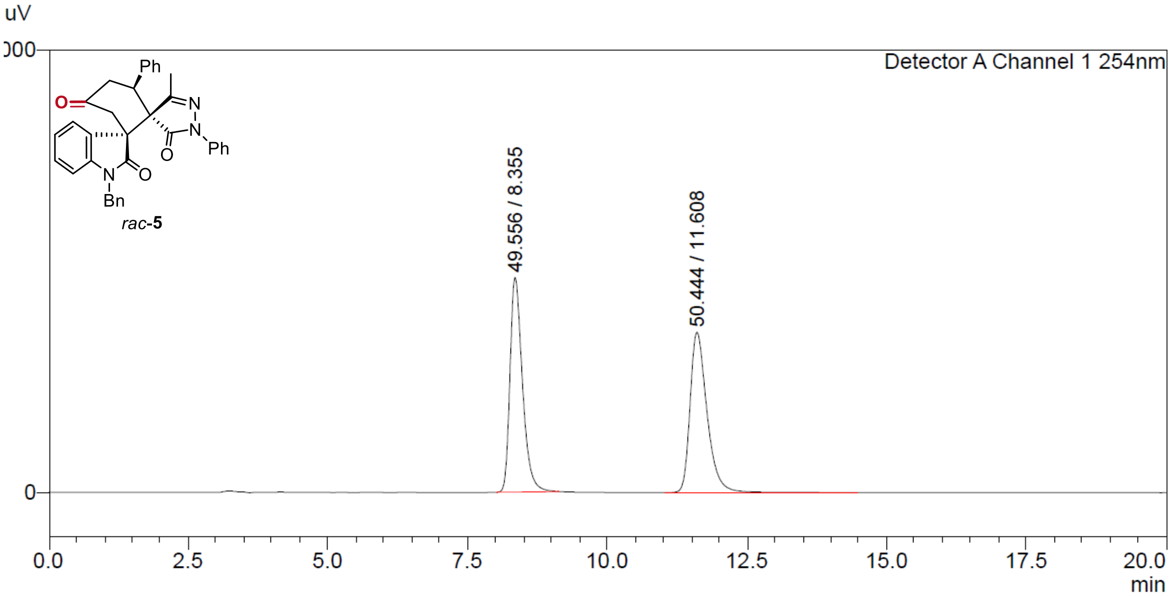

Peak Analysis Report

Detector A Channel 1 254nm

No.	Ret. Time	Height (mAu)	Area (mAu*min)	Rel. Area (%)
1	8.425	280	3950	0.043
2	11.607	432643	9194245	99.957
Total		432922	9198195	100.000

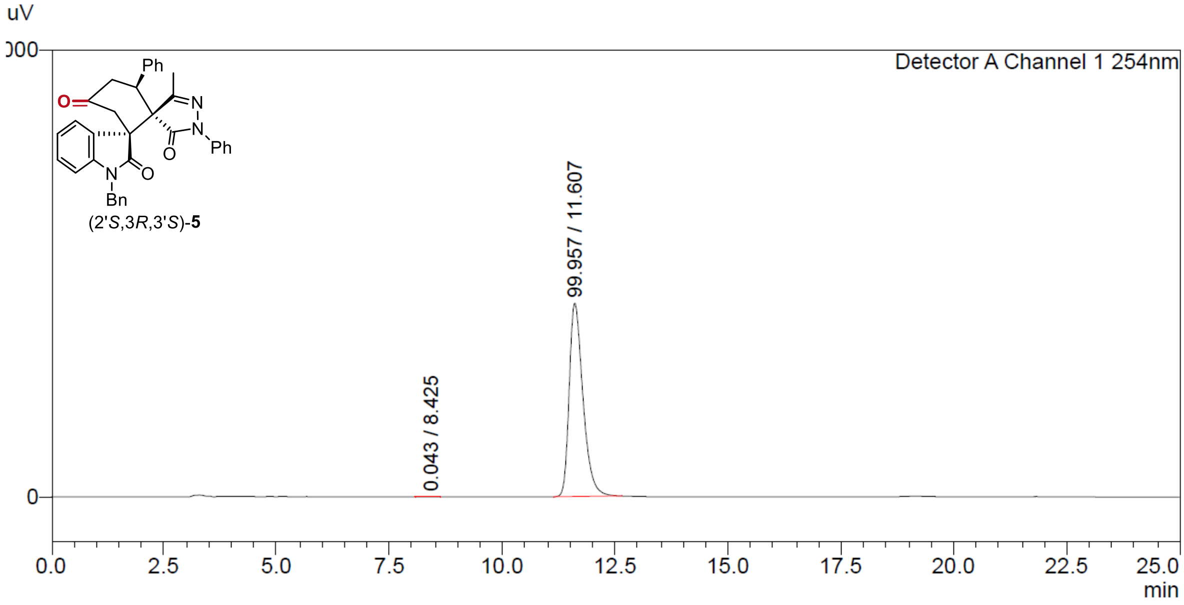

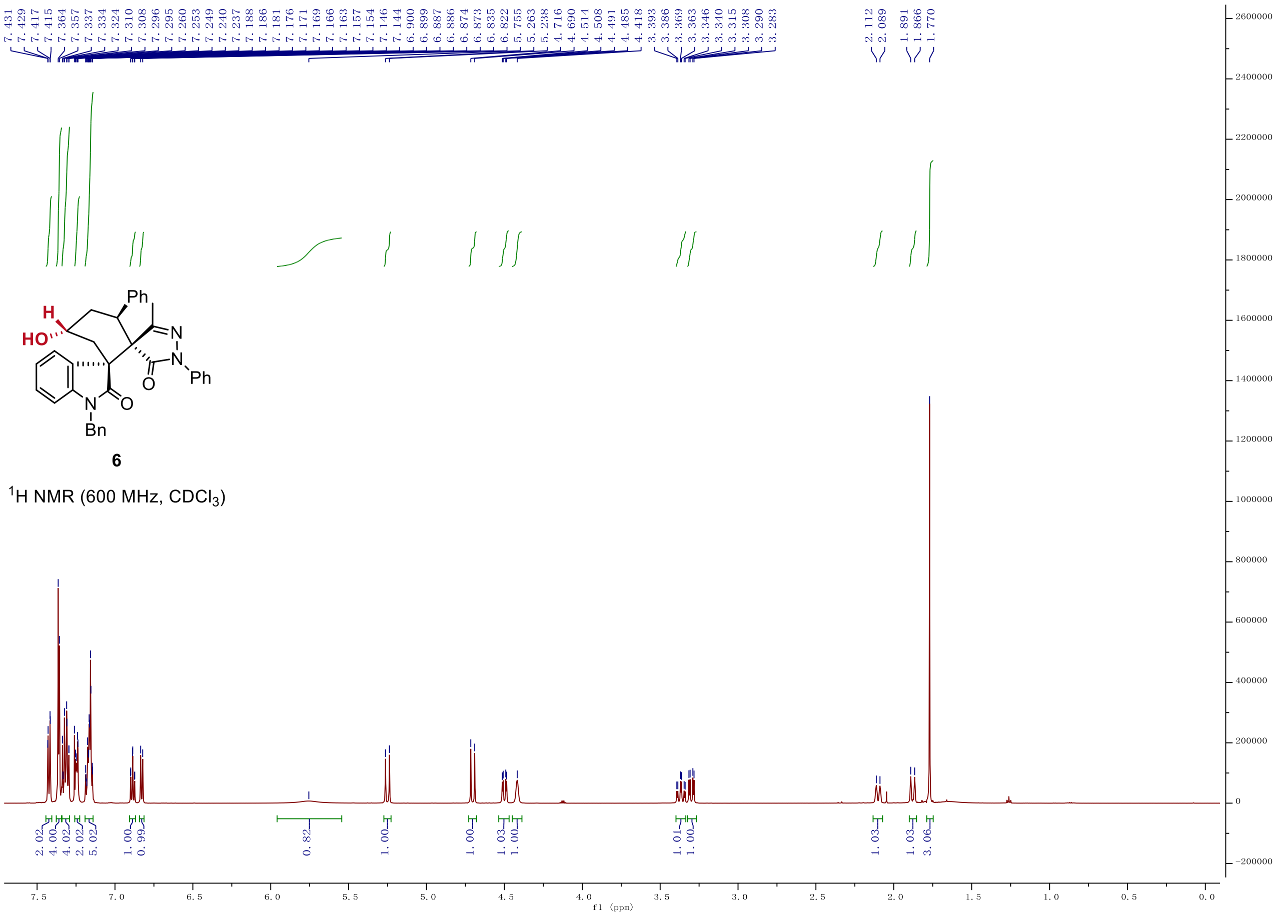

6

^{1}H NMR (600 MHz, $CDCl_3$)

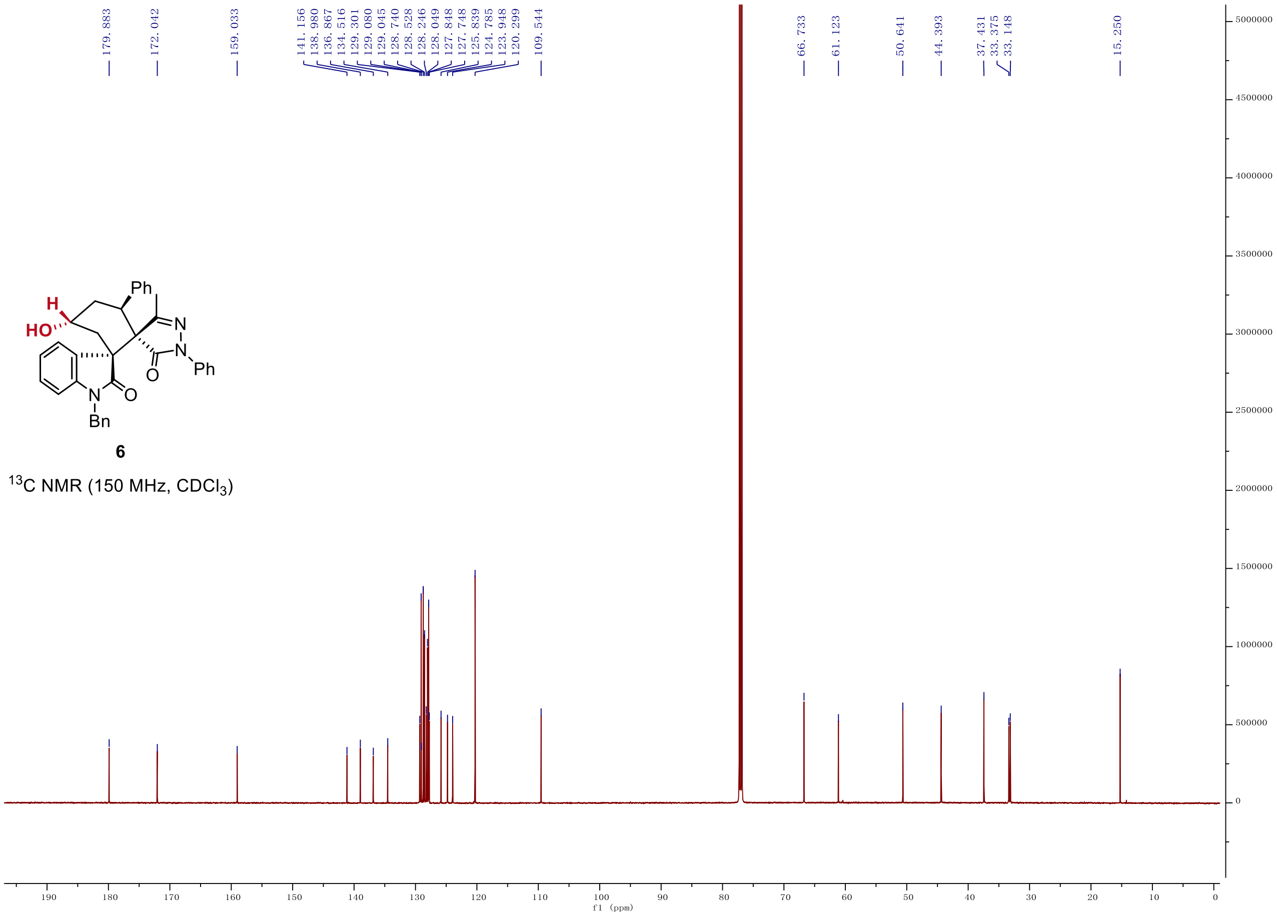

6

^{13}C NMR (150 MHz, $CDCl_3$)

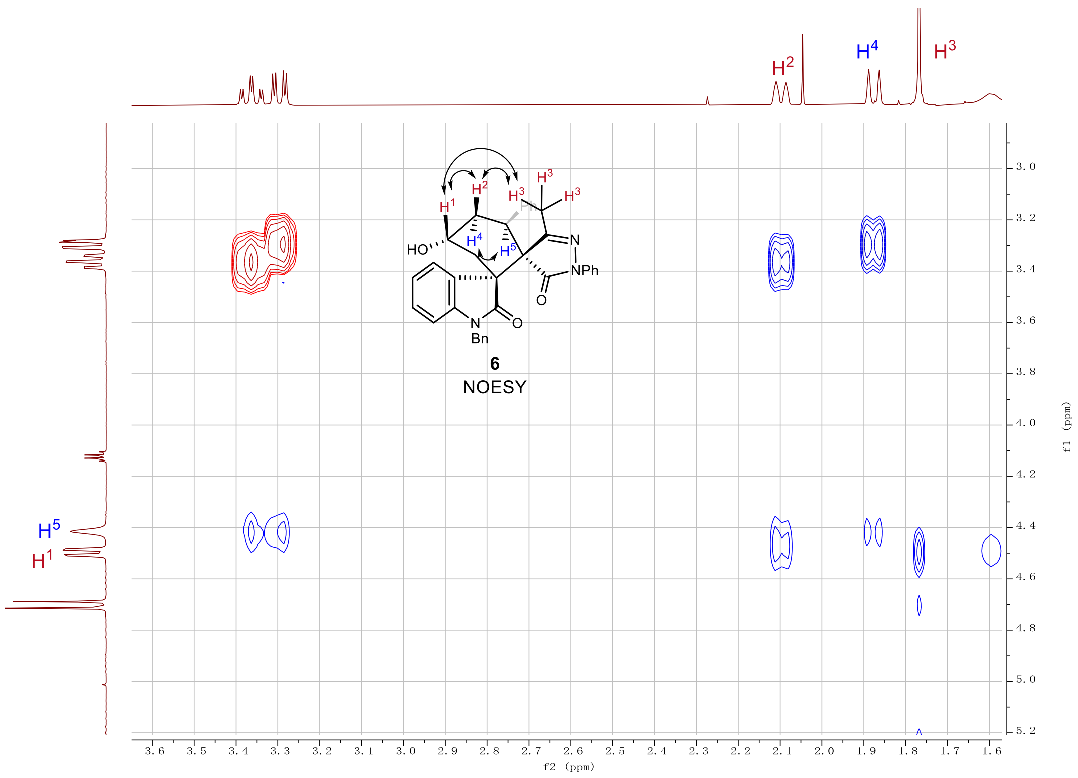

Peak Analysis Report

Detector A Channel 1 254nm

No.	Ret. Time	Height (mAu)	Area (mAu*min)	Rel. Area (%)
1	10.774	742836	16324035	49.863
2	29.016	227006	16413457	50.137
Total		969842	32737492	100.000

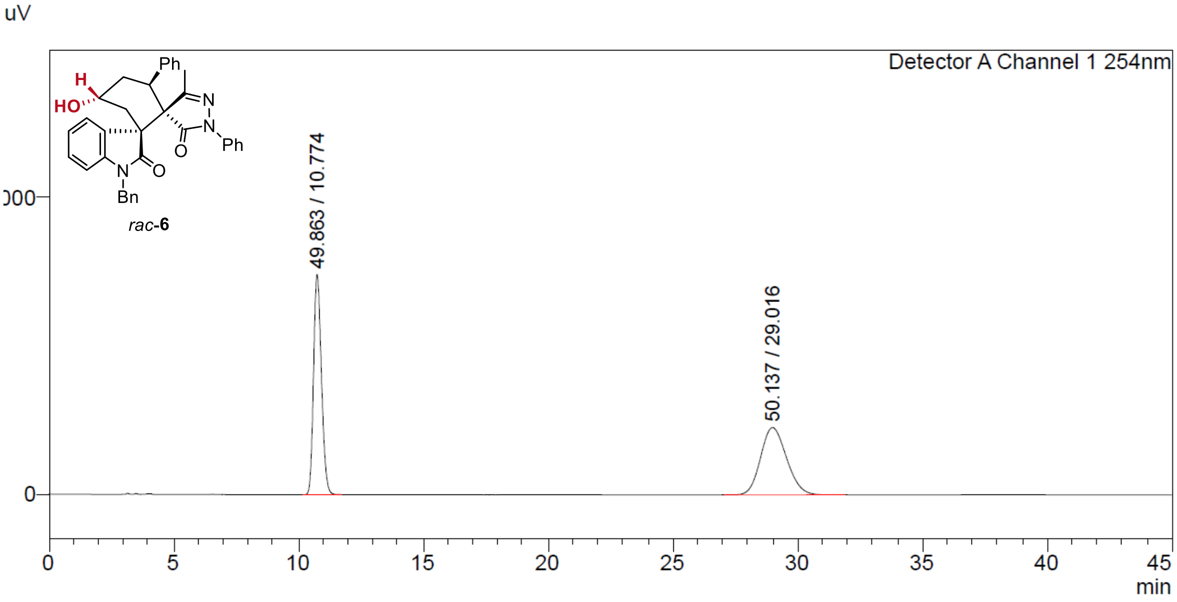

Peak Analysis Report

Detector A Channel 1 254nm

No.	Ret. Time	Height (mAu)	Area (mAu*min)	Rel. Area (%)
1	10.812	295	6654	0.036
2	28.816	262690	18707751	99.964
Total		262985	18714406	100.000

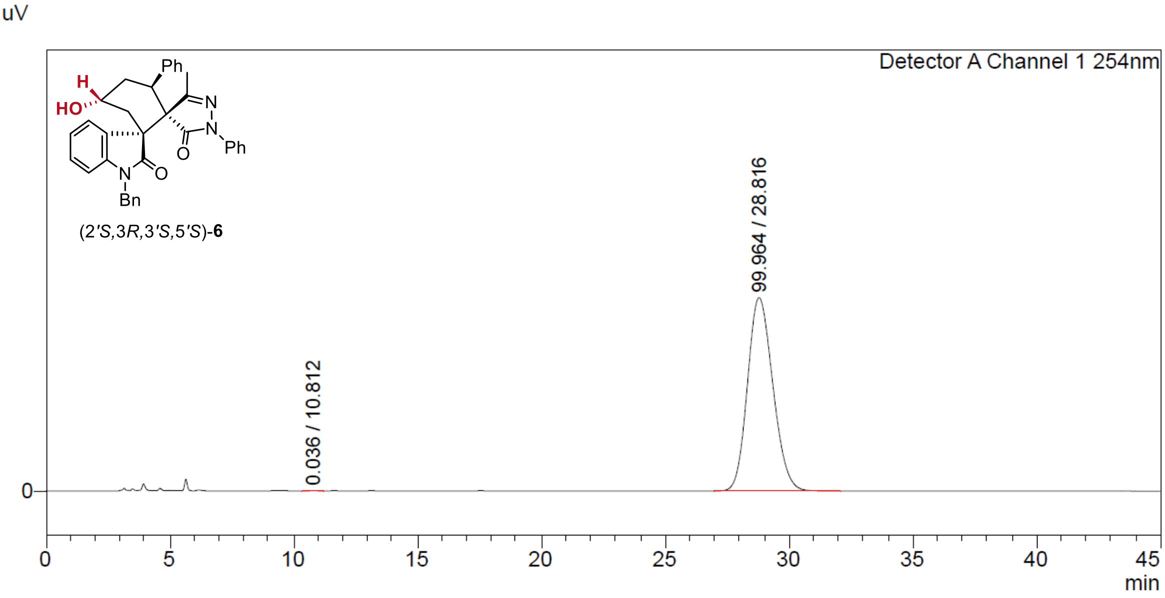

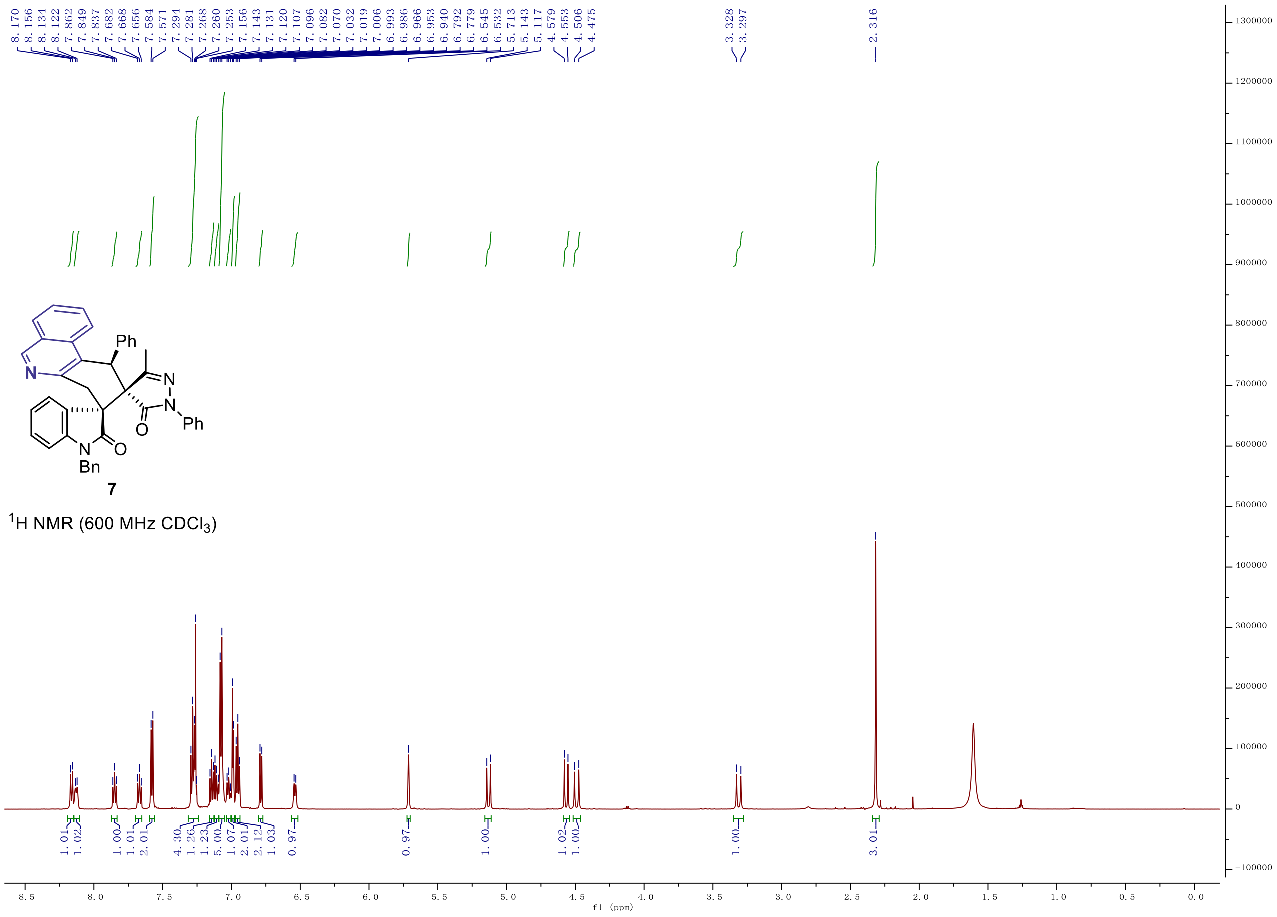

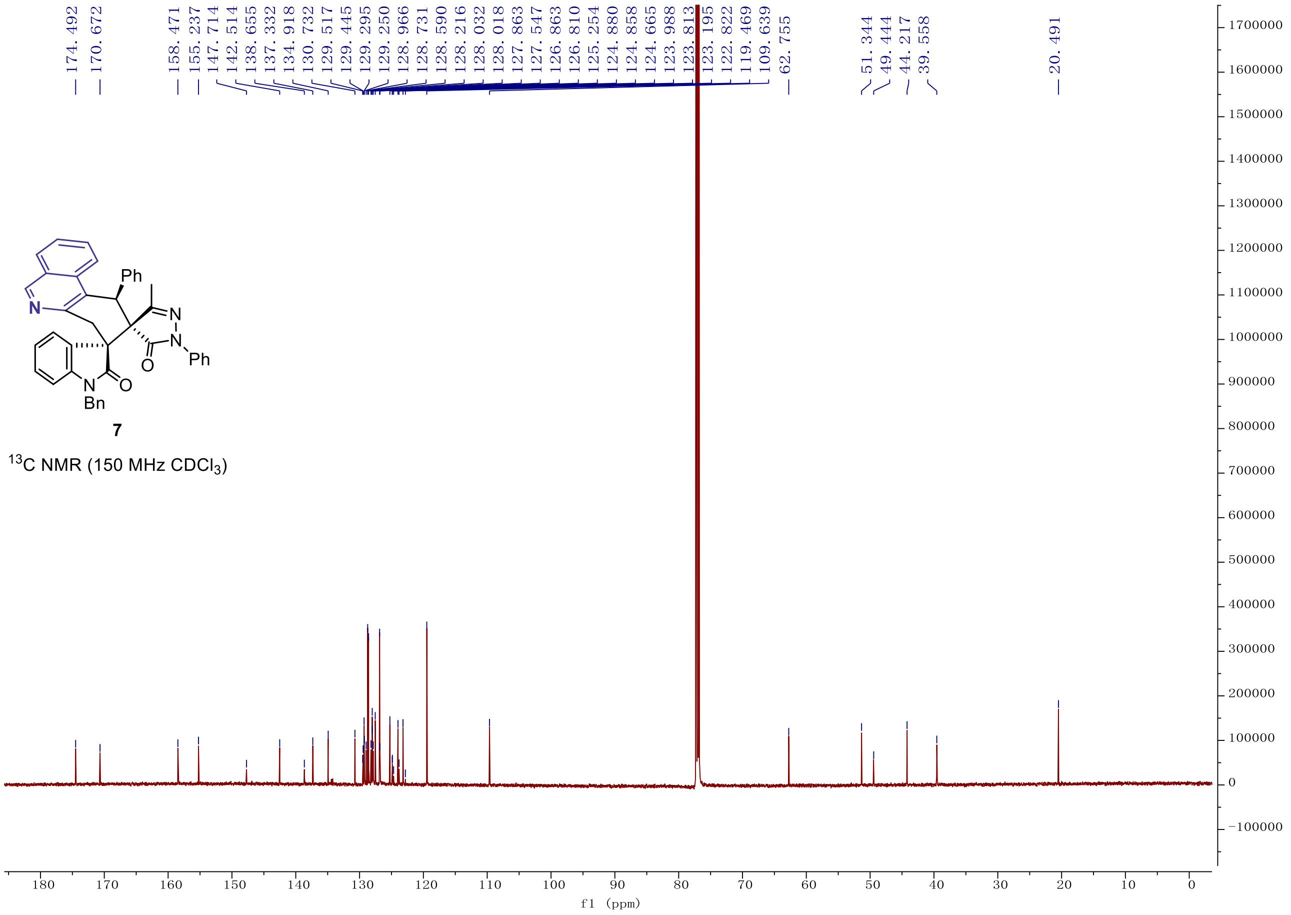

7

^{13}C NMR (150 MHz $CDCl_3$)

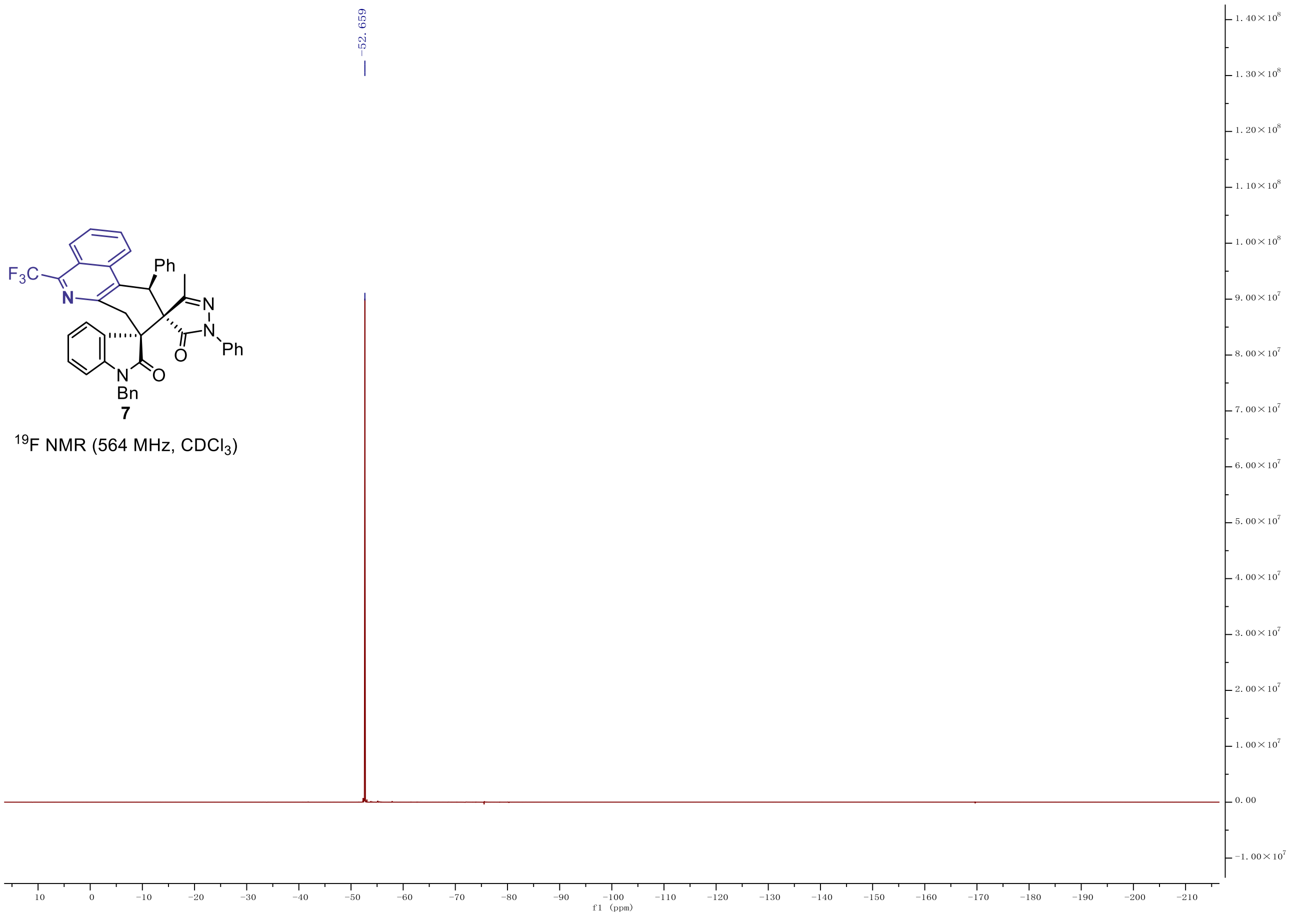

7

^{19}F NMR (564 MHz, $CDCl_3$)

Peak Analysis Report

Detector A Channel 1 254nm

No.	Ret. Time	Height (mAu)	Area (mAu*min)	Rel. Area (%)
1	18.950	95817	6082039	49.601
2	25.325	103632	6179830	50.399
Total		199449	12261869	100.000

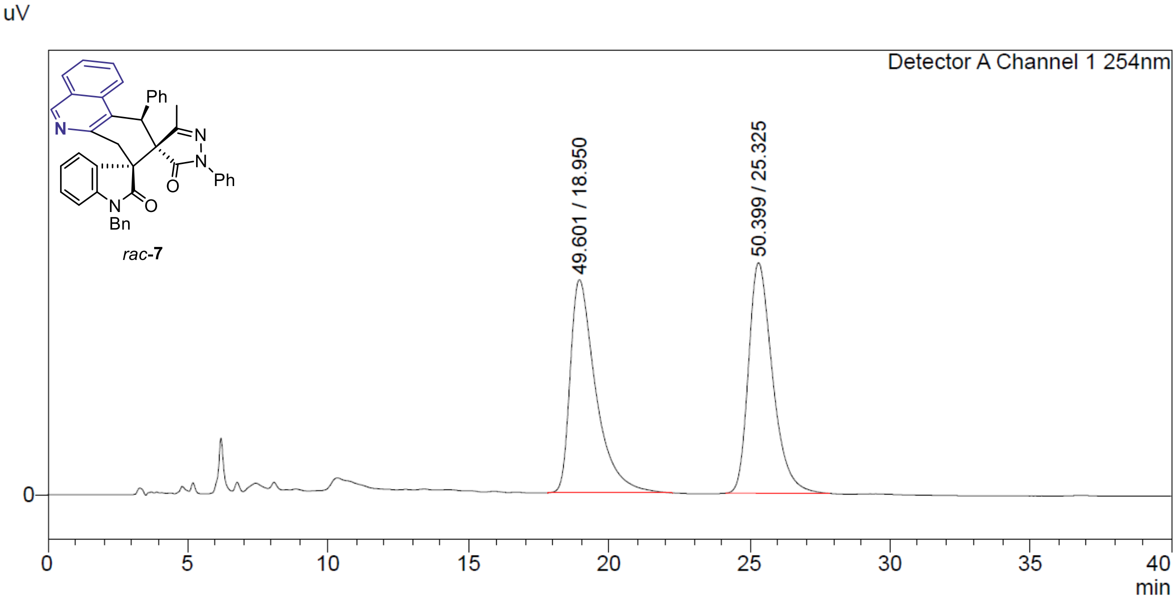

Peak Analysis Report

Detector A Channel 1 254nm

No.	Ret. Time	Height (mAu)	Area (mAu*min)	Rel. Area (%)
1	19.096	153	5138	0.118
2	25.229	72897	4337488	99.882
Total		73049	4342627	100.000

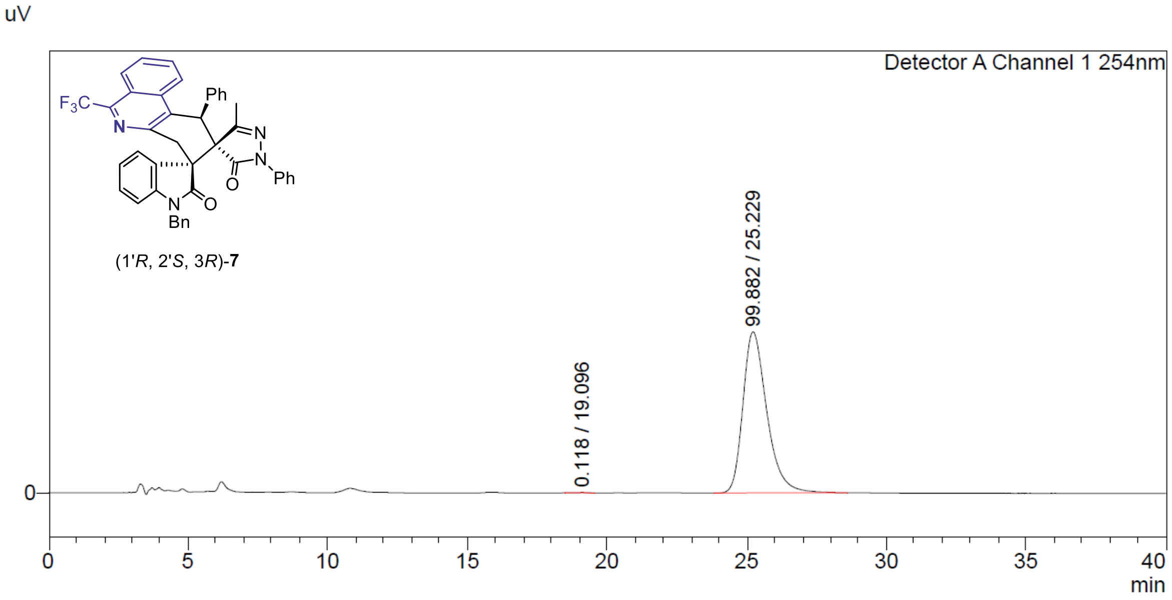

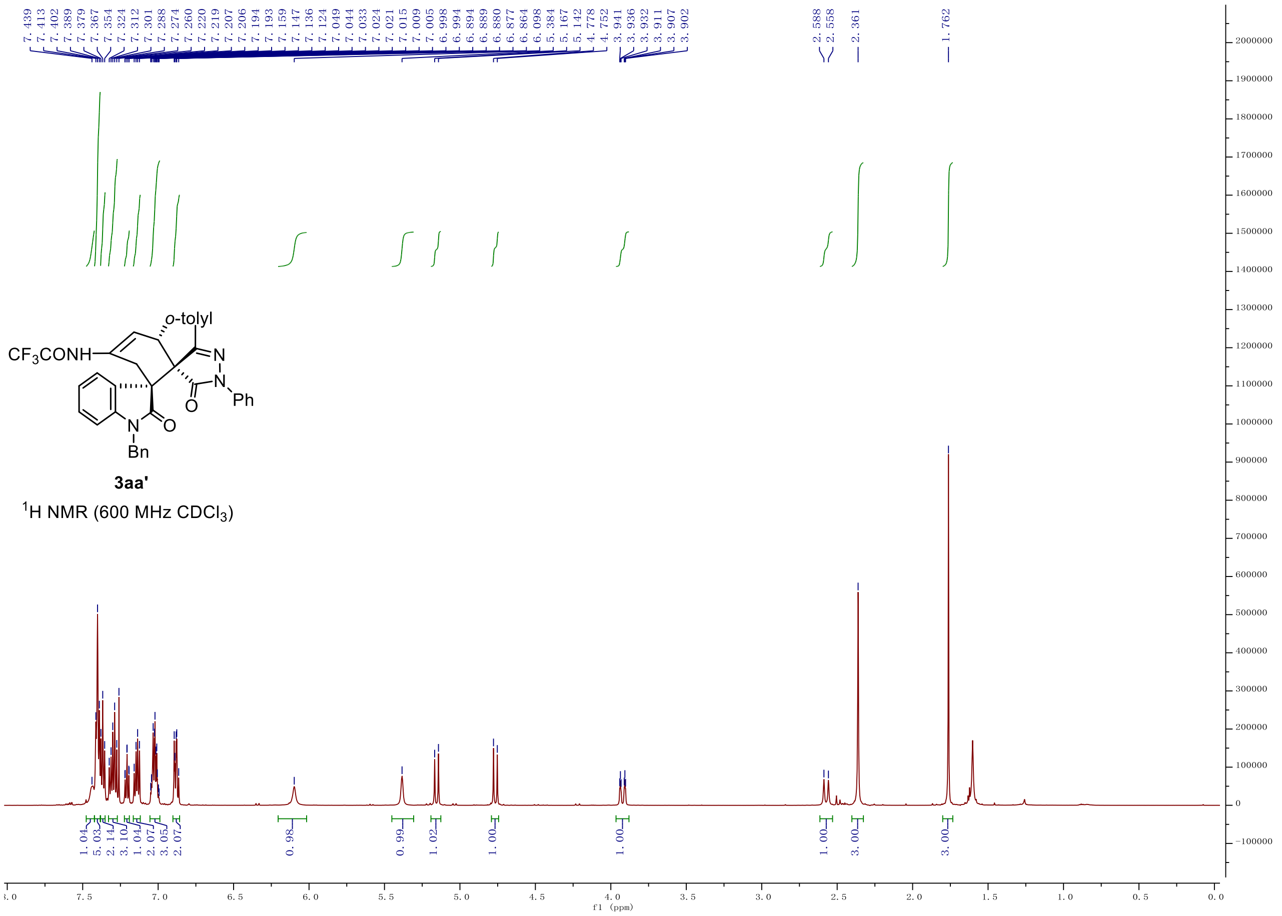
7.439 7.413 7.402 7.389 7.379 7.367 7.354 7.324 7.312 7.301 7.288 7.274 7.260 7.220 7.219 7.207 7.206 7.194 7.193 7.159 7.147 7.136 7.124 7.049 7.044 7.033 7.024 7.021 7.015 7.009 7.005 6.998 6.994 6.894 6.889 6.880 6.877 6.864 6.098 5.384 5.167 5.142 4.778 4.752 3.941 3.936 3.932 3.911 3.907 3.902
2.588 2.558
2.361
1.762
o-tolyl
CF3CONH
N
N
Ph
O
N
O
Bn
3aa'
1H NMR (600 MHz CDCl3)
1.04 5.03 2.14 3.10 1.04 2.07 3.05 2.07
0.98
0.99
1.02
1.00
1.00
1.00
3.00
3.00
f1 (ppm)

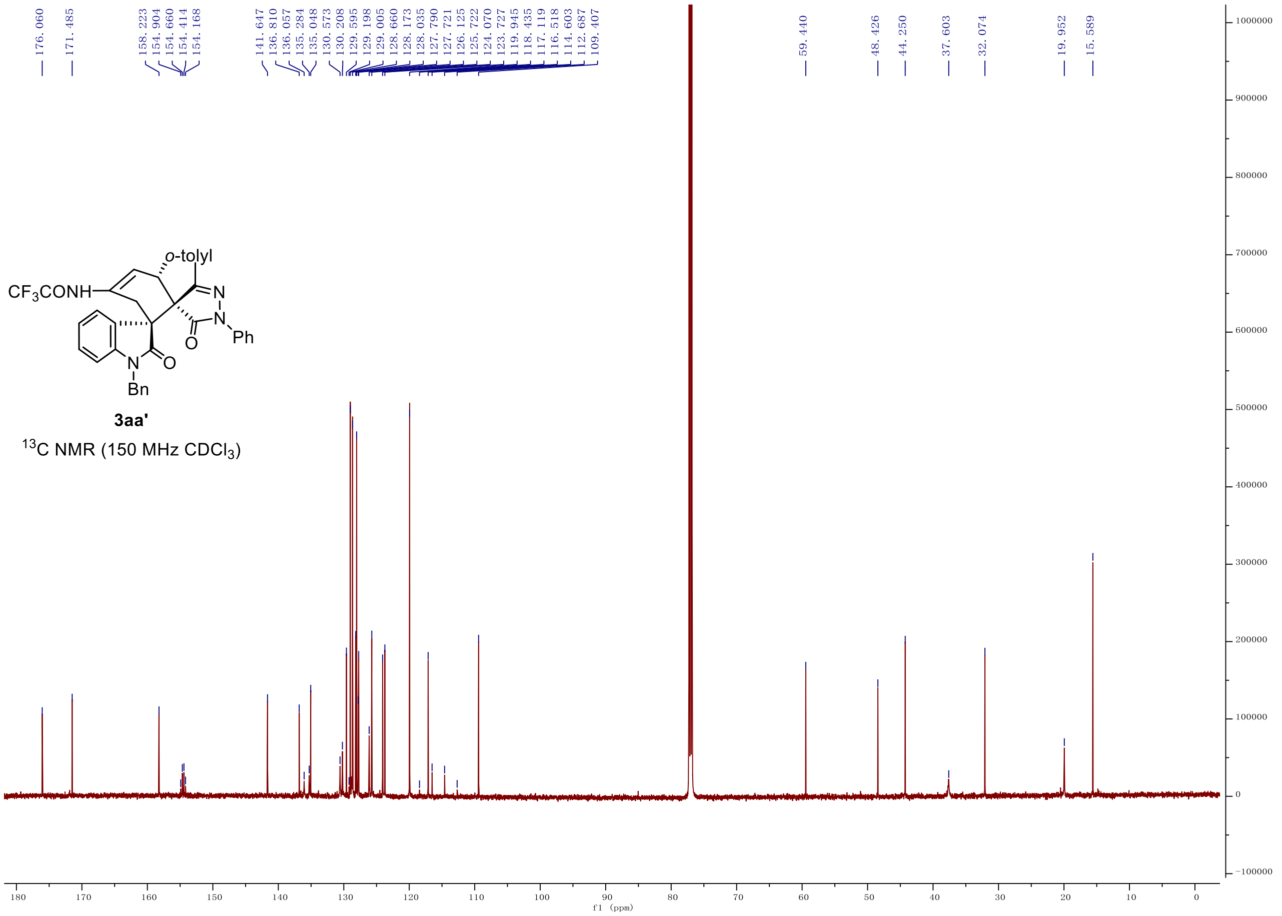
176.060
171.485
158.223 154.904 154.660 154.414 154.168
141.647 136.810 136.057 135.284 135.048 130.573 130.208 129.595 129.198 129.005 128.660 128.173 128.035 127.790 127.721 126.125 125.722 124.070 123.727 119.945 118.435 117.119 116.518 114.603 112.687 109.407
59.440
48.426
44.250
37.603
32.074
19.952
15.589
o-tolyl
CF3CONH
N
N
Ph
O
N
O
Bn
3aa'
13C NMR (150 MHz CDCl3)
f1 (ppm)

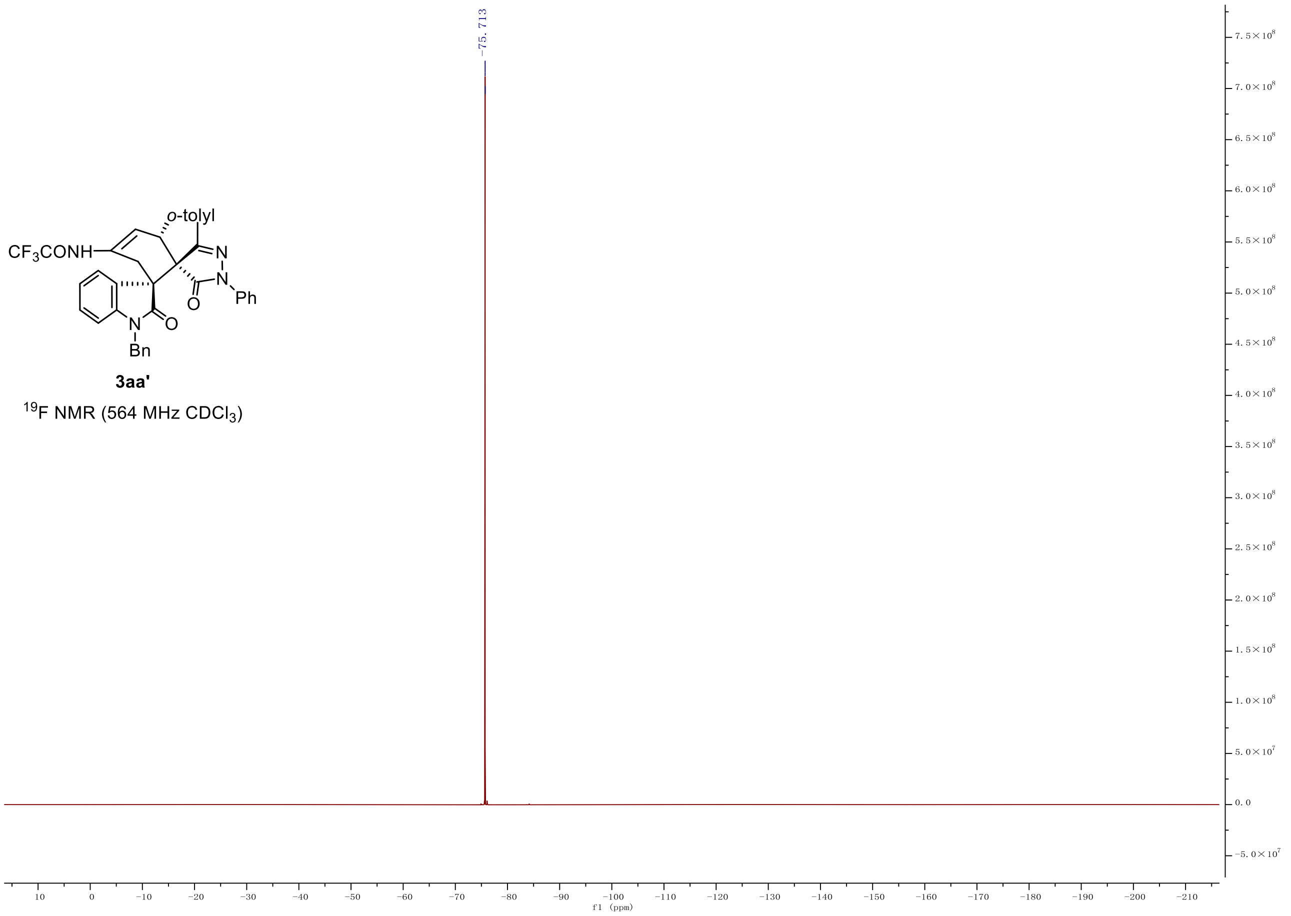

3aa'

^{19}F NMR (564 MHz $CDCl_3$)

Peak Analysis Report

Detector A Channel 1 254nm

No.	Ret. Time	Height (mAu)	Area (mAu*min)	Rel. Area (%)
1	6.109	345383	4224257	50.302
2	6.564	283930	4173554	49.698
Total		629312	8397811	100.000

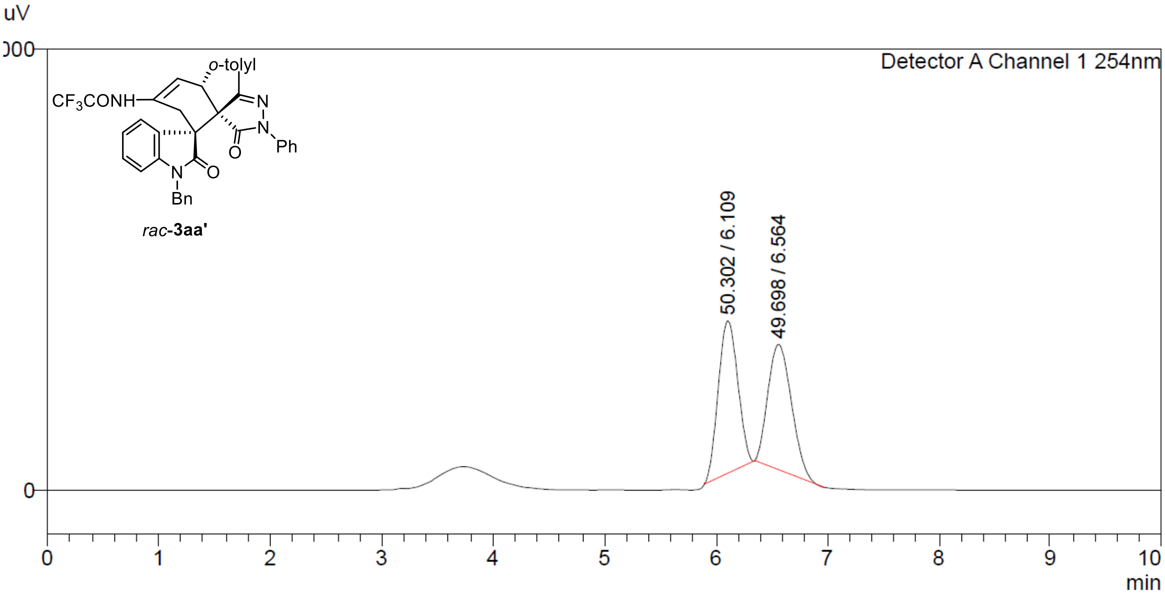

rac-**3aa'**

Peak Analysis Report

Detector A Channel 1 254nm

No.	Ret. Time	Height (mAu)	Area (mAu*min)	Rel. Area (%)
1	6.054	-56	1885	0.003
2	6.468	2869125	55584225	99.997
Total		2869068	55586110	100.000

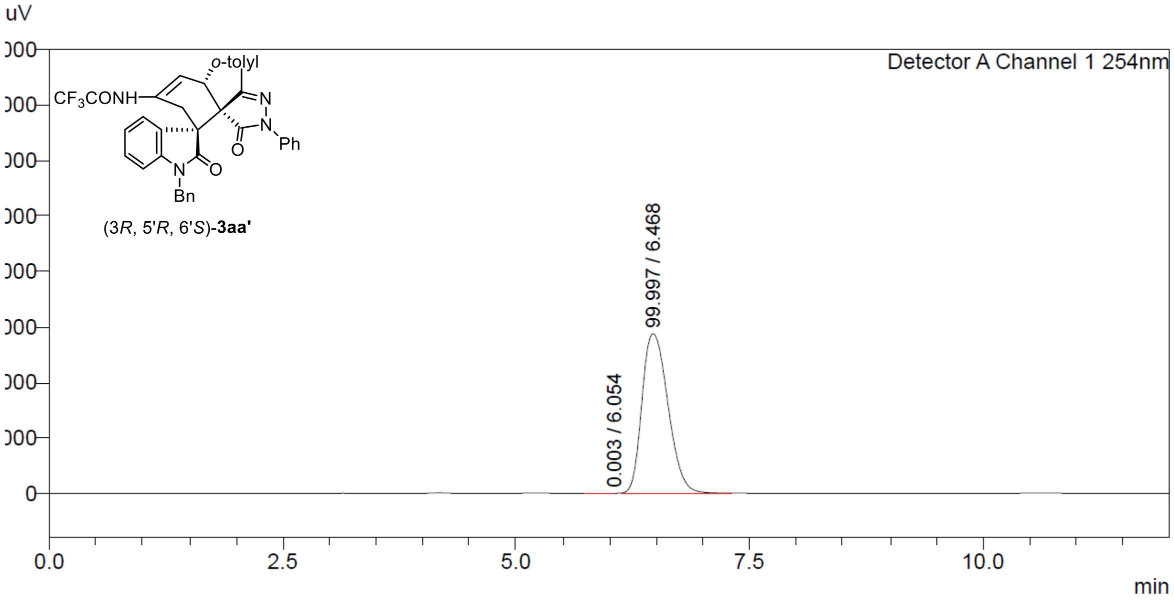

Peak Analysis Report

Detector A Channel 1 254nm

No.	Ret. Time	Height (mAu)	Area (mAu*min)	Rel. Area (%)
1	6.102	360820	5386602	99.189
2	6.513	7016	44061	0.811
Total		367836	5430663	100.000

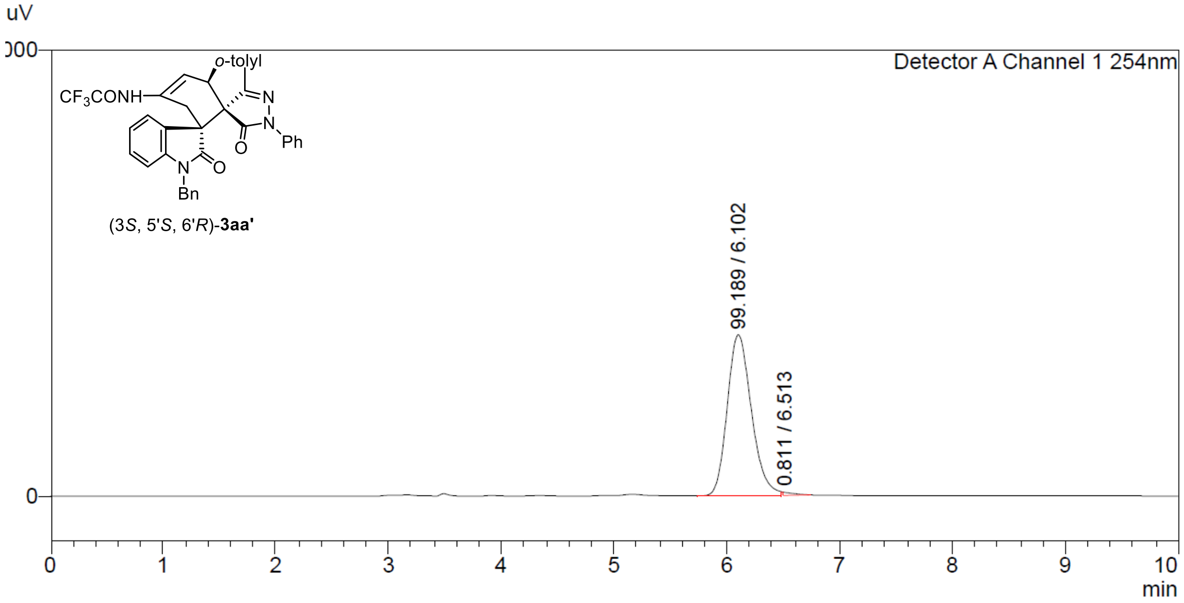

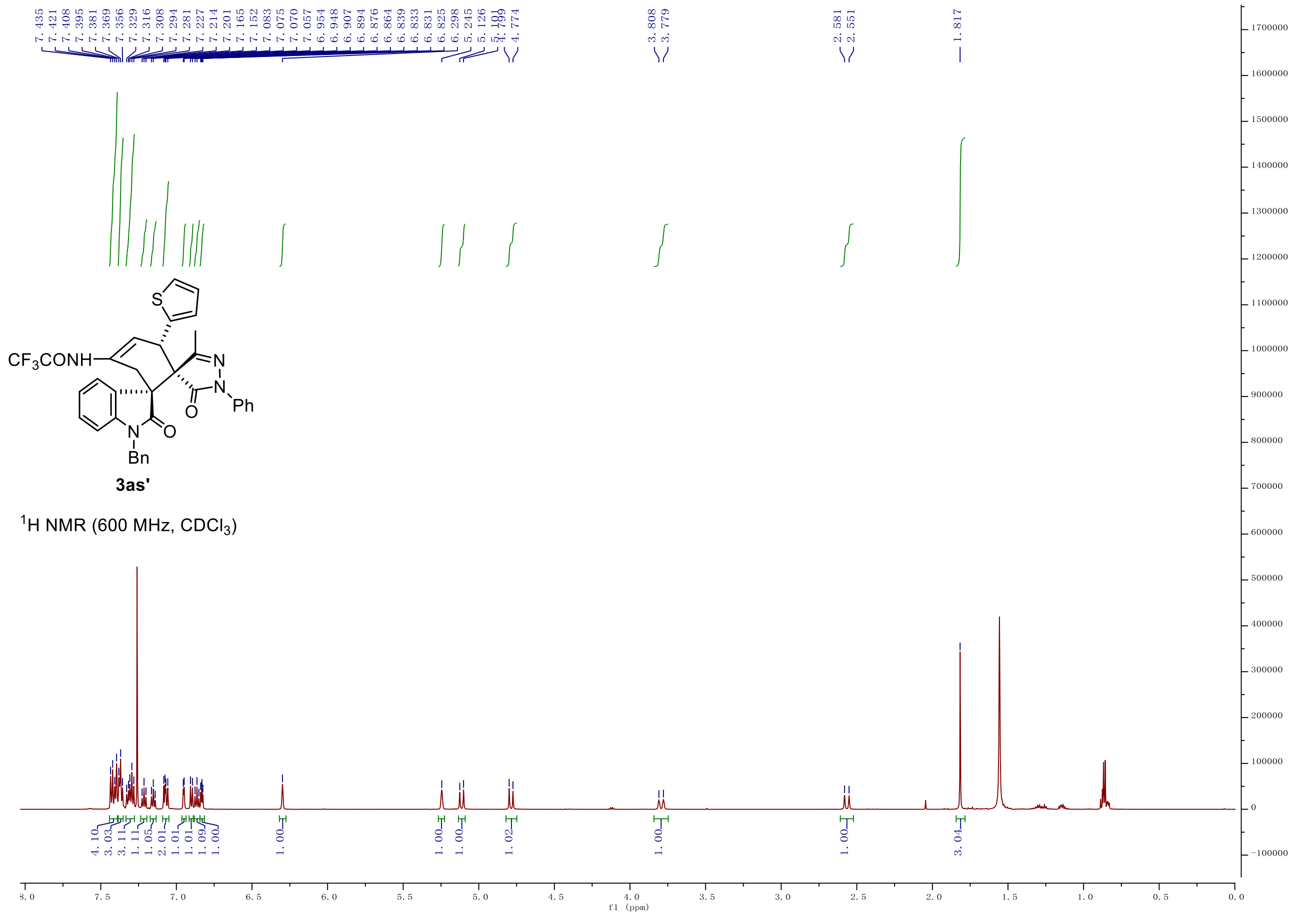

7.435 7.421 7.408 7.395 7.381 7.369 7.356 7.329 7.316 7.308 7.294 7.281 7.227 7.214 7.201 7.165 7.152 7.083 7.075 7.070 7.057 6.954 6.948 6.907 6.894 6.876 6.864 6.839 6.833 6.831 6.825 6.298 5.245 5.126 5.101 4.799 4.774
3.808 3.779
2.581 2.551
1.817
CF3CONH
S
N
N
Ph
O
O
N
Bn
3as'
1H NMR (600 MHz, CDCl3)
4.10 3.03 3.11 1.11 1.05 2.01 1.01 1.01 1.09 1.00 1.00 1.00 1.00 1.02 1.00 1.00 3.04
f1 (ppm)

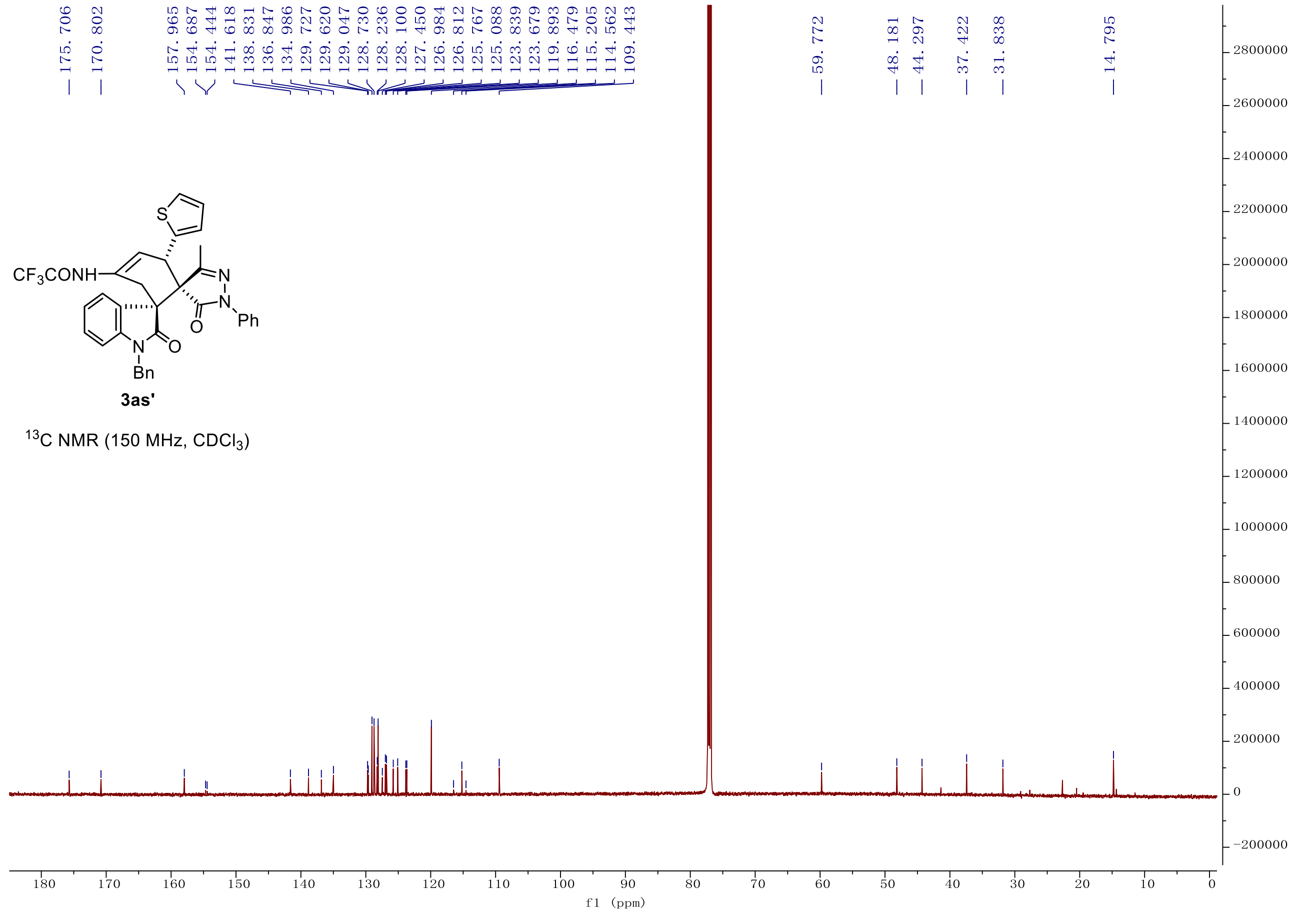

175.706 170.802 157.965 154.687 154.444 141.618 138.831 136.847 134.986 129.727 129.620 129.047 128.730 128.236 128.100 127.450 126.984 126.812 125.767 125.088 123.839 123.679 119.893 116.479 115.205 114.562 109.443
59.772 48.181 44.297 37.422 31.838 14.795
CF3CONH
S
N
N
Ph
O
O
N
Bn
3as'
13C NMR (150 MHz, CDCl3)
f1 (ppm)

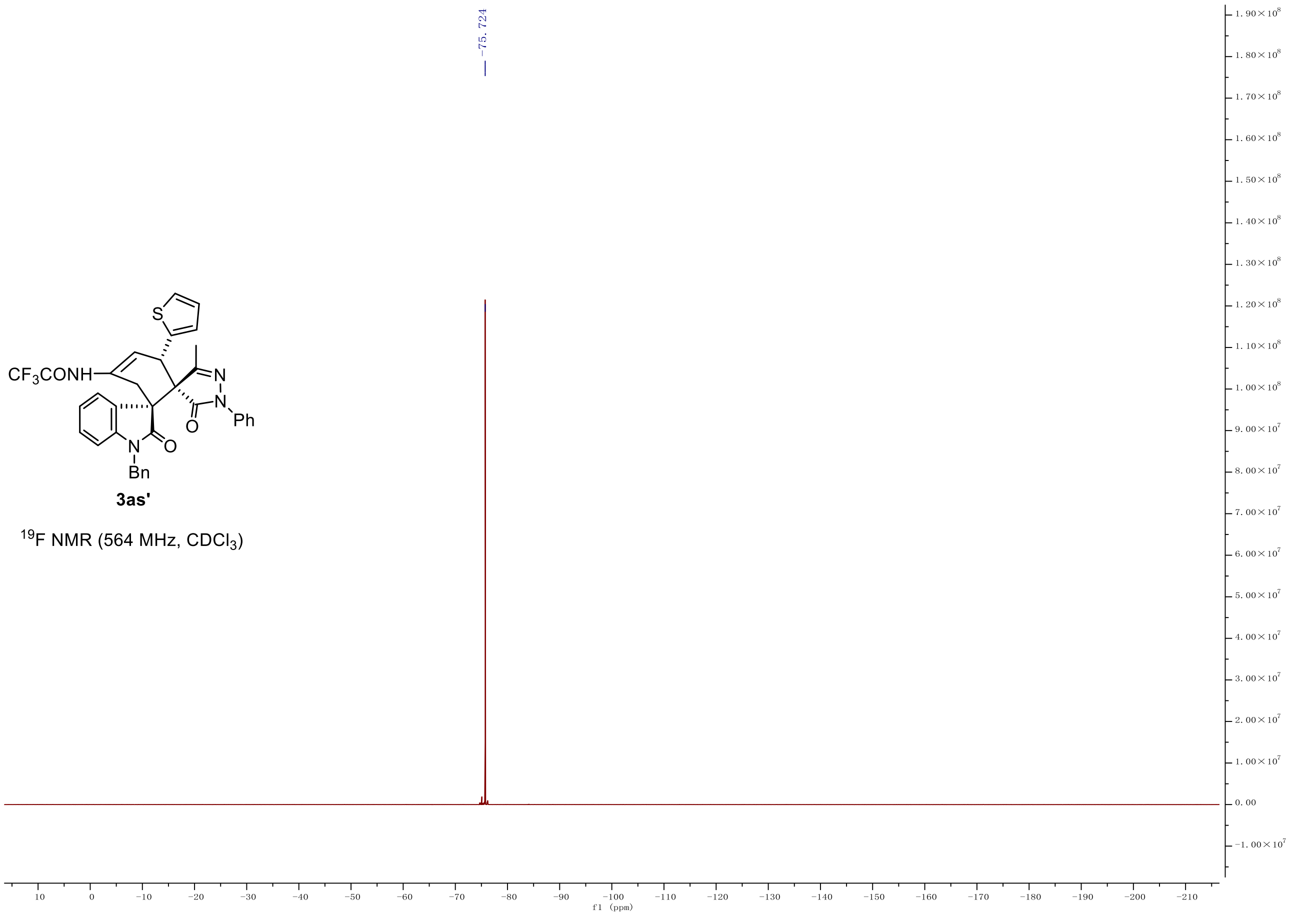

Peak Analysis Report

Detector A Channel 1 254nm

No.	Ret. Time	Height (mAu)	Area (mAu*min)	Rel. Area (%)
1	8.933	978654	22735813	50.702
2	9.960	690613	22106146	49.298
Total		1669267	44841959	100.000

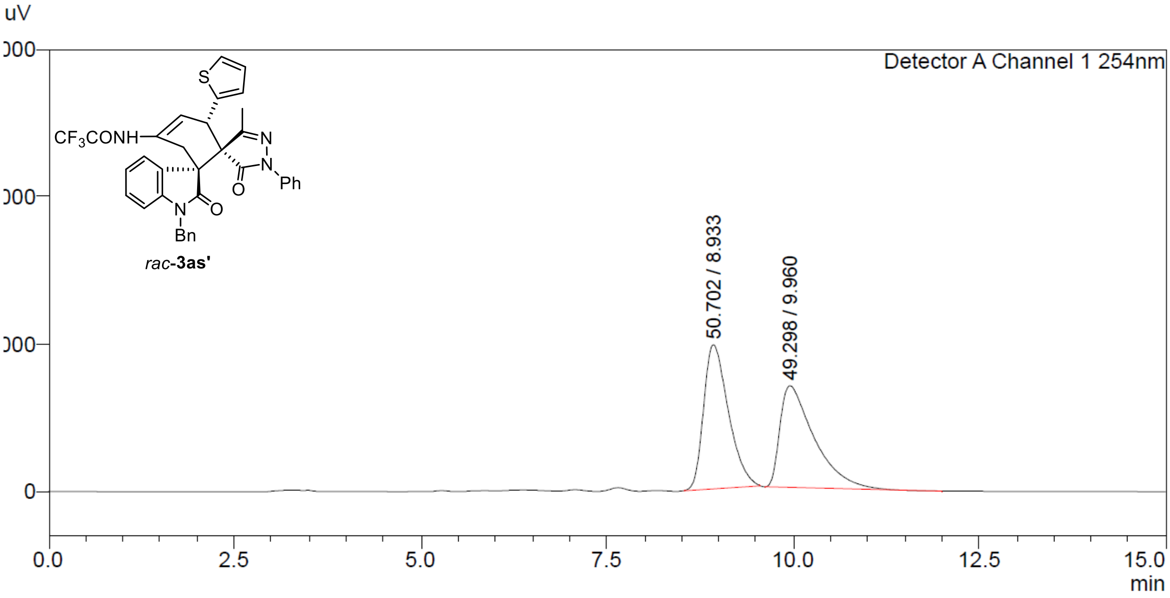

Peak Analysis Report

Detector A Channel 1 254nm

No.	Ret. Time	Height (mAu)	Area (mAu*min)	Rel. Area (%)
1	9.002	347754	8946022	99.594
2	10.438	1267	36428	0.406
Total		349020	8982450	100.000

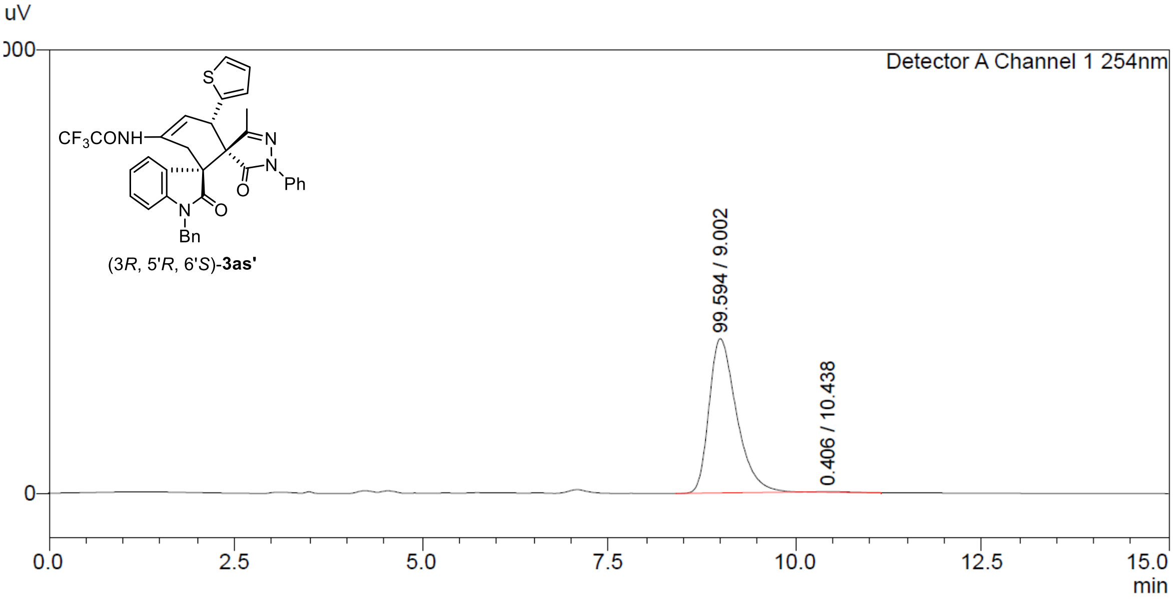

Peak Analysis Report

Detector A Channel 1 254nm

No.	Ret. Time	Height (mAu)	Area (mAu*min)	Rel. Area (%)
1	9.126	4498	111317	0.894
2	10.101	349158	12347024	99.106
Total		353656	12458341	100.000

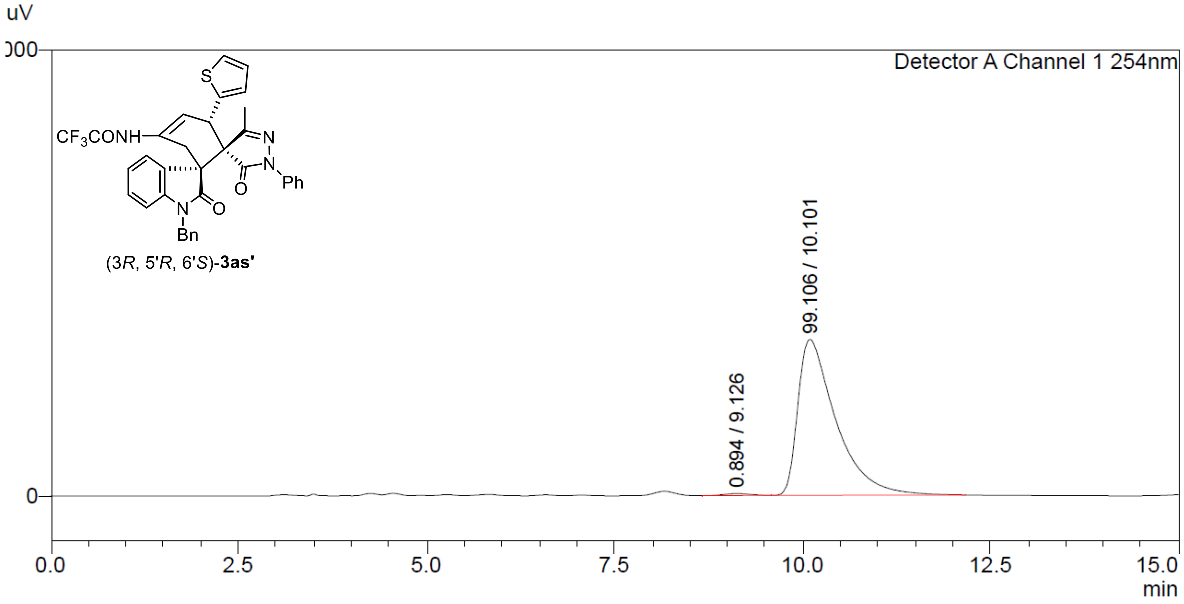

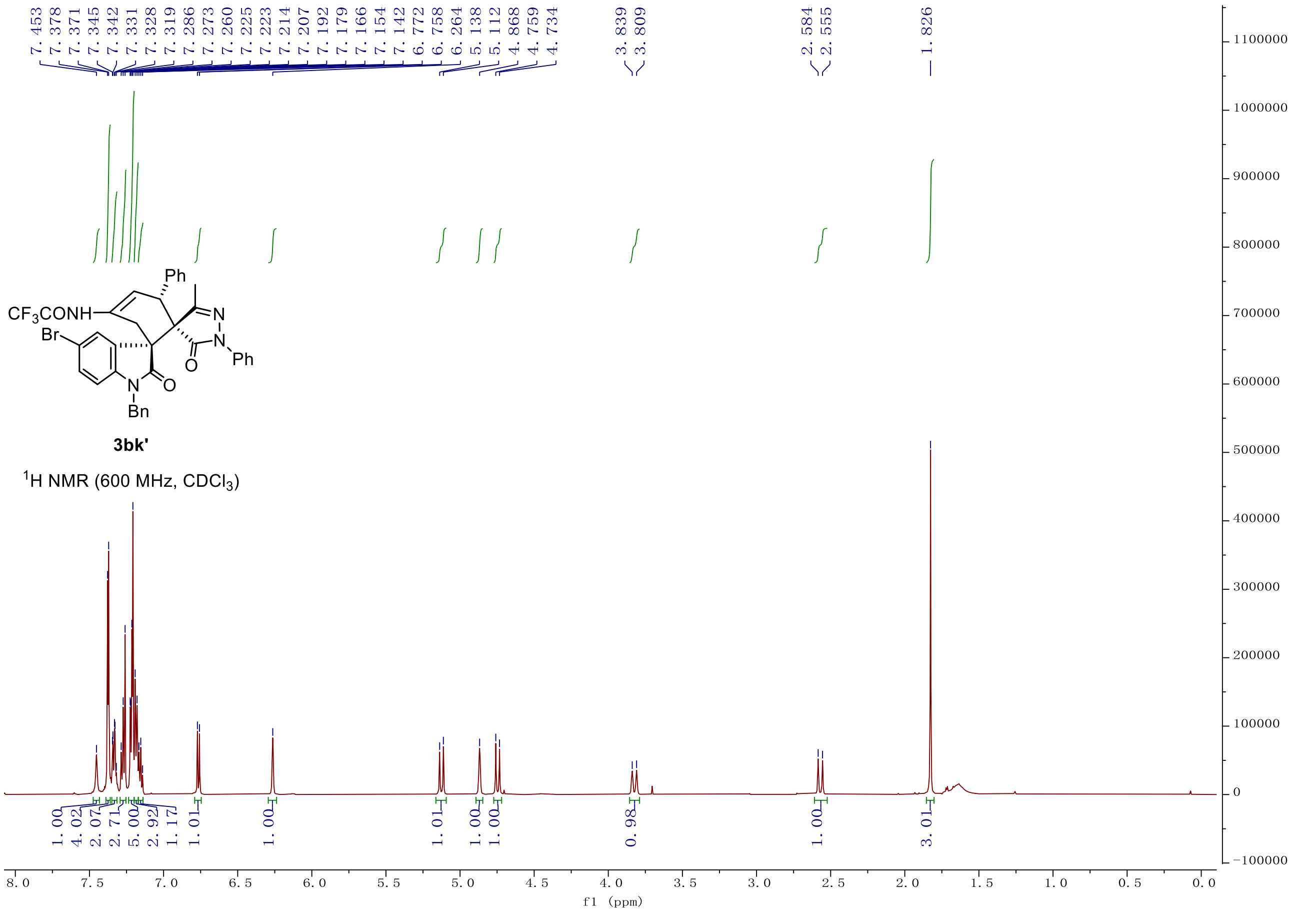
7.453
7.378
7.371
7.345
7.342
7.331
7.328
7.319
7.286
7.273
7.260
7.225
7.223
7.214
7.207
7.192
7.179
7.166
7.154
7.142
6.772
6.758
6.264
5.138
5.112
4.868
4.759
4.734
3.839
3.809
2.584
2.555
1.826
CF3CONH
Br
Ph
N
N
Ph
O
O
N
Bn
3bk'
1H NMR (600 MHz, CDCl3)
1.00
4.02
2.07
2.71
5.00
2.92
1.17
1.01
1.00
1.01
1.00
1.00
0.98
1.00
3.01
f1 (ppm)

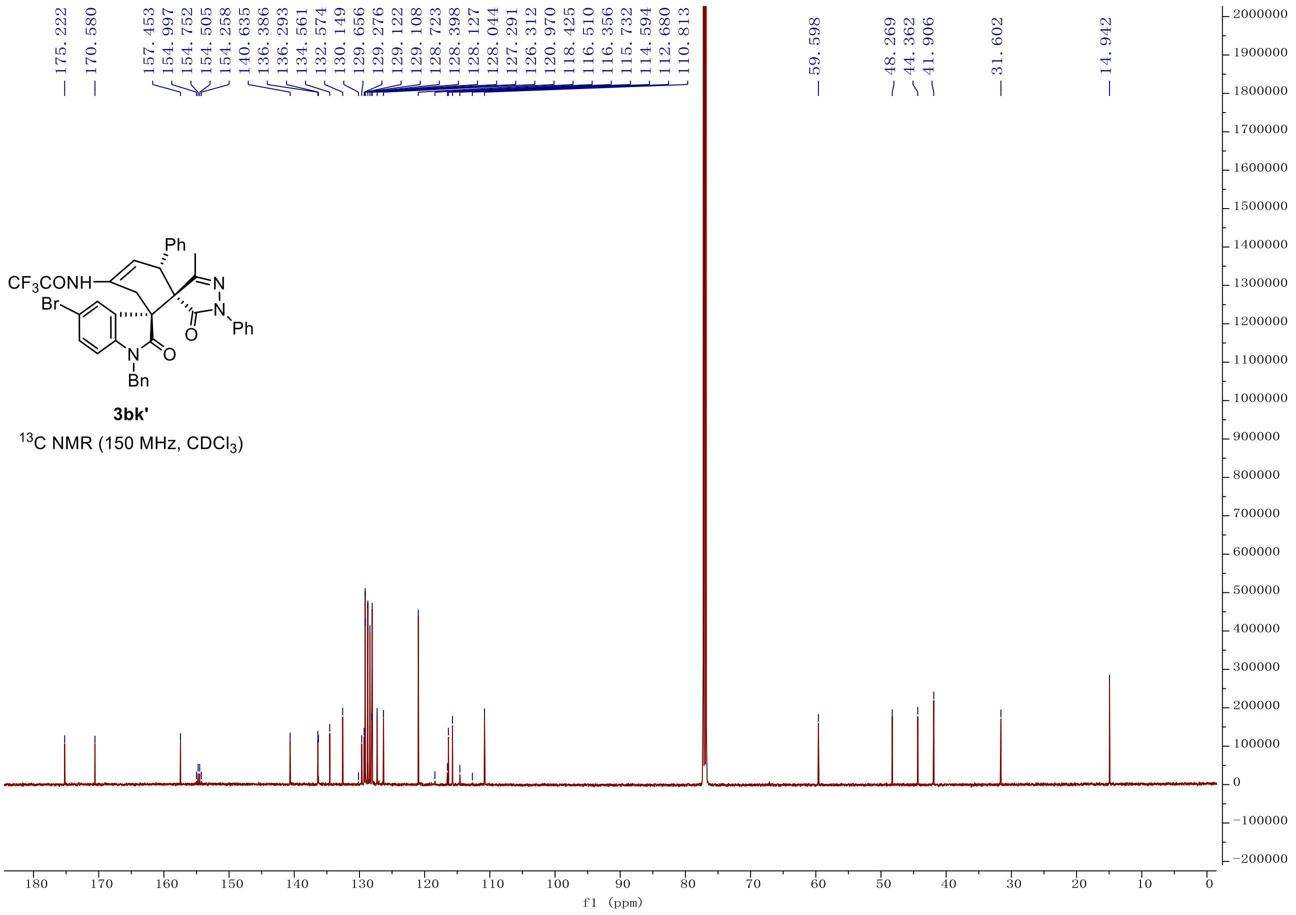
175.222
170.580
157.453
154.997
154.752
154.505
154.258
140.635
136.386
136.293
134.561
132.574
130.149
129.656
129.276
129.122
129.108
128.723
128.398
128.127
128.044
127.291
126.312
120.970
118.425
116.510
116.356
115.732
114.594
112.680
110.813
59.598
48.269
44.362
41.906
31.602
14.942
CF3CONH
Br
Ph
N
N
Ph
O
O
N
Bn
3bk'
13C NMR (150 MHz, CDCl3)
f1 (ppm)

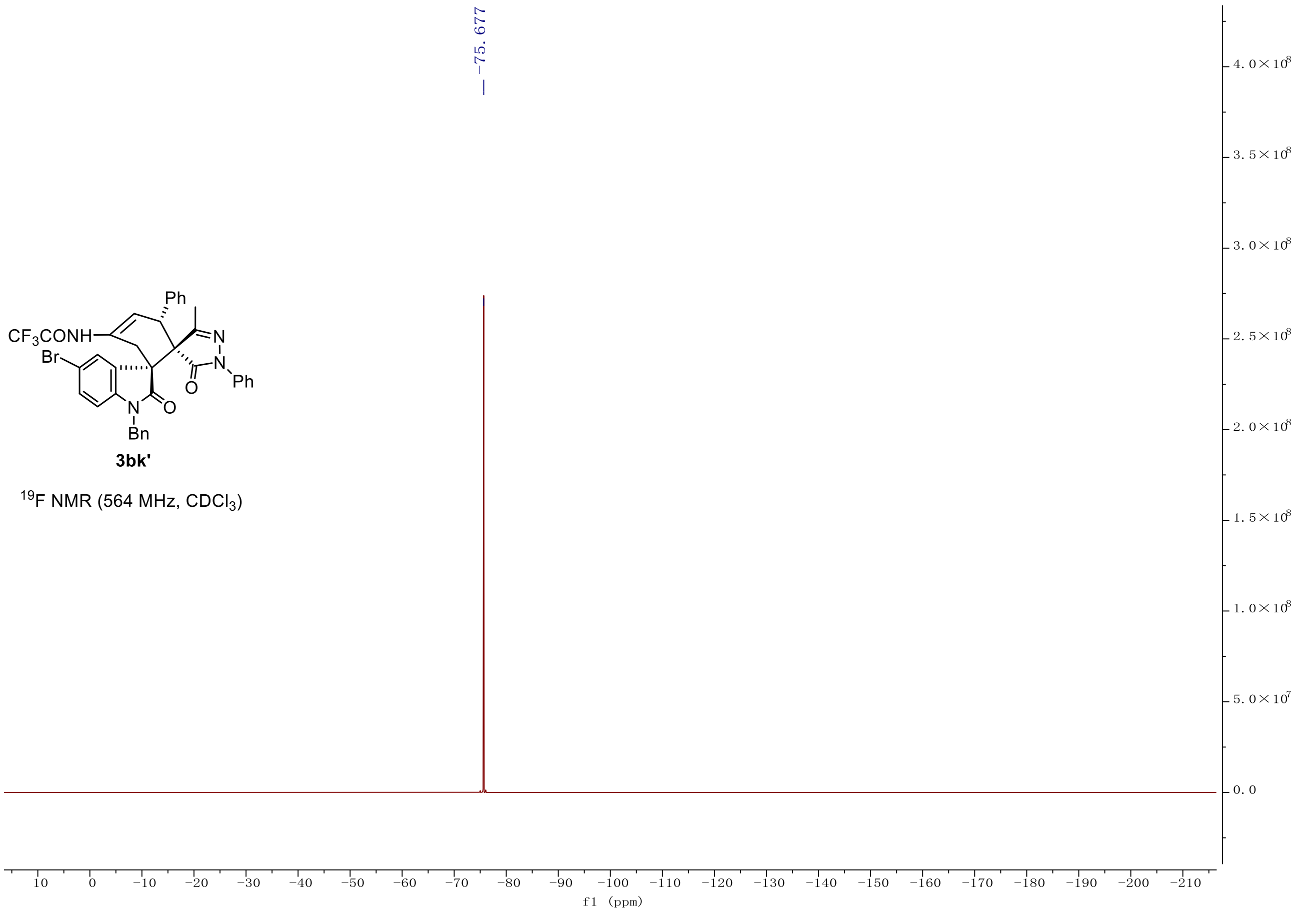

Peak Analysis Report

Detector A Channel 1 254nm

No.	Ret. Time	Height (mAu)	Area (mAu*min)	Rel. Area (%)
1	10.868	43174	1365309	22.944
2	12.052	58553	1665261	27.985
3	14.000	42745	1629970	27.392
4	15.421	33873	1290029	21.679
Total		178346	5950569	100.000

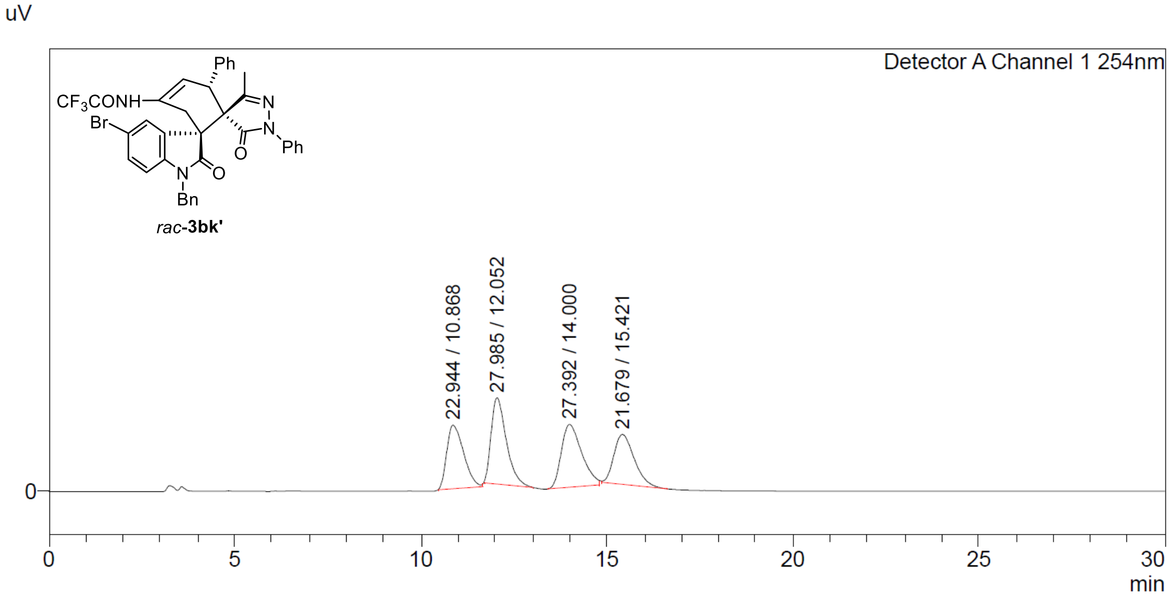

Peak Analysis Report

Detector A Channel 1 254nm

No.	Ret. Time	Height (mAu)	Area (mAu*min)	Rel. Area (%)
1	10.821	10251	322292	1.221
2	14.951	640891	26064653	98.779
Total		651142	26386946	100.000

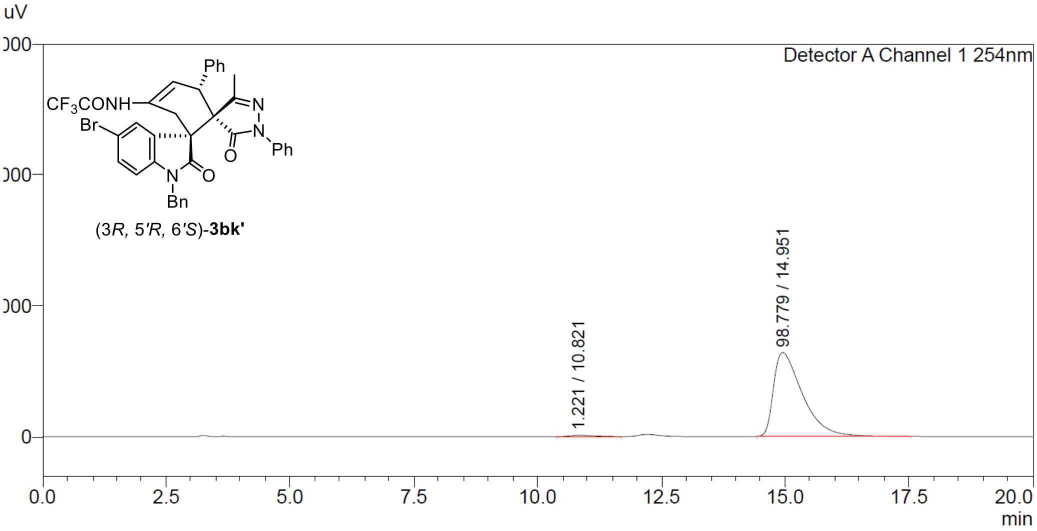

Peak Analysis Report

Detector A Channel 1 254nm

No.	Ret. Time	Height (mAu)	Area (mAu*min)	Rel. Area (%)
1	10.514	1594708	49972453	98.947
2	14.185	16931	531679	1.053
Total		1611640	50504133	100.000

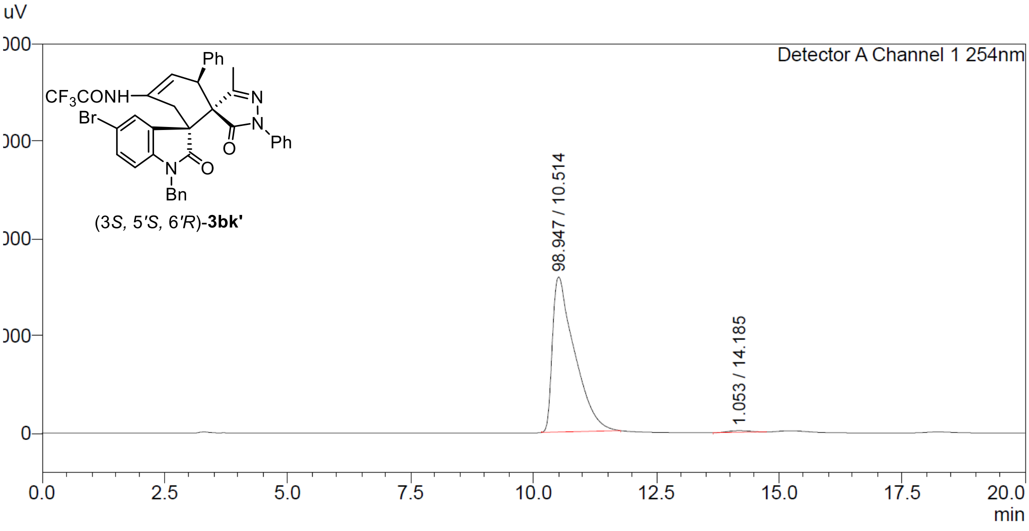

Supplement: Supplementary file 1 — Supplementary Text Tables S1 to S3 Figs. S1 and S2 [file sciadv.adt5997_sm.pdf]
